# Genetical Genomics Identifies the Genetic Architecture for Growth and Weevil Resistance in Spruce

**DOI:** 10.1371/journal.pone.0044397

**Published:** 2012-09-04

**Authors:** Ilga Porth, Richard White, Barry Jaquish, René Alfaro, Carol Ritland, Kermit Ritland

**Affiliations:** 1 Department of Forest Sciences, University of British Columbia, Vancouver, British Columbia, Canada; 2 Department of Statistics, University of British Columbia, Vancouver, British Columbia, Canada; 3 Kalamalka Forestry Centre, British Columbia Ministry of Forests, Lands and Natural Resource Operations, Vernon, British Columbia, Canada; 4 Pacific Forestry Centre, Canadian Forest Service, Victoria, British Columbia, Canada; University of Umeå, Sweden

## Abstract

In plants, relationships between resistance to herbivorous insect pests and growth are typically controlled by complex interactions between genetically correlated traits. These relationships often result in tradeoffs in phenotypic expression. In this study we used genetical genomics to elucidate genetic relationships between tree growth and resistance to white pine terminal weevil (*Pissodes strobi* Peck.) in a pedigree population of interior spruce (*Picea glauca, P*. *engelmannii and their hybrids*) that was growing at Vernon, B.C. and segregating for weevil resistance. Genetical genomics uses genetic perturbations caused by allelic segregation in pedigrees to co-locate quantitative trait loci (QTLs) for gene expression and quantitative traits. Bark tissue of apical leaders from 188 trees was assayed for gene expression using a 21.8K spruce EST-spotted microarray; the same individuals were genotyped for 384 SNP markers for the genetic map. Many of the expression QTLs (eQTL) co-localized with resistance trait QTLs. For a composite resistance phenotype of six attack and oviposition traits, 149 positional candidate genes were identified. Resistance and growth QTLs also overlapped with eQTL hotspots along the genome suggesting that: 1) genetic pleiotropy of resistance and growth traits in interior spruce was substantial, and 2) master regulatory genes were important for weevil resistance in spruce. These results will enable future work on functional genetic studies of insect resistance in spruce, and provide valuable information about candidate genes for genetic improvement of spruce.

## Introduction

Plants are sessile organisms that have evolved many resistance mechanisms to defend against insect pests. These resistance mechanisms are genetically complex and involve interactions between both host and pests [1], [2]. Recently, a “cost-benefit” paradigm for resistance has emerged to enhance our understanding of these interactions [3]–[7]. This paradigm suggests that tradeoffs in the cost-benefit paradigm may be due to correlated selection (favored trait combinations) and spatio-temporal heterogeneity of the environment [8]. Theoretical approaches have also described the importance of resource allocation within biosynthetic pathways for the evolution of resistance [9].

Meta-analyses of plant-herbivore defenses suggest that trade-offs exist between constitutive and induced defenses. More competitive species tend to exhibit lower constitutive and higher induced resistance than less competitive species ([10], [11]). It has also been hypothesized that both constitutive and induced resistance are influenced by selection on traits that alter plant growth rates [12]. In spruce (*Picea* spp.), constitutive and induced defenses are thought to follow sequentially [13]. Strength and rapidity of traumatic resinosis have often been related to resistance. Nevertheless, Alfaro [13] suggested that in response to wounding, some resistant trees failed to produce the traumatic response and some susceptible trees responded with an unexpectedly intensified response.

Phenotypic and genetic relationships between growth and resistance to white pine terminal weevil (*Pissodes strobi* Peck.) have been intensively studied in interior spruce (*Picea glauca* [Moench] Voss, *P. engelmannii* Parry and their hybrids); however, results have been inconsistent and seemingly contradictory. Kiss and Yanchuk [14] found a negative genetic correlation between mean family height and weevil damage in interior spruce, while King et al. [15] reported a positive phenotypic but a strong negative genetic correlation between attack level and leader height. Alfaro et al. [16] reported better developed bark resin canals in fast-growing trees, while Lieutier et al. [17] concluded that there is no relationship between tree growth and resistance in Norway spruce (*Picea abies*. ((L.) Karst.). Vandersar and Borden [18] suggested that weevils prefer faster growing trees, and more recently He and Alfaro [19] found a higher survival time for shorter trees. In Sitka spruce (*Picea sitchensis* (Bong.) Carr.), genetic resistance was most pronounced in families with average height growth [20]. This finding is interesting, since improved growth rate in spruce trees has lead to a higher predisposition to weevil attacks [21].

Trade-offs between correlated traits may be due to genetic and/or phenotypic variation. At the least, genetically correlated traits share quantitative trait loci (QTLs). However, pleiotropic genes, which control the hubs in such trade-offs, are difficult to distinguish from the confounding physically linked loci within a shared QTL. Moreover, biometric correlations and QTLs do not always concur because of the presence of obscuring antagonistic QTLs [22]. This failure to detect significant correlations may indicate the extent of independent variation between two traits and not necessarily the absence of a tradeoff. Other interacting factors can remain undetected [8]. In other species, pleiotropy and genetic correlations may be present. Examples include dehydration avoidance and flowering time in *Arabidopsis thaliana* [23], resistance and tolerance to herbivory in the common morning glory [24], and growth rate and flowering in *A. thaliana* [25]. While *A. thaliana* has facilitated research on tradeoffs for life-history traits in annual plants with short life cycles, research on long-lived forest trees promises new perspectives on molecular mechanisms of life-history control in non-model species [26].

In this paper, we present results on the use of genetical genomics to investigate a question of fundamental importance in plant genomics: How do genes underlying a pathway to pest resistance concertedly function? We investigated growth and insect resistance as a trait pair that defines the life history of interior spruce, a commercially valuable and ecologically important coniferous tree species. We show how genetical genomics can provide a fine-scale analysis of the genetic architecture in the study of pest resistance cost-benefit tradeoffs. Genetical genomics assays thousands of traits (gene expression levels) [27] and these “expression phenotypes” are subjected to standard QTL analysis. We use genetical genomics to infer the nature of resistance of interior spruce to the white pine weevil. We infer expression QTLs (eQTL) in segregating crosses of interior spruce, with variable resistance to white pine weevil. In this analysis, the positions of eQTLs indicate regions that harbor regulatory elements that control expression of genes in the same pathway. In the case of *cis*-regulation, the genomic location of the eQTL coincides with the physical location of the regulated gene, while *trans*-acting eQTLs identify regulatory elements for the gene elsewhere in the genome. The distribution of eQTLs may spread evenly on the genome or may appear in clusters or “hotspots”, depending on the genetic architecture of these gene interactions [28]. At the QTL level, the action of a gene might suggest pleiotropy because multiple traits are affected. We search for pleiotropy based on common candidate genes between resistance and growth. Furthermore, a positive correlation between growth rate, and attack and oviposition rate (our resistance measure) might indicate a tradeoff between growth and defenses. This tradeoff could be due to the increased carbon cost required for higher defense chemical levels. We also search for master regulons that underlie *trans*-eQTL hotspots (“hubs”) which tend to be at the center of gene expression networks (network eQTLs).

## Results

### Correlations between Phenotypic Estimates

The phenotypic response data consisted of tree height measurements and weevil attack and oviposition counts. Tree height measures were taken at the time of planting in 1995 and at three and five growing seasons thereafter. Leader length was measured in year five preceding the artificial augmentation of a local weevil population in October of the same year (hgt_1995, hgt_1997, hgt_1999, and ldr_99, respectively). Weevil attack rates counted in 2000 and 2001 were classified as successful top kills, failure to kill the leader, and no attack (atk_2000, atk_2001). In the same years, oviposition was assessed (egg_2000, egg_2001) and egg counts along the leaders were summarized into five discrete classes. The sum of weevil attacks and the sum of oviposition for 2000 and 2001 were also used as response traits (sum_atk, and sum_egg).

Forty-five pairwise phenotypic correlations were estimated for individual and sum traits (**Table 1**). In general, correlations between egg counts and attack classification were strong, between initial height (hgt 1995) and later growth correlation was weak, and between hgt_1999, ldr_99 and hgt_1997 correlations were strong. Correlations between growth (ldr 99 and hgt 1999) and attack (resistance) traits (atk_2000, egg_2000, sum_atk and sum egg) were generally positive (**Table 1** **)**. Collectively, these results suggest a negative relationship between growth and the actual pest resistance (fewer attacks).

**Table 1:** Pearson’s correlation coefficients for phenotypic (*above* diagonal) and for QTL correlations (*below* diagonal).

	ldr_99	hgt_1995	hgt_1997	hgt_1999	atk_2000	atk_2001	sum_atk	egg_2000	egg_2001	sum_egg
**ldr_99**		0.028	0.447	0.823	0.300	0.068	0.274	0.392	0.037	0.331
**hgt_1995**	0.035		0.357	0.142	−0.064	0.057	−0.009	−0.042	0.070	0.014
**hgt_1997**	0.034	0.095		0.723	0.089	0.163	0.181	0.093	0.235	0.231
**hgt_1999**	0.207	0.074	0.327		0.283	0.109	0.290	0.319	0.121	0.331
**atk_2000**	0.158	0.071	0.113	0.146		−0.050	0.719	0.782	−0.103	0.541
**atk_2001**	−0.042	−0.127	0.105	0.075	0.170		0.658	−0.001	0.799	0.540
**sum_atk**	0.218	0.141	0.169	0.056	0.560	0.178		0.589	0.478	0.784
**egg_2000**	−0.044	−0.106	0.184	0.255	0.465	0.282	0.348		−0.065	0.738
**egg_2001**	0.051	−0.121	0.133	0.111	0.198	0.634	0.032	0.250		0.625
**sum_egg**	0.177	0.131	0.352	0.183	0.357	0.283	0.309	0.279	0.166	

### QTL Mapping

The spruce mapping population was genotyped for 384 SNP loci. These SNPs had been detected within expressed sequence tag (EST) contigs that represented assemblies of short expressed sequences with predicted open reading frames. These ESTs originated from the spruce Treenomix EST database (K. Ritland, personal communication). The genotypic information was used to estimate pairwise recombination rates between SNP loci and construct a framework genetic linkage map to localize quantitative trait loci (QTLs). Details about the genetic linkage map and the annotation of the mapped contigs can be found in the supplement material of [29]. The phenotypic variations that were obtained from four tree height, three weevil attack, three oviposition (see above), and extensive quantitative gene expression (21,840 transcripts) measures were mapped to the established genetic linkage map of the factorial progeny. The QTLs were mapped by using a likelihood function to assess the phenotype effect conditioned on the genotypic variation. A (e)QTL was significant with a LOD ≥3.84. In total, we identified 132,100 significant eQTLs (see **File S1** and legends in **Table S1**).

**Figure 1:**
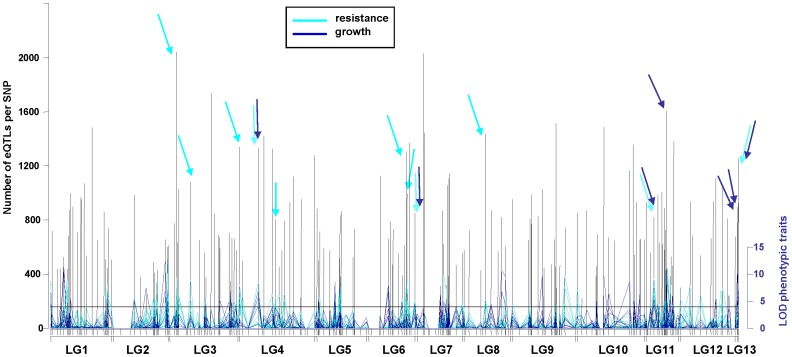
EQTL density map with overlapping positions of pQTLs at individual marker positions. Linkage groups (LG1-13) are displayed horizontally, black bars indicate SNP marker positions in linkage groups; arrows mark positions with at least three pQTLs (LOD ≥3.84, i.e. values above horizontal line) and eQTL numbers >786.

For each SNP locus along the genetic linkage map, we superimposed the mapped phenotypic trait QTLs (pQTLs) with the counts of significant eQTLs (**Figure 1**), and identified hubs of trait variation at several SNP loci that comprised of multiple pQTLs and an extensive accumulation of eQTLs. A goodness-of-fit test that assumed a uniform distribution was performed to test whether the observed frequencies of eQTLs along the linkage map differed significantly from the expected value. Then we used all detected eQTLs and all marker loci (see above) in a randomization procedure to assess the maximum number of eQTLs within eQTL clusters. According to this randomly generated data set, “eQTL hotspots” would be declared if the number of eQTLs at a given locus exceeded 630. However, we arbitrarily used a cutoff value of 786. This number was simply the value where the expected average value was exceeded by 50%. In this way, we focused on fewer hubs of *trans*-eQTLs that are associated with important regulators of quantitative trait variation. Fourteen loci coincided with eQTL hubs and with at least three pQTLs (**Figure 1**).

### Hotspots of Phenotypic Trait Variation

**Figure 2:**
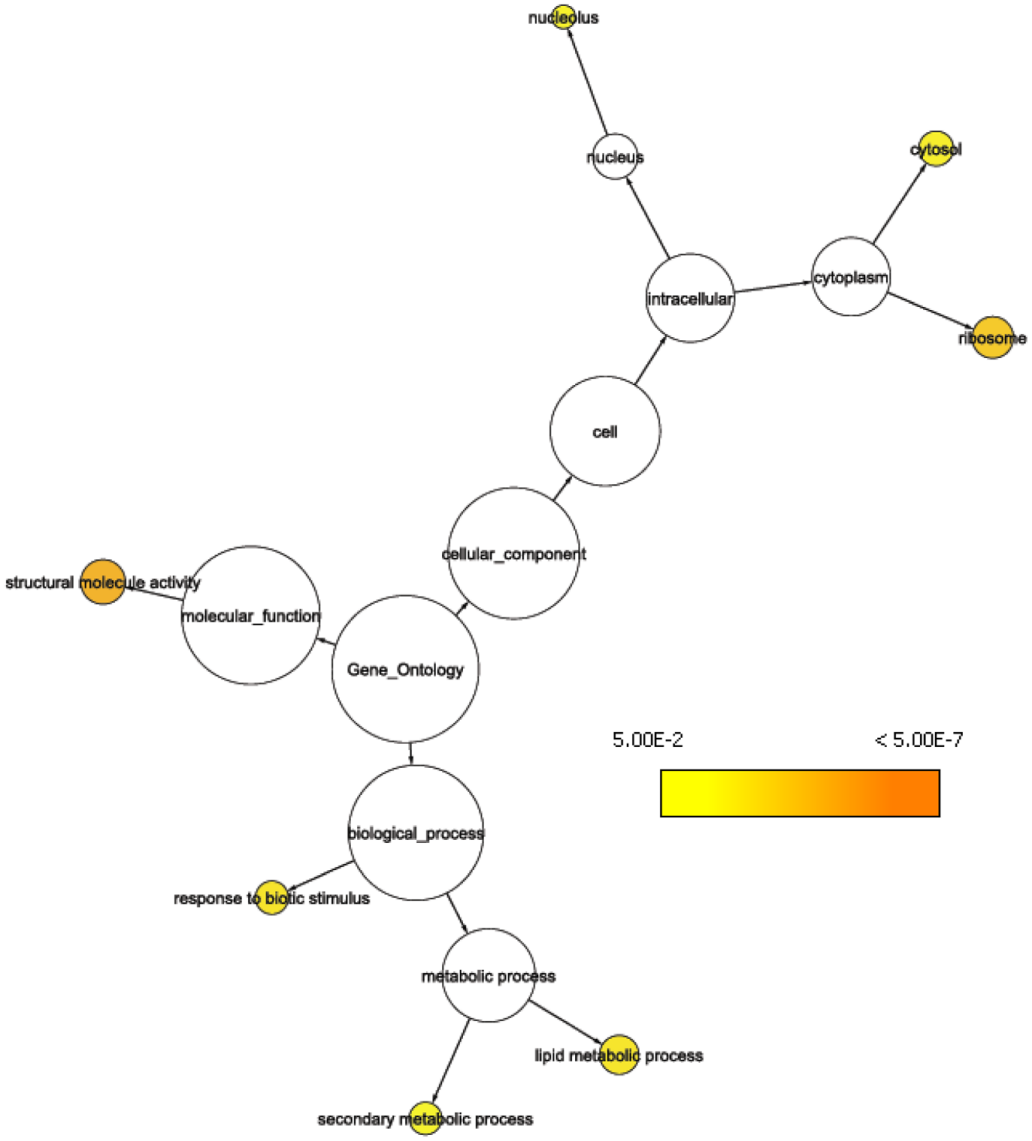
GO tree representation showing significantly (p ≤ 0.05) overrepresented GO categories at gene locus PiglCCoAOMT-1. Representing the *trans* eQTL-hotspot on LG6 (WS0031_301, SNP marker Contig_4096_434).

**Figure 3:**
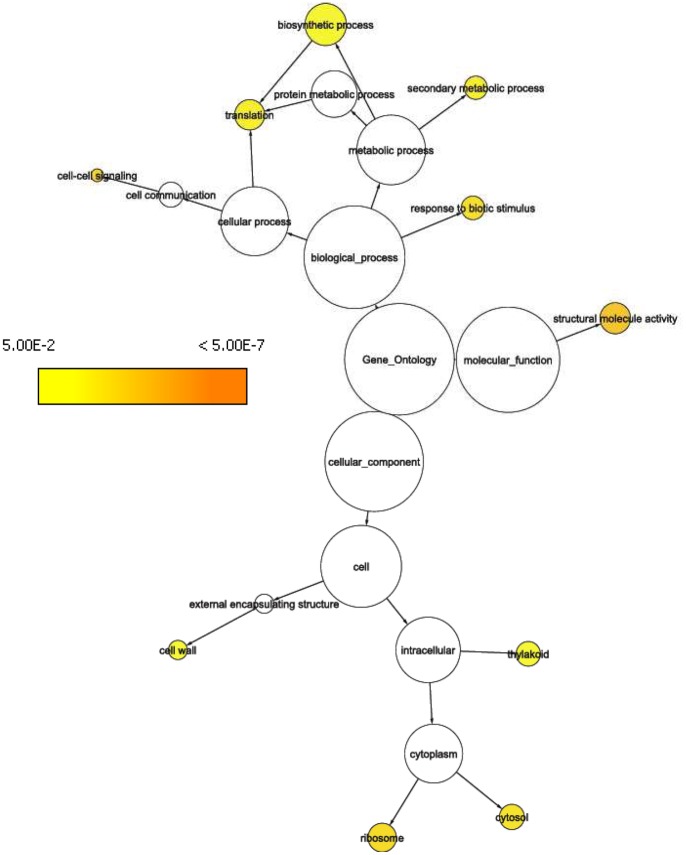
GO tree representation showing significantly (p ≤ 0.05) overrepresented GO categories at gene locus PiglCCoAOMT-2. Representing the *trans* eQTL-hotspot on LG6 (WS0064_O09, SNP marker CCoAOMT_1_320).

From these 14 loci, seven loci were associated exclusively with resistance pQTLs, three loci with growth pQTLs, while four loci with pQTLs from individual traits of both growth and resistance, respectively (**Figure 1**). The composition of two *trans*-eQTL hotspots with extensive resistance pQTL overlap was analyzed in more detail (see below and **Figure 2**, and **Figure 3** **; Table S2**, and **Table S3**).

At eight map positions, at least four pQTLs overlapped with eQTL hotspots on five different linkage groups (LG): SNP44 on LG3 (termed Contig_2685_179 and annotated as unknown gene [29]; accumulation of eQTLs from 2040 transcripts, and resistance pQTLs), SNP71 on LG4 (Contig_486_336, no hit [29];1341 transcripts, resistance pQTLs), SNP74 on LG4 (Contig_1623_510, unknown gene [29]; 1333 transcripts, resistance and growth pQTLs), SNP124 on LG6 (Contig_4096_434, caffeoyl-CoA 3-O-methyltransferase CCoAOMT [29];1307 transcripts, resistance pQTLs), SNP125 on LG6 (CCoAOMT_1_320, CCoAOMT [29]; 992 transcripts, resistance pQTLs), SNP210 on LG11 (Contig_1761_256, ubiquitin conjugating enzyme 2 [29]; 820 transcripts, resistance and growth pQTLs), SNP224 on LG11 (Contig_4216_318, predicted protein [29]; 1605 transcripts, growth pQTLs), and SNP252 on LG13 (Contig_1305_426, very weak similarity to cycloidea-like gene [29]; 1253 transcripts, resistance and growth pQTLs).

The two resistance trait associated eQTL hotspots on LG6 were localized at two annotated *bona fide* CCoAOMT genes that are separated by only 1.5 centiMorgan map distance, **Figure 1**. In a previous study that focused on spruce gene families from the phenylpropanoid pathway, we identified the same region enriched for *trans*-eQTLs [29]. Both *CCoAOMT* genes also have *cis*-eQTLs that might represent promoter polymorphisms regulating the differential gene expression in those two loci. At the two different CCoAOMT loci a common set of pQTLs as well as eQTLs clustered (53% and 36% of their accumulated *trans*-eQTLs were generated by the same transcripts, respectively). We compared gene annotations to the 8366 unique *Arabidopsis* annotations from the entire microarray. For both loci the categories ‘structural molecule activity’, ‘secondary metabolic process’, ‘ribosome’, ‘response to biotic stimulus’, and ‘cytosol’ were overrepresented. A Fisher’s exact test was employed to assess significance. Details about the overrepresented ‘GOSlim Plant’ categories within the two eQTL hotspots can be found in **Figure 2** & **Figure 3**. Five jasmonate-ZIM-domain protein (JAZ) genes (WS0105_K14, WS00918_B02, WS0063_E19, WS00918_P17, and WS00919_H21) contributed with eQTLs to this hotspot region, and expression variation for three JAZ genes mapped to both CCoAOMT loci (WS00918_B02, WS00918_P17, and WS00919_H21). Three JAZ genes (WS00919_H21, WS0063_E19, WS00918_P17) were candidate genes directly associated with phenotypic variation for ht_1999, atk_2000 and atk_2001 traits, respectively (**Table S2**, **Table S3**, and **Table S4**). Transcripts from three putative carbonic anhydrase genes (WS00110_A15, WS00928_K21, and WS00936_A24) mapped *trans*-eQTLs to both hotspot locations.

Two carbonic anhydrase loci on LG4, SNP78 and SNP83 (contig_2079_440 and contig_103_602, [29]) are associated with extensive gene expression variation (**Figure S1** & **Figure S2**). At SNP78, three resistance traits mapped significant pQTLs. Seven overrepresented GO categories were in common between eQTL hotspots of carbonic anhydrase SNP83 and the CCoAOMT locus SNP125 (‘biosynthetic process’, ‘cell wall’, ‘secondary metabolic process’ and ‘translation’, e.g.), **Figure 3** and **Figure S2**. At locus SNP125, eQTLs from ethylene-responsive element binding factors (ERFs) linked to defensive gene expression were identified; specifically ERFs from group IX (ERF3, ERF4, ERF7).

The cluster of three phenotype associated eQTL hotspots on LG13 deserves further attention (**Figure 1**), yet we were unable to relate this group of markers to any of the 12 major linkage groups. This might be due to a limited number of segregating markers. At two SNP markers on LG13, SNP250 and SNP251 (WS0021_L13_301 and Contig_2062_390, [29]), related to sequences of glutamate decarboxylase (GAD) genes, significant growth variation and extensive expression variation co-localized.

On four spruce linkage groups (LG4, LG6, LG11 and LG13) hotspots of expression variation associated with QTLs from both growth and resistance traits (**Figure 1**). The pQTLs from resistance traits explained a higher % variation of the trait variation than pQTLs from growth traits. The largest pQTL at any of these eQTL hotspots was identified for sum_egg at SNP44 (on LG 3) and explained 10.3% of the trait variation. Also, for traits atk_2000, sum_atk, egg_2000 as well as for egg_2001 pQTLs explaining a higher portion of phenotypic variance (4.6–7.5%) mapped to this locus. Along the entire linkage map, most eQTLs mapped to this locus (2040 transcripts). At two loci, SNP74 (LG4) and SNP252 (LG13), the pQTLs from the same five traits (hgt_1997, hgt_1999, ldr_99, atk_2000 and egg_2000) were associated with the individual eQTL hotspots. On LG6, pQTLs from ldr_99, atk_2000 and sum_egg, while on LG11, pQTLs from hgt_1999, ldr_99, atk_2000, sum_atk and egg_2000 associated with the eQTL hotspot. At SNP210 related to ubiquitin conjugating enzyme 2 (LG11), the identified eQTL hotspot was associated with three resistance and two growth traits. In all four cases of eQTL hotspots where several pQTLs for growth and resistance traits collocated, the allelic effects of the pQTls for growth and resistance traits had the same sign. This indicates a positive correlation between the growth trait variation (as assessed by height data) and the resistance trait variation (from attack rates and oviposition data).

### Positional Candidate Genes for Resistance and Growth Trait Variation

The combination of phenotype and gene expression datasets facilitates studying the genetic control of phenotypic traits of interest [30]. “Positional” candidate genes can be identified as genes for which transcript abundance correlates extensively with the quantitative phenotype.

Here, the positional candidate genes were identified by collocation of at least 40% of their eQTLs with pQTLs based on the criteria for identifying significant QTLs and running 10,000 randomizations (p ≤ 0.05), see Material and Methods. Thus, extensive co-segregation of transcript variation with the phenotypic trait of interest identifies positional candidate genes that are directly underlying the trait. We arbitrarily defined map intervals of 10 cM to measure collocation (on average 2 SNPs were binned into 10 cM). Thus, within a resolution window of 10 cM at a given SNP locus we determined the significance of co-localizations between the expression variation that was detected at a certain EST that was spotted on the microarray (gene spot) and the phenotypic trait variation (p ≤ 0.05). We screened 21,840 transcripts that represented distinct ESTs spotted on the microarray for co-localization with growth traits and for co-localization with resistance traits, respectively, and identified 1621 and 2002 distinct ESTs, respectively. These numbers comprised of the following trait associations: ldr_99 (217 gene spots), hgt_1995 (254), hgt_1997 (385), hgt_1999 (878); atk_2000 (346), atk_2001 (311), sum_atk (546), egg_2000 (361), egg_2001 (335), sum_egg (584), respectively (**Table S4**). Not unusual for conifers, many of those spruce genes had no *Arabidopsis* homologues. In total, 1191 gene spots from co-localizations with resistance traits gave hits with unique *Arabidopsis* entries (59%); 1000 gene spots from co-localizations with growth traits had unique annotations (62%).

**Figure 4:**
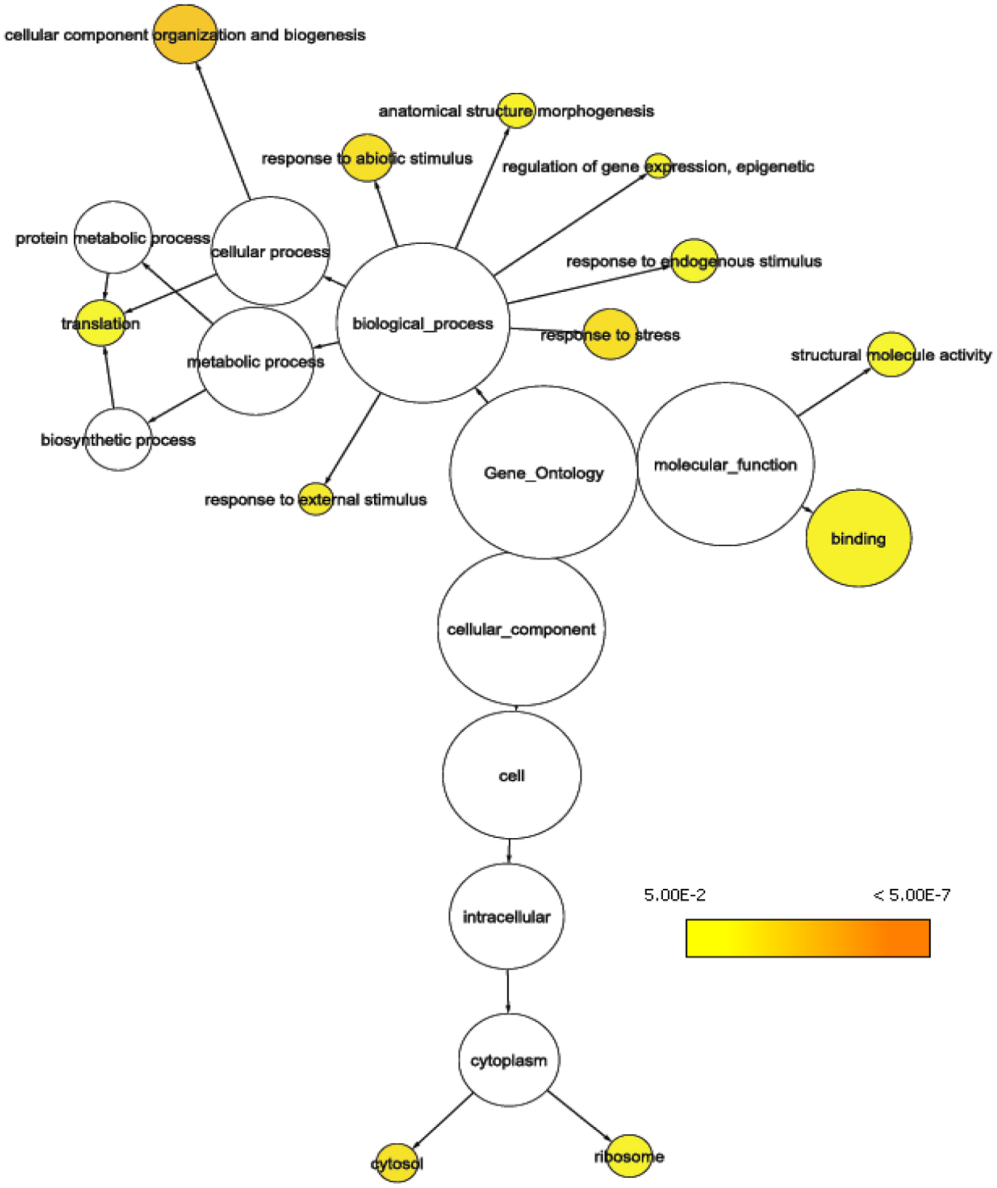
GO tree representation indicating significantly (p ≤ 0.05) overrepresented categories from colocalizations with resistance traits.

**Figure 5:**
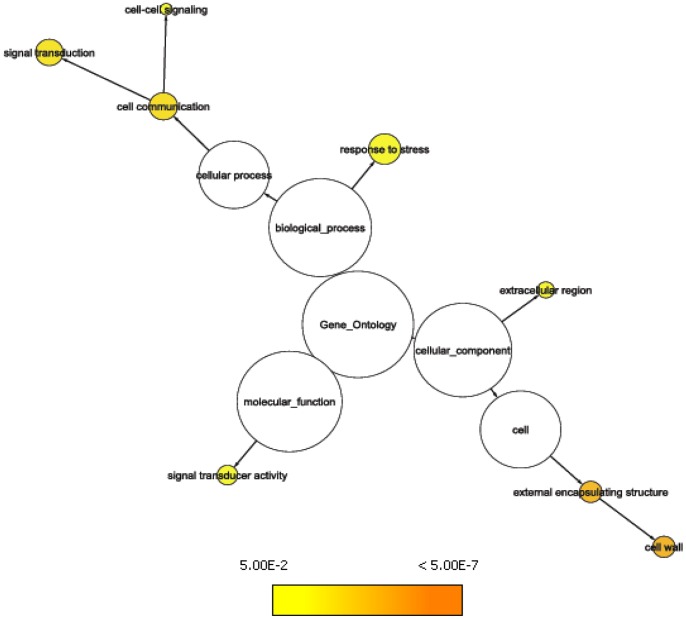
GO tree representation indicating significantly (p ≤ 0.05) overrepresented categories from colocalizations with growth traits.

We compared annotations independently for resistance and growth with the unique *Arabidopsis* annotations from the array. Twelve, and eight GOSlim Plants categories, respectively, were overrepresented among genes with expression variation significantly associated with individual phenotypic traits. The categories for resistance trait associations involved: ‘response to stress’ (64 compared to a total of 329 genes on the array), ‘response to abiotic stimulus’ (54/272), ‘cellular component organization and biogenesis’ (94/479) and ‘cytosol’ (32/145) as well as ‘binding’ (298/1908); **Figure 4** and **Table S5**. Growth trait categories were related to ‘extracellular region’ (8/32), ‘signal transducer activity’ (15/78), ‘cell communication’ (38/204), ‘response to stress’ (51/329) categories and the ‘cell wall’ category (19/63); **Figure 5** and **Table S5**.

In the gene lists a large number of kinases, phosphatases as well as transcription factors were identified; **Table S4**. Phosphorylation and dephosphorylation are important steps in various biosynthetic processes, and in signal transduction cascades within the organism. For resistance traits we counted 104, for growth traits 91 transcription (-related) factors/proteins. We identified 49 kinases associated with the growth traits, whereas 63 with the resistance traits. Phosphatases totaled to 22 candidate genes both for growth and for resistance traits, respectively.

Several multi-gene families were highly represented in both growth and resistance traits associations: among others GDSL-motif lipase/hydrolase, glycosyl hydrolase (GH), leucine-rich repeat (LRR) proteins, oxidoreductases, pentatricopeptide (PPR) repeat-containing proteins, disease resistance family proteins, DNAJ heat shock family proteins.

A number of auxin-related genes (including ABC transporters) co-localized with phenotypic trait variation: for resistance we found 19, for growth 15 co-localizing. A number of genes involved in embryo arrest/deficiency also co-localized with the phenotypes: 19 for resistance and 22 for growth traits. Jasmonic acid (JA)-forming and ethylene-forming/−responsive genes were also identified candidate genes based on collocations with the respective phenotypic traits.

The isoprenoid biosynthesis pathway generates many compounds relevant to plant defenses (terpenoids, tocopherol, e.g.) but also the precursors of ‘plant hormones’ like gibberellins (GA) and abscisic acid (ABA). Eight biosynthetic genes co-localized with resistance traits and six exclusively with the hgt_1999 trait. In addition, both for growth and resistance traits, four GA-regulated/GA signaling-regulating, and two ABA-related proteins were identified as potential candidates.

The phenylpropanoid metabolic pathway provides various specialized metabolites important in plant development, polymeric lignin for structural support, anthocyanins for pigmentation, flavonoids with various protective functions, and antimicrobial phytoalexins [31]. Thirty-seven putative gene family members associated with individual resistance traits, while 25 with growth traits. For the traits related to egg counts (26 transcripts) and to height in 1999 (15 transcripts) the highest number of candidates from this pathway was identified.

Genes that are significant for both resistance and growth traits (p ≤ 0.05; 10,000 randomizations, see Materials and Methods) are summarized in **Table S6** (see **Table S4** for comprehensive results). In sum, 244 genes were identified. The majority of these genes co-localized with Ht 1999, and also co-localized with atk_2000, egg_2000, respectively, resistance traits assessed in the next growth season. Since many identified genes have no angiosperm counterparts, they likely represent novel conifer-specific genes at the pivotal points of growth variation and defense. For one DNAJ heat shock family protein its eQTLs significantly co-localized with QTLs from as much as seven individual traits (**Table S6**). The functions of these chaperones are related to environmental challenges (involving stress tolerance) but are manifold due to the complexity of the whole gene family. Among the candidates in common between resistance and growth traits were genes involved in normal/optimal plant growth, stress signaling, defense, stress tolerance, glycine betaine synthesis, DNA repair, transcription regulation, post-transcriptional regulations, protein degradation as well as expansins with a proposed function related to cell wall architecture during rapid tissue expansion (**Table S6**).

Significant associations with all four individual growth and six individual resistance traits, respectively, allowed us to robustly identify 149, and 99 candidate genes for the composite ‘resistance’ and ‘growth’ phenotype, respectively (**Table S7** and **Table S8**). Gene identities with annotations are provided in **Table 2** and **Table 3**. For about half of the gene spots identified as candidates no putative functions were unraveled by BLAST searches. Candidate genes for ‘resistance’ and ‘growth’ differ markedly in their functions. While collocations of expression with growth variation were predominantly found for gene products involved in (post-) transcription and post translational regulation (in total 18), collocations with resistance variation identified a larger number of biosynthetic proteins (17), signaling (20) and transporter/transport related molecules (13). We found that 19 genes are positional candidates for the composite ‘resistance’ phenotype, but are additionally associated with other growth trait(s); these genes are typically involved in signaling, transport and biosynthesis related processes. The 29 genes that are positional candidates for the composite ‘growth’ phenotype and at the same time associated with individual resistance traits have proposed functions in transcriptional or (post-) translational control, growth and cell wall remodeling.

**Table 2:** Display of 80 positional candidate genes (AGI annotated) for the composite resistance phenotype, p ≤ 0.1.

Gene id	P-value	E-value	AGI #	Annotation	Putative function
WS0262_L21	0.051	2.9E–45	AT2G38240	oxidoreductase, 2OG-Fe(II) oxygenase family protein	biosynthesis
WS00922_A20	0.061	1.4E–54	AT3G06810	acyl-CoA dehydrogenase-related	biosynthesis
WS01033_F16	0.065	5.30E–023	AT3G03780	AtMS2 (Arabidopsis thaliana methionine synthase 2)	biosynthesis
WS00818_F07	0.074	1.20E–073	AT2G43710	FAB2, SSI2 SSI2 (fatty acid biosynthesis 2); acyl-[acyl-carrier-protein] desaturase	biosynthesis
WS00812_J14	0.082	7.80E–052	AT3G04520	THA2 (THREONINE ALDOLASE 2)	biosynthesis
WS00113_D16	0.082	2.80E–111	AT5G08370	ATAGAL2 (ARABIDOPSIS THALIANA ALPHA-GALACTOSIDASE 2)	biosynthesis
WS0043_N05	0.084	2.3E–111	AT3G17390	SAMS3, MAT4, MTO3 MTO3 (S-adenosylmethionine synthase 3); methionine adenosyltransferase	biosynthesis
WS0073_B10	0.086	1.40E–027	AT1G14550	anionic peroxidase, putative	biosynthesis
WS00932_K15	0.086	7.5E–43	AT4G37970	mannitol dehydrogenase, putative	biosynthesis
WS01016_H01	0.089	5.50E–109	AT5G60540	EMB2407, ATPDX2, PDX2 ATPDX2/EMB2407/PDX2 (PYRIDOXINE BIOSYNTHESIS 2)	biosynthesis
WS00816_E04	0.092	5.4E–30	AT1G08250	prephenate dehydratase family protein	biosynthesis
WS00725_B17	0.093	2.80E–054	AT5G42800	TT3, M318, DFR DFR (DIHYDROFLAVONOL 4-REDUCTASE); dihydrokaempferol 4-reductase	biosynthesis
WS0045_J16	0.094	1.90E–031	AT5G04330	cytochrome P450, putative/ferulate-5-hydroxylase, putative	biosynthesis
WS0094_G24	0.096	1.50E–126	AT5G17990	PAT1, TRP1 TRP1 (TRYPTOPHAN BIOSYNTHESIS 1); anthranilate phosphoribosyltransferase	biosynthesis
WS0023_B12	0.097	5.50E–074	AT1G23800	ALDH2B, ALDH2B7 ALDH2B7 (Aldehyde dehydrogenase 2B7)	biosynthesis
WS0107_F01	0.097	3.1E–22	AT4G37970	mannitol dehydrogenase, putative	biosynthesis
WS0076_F23	0.098	4.9E–36	AT5G19730	pectinesterase family protein	cell wall
WS0105_N22	0.100	2.60E–138	AT1G77380	AAP3 (amino acid permease 3); amino acid permease	transport
WS00721_A21	0.100	1.6E–76	AT1G77120	ADH, ATADH, ADH1 ADH1 (ALCOHOL DEHYDROGENASE 1)	biosynthesis
WS0044_N09	0.083	7.2E–10	AT1G60390	BURP domain-containing protein/polygalacturonase, putative	cell wall, stress(?)
WS0086_K19	0.097	6.70E–046	AT1G27120	galactosyltransferase family protein	cell wall
WS0092_M11	0.064	6.3E–39	AT2G30410	KIS (KIESEL); unfolded protein binding	growth
WS0061_B17	0.073	1.40E–055	AT2G21530	forkhead-associated domain-containing protein	growth, development
WS01031_N10	0.079	4.10E–049	AT2G04030	EMB1956, CR88 CR88 (EMBRYO DEFECTIVE 1956); ATP binding	growth
WS0261_O16	0.088	6.40E–164	AT5G67270	ATEB1C (MICROTUBULE END BINDING PROTEIN 1); microtubule binding	growth
WS01021_K16	0.088	5.90E–196	AT2G33150	PED1, KAT2 PED1 (PEROXISOME DEFECTIVE 1); acetyl-CoA C-acyltransferase	growth
WS0261_G19	0.088	4.7E–34	AT5G53940	yippee family protein	growth, stress
WS00813_E05	0.060	5.80E–102	AT4G09670	oxidoreductase family protein	miscellaneous
WS0017_K15	0.066	5.80E–006	AT5G40470	similar to F-box family protein (FBL4) [Arabidopsis thaliana]	miscellaneous
WS00728_E10	0.076	1.0E–20	AT5G02450	60S ribosomal protein L36 (RPL36C)	miscellaneous
WS00921_L16	0.077	2.5E–19	AT1G27620	transferase family protein	miscellaneous
WS00912_K11	0.080	2.1E–47	AT2G19680	mitochondrial ATP synthase g subunit family protein	miscellaneous
WS00927_K21	0.082	2.10E–026	AT3G53850	similar to integral membrane protein, putative [Arabidopsis thaliana]	miscellaneous
WS00712_A12	0.091	8.40E–084	AT1G60420	DC1 domain-containing protein	miscellaneous
WS0032_G24	0.095	3.90E–057	AT2G37270	ATRPS5B ATRPS5B (RIBOSOMAL PROTEIN 5B); structural constituent of ribosome	miscellaneous
WS0057_N15	0.096	1.60E–102	AT1G10780	F-box family protein	miscellaneous
WS01032_N12	0.098	1.10E–053	AT1G44910	protein binding	miscellaneous
WS0039_A22	0.099	1.60E–116	AT5G53490	thylakoid lumenal 17.4 kDa protein, chloroplast	miscellaneous
WS00932_M11	0.061	5.10E–006	AT5G01020	protein kinase family protein	signaling
WS01011_I05	0.062	2.4E–23	AT1G73500	ATMKK9 ATMKK9 (Arabidopsis thaliana MAP kinase kinase 9)	signaling
WS00924_L15	0.071	1.20E–070	AT1G79110	protein binding/zinc ion binding	signaling
IS0011_J12	0.072	6.3E–28	AT5G53590	auxin-responsive family protein	signaling
WS0048_K17	0.074	1.70E–086	AT1G60490	ATVPS34 ATVPS34 (Arabidopsis thaliana vacuolar protein sorting 34); phosphatidylinositol 3–kinase	signaling
WS00824_D10	0.081	5.10E–041	AT5G14930	GENE101, SAG101 SAG101 (SENESCENCE–ASSOCIATED GENE 101);triacylglycerol lipase	signaling, stress
WS0016_M07	0.084	5.90E–030	AT5G14930	GENE101, SAG101 SAG101 (SENESCENCE–ASSOCIATED GENE 101);triacylglycerol lipase	signaling, stress
WS00946_N19	0.088	1.00E–079	AT2G38010	ceramidase family protein	signaling, stress
WS00922_M10	0.090	1.30E–043	AT2G32800	AP4.3A AP4.3A; ATP binding/protein kinase	signaling
WS00928_C13	0.091	1.4E–36	AT2G33040	ATP synthase gamma chain, mitochondrial (ATPC)	signaling
WS0063_H05	0.091	7.90E–075	AT4G15415	serine/threonine protein phosphatase 2A (PP2A) regulatory subunit B' (B'gamma)	signaling
WS0063_N04	0.093	3.20E–039	AT1G16670	protein kinase family protein	signaling
WS00924_P23	0.093	2.6E–45	AT5G26751	ATSK11 (Arabidopsis thaliana SHAGGY-related kinase 11); protein kinase	signaling
WS0261_J01	0.094	2.0E–85	AT1G73500	ATMKK9 (Arabidopsis thaliana MAP kinase kinase 9)	signaling
WS0097_P15	0.096	6.10E–024	AT3G12690	protein kinase, putative	signaling
WS0268_O17	0.096	6.80E–037	AT3G22190	IQD5 IQD5 (IQ-domain 5); calmodulin binding	signaling
WS00930_C14	0.096	5.80E–161	AT3G50960	similar to Thioredoxin domain 2 [Medicago truncatula]	signaling
WS0089_G23	0.097	2.6E–38	AT1G66410	ACAM-4, CAM4 CAM4 (CALMODULIN 4); calcium ion binding	signaling
WS00111_O11	0.099	1.80E–087	AT2G30020	protein phosphatase 2C, putative/PP2C, putative	signaling
WS01041_M05	0.099	5.40E–083	AT2G30020	protein phosphatase 2C, putative/PP2C, putative	signaling
WS00922_P23	0.069	2.80E–110	AT5G01230	FtsJ-like methyltransferase family protein	stress
WS01021_F15	0.092	3.00E–133	AT3G62550	universal stress protein (USP) family protein	stress
WS0263_B07	0.044	9.0E–21	AT4G23330	eukaryotic translation initiation factor-related	transcriptional, translational
WS0061_C09	0.061	6.90E–013	AT4G20970	basic helix-loop-helix (bHLH) family protein	transcriptional
WS00924_K23	0.074	8.00E–050	AT3G49430	SRP34A SRP34A (SER/ARG-RICH PROTEIN 34A); RNA binding	transcriptional
WS0078_K12	0.077	2.3E–11	AT1G10200	transcription factor LIM, putative	transcriptional
WS0087_O15	0.078	7.00E–051	AT5G47390	myb family transcription factor	transcriptional
WS00916_N06	0.083	1.2E–17	AT5G06550	similar to transcription factor jumonji (jmjC) domain-containing protein [Arabidopsis thaliana]	transcriptional
WS00826_M02	0.083	6.60E–065	AT1G13690	ATE1 (ATPase E1); nucleic acid binding	transcriptional
WS00825_H14	0.087	1.6E–32	AT1G29250	nucleic acid binding	transcriptional
WS0107_C03	0.062	5.00E–025	AT3G07490	AGD11 (ARF-GAP DOMAIN 11); calcium ion binding	transport
WS01013_E24	0.076	4.20E–103	AT2G35800	mitochondrial substrate carrier family protein	transport
WS00927_L04	0.076	1.60E–129	AT5G19760	dicarboxylate/tricarboxylate carrier (DTC)	transport
WS00939_B16	0.076	1.60E–158	AT2G20930	similar to intracellular transporter [Arabidopsis thaliana]	transport
WS0071_O18	0.081	1.90E–127	AT1G73030	SNF7 family protein	transport
WS0017_I09	0.085	4.80E–091	AT4G04860	DER2.2 Der1-like family protein/degradation in the ER-like family protein	transport, stress
WS00919_L11	0.085	1.00E–159	AT1G72280	AERO1 AERO1 (ARABIDOPSIS ENDOPLASMIC RETICULUM OXIDOREDUCTINS 1)	transport
WS0081_G16	0.088	2.40E–096	AT5G46630	clathrin adaptor complexes medium subunit family protein	transport
WS0105_B03	0.090	1.80E–069	AT5G12130	PDE149 PDE149 (PIGMENT DEFECTIVE 149)	transport
WS01012_D02	0.092	1.70E–088	AT4G02050	sugar transporter, putative	transport
WS00712_P20	0.095	8.10E–105	AT3G48420	haloacid dehalogenase-like hydrolase family protein	transport
WS0106_H16	0.100	3.80E–023	AT1G30690	SEC14 cytosolic factor family protein/phosphoglyceride transfer family protein	transport

**Table 3:** Display of 49 positional candidate genes (AGI annotated) for the composite growth phenotype, p ≤ 0.1.

Gene id	P-value	E-value	AGI #	Annotation	Putative function
WS00924_B02	0.084	6.30E–101	AT2G24210	TPS10 (TERPENE SYNTHASE 10); myrcene/(E)-beta-ocimene synthase	biosynthesis
WS00923_K11	0.089	3.6E–83	AT5G03860	malate synthase, putative	biosynthesis
WS00924_G02	0.092	4.3E–85	AT4G35630	PSAT (phosphoserine aminotransferase); phosphoserine transaminase	biosynthesis
WS0079_D01	0.092	8.10E–160	AT3G54050	fructose-1,6-bisphosphatase, putative/D-fructose-1,6-bisphosphate 1-phosphohydrolase, putative	biosynthesis
WS00945_C13	0.108	1.30E–165	AT1G79500	AtkdsA1 (Arabidopsis thaliana KDO-8-phosphate synthase A1); 3-deoxy-8-phosphooctulonate synthase	biosynthesis
WS00821_F22	0.058	1.60E–108	AT1G32860	glycosyl hydrolase family 17 protein	cell wall, remodeling(?)
WS00818_M19	0.095	2.50E–085	AT1G26770	ATEXPA10 (ARABIDOPSIS THALIANA EXPANSIN A10)	cell wall
WS0014_E13	0.108	7.40E–089	AT2G37870	protease inhibitor/seed storage/lipid transfer protein (LTP) family protein	cell wall, remodeling(?)
WS0087_G23	0.071	2.90E–058	AT3G06930	protein arginine N-methyltransferase family protein	plant development
WS00819_F17	0.095	1.10E–031	AT4G27745	Identical to Protein yippee-like At4g27740 [Arabidopsis Thaliana]	growth
WS00712_K23	0.100	6.6E–25	AT1G28480	glutaredoxin family protein	growth, development
WS00922_F02	0.104	1.70E–151	AT5G62390	ATBAG7 (ARABIDOPSIS THALIANA BCL-2-ASSOCIATED ATHANOGENE 7); calmodulin binding	growth arrest
WS0084_L12	0.104	2.30E–087	AT3G61780	EMB1703 (EMBRYO DEFECTIVE 1703)	growth arrest
WS00716_E11	0.106	3.00E–146	AT4G26850	VTC2 (VITAMIN C DEFECTIVE 2)	growth related
WS0264_I07	0.108	3.90E–080	AT1G60170	EMB1220 (EMBRYO DEFECTIVE 1220)	growth arrest
WS0083_N10	0.055	8.40E–095	AT2G18360	hydrolase, alpha/beta fold family protein	miscellaneous
WS00821_F12	0.081	2.4E–42	AT3G07480	electron carrier/iron ion binding	miscellaneous
WS01037_M20	0.085	1.1E–30	ATMG00810	similar to protein kinase family protein [Arabidopsis thaliana]	miscellaneous
WS00728_D14	0.092	3.7E–41	AT4G34670	40S ribosomal protein S3A (RPS3aB)	miscellaneous
WS01034_K20	0.095	6.70E–009	AT4G19380	alcohol oxidase-related	miscellaneous
WS00815_F18	0.100	3.00E–072	AT2G19750	40S ribosomal protein S30 (RPS30A)	miscellaneous
WS0041_I12	0.101	3.8E–08	AT1G12810	proline-rich family protein	miscellaneous
WS00926_B01	0.103	9.4E–57	AT4G18100	60S ribosomal protein L32 (RPL32A)	miscellaneous
WS0056_L17	0.104	1.20E–114	AT1G66530	arginyl-tRNA synthetase, putative/arginine–tRNA ligase, putative	miscellaneous
WS0097_I03	0.108	8.9E–08	AT5G54600	50S ribosomal protein L24, chloroplast (CL24)	miscellaneous
WS00819_E15	0.109	8.60E–053	AT4G38250	amino acid transporter family protein	miscellaneous
WS00112_E05	0.037	2.50E–062	AT2G22360	DNAJ heat shock family protein	posttranslational
WS01025_F14	0.067	1.60E–181	AT3G07780	protein binding/zinc ion binding	posttranslational
WS0011_I04	0.074	4.10E–010	AT3G54850	armadillo/beta-catenin repeat family protein/U-box domain-containingfamily protein	posttranslational
WS0047_F24	0.089	6.4E–24	AT3G06130	heavy-metal-associated domain-containing protein	posttranslational
WS00733_J11	0.090	9.6E–44	AT1G75690	chaperone protein dnaJ-related	posttranslational
WS0024_O12	0.093	5.30E–122	AT5G45390	NCLPP3, NCLPP4, CLPP4 | CLPP4 (Clp protease proteolytic subunit 4)	posttranslational
WS01024_O16	0.103	4.9E–64	AT1G77460	C2 domain-containing protein/armadillo/beta-catenin repeat family protein	posttranslational
WS00815_B15	0.109	2.7E–19	AT1G01490	heavy-metal-associated domain-containing protein	posttranslational
WS0089_E10	0.102	6.50E–043	AT2G46225	ABI1L1 (ABI-1-LIKE 1)	signaling
WS00919_K24	0.104	9.20E–119	AT3G59520	rhomboid family protein	signaling
WS0104_D02	0.107	7.4E–16	AT1G61370	S-locus lectin protein kinase family protein	signaling
WS00928_J07	0.078	3.90E–152	AT1G17440	transcription initiation factor IID (TFIID) subunit A family protein	transcriptional/posttranscriptional
WS00912_K01	0.089	1.70E–019	AT2G41900	zinc finger (CCCH-type) family protein	transcriptional/posttranscriptional
WS00917_G03	0.091	3.90E–120	AT1G01350	zinc finger (CCCH-type/C3HC4-type RING finger) family protein	transcriptional/posttranscriptional
WS0097_H22	0.092	1.7E–30	AT4G25500	ATRSP35 (Arabidopsis thaliana arginine/serine-rich splicing factor 35)	transcriptional/posttranscriptional
WS00910_O08	0.096	7.00E–048	AT5G08390	similar to transducin family protein/WD-40 repeat family protein [Arabidopsis thaliana]	transcriptional/posttranscriptional
WS00815_A12	0.099	7.20E–120	AT1G20110	zinc finger (FYVE type) family protein	transcriptional/posttranscriptional
WS01031_K02	0.102	1.80E–139	AT3G26935	zinc finger (DHHC type) family protein	transcriptional/posttranscriptional
WS0261_F02	0.103	5.50E–089	AT3G10760	myb family transcription factor	transcriptional/posttranscriptional
WS00922_N21	0.106	1.0E–23	AT3G28917	MIF2 (MINI ZINC FINGER 2); DNA binding	transcriptional/posttranscriptional
WS0099_L07	0.106	2.70E–104	AT2G27110	FRS3 (FAR1-RELATED SEQUENCE 3); zinc ion binding	transcriptional/posttranscriptional
IS0014_L17	0.086	6.40E–100	AT2G21600	ATRER1B (Arabidopsis thaliana endoplasmatic reticulum retrieval protein 1B)	transport, vesicle trafficking
WS00917_J14	0.097	1.10E–020	AT1G33475	Identical to Probable VAMP-like protein At1g33485 [Arabidopsis Thaliana]	transport, vesicle trafficking

### Correlations between Co-localization Estimates

We determined genetic (QTL) correlations based on co-localization estimates between gene expression and trait variation, **Table 1**. The correlation between the general ‘growth’ and ’resistance’ trait based on associated expression variation of transcripts was significant (R = 0.251). However, the positional candidates for the general ‘growth’ and ‘resistance’ phenotypes were distinct. Co-localization estimates for hgt_1997, ldr_99 and hgt_1999, respectively, correlated with co-localization estimates for atk_2000, egg_2000, sum_atk as well as sum_egg traits. This means that a significant fraction of their eQTLs co-localize with both growth and resistance QTLs. Overall, 12% of the genes that were positional candidates for individual height growth traits were also positional candidates for individual resistance traits, and 15% of the genes that were positional candidates for resistance traits were also positional candidates for height growth traits.

## Discussion

Our work on spruce weevil resistance follows similar work on eucalyptus [32] and poplar ([33], [34]). Ours is the first study of expression QTLs for resistance in a conifer. Despite the enormous genome size of conifers (ca. 20 billion base pairs) studies on the transcriptome of conifers are just as manageable as those with angiosperms with much smaller genome sizes. Here we utilized a third generation microarray spotted with 21,840 spruce ESTs in combination with a multiplexed genotyping approach to examine expression QTLs involved with weevil resistance and height growth. This work represents an extended study of [29] that previously focused in detail on the phenylpropanoid pathway and related genes with respect to pest resistance in spruce.

In our experiment, we harvested plant material in spring at the optimal time point at which early seasonal growth and natural onset of weevil attacks coincided. Our microarray consisted of 70% cDNA from untreated tissue of many tissue types; no overrepresentation of specific metabolic pathways was attempted. The issue of cross-hybridizations in microarray experiments due to high nucleotide similarity [35], is reduced in genetical genomics because of the randomized genetic background, high sample size and the statistical procedures. Cross-species comparisons of QTL are also possible based on the white spruce/loblolly pine comparative mapping project (K. Ritland, pers. comm.). Linkage groups one to twelve (LG1-12) were assigned following [36] to facilitate comparisons within the family of *Pinaceae*.

The main focus of the present work was scanning the genome for transcripts whose abundance correlated with the quantitative phenotype in order to identify transcripts associated with the phenotype ([37]). We assigned groups of genes that significantly associated with individual resistance and growth traits, respectively, into functional, cellular component, and biological process GO categories. These genes were co-regulated and likely have combined functions in the studied phenotypes. Thus, the present work elucidates functional associations among genes and provides a comprehensive study to the evolution of transcription regulation in spruce. Overall we found that a significant fraction of eQTLs were in common between the general ‘growth’ and ‘resistance’ phenotypes. This result was based on expression variation from all studied transcripts. This provides evidence for genetic pleiotropy of resistance and growth traits in interior spruce. In terms of directionality of gene expression with phenotypic trait variation, we found that the mean of correlations between transcript expression and quantitative traits was zero, however overall we found a wide distribution of correlations where some genes showed a clear positive, whereas others showed a clear negative correlation with traits (K. Ritland, personal communication).

### Contribution from Single Gene QTL

From the myriad of candidate genes that were identified for the individual resistance traits (**Table S4**; **Figure 4**), our study also identified an array of single genes that were associated with both resistance and growth phenotype (**Table S6** and **Table S4**). The identification of shared candidates suggests that several of the general functionalities (notably, the signaling systems, [38]) important to normal plant development are also adopted for defense mechanisms. Among those ‘pleiotropic’ genes many had functionalities that prevalently involve signaling, transcription factor activity, functions in transcription/translation (including RNA editing), stress/stimulus response, as well as transport and cell wall functions. Transcription regulators have been suggested to be key targets for plant evolution ([39], [40]). Their high representation in our gene lists reinforces their broad importance to the sessile organism’s potential to optimize growth under the given environmental conditions.

### Multigene Family QTL Contributions

Large multi-gene families were represented in the associations with both growth and resistance traits. These involved GDSL-motif lipase/hydrolase, GHs, LRR proteins, oxidoreductases, PPR proteins, disease resistance proteins, and DNAJ heat shock family proteins. While GDSL, LRR, PPR and DNAJ proteins were equally represented among growth and resistance candidate genes, respectively, GH, oxidoreductases, and disease resistance proteins contributed twice as many resistance candidates than growth candidates. From this diverse group of disease resistance genes, two spruce sequences with similarity to f-family dirigent proteins that are implicated in constitutive resistance [41] were identified as candidates for weevil resistance. Several individual members were directly associated with both phenotypes and hence pleiotropic (one member in each case for GDSL-motif lipase/hydrolase, disease resistance proteins, and DNAJ heat shock family proteins; two each for GHs, and oxidoreductases; four each for LRR proteins, and PPR proteins). The spruce DNAJ heat shock protein co-localized with expression variation of seven individual traits (both resistance and growth), **Table S6**. Most of these candidates have previously been reported to be involved in defense reactions, some with proposed antifungal properties [42]–[47].

### Dominant Themes among Gene Functions Associated with the Resistance Phenotype

The statistically overrepresented ontology categories among genes that were associated with resistance phenotypic traits revealed dominant themes in gene expression that involved response to all sorts of stimuli (biotic, abiotic, external, and endogenous), epigenetic gene expression, and translation (ribosome) for the *de novo* generation of gene products. Additionally, remodeling processes that involve the (re-) organization of cellular components, and anatomical structures by proteins that provide binding functions and other structural molecule activities are important components of defense (**Figure 4**). Interestingly, signaling is the most common molecular function and cellular process among growth phenotype associated genes (**Figure 5**). For genes associated with the composite weevil resistance phenotype, functions in signaling were also predominant (**Table 2**). There is evidence that signaling pathways in defense reactions are co-opted from normal developmental processes, see also above. Genes that have signaling functions and are negative regulators of ABA responsiveness [48] were identified (phosphatase 2C protein for resistance, while ABI-1-LIKE 1 for growth, **Table 2** and **Table 3**). It is assumed that these gene functions allow for the fine-tuning of stress responses. We could also identify an ATP synthase (**Table 2**). Recently, it was shown that the initiation of multiple defense elicitors in the host is triggered by herbivore proteolysis of a plant ATP synthase [49].

Biosynthesis is also an important function for genes associated with the composite resistance phenotype (**Table 2**). Two genes annotated as mannitol dehydrogenase were identified. Mannitol dehydrogenase counteracts the fungal suppression of the reactive oxygen species that are generated during host defenses [50]. Furthermore, several genes linked to the general phenylpropanoid pathway, ([32], [31], [51]) and to flavonoid and isoflavonoid biosynthesis [52] were positional candidates for the general resistance trait.

### Significance of Phenylpropanoid vs. Terpenoid QTL for Constitutive Resistance

The observed eQTLs were the result of constitutive differences in gene expression. The importance of polyphenolics for constitutive defenses is reflected in a higher number of gene family members of the phenylpropanoid pathway (by homology to *A. thaliana* genes) whose eQTL co-localized with resistance QTLs. In this work, seven genes related to phenolics or flavonoid biosynthesis were positional candidates for the general resistance trait (**Table 2**). In contrast, no candidate gene for the resistance trait *per se* could be identified from the terpenoid pathway. Hence, we feel that this might reflect the higher importance of the phenolics over the terpenoid pathway in established resistance against this herbivore. We have previously suggested that monolignol formation may play an important role in defense reactions against the stem borer *Pissodes strobi* [29]. Based on in-depth analysis of genes involved in the shikimate pathway, monolignol biosynthesis and downstream condensation reactions as well as lignan formation with respect to weevil resistance, we further conclude that gene family members that were duplicated in spruce may have acquired temporally and spatially diverse functions in defense [29].

### Trans-eQTL Hotspots and their Significance

Certain phenotypes may be affected by gene expression regulators located within eQTL hotspots [30]. Although several previously conducted eQTL studies suggest that *cis*-eQTLs might have a greater effect on the phenotype than *trans*-eQTLs [53], *trans*-eQTLs are important for our understanding of the complexity of phenotypes [54]. For example, by comparing the levels of *trans*-eQTLs for each gene the global regulatory hierarchy can be assessed [53]. While *cis*-eQTLs are physically linked to the causative locus of the phenotype, *trans*-eQTLs can identify many downstream genes and reveal unknown pathways. In our study, we were mainly limited to the detection of *trans*-eQTLs, since the majority of SNP loci on our genetic linkage map could not be annotated [29]. This limitation is due to the fact that we were working with a non-model species and in particular with a conifer of immense genome size for which the genome sequencing has yet to be completed (http://www.congenie.org/).

Several trait-associated SNPs that were enriched for *trans*-eQTLs were identified in our study. At seven map positions, hotspots of expression variation coincided with QTLs from multiple resistance traits (LG3, LG4, LG6 and LG8). At eight SNP positions at least four pQTLs overlapped with eQTL hotspots. On four spruce linkage groups (LG4, LG6, LG11 and LG13) hotspots of expression variation associated with QTLs from both growth and resistance traits (**Figure 1**). This indicates *gene expression regulators* [30].

For example, on LG13 the two SNP loci underlying extensive expression and growth variation (i.e. large number of mapped QTLs) are derived from GAD enzymes whose activity regulation is vital for normal plant development. This allows response to external stimuli [55]. In addition, the enzyme may also function in a host deterrence reaction towards herbivore attack [56]. The eQTL hotspot on LG11 was associated with three resistance traits and two growth traits. The SNP that is located within the eQTL hotspot region is a gene that plays an important role within the ubiquitin/proteasome system, regulating developmental processes in plants, but also involved in biotic defense responses [57]. SNP markers derived from two different contigs Contig_4096_434 and CCoAOMT_1_320, respectively, clustered on LG6 and represent two of the three lignin-forming CCoAOMT genes in spruce [29]. We identified *cis*-eQTLs for these genes as well as *trans*-eQTLs generated from a multitude of genes that mapped to the two loci. Both loci are also hotspots of weevil resistance QTLs. A GO analysis of the transcripts associated with the two *trans*-eQTL hotspots revealed significant over-representation of several molecular function, cellular component, and biological process GO categories. The fact that 36% and 53%, respectively, of the mapped *trans*-eQTLs were in common between CCoAOMT-1 and CCoAOMT-2 suggests extensive interactions between both CCoAOMT loci via eQTLs from a multitude of genes. This was also reflected by common GO categories that were overrepresented such as ‘secondary metabolic process’ and ‘response to biotic stimulus’ in both gene expression networks centered at these two SNPs (**Figure 2** & **Figure 3**). Thus, this demonstrates how epistasis between gene loci works at the transcriptional level by linking *cis*-eQTLs via *trans*-regulatory interactions. In the case of CCoAOMT-1 and CCoAOMT-2, three resistance pQTLs also contributed to this epistatic interaction. CCoAOMT-1 and CCoAOMT-2 represent both metabolic pathway–specific *trans*-eQTL hotspots [29] and based on the present study, they represent important global *trans*-eQTL hotspots that are of interest for pest resistance in spruce.

### Jasmonate Signaling and its Central Role in Defense

We also identified JAZ genes that may play a role in the gene-gene interaction network between both CCoAOMT loci. JAZ genes were identified as central regulators of JA-mediated anti-insect defense [58]. Three JAZ genes were candidate genes directly associated with phenotypic trait variation. JA signaling is activated by repressor (i. e. JAZ) removal from the ubiquitin ligase complex [59]. Carbonic anhydrase genes are other important genes that have roles in jasmonate signaling and also in ethylene signaling [60], and in our study these genes mapped *trans*-eQTLs to both CCoAOMT hotspot locations. A previous study found that carbonic anhydrase genes were induced in spruce under stress treatments (budworm, weevil feeding, and mechanical wounding) [35]. At the second CCoAOMT locus on LG6, eQTLs from ERFs and specifically those from group IX [61] that represent known transcription repressors (ERF3, ERF4, ERF7) [62] were found.

In our study, transcriptional activators related to ethylene response (ERF-1, ERF-2, EIN5, [63]) as well as regulators for ethylene biosynthesis *per se* (RUB1, RUB1-conjugating enzyme, [64]) were found to co-localize exclusively with resistance traits. The hormonal cross-talks with JA (involving salicylic acid, ethylene, abscisic acid, and auxin) during growth and development as well as during adaptation to stress are highly complex [59], [65]. In *Arabidopsis*, the major players in JA-mediated plant defense are tightly linked [66]. The differential regulation of certain components/steps in the pathway is expected to generate distinct responses to different stimuli (reproductive development, growth or defenses, [59], [66]). Thus, establishing and maintaining defenses involves signaling systems that are co-opted from developmental processes [38].

### Conclusions

Although genomics studies on forest trees have traditionally focused on wood attributes [67]–[70], the genomics of environmental challenges has recently gained importance [34], [71]. Biotic stressors, herbivores and their accompanying pathogens pose an increased threat to tree populations, and knowledge of the genomic architecture can inform the management and breeding practices of conifers, as well as increase our general understanding of the evolution and adaptation of conifer species. Our result will add to this second vary important layer of genomics in forestry.

We have utilized expression QTL mapping to identify candidate genes. This will facilitate targeted association studies to further understand the genetic basis of host resistance to pests, the genomic basis of pest resistance. These results will enable both further functional studies to the nature of insect resistance in spruce, and provide valuable information about candidate genes for genetic improvement of spruce.

We identified several master regulators that underlie the genetic pleiotropy of pest resistance and developmental processes. Several candidate genes from the JA signaling pathway were identified for which we could show that central regulators of this pathway are contributing to extensive gene-gene interaction networks. Plant JA signaling provides a rapid response to various external stimuli [72] and is central to all biotic stress responses that directly influence the performance of the pest or contribute indirect defense responses to attract predators or herbivore parasitoids [73]. Importantly, this signaling pathway is not defense specific, but co-opted from normal developmental processes such as reproductive development [72]. In this way, the induction of defenses against herbivores or pathogens remains highly cost effective [38].

This work identified several pleiotropic genes as candidate genes whose proposed functions are important in stress response or disease resistance. In addition, our study revealed the presence of master genes which influence the global transcriptome. These genes are in “hotspots”, sometimes linked to annotated loci which were in turn further annotated to developmental and defense associated processes. Since resistance and growth QTLs overlapped with eQTL hotspots along the genome, this suggests that: 1) genetic pleiotropy of resistance and growth traits in interior spruce was substantial, and 2) master regulatory genes were important for weevil resistance in spruce. Knowledge about the exact function of these master regulons in the conifer genome needs further investigation; however knock-out mutants for largely pleiotropic genes were shown to be largely lethal or exhibit highly deleterious phenotypes [53].

## Materials and Methods

### Interior Spruce Pedigree

Experimental interior spruce populations originated from a controlled-cross progeny trial established in 1995 at Kalamalka Research Station in Vernon, BC, Canada [74]. The parental trees were selected from individuals previously ranked for weevil-resistance in open-pollinated progeny tests [14]. Out of twenty crosses segregating for weevil-resistance [74], four families with wide segregation for weevil resistance arranged as 2x2 factorial were harvested in May 2006 for gene expression profiling: cross 26 (♀PG87*♂PG165), cross 27 (♀PG87*♂PG117), cross 29 (♀PG21*♂PG165) and cross 32 (♀PG21*♂PG117). From 417 offspring of a 3x2 factorial (including the additional crosses 22 and 25), genomic DNA was isolated from flushing bud/needle tissue according to the cetyltrimethylammonium bromide (CTAB) method [75]. The studied trees represented individual genotypes that were planted in randomized plots within three replicate blocks in the field [74]. A detailed layout of the study site that shows the randomized location of plots for the QTL mapping families PG87*PG165 (cross 26), PG87*PG117 (cross 27), PG21*PG165 (cross29) and PG21*PG117 (cross32) within the replicate blocks can be found in [29].

A set of 384 SNPs were identified *in silico* from a collection of ESTs from the Treenomix EST database (K. Ritland pers.comm.) that were all derived from a single tree (PG 29). The genomic DNA was then genotyped for these SNPs using the multiplexed Illumina platform at the CMMT Genotyping and Gene Expression Core Facility, Centre for Molecular Medicine and Therapeutics, Vancouver, BC. Recombination rates were determined by joint likelihood [76] for each pair of loci and a consensus genetic map of 252 SNPs was constructed using JoinMap 3.0 [77], see [29] for details. Of all putative SNP loci, 73.4–76.0% were confirmed and included in the analysis; 394 individuals were true full-sibs. Those that could not be confirmed as full-sibs in the respective crosses (cross 26: 7%, cross 27: 10%, cross 29: 4%, and cross 32: 1% of the trees alive in 2006) were removed before phenotyping. The majority of spruce gene markers (i.e. the SNPs) that were used to build the framework map could not be annotated. This involved 67% of the ESTs when we used the TAIR7 database, while 54% of the ESTs when we used Viridiplantae databases [29].

### Measures for Tree Height, Weevil Attack and Oviposition

The trial was screened for resistance to terminal leader weevil following the method described by [20]. In short, a population of weevils was raised in summer 1999 at the Canadian Forest Service (Pacific Forestry Centre), Victoria and released onto all test trees in fall 1999. Attack rates, egg counts and top kills were recorded in 2000–2004. Growth measurements included the initial tree height in 1995 (year one), and heights in years three, and five as well as leader length in year five preceding the artificial augmentation of the local weevil population in October of the same year (hgt_1995, hgt_1997, hgt_1999, and ldr_99, respectively). Attack rates in 2000 and 2001 (atk_2000, atk_2001) were classified as successful ‘top kills’, ‘failure’ to kill the leader and ‘no attack’ [74]. In addition, for the same years oviposition on the leaders was recorded (egg_2000 and egg_2001) by counting egg punctures into five discrete classes: 1 = 1–25, 2 = 26–50, 3 = 51–75, 4 = 76–100, 5 = 101 and more. Egg punctures contain egg covering fecal plugs and are easily distinguished from feeding punctures, which are not covered. The sums of weevil attacks and oviposition for 2000 and 2001 were also used as ‘resistance’ traits (sum_atk and sum_egg).

### Tissue Collection, RNA Preparation, Microarray, Gene Expression Profiling

Tree material within a replicate block was sampled in a randomized fashion among the plots (i.e. crosses). Terminal leaders from trees in a block were collected in the mornings of May 16, 17 and 18, 2006, respectively. The weather was consistent among these days. Bark/phloem tissue was immediately harvested on site from cut leaders as described previously ([35]; [78]), flash frozen in liquid nitrogen, and stored at −80°C until processed. Total RNA from unattacked individuals was isolated following the protocol of [79] and quantified using NanoDrop® ND-1000 Spectrophotometer; RNA integrity was evaluated using the Agilent 2100 Bioanalyzer. The 21,840 spruce ESTs on the array involved elements from 12 different cDNA libraries, built from different tissues (bark, phloem, xylem), which were under different developmental stages, as well as wound/methyljasmonate treated (ca. 6,500 elements) and untreated (ca. 15,400). Complete details of cDNA microarray fabrication and quality control are described elsewhere (S. Ralph and co-workers, Gene Expression Omnibus database GEO: GPL5423 and http://www.treenomix.ca/). Labeling reactions, hybridizations, slide washes as well as scanning of slides were carried out as described in [35]. Fluorescent intensity data were extracted by using the ImaGene 6.0 software (Biodiscovery, El Segundo, USA). Signal intensity measurements were deposited in the Gene Expression Omnibus database under the accession number GSE22116.

### Microarray Experimental Design and Pre-processing of Expression Data

Our experimental design is based on *a priori* known genotypes. Testing six genotyped crosses and using the previously collected phenotypic data (see above), we determined that genotype differences between most and least resistant progeny were highest in crosses 26, 27, 29 and 32. Since we used two-color microarrays, direct comparisons between Cy3-Cy5 labeled samples were required. A distant pair design for microarray analysis that maximized direct comparisons between different alleles at each locus was originally introduced by [80] and was modified in our study for outbred individuals. We estimated the genetic distance for possible probe-pairs genome-wide by using all segregating SNP loci (122 on average) and such we maximized the number of distant pairs in a given cross. A 25% improvement over random pairing was achieved. We also balanced the two dyes across the three replicate blocks (i.e. sampling on three different days), the different batches of microarray fabrication and different experimenters (see below). Our design resulted in 94 hybridizations profiling 48 individuals in cross 26, 36 in cross 27, and 50 in cross 29 as well as 54 individuals in cross 32 [29].

After quantification of the signal intensities in each array the local background was subtracted for each subgrid. Data were normalized to compensate for non-linearity of intensity distributions using the variance stabilizing normalization method [81]. We performed a single normalization of 188 columns of data. In this way each channel had a similar and array-independent overall expression level and variance. Signal intensities are deposited under the GEO accession number GSE22116. The linear model 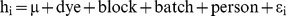



with *μ* as the overall mean was then fit to the normalized intensities of each gene i (*h_i_*) in the Cy3 and Cy5 channels to account for technical effects within the experiment (gene-specific ‘dye’ effect, replicate ‘block’, microarray fabrication ‘batch’, experimenter ‘person’ are all fixed effects). The residuals were used in the subsequent QTL analysis. All of the above statistics was carried out using the R statistical package (www.r-project.org).

### QTL Detection

A program was written in FORTRAN by K. Ritland for QTL mapping in the 3×2 factorial (for resistance and growth traits) and 2x2 factorial design (for gene expression traits). This program inferred QTL maps for each of the parents of the factorial. QTLs were mapped in the progeny by employing a likelihood function of the trait level (gene expression, other traits) conditioned on genotype of progeny, and compared to the likelihood of unconditioned genotype of progeny (no association of traits with progeny genotype) to give a log-odds (LOD) ratio. Due to the large number of gene expression traits, a single-marker model instead of an interval mapping approach was used, and QTLs were binned into 10 cM marker intervals, thus avoiding having two QTLs assigned to adjacent markers due to linkage of two markers to one QTL. We used R (www.r-project.org) to display QTL density maps. A QTL was significant at LOD ≥3.84 and had to be detected for a minimum of one parent in the factorial ([29], and **Table S1**). A goodness-of-fit test assuming a uniform distribution was performed to test whether the observed frequencies of eQTLs along the linkage map differed significantly from the expected value. Following the rejection of this null hypothesis ( χ^2^ = 96678, df = 251, p-value <2.2e-16), we declared “eQTL hotspots” if the number of eQTLs at a given locus exceeded the expected average by 50%. These numbers are significantly above the maximum number within eQTL clusters (i.e. 630) from a randomly generated data set using all 132,100 detected eQTLs, 252 markers, and running 1,000 replicates. The positional candidate genes were identified by collocation of at least 40% of their eQTLs with phenotypic trait QTLs based on the criteria for identifying significant QTLs (see above) and running 10,000 randomizations (p ≤ 0.05).

### Other Statistical Analyses

Phenotypic trait correlations were determined using SAS/STAT software, version 9.1.3 of the SAS system for Windows® (SAS Institute Inc., Cary, NC, USA). The cytoscape 2.5.1 plug-in BINGO [82] was used and a hypergeometric test was performed to determine statistically overrepresented Gene Ontology (GO) terms within the GOSlim Plants ontology for spruce genes with *Arabidopsis* homologs. In our case, this involved comparing the nearest Arabidopsis homologs for all genes that showed significant association of their expression variation with the previously assessed phenotypic trait variation (tree height, weevil attack, and oviposition) to all Arabidopsis homologs on the microarray.

## Supporting Information

Figure S1 **GO tree representation showing significantly (p ≤ 0.05) overrepresented GO categories within the** ***trans*** **eQTL-hotspot at the carbonic anhydrase gene locus contig_2079_440 (803 eQTLs) on LG4, for color code see** **Figures 2** **,** **3** **,** **4** **and** **5** **in main text.**
(TIFF)

Figure S2 **GO tree representation showing significantly (p ≤ 0.05) overrepresented GO categories within the** ***trans*** **eQTL-hotspot at the carbonic anhydrase gene locus contig_103_602 (1122 eQTLs) on LG4, for color code see** **Figures 2** **,** **3** **,** **4** **and** **5** **in main text.**
(TIFF)

File S1 **Comprehensive list of all 132,100 significant eQTLs (legends can be found in Table S1).**
(PDF)

Table S1 **Significant QTLs for gene expression (LOD ≥3.84); allele effect, and % phenotypic variation explained by QTL are given in File S1.**
(XLS)

Table S2 **Comprehensive list of eQTLs with annotations at locus Contig_4096_434 (see also** **Figure 2** **).**
(XLS)

Table S3 **Comprehensive list of eQTLs with annotations at locus CCoAOMT_1_320 (see also** **Figure 3** **).**
(XLS)

Table S4 **Comprehensive results for collocation estimations, with p-values.**
(XLS)

Table S5 **Statistically overrepresented Gene Ontology terms in the GOSlim Plant ontology for genes with expression variation co-localizing with resistance and growth traits, respectively, as presented in** **Figure 4** **and** **Figure 5** **.**
(XLS)

Table S6 **Display of genes that are candidates for different resistance and growth traits (p≤0.05), for at least three phenotypic traits (ldr_99, hgt_1995, hgt_1997, hgt_1999, atk_2000, atk_2001, sum_atk, egg_2000, egg_2001, and sum_egg, respectively).**
(XLS)

Table S7 **Complete list of the identified 149 positional candidate genes for the general resistance trait, (p ≤ 0.1).**
(XLS)

Table S8 **Complete list of identified 99 positional candidate genes for the general growth trait, (p ≤ 0.1).**
(XLS)

## References

[1] Dicke M (2000) Chemical ecology of host-plant selection by herbivorous arthropods: a multitrophic perspective. Biochemical Systematics and Ecology 28: 601–617. 10854737 10.1016/s0305-1978(99)00106-4

[2] Cornell HV, Hawkins BA (2003) Herbivore responses to plant secondary compounds: A test of phytochemical coevolution theory. American Naturalist 161: 507–522. 10.1086/368346 12776881

[3] Strauss SY, Rudgers JA, Lau JA, Irwin RE (2002) Direct and ecological costs of resistance to herbivory. Trends in Ecology & Evolution 17: 278–285.

[4] Zangerl AR, Arntz AM, Berenbaum MR (1997) Physiological price of an induced chemical defense: Photosynthesis, respiration, biosynthesis, and growth. Oecologia 109: 433–441. 28307541 10.1007/s004420050103

[5] Bergelson J, Purrington CB (1996) Surveying patterns in the cost of resistance in plants. American Naturalist 148: 536–558.

[6] Mole S (1994) Tradeoffs and constraints in plant-herbivore defense theory - a life history perspective. Oikos 71: 3–12.

[7] Herms DA, Mattson WJ (1992) The dilemma of plants - to grow or defend. Quarterly Review of Biology 67: 478–478.

[8] Roff DA, Fairbairn DJ (2007) The evolution of trade-offs: where are we? Journal of Evolutionary Biology 20: 433–447. 17305809 10.1111/j.1420-9101.2006.01255.x

[9] Worley AC, Houle D, Barrett SCH (2003) Consequences of hierarchical allocation for the evolution of life-history traits. American Naturalist 161: 153–167. 10.1086/345461 12650469

[10] Koricheva J, Nykanen H, Gianoli E (2004) Meta-analysis of trade-offs among plant antiherbivore defenses: Are plants jacks-of-all-trades, masters of all? American Naturalist 163: E64–E75. 10.1086/382601 15122510

[11] Kempel A, Schaedler M, Chrobock T, Fischer M, van Kleunen M (2011) Tradeoffs associated with constitutive and induced plant resistance against herbivory. Proceedings of the National Academy of Sciences of the United States of America 108: 5685–5689. 21389269 10.1073/pnas.1016508108 PMC3078369

[12] Van Zandt PA (2007) Plant defense, growth, and habitat: A comparative assessment of constitutive and induced resistance. Ecology 88: 1984–1993. 17824430 10.1890/06-1329.1

[13] Alfaro RI (1995) An induced defense reaction in white spruce to attack by the white-pine weevil, Pissodes strobi. Canadian Journal of Forest Research-Revue Canadienne De Recherche Forestiere 25: 1725–1730.

[14] Kiss GK, Yanchuk AD (1991) Preliminary evaluation of genetic-variation of weevil resistance in interior spruce in British-Columbia. Canadian Journal of Forest Research-Revue Canadienne De Recherche Forestiere 21: 230–234.

[15] King JN, Yanchuk AD, Kiss GK, Alfaro RI (1997) Genetic and phenotypic relationships between weevil (Pissodes strobi) resistance and height growth in spruce populations of British Columbia. Canadian Journal of Forest Research-Revue Canadienne De Recherche Forestiere 27: 732–739.

[16] Alfaro RI, He FL, Tomlin E, Kiss G (1997) White spruce resistance to white pine weevil related to bark resin canal density. Canadian Journal of Botany-Revue Canadienne De Botanique 75: 568–573.

[17] Lieutier F, Brignolas F, Sauvard D, Yart A, Galet C, et al (2003) Intra- and inter-provenance variability in phloem phenols of Picea abies and relationship to a bark beetle-associated fungus. Tree Physiology 23: 247–256. 12566260 10.1093/treephys/23.4.247

[18] Vandersar TJD, Borden JH (1977) Visual orientation of Pissodes-strobi Peck (Coleoptera curculionidae) in relation to host selection behavior. Canadian Journal of Zoology-Revue Canadienne De Zoologie 55: 2042–2049.

[19] He FL, Alfaro RI (2000) White pine weevil attack on white spruce: A survival time analysis. Ecological Applications 10: 225–232.

[20] Alfaro RI, King JN, Brown RG, Buddingh SM (2008) Screening of Sitka spruce genotypes for resistance to the white pine weevil using artificial infestations. Forest Ecology and Management 255: 1749–1758.

[21] vanAkker L, Alfaro RI, Brockley R (2004) Effects of fertilization on resin canal defences and incidence of Pissodes strobi attack in interior spruce. Canadian Journal of Forest Research-Revue Canadienne De Recherche Forestiere 34: 855–862.

[22] Gardner KM, Latta RG (2007) Shared quantitative trait loci underlying the genetic correlation between continuous traits. Molecular Ecology 16: 4195–4209. 17850272 10.1111/j.1365-294X.2007.03499.x

[23] McKay JK, Richards JH, Mitchell-Olds T (2003) Genetics of drought adaptation in Arabidopsis thaliana: I. Pleiotropy contributes to genetic correlations among ecological traits. Molecular Ecology 12: 1137–1151. 12694278 10.1046/j.1365-294x.2003.01833.x

[24] Tiffin P, Rausher MD (1999) Genetic constraints and selection acting on tolerance to herbivory in the common morning glory Ipomoea purpurea. American Naturalist 154: 700–716. 10.1086/303271 10600614

[25] MitchellOlds T (1996) Genetic constraints on life-history evolution: Quantitative-trait loci influencing growth and flowering in Arabidopsis thaliana. Evolution 50: 140–145. 28568847 10.1111/j.1558-5646.1996.tb04480.x

[26] Karrenberg S, Widmer A (2008) Ecologically relevant genetic variation from a non-Arabidopsis perspective. Current Opinion in Plant Biology 11: 156–162. 18329951 10.1016/j.pbi.2008.01.004

[27] Rockman MV, Kruglyak L (2006) Genetics of global gene expression. Nature Reviews Genetics 7: 862–872. 10.1038/nrg1964 17047685

[28] Farrall M (2004) Quantitative genetic variation: a post-modern view. Human Molecular Genetics 13: R1–R7. 14962979 10.1093/hmg/ddh084

[29] Porth I, Hamberger B, White R, Ritland K (2011) Defense mechanisms against herbivory in Picea: sequence evolution and expression regulation of gene family members in the phenylpropanoid pathway. BMC Genomics 12: 608. 22177423 10.1186/1471-2164-12-608 PMC3288119

[30] Potokina E, Druka A, Luo ZW, Wise R, Waugh R, et al (2008) Gene expression quantitative trait locus analysis of 16,000 barley genes reveals a complex pattern of genome-wide transcriptional regulation. Plant Journal 53: 90–101. 17944808 10.1111/j.1365-313X.2007.03315.x

[31] Ferrer JL, Austin MB, Stewart C, Noe JP (2008) Structure and function of enzymes involved in the biosynthesis of phenylpropanoids. Plant Physiology and Biochemistry 46: 356–370. 18272377 10.1016/j.plaphy.2007.12.009 PMC2860624

[32] Kirst M, Myburg AA, De Leon JPG, Kirst ME, Scott J, et al (2004) Coordinated genetic regulation of growth and lignin revealed by quantitative trait locus analysis of cDNA microarray data in an interspecific backcross of eucalyptus. Plant Physiology 135: 2368–2378. 15299141 10.1104/pp.103.037960 PMC520804

[33] Drost DR, Benedict CI, Berg A, Novaes E, Novaes CRDB, et al (2010) Diversification in the genetic architecture of gene expression and transcriptional networks in organ differentiation of Populus. Proceedings of the National Academy of Sciences of the United States of America 107: 8492–8497. 20404162 10.1073/pnas.0914709107 PMC2889559

[34] Street NR, Skogstrom O, Sjodin A, Tucker J, Rodriguez-Acosta M, et al (2006) The genetics and genomics of the drought response in Populus. Plant Journal 48: 321–341. 17005011 10.1111/j.1365-313X.2006.02864.x

[35] Ralph SG, Yueh H, Friedmann M, Aeschliman D, Zeznik JA, et al (2006) Conifer defence against insects: microarray gene expression profiling of Sitka spruce (*Picea sitchensis*) induced by mechanical wounding or feeding by spruce budworms (*Choristoneura occidentalis*) or white pine weevils (*Pissodes strobi*) reveals large-scale changes of the host transcriptome. Plant Cell and Environment 29: 1545–1570. 10.1111/j.1365-3040.2006.01532.x 16898017

[36] Sewell MM, Sherman BK, Neale DB (1999) A consensus map for loblolly pine (Pinus taeda L.). I. Construction and integration of individual linkage maps from two outbred three-generation pedigrees. Genetics 151: 321–330. 9872970 10.1093/genetics/151.1.321 PMC1460451

[37] Gibson G, Weir B (2005) The quantitative genetics of transcription. Trends in Genetics 21: 616–623. 16154229 10.1016/j.tig.2005.08.010

[38] Steppuhn A, Baldwin I (2008) Induced defenses and the cost-benefit paradigm. In: Schaller A, editor. Induced Plant Resistance to Herbivory: Springer. 61–83.

[39] Doebley J, Lukens L (1998) Transcriptional regulators and the evolution of plant form. Plant Cell 10: 1075–1082. 9668128 10.1105/tpc.10.7.1075 PMC1464652

[40] Chen K, Rajewsky N (2007) The evolution of gene regulation by transcription factors and microRNAs. Nature Reviews Genetics 8: 93–103. 10.1038/nrg1990 17230196

[41] Ralph SG, Jancsik S, Bohlmann J (2007) Dirigent proteins in conifer defense II: Extended gene discovery, phylogeny, and constitutive and stress-induced gene expression in spruce (Picea spp.). Phytochemistry 68: 1975–1991. 17590394 10.1016/j.phytochem.2007.04.042

[42] Naranjo MA, Forment J, Roldan M, Serrano R, Vicente O (2006) Overexpression of Arabidopsis thaliana LTL1, a salt-induced gene encoding a GDSL-motif lipase, increases salt tolerance in yeast and transgenic plants. Plant Cell and Environment 29: 1890–1900. 10.1111/j.1365-3040.2006.01565.x 16930315

[43] Lipka V, Dittgen J, Bednarek P, Bhat R, Wiermer M, et al (2005) Pre- and postinvasion defenses both contribute to nonhost resistance in Arabidopsis. Science 310: 1180–1183. 16293760 10.1126/science.1119409

[44] Shanmugam V (2005) Role of extracytoplasmic leucine rich repeat proteins in plant defence mechanisms. Microbiological Research 160: 83–94. 15782942 10.1016/j.micres.2004.09.014

[45] Saha D, Prasad AM, Srinivasan R (2007) Pentatricopeptide repeat proteins and their emerging roles in plants. Plant Physiology and Biochemistry 45: 521–534. 17560114 10.1016/j.plaphy.2007.03.026

[46] Belkhadir Y, Subramaniam R, Dangl JL (2004) Plant disease resistance protein signaling: NBS-LRR proteins and their partners. Current Opinion in Plant Biology 7: 391–399. 15231261 10.1016/j.pbi.2004.05.009

[47] Miernyk JA (2001) The J-domain proteins of Arabidopsis thaliana: an unexpectedly large and diverse family of chaperones. Cell Stress & Chaperones 6: 209–218. 11599562 10.1379/1466-1268(2001)006<0209:tjdpoa>2.0.co;2 PMC434402

[48] Gosti F, Beaudoin N, Serizet C, Webb AAR, Vartanian N, et al (1999) ABI1 protein phosphatase 2C is a negative regulator of abscisic acid signaling. Plant Cell 11: 1897–1909. 10521520 10.1105/tpc.11.10.1897 PMC144098

[49] Schmelz EA, LeClere S, Carroll MJ, Alborn HT, Teal PEA (2007) Cowpea chloroplastic ATP synthase is the source of multiple plant defense elicitors during insect herbivory. Plant Physiology 144: 793–805. 17369425 10.1104/pp.107.097154 PMC1914193

[50] Jennings DB, Ehrenshaft M, Pharr DM, Williamson JD (1998) Roles for mannitol and mannitol dehydrogenase in active oxygen-mediated plant defense. Proceedings of the National Academy of Sciences of the United States of America 95: 15129–15133. 9844027 10.1073/pnas.95.25.15129 PMC24587

[51] Koutaniemi S, Warinowski T, Karkonen A, Alatalo E, Fossdal CG, et al (2007) Expression profiling of the lignin biosynthetic pathway in Norway spruce using EST sequencing and real-time RT-PCR. Plant Molecular Biology 65: 311–328. 17764001 10.1007/s11103-007-9220-5

[52] Zhao JM, Last RL (1996) Coordinate regulation of the tryptophan biosynthetic pathway and indolic phytoalexin accumulation in Arabidopsis. Plant Cell 8: 2235–2244. 8989880 10.1105/tpc.8.12.2235 PMC161348

[53] Kliebenstein D (2009) Quantitative Genomics: Analyzing Intraspecific Variation Using Global Gene Expression Polymorphisms or eQTLs. Annual Review of Plant Biology 60: 93–114. 10.1146/annurev.arplant.043008.092114 19012536

[54] Fehrmann RSN, Jansen RC, Veldink JH, Westra H-J, Arends D, et al (2011) *Trans*-eQTLs Reveal That Independent Genetic Variants Associated with a Complex Phenotype Converge on Intermediate Genes, with a Major Role for the HLA. PLoS Genet 7: e1002197. 21829388 10.1371/journal.pgen.1002197 PMC3150446

[55] Baum G, LevYadun S, Fridmann Y, Arazi T, Katsnelson H, et al (1996) Calmodulin binding to glutamate decarboxylase is required for regulation of glutamate and GABA metabolism and normal development in plants. Embo Journal 15: 2988–2996. 8670800 PMC450240

[56] Bown AW, MacGregor KB, Shelp BJ (2006) Gamma-aminobutyrate: defense against invertebrate pests? Trends in Plant Science 11: 424–427. 16890474 10.1016/j.tplants.2006.07.002

[57] Dreher K, Callis J (2007) Ubiquitin, hormones and biotic stress in plants. Annals of Botany 99: 787–822. 17220175 10.1093/aob/mcl255 PMC2802907

[58] Chung HS, Koo AJK, Gao XL, Jayanty S, Thines B, et al (2008) Regulation and function of Arabidopsis JASMONATE ZIM-domain genes in response to wounding and herbivory. Plant Physiology 146: 952–964. 18223147 10.1104/pp.107.115691 PMC2259048

[59] Kazan K, Manners JM (2008) Jasmonate signaling: toward an integrated view. Plant Physiology 146: 1459–1468. 18390489 10.1104/pp.107.115717 PMC2287326

[60] Ferreira FJ, Guo C, Coleman JR (2008) Reduction of plastid-localized carbonic anhydrase activity results in reduced Arabidopsis seedling survivorship. Plant Physiology 147: 585–594. 18434607 10.1104/pp.108.118661 PMC2409021

[61] Nakano T, Suzuki K, Fujimura T, Shinshi H (2006) Genome-wide analysis of the ERF gene family in Arabidopsis and rice. Plant Physiology 140: 411–432. 16407444 10.1104/pp.105.073783 PMC1361313

[62] Yang Z, Tian LN, Latoszek-Green M, Brown D, Wu KQ (2005) Arabidopsis ERF4 is a transcriptional repressor capable of modulating ethylene and abscisic acid responses. Plant Molecular Biology 58: 585–596. 16021341 10.1007/s11103-005-7294-5

[63] Adams E, Devoto A, Turner J (2007) Analysis of a novel ethylene-induced COI1-dependent signalling pathway in Arabidopsis thaliana. In: al Re, editor. Advances in Plant Ethylene Research: Proceedings of the 7th International Symposium on the Plant Hormone Ethylene: Springer. 81–87.

[64] Chae H, Kieber J (2005) Eto Brute? Role of ACS turnover in regulating ethylene biosynthesis. Trends in Plant Science 10. 10.1016/j.tplants.2005.04.006 15949763

[65] Pauwels L, Goossens A (2011) The JAZ Proteins: A Crucial Interface in the Jasmonate Signaling Cascade. The Plant Cell Online 23: 3089–3100. 10.1105/tpc.111.089300 PMC3203442 21963667

[66] Xiao S, Dai LY, Liu FQ, Wang Z, Peng W, et al (2004) COS1: An Arabidopsis Coronatine insensitive1 suppressor essential for regulation of jasmonate-mediated plant defense and senescence. Plant Cell 16: 1132–1142. 15075400 10.1105/tpc.020370 PMC423205

[67] Gonzalez-Martinez SC, Wheeler NC, Ersoz E, Nelson CD, Neale DB (2007) Association genetics in Pinus taeda L. I. Wood property traits. Genetics 175: 399–409. 17110498 10.1534/genetics.106.061127 PMC1775017

[68] Pot D, McMillan L, Echt C, Le Provost G, Garnier-Gere P, et al (2005) Nucleotide variation in genes involved in wood formation in two pine species. New Phytologist 167: 101–112. 15948834 10.1111/j.1469-8137.2005.01417.x

[69] Chagne D, Brown G, Lalanne C, Madur D, Pot D, et al (2003) Comparative genome and QTL mapping between maritime and loblolly pines. Molecular Breeding 12: 185–195.

[70] Sewell MM, Davis MF, Tuskan GA, Wheeler NC, Elam CC, et al (2002) Identification of QTLs influencing wood property traits in loblolly pine (Pinus taeda L.). II. Chemical wood properties. Theoretical and Applied Genetics 104: 214–222. 12582689 10.1007/s001220100697

[71] Holliday JA, Ralph SG, White R, Bohlmann J, Aitken SN (2008) Global monitoring of autumn gene expression within and among phenotypically divergent populations of Sitka spruce (Picea sitchensis). New Phytologist 178: 103–122. 18194148 10.1111/j.1469-8137.2007.02346.x

[72] Howe GA, Jander G (2008) Plant immunity to insect herbivores. Annual Review of Plant Biology. Palo Alto: Annual Reviews. 41–66. 10.1146/annurev.arplant.59.032607.092825 18031220

[73] Thaler JS, Farag MA, Pare PW, Dicke M (2002) Jasmonate-deficient plants have reduced direct and indirect defences against herbivores. Ecology Letters 5: 764–774.

[74] Alfaro RI, VanAkker L, Jaquish B, King J (2004) Weevil resistance of progeny derived from putatively resistant and susceptible interior spruce parents. Forest Ecology and Management 202: 369–377.

[75] Doyle J, Doyle J (1990) Isolation of plant DNA from fresh tissue. *Focus* 12: 13–15.

[76] Hu XS, Goodwillie C, Ritland KM (2004) Joining genetic linkage maps using a joint likelihood function. Theoretical and Applied Genetics 109: 996–1004. 15221138 10.1007/s00122-004-1705-x

[77] Stam P (1993) Construction of integrated genetic-linkage maps by means of a new computer package - Joinmap. Plant Journal 3: 739–744.

[78] Ralph S, Park JY, Bohlmann J, Mansfield SD (2006) Dirigent proteins in conifer defense: gene discovery, phylogeny, and differential wound- and insect-induced expression of a family of DIR and DIR-like genes in spruce (Picea spp.). Plant Molecular Biology 60: 21–40. 16463097 10.1007/s11103-005-2226-y

[79] Kolosova N, Miller B, Ralph S, Ellis BE, Douglas C, et al (2004) Isolation of high-quality RNA from gymnosperm and angiosperm trees. Biotechniques 36: 821–824. 15152602 10.2144/04365ST06

[80] Fu JY, Jansen RC (2006) Optimal design and analysis of genetic studies on gene expression. Genetics 172: 1993–1999. 16361243 10.1534/genetics.105.047001 PMC1456314

[81] Huber W, von Heydebreck A, Sultmann H, Poustka A, Vingron M (2002) Variance stabilization applied to microarray data calibration and to the quantification of differential expression. Bioinformatics 18: S96–S104. 12169536 10.1093/bioinformatics/18.suppl_1.s96

[82] Maere S, Heymans K, Kuiper M (2005) BiNGO: a cytoscape plugin to assess overrepresentation of gene ontology categories in biological networks. Bioinformatics 21: 3448–3449. 15972284 10.1093/bioinformatics/bti551

