# Supplementary figures and images for: Genetical Genomics Identifies the Genetic Architecture for Growth and Weevil Resistance in Spruce
Source: PLoS One. 2012 Sep 4;7(9):e44397. doi: 10.1371/journal.pone.0044397 (PMC3433439; doi:10.1371/journal.pone.0044397)

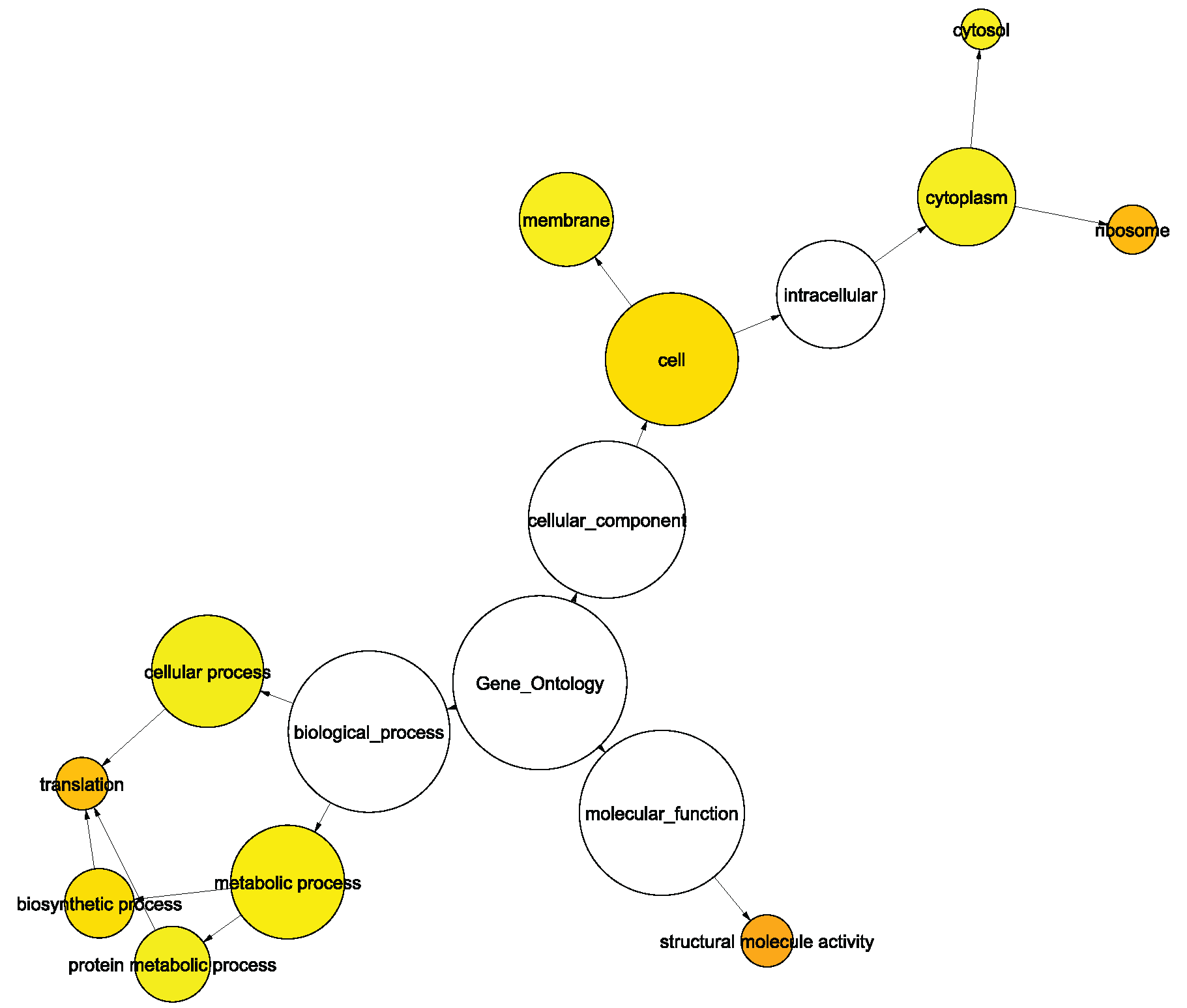

Supplement: Figure S1 — GO tree representation showing significantly (p ≤ 0.05) overrepresented GO categories within the trans eQTL-hotspot at the carbonic anhydrase gene locus contig_2079_440 (803 eQTLs) on LG4, for color code see Figures 2 , 3 , 4 and 5 in main text. (TIFF) [file pone.0044397.s001.tiff]

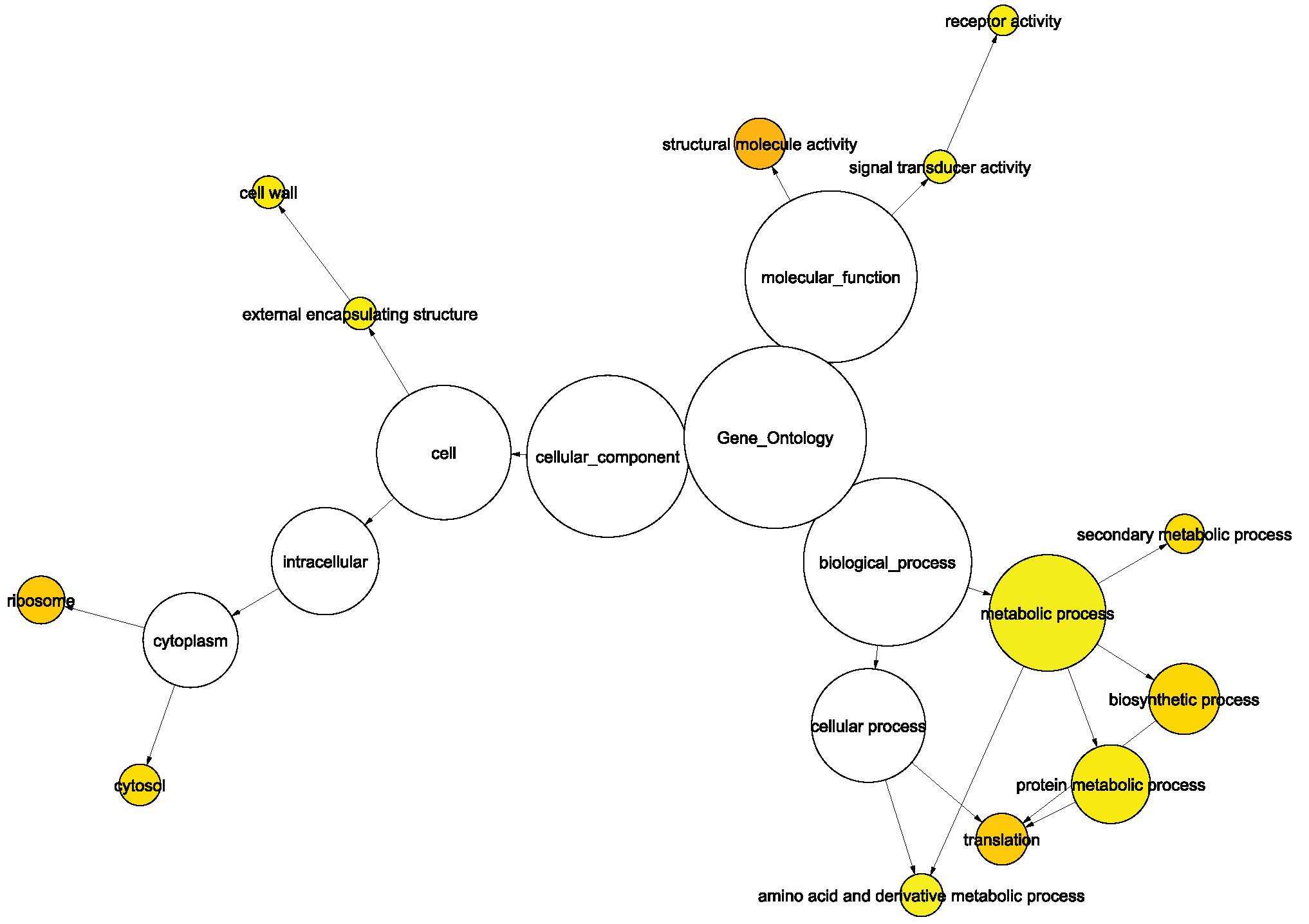

Supplement: Figure S2 — GO tree representation showing significantly (p ≤ 0.05) overrepresented GO categories within the trans eQTL-hotspot at the carbonic anhydrase gene locus contig_103_602 (1122 eQTLs) on LG4, for color code see Figures 2 , 3 , 4 and 5 in main text. (TIFF) [file pone.0044397.s002.tiff]
