# Supplementary material for: Genetical Genomics Identifies the Genetic Architecture for Growth and Weevil Resistance in Spruce
Source: PLoS One. 2012 Sep 4;7(9):e44397. doi: 10.1371/journal.pone.0044397 (PMC3433439; doi:10.1371/journal.pone.0044397)
Supplement: File S1 — Comprehensive list of all 132,100 significant eQTLs (legends can be found in Table S1). (PDF) [file pone.0044397.s003.pdf]

1	38.326	6	2	0.325	0.065	8.399	1	WS00712_F05
4	116.928	82	2	-0.223	0.034	4.281	1	WS00712_F05
5	57.017	102	2	0.230	0.042	5.461	1	WS00712_F05
6	56.188	120	2	0.297	0.060	6.010	1	WS00712_F05
7	54.200	136	2	0.254	0.056	3.929	1	WS00712_F05
10	78.479	193	3	-0.429	0.110	11.604	1	WS00712_F05
10	86.151	195	3	0.281	0.064	6.802	1	WS00712_F05
12	22.629	237	1	0.298	0.074	4.620	1	WS00712_F05
4	142.917	85	1	0.318	0.072	4.472	2	WS00712_L14
5	57.017	102	2	0.174	0.031	3.960	2	WS00712_L14
11	27.405	204	4	0.324	0.075	5.443	2	WS00712_L14
13	3.386	245	1	0.253	0.074	4.682	2	WS00712_L14
1	77.177	15	2	-0.173	0.042	3.924	3	WS0094_O21
3	14.500	43	1	-0.221	0.042	3.873	3	WS0094_O21
7	74.560	143	1	-0.223	0.045	4.223	3	WS0094_O21
8	87.463	158	2	0.259	0.072	6.712	3	WS0094_O21
9	59.963	177	4	-0.296	0.073	7.293	3	WS0094_O21
13	3.386	245	1	0.302	0.081	7.534	3	WS0094_O21
1	133.992	24	2	0.231	0.040	4.580	4	WS0057_H07
2	48.839	29	4	-0.263	0.056	5.956	4	WS0057_H07
4	0.000	71	4	0.224	0.039	4.156	4	WS0057_H07
4	97.576	80	2	0.205	0.032	5.338	4	WS0057_H07
4	103.719	81	4	0.230	0.040	4.552	4	WS0057_H07
5	56.008	101	2	0.225	0.044	5.166	4	WS0057_H07
6	45.897	117	4	0.260	0.045	4.743	4	WS0057_H07
9	61.113	178	1	0.379	0.113	4.708	4	WS0057_H07
10	35.204	189	1	0.212	0.061	4.473	4	WS0057_H07
10	78.479	193	3	-0.292	0.065	5.311	4	WS0057_H07
1	43.578	8	2	0.229	0.045	4.333	5	WS01017_D02
1	69.854	13	2	-0.263	0.068	6.663	5	WS01017_D02
1	71.592	14	2	-0.261	0.068	6.623	5	WS01017_D02
1	77.177	15	2	-0.279	0.078	7.583	5	WS01017_D02
1	95.572	17	2	-0.241	0.041	4.017	5	WS01017_D02
3	47.928	49	1	0.335	0.132	11.959	5	WS01017_D02
4	75.958	77	3	-0.396	0.129	7.231	5	WS01017_D02
4	116.928	82	2	-0.260	0.055	5.497	5	WS01017_D02
5	57.017	102	2	0.277	0.071	7.304	5	WS01017_D02
6	45.897	117	1	0.200	0.043	3.858	5	WS01017_D02
9	59.963	177	4	-0.279	0.071	6.999	5	WS01017_D02
11	17.088	203	1	-0.211	0.058	5.196	5	WS01017_D02
11	45.989	216	1	-0.234	0.045	4.069	5	WS01017_D02
12	22.629	237	1	0.276	0.064	5.769	5	WS01017_D02
14	44.898	251	3	0.267	0.052	4.380	5	WS01017_D02
2	127.147	40	3	-0.225	0.050	6.752	6	WS0058_C01
3	95.157	59	2	0.172	0.039	4.828	6	WS0058_C01
3	112.868	61	2	0.144	0.028	3.876	6	WS0058_C01
4	116.928	82	2	0.263	0.035	4.791	6	WS0058_C01
4	124.132	83	2	0.301	0.061	6.741	6	WS0058_C01
5	52.820	99	3	0.276	0.042	5.743	6	WS0058_C01
5	86.669	106	2	0.215	0.046	4.552	6	WS0058_C01
6	49.100	118	2	0.262	0.060	5.903	6	WS0058_C01
6	69.073	121	3	-0.231	0.045	6.169	6	WS0058_C01
6	95.249	126	1	0.344	0.106	4.002	6	WS0058_C01
11	64.014	222	3	-0.176	0.031	4.156	6	WS0058_C01

1	69.854	13	2	-0.225	0.046	5.852	7	WS00712_O19
1	71.592	14	2	-0.220	0.045	5.657	7	WS00712_O19
1	77.177	15	2	-0.256	0.061	7.652	7	WS00712_O19
3	148.745	68	1	0.275	0.096	5.837	7	WS00712_O19
3	154.505	69	1	0.237	0.065	4.008	7	WS00712_O19
6	26.350	115	2	-0.219	0.038	4.850	7	WS00712_O19
10	93.347	196	1	-0.452	0.120	4.899	7	WS00712_O19
11	27.405	204	4	0.351	0.069	6.255	7	WS00712_O19
11	42.600	213	3	-0.247	0.046	4.508	7	WS00712_O19
1	29.800	5	2	0.320	0.043	5.846	8	WS00712_P10
1	38.326	6	2	-0.164	0.030	3.956	8	WS00712_P10
3	47.928	49	1	0.252	0.089	4.943	8	WS00712_P10
4	0.000	71	4	-0.286	0.051	4.606	8	WS00712_P10
4	82.462	78	2	0.230	0.032	4.782	8	WS00712_P10
5	16.219	93	2	0.243	0.030	4.021	8	WS00712_P10
6	106.702	128	2	0.457	0.080	5.706	8	WS00712_P10
14	46.637	252	3	0.259	0.064	6.479	8	WS00712_P10
4	6.032	72	4	-0.212	0.057	4.792	9	WS00713_C11
4	82.462	78	2	0.227	0.059	5.837	9	WS00713_C11
4	92.039	79	2	0.193	0.040	4.038	9	WS00713_C11
7	15.555	130	2	0.290	0.061	6.068	9	WS00713_C11
14	44.898	251	3	0.276	0.062	6.512	9	WS00713_C11
3	104.681	60	2	-0.231	0.056	6.220	10	WS00713_D02
3	112.868	61	2	0.251	0.073	8.071	10	WS00713_D02
3	114.747	62	2	0.251	0.054	5.998	10	WS00713_D02
4	173.929	88	2	-0.211	0.042	4.710	10	WS00713_D02
5	84.347	105	2	-0.202	0.047	4.222	10	WS00713_D02
5	86.669	106	2	0.327	0.105	6.073	10	WS00713_D02
9	36.227	165	4	-0.235	0.060	5.197	10	WS00713_D02
10	80.088	194	2	-0.215	0.032	3.894	10	WS00713_D02
11	29.069	205	1	0.278	0.048	3.876	10	WS00713_D02
11	45.989	216	1	-0.265	0.080	6.114	10	WS00713_D02
1	45.794	9	1	-0.316	0.116	6.870	11	WS00713_G13
1	107.333	19	3	0.255	0.061	8.238	11	WS00713_G13
1	127.805	21	3	0.223	0.029	3.922	11	WS00713_G13
3	9.953	42	3	0.164	0.037	4.942	11	WS00713_G13
5	57.017	102	2	-0.267	0.034	4.474	11	WS00713_G13
7	17.610	131	3	-0.276	0.038	3.943	11	WS00713_G13
10	70.246	190	2	0.190	0.032	4.117	11	WS00713_G13
12	6.242	234	2	0.240	0.044	5.997	11	WS00713_G13
14	44.898	251	1	0.278	0.095	5.553	11	WS00713_G13
2	120.617	38	3	0.200	0.033	4.030	12	WS00713_H21
2	127.147	40	3	-0.219	0.046	5.561	12	WS00713_H21
3	9.953	42	3	0.192	0.035	4.276	12	WS00713_H21
4	124.132	83	2	0.239	0.052	4.328	12	WS00713_H21
11	27.405	204	4	0.282	0.082	5.361	12	WS00713_H21
11	42.600	213	3	-0.201	0.035	4.242	12	WS00713_H21
14	43.392	250	1	-0.273	0.113	4.686	12	WS00713_H21
1	77.177	15	2	-0.197	0.032	4.175	13	WS00713_K03
2	127.147	40	3	-0.223	0.037	4.346	13	WS00713_K03
4	82.462	78	2	0.251	0.039	4.787	13	WS00713_K03
5	86.669	106	2	0.306	0.077	6.560	13	WS00713_K03
6	106.702	128	2	0.495	0.106	7.285	14	WS00713_L20
7	74.560	143	1	0.335	0.061	5.628	14	WS00713_L20

9	28.608	170	3	0.290	0.045	5.066	14	WS00713_L20
9	81.073	183	3	-0.246	0.044	5.252	14	WS00713_L20
11	29.069	205	1	0.316	0.059	5.527	14	WS00713_L20
11	45.989	216	1	-0.267	0.072	6.508	14	WS00713_L20
11	64.014	222	3	0.302	0.062	7.163	14	WS00713_L20
14	0.000	246	1	0.253	0.059	5.337	14	WS00713_L20
1	29.800	5	2	0.305	0.050	6.436	15	WS00946_G04
1	38.326	6	2	-0.216	0.037	4.866	15	WS00946_G04
1	133.992	24	2	-0.238	0.044	5.851	15	WS00946_G04
2	120.617	38	3	0.248	0.040	4.945	15	WS00946_G04
2	127.147	40	3	-0.296	0.075	9.299	15	WS00946_G04
3	69.071	52	1	0.342	0.145	6.105	15	WS00946_G04
4	116.928	82	2	0.254	0.039	5.092	15	WS00946_G04
5	52.820	99	3	0.224	0.033	4.129	15	WS00946_G04
5	54.072	100	4	0.182	0.067	4.216	15	WS00946_G04
7	17.610	131	3	-0.297	0.046	3.866	15	WS00946_G04
10	21.864	188	3	0.198	0.034	4.106	15	WS00946_G04
3	9.953	42	3	0.171	0.035	4.345	16	WS00817_N21
4	116.928	82	2	0.314	0.052	7.286	16	WS00817_N21
5	84.347	105	2	-0.223	0.054	7.388	16	WS00817_N21
5	86.669	106	2	0.347	0.103	9.319	16	WS00817_N21
10	9.580	187	2	0.190	0.080	3.868	16	WS00817_N21
12	6.242	234	2	0.192	0.031	4.554	16	WS00817_N21
6	49.100	118	2	0.191	0.059	4.925	17	WS00945_A17
10	35.204	189	2	-0.195	0.052	5.692	17	WS00945_A17
1	45.794	9	1	-0.334	0.111	8.495	18	WS00818_E03
4	116.928	82	2	0.316	0.047	5.473	18	WS00818_E03
4	142.917	85	1	-0.270	0.060	4.445	18	WS00818_E03
5	31.191	95	1	-0.204	0.071	5.094	18	WS00818_E03
5	54.072	100	4	-0.252	0.039	4.006	18	WS00818_E03
7	15.555	130	2	0.353	0.058	7.174	18	WS00818_E03
12	28.493	228	2	0.150	0.058	3.865	18	WS00818_E03
1	29.800	5	2	0.278	0.042	4.626	19	WS00922_K06
1	38.326	6	2	-0.228	0.044	4.968	19	WS00922_K06
6	69.073	121	3	-0.220	0.038	4.391	19	WS00922_K06
10	9.580	187	2	0.302	0.123	4.710	19	WS00922_K06
10	21.864	188	1	0.275	0.055	4.121	19	WS00922_K06
11	27.405	204	1	0.345	0.086	6.385	19	WS00922_K06
11	29.069	205	1	0.312	0.108	8.070	19	WS00922_K06
11	45.989	216	1	-0.259	0.097	7.258	19	WS00922_K06
11	60.464	221	1	0.303	0.059	4.464	19	WS00922_K06
14	44.898	251	1	0.259	0.058	4.245	19	WS00922_K06
14	46.637	252	3	0.228	0.039	4.530	19	WS00922_K06
3	47.928	49	1	0.295	0.078	6.878	20	WS00922_N08
4	0.000	71	4	-0.225	0.097	7.164	20	WS00922_N08
6	69.073	121	4	-0.162	0.049	4.001	20	WS00922_N08
8	50.001	154	1	0.247	0.050	4.703	20	WS00922_N08
10	9.580	187	3	-0.421	0.133	7.775	20	WS00922_N08
10	78.479	193	3	-0.440	0.068	7.836	20	WS00922_N08
11	35.604	207	3	-0.154	0.040	4.531	20	WS00922_N08
11	64.014	222	3	-0.227	0.042	4.761	20	WS00922_N08
3	80.211	55	4	-0.206	0.060	4.954	21	WS00814_I08
7	15.555	130	2	0.284	0.043	4.550	21	WS00814_I08
7	17.610	131	3	0.254	0.070	4.657	21	WS00814_I08

7	70.278	141	3	0.236	0.051	5.397	21	WS00814_I08
7	91.487	146	3	0.176	0.041	4.316	21	WS00814_I08
10	78.479	193	3	-0.246	0.050	5.236	21	WS00814_I08
12	22.441	227	3	-0.271	0.057	5.779	21	WS00814_I08
12	6.242	234	2	0.189	0.035	4.299	21	WS00814_I08
13	3.386	245	1	0.328	0.099	8.004	21	WS00814_I08
2	48.839	29	4	0.246	0.051	4.869	22	WS00814_J01
2	95.880	34	3	0.242	0.064	7.178	22	WS00814_J01
2	101.076	35	3	-0.212	0.042	4.240	22	WS00814_J01
3	47.928	49	1	0.305	0.091	7.895	22	WS00814_J01
5	31.191	95	1	-0.235	0.057	4.921	22	WS00814_J01
6	89.401	125	1	-0.316	0.054	4.744	22	WS00814_J01
6	95.249	126	1	0.388	0.114	5.171	22	WS00814_J01
7	15.555	130	2	0.220	0.049	4.953	22	WS00814_J01
11	29.069	205	1	0.309	0.054	4.800	22	WS00814_J01
6	25.914	114	1	-0.205	0.058	6.202	23	WS00814_L10
7	73.169	142	1	0.509	0.064	4.244	23	WS00814_L10
9	61.113	178	1	0.400	0.061	4.173	23	WS00814_L10
10	78.479	193	3	-0.276	0.045	4.010	23	WS00814_L10
1	107.333	19	3	0.220	0.036	4.257	24	WS00814_M21
11	27.405	204	4	0.247	0.059	4.622	24	WS00814_M21
11	45.989	216	1	-0.217	0.064	5.699	24	WS00814_M21
1	95.572	17	3	0.230	0.039	3.932	25	WS00814_P08
1	107.333	19	3	0.176	0.038	4.240	25	WS00814_P08
9	2.011	167	4	-0.208	0.052	3.963	25	WS00814_P08
11	64.014	222	3	-0.271	0.056	6.263	25	WS00814_P08
13	3.386	245	1	0.216	0.043	4.049	25	WS00814_P08
3	20.512	46	2	0.236	0.034	4.080	26	WS00814_P22
4	6.032	72	4	-0.191	0.053	5.557	26	WS00814_P22
4	75.958	77	3	-0.372	0.124	6.073	26	WS00814_P22
7	75.883	144	2	0.500	0.097	7.060	26	WS00814_P22
7	105.477	147	2	-0.332	0.115	7.787	26	WS00814_P22
11	45.989	216	1	-0.297	0.107	7.607	26	WS00814_P22
14	44.898	251	3	0.246	0.050	4.245	26	WS00814_P22
3	141.443	65	3	-0.358	0.091	5.033	27	WS00815_B18
4	97.576	80	2	0.192	0.065	6.942	27	WS00815_B18
4	124.132	83	1	0.625	0.220	7.827	27	WS00815_B18
5	3.484	90	2	0.233	0.107	5.475	27	WS00815_B18
7	17.610	131	3	0.343	0.112	5.215	27	WS00815_B18
7	67.154	140	4	0.422	0.163	5.813	27	WS00815_B18
7	75.883	144	2	-0.706	0.203	12.230	27	WS00815_B18
7	75.883	144	4	0.352	0.099	8.494	27	WS00815_B18
9	36.227	165	3	-0.137	0.039	3.936	27	WS00815_B18
9	36.227	165	4	0.269	0.049	4.395	27	WS00815_B18
9	69.046	181	2	-0.316	0.108	5.212	27	WS00815_B18
11	35.604	207	3	-0.273	0.061	6.207	27	WS00815_B18
11	37.143	209	3	0.281	0.065	6.639	27	WS00815_B18
11	42.600	213	3	-0.305	0.076	7.719	27	WS00815_B18
12	22.441	227	1	-0.266	0.052	4.798	27	WS00815_B18
12	28.493	228	2	0.352	0.191	9.745	27	WS00815_B18
13	1.815	244	2	0.239	0.041	3.962	27	WS00815_B18
14	0.000	246	1	-0.272	0.052	4.785	27	WS00815_B18
1	50.955	10	1	0.232	0.075	5.404	28	WS00815_C18
1	82.301	16	3	-0.231	0.034	4.430	28	WS00815_C18

1	107.333	19	3	0.208	0.031	4.001	28	WS00815_C18
2	120.617	38	3	0.199	0.034	4.405	28	WS00815_C18
2	127.147	40	3	-0.205	0.049	6.213	28	WS00815_C18
3	47.928	49	1	0.201	0.060	4.326	28	WS00815_C18
3	95.157	59	2	0.201	0.036	3.910	28	WS00815_C18
4	124.132	83	2	0.250	0.071	4.390	28	WS00815_C18
4	142.917	85	1	-0.207	0.054	3.900	28	WS00815_C18
5	31.191	95	1	-0.207	0.065	4.664	28	WS00815_C18
5	86.669	106	2	0.342	0.065	5.116	28	WS00815_C18
6	106.702	128	2	0.455	0.066	5.165	28	WS00815_C18
11	27.405	204	1	0.300	0.073	5.224	28	WS00815_C18
11	29.069	205	1	0.306	0.091	6.624	28	WS00815_C18
11	45.989	216	1	-0.261	0.070	5.074	28	WS00815_C18
12	22.441	227	1	-0.228	0.064	4.653	28	WS00815_C18
1	107.333	19	3	0.180	0.042	4.373	29	WS00815_H08
1	127.805	21	3	0.205	0.042	4.425	29	WS00815_H08
3	104.681	60	2	-0.174	0.036	4.016	29	WS00815_H08
3	112.868	61	2	0.179	0.044	4.904	29	WS00815_H08
3	114.747	62	2	0.226	0.040	4.521	29	WS00815_H08
5	56.008	101	2	0.189	0.046	4.916	29	WS00815_H08
6	26.350	115	2	-0.206	0.036	4.067	29	WS00815_H08
7	59.789	137	3	-0.204	0.039	4.095	29	WS00815_H08
11	46.026	217	1	-0.275	0.075	5.656	29	WS00815_H08
1	38.326	6	2	-0.223	0.032	4.492	30	WS00815_N10
1	62.128	11	2	-0.271	0.059	7.187	30	WS00815_N10
1	69.854	13	2	-0.187	0.036	5.075	30	WS00815_N10
1	71.592	14	2	-0.204	0.043	6.003	30	WS00815_N10
1	77.177	15	2	-0.189	0.037	5.198	30	WS00815_N10
2	120.617	38	3	0.207	0.032	4.854	30	WS00815_N10
2	127.147	40	3	-0.267	0.074	10.896	30	WS00815_N10
3	95.157	59	2	0.182	0.035	4.644	30	WS00815_N10
3	112.868	61	2	0.191	0.032	4.475	30	WS00815_N10
4	124.132	83	2	0.188	0.031	5.169	30	WS00815_N10
5	52.820	99	3	0.263	0.049	7.303	30	WS00815_N10
6	49.100	118	2	0.285	0.044	4.927	30	WS00815_N10
6	95.249	126	1	0.347	0.132	5.172	30	WS00815_N10
11	27.405	204	1	0.372	0.141	6.768	30	WS00815_N10
11	29.069	205	1	0.281	0.115	5.467	30	WS00815_N10
11	45.989	216	1	-0.198	0.095	4.507	30	WS00815_N10
1	22.419	4	3	0.354	0.089	5.241	31	WS00715_I03
3	47.928	49	1	-0.247	0.062	5.568	31	WS00715_I03
4	103.719	81	4	0.256	0.054	4.867	31	WS00715_I03
4	124.132	83	2	-0.295	0.092	3.948	31	WS00715_I03
5	31.191	95	1	0.204	0.044	3.932	31	WS00715_I03
6	25.914	114	1	0.227	0.047	4.270	31	WS00715_I03
6	81.996	123	3	-0.513	0.203	11.889	31	WS00715_I03
7	15.555	130	2	-0.276	0.054	5.339	31	WS00715_I03
7	15.555	130	3	0.270	0.046	4.876	31	WS00715_I03
9	17.951	169	1	-0.330	0.061	5.489	31	WS00715_I03
10	93.347	196	2	-0.479	0.122	5.280	31	WS00715_I03
11	0.000	199	3	0.223	0.053	3.925	31	WS00715_I03
11	40.960	210	2	-0.461	0.107	6.412	31	WS00715_I03
11	40.960	210	4	0.331	0.062	5.488	31	WS00715_I03
12	14.406	230	3	-0.289	0.049	5.008	31	WS00715_I03

2	75.550	31	2	-0.316	0.064	4.623	32	WS00921_B20
2	93.504	32	2	-0.217	0.039	3.861	32	WS00921_B20
3	137.891	64	1	-0.217	0.043	3.856	32	WS00921_B20
3	141.443	65	3	-0.353	0.095	5.952	32	WS00921_B20
8	50.001	154	1	-0.247	0.049	4.702	32	WS00921_B20
9	55.358	175	3	0.825	0.240	14.261	32	WS00921_B20
10	35.204	189	1	0.295	0.055	5.046	32	WS00921_B20
11	9.237	202	4	0.227	0.045	3.842	32	WS00921_B20
12	28.493	228	2	0.263	0.082	4.540	32	WS00921_B20
13	1.815	244	2	0.228	0.049	4.837	32	WS00921_B20
2	127.147	40	3	-0.199	0.056	5.493	33	WS00716_A08
4	0.000	71	4	0.246	0.044	4.022	33	WS00716_A08
4	124.132	83	1	-0.546	0.182	5.962	33	WS00716_A08
6	56.188	120	1	0.217	0.066	4.654	33	WS00716_A08
6	87.891	124	2	-0.260	0.065	7.592	33	WS00716_A08
6	89.401	125	2	-0.363	0.083	8.971	33	WS00716_A08
10	80.088	194	1	0.269	0.060	4.250	33	WS00716_A08
11	42.600	213	3	-0.167	0.040	3.876	33	WS00716_A08
12	14.406	230	4	0.306	0.071	4.899	33	WS00716_A08
2	120.617	38	3	0.211	0.032	4.702	34	WS00921_G11
2	127.147	40	3	-0.286	0.081	11.895	34	WS00921_G11
4	124.132	83	2	0.237	0.053	5.058	34	WS00921_G11
5	31.191	95	1	-0.242	0.062	4.313	34	WS00921_G11
5	52.820	99	3	0.279	0.054	7.709	34	WS00921_G11
6	49.100	118	2	0.276	0.048	4.334	34	WS00921_G11
6	95.249	126	1	0.421	0.153	9.006	34	WS00921_G11
9	36.227	165	3	-0.246	0.029	4.330	34	WS00921_G11
11	27.405	204	1	0.316	0.087	6.165	34	WS00921_G11
3	148.745	68	1	0.202	0.056	4.057	35	WS00945_F05
4	82.462	78	2	0.185	0.035	4.274	35	WS00945_F05
6	45.897	117	4	-0.295	0.069	5.268	35	WS00945_F05
6	87.891	124	2	-0.172	0.040	4.684	35	WS00945_F05
8	39.090	152	4	-0.158	0.043	4.033	35	WS00945_F05
10	9.580	187	2	0.465	0.176	10.594	35	WS00945_F05
10	21.864	188	3	0.171	0.040	4.453	35	WS00945_F05
11	41.984	212	2	-0.219	0.036	4.266	35	WS00945_F05
11	45.989	216	2	-0.234	0.040	4.879	35	WS00945_F05
11	60.464	221	2	-0.249	0.038	4.898	35	WS00945_F05
12	28.493	228	2	0.341	0.104	6.237	35	WS00945_F05
2	102.814	36	1	-0.230	0.046	4.076	36	WS00945_G01
3	14.500	43	1	-0.269	0.054	4.250	36	WS00945_G01
3	141.443	65	3	-0.306	0.085	4.769	36	WS00945_G01
3	144.593	66	4	0.169	0.037	4.660	36	WS00945_G01
3	148.745	68	4	-0.272	0.053	4.564	36	WS00945_G01
6	56.188	120	1	0.302	0.068	5.381	36	WS00945_G01
9	32.356	171	2	-0.189	0.040	4.322	36	WS00945_G01
10	21.864	188	1	0.213	0.056	4.046	36	WS00945_G01
10	21.864	188	3	0.258	0.067	5.796	36	WS00945_G01
11	42.600	213	3	-0.256	0.059	5.459	36	WS00945_G01
11	48.979	218	2	0.260	0.049	5.323	36	WS00945_G01
12	28.493	228	2	0.273	0.063	4.620	36	WS00945_G01
1	62.128	11	1	-0.464	0.205	4.093	37	WS00945_J06
4	55.925	76	1	-0.307	0.087	7.248	37	WS00945_J06
5	52.820	99	3	0.190	0.034	4.429	37	WS00945_J06

7	62.787	139	4	0.188	0.050	4.587	37	WS00945_J06
11	27.405	204	1	0.242	0.050	3.996	37	WS00945_J06
11	37.143	209	3	0.214	0.043	5.031	37	WS00945_J06
11	64.014	222	3	-0.234	0.037	4.350	37	WS00945_J06
14	44.898	251	3	0.274	0.049	5.880	37	WS00945_J06
2	93.504	32	4	-0.219	0.044	3.979	38	WS00945_K22
2	127.147	40	3	-0.179	0.043	4.215	38	WS00945_K22
3	9.953	42	3	0.168	0.055	5.407	38	WS00945_K22
4	103.719	81	4	-0.182	0.045	4.124	38	WS00945_K22
4	116.928	82	2	0.294	0.038	4.399	38	WS00945_K22
5	3.484	90	2	-0.240	0.060	3.914	38	WS00945_K22
6	26.350	115	2	-0.186	0.043	5.014	38	WS00945_K22
6	95.249	126	1	0.427	0.153	7.268	38	WS00945_K22
9	2.011	167	4	-0.197	0.046	4.107	38	WS00945_K22
11	64.014	222	4	-0.305	0.057	4.476	38	WS00945_K22
3	9.953	42	3	0.232	0.046	5.218	39	WS00728_A15
3	47.928	49	1	0.261	0.065	5.256	39	WS00728_A15
3	148.745	68	1	0.298	0.054	4.637	39	WS00728_A15
4	75.958	77	3	-0.335	0.069	4.625	39	WS00728_A15
5	54.072	100	4	0.288	0.057	4.358	39	WS00728_A15
6	95.249	126	1	0.409	0.143	6.902	39	WS00728_A15
10	35.204	189	2	0.187	0.037	4.484	39	WS00728_A15
10	70.246	190	2	0.264	0.035	3.919	39	WS00728_A15
10	78.479	193	3	-0.360	0.045	5.244	39	WS00728_A15
10	86.151	195	3	0.125	0.035	3.933	39	WS00728_A15
10	93.347	196	2	0.492	0.086	5.399	39	WS00728_A15
11	17.088	203	1	-0.265	0.067	5.414	39	WS00728_A15
11	29.069	205	1	0.245	0.053	4.510	39	WS00728_A15
1	22.419	4	3	0.343	0.118	6.392	40	WS00728_C16
3	9.953	42	4	0.267	0.059	4.657	40	WS00728_C16
3	14.500	43	1	0.318	0.087	7.986	40	WS00728_C16
3	17.158	45	1	-0.253	0.048	4.460	40	WS00728_C16
3	80.211	55	4	0.213	0.050	3.955	40	WS00728_C16
3	148.745	68	1	-0.315	0.066	6.086	40	WS00728_C16
3	154.505	69	1	-0.327	0.066	6.042	40	WS00728_C16
5	52.820	99	3	-0.372	0.090	7.575	40	WS00728_C16
6	81.996	123	3	-0.320	0.116	6.265	40	WS00728_C16
9	17.951	169	1	-0.347	0.070	6.457	40	WS00728_C16
9	58.045	176	1	-0.281	0.074	3.996	40	WS00728_C16
9	69.046	181	1	0.323	0.100	5.417	40	WS00728_C16
9	81.073	183	1	-0.210	0.052	4.844	40	WS00728_C16
11	45.989	216	1	-0.242	0.047	4.359	40	WS00728_C16
12	22.441	227	1	-0.220	0.047	4.328	40	WS00728_C16
12	14.406	230	4	0.342	0.066	5.315	40	WS00728_C16
2	120.617	38	3	0.251	0.058	5.943	41	WS00728_G23
3	112.868	61	2	0.225	0.035	3.869	41	WS00728_G23
6	89.401	125	1	-0.277	0.056	4.567	41	WS00728_G23
6	95.249	126	1	0.329	0.092	4.736	41	WS00728_G23
10	78.479	193	3	-0.406	0.071	7.207	41	WS00728_G23
10	86.151	195	3	0.162	0.043	4.327	41	WS00728_G23
11	27.405	204	1	0.347	0.078	6.253	41	WS00728_G23
11	29.069	205	1	0.313	0.081	6.490	41	WS00728_G23
11	45.989	216	1	-0.238	0.072	5.758	41	WS00728_G23
13	1.815	244	4	-0.210	0.047	3.947	41	WS00728_G23

1	127.805	21	4	0.237	0.058	5.513	42	WS00728_I15
4	75.958	77	3	-0.328	0.124	6.827	42	WS00728_I15
9	49.013	173	2	0.203	0.032	3.952	42	WS00728_I15
11	27.405	204	4	0.388	0.092	8.906	42	WS00728_I15
12	14.406	230	4	-0.308	0.052	5.221	42	WS00728_I15
1	45.794	9	3	-0.182	0.041	4.417	43	WS00928_H06
4	75.958	77	3	-0.211	0.069	4.286	43	WS00928_H06
7	15.555	130	2	0.312	0.038	4.737	43	WS00928_H06
11	45.989	216	1	-0.227	0.066	4.164	43	WS00928_H06
14	46.637	252	4	0.353	0.062	5.159	43	WS00928_H06
3	47.928	49	1	0.242	0.053	4.022	44	WS00928_H13
4	75.958	77	3	-0.281	0.083	5.313	44	WS00928_H13
6	89.401	125	1	-0.336	0.084	6.272	44	WS00928_H13
6	95.249	126	1	0.528	0.229	10.477	44	WS00928_H13
9	61.113	178	1	-0.350	0.207	6.075	44	WS00928_H13
9	81.073	183	1	-0.416	0.127	9.506	44	WS00928_H13
10	78.479	193	3	-0.249	0.039	4.272	44	WS00928_H13
10	80.088	194	2	-0.206	0.029	4.317	44	WS00928_H13
2	48.839	29	4	-0.255	0.065	5.543	45	WS00928_I22
9	17.951	169	1	-0.177	0.045	3.979	45	WS00928_I22
9	55.358	175	3	0.372	0.064	3.885	45	WS00928_I22
9	58.045	176	1	-0.217	0.067	3.924	45	WS00928_I22
10	70.246	190	2	-0.285	0.056	5.521	45	WS00928_I22
10	80.088	194	2	0.267	0.044	4.489	45	WS00928_I22
11	0.000	199	3	0.457	0.110	11.200	45	WS00928_I22
11	17.088	203	1	0.280	0.074	6.628	45	WS00928_I22
11	27.405	204	1	-0.391	0.090	8.049	45	WS00928_I22
11	29.069	205	1	-0.308	0.073	6.577	45	WS00928_I22
11	45.989	216	1	0.201	0.045	4.023	45	WS00928_I22
3	47.928	49	1	0.323	0.120	9.007	46	WS00928_J10
4	75.958	77	3	-0.208	0.061	4.110	46	WS00928_J10
5	31.191	95	1	-0.243	0.072	5.389	46	WS00928_J10
5	56.008	101	1	-0.221	0.063	4.765	46	WS00928_J10
7	15.555	130	2	0.343	0.051	5.724	46	WS00928_J10
7	15.555	130	3	-0.212	0.041	4.758	46	WS00928_J10
8	50.001	154	1	0.241	0.063	5.142	46	WS00928_J10
11	29.069	205	2	-0.252	0.041	4.516	46	WS00928_J10
11	40.960	210	2	0.408	0.095	4.532	46	WS00928_J10
1	107.333	19	3	0.242	0.054	4.893	47	WS00928_K17
3	14.500	43	1	0.315	0.069	5.610	47	WS00928_K17
3	17.158	45	1	-0.303	0.098	7.941	47	WS00928_K17
3	47.928	49	1	-0.242	0.067	5.438	47	WS00928_K17
12	51.382	242	1	-0.346	0.135	3.965	47	WS00928_K17
4	142.917	85	1	-0.239	0.063	4.799	48	WS00928_L03
6	26.350	115	2	-0.228	0.051	5.713	48	WS00928_L03
6	87.891	124	2	0.252	0.057	6.381	48	WS00928_L03
6	89.401	125	2	0.284	0.044	4.279	48	WS00928_L03
7	67.154	140	4	-0.348	0.134	5.284	48	WS00928_L03
7	73.169	142	1	0.493	0.099	3.888	48	WS00928_L03
7	75.883	144	4	-0.475	0.120	12.019	48	WS00928_L03
9	55.358	175	1	0.275	0.054	4.202	48	WS00928_L03
11	46.026	217	1	-0.301	0.099	7.557	48	WS00928_L03
2	48.839	29	4	0.207	0.057	4.104	49	WS00928_M11
2	127.147	40	3	-0.256	0.054	6.275	49	WS00928_M11

3	47.928	49	1	0.255	0.058	4.732	49	WS00928_M11
4	55.925	76	1	-0.288	0.068	5.896	49	WS00928_M11
4	82.462	78	1	-0.220	0.049	4.007	49	WS00928_M11
5	31.191	95	1	-0.288	0.078	6.435	49	WS00928_M11
6	95.249	126	1	0.304	0.080	4.706	49	WS00928_M11
10	80.088	194	1	0.270	0.053	4.372	49	WS00928_M11
11	27.405	204	1	0.423	0.119	9.776	49	WS00928_M11
11	29.069	205	1	0.273	0.086	7.055	49	WS00928_M11
11	40.960	210	2	0.378	0.080	4.113	49	WS00928_M11
11	45.989	216	1	-0.182	0.051	4.131	49	WS00928_M11
1	40.464	7	3	0.224	0.033	4.620	50	WS00928_M20
1	43.578	8	3	0.246	0.040	5.892	50	WS00928_M20
1	62.128	11	2	-0.247	0.045	5.080	50	WS00928_M20
2	127.147	40	3	-0.209	0.039	5.446	50	WS00928_M20
5	52.820	99	3	0.256	0.049	6.921	50	WS00928_M20
5	86.669	106	2	0.298	0.056	6.287	50	WS00928_M20
6	49.100	118	2	0.290	0.051	5.830	50	WS00928_M20
1	82.301	16	3	-0.239	0.029	4.308	51	WS00810_A03
1	107.333	19	3	0.239	0.039	5.873	51	WS00810_A03
3	95.157	59	2	0.202	0.041	5.687	51	WS00810_A03
3	104.681	60	2	-0.201	0.036	4.787	51	WS00810_A03
3	112.868	61	2	0.206	0.045	6.017	51	WS00810_A03
3	114.747	62	2	0.234	0.043	5.779	51	WS00810_A03
5	56.008	101	2	0.183	0.035	4.114	51	WS00810_A03
5	57.017	102	2	-0.248	0.037	4.858	51	WS00810_A03
6	69.073	121	3	-0.256	0.054	8.058	51	WS00810_A03
6	89.401	125	1	-0.298	0.108	5.809	51	WS00810_A03
6	95.249	126	1	0.412	0.178	7.283	51	WS00810_A03
8	87.463	158	2	-0.209	0.030	3.897	51	WS00810_A03
11	17.088	203	1	-0.249	0.079	4.255	51	WS00810_A03
11	27.405	204	1	0.361	0.104	5.660	51	WS00810_A03
11	29.069	205	1	0.330	0.124	6.716	51	WS00810_A03
12	6.242	234	2	0.202	0.031	4.433	51	WS00810_A03
3	20.512	46	2	0.170	0.042	4.582	52	WS00928_P18
6	87.891	124	2	-0.235	0.042	4.637	52	WS00928_P18
6	106.702	128	2	0.377	0.053	4.244	52	WS00928_P18
9	81.073	183	3	-0.214	0.033	4.140	52	WS00928_P18
1	69.854	13	2	-0.161	0.034	4.407	53	WS00810_F02
1	71.592	14	2	-0.180	0.042	5.437	53	WS00810_F02
1	77.177	15	2	-0.218	0.058	7.526	53	WS00810_F02
1	107.333	19	3	0.237	0.051	6.354	53	WS00810_F02
2	120.617	38	3	0.235	0.037	4.665	53	WS00810_F02
2	127.147	40	3	-0.225	0.047	5.805	53	WS00810_F02
6	26.350	115	2	-0.245	0.061	7.938	53	WS00810_F02
6	49.100	118	2	0.384	0.115	9.095	53	WS00810_F02
1	29.800	5	1	0.290	0.048	4.026	54	WS00929_C22
3	148.745	68	4	0.271	0.109	6.952	54	WS00929_C22
6	49.100	118	2	0.416	0.159	10.232	54	WS00929_C22
8	62.580	155	1	-0.337	0.267	6.395	54	WS00929_C22
8	78.389	157	1	-0.585	0.307	25.552	54	WS00929_C22
8	87.463	158	1	0.542	0.267	22.227	54	WS00929_C22
9	17.951	169	2	0.215	0.046	5.157	54	WS00929_C22
9	28.608	170	2	-0.245	0.050	5.361	54	WS00929_C22
10	35.204	189	1	0.214	0.050	4.110	54	WS00929_C22

10	70.246	190	2	-0.250	0.048	5.167	54	WS00929_C22
11	27.405	204	1	0.442	0.135	11.213	54	WS00929_C22
11	29.069	205	1	0.265	0.095	7.855	54	WS00929_C22
11	43.491	214	4	-0.276	0.086	4.844	54	WS00929_C22
11	45.989	216	1	-0.210	0.074	6.146	54	WS00929_C22
1	29.800	5	2	0.277	0.050	6.375	55	WS01021_I15
1	38.326	6	2	-0.287	0.054	7.010	55	WS01021_I15
1	40.464	7	3	0.249	0.044	5.523	55	WS01021_I15
1	43.578	8	2	0.260	0.043	5.443	55	WS01021_I15
1	62.128	11	2	-0.265	0.053	4.657	55	WS01021_I15
1	69.854	13	2	-0.184	0.035	4.506	55	WS01021_I15
1	71.592	14	2	-0.190	0.037	4.833	55	WS01021_I15
1	77.177	15	2	-0.185	0.035	4.538	55	WS01021_I15
5	0.000	89	3	-0.273	0.039	4.968	55	WS01021_I15
9	69.046	181	2	0.266	0.048	4.388	55	WS01021_I15
3	15.147	44	2	0.221	0.068	3.928	56	WS01021_N09
4	116.928	82	2	0.247	0.048	4.876	56	WS01021_N09
4	124.132	83	1	-0.487	0.108	4.936	56	WS01021_N09
7	15.555	130	2	-0.290	0.050	5.061	56	WS01021_N09
9	0.000	166	1	0.144	0.052	4.481	56	WS01021_N09
10	78.479	193	3	0.279	0.050	4.408	56	WS01021_N09
1	29.800	5	2	0.279	0.044	5.293	57	WS0102_I20
1	38.326	6	2	-0.265	0.046	5.588	57	WS0102_I20
1	95.572	17	2	0.284	0.037	4.448	57	WS0102_I20
2	93.504	32	2	0.214	0.033	3.978	57	WS0102_I20
4	103.719	81	4	0.183	0.043	3.860	57	WS0102_I20
5	31.191	95	1	0.205	0.065	4.352	57	WS0102_I20
6	25.742	113	3	0.290	0.078	8.055	57	WS0102_I20
6	69.073	121	3	-0.248	0.050	5.187	57	WS0102_I20
10	86.151	195	3	-0.240	0.058	5.965	57	WS0102_I20
1	133.992	24	2	-0.199	0.043	5.124	58	WS0102_J13
1	141.021	25	2	0.164	0.033	3.952	58	WS0102_J13
2	93.504	32	2	-0.291	0.073	8.742	58	WS0102_J13
2	102.814	36	2	-0.300	0.065	8.180	58	WS0102_J13
2	126.181	39	2	0.358	0.078	9.451	58	WS0102_J13
3	69.071	52	1	0.382	0.132	6.087	58	WS0102_J13
3	83.563	56	3	0.237	0.051	5.912	58	WS0102_J13
3	95.157	59	2	0.175	0.042	4.622	58	WS0102_J13
3	116.958	63	4	-0.266	0.090	6.441	58	WS0102_J13
5	7.672	92	4	0.292	0.080	4.780	58	WS0102_J13
7	91.487	146	3	0.159	0.043	4.914	58	WS0102_J13
8	87.463	158	2	-0.234	0.040	4.806	58	WS0102_J13
8	95.937	159	3	0.294	0.082	9.465	58	WS0102_J13
10	93.347	196	2	-0.474	0.080	5.599	58	WS0102_J13
4	6.032	72	4	-0.226	0.083	6.405	59	WS0102_L06
4	43.146	74	3	-0.234	0.038	4.296	59	WS0102_L06
6	95.249	126	1	0.374	0.088	5.558	59	WS0102_L06
8	50.001	154	1	0.280	0.058	5.809	59	WS0102_L06
10	78.479	193	3	-0.382	0.071	7.883	59	WS0102_L06
10	86.151	195	3	0.156	0.036	4.007	59	WS0102_L06
11	64.014	222	3	-0.177	0.045	4.977	59	WS0102_L06
13	0.000	243	4	-0.276	0.118	3.882	59	WS0102_L06
5	0.000	89	3	0.299	0.066	7.675	60	WS0102_L22
5	84.347	105	2	0.189	0.048	5.127	60	WS0102_L22

11	42.600	213	3	0.199	0.036	4.098	60	WS0102_L22
11	43.491	214	3	0.291	0.082	9.350	60	WS0102_L22
12	14.406	230	3	0.253	0.036	4.133	60	WS0102_L22
2	63.261	30	3	-0.218	0.040	3.886	61	WS0102_N13
2	75.550	31	2	-0.224	0.039	4.404	61	WS0102_N13
3	95.157	59	2	0.223	0.054	6.259	61	WS0102_N13
3	104.681	60	2	-0.193	0.037	4.136	61	WS0102_N13
11	43.491	214	3	0.241	0.055	5.422	61	WS0102_N13
11	64.014	222	3	-0.296	0.060	5.902	61	WS0102_N13
2	95.880	34	1	0.238	0.053	4.603	62	WS0102_O03
2	120.617	38	3	0.340	0.077	7.338	62	WS0102_O03
2	127.147	40	3	-0.335	0.099	9.424	62	WS0102_O03
3	80.211	55	4	-0.182	0.048	4.455	62	WS0102_O03
4	55.925	76	1	-0.229	0.060	4.577	62	WS0102_O03
4	82.462	78	1	-0.271	0.097	8.099	62	WS0102_O03
6	45.897	117	1	0.355	0.092	7.927	62	WS0102_O03
7	54.200	136	2	0.313	0.119	4.917	62	WS0102_O03
8	62.580	155	1	0.418	0.184	5.588	62	WS0102_O03
9	0.000	166	1	0.179	0.047	3.878	62	WS0102_O03
10	78.479	193	3	-0.302	0.054	5.080	62	WS0102_O03
14	0.000	246	2	-0.183	0.045	4.696	62	WS0102_O03
1	38.326	6	2	0.199	0.041	3.957	63	WS0102_P22
2	120.617	38	3	0.295	0.072	6.616	63	WS0102_P22
2	127.147	40	3	-0.239	0.069	6.304	63	WS0102_P22
3	47.928	49	1	0.159	0.052	4.656	63	WS0102_P22
4	82.462	78	1	-0.326	0.075	6.848	63	WS0102_P22
6	95.249	126	1	0.248	0.086	4.422	63	WS0102_P22
7	15.555	130	2	0.448	0.124	12.175	63	WS0102_P22
10	78.479	193	3	-0.344	0.059	5.661	63	WS0102_P22
10	86.151	195	3	0.191	0.042	3.899	63	WS0102_P22
10	104.214	198	2	0.264	0.047	5.757	63	WS0102_P22
1	69.854	13	2	-0.214	0.045	5.080	64	WS00712_A10
1	71.592	14	2	-0.215	0.046	5.191	64	WS00712_A10
1	77.177	15	2	-0.205	0.042	4.663	64	WS00712_A10
1	82.301	16	4	0.340	0.059	6.857	64	WS00712_A10
4	92.039	79	2	-0.152	0.044	4.810	64	WS00712_A10
4	97.576	80	2	0.244	0.063	7.499	64	WS00712_A10
8	11.974	150	1	0.236	0.051	3.861	64	WS00712_A10
10	70.246	190	2	0.188	0.062	7.126	64	WS00712_A10
10	86.151	195	3	0.286	0.060	5.505	64	WS00712_A10
10	93.347	196	2	0.456	0.084	4.561	64	WS00712_A10
3	47.928	49	1	0.276	0.074	4.248	65	WS0264_N10
4	75.958	77	3	-0.397	0.156	10.264	65	WS0264_N10
6	56.188	120	2	0.217	0.034	5.101	65	WS0264_N10
9	59.963	177	4	-0.404	0.116	10.558	65	WS0264_N10
10	80.088	194	2	-0.278	0.039	5.869	65	WS0264_N10
11	35.604	207	3	-0.155	0.044	4.175	65	WS0264_N10
11	37.143	209	3	0.153	0.043	4.093	65	WS0264_N10
11	46.026	217	1	-0.203	0.091	5.139	65	WS0264_N10
3	20.512	46	2	0.194	0.036	4.721	66	WS0264_N22
4	116.928	82	2	-0.193	0.034	4.388	66	WS0264_N22
5	52.820	99	3	0.275	0.047	4.976	66	WS0264_N22
5	56.008	101	2	-0.256	0.048	5.576	66	WS0264_N22
6	106.702	128	2	-0.406	0.054	4.146	66	WS0264_N22

11	40.960	210	2	0.373	0.065	3.965	66	WS0264_N22
13	1.815	244	2	-0.273	0.032	4.195	66	WS0264_N22
4	75.958	77	3	-0.228	0.065	3.911	67	WS0264_P03
4	103.719	81	4	-0.223	0.041	4.164	67	WS0264_P03
8	50.001	154	1	0.269	0.069	5.390	67	WS0264_P03
9	2.011	167	4	-0.294	0.071	6.868	67	WS0264_P03
10	35.204	189	1	0.285	0.084	5.941	67	WS0264_P03
11	9.237	202	4	-0.235	0.062	5.935	67	WS0264_P03
11	27.405	204	4	0.342	0.082	7.059	67	WS0264_P03
11	64.014	222	4	-0.227	0.049	4.555	67	WS0264_P03
13	0.000	243	4	0.318	0.135	5.302	67	WS0264_P03
14	43.392	250	1	-0.290	0.142	4.442	67	WS0264_P03
3	14.500	43	1	-0.180	0.048	4.040	68	WS0264_P15
3	17.158	45	1	0.233	0.049	4.194	68	WS0264_P15
4	116.928	82	2	0.272	0.048	4.928	68	WS0264_P15
4	124.132	83	2	0.291	0.068	5.449	68	WS0264_P15
7	17.610	131	3	0.404	0.148	10.330	68	WS0264_P15
8	87.463	158	1	0.239	0.057	4.795	68	WS0264_P15
9	69.046	181	2	0.321	0.067	4.420	68	WS0264_P15
10	78.479	193	3	-0.326	0.077	7.928	68	WS0264_P15
10	86.151	195	3	0.215	0.042	4.378	68	WS0264_P15
11	46.026	217	1	-0.260	0.071	6.025	68	WS0264_P15
2	120.617	38	3	0.241	0.051	4.944	69	WS00824_A17
3	47.928	49	1	0.265	0.072	5.349	69	WS00824_A17
4	75.958	77	3	-0.352	0.123	5.511	69	WS00824_A17
10	78.479	193	3	-0.333	0.045	4.382	69	WS00824_A17
11	27.405	204	1	0.307	0.068	4.979	69	WS00824_A17
11	29.069	205	1	0.228	0.064	4.730	69	WS00824_A17
1	127.805	21	4	0.280	0.047	5.212	70	WS00824_B05
3	47.928	49	1	0.252	0.052	4.288	70	WS00824_B05
6	81.996	123	4	-0.445	0.137	5.472	70	WS00824_B05
7	15.555	130	3	-0.227	0.052	4.016	70	WS00824_B05
8	62.580	155	1	0.394	0.107	4.391	70	WS00824_B05
9	55.358	175	1	-0.347	0.076	6.315	70	WS00824_B05
9	55.358	175	3	0.459	0.125	5.758	70	WS00824_B05
10	95.795	197	4	0.215	0.041	4.526	70	WS00824_B05
11	40.960	210	2	0.414	0.067	4.994	70	WS00824_B05
11	40.960	210	4	-0.349	0.056	6.373	70	WS00824_B05
12	28.493	228	2	0.365	0.078	5.508	70	WS00824_B05
1	127.805	21	4	0.272	0.057	4.630	71	WS00824_C15
2	127.147	40	3	-0.162	0.043	4.384	71	WS00824_C15
3	112.868	61	2	0.261	0.042	5.161	71	WS00824_C15
4	75.958	77	3	-0.298	0.086	4.745	71	WS00824_C15
6	89.401	125	1	-0.268	0.079	5.386	71	WS00824_C15
6	95.249	126	1	0.386	0.126	5.860	71	WS00824_C15
7	73.169	142	2	-0.393	0.062	4.006	71	WS00824_C15
7	74.560	143	2	0.240	0.040	5.136	71	WS00824_C15
7	75.883	144	2	0.382	0.063	4.029	71	WS00824_C15
9	49.013	173	2	0.216	0.053	5.763	71	WS00824_C15
11	27.405	204	1	0.321	0.064	4.479	71	WS00824_C15
1	43.578	8	2	0.282	0.043	4.739	72	WS00824_D03
4	75.958	77	3	-0.297	0.113	5.345	72	WS00824_D03
5	31.191	95	1	-0.250	0.063	4.947	72	WS00824_D03
7	15.555	130	3	-0.310	0.096	9.125	72	WS00824_D03

10	78.479	193	4	-0.221	0.049	4.510	72	WS00824_D03
2	127.147	40	3	-0.194	0.031	3.976	73	WS0104_I10
6	49.100	118	2	0.322	0.105	8.257	73	WS0104_I10
11	27.405	204	1	0.437	0.128	8.843	73	WS0104_I10
11	29.069	205	1	0.315	0.097	6.773	73	WS0104_I10
11	45.989	216	1	-0.204	0.079	5.419	73	WS0104_I10
3	47.928	49	1	0.237	0.048	4.544	74	WS0104_J02
4	0.000	71	4	-0.241	0.060	4.131	74	WS0104_J02
5	52.820	99	3	0.311	0.042	4.716	74	WS0104_J02
9	81.073	183	3	-0.253	0.048	5.467	74	WS0104_J02
12	38.543	232	4	0.333	0.149	5.454	74	WS0104_J02
2	127.147	40	3	-0.238	0.090	9.190	75	WS0104_L04
3	154.505	69	4	-0.213	0.074	6.419	75	WS0104_L04
6	95.249	126	1	0.359	0.085	4.183	75	WS0104_L04
9	28.608	170	2	-0.347	0.073	8.547	75	WS0104_L04
9	32.356	171	2	-0.264	0.050	5.724	75	WS0104_L04
9	49.013	173	2	0.276	0.095	10.220	75	WS0104_L04
11	0.000	199	2	0.263	0.044	5.320	75	WS0104_L04
11	17.088	203	1	-0.296	0.060	4.562	75	WS0104_L04
11	27.405	204	1	0.540	0.198	15.235	75	WS0104_L04
11	29.069	205	1	0.396	0.128	9.682	75	WS0104_L04
11	45.989	216	1	-0.230	0.104	7.837	75	WS0104_L04
3	17.158	45	1	0.257	0.050	4.200	76	WS0104_L23
5	52.820	99	3	0.364	0.054	6.290	76	WS0104_L23
1	107.333	19	4	-0.213	0.085	4.219	77	WS0104_N11
3	14.500	43	1	-0.238	0.059	5.294	77	WS0104_N11
3	17.158	45	1	0.262	0.057	5.143	77	WS0104_N11
6	25.914	114	1	-0.256	0.054	4.872	77	WS0104_N11
7	59.789	137	3	-0.197	0.031	3.924	77	WS0104_N11
11	27.405	204	1	0.478	0.139	12.620	77	WS0104_N11
11	29.069	205	1	0.305	0.088	8.071	77	WS0104_N11
11	45.989	216	1	-0.281	0.097	8.767	77	WS0104_N11
14	0.000	246	1	0.249	0.049	4.455	77	WS0104_N11
1	2.788	2	4	0.200	0.039	4.872	78	WS0104_N21
1	62.128	11	1	-0.418	0.060	4.108	78	WS0104_N21
3	9.953	42	4	-0.190	0.038	4.393	78	WS0104_N21
3	14.500	43	1	-0.179	0.045	4.997	78	WS0104_N21
4	92.039	79	4	-0.271	0.038	4.280	78	WS0104_N21
4	124.132	83	1	-0.650	0.134	9.136	78	WS0104_N21
5	54.072	100	4	0.220	0.040	4.501	78	WS0104_N21
5	57.017	102	4	-0.316	0.043	4.867	78	WS0104_N21
9	36.227	165	4	-0.282	0.048	5.443	78	WS0104_N21
9	28.608	170	2	-0.231	0.059	4.632	78	WS0104_N21
11	29.069	205	1	0.255	0.038	4.265	78	WS0104_N21
12	38.543	232	4	0.342	0.103	7.012	78	WS0104_N21
1	107.333	19	3	0.204	0.036	3.950	79	WS0105_A11
4	75.958	77	3	-0.310	0.070	4.041	79	WS0105_A11
5	31.191	95	1	-0.189	0.060	4.393	79	WS0105_A11
6	25.914	114	1	-0.202	0.061	4.448	79	WS0105_A11
10	35.204	189	1	0.329	0.080	5.912	79	WS0105_A11
11	37.143	209	3	0.182	0.038	4.155	79	WS0105_A11
11	42.600	213	3	-0.184	0.035	3.916	79	WS0105_A11
14	43.392	250	1	-0.330	0.094	5.049	79	WS0105_A11
11	27.405	204	1	0.301	0.058	4.897	80	WS0105_B03

11	29.069	205	1	0.248	0.063	5.319	80	WS0105_B03
11	45.989	216	1	-0.205	0.056	4.671	80	WS0105_B03
11	46.026	217	1	-0.223	0.058	4.880	80	WS0105_B03
1	29.800	5	1	0.238	0.060	4.725	81	WS00731_I15
1	38.326	6	2	0.275	0.049	5.311	81	WS00731_I15
1	71.592	14	2	0.206	0.035	3.890	81	WS00731_I15
1	77.177	15	2	0.221	0.042	4.576	81	WS00731_I15
1	107.333	19	3	0.236	0.038	4.931	81	WS00731_I15
3	47.928	49	1	0.247	0.050	4.001	81	WS00731_I15
4	0.000	71	4	-0.197	0.068	4.067	81	WS00731_I15
6	89.401	125	1	-0.255	0.055	4.300	81	WS00731_I15
10	78.479	193	3	-0.302	0.054	6.807	81	WS00731_I15
11	43.491	214	3	0.175	0.037	4.744	81	WS00731_I15
11	64.014	222	3	-0.196	0.044	5.619	81	WS00731_I15
9	59.963	177	4	-0.298	0.060	4.369	82	WS00112_M03
10	78.479	193	3	-0.469	0.103	11.762	82	WS00112_M03
10	86.151	195	3	0.233	0.059	6.755	82	WS00112_M03
14	46.637	252	4	0.357	0.108	6.795	82	WS00112_M03
1	29.800	5	1	0.215	0.059	4.871	83	WS00732_E21
1	107.333	19	3	0.235	0.040	4.480	83	WS00732_E21
4	6.032	72	4	-0.173	0.054	4.060	83	WS00732_E21
6	56.188	120	2	0.222	0.035	3.913	83	WS00732_E21
6	95.249	126	1	0.385	0.088	5.343	83	WS00732_E21
9	28.608	170	2	-0.261	0.042	4.554	83	WS00732_E21
11	29.069	205	1	0.283	0.049	4.091	83	WS00732_E21
11	64.014	222	3	-0.151	0.040	4.494	83	WS00732_E21
1	69.854	13	2	-0.180	0.035	4.048	84	WS00113_G22
1	71.592	14	2	-0.191	0.040	4.588	84	WS00113_G22
2	63.261	30	3	0.217	0.059	5.794	84	WS00113_G22
4	116.928	82	2	0.319	0.048	5.620	84	WS00113_G22
4	124.132	83	2	0.359	0.097	5.018	84	WS00113_G22
6	25.914	114	1	-0.224	0.056	4.087	84	WS00113_G22
6	45.897	117	1	0.239	0.072	5.347	84	WS00113_G22
11	29.069	205	1	0.275	0.092	6.779	84	WS00113_G22
1	107.333	19	3	0.283	0.063	7.190	85	WS01040_A15
2	120.617	38	3	0.259	0.063	7.043	85	WS01040_A15
2	127.147	40	3	-0.144	0.035	3.932	85	WS01040_A15
3	47.928	49	1	0.316	0.084	7.684	85	WS01040_A15
4	75.958	77	3	-0.366	0.140	7.196	85	WS01040_A15
5	31.191	95	1	-0.244	0.052	4.762	85	WS01040_A15
6	89.401	125	1	-0.283	0.046	4.232	85	WS01040_A15
6	95.249	126	1	0.388	0.094	5.841	85	WS01040_A15
8	50.001	154	1	0.243	0.044	4.660	85	WS01040_A15
9	0.000	166	2	0.237	0.061	5.501	85	WS01040_A15
9	2.011	167	4	-0.213	0.060	4.504	85	WS01040_A15
9	55.358	175	3	0.462	0.089	4.118	85	WS01040_A15
10	78.479	193	3	-0.295	0.041	4.673	85	WS01040_A15
11	29.069	205	1	0.302	0.061	5.665	85	WS01040_A15
11	45.989	216	1	-0.206	0.053	4.831	85	WS01040_A15
2	126.181	39	4	-0.725	0.216	13.842	86	WS01040_I17
4	82.462	78	1	-0.212	0.059	5.174	86	WS01040_I17
4	142.917	85	1	0.322	0.074	6.533	86	WS01040_I17
5	3.484	90	2	0.297	0.084	5.311	86	WS01040_I17
5	56.008	101	1	-0.266	0.053	4.751	86	WS01040_I17

8	50.001	154	1	0.297	0.075	6.929	86	WS01040_I17
9	55.358	175	1	-0.280	0.044	3.966	86	WS01040_I17
10	21.864	188	1	0.233	0.065	5.713	86	WS01040_I17
10	104.214	198	4	0.275	0.029	4.540	86	WS01040_I17
11	43.491	214	4	-0.277	0.060	6.337	86	WS01040_I17
11	44.884	215	4	0.318	0.054	5.459	86	WS01040_I17
11	48.979	218	4	-0.311	0.053	5.150	86	WS01040_I17
11	57.661	220	4	0.383	0.145	15.369	86	WS01040_I17
11	64.014	222	4	-0.441	0.144	13.125	86	WS01040_I17
12	14.406	230	3	-0.228	0.059	4.796	86	WS01040_I17
3	15.147	44	2	-0.182	0.072	4.402	87	WS0015_D01
3	69.071	52	1	-0.311	0.120	4.080	87	WS0015_D01
4	116.928	82	4	0.288	0.039	4.447	87	WS0015_D01
6	56.188	120	2	0.272	0.044	4.575	87	WS0015_D01
7	15.555	130	2	0.330	0.053	5.774	87	WS0015_D01
7	15.555	130	3	-0.273	0.067	5.922	87	WS0015_D01
12	38.543	232	4	0.314	0.099	4.627	87	WS0015_D01
1	107.333	19	3	0.267	0.047	5.791	88	WS0081_C01
4	6.032	72	4	-0.154	0.066	4.454	88	WS0081_C01
6	89.401	125	1	-0.255	0.040	4.034	88	WS0081_C01
6	95.249	126	1	0.452	0.109	8.300	88	WS0081_C01
11	0.000	199	3	-0.383	0.069	7.790	88	WS0081_C01
11	27.405	204	1	0.536	0.156	16.160	88	WS0081_C01
11	29.069	205	1	0.419	0.117	11.954	88	WS0081_C01
11	45.989	216	1	-0.300	0.109	10.992	88	WS0081_C01
14	22.402	248	3	0.203	0.049	5.811	88	WS0081_C01
3	15.147	44	2	-0.317	0.097	5.989	89	WS0015_P14
4	75.958	77	3	-0.271	0.082	4.581	89	WS0015_P14
7	15.555	130	3	-0.232	0.069	5.466	89	WS0015_P14
9	59.963	177	4	-0.230	0.049	5.283	89	WS0015_P14
9	81.073	183	1	-0.260	0.086	5.499	89	WS0015_P14
11	45.989	216	1	-0.215	0.063	4.034	89	WS0015_P14
1	127.805	21	4	0.221	0.052	4.364	90	WS0081_J24
2	48.839	29	4	0.289	0.066	5.683	90	WS0081_J24
3	47.928	49	1	0.287	0.072	7.114	90	WS0081_J24
5	57.017	102	2	0.224	0.047	4.129	90	WS0081_J24
8	0.000	148	4	-0.213	0.051	4.540	90	WS0081_J24
8	87.463	158	2	0.222	0.059	4.238	90	WS0081_J24
9	49.013	173	1	0.222	0.039	4.033	90	WS0081_J24
9	81.073	183	1	0.368	0.082	8.497	90	WS0081_J24
9	81.073	183	3	-0.273	0.058	5.752	90	WS0081_J24
11	35.604	207	3	-0.374	0.149	14.809	90	WS0081_J24
11	37.143	209	3	0.396	0.166	16.484	90	WS0081_J24
11	42.600	213	3	-0.337	0.112	11.136	90	WS0081_J24
3	47.928	49	1	0.263	0.063	4.601	91	WS00929_G04
4	75.958	77	3	-0.339	0.095	6.328	91	WS00929_G04
5	52.820	99	3	0.248	0.042	4.676	91	WS00929_G04
9	59.963	177	4	-0.314	0.091	5.213	91	WS00929_G04
10	80.088	194	2	-0.270	0.049	5.493	91	WS00929_G04
11	27.405	204	1	0.328	0.063	4.763	91	WS00929_G04
11	29.069	205	1	0.304	0.074	5.431	91	WS00929_G04
11	45.989	216	1	-0.171	0.053	3.864	91	WS00929_G04
11	56.921	219	2	0.282	0.065	4.209	91	WS00929_G04
1	127.805	21	4	0.213	0.046	4.207	92	WS00929_J04

3	9.953	42	4	-0.218	0.048	4.358	92	WS00929_J04
4	116.928	82	4	0.308	0.052	4.371	92	WS00929_J04
4	173.929	88	2	-0.201	0.041	4.676	92	WS00929_J04
5	7.672	92	4	0.222	0.044	4.074	92	WS00929_J04
6	87.891	124	2	0.206	0.039	4.451	92	WS00929_J04
7	15.555	130	3	-0.286	0.065	6.038	92	WS00929_J04
9	59.963	177	4	-0.239	0.047	4.309	92	WS00929_J04
11	27.405	204	4	0.263	0.049	4.341	92	WS00929_J04
11	37.143	209	3	0.201	0.042	3.964	92	WS00929_J04
13	3.386	245	1	0.277	0.091	6.831	92	WS00929_J04
2	63.261	30	3	0.246	0.052	4.301	93	WS0019_J21
9	17.951	169	2	0.191	0.056	6.988	93	WS0019_J21
9	28.608	170	2	-0.406	0.099	11.314	93	WS0019_J21
9	28.608	170	3	0.308	0.065	5.488	93	WS0019_J21
9	49.013	173	2	0.185	0.066	5.328	93	WS0019_J21
9	81.073	183	1	0.229	0.068	5.609	93	WS0019_J21
10	95.795	197	4	0.212	0.056	6.046	93	WS0019_J21
11	17.088	203	1	-0.311	0.052	4.354	93	WS0019_J21
11	27.405	204	1	0.332	0.073	5.957	93	WS0019_J21
11	29.069	205	1	0.217	0.049	3.983	93	WS0019_J21
11	43.491	214	4	-0.180	0.044	4.568	93	WS0019_J21
11	44.884	215	4	0.265	0.034	4.136	93	WS0019_J21
11	56.921	219	1	-0.435	0.137	4.685	93	WS0019_J21
11	57.661	220	4	0.122	0.040	4.246	93	WS0019_J21
11	64.014	222	4	-0.225	0.046	5.474	93	WS0019_J21
1	131.686	23	2	0.464	0.088	5.308	94	WS0019_L07
1	133.992	24	2	0.271	0.065	6.688	94	WS0019_L07
1	141.021	25	2	-0.207	0.040	4.085	94	WS0019_L07
2	95.880	34	1	0.223	0.046	3.933	94	WS0019_L07
3	148.745	68	1	-0.204	0.047	4.045	94	WS0019_L07
4	43.146	74	3	0.235	0.064	6.166	94	WS0019_L07
4	55.925	76	1	0.216	0.048	4.211	94	WS0019_L07
5	54.072	100	4	-0.190	0.044	4.034	94	WS0019_L07
7	17.610	131	3	0.282	0.097	4.066	94	WS0019_L07
11	27.405	204	4	0.415	0.129	10.012	94	WS0019_L07
12	14.406	230	3	-0.238	0.048	4.640	94	WS0019_L07
12	6.242	234	2	-0.265	0.040	4.107	94	WS0019_L07
5	31.191	95	1	-0.249	0.035	3.940	95	WS0019_O24
5	42.979	97	1	0.296	0.047	5.264	95	WS0019_O24
10	78.479	193	4	-0.199	0.054	5.061	95	WS0019_O24
11	27.405	204	1	0.437	0.082	9.405	95	WS0019_O24
11	29.069	205	1	0.326	0.066	7.406	95	WS0019_O24
11	45.989	216	1	-0.195	0.060	6.600	95	WS0019_O24
14	0.000	246	1	0.319	0.054	6.151	95	WS0019_O24
3	47.928	49	1	-0.226	0.083	7.419	96	WS00110_A20
6	49.100	118	4	0.274	0.106	4.116	96	WS00110_A20
6	56.188	120	1	-0.269	0.049	4.410	96	WS00110_A20
6	81.996	123	4	0.278	0.103	4.270	96	WS00110_A20
9	2.011	167	4	0.149	0.044	3.907	96	WS00110_A20
11	27.405	204	1	0.337	0.064	5.792	96	WS00110_A20
11	29.069	205	1	0.207	0.047	4.197	96	WS00110_A20
11	45.989	216	1	-0.156	0.048	4.241	96	WS00110_A20
14	22.402	248	4	0.228	0.055	5.044	96	WS00110_A20
1	40.464	7	4	-0.261	0.046	4.312	97	WS00110_D11

3	137.891	64	1	-0.276	0.053	4.588	97	WS00110_D11
3	148.745	68	1	0.270	0.049	4.472	97	WS00110_D11
5	7.672	92	4	-0.335	0.078	6.502	97	WS00110_D11
5	52.820	99	3	0.287	0.056	4.933	97	WS00110_D11
8	0.000	148	4	-0.159	0.049	4.100	97	WS00110_D11
8	95.937	159	3	0.236	0.041	4.278	97	WS00110_D11
9	28.608	170	3	0.287	0.053	5.807	97	WS00110_D11
10	9.580	187	3	0.195	0.076	4.438	97	WS00110_D11
12	14.406	230	3	-0.283	0.042	4.406	97	WS00110_D11
14	0.000	246	2	-0.257	0.056	6.227	97	WS00110_D11
4	116.928	82	4	0.324	0.042	4.791	98	WS00110_E22
4	173.929	88	2	-0.156	0.050	4.108	98	WS00110_E22
11	0.000	199	2	0.239	0.052	4.309	98	WS00110_E22
11	40.960	210	4	-0.296	0.062	5.496	98	WS00110_E22
11	44.884	215	4	0.245	0.039	4.075	98	WS00110_E22
11	48.979	218	4	-0.262	0.040	4.183	98	WS00110_E22
11	57.661	220	4	0.255	0.055	5.648	98	WS00110_E22
12	38.543	232	4	0.546	0.269	12.295	98	WS00110_E22
1	107.333	19	3	0.279	0.056	4.624	99	WS00110_J06
4	6.032	72	4	-0.166	0.047	4.941	99	WS00110_J06
6	69.073	121	3	-0.255	0.052	4.264	99	WS00110_J06
6	89.401	125	1	-0.369	0.078	6.715	99	WS00110_J06
6	95.249	126	1	0.373	0.114	4.696	99	WS00110_J06
7	15.555	130	2	0.250	0.052	5.295	99	WS00110_J06
7	74.560	143	1	-0.178	0.046	3.958	99	WS00110_J06
8	50.001	154	1	0.289	0.077	6.984	99	WS00110_J06
10	78.479	193	3	-0.332	0.060	4.897	99	WS00110_J06
11	17.088	203	1	-0.244	0.055	4.670	99	WS00110_J06
11	29.069	205	1	0.308	0.049	4.338	99	WS00110_J06
4	116.928	82	2	-0.193	0.034	4.324	100	WS00110_K19
6	56.188	120	2	0.270	0.044	4.828	100	WS00110_K19
9	59.963	177	4	-0.284	0.077	4.990	100	WS00110_K19
10	78.479	193	4	-0.245	0.072	4.950	100	WS00110_K19
11	17.088	203	1	-0.273	0.065	4.133	100	WS00110_K19
11	29.069	205	1	0.291	0.068	4.417	100	WS00110_K19
4	0.000	71	4	-0.171	0.042	4.272	101	WS0016_H06
7	74.560	143	1	-0.302	0.122	8.914	101	WS0016_H06
9	59.963	177	4	-0.311	0.070	6.912	101	WS0016_H06
11	64.014	222	4	0.308	0.052	3.922	101	WS0016_H06
1	107.333	19	3	0.203	0.061	5.823	102	WS0016_J03
3	9.953	42	4	-0.536	0.283	22.923	102	WS0016_J03
3	17.158	45	1	-0.310	0.127	11.986	102	WS0016_J03
3	137.891	64	1	0.370	0.049	4.680	102	WS0016_J03
6	56.188	120	2	0.287	0.042	3.952	102	WS0016_J03
9	59.963	177	4	-0.244	0.051	4.700	102	WS0016_J03
11	9.237	202	4	-0.198	0.051	4.644	102	WS0016_J03
12	22.441	227	1	0.334	0.096	9.517	102	WS0016_J03
14	46.637	252	4	0.217	0.046	4.396	102	WS0016_J03
4	6.032	72	4	-0.137	0.042	4.505	103	WS0016_M15
10	70.246	190	4	-0.342	0.053	5.719	103	WS0016_M15
11	17.088	203	1	-0.209	0.046	3.946	103	WS0016_M15
11	29.069	205	1	0.342	0.071	6.348	103	WS0016_M15
11	45.989	216	1	-0.256	0.069	5.925	103	WS0016_M15
3	144.593	66	4	-0.224	0.044	3.939	104	WS0016_O17

6	56.188	120	2	0.402	0.090	10.718	104	WS0016_O17
7	15.555	130	3	-0.264	0.067	6.052	104	WS0016_O17
11	17.088	203	1	-0.304	0.088	6.124	104	WS0016_O17
11	29.069	205	1	0.215	0.069	4.772	104	WS0016_O17
1	50.955	10	1	-0.199	0.059	3.955	105	WS0017_D24
1	62.128	11	1	-0.593	0.171	6.710	105	WS0017_D24
2	101.076	35	3	0.250	0.052	3.891	105	WS0017_D24
2	120.617	38	3	0.202	0.066	4.933	105	WS0017_D24
2	127.147	40	3	-0.199	0.065	4.813	105	WS0017_D24
3	80.211	55	4	-0.239	0.041	4.702	105	WS0017_D24
12	14.406	230	4	-0.225	0.034	3.955	105	WS0017_D24
12	38.543	232	4	0.433	0.206	8.097	105	WS0017_D24
13	3.386	245	1	0.242	0.057	3.857	105	WS0017_D24
1	107.333	19	3	0.229	0.043	4.539	106	WS0017_E15
2	120.617	38	3	0.189	0.041	4.286	106	WS0017_E15
3	47.928	49	1	0.272	0.065	5.373	106	WS0017_E15
9	36.227	165	4	-0.246	0.055	4.666	106	WS0017_E15
9	59.963	177	4	-0.254	0.053	3.907	106	WS0017_E15
11	0.000	199	3	-0.296	0.040	3.986	106	WS0017_E15
11	17.088	203	1	-0.246	0.051	4.218	106	WS0017_E15
11	29.069	205	1	0.304	0.070	5.774	106	WS0017_E15
11	45.989	216	1	-0.186	0.052	4.241	106	WS0017_E15
11	56.921	219	2	0.282	0.093	3.857	106	WS0017_E15
2	120.617	38	3	0.211	0.063	5.444	107	WS0017_I01
3	14.500	43	1	-0.167	0.044	3.960	107	WS0017_I01
3	17.158	45	1	0.214	0.044	3.976	107	WS0017_I01
3	47.928	49	1	0.203	0.042	3.845	107	WS0017_I01
6	95.249	126	1	0.442	0.187	7.971	107	WS0017_I01
9	59.963	177	4	-0.301	0.071	5.154	107	WS0017_I01
10	104.214	198	4	-0.310	0.049	4.203	107	WS0017_I01
11	17.088	203	2	-0.232	0.052	4.607	107	WS0017_I01
3	95.157	59	2	0.152	0.035	3.905	108	WS0017_J07
3	104.681	60	2	-0.234	0.039	4.526	108	WS0017_J07
5	84.347	105	1	0.255	0.059	4.404	108	WS0017_J07
6	87.891	124	2	0.253	0.044	5.091	108	WS0017_J07
7	17.610	131	3	-0.397	0.099	7.306	108	WS0017_J07
9	2.011	167	4	-0.261	0.057	4.407	108	WS0017_J07
10	78.479	193	3	0.338	0.052	5.802	108	WS0017_J07
10	86.151	195	3	-0.212	0.037	4.166	108	WS0017_J07
14	0.000	246	2	-0.208	0.034	4.154	108	WS0017_J07
2	127.147	40	3	0.256	0.064	5.723	109	WS00729_E06
3	14.500	43	1	0.263	0.091	5.506	109	WS00729_E06
3	15.147	44	2	0.299	0.097	7.087	109	WS00729_E06
3	17.158	45	1	-0.264	0.084	5.070	109	WS00729_E06
5	0.000	89	3	0.219	0.058	5.130	109	WS00729_E06
11	35.604	207	3	0.291	0.085	7.658	109	WS00729_E06
11	37.143	209	3	-0.281	0.079	7.095	109	WS00729_E06
11	42.600	213	3	0.238	0.053	4.790	109	WS00729_E06
1	29.800	5	2	-0.202	0.041	4.698	110	WS00834_D02
1	38.326	6	2	0.289	0.044	4.864	110	WS00834_D02
2	126.181	39	4	-0.529	0.128	5.882	110	WS00834_D02
6	45.897	117	4	-0.249	0.062	6.348	110	WS00834_D02
6	54.066	119	4	-0.314	0.086	8.083	110	WS00834_D02
9	0.000	166	2	0.189	0.037	4.221	110	WS00834_D02

9	2.011	167	4	-0.283	0.059	5.557	110	WS00834_D02
10	78.479	193	3	-0.259	0.060	5.601	110	WS00834_D02
11	9.237	202	4	-0.291	0.072	6.809	110	WS00834_D02
11	27.405	204	1	0.394	0.102	7.667	110	WS00834_D02
11	29.069	205	1	0.235	0.057	4.286	110	WS00834_D02
11	35.604	207	3	-0.227	0.063	5.897	110	WS00834_D02
11	37.143	209	3	0.232	0.065	6.156	110	WS00834_D02
11	41.984	212	2	-0.253	0.046	5.252	110	WS00834_D02
11	42.600	213	3	-0.195	0.044	4.121	110	WS00834_D02
3	141.443	65	3	0.325	0.183	4.960	111	WS00940_C15
3	154.505	69	1	-0.221	0.075	4.511	111	WS00940_C15
3	154.505	69	4	0.275	0.040	4.238	111	WS00940_C15
4	97.576	80	2	0.254	0.048	5.668	111	WS00940_C15
7	105.477	147	2	-0.215	0.066	4.725	111	WS00940_C15
12	22.441	227	3	0.327	0.100	8.593	111	WS00940_C15
14	22.402	248	4	-0.467	0.093	9.326	111	WS00940_C15
14	46.637	252	4	-0.463	0.096	10.058	111	WS00940_C15
2	120.617	38	3	0.253	0.064	6.771	112	WS00834_N05
2	127.147	40	3	-0.228	0.086	9.085	112	WS00834_N05
3	9.953	42	3	0.216	0.041	4.326	112	WS00834_N05
5	31.191	95	1	-0.233	0.050	4.368	112	WS00834_N05
6	95.249	126	1	0.394	0.102	6.627	112	WS00834_N05
8	62.580	155	1	0.277	0.180	4.104	112	WS00834_N05
11	0.000	199	3	-0.309	0.058	4.485	112	WS00834_N05
11	27.405	204	1	0.525	0.185	16.673	112	WS00834_N05
11	29.069	205	1	0.382	0.114	10.009	112	WS00834_N05
11	37.143	209	3	0.144	0.044	4.597	112	WS00834_N05
11	45.989	216	1	-0.292	0.102	8.916	112	WS00834_N05
4	43.146	74	3	0.283	0.032	4.007	113	WS0094_G23
6	56.188	120	1	0.304	0.039	4.080	113	WS0094_G23
11	40.960	210	2	0.531	0.144	8.586	113	WS0094_G23
12	0.000	226	3	0.233	0.056	6.180	113	WS0094_G23
14	43.392	250	1	-0.440	0.098	6.650	113	WS0094_G23
2	127.147	40	3	-0.232	0.050	5.181	114	WS0094_L01
3	14.500	43	1	-0.350	0.073	7.512	114	WS0094_L01
3	15.147	44	2	0.337	0.111	5.525	114	WS0094_L01
3	17.158	45	1	0.309	0.085	8.789	114	WS0094_L01
3	154.505	69	1	0.253	0.057	5.805	114	WS0094_L01
4	55.925	76	1	-0.358	0.108	11.066	114	WS0094_L01
5	31.191	95	1	-0.198	0.039	4.006	114	WS0094_L01
6	87.891	124	2	-0.201	0.046	3.909	114	WS0094_L01
6	95.249	126	1	0.258	0.069	5.061	114	WS0094_L01
9	36.227	165	3	-0.211	0.041	4.282	114	WS0094_L01
10	77.676	192	1	0.275	0.071	7.342	114	WS0094_L01
10	80.088	194	1	0.385	0.105	10.840	114	WS0094_L01
11	27.405	204	1	0.299	0.073	7.490	114	WS0094_L01
11	45.989	216	1	-0.223	0.047	4.859	114	WS0094_L01
11	64.014	222	3	-0.295	0.060	6.264	114	WS0094_L01
14	22.402	248	3	0.267	0.042	4.438	114	WS0094_L01
6	56.188	120	2	0.278	0.040	5.326	115	WS0018_D18
9	0.000	166	2	-0.199	0.033	4.325	115	WS0018_D18
11	27.405	204	1	0.306	0.084	4.757	115	WS0018_D18
14	0.000	246	1	0.299	0.079	4.525	115	WS0018_D18
2	127.147	40	3	-0.142	0.043	4.299	116	WS00940_O14

3	9.953	42	3	0.251	0.070	6.951	116	WS00940_O14
3	9.953	42	4	0.192	0.047	4.071	116	WS00940_O14
3	47.928	49	1	0.246	0.041	5.097	116	WS00940_O14
4	75.958	77	3	-0.329	0.153	3.957	116	WS00940_O14
5	31.191	95	1	-0.276	0.056	6.880	116	WS00940_O14
6	95.249	126	1	0.451	0.118	8.818	116	WS00940_O14
11	27.405	204	1	0.438	0.090	11.248	116	WS00940_O14
11	29.069	205	1	0.317	0.055	6.787	116	WS00940_O14
11	45.989	216	1	-0.267	0.074	9.006	116	WS00940_O14
14	0.000	246	1	0.260	0.042	5.238	116	WS00940_O14
1	107.333	19	3	0.190	0.043	3.922	117	WS0019_C10
1	123.316	20	4	-0.310	0.057	3.843	117	WS0019_C10
2	126.181	39	4	0.549	0.125	7.981	117	WS0019_C10
3	9.953	42	4	-0.128	0.042	4.026	117	WS0019_C10
7	62.787	139	4	-0.212	0.055	5.600	117	WS0019_C10
8	50.001	154	2	-0.236	0.079	7.998	117	WS0019_C10
10	9.580	187	2	0.222	0.066	4.346	117	WS0019_C10
11	46.026	217	1	-0.241	0.044	3.854	117	WS0019_C10
11	56.921	219	2	-0.285	0.106	4.008	117	WS0019_C10
13	1.815	244	2	-0.230	0.040	4.139	117	WS0019_C10
14	43.392	250	1	-0.281	0.097	5.542	117	WS0019_C10
2	127.147	40	3	-0.237	0.060	5.669	118	WS00941_H03
3	9.953	42	3	0.228	0.074	7.054	118	WS00941_H03
3	15.147	44	2	0.228	0.058	4.303	118	WS00941_H03
3	20.512	46	2	0.213	0.044	4.637	118	WS00941_H03
4	55.925	76	1	-0.278	0.051	4.043	118	WS00941_H03
5	31.191	95	1	-0.288	0.063	5.300	118	WS00941_H03
6	95.249	126	1	0.313	0.072	4.724	118	WS00941_H03
11	27.405	204	1	0.358	0.089	7.492	118	WS00941_H03
1	29.800	5	1	0.237	0.047	4.812	119	WS00923_E18
1	95.572	17	3	0.215	0.043	5.353	119	WS00923_E18
1	107.333	19	3	0.209	0.034	4.298	119	WS00923_E18
2	102.814	36	1	0.199	0.043	4.402	119	WS00923_E18
8	50.001	154	1	0.268	0.054	5.566	119	WS00923_E18
10	9.580	187	3	-0.378	0.069	5.461	119	WS00923_E18
10	78.479	193	3	-0.256	0.037	4.602	119	WS00923_E18
11	29.069	205	1	0.274	0.051	5.271	119	WS00923_E18
11	45.989	216	1	-0.185	0.039	3.975	119	WS00923_E18
1	133.992	24	2	0.183	0.058	4.940	120	WS00923_I04
4	75.958	77	3	-0.350	0.084	4.305	120	WS00923_I04
6	95.249	126	1	0.421	0.157	8.103	120	WS00923_I04
10	78.479	193	3	-0.355	0.041	4.228	120	WS00923_I04
11	29.069	205	1	0.299	0.044	4.574	120	WS00923_I04
11	64.014	222	3	-0.225	0.054	5.503	120	WS00923_I04
1	77.177	15	2	-0.173	0.036	4.885	121	WS0022_B01
4	75.958	77	3	-0.336	0.129	7.154	121	WS0022_B01
9	59.963	177	4	-0.292	0.070	7.815	121	WS0022_B01
11	60.464	221	2	0.239	0.033	4.133	121	WS0022_B01
11	64.014	222	4	0.287	0.056	6.009	121	WS0022_B01
12	6.242	234	2	0.253	0.042	6.343	121	WS0022_B01
14	22.402	248	4	0.275	0.042	4.806	121	WS0022_B01
1	45.794	9	1	-0.233	0.071	5.298	122	WS0022_B23
1	62.128	11	2	0.270	0.076	4.787	122	WS0022_B23
9	59.963	177	4	-0.211	0.052	4.110	122	WS0022_B23

10	80.088	194	2	-0.245	0.033	3.954	122	WS0022_B23
11	56.921	219	2	0.311	0.098	6.724	122	WS0022_B23
4	75.958	77	3	-0.289	0.080	4.846	123	WS0022_F17
6	25.914	114	1	-0.233	0.047	3.943	123	WS0022_F17
7	75.883	144	4	-0.392	0.080	6.055	123	WS0022_F17
11	40.960	210	2	-0.392	0.092	4.322	123	WS0022_F17
11	46.026	217	1	-0.341	0.125	10.448	123	WS0022_F17
1	82.301	16	3	-0.248	0.045	3.999	124	WS0022_G14
3	9.953	42	4	0.245	0.059	6.311	124	WS0022_G14
3	47.928	49	1	0.263	0.073	4.238	124	WS0022_G14
5	31.191	95	1	-0.253	0.070	4.060	124	WS0022_G14
6	56.188	120	2	0.228	0.029	4.408	124	WS0022_G14
7	15.555	130	3	-0.199	0.069	4.451	124	WS0022_G14
10	35.204	189	2	-0.195	0.030	4.208	124	WS0022_G14
2	127.147	40	3	-0.169	0.040	4.171	125	WS0022_I13
3	83.563	56	3	-0.178	0.039	4.072	125	WS0022_I13
6	95.249	126	1	0.360	0.101	4.210	125	WS0022_I13
7	17.610	131	3	-0.350	0.084	5.446	125	WS0022_I13
7	75.883	144	2	0.423	0.081	5.241	125	WS0022_I13
9	59.963	177	4	-0.216	0.046	3.911	125	WS0022_I13
11	17.088	203	2	-0.167	0.035	4.738	125	WS0022_I13
11	29.069	205	2	-0.248	0.058	6.959	125	WS0022_I13
11	35.604	207	3	-0.162	0.039	4.055	125	WS0022_I13
12	22.441	227	1	0.279	0.080	4.954	125	WS0022_I13
7	15.555	130	3	-0.292	0.061	6.205	126	WS0022_J05
7	59.789	137	3	-0.255	0.076	8.261	126	WS0022_J05
7	73.169	142	2	-0.490	0.096	4.704	126	WS0022_J05
7	74.560	143	2	0.312	0.066	7.341	126	WS0022_J05
7	75.883	144	2	0.498	0.107	6.729	126	WS0022_J05
10	78.479	193	4	-0.194	0.061	4.851	126	WS0022_J05
1	2.788	2	4	-0.203	0.047	5.867	127	WS0022_L16
1	131.686	23	2	0.396	0.055	6.066	127	WS0022_L16
1	133.992	24	2	0.250	0.041	6.275	127	WS0022_L16
1	141.021	25	2	-0.209	0.029	4.446	127	WS0022_L16
3	80.211	55	4	0.171	0.033	4.157	127	WS0022_L16
6	26.350	115	2	0.227	0.039	5.918	127	WS0022_L16
11	57.661	220	4	0.156	0.031	3.872	127	WS0022_L16
11	64.014	222	4	-0.252	0.043	4.563	127	WS0022_L16
1	62.128	11	2	0.294	0.088	3.992	128	WS0022_M11
1	131.686	23	1	-0.645	0.131	7.071	128	WS0022_M11
3	144.593	66	4	-0.183	0.050	5.493	128	WS0022_M11
3	154.505	69	4	-0.294	0.065	7.058	128	WS0022_M11
4	124.132	83	1	0.447	0.077	4.142	128	WS0022_M11
4	124.132	83	2	-0.270	0.088	4.937	128	WS0022_M11
4	142.917	85	1	-0.195	0.053	4.629	128	WS0022_M11
8	0.000	148	4	0.248	0.045	4.665	128	WS0022_M11
9	81.073	183	1	-0.206	0.052	4.518	128	WS0022_M11
11	45.989	216	1	-0.228	0.048	4.194	128	WS0022_M11
14	46.637	252	4	0.427	0.077	7.082	128	WS0022_M11
1	127.805	21	4	0.200	0.061	4.460	129	WS00821_E14
3	9.953	42	3	0.174	0.044	5.147	129	WS00821_E14
3	47.928	49	1	0.250	0.047	4.720	129	WS00821_E14
3	144.647	67	1	0.359	0.110	3.923	129	WS00821_E14
6	56.188	120	2	0.232	0.048	4.171	129	WS00821_E14

6	81.996	123	4	-0.253	0.105	3.928	129	WS00821_E14
6	95.249	126	1	0.376	0.083	5.371	129	WS00821_E14
9	17.951	169	2	0.215	0.045	4.133	129	WS00821_E14
9	28.608	170	2	-0.267	0.068	6.134	129	WS00821_E14
9	32.356	171	2	-0.218	0.052	4.585	129	WS00821_E14
11	27.405	204	1	0.467	0.099	10.046	129	WS00821_E14
11	29.069	205	1	0.352	0.080	8.018	129	WS00821_E14
11	45.989	216	1	-0.299	0.104	10.313	129	WS00821_E14
11	60.464	221	1	0.263	0.054	5.409	129	WS00821_E14
11	64.014	222	3	-0.159	0.033	3.934	129	WS00821_E14
1	45.794	9	1	-0.223	0.058	4.180	130	WS00821_F01
3	15.147	44	2	-0.258	0.104	4.932	130	WS00821_F01
6	25.914	114	1	-0.284	0.086	6.241	130	WS00821_F01
7	15.555	130	3	-0.206	0.049	4.949	130	WS00821_F01
10	70.246	190	2	0.227	0.044	5.115	130	WS00821_F01
11	37.143	209	3	0.192	0.038	4.029	130	WS00821_F01
13	0.000	243	4	0.405	0.144	4.768	130	WS00821_F01
3	47.928	49	1	0.256	0.063	6.656	131	WS00821_G10
4	75.958	77	3	-0.273	0.080	4.067	131	WS00821_G10
6	95.249	126	1	0.280	0.088	4.092	131	WS00821_G10
7	15.555	130	3	-0.210	0.042	3.866	131	WS00821_G10
11	45.989	216	1	-0.198	0.037	3.846	131	WS00821_G10
12	0.000	233	1	-0.211	0.045	4.736	131	WS00821_G10
14	0.000	246	1	0.205	0.037	3.927	131	WS00821_G10
2	93.504	32	4	-0.196	0.040	4.388	132	WS00821_H03
3	9.953	42	3	0.225	0.058	4.820	132	WS00821_H03
3	141.443	65	4	0.299	0.076	5.949	132	WS00821_H03
3	154.505	69	1	0.262	0.034	4.479	132	WS00821_H03
4	55.925	76	1	-0.256	0.053	6.737	132	WS00821_H03
4	75.958	77	3	-0.355	0.178	4.839	132	WS00821_H03
5	31.191	95	1	-0.168	0.030	3.886	132	WS00821_H03
9	0.000	166	1	0.259	0.034	4.561	132	WS00821_H03
11	27.405	204	1	0.335	0.047	6.271	132	WS00821_H03
11	29.069	205	1	0.246	0.031	4.137	132	WS00821_H03
11	40.960	210	4	0.250	0.039	4.018	132	WS00821_H03
11	56.921	219	1	0.333	0.067	5.048	132	WS00821_H03
12	22.441	227	1	-0.210	0.035	4.581	132	WS00821_H03
12	51.382	242	1	-0.319	0.068	5.094	132	WS00821_H03
1	2.788	2	4	-0.135	0.048	4.179	133	WS00821_I20
2	48.839	29	4	0.285	0.046	4.203	133	WS00821_I20
3	14.500	43	2	-0.326	0.098	9.035	133	WS00821_I20
3	15.147	44	2	0.312	0.149	10.057	133	WS00821_I20
3	20.512	46	2	0.385	0.113	12.139	133	WS00821_I20
3	95.157	59	2	-0.262	0.044	5.785	133	WS00821_I20
3	104.681	60	2	0.151	0.045	4.711	133	WS00821_I20
6	69.073	121	3	0.323	0.085	8.288	133	WS00821_I20
7	15.555	130	2	-0.398	0.089	9.578	133	WS00821_I20
8	50.001	154	2	-0.255	0.046	3.955	133	WS00821_I20
9	69.046	181	1	-0.334	0.067	3.979	133	WS00821_I20
11	0.000	199	2	0.315	0.045	5.033	133	WS00821_I20
11	43.491	214	4	-0.188	0.051	4.396	133	WS00821_I20
1	77.177	15	2	0.248	0.064	6.398	134	WS00821_J10
1	95.572	17	2	0.343	0.073	7.583	134	WS00821_J10
3	14.500	43	2	-0.191	0.055	5.384	134	WS00821_J10

3	95.157	59	2	-0.225	0.049	5.515	134	WS00821_J10
6	95.249	126	1	0.429	0.141	7.091	134	WS00821_J10
9	59.963	177	1	0.299	0.047	4.319	134	WS00821_J10
9	69.046	181	1	-0.378	0.092	4.735	134	WS00821_J10
11	27.405	204	1	0.404	0.082	7.417	134	WS00821_J10
11	29.069	205	1	0.338	0.081	7.373	134	WS00821_J10
11	45.989	216	1	-0.315	0.120	10.580	134	WS00821_J10
14	9.923	247	1	-0.234	0.044	3.866	134	WS00821_J10
1	82.301	16	3	0.230	0.057	4.656	135	WS00821_L03
1	95.572	17	3	-0.295	0.080	5.414	135	WS00821_L03
1	107.333	19	3	-0.299	0.100	8.148	135	WS00821_L03
1	107.333	19	4	-0.213	0.049	5.229	135	WS00821_L03
1	127.805	21	3	-0.250	0.073	5.951	135	WS00821_L03
3	112.868	61	2	-0.204	0.043	3.990	135	WS00821_L03
3	141.443	65	4	0.391	0.097	4.408	135	WS00821_L03
7	105.477	147	2	-0.279	0.066	4.056	135	WS00821_L03
8	39.090	152	4	-0.182	0.038	4.064	135	WS00821_L03
8	62.580	155	1	-0.311	0.098	4.472	135	WS00821_L03
2	95.880	34	1	0.251	0.048	5.337	136	WS00821_L12
2	127.147	40	3	-0.220	0.065	5.634	136	WS00821_L12
3	47.928	49	1	0.248	0.043	4.772	136	WS00821_L12
3	144.647	67	1	0.281	0.062	4.338	136	WS00821_L12
11	27.405	204	1	0.322	0.048	5.390	136	WS00821_L12
11	45.989	216	1	-0.226	0.062	6.814	136	WS00821_L12
13	1.815	244	4	0.258	0.051	4.343	136	WS00821_L12
14	0.000	246	1	0.275	0.050	5.650	136	WS00821_L12
2	127.147	40	3	-0.215	0.052	5.579	137	WS01016_F09
3	9.953	42	3	0.228	0.060	6.380	137	WS01016_F09
3	15.147	44	2	0.204	0.056	3.916	137	WS01016_F09
5	31.191	95	1	-0.257	0.048	4.631	137	WS01016_F09
6	95.249	126	1	0.299	0.059	4.530	137	WS01016_F09
7	75.883	144	2	-0.379	0.061	3.893	137	WS01016_F09
9	59.963	177	4	0.220	0.049	4.019	137	WS01016_F09
11	27.405	204	1	0.307	0.055	5.266	137	WS01016_F09
11	64.014	222	3	-0.205	0.047	4.957	137	WS01016_F09
14	22.402	248	3	0.206	0.039	4.107	137	WS01016_F09
2	127.147	40	3	-0.297	0.063	7.636	138	WS01016_K13
6	25.742	113	3	0.266	0.049	5.993	138	WS01016_K13
6	95.249	126	1	0.313	0.084	4.342	138	WS01016_K13
8	50.001	154	1	0.255	0.040	3.872	138	WS01016_K13
9	49.013	173	2	0.149	0.038	4.293	138	WS01016_K13
10	70.246	190	2	0.224	0.057	6.209	138	WS01016_K13
10	78.479	193	3	-0.173	0.033	3.959	138	WS01016_K13
10	86.151	195	3	0.231	0.035	4.303	138	WS01016_K13
10	93.347	196	2	0.432	0.062	4.316	138	WS01016_K13
11	9.237	202	4	-0.229	0.090	6.044	138	WS01016_K13
11	27.405	204	4	0.326	0.099	4.013	138	WS01016_K13
11	64.014	222	3	-0.224	0.041	4.916	138	WS01016_K13
3	148.745	68	1	0.372	0.072	5.886	139	WS0085_A22
3	154.505	69	1	0.358	0.052	4.090	139	WS0085_A22
4	43.146	74	3	-0.274	0.053	5.624	139	WS0085_A22
5	57.017	102	2	0.249	0.060	6.579	139	WS0085_A22
8	50.001	154	1	0.290	0.051	4.624	139	WS0085_A22
8	95.937	159	3	0.362	0.092	9.684	139	WS0085_A22

11	0.000	199	2	0.330	0.058	6.687	139	WS0085_A22
11	0.000	199	3	-0.339	0.053	5.872	139	WS0085_A22
1	62.128	11	1	-0.451	0.106	3.871	140	WS0023_D20
3	47.928	49	1	0.253	0.061	4.604	140	WS0023_D20
4	6.032	72	4	-0.198	0.039	4.115	140	WS0023_D20
4	142.917	85	3	0.243	0.051	4.154	140	WS0023_D20
7	15.555	130	2	0.251	0.046	5.181	140	WS0023_D20
10	70.246	190	4	-0.308	0.059	6.116	140	WS0023_D20
10	78.479	193	4	-0.266	0.076	7.143	140	WS0023_D20
10	95.795	197	4	0.197	0.041	4.367	140	WS0023_D20
3	148.745	68	1	0.217	0.043	4.073	141	WS0085_G24
7	15.555	130	2	0.261	0.043	4.015	141	WS0085_G24
8	50.001	154	1	0.228	0.051	5.598	141	WS0085_G24
10	78.479	193	3	-0.268	0.044	5.058	141	WS0085_G24
10	93.347	196	1	-0.410	0.066	4.072	141	WS0085_G24
11	0.000	199	3	-0.220	0.036	4.006	141	WS0085_G24
11	43.491	214	3	0.253	0.049	5.603	141	WS0085_G24
11	64.014	222	3	-0.326	0.067	7.690	141	WS0085_G24
1	62.128	11	1	-0.710	0.197	9.574	142	WS0023_L07
2	126.181	39	4	-0.555	0.167	8.541	142	WS0023_L07
3	47.928	49	1	0.263	0.055	4.795	142	WS0023_L07
4	82.462	78	1	-0.197	0.086	7.407	142	WS0023_L07
4	142.917	85	1	0.282	0.103	8.782	142	WS0023_L07
6	56.188	120	2	0.280	0.055	5.531	142	WS0023_L07
6	106.702	128	2	-0.511	0.121	6.655	142	WS0023_L07
8	0.000	148	4	-0.303	0.071	7.039	142	WS0023_L07
11	9.237	202	4	-0.195	0.050	4.932	142	WS0023_L07
11	64.014	222	4	-0.202	0.063	6.352	142	WS0023_L07
12	22.441	227	1	0.165	0.053	4.544	142	WS0023_L07
1	40.464	7	3	-0.224	0.054	5.623	143	WS0105_P09
1	43.578	8	3	-0.245	0.064	6.728	143	WS0105_P09
1	45.794	9	1	0.228	0.045	4.221	143	WS0105_P09
2	63.261	30	3	-0.222	0.042	4.401	143	WS0105_P09
3	137.891	64	1	-0.276	0.061	5.657	143	WS0105_P09
3	141.443	65	3	-0.321	0.080	4.977	143	WS0105_P09
4	103.719	81	4	0.198	0.056	4.771	143	WS0105_P09
4	142.917	85	1	0.228	0.050	4.750	143	WS0105_P09
11	27.405	204	1	-0.274	0.048	4.555	143	WS0105_P09
11	45.989	216	1	0.224	0.053	5.050	143	WS0105_P09
12	22.629	237	1	-0.209	0.045	4.258	143	WS0105_P09
12	32.473	238	1	-0.227	0.051	4.837	143	WS0105_P09
12	51.382	242	1	0.439	0.205	6.667	143	WS0105_P09
1	2.788	2	4	0.205	0.054	6.030	144	WS0106_E14
1	38.326	6	4	0.371	0.108	9.960	144	WS0106_E14
1	40.464	7	4	-0.426	0.131	11.596	144	WS0106_E14
3	9.953	42	3	-0.255	0.075	6.812	144	WS0106_E14
7	59.789	137	3	0.179	0.046	4.150	144	WS0106_E14
7	74.560	143	1	-0.307	0.066	5.087	144	WS0106_E14
11	27.405	204	1	-0.362	0.106	8.209	144	WS0106_E14
11	29.069	205	1	-0.283	0.095	7.291	144	WS0106_E14
11	45.989	216	1	0.218	0.070	5.365	144	WS0106_E14
12	6.242	234	4	-0.287	0.054	4.526	144	WS0106_E14
2	75.550	31	1	-0.275	0.074	5.569	145	WS00923_L21
9	2.011	167	4	-0.149	0.038	3.972	145	WS00923_L21

10	9.580	187	3	0.310	0.131	5.085	145	WS00923_L21
12	14.406	230	3	-0.279	0.063	5.131	145	WS00923_L21
1	62.128	11	2	0.305	0.118	5.951	146	WS00923_P03
1	82.301	16	3	-0.300	0.043	5.230	146	WS00923_P03
5	31.191	95	1	-0.253	0.042	4.115	146	WS00923_P03
5	57.017	102	2	-0.285	0.048	4.429	146	WS00923_P03
6	89.401	125	1	-0.301	0.071	6.739	146	WS00923_P03
6	95.249	126	1	0.480	0.127	8.010	146	WS00923_P03
9	28.608	170	2	-0.242	0.047	4.547	146	WS00923_P03
11	27.405	204	1	0.557	0.154	15.204	146	WS00923_P03
11	29.069	205	1	0.400	0.114	10.953	146	WS00923_P03
11	29.069	205	2	-0.208	0.048	4.475	146	WS00923_P03
11	43.491	214	4	-0.186	0.063	4.273	146	WS00923_P03
11	45.989	216	1	-0.211	0.079	7.485	146	WS00923_P03
2	95.880	34	1	0.206	0.033	4.476	147	WS00929_K18
3	47.928	49	1	0.310	0.068	9.281	147	WS00929_K18
3	137.891	64	1	0.280	0.042	5.711	147	WS00929_K18
3	144.647	67	1	0.312	0.074	7.221	147	WS00929_K18
4	142.917	85	1	-0.243	0.036	4.910	147	WS00929_K18
11	27.405	204	1	0.298	0.034	4.749	147	WS00929_K18
11	45.989	216	1	-0.214	0.044	5.993	147	WS00929_K18
12	51.382	242	1	-0.277	0.061	6.012	147	WS00929_K18
13	0.000	243	4	0.249	0.044	4.285	147	WS00929_K18
14	0.000	246	1	0.235	0.036	4.944	147	WS00929_K18
9	81.073	183	1	-0.288	0.060	5.921	148	WS00929_L02
10	78.479	193	4	-0.274	0.053	5.235	148	WS00929_L02
12	0.000	233	1	-0.231	0.047	4.616	148	WS00929_L02
12	51.382	242	1	-0.300	0.071	4.308	148	WS00929_L02
3	47.928	49	1	0.290	0.060	7.139	149	WS00929_M08
4	142.917	85	1	-0.205	0.036	4.235	149	WS00929_M08
5	31.191	95	1	-0.208	0.033	3.904	149	WS00929_M08
6	95.249	126	1	0.435	0.113	7.638	149	WS00929_M08
11	27.405	204	1	0.428	0.082	9.951	149	WS00929_M08
11	29.069	205	1	0.323	0.059	7.136	149	WS00929_M08
11	45.989	216	1	-0.276	0.079	9.378	149	WS00929_M08
3	9.953	42	3	0.153	0.052	4.560	150	WS00929_M20
7	70.278	141	3	0.199	0.053	4.646	150	WS00929_M20
11	27.405	204	1	0.327	0.056	5.612	150	WS00929_M20
11	29.069	205	1	0.244	0.047	4.726	150	WS00929_M20
11	43.491	214	4	-0.229	0.051	5.031	150	WS00929_M20
11	45.989	216	1	-0.252	0.085	8.378	150	WS00929_M20
11	60.464	221	1	0.264	0.039	3.895	150	WS00929_M20
1	123.316	20	4	0.263	0.063	4.972	151	WS00929_O01
4	116.928	82	4	0.245	0.051	5.671	151	WS00929_O01
4	142.917	85	1	0.267	0.090	6.762	151	WS00929_O01
5	42.979	97	2	-0.256	0.052	5.554	151	WS00929_O01
8	0.000	148	4	-0.441	0.140	15.647	151	WS00929_O01
10	93.347	196	1	-0.411	0.097	4.079	151	WS00929_O01
11	57.661	220	4	0.186	0.044	4.845	151	WS00929_O01
11	64.014	222	4	-0.240	0.058	6.192	151	WS00929_O01
12	14.406	230	4	0.196	0.047	5.730	151	WS00929_O01
14	43.392	250	2	-0.222	0.047	5.646	151	WS00929_O01
2	75.550	31	2	-0.258	0.047	4.267	152	WS00929_O08
4	75.958	77	3	-0.314	0.179	4.535	152	WS00929_O08

9	36.227	165	4	0.330	0.053	4.274	152	WS00929_O08
9	59.963	177	4	0.254	0.036	4.139	152	WS00929_O08
11	45.989	216	1	-0.253	0.058	5.733	152	WS00929_O08
13	0.000	243	4	0.346	0.079	4.206	152	WS00929_O08
1	2.788	2	4	-0.202	0.057	5.222	153	WS00929_P13
1	40.464	7	3	0.146	0.037	3.908	153	WS00929_P13
1	43.578	8	3	0.178	0.052	4.113	153	WS00929_P13
11	27.405	204	1	0.414	0.091	8.629	153	WS00929_P13
11	29.069	205	1	0.328	0.075	7.043	153	WS00929_P13
11	43.491	214	4	-0.174	0.054	4.434	153	WS00929_P13
11	45.989	216	1	-0.196	0.063	5.812	153	WS00929_P13
4	124.132	83	1	0.492	0.128	4.974	154	WS00930_A02
4	142.917	85	1	-0.252	0.040	4.611	154	WS00930_A02
1	95.572	17	3	0.207	0.038	4.823	155	WS0082_I06
1	107.333	19	3	0.229	0.045	5.789	155	WS0082_I06
3	47.928	49	1	0.292	0.083	6.214	155	WS0082_I06
8	50.001	154	1	0.290	0.075	6.652	155	WS0082_I06
9	0.000	166	2	0.209	0.052	6.063	155	WS0082_I06
9	69.046	181	2	0.286	0.056	4.522	155	WS0082_I06
10	78.479	193	3	-0.357	0.077	9.900	155	WS0082_I06
10	86.151	195	3	0.228	0.046	5.881	155	WS0082_I06
11	43.491	214	3	0.189	0.035	4.518	155	WS0082_I06
12	22.441	227	3	-0.258	0.039	4.398	155	WS0082_I06
12	6.242	234	2	0.227	0.036	3.887	155	WS0082_I06
1	107.333	19	3	0.189	0.036	3.948	156	WS0082_J01
2	120.617	38	3	0.217	0.041	4.439	156	WS0082_J01
3	144.593	66	4	-0.288	0.100	8.362	156	WS0082_J01
3	148.745	68	4	0.286	0.047	4.089	156	WS0082_J01
3	154.505	69	4	-0.333	0.086	6.782	156	WS0082_J01
5	52.820	99	3	-0.265	0.052	5.116	156	WS0082_J01
6	89.401	125	1	-0.289	0.053	3.982	156	WS0082_J01
7	74.560	143	2	0.246	0.051	5.516	156	WS0082_J01
10	78.479	193	4	-0.315	0.076	5.095	156	WS0082_J01
11	17.088	203	1	-0.292	0.105	7.716	156	WS0082_J01
11	29.069	205	1	0.243	0.052	3.944	156	WS0082_J01
12	22.441	227	3	-0.255	0.049	5.562	156	WS0082_J01
4	124.132	83	1	0.532	0.148	5.693	157	WS0082_L14
6	81.996	123	4	0.409	0.120	4.212	157	WS0082_L14
10	78.479	193	3	-0.283	0.044	4.340	157	WS0082_L14
11	45.989	216	1	-0.238	0.052	4.743	157	WS0082_L14
2	120.617	38	3	0.175	0.039	3.844	158	WS0082_M05
4	6.032	72	4	-0.224	0.079	7.335	158	WS0082_M05
5	31.191	95	1	-0.204	0.045	3.877	158	WS0082_M05
6	95.249	126	1	0.326	0.081	4.028	158	WS0082_M05
8	50.001	154	1	0.285	0.080	7.859	158	WS0082_M05
9	0.000	166	2	0.217	0.039	4.020	158	WS0082_M05
10	86.151	195	3	0.147	0.043	4.092	158	WS0082_M05
11	0.000	199	3	-0.310	0.046	4.209	158	WS0082_M05
11	29.069	205	1	0.313	0.058	5.187	158	WS0082_M05
11	45.989	216	1	-0.219	0.052	4.545	158	WS0082_M05
14	22.402	248	3	0.193	0.053	4.996	158	WS0082_M05
2	48.839	29	1	0.146	0.039	3.900	159	WS0082_O08
3	47.928	49	1	0.267	0.045	4.597	159	WS0082_O08
3	148.745	68	1	0.331	0.045	4.691	159	WS0082_O08

5	31.191	95	1	-0.277	0.051	5.219	159	WS0082_O08
6	95.249	126	1	0.363	0.053	3.987	159	WS0082_O08
9	59.963	177	4	-0.260	0.073	4.987	159	WS0082_O08
10	35.204	189	1	-0.337	0.052	5.352	159	WS0082_O08
10	78.479	193	4	-0.259	0.078	4.712	159	WS0082_O08
11	64.014	222	3	-0.160	0.042	4.642	159	WS0082_O08
1	107.333	19	3	0.248	0.053	5.658	160	WS0082_O18
2	48.839	29	4	0.247	0.057	5.116	160	WS0082_O18
2	126.181	39	4	-0.398	0.078	4.538	160	WS0082_O18
3	144.593	66	4	-0.179	0.048	4.003	160	WS0082_O18
3	154.505	69	4	-0.220	0.062	5.617	160	WS0082_O18
10	70.246	190	2	0.241	0.046	5.452	160	WS0082_O18
10	80.088	194	2	-0.245	0.030	4.450	160	WS0082_O18
11	27.405	204	1	0.444	0.131	9.278	160	WS0082_O18
11	29.069	205	1	0.350	0.105	7.307	160	WS0082_O18
11	29.069	205	2	-0.228	0.049	5.806	160	WS0082_O18
11	45.989	216	1	-0.171	0.066	4.565	160	WS0082_O18
12	22.441	227	1	0.293	0.062	4.409	160	WS0082_O18
12	22.441	227	3	-0.242	0.038	4.231	160	WS0082_O18
6	89.401	125	1	-0.224	0.040	4.094	161	WS0083_B09
6	95.249	126	1	0.314	0.056	4.673	161	WS0083_B09
7	74.560	143	1	-0.232	0.051	5.199	161	WS0083_B09
9	49.013	173	2	0.167	0.050	4.205	161	WS0083_B09
10	70.246	190	2	0.188	0.050	4.218	161	WS0083_B09
11	43.491	214	3	0.283	0.062	8.589	161	WS0083_B09
11	44.884	215	3	-0.212	0.043	5.864	161	WS0083_B09
11	64.014	222	3	-0.294	0.072	9.820	161	WS0083_B09
1	2.788	2	4	-0.143	0.034	4.277	162	WS0083_C14
1	29.800	5	1	0.185	0.060	4.922	162	WS0083_C14
1	107.333	19	3	0.227	0.039	4.256	162	WS0083_C14
6	69.073	121	3	-0.176	0.040	4.266	162	WS0083_C14
6	89.401	125	1	-0.346	0.090	7.498	162	WS0083_C14
6	95.249	126	1	0.372	0.080	4.323	162	WS0083_C14
9	28.608	170	2	-0.220	0.037	4.141	162	WS0083_C14
9	59.963	177	1	0.324	0.044	3.931	162	WS0083_C14
9	59.963	177	4	-0.235	0.049	4.292	162	WS0083_C14
10	80.088	194	2	-0.260	0.036	4.787	162	WS0083_C14
11	27.405	204	1	0.420	0.068	6.074	162	WS0083_C14
11	29.069	205	1	0.330	0.073	6.161	162	WS0083_C14
11	29.069	205	2	-0.200	0.043	4.695	162	WS0083_C14
11	40.960	210	4	-0.241	0.055	4.403	162	WS0083_C14
11	43.491	214	4	-0.231	0.085	6.038	162	WS0083_C14
11	44.884	215	4	0.269	0.074	6.844	162	WS0083_C14
11	45.989	216	1	-0.143	0.052	4.314	162	WS0083_C14
11	57.661	220	4	0.201	0.053	4.280	162	WS0083_C14
6	89.401	125	1	-0.337	0.070	6.069	163	WS00824_O10
9	0.000	163	1	-0.425	0.073	4.112	163	WS00824_O10
9	28.608	170	2	-0.337	0.093	10.286	163	WS00824_O10
9	28.608	170	3	0.441	0.088	9.471	163	WS00824_O10
9	49.013	173	2	0.143	0.041	3.984	163	WS00824_O10
9	59.963	177	4	-0.215	0.053	6.113	163	WS00824_O10
11	43.491	214	4	-0.157	0.048	4.454	163	WS00824_O10
11	44.884	215	4	0.172	0.039	4.096	163	WS00824_O10
11	45.989	216	1	-0.137	0.046	3.924	163	WS00824_O10

12	22.441	227	3	-0.289	0.059	5.607	163	WS00824_O10
2	126.181	39	4	-0.389	0.062	5.208	164	WS00824_O24
3	15.147	44	2	-0.262	0.050	4.240	164	WS00824_O24
3	47.928	49	1	0.311	0.121	6.941	164	WS00824_O24
4	75.958	77	3	-0.297	0.103	4.442	164	WS00824_O24
5	31.191	95	1	-0.263	0.087	5.024	164	WS00824_O24
6	95.249	126	1	0.328	0.103	4.504	164	WS00824_O24
9	59.963	177	4	-0.287	0.070	6.364	164	WS00824_O24
11	27.405	204	1	0.331	0.085	4.932	164	WS00824_O24
11	57.661	220	4	0.218	0.047	4.747	164	WS00824_O24
1	43.578	8	3	0.210	0.037	4.989	165	WS01028_A21
2	127.147	40	3	-0.205	0.033	4.425	165	WS01028_A21
3	47.928	49	1	0.261	0.045	4.764	165	WS01028_A21
6	89.401	125	1	-0.294	0.063	6.633	165	WS01028_A21
6	95.249	126	1	0.325	0.060	4.929	165	WS01028_A21
11	27.405	204	1	0.341	0.051	5.436	165	WS01028_A21
11	29.069	205	1	0.211	0.036	3.870	165	WS01028_A21
11	64.014	222	3	-0.229	0.045	6.066	165	WS01028_A21
1	95.572	17	2	-0.206	0.038	4.175	166	WS01028_B05
2	126.181	39	4	-0.385	0.064	5.118	166	WS01028_B05
2	127.147	40	4	0.238	0.039	4.411	166	WS01028_B05
3	15.147	44	2	-0.311	0.069	5.564	166	WS01028_B05
3	154.505	69	1	-0.253	0.053	4.138	166	WS01028_B05
4	75.958	77	3	-0.218	0.136	4.580	166	WS01028_B05
6	56.188	120	2	0.287	0.042	4.615	166	WS01028_B05
6	106.702	128	2	-0.439	0.130	4.786	166	WS01028_B05
10	78.479	193	4	-0.133	0.035	3.925	166	WS01028_B05
1	123.316	20	4	-0.232	0.048	4.619	167	WS01028_C18
3	15.147	44	2	-0.337	0.087	8.208	167	WS01028_C18
6	95.249	126	1	0.365	0.187	4.186	167	WS01028_C18
7	75.883	144	2	0.331	0.044	4.240	167	WS01028_C18
9	59.963	177	4	-0.294	0.058	7.302	167	WS01028_C18
10	80.088	194	2	-0.259	0.038	5.122	167	WS01028_C18
11	40.960	210	2	-0.478	0.074	7.204	167	WS01028_C18
1	50.955	10	1	-0.181	0.047	3.908	168	WS01028_D03
3	144.593	66	4	0.227	0.071	7.393	168	WS01028_D03
3	154.505	69	4	0.305	0.074	6.561	168	WS01028_D03
4	142.917	85	1	0.246	0.065	5.514	168	WS01028_D03
5	56.008	101	1	-0.284	0.059	4.954	168	WS01028_D03
6	81.996	123	3	-0.277	0.119	4.899	168	WS01028_D03
6	106.702	128	2	-0.446	0.097	4.942	168	WS01028_D03
7	62.787	139	4	-0.355	0.059	3.848	168	WS01028_D03
9	28.608	170	3	-0.249	0.049	3.878	168	WS01028_D03
12	22.441	227	1	0.231	0.055	4.506	168	WS01028_D03
3	14.500	43	2	-0.379	0.119	10.048	169	WS01028_E24
3	20.512	46	2	0.288	0.110	9.752	169	WS01028_E24
3	47.928	49	1	0.237	0.050	4.955	169	WS01028_E24
3	154.505	69	1	0.263	0.069	6.989	169	WS01028_E24
4	0.000	71	4	-0.208	0.046	4.368	169	WS01028_E24
4	55.925	76	1	-0.290	0.064	6.103	169	WS01028_E24
4	82.462	78	1	-0.224	0.057	5.592	169	WS01028_E24
6	69.073	121	3	0.279	0.052	4.861	169	WS01028_E24
11	27.405	204	1	0.262	0.054	5.394	169	WS01028_E24
3	47.928	49	1	0.211	0.043	4.095	170	WS01028_F13

11	45.989	216	1	-0.261	0.070	6.627	170	WS01028_F13
12	28.493	228	2	-0.407	0.150	7.242	170	WS01028_F13
14	0.000	246	1	0.264	0.060	5.784	170	WS01028_F13
1	77.177	15	2	0.204	0.049	5.030	171	WS0058_K16
1	95.572	17	2	0.286	0.051	5.299	171	WS0058_K16
3	15.147	44	2	0.235	0.056	4.018	171	WS0058_K16
4	6.032	72	4	-0.217	0.048	5.112	171	WS0058_K16
7	105.477	147	2	-0.251	0.061	4.486	171	WS0058_K16
9	59.963	177	1	0.265	0.052	4.278	171	WS0058_K16
9	69.046	181	1	-0.307	0.084	4.312	171	WS0058_K16
11	27.405	204	1	0.427	0.140	12.264	171	WS0058_K16
11	27.405	204	4	-0.296	0.058	6.179	171	WS0058_K16
11	29.069	205	1	0.240	0.056	4.707	171	WS0058_K16
11	45.989	216	1	-0.261	0.080	6.716	171	WS0058_K16
11	64.014	222	3	-0.331	0.113	9.098	171	WS0058_K16
3	9.953	42	3	0.194	0.046	4.340	172	WS01014_C07
3	15.147	44	2	0.281	0.096	6.032	172	WS01014_C07
3	20.512	46	2	0.231	0.049	3.965	172	WS01014_C07
6	95.249	126	1	0.388	0.088	6.759	172	WS01014_C07
11	0.000	199	2	0.260	0.057	4.663	172	WS01014_C07
11	0.000	199	3	-0.275	0.046	4.382	172	WS01014_C07
11	27.405	204	1	0.537	0.154	16.945	172	WS01014_C07
11	29.069	205	1	0.389	0.100	10.906	172	WS01014_C07
11	45.989	216	1	-0.321	0.110	11.791	172	WS01014_C07
11	60.464	221	1	0.253	0.047	5.114	172	WS01014_C07
1	62.128	11	2	0.303	0.073	4.853	173	WS00924_B22
2	63.261	30	3	0.237	0.030	3.916	173	WS00924_B22
3	14.500	43	2	-0.300	0.057	6.161	173	WS00924_B22
3	95.157	59	2	-0.220	0.033	4.189	173	WS00924_B22
6	56.188	120	2	0.219	0.034	4.632	173	WS00924_B22
6	95.249	126	1	0.370	0.079	4.684	173	WS00924_B22
11	27.405	204	1	0.426	0.112	8.604	173	WS00924_B22
11	29.069	205	1	0.321	0.086	6.357	173	WS00924_B22
11	45.989	216	1	-0.163	0.062	4.587	173	WS00924_B22
1	131.686	23	2	-0.497	0.091	5.811	174	WS00924_F08
5	56.008	101	2	-0.317	0.086	9.430	174	WS00924_F08
5	57.017	102	2	0.243	0.061	6.441	174	WS00924_F08
9	49.013	173	1	-0.224	0.067	5.128	174	WS00924_F08
10	93.347	196	2	0.510	0.131	5.929	174	WS00924_F08
10	104.214	198	2	-0.236	0.035	3.928	174	WS00924_F08
11	27.405	204	1	-0.218	0.051	3.947	174	WS00924_F08
12	38.543	232	4	-0.352	0.138	5.644	174	WS00924_F08
1	38.326	6	2	0.245	0.030	4.282	175	WS00924_H01
4	124.132	83	2	0.228	0.039	4.575	175	WS00924_H01
6	89.401	125	1	-0.268	0.098	4.421	175	WS00924_H01
7	15.555	130	2	0.222	0.034	4.757	175	WS00924_H01
9	0.000	166	2	0.256	0.058	8.680	175	WS00924_H01
10	78.479	193	3	-0.437	0.100	14.164	175	WS00924_H01
10	86.151	195	3	0.226	0.053	7.506	175	WS00924_H01
10	93.347	196	2	0.486	0.059	6.719	175	WS00924_H01
11	0.000	199	2	0.209	0.035	5.300	175	WS00924_H01
11	43.491	214	3	0.232	0.059	8.363	175	WS00924_H01
11	64.014	222	3	-0.164	0.028	4.056	175	WS00924_H01
12	6.242	234	2	0.280	0.049	6.738	175	WS00924_H01

13	1.815	244	2	0.231	0.040	5.678	175	WS00924_H01
2	120.617	38	3	0.171	0.045	4.951	176	WS00924_H11
2	127.147	40	3	-0.189	0.057	6.330	176	WS00924_H11
3	104.681	60	2	-0.291	0.060	6.783	176	WS00924_H11
3	112.868	61	2	0.285	0.065	7.327	176	WS00924_H11
3	114.747	62	2	0.199	0.053	5.963	176	WS00924_H11
4	75.958	77	3	-0.349	0.141	7.617	176	WS00924_H11
6	89.401	125	1	-0.291	0.073	5.636	176	WS00924_H11
6	95.249	126	1	0.431	0.108	6.254	176	WS00924_H11
8	50.001	154	1	0.265	0.047	4.178	176	WS00924_H11
9	0.000	166	2	0.185	0.030	4.452	176	WS00924_H11
9	28.608	170	2	-0.249	0.033	3.903	176	WS00924_H11
9	49.013	173	2	0.261	0.086	8.880	176	WS00924_H11
10	78.479	193	3	-0.299	0.051	5.622	176	WS00924_H11
10	86.151	195	3	0.150	0.039	4.306	176	WS00924_H11
11	27.405	204	1	0.370	0.055	4.527	176	WS00924_H11
11	29.069	205	1	0.338	0.069	5.280	176	WS00924_H11
11	29.069	205	2	-0.163	0.042	4.762	176	WS00924_H11
1	95.572	17	3	0.291	0.047	5.558	177	WS00924_I13
1	107.333	19	3	0.221	0.050	6.014	177	WS00924_I13
3	47.928	49	1	0.206	0.054	4.765	177	WS00924_I13
4	75.958	77	3	-0.330	0.109	6.239	177	WS00924_I13
5	31.191	95	1	-0.219	0.061	5.350	177	WS00924_I13
6	25.914	114	1	-0.264	0.079	6.958	177	WS00924_I13
7	15.555	130	2	0.250	0.039	3.916	177	WS00924_I13
7	75.883	144	4	-0.325	0.088	5.172	177	WS00924_I13
10	78.479	193	3	-0.273	0.035	4.229	177	WS00924_I13
13	3.386	245	1	0.286	0.074	6.582	177	WS00924_I13
1	133.992	24	2	0.233	0.037	4.425	178	WS00924_J03
1	141.021	25	2	-0.210	0.033	3.917	178	WS00924_J03
2	120.617	38	3	0.165	0.045	4.356	178	WS00924_J03
4	75.958	77	3	-0.307	0.086	4.644	178	WS00924_J03
6	25.914	114	1	-0.247	0.058	4.019	178	WS00924_J03
8	0.000	148	4	0.309	0.066	6.149	178	WS00924_J03
9	0.000	166	2	0.207	0.039	6.317	178	WS00924_J03
9	49.013	173	2	0.186	0.035	3.943	178	WS00924_J03
11	0.000	199	3	-0.427	0.074	6.600	178	WS00924_J03
1	62.128	11	1	-0.462	0.116	4.081	179	WS00924_K03
4	142.917	85	1	0.238	0.069	5.643	179	WS00924_K03
4	173.929	88	2	-0.208	0.052	5.518	179	WS00924_K03
5	0.000	89	1	-0.314	0.056	4.724	179	WS00924_K03
5	0.000	89	3	0.204	0.041	4.162	179	WS00924_K03
6	69.073	121	3	0.281	0.074	7.503	179	WS00924_K03
7	17.610	131	3	-0.243	0.075	4.126	179	WS00924_K03
7	59.789	137	3	-0.248	0.069	6.931	179	WS00924_K03
9	49.013	173	2	0.214	0.046	4.375	179	WS00924_K03
10	70.246	190	4	-0.272	0.084	7.572	179	WS00924_K03
12	6.242	234	4	0.375	0.061	5.150	179	WS00924_K03
14	43.392	250	1	-0.332	0.101	4.670	179	WS00924_K03
1	62.128	11	1	-0.484	0.097	4.759	180	WS00924_K15
1	131.686	23	1	0.661	0.159	7.754	180	WS00924_K15
9	0.000	166	1	0.198	0.069	5.458	180	WS00924_K15
11	64.014	222	3	-0.230	0.060	4.395	180	WS00924_K15
1	50.955	10	1	-0.190	0.038	3.868	181	WS00924_M10

1	131.686	23	1	0.508	0.130	4.408	181	WS00924_M10
3	47.928	49	1	0.268	0.056	5.671	181	WS00924_M10
6	95.249	126	1	0.376	0.095	6.396	181	WS00924_M10
8	50.001	154	1	0.257	0.049	5.208	181	WS00924_M10
9	58.045	176	1	0.275	0.080	5.331	181	WS00924_M10
9	69.046	181	1	-0.328	0.069	4.655	181	WS00924_M10
11	27.405	204	1	0.349	0.058	5.903	181	WS00924_M10
11	29.069	205	1	0.247	0.042	4.289	181	WS00924_M10
11	43.491	214	3	0.233	0.065	6.755	181	WS00924_M10
11	44.884	215	3	-0.215	0.048	5.056	181	WS00924_M10
11	64.014	222	3	-0.345	0.106	11.025	181	WS00924_M10
2	48.839	29	1	-0.304	0.054	4.562	182	WS00924_M23
4	140.962	84	3	0.269	0.062	5.565	182	WS00924_M23
5	52.820	99	1	0.191	0.046	3.987	182	WS00924_M23
5	52.820	99	3	-0.245	0.060	5.236	182	WS00924_M23
6	89.401	125	1	-0.227	0.051	4.291	182	WS00924_M23
6	106.702	128	2	-0.394	0.075	3.949	182	WS00924_M23
9	2.011	167	4	0.224	0.060	5.857	182	WS00924_M23
1	69.854	13	2	-0.213	0.042	4.462	183	WS00925_B24
1	71.592	14	2	-0.210	0.041	4.337	183	WS00925_B24
1	95.572	17	2	-0.262	0.043	4.550	183	WS00925_B24
1	123.316	20	4	-0.288	0.060	4.432	183	WS00925_B24
1	131.686	23	1	-0.521	0.149	4.617	183	WS00925_B24
3	9.953	42	4	0.275	0.069	6.706	183	WS00925_B24
3	47.928	49	1	-0.250	0.055	4.530	183	WS00925_B24
3	116.958	63	4	-0.212	0.038	4.021	183	WS00925_B24
6	25.914	114	1	0.255	0.057	4.675	183	WS00925_B24
6	81.996	123	4	0.323	0.127	3.884	183	WS00925_B24
8	95.937	159	3	-0.218	0.049	4.101	183	WS00925_B24
10	86.151	195	3	0.282	0.080	6.682	183	WS00925_B24
11	0.000	199	3	0.276	0.050	4.064	183	WS00925_B24
12	28.493	228	2	0.268	0.064	4.771	183	WS00925_B24
2	93.504	32	2	0.259	0.041	4.192	184	WS00925_F01
2	101.076	35	4	-0.201	0.054	5.314	184	WS00925_F01
6	87.891	124	2	0.257	0.079	8.019	184	WS00925_F01
6	89.401	125	2	0.298	0.062	6.228	184	WS00925_F01
7	15.555	130	3	-0.327	0.077	6.355	184	WS00925_F01
7	59.789	137	3	-0.315	0.084	7.913	184	WS00925_F01
7	70.278	141	3	0.262	0.059	5.584	184	WS00925_F01
11	0.000	199	2	-0.318	0.053	5.545	184	WS00925_F01
11	27.405	204	1	0.216	0.047	4.093	184	WS00925_F01
11	29.069	205	1	0.176	0.048	4.165	184	WS00925_F01
11	45.989	216	1	-0.284	0.073	6.287	184	WS00925_F01
11	64.014	222	3	-0.286	0.059	5.635	184	WS00925_F01
13	1.815	244	2	-0.379	0.082	8.419	184	WS00925_F01
3	47.928	49	1	0.223	0.045	4.629	185	WS0031_K05
3	116.958	63	4	-0.216	0.041	4.799	185	WS0031_K05
4	92.039	79	4	0.225	0.048	4.663	185	WS0031_K05
4	142.917	85	1	-0.301	0.057	5.835	185	WS0031_K05
6	25.914	114	1	-0.215	0.039	4.030	185	WS0031_K05
10	70.246	190	4	-0.282	0.043	4.984	185	WS0031_K05
10	78.479	193	4	-0.280	0.061	6.574	185	WS0031_K05
12	0.000	233	1	-0.237	0.054	5.519	185	WS0031_K05
14	0.000	246	1	0.265	0.057	5.849	185	WS0031_K05

4	55.925	76	1	0.244	0.048	4.417	186	WS01019_E18
4	124.132	83	1	-0.629	0.183	8.035	186	WS01019_E18
8	39.090	152	4	-0.250	0.058	5.690	186	WS01019_E18
9	0.000	163	1	-0.652	0.168	9.776	186	WS01019_E18
1	62.128	11	1	0.582	0.123	6.554	187	WS0032_J21
4	0.000	71	4	0.275	0.089	8.548	187	WS0032_J21
4	75.958	77	3	-0.420	0.127	7.606	187	WS0032_J21
9	3.127	168	1	-0.454	0.086	6.169	187	WS0032_J21
9	49.013	173	2	0.276	0.063	6.211	187	WS0032_J21
11	17.088	203	1	-0.232	0.068	5.804	187	WS0032_J21
11	27.405	204	1	0.299	0.062	5.272	187	WS0032_J21
11	29.069	205	1	0.359	0.107	9.233	187	WS0032_J21
11	45.989	216	1	-0.230	0.048	4.126	187	WS0032_J21
11	46.026	217	1	-0.253	0.053	4.564	187	WS0032_J21
12	0.000	233	1	-0.180	0.045	3.852	187	WS0032_J21
13	1.815	244	4	-0.184	0.052	4.074	187	WS0032_J21
14	44.898	251	3	0.311	0.062	5.776	187	WS0032_J21
2	95.880	34	1	0.265	0.043	4.732	188	WS01019_N08
2	127.147	40	3	-0.190	0.050	4.575	188	WS01019_N08
3	47.928	49	1	0.309	0.052	5.756	188	WS01019_N08
4	6.032	72	4	-0.223	0.053	5.052	188	WS01019_N08
4	92.039	79	4	0.266	0.042	4.043	188	WS01019_N08
9	28.608	170	2	-0.217	0.049	4.216	188	WS01019_N08
9	49.013	173	2	0.203	0.082	4.016	188	WS01019_N08
9	59.963	177	1	0.341	0.053	5.910	188	WS01019_N08
9	69.046	181	1	-0.398	0.100	4.956	188	WS01019_N08
9	69.046	181	2	0.344	0.103	4.771	188	WS01019_N08
10	86.151	195	3	0.190	0.047	4.347	188	WS01019_N08
11	27.405	204	1	0.365	0.054	6.054	188	WS01019_N08
11	45.989	216	1	-0.145	0.039	4.214	188	WS01019_N08
2	63.261	30	3	0.223	0.049	4.971	189	WS00925_N10
3	47.928	49	1	0.385	0.108	11.664	189	WS00925_N10
3	144.647	67	1	0.341	0.090	4.508	189	WS00925_N10
13	0.000	243	4	0.338	0.086	4.320	189	WS00925_N10
14	0.000	246	1	0.278	0.051	5.539	189	WS00925_N10
1	2.788	2	4	0.261	0.060	4.909	190	WS00925_N17
1	29.800	5	2	-0.387	0.116	10.116	190	WS00925_N17
1	38.326	6	2	0.309	0.073	7.951	190	WS00925_N17
1	40.464	7	4	-0.329	0.080	6.048	190	WS00925_N17
1	62.128	11	2	0.307	0.103	3.867	190	WS00925_N17
2	101.076	35	3	-0.212	0.043	3.861	190	WS00925_N17
2	127.147	40	3	0.213	0.061	5.568	190	WS00925_N17
3	104.681	60	2	0.251	0.055	6.327	190	WS00925_N17
3	112.868	61	2	-0.281	0.073	8.399	190	WS00925_N17
3	114.747	62	2	-0.284	0.080	9.250	190	WS00925_N17
4	116.928	82	2	-0.233	0.042	4.916	190	WS00925_N17
7	62.787	139	4	-0.218	0.029	4.185	190	WS00925_N17
10	21.864	188	1	-0.263	0.053	3.936	190	WS00925_N17
10	35.204	189	1	-0.228	0.074	5.338	190	WS00925_N17
14	0.000	246	1	-0.252	0.065	4.724	190	WS00925_N17
1	22.419	4	3	0.272	0.100	4.591	191	WS00925_O21
1	29.800	5	2	-0.313	0.054	5.118	191	WS00925_O21
1	38.326	6	2	0.218	0.038	4.429	191	WS00925_O21
1	40.464	7	4	-0.237	0.042	3.989	191	WS00925_O21

1	43.578	8	2	-0.243	0.042	4.986	191	WS00925_O21
3	95.157	59	2	-0.175	0.036	4.369	191	WS00925_O21
4	124.132	83	2	-0.301	0.086	4.356	191	WS00925_O21
6	106.702	128	2	-0.416	0.083	4.508	191	WS00925_O21
8	39.090	152	4	-0.304	0.095	8.422	191	WS00925_O21
8	87.463	158	2	-0.245	0.058	6.583	191	WS00925_O21
9	49.013	173	1	-0.249	0.064	5.105	191	WS00925_O21
10	9.580	187	2	-0.302	0.073	4.722	191	WS00925_O21
10	70.246	190	2	-0.234	0.042	4.900	191	WS00925_O21
11	40.960	210	4	0.271	0.052	3.888	191	WS00925_O21
11	45.989	216	1	0.242	0.089	6.215	191	WS00925_O21
14	43.392	250	2	-0.305	0.093	9.121	191	WS00925_O21
14	44.898	251	3	0.296	0.064	6.183	191	WS00925_O21
14	46.637	252	4	-0.262	0.055	4.846	191	WS00925_O21
1	69.854	13	2	-0.252	0.043	4.702	192	WS00925_P05
1	71.592	14	2	-0.272	0.055	5.960	192	WS00925_P05
1	77.177	15	2	-0.255	0.045	4.968	192	WS00925_P05
1	95.572	17	2	-0.293	0.052	5.771	192	WS00925_P05
1	107.333	19	4	-0.328	0.107	9.100	192	WS00925_P05
1	123.316	20	4	-0.317	0.069	3.915	192	WS00925_P05
3	80.211	55	4	-0.255	0.064	5.572	192	WS00925_P05
5	7.672	92	1	-0.266	0.045	4.685	192	WS00925_P05
5	7.672	92	4	0.332	0.069	6.259	192	WS00925_P05
7	75.883	144	2	0.575	0.145	9.222	192	WS00925_P05
7	75.883	144	4	-0.382	0.066	5.961	192	WS00925_P05
8	11.974	150	1	-0.141	0.051	4.033	192	WS00925_P05
9	58.045	176	1	-0.232	0.084	4.007	192	WS00925_P05
9	59.963	177	1	-0.312	0.072	5.885	192	WS00925_P05
9	69.046	181	1	0.430	0.144	6.930	192	WS00925_P05
9	69.046	181	2	0.213	0.078	4.126	192	WS00925_P05
10	80.088	194	2	-0.216	0.053	5.654	192	WS00925_P05
12	51.382	242	1	-0.434	0.145	4.724	192	WS00925_P05
13	3.386	245	1	0.295	0.079	6.351	192	WS00925_P05
2	120.617	38	3	0.222	0.039	3.953	193	WS00926_A12
3	47.928	49	1	0.247	0.061	6.040	193	WS00926_A12
4	0.000	71	4	-0.239	0.064	5.483	193	WS00926_A12
4	75.958	77	3	-0.327	0.092	4.322	193	WS00926_A12
4	124.132	83	1	0.453	0.093	4.095	193	WS00926_A12
6	45.897	117	1	0.259	0.063	6.100	193	WS00926_A12
6	49.100	118	4	0.442	0.125	5.291	193	WS00926_A12
7	67.154	140	4	0.448	0.126	5.602	193	WS00926_A12
7	73.169	142	1	-0.512	0.099	4.358	193	WS00926_A12
7	75.883	144	4	0.340	0.111	8.977	193	WS00926_A12
8	50.001	154	1	0.325	0.096	11.090	193	WS00926_A12
10	77.676	192	1	-0.194	0.047	4.678	193	WS00926_A12
10	104.214	198	4	-0.254	0.046	4.321	193	WS00926_A12
1	107.333	19	3	0.221	0.052	6.071	194	WS00926_A20
4	75.958	77	3	-0.265	0.071	4.233	194	WS00926_A20
6	25.914	114	1	-0.202	0.059	4.088	194	WS00926_A20
7	15.555	130	3	-0.317	0.070	6.986	194	WS00926_A20
8	50.001	154	1	0.192	0.056	3.910	194	WS00926_A20
9	59.963	177	4	-0.247	0.071	5.094	194	WS00926_A20
10	78.479	193	3	-0.384	0.078	9.006	194	WS00926_A20
11	35.604	207	3	-0.256	0.057	6.596	194	WS00926_A20

11	42.600	213	3	-0.258	0.053	6.133	194	WS00926_A20
1	29.800	5	1	0.259	0.090	6.625	195	WS00926_B22
1	40.464	7	4	0.277	0.050	4.301	195	WS00926_B22
1	107.333	19	3	0.262	0.055	5.894	195	WS00926_B22
6	56.188	120	2	0.236	0.042	4.346	195	WS00926_B22
9	59.963	177	4	-0.358	0.105	7.121	195	WS00926_B22
10	70.246	190	2	0.223	0.037	4.165	195	WS00926_B22
11	27.405	204	1	0.373	0.072	5.620	195	WS00926_B22
11	29.069	205	1	0.340	0.074	5.558	195	WS00926_B22
11	45.989	216	1	-0.152	0.054	4.023	195	WS00926_B22
1	107.333	19	3	0.252	0.064	5.430	196	WS00926_C04
3	15.147	44	2	-0.452	0.155	10.141	196	WS00926_C04
6	89.401	125	1	-0.302	0.071	5.103	196	WS00926_C04
9	59.963	177	4	-0.271	0.050	5.186	196	WS00926_C04
10	78.479	193	4	-0.216	0.054	4.959	196	WS00926_C04
11	44.884	215	4	0.221	0.039	3.842	196	WS00926_C04
11	57.661	220	4	0.264	0.067	6.919	196	WS00926_C04
1	29.800	5	1	0.252	0.062	5.300	197	WS0263_C04
1	107.333	19	3	0.234	0.048	4.618	197	WS0263_C04
2	120.617	38	3	0.202	0.040	3.864	197	WS0263_C04
3	83.563	56	3	-0.181	0.041	3.960	197	WS0263_C04
9	59.963	177	4	-0.338	0.084	7.615	197	WS0263_C04
1	107.333	19	3	0.251	0.045	4.335	198	WS0263_C20
2	93.504	32	4	0.448	0.091	7.308	198	WS0263_C20
6	89.401	125	1	-0.346	0.065	5.503	198	WS0263_C20
7	74.560	143	1	-0.173	0.054	4.473	198	WS0263_C20
7	75.883	144	2	0.541	0.131	8.086	198	WS0263_C20
9	28.608	170	3	0.331	0.074	5.933	198	WS0263_C20
10	35.204	189	1	-0.307	0.050	4.233	198	WS0263_C20
10	70.246	190	4	-0.248	0.040	4.327	198	WS0263_C20
10	78.479	193	4	-0.395	0.150	13.026	198	WS0263_C20
10	95.795	197	4	0.149	0.049	4.669	198	WS0263_C20
1	29.800	5	1	0.256	0.049	3.859	199	WS0263_E15
3	137.891	64	3	0.230	0.044	4.044	199	WS0263_E15
4	0.000	71	4	0.233	0.072	5.711	199	WS0263_E15
5	31.191	95	1	-0.189	0.049	3.844	199	WS0263_E15
10	78.479	193	4	-0.285	0.084	6.768	199	WS0263_E15
10	95.795	197	4	0.182	0.057	4.491	199	WS0263_E15
1	69.854	13	2	-0.198	0.043	4.720	200	WS0263_F01
1	71.592	14	2	-0.227	0.057	6.278	200	WS0263_F01
1	77.177	15	2	-0.257	0.073	7.974	200	WS0263_F01
3	9.953	42	4	0.297	0.078	7.333	200	WS0263_F01
4	0.000	71	4	0.204	0.042	4.446	200	WS0263_F01
4	75.958	77	3	-0.410	0.173	8.303	200	WS0263_F01
5	31.191	95	1	-0.209	0.053	4.149	200	WS0263_F01
5	42.979	97	1	0.313	0.058	4.517	200	WS0263_F01
5	52.820	99	1	0.258	0.054	4.381	200	WS0263_F01
7	15.555	130	3	-0.216	0.056	4.180	200	WS0263_F01
7	74.560	143	1	0.277	0.051	4.009	200	WS0263_F01
10	78.479	193	4	-0.236	0.051	4.303	200	WS0263_F01
11	56.921	219	1	-0.290	0.100	4.326	200	WS0263_F01
12	0.000	233	1	-0.228	0.063	4.930	200	WS0263_F01
14	0.000	246	1	0.283	0.082	6.386	200	WS0263_F01
1	133.992	24	2	0.177	0.050	4.066	201	WS0263_G08

3	83.563	56	3	-0.216	0.045	4.476	201	WS0263_G08
4	55.925	76	1	-0.313	0.091	9.295	201	WS0263_G08
4	75.958	77	3	-0.431	0.207	10.333	201	WS0263_G08
4	82.462	78	1	-0.269	0.044	4.720	201	WS0263_G08
4	124.132	83	1	0.416	0.071	4.054	201	WS0263_G08
5	31.191	95	1	-0.195	0.047	4.958	201	WS0263_G08
6	106.702	128	2	-0.414	0.085	4.312	201	WS0263_G08
9	17.951	169	1	0.223	0.041	4.362	201	WS0263_G08
9	69.046	181	2	0.289	0.090	4.392	201	WS0263_G08
14	44.898	251	3	0.218	0.043	4.481	201	WS0263_G08
1	29.800	5	1	0.181	0.040	4.004	202	WS0263_G23
4	75.958	77	3	-0.297	0.126	5.565	202	WS0263_G23
6	89.401	125	1	-0.281	0.054	5.513	202	WS0263_G23
6	95.249	126	1	0.478	0.110	8.616	202	WS0263_G23
11	0.000	199	3	-0.353	0.050	4.596	202	WS0263_G23
11	27.405	204	1	0.564	0.142	14.792	202	WS0263_G23
11	29.069	205	1	0.453	0.135	13.796	202	WS0263_G23
11	45.989	216	1	-0.235	0.083	8.393	202	WS0263_G23
1	82.301	16	3	-0.194	0.041	4.478	203	WS0263_I20
1	133.992	24	2	0.276	0.062	6.414	203	WS0263_I20
3	20.512	46	2	0.290	0.049	5.317	203	WS0263_I20
3	47.928	49	1	0.266	0.060	5.060	203	WS0263_I20
4	92.039	79	4	0.285	0.082	6.868	203	WS0263_I20
6	89.401	125	1	-0.224	0.053	4.579	203	WS0263_I20
6	95.249	126	1	0.384	0.123	6.770	203	WS0263_I20
8	50.001	154	1	0.287	0.068	6.117	203	WS0263_I20
11	35.604	207	3	-0.154	0.042	4.460	203	WS0263_I20
11	37.143	209	3	0.152	0.041	4.343	203	WS0263_I20
11	64.014	222	3	-0.277	0.073	7.914	203	WS0263_I20
14	22.402	248	3	0.267	0.061	6.518	203	WS0263_I20
1	95.572	17	3	0.280	0.047	4.715	204	WS0263_J03
1	107.333	19	3	0.238	0.051	5.843	204	WS0263_J03
5	31.191	95	1	-0.209	0.069	4.468	204	WS0263_J03
6	89.401	125	1	-0.295	0.078	5.052	204	WS0263_J03
7	15.555	130	2	0.211	0.033	4.035	204	WS0263_J03
11	27.405	204	1	0.261	0.060	3.899	204	WS0263_J03
11	29.069	205	1	0.261	0.083	5.341	204	WS0263_J03
11	45.989	216	1	-0.266	0.087	5.615	204	WS0263_J03
11	45.989	216	2	-0.214	0.044	5.468	204	WS0263_J03
12	0.000	226	3	0.199	0.034	3.899	204	WS0263_J03
1	22.419	4	3	0.322	0.066	4.025	205	WS0084_E12
2	48.839	29	4	0.220	0.073	5.590	205	WS0084_E12
2	127.147	40	3	-0.232	0.038	4.302	205	WS0084_E12
3	14.500	43	1	-0.259	0.062	5.292	205	WS0084_E12
3	17.158	45	1	0.271	0.051	4.490	205	WS0084_E12
5	42.979	97	1	0.212	0.046	3.999	205	WS0084_E12
6	89.401	125	1	-0.287	0.084	7.201	205	WS0084_E12
6	95.249	126	1	0.341	0.089	5.455	205	WS0084_E12
7	70.278	141	3	0.220	0.036	4.112	205	WS0084_E12
8	50.001	154	1	0.232	0.036	3.926	205	WS0084_E12
11	27.405	204	1	0.291	0.043	3.889	205	WS0084_E12
11	35.604	207	3	-0.222	0.035	4.055	205	WS0084_E12
11	64.014	222	3	-0.225	0.050	5.628	205	WS0084_E12
4	124.132	83	2	-0.330	0.134	5.891	206	WS0084_K23

11	0.000	199	2	-0.342	0.069	6.507	206 WS0084_K23
11	0.000	199	3	0.234	0.035	4.036	206 WS0084_K23
14	43.392	250	1	-0.244	0.060	3.930	206 WS0084_K23
2	95.880	34	1	0.212	0.033	3.984	207 WS01015_L15
2	102.814	36	1	0.189	0.035	3.883	207 WS01015_L15
3	47.928	49	1	0.333	0.080	9.611	207 WS01015_L15
3	141.443	65	4	0.274	0.047	4.683	207 WS01015_L15
3	144.647	67	1	0.245	0.043	3.927	207 WS01015_L15
6	25.914	114	1	-0.268	0.048	5.791	207 WS01015_L15
12	38.543	232	4	-0.251	0.047	4.333	207 WS01015_L15
1	95.572	17	2	-0.306	0.050	5.767	208 WS01015_P20
1	127.805	21	3	-0.217	0.063	5.087	208 WS01015_P20
2	48.839	29	4	-0.407	0.099	8.946	208 WS01015_P20
3	9.953	42	3	-0.215	0.049	3.954	208 WS01015_P20
9	36.227	165	4	0.265	0.039	4.802	208 WS01015_P20
9	81.073	183	1	-0.371	0.115	8.553	208 WS01015_P20
10	93.347	196	1	0.423	0.102	4.305	208 WS01015_P20
11	17.088	203	1	0.229	0.052	3.843	208 WS01015_P20
11	17.088	203	2	-0.249	0.052	5.712	208 WS01015_P20
12	22.629	237	1	-0.252	0.086	6.291	208 WS01015_P20
12	32.473	238	1	-0.207	0.058	4.216	208 WS01015_P20
1	107.333	19	4	-0.231	0.048	4.219	209 IS0011_G01
3	9.953	42	3	0.286	0.085	8.645	209 IS0011_G01
3	137.891	64	1	0.259	0.055	5.194	209 IS0011_G01
4	173.929	88	2	-0.199	0.049	4.612	209 IS0011_G01
6	49.100	118	4	-0.399	0.176	6.883	209 IS0011_G01
11	0.000	199	3	-0.278	0.053	5.150	209 IS0011_G01
11	27.405	204	1	0.325	0.056	5.361	209 IS0011_G01
11	29.069	205	1	0.237	0.045	4.251	209 IS0011_G01
11	45.989	216	1	-0.255	0.071	6.672	209 IS0011_G01
1	43.578	8	3	0.149	0.035	3.994	210 IS0012_A11
2	120.617	38	3	0.226	0.068	7.497	210 IS0012_A11
4	0.000	71	4	-0.220	0.065	5.062	210 IS0012_A11
4	124.132	83	1	0.550	0.187	6.097	210 IS0012_A11
10	78.479	193	3	-0.327	0.045	5.019	210 IS0012_A11
10	86.151	195	3	0.135	0.041	4.538	210 IS0012_A11
11	29.069	205	1	0.334	0.071	6.385	210 IS0012_A11
11	45.989	216	1	-0.180	0.053	4.449	210 IS0012_A11
14	44.898	251	3	0.259	0.044	4.599	210 IS0012_A11
4	6.032	72	4	-0.205	0.043	4.398	211 WS0043_N05
9	49.013	173	2	0.153	0.029	3.854	211 WS0043_N05
4	124.132	83	1	0.503	0.124	5.087	212 WS00927_J03
6	95.249	126	1	0.348	0.100	4.695	212 WS00927_J03
7	15.555	130	2	0.276	0.043	4.343	212 WS00927_J03
9	0.000	166	2	0.164	0.042	5.144	212 WS00927_J03
10	70.246	190	2	0.205	0.044	4.519	212 WS00927_J03
10	78.479	193	3	-0.336	0.071	7.158	212 WS00927_J03
10	86.151	195	3	0.196	0.048	4.837	212 WS00927_J03
11	27.405	204	1	0.314	0.049	4.389	212 WS00927_J03
11	29.069	205	1	0.345	0.090	7.977	212 WS00927_J03
11	45.989	216	1	-0.213	0.045	3.984	212 WS00927_J03
2	93.504	32	4	0.350	0.077	4.285	213 WS0044_G08
7	17.610	131	3	-0.313	0.096	6.160	213 WS0044_G08
10	70.246	190	2	0.283	0.054	4.809	213 WS0044_G08

1	82.301	16	3	-0.212	0.039	4.783	214	WS00927_M16
2	120.617	38	3	0.242	0.045	5.473	214	WS00927_M16
2	127.147	40	3	-0.170	0.045	5.465	214	WS00927_M16
3	17.158	45	1	0.224	0.046	3.917	214	WS00927_M16
3	47.928	49	1	0.238	0.055	4.596	214	WS00927_M16
6	89.401	125	1	-0.355	0.084	7.207	214	WS00927_M16
6	95.249	126	1	0.464	0.166	9.531	214	WS00927_M16
9	49.013	173	2	0.203	0.041	4.226	214	WS00927_M16
10	78.479	193	3	-0.448	0.077	9.530	214	WS00927_M16
11	27.405	204	1	0.297	0.053	4.533	214	WS00927_M16
11	29.069	205	1	0.298	0.060	5.046	214	WS00927_M16
11	35.604	207	3	-0.174	0.047	5.734	214	WS00927_M16
11	37.143	209	3	0.139	0.033	4.036	214	WS00927_M16
11	43.491	214	3	0.143	0.034	4.157	214	WS00927_M16
11	64.014	222	3	-0.202	0.046	5.595	214	WS00927_M16
1	43.578	8	2	-0.207	0.042	4.039	215	WS01024_G04
4	97.576	80	2	0.186	0.035	4.623	215	WS01024_G04
6	87.891	124	2	-0.212	0.056	5.355	215	WS01024_G04
6	95.249	126	1	0.438	0.138	8.598	215	WS01024_G04
8	50.001	154	1	0.232	0.043	4.182	215	WS01024_G04
11	27.405	204	1	0.306	0.048	4.496	215	WS01024_G04
11	29.069	205	1	0.278	0.052	4.772	215	WS01024_G04
11	64.014	222	3	-0.286	0.073	8.001	215	WS01024_G04
1	71.592	14	2	0.189	0.036	4.415	216	WS01024_G17
1	123.316	20	4	0.359	0.107	8.058	216	WS01024_G17
3	20.512	46	2	-0.216	0.047	5.789	216	WS01024_G17
3	47.928	49	1	0.235	0.067	4.405	216	WS01024_G17
4	82.462	78	1	-0.229	0.065	4.267	216	WS01024_G17
10	104.214	198	4	0.215	0.022	5.499	216	WS01024_G17
11	64.014	222	4	-0.257	0.039	3.925	216	WS01024_G17
12	28.493	228	2	-0.308	0.081	6.137	216	WS01024_G17
1	43.578	8	2	-0.230	0.046	4.810	217	WS01024_I11
1	123.316	20	4	0.353	0.108	6.193	217	WS01024_I11
2	126.181	39	2	0.200	0.041	4.185	217	WS01024_I11
4	6.032	72	4	0.265	0.070	5.772	217	WS01024_I11
6	56.188	120	1	0.243	0.045	3.911	217	WS01024_I11
7	74.560	143	1	0.344	0.106	9.161	217	WS01024_I11
3	14.500	43	1	-0.173	0.047	4.173	218	WS01024_I20
5	0.000	89	3	-0.182	0.038	4.549	218	WS01024_I20
6	49.100	118	4	-0.300	0.129	4.324	218	WS01024_I20
10	35.204	189	2	-0.193	0.037	3.868	218	WS01024_I20
11	27.405	204	1	0.299	0.042	3.893	218	WS01024_I20
11	29.069	205	1	0.266	0.055	4.970	218	WS01024_I20
1	127.805	21	3	0.286	0.076	8.376	219	WS01024_K21
3	14.500	43	2	-0.236	0.045	3.861	219	WS01024_K21
3	15.147	44	2	0.242	0.076	4.044	219	WS01024_K21
5	0.000	89	3	0.330	0.071	7.833	219	WS01024_K21
8	87.463	158	2	-0.282	0.071	6.954	219	WS01024_K21
9	81.073	183	3	-0.201	0.036	3.951	219	WS01024_K21
11	17.088	203	1	0.274	0.089	7.603	219	WS01024_K21
11	42.600	213	3	0.211	0.048	5.305	219	WS01024_K21
6	87.891	124	2	0.202	0.044	5.069	220	WS01024_L13
9	28.608	170	3	-0.278	0.048	5.511	220	WS01024_L13
10	86.151	195	3	0.303	0.053	6.138	220	WS01024_L13

12	0.000	226	3	-0.210	0.045	5.105	220	WS01024_L13
3	114.747	62	2	-0.154	0.044	4.063	221	WS01024_M15
4	43.146	74	3	-0.180	0.043	4.237	221	WS01024_M15
6	76.376	122	1	0.396	0.175	7.307	221	WS01024_M15
6	81.996	123	3	0.286	0.122	6.513	221	WS01024_M15
7	15.555	130	3	-0.277	0.086	7.766	221	WS01024_M15
11	17.088	203	1	0.153	0.042	3.936	221	WS01024_M15
11	46.026	217	1	0.262	0.039	3.872	221	WS01024_M15
13	1.815	244	4	0.222	0.064	5.796	221	WS01024_M15
2	63.261	30	3	0.208	0.031	4.141	222	WS01024_M24
2	95.880	34	3	0.227	0.041	5.130	222	WS01024_M24
3	47.928	49	1	0.218	0.071	4.641	222	WS01024_M24
3	154.505	69	1	0.267	0.069	4.645	222	WS01024_M24
4	55.925	76	1	-0.225	0.068	4.148	222	WS01024_M24
9	49.013	173	2	0.291	0.059	5.665	222	WS01024_M24
10	70.246	190	2	0.226	0.037	4.551	222	WS01024_M24
10	78.479	193	3	-0.257	0.035	4.709	222	WS01024_M24
10	86.151	195	3	0.204	0.039	5.118	222	WS01024_M24
11	29.069	205	1	0.265	0.074	4.915	222	WS01024_M24
12	6.242	234	2	0.261	0.041	5.133	222	WS01024_M24
1	131.686	23	1	0.521	0.104	4.618	223	WS01016_N01
3	9.953	42	3	0.298	0.105	10.549	223	WS01016_N01
3	47.928	49	1	0.327	0.076	7.923	223	WS01016_N01
3	137.891	64	3	0.299	0.087	7.720	223	WS01016_N01
3	141.443	65	3	0.471	0.151	9.018	223	WS01016_N01
6	95.249	126	1	0.423	0.115	6.911	223	WS01016_N01
10	21.864	188	1	-0.239	0.044	4.605	223	WS01016_N01
11	27.405	204	1	0.296	0.040	4.299	223	WS01016_N01
2	95.880	34	1	0.203	0.037	5.344	224	WS01016_N12
3	47.928	49	1	0.197	0.037	5.322	224	WS01016_N12
4	124.132	83	1	0.540	0.072	7.841	224	WS01016_N12
4	142.917	85	1	-0.337	0.069	10.003	224	WS01016_N12
5	57.017	102	4	0.224	0.039	5.121	224	WS01016_N12
12	0.000	233	1	-0.165	0.027	3.944	224	WS01016_N12
14	0.000	246	1	0.226	0.042	6.164	224	WS01016_N12
3	9.953	42	4	0.187	0.066	5.368	225	WS01016_P20
6	69.073	121	3	0.374	0.100	10.001	225	WS01016_P20
9	49.013	173	2	0.207	0.058	4.572	225	WS01016_P20
12	22.441	227	3	0.254	0.053	5.337	225	WS01016_P20
1	2.788	2	4	-0.279	0.050	5.498	226	WS01018_A07
1	22.419	4	3	0.402	0.083	4.420	226	WS01018_A07
4	142.917	85	1	-0.231	0.045	5.377	226	WS01018_A07
6	95.249	126	1	0.387	0.102	5.294	226	WS01018_A07
9	59.963	177	1	0.334	0.053	6.383	226	WS01018_A07
11	27.405	204	1	0.458	0.088	10.561	226	WS01018_A07
11	29.069	205	1	0.393	0.087	10.518	226	WS01018_A07
11	45.989	216	1	-0.320	0.104	12.335	226	WS01018_A07
11	60.464	221	1	0.196	0.035	4.143	226	WS01018_A07
12	38.543	232	4	-0.400	0.096	6.578	226	WS01018_A07
14	0.000	246	1	0.253	0.042	4.993	226	WS01018_A07
2	95.880	34	1	0.243	0.045	4.693	227	WS01018_B19
6	95.249	126	1	0.355	0.116	6.793	227	WS01018_B19
8	11.974	150	1	-0.255	0.049	5.068	227	WS01018_B19
11	27.405	204	1	0.428	0.111	11.639	227	WS01018_B19

11	29.069	205	1	0.272	0.060	6.193	227	WS01018_B19
11	45.989	216	1	-0.245	0.063	6.552	227	WS01018_B19
11	64.014	222	3	-0.233	0.059	4.938	227	WS01018_B19
14	46.637	252	4	-0.312	0.066	6.916	227	WS01018_B19
3	17.158	45	1	0.178	0.051	4.370	228	WS01018_C07
4	55.925	76	1	-0.242	0.081	6.601	228	WS01018_C07
4	82.462	78	1	-0.210	0.053	4.584	228	WS01018_C07
5	7.672	92	1	-0.232	0.041	3.924	228	WS01018_C07
5	16.219	93	1	-0.264	0.060	5.180	228	WS01018_C07
5	31.191	95	1	-0.218	0.076	6.482	228	WS01018_C07
6	25.742	113	3	-0.254	0.075	9.102	228	WS01018_C07
6	87.891	124	2	0.160	0.042	4.271	228	WS01018_C07
14	0.000	246	1	0.171	0.048	4.057	228	WS01018_C07
1	62.128	11	1	-0.716	0.194	10.228	229	WS01018_E04
1	107.333	19	4	-0.187	0.060	6.475	229	WS01018_E04
1	131.686	23	1	0.526	0.095	5.002	229	WS01018_E04
3	47.928	49	1	0.207	0.044	3.937	229	WS01018_E04
5	52.820	99	1	-0.279	0.046	4.267	229	WS01018_E04
6	89.401	125	2	0.254	0.048	4.276	229	WS01018_E04
7	62.787	139	4	0.194	0.030	4.415	229	WS01018_E04
7	75.883	144	2	-0.367	0.058	4.350	229	WS01018_E04
7	75.883	144	4	0.302	0.036	4.414	229	WS01018_E04
8	62.580	155	1	0.421	0.090	4.796	229	WS01018_E04
9	0.000	166	1	0.260	0.117	10.373	229	WS01018_E04
9	81.073	183	1	0.213	0.048	4.192	229	WS01018_E04
11	40.960	210	2	0.535	0.117	8.473	229	WS01018_E04
11	43.491	214	4	0.104	0.026	4.187	229	WS01018_E04
3	9.953	42	3	0.136	0.038	4.150	230	WS01018_E13
3	14.500	43	2	-0.338	0.073	5.762	230	WS01018_E13
3	15.147	44	2	0.320	0.143	7.837	230	WS01018_E13
3	20.512	46	2	0.339	0.131	12.495	230	WS01018_E13
3	95.157	59	2	-0.268	0.051	5.971	230	WS01018_E13
4	142.917	85	3	-0.170	0.040	3.971	230	WS01018_E13
6	95.249	126	1	0.450	0.134	9.095	230	WS01018_E13
7	15.555	130	2	-0.267	0.052	5.017	230	WS01018_E13
7	59.789	137	3	-0.241	0.063	6.932	230	WS01018_E13
9	81.073	183	1	0.205	0.047	4.355	230	WS01018_E13
11	27.405	204	1	0.369	0.080	7.549	230	WS01018_E13
11	27.405	204	4	-0.333	0.087	6.932	230	WS01018_E13
11	35.604	207	3	-0.186	0.036	3.962	230	WS01018_E13
11	37.143	209	3	0.227	0.053	5.890	230	WS01018_E13
1	40.464	7	4	0.248	0.061	5.693	231	WS0086_A02
3	14.500	43	1	0.271	0.070	5.331	231	WS0086_A02
3	47.928	49	1	0.244	0.053	4.076	231	WS0086_A02
3	148.745	68	4	0.309	0.079	4.732	231	WS0086_A02
9	59.963	177	4	-0.305	0.077	7.144	231	WS0086_A02
1	107.333	19	3	0.248	0.043	4.853	232	WS0086_G22
4	75.958	77	3	-0.319	0.094	4.825	232	WS0086_G22
6	89.401	125	1	-0.384	0.054	5.542	232	WS0086_G22
6	95.249	126	1	0.334	0.071	4.385	232	WS0086_G22
8	50.001	154	1	0.280	0.075	7.658	232	WS0086_G22
9	59.963	177	4	-0.210	0.054	4.590	232	WS0086_G22
11	0.000	199	3	-0.335	0.040	4.240	232	WS0086_G22
11	29.069	205	1	0.327	0.049	5.046	232	WS0086_G22

11	45.989	216	1	-0.299	0.084	8.259	232	WS0086_G22
11	60.464	221	1	0.198	0.044	4.301	232	WS0086_G22
11	64.014	222	3	-0.161	0.035	3.855	232	WS0086_G22
1	38.326	6	2	0.228	0.060	4.733	233	IS0014_M20
1	71.592	14	2	0.192	0.054	4.092	233	IS0014_M20
3	47.928	49	1	0.320	0.077	8.658	233	IS0014_M20
3	137.891	64	1	0.193	0.036	4.010	233	IS0014_M20
4	124.132	83	1	0.702	0.168	11.063	233	IS0014_M20
6	89.401	125	1	-0.366	0.063	7.131	233	IS0014_M20
6	95.249	126	1	0.391	0.109	5.162	233	IS0014_M20
8	11.974	150	1	-0.236	0.037	4.132	233	IS0014_M20
9	0.000	166	2	0.163	0.038	3.859	233	IS0014_M20
10	70.246	190	2	0.214	0.051	3.999	233	IS0014_M20
10	78.479	193	3	-0.284	0.047	4.388	233	IS0014_M20
11	29.069	205	1	0.287	0.039	4.524	233	IS0014_M20
12	14.406	230	4	-0.185	0.038	3.861	233	IS0014_M20
1	15.589	3	2	0.220	0.043	4.354	234	WS0041_E22
1	29.800	5	1	0.238	0.073	6.291	234	WS0041_E22
1	40.464	7	4	0.312	0.059	5.001	234	WS0041_E22
1	82.301	16	3	-0.264	0.050	5.014	234	WS0041_E22
3	47.928	49	1	0.319	0.079	6.892	234	WS0041_E22
6	69.073	121	4	-0.253	0.074	6.266	234	WS0041_E22
6	89.401	125	1	-0.404	0.110	9.463	234	WS0041_E22
6	95.249	126	1	0.368	0.089	4.385	234	WS0041_E22
9	28.608	170	3	0.328	0.060	4.053	234	WS0041_E22
9	59.963	177	4	-0.459	0.161	12.187	234	WS0041_E22
10	78.479	193	3	-0.269	0.045	4.480	234	WS0041_E22
10	78.479	193	4	-0.201	0.053	4.675	234	WS0041_E22
10	86.151	195	3	0.201	0.062	6.139	234	WS0041_E22
11	60.464	221	2	0.201	0.052	4.840	234	WS0041_E22
1	38.326	6	2	0.269	0.061	5.888	235	WS01018_G05
2	127.147	40	3	-0.208	0.050	5.284	235	WS01018_G05
3	95.157	59	2	0.202	0.049	4.118	235	WS01018_G05
4	55.925	76	1	-0.228	0.048	4.159	235	WS01018_G05
6	95.249	126	1	0.362	0.109	6.931	235	WS01018_G05
11	27.405	204	1	0.341	0.071	6.523	235	WS01018_G05
11	29.069	205	1	0.295	0.072	6.624	235	WS01018_G05
11	35.604	207	3	-0.177	0.036	3.873	235	WS01018_G05
11	45.989	216	1	-0.224	0.050	4.624	235	WS01018_G05
11	64.014	222	3	-0.287	0.063	6.747	235	WS01018_G05
3	47.928	49	1	0.245	0.065	8.538	236	WS01018_H06
3	69.071	52	1	0.221	0.049	4.271	236	WS01018_H06
4	43.146	74	3	0.186	0.036	4.358	236	WS01018_H06
4	82.462	78	1	-0.232	0.037	4.913	236	WS01018_H06
5	31.191	95	1	-0.229	0.059	7.838	236	WS01018_H06
9	3.127	168	1	-0.298	0.045	4.239	236	WS01018_H06
9	28.608	170	3	-0.279	0.055	6.777	236	WS01018_H06
11	35.604	207	3	-0.240	0.057	6.878	236	WS01018_H06
11	37.143	209	3	0.251	0.062	7.518	236	WS01018_H06
13	3.386	245	1	0.338	0.070	9.323	236	WS01018_H06
4	75.958	77	3	-0.393	0.121	6.518	237	WS01018_J12
6	49.100	118	4	-0.337	0.212	5.612	237	WS01018_J12
6	95.249	126	1	0.350	0.085	4.853	237	WS01018_J12
7	75.883	144	2	0.420	0.090	4.644	237	WS01018_J12

8	39.090	152	4	0.131	0.050	3.869	237	WS01018_J12
8	50.001	154	2	0.193	0.053	5.003	237	WS01018_J12
12	22.441	227	3	-0.193	0.041	4.291	237	WS01018_J12
13	1.815	244	4	-0.267	0.093	7.716	237	WS01018_J12
2	63.261	30	3	0.257	0.061	8.018	238	WS01018_J24
3	20.512	46	2	0.134	0.028	4.121	238	WS01018_J24
3	95.157	59	2	-0.237	0.043	5.914	238	WS01018_J24
4	116.928	82	2	0.369	0.076	11.131	238	WS01018_J24
4	124.132	83	2	0.388	0.101	9.827	238	WS01018_J24
9	69.046	181	2	0.367	0.076	7.832	238	WS01018_J24
10	78.479	193	3	-0.177	0.033	4.241	238	WS01018_J24
3	9.953	42	3	0.305	0.070	8.777	239	WS01018_L10
3	14.500	43	1	-0.198	0.035	4.334	239	WS01018_L10
3	17.158	45	1	0.175	0.036	4.494	239	WS01018_L10
6	89.401	125	1	-0.294	0.031	3.996	239	WS01018_L10
6	95.249	126	1	0.550	0.188	17.662	239	WS01018_L10
10	78.479	193	3	-0.328	0.033	4.346	239	WS01018_L10
1	62.128	11	1	-0.503	0.145	4.927	240	WS01018_L23
3	9.953	42	3	0.141	0.037	4.083	240	WS01018_L23
3	144.647	67	1	0.331	0.134	4.492	240	WS01018_L23
5	31.191	95	1	-0.232	0.057	4.076	240	WS01018_L23
5	57.017	102	2	-0.288	0.046	5.540	240	WS01018_L23
7	15.555	130	3	-0.220	0.045	4.762	240	WS01018_L23
11	40.960	210	2	0.491	0.144	6.405	240	WS01018_L23
3	20.512	46	2	0.258	0.066	6.147	241	WS01018_N24
6	89.401	125	1	-0.210	0.048	4.558	241	WS01018_N24
6	95.249	126	1	0.474	0.169	12.126	241	WS01018_N24
9	2.011	167	4	0.171	0.055	4.618	241	WS01018_N24
2	63.261	30	3	0.340	0.100	7.466	242	WS01018_O17
2	95.880	34	1	0.229	0.038	4.503	242	WS01018_O17
3	9.953	42	3	-0.287	0.073	5.389	242	WS01018_O17
3	47.928	49	1	0.308	0.060	7.153	242	WS01018_O17
3	137.891	64	1	0.332	0.060	7.191	242	WS01018_O17
3	144.647	67	1	0.335	0.088	6.998	242	WS01018_O17
4	0.000	71	4	-0.281	0.079	9.019	242	WS01018_O17
6	95.249	126	1	0.348	0.107	4.097	242	WS01018_O17
8	11.974	150	1	-0.329	0.060	7.225	242	WS01018_O17
10	70.246	190	4	-0.285	0.044	4.438	242	WS01018_O17
10	86.151	195	3	0.160	0.057	4.208	242	WS01018_O17
10	93.347	196	1	0.403	0.104	3.896	242	WS01018_O17
11	27.405	204	1	0.279	0.032	3.884	242	WS01018_O17
12	0.000	233	1	-0.260	0.043	5.119	242	WS01018_O17
13	0.000	243	4	0.315	0.075	5.936	242	WS01018_O17
14	0.000	246	1	0.256	0.039	4.659	242	WS01018_O17
5	31.191	95	1	-0.247	0.059	7.773	243	WS00936_D01
6	25.742	113	3	-0.239	0.055	5.488	243	WS00936_D01
6	25.914	114	1	-0.276	0.063	8.254	243	WS00936_D01
9	36.227	165	3	-0.250	0.043	4.321	243	WS00936_D01
11	60.464	221	1	0.276	0.043	5.614	243	WS00936_D01
1	2.788	2	4	-0.253	0.042	5.593	244	WS00936_F03
2	48.839	29	4	0.280	0.045	6.578	244	WS00936_F03
4	116.928	82	4	-0.413	0.074	7.420	244	WS00936_F03
6	95.249	126	1	0.412	0.166	6.319	244	WS00936_F03
8	11.974	150	1	-0.258	0.046	4.574	244	WS00936_F03

11	29.069	205	2	-0.199	0.058	4.622	244	WS00936_F03
11	41.984	212	2	-0.266	0.074	6.617	244	WS00936_F03
11	45.989	216	2	-0.288	0.080	5.504	244	WS00936_F03
12	38.543	232	4	-0.329	0.072	4.370	244	WS00936_F03
1	69.854	13	2	0.167	0.035	4.142	245	WS00936_I22
1	71.592	14	2	0.183	0.042	4.985	245	WS00936_I22
1	95.572	17	2	0.262	0.046	5.375	245	WS00936_I22
1	123.316	20	4	0.322	0.099	7.284	245	WS00936_I22
2	93.504	32	4	-0.290	0.043	4.752	245	WS00936_I22
4	103.719	81	4	-0.217	0.040	4.695	245	WS00936_I22
12	28.493	228	2	-0.382	0.108	8.018	245	WS00936_I22
13	0.000	243	4	0.329	0.101	4.154	245	WS00936_I22
3	83.563	56	3	0.174	0.062	5.470	246	WS00936_K15
3	144.593	66	4	0.191	0.065	6.016	246	WS00936_K15
3	154.505	69	4	0.110	0.039	4.209	246	WS00936_K15
4	43.146	74	3	0.290	0.062	5.532	246	WS00936_K15
4	103.719	81	4	-0.258	0.046	4.389	246	WS00936_K15
5	7.672	92	1	-0.326	0.056	4.494	246	WS00936_K15
6	45.897	117	1	0.309	0.054	4.582	246	WS00936_K15
7	62.787	139	2	0.249	0.054	5.317	246	WS00936_K15
8	50.001	154	2	-0.150	0.054	6.747	246	WS00936_K15
11	17.088	203	2	0.139	0.046	5.372	246	WS00936_K15
12	22.629	237	1	0.324	0.057	4.514	246	WS00936_K15
1	43.578	8	3	0.233	0.039	4.617	247	WS00936_P09
3	14.500	43	1	-0.314	0.043	4.608	247	WS00936_P09
4	55.925	76	1	-0.132	0.041	4.164	247	WS00936_P09
4	103.719	81	4	-0.262	0.046	4.564	247	WS00936_P09
4	124.132	83	1	0.666	0.220	9.141	247	WS00936_P09
4	142.917	85	1	-0.258	0.057	5.915	247	WS00936_P09
4	173.929	88	2	-0.554	0.286	24.324	247	WS00936_P09
7	59.789	137	3	-0.242	0.050	4.568	247	WS00936_P09
9	0.000	163	1	0.503	0.089	5.570	247	WS00936_P09
9	28.608	170	3	-0.226	0.050	3.877	247	WS00936_P09
12	22.441	227	1	-0.245	0.059	6.195	247	WS00936_P09
1	22.419	4	3	0.401	0.116	6.852	248	WS00937_A17
1	43.578	8	3	0.245	0.062	5.309	248	WS00937_A17
3	20.512	46	2	0.193	0.045	4.129	248	WS00937_A17
3	95.157	59	2	-0.173	0.035	4.402	248	WS00937_A17
4	55.925	76	1	-0.274	0.062	6.018	248	WS00937_A17
4	82.462	78	1	-0.239	0.043	4.084	248	WS00937_A17
4	173.929	88	2	-0.225	0.061	5.601	248	WS00937_A17
6	54.066	119	4	-0.281	0.069	6.640	248	WS00937_A17
11	27.405	204	1	0.363	0.076	7.159	248	WS00937_A17
11	27.405	204	4	-0.229	0.053	5.217	248	WS00937_A17
11	29.069	205	1	0.243	0.048	4.626	248	WS00937_A17
11	45.989	216	1	-0.198	0.042	4.019	248	WS00937_A17
12	28.493	228	2	-0.299	0.076	4.507	248	WS00937_A17
14	22.402	248	4	0.243	0.045	4.122	248	WS00937_A17
2	48.839	29	1	-0.319	0.051	5.645	249	WS00937_E06
2	102.814	36	1	0.273	0.041	4.465	249	WS00937_E06
3	9.953	42	4	0.247	0.068	6.415	249	WS00937_E06
5	0.000	89	1	-0.277	0.046	5.098	249	WS00937_E06
6	45.897	117	1	0.282	0.056	6.162	249	WS00937_E06
6	49.100	118	4	0.465	0.201	9.991	249	WS00937_E06

6	54.066	119	4	0.246	0.050	4.557	249	WS00937_E06
9	0.000	163	1	-0.492	0.095	5.404	249	WS00937_E06
9	0.000	163	2	-0.448	0.101	4.021	249	WS00937_E06
9	3.127	168	1	0.369	0.087	4.980	249	WS00937_E06
11	42.600	213	3	-0.233	0.046	4.452	249	WS00937_E06
12	51.382	242	1	-0.263	0.082	4.257	249	WS00937_E06
4	0.000	71	4	-0.300	0.076	4.754	250	WS00937_F04
6	45.897	117	1	0.239	0.055	4.819	250	WS00937_F04
6	81.996	123	3	0.299	0.088	5.199	250	WS00937_F04
10	9.580	187	3	-0.287	0.071	3.996	250	WS00937_F04
10	93.347	196	2	0.407	0.058	3.981	250	WS00937_F04
11	43.491	214	3	0.228	0.040	5.126	250	WS00937_F04
1	82.301	16	3	-0.247	0.069	7.028	251	WS00826_P04
1	95.572	17	3	0.286	0.050	5.208	251	WS00826_P04
1	107.333	19	3	0.262	0.066	6.708	251	WS00826_P04
2	93.504	32	2	-0.243	0.042	4.555	251	WS00826_P04
3	47.928	49	1	0.169	0.060	5.053	251	WS00826_P04
3	95.157	59	2	0.224	0.039	3.860	251	WS00826_P04
6	45.897	117	1	0.269	0.046	4.006	251	WS00826_P04
8	50.001	154	1	0.163	0.057	4.532	251	WS00826_P04
10	78.479	193	3	-0.425	0.078	8.076	251	WS00826_P04
11	64.014	222	3	-0.243	0.042	4.327	251	WS00826_P04
12	38.543	232	4	-0.411	0.108	4.351	251	WS00826_P04
2	120.617	38	3	0.252	0.064	6.735	252	WS00720_B24
2	127.147	40	3	-0.166	0.039	4.151	252	WS00720_B24
3	47.928	49	1	0.350	0.102	8.662	252	WS00720_B24
4	0.000	71	4	-0.233	0.076	6.244	252	WS00720_B24
6	89.401	125	1	-0.362	0.100	8.532	252	WS00720_B24
8	50.001	154	1	0.345	0.095	8.670	252	WS00720_B24
11	35.604	207	3	-0.163	0.039	4.114	252	WS00720_B24
13	1.815	244	2	0.228	0.044	4.436	252	WS00720_B24
14	43.392	250	2	-0.272	0.059	5.859	252	WS00720_B24
14	46.637	252	4	-0.266	0.064	4.917	252	WS00720_B24
1	29.800	5	1	0.214	0.043	4.010	253	WS00720_F05
1	38.326	6	2	0.236	0.047	4.452	253	WS00720_F05
2	120.617	38	3	0.200	0.040	4.637	253	WS00720_F05
2	127.147	40	3	-0.200	0.051	5.938	253	WS00720_F05
3	47.928	49	1	0.255	0.054	5.113	253	WS00720_F05
6	89.401	125	1	-0.360	0.073	6.913	253	WS00720_F05
6	95.249	126	1	0.405	0.100	6.615	253	WS00720_F05
8	50.001	154	1	0.283	0.065	6.792	253	WS00720_F05
10	78.479	193	3	-0.293	0.045	5.253	253	WS00720_F05
11	27.405	204	1	0.327	0.043	4.251	253	WS00720_F05
11	29.069	205	1	0.296	0.052	5.015	253	WS00720_F05
11	43.491	214	3	0.164	0.037	4.319	253	WS00720_F05
11	64.014	222	3	-0.257	0.068	7.952	253	WS00720_F05
1	69.854	13	2	0.205	0.042	4.525	254	WS00720_G20
1	71.592	14	2	0.226	0.053	5.658	254	WS00720_G20
2	48.839	29	4	0.267	0.061	4.336	254	WS00720_G20
3	80.211	55	4	0.221	0.044	3.873	254	WS00720_G20
5	16.219	93	2	0.284	0.072	7.734	254	WS00720_G20
8	50.001	154	1	0.244	0.047	4.269	254	WS00720_G20
10	78.479	193	3	-0.273	0.043	4.318	254	WS00720_G20
11	64.014	222	3	-0.227	0.042	4.156	254	WS00720_G20

1	38.326	6	4	-0.241	0.051	4.166	255	WS00720_K24
1	127.805	21	4	0.248	0.064	6.092	255	WS00720_K24
3	47.928	49	1	0.267	0.060	5.721	255	WS00720_K24
3	144.647	67	1	0.404	0.190	8.204	255	WS00720_K24
4	92.039	79	2	-0.206	0.056	5.283	255	WS00720_K24
4	97.576	80	2	0.197	0.037	3.901	255	WS00720_K24
9	0.000	166	1	0.215	0.052	4.914	255	WS00720_K24
9	59.963	177	4	-0.239	0.049	3.952	255	WS00720_K24
13	1.815	244	4	0.209	0.043	4.203	255	WS00720_K24
1	43.578	8	2	-0.300	0.069	6.620	256	WS00720_N04
1	43.578	8	3	0.208	0.044	4.819	256	WS00720_N04
1	82.301	16	3	-0.252	0.061	6.632	256	WS00720_N04
3	47.928	49	1	0.324	0.109	10.077	256	WS00720_N04
5	86.669	106	4	0.267	0.167	5.227	256	WS00720_N04
6	95.249	126	1	0.398	0.115	7.093	256	WS00720_N04
10	78.479	193	3	-0.297	0.038	4.181	256	WS00720_N04
10	86.151	195	3	0.197	0.055	5.968	256	WS00720_N04
3	95.157	59	2	0.254	0.073	5.809	257	WS00721_B01
6	95.249	126	1	0.334	0.088	4.973	257	WS00721_B01
8	87.463	158	2	-0.171	0.045	3.965	257	WS00721_B01
9	55.358	175	3	-0.459	0.106	5.876	257	WS00721_B01
3	47.928	49	1	0.231	0.047	4.401	258	WS00721_C10
6	95.249	126	1	0.435	0.137	7.918	258	WS00721_C10
10	78.479	193	3	-0.301	0.052	5.245	258	WS00721_C10
11	29.069	205	1	0.263	0.041	3.905	258	WS00721_C10
1	82.301	16	3	-0.179	0.049	4.398	259	WS0041_M16
3	83.563	56	3	-0.158	0.046	4.075	259	WS0041_M16
4	124.132	83	1	0.431	0.095	4.159	259	WS0041_M16
8	39.090	152	4	0.170	0.048	4.704	259	WS0041_M16
9	3.127	168	1	-0.446	0.078	5.058	259	WS0041_M16
10	78.479	193	4	-0.375	0.100	8.616	259	WS0041_M16
5	56.008	101	2	0.191	0.043	4.372	260	WS0042_E08
5	57.017	102	2	-0.282	0.054	5.775	260	WS0042_E08
7	75.883	144	2	0.453	0.113	5.272	260	WS0042_E08
8	95.937	159	3	-0.210	0.041	4.108	260	WS0042_E08
9	81.073	183	3	0.320	0.074	5.755	260	WS0042_E08
11	40.960	210	2	0.447	0.116	5.674	260	WS0042_E08
13	3.386	245	1	0.261	0.067	5.383	260	WS0042_E08
1	82.301	16	3	-0.251	0.038	4.180	261	WS00816_D13
3	9.953	42	3	0.192	0.046	5.043	261	WS00816_D13
3	14.500	43	1	-0.221	0.052	4.975	261	WS00816_D13
6	89.401	125	1	-0.344	0.053	5.155	261	WS00816_D13
6	95.249	126	1	0.359	0.090	6.434	261	WS00816_D13
11	17.088	203	2	-0.271	0.076	5.670	261	WS00816_D13
11	27.405	204	1	0.324	0.050	4.831	261	WS00816_D13
11	29.069	205	1	0.290	0.055	5.452	261	WS00816_D13
11	29.069	205	2	-0.228	0.054	4.951	261	WS00816_D13
11	45.989	216	1	-0.242	0.053	5.206	261	WS00816_D13
11	64.014	222	3	-0.247	0.055	6.035	261	WS00816_D13
1	50.955	10	1	0.210	0.067	5.779	262	WS00816_J13
3	144.647	67	1	0.490	0.258	10.869	262	WS00816_J13
3	148.745	68	1	-0.304	0.044	3.860	262	WS00816_J13
4	142.917	85	1	-0.263	0.045	3.859	262	WS00816_J13
5	52.820	99	1	-0.245	0.045	3.993	262	WS00816_J13

6	56.188	120	2	0.256	0.046	5.154	262	WS00816_J13
7	74.560	143	1	0.215	0.059	5.173	262	WS00816_J13
8	87.463	158	2	0.282	0.063	6.095	262	WS00816_J13
9	3.127	168	1	0.450	0.150	6.540	262	WS00816_J13
9	17.951	169	1	-0.268	0.067	5.965	262	WS00816_J13
9	17.951	169	2	0.361	0.046	6.270	262	WS00816_J13
9	81.073	183	3	-0.227	0.079	6.226	262	WS00816_J13
10	80.088	194	1	0.208	0.069	5.967	262	WS00816_J13
10	104.214	198	4	0.334	0.056	4.227	262	WS00816_J13
11	40.960	210	2	0.375	0.061	3.930	262	WS00816_J13
11	43.491	214	4	0.110	0.033	4.556	262	WS00816_J13
1	2.788	2	4	-0.259	0.049	4.886	263	WS0262_M05
1	45.794	9	1	-0.334	0.104	11.161	263	WS0262_M05
1	45.794	9	3	0.251	0.054	4.696	263	WS0262_M05
3	144.647	67	1	0.380	0.066	4.016	263	WS0262_M05
4	55.925	76	1	-0.215	0.037	4.136	263	WS0262_M05
4	124.132	83	1	0.599	0.143	8.497	263	WS0262_M05
5	16.219	93	2	0.257	0.075	6.629	263	WS0262_M05
6	87.891	124	2	0.225	0.063	5.291	263	WS0262_M05
6	95.249	126	1	0.355	0.101	4.526	263	WS0262_M05
11	0.000	199	3	-0.421	0.101	8.640	263	WS0262_M05
11	17.088	203	1	-0.283	0.069	7.160	263	WS0262_M05
11	27.405	204	1	0.425	0.080	8.468	263	WS0262_M05
11	29.069	205	1	0.399	0.095	10.044	263	WS0262_M05
11	45.989	216	1	-0.315	0.096	9.960	263	WS0262_M05
12	22.441	227	1	-0.264	0.062	6.454	263	WS0262_M05
13	3.386	245	1	-0.139	0.044	4.520	263	WS0262_M05
14	0.000	246	1	0.308	0.073	7.613	263	WS0262_M05
14	22.402	248	3	0.237	0.083	7.420	263	WS0262_M05
1	69.854	13	2	-0.255	0.036	4.891	264	WS0263_A08
1	71.592	14	2	-0.288	0.046	6.186	264	WS0263_A08
1	77.177	15	2	-0.330	0.068	9.023	264	WS0263_A08
1	95.572	17	2	-0.342	0.063	8.631	264	WS0263_A08
1	131.686	23	2	-0.472	0.083	6.764	264	WS0263_A08
1	133.992	24	2	-0.479	0.120	16.192	264	WS0263_A08
1	141.021	25	2	0.389	0.085	11.438	264	WS0263_A08
3	14.500	43	2	0.283	0.044	5.640	264	WS0263_A08
3	154.505	69	4	0.159	0.055	4.671	264	WS0263_A08
4	75.958	77	3	-0.351	0.074	4.581	264	WS0263_A08
8	11.974	150	1	-0.125	0.074	4.173	264	WS0263_A08
9	0.000	163	1	0.452	0.102	4.537	264	WS0263_A08
9	69.046	181	2	0.178	0.068	4.751	264	WS0263_A08
10	93.347	196	1	-0.487	0.129	5.715	264	WS0263_A08
12	0.000	226	3	0.306	0.049	5.437	264	WS0263_A08
14	44.898	251	3	0.275	0.043	4.181	264	WS0263_A08
1	133.992	24	2	0.199	0.041	3.868	265	WS0038_I06
4	116.928	82	4	-0.236	0.061	4.738	265	WS0038_I06
4	124.132	83	1	0.540	0.123	6.158	265	WS0038_I06
5	0.000	89	3	0.281	0.042	4.930	265	WS0038_I06
6	56.188	120	2	0.245	0.054	4.785	265	WS0038_I06
7	75.883	144	2	0.451	0.129	5.593	265	WS0038_I06
9	0.000	166	2	0.189	0.043	3.991	265	WS0038_I06
9	3.127	168	1	-0.490	0.132	8.346	265	WS0038_I06
9	59.963	177	4	-0.237	0.054	5.076	265	WS0038_I06

10	78.479	193	3	-0.362	0.077	7.581	265	WS0038_I06
10	95.795	197	4	0.221	0.070	6.211	265	WS0038_I06
11	46.026	217	1	-0.227	0.046	4.347	265	WS0038_I06
14	46.637	252	4	0.284	0.048	4.366	265	WS0038_I06
1	29.800	5	2	-0.360	0.072	4.793	266	WS0039_B20
1	38.326	6	2	0.331	0.092	9.226	266	WS0039_B20
1	50.955	10	1	-0.231	0.052	4.634	266	WS0039_B20
6	89.401	125	1	-0.322	0.070	6.177	266	WS0039_B20
6	95.249	126	1	0.296	0.076	5.004	266	WS0039_B20
6	106.702	128	3	0.221	0.040	4.749	266	WS0039_B20
8	11.974	150	1	-0.291	0.075	6.605	266	WS0039_B20
8	50.001	154	1	0.225	0.050	4.722	266	WS0039_B20
9	59.963	177	4	-0.300	0.099	5.958	266	WS0039_B20
10	78.479	193	3	-0.360	0.054	6.716	266	WS0039_B20
10	86.151	195	3	0.179	0.034	4.214	266	WS0039_B20
11	0.000	199	3	-0.280	0.034	4.057	266	WS0039_B20
14	22.402	248	3	0.255	0.045	5.558	266	WS0039_B20
2	127.147	40	3	-0.202	0.036	4.576	267	WS00812_D06
4	75.958	77	3	-0.276	0.084	5.602	267	WS00812_D06
8	50.001	154	1	0.244	0.054	5.173	267	WS00812_D06
10	78.479	193	3	-0.257	0.040	5.097	267	WS00812_D06
11	27.405	204	1	0.349	0.071	6.047	267	WS00812_D06
11	29.069	205	1	0.267	0.066	5.651	267	WS00812_D06
11	35.604	207	3	-0.203	0.038	4.844	267	WS00812_D06
11	42.600	213	3	-0.209	0.037	4.752	267	WS00812_D06
11	64.014	222	3	-0.213	0.034	4.369	267	WS00812_D06
1	107.333	19	3	0.274	0.056	6.485	268	WS00812_E09
2	126.181	39	2	0.228	0.042	4.403	268	WS00812_E09
4	75.958	77	3	-0.251	0.065	3.888	268	WS00812_E09
6	89.401	125	1	-0.323	0.066	5.601	268	WS00812_E09
10	80.088	194	2	-0.223	0.038	3.951	268	WS00812_E09
11	17.088	203	1	-0.268	0.061	5.096	268	WS00812_E09
11	17.088	203	2	-0.214	0.048	4.640	268	WS00812_E09
11	27.405	204	1	0.392	0.089	7.585	268	WS00812_E09
11	29.069	205	1	0.366	0.106	8.930	268	WS00812_E09
11	29.069	205	2	-0.219	0.056	5.893	268	WS00812_E09
11	45.989	216	1	-0.206	0.057	4.750	268	WS00812_E09
11	64.014	222	3	-0.180	0.037	4.176	268	WS00812_E09
1	82.301	16	3	-0.212	0.056	5.706	269	WS00812_G19
1	95.572	17	3	0.270	0.066	6.802	269	WS00812_G19
2	120.617	38	3	0.193	0.041	4.263	269	WS00812_G19
9	0.000	166	2	0.212	0.050	5.488	269	WS00812_G19
10	78.479	193	3	-0.369	0.061	6.294	269	WS00812_G19
10	86.151	195	3	0.172	0.046	4.690	269	WS00812_G19
11	17.088	203	1	-0.178	0.050	4.688	269	WS00812_G19
11	27.405	204	1	0.349	0.074	6.995	269	WS00812_G19
11	29.069	205	1	0.351	0.079	7.531	269	WS00812_G19
11	45.989	216	1	-0.277	0.057	5.417	269	WS00812_G19
3	47.928	49	1	0.231	0.061	6.004	270	WS00812_H19
4	6.032	72	4	-0.156	0.046	4.495	270	WS00812_H19
4	75.958	77	3	-0.312	0.095	3.975	270	WS00812_H19
6	89.401	125	1	-0.381	0.056	5.727	270	WS00812_H19
6	95.249	126	1	0.351	0.094	4.678	270	WS00812_H19
9	81.073	183	3	0.182	0.046	4.112	270	WS00812_H19

11	17.088	203	1	-0.280	0.084	8.302	270	WS00812_H19
11	29.069	205	1	0.378	0.081	8.271	270	WS00812_H19
11	45.989	216	1	-0.311	0.078	7.743	270	WS00812_H19
11	64.014	222	3	-0.200	0.056	5.081	270	WS00812_H19
12	28.493	228	2	-0.318	0.113	5.302	270	WS00812_H19
3	9.953	42	4	-0.264	0.104	7.540	271	WS00812_K01
3	69.071	52	1	0.370	0.101	6.567	271	WS00812_K01
3	137.891	64	1	0.416	0.091	10.168	271	WS00812_K01
4	6.032	72	4	-0.342	0.099	8.096	271	WS00812_K01
4	55.925	76	1	0.920	0.622	68.737	271	WS00812_K01
4	82.462	78	1	0.372	0.119	13.344	271	WS00812_K01
4	92.039	79	2	-0.191	0.068	5.124	271	WS00812_K01
6	81.996	123	4	-0.354	0.100	4.427	271	WS00812_K01
12	22.441	227	1	0.265	0.046	5.297	271	WS00812_K01
14	46.637	252	4	0.298	0.069	5.900	271	WS00812_K01
2	120.617	38	4	0.286	0.039	4.332	272	WS00812_K18
3	137.891	64	3	-0.263	0.076	3.893	272	WS00812_K18
5	54.072	100	4	-0.663	0.362	44.651	272	WS00812_K18
5	57.017	102	4	0.507	0.159	16.205	272	WS00812_K18
5	86.669	106	4	-0.863	0.555	22.866	272	WS00812_K18
9	3.127	168	1	-0.409	0.130	5.422	272	WS00812_K18
9	32.356	171	2	-0.170	0.039	3.943	272	WS00812_K18
9	81.073	183	3	0.257	0.089	5.598	272	WS00812_K18
12	51.382	242	1	-0.345	0.114	5.216	272	WS00812_K18
14	22.402	248	4	0.381	0.075	8.787	272	WS00812_K18
14	46.637	252	4	0.462	0.105	12.998	272	WS00812_K18
1	43.578	8	2	-0.239	0.071	5.915	273	WS00812_N16
1	82.301	16	3	-0.226	0.040	4.188	273	WS00812_N16
1	131.686	23	1	0.484	0.096	3.986	273	WS00812_N16
3	47.928	49	1	0.249	0.044	4.665	273	WS00812_N16
5	31.191	95	1	-0.300	0.066	6.886	273	WS00812_N16
6	95.249	126	1	0.429	0.141	8.917	273	WS00812_N16
8	50.001	154	1	0.262	0.050	5.222	273	WS00812_N16
9	36.227	165	3	-0.274	0.045	4.796	273	WS00812_N16
9	0.000	166	2	0.215	0.045	3.851	273	WS00812_N16
10	86.151	195	3	0.176	0.041	4.267	273	WS00812_N16
11	56.921	219	2	0.287	0.092	3.878	273	WS00812_N16
13	0.000	243	4	-0.448	0.177	7.341	273	WS00812_N16
13	1.815	244	2	0.236	0.063	5.495	273	WS00812_N16
1	15.589	3	2	0.247	0.043	5.222	274	WS00812_O20
1	62.128	11	2	0.332	0.170	11.130	274	WS00812_O20
2	102.814	36	2	-0.179	0.059	6.708	274	WS00812_O20
3	20.512	46	2	0.141	0.044	5.276	274	WS00812_O20
3	95.157	59	2	-0.245	0.036	4.976	274	WS00812_O20
5	31.191	95	1	-0.370	0.123	8.485	274	WS00812_O20
5	42.979	97	1	0.262	0.071	4.799	274	WS00812_O20
5	52.820	99	1	0.270	0.085	5.668	274	WS00812_O20
5	56.008	101	1	-0.205	0.069	4.773	274	WS00812_O20
11	27.405	204	1	0.334	0.071	4.860	274	WS00812_O20
11	29.069	205	1	0.245	0.078	5.463	274	WS00812_O20
1	38.326	6	2	0.210	0.055	3.901	275	WS0097_M15
1	82.301	16	3	-0.277	0.042	5.329	275	WS0097_M15
2	127.147	40	3	-0.182	0.037	4.669	275	WS0097_M15
4	142.917	85	1	-0.214	0.036	4.275	275	WS0097_M15

5	31.191	95	1	-0.261	0.061	7.208	275	WS0097_M15
6	89.401	125	1	-0.290	0.035	4.239	275	WS0097_M15
6	95.249	126	1	0.371	0.079	6.979	275	WS0097_M15
11	27.405	204	1	0.386	0.065	7.700	275	WS0097_M15
11	29.069	205	1	0.319	0.061	7.294	275	WS0097_M15
11	45.989	216	1	-0.260	0.059	6.943	275	WS0097_M15
11	64.014	222	3	-0.247	0.056	6.985	275	WS0097_M15
1	2.788	2	4	-0.214	0.050	4.651	276	WS0097_N01
1	131.686	23	1	0.714	0.231	8.660	276	WS0097_N01
6	25.914	114	1	-0.278	0.057	4.820	276	WS0097_N01
8	0.000	148	4	-0.238	0.053	4.032	276	WS0097_N01
1	22.419	4	4	-0.336	0.065	4.249	277	WS0097_P05
1	38.326	6	4	-0.300	0.047	4.089	277	WS0097_P05
1	69.854	13	2	-0.194	0.037	4.033	277	WS0097_P05
2	95.880	34	3	0.301	0.066	5.244	277	WS0097_P05
3	47.928	49	1	0.204	0.051	4.122	277	WS0097_P05
5	54.072	100	4	-0.231	0.052	4.827	277	WS0097_P05
6	76.376	122	1	0.368	0.077	5.030	277	WS0097_P05
6	89.401	125	1	-0.380	0.098	7.872	277	WS0097_P05
8	11.974	150	1	-0.211	0.048	3.861	277	WS0097_P05
8	62.580	155	1	0.304	0.090	5.941	277	WS0097_P05
9	3.127	168	1	-0.349	0.072	5.090	277	WS0097_P05
10	78.479	193	3	-0.207	0.044	4.194	277	WS0097_P05
10	86.151	195	3	0.217	0.047	4.446	277	WS0097_P05
1	43.578	8	2	0.175	0.046	4.186	278	WS0097_P18
9	0.000	166	2	0.143	0.052	5.595	278	WS0097_P18
10	78.479	193	4	-0.274	0.081	6.184	278	WS0097_P18
11	0.000	199	3	-0.215	0.046	5.462	278	WS0097_P18
1	123.316	20	4	0.281	0.062	4.124	279	WS0099_B04
2	48.839	29	1	0.315	0.106	8.918	279	WS0099_B04
3	17.158	45	1	0.296	0.069	5.952	279	WS0099_B04
3	47.928	49	1	0.345	0.099	8.475	279	WS0099_B04
6	95.249	126	1	0.397	0.139	7.185	279	WS0099_B04
9	58.045	176	1	0.242	0.080	4.117	279	WS0099_B04
12	28.493	228	2	-0.263	0.070	4.713	279	WS0099_B04
1	2.788	2	4	-0.343	0.134	13.156	280	WS0099_B20
1	22.419	4	4	-0.339	0.116	5.901	280	WS0099_B20
11	27.405	204	1	0.427	0.082	8.437	280	WS0099_B20
11	29.069	205	1	0.345	0.082	8.329	280	WS0099_B20
11	45.989	216	1	-0.170	0.050	4.976	280	WS0099_B20
1	2.788	2	4	-0.222	0.053	5.993	281	WS0099_E06
3	47.928	49	1	0.282	0.061	5.659	281	WS0099_E06
6	89.401	125	1	-0.245	0.059	5.418	281	WS0099_E06
8	50.001	154	2	-0.235	0.058	4.123	281	WS0099_E06
1	2.788	2	4	-0.189	0.056	5.278	282	WS0099_E17
6	81.996	123	3	-0.336	0.092	5.186	282	WS0099_E17
8	50.001	154	2	-0.293	0.077	6.677	282	WS0099_E17
8	62.580	155	1	-0.256	0.126	4.244	282	WS0099_E17
8	87.463	158	2	0.157	0.041	4.284	282	WS0099_E17
11	27.405	204	1	0.306	0.059	5.269	282	WS0099_E17
2	63.261	30	3	0.202	0.048	4.480	283	WS00932_P24
3	15.147	44	2	0.316	0.076	3.992	283	WS00932_P24
3	20.512	46	2	0.171	0.049	4.598	283	WS00932_P24
3	95.157	59	2	-0.217	0.064	6.789	283	WS00932_P24

3	104.681	60	2	0.283	0.082	7.726	283	WS00932_P24
3	112.868	61	2	-0.263	0.064	6.003	283	WS00932_P24
3	114.747	62	2	-0.243	0.088	8.218	283	WS00932_P24
4	55.925	76	1	-0.209	0.043	3.946	283	WS00932_P24
7	74.560	143	1	0.232	0.047	4.377	283	WS00932_P24
11	40.960	210	2	0.389	0.069	4.076	283	WS00932_P24
11	45.989	216	1	-0.230	0.050	4.671	283	WS00932_P24
14	9.923	247	1	-0.211	0.044	4.154	283	WS00932_P24
14	43.392	250	2	-0.226	0.061	5.643	283	WS00932_P24
14	46.637	252	4	-0.248	0.055	4.403	283	WS00932_P24
3	47.928	49	1	0.186	0.043	4.170	284	WS00933_B02
4	103.719	81	4	-0.247	0.067	5.845	284	WS00933_B02
9	36.227	165	3	0.229	0.054	5.522	284	WS00933_B02
12	38.543	232	4	0.264	0.104	4.824	284	WS00933_B02
14	44.898	251	1	0.211	0.047	4.486	284	WS00933_B02
1	38.326	6	2	0.220	0.038	4.060	285	WS00933_E18
6	56.188	120	2	0.201	0.039	4.452	285	WS00933_E18
8	50.001	154	1	0.240	0.050	4.319	285	WS00933_E18
10	9.580	187	3	-0.402	0.098	5.325	285	WS00933_E18
10	70.246	190	2	0.247	0.043	4.515	285	WS00933_E18
10	78.479	193	3	-0.358	0.067	7.166	285	WS00933_E18
11	29.069	205	1	0.306	0.060	5.047	285	WS00933_E18
11	43.491	214	3	0.156	0.041	4.343	285	WS00933_E18
11	64.014	222	3	-0.206	0.055	5.870	285	WS00933_E18
1	107.333	19	3	0.226	0.037	4.067	286	WS00933_F17
8	50.001	154	1	0.211	0.058	6.314	286	WS00933_F17
9	59.963	177	4	-0.206	0.053	3.853	286	WS00933_F17
10	9.580	187	3	-0.406	0.125	6.693	286	WS00933_F17
10	70.246	190	2	0.316	0.082	7.183	286	WS00933_F17
10	78.479	193	3	-0.393	0.067	7.262	286	WS00933_F17
12	22.629	237	1	0.242	0.037	3.883	286	WS00933_F17
12	51.382	242	1	-0.526	0.143	7.080	286	WS00933_F17
14	0.000	246	2	0.239	0.058	4.232	286	WS00933_F17
1	43.578	8	2	-0.238	0.056	6.586	287	WS00933_J24
2	48.839	29	4	-0.262	0.066	5.248	287	WS00933_J24
2	102.814	36	1	0.317	0.075	5.613	287	WS00933_J24
3	144.593	66	4	-0.206	0.054	5.032	287	WS00933_J24
3	148.745	68	4	0.232	0.048	4.379	287	WS00933_J24
3	154.505	69	4	-0.226	0.050	4.528	287	WS00933_J24
10	35.204	189	1	-0.301	0.098	7.533	287	WS00933_J24
10	104.214	198	4	-0.191	0.028	4.611	287	WS00933_J24
11	43.491	214	3	0.201	0.055	5.418	287	WS00933_J24
12	6.242	234	2	0.277	0.033	5.149	287	WS00933_J24
14	0.000	246	2	0.263	0.063	6.006	287	WS00933_J24
1	29.800	5	1	0.285	0.073	6.411	288	WS00933_K23
1	95.572	17	3	0.329	0.098	9.688	288	WS00933_K23
1	107.333	19	3	0.272	0.053	6.027	288	WS00933_K23
2	126.181	39	2	-0.283	0.039	3.998	288	WS00933_K23
4	116.928	82	4	-0.176	0.048	4.037	288	WS00933_K23
6	25.914	114	1	-0.302	0.093	8.053	288	WS00933_K23
6	45.897	117	1	0.255	0.075	6.455	288	WS00933_K23
8	50.001	154	1	0.283	0.081	6.931	288	WS00933_K23
9	69.046	181	2	0.180	0.075	4.298	288	WS00933_K23
9	81.073	183	3	0.146	0.036	4.154	288	WS00933_K23

11	35.604	207	3	-0.122	0.039	4.249	288	WS00933_K23
11	42.600	213	3	-0.164	0.056	6.163	288	WS00933_K23
12	22.441	227	3	-0.148	0.039	5.191	288	WS00933_K23
1	43.578	8	3	0.292	0.044	4.746	289	WS00933_O09
1	69.854	13	2	-0.344	0.130	14.315	289	WS00933_O09
1	71.592	14	2	-0.338	0.127	13.973	289	WS00933_O09
1	77.177	15	2	-0.318	0.111	12.278	289	WS00933_O09
1	82.301	16	3	-0.310	0.051	5.399	289	WS00933_O09
2	127.147	40	3	-0.237	0.039	4.181	289	WS00933_O09
3	95.157	59	2	0.202	0.044	5.135	289	WS00933_O09
4	92.039	79	2	-0.163	0.042	4.728	289	WS00933_O09
9	0.000	166	1	0.216	0.077	5.955	289	WS00933_O09
9	32.356	171	2	-0.183	0.038	4.160	289	WS00933_O09
10	78.479	193	3	-0.179	0.038	3.864	289	WS00933_O09
11	43.491	214	3	0.252	0.044	4.777	289	WS00933_O09
11	64.014	222	3	-0.335	0.083	8.551	289	WS00933_O09
14	22.402	248	4	-0.424	0.091	4.940	289	WS00933_O09
4	124.132	83	1	0.623	0.213	7.758	290	WS00933_P09
4	142.917	85	1	-0.284	0.080	7.522	290	WS00933_P09
7	15.555	130	2	0.217	0.041	3.879	290	WS00933_P09
10	9.580	187	3	-0.400	0.115	6.676	290	WS00933_P09
10	78.479	193	3	-0.385	0.060	7.057	290	WS00933_P09
10	86.151	195	3	0.156	0.034	3.908	290	WS00933_P09
12	22.441	227	1	-0.225	0.052	4.919	290	WS00933_P09
1	131.686	23	2	-0.463	0.079	5.207	291	WS00931_G09
3	9.953	42	4	-0.285	0.076	7.775	291	WS00931_G09
3	17.158	45	1	-0.268	0.068	4.939	291	WS00931_G09
6	89.401	125	2	-0.245	0.040	4.692	291	WS00931_G09
6	95.249	126	1	-0.318	0.114	4.119	291	WS00931_G09
7	17.610	131	3	-0.243	0.106	5.246	291	WS00931_G09
7	73.169	142	1	-0.505	0.111	4.082	291	WS00931_G09
8	39.090	152	4	0.203	0.043	4.379	291	WS00931_G09
10	80.088	194	2	-0.217	0.054	6.034	291	WS00931_G09
10	95.795	197	4	0.205	0.042	4.242	291	WS00931_G09
14	44.898	251	3	-0.185	0.052	4.462	291	WS00931_G09
1	62.128	11	2	0.365	0.105	4.568	292	WS00931_K08
1	127.805	21	4	-0.280	0.054	4.620	292	WS00931_K08
2	95.880	34	3	0.266	0.060	5.345	292	WS00931_K08
3	17.158	45	1	-0.254	0.060	4.139	292	WS00931_K08
9	49.013	173	2	-0.279	0.080	8.311	292	WS00931_K08
11	0.000	199	2	-0.265	0.034	4.123	292	WS00931_K08
11	27.405	204	4	-0.237	0.060	5.189	292	WS00931_K08
1	123.316	20	4	-0.304	0.071	5.433	293	WS01025_M20
1	127.805	21	4	0.251	0.048	5.304	293	WS01025_M20
4	82.462	78	2	-0.199	0.044	5.117	293	WS01025_M20
5	0.000	89	1	0.177	0.050	3.877	293	WS01025_M20
6	106.702	128	3	0.318	0.076	5.019	293	WS01025_M20
7	15.555	130	2	-0.252	0.043	4.737	293	WS01025_M20
9	69.046	181	1	0.325	0.092	3.974	293	WS01025_M20
10	35.204	189	2	0.291	0.052	4.334	293	WS01025_M20
11	45.989	216	1	0.213	0.053	4.079	293	WS01025_M20
13	0.000	243	4	-0.448	0.160	5.551	293	WS01025_M20
1	62.128	11	1	-0.477	0.111	4.318	294	WS01026_B10
1	95.572	17	3	0.242	0.043	4.079	294	WS01026_B10

3	14.500	43	1	-0.267	0.051	4.879	294	WS01026_B10
6	69.073	121	4	0.241	0.080	5.197	294	WS01026_B10
2	126.181	39	4	-0.361	0.067	4.311	295	WS0076_B09
7	15.555	130	2	0.282	0.059	5.406	295	WS0076_B09
11	17.088	203	1	0.209	0.050	4.834	295	WS0076_B09
2	120.617	38	3	0.346	0.092	10.212	296	WS0076_B17
2	127.147	40	3	-0.302	0.097	10.797	296	WS0076_B17
3	47.928	49	1	0.343	0.090	8.588	296	WS0076_B17
5	31.191	95	1	-0.309	0.075	7.142	296	WS0076_B17
6	89.401	125	1	-0.259	0.058	5.519	296	WS0076_B17
6	95.249	126	1	0.355	0.077	5.679	296	WS0076_B17
9	17.951	169	2	0.246	0.046	4.419	296	WS0076_B17
11	27.405	204	1	0.351	0.074	7.169	296	WS0076_B17
11	43.491	214	4	-0.208	0.059	4.314	296	WS0076_B17
11	57.661	220	4	0.246	0.071	5.445	296	WS0076_B17
1	50.955	10	1	-0.262	0.064	5.967	297	WS0076_D02
2	120.617	38	3	0.201	0.043	4.437	297	WS0076_D02
2	127.147	40	3	-0.181	0.058	5.973	297	WS0076_D02
3	47.928	49	1	0.283	0.076	7.000	297	WS0076_D02
3	137.891	64	3	0.236	0.056	5.899	297	WS0076_D02
5	52.820	99	1	0.261	0.042	4.018	297	WS0076_D02
5	84.347	105	1	-0.230	0.050	4.655	297	WS0076_D02
6	95.249	126	1	0.583	0.249	15.546	297	WS0076_D02
9	0.000	166	2	0.249	0.059	6.238	297	WS0076_D02
10	86.151	195	3	0.194	0.060	6.173	297	WS0076_D02
12	28.493	228	2	-0.278	0.101	5.559	297	WS0076_D02
14	22.402	248	3	0.178	0.040	4.068	297	WS0076_D02
14	43.392	250	2	-0.208	0.048	4.493	297	WS0076_D02
14	44.898	251	3	0.239	0.039	4.125	297	WS0076_D02
1	22.419	4	3	0.431	0.084	5.167	298	WS0076_D09
1	29.800	5	1	0.162	0.058	4.264	298	WS0076_D09
1	40.464	7	3	0.207	0.080	7.094	298	WS0076_D09
1	43.578	8	3	0.218	0.088	8.020	298	WS0076_D09
1	45.794	9	3	-0.264	0.060	5.147	298	WS0076_D09
3	137.891	64	3	0.247	0.050	4.174	298	WS0076_D09
3	154.505	69	4	-0.090	0.028	4.174	298	WS0076_D09
6	89.401	125	1	-0.344	0.129	9.696	298	WS0076_D09
6	89.401	125	2	-0.307	0.041	4.855	298	WS0076_D09
6	95.249	126	1	0.529	0.176	10.279	298	WS0076_D09
10	104.214	198	2	-0.186	0.032	3.946	298	WS0076_D09
11	0.000	199	2	0.279	0.047	5.722	298	WS0076_D09
11	27.405	204	1	0.442	0.096	7.469	298	WS0076_D09
11	29.069	205	1	0.259	0.051	3.901	298	WS0076_D09
11	43.491	214	4	-0.136	0.043	4.273	298	WS0076_D09
11	45.989	216	1	-0.135	0.059	4.336	298	WS0076_D09
12	14.406	230	3	-0.327	0.049	4.480	298	WS0076_D09
1	29.800	5	1	0.233	0.059	5.196	299	WS0076_E23
1	50.955	10	1	-0.188	0.061	5.321	299	WS0076_E23
1	95.572	17	2	0.251	0.042	4.348	299	WS0076_E23
2	95.880	34	1	0.296	0.045	3.946	299	WS0076_E23
2	102.814	36	1	0.247	0.065	6.342	299	WS0076_E23
2	126.181	39	4	0.496	0.098	5.679	299	WS0076_E23
3	14.500	43	2	-0.193	0.041	4.117	299	WS0076_E23
3	15.147	44	2	0.232	0.085	6.104	299	WS0076_E23

3	20.512	46	2	0.259	0.056	5.624	299	WS0076_E23
5	7.672	92	1	0.268	0.051	4.287	299	WS0076_E23
6	25.914	114	1	0.171	0.052	4.553	299	WS0076_E23
6	69.073	121	3	0.249	0.046	4.095	299	WS0076_E23
7	105.477	147	2	-0.227	0.080	5.443	299	WS0076_E23
9	49.013	173	1	0.289	0.049	4.795	299	WS0076_E23
9	58.045	176	1	0.256	0.088	4.916	299	WS0076_E23
9	69.046	181	1	-0.312	0.067	3.853	299	WS0076_E23
10	9.580	187	3	0.234	0.077	4.958	299	WS0076_E23
10	86.151	195	3	0.184	0.047	4.135	299	WS0076_E23
11	43.491	214	4	-0.220	0.054	5.465	299	WS0076_E23
13	1.815	244	2	-0.227	0.040	3.945	299	WS0076_E23
1	2.788	2	4	-0.238	0.050	4.367	300	WS0076_F12
3	17.158	45	1	0.250	0.064	6.720	300	WS0076_F12
3	95.157	59	2	-0.188	0.061	5.250	300	WS0076_F12
3	114.747	62	2	-0.213	0.047	3.951	300	WS0076_F12
9	36.227	165	3	-0.263	0.041	4.380	300	WS0076_F12
11	45.989	216	1	-0.227	0.038	3.988	300	WS0076_F12
12	22.441	227	1	-0.247	0.052	5.656	300	WS0076_F12
1	69.854	13	2	-0.154	0.032	3.878	301	WS0076_G17
1	71.592	14	2	-0.156	0.033	3.984	301	WS0076_G17
1	77.177	15	2	-0.174	0.040	4.821	301	WS0076_G17
1	127.805	21	3	-0.160	0.048	4.103	301	WS0076_G17
3	137.891	64	3	0.319	0.083	7.035	301	WS0076_G17
4	142.917	85	3	-0.271	0.067	5.275	301	WS0076_G17
4	173.929	88	2	-0.168	0.035	4.205	301	WS0076_G17
6	95.249	126	1	0.379	0.129	5.726	301	WS0076_G17
7	17.610	131	3	-0.360	0.149	6.062	301	WS0076_G17
9	49.013	173	1	0.263	0.071	4.905	301	WS0076_G17
11	17.088	203	2	-0.235	0.059	4.365	301	WS0076_G17
11	27.405	204	1	0.401	0.108	7.282	301	WS0076_G17
11	29.069	205	1	0.359	0.121	8.075	301	WS0076_G17
11	45.989	216	1	-0.350	0.199	13.272	301	WS0076_G17
12	14.406	230	3	-0.277	0.051	4.371	301	WS0076_G17
1	77.177	15	2	0.252	0.049	4.471	302	WS0076_H06
1	127.805	21	4	0.152	0.029	3.993	302	WS0076_H06
3	47.928	49	1	0.314	0.058	5.909	302	WS0076_H06
6	95.249	126	1	0.440	0.126	7.242	302	WS0076_H06
7	105.477	147	2	-0.373	0.169	7.246	302	WS0076_H06
2	101.076	35	4	-0.231	0.049	5.818	303	WS0073_O03
3	144.593	66	4	-0.229	0.044	4.830	303	WS0073_O03
3	154.505	69	4	-0.272	0.059	5.415	303	WS0073_O03
8	87.463	158	2	0.166	0.032	3.999	303	WS0073_O03
9	0.000	166	2	-0.252	0.039	4.674	303	WS0073_O03
9	59.963	177	4	-0.282	0.081	8.266	303	WS0073_O03
4	75.958	77	3	-0.301	0.074	4.429	304	WS0073_O22
5	3.484	90	2	0.397	0.123	7.062	304	WS0073_O22
8	50.001	154	1	0.270	0.064	5.160	304	WS0073_O22
10	78.479	193	3	-0.314	0.050	4.956	304	WS0073_O22
1	123.316	20	4	0.311	0.137	5.360	305	WS0074_B14
2	127.147	40	3	-0.217	0.041	4.711	305	WS0074_B14
3	15.147	44	2	-0.327	0.148	5.788	305	WS0074_B14
4	92.039	79	2	-0.185	0.039	4.382	305	WS0074_B14
6	56.188	120	2	0.308	0.060	6.221	305	WS0074_B14

9	81.073	183	3	-0.221	0.041	4.833	305	WS0074_B14
10	78.479	193	3	-0.285	0.054	6.239	305	WS0074_B14
11	27.405	204	1	0.442	0.135	10.430	305	WS0074_B14
11	29.069	205	1	0.242	0.070	5.366	305	WS0074_B14
11	35.604	207	3	-0.232	0.048	5.579	305	WS0074_B14
11	37.143	209	3	0.208	0.038	4.420	305	WS0074_B14
1	29.800	5	1	0.309	0.058	6.180	306	WS0074_C05
5	0.000	89	1	-0.197	0.040	4.228	306	WS0074_C05
5	16.219	93	1	-0.261	0.040	4.313	306	WS0074_C05
11	35.604	207	3	-0.210	0.039	4.884	306	WS0074_C05
11	46.026	217	1	0.233	0.036	3.909	306	WS0074_C05
12	14.406	230	3	-0.308	0.045	5.718	306	WS0074_C05
1	15.589	3	2	-0.293	0.088	8.773	307	WS0074_E10
1	22.419	4	4	-0.432	0.255	5.081	307	WS0074_E10
1	95.572	17	2	-0.396	0.102	10.387	307	WS0074_E10
2	126.181	39	4	-0.443	0.095	5.318	307	WS0074_E10
3	14.500	43	1	-0.360	0.088	7.803	307	WS0074_E10
3	17.158	45	1	0.332	0.124	11.024	307	WS0074_E10
4	82.462	78	1	-0.304	0.083	7.377	307	WS0074_E10
7	59.789	137	3	-0.326	0.098	10.711	307	WS0074_E10
7	70.278	141	3	0.225	0.048	5.215	307	WS0074_E10
8	11.974	150	1	-0.218	0.054	4.778	307	WS0074_E10
9	0.000	166	1	0.272	0.067	5.971	307	WS0074_E10
10	95.795	197	4	-0.197	0.052	4.072	307	WS0074_E10
11	35.604	207	3	-0.220	0.042	4.548	307	WS0074_E10
11	42.600	213	3	-0.219	0.038	4.186	307	WS0074_E10
11	64.014	222	3	-0.287	0.053	5.794	307	WS0074_E10
14	0.000	246	1	-0.258	0.052	4.617	307	WS0074_E10
3	47.928	49	1	0.280	0.063	5.368	308	WS0074_E20
6	89.401	125	1	-0.268	0.050	4.254	308	WS0074_E20
8	50.001	154	1	0.247	0.045	4.134	308	WS0074_E20
9	59.963	177	4	-0.223	0.053	4.326	308	WS0074_E20
10	78.479	193	3	-0.224	0.037	3.978	308	WS0074_E20
11	27.405	204	1	0.364	0.052	4.622	308	WS0074_E20
11	29.069	205	1	0.301	0.060	5.098	308	WS0074_E20
11	43.491	214	3	0.157	0.037	3.943	308	WS0074_E20
11	64.014	222	3	-0.211	0.064	6.890	308	WS0074_E20
3	47.928	49	1	0.243	0.044	4.341	309	WS0074_G17
7	15.555	130	3	-0.187	0.048	4.333	309	WS0074_G17
8	50.001	154	1	0.258	0.048	4.951	309	WS0074_G17
11	64.014	222	3	-0.215	0.059	5.313	309	WS0074_G17
13	3.386	245	1	0.256	0.047	4.677	309	WS0074_G17
2	120.617	38	3	0.168	0.062	5.383	310	WS0074_H04
3	154.505	69	4	-0.194	0.049	5.090	310	WS0074_H04
4	124.132	83	1	0.465	0.097	4.651	310	WS0074_H04
5	31.191	95	1	-0.226	0.042	3.978	310	WS0074_H04
6	95.249	126	1	0.553	0.220	10.198	310	WS0074_H04
9	3.127	168	1	-0.489	0.118	6.100	310	WS0074_H04
11	17.088	203	1	-0.284	0.064	6.021	310	WS0074_H04
11	29.069	205	1	0.416	0.103	9.883	310	WS0074_H04
11	45.989	216	1	-0.261	0.083	7.688	310	WS0074_H04
14	46.637	252	4	0.319	0.040	4.293	310	WS0074_H04
1	82.301	16	3	-0.221	0.036	3.885	311	WS00819_A06
5	31.191	95	1	-0.258	0.062	5.276	311	WS00819_A06

6	95.249	126	1	0.402	0.103	6.274	311	WS00819_A06
9	28.608	170	2	-0.302	0.061	6.332	311	WS00819_A06
11	27.405	204	1	0.445	0.108	9.288	311	WS00819_A06
11	29.069	205	1	0.423	0.131	11.228	311	WS00819_A06
11	45.989	216	1	-0.268	0.092	7.806	311	WS00819_A06
1	107.333	19	3	0.237	0.044	4.933	312	WS00912_G06
3	47.928	49	1	0.328	0.081	7.956	312	WS00912_G06
5	31.191	95	1	-0.274	0.056	5.493	312	WS00912_G06
6	89.401	125	1	-0.301	0.055	5.376	312	WS00912_G06
6	95.249	126	1	0.418	0.105	6.669	312	WS00912_G06
11	27.405	204	1	0.397	0.068	6.739	312	WS00912_G06
11	29.069	205	1	0.355	0.082	8.058	312	WS00912_G06
11	45.989	216	1	-0.175	0.045	4.350	312	WS00912_G06
1	43.578	8	2	0.213	0.050	5.261	313	WS00819_F18
3	15.147	44	2	-0.281	0.100	5.313	313	WS00819_F18
3	80.211	55	4	-0.299	0.088	9.699	313	WS00819_F18
3	144.647	67	1	0.429	0.133	7.111	313	WS00819_F18
5	57.017	102	4	0.293	0.047	4.790	313	WS00819_F18
5	86.669	106	4	-0.339	0.086	4.745	313	WS00819_F18
6	56.188	120	2	0.310	0.066	6.602	313	WS00819_F18
8	50.001	154	2	-0.220	0.057	5.775	313	WS00819_F18
8	87.463	158	2	0.353	0.116	11.659	313	WS00819_F18
9	36.227	165	3	0.227	0.062	4.782	313	WS00819_F18
9	17.951	169	1	-0.325	0.075	6.397	313	WS00819_F18
9	17.951	169	2	0.362	0.077	8.924	313	WS00819_F18
10	80.088	194	1	0.265	0.070	5.949	313	WS00819_F18
8	11.974	150	1	-0.159	0.059	4.028	314	WS00912_K21
8	87.463	158	2	-0.315	0.116	14.033	314	WS00912_K21
8	95.937	159	3	-0.338	0.079	9.142	314	WS00912_K21
14	44.898	251	3	0.244	0.043	4.883	314	WS00912_K21
1	131.686	23	1	0.483	0.130	3.967	315	WS00829_M18
2	127.147	40	3	-0.218	0.050	5.170	315	WS00829_M18
4	55.925	76	1	-0.239	0.052	4.808	315	WS00829_M18
6	95.249	126	1	0.315	0.089	6.403	315	WS00829_M18
8	11.974	150	1	-0.272	0.068	6.920	315	WS00829_M18
8	39.090	152	4	0.215	0.048	4.005	315	WS00829_M18
11	27.405	204	1	0.282	0.049	5.039	315	WS00829_M18
11	64.014	222	3	-0.257	0.045	4.743	315	WS00829_M18
1	29.800	5	1	0.166	0.053	4.498	316	WS00830_H06
1	69.854	13	2	0.318	0.079	8.132	316	WS00830_H06
1	71.592	14	2	0.299	0.068	7.061	316	WS00830_H06
1	77.177	15	2	0.253	0.041	4.267	316	WS00830_H06
1	95.572	17	2	0.295	0.030	3.943	316	WS00830_H06
2	120.617	38	4	0.226	0.038	4.443	316	WS00830_H06
4	55.925	76	1	0.217	0.098	8.581	316	WS00830_H06
4	82.462	78	1	0.137	0.054	4.801	316	WS00830_H06
6	89.401	125	2	-0.378	0.049	4.892	316	WS00830_H06
9	0.000	163	1	-0.443	0.075	4.333	316	WS00830_H06
11	17.088	203	2	-0.134	0.045	5.787	316	WS00830_H06
11	56.921	219	2	0.405	0.173	6.193	316	WS00830_H06
13	1.815	244	2	0.367	0.086	8.949	316	WS00830_H06
1	22.419	4	4	0.372	0.205	4.554	317	WS00931_O13
1	127.805	21	4	-0.221	0.065	4.979	317	WS00931_O13
3	95.157	59	2	-0.239	0.063	7.731	317	WS00931_O13

3	104.681	60	2	0.243	0.045	5.446	317	WS00931_O13
4	116.928	82	2	-0.258	0.043	5.295	317	WS00931_O13
7	15.555	130	3	0.219	0.057	6.167	317	WS00931_O13
7	17.610	131	3	0.362	0.082	5.649	317	WS00931_O13
9	55.358	175	1	0.308	0.095	6.463	317	WS00931_O13
9	61.113	178	1	-0.400	0.231	5.191	317	WS00931_O13
2	75.550	31	2	-0.300	0.046	4.927	318	WS01029_D10
5	52.820	99	3	0.257	0.047	3.929	318	WS01029_D10
6	87.891	124	2	0.164	0.036	4.070	318	WS01029_D10
9	3.127	168	1	-0.395	0.176	6.652	318	WS01029_D10
11	64.014	222	4	0.319	0.080	7.247	318	WS01029_D10
1	107.333	19	3	0.178	0.043	3.945	319	WS00913_L24
4	92.039	79	2	-0.229	0.042	4.795	319	WS00913_L24
8	50.001	154	2	-0.212	0.044	4.865	319	WS00913_L24
10	104.214	198	2	-0.183	0.025	4.077	319	WS00913_L24
2	126.181	39	2	0.303	0.049	5.188	320	WS00914_C08
3	144.647	67	1	0.381	0.164	5.335	320	WS00914_C08
3	148.745	68	1	-0.240	0.053	4.479	320	WS00914_C08
3	154.505	69	1	-0.235	0.051	4.361	320	WS00914_C08
4	6.032	72	4	0.195	0.056	4.914	320	WS00914_C08
4	82.462	78	2	-0.144	0.048	4.233	320	WS00914_C08
4	92.039	79	2	-0.254	0.074	7.653	320	WS00914_C08
4	97.576	80	2	0.250	0.067	7.696	320	WS00914_C08
4	140.962	84	3	0.235	0.043	4.309	320	WS00914_C08
5	86.669	106	4	-0.311	0.115	4.142	320	WS00914_C08
6	56.188	120	2	0.331	0.066	6.652	320	WS00914_C08
7	105.477	147	2	0.222	0.086	4.694	320	WS00914_C08
8	87.463	158	2	0.290	0.079	7.944	320	WS00914_C08
11	43.491	214	4	0.223	0.072	5.964	320	WS00914_C08
3	47.928	49	1	0.353	0.116	10.566	321	WS0052_I10
3	144.593	66	4	-0.217	0.054	4.917	321	WS0052_I10
9	59.963	177	4	-0.233	0.069	5.560	321	WS0052_I10
11	43.491	214	3	0.190	0.037	4.019	321	WS0052_I10
5	57.017	102	4	0.211	0.041	4.133	322	WS0052_J24
9	59.963	177	4	-0.294	0.064	6.507	322	WS0052_J24
11	27.405	204	4	0.304	0.059	5.721	322	WS0052_J24
11	29.069	205	1	0.294	0.078	5.263	322	WS0052_J24
11	45.989	216	1	-0.202	0.070	4.749	322	WS0052_J24
14	46.637	252	4	0.332	0.064	6.670	322	WS0052_J24
8	50.001	154	1	0.274	0.113	11.934	323	WS0052_P13
8	78.389	157	1	0.301	0.048	4.962	323	WS0052_P13
8	87.463	158	1	-0.286	0.050	5.254	323	WS0052_P13
9	28.608	170	2	0.230	0.046	4.517	323	WS0052_P13
10	80.088	194	2	-0.197	0.050	4.806	323	WS0052_P13
11	9.237	202	4	-0.446	0.295	23.070	323	WS0052_P13
11	17.088	203	1	0.368	0.083	8.529	323	WS0052_P13
11	27.405	204	1	-0.529	0.134	13.677	323	WS0052_P13
11	27.405	204	4	0.692	0.315	28.524	323	WS0052_P13
11	29.069	205	1	-0.425	0.116	11.933	323	WS0052_P13
11	35.604	207	3	-0.291	0.057	6.370	323	WS0052_P13
11	37.143	209	3	0.270	0.053	5.822	323	WS0052_P13
11	42.600	213	3	-0.289	0.057	6.313	323	WS0052_P13
11	45.989	216	1	0.208	0.079	7.952	323	WS0052_P13
11	46.026	217	1	0.147	0.045	4.564	323	WS0052_P13

14	22.402	248	3	0.347	0.042	4.892	323	WS0052_P13
1	40.464	7	4	0.271	0.077	3.989	324	WS0053_A13
1	62.128	11	2	0.248	0.061	4.689	324	WS0053_A13
1	107.333	19	3	0.162	0.031	4.237	324	WS0053_A13
3	141.443	65	3	-0.257	0.069	4.090	324	WS0053_A13
10	9.580	187	3	-0.269	0.070	4.629	324	WS0053_A13
10	78.479	193	3	-0.222	0.033	4.502	324	WS0053_A13
10	86.151	195	3	0.218	0.028	3.946	324	WS0053_A13
1	62.128	11	1	-0.527	0.093	5.421	325	WS0053_D12
2	120.617	38	3	0.248	0.060	4.420	325	WS0053_D12
2	127.147	40	3	-0.246	0.065	4.708	325	WS0053_D12
3	47.928	49	1	0.349	0.115	10.556	325	WS0053_D12
4	55.925	76	1	-0.239	0.045	4.456	325	WS0053_D12
4	75.958	77	3	-0.308	0.167	6.524	325	WS0053_D12
4	82.462	78	1	-0.229	0.053	4.879	325	WS0053_D12
5	31.191	95	1	-0.212	0.042	3.890	325	WS0053_D12
6	89.401	125	2	0.244	0.048	4.700	325	WS0053_D12
8	50.001	154	1	0.285	0.069	7.027	325	WS0053_D12
12	22.629	237	1	0.227	0.055	5.025	325	WS0053_D12
1	107.333	19	3	0.237	0.044	4.992	326	WS0053_E13
2	126.181	39	2	0.290	0.056	5.612	326	WS0053_E13
4	6.032	72	4	-0.197	0.058	4.266	326	WS0053_E13
4	43.146	74	3	-0.277	0.060	6.914	326	WS0053_E13
7	17.610	131	3	-0.327	0.078	4.365	326	WS0053_E13
11	56.921	219	2	0.279	0.099	4.881	326	WS0053_E13
3	15.147	44	2	0.270	0.118	4.600	327	WS0053_J14
3	20.512	46	2	0.249	0.060	5.469	327	WS0053_J14
3	104.681	60	2	0.204	0.045	4.101	327	WS0053_J14
4	116.928	82	2	-0.233	0.046	4.173	327	WS0053_J14
4	124.132	83	1	0.458	0.076	4.740	327	WS0053_J14
4	173.929	88	2	0.210	0.057	5.227	327	WS0053_J14
6	26.350	115	2	0.216	0.055	5.029	327	WS0053_J14
6	49.100	118	2	-0.353	0.138	7.571	327	WS0053_J14
6	95.249	126	1	0.459	0.179	6.236	327	WS0053_J14
9	3.127	168	1	-0.421	0.069	4.748	327	WS0053_J14
11	17.088	203	1	-0.242	0.055	5.329	327	WS0053_J14
11	29.069	205	1	0.393	0.082	8.196	327	WS0053_J14
11	45.989	216	1	-0.256	0.066	6.397	327	WS0053_J14
11	56.921	219	2	0.317	0.099	6.222	327	WS0053_J14
1	43.578	8	2	0.259	0.035	4.633	328	WS0053_K19
1	71.592	14	2	-0.171	0.032	4.202	328	WS0053_K19
1	77.177	15	2	-0.205	0.046	5.947	328	WS0053_K19
3	9.953	42	4	-0.222	0.045	5.027	328	WS0053_K19
3	15.147	44	2	-0.263	0.057	4.502	328	WS0053_K19
7	17.610	131	3	-0.339	0.104	5.391	328	WS0053_K19
9	59.963	177	4	-0.335	0.087	9.731	328	WS0053_K19
10	80.088	194	2	-0.294	0.048	5.994	328	WS0053_K19
11	27.405	204	4	0.297	0.050	5.727	328	WS0053_K19
11	56.921	219	2	0.270	0.077	3.926	328	WS0053_K19
3	47.928	49	1	0.292	0.095	9.890	329	WS01033_G12
4	75.958	77	3	-0.334	0.085	3.884	329	WS01033_G12
6	95.249	126	1	0.423	0.153	9.917	329	WS01033_G12
8	11.974	150	1	-0.254	0.065	6.827	329	WS01033_G12
8	87.463	158	2	-0.212	0.069	5.341	329	WS01033_G12

9	0.000	166	2	0.313	0.094	6.774	329	WS01033_G12
10	78.479	193	3	-0.395	0.049	5.502	329	WS01033_G12
10	86.151	195	3	0.163	0.040	4.376	329	WS01033_G12
3	104.681	60	2	0.219	0.037	4.175	330	WS01033_H09
3	114.747	62	2	-0.187	0.039	4.413	330	WS01033_H09
7	17.610	131	3	0.292	0.050	4.353	330	WS01033_H09
7	75.883	144	4	0.250	0.084	3.855	330	WS01033_H09
10	21.864	188	3	-0.197	0.029	4.102	330	WS01033_H09
10	86.151	195	3	0.253	0.039	5.598	330	WS01033_H09
12	14.406	230	3	0.251	0.028	3.947	330	WS01033_H09
14	0.000	246	2	0.245	0.052	5.975	330	WS01033_H09
1	40.464	7	3	0.438	0.122	15.411	331	WS01033_K02
1	43.578	8	3	0.471	0.146	16.282	331	WS01033_K02
1	45.794	9	3	-0.245	0.059	3.921	331	WS01033_K02
1	69.854	13	2	0.282	0.077	6.190	331	WS01033_K02
1	71.592	14	2	0.287	0.081	6.540	331	WS01033_K02
1	77.177	15	2	0.241	0.057	4.599	331	WS01033_K02
1	131.686	23	1	0.679	0.250	7.838	331	WS01033_K02
3	47.928	49	1	0.386	0.096	10.464	331	WS01033_K02
4	55.925	76	1	-0.265	0.037	4.097	331	WS01033_K02
5	0.000	89	1	-0.328	0.061	6.941	331	WS01033_K02
5	0.000	89	3	0.283	0.032	4.222	331	WS01033_K02
5	31.191	95	1	-0.305	0.055	6.041	331	WS01033_K02
6	95.249	126	1	0.392	0.097	7.394	331	WS01033_K02
9	0.000	166	1	0.262	0.054	5.855	331	WS01033_K02
11	17.088	203	2	-0.252	0.117	8.820	331	WS01033_K02
11	29.069	205	2	-0.255	0.069	4.421	331	WS01033_K02
11	56.921	219	2	0.274	0.132	3.968	331	WS01033_K02
14	44.898	251	1	0.339	0.052	5.785	331	WS01033_K02
1	45.794	9	1	-0.319	0.087	6.818	332	WS01033_L04
1	62.128	11	1	-0.456	0.104	3.955	332	WS01033_L04
1	82.301	16	3	-0.285	0.038	4.038	332	WS01033_L04
1	95.572	17	3	0.242	0.049	5.777	332	WS01033_L04
3	14.500	43	1	-0.316	0.069	5.144	332	WS01033_L04
3	14.500	43	2	0.160	0.039	4.417	332	WS01033_L04
3	17.158	45	1	0.228	0.062	4.585	332	WS01033_L04
3	47.928	49	1	0.230	0.068	5.050	332	WS01033_L04
4	116.928	82	2	0.275	0.038	4.280	332	WS01033_L04
9	17.951	169	1	0.318	0.075	5.551	332	WS01033_L04
9	28.608	170	2	0.240	0.032	3.925	332	WS01033_L04
9	28.608	170	3	-0.243	0.039	3.896	332	WS01033_L04
10	70.246	190	2	0.203	0.055	6.204	332	WS01033_L04
10	93.347	196	2	0.489	0.089	5.691	332	WS01033_L04
11	17.088	203	1	0.264	0.074	5.485	332	WS01033_L04
11	48.979	218	2	0.154	0.038	4.376	332	WS01033_L04
1	62.128	11	1	-0.477	0.123	4.328	333	WS01033_P06
1	95.572	17	3	0.284	0.045	4.266	333	WS01033_P06
2	93.504	32	2	0.231	0.032	3.867	333	WS01033_P06
3	154.505	69	4	0.154	0.044	4.372	333	WS01033_P06
4	116.928	82	2	0.244	0.037	4.201	333	WS01033_P06
11	40.960	210	2	0.368	0.060	4.079	333	WS01033_P06
1	50.955	10	1	-0.221	0.050	3.845	334	WS01034_A12
1	133.992	24	2	-0.203	0.034	3.842	334	WS01034_A12
1	141.021	25	2	0.231	0.046	5.154	334	WS01034_A12

2	75.550	31	1	0.253	0.063	4.831	334	WS01034_A12
3	47.928	49	1	0.247	0.077	5.884	334	WS01034_A12
5	42.979	97	2	-0.308	0.054	5.379	334	WS01034_A12
11	17.088	203	1	0.223	0.053	4.004	334	WS01034_A12
14	22.402	248	4	-0.383	0.073	6.912	334	WS01034_A12
14	46.637	252	4	-0.391	0.082	8.013	334	WS01034_A12
2	120.617	38	3	-0.176	0.044	4.336	335	WS01034_D08
4	75.958	77	3	0.316	0.114	4.772	335	WS01034_D08
4	92.039	79	2	-0.305	0.099	9.811	335	WS01034_D08
4	173.929	88	2	-0.211	0.047	4.455	335	WS01034_D08
6	25.914	114	1	0.323	0.094	8.783	335	WS01034_D08
9	36.227	165	4	0.247	0.080	7.724	335	WS01034_D08
11	17.088	203	1	0.265	0.061	5.721	335	WS01034_D08
11	29.069	205	1	-0.307	0.050	4.879	335	WS01034_D08
11	44.884	215	3	-0.276	0.043	4.496	335	WS01034_D08
12	22.441	227	3	0.221	0.045	4.487	335	WS01034_D08
12	28.493	228	2	-0.401	0.140	7.357	335	WS01034_D08
1	45.794	9	1	0.211	0.062	3.883	336	WS01034_F01
3	15.147	44	2	0.298	0.092	5.678	336	WS01034_F01
5	3.484	90	2	-0.247	0.076	4.482	336	WS01034_F01
6	54.066	119	4	0.238	0.053	4.677	336	WS01034_F01
6	69.073	121	4	-0.315	0.077	6.720	336	WS01034_F01
7	75.883	144	4	0.286	0.047	4.039	336	WS01034_F01
10	70.246	190	2	-0.247	0.034	4.257	336	WS01034_F01
10	80.088	194	2	0.289	0.041	4.494	336	WS01034_F01
10	93.347	196	2	-0.484	0.080	5.375	336	WS01034_F01
12	22.441	227	3	0.227	0.043	4.376	336	WS01034_F01
12	6.242	234	2	-0.189	0.030	3.962	336	WS01034_F01
12	22.629	237	1	-0.211	0.067	4.072	336	WS01034_F01
14	0.000	246	2	-0.195	0.040	4.925	336	WS01034_F01
3	20.512	46	2	0.224	0.049	4.295	337	WS0078_B06
4	0.000	71	4	0.186	0.062	4.093	337	WS0078_B06
6	69.073	121	3	0.186	0.039	4.772	337	WS0078_B06
9	0.000	166	2	0.266	0.074	6.374	337	WS0078_B06
10	21.864	188	1	-0.255	0.051	5.262	337	WS0078_B06
10	78.479	193	3	-0.482	0.077	9.413	337	WS0078_B06
10	86.151	195	3	0.210	0.034	4.127	337	WS0078_B06
2	120.617	38	4	0.295	0.066	5.540	338	WS0078_B23
4	82.462	78	2	0.293	0.068	8.467	338	WS0078_B23
4	97.576	80	2	-0.272	0.061	9.046	338	WS0078_B23
4	103.719	81	4	-0.208	0.040	3.966	338	WS0078_B23
9	36.227	165	4	-0.261	0.038	4.048	338	WS0078_B23
10	95.795	197	4	0.255	0.052	5.027	338	WS0078_B23
12	28.493	228	2	0.240	0.064	4.820	338	WS0078_B23
1	2.788	2	4	-0.250	0.057	4.051	339	WS0078_D12
1	40.464	7	3	0.211	0.049	5.388	339	WS0078_D12
1	43.578	8	3	0.222	0.055	6.023	339	WS0078_D12
1	45.794	9	3	-0.239	0.037	4.211	339	WS0078_D12
2	63.261	30	3	0.242	0.035	3.910	339	WS0078_D12
3	47.928	49	1	0.249	0.057	6.597	339	WS0078_D12
3	95.157	59	2	-0.208	0.073	5.894	339	WS0078_D12
3	104.681	60	2	0.210	0.058	4.140	339	WS0078_D12
4	55.925	76	1	-0.244	0.048	5.112	339	WS0078_D12
4	124.132	83	1	0.607	0.173	7.563	339	WS0078_D12

4	142.917	85	1	-0.314	0.082	9.311	339	WS0078_D12
6	95.249	126	1	0.391	0.091	6.589	339	WS0078_D12
6	106.702	128	2	0.419	0.118	4.583	339	WS0078_D12
9	3.127	168	1	-0.392	0.077	3.958	339	WS0078_D12
11	27.405	204	1	0.351	0.055	6.404	339	WS0078_D12
11	29.069	205	1	0.296	0.050	5.909	339	WS0078_D12
11	45.989	216	1	-0.270	0.060	6.895	339	WS0078_D12
12	22.629	237	1	0.219	0.035	4.105	339	WS0078_D12
2	127.147	40	3	-0.235	0.059	7.104	340	WS0078_D24
8	87.463	158	2	-0.239	0.058	4.679	340	WS0078_D24
11	27.405	204	1	0.482	0.141	15.539	340	WS0078_D24
11	29.069	205	1	0.350	0.092	9.937	340	WS0078_D24
11	45.989	216	1	-0.296	0.099	10.661	340	WS0078_D24
1	107.333	19	3	0.242	0.042	4.682	341	WS0078_F19
2	120.617	38	3	0.205	0.047	5.284	341	WS0078_F19
4	0.000	71	4	-0.233	0.054	4.155	341	WS0078_F19
4	75.958	77	3	-0.289	0.095	4.999	341	WS0078_F19
6	89.401	125	1	-0.290	0.043	4.001	341	WS0078_F19
8	50.001	154	1	0.306	0.075	7.515	341	WS0078_F19
9	59.963	177	4	-0.357	0.117	7.654	341	WS0078_F19
10	9.580	187	3	-0.412	0.095	5.218	341	WS0078_F19
10	70.246	190	2	0.241	0.047	4.604	341	WS0078_F19
10	78.479	193	3	-0.273	0.040	4.489	341	WS0078_F19
11	29.069	205	1	0.282	0.048	4.430	341	WS0078_F19
11	64.014	222	3	-0.192	0.044	4.903	341	WS0078_F19
1	40.464	7	4	0.245	0.074	4.556	342	WS0078_G10
1	131.686	23	1	0.547	0.123	5.103	342	WS0078_G10
2	48.839	29	1	-0.524	0.142	17.436	342	WS0078_G10
2	63.261	30	3	0.224	0.040	4.590	342	WS0078_G10
2	75.550	31	1	0.645	0.288	33.595	342	WS0078_G10
2	75.550	31	2	-0.410	0.230	16.362	342	WS0078_G10
2	93.504	32	2	-0.187	0.060	4.312	342	WS0078_G10
2	95.880	34	1	0.436	0.094	10.574	342	WS0078_G10
2	102.814	36	1	0.654	0.278	32.944	342	WS0078_G10
6	45.897	117	1	0.208	0.039	4.506	342	WS0078_G10
9	0.000	163	1	-0.592	0.108	8.095	342	WS0078_G10
11	27.405	204	1	0.294	0.043	5.135	342	WS0078_G10
12	6.242	234	4	0.346	0.068	3.869	342	WS0078_G10
1	123.316	20	4	0.422	0.128	7.847	343	WS0078_J19
3	154.505	69	4	-0.213	0.063	4.431	343	WS0078_J19
4	6.032	72	4	-0.179	0.055	4.563	343	WS0078_J19
7	17.610	131	3	-0.220	0.074	3.893	343	WS0078_J19
8	50.001	154	1	0.228	0.057	4.615	343	WS0078_J19
10	9.580	187	2	-0.288	0.102	6.207	343	WS0078_J19
10	78.479	193	3	-0.385	0.060	6.342	343	WS0078_J19
11	64.014	222	3	-0.240	0.056	5.867	343	WS0078_J19
11	64.014	222	4	-0.258	0.059	4.865	343	WS0078_J19
1	2.788	2	3	-0.278	0.099	9.879	344	WS0078_K09
2	120.617	38	3	-0.265	0.054	5.441	344	WS0078_K09
2	126.181	39	2	0.243	0.049	4.134	344	WS0078_K09
4	124.132	83	1	0.438	0.086	3.864	344	WS0078_K09
5	16.219	93	1	0.230	0.052	5.410	344	WS0078_K09
5	31.191	95	1	0.212	0.037	3.895	344	WS0078_K09
5	86.669	106	4	-0.458	0.140	5.609	344	WS0078_K09

6	49.100	118	4	0.408	0.113	5.157	344	WS0078_K09
7	54.200	136	2	0.271	0.107	4.297	344	WS0078_K09
7	62.787	139	2	0.329	0.094	8.451	344	WS0078_K09
7	74.560	143	1	-0.252	0.051	5.341	344	WS0078_K09
8	50.001	154	2	-0.183	0.056	4.776	344	WS0078_K09
10	35.204	189	1	-0.219	0.040	4.177	344	WS0078_K09
10	70.246	190	2	0.314	0.110	9.278	344	WS0078_K09
11	40.960	210	4	0.299	0.055	4.808	344	WS0078_K09
11	43.491	214	3	0.221	0.050	5.001	344	WS0078_K09
11	43.491	214	4	0.278	0.086	7.516	344	WS0078_K09
11	44.884	215	3	-0.238	0.057	5.716	344	WS0078_K09
11	44.884	215	4	-0.318	0.050	4.400	344	WS0078_K09
11	57.661	220	4	-0.221	0.079	6.924	344	WS0078_K09
11	64.014	222	3	-0.242	0.055	5.471	344	WS0078_K09
11	64.014	222	4	0.305	0.080	7.022	344	WS0078_K09
12	6.242	234	2	0.204	0.042	3.909	344	WS0078_K09
12	51.382	242	1	-0.364	0.097	4.395	344	WS0078_K09
3	137.891	64	3	0.299	0.064	4.626	345	WS00911_L17
4	75.958	77	3	-0.326	0.094	5.599	345	WS00911_L17
6	106.702	128	2	0.404	0.069	4.264	345	WS00911_L17
9	59.963	177	4	-0.252	0.047	4.276	345	WS00911_L17
10	78.479	193	3	-0.281	0.040	3.901	345	WS00911_L17
10	80.088	194	2	-0.243	0.042	5.265	345	WS00911_L17
14	46.637	252	4	0.332	0.074	5.551	345	WS00911_L17
2	48.839	29	1	0.195	0.049	4.588	346	WS00912_A14
4	124.132	83	1	0.577	0.139	7.039	346	WS00912_A14
10	78.479	193	4	-0.316	0.065	3.956	346	WS00912_A14
10	80.088	194	2	-0.305	0.060	5.389	346	WS00912_A14
12	22.441	227	3	-0.267	0.050	4.402	346	WS00912_A14
12	14.406	230	4	-0.350	0.102	8.809	346	WS00912_A14
1	38.326	6	2	0.187	0.055	4.232	347	WS00828_F12
3	14.500	43	1	-0.222	0.041	4.558	347	WS00828_F12
3	17.158	45	1	0.183	0.036	4.049	347	WS00828_F12
3	47.928	49	1	0.253	0.067	7.547	347	WS00828_F12
3	69.071	52	1	0.288	0.058	4.922	347	WS00828_F12
4	55.925	76	1	-0.200	0.041	4.464	347	WS00828_F12
5	31.191	95	1	-0.346	0.118	13.286	347	WS00828_F12
8	50.001	154	1	0.227	0.054	6.023	347	WS00828_F12
12	22.629	237	1	0.234	0.037	4.180	347	WS00828_F12
13	3.386	245	1	0.300	0.054	6.127	347	WS00828_F12
1	107.333	19	3	0.225	0.045	4.041	348	WS01031_F07
3	15.147	44	2	-0.431	0.128	9.907	348	WS01031_F07
3	47.928	49	1	0.259	0.068	4.654	348	WS01031_F07
6	56.188	120	2	0.240	0.039	4.272	348	WS01031_F07
6	89.401	125	1	-0.283	0.057	3.959	348	WS01031_F07
8	39.090	152	4	0.251	0.085	8.262	348	WS01031_F07
9	28.608	170	2	-0.334	0.080	9.784	348	WS01031_F07
9	28.608	170	3	0.278	0.043	3.961	348	WS01031_F07
9	59.963	177	4	-0.286	0.066	6.898	348	WS01031_F07
11	29.069	205	2	-0.240	0.059	6.580	348	WS01031_F07
11	57.661	220	4	0.222	0.042	4.109	348	WS01031_F07
12	28.493	228	2	-0.251	0.062	4.887	348	WS01031_F07
2	127.147	40	3	-0.169	0.061	5.192	349	WS00829_B23
3	95.157	59	2	0.286	0.089	8.364	349	WS00829_B23

4	55.925	76	1	-0.242	0.053	3.901	349	WS00829_B23
6	95.249	126	1	0.427	0.168	7.272	349	WS00829_B23
8	39.090	152	4	0.213	0.067	6.892	349	WS00829_B23
9	36.227	165	3	-0.272	0.053	4.590	349	WS00829_B23
11	0.000	199	3	-0.303	0.053	4.368	349	WS00829_B23
11	27.405	204	1	0.329	0.058	4.650	349	WS00829_B23
1	133.992	24	2	0.168	0.047	4.361	350	WS01031_J04
4	55.925	76	1	-0.227	0.068	6.053	350	WS01031_J04
4	82.462	78	1	-0.195	0.046	4.423	350	WS01031_J04
4	142.917	85	1	-0.236	0.044	4.397	350	WS01031_J04
5	0.000	89	1	-0.258	0.061	5.965	350	WS01031_J04
5	16.219	93	1	-0.272	0.055	5.317	350	WS01031_J04
6	95.249	126	1	0.262	0.110	6.249	350	WS01031_J04
7	74.560	143	1	0.172	0.042	4.036	350	WS01031_J04
8	62.580	155	1	0.409	0.181	7.004	350	WS01031_J04
10	78.479	193	3	-0.202	0.039	3.841	350	WS01031_J04
10	86.151	195	3	0.286	0.050	5.030	350	WS01031_J04
4	75.958	77	3	-0.287	0.086	4.261	351	WS00825_E09
4	124.132	83	1	0.530	0.150	5.778	351	WS00825_E09
4	142.917	85	1	-0.282	0.089	7.428	351	WS00825_E09
6	49.100	118	4	0.423	0.112	3.931	351	WS00825_E09
11	45.989	216	1	-0.259	0.067	5.544	351	WS00825_E09
3	47.928	49	1	0.270	0.050	3.961	352	WS00825_F01
4	75.958	77	3	-0.213	0.057	4.038	352	WS00825_F01
9	59.963	177	4	-0.298	0.084	4.081	352	WS00825_F01
9	69.046	181	2	0.268	0.056	4.478	352	WS00825_F01
10	70.246	190	2	0.329	0.083	9.292	352	WS00825_F01
10	78.479	193	3	-0.257	0.040	5.193	352	WS00825_F01
12	22.629	237	1	0.173	0.064	4.866	352	WS00825_F01
12	32.473	238	1	0.161	0.056	4.312	352	WS00825_F01
11	0.000	199	3	-0.322	0.054	5.015	353	WS00825_G22
11	17.088	203	1	-0.260	0.060	6.030	353	WS00825_G22
11	27.405	204	1	0.334	0.051	5.241	353	WS00825_G22
11	29.069	205	1	0.350	0.092	9.370	353	WS00825_G22
11	40.960	210	4	-0.250	0.063	5.538	353	WS00825_G22
11	45.989	216	1	-0.290	0.083	8.371	353	WS00825_G22
1	62.128	11	1	-0.549	0.116	7.282	354	WS00825_H17
3	15.147	44	2	-0.206	0.063	4.085	354	WS00825_H17
3	144.647	67	1	0.342	0.111	7.003	354	WS00825_H17
3	154.505	69	1	-0.335	0.088	8.575	354	WS00825_H17
5	0.000	89	1	-0.344	0.077	7.536	354	WS00825_H17
5	16.219	93	1	-0.265	0.065	6.289	354	WS00825_H17
6	45.897	117	1	0.299	0.065	6.237	354	WS00825_H17
6	49.100	118	4	0.360	0.120	7.883	354	WS00825_H17
6	89.401	125	1	0.285	0.045	4.375	354	WS00825_H17
9	0.000	166	1	0.175	0.046	4.403	354	WS00825_H17
9	55.358	175	1	-0.285	0.053	5.149	354	WS00825_H17
10	21.864	188	1	-0.330	0.051	4.969	354	WS00825_H17
13	3.386	245	1	-0.299	0.064	6.231	354	WS00825_H17
1	40.464	7	4	0.349	0.046	4.501	355	WS00825_J18
2	95.880	34	1	-0.320	0.050	4.764	355	WS00825_J18
4	142.917	85	1	-0.196	0.041	3.901	355	WS00825_J18
5	16.219	93	2	0.239	0.043	4.263	355	WS00825_J18
5	86.669	106	4	-0.482	0.143	7.662	355	WS00825_J18

7	54.200	136	2	0.271	0.078	3.849	355	WS00825_J18
7	62.787	139	4	-0.257	0.052	3.926	355	WS00825_J18
7	67.154	140	4	-0.351	0.112	7.974	355	WS00825_J18
7	75.883	144	2	0.442	0.120	5.647	355	WS00825_J18
7	75.883	144	4	-0.374	0.084	9.652	355	WS00825_J18
9	2.011	167	4	0.290	0.052	6.030	355	WS00825_J18
9	55.358	175	1	0.298	0.049	4.776	355	WS00825_J18
9	59.963	177	4	-0.256	0.069	8.365	355	WS00825_J18
9	61.113	178	1	-0.230	0.066	4.653	355	WS00825_J18
11	45.989	216	1	-0.257	0.057	5.453	355	WS00825_J18
12	22.629	237	1	0.252	0.054	5.164	355	WS00825_J18
12	32.473	238	1	0.229	0.043	4.102	355	WS00825_J18
12	51.382	242	1	-0.398	0.108	7.578	355	WS00825_J18
3	47.928	49	1	0.212	0.067	4.702	356	WS00825_K05
4	142.917	85	1	-0.217	0.056	4.026	356	WS00825_K05
6	25.914	114	1	-0.262	0.093	6.526	356	WS00825_K05
6	45.897	117	1	0.281	0.099	7.179	356	WS00825_K05
7	15.555	130	3	-0.221	0.041	5.051	356	WS00825_K05
9	49.013	173	2	-0.203	0.041	4.673	356	WS00825_K05
1	43.578	8	2	-0.257	0.030	4.128	357	WS00825_M13
1	69.854	13	2	0.181	0.032	4.375	357	WS00825_M13
1	71.592	14	2	0.183	0.033	4.520	357	WS00825_M13
1	77.177	15	2	0.174	0.031	4.137	357	WS00825_M13
1	131.686	23	2	-0.423	0.060	4.333	357	WS00825_M13
1	141.021	25	2	0.226	0.036	4.881	357	WS00825_M13
2	93.504	32	2	0.220	0.028	3.858	357	WS00825_M13
2	126.181	39	2	-0.248	0.044	5.639	357	WS00825_M13
2	126.181	39	4	0.756	0.200	16.495	357	WS00825_M13
5	16.219	93	2	0.203	0.031	4.094	357	WS00825_M13
6	106.702	128	2	0.567	0.140	8.417	357	WS00825_M13
8	95.937	159	3	-0.303	0.085	8.396	357	WS00825_M13
9	17.951	169	2	-0.245	0.056	6.524	357	WS00825_M13
9	49.013	173	2	-0.202	0.032	4.218	357	WS00825_M13
10	70.246	190	2	0.219	0.030	4.155	357	WS00825_M13
10	80.088	194	2	-0.250	0.028	3.847	357	WS00825_M13
11	64.014	222	4	0.253	0.045	3.977	357	WS00825_M13
12	0.000	226	3	-0.201	0.044	4.389	357	WS00825_M13
1	62.128	11	1	-0.598	0.195	6.809	358	WS00825_M22
2	63.261	30	3	0.211	0.037	4.271	358	WS00825_M22
2	95.880	34	3	0.230	0.038	3.841	358	WS00825_M22
3	47.928	49	1	0.227	0.039	3.883	358	WS00825_M22
6	45.897	117	1	0.207	0.040	3.923	358	WS00825_M22
11	27.405	204	1	0.318	0.057	5.627	358	WS00825_M22
12	6.242	234	2	-0.245	0.036	4.090	358	WS00825_M22
12	22.629	237	1	0.187	0.041	4.061	358	WS00825_M22
12	32.473	238	1	0.205	0.048	4.700	358	WS00825_M22
3	104.681	60	2	0.231	0.043	5.014	359	WS0262_B21
4	116.928	82	4	0.284	0.066	6.214	359	WS0262_B21
5	3.484	90	2	-0.368	0.072	5.256	359	WS0262_B21
5	16.219	93	2	0.298	0.047	5.538	359	WS0262_B21
13	3.386	245	1	0.209	0.113	8.068	359	WS0262_B21
2	127.147	40	3	-0.197	0.058	6.147	360	WS0262_C18
3	116.958	63	4	-0.210	0.052	4.236	360	WS0262_C18
4	124.132	83	2	0.233	0.087	4.561	360	WS0262_C18

8	11.974	150	1	-0.247	0.053	4.698	360	WS0262_C18
11	27.405	204	1	0.323	0.068	6.112	360	WS0262_C18
11	29.069	205	1	0.277	0.058	5.231	360	WS0262_C18
11	45.989	216	1	-0.239	0.067	5.907	360	WS0262_C18
13	3.386	245	1	-0.177	0.051	4.507	360	WS0262_C18
1	40.464	7	4	0.430	0.086	7.931	361	WS0262_E08
2	120.617	38	3	0.273	0.047	4.436	361	WS0262_E08
8	50.001	154	1	0.179	0.067	5.826	361	WS0262_E08
9	59.963	177	4	-0.337	0.091	8.956	361	WS0262_E08
10	78.479	193	3	-0.346	0.046	4.357	361	WS0262_E08
11	0.000	199	3	-0.238	0.049	4.406	361	WS0262_E08
11	29.069	205	1	0.258	0.065	5.048	361	WS0262_E08
11	35.604	207	3	-0.175	0.050	4.540	361	WS0262_E08
11	42.600	213	3	-0.208	0.063	5.710	361	WS0262_E08
12	51.382	242	1	-0.404	0.136	4.596	361	WS0262_E08
1	77.177	15	2	-0.182	0.036	4.040	362	WS0262_E17
1	95.572	17	2	-0.343	0.081	9.085	362	WS0262_E17
3	47.928	49	1	0.252	0.074	5.574	362	WS0262_E17
3	154.505	69	4	-0.211	0.037	4.243	362	WS0262_E17
9	59.963	177	4	-0.368	0.105	11.571	362	WS0262_E17
9	3.127	168	1	-0.439	0.110	5.331	363	WS0262_F23
10	78.479	193	3	-0.426	0.079	7.376	363	WS0262_F23
10	80.088	194	2	-0.197	0.041	4.698	363	WS0262_F23
14	0.000	246	2	0.199	0.042	4.803	363	WS0262_F23
1	15.589	3	2	-0.263	0.069	6.774	364	WS0262_G19
1	22.419	4	3	-0.552	0.247	13.790	364	WS0262_G19
1	29.800	5	1	0.325	0.058	5.225	364	WS0262_G19
1	29.800	5	2	0.577	0.234	23.156	364	WS0262_G19
1	38.326	6	2	-0.521	0.211	20.218	364	WS0262_G19
1	40.464	7	3	-0.302	0.052	5.885	364	WS0262_G19
1	40.464	7	4	0.344	0.103	7.670	364	WS0262_G19
1	43.578	8	2	0.338	0.111	11.484	364	WS0262_G19
1	43.578	8	3	-0.361	0.085	9.652	364	WS0262_G19
1	45.794	9	3	0.475	0.142	11.646	364	WS0262_G19
1	50.955	10	1	-0.334	0.092	8.197	364	WS0262_G19
1	62.128	11	2	-0.443	0.164	5.789	364	WS0262_G19
1	69.854	13	2	-0.256	0.064	6.360	364	WS0262_G19
1	71.592	14	2	-0.257	0.066	6.521	364	WS0262_G19
5	0.000	89	3	-0.241	0.044	4.061	364	WS0262_G19
5	57.017	102	4	0.260	0.078	3.845	364	WS0262_G19
6	45.897	117	1	0.236	0.046	4.314	364	WS0262_G19
6	69.073	121	3	0.263	0.042	4.751	364	WS0262_G19
7	17.610	131	3	-0.362	0.108	6.180	364	WS0262_G19
7	54.200	136	2	-0.284	0.068	3.898	364	WS0262_G19
7	105.477	147	2	0.209	0.093	3.859	364	WS0262_G19
8	87.463	158	2	0.296	0.073	6.954	364	WS0262_G19
9	0.000	163	1	-0.433	0.074	4.136	364	WS0262_G19
9	81.073	183	3	0.280	0.045	4.678	364	WS0262_G19
10	21.864	188	1	0.289	0.053	4.657	364	WS0262_G19
10	93.347	196	1	-0.576	0.143	7.971	364	WS0262_G19
11	17.088	203	2	0.218	0.064	6.102	364	WS0262_G19
11	35.604	207	3	-0.213	0.035	3.968	364	WS0262_G19
13	1.815	244	2	-0.298	0.069	6.814	364	WS0262_G19
13	1.815	244	4	0.288	0.074	5.950	364	WS0262_G19

1	2.788	2	3	-0.205	0.044	4.243	365	WS0262_I08
9	81.073	183	1	-0.240	0.058	4.819	365	WS0262_I08
11	17.088	203	1	-0.210	0.055	4.600	365	WS0262_I08
11	27.405	204	1	0.287	0.058	5.004	365	WS0262_I08
11	29.069	205	1	0.276	0.058	4.964	365	WS0262_I08
11	43.491	214	3	-0.294	0.071	6.988	365	WS0262_I08
11	44.884	215	3	0.261	0.056	5.433	365	WS0262_I08
11	45.989	216	1	-0.246	0.055	4.616	365	WS0262_I08
11	46.026	217	1	-0.246	0.050	4.212	365	WS0262_I08
12	51.382	242	1	-0.385	0.101	4.164	365	WS0262_I08
1	123.316	20	4	0.309	0.097	5.013	366	WS0262_I23
3	47.928	49	1	0.189	0.058	4.358	366	WS0262_I23
4	75.958	77	3	-0.321	0.072	4.442	366	WS0262_I23
4	124.132	83	1	0.450	0.101	4.076	366	WS0262_I23
6	95.249	126	1	0.353	0.137	4.771	366	WS0262_I23
11	17.088	203	1	-0.253	0.095	7.142	366	WS0262_I23
11	29.069	205	1	0.396	0.111	8.611	366	WS0262_I23
11	45.989	216	1	-0.276	0.076	5.697	366	WS0262_I23
12	28.493	228	2	-0.274	0.080	4.124	366	WS0262_I23
6	56.188	120	2	0.287	0.034	4.290	367	WS01030_I11
6	95.249	126	1	0.278	0.147	4.740	367	WS01030_I11
7	59.789	137	3	-0.251	0.046	4.702	367	WS01030_I11
7	70.278	141	3	0.310	0.077	7.806	367	WS01030_I11
8	11.974	150	1	-0.258	0.064	4.076	367	WS01030_I11
10	70.246	190	2	0.173	0.039	4.853	367	WS01030_I11
10	104.214	198	2	-0.256	0.032	4.719	367	WS01030_I11
11	35.604	207	3	-0.276	0.054	5.502	367	WS01030_I11
11	40.960	210	2	0.496	0.115	7.167	367	WS01030_I11
11	64.014	222	3	-0.283	0.066	6.645	367	WS01030_I11
1	2.788	2	4	-0.185	0.040	3.945	368	WS01030_J07
1	38.326	6	4	-0.216	0.074	5.760	368	WS01030_J07
1	40.464	7	4	0.302	0.116	10.179	368	WS01030_J07
1	43.578	8	2	0.179	0.044	4.082	368	WS01030_J07
1	62.128	11	1	-0.444	0.082	4.124	368	WS01030_J07
4	92.039	79	2	-0.148	0.047	4.634	368	WS01030_J07
4	97.576	80	2	0.232	0.055	5.061	368	WS01030_J07
4	116.928	82	2	0.295	0.053	5.285	368	WS01030_J07
6	45.897	117	1	0.295	0.045	4.312	368	WS01030_J07
8	11.974	150	1	-0.236	0.041	3.855	368	WS01030_J07
9	17.951	169	2	0.283	0.038	5.358	368	WS01030_J07
9	55.358	175	1	-0.346	0.051	4.904	368	WS01030_J07
9	55.358	175	3	0.387	0.091	4.524	368	WS01030_J07
10	78.479	193	4	-0.194	0.048	5.586	368	WS01030_J07
11	40.960	210	2	0.615	0.195	11.291	368	WS01030_J07
11	40.960	210	4	-0.380	0.065	6.430	368	WS01030_J07
11	48.979	218	2	0.122	0.039	3.972	368	WS01030_J07
11	56.921	219	1	-0.405	0.091	4.559	368	WS01030_J07
12	6.242	234	4	0.333	0.047	4.210	368	WS01030_J07
3	47.928	49	1	0.260	0.057	4.834	369	WS01030_L01
4	75.958	77	3	-0.298	0.092	4.588	369	WS01030_L01
6	95.249	126	1	0.354	0.079	4.122	369	WS01030_L01
9	49.013	173	1	0.242	0.047	4.159	369	WS01030_L01
9	58.045	176	1	0.178	0.077	3.875	369	WS01030_L01
9	59.963	177	4	-0.311	0.081	4.889	369	WS01030_L01

11	27.405	204	1	0.377	0.054	4.899	369	WS01030_L01
11	29.069	205	1	0.331	0.067	5.793	369	WS01030_L01
11	42.600	213	3	-0.144	0.041	4.138	369	WS01030_L01
12	51.382	242	1	-0.366	0.119	4.092	369	WS01030_L01
1	22.419	4	3	0.319	0.056	4.135	370	WS01030_L12
2	127.147	40	3	-0.158	0.037	3.895	370	WS01030_L12
4	55.925	76	1	-0.257	0.050	4.685	370	WS01030_L12
5	31.191	95	1	-0.239	0.049	4.777	370	WS01030_L12
6	89.401	125	1	-0.368	0.075	7.429	370	WS01030_L12
6	95.249	126	1	0.431	0.112	8.117	370	WS01030_L12
8	11.974	150	1	-0.227	0.040	3.892	370	WS01030_L12
8	39.090	152	4	0.209	0.068	5.445	370	WS01030_L12
11	27.405	204	1	0.384	0.068	6.783	370	WS01030_L12
11	29.069	205	1	0.321	0.068	6.696	370	WS01030_L12
11	45.989	216	1	-0.208	0.050	4.853	370	WS01030_L12
11	64.014	222	3	-0.274	0.077	8.200	370	WS01030_L12
1	38.326	6	2	0.207	0.052	4.206	371	WS01030_N04
6	89.401	125	1	-0.316	0.042	4.727	371	WS01030_N04
6	95.249	126	1	0.457	0.122	9.045	371	WS01030_N04
7	74.560	143	1	-0.198	0.037	4.013	371	WS01030_N04
11	44.884	215	3	-0.181	0.038	4.171	371	WS01030_N04
11	64.014	222	3	-0.266	0.076	8.395	371	WS01030_N04
1	131.686	23	1	0.521	0.122	4.701	372	WS01030_O03
2	120.617	38	3	0.280	0.054	6.147	372	WS01030_O03
2	127.147	40	3	-0.286	0.071	8.050	372	WS01030_O03
3	9.953	42	4	0.167	0.051	3.899	372	WS01030_O03
4	55.925	76	1	-0.249	0.043	4.242	372	WS01030_O03
4	82.462	78	1	-0.247	0.058	6.222	372	WS01030_O03
5	16.219	93	1	-0.217	0.040	4.313	372	WS01030_O03
5	31.191	95	1	-0.324	0.076	8.164	372	WS01030_O03
6	89.401	125	1	-0.258	0.059	6.322	372	WS01030_O03
6	95.249	126	1	0.325	0.091	6.226	372	WS01030_O03
7	59.789	137	3	-0.193	0.037	4.146	372	WS01030_O03
8	11.974	150	1	-0.272	0.047	5.028	372	WS01030_O03
9	0.000	166	1	0.306	0.077	8.265	372	WS01030_O03
11	27.405	204	1	0.290	0.050	5.351	372	WS01030_O03
11	35.604	207	3	-0.249	0.056	6.341	372	WS01030_O03
11	37.143	209	3	0.223	0.044	5.015	372	WS01030_O03
1	38.326	6	2	-0.184	0.040	4.455	373	WS01031_A08
2	63.261	30	3	-0.322	0.075	8.695	373	WS01031_A08
10	93.347	196	1	0.414	0.078	4.105	373	WS01031_A08
11	40.960	210	2	-0.376	0.080	3.971	373	WS01031_A08
14	44.898	251	3	-0.261	0.036	3.955	373	WS01031_A08
1	29.800	5	1	0.269	0.065	4.736	374	WS01031_A24
1	77.177	15	2	0.218	0.036	4.228	374	WS01031_A24
3	141.443	65	3	0.290	0.076	4.077	374	WS01031_A24
4	75.958	77	3	0.393	0.158	8.868	374	WS01031_A24
4	103.719	81	4	0.250	0.073	5.561	374	WS01031_A24
5	16.219	93	2	-0.280	0.045	4.710	374	WS01031_A24
5	84.347	105	2	0.144	0.034	4.205	374	WS01031_A24
6	26.350	115	2	0.224	0.034	4.007	374	WS01031_A24
7	15.555	130	3	0.399	0.106	9.437	374	WS01031_A24
9	2.011	167	4	-0.256	0.050	4.017	374	WS01031_A24
10	80.088	194	2	0.233	0.036	4.015	374	WS01031_A24

11	37.143	209	3	-0.216	0.041	4.505	374	WS01031_A24
11	48.979	218	2	0.228	0.039	4.483	374	WS01031_A24
12	22.629	237	1	-0.282	0.065	4.804	374	WS01031_A24
14	44.898	251	3	-0.275	0.060	6.413	374	WS01031_A24
1	22.419	4	3	0.283	0.100	6.385	375	WS00821_P10
1	38.326	6	2	0.182	0.051	4.784	375	WS00821_P10
3	137.891	64	3	0.231	0.058	6.879	375	WS00821_P10
8	87.463	158	2	-0.117	0.042	4.029	375	WS00821_P10
11	17.088	203	2	-0.317	0.057	3.972	375	WS00821_P10
11	27.405	204	1	0.260	0.064	6.066	375	WS00821_P10
11	29.069	205	1	0.367	0.115	10.841	375	WS00821_P10
11	29.069	205	2	-0.403	0.176	16.499	375	WS00821_P10
11	41.984	212	2	-0.196	0.091	8.538	375	WS00821_P10
11	45.989	216	1	-0.414	0.095	9.155	375	WS00821_P10
11	45.989	216	2	-0.201	0.093	8.829	375	WS00821_P10
11	56.921	219	2	0.455	0.173	6.163	375	WS00821_P10
12	28.493	228	2	-0.354	0.084	3.895	375	WS00821_P10
14	22.402	248	3	0.270	0.040	4.716	375	WS00821_P10
14	43.392	250	1	-0.511	0.196	12.506	375	WS00821_P10
2	48.839	29	4	-0.371	0.094	8.453	376	WS01027_G11
6	54.066	119	4	-0.207	0.060	5.529	376	WS01027_G11
6	69.073	121	4	0.329	0.106	9.890	376	WS01027_G11
7	15.555	130	2	0.246	0.044	4.400	376	WS01027_G11
7	59.789	137	3	-0.275	0.067	6.439	376	WS01027_G11
7	70.278	141	3	0.247	0.057	5.444	376	WS01027_G11
7	91.487	146	3	0.225	0.044	4.184	376	WS01027_G11
1	45.794	9	3	-0.200	0.047	4.732	377	WS00823_K13
4	82.462	78	2	0.292	0.079	5.545	377	WS00823_K13
4	97.576	80	2	-0.171	0.042	4.800	377	WS00823_K13
4	124.132	83	1	-0.464	0.100	4.471	377	WS00823_K13
6	87.891	124	2	0.274	0.063	6.434	377	WS00823_K13
6	106.702	128	2	-0.467	0.091	5.576	377	WS00823_K13
7	74.560	143	1	-0.419	0.134	11.644	377	WS00823_K13
8	87.463	158	1	-0.183	0.050	4.297	377	WS00823_K13
9	0.000	166	1	0.194	0.048	4.116	377	WS00823_K13
11	56.921	219	1	0.296	0.124	5.496	377	WS00823_K13
13	3.386	245	1	0.189	0.047	4.109	377	WS00823_K13
14	0.000	246	1	-0.370	0.154	13.377	377	WS00823_K13
2	75.550	31	1	0.309	0.061	5.406	378	WS00823_P15
4	142.917	85	1	-0.253	0.049	4.297	378	WS00823_P15
6	87.891	124	2	0.193	0.057	5.724	378	WS00823_P15
6	89.401	125	2	0.483	0.118	11.657	378	WS00823_P15
9	55.358	175	1	0.267	0.045	3.976	378	WS00823_P15
9	59.963	177	1	-0.245	0.057	5.198	378	WS00823_P15
1	95.572	17	3	0.259	0.061	6.303	379	WS00822_O23
2	102.814	36	1	0.305	0.072	8.794	379	WS00822_O23
3	47.928	49	1	0.210	0.043	5.522	379	WS00822_O23
4	6.032	72	4	-0.215	0.053	4.197	379	WS00822_O23
6	25.914	114	1	-0.225	0.042	5.458	379	WS00822_O23
8	11.974	150	1	-0.281	0.058	7.561	379	WS00822_O23
10	9.580	187	3	-0.378	0.079	6.580	379	WS00822_O23
10	78.479	193	3	-0.266	0.036	4.029	379	WS00822_O23
10	93.347	196	1	0.452	0.070	5.666	379	WS00822_O23
11	0.000	199	3	-0.303	0.040	4.743	379	WS00822_O23

11	29.069	205	1	0.237	0.031	4.045	379	WS00822_O23
1	38.326	6	2	0.210	0.034	4.102	380	WS00823_E13
1	123.316	20	4	0.481	0.152	11.472	380	WS00823_E13
6	81.996	123	3	-0.365	0.100	4.507	380	WS00823_E13
10	70.246	190	2	0.290	0.046	5.623	380	WS00823_E13
12	28.493	228	2	-0.198	0.053	4.001	380	WS00823_E13
4	92.039	79	2	-0.240	0.038	4.335	381	WS00916_K21
6	49.100	118	2	0.310	0.079	4.599	381	WS00916_K21
6	56.188	120	2	0.281	0.063	6.694	381	WS00916_K21
6	69.073	121	3	-0.240	0.042	4.139	381	WS00916_K21
7	75.883	144	2	0.474	0.095	5.776	381	WS00916_K21
7	75.883	144	4	-0.321	0.055	4.995	381	WS00916_K21
7	105.477	147	2	0.326	0.092	5.146	381	WS00916_K21
9	59.963	177	4	-0.267	0.061	5.614	381	WS00916_K21
9	69.046	181	2	-0.328	0.100	6.129	381	WS00916_K21
10	78.479	193	3	-0.281	0.044	4.367	381	WS00916_K21
10	104.214	198	2	-0.212	0.039	4.096	381	WS00916_K21
11	29.069	205	2	-0.203	0.047	4.343	381	WS00916_K21
11	35.604	207	3	-0.207	0.063	6.226	381	WS00916_K21
11	37.143	209	3	0.160	0.041	4.102	381	WS00916_K21
11	41.984	212	2	-0.228	0.046	5.213	381	WS00916_K21
11	42.600	213	3	-0.164	0.040	3.964	381	WS00916_K21
11	43.491	214	3	0.217	0.070	6.905	381	WS00916_K21
11	44.884	215	3	-0.170	0.045	4.463	381	WS00916_K21
11	64.014	222	3	-0.269	0.090	8.917	381	WS00916_K21
1	82.301	16	3	-0.195	0.033	4.308	382	WS00917_B01
3	47.928	49	1	0.231	0.054	5.138	382	WS00917_B01
5	31.191	95	1	-0.246	0.062	5.903	382	WS00917_B01
6	89.401	125	1	-0.269	0.044	4.160	382	WS00917_B01
6	95.249	126	1	0.374	0.108	7.153	382	WS00917_B01
8	11.974	150	1	-0.252	0.053	5.038	382	WS00917_B01
10	78.479	193	3	-0.437	0.073	9.475	382	WS00917_B01
10	86.151	195	3	0.192	0.043	5.607	382	WS00917_B01
11	35.604	207	3	-0.192	0.049	6.274	382	WS00917_B01
11	37.143	209	3	0.172	0.040	5.211	382	WS00917_B01
11	64.014	222	3	-0.298	0.077	9.969	382	WS00917_B01
3	9.953	42	4	-0.181	0.059	4.824	383	WS00917_N15
5	0.000	89	3	-0.179	0.029	4.280	383	WS00917_N15
5	7.672	92	1	-0.227	0.072	5.363	383	WS00917_N15
9	59.963	177	4	-0.236	0.055	4.360	383	WS00917_N15
11	17.088	203	1	-0.216	0.065	4.541	383	WS00917_N15
11	29.069	205	1	0.374	0.088	6.426	383	WS00917_N15
11	45.989	216	1	-0.281	0.091	6.354	383	WS00917_N15
1	2.788	2	4	-0.200	0.037	4.499	384	WS00917_O05
3	47.928	49	1	0.272	0.063	4.862	384	WS00917_O05
3	154.505	69	4	-0.187	0.046	4.797	384	WS00917_O05
6	89.401	125	1	-0.318	0.067	5.201	384	WS00917_O05
7	74.560	143	2	0.183	0.040	5.286	384	WS00917_O05
9	59.963	177	4	-0.390	0.143	13.280	384	WS00917_O05
10	78.479	193	4	-0.264	0.068	5.792	384	WS00917_O05
10	80.088	194	2	-0.283	0.046	5.368	384	WS00917_O05
11	27.405	204	1	0.338	0.048	3.976	384	WS00917_O05
11	29.069	205	1	0.279	0.050	3.986	384	WS00917_O05
11	45.989	216	1	-0.178	0.059	4.524	384	WS00917_O05

12	22.441	227	3	-0.242	0.042	3.934	384	WS00917_O05
1	62.128	11	1	-0.541	0.119	6.485	385	WS00918_B02
3	144.647	67	1	0.282	0.077	4.176	385	WS00918_B02
5	0.000	89	1	-0.307	0.076	6.368	385	WS00918_B02
6	45.897	117	1	0.247	0.053	4.531	385	WS00918_B02
6	49.100	118	4	0.273	0.073	4.971	385	WS00918_B02
6	87.891	124	2	0.259	0.063	6.648	385	WS00918_B02
6	89.401	125	2	0.294	0.052	4.511	385	WS00918_B02
8	62.580	155	1	0.332	0.084	4.536	385	WS00918_B02
9	0.000	166	1	0.231	0.073	6.038	385	WS00918_B02
11	40.960	210	2	0.501	0.088	6.813	385	WS00918_B02
11	43.491	214	4	0.178	0.047	5.934	385	WS00918_B02
3	144.593	66	4	-0.191	0.049	4.464	386	WS00918_B17
3	154.505	69	4	-0.191	0.044	3.949	386	WS00918_B17
9	59.963	177	4	-0.272	0.078	7.110	386	WS00918_B17
11	27.405	204	1	0.322	0.043	4.147	386	WS00918_B17
11	29.069	205	1	0.295	0.067	6.271	386	WS00918_B17
11	44.884	215	4	0.225	0.050	4.142	386	WS00918_B17
2	120.617	38	3	0.226	0.043	4.386	387	WS00918_D23
2	127.147	40	3	-0.209	0.061	6.183	387	WS00918_D23
3	47.928	49	1	0.324	0.100	10.486	387	WS00918_D23
4	55.925	76	1	-0.264	0.060	5.810	387	WS00918_D23
4	75.958	77	3	-0.388	0.163	6.805	387	WS00918_D23
4	82.462	78	1	-0.320	0.066	6.985	387	WS00918_D23
5	31.191	95	1	-0.293	0.087	9.071	387	WS00918_D23
6	89.401	125	1	-0.283	0.044	4.588	387	WS00918_D23
6	95.249	126	1	0.432	0.159	9.588	387	WS00918_D23
11	27.405	204	1	0.332	0.055	5.808	387	WS00918_D23
11	29.069	205	1	0.306	0.066	6.921	387	WS00918_D23
11	45.989	216	1	-0.247	0.051	5.371	387	WS00918_D23
12	28.493	228	2	-0.279	0.104	4.309	387	WS00918_D23
4	0.000	71	4	-0.199	0.054	3.874	388	WS00918_E15
6	95.249	126	1	0.324	0.116	3.874	388	WS00918_E15
9	0.000	163	2	-0.452	0.052	4.319	388	WS00918_E15
10	70.246	190	2	0.218	0.043	5.131	388	WS00918_E15
10	70.246	190	4	-0.266	0.067	4.947	388	WS00918_E15
12	6.242	234	2	0.181	0.032	4.567	388	WS00918_E15
13	1.815	244	2	-0.226	0.033	4.122	388	WS00918_E15
1	45.794	9	1	0.236	0.065	5.499	389	WS00918_H13
1	50.955	10	1	-0.189	0.059	4.911	389	WS00918_H13
1	77.177	15	2	0.258	0.057	6.018	389	WS00918_H13
1	95.572	17	2	0.270	0.047	4.898	389	WS00918_H13
3	83.563	56	3	-0.291	0.096	7.931	389	WS00918_H13
6	81.996	123	3	0.245	0.075	3.990	389	WS00918_H13
7	15.555	130	2	-0.285	0.042	4.476	389	WS00918_H13
10	93.347	196	1	0.484	0.105	5.635	389	WS00918_H13
11	0.000	199	2	0.306	0.050	5.304	389	WS00918_H13
6	87.891	124	2	0.226	0.043	4.770	390	WS00918_I20
6	89.401	125	2	0.243	0.043	5.135	390	WS00918_I20
6	106.702	128	2	-0.520	0.084	7.214	390	WS00918_I20
7	59.789	137	3	0.143	0.031	4.084	390	WS00918_I20
9	59.963	177	1	-0.246	0.052	3.985	390	WS00918_I20
3	9.953	42	3	0.251	0.068	6.339	391	WS00914_F24
3	17.158	45	1	0.237	0.045	4.403	391	WS00914_F24

6	95.249	126	1	0.385	0.112	6.962	391	WS00914_F24
7	75.883	144	2	0.423	0.080	4.906	391	WS00914_F24
9	49.013	173	2	0.222	0.052	4.354	391	WS00914_F24
1	45.794	9	3	-0.243	0.053	5.741	392	WS00914_G13
3	95.157	59	2	-0.234	0.058	6.959	392	WS00914_G13
4	75.958	77	3	-0.351	0.106	6.447	392	WS00914_G13
6	45.897	117	4	0.246	0.050	4.763	392	WS00914_G13
6	95.249	126	1	0.353	0.100	3.930	392	WS00914_G13
11	17.088	203	1	-0.282	0.086	6.219	392	WS00914_G13
11	27.405	204	1	0.379	0.084	6.318	392	WS00914_G13
11	29.069	205	1	0.377	0.090	6.859	392	WS00914_G13
11	45.989	216	1	-0.238	0.077	5.563	392	WS00914_G13
12	22.441	227	3	-0.326	0.110	11.230	392	WS00914_G13
14	22.402	248	4	0.324	0.042	4.108	392	WS00914_G13
14	46.637	252	4	0.354	0.060	5.128	392	WS00914_G13
2	127.147	40	3	-0.161	0.047	4.876	393	WS00914_I23
3	9.953	42	3	0.243	0.058	6.048	393	WS00914_I23
6	95.249	126	1	0.451	0.126	9.864	393	WS00914_I23
8	39.090	152	4	0.160	0.052	4.314	393	WS00914_I23
9	36.227	165	3	-0.298	0.048	5.144	393	WS00914_I23
11	27.405	204	1	0.386	0.096	9.733	393	WS00914_I23
11	27.405	204	4	-0.300	0.058	4.930	393	WS00914_I23
11	29.069	205	1	0.310	0.071	7.034	393	WS00914_I23
11	45.989	216	1	-0.263	0.077	7.539	393	WS00914_I23
12	28.493	228	2	-0.215	0.067	4.239	393	WS00914_I23
14	22.402	248	3	0.218	0.049	5.091	393	WS00914_I23
1	22.419	4	4	0.331	0.042	4.091	394	WS00914_J14
1	131.686	23	1	0.418	0.055	4.194	394	WS00914_J14
3	9.953	42	4	0.203	0.068	7.011	394	WS00914_J14
4	55.925	76	1	-0.254	0.048	5.144	394	WS00914_J14
7	59.789	137	3	-0.258	0.062	4.425	394	WS00914_J14
10	95.795	197	4	-0.174	0.046	5.351	394	WS00914_J14
12	6.242	234	4	0.482	0.096	6.331	394	WS00914_J14
14	22.402	248	4	-0.345	0.041	5.578	394	WS00914_J14
14	46.637	252	4	-0.537	0.122	15.254	394	WS00914_J14
1	62.128	11	1	-0.482	0.090	4.594	395	WS00914_L22
1	123.316	20	4	0.205	0.069	4.753	395	WS00914_L22
4	82.462	78	1	-0.123	0.048	4.238	395	WS00914_L22
4	116.928	82	4	0.349	0.089	7.295	395	WS00914_L22
4	142.917	85	1	0.213	0.049	4.248	395	WS00914_L22
6	45.897	117	4	-0.226	0.030	4.425	395	WS00914_L22
6	106.702	128	2	-0.472	0.184	6.546	395	WS00914_L22
8	0.000	148	4	-0.261	0.033	4.155	395	WS00914_L22
11	46.026	217	1	0.254	0.082	7.131	395	WS00914_L22
2	102.814	36	1	0.264	0.052	4.299	396	WS00914_M10
4	55.925	76	1	0.216	0.059	4.728	396	WS00914_M10
6	26.350	115	2	0.229	0.045	4.779	396	WS00914_M10
7	15.555	130	3	0.250	0.056	5.801	396	WS00914_M10
7	91.487	146	3	0.227	0.044	4.511	396	WS00914_M10
9	61.113	178	1	-0.413	0.172	4.102	396	WS00914_M10
10	78.479	193	3	-0.322	0.046	4.768	396	WS00914_M10
10	86.151	195	3	0.269	0.075	7.724	396	WS00914_M10
11	17.088	203	1	0.275	0.094	7.701	396	WS00914_M10
11	29.069	205	1	-0.251	0.062	5.104	396	WS00914_M10

14	22.402	248	3	-0.255	0.045	4.620	396	WS00914_M10
3	15.147	44	2	-0.357	0.161	10.236	397	WS00914_O20
3	20.512	46	2	-0.242	0.036	4.145	397	WS00914_O20
3	114.747	62	2	0.250	0.039	4.543	397	WS00914_O20
8	50.001	154	1	0.278	0.075	6.346	397	WS00914_O20
9	59.963	177	4	-0.320	0.105	10.328	397	WS00914_O20
10	35.204	189	2	-0.258	0.061	6.173	397	WS00914_O20
10	70.246	190	2	0.217	0.060	6.734	397	WS00914_O20
12	51.382	242	1	-0.560	0.326	11.811	397	WS00914_O20
2	102.814	36	1	0.211	0.051	5.059	398	WS00914_P22
2	102.814	36	2	-0.232	0.065	6.027	398	WS00914_P22
2	126.181	39	2	0.283	0.051	4.828	398	WS00914_P22
3	9.953	42	3	-0.269	0.061	4.848	398	WS00914_P22
3	15.147	44	2	-0.279	0.081	5.487	398	WS00914_P22
5	57.017	102	4	0.268	0.054	4.607	398	WS00914_P22
9	36.227	165	4	0.274	0.037	4.371	398	WS00914_P22
9	17.951	169	2	0.240	0.046	3.980	398	WS00914_P22
9	59.963	177	4	-0.299	0.076	8.442	398	WS00914_P22
11	27.405	204	1	0.409	0.099	9.320	398	WS00914_P22
11	29.069	205	1	0.377	0.126	11.869	398	WS00914_P22
11	29.069	205	2	-0.342	0.105	9.863	398	WS00914_P22
11	41.984	212	2	-0.316	0.104	9.691	398	WS00914_P22
11	45.989	216	1	-0.211	0.058	5.430	398	WS00914_P22
11	45.989	216	2	-0.269	0.068	6.336	398	WS00914_P22
11	60.464	221	2	-0.304	0.070	5.501	398	WS00914_P22
12	51.382	242	1	-0.327	0.111	5.164	398	WS00914_P22
1	38.326	6	4	-0.246	0.055	3.877	399	WS00919_K09
1	40.464	7	4	0.266	0.057	4.176	399	WS00919_K09
3	47.928	49	1	0.258	0.052	5.300	399	WS00919_K09
5	7.672	92	4	0.198	0.053	3.926	399	WS00919_K09
5	42.979	97	2	0.257	0.047	3.867	399	WS00919_K09
6	25.914	114	1	-0.261	0.048	4.871	399	WS00919_K09
8	87.463	158	2	0.192	0.049	4.275	399	WS00919_K09
1	131.686	23	1	0.613	0.135	6.746	400	WS00919_L04
2	126.181	39	4	-0.390	0.109	4.660	400	WS00919_L04
2	127.147	40	3	-0.225	0.042	4.182	400	WS00919_L04
3	47.928	49	1	0.236	0.065	5.146	400	WS00919_L04
3	95.157	59	2	0.250	0.067	7.117	400	WS00919_L04
5	57.017	102	2	-0.287	0.048	5.442	400	WS00919_L04
5	57.017	102	4	0.270	0.053	4.927	400	WS00919_L04
6	45.897	117	1	0.247	0.061	5.202	400	WS00919_L04
7	59.789	137	3	-0.259	0.054	5.249	400	WS00919_L04
9	0.000	166	1	0.257	0.072	5.723	400	WS00919_L04
10	70.246	190	2	0.186	0.050	5.491	400	WS00919_L04
11	40.960	210	2	0.418	0.110	4.663	400	WS00919_L04
11	56.921	219	1	0.317	0.089	4.450	400	WS00919_L04
14	46.637	252	4	-0.316	0.069	6.122	400	WS00919_L04
3	95.157	59	2	0.165	0.040	4.070	401	WS00919_M23
6	69.073	121	3	-0.198	0.039	3.905	401	WS00919_M23
6	89.401	125	1	-0.238	0.053	4.146	401	WS00919_M23
6	95.249	126	1	0.306	0.078	3.967	401	WS00919_M23
8	11.974	150	1	-0.272	0.056	4.492	401	WS00919_M23
11	17.088	203	2	-0.201	0.046	4.051	401	WS00919_M23
11	27.405	204	1	0.343	0.067	5.355	401	WS00919_M23

11	29.069	205	1	0.266	0.069	5.401	401	WS00919_M23
11	29.069	205	2	-0.237	0.051	5.571	401	WS00919_M23
11	43.491	214	3	0.202	0.047	4.637	401	WS00919_M23
11	64.014	222	3	-0.305	0.093	9.232	401	WS00919_M23
1	95.572	17	3	0.257	0.048	4.438	402	WS00919_N18
1	127.805	21	4	0.281	0.065	5.403	402	WS00919_N18
4	92.039	79	2	-0.281	0.088	7.508	402	WS00919_N18
4	97.576	80	2	0.244	0.083	6.797	402	WS00919_N18
4	116.928	82	2	0.248	0.064	5.417	402	WS00919_N18
9	36.227	165	4	0.252	0.064	6.465	402	WS00919_N18
9	0.000	166	1	0.153	0.044	4.677	402	WS00919_N18
10	70.246	190	4	-0.218	0.052	4.880	402	WS00919_N18
14	22.402	248	4	-0.356	0.058	6.061	402	WS00919_N18
14	46.637	252	4	-0.287	0.043	4.585	402	WS00919_N18
1	2.788	2	4	-0.186	0.051	4.153	403	WS00920_B03
1	131.686	23	2	0.485	0.069	5.453	403	WS00920_B03
2	126.181	39	4	-0.411	0.058	4.961	403	WS00920_B03
5	57.017	102	2	-0.214	0.028	4.172	403	WS00920_B03
7	15.555	130	2	-0.204	0.026	3.858	403	WS00920_B03
7	17.610	131	3	-0.293	0.074	4.572	403	WS00920_B03
11	9.237	202	4	-0.308	0.094	8.838	403	WS00920_B03
11	27.405	204	4	0.302	0.083	7.980	403	WS00920_B03
3	20.512	46	2	-0.208	0.045	4.742	404	WS00920_B17
3	47.928	49	1	0.250	0.052	4.297	404	WS00920_B17
3	95.157	59	2	0.184	0.048	5.060	404	WS00920_B17
6	95.249	126	1	0.496	0.203	10.434	404	WS00920_B17
11	27.405	204	1	0.320	0.049	4.155	404	WS00920_B17
11	64.014	222	3	-0.220	0.063	6.031	404	WS00920_B17
14	22.402	248	4	-0.385	0.096	8.181	404	WS00920_B17
6	25.914	114	1	0.188	0.053	3.916	405	WS00920_D22
9	61.113	178	1	-0.437	0.133	5.338	405	WS00920_D22
11	9.237	202	4	-0.235	0.046	4.570	405	WS00920_D22
13	0.000	243	4	0.261	0.102	4.019	405	WS00920_D22
13	1.815	244	2	-0.205	0.044	5.070	405	WS00920_D22
2	126.181	39	4	-0.472	0.087	8.516	406	WS00920_E10
3	15.147	44	2	-0.227	0.043	4.064	406	WS00920_E10
3	95.157	59	2	0.174	0.032	4.201	406	WS00920_E10
3	104.681	60	2	-0.224	0.032	4.390	406	WS00920_E10
3	112.868	61	2	0.157	0.029	3.971	406	WS00920_E10
6	56.188	120	2	0.306	0.046	6.527	406	WS00920_E10
7	59.789	137	3	-0.204	0.060	4.445	406	WS00920_E10
7	70.278	141	3	0.214	0.070	5.142	406	WS00920_E10
7	75.883	144	2	0.386	0.039	3.951	406	WS00920_E10
8	39.090	152	4	0.266	0.055	6.242	406	WS00920_E10
9	17.951	169	2	0.250	0.035	4.382	406	WS00920_E10
11	27.405	204	1	0.506	0.253	13.344	406	WS00920_E10
11	27.405	204	4	-0.218	0.041	4.716	406	WS00920_E10
11	29.069	205	1	0.251	0.100	5.181	406	WS00920_E10
11	35.604	207	3	-0.305	0.127	9.351	406	WS00920_E10
11	37.143	209	3	0.266	0.096	7.119	406	WS00920_E10
11	42.600	213	3	-0.231	0.067	4.974	406	WS00920_E10
1	50.955	10	1	-0.149	0.056	3.909	407	WS0054_P20
3	104.681	60	2	-0.231	0.034	4.023	407	WS0054_P20
3	112.868	61	2	0.235	0.035	4.177	407	WS0054_P20

4	142.917	85	1	0.261	0.059	4.144	407	WS0054_P20
6	49.100	118	4	-0.382	0.130	4.412	407	WS0054_P20
8	50.001	154	2	0.188	0.057	6.615	407	WS0054_P20
9	59.963	177	4	-0.261	0.040	4.353	407	WS0054_P20
2	120.617	38	3	0.207	0.048	4.770	408	WS0261_A14
2	127.147	40	3	-0.194	0.056	5.503	408	WS0261_A14
3	47.928	49	1	0.223	0.061	5.313	408	WS0261_A14
4	124.132	83	1	0.478	0.133	4.565	408	WS0261_A14
5	31.191	95	1	-0.239	0.071	6.210	408	WS0261_A14
9	0.000	166	2	0.214	0.046	5.050	408	WS0261_A14
12	22.441	227	1	-0.260	0.068	5.971	408	WS0261_A14
1	29.800	5	1	0.265	0.054	5.372	409	WS0261_C08
1	127.805	21	4	-0.230	0.066	5.849	409	WS0261_C08
1	131.686	23	1	-0.593	0.106	6.036	409	WS0261_C08
3	83.563	56	3	-0.241	0.062	6.271	409	WS0261_C08
4	142.917	85	3	0.276	0.052	4.879	409	WS0261_C08
6	56.188	120	2	0.253	0.054	4.552	409	WS0261_C08
6	87.891	124	2	0.214	0.044	3.936	409	WS0261_C08
7	75.883	144	2	-0.413	0.133	4.322	409	WS0261_C08
8	87.463	158	1	0.259	0.050	4.970	409	WS0261_C08
9	36.227	165	3	0.209	0.051	5.173	409	WS0261_C08
9	3.127	168	1	-0.352	0.097	4.749	409	WS0261_C08
10	104.214	198	4	-0.365	0.095	8.517	409	WS0261_C08
1	82.301	16	3	-0.349	0.075	8.130	410	WS0261_C24
3	9.953	42	3	0.190	0.043	4.624	410	WS0261_C24
4	75.958	77	3	-0.256	0.069	5.320	410	WS0261_C24
4	82.462	78	1	-0.275	0.085	5.391	410	WS0261_C24
5	56.008	101	2	0.178	0.038	4.709	410	WS0261_C24
6	106.702	128	2	-0.410	0.059	4.660	410	WS0261_C24
7	17.610	131	3	-0.339	0.081	6.301	410	WS0261_C24
8	50.001	154	1	0.220	0.061	4.134	410	WS0261_C24
8	87.463	158	2	-0.226	0.040	4.255	410	WS0261_C24
9	49.013	173	2	0.253	0.049	6.182	410	WS0261_C24
11	42.600	213	3	-0.220	0.042	4.533	410	WS0261_C24
1	2.788	2	4	-0.275	0.077	6.373	411	WS0261_E02
1	123.316	20	4	0.294	0.057	3.859	411	WS0261_E02
2	127.147	40	3	-0.169	0.046	4.517	411	WS0261_E02
4	75.958	77	3	-0.364	0.132	6.554	411	WS0261_E02
6	95.249	126	1	0.310	0.080	3.984	411	WS0261_E02
10	80.088	194	2	-0.225	0.038	4.199	411	WS0261_E02
11	27.405	204	1	0.452	0.151	10.827	411	WS0261_E02
11	29.069	205	1	0.333	0.107	7.578	411	WS0261_E02
1	29.800	5	1	0.205	0.046	3.950	412	WS0261_E10
2	127.147	40	3	-0.227	0.058	6.719	412	WS0261_E10
3	47.928	49	1	0.318	0.068	5.939	412	WS0261_E10
5	52.820	99	3	0.248	0.034	3.847	412	WS0261_E10
11	27.405	204	1	0.314	0.049	4.317	412	WS0261_E10
11	56.921	219	2	0.303	0.108	6.474	412	WS0261_E10
1	50.955	10	1	-0.222	0.039	3.897	413	WS0261_F21
2	127.147	40	3	-0.173	0.054	4.648	413	WS0261_F21
3	47.928	49	1	0.218	0.048	4.721	413	WS0261_F21
4	75.958	77	3	-0.384	0.159	5.874	413	WS0261_F21
4	124.132	83	1	0.451	0.082	4.108	413	WS0261_F21
4	142.917	85	1	-0.263	0.044	4.388	413	WS0261_F21

6	95.249	126	1	0.329	0.113	5.529	413	WS0261_F21
10	86.151	195	3	0.225	0.080	6.842	413	WS0261_F21
1	82.301	16	3	-0.268	0.048	4.793	414	WS0261_G14
3	47.928	49	1	0.231	0.069	4.617	414	WS0261_G14
6	56.188	120	2	0.259	0.037	4.552	414	WS0261_G14
8	50.001	154	1	0.215	0.059	3.869	414	WS0261_G14
14	0.000	246	1	0.233	0.065	4.308	414	WS0261_G14
2	63.261	30	3	0.288	0.051	4.791	415	WS0101_G20
3	83.563	56	3	-0.145	0.043	3.843	415	WS0101_G20
5	31.191	95	1	-0.175	0.039	4.441	415	WS0101_G20
6	95.249	126	1	0.372	0.099	6.791	415	WS0101_G20
11	27.405	204	1	0.375	0.082	9.642	415	WS0101_G20
11	29.069	205	1	0.331	0.063	7.403	415	WS0101_G20
11	45.989	216	1	-0.323	0.075	8.670	415	WS0101_G20
14	0.000	246	1	0.183	0.039	4.479	415	WS0101_G20
9	36.227	165	3	-0.288	0.063	6.745	416	WS0101_M03
11	27.405	204	1	0.275	0.057	6.325	416	WS0101_M03
11	64.014	222	3	-0.238	0.043	4.587	416	WS0101_M03
1	95.572	17	3	0.261	0.051	4.229	417	WS0079_M02
2	127.147	40	3	-0.169	0.040	4.333	417	WS0079_M02
6	49.100	118	2	0.318	0.082	4.090	417	WS0079_M02
6	56.188	120	2	0.242	0.060	5.498	417	WS0079_M02
7	17.610	131	3	-0.300	0.107	5.252	417	WS0079_M02
7	59.789	137	3	-0.636	0.446	48.561	417	WS0079_M02
7	62.787	139	4	-0.612	0.319	25.589	417	WS0079_M02
7	67.154	140	4	-0.548	0.352	12.992	417	WS0079_M02
7	70.278	141	3	0.546	0.351	38.143	417	WS0079_M02
7	73.169	142	2	0.518	0.152	4.014	417	WS0079_M02
7	74.560	143	1	-0.323	0.102	9.856	417	WS0079_M02
7	75.883	144	4	-0.609	0.254	20.470	417	WS0079_M02
7	91.487	146	3	0.396	0.151	16.395	417	WS0079_M02
9	32.356	171	2	-0.189	0.048	4.360	417	WS0079_M02
11	56.921	219	1	0.417	0.130	4.859	417	WS0079_M02
11	64.014	222	3	-0.205	0.046	5.007	417	WS0079_M02
12	14.406	230	3	-0.360	0.076	8.292	417	WS0079_M02
12	6.242	234	4	0.193	0.054	4.210	417	WS0079_M02
1	123.316	20	4	0.298	0.076	4.265	418	WS00938_M17
3	95.157	59	2	0.197	0.050	5.114	418	WS00938_M17
7	74.560	143	1	-0.238	0.055	4.448	418	WS00938_M17
8	11.974	150	1	-0.242	0.049	4.024	418	WS00938_M17
9	59.963	177	4	-0.259	0.055	4.827	418	WS00938_M17
10	78.479	193	3	-0.331	0.064	6.396	418	WS00938_M17
11	43.491	214	3	0.202	0.053	5.232	418	WS00938_M17
11	64.014	222	3	-0.273	0.071	7.126	418	WS00938_M17
1	38.326	6	2	0.190	0.036	3.878	419	WS0092_C11
2	120.617	38	3	0.223	0.048	4.949	419	WS0092_C11
3	47.928	49	1	0.222	0.052	4.170	419	WS0092_C11
3	95.157	59	2	0.212	0.053	5.218	419	WS0092_C11
6	95.249	126	1	0.350	0.103	5.146	419	WS0092_C11
8	50.001	154	1	0.235	0.056	4.854	419	WS0092_C11
9	59.963	177	4	-0.263	0.054	4.404	419	WS0092_C11
10	9.580	187	3	-0.371	0.103	4.009	419	WS0092_C11
10	78.479	193	3	-0.394	0.064	6.673	419	WS0092_C11
11	64.014	222	3	-0.187	0.038	3.898	419	WS0092_C11

6	89.401	125	1	-0.295	0.080	6.089	420	WS00939_F21
6	95.249	126	1	0.438	0.147	7.083	420	WS00939_F21
7	74.560	143	1	-0.176	0.056	4.144	420	WS00939_F21
8	11.974	150	1	-0.273	0.050	3.869	420	WS00939_F21
10	78.479	193	3	-0.328	0.043	4.395	420	WS00939_F21
11	17.088	203	2	-0.197	0.049	4.643	420	WS00939_F21
1	29.800	5	2	-0.293	0.050	4.297	421	WS0261_I01
1	38.326	6	2	0.275	0.058	6.330	421	WS0261_I01
3	47.928	49	1	0.294	0.085	6.819	421	WS0261_I01
4	75.958	77	3	-0.271	0.068	4.184	421	WS0261_I01
6	89.401	125	1	-0.286	0.058	4.699	421	WS0261_I01
6	95.249	126	1	0.315	0.102	4.211	421	WS0261_I01
8	11.974	150	1	-0.275	0.066	5.296	421	WS0261_I01
8	50.001	154	1	0.246	0.054	4.911	421	WS0261_I01
9	59.963	177	4	-0.233	0.050	4.609	421	WS0261_I01
10	78.479	193	3	-0.336	0.064	6.530	421	WS0261_I01
11	64.014	222	3	-0.243	0.062	6.332	421	WS0261_I01
1	107.333	19	3	0.238	0.045	4.858	422	WS0261_I17
3	47.928	49	1	0.279	0.068	6.233	422	WS0261_I17
3	154.505	69	4	-0.187	0.045	3.967	422	WS0261_I17
6	89.401	125	1	-0.328	0.060	5.519	422	WS0261_I17
8	11.974	150	1	-0.236	0.044	4.005	422	WS0261_I17
9	59.963	177	4	-0.323	0.089	7.420	422	WS0261_I17
11	17.088	203	1	-0.237	0.048	4.403	422	WS0261_I17
11	27.405	204	1	0.419	0.091	8.432	422	WS0261_I17
11	29.069	205	1	0.382	0.104	9.545	422	WS0261_I17
11	45.989	216	1	-0.211	0.052	4.699	422	WS0261_I17
1	38.326	6	2	0.195	0.036	4.488	423	WS0261_J24
2	120.617	38	3	0.198	0.052	4.318	423	WS0261_J24
2	127.147	40	3	-0.148	0.051	4.183	423	WS0261_J24
3	104.681	60	2	-0.213	0.042	5.203	423	WS0261_J24
3	112.868	61	2	0.262	0.048	5.997	423	WS0261_J24
3	114.747	62	2	0.153	0.035	4.349	423	WS0261_J24
6	95.249	126	1	0.378	0.158	5.811	423	WS0261_J24
8	11.974	150	1	-0.248	0.073	4.660	423	WS0261_J24
9	49.013	173	2	0.254	0.050	5.909	423	WS0261_J24
9	59.963	177	4	-0.360	0.087	9.076	423	WS0261_J24
10	78.479	193	3	-0.331	0.055	4.538	423	WS0261_J24
11	17.088	203	2	-0.223	0.041	4.087	423	WS0261_J24
11	64.014	222	3	-0.211	0.056	4.652	423	WS0261_J24
2	120.617	38	3	0.220	0.048	4.886	424	WS0261_K11
2	127.147	40	3	-0.261	0.072	7.291	424	WS0261_K11
3	95.157	59	2	0.165	0.038	4.958	424	WS0261_K11
9	49.013	173	2	0.219	0.051	6.536	424	WS0261_K11
10	9.580	187	2	-0.360	0.112	7.586	424	WS0261_K11
11	0.000	199	3	-0.317	0.049	4.993	424	WS0261_K11
11	9.237	202	4	-0.247	0.061	5.284	424	WS0261_K11
1	38.326	6	4	-0.252	0.040	4.405	425	WS0261_M02
1	62.128	11	1	-0.532	0.098	5.458	425	WS0261_M02
3	80.211	55	4	-0.186	0.043	4.728	425	WS0261_M02
4	6.032	72	4	0.262	0.073	8.103	425	WS0261_M02
5	57.017	102	2	-0.254	0.057	6.170	425	WS0261_M02
5	57.017	102	4	0.409	0.086	9.473	425	WS0261_M02
6	56.188	120	2	0.256	0.056	5.538	425	WS0261_M02

8	50.001	154	2	-0.206	0.057	5.619	425	WS0261_M02
8	87.463	158	2	0.276	0.069	6.787	425	WS0261_M02
9	17.951	169	1	-0.361	0.088	7.764	425	WS0261_M02
9	17.951	169	2	0.390	0.085	9.803	425	WS0261_M02
10	77.676	192	1	0.212	0.046	4.121	425	WS0261_M02
10	80.088	194	1	0.221	0.053	4.741	425	WS0261_M02
10	104.214	198	4	0.242	0.031	4.621	425	WS0261_M02
11	48.979	218	4	0.255	0.051	5.865	425	WS0261_M02
12	38.543	232	4	-0.287	0.081	4.416	425	WS0261_M02
12	0.000	233	1	0.209	0.045	3.986	425	WS0261_M02
14	22.402	248	4	-0.431	0.110	11.519	425	WS0261_M02
1	29.800	5	1	0.213	0.052	4.544	426	WS0261_M19
3	47.928	49	1	0.264	0.049	4.300	426	WS0261_M19
4	75.958	77	3	-0.438	0.179	8.827	426	WS0261_M19
5	31.191	95	1	-0.319	0.078	6.896	426	WS0261_M19
6	95.249	126	1	0.456	0.113	7.404	426	WS0261_M19
9	28.608	170	2	-0.330	0.051	5.331	426	WS0261_M19
11	27.405	204	1	0.448	0.119	10.769	426	WS0261_M19
11	29.069	205	1	0.384	0.094	8.320	426	WS0261_M19
1	29.800	5	1	0.217	0.039	3.959	427	WS0261_O10
1	123.316	20	4	0.295	0.084	4.436	427	WS0261_O10
6	95.249	126	1	0.378	0.120	5.421	427	WS0261_O10
9	59.963	177	4	-0.459	0.161	18.657	427	WS0261_O10
3	15.147	44	2	-0.347	0.079	3.990	428	WS0261_O18
4	0.000	71	4	0.277	0.052	5.146	428	WS0261_O18
7	15.555	130	3	-0.183	0.057	5.159	428	WS0261_O18
7	17.610	131	3	-0.301	0.080	4.066	428	WS0261_O18
7	62.787	139	2	-0.220	0.045	5.053	428	WS0261_O18
7	74.560	143	2	0.283	0.057	6.766	428	WS0261_O18
9	59.963	177	4	-0.282	0.057	5.923	428	WS0261_O18
11	27.405	204	1	0.276	0.049	3.898	428	WS0261_O18
12	0.000	226	3	0.165	0.051	4.644	428	WS0261_O18
14	43.392	250	2	0.171	0.043	4.450	428	WS0261_O18
6	95.249	126	1	0.385	0.102	6.816	429	WS00920_H02
8	11.974	150	1	-0.263	0.065	6.209	429	WS00920_H02
11	27.405	204	1	0.362	0.065	6.209	429	WS00920_H02
11	29.069	205	1	0.334	0.077	7.394	429	WS00920_H02
11	43.491	214	3	0.184	0.042	4.616	429	WS00920_H02
11	60.464	221	1	0.213	0.055	5.226	429	WS00920_H02
11	64.014	222	3	-0.274	0.072	7.962	429	WS00920_H02
1	82.301	16	3	-0.258	0.041	5.151	430	WS00920_H21
3	95.157	59	2	0.249	0.065	6.349	430	WS00920_H21
7	17.610	131	3	-0.261	0.060	4.370	430	WS00920_H21
1	50.955	10	1	-0.235	0.068	5.804	431	WS00920_J18
3	80.211	55	4	0.310	0.065	6.045	431	WS00920_J18
7	75.883	144	2	-0.383	0.068	4.268	431	WS00920_J18
9	17.951	169	2	0.250	0.043	4.539	431	WS00920_J18
11	17.088	203	1	0.281	0.093	7.945	431	WS00920_J18
11	17.088	203	2	-0.175	0.038	3.922	431	WS00920_J18
12	6.242	234	4	0.319	0.055	4.543	431	WS00920_J18
3	47.928	49	1	0.235	0.042	4.061	432	WS00920_K10
5	31.191	95	1	-0.243	0.044	4.224	432	WS00920_K10
6	95.249	126	1	0.502	0.152	10.996	432	WS00920_K10
11	27.405	204	1	0.398	0.080	7.877	432	WS00920_K10

11	29.069	205	1	0.275	0.047	4.542	432	WS00920_K10
11	64.014	222	3	-0.216	0.059	6.553	432	WS00920_K10
12	28.493	228	2	-0.281	0.119	6.192	432	WS00920_K10
1	29.800	5	1	0.276	0.047	3.869	433	WS00920_M05
1	107.333	19	3	-0.217	0.046	4.241	433	WS00920_M05
8	50.001	154	1	-0.226	0.068	5.606	433	WS00920_M05
12	22.629	237	1	-0.261	0.059	4.807	433	WS00920_M05
12	32.473	238	1	-0.255	0.056	4.543	433	WS00920_M05
14	22.402	248	4	-0.557	0.123	8.416	433	WS00920_M05
8	11.974	150	1	-0.221	0.044	4.456	434	WS00920_M19
8	87.463	158	2	-0.215	0.055	4.027	434	WS00920_M19
9	59.963	177	4	0.222	0.045	4.205	434	WS00920_M19
12	28.493	228	2	-0.264	0.065	4.095	434	WS00920_M19
6	95.249	126	1	0.322	0.066	4.664	435	WS00920_O20
8	39.090	152	4	0.175	0.043	4.074	435	WS00920_O20
11	27.405	204	1	0.394	0.086	8.594	435	WS00920_O20
11	27.405	204	4	-0.291	0.065	6.419	435	WS00920_O20
11	29.069	205	1	0.260	0.045	4.518	435	WS00920_O20
11	45.989	216	1	-0.215	0.053	5.205	435	WS00920_O20
3	14.500	43	1	-0.337	0.089	8.090	436	WS00920_P08
3	17.158	45	1	0.211	0.046	4.176	436	WS00920_P08
3	80.211	55	4	0.259	0.062	5.206	436	WS00920_P08
3	137.891	64	3	0.314	0.060	5.335	436	WS00920_P08
5	31.191	95	1	-0.225	0.057	5.194	436	WS00920_P08
9	28.608	170	3	-0.302	0.071	7.307	436	WS00920_P08
9	58.045	176	1	0.319	0.068	5.017	436	WS00920_P08
9	69.046	181	1	-0.268	0.066	4.853	436	WS00920_P08
1	95.572	17	2	0.220	0.052	4.580	437	WS02612_H07
3	15.147	44	2	0.409	0.111	8.417	437	WS02612_H07
3	20.512	46	2	0.226	0.089	7.907	437	WS02612_H07
3	148.745	68	4	0.264	0.035	4.337	437	WS02612_H07
4	142.917	85	1	0.277	0.055	5.334	437	WS02612_H07
5	86.669	106	4	0.424	0.091	4.641	437	WS02612_H07
6	49.100	118	4	-0.493	0.140	7.131	437	WS02612_H07
8	87.463	158	2	-0.270	0.073	6.043	437	WS02612_H07
9	59.963	177	4	0.341	0.060	6.716	437	WS02612_H07
11	27.405	204	1	0.296	0.088	8.634	437	WS02612_H07
11	29.069	205	1	0.155	0.045	4.396	437	WS02612_H07
11	40.960	210	2	0.448	0.080	5.975	437	WS02612_H07
11	40.960	210	4	-0.301	0.036	4.450	437	WS02612_H07
11	56.921	219	1	-0.392	0.083	4.380	437	WS02612_H07
12	14.406	230	4	0.253	0.061	7.571	437	WS02612_H07
1	29.800	5	2	-0.199	0.039	4.583	438	WS02613_H16
5	57.017	102	4	0.224	0.041	3.997	438	WS02613_H16
6	69.073	121	3	-0.311	0.096	9.287	438	WS02613_H16
7	17.610	131	3	0.242	0.083	5.087	438	WS02613_H16
12	6.242	234	4	0.223	0.042	6.137	438	WS02613_H16
1	127.805	21	4	-0.225	0.051	5.183	439	WS0091_C05
1	131.686	23	2	-0.488	0.084	5.224	439	WS0091_C05
3	95.157	59	2	-0.221	0.054	5.573	439	WS0091_C05
3	141.443	65	3	0.329	0.098	4.663	439	WS0091_C05
9	32.356	171	2	0.180	0.038	4.083	439	WS0091_C05
1	40.464	7	4	0.197	0.032	4.396	440	WS00928_B22
1	45.794	9	1	-0.366	0.083	7.810	440	WS00928_B22

3	47.928	49	1	0.268	0.090	8.254	440	WS00928_B22
3	104.681	60	2	-0.171	0.042	3.987	440	WS00928_B22
3	144.647	67	1	0.331	0.122	5.788	440	WS00928_B22
4	116.928	82	4	0.319	0.074	6.513	440	WS00928_B22
5	57.017	102	4	0.289	0.049	5.265	440	WS00928_B22
6	49.100	118	4	0.388	0.166	7.926	440	WS00928_B22
6	87.891	124	2	0.236	0.075	7.170	440	WS00928_B22
6	89.401	125	2	0.329	0.061	4.361	440	WS00928_B22
6	106.702	128	2	-0.380	0.111	4.129	440	WS00928_B22
7	105.477	147	2	0.199	0.081	4.919	440	WS00928_B22
8	0.000	148	4	-0.280	0.047	5.183	440	WS00928_B22
11	46.026	217	1	0.264	0.079	7.320	440	WS00928_B22
13	1.815	244	4	0.226	0.039	4.067	440	WS00928_B22
1	38.326	6	2	0.196	0.040	4.300	441	WS0091_H09
2	120.617	38	3	0.211	0.051	4.963	441	WS0091_H09
2	127.147	40	3	-0.169	0.049	4.781	441	WS0091_H09
3	47.928	49	1	0.278	0.091	7.529	441	WS0091_H09
3	95.157	59	2	0.213	0.047	4.781	441	WS0091_H09
4	0.000	71	4	-0.244	0.044	4.057	441	WS0091_H09
8	50.001	154	1	0.235	0.062	5.449	441	WS0091_H09
9	59.963	177	4	-0.233	0.046	4.025	441	WS0091_H09
10	78.479	193	3	-0.346	0.054	5.272	441	WS0091_H09
12	51.382	242	1	-0.425	0.134	5.045	441	WS0091_H09
1	62.128	11	2	0.359	0.103	5.040	442	WS00928_F16
1	95.572	17	3	0.269	0.054	6.674	442	WS00928_F16
1	107.333	19	3	0.298	0.074	8.662	442	WS00928_F16
2	120.617	38	3	0.368	0.093	10.972	442	WS00928_F16
2	126.181	39	2	-0.248	0.044	3.947	442	WS00928_F16
2	127.147	40	3	-0.197	0.044	5.185	442	WS00928_F16
3	47.928	49	1	0.189	0.054	5.366	442	WS00928_F16
4	140.962	84	3	0.170	0.034	3.970	442	WS00928_F16
4	142.917	85	3	0.209	0.042	5.221	442	WS00928_F16
6	81.996	123	3	-0.546	0.237	15.447	442	WS00928_F16
6	106.702	128	3	0.345	0.088	10.235	442	WS00928_F16
7	75.883	144	2	0.423	0.126	4.959	442	WS00928_F16
8	39.090	152	4	0.239	0.089	6.280	442	WS00928_F16
8	50.001	154	1	0.288	0.099	9.931	442	WS00928_F16
10	21.864	188	3	-0.180	0.031	3.870	442	WS00928_F16
10	70.246	190	2	0.284	0.061	5.502	442	WS00928_F16
10	70.246	190	4	-0.320	0.060	4.682	442	WS00928_F16
12	51.382	242	1	-0.391	0.107	3.844	442	WS00928_F16
1	38.326	6	2	0.211	0.066	4.137	443	WS0092_N14
2	93.504	32	4	0.342	0.077	7.638	443	WS0092_N14
2	120.617	38	4	0.346	0.077	6.812	443	WS0092_N14
4	75.958	77	3	-0.294	0.101	3.886	443	WS0092_N14
11	29.069	205	1	0.268	0.046	5.819	443	WS0092_N14
11	42.600	213	3	-0.166	0.048	4.198	443	WS0092_N14
13	3.386	245	1	0.321	0.057	7.245	443	WS0092_N14
1	95.572	17	3	0.236	0.035	3.946	444	WS0092_O01
2	120.617	38	3	0.214	0.035	4.233	444	WS0092_O01
3	47.928	49	1	0.302	0.097	8.474	444	WS0092_O01
4	75.958	77	3	-0.310	0.086	5.290	444	WS0092_O01
9	59.963	177	4	-0.248	0.074	4.871	444	WS0092_O01
10	70.246	190	2	0.237	0.040	3.988	444	WS0092_O01

1	38.326	6	4	-0.350	0.043	5.036	445	WS0093_A07
6	25.914	114	1	-0.276	0.075	8.238	445	WS0093_A07
6	76.376	122	1	-0.268	0.068	5.297	445	WS0093_A07
7	73.169	142	1	0.627	0.093	7.285	445	WS0093_A07
11	17.088	203	1	-0.275	0.079	8.635	445	WS0093_A07
12	6.242	234	4	0.163	0.031	3.875	445	WS0093_A07
12	51.382	242	1	-0.381	0.088	6.796	445	WS0093_A07
13	0.000	243	4	-0.237	0.062	4.802	445	WS0093_A07
2	102.814	36	1	0.294	0.053	4.488	446	WS0093_B06
6	45.897	117	1	0.292	0.056	4.918	446	WS0093_B06
6	106.702	128	3	0.271	0.049	4.156	446	WS0093_B06
9	17.951	169	2	0.272	0.050	5.335	446	WS0093_B06
9	59.963	177	4	-0.280	0.058	4.827	446	WS0093_B06
10	21.864	188	1	-0.284	0.048	4.247	446	WS0093_B06
10	35.204	189	1	-0.217	0.047	4.146	446	WS0093_B06
10	78.479	193	4	-0.275	0.092	7.137	446	WS0093_B06
10	80.088	194	1	-0.275	0.047	4.186	446	WS0093_B06
13	0.000	243	4	0.312	0.130	4.196	446	WS0093_B06
3	80.211	55	4	0.235	0.037	4.011	447	WS0093_C22
3	144.593	66	4	-0.250	0.046	5.427	447	WS0093_C22
4	103.719	81	4	-0.250	0.047	5.151	447	WS0093_C22
4	116.928	82	4	0.230	0.043	4.662	447	WS0093_C22
5	54.072	100	4	0.217	0.058	6.200	447	WS0093_C22
7	75.883	144	4	0.355	0.069	6.006	447	WS0093_C22
8	11.974	150	1	-0.295	0.071	7.730	447	WS0093_C22
10	70.246	190	4	-0.427	0.125	13.335	447	WS0093_C22
10	78.479	193	4	-0.331	0.111	11.205	447	WS0093_C22
12	14.406	230	4	-0.312	0.046	4.248	447	WS0093_C22
2	102.814	36	1	0.297	0.039	4.470	448	WS0093_D07
5	52.820	99	3	-0.255	0.042	4.627	448	WS0093_D07
6	45.897	117	1	0.321	0.045	4.697	448	WS0093_D07
7	15.555	130	2	0.222	0.049	4.192	448	WS0093_D07
10	70.246	190	4	-0.173	0.049	3.940	448	WS0093_D07
10	86.151	195	3	0.240	0.038	4.213	448	WS0093_D07
11	56.921	219	2	-0.275	0.080	4.558	448	WS0093_D07
1	2.788	2	4	-0.256	0.045	3.858	449	WS0093_F21
1	95.572	17	3	0.199	0.046	4.364	449	WS0093_F21
4	124.132	83	1	0.530	0.119	5.689	449	WS0093_F21
4	142.917	85	1	-0.207	0.036	4.070	449	WS0093_F21
6	69.073	121	3	-0.248	0.044	4.201	449	WS0093_F21
6	89.401	125	1	-0.441	0.089	10.181	449	WS0093_F21
8	11.974	150	1	-0.251	0.050	5.654	449	WS0093_F21
8	39.090	152	4	0.214	0.064	5.935	449	WS0093_F21
9	3.127	168	1	-0.368	0.070	4.139	449	WS0093_F21
9	59.963	177	4	-0.201	0.042	4.116	449	WS0093_F21
11	27.405	204	1	0.337	0.047	5.322	449	WS0093_F21
11	29.069	205	1	0.334	0.067	7.710	449	WS0093_F21
11	45.989	216	1	-0.262	0.054	6.139	449	WS0093_F21
12	14.406	230	4	-0.298	0.079	7.149	449	WS0093_F21
1	131.686	23	1	0.480	0.148	3.912	450	WS0093_G10
4	116.928	82	4	0.208	0.055	4.231	450	WS0093_G10
5	57.017	102	2	-0.332	0.062	7.748	450	WS0093_G10
9	0.000	163	2	-0.464	0.059	4.264	450	WS0093_G10
13	1.815	244	4	0.154	0.051	3.935	450	WS0093_G10

2	93.504	32	4	-0.302	0.057	4.874	451	WS0092_G11
4	116.928	82	2	0.268	0.049	5.295	451	WS0092_G11
6	49.100	118	4	0.401	0.188	6.760	451	WS0092_G11
6	56.188	120	2	0.186	0.032	4.987	451	WS0092_G11
6	69.073	121	3	0.239	0.049	4.724	451	WS0092_G11
6	87.891	124	2	0.156	0.046	5.063	451	WS0092_G11
6	89.401	125	2	0.319	0.047	5.230	451	WS0092_G11
8	50.001	154	2	-0.319	0.151	16.961	451	WS0092_G11
8	87.463	158	2	0.289	0.047	4.143	451	WS0092_G11
9	28.608	170	2	0.312	0.048	5.755	451	WS0092_G11
9	28.608	170	3	-0.323	0.060	6.311	451	WS0092_G11
11	40.960	210	2	0.427	0.086	4.885	451	WS0092_G11
1	123.316	20	4	0.291	0.087	6.316	452	WS0092_L06
1	127.805	21	4	-0.316	0.076	6.121	452	WS0092_L06
1	131.686	23	2	-0.446	0.066	4.823	452	WS0092_L06
5	3.484	90	2	0.207	0.053	3.841	452	WS0092_L06
5	7.672	92	4	-0.213	0.053	5.295	452	WS0092_L06
9	59.963	177	4	-0.272	0.082	8.055	452	WS0092_L06
11	9.237	202	4	0.232	0.053	4.993	452	WS0092_L06
11	27.405	204	4	-0.257	0.065	5.600	452	WS0092_L06
11	29.069	205	2	0.162	0.041	4.931	452	WS0092_L06
3	47.928	49	1	0.226	0.043	4.959	453	WS01013_F10
3	80.211	55	4	0.225	0.051	5.237	453	WS01013_F10
3	137.891	64	1	0.239	0.040	4.632	453	WS01013_F10
3	144.647	67	1	0.396	0.114	5.720	453	WS01013_F10
6	81.996	123	4	-0.298	0.091	4.642	453	WS01013_F10
6	95.249	126	1	0.337	0.082	5.429	453	WS01013_F10
8	11.974	150	1	-0.256	0.046	5.328	453	WS01013_F10
11	27.405	204	1	0.512	0.129	15.256	453	WS01013_F10
11	27.405	204	4	-0.239	0.048	5.056	453	WS01013_F10
11	29.069	205	1	0.319	0.065	7.646	453	WS01013_F10
11	45.989	216	1	-0.245	0.056	6.463	453	WS01013_F10
13	0.000	243	4	0.351	0.080	4.049	453	WS01013_F10
1	40.464	7	3	0.217	0.039	5.275	454	WS0269_B14
1	40.464	7	4	0.300	0.091	4.812	454	WS0269_B14
1	45.794	9	3	-0.208	0.034	4.471	454	WS0269_B14
4	116.928	82	2	-0.232	0.037	4.176	454	WS0269_B14
4	124.132	83	2	-0.275	0.063	4.111	454	WS0269_B14
5	56.008	101	1	-0.185	0.056	4.144	454	WS0269_B14
5	57.017	102	2	0.218	0.038	4.302	454	WS0269_B14
9	59.963	177	1	0.347	0.100	7.590	454	WS0269_B14
9	69.046	181	1	-0.316	0.068	4.083	454	WS0269_B14
14	0.000	246	2	0.216	0.053	6.034	454	WS0269_B14
14	44.898	251	1	0.263	0.074	5.508	454	WS0269_B14
1	22.419	4	3	0.383	0.072	5.300	455	WS01013_K21
1	95.572	17	2	0.253	0.042	4.000	455	WS01013_K21
3	9.953	42	3	0.167	0.042	4.082	455	WS01013_K21
6	95.249	126	1	0.453	0.116	8.503	455	WS01013_K21
7	15.555	130	2	-0.264	0.046	4.319	455	WS01013_K21
7	15.555	130	3	0.276	0.047	4.809	455	WS01013_K21
8	87.463	158	2	-0.243	0.056	5.128	455	WS01013_K21
11	27.405	204	1	0.430	0.091	9.001	455	WS01013_K21
11	27.405	204	4	-0.265	0.047	4.483	455	WS01013_K21
11	29.069	205	1	0.328	0.067	6.570	455	WS01013_K21

11	45.989	216	1	-0.177	0.046	4.393	455	WS01013_K21
2	126.181	39	4	0.293	0.059	4.053	456	WS0269_N15
6	87.891	124	2	-0.257	0.048	5.236	456	WS0269_N15
6	89.401	125	2	-0.278	0.049	5.358	456	WS0269_N15
7	15.555	130	2	-0.324	0.052	5.715	456	WS0269_N15
7	73.169	142	1	0.562	0.186	4.850	456	WS0269_N15
11	27.405	204	1	0.356	0.111	9.021	456	WS0269_N15
11	29.069	205	1	0.299	0.087	7.219	456	WS0269_N15
11	45.989	216	1	-0.311	0.085	6.873	456	WS0269_N15
11	60.464	221	1	0.277	0.085	6.798	456	WS0269_N15
1	95.572	17	3	0.200	0.038	3.921	457	WS00712_D21
1	107.333	19	3	0.215	0.042	4.403	457	WS00712_D21
3	17.158	45	1	0.249	0.046	4.723	457	WS00712_D21
5	7.672	92	4	0.259	0.057	4.001	457	WS00712_D21
12	6.242	234	4	-0.301	0.064	4.112	457	WS00712_D21
1	107.333	19	3	0.219	0.041	4.107	458	WS00712_K05
3	9.953	42	3	0.215	0.059	5.772	458	WS00712_K05
3	80.211	55	4	-0.296	0.070	6.318	458	WS00712_K05
5	57.017	102	4	-0.301	0.062	4.434	458	WS00712_K05
8	87.463	158	1	0.241	0.057	4.176	458	WS00712_K05
11	27.405	204	1	0.278	0.063	4.773	458	WS00712_K05
11	29.069	205	1	0.264	0.055	3.997	458	WS00712_K05
11	45.989	216	1	-0.236	0.089	6.404	458	WS00712_K05
14	0.000	246	1	0.290	0.072	5.210	458	WS00712_K05
1	45.794	9	3	-0.230	0.068	6.219	459	WS0094_N21
7	17.610	131	3	0.277	0.108	4.517	459	WS0094_N21
11	45.989	216	1	-0.229	0.040	4.132	459	WS0094_N21
14	22.402	248	4	0.339	0.038	4.648	459	WS0094_N21
1	15.589	3	2	-0.195	0.048	4.827	460	WS0057_F14
2	63.261	30	3	0.227	0.039	4.369	460	WS0057_F14
2	127.147	40	3	-0.178	0.037	4.111	460	WS0057_F14
4	0.000	71	4	0.235	0.063	4.886	460	WS0057_F14
5	31.191	95	1	-0.253	0.063	5.401	460	WS0057_F14
5	54.072	100	4	0.220	0.055	4.224	460	WS0057_F14
6	95.249	126	1	0.388	0.111	7.881	460	WS0057_F14
10	70.246	190	2	-0.279	0.052	5.318	460	WS0057_F14
11	27.405	204	1	0.263	0.054	4.753	460	WS0057_F14
11	27.405	204	4	-0.254	0.056	4.327	460	WS0057_F14
11	45.989	216	1	-0.233	0.068	5.854	460	WS0057_F14
1	22.419	4	4	-0.488	0.224	7.743	461	WS01017_B23
1	38.326	6	4	-0.299	0.068	4.997	461	WS01017_B23
1	40.464	7	4	0.255	0.050	3.965	461	WS01017_B23
8	87.463	158	2	0.194	0.051	4.499	461	WS01017_B23
11	17.088	203	1	-0.279	0.070	6.058	461	WS01017_B23
3	95.157	59	2	-0.213	0.028	3.913	462	WS0057_P23
4	75.958	77	3	-0.302	0.087	4.379	462	WS0057_P23
8	95.937	159	3	0.234	0.035	3.917	462	WS0057_P23
11	27.405	204	1	0.482	0.152	11.533	462	WS0057_P23
11	27.405	204	4	-0.332	0.074	6.260	462	WS0057_P23
11	29.069	205	1	0.289	0.069	5.039	462	WS0057_P23
11	41.984	212	2	-0.230	0.035	4.146	462	WS0057_P23
11	45.989	216	1	-0.146	0.055	4.021	462	WS0057_P23
3	116.958	63	4	-0.196	0.051	4.576	463	WS00922_B17
4	75.958	77	3	-0.501	0.185	9.177	463	WS00922_B17

4	116.928	82	2	-0.290	0.040	4.639	463	WS00922_B17
5	31.191	95	1	-0.269	0.082	6.508	463	WS00922_B17
5	57.017	102	2	0.235	0.034	4.107	463	WS00922_B17
6	95.249	126	1	0.330	0.076	3.905	463	WS00922_B17
11	0.000	199	3	-0.353	0.073	7.166	463	WS00922_B17
11	17.088	203	1	-0.232	0.055	4.321	463	WS00922_B17
11	29.069	205	1	0.302	0.055	4.717	463	WS00922_B17
11	45.989	216	1	-0.231	0.064	5.085	463	WS00922_B17
14	0.000	246	1	0.301	0.089	7.082	463	WS00922_B17
1	40.464	7	3	0.192	0.034	4.217	464	WS00922_C04
1	43.578	8	3	0.193	0.035	4.549	464	WS00922_C04
3	9.953	42	3	0.179	0.030	3.865	464	WS00922_C04
3	14.500	43	1	-0.255	0.054	5.035	464	WS00922_C04
4	0.000	71	4	0.201	0.064	3.860	464	WS00922_C04
5	31.191	95	1	-0.217	0.046	4.317	464	WS00922_C04
11	27.405	204	1	0.355	0.099	9.212	464	WS00922_C04
11	27.405	204	4	-0.229	0.077	4.728	464	WS00922_C04
11	29.069	205	1	0.234	0.057	5.258	464	WS00922_C04
11	45.989	216	1	-0.266	0.074	6.831	464	WS00922_C04
14	22.402	248	3	0.249	0.031	3.934	464	WS00922_C04
14	43.392	250	1	-0.275	0.075	6.187	464	WS00922_C04
14	44.898	251	3	0.204	0.030	3.904	464	WS00922_C04
3	9.953	42	3	0.179	0.044	4.420	465	WS00922_D14
3	15.147	44	2	0.306	0.103	6.390	465	WS00922_D14
3	20.512	46	2	0.218	0.039	4.386	465	WS00922_D14
3	95.157	59	2	-0.283	0.072	8.539	465	WS00922_D14
3	104.681	60	2	0.289	0.089	9.951	465	WS00922_D14
3	114.747	62	2	-0.226	0.041	4.660	465	WS00922_D14
3	148.745	68	1	0.301	0.051	3.921	465	WS00922_D14
3	148.745	68	4	-0.243	0.053	4.727	465	WS00922_D14
3	154.505	69	1	0.310	0.052	4.236	465	WS00922_D14
4	75.958	77	3	-0.317	0.109	5.414	465	WS00922_D14
4	97.576	80	2	0.210	0.039	4.930	465	WS00922_D14
9	69.046	181	2	0.272	0.086	4.545	465	WS00922_D14
11	45.989	216	1	-0.205	0.060	4.548	465	WS00922_D14
1	82.301	16	3	-0.208	0.034	3.896	466	WS00922_D21
3	9.953	42	3	0.227	0.036	4.173	466	WS00922_D21
4	75.958	77	3	-0.304	0.061	3.850	466	WS00922_D21
4	92.039	79	2	-0.285	0.040	4.520	466	WS00922_D21
4	97.576	80	2	0.194	0.038	4.245	466	WS00922_D21
6	87.891	124	2	-0.191	0.036	4.037	466	WS00922_D21
10	93.347	196	1	-0.417	0.078	4.174	466	WS00922_D21
12	14.406	230	3	-0.212	0.037	4.317	466	WS00922_D21
1	45.794	9	1	-0.338	0.076	6.911	467	WS00922_E23
3	95.157	59	2	-0.222	0.040	4.011	467	WS00922_E23
3	112.868	61	2	-0.207	0.046	4.526	467	WS00922_E23
3	148.745	68	1	0.313	0.060	5.547	467	WS00922_E23
5	0.000	89	1	-0.241	0.062	6.040	467	WS00922_E23
5	0.000	89	3	0.333	0.044	5.741	467	WS00922_E23
7	54.200	136	2	0.285	0.086	5.636	467	WS00922_E23
8	39.090	152	4	-0.248	0.078	4.391	467	WS00922_E23
8	50.001	154	2	-0.212	0.040	4.210	467	WS00922_E23
9	0.000	166	2	0.181	0.039	4.209	467	WS00922_E23
9	28.608	170	3	0.261	0.033	4.303	467	WS00922_E23

14	46.637	252	3	0.190	0.035	4.561	467	WS00922_E23
4	6.032	72	4	0.214	0.043	4.070	468	WS00922_F08
4	92.039	79	2	-0.250	0.037	4.336	468	WS00922_F08
4	97.576	80	2	0.186	0.039	5.110	468	WS00922_F08
4	173.929	88	2	-0.228	0.048	5.593	468	WS00922_F08
9	3.127	168	1	0.370	0.185	3.841	468	WS00922_F08
9	17.951	169	2	0.236	0.041	4.385	468	WS00922_F08
9	81.073	183	3	-0.255	0.049	4.604	468	WS00922_F08
10	35.204	189	1	0.334	0.096	6.793	468	WS00922_F08
11	27.405	204	1	0.320	0.119	8.698	468	WS00922_F08
11	37.143	209	3	0.205	0.049	4.546	468	WS00922_F08
11	64.014	222	4	-0.279	0.044	4.213	468	WS00922_F08
3	15.147	44	2	0.358	0.104	6.509	469	WS00922_G19
3	20.512	46	2	0.189	0.035	4.196	469	WS00922_G19
3	95.157	59	2	-0.192	0.039	5.267	469	WS00922_G19
3	104.681	60	2	0.269	0.062	7.503	469	WS00922_G19
3	114.747	62	2	-0.196	0.035	4.240	469	WS00922_G19
3	116.958	63	4	-0.212	0.054	4.662	469	WS00922_G19
4	6.032	72	4	0.203	0.050	4.280	469	WS00922_G19
4	82.462	78	1	-0.228	0.085	5.690	469	WS00922_G19
5	0.000	89	1	-0.293	0.080	5.630	469	WS00922_G19
5	16.219	93	1	-0.235	0.067	4.471	469	WS00922_G19
9	69.046	181	2	0.344	0.130	7.250	469	WS00922_G19
9	81.073	183	3	-0.260	0.054	5.239	469	WS00922_G19
10	35.204	189	1	0.246	0.061	4.093	469	WS00922_G19
13	1.815	244	4	0.272	0.075	4.769	469	WS00922_G19
1	62.128	11	1	-0.466	0.094	4.167	470	WS00922_H03
3	15.147	44	2	0.307	0.088	4.025	470	WS00922_H03
3	95.157	59	2	-0.279	0.107	10.416	470	WS00922_H03
3	104.681	60	2	0.247	0.059	5.708	470	WS00922_H03
4	92.039	79	4	-0.336	0.059	5.678	470	WS00922_H03
5	0.000	89	3	0.275	0.044	3.847	470	WS00922_H03
13	1.815	244	2	0.259	0.054	5.061	470	WS00922_H03
9	32.356	171	2	-0.207	0.044	5.125	471	WS00946_E01
10	21.864	188	1	0.199	0.058	4.005	471	WS00946_E01
11	56.921	219	2	-0.376	0.088	6.256	471	WS00946_E01
12	38.543	232	4	0.378	0.103	3.937	471	WS00946_E01
1	131.686	23	2	0.409	0.057	3.970	472	WS00817_M13
2	48.839	29	1	0.226	0.057	4.641	472	WS00817_M13
2	63.261	30	3	-0.210	0.057	4.457	472	WS00817_M13
3	154.505	69	1	0.384	0.123	10.356	472	WS00817_M13
6	25.914	114	1	-0.204	0.052	4.174	472	WS00817_M13
6	45.897	117	1	0.212	0.051	4.119	472	WS00817_M13
7	75.883	144	2	0.411	0.061	4.676	472	WS00817_M13
1	40.464	7	4	0.304	0.040	4.022	473	WS00946_N12
6	25.914	114	1	-0.296	0.093	8.008	473	WS00946_N12
10	70.246	190	2	0.233	0.039	4.060	473	WS00946_N12
11	0.000	199	2	-0.261	0.049	4.966	473	WS00946_N12
12	22.629	237	1	0.270	0.069	5.958	473	WS00946_N12
2	48.839	29	1	0.339	0.129	10.333	474	WS00818_C22
2	63.261	30	3	-0.157	0.046	4.437	474	WS00818_C22
2	102.814	36	1	-0.305	0.047	3.959	474	WS00818_C22
6	25.914	114	1	-0.310	0.109	8.624	474	WS00818_C22
6	106.702	128	2	0.521	0.125	6.955	474	WS00818_C22

9	28.608	170	3	0.279	0.059	5.241	474	WS00818_C22
9	81.073	183	3	-0.267	0.041	3.978	474	WS00818_C22
10	78.479	193	3	-0.423	0.072	7.093	474	WS00818_C22
11	9.237	202	4	0.167	0.051	4.599	474	WS00818_C22
2	63.261	30	3	-0.272	0.075	7.367	475	WS00922_J06
6	69.073	121	3	-0.312	0.095	9.322	475	WS00922_J06
6	81.996	123	3	-0.292	0.081	3.919	475	WS00922_J06
6	106.702	128	2	-0.456	0.101	5.265	475	WS00922_J06
7	54.200	136	2	0.310	0.105	5.250	475	WS00922_J06
7	74.560	143	1	-0.228	0.046	3.849	475	WS00922_J06
5	31.191	95	1	-0.259	0.065	4.969	476	WS00922_M15
5	84.347	105	2	-0.310	0.090	10.175	476	WS00922_M15
5	86.669	106	2	0.354	0.135	6.084	476	WS00922_M15
5	3.484	90	2	-0.412	0.131	8.286	478	WS01017_O10
5	16.219	93	2	0.337	0.055	4.912	478	WS01017_O10
6	25.914	114	1	-0.255	0.059	4.327	478	WS01017_O10
9	55.358	175	3	0.439	0.096	4.224	478	WS01017_O10
11	37.143	209	3	0.253	0.052	5.276	478	WS01017_O10
11	56.921	219	1	-0.370	0.191	4.656	478	WS01017_O10
14	0.000	246	1	0.269	0.063	4.661	478	WS01017_O10
2	63.261	30	3	0.215	0.036	4.213	479	WS01017_P13
3	95.157	59	2	-0.224	0.045	4.712	479	WS01017_P13
5	7.672	92	4	0.246	0.070	4.214	479	WS01017_P13
5	31.191	95	1	-0.262	0.057	5.030	479	WS01017_P13
11	0.000	199	3	-0.299	0.043	4.990	479	WS01017_P13
11	27.405	204	1	0.311	0.055	4.827	479	WS01017_P13
11	29.069	205	1	0.235	0.047	4.118	479	WS01017_P13
11	45.989	216	1	-0.205	0.060	5.208	479	WS01017_P13
12	22.629	237	1	0.197	0.053	4.623	479	WS01017_P13
4	75.958	77	3	-0.240	0.071	4.587	480	WS01017_P24
5	31.191	95	1	-0.261	0.067	4.810	480	WS01017_P24
2	95.880	34	3	0.253	0.041	4.202	481	WS00814_B13
4	82.462	78	2	0.194	0.043	4.308	481	WS00814_B13
4	124.132	83	1	-0.446	0.143	3.974	481	WS00814_B13
9	49.013	173	1	0.245	0.049	4.303	481	WS00814_B13
9	59.963	177	1	0.283	0.050	4.464	481	WS00814_B13
11	27.405	204	1	0.334	0.070	6.211	481	WS00814_B13
11	29.069	205	1	0.270	0.063	5.567	481	WS00814_B13
12	22.629	237	1	0.193	0.051	4.519	481	WS00814_B13
12	32.473	238	1	0.186	0.048	4.214	481	WS00814_B13
13	3.386	245	1	0.236	0.045	3.983	481	WS00814_B13
6	26.350	115	2	-0.229	0.072	7.376	482	WS00814_C05
11	0.000	199	3	-0.353	0.063	7.551	482	WS00814_C05
11	17.088	203	1	-0.236	0.045	3.934	482	WS00814_C05
11	27.405	204	1	0.298	0.047	4.058	482	WS00814_C05
11	29.069	205	1	0.257	0.053	4.605	482	WS00814_C05
13	3.386	245	1	0.255	0.045	3.930	482	WS00814_C05
1	69.854	13	2	0.196	0.035	3.911	483	WS00814_E07
3	9.953	42	3	0.257	0.077	8.936	483	WS00814_E07
3	95.157	59	2	-0.260	0.064	7.330	483	WS00814_E07
4	97.576	80	2	0.203	0.037	4.992	483	WS00814_E07
4	116.928	82	2	0.298	0.049	5.505	483	WS00814_E07
7	17.610	131	3	0.264	0.088	5.515	483	WS00814_E07
7	70.278	141	3	0.216	0.038	4.395	483	WS00814_E07

11	27.405	204	1	0.281	0.060	4.505	483	WS00814_E07
13	1.815	244	2	0.210	0.049	5.463	483	WS00814_E07
14	22.402	248	3	0.250	0.034	4.007	483	WS00814_E07
2	126.181	39	4	0.406	0.101	4.221	484	WS00814_F15
3	9.953	42	3	0.174	0.033	4.004	484	WS00814_F15
3	95.157	59	2	-0.197	0.050	4.529	484	WS00814_F15
3	154.505	69	1	0.299	0.077	7.750	484	WS00814_F15
4	124.132	83	2	0.292	0.069	3.886	484	WS00814_F15
4	142.917	85	1	-0.357	0.090	9.344	484	WS00814_F15
5	7.672	92	1	-0.289	0.062	5.987	484	WS00814_F15
5	7.672	92	4	0.278	0.060	4.161	484	WS00814_F15
5	16.219	93	1	-0.227	0.048	4.838	484	WS00814_F15
7	17.610	131	3	0.222	0.089	3.908	484	WS00814_F15
11	40.960	210	2	0.421	0.102	4.905	484	WS00814_F15
11	40.960	210	4	-0.284	0.073	5.010	484	WS00814_F15
14	43.392	250	1	-0.269	0.060	4.605	484	WS00814_F15
2	75.550	31	2	-0.254	0.049	5.646	485	WS00815_G02
2	93.504	32	4	-0.288	0.054	4.921	485	WS00815_G02
3	144.593	66	4	-0.212	0.048	3.982	485	WS00815_G02
11	40.960	210	2	0.434	0.095	5.369	485	WS00815_G02
11	40.960	210	4	-0.380	0.087	8.016	485	WS00815_G02
11	56.921	219	1	-0.409	0.140	5.340	485	WS00815_G02
12	22.441	227	1	-0.311	0.054	4.051	485	WS00815_G02
12	22.441	227	3	0.207	0.055	5.482	485	WS00815_G02
12	38.543	232	4	0.498	0.210	7.965	485	WS00815_G02
12	51.382	242	1	0.355	0.109	4.123	485	WS00815_G02
1	50.955	10	1	0.176	0.057	4.565	486	WS00815_M11
2	126.181	39	4	0.476	0.096	4.806	486	WS00815_M11
3	95.157	59	2	-0.195	0.059	6.269	486	WS00815_M11
5	86.669	106	4	-0.397	0.091	3.856	486	WS00815_M11
7	17.610	131	3	0.315	0.131	5.329	486	WS00815_M11
10	9.580	187	2	0.216	0.075	4.992	486	WS00815_M11
10	80.088	194	1	0.254	0.063	5.026	486	WS00815_M11
3	9.953	42	3	0.215	0.043	4.930	487	WS00715_G03
3	69.071	52	1	-0.260	0.074	3.925	487	WS00715_G03
3	116.958	63	4	-0.224	0.062	4.567	487	WS00715_G03
4	75.958	77	3	-0.249	0.065	3.951	487	WS00715_G03
4	92.039	79	2	-0.214	0.038	4.303	487	WS00715_G03
4	173.929	88	2	-0.230	0.056	6.195	487	WS00715_G03
11	45.989	216	1	-0.272	0.091	7.023	487	WS00715_G03
12	14.406	230	3	-0.229	0.046	5.197	487	WS00715_G03
14	44.898	251	3	0.230	0.042	4.241	487	WS00715_G03
2	63.261	30	3	0.289	0.084	8.989	488	WS00921_A17
3	14.500	43	2	-0.293	0.046	5.446	488	WS00921_A17
3	15.147	44	2	0.303	0.095	5.300	488	WS00921_A17
3	20.512	46	2	0.286	0.082	10.209	488	WS00921_A17
3	95.157	59	2	-0.265	0.053	7.328	488	WS00921_A17
3	104.681	60	2	0.197	0.047	5.825	488	WS00921_A17
3	114.747	62	2	-0.170	0.035	4.381	488	WS00921_A17
4	97.576	80	2	0.211	0.030	4.040	488	WS00921_A17
6	69.073	121	3	0.258	0.039	4.199	488	WS00921_A17
9	69.046	181	2	0.385	0.099	4.329	488	WS00921_A17
11	27.405	204	4	-0.335	0.093	6.152	488	WS00921_A17
1	2.788	2	3	0.278	0.067	6.928	489	WS00715_N22

1	69.854	13	2	0.213	0.045	5.175	489	WS00715_N22
1	77.177	15	2	0.223	0.050	5.758	489	WS00715_N22
2	48.839	29	4	0.354	0.139	11.382	489	WS00715_N22
6	106.702	128	2	-0.459	0.094	5.418	489	WS00715_N22
8	50.001	154	1	0.289	0.098	5.949	489	WS00715_N22
9	49.013	173	1	-0.222	0.067	5.592	489	WS00715_N22
9	81.073	183	1	-0.244	0.055	4.041	489	WS00715_N22
12	6.242	234	2	0.283	0.056	6.976	489	WS00715_N22
1	62.128	11	1	-0.502	0.125	4.789	490	WS00921_F03
1	123.316	20	4	0.186	0.047	4.061	490	WS00921_F03
2	127.147	40	4	-0.141	0.032	4.205	490	WS00921_F03
3	144.593	66	4	0.220	0.066	8.040	490	WS00921_F03
3	148.745	68	4	-0.271	0.072	8.353	490	WS00921_F03
4	82.462	78	1	-0.187	0.068	4.983	490	WS00921_F03
4	97.576	80	2	0.171	0.038	4.372	490	WS00921_F03
4	116.928	82	4	0.331	0.064	5.745	490	WS00921_F03
5	0.000	89	1	-0.262	0.062	4.631	490	WS00921_F03
5	16.219	93	1	-0.219	0.056	4.092	490	WS00921_F03
7	105.477	147	2	0.203	0.054	4.621	490	WS00921_F03
9	0.000	166	1	0.164	0.055	4.080	490	WS00921_F03
13	0.000	243	4	0.317	0.120	4.597	490	WS00921_F03
3	137.891	64	3	0.232	0.051	5.087	491	WS00716_D15
4	173.929	88	2	-0.238	0.053	6.723	491	WS00716_D15
5	54.072	100	4	0.250	0.057	5.092	491	WS00716_D15
8	87.463	158	2	-0.207	0.033	3.895	491	WS00716_D15
9	59.963	177	4	0.235	0.050	4.555	491	WS00716_D15
10	70.246	190	2	-0.248	0.040	4.969	491	WS00716_D15
11	45.989	216	1	-0.211	0.070	4.357	491	WS00716_D15
14	44.898	251	3	0.267	0.053	5.269	491	WS00716_D15
2	75.550	31	2	-0.238	0.043	3.904	492	WS00716_E03
3	9.953	42	3	0.238	0.044	4.302	492	WS00716_E03
3	116.958	63	4	-0.198	0.044	4.031	492	WS00716_E03
4	173.929	88	2	-0.252	0.060	6.495	492	WS00716_E03
6	81.996	123	3	0.357	0.108	6.335	492	WS00716_E03
3	137.891	64	3	0.232	0.050	5.115	493	WS00716_G01
4	0.000	71	4	0.256	0.065	5.530	493	WS00716_G01
4	173.929	88	2	-0.254	0.059	6.837	493	WS00716_G01
5	54.072	100	4	0.232	0.064	5.409	493	WS00716_G01
5	57.017	102	4	-0.258	0.065	5.649	493	WS00716_G01
6	81.996	123	3	0.298	0.086	4.580	493	WS00716_G01
10	70.246	190	2	-0.230	0.037	4.232	493	WS00716_G01
11	27.405	204	4	-0.304	0.066	4.469	493	WS00716_G01
14	44.898	251	3	0.235	0.041	4.152	493	WS00716_G01
1	127.805	21	3	0.238	0.038	4.182	494	WS00716_G17
2	63.261	30	3	-0.275	0.068	7.269	494	WS00716_G17
4	82.462	78	2	-0.285	0.084	10.134	494	WS00716_G17
4	97.576	80	2	0.240	0.039	5.021	494	WS00716_G17
4	142.917	85	1	0.273	0.074	5.082	494	WS00716_G17
7	91.487	146	3	-0.248	0.041	4.445	494	WS00716_G17
12	6.242	234	4	0.253	0.052	4.104	494	WS00716_G17
14	0.000	246	2	0.145	0.034	4.055	494	WS00716_G17
1	15.589	3	2	-0.230	0.039	4.012	495	WS00716_I17
1	95.572	17	2	0.298	0.064	6.536	495	WS00716_I17
2	126.181	39	4	0.391	0.083	3.954	495	WS00716_I17

3	15.147	44	2	0.324	0.097	5.218	495	WS00716_I17
8	0.000	148	4	0.296	0.064	6.242	495	WS00716_I17
8	50.001	154	2	0.273	0.057	6.287	495	WS00716_I17
8	87.463	158	2	-0.175	0.054	5.835	495	WS00716_I17
9	59.963	177	4	0.348	0.085	8.193	495	WS00716_I17
11	45.989	216	1	-0.283	0.056	4.903	495	WS00716_I17
11	60.464	221	1	0.220	0.066	5.667	495	WS00716_I17
1	82.301	16	4	-0.334	0.055	3.903	496	WS00716_J08
2	126.181	39	2	-0.281	0.040	4.800	496	WS00716_J08
2	126.181	39	4	0.535	0.104	8.768	496	WS00716_J08
3	15.147	44	2	0.221	0.083	6.503	496	WS00716_J08
4	55.925	76	1	-0.348	0.110	7.319	496	WS00716_J08
5	31.191	95	1	-0.246	0.060	4.027	496	WS00716_J08
6	87.891	124	2	-0.199	0.048	5.824	496	WS00716_J08
6	89.401	125	2	-0.389	0.072	7.798	496	WS00716_J08
10	35.204	189	2	0.275	0.055	5.588	496	WS00716_J08
10	70.246	190	2	-0.244	0.058	7.088	496	WS00716_J08
11	0.000	199	3	-0.337	0.082	7.358	496	WS00716_J08
11	27.405	204	1	0.329	0.094	6.585	496	WS00716_J08
11	29.069	205	1	0.247	0.068	4.622	496	WS00716_J08
3	14.500	43	1	-0.290	0.072	6.742	497	WS00716_L16
3	15.147	44	2	0.314	0.079	4.781	497	WS00716_L16
3	17.158	45	1	0.232	0.047	4.438	497	WS00716_L16
6	95.249	126	1	0.278	0.051	4.084	497	WS00716_L16
8	87.463	158	2	-0.223	0.051	4.537	497	WS00716_L16
11	9.237	202	4	0.271	0.083	6.301	497	WS00716_L16
11	27.405	204	1	0.296	0.057	5.340	497	WS00716_L16
11	27.405	204	4	-0.300	0.103	7.979	497	WS00716_L16
11	29.069	205	1	0.238	0.046	4.318	497	WS00716_L16
11	45.989	216	1	-0.251	0.070	6.565	497	WS00716_L16
11	64.014	222	3	-0.203	0.040	4.509	497	WS00716_L16
1	69.854	13	2	-0.180	0.031	3.911	498	WS00716_M07
1	77.177	15	2	-0.245	0.060	7.533	498	WS00716_M07
1	95.572	17	2	-0.312	0.042	5.416	498	WS00716_M07
1	95.572	17	3	0.280	0.075	7.302	498	WS00716_M07
3	9.953	42	3	-0.268	0.056	5.466	498	WS00716_M07
3	14.500	43	1	-0.248	0.086	5.296	498	WS00716_M07
5	86.669	106	2	0.218	0.075	4.118	498	WS00716_M07
11	0.000	199	2	-0.304	0.034	4.377	498	WS00716_M07
12	28.493	228	2	0.276	0.100	7.115	498	WS00716_M07
13	3.386	245	1	0.275	0.074	4.590	498	WS00716_M07
4	82.462	78	2	0.172	0.038	3.874	499	WS00716_O14
6	95.249	126	1	0.320	0.070	4.192	499	WS00716_O14
7	59.789	137	3	-0.231	0.069	7.811	499	WS00716_O14
1	71.592	14	2	0.176	0.039	3.855	500	WS00716_P14
2	48.839	29	1	0.201	0.049	4.325	500	WS00716_P14
3	137.891	64	3	-0.175	0.039	4.173	500	WS00716_P14
6	95.249	126	1	0.345	0.120	4.984	500	WS00716_P14
7	17.610	131	3	0.201	0.063	3.925	500	WS00716_P14
12	6.242	234	2	0.218	0.050	5.620	500	WS00716_P14
13	1.815	244	2	0.190	0.050	4.946	500	WS00716_P14
13	3.386	245	1	0.305	0.063	5.690	500	WS00716_P14
3	9.953	42	3	-0.349	0.105	11.803	501	WS0089_C18
3	9.953	42	4	-0.153	0.055	4.136	501	WS0089_C18

3	112.868	61	2	0.171	0.034	3.965	501	WS0089_C18
3	114.747	62	2	0.211	0.035	4.119	501	WS0089_C18
5	56.008	101	2	0.192	0.039	4.506	501	WS0089_C18
6	25.742	113	3	0.193	0.037	4.132	501	WS0089_C18
6	69.073	121	3	-0.350	0.067	7.649	501	WS0089_C18
10	35.204	189	2	-0.263	0.038	3.988	501	WS0089_C18
12	22.441	227	3	-0.223	0.034	3.906	501	WS0089_C18
12	6.242	234	2	0.243	0.037	5.596	501	WS0089_C18
2	48.839	29	1	0.220	0.053	4.043	502	WS0089_D22
2	93.504	32	2	0.290	0.050	5.478	502	WS0089_D22
12	6.242	234	2	0.232	0.034	3.935	502	WS0089_D22
2	120.617	38	4	0.281	0.063	4.936	503	WS0089_G23
5	52.820	99	3	-0.265	0.046	4.002	503	WS0089_G23
11	56.921	219	2	-0.409	0.191	9.542	503	WS0089_G23
1	62.128	11	2	0.284	0.095	4.136	504	WS0089_H19
1	107.333	19	3	0.175	0.042	4.287	504	WS0089_H19
3	9.953	42	3	-0.226	0.053	5.511	504	WS0089_H19
4	124.132	83	1	-0.446	0.123	3.973	504	WS0089_H19
6	26.350	115	2	0.206	0.050	4.809	504	WS0089_H19
6	87.891	124	2	-0.204	0.042	4.004	504	WS0089_H19
7	70.278	141	3	0.205	0.039	4.056	504	WS0089_H19
7	91.487	146	3	0.210	0.039	3.993	504	WS0089_H19
10	35.204	189	1	0.304	0.059	5.387	504	WS0089_H19
10	80.088	194	1	0.282	0.059	5.395	504	WS0089_H19
5	56.008	101	2	-0.233	0.043	4.414	505	WS0089_K03
5	57.017	102	2	0.275	0.080	8.503	505	WS0089_K03
6	25.914	114	1	-0.224	0.048	4.028	505	WS0089_K03
7	17.610	131	3	0.331	0.153	6.576	505	WS0089_K03
8	0.000	148	4	0.210	0.047	4.488	505	WS0089_K03
13	3.386	245	1	0.251	0.050	4.222	505	WS0089_K03
3	95.157	59	2	-0.270	0.065	7.015	506	WS0089_L06
3	104.681	60	2	0.156	0.040	3.966	506	WS0089_L06
6	89.401	125	1	-0.261	0.062	5.505	506	WS0089_L06
10	35.204	189	1	0.204	0.049	4.313	506	WS0089_L06
12	14.406	230	3	-0.322	0.056	6.762	506	WS0089_L06
13	0.000	243	4	-0.384	0.199	4.021	506	WS0089_L06
3	95.157	59	2	-0.221	0.045	4.980	507	WS0089_O21
5	31.191	95	1	-0.265	0.061	5.336	507	WS0089_O21
5	42.979	97	1	0.239	0.050	4.459	507	WS0089_O21
10	21.864	188	3	0.223	0.036	4.289	507	WS0089_O21
10	35.204	189	1	0.212	0.045	3.961	507	WS0089_O21
11	37.143	209	3	0.207	0.052	5.829	507	WS0089_O21
14	43.392	250	1	-0.237	0.056	3.845	507	WS0089_O21
2	126.181	39	4	0.405	0.085	4.113	508	WS00928_P01
2	127.147	40	3	-0.154	0.044	4.754	508	WS00928_P01
3	83.563	56	3	-0.208	0.066	7.070	508	WS00928_P01
4	75.958	77	3	-0.334	0.078	4.766	508	WS00928_P01
4	173.929	88	2	-0.165	0.034	3.885	508	WS00928_P01
5	31.191	95	1	-0.272	0.080	5.883	508	WS00928_P01
6	69.073	121	3	0.184	0.036	3.882	508	WS00928_P01
1	69.854	13	2	0.268	0.065	6.663	509	WS00810_D16
1	71.592	14	2	0.271	0.067	6.904	509	WS00810_D16
1	77.177	15	2	0.264	0.062	6.346	509	WS00810_D16
3	95.157	59	2	-0.271	0.059	7.094	509	WS00810_D16

3	114.747	62	2	-0.211	0.040	4.131	509	WS00810_D16
5	31.191	95	1	-0.334	0.095	8.180	509	WS00810_D16
11	27.405	204	1	0.305	0.068	6.050	509	WS00810_D16
11	27.405	204	4	-0.315	0.075	5.207	509	WS00810_D16
11	45.989	216	1	-0.185	0.058	4.998	509	WS00810_D16
12	14.406	230	3	-0.254	0.036	4.290	509	WS00810_D16
14	0.000	246	1	0.261	0.053	4.606	509	WS00810_D16
4	140.962	84	3	-0.138	0.045	5.377	510	WS00929_C02
5	31.191	95	1	-0.274	0.056	4.398	510	WS00929_C02
5	56.008	101	1	-0.213	0.061	4.627	510	WS00929_C02
6	25.914	114	1	-0.303	0.049	4.025	510	WS00929_C02
7	15.555	130	3	-0.225	0.052	6.348	510	WS00929_C02
9	55.358	175	3	0.711	0.191	9.345	510	WS00929_C02
11	37.143	209	3	0.211	0.038	4.603	510	WS00929_C02
3	83.563	56	3	-0.181	0.043	3.890	511	WS01021_H15
3	137.891	64	3	0.276	0.045	3.971	511	WS01021_H15
5	16.219	93	2	0.207	0.040	4.282	511	WS01021_H15
3	14.500	43	2	-0.210	0.045	5.048	512	WS01021_M04
5	84.347	105	1	-0.173	0.059	4.402	512	WS01021_M04
6	25.742	113	3	-0.264	0.042	4.313	512	WS01021_M04
6	95.249	126	1	0.469	0.189	7.678	512	WS01021_M04
8	39.090	152	4	-0.294	0.047	4.461	512	WS01021_M04
10	35.204	189	2	0.312	0.074	8.866	512	WS01021_M04
13	0.000	243	4	-0.321	0.132	4.498	512	WS01021_M04
3	80.211	55	4	0.245	0.067	5.993	513	WS0063_P06
3	104.681	60	2	0.134	0.043	4.158	513	WS0063_P06
6	25.914	114	1	-0.281	0.044	4.216	513	WS0063_P06
6	87.891	124	2	-0.214	0.087	8.327	513	WS0063_P06
6	89.401	125	2	-0.435	0.104	8.905	513	WS0063_P06
11	64.014	222	4	-0.203	0.060	5.429	513	WS0063_P06
12	6.242	234	2	-0.208	0.032	4.142	513	WS0063_P06
6	25.914	114	1	-0.342	0.100	9.211	514	WS0064_A13
7	15.555	130	3	-0.256	0.042	5.129	514	WS0064_A13
8	50.001	154	1	0.225	0.043	4.276	514	WS0064_A13
10	70.246	190	2	0.273	0.054	5.190	514	WS0064_A13
14	43.392	250	1	-0.241	0.063	4.252	514	WS0064_A13
4	82.462	78	1	0.295	0.098	7.599	515	WS0064_D20
11	0.000	199	2	-0.205	0.039	4.279	515	WS0064_D20
12	0.000	233	1	-0.257	0.062	4.815	515	WS0064_D20
1	43.578	8	2	0.227	0.037	3.841	516	WS0064_E20
1	95.572	17	2	-0.196	0.041	4.113	516	WS0064_E20
4	142.917	85	1	0.217	0.049	4.307	516	WS0064_E20
5	0.000	89	3	-0.277	0.046	4.563	516	WS0064_E20
10	78.479	193	3	0.339	0.063	6.304	516	WS0064_E20
4	43.146	74	3	-0.210	0.057	5.592	517	WS0064_I01
4	82.462	78	1	0.400	0.134	12.053	517	WS0064_I01
9	2.011	167	4	0.229	0.065	5.781	517	WS0064_I01
10	70.246	190	2	0.283	0.062	6.128	517	WS0064_I01
11	48.979	218	2	0.243	0.050	4.934	517	WS0064_I01
12	28.493	228	2	0.288	0.085	4.411	517	WS0064_I01
2	48.839	29	1	0.278	0.055	6.013	518	WS0064_J06
3	15.147	44	2	-0.257	0.136	5.110	518	WS0064_J06
3	144.647	67	1	0.284	0.148	3.915	518	WS0064_J06
6	56.188	120	2	0.308	0.075	5.180	518	WS0064_J06

8	50.001	154	1	-0.209	0.041	4.900	518	WS0064_J06
8	50.001	154	2	-0.229	0.078	6.143	518	WS0064_J06
8	87.463	158	2	0.230	0.060	4.407	518	WS0064_J06
9	36.227	165	3	0.216	0.035	4.227	518	WS0064_J06
9	17.951	169	1	-0.218	0.038	4.100	518	WS0064_J06
9	17.951	169	2	0.363	0.097	8.804	518	WS0064_J06
10	9.580	187	3	0.252	0.050	4.419	518	WS0064_J06
10	77.676	192	1	0.183	0.037	4.022	518	WS0064_J06
10	80.088	194	1	0.254	0.053	5.766	518	WS0064_J06
12	14.406	230	3	-0.210	0.035	4.228	518	WS0064_J06
1	29.800	5	1	-0.203	0.094	4.660	519	WS0064_M15
1	69.854	13	2	-0.291	0.053	7.407	519	WS0064_M15
1	71.592	14	2	-0.286	0.052	7.190	519	WS0064_M15
1	77.177	15	2	-0.308	0.057	7.937	519	WS0064_M15
1	82.301	16	4	0.345	0.053	4.628	519	WS0064_M15
2	75.550	31	2	-0.721	0.236	32.625	519	WS0064_M15
2	93.504	32	2	-0.291	0.051	7.018	519	WS0064_M15
2	102.814	36	2	-0.336	0.067	10.429	519	WS0064_M15
3	14.500	43	2	0.224	0.038	5.425	519	WS0064_M15
3	15.147	44	2	-0.138	0.037	3.930	519	WS0064_M15
3	104.681	60	2	-0.189	0.054	7.445	519	WS0064_M15
3	114.747	62	2	0.255	0.039	5.425	519	WS0064_M15
6	56.188	120	2	0.167	0.026	4.563	519	WS0064_M15
9	28.608	170	2	0.277	0.041	5.826	519	WS0064_M15
11	43.491	214	4	0.164	0.039	4.712	519	WS0064_M15
1	133.992	24	2	0.197	0.039	4.458	520	WS0064_N10
3	47.928	49	1	0.242	0.083	5.998	520	WS0064_N10
3	95.157	59	2	-0.207	0.052	6.500	520	WS0064_N10
4	75.958	77	3	-0.353	0.094	4.604	520	WS0064_N10
5	16.219	93	2	0.242	0.056	4.389	520	WS0064_N10
5	31.191	95	1	-0.212	0.068	4.922	520	WS0064_N10
6	25.742	113	3	-0.244	0.047	4.547	520	WS0064_N10
14	0.000	246	1	0.198	0.054	3.863	520	WS0064_N10
3	15.147	44	2	0.309	0.089	5.453	521	WS0091_J10
4	142.917	85	3	-0.279	0.052	5.106	521	WS0091_J10
6	89.401	125	2	-0.198	0.039	4.203	521	WS0091_J10
10	78.479	193	3	0.257	0.040	3.920	521	WS0091_J10
11	0.000	199	3	-0.249	0.053	3.909	521	WS0091_J10
11	9.237	202	4	0.249	0.057	5.069	521	WS0091_J10
11	45.989	216	1	-0.256	0.074	5.809	521	WS0091_J10
1	95.572	17	2	0.289	0.044	4.315	522	WS0091_J21
2	120.617	38	3	0.299	0.099	8.398	522	WS0091_J21
2	127.147	40	3	-0.329	0.173	14.566	522	WS0091_J21
3	83.563	56	3	-0.183	0.060	5.049	522	WS0091_J21
5	3.484	90	2	-0.254	0.093	5.928	522	WS0091_J21
5	31.191	95	1	-0.215	0.043	4.014	522	WS0091_J21
6	95.249	126	1	0.300	0.075	3.988	522	WS0091_J21
8	62.580	155	1	0.323	0.129	5.078	522	WS0091_J21
9	36.227	165	4	-0.227	0.045	5.362	522	WS0091_J21
10	104.214	198	2	0.296	0.041	3.871	522	WS0091_J21
11	17.088	203	1	-0.258	0.058	5.426	522	WS0091_J21
11	27.405	204	1	0.334	0.075	6.825	522	WS0091_J21
11	29.069	205	1	0.315	0.078	7.290	522	WS0091_J21
11	45.989	216	1	-0.283	0.088	8.096	522	WS0091_J21

14	0.000	246	1	0.252	0.053	4.898	522	WS0091_J21
5	31.191	95	1	-0.366	0.114	8.931	523	WS0091_M04
6	25.742	113	3	-0.211	0.040	4.067	523	WS0091_M04
6	54.066	119	4	-0.223	0.048	4.118	523	WS0091_M04
7	75.883	144	2	-0.381	0.057	4.254	523	WS0091_M04
10	104.214	198	4	0.278	0.027	4.436	523	WS0091_M04
4	124.132	83	1	-0.605	0.132	7.569	524	WS0091_M20
9	55.358	175	1	0.375	0.092	9.656	524	WS0091_M20
11	56.921	219	1	-0.287	0.078	4.407	524	WS0091_M20
12	6.242	234	4	-0.436	0.099	9.267	524	WS0091_M20
14	44.898	251	3	0.280	0.066	4.726	524	WS0091_M20
1	29.800	5	1	-0.231	0.055	3.854	525	WS0091_P08
1	45.794	9	1	-0.291	0.062	4.401	525	WS0091_P08
1	50.955	10	1	0.199	0.057	3.944	525	WS0091_P08
2	101.076	35	4	0.408	0.104	8.825	525	WS0091_P08
2	102.814	36	2	-0.262	0.065	7.173	525	WS0091_P08
3	80.211	55	4	-0.170	0.040	4.015	525	WS0091_P08
3	95.157	59	2	-0.197	0.045	5.144	525	WS0091_P08
3	104.681	60	2	0.200	0.033	3.874	525	WS0091_P08
3	112.868	61	2	-0.231	0.037	4.378	525	WS0091_P08
3	114.747	62	2	-0.174	0.044	5.197	525	WS0091_P08
3	137.891	64	3	-0.282	0.083	7.325	525	WS0091_P08
10	9.580	187	2	0.288	0.078	6.104	525	WS0091_P08
1	133.992	24	2	0.209	0.050	4.848	526	WS0094_A05
1	141.021	25	2	-0.205	0.051	4.857	526	WS0094_A05
4	116.928	82	4	0.300	0.067	5.275	526	WS0094_A05
5	7.672	92	4	0.220	0.053	4.831	526	WS0094_A05
6	69.073	121	3	0.225	0.044	4.573	526	WS0094_A05
7	62.787	139	4	0.238	0.043	4.422	526	WS0094_A05
7	75.883	144	4	0.295	0.057	4.112	526	WS0094_A05
9	2.011	167	4	-0.213	0.046	3.842	526	WS0094_A05
10	9.580	187	3	-0.230	0.077	4.337	526	WS0094_A05
13	0.000	243	4	0.303	0.122	4.273	526	WS0094_A05
3	116.958	63	4	-0.187	0.061	4.457	527	WS0094_C03
4	140.962	84	3	-0.195	0.038	4.299	527	WS0094_C03
4	173.929	88	2	-0.192	0.042	4.879	527	WS0094_C03
6	25.914	114	1	-0.240	0.056	4.110	527	WS0094_C03
9	59.963	177	4	0.219	0.061	4.387	527	WS0094_C03
10	78.479	193	3	0.256	0.037	4.263	527	WS0094_C03
11	27.405	204	1	0.287	0.073	5.370	527	WS0094_C03
11	43.491	214	3	-0.220	0.042	4.845	527	WS0094_C03
11	45.989	216	1	-0.193	0.057	4.168	527	WS0094_C03
14	44.898	251	3	0.267	0.045	5.078	527	WS0094_C03
1	38.326	6	4	0.226	0.039	4.059	528	WS0094_C12
1	40.464	7	4	-0.243	0.044	4.304	528	WS0094_C12
2	75.550	31	1	-0.216	0.038	3.910	528	WS0094_C12
3	141.443	65	4	0.294	0.066	4.133	528	WS0094_C12
4	82.462	78	2	0.330	0.108	9.002	528	WS0094_C12
6	25.914	114	1	-0.326	0.085	8.899	528	WS0094_C12
6	76.376	122	1	-0.259	0.063	3.856	528	WS0094_C12
7	67.154	140	4	-0.347	0.090	5.592	528	WS0094_C12
8	11.974	150	1	0.302	0.072	7.471	528	WS0094_C12
9	2.011	167	4	-0.194	0.050	5.104	528	WS0094_C12
11	40.960	210	2	0.463	0.141	5.732	528	WS0094_C12

11	46.026	217	1	-0.238	0.048	4.973	528	WS0094_C12
12	32.473	238	1	0.220	0.048	4.937	528	WS0094_C12
3	137.891	64	3	0.332	0.062	7.401	529	WS0012_J20
6	69.073	121	3	0.268	0.031	4.038	529	WS0012_J20
10	70.246	190	2	-0.278	0.036	4.141	529	WS0012_J20
10	80.088	194	2	0.290	0.067	7.368	529	WS0012_J20
11	27.405	204	4	-0.287	0.125	4.907	529	WS0012_J20
11	45.989	216	1	-0.217	0.060	4.349	529	WS0012_J20
12	0.000	226	3	-0.214	0.031	3.953	529	WS0012_J20
3	20.512	46	2	0.198	0.036	3.939	530	WS0012_L13
3	69.071	52	1	-0.308	0.072	3.939	530	WS0012_L13
4	75.958	77	3	-0.389	0.122	7.729	530	WS0012_L13
5	54.072	100	4	0.184	0.056	3.924	530	WS0012_L13
7	15.555	130	3	-0.285	0.036	4.409	530	WS0012_L13
7	59.789	137	3	-0.215	0.041	4.777	530	WS0012_L13
11	37.143	209	3	0.212	0.040	4.660	530	WS0012_L13
3	15.147	44	2	0.252	0.076	4.957	531	WS0013_B24
3	137.891	64	3	0.258	0.064	5.335	531	WS0013_B24
4	0.000	71	4	0.267	0.068	6.428	531	WS0013_B24
4	173.929	88	2	-0.194	0.042	4.656	531	WS0013_B24
5	54.072	100	4	0.291	0.066	6.317	531	WS0013_B24
5	57.017	102	4	-0.394	0.103	9.917	531	WS0013_B24
8	87.463	158	2	-0.244	0.049	3.924	531	WS0013_B24
10	70.246	190	2	-0.227	0.039	4.286	531	WS0013_B24
11	9.237	202	4	0.178	0.049	4.656	531	WS0013_B24
11	27.405	204	1	0.301	0.063	5.072	531	WS0013_B24
11	27.405	204	4	-0.346	0.075	7.203	531	WS0013_B24
11	45.989	216	1	-0.260	0.089	6.839	531	WS0013_B24
14	44.898	251	3	0.269	0.053	4.951	531	WS0013_B24
1	50.955	10	1	-0.224	0.054	4.141	532	WS0013_C16
1	95.572	17	2	-0.208	0.034	3.878	532	WS0013_C16
3	14.500	43	1	-0.294	0.075	5.713	532	WS0013_C16
5	52.820	99	3	0.272	0.058	6.399	532	WS0013_C16
5	57.017	102	2	0.262	0.051	5.667	532	WS0013_C16
7	59.789	137	3	-0.193	0.045	4.960	532	WS0013_C16
10	78.479	193	3	0.315	0.047	5.205	532	WS0013_C16
11	46.026	217	1	-0.239	0.060	4.560	532	WS0013_C16
12	14.406	230	3	-0.223	0.036	3.982	532	WS0013_C16
1	62.128	11	1	-0.663	0.154	10.692	533	WS0013_J10
9	0.000	166	1	0.250	0.064	6.719	533	WS0013_J10
11	48.979	218	4	0.163	0.032	4.439	533	WS0013_J10
2	127.147	40	3	-0.243	0.046	5.300	534	WS0013_K22
4	116.928	82	2	0.316	0.050	5.602	534	WS0013_K22
4	124.132	83	2	0.405	0.119	6.118	534	WS0013_K22
4	173.929	88	2	-0.269	0.107	11.863	534	WS0013_K22
5	42.979	97	1	0.301	0.107	8.262	534	WS0013_K22
5	52.820	99	1	0.210	0.071	5.315	534	WS0013_K22
5	56.008	101	1	-0.300	0.086	6.663	534	WS0013_K22
5	84.347	105	1	-0.190	0.076	5.841	534	WS0013_K22
9	32.356	171	2	-0.246	0.044	4.888	534	WS0013_K22
9	49.013	173	2	0.204	0.062	6.718	534	WS0013_K22
12	22.441	227	3	0.202	0.044	4.934	534	WS0013_K22
1	62.128	11	1	-0.686	0.240	9.044	535	WS0014_B10
3	9.953	42	4	0.234	0.055	4.370	535	WS0014_B10

6	45.897	117	1	0.269	0.054	4.729	535	WS0014_B10
6	69.073	121	3	0.341	0.062	6.371	535	WS0014_B10
6	81.996	123	3	0.298	0.081	3.841	535	WS0014_B10
6	87.891	124	2	0.231	0.049	5.064	535	WS0014_B10
7	15.555	130	3	-0.318	0.070	7.291	535	WS0014_B10
7	75.883	144	2	-0.375	0.074	3.932	535	WS0014_B10
11	27.405	204	4	-0.207	0.054	4.551	535	WS0014_B10
13	1.815	244	4	0.231	0.075	6.537	535	WS0014_B10
13	3.386	245	1	-0.288	0.052	4.512	535	WS0014_B10
1	131.686	23	1	0.503	0.128	4.309	536	WS0014_D05
3	9.953	42	4	0.239	0.109	7.041	536	WS0014_D05
3	69.071	52	1	-0.384	0.092	5.388	536	WS0014_D05
3	116.958	63	4	-0.153	0.053	4.382	536	WS0014_D05
4	92.039	79	2	-0.242	0.052	5.319	536	WS0014_D05
6	25.914	114	1	-0.219	0.043	3.953	536	WS0014_D05
6	81.996	123	3	0.327	0.077	4.553	536	WS0014_D05
7	15.555	130	3	-0.298	0.038	4.170	536	WS0014_D05
9	59.963	177	4	0.237	0.061	4.376	536	WS0014_D05
10	86.151	195	3	-0.155	0.037	3.888	536	WS0014_D05
11	43.491	214	3	-0.166	0.045	4.709	536	WS0014_D05
12	6.242	234	2	-0.198	0.043	5.136	536	WS0014_D05
12	6.242	234	4	0.391	0.061	4.079	536	WS0014_D05
14	46.637	252	4	-0.449	0.099	8.616	536	WS0014_D05
1	2.788	2	4	-0.128	0.034	3.892	537	WS00731_G19
1	22.419	4	4	-0.509	0.220	11.394	537	WS00731_G19
1	127.805	21	4	-0.169	0.054	4.383	537	WS00731_G19
2	126.181	39	2	0.254	0.060	4.327	537	WS00731_G19
3	14.500	43	1	-0.419	0.182	21.066	537	WS00731_G19
3	17.158	45	1	0.664	0.257	30.034	537	WS00731_G19
6	81.996	123	4	0.352	0.121	6.007	537	WS00731_G19
8	78.389	157	1	-0.311	0.049	5.849	537	WS00731_G19
10	70.246	190	4	0.274	0.055	4.574	537	WS00731_G19
11	27.405	204	1	0.386	0.057	6.808	537	WS00731_G19
11	29.069	205	1	0.313	0.048	5.728	537	WS00731_G19
11	45.989	216	1	-0.145	0.039	4.471	537	WS00731_G19
11	46.026	217	1	-0.180	0.049	5.661	537	WS00731_G19
12	14.406	230	4	0.327	0.070	6.180	537	WS00731_G19
2	127.147	40	3	-0.222	0.050	4.483	538	WS00112_J03
3	83.563	56	3	-0.236	0.050	4.488	538	WS00112_J03
4	173.929	88	2	-0.188	0.052	5.398	538	WS00112_J03
5	3.484	90	2	-0.274	0.071	4.710	538	WS00112_J03
5	42.979	97	1	0.299	0.071	5.854	538	WS00112_J03
5	42.979	97	2	0.213	0.040	4.196	538	WS00112_J03
14	0.000	246	1	0.303	0.069	5.862	538	WS00112_J03
1	38.326	6	4	-0.197	0.038	4.884	539	WS00732_A23
3	148.745	68	4	0.236	0.041	3.951	539	WS00732_A23
5	56.008	101	2	-0.235	0.037	4.321	539	WS00732_A23
6	26.350	115	2	-0.166	0.039	4.615	539	WS00732_A23
6	69.073	121	3	-0.195	0.056	4.607	539	WS00732_A23
10	35.204	189	2	-0.215	0.040	4.050	539	WS00732_A23
10	77.676	192	1	0.242	0.055	3.874	539	WS00732_A23
14	46.637	252	4	-0.285	0.044	4.277	539	WS00732_A23
3	141.443	65	3	-0.378	0.144	8.009	540	WS00113_D12
4	116.928	82	2	0.282	0.044	5.040	540	WS00113_D12

5	7.672	92	4	0.301	0.071	5.824	540	WS00113_D12
6	81.996	123	3	-0.270	0.083	4.611	540	WS00113_D12
2	48.839	29	4	-0.219	0.032	3.849	541	WS00941_O23
5	7.672	92	1	-0.241	0.082	6.006	541	WS00941_O23
7	62.787	139	4	-0.215	0.041	3.964	541	WS00941_O23
8	39.090	152	4	-0.323	0.096	8.726	541	WS00941_O23
8	95.937	159	3	0.218	0.044	4.285	541	WS00941_O23
9	55.358	175	1	0.254	0.055	4.381	541	WS00941_O23
9	69.046	181	2	-0.264	0.098	6.177	541	WS00941_O23
11	0.000	199	2	0.239	0.043	4.704	541	WS00941_O23
12	6.242	234	2	0.219	0.046	5.089	541	WS00941_O23
12	51.382	242	1	0.301	0.107	4.332	541	WS00941_O23
3	9.953	42	4	0.248	0.040	4.279	542	WS01040_G24
3	144.593	66	4	0.164	0.052	5.880	542	WS01040_G24
3	148.745	68	4	-0.242	0.058	7.605	542	WS01040_G24
3	154.505	69	4	0.204	0.047	4.666	542	WS01040_G24
4	55.925	76	1	-0.220	0.056	5.058	542	WS01040_G24
4	82.462	78	1	-0.179	0.068	6.111	542	WS01040_G24
4	103.719	81	4	-0.323	0.058	6.890	542	WS01040_G24
4	116.928	82	4	0.297	0.072	7.816	542	WS01040_G24
6	49.100	118	4	0.430	0.202	10.616	542	WS01040_G24
6	106.702	128	2	-0.367	0.120	4.067	542	WS01040_G24
8	0.000	148	4	-0.349	0.065	8.092	542	WS01040_G24
8	50.001	154	2	-0.191	0.053	5.084	542	WS01040_G24
8	87.463	158	2	0.228	0.045	4.174	542	WS01040_G24
13	1.815	244	4	0.285	0.073	9.461	542	WS01040_G24
2	101.076	35	3	0.256	0.042	5.400	543	WS0014_M21
2	120.617	38	3	0.311	0.062	8.087	543	WS0014_M21
2	127.147	40	3	-0.295	0.079	10.209	543	WS0014_M21
3	148.745	68	1	0.258	0.050	4.472	543	WS0014_M21
5	84.347	105	1	-0.175	0.047	4.128	543	WS0014_M21
6	45.897	117	1	0.251	0.059	5.219	543	WS0014_M21
6	69.073	121	3	0.306	0.050	6.527	543	WS0014_M21
12	14.406	230	3	-0.212	0.031	4.072	543	WS0014_M21
12	22.629	237	1	-0.260	0.051	4.499	543	WS0014_M21
14	22.402	248	3	0.251	0.044	5.735	543	WS0014_M21
14	43.392	250	1	-0.260	0.081	4.951	543	WS0014_M21
1	95.572	17	3	0.369	0.077	9.657	544	WS0109_P19
1	107.333	19	3	0.164	0.034	4.274	544	WS0109_P19
5	56.008	101	2	-0.237	0.050	5.472	544	WS0109_P19
7	59.789	137	3	-0.250	0.048	6.072	544	WS0109_P19
7	91.487	146	3	0.179	0.034	4.270	544	WS0109_P19
9	0.000	163	2	0.467	0.065	4.215	544	WS0109_P19
9	55.358	175	3	0.536	0.132	8.054	544	WS0109_P19
10	93.347	196	1	-0.406	0.068	3.972	544	WS0109_P19
11	35.604	207	3	-0.244	0.041	5.182	544	WS0109_P19
11	37.143	209	3	0.281	0.056	7.109	544	WS0109_P19
11	42.600	213	3	-0.266	0.047	5.893	544	WS0109_P19
2	102.814	36	1	0.222	0.043	4.521	545	WS0015_L09
9	81.073	183	3	-0.223	0.042	5.483	545	WS0015_L09
10	35.204	189	1	0.248	0.064	6.028	545	WS0015_L09
14	44.898	251	3	0.205	0.041	5.342	545	WS0015_L09
2	127.147	40	3	-0.167	0.044	5.024	546	WS0081_H23
3	15.147	44	2	0.231	0.095	5.491	546	WS0081_H23

4	142.917	85	3	-0.234	0.035	3.844	546	WS0081_H23
8	87.463	158	2	-0.302	0.071	7.666	546	WS0081_H23
10	70.246	190	2	-0.211	0.042	4.635	546	WS0081_H23
11	27.405	204	1	0.343	0.090	7.316	546	WS0081_H23
11	29.069	205	1	0.257	0.058	4.609	546	WS0081_H23
11	45.989	216	1	-0.232	0.085	6.657	546	WS0081_H23
14	44.898	251	3	0.303	0.050	5.585	546	WS0081_H23
2	127.147	40	3	-0.247	0.051	6.773	547	WS00929_F06
3	137.891	64	3	0.311	0.036	5.249	547	WS00929_F06
4	75.958	77	3	-0.297	0.093	6.333	547	WS00929_F06
4	173.929	88	2	-0.148	0.041	4.077	547	WS00929_F06
5	31.191	95	1	-0.257	0.049	4.357	547	WS00929_F06
9	49.013	173	2	0.194	0.055	5.780	547	WS00929_F06
10	35.204	189	1	0.256	0.072	6.235	547	WS00929_F06
11	0.000	199	3	-0.252	0.033	4.379	547	WS00929_F06
11	64.014	222	4	-0.236	0.103	5.392	547	WS00929_F06
14	0.000	246	1	0.303	0.060	5.317	547	WS00929_F06
14	44.898	251	3	0.222	0.035	4.944	547	WS00929_F06
2	48.839	29	1	-0.268	0.053	5.257	548	WS00929_I11
2	127.147	40	3	-0.196	0.048	5.254	548	WS00929_I11
3	69.071	52	1	-0.322	0.101	6.804	548	WS00929_I11
3	83.563	56	3	-0.183	0.038	4.178	548	WS00929_I11
3	137.891	64	3	0.215	0.045	4.865	548	WS00929_I11
5	42.979	97	1	0.301	0.044	4.320	548	WS00929_I11
6	87.891	124	2	-0.210	0.046	4.192	548	WS00929_I11
9	69.046	181	2	0.250	0.116	4.450	548	WS00929_I11
10	35.204	189	1	0.322	0.071	6.934	548	WS00929_I11
11	43.491	214	3	-0.233	0.040	4.450	548	WS00929_I11
11	45.989	216	1	-0.293	0.079	7.680	548	WS00929_I11
12	14.406	230	4	0.382	0.095	6.228	548	WS00929_I11
14	22.402	248	3	0.247	0.039	4.247	548	WS00929_I11
1	45.794	9	1	-0.286	0.063	4.926	549	WS00732_K06
2	48.839	29	4	0.369	0.108	8.924	549	WS00732_K06
5	31.191	95	1	-0.296	0.089	6.857	549	WS00732_K06
7	17.610	131	3	-0.277	0.083	3.852	549	WS00732_K06
7	59.789	137	3	-0.191	0.044	4.618	549	WS00732_K06
8	50.001	154	1	0.244	0.059	3.945	549	WS00732_K06
9	36.227	165	3	-0.246	0.041	4.300	549	WS00732_K06
11	43.491	214	3	-0.280	0.073	7.710	549	WS00732_K06
11	44.884	215	3	0.299	0.060	6.363	549	WS00732_K06
11	45.989	216	2	-0.217	0.039	4.359	549	WS00732_K06
12	14.406	230	3	-0.349	0.088	9.235	549	WS00732_K06
14	43.392	250	1	-0.424	0.194	11.431	549	WS00732_K06
1	62.128	11	2	0.251	0.093	5.080	550	WS00732_M21
1	95.572	17	3	0.239	0.061	6.989	550	WS00732_M21
1	107.333	19	3	0.181	0.033	3.965	550	WS00732_M21
5	0.000	89	1	-0.191	0.051	4.064	550	WS00732_M21
5	31.191	95	1	-0.291	0.076	5.632	550	WS00732_M21
6	25.914	114	1	-0.293	0.066	4.936	550	WS00732_M21
6	56.188	120	2	0.314	0.050	5.695	550	WS00732_M21
7	54.200	136	2	0.217	0.075	4.797	550	WS00732_M21
7	74.560	143	2	0.129	0.028	3.961	550	WS00732_M21
8	50.001	154	2	-0.226	0.042	3.845	550	WS00732_M21
8	87.463	158	2	0.183	0.045	5.303	550	WS00732_M21

9	55.358	175	3	0.466	0.096	5.211	550	WS00732_M21
4	82.462	78	2	0.259	0.054	5.367	551	WS00733_D04
4	103.719	81	4	-0.222	0.057	3.928	551	WS00733_D04
7	54.200	136	2	0.222	0.086	4.492	551	WS00733_D04
7	73.169	142	1	-0.527	0.159	4.438	551	WS00733_D04
9	59.963	177	1	-0.203	0.047	4.188	551	WS00733_D04
9	69.046	181	1	0.250	0.070	4.293	551	WS00733_D04
10	95.795	197	4	0.264	0.069	5.133	551	WS00733_D04
11	60.464	221	2	-0.274	0.056	5.549	551	WS00733_D04
13	1.815	244	4	0.233	0.067	5.436	551	WS00733_D04
5	31.191	95	1	-0.212	0.047	4.787	552	WS00733_F10
5	56.008	101	1	-0.300	0.075	7.656	552	WS00733_F10
5	84.347	105	1	-0.204	0.039	4.024	552	WS00733_F10
8	50.001	154	1	0.214	0.041	4.734	552	WS00733_F10
9	55.358	175	3	0.505	0.078	6.513	552	WS00733_F10
10	35.204	189	1	0.241	0.039	4.015	552	WS00733_F10
11	37.143	209	3	0.227	0.044	6.297	552	WS00733_F10
14	43.392	250	1	-0.238	0.044	3.856	552	WS00733_F10
14	44.898	251	3	0.220	0.030	4.270	552	WS00733_F10
1	50.955	10	1	0.268	0.073	6.338	553	WS00733_J17
1	62.128	11	2	0.300	0.143	4.666	553	WS00733_J17
1	69.854	13	2	0.172	0.038	3.853	553	WS00733_J17
1	71.592	14	2	0.176	0.040	4.063	553	WS00733_J17
1	133.992	24	2	0.246	0.059	5.969	553	WS00733_J17
5	0.000	89	1	-0.239	0.045	4.001	553	WS00733_J17
7	75.883	144	4	-0.227	0.046	3.922	553	WS00733_J17
11	0.000	199	2	-0.252	0.045	4.650	553	WS00733_J17
4	97.576	80	2	0.208	0.041	4.746	554	WS00733_L03
5	0.000	89	1	-0.306	0.055	4.161	554	WS00733_L03
6	81.996	123	3	0.341	0.102	4.931	554	WS00733_L03
9	55.358	175	3	0.515	0.143	4.991	554	WS00733_L03
10	35.204	189	1	0.301	0.072	5.141	554	WS00733_L03
3	15.147	44	2	0.242	0.065	3.988	555	WS00735_B01
4	55.925	76	1	-0.288	0.072	5.416	555	WS00735_B01
5	31.191	95	1	-0.306	0.087	7.190	555	WS00735_B01
6	95.249	126	1	0.304	0.060	3.935	555	WS00735_B01
8	87.463	158	2	-0.268	0.059	6.284	555	WS00735_B01
14	44.898	251	3	0.266	0.057	6.310	555	WS00735_B01
4	97.576	80	2	0.270	0.054	5.942	556	WS00735_C02
7	59.789	137	3	-0.231	0.043	4.429	556	WS00735_C02
9	81.073	183	1	0.191	0.062	5.594	556	WS00735_C02
10	35.204	189	1	0.285	0.068	5.977	556	WS00735_C02
10	77.676	192	1	0.264	0.116	10.185	556	WS00735_C02
10	80.088	194	1	0.445	0.148	13.097	556	WS00735_C02
12	14.406	230	3	-0.255	0.065	6.613	556	WS00735_C02
3	83.563	56	3	-0.254	0.071	7.430	557	WS0105_C24
6	45.897	117	1	-0.275	0.044	4.106	557	WS0105_C24
7	62.787	139	4	-0.322	0.057	4.374	557	WS0105_C24
7	75.883	144	2	0.407	0.074	3.994	557	WS0105_C24
9	28.608	170	2	-0.309	0.052	5.214	557	WS0105_C24
9	49.013	173	2	0.170	0.061	6.857	557	WS0105_C24
11	27.405	204	1	0.450	0.136	12.601	557	WS0105_C24
11	29.069	205	1	0.254	0.066	5.914	557	WS0105_C24
12	22.441	227	1	0.233	0.054	4.865	557	WS0105_C24

1	45.794	9	3	-0.214	0.033	3.980	558	WS0105_D12
4	75.958	77	3	-0.309	0.099	6.264	558	WS0105_D12
8	78.389	157	1	-0.288	0.063	4.337	558	WS0105_D12
11	27.405	204	1	0.412	0.130	8.926	558	WS0105_D12
11	29.069	205	1	0.294	0.086	5.751	558	WS0105_D12
11	45.989	216	1	-0.158	0.066	4.474	558	WS0105_D12
2	102.814	36	1	0.198	0.049	4.115	559	WS0105_F10
3	15.147	44	2	0.333	0.104	5.988	559	WS0105_F10
3	20.512	46	2	0.186	0.050	5.279	559	WS0105_F10
11	27.405	204	1	0.380	0.098	8.096	559	WS0105_F10
11	27.405	204	4	-0.232	0.056	4.524	559	WS0105_F10
11	29.069	205	1	0.206	0.048	3.920	559	WS0105_F10
11	40.960	210	2	0.442	0.082	5.459	559	WS0105_F10
11	45.989	216	1	-0.182	0.061	4.959	559	WS0105_F10
11	45.989	216	1	-0.165	0.049	4.496	560	WS0105_G01
3	95.157	59	2	-0.254	0.042	5.420	561	WS0105_I05
4	0.000	71	4	0.268	0.062	4.524	561	WS0105_I05
7	17.610	131	3	0.214	0.064	4.541	561	WS0105_I05
9	32.356	171	2	-0.217	0.033	3.973	561	WS0105_I05
1	38.326	6	4	-0.268	0.048	4.093	562	WS0105_I17
3	47.928	49	1	0.234	0.050	4.922	562	WS0105_I17
4	124.132	83	1	-0.480	0.095	4.954	562	WS0105_I17
6	25.914	114	1	-0.257	0.052	5.184	562	WS0105_I17
6	54.066	119	4	0.224	0.038	4.019	562	WS0105_I17
7	62.787	139	4	-0.340	0.069	9.033	562	WS0105_I17
13	3.386	245	1	0.357	0.101	10.096	562	WS0105_I17
14	22.402	248	3	-0.300	0.067	5.480	562	WS0105_I17
14	46.637	252	3	0.286	0.071	5.776	562	WS0105_I17
1	40.464	7	3	0.265	0.041	5.224	563	WS0105_K11
1	43.578	8	3	0.273	0.046	5.310	563	WS0105_K11
3	83.563	56	3	-0.216	0.041	5.253	563	WS0105_K11
4	75.958	77	3	-0.318	0.095	4.812	563	WS0105_K11
5	31.191	95	1	-0.256	0.062	5.499	563	WS0105_K11
6	69.073	121	3	0.251	0.038	4.775	563	WS0105_K11
8	87.463	158	2	-0.226	0.047	4.664	563	WS0105_K11
9	49.013	173	2	0.193	0.040	4.000	563	WS0105_K11
14	22.402	248	3	0.250	0.038	4.840	563	WS0105_K11
14	43.392	250	1	-0.302	0.086	6.475	563	WS0105_K11
3	83.563	56	3	-0.219	0.037	4.228	564	WS0105_L05
4	43.146	74	3	0.196	0.035	3.962	564	WS0105_L05
4	75.958	77	3	-0.256	0.087	5.069	564	WS0105_L05
4	116.928	82	4	0.367	0.086	5.070	564	WS0105_L05
5	31.191	95	1	-0.224	0.043	3.866	564	WS0105_L05
7	74.560	143	1	0.298	0.057	5.097	564	WS0105_L05
10	35.204	189	1	0.281	0.075	6.686	564	WS0105_L05
12	14.406	230	3	-0.280	0.055	6.301	564	WS0105_L05
14	0.000	246	1	0.329	0.087	7.794	564	WS0105_L05
3	9.953	42	3	0.166	0.038	3.974	565	WS00729_B08
3	116.958	63	4	-0.195	0.049	4.057	565	WS00729_B08
3	137.891	64	3	0.296	0.053	5.429	565	WS00729_B08
4	173.929	88	2	-0.212	0.052	6.089	565	WS00729_B08
5	31.191	95	1	-0.284	0.085	6.118	565	WS00729_B08
11	27.405	204	1	0.285	0.067	4.928	565	WS00729_B08
11	45.989	216	1	-0.196	0.063	4.532	565	WS00729_B08

2	101.076	35	3	0.234	0.049	5.060	566	WS00834_A08
2	126.181	39	2	-0.290	0.092	8.508	566	WS00834_A08
2	126.181	39	4	0.607	0.193	10.063	566	WS00834_A08
2	127.147	40	3	-0.236	0.042	4.264	566	WS00834_A08
3	116.958	63	4	-0.203	0.054	4.619	566	WS00834_A08
5	86.669	106	4	0.444	0.148	6.206	566	WS00834_A08
6	69.073	121	3	0.333	0.069	7.121	566	WS00834_A08
8	62.580	155	1	0.510	0.190	7.751	566	WS00834_A08
9	0.000	163	2	0.548	0.125	4.804	566	WS00834_A08
9	2.011	167	4	-0.221	0.048	4.098	566	WS00834_A08
9	81.073	183	1	0.309	0.064	6.071	566	WS00834_A08
9	81.073	183	3	-0.184	0.042	4.406	566	WS00834_A08
10	70.246	190	2	-0.292	0.055	5.241	566	WS00834_A08
11	40.960	210	2	0.408	0.093	4.586	566	WS00834_A08
11	40.960	210	4	-0.298	0.054	4.774	566	WS00834_A08
14	22.402	248	3	0.293	0.053	5.482	566	WS00834_A08
1	62.128	11	2	0.302	0.124	7.037	567	WS00729_P12
3	9.953	42	4	0.213	0.067	4.661	567	WS00729_P12
3	95.157	59	2	-0.258	0.061	7.475	567	WS00729_P12
5	0.000	89	1	-0.258	0.059	5.193	567	WS00729_P12
5	0.000	89	3	0.316	0.041	4.810	567	WS00729_P12
5	31.191	95	1	-0.311	0.096	7.517	567	WS00729_P12
6	25.914	114	1	-0.317	0.092	7.236	567	WS00729_P12
6	54.066	119	4	-0.213	0.082	5.997	567	WS00729_P12
6	69.073	121	4	0.234	0.058	4.189	567	WS00729_P12
14	46.637	252	4	-0.334	0.072	4.701	567	WS00729_P12
3	9.953	42	3	0.159	0.036	4.123	568	WS00834_L07
4	0.000	71	4	0.225	0.064	4.692	568	WS00834_L07
4	173.929	88	2	-0.266	0.079	8.969	568	WS00834_L07
10	35.204	189	1	0.231	0.058	4.314	568	WS00834_L07
10	78.479	193	3	0.386	0.055	6.347	568	WS00834_L07
11	27.405	204	1	0.293	0.083	6.297	568	WS00834_L07
11	29.069	205	1	0.196	0.055	4.115	568	WS00834_L07
11	43.491	214	3	-0.165	0.037	4.207	568	WS00834_L07
11	45.989	216	1	-0.272	0.105	7.867	568	WS00834_L07
3	95.157	59	2	-0.190	0.031	4.278	569	WS0094_F20
3	114.747	62	2	-0.191	0.041	4.653	569	WS0094_F20
3	116.958	63	4	-0.200	0.061	4.630	569	WS0094_F20
4	173.929	88	2	-0.242	0.066	7.542	569	WS0094_F20
6	81.996	123	3	0.312	0.104	5.865	569	WS0094_F20
11	43.491	214	3	-0.173	0.036	4.027	569	WS0094_F20
11	45.989	216	1	-0.199	0.062	4.481	569	WS0094_F20
3	137.891	64	3	0.287	0.047	5.468	570	WS0094_K05
8	78.389	157	1	-0.264	0.054	5.074	570	WS0094_K05
11	27.405	204	4	-0.335	0.088	5.234	570	WS0094_K05
11	43.491	214	3	-0.222	0.047	5.285	570	WS0094_K05
14	43.392	250	1	-0.272	0.080	5.295	570	WS0094_K05
14	44.898	251	3	0.227	0.047	5.138	570	WS0094_K05
4	0.000	71	4	0.354	0.100	8.553	571	WS0018_B21
4	173.929	88	2	-0.147	0.039	4.130	571	WS0018_B21
9	28.608	170	2	-0.259	0.047	5.073	571	WS0018_B21
9	49.013	173	2	0.179	0.048	5.541	571	WS0018_B21
12	14.406	230	3	-0.292	0.056	5.767	571	WS0018_B21
12	14.406	230	4	0.203	0.048	4.032	571	WS0018_B21

1	82.301	16	4	-0.340	0.081	9.465	572	WS00940_L22
2	63.261	30	3	-0.197	0.055	3.983	572	WS00940_L22
2	101.076	35	4	-0.336	0.069	7.524	572	WS00940_L22
3	9.953	42	4	-0.237	0.047	5.550	572	WS00940_L22
3	47.928	49	1	-0.272	0.108	7.664	572	WS00940_L22
3	144.593	66	4	-0.199	0.039	4.743	572	WS00940_L22
4	55.925	76	1	0.220	0.067	4.367	572	WS00940_L22
4	103.719	81	4	0.269	0.061	7.455	572	WS00940_L22
4	116.928	82	4	-0.322	0.045	5.219	572	WS00940_L22
11	9.237	202	4	-0.221	0.036	4.141	572	WS00940_L22
2	48.839	29	1	-0.300	0.056	4.554	573	WS0018_P01
3	20.512	46	2	-0.196	0.039	4.205	573	WS0018_P01
3	137.891	64	3	0.294	0.054	6.226	573	WS0018_P01
5	0.000	89	1	-0.209	0.052	4.506	573	WS0018_P01
5	0.000	89	3	0.269	0.036	4.114	573	WS0018_P01
5	54.072	100	4	0.225	0.065	4.561	573	WS0018_P01
5	57.017	102	4	-0.314	0.095	6.494	573	WS0018_P01
9	36.227	165	3	0.248	0.058	6.763	573	WS0018_P01
14	44.898	251	3	0.234	0.039	4.432	573	WS0018_P01
3	14.500	43	2	-0.293	0.074	7.911	574	WS00941_E19
3	15.147	44	2	0.315	0.102	6.007	574	WS00941_E19
3	20.512	46	2	0.260	0.053	6.178	574	WS00941_E19
5	57.017	102	4	-0.313	0.090	7.025	574	WS00941_E19
5	84.347	105	1	-0.238	0.064	4.553	574	WS00941_E19
11	37.143	209	3	0.178	0.042	4.457	574	WS00941_E19
12	6.242	234	2	-0.316	0.062	8.584	574	WS00941_E19
12	6.242	234	4	0.238	0.057	4.001	574	WS00941_E19
3	47.928	49	1	0.237	0.045	5.089	575	WS00923_E02
3	95.157	59	2	-0.189	0.051	4.423	575	WS00923_E02
4	124.132	83	1	0.460	0.105	4.446	575	WS00923_E02
4	142.917	85	1	-0.252	0.051	5.635	575	WS00923_E02
5	31.191	95	1	-0.261	0.058	6.532	575	WS00923_E02
6	89.401	125	1	-0.302	0.039	4.474	575	WS00923_E02
6	95.249	126	1	0.393	0.099	6.894	575	WS00923_E02
11	27.405	204	1	0.339	0.048	5.387	575	WS00923_E02
11	29.069	205	1	0.283	0.047	5.305	575	WS00923_E02
11	45.989	216	1	-0.249	0.058	6.461	575	WS00923_E02
13	1.815	244	4	0.326	0.061	3.925	575	WS00923_E02
1	82.301	16	3	-0.286	0.042	5.081	576	WS00923_H13
1	95.572	17	2	0.187	0.038	3.901	576	WS00923_H13
3	14.500	43	1	-0.327	0.071	5.956	576	WS00923_H13
3	17.158	45	1	0.234	0.060	4.977	576	WS00923_H13
5	16.219	93	1	-0.220	0.051	4.205	576	WS00923_H13
5	31.191	95	1	-0.251	0.075	6.237	576	WS00923_H13
9	36.227	165	3	-0.233	0.040	4.845	576	WS00923_H13
11	41.984	212	2	-0.178	0.038	3.989	576	WS00923_H13
2	126.181	39	2	-0.272	0.054	6.477	577	WS00820_L03
3	95.157	59	2	-0.282	0.069	8.938	577	WS00820_L03
3	104.681	60	2	0.203	0.051	6.023	577	WS00820_L03
3	114.747	62	2	-0.233	0.040	4.751	577	WS00820_L03
3	137.891	64	3	0.229	0.035	4.719	577	WS00820_L03
5	52.820	99	1	0.205	0.060	4.471	577	WS00820_L03
11	27.405	204	1	0.287	0.062	4.393	577	WS00820_L03
11	48.979	218	2	0.225	0.036	4.308	577	WS00820_L03

14	44.898	251	3	0.314	0.057	7.282	577	WS00820_L03
1	123.316	20	4	0.258	0.065	5.674	578	WS00820_L17
3	15.147	44	2	0.407	0.096	8.334	578	WS00820_L17
3	20.512	46	2	0.163	0.039	4.671	578	WS00820_L17
3	95.157	59	2	-0.262	0.062	8.323	578	WS00820_L17
3	104.681	60	2	0.233	0.057	6.974	578	WS00820_L17
4	6.032	72	4	0.172	0.038	4.218	578	WS00820_L17
4	55.925	76	1	-0.293	0.082	5.348	578	WS00820_L17
4	82.462	78	1	-0.211	0.071	4.693	578	WS00820_L17
5	86.669	106	4	0.416	0.199	4.785	578	WS00820_L17
7	75.883	144	2	-0.387	0.046	4.190	578	WS00820_L17
10	35.204	189	1	0.310	0.123	8.122	578	WS00820_L17
11	40.960	210	2	0.430	0.055	5.149	578	WS00820_L17
11	64.014	222	4	-0.269	0.059	6.467	578	WS00820_L17
13	1.815	244	4	0.197	0.043	4.199	578	WS00820_L17
2	127.147	40	3	-0.181	0.048	4.795	579	WS00820_N12
3	14.500	43	2	-0.175	0.039	4.339	579	WS00820_N12
3	15.147	44	2	0.290	0.116	7.501	579	WS00820_N12
3	20.512	46	2	0.331	0.069	8.027	579	WS00820_N12
3	104.681	60	2	0.189	0.035	4.004	579	WS00820_N12
4	55.925	76	1	-0.271	0.073	5.114	579	WS00820_N12
5	86.669	106	4	0.380	0.197	5.001	579	WS00820_N12
7	62.787	139	4	0.237	0.046	5.093	579	WS00820_N12
7	74.560	143	1	0.236	0.055	4.084	579	WS00820_N12
8	62.580	155	1	0.427	0.244	5.908	579	WS00820_N12
10	35.204	189	1	0.240	0.057	4.249	579	WS00820_N12
11	27.405	204	1	0.338	0.119	9.424	579	WS00820_N12
11	27.405	204	4	-0.330	0.057	4.989	579	WS00820_N12
11	45.989	216	1	-0.195	0.052	3.846	579	WS00820_N12
2	63.261	30	3	0.232	0.044	4.591	580	WS00820_O04
3	17.158	45	1	0.225	0.052	3.949	580	WS00820_O04
3	69.071	52	1	-0.263	0.076	4.529	580	WS00820_O04
4	173.929	88	2	-0.198	0.043	4.755	580	WS00820_O04
5	54.072	100	4	0.227	0.052	4.407	580	WS00820_O04
9	59.963	177	4	0.237	0.048	4.186	580	WS00820_O04
11	17.088	203	1	-0.221	0.051	3.891	580	WS00820_O04
11	27.405	204	1	0.473	0.185	14.412	580	WS00820_O04
11	29.069	205	1	0.350	0.142	10.845	580	WS00820_O04
11	35.604	207	3	-0.186	0.043	4.383	580	WS00820_O04
11	37.143	209	3	0.219	0.058	5.964	580	WS00820_O04
11	41.984	212	2	-0.345	0.112	12.480	580	WS00820_O04
11	45.989	216	1	-0.450	0.247	18.870	580	WS00820_O04
11	45.989	216	2	-0.314	0.088	9.779	580	WS00820_O04
11	60.464	221	1	0.507	0.198	15.166	580	WS00820_O04
11	60.464	221	2	-0.246	0.034	3.856	580	WS00820_O04
4	0.000	71	4	0.250	0.051	4.490	581	WS00820_P12
4	173.929	88	2	-0.236	0.060	6.942	581	WS00820_P12
5	57.017	102	4	-0.240	0.056	4.443	581	WS00820_P12
10	70.246	190	2	-0.260	0.050	5.906	581	WS00820_P12
11	27.405	204	1	0.324	0.111	8.303	581	WS00820_P12
11	27.405	204	4	-0.319	0.071	6.147	581	WS00820_P12
11	37.143	209	3	0.176	0.040	3.978	581	WS00820_P12
11	45.989	216	1	-0.249	0.090	6.409	581	WS00820_P12
14	22.402	248	3	0.254	0.040	4.042	581	WS00820_P12

2	95.880	34	1	0.384	0.066	6.513	582	WS00821_A07
2	95.880	34	3	-0.176	0.037	4.543	582	WS00821_A07
2	120.617	38	3	0.271	0.048	5.867	582	WS00821_A07
2	127.147	40	3	-0.261	0.046	5.564	582	WS00821_A07
3	15.147	44	2	0.281	0.083	4.220	582	WS00821_A07
6	81.996	123	3	-0.333	0.103	7.117	582	WS00821_A07
9	55.358	175	3	0.653	0.149	8.061	582	WS00821_A07
9	59.963	177	4	0.244	0.062	4.060	582	WS00821_A07
10	35.204	189	1	0.276	0.056	5.474	582	WS00821_A07
10	77.676	192	1	0.183	0.042	4.095	582	WS00821_A07
1	107.333	19	4	-0.293	0.101	9.637	583	WS00821_C07
2	127.147	40	3	-0.239	0.053	4.989	583	WS00821_C07
3	20.512	46	2	0.197	0.045	4.965	583	WS00821_C07
4	82.462	78	2	-0.191	0.046	5.177	583	WS00821_C07
4	92.039	79	2	-0.187	0.041	4.647	583	WS00821_C07
4	97.576	80	2	0.250	0.044	4.966	583	WS00821_C07
10	21.864	188	3	0.159	0.042	4.247	583	WS00821_C07
11	27.405	204	4	-0.325	0.068	6.414	583	WS00821_C07
11	56.921	219	2	-0.362	0.106	6.104	583	WS00821_C07
2	126.181	39	4	0.381	0.053	4.011	584	WS00821_C17
2	127.147	40	3	-0.172	0.045	3.893	584	WS00821_C17
3	15.147	44	2	0.396	0.152	12.577	584	WS00821_C17
3	20.512	46	2	0.279	0.052	6.474	584	WS00821_C17
4	173.929	88	2	-0.210	0.038	4.668	584	WS00821_C17
5	54.072	100	4	0.220	0.040	4.062	584	WS00821_C17
6	87.891	124	2	-0.225	0.042	5.183	584	WS00821_C17
10	35.204	189	1	0.267	0.071	4.550	584	WS00821_C17
10	70.246	190	2	-0.240	0.046	5.581	584	WS00821_C17
1	82.301	16	4	-0.360	0.081	7.799	585	WS00110_P23
5	0.000	89	1	-0.376	0.087	7.401	585	WS00110_P23
5	16.219	93	1	-0.284	0.092	7.700	585	WS00110_P23
5	31.191	95	1	-0.337	0.077	6.596	585	WS00110_P23
7	15.555	130	2	-0.279	0.040	4.331	585	WS00110_P23
9	3.127	168	1	0.433	0.114	5.314	585	WS00110_P23
9	55.358	175	1	-0.335	0.062	5.339	585	WS00110_P23
10	21.864	188	3	0.186	0.048	4.633	585	WS00110_P23
11	37.143	209	3	0.186	0.044	4.086	585	WS00110_P23
5	0.000	89	1	-0.323	0.068	5.425	586	WS00111_B24
5	0.000	89	3	0.280	0.044	4.888	586	WS00111_B24
7	17.610	131	3	0.275	0.077	5.330	586	WS00111_B24
1	62.128	11	1	-0.494	0.097	4.813	587	WS00111_G11
3	95.157	59	2	-0.197	0.047	5.282	587	WS00111_G11
3	104.681	60	2	0.214	0.046	4.692	587	WS00111_G11
3	112.868	61	2	-0.223	0.044	4.544	587	WS00111_G11
3	114.747	62	2	-0.173	0.042	4.248	587	WS00111_G11
3	148.745	68	4	-0.250	0.053	5.291	587	WS00111_G11
3	154.505	69	4	0.236	0.046	4.695	587	WS00111_G11
5	31.191	95	1	-0.248	0.070	6.025	587	WS00111_G11
6	69.073	121	3	0.352	0.085	7.353	587	WS00111_G11
6	106.702	128	2	-0.408	0.087	4.545	587	WS00111_G11
7	70.278	141	3	-0.231	0.082	7.044	587	WS00111_G11
8	50.001	154	1	0.254	0.065	5.335	587	WS00111_G11
9	3.127	168	1	0.315	0.088	4.460	587	WS00111_G11
13	1.815	244	4	0.232	0.046	4.385	587	WS00111_G11

14	43.392	250	1	-0.333	0.155	5.616	587	WS00111_G11
14	44.898	251	3	0.287	0.081	6.848	587	WS00111_G11
1	40.464	7	3	0.202	0.039	4.861	588	WS00111_I06
3	83.563	56	3	-0.202	0.039	4.866	588	WS00111_I06
8	87.463	158	2	-0.220	0.042	4.114	588	WS00111_I06
12	14.406	230	3	-0.236	0.035	4.435	588	WS00111_I06
3	83.563	56	3	-0.208	0.032	3.991	589	WS00111_M15
3	95.157	59	2	-0.217	0.043	5.292	589	WS00111_M15
3	116.958	63	4	-0.193	0.073	4.561	589	WS00111_M15
4	173.929	88	2	-0.168	0.037	3.906	589	WS00111_M15
9	55.358	175	3	0.458	0.079	4.239	589	WS00111_M15
10	35.204	189	1	0.260	0.071	5.790	589	WS00111_M15
11	27.405	204	1	0.239	0.053	4.380	589	WS00111_M15
11	40.960	210	2	0.376	0.079	3.964	589	WS00111_M15
11	45.989	216	1	-0.181	0.048	3.900	589	WS00111_M15
12	14.406	230	3	-0.196	0.035	4.308	589	WS00111_M15
3	9.953	42	3	0.187	0.052	6.649	590	WS00111_O01
3	95.157	59	2	-0.273	0.062	6.598	590	WS00111_O01
5	31.191	95	1	-0.252	0.047	4.059	590	WS00111_O01
5	42.979	97	1	0.270	0.064	5.493	590	WS00111_O01
7	105.477	147	2	-0.227	0.110	4.352	590	WS00111_O01
11	43.491	214	3	-0.261	0.048	6.228	590	WS00111_O01
11	44.884	215	3	0.255	0.030	3.995	590	WS00111_O01
12	14.406	230	3	-0.271	0.033	4.318	590	WS00111_O01
1	43.578	8	3	0.198	0.028	4.009	591	WS00112_C09
3	83.563	56	3	-0.220	0.036	5.262	591	WS00112_C09
4	75.958	77	3	-0.298	0.094	5.726	591	WS00112_C09
5	56.008	101	1	-0.216	0.046	4.504	591	WS00112_C09
6	81.996	123	3	0.203	0.046	3.868	591	WS00112_C09
7	70.278	141	3	-0.195	0.034	4.935	591	WS00112_C09
9	28.608	170	3	0.257	0.040	4.421	591	WS00112_C09
9	55.358	175	3	0.535	0.087	7.402	591	WS00112_C09
10	35.204	189	1	0.197	0.041	3.998	591	WS00112_C09
14	44.898	251	3	0.213	0.029	4.308	591	WS00112_C09
2	48.839	29	1	-0.234	0.047	4.002	592	WS00112_E01
2	127.147	40	3	-0.250	0.043	5.261	592	WS00112_E01
5	0.000	89	1	-0.244	0.046	4.041	592	WS00112_E01
5	56.008	101	1	-0.273	0.063	5.442	592	WS00112_E01
9	36.227	165	3	0.236	0.046	5.540	592	WS00112_E01
9	59.963	177	4	-0.220	0.060	3.930	592	WS00112_E01
10	35.204	189	1	0.293	0.066	5.607	592	WS00112_E01
1	22.419	4	4	0.457	0.134	6.836	593	WS01016_D21
1	38.326	6	4	0.350	0.034	4.265	593	WS01016_D21
2	127.147	40	3	-0.244	0.039	3.914	593	WS01016_D21
3	9.953	42	4	0.200	0.044	3.861	593	WS01016_D21
4	6.032	72	4	0.153	0.056	5.397	593	WS01016_D21
4	43.146	74	3	0.297	0.058	5.531	593	WS01016_D21
4	55.925	76	1	-0.305	0.095	9.452	593	WS01016_D21
6	69.073	121	3	0.297	0.055	5.115	593	WS01016_D21
7	62.787	139	4	0.181	0.041	4.163	593	WS01016_D21
7	67.154	140	4	0.225	0.100	4.379	593	WS01016_D21
7	73.169	142	1	-0.533	0.103	4.551	593	WS01016_D21
7	75.883	144	2	-0.455	0.099	5.797	593	WS01016_D21
7	75.883	144	4	0.412	0.078	7.855	593	WS01016_D21

8	87.463	158	2	-0.201	0.047	4.312	593	WS01016_D21
11	17.088	203	1	0.267	0.058	5.517	593	WS01016_D21
14	44.898	251	3	0.160	0.039	3.983	593	WS01016_D21
3	14.500	43	1	-0.223	0.068	5.091	594	WS01016_J10
3	17.158	45	1	0.290	0.069	5.197	594	WS01016_J10
11	27.405	204	1	0.318	0.061	4.621	594	WS01016_J10
1	38.326	6	2	0.225	0.067	6.255	595	WS0084_P04
1	127.805	21	3	-0.179	0.042	3.989	595	WS0084_P04
4	140.962	84	3	0.200	0.042	3.973	595	WS0084_P04
9	55.358	175	3	-0.509	0.137	4.686	595	WS0084_P04
12	6.242	234	2	-0.301	0.084	6.182	595	WS0084_P04
12	22.629	237	1	0.366	0.099	9.728	595	WS0084_P04
12	32.473	238	1	0.382	0.109	10.764	595	WS0084_P04
12	51.382	242	1	-0.600	0.210	10.305	595	WS0084_P04
2	127.147	40	3	-0.213	0.041	4.031	596	WS0023_B12
5	7.672	92	1	-0.271	0.063	5.032	596	WS0023_B12
5	42.979	97	2	0.215	0.049	4.460	596	WS0023_B12
5	54.072	100	4	-0.265	0.061	5.551	596	WS0023_B12
5	56.008	101	2	0.171	0.044	4.120	596	WS0023_B12
5	57.017	102	2	-0.255	0.042	4.961	596	WS0023_B12
6	81.996	123	4	0.394	0.198	5.191	596	WS0023_B12
8	78.389	157	1	-0.276	0.090	6.543	596	WS0023_B12
11	41.984	212	2	0.228	0.053	6.039	596	WS0023_B12
11	43.491	214	4	0.194	0.050	4.227	596	WS0023_B12
11	57.661	220	4	-0.307	0.101	9.107	596	WS0023_B12
11	64.014	222	4	0.324	0.073	5.955	596	WS0023_B12
1	95.572	17	2	0.208	0.070	6.065	597	WS0085_E24
3	14.500	43	1	-0.271	0.040	4.053	597	WS0085_E24
3	17.158	45	1	0.212	0.063	6.354	597	WS0085_E24
5	16.219	93	2	0.291	0.058	4.972	597	WS0085_E24
7	59.789	137	3	-0.257	0.057	5.899	597	WS0085_E24
10	77.676	192	1	0.225	0.052	5.212	597	WS0085_E24
10	80.088	194	1	0.196	0.042	4.293	597	WS0085_E24
11	29.069	205	1	0.187	0.046	4.655	597	WS0085_E24
11	45.989	216	1	-0.304	0.062	6.287	597	WS0085_E24
12	22.629	237	1	-0.157	0.043	4.322	597	WS0085_E24
14	0.000	246	1	0.273	0.094	9.518	597	WS0085_E24
2	75.550	31	2	-0.214	0.031	4.273	598	WS0023_I17
2	95.880	34	1	0.315	0.076	4.804	598	WS0023_I17
2	127.147	40	3	-0.224	0.039	4.451	598	WS0023_I17
4	0.000	71	4	0.276	0.070	5.278	598	WS0023_I17
5	31.191	95	1	-0.275	0.066	4.246	598	WS0023_I17
8	50.001	154	2	-0.231	0.035	4.544	598	WS0023_I17
1	15.589	3	2	-0.250	0.051	5.736	599	WS0105_O03
3	83.563	56	3	0.165	0.038	4.387	599	WS0105_O03
3	95.157	59	2	-0.171	0.046	5.052	599	WS0105_O03
4	0.000	71	4	0.173	0.088	6.389	599	WS0105_O03
4	116.928	82	2	0.253	0.041	4.654	599	WS0105_O03
7	59.789	137	3	-0.311	0.071	8.145	599	WS0105_O03
7	70.278	141	3	0.259	0.049	5.705	599	WS0105_O03
9	59.963	177	4	0.373	0.070	5.057	599	WS0105_O03
10	80.088	194	1	0.247	0.069	5.240	599	WS0105_O03
13	1.815	244	2	0.169	0.035	3.924	599	WS0105_O03
4	116.928	82	2	0.324	0.047	5.182	600	WS0106_D18

11	27.405	204	1	0.212	0.063	5.163	600	WS0106_D18
11	40.960	210	2	0.381	0.092	5.314	600	WS0106_D18
14	43.392	250	1	-0.311	0.077	4.153	600	WS0106_D18
1	62.128	11	2	0.339	0.107	6.879	601	WS00923_K23
1	95.572	17	3	0.228	0.037	4.826	601	WS00923_K23
7	54.200	136	2	0.259	0.071	5.821	601	WS00923_K23
7	70.278	141	3	0.230	0.038	4.448	601	WS00923_K23
9	69.046	181	1	0.352	0.124	4.180	601	WS00923_K23
2	127.147	40	3	-0.262	0.050	5.821	602	WS00923_O05
3	9.953	42	3	0.191	0.039	4.510	602	WS00923_O05
3	137.891	64	3	0.305	0.053	4.838	602	WS00923_O05
4	0.000	71	4	0.263	0.082	5.955	602	WS00923_O05
5	31.191	95	1	-0.224	0.059	4.035	602	WS00923_O05
8	78.389	157	1	-0.221	0.057	3.895	602	WS00923_O05
8	95.937	159	3	-0.258	0.050	5.782	602	WS00923_O05
11	45.989	216	1	-0.225	0.075	5.102	602	WS00923_O05
1	2.788	2	4	-0.110	0.032	4.329	603	WS0107_L16
1	22.419	4	4	-0.290	0.141	6.748	603	WS0107_L16
1	29.800	5	1	0.190	0.051	5.005	603	WS0107_L16
1	69.854	13	2	0.285	0.053	4.991	603	WS0107_L16
1	71.592	14	2	0.295	0.061	5.621	603	WS0107_L16
1	77.177	15	2	0.324	0.080	7.398	603	WS0107_L16
3	95.157	59	2	-0.265	0.046	4.782	603	WS0107_L16
4	92.039	79	2	-0.151	0.045	4.130	603	WS0107_L16
8	50.001	154	2	-0.261	0.053	5.647	603	WS0107_L16
8	78.389	157	1	-0.290	0.043	4.452	603	WS0107_L16
11	17.088	203	1	-0.284	0.046	4.869	603	WS0107_L16
11	27.405	204	1	0.459	0.117	11.705	603	WS0107_L16
11	29.069	205	1	0.238	0.055	5.422	603	WS0107_L16
11	40.960	210	2	0.393	0.081	4.567	603	WS0107_L16
12	32.473	238	1	0.138	0.048	4.663	603	WS0107_L16
13	3.386	245	1	-0.211	0.043	4.242	603	WS0107_L16
14	0.000	246	2	-0.317	0.076	7.402	603	WS0107_L16
14	43.392	250	2	0.103	0.037	3.879	603	WS0107_L16
3	69.071	52	1	-0.279	0.068	4.350	604	WS0107_M05
5	31.191	95	1	-0.229	0.052	6.210	604	WS0107_M05
5	42.979	97	1	0.286	0.041	4.965	604	WS0107_M05
9	55.358	175	3	0.464	0.084	4.426	604	WS0107_M05
11	40.960	210	2	0.430	0.142	5.084	604	WS0107_M05
11	40.960	210	4	-0.334	0.090	8.019	604	WS0107_M05
14	44.898	251	3	0.282	0.043	4.104	604	WS0107_M05
2	127.147	40	3	-0.236	0.047	5.136	605	WS0107_O15
3	9.953	42	3	0.213	0.047	5.135	605	WS0107_O15
3	137.891	64	3	0.250	0.040	4.321	605	WS0107_O15
4	173.929	88	2	-0.194	0.044	4.449	605	WS0107_O15
6	81.996	123	3	0.305	0.096	5.672	605	WS0107_O15
11	37.143	209	3	0.214	0.041	4.430	605	WS0107_O15
11	43.491	214	3	-0.179	0.038	4.115	605	WS0107_O15
11	45.989	216	1	-0.250	0.074	6.427	605	WS0107_O15
14	44.898	251	3	0.205	0.041	4.233	605	WS0107_O15
3	112.868	61	2	-0.181	0.038	3.904	606	WS0107_P02
3	114.747	62	2	-0.230	0.050	5.181	606	WS0107_P02
3	116.958	63	4	-0.198	0.054	4.232	606	WS0107_P02
3	137.891	64	3	0.248	0.043	4.708	606	WS0107_P02

4	173.929	88	2	-0.221	0.054	5.544	606	WS0107_P02
6	81.996	123	3	0.325	0.102	6.246	606	WS0107_P02
11	27.405	204	1	0.211	0.051	4.455	606	WS0107_P02
11	45.989	216	1	-0.245	0.069	5.869	606	WS0107_P02
11	64.014	222	4	-0.304	0.067	5.220	606	WS0107_P02
1	133.992	24	2	-0.193	0.040	3.875	607	WS0108_B15
3	144.593	66	4	0.164	0.047	4.628	607	WS0108_B15
3	148.745	68	1	-0.265	0.051	4.592	607	WS0108_B15
3	154.505	69	1	-0.275	0.060	5.498	607	WS0108_B15
4	103.719	81	4	-0.263	0.056	5.239	607	WS0108_B15
5	7.672	92	1	-0.224	0.041	5.026	607	WS0108_B15
6	49.100	118	4	0.455	0.208	9.856	607	WS0108_B15
6	56.188	120	1	-0.188	0.044	3.950	607	WS0108_B15
6	69.073	121	4	-0.265	0.045	4.397	607	WS0108_B15
7	75.883	144	2	0.452	0.103	5.123	607	WS0108_B15
10	21.864	188	1	0.268	0.057	5.241	607	WS0108_B15
11	9.237	202	4	0.251	0.042	3.958	607	WS0108_B15
11	43.491	214	3	-0.153	0.043	4.068	607	WS0108_B15
11	44.884	215	3	0.292	0.063	6.022	607	WS0108_B15
5	31.191	95	1	-0.305	0.065	6.295	608	WS0108_C10
5	56.008	101	1	-0.219	0.050	4.822	608	WS0108_C10
8	11.974	150	1	0.165	0.041	3.968	608	WS0108_C10
8	50.001	154	1	0.271	0.052	4.763	608	WS0108_C10
14	44.898	251	3	0.277	0.053	5.517	608	WS0108_C10
1	22.419	4	3	-0.426	0.174	8.574	609	WS0108_E02
1	22.419	4	4	0.409	0.188	6.262	609	WS0108_E02
1	95.572	17	2	-0.287	0.049	5.200	609	WS0108_E02
2	101.076	35	3	-0.246	0.046	4.072	609	WS0108_E02
2	120.617	38	3	-0.248	0.059	5.149	609	WS0108_E02
2	127.147	40	3	0.196	0.061	5.303	609	WS0108_E02
3	15.147	44	2	-0.226	0.067	4.505	609	WS0108_E02
6	26.350	115	2	0.206	0.048	5.048	609	WS0108_E02
1	127.805	21	3	0.209	0.038	5.259	610	WS0108_E22
3	69.071	52	1	-0.298	0.055	4.652	610	WS0108_E22
3	148.745	68	1	-0.283	0.047	4.976	610	WS0108_E22
4	173.929	88	2	-0.221	0.064	5.341	610	WS0108_E22
6	81.996	123	3	0.296	0.054	4.540	610	WS0108_E22
9	81.073	183	3	-0.304	0.062	7.531	610	WS0108_E22
10	78.479	193	3	0.305	0.029	4.004	610	WS0108_E22
12	22.441	227	3	0.236	0.035	4.748	610	WS0108_E22
14	22.402	248	3	0.281	0.030	4.212	610	WS0108_E22
3	14.500	43	1	-0.237	0.064	4.672	611	WS0109_C13
3	15.147	44	2	0.317	0.072	5.005	611	WS0109_C13
3	17.158	45	1	0.238	0.056	4.111	611	WS0109_C13
3	148.745	68	4	-0.216	0.042	4.165	611	WS0109_C13
4	0.000	71	4	0.245	0.060	5.857	611	WS0109_C13
4	173.929	88	2	-0.279	0.083	9.573	611	WS0109_C13
5	52.820	99	3	0.266	0.049	4.349	611	WS0109_C13
11	27.405	204	1	0.270	0.057	4.077	611	WS0109_C13
11	27.405	204	4	-0.193	0.039	3.856	611	WS0109_C13
1	45.794	9	1	-0.194	0.039	3.864	612	WS0109_D12
1	127.805	21	4	0.173	0.051	5.262	612	WS0109_D12
3	47.928	49	1	0.287	0.067	6.680	612	WS0109_D12
6	81.996	123	4	-0.288	0.085	5.802	612	WS0109_D12

10	78.479	193	4	-0.258	0.051	4.819	612	WS0109_D12
14	0.000	246	1	0.322	0.078	7.710	612	WS0109_D12
14	44.898	251	1	0.240	0.050	5.012	612	WS0109_D12
1	38.326	6	4	-0.298	0.051	5.218	613	WS0109_G01
1	40.464	7	4	0.334	0.065	7.233	613	WS0109_G01
3	9.953	42	4	-0.185	0.039	4.994	613	WS0109_G01
3	17.158	45	1	0.272	0.051	4.547	613	WS0109_G01
3	148.745	68	4	0.272	0.059	6.800	613	WS0109_G01
3	154.505	69	4	-0.226	0.046	4.917	613	WS0109_G01
4	116.928	82	4	-0.256	0.051	4.990	613	WS0109_G01
5	0.000	89	1	0.297	0.059	5.315	613	WS0109_G01
5	54.072	100	4	-0.168	0.042	4.297	613	WS0109_G01
7	54.200	136	2	-0.354	0.112	4.693	613	WS0109_G01
7	74.560	143	1	-0.214	0.081	7.049	613	WS0109_G01
7	75.883	144	4	-0.342	0.058	5.459	613	WS0109_G01
11	46.026	217	1	-0.208	0.062	5.372	613	WS0109_G01
11	64.014	222	4	0.306	0.065	7.889	613	WS0109_G01
12	6.242	234	2	0.175	0.041	5.249	613	WS0109_G01
13	3.386	245	1	0.275	0.042	3.883	613	WS0109_G01
3	95.157	59	2	-0.210	0.029	3.916	614	WS0109_H06
5	7.672	92	4	0.241	0.065	4.122	615	WS0109_I22
11	45.989	216	1	-0.213	0.060	4.021	615	WS0109_I22
1	22.419	4	3	-0.251	0.070	3.910	616	WS0109_J11
3	144.647	67	1	0.340	0.098	4.214	616	WS0109_J11
4	142.917	85	1	-0.264	0.045	4.521	616	WS0109_J11
6	49.100	118	4	0.337	0.098	4.166	616	WS0109_J11
10	93.347	196	2	0.441	0.092	3.963	616	WS0109_J11
11	60.464	221	2	0.235	0.050	4.520	616	WS0109_J11
14	0.000	246	1	0.254	0.051	5.042	616	WS0109_J11
10	35.204	189	1	0.236	0.062	5.458	617	WS0109_M03
11	0.000	199	2	0.215	0.039	3.880	617	WS0109_M03
11	27.405	204	1	0.297	0.058	5.062	617	WS0109_M03
11	27.405	204	4	-0.201	0.054	3.948	617	WS0109_M03
11	45.989	216	1	-0.256	0.070	6.195	617	WS0109_M03
14	43.392	250	1	-0.243	0.061	4.099	617	WS0109_M03
1	22.419	4	3	-0.281	0.068	4.470	618	WS0109_M16
2	63.261	30	3	0.201	0.035	4.117	618	WS0109_M16
4	92.039	79	2	-0.232	0.045	4.725	618	WS0109_M16
4	116.928	82	4	0.305	0.098	7.028	618	WS0109_M16
4	173.929	88	2	-0.238	0.080	8.140	618	WS0109_M16
6	69.073	121	3	0.247	0.036	4.156	618	WS0109_M16
9	49.013	173	2	0.167	0.038	4.037	618	WS0109_M16
10	77.676	192	1	0.192	0.051	4.362	618	WS0109_M16
10	80.088	194	1	0.304	0.067	5.757	618	WS0109_M16
11	27.405	204	1	0.252	0.051	4.396	618	WS0109_M16
3	9.953	42	3	0.214	0.048	5.451	619	WS0265_G13
4	140.962	84	3	-0.174	0.034	3.861	619	WS0265_G13
9	59.963	177	4	0.255	0.057	4.141	619	WS0265_G13
11	27.405	204	1	0.255	0.062	5.812	619	WS0265_G13
11	45.989	216	1	-0.206	0.049	4.504	619	WS0265_G13
14	22.402	248	3	0.278	0.044	5.066	619	WS0265_G13
14	43.392	250	1	-0.343	0.111	8.320	619	WS0265_G13
1	15.589	3	2	-0.237	0.060	6.088	620	WS0265_I19
3	15.147	44	2	0.292	0.098	4.643	620	WS0265_I19

4	0.000	71	4	0.308	0.112	10.317	620	WS0265_I19
4	173.929	88	2	-0.225	0.050	5.063	620	WS0265_I19
6	45.897	117	1	-0.323	0.077	6.852	620	WS0265_I19
6	49.100	118	4	-0.355	0.190	7.615	620	WS0265_I19
11	0.000	199	2	0.284	0.061	6.193	620	WS0265_I19
11	27.405	204	1	0.323	0.076	6.633	620	WS0265_I19
11	29.069	205	1	0.312	0.075	6.600	620	WS0265_I19
11	45.989	216	1	-0.319	0.103	8.895	620	WS0265_I19
11	56.921	219	1	-0.234	0.100	3.975	620	WS0265_I19
14	22.402	248	3	0.229	0.045	4.281	620	WS0265_I19
3	20.512	46	2	0.132	0.043	4.158	621	WS0265_O13
5	42.979	97	1	-0.341	0.057	5.333	621	WS0265_O13
5	52.820	99	1	-0.332	0.056	5.111	621	WS0265_O13
5	56.008	101	1	0.202	0.066	5.907	621	WS0265_O13
5	84.347	105	1	0.314	0.108	9.742	621	WS0265_O13
6	81.996	123	4	-0.585	0.197	10.969	621	WS0265_O13
9	3.127	168	1	0.393	0.092	4.497	621	WS0265_O13
1	40.464	7	4	0.340	0.050	3.949	622	WS0266_A18
1	45.794	9	3	-0.248	0.062	5.949	622	WS0266_A18
1	95.572	17	3	0.442	0.174	14.632	622	WS0266_A18
1	107.333	19	3	0.306	0.101	9.461	622	WS0266_A18
3	15.147	44	2	-0.382	0.198	10.580	622	WS0266_A18
7	62.787	139	4	-0.217	0.046	4.228	622	WS0266_A18
9	28.608	170	3	0.300	0.061	5.078	622	WS0266_A18
10	9.580	187	2	0.231	0.076	4.053	622	WS0266_A18
12	51.382	242	1	-0.386	0.115	4.699	622	WS0266_A18
3	137.891	64	3	0.275	0.056	5.160	623	WS0266_G05
5	84.347	105	1	-0.236	0.049	4.364	623	WS0266_G05
6	25.914	114	1	-0.238	0.058	5.142	623	WS0266_G05
6	69.073	121	3	0.237	0.040	3.946	623	WS0266_G05
6	81.996	123	4	0.372	0.080	3.971	623	WS0266_G05
7	15.555	130	3	-0.208	0.044	3.933	623	WS0266_G05
7	73.169	142	1	0.734	0.165	8.748	623	WS0266_G05
7	75.883	144	2	0.427	0.119	4.709	623	WS0266_G05
7	75.883	144	4	-0.294	0.055	4.882	623	WS0266_G05
14	44.898	251	3	0.224	0.054	5.213	623	WS0266_G05
4	116.928	82	2	0.247	0.046	4.529	624	WS0266_J01
5	16.219	93	1	-0.201	0.044	3.985	624	WS0266_J01
5	31.191	95	1	-0.223	0.054	4.859	624	WS0266_J01
7	17.610	131	3	0.304	0.107	5.766	624	WS0266_J01
8	50.001	154	2	-0.229	0.067	6.427	624	WS0266_J01
10	35.204	189	1	0.278	0.058	5.249	624	WS0266_J01
11	45.989	216	1	-0.213	0.045	4.095	624	WS0266_J01
14	44.898	251	3	0.258	0.060	6.238	624	WS0266_J01
1	131.686	23	2	-0.423	0.090	4.298	625	WS0266_O10
3	9.953	42	3	0.164	0.030	3.850	625	WS0266_O10
3	116.958	63	4	-0.191	0.065	3.925	625	WS0266_O10
4	82.462	78	2	0.243	0.052	4.479	625	WS0266_O10
6	81.996	123	3	0.246	0.060	4.303	625	WS0266_O10
9	55.358	175	3	0.474	0.080	5.353	625	WS0266_O10
9	69.046	181	2	0.311	0.078	4.341	625	WS0266_O10
10	35.204	189	1	0.239	0.057	4.809	625	WS0266_O10
14	43.392	250	1	-0.244	0.057	4.093	625	WS0266_O10
14	44.898	251	3	0.215	0.041	5.199	625	WS0266_O10

1	69.854	13	2	0.207	0.038	4.808	626	WS0266_P21
1	71.592	14	2	0.201	0.036	4.578	626	WS0266_P21
1	77.177	15	2	0.202	0.036	4.559	626	WS0266_P21
1	123.316	20	4	0.263	0.044	4.132	626	WS0266_P21
1	131.686	23	2	-0.499	0.088	8.307	626	WS0266_P21
2	48.839	29	4	0.277	0.047	4.941	626	WS0266_P21
3	116.958	63	4	-0.211	0.056	6.246	626	WS0266_P21
5	54.072	100	4	0.224	0.040	4.396	626	WS0266_P21
8	50.001	154	2	-0.224	0.045	5.599	626	WS0266_P21
11	27.405	204	4	-0.319	0.061	4.187	626	WS0266_P21
1	95.572	17	2	-0.285	0.066	7.730	627	WS0058_I05
3	9.953	42	4	0.273	0.095	8.499	627	WS0058_I05
3	116.958	63	4	-0.245	0.075	7.053	627	WS0058_I05
3	137.891	64	3	0.271	0.053	4.536	627	WS0058_I05
11	0.000	199	2	-0.307	0.059	6.810	627	WS0058_I05
11	27.405	204	4	-0.310	0.071	5.206	627	WS0058_I05
12	28.493	228	2	0.301	0.068	5.113	627	WS0058_I05
12	6.242	234	4	0.334	0.063	6.219	627	WS0058_I05
1	29.800	5	2	-0.231	0.040	4.607	628	WS01014_A23
1	62.128	11	2	0.291	0.092	4.862	628	WS01014_A23
2	101.076	35	4	-0.210	0.037	3.893	628	WS01014_A23
9	17.951	169	1	-0.262	0.071	4.851	628	WS01014_A23
9	32.356	171	2	0.196	0.041	4.830	628	WS01014_A23
9	59.963	177	4	-0.284	0.067	6.505	628	WS01014_A23
10	21.864	188	1	0.236	0.060	4.156	628	WS01014_A23
10	78.479	193	3	-0.261	0.052	4.526	628	WS01014_A23
12	38.543	232	4	-0.401	0.199	6.695	628	WS01014_A23
3	137.891	64	3	0.349	0.077	4.951	629	WS00924_B07
4	124.132	83	2	-0.323	0.115	4.807	629	WS00924_B07
7	15.555	130	3	-0.316	0.067	4.988	629	WS00924_B07
7	17.610	131	3	-0.345	0.114	5.776	629	WS00924_B07
7	75.883	144	4	-0.327	0.078	5.957	629	WS00924_B07
8	39.090	152	4	-0.212	0.066	5.233	629	WS00924_B07
9	59.963	177	4	0.363	0.083	4.818	629	WS00924_B07
11	60.464	221	2	-0.213	0.044	4.676	629	WS00924_B07
1	15.589	3	2	-0.275	0.032	3.909	630	WS00924_E07
3	137.891	64	3	0.126	0.035	4.175	630	WS00924_E07
4	142.917	85	3	-0.245	0.036	4.116	630	WS00924_E07
5	16.219	93	2	0.247	0.028	4.333	630	WS00924_E07
6	69.073	121	4	0.161	0.047	5.696	630	WS00924_E07
7	73.169	142	1	0.543	0.126	4.717	630	WS00924_E07
11	9.237	202	4	0.298	0.079	8.025	630	WS00924_E07
11	29.069	205	1	0.247	0.056	4.360	630	WS00924_E07
11	45.989	216	1	-0.448	0.101	8.349	630	WS00924_E07
11	56.921	219	2	0.261	0.048	4.014	630	WS00924_E07
11	60.464	221	1	0.210	0.092	7.153	630	WS00924_E07
3	154.505	69	1	0.218	0.046	4.021	631	WS0033_E24
4	55.925	76	1	-0.294	0.073	6.808	631	WS0033_E24
5	3.484	90	2	-0.240	0.063	4.068	631	WS0033_E24
5	31.191	95	1	-0.229	0.052	4.536	631	WS0033_E24
12	6.242	234	2	-0.228	0.033	3.951	631	WS0033_E24
1	40.464	7	3	0.225	0.043	5.361	632	WS0033_H15
1	43.578	8	3	0.229	0.045	5.431	632	WS0033_H15
3	83.563	56	3	-0.212	0.039	4.824	632	WS0033_H15

3	104.681	60	2	0.171	0.038	4.032	632	WS0033_H15
3	114.747	62	2	-0.198	0.040	4.271	632	WS0033_H15
11	0.000	199	3	-0.311	0.049	4.010	632	WS0033_H15
11	43.491	214	3	-0.191	0.031	3.841	632	WS0033_H15
12	14.406	230	3	-0.231	0.036	4.547	632	WS0033_H15
14	22.402	248	3	0.219	0.032	4.033	632	WS0033_H15
3	9.953	42	4	0.276	0.066	5.300	633	WS0034_B09
3	17.158	45	1	0.231	0.047	3.916	633	WS0034_B09
4	173.929	88	2	-0.197	0.047	4.910	633	WS0034_B09
6	81.996	123	4	0.402	0.142	5.955	633	WS0034_B09
11	45.989	216	1	-0.303	0.116	9.682	633	WS0034_B09
3	20.512	46	2	0.194	0.042	5.071	634	WS0034_C09
3	104.681	60	2	0.158	0.037	4.416	634	WS0034_C09
3	112.868	61	2	-0.204	0.032	3.889	634	WS0034_C09
3	114.747	62	2	-0.189	0.040	4.830	634	WS0034_C09
11	27.405	204	1	0.302	0.069	4.752	634	WS0034_C09
11	40.960	210	2	0.411	0.072	4.677	634	WS0034_C09
11	45.989	216	1	-0.200	0.086	5.836	634	WS0034_C09
12	14.406	230	3	-0.253	0.041	4.636	634	WS0034_C09
3	15.147	44	2	0.322	0.089	7.695	635	WS0034_I05
3	20.512	46	2	0.252	0.046	5.696	635	WS0034_I05
3	104.681	60	2	0.207	0.042	5.157	635	WS0034_I05
3	114.747	62	2	-0.209	0.032	3.912	635	WS0034_I05
3	116.958	63	4	-0.173	0.038	3.890	635	WS0034_I05
11	27.405	204	1	0.312	0.100	6.744	635	WS0034_I05
11	27.405	204	4	-0.304	0.064	6.647	635	WS0034_I05
11	40.960	210	2	0.439	0.057	5.308	635	WS0034_I05
11	45.989	216	1	-0.253	0.099	6.533	635	WS0034_I05
12	6.242	234	2	-0.262	0.041	5.938	635	WS0034_I05
1	95.572	17	3	0.230	0.038	4.285	636	WS0034_L05
1	107.333	19	3	0.257	0.052	5.519	636	WS0034_L05
5	42.979	97	2	-0.188	0.033	3.885	636	WS0034_L05
5	57.017	102	2	0.268	0.047	5.443	636	WS0034_L05
7	54.200	136	2	0.344	0.091	5.112	636	WS0034_L05
7	62.787	139	4	-0.268	0.080	7.083	636	WS0034_L05
9	2.011	167	4	-0.215	0.055	4.479	636	WS0034_L05
10	78.479	193	4	-0.233	0.062	4.944	636	WS0034_L05
10	95.795	197	4	0.220	0.075	6.125	636	WS0034_L05
3	116.958	63	4	-0.269	0.086	6.173	637	WS0035_A13
3	137.891	64	1	-0.253	0.046	4.639	637	WS0035_A13
3	137.891	64	3	0.264	0.048	5.612	637	WS0035_A13
4	43.146	74	3	0.229	0.045	5.221	637	WS0035_A13
4	140.962	84	3	-0.251	0.048	5.601	637	WS0035_A13
5	16.219	93	1	-0.220	0.047	4.673	637	WS0035_A13
6	25.914	114	1	-0.244	0.059	5.911	637	WS0035_A13
7	74.560	143	1	0.230	0.048	4.749	637	WS0035_A13
10	35.204	189	1	0.280	0.049	4.913	637	WS0035_A13
3	69.071	52	1	-0.382	0.098	5.244	638	WS0035_C16
4	173.929	88	2	-0.221	0.037	4.046	638	WS0035_C16
9	0.000	163	1	0.429	0.078	4.084	638	WS0035_C16
9	36.227	165	4	0.256	0.064	4.623	638	WS0035_C16
9	55.358	175	3	0.393	0.076	3.919	638	WS0035_C16
11	45.989	216	1	-0.253	0.051	4.272	638	WS0035_C16
1	95.572	17	2	0.190	0.037	4.795	639	WS00925_B04

3	148.745	68	4	-0.197	0.051	3.919	639	WS00925_B04
4	173.929	88	2	-0.244	0.038	4.995	639	WS00925_B04
5	52.820	99	3	0.415	0.126	12.938	639	WS00925_B04
6	106.702	128	2	0.475	0.089	5.906	639	WS00925_B04
7	54.200	136	2	-0.240	0.062	3.959	639	WS00925_B04
9	17.951	169	1	-0.335	0.081	4.868	639	WS00925_B04
9	69.046	181	2	-0.303	0.070	4.598	639	WS00925_B04
9	81.073	183	3	-0.228	0.038	4.146	639	WS00925_B04
10	78.479	193	3	0.327	0.074	7.588	639	WS00925_B04
10	86.151	195	3	-0.211	0.048	4.953	639	WS00925_B04
11	35.604	207	3	-0.246	0.056	5.828	639	WS00925_B04
11	37.143	209	3	0.248	0.058	5.959	639	WS00925_B04
11	42.600	213	3	-0.257	0.058	5.984	639	WS00925_B04
11	60.464	221	1	0.195	0.067	4.043	639	WS00925_B04
3	9.953	42	3	0.223	0.047	4.196	640	WS00925_E06
3	116.958	63	4	-0.181	0.045	4.414	640	WS00925_E06
3	137.891	64	3	0.215	0.049	4.410	640	WS00925_E06
4	173.929	88	2	-0.262	0.069	8.529	640	WS00925_E06
7	62.787	139	4	0.255	0.051	5.625	640	WS00925_E06
11	27.405	204	1	0.328	0.117	8.241	640	WS00925_E06
11	27.405	204	4	-0.326	0.062	6.020	640	WS00925_E06
11	45.989	216	1	-0.242	0.092	5.985	640	WS00925_E06
3	95.157	59	2	-0.206	0.046	4.304	641	WS0031_E20
3	104.681	60	2	0.261	0.080	7.760	641	WS0031_E20
3	112.868	61	2	-0.276	0.082	7.929	641	WS0031_E20
3	114.747	62	2	-0.251	0.071	6.871	641	WS0031_E20
4	75.958	77	3	-0.287	0.075	5.602	641	WS0031_E20
6	106.702	128	2	0.372	0.057	4.299	641	WS0031_E20
9	0.000	166	2	0.189	0.045	4.836	641	WS0031_E20
9	55.358	175	3	0.650	0.165	9.382	641	WS0031_E20
10	9.580	187	3	-0.240	0.071	4.251	641	WS0031_E20
11	37.143	209	3	0.213	0.029	3.989	641	WS0031_E20
11	40.960	210	2	0.435	0.154	3.908	641	WS0031_E20
11	40.960	210	4	-0.360	0.136	7.588	641	WS0031_E20
11	56.921	219	1	-0.493	0.195	5.950	641	WS0031_E20
13	3.386	245	1	0.159	0.047	4.227	641	WS0031_E20
3	9.953	42	3	0.222	0.040	4.416	642	WS01019_D09
3	104.681	60	2	0.194	0.036	4.320	642	WS01019_D09
3	114.747	62	2	-0.224	0.040	4.729	642	WS01019_D09
4	173.929	88	2	-0.223	0.050	5.925	642	WS01019_D09
5	57.017	102	2	0.246	0.034	4.009	642	WS01019_D09
9	81.073	183	3	-0.257	0.041	4.077	642	WS01019_D09
10	35.204	189	1	0.304	0.094	6.544	642	WS01019_D09
11	27.405	204	1	0.237	0.066	4.700	642	WS01019_D09
11	27.405	204	4	-0.288	0.071	5.575	642	WS01019_D09
1	45.794	9	1	0.372	0.093	8.334	643	WS0032_F01
1	45.794	9	3	-0.249	0.053	6.009	643	WS0032_F01
6	106.702	128	2	0.448	0.079	5.081	643	WS0032_F01
13	1.815	244	2	0.268	0.063	6.264	643	WS0032_F01
13	3.386	245	1	0.238	0.069	6.160	643	WS0032_F01
14	0.000	246	1	-0.239	0.045	4.071	643	WS0032_F01
14	44.898	251	3	0.206	0.039	3.917	643	WS0032_F01
2	95.880	34	1	0.346	0.054	6.054	644	WS01019_K17
2	102.814	36	2	-0.222	0.064	4.367	644	WS01019_K17

3	148.745	68	1	-0.290	0.042	4.848	644	WS01019_K17
5	0.000	89	1	-0.263	0.033	3.841	644	WS01019_K17
9	55.358	175	3	0.587	0.101	9.681	644	WS01019_K17
12	14.406	230	3	-0.193	0.038	5.193	644	WS01019_K17
14	0.000	246	1	0.248	0.049	5.559	644	WS01019_K17
3	83.563	56	3	-0.247	0.065	6.955	645	WS01014_O21
3	95.157	59	2	-0.200	0.036	5.054	645	WS01014_O21
3	137.891	64	3	0.215	0.043	4.684	645	WS01014_O21
4	0.000	71	4	0.254	0.049	4.025	645	WS01014_O21
4	140.962	84	3	-0.210	0.036	3.910	645	WS01014_O21
4	173.929	88	2	-0.259	0.074	8.796	645	WS01014_O21
6	87.891	124	2	-0.195	0.043	5.078	645	WS01014_O21
6	89.401	125	2	-0.283	0.041	4.857	645	WS01014_O21
7	74.560	143	1	0.292	0.074	5.185	645	WS01014_O21
10	35.204	189	1	0.265	0.075	5.163	645	WS01014_O21
10	70.246	190	2	-0.199	0.035	4.178	645	WS01014_O21
11	27.405	204	1	0.248	0.075	5.569	645	WS01014_O21
11	27.405	204	4	-0.352	0.072	5.736	645	WS01014_O21
11	45.989	216	1	-0.207	0.071	4.896	645	WS01014_O21
1	127.805	21	3	0.207	0.050	4.720	646	WS01014_P12
2	48.839	29	1	-0.245	0.057	4.361	646	WS01014_P12
3	14.500	43	2	-0.341	0.092	9.604	646	WS01014_P12
3	20.512	46	2	0.282	0.064	7.057	646	WS01014_P12
3	148.745	68	4	0.191	0.052	4.492	646	WS01014_P12
4	82.462	78	2	-0.234	0.048	5.634	646	WS01014_P12
5	52.820	99	1	0.182	0.052	4.112	646	WS01014_P12
5	52.820	99	3	-0.291	0.057	5.468	646	WS01014_P12
10	9.580	187	2	-0.281	0.098	5.790	646	WS01014_P12
10	35.204	189	2	0.261	0.046	4.241	646	WS01014_P12
10	78.479	193	3	-0.343	0.066	6.278	646	WS01014_P12
10	86.151	195	3	0.226	0.049	4.661	646	WS01014_P12
11	43.491	214	4	-0.276	0.068	4.581	646	WS01014_P12
11	46.026	217	1	-0.251	0.090	6.954	646	WS01014_P12
12	22.441	227	3	0.275	0.070	7.009	646	WS01014_P12
12	22.629	237	1	-0.348	0.112	8.680	646	WS01014_P12
12	32.473	238	1	-0.249	0.053	4.162	646	WS01014_P12
1	62.128	11	1	-0.683	0.217	8.929	647	WS01015_A14
1	107.333	19	4	-0.244	0.082	8.488	647	WS01015_A14
2	126.181	39	2	-0.230	0.051	5.181	647	WS01015_A14
3	69.071	52	1	-0.332	0.092	4.142	647	WS01015_A14
3	95.157	59	2	-0.198	0.042	4.517	647	WS01015_A14
3	104.681	60	2	0.225	0.056	5.759	647	WS01015_A14
3	112.868	61	2	-0.236	0.050	5.143	647	WS01015_A14
3	114.747	62	2	-0.204	0.054	5.478	647	WS01015_A14
3	148.745	68	4	-0.233	0.042	3.973	647	WS01015_A14
4	92.039	79	2	-0.187	0.040	4.210	647	WS01015_A14
4	97.576	80	2	0.196	0.041	4.078	647	WS01015_A14
6	69.073	121	3	0.348	0.083	7.311	647	WS01015_A14
9	0.000	166	1	0.143	0.048	4.119	647	WS01015_A14
9	81.073	183	1	0.204	0.046	3.879	647	WS01015_A14
10	35.204	189	1	0.360	0.090	7.696	647	WS01015_A14
11	17.088	203	1	0.206	0.046	3.916	647	WS01015_A14
13	1.815	244	4	0.229	0.067	6.805	647	WS01015_A14
14	43.392	250	1	-0.412	0.154	6.862	647	WS01015_A14

14	46.637	252	4	-0.301	0.040	3.900	647	WS01015_A14
1	62.128	11	1	-0.481	0.064	4.932	648	WS01015_B08
2	48.839	29	4	0.251	0.048	4.428	648	WS01015_B08
2	93.504	32	4	-0.306	0.054	5.010	648	WS01015_B08
3	116.958	63	4	-0.201	0.045	4.513	648	WS01015_B08
3	137.891	64	1	0.300	0.034	4.589	648	WS01015_B08
3	144.647	67	1	0.259	0.068	5.057	648	WS01015_B08
4	103.719	81	4	-0.210	0.043	4.414	648	WS01015_B08
4	116.928	82	4	0.350	0.101	9.645	648	WS01015_B08
7	62.787	139	4	0.235	0.044	4.928	648	WS01015_B08
7	74.560	143	1	0.207	0.031	4.158	648	WS01015_B08
7	75.883	144	4	0.306	0.060	5.805	648	WS01015_B08
11	64.014	222	4	-0.328	0.083	8.210	648	WS01015_B08
12	38.543	232	4	0.331	0.060	4.617	648	WS01015_B08
13	0.000	243	4	0.371	0.128	9.623	648	WS01015_B08
13	3.386	245	1	-0.233	0.035	4.708	648	WS01015_B08
1	50.955	10	1	-0.197	0.062	4.198	649	WS01015_D09
4	124.132	83	1	-0.510	0.169	5.214	649	WS01015_D09
5	57.017	102	2	0.206	0.045	5.519	649	WS01015_D09
5	57.017	102	4	-0.241	0.061	4.080	649	WS01015_D09
6	49.100	118	4	-0.348	0.127	3.926	649	WS01015_D09
6	81.996	123	3	-0.338	0.131	4.832	649	WS01015_D09
8	50.001	154	2	-0.221	0.044	5.456	649	WS01015_D09
8	87.463	158	2	0.240	0.055	6.696	649	WS01015_D09
9	0.000	166	1	0.259	0.076	5.140	649	WS01015_D09
10	104.214	198	2	-0.234	0.034	4.375	649	WS01015_D09
11	35.604	207	3	-0.203	0.036	4.287	649	WS01015_D09
12	0.000	226	3	-0.226	0.035	4.210	649	WS01015_D09
14	0.000	246	2	0.234	0.064	7.618	649	WS01015_D09
3	20.512	46	2	0.168	0.055	4.479	650	WS01015_E08
4	0.000	71	4	0.221	0.060	4.673	650	WS01015_E08
5	7.672	92	4	0.276	0.061	4.799	650	WS01015_E08
6	25.914	114	1	0.238	0.051	5.348	650	WS01015_E08
10	9.580	187	3	0.384	0.094	7.353	650	WS01015_E08
10	35.204	189	1	0.265	0.038	4.080	650	WS01015_E08
12	32.473	238	1	-0.262	0.060	6.389	650	WS01015_E08
1	43.578	8	3	0.261	0.038	4.251	651	WS01015_G06
1	69.854	13	2	-0.222	0.075	5.901	651	WS01015_G06
1	71.592	14	2	-0.207	0.066	5.186	651	WS01015_G06
1	77.177	15	2	-0.200	0.061	4.808	651	WS01015_G06
1	107.333	19	4	-0.168	0.054	4.126	651	WS01015_G06
1	131.686	23	1	0.518	0.118	4.567	651	WS01015_G06
6	25.914	114	1	0.223	0.044	4.739	651	WS01015_G06
9	0.000	166	1	0.236	0.062	6.757	651	WS01015_G06
9	28.608	170	3	-0.235	0.042	4.333	651	WS01015_G06
10	9.580	187	3	0.398	0.108	7.607	651	WS01015_G06
10	86.151	195	3	0.340	0.084	9.572	651	WS01015_G06
10	93.347	196	1	0.400	0.056	3.912	651	WS01015_G06
12	0.000	226	3	-0.225	0.048	5.439	651	WS01015_G06
12	22.441	227	3	0.261	0.049	5.555	651	WS01015_G06
12	22.629	237	1	-0.236	0.046	5.053	651	WS01015_G06
12	32.473	238	1	-0.253	0.053	5.763	651	WS01015_G06
3	14.500	43	1	-0.224	0.068	4.596	652	WS01015_G22
4	173.929	88	2	-0.177	0.036	4.288	652	WS01015_G22

11	27.405	204	1	0.255	0.060	4.132	652	WS01015_G22
11	45.989	216	1	-0.186	0.058	3.923	652	WS01015_G22
2	93.504	32	4	-0.274	0.069	5.503	653	WS0055_O24
4	75.958	77	3	-0.290	0.104	5.529	653	WS0055_O24
4	124.132	83	1	0.610	0.230	7.438	653	WS0055_O24
4	142.917	85	1	-0.334	0.084	7.094	653	WS0055_O24
6	25.742	113	3	-0.225	0.044	4.585	653	WS0055_O24
11	45.989	216	1	-0.202	0.048	4.035	653	WS0055_O24
11	60.464	221	2	0.207	0.033	3.850	653	WS0055_O24
12	22.441	227	1	-0.237	0.047	3.972	653	WS0055_O24
2	126.181	39	2	-0.227	0.046	4.821	654	WS0045_A18
2	126.181	39	4	0.398	0.103	4.361	654	WS0045_A18
9	69.046	181	2	0.309	0.082	5.173	654	WS0045_A18
3	9.953	42	4	0.224	0.078	6.996	655	WS0045_E01
3	20.512	46	2	0.213	0.039	4.005	655	WS0045_E01
4	55.925	76	1	-0.251	0.065	5.628	655	WS0045_E01
7	59.789	137	3	-0.227	0.046	4.719	655	WS0045_E01
9	0.000	166	1	0.175	0.058	4.954	655	WS0045_E01
12	6.242	234	2	-0.266	0.056	5.662	655	WS0045_E01
14	43.392	250	1	-0.314	0.072	3.851	655	WS0045_E01
14	46.637	252	4	-0.339	0.054	5.024	655	WS0045_E01
1	62.128	11	1	-0.489	0.080	4.877	656	WS0045_E24
2	120.617	38	4	-0.272	0.029	4.383	656	WS0045_E24
3	148.745	68	4	-0.251	0.048	6.822	656	WS0045_E24
3	154.505	69	4	0.243	0.051	6.285	656	WS0045_E24
4	116.928	82	4	0.227	0.037	4.383	656	WS0045_E24
6	69.073	121	3	0.313	0.088	5.803	656	WS0045_E24
8	50.001	154	1	0.223	0.059	5.321	656	WS0045_E24
9	0.000	166	1	0.200	0.075	7.188	656	WS0045_E24
10	21.864	188	1	-0.336	0.059	5.779	656	WS0045_E24
11	17.088	203	1	0.361	0.099	9.522	656	WS0045_E24
11	29.069	205	1	-0.365	0.085	8.331	656	WS0045_E24
12	28.493	228	2	0.238	0.081	4.944	656	WS0045_E24
12	22.629	237	1	0.260	0.053	5.133	656	WS0045_E24
12	32.473	238	1	0.243	0.044	4.249	656	WS0045_E24
1	40.464	7	3	0.294	0.049	5.858	657	WS0045_I16
1	43.578	8	3	0.302	0.054	6.010	657	WS0045_I16
4	55.925	76	1	-0.255	0.045	4.108	657	WS0045_I16
4	140.962	84	3	-0.265	0.053	6.348	657	WS0045_I16
4	173.929	88	2	-0.156	0.043	3.868	657	WS0045_I16
11	0.000	199	3	-0.234	0.043	5.235	657	WS0045_I16
11	27.405	204	1	0.310	0.083	8.427	657	WS0045_I16
2	120.617	38	4	-0.275	0.066	4.087	658	WS0045_K01
2	126.181	39	2	-0.248	0.047	4.542	658	WS0045_K01
2	127.147	40	4	-0.315	0.086	6.172	658	WS0045_K01
3	137.891	64	3	0.246	0.054	3.956	658	WS0045_K01
4	142.917	85	1	-0.219	0.047	4.330	658	WS0045_K01
6	95.249	126	1	0.301	0.103	4.022	658	WS0045_K01
9	55.358	175	1	0.334	0.055	5.227	658	WS0045_K01
11	45.989	216	1	-0.287	0.071	6.624	658	WS0045_K01
12	22.441	227	3	0.289	0.052	5.191	658	WS0045_K01
13	0.000	243	4	0.442	0.093	5.111	658	WS0045_K01
13	3.386	245	1	-0.151	0.044	4.108	658	WS0045_K01
3	69.071	52	1	-0.378	0.105	6.108	659	WS0045_O11

4	173.929	88	2	-0.281	0.082	7.760	659	WS0045_O11
6	69.073	121	3	0.226	0.042	4.226	659	WS0045_O11
11	45.989	216	1	-0.282	0.073	6.789	659	WS0045_O11
11	57.661	220	4	-0.245	0.054	4.809	659	WS0045_O11
11	64.014	222	4	0.303	0.052	4.496	659	WS0045_O11
12	22.441	227	3	0.252	0.042	4.079	659	WS0045_O11
14	22.402	248	3	0.322	0.051	5.244	659	WS0045_O11
14	43.392	250	1	-0.373	0.103	6.019	659	WS0045_O11
14	44.898	251	3	0.256	0.045	4.472	659	WS0045_O11
2	95.880	34	1	-0.346	0.082	7.024	660	WS0045_P07
3	15.147	44	2	0.327	0.068	5.297	660	WS0045_P07
3	20.512	46	2	0.172	0.047	4.760	660	WS0045_P07
5	3.484	90	2	-0.328	0.080	5.272	660	WS0045_P07
5	84.347	105	1	-0.232	0.051	4.381	660	WS0045_P07
9	36.227	165	4	-0.193	0.027	3.971	660	WS0045_P07
9	0.000	166	1	0.237	0.045	3.916	660	WS0045_P07
3	104.681	60	2	0.203	0.037	4.022	661	WS0084_D01
3	114.747	62	2	-0.192	0.037	3.967	661	WS0084_D01
4	55.925	76	1	-0.267	0.076	5.527	661	WS0084_D01
4	82.462	78	1	-0.246	0.090	7.172	661	WS0084_D01
5	31.191	95	1	-0.234	0.068	5.416	661	WS0084_D01
6	69.073	121	3	0.266	0.052	4.993	661	WS0084_D01
8	62.580	155	1	0.393	0.163	4.391	661	WS0084_D01
9	36.227	165	3	-0.217	0.055	5.313	661	WS0084_D01
9	69.046	181	2	0.270	0.098	4.279	661	WS0084_D01
10	70.246	190	2	-0.229	0.038	4.173	661	WS0084_D01
14	22.402	248	3	0.282	0.046	4.501	661	WS0084_D01
14	43.392	250	1	-0.419	0.149	7.973	661	WS0084_D01
14	44.898	251	3	0.289	0.066	6.074	661	WS0084_D01
3	9.953	42	3	0.243	0.047	4.417	662	WS0084_J11
4	0.000	71	4	0.265	0.052	4.974	662	WS0084_J11
4	173.929	88	2	-0.229	0.057	5.980	662	WS0084_J11
8	87.463	158	2	-0.231	0.049	5.176	662	WS0084_J11
9	81.073	183	1	0.252	0.047	3.965	662	WS0084_J11
10	70.246	190	2	-0.316	0.096	10.286	662	WS0084_J11
11	27.405	204	1	0.222	0.052	4.568	662	WS0084_J11
11	45.989	216	1	-0.260	0.082	6.811	662	WS0084_J11
14	22.402	248	3	0.275	0.052	4.869	662	WS0084_J11
1	2.788	2	4	0.221	0.056	5.742	663	WS01015_K11
1	22.419	4	3	0.363	0.122	5.757	663	WS01015_K11
1	29.800	5	2	-0.213	0.056	5.741	663	WS01015_K11
1	107.333	19	3	-0.287	0.076	7.517	663	WS01015_K11
2	48.839	29	4	-0.311	0.066	5.809	663	WS01015_K11
2	126.181	39	2	0.306	0.051	5.655	663	WS01015_K11
2	126.181	39	4	-0.473	0.109	7.134	663	WS01015_K11
3	80.211	55	4	0.247	0.050	4.544	663	WS01015_K11
4	82.462	78	2	-0.194	0.062	6.420	663	WS01015_K11
4	97.576	80	2	0.298	0.078	10.673	663	WS01015_K11
6	81.996	123	3	-0.331	0.106	5.016	663	WS01015_K11
6	106.702	128	2	-0.514	0.130	7.167	663	WS01015_K11
11	0.000	199	3	0.209	0.046	4.578	663	WS01015_K11
11	27.405	204	1	-0.220	0.051	3.917	663	WS01015_K11
11	45.989	216	1	0.240	0.057	4.412	663	WS01015_K11
1	15.589	3	2	0.268	0.059	7.106	664	WS01015_O11

1	29.800	5	2	-0.188	0.033	4.018	664	WS01015_O11
1	43.578	8	2	-0.208	0.035	4.233	664	WS01015_O11
1	71.592	14	2	0.222	0.039	4.742	664	WS01015_O11
1	77.177	15	2	0.269	0.062	7.433	664	WS01015_O11
1	95.572	17	3	0.302	0.091	7.892	664	WS01015_O11
3	15.147	44	2	0.342	0.100	7.009	664	WS01015_O11
5	56.008	101	1	-0.219	0.061	4.139	664	WS01015_O11
5	57.017	102	2	-0.242	0.039	4.710	664	WS01015_O11
6	25.742	113	3	-0.215	0.051	4.435	664	WS01015_O11
8	50.001	154	2	-0.232	0.041	4.902	664	WS01015_O11
11	46.026	217	1	-0.216	0.079	5.339	664	WS01015_O11
1	15.589	3	2	0.168	0.034	3.905	665	IS0011_C01
1	29.800	5	2	-0.270	0.041	4.677	665	IS0011_C01
2	48.839	29	1	0.288	0.082	5.954	665	IS0011_C01
5	57.017	102	4	0.226	0.053	4.284	665	IS0011_C01
9	17.951	169	1	-0.292	0.054	3.919	665	IS0011_C01
9	59.963	177	4	-0.257	0.049	4.631	665	IS0011_C01
9	69.046	181	2	-0.337	0.113	5.404	665	IS0011_C01
11	27.405	204	1	-0.249	0.080	5.879	665	IS0011_C01
11	29.069	205	1	-0.173	0.057	4.114	665	IS0011_C01
11	40.960	210	4	0.306	0.050	4.685	665	IS0011_C01
11	43.491	214	3	0.181	0.046	4.310	665	IS0011_C01
12	6.242	234	2	0.241	0.038	4.873	665	IS0011_C01
3	14.500	43	1	-0.226	0.050	4.172	666	IS0011_O06
3	14.500	43	2	-0.196	0.038	3.874	666	IS0011_O06
5	86.669	106	4	-0.388	0.123	4.134	666	IS0011_O06
7	67.154	140	4	-0.291	0.105	3.875	666	IS0011_O06
7	75.883	144	4	-0.437	0.130	9.228	666	IS0011_O06
8	39.090	152	4	-0.246	0.058	4.703	666	IS0011_O06
9	2.011	167	4	0.298	0.093	7.529	666	IS0011_O06
14	0.000	246	1	0.206	0.047	3.966	666	IS0011_O06
1	15.589	3	2	0.184	0.038	3.914	667	WS0043_L07
2	63.261	30	3	-0.217	0.045	4.167	667	WS0043_L07
2	75.550	31	2	-0.280	0.051	5.260	667	WS0043_L07
2	101.076	35	4	-0.320	0.090	6.736	667	WS0043_L07
2	102.814	36	2	0.327	0.057	5.654	667	WS0043_L07
2	126.181	39	2	-0.277	0.068	7.101	667	WS0043_L07
3	15.147	44	2	-0.353	0.118	6.902	667	WS0043_L07
3	114.747	62	2	0.212	0.038	3.879	667	WS0043_L07
3	116.958	63	4	0.219	0.053	5.034	667	WS0043_L07
5	42.979	97	1	0.307	0.050	4.292	667	WS0043_L07
8	50.001	154	2	-0.188	0.038	3.844	667	WS0043_L07
10	21.864	188	1	-0.261	0.052	4.219	667	WS0043_L07
13	0.000	243	4	-0.342	0.122	3.952	667	WS0043_L07
1	77.177	15	2	0.183	0.036	4.988	668	WS00927_I10
1	123.316	20	4	-0.202	0.034	3.878	668	WS00927_I10
2	126.181	39	4	0.461	0.058	6.735	668	WS00927_I10
3	80.211	55	4	-0.221	0.031	4.170	668	WS00927_I10
4	103.719	81	4	-0.206	0.047	6.602	668	WS00927_I10
4	116.928	82	4	0.368	0.052	7.241	668	WS00927_I10
5	42.979	97	2	-0.193	0.031	4.223	668	WS00927_I10
5	54.072	100	4	0.219	0.034	4.567	668	WS00927_I10
6	54.066	119	4	0.190	0.038	5.098	668	WS00927_I10
6	69.073	121	4	-0.224	0.033	4.240	668	WS00927_I10

7	105.477	147	2	-0.308	0.067	7.669	668	WS00927_I10
9	59.963	177	4	0.272	0.054	6.199	668	WS00927_I10
11	43.491	214	4	-0.206	0.043	4.439	668	WS00927_I10
12	6.242	234	2	-0.196	0.031	4.426	668	WS00927_I10
14	43.392	250	2	0.201	0.030	4.279	668	WS00927_I10
2	93.504	32	2	-0.230	0.040	5.315	669	WS0044_E02
6	25.742	113	3	-0.244	0.050	4.867	669	WS0044_E02
13	1.815	244	2	-0.270	0.060	8.154	669	WS0044_E02
14	43.392	250	2	-0.186	0.032	4.346	669	WS0044_E02
1	45.794	9	1	-0.274	0.065	7.073	670	WS00927_L14
2	93.504	32	4	-0.195	0.045	4.382	670	WS00927_L14
3	14.500	43	1	-0.247	0.041	4.435	670	WS00927_L14
3	17.158	45	1	0.193	0.048	5.199	670	WS00927_L14
3	137.891	64	1	0.238	0.048	5.253	670	WS00927_L14
3	137.891	64	3	-0.226	0.044	4.004	670	WS00927_L14
8	50.001	154	2	-0.143	0.049	3.953	670	WS00927_L14
11	29.069	205	1	0.238	0.039	4.248	670	WS00927_L14
11	64.014	222	3	-0.221	0.044	4.096	670	WS00927_L14
12	22.441	227	1	-0.228	0.048	5.287	670	WS00927_L14
14	0.000	246	1	0.256	0.075	8.155	670	WS00927_L14
2	93.504	32	4	0.239	0.044	4.478	671	WS00910_O10
3	47.928	49	1	0.368	0.095	9.900	671	WS00910_O10
3	69.071	52	1	0.315	0.094	4.831	671	WS00910_O10
4	92.039	79	4	0.307	0.040	3.899	671	WS00910_O10
4	124.132	83	1	-0.715	0.205	10.728	671	WS00910_O10
4	142.917	85	1	0.280	0.060	6.319	671	WS00910_O10
5	3.484	90	2	0.295	0.090	4.046	671	WS00910_O10
6	49.100	118	4	-0.419	0.109	5.254	671	WS00910_O10
6	81.996	123	3	-0.307	0.088	4.483	671	WS00910_O10
8	39.090	152	4	-0.239	0.056	5.429	671	WS00910_O10
9	49.013	173	1	-0.221	0.061	7.114	671	WS00910_O10
11	43.491	214	3	0.211	0.049	4.440	671	WS00910_O10
11	45.989	216	1	0.343	0.085	8.791	671	WS00910_O10
12	6.242	234	2	0.230	0.042	3.912	671	WS00910_O10
1	43.578	8	2	0.348	0.088	8.655	672	WS00910_P09
1	69.854	13	2	-0.172	0.040	3.850	672	WS00910_P09
1	71.592	14	2	-0.177	0.042	4.104	672	WS00910_P09
1	95.572	17	2	-0.228	0.043	4.174	672	WS00910_P09
3	144.647	67	1	-0.366	0.108	4.387	672	WS00910_P09
5	3.484	90	2	0.253	0.056	4.028	672	WS00910_P09
5	31.191	95	1	-0.219	0.043	3.891	672	WS00910_P09
5	54.072	100	4	0.254	0.059	6.652	672	WS00910_P09
5	57.017	102	4	-0.286	0.056	4.722	672	WS00910_P09
11	40.960	210	2	0.388	0.060	4.558	672	WS00910_P09
11	60.464	221	1	0.269	0.044	4.013	672	WS00910_P09
12	6.242	234	2	-0.249	0.048	4.885	672	WS00910_P09
12	6.242	234	4	0.290	0.038	4.558	672	WS00910_P09
2	126.181	39	4	-0.517	0.148	6.673	673	WS00911_A23
3	14.500	43	1	0.336	0.078	6.816	673	WS00911_A23
3	17.158	45	1	-0.212	0.059	5.139	673	WS00911_A23
5	84.347	105	2	0.164	0.043	4.373	673	WS00911_A23
6	25.742	113	3	-0.223	0.040	4.701	673	WS00911_A23
6	69.073	121	3	0.276	0.038	4.498	673	WS00911_A23
9	32.356	171	2	-0.243	0.056	5.617	673	WS00911_A23

11	64.014	222	3	0.280	0.039	4.730	673	WS00911_A23
12	22.441	227	1	-0.297	0.054	4.872	673	WS00911_A23
12	22.441	227	3	0.216	0.036	4.336	673	WS00911_A23
9	28.608	170	2	-0.283	0.050	5.926	674	WS00911_B16
11	27.405	204	1	0.263	0.055	3.957	674	WS00911_B16
11	37.143	209	3	0.196	0.045	4.495	674	WS00911_B16
12	14.406	230	3	-0.238	0.040	4.000	674	WS00911_B16
13	1.815	244	4	-0.266	0.046	4.092	674	WS00911_B16
1	2.788	2	4	-0.241	0.106	7.878	675	WS00911_D11
1	40.464	7	3	0.194	0.038	4.101	675	WS00911_D11
1	43.578	8	3	0.171	0.029	3.861	675	WS00911_D11
4	55.925	76	1	-0.290	0.062	5.369	675	WS00911_D11
5	52.820	99	1	0.249	0.063	5.948	675	WS00911_D11
6	89.401	125	1	-0.235	0.044	4.088	675	WS00911_D11
11	0.000	199	3	-0.350	0.069	7.253	675	WS00911_D11
11	27.405	204	1	0.345	0.072	6.687	675	WS00911_D11
11	27.405	204	4	-0.295	0.048	4.141	675	WS00911_D11
11	45.989	216	1	-0.167	0.045	4.104	675	WS00911_D11
1	127.805	21	3	-0.241	0.060	4.747	676	WS00911_D24
2	75.550	31	1	0.246	0.052	3.906	676	WS00911_D24
3	9.953	42	4	0.204	0.054	5.938	676	WS00911_D24
4	82.462	78	2	-0.301	0.073	8.385	676	WS00911_D24
4	116.928	82	4	0.262	0.043	4.215	676	WS00911_D24
5	42.979	97	2	-0.185	0.041	4.468	676	WS00911_D24
6	87.891	124	2	-0.235	0.054	6.087	676	WS00911_D24
6	89.401	125	2	-0.388	0.098	9.590	676	WS00911_D24
7	74.560	143	1	0.241	0.058	4.372	676	WS00911_D24
8	62.580	155	1	0.518	0.224	8.302	676	WS00911_D24
10	9.580	187	3	0.306	0.106	3.849	676	WS00911_D24
10	80.088	194	1	0.228	0.057	4.246	676	WS00911_D24
11	43.491	214	3	-0.303	0.116	9.077	676	WS00911_D24
11	43.491	214	4	-0.211	0.039	4.238	676	WS00911_D24
11	44.884	215	3	0.296	0.102	7.998	676	WS00911_D24
11	64.014	222	3	0.294	0.092	7.241	676	WS00911_D24
11	64.014	222	4	-0.256	0.036	3.900	676	WS00911_D24
12	6.242	234	4	0.285	0.046	4.506	676	WS00911_D24
1	45.794	9	1	-0.259	0.045	4.540	677	WS00911_G12
2	120.617	38	4	-0.360	0.086	7.254	677	WS00911_G12
3	80.211	55	4	0.219	0.048	4.409	677	WS00911_G12
4	0.000	71	4	-0.222	0.043	3.913	677	WS00911_G12
4	124.132	83	1	0.749	0.223	11.341	677	WS00911_G12
4	142.917	85	1	-0.329	0.046	4.753	677	WS00911_G12
6	49.100	118	4	0.393	0.131	6.528	677	WS00911_G12
6	69.073	121	3	0.272	0.064	6.185	677	WS00911_G12
8	50.001	154	2	-0.188	0.053	4.614	677	WS00911_G12
12	22.441	227	1	-0.272	0.045	4.506	677	WS00911_G12
12	22.441	227	3	0.257	0.057	5.652	677	WS00911_G12
14	44.898	251	3	0.247	0.045	4.263	677	WS00911_G12
1	71.592	14	2	0.205	0.033	3.878	678	WS00911_H04
4	82.462	78	2	0.222	0.051	6.406	678	WS00911_H04
4	173.929	88	2	-0.245	0.074	8.666	678	WS00911_H04
5	0.000	89	1	-0.348	0.087	6.966	678	WS00911_H04
5	0.000	89	3	0.311	0.065	6.097	678	WS00911_H04
5	3.484	90	2	-0.231	0.062	4.440	678	WS00911_H04

6	81.996	123	3	0.294	0.081	3.963	678	WS00911_H04
7	15.555	130	2	0.169	0.035	4.161	678	WS00911_H04
10	70.246	190	2	-0.209	0.032	3.853	678	WS00911_H04
1	95.572	17	2	-0.281	0.044	4.596	679	WS0086_K04
5	0.000	89	1	-0.243	0.062	5.618	679	WS0086_K04
6	25.914	114	1	-0.265	0.063	5.419	679	WS0086_K04
9	55.358	175	3	0.531	0.124	6.118	679	WS0086_K04
10	78.479	193	4	-0.225	0.052	4.127	679	WS0086_K04
12	22.441	227	1	0.308	0.062	5.406	679	WS0086_K04
12	14.406	230	3	-0.218	0.038	4.234	679	WS0086_K04
14	0.000	246	1	0.270	0.066	5.637	679	WS0086_K04
2	127.147	40	3	-0.182	0.052	5.356	680	WS0086_K24
3	83.563	56	3	-0.259	0.085	8.772	680	WS0086_K24
4	124.132	83	1	0.501	0.108	5.457	680	WS0086_K24
4	140.962	84	3	-0.183	0.038	3.882	680	WS0086_K24
4	173.929	88	2	-0.204	0.063	4.166	680	WS0086_K24
7	74.560	143	1	0.376	0.063	7.771	680	WS0086_K24
9	81.073	183	3	-0.263	0.044	4.613	680	WS0086_K24
11	27.405	204	1	0.329	0.053	6.518	680	WS0086_K24
11	29.069	205	1	0.296	0.050	6.177	680	WS0086_K24
11	45.989	216	1	-0.318	0.079	9.596	680	WS0086_K24
14	0.000	246	1	0.271	0.056	6.863	680	WS0086_K24
4	55.925	76	1	-0.273	0.074	5.940	681	WS0086_N10
5	52.820	99	1	0.280	0.056	4.426	681	WS0086_N10
5	84.347	105	1	-0.253	0.070	5.503	681	WS0086_N10
6	26.350	115	2	0.226	0.047	5.195	681	WS0086_N10
7	17.610	131	3	0.247	0.104	4.440	681	WS0086_N10
9	59.963	177	4	0.345	0.061	5.687	681	WS0086_N10
10	95.795	197	4	-0.188	0.038	3.890	681	WS0086_N10
11	9.237	202	4	0.244	0.046	4.763	681	WS0086_N10
11	27.405	204	4	-0.237	0.059	4.833	681	WS0086_N10
11	45.989	216	1	-0.232	0.067	5.223	681	WS0086_N10
14	22.402	248	3	0.295	0.087	7.427	681	WS0086_N10
1	71.592	14	2	0.206	0.045	4.531	682	WS0086_O03
1	77.177	15	2	0.192	0.038	3.841	682	WS0086_O03
4	82.462	78	1	-0.211	0.058	4.980	682	WS0086_O03
9	59.963	177	1	-0.246	0.046	3.988	682	WS0086_O03
9	81.073	183	3	-0.327	0.085	10.370	682	WS0086_O03
10	35.204	189	1	0.236	0.053	4.567	682	WS0086_O03
11	27.405	204	1	0.223	0.050	4.355	682	WS0086_O03
11	40.960	210	2	0.470	0.130	6.133	682	WS0086_O03
12	0.000	233	1	0.204	0.055	4.735	682	WS0086_O03
1	82.301	16	4	-0.293	0.057	5.317	683	WS0087_A10
4	124.132	83	1	-0.548	0.137	6.105	683	WS0087_A10
4	142.917	85	1	0.357	0.118	9.598	683	WS0087_A10
4	173.929	88	2	-0.181	0.052	5.562	683	WS0087_A10
5	0.000	89	1	-0.289	0.060	4.798	683	WS0087_A10
5	54.072	100	4	0.203	0.037	3.919	683	WS0087_A10
5	86.669	106	4	0.468	0.146	6.519	683	WS0087_A10
9	2.011	167	4	-0.373	0.095	10.243	683	WS0087_A10
9	3.127	168	1	0.551	0.213	9.308	683	WS0087_A10
9	49.013	173	2	0.270	0.087	8.858	683	WS0087_A10
9	55.358	175	1	-0.288	0.052	4.224	683	WS0087_A10
11	0.000	199	3	-0.299	0.066	5.324	683	WS0087_A10

14	22.402	248	3	0.329	0.112	9.030	683	WS0087_A10
1	82.301	16	4	-0.237	0.059	5.087	684	WS0087_B09
2	102.814	36	1	0.304	0.085	10.347	684	WS0087_B09
5	31.191	95	1	-0.378	0.076	9.285	684	WS0087_B09
6	56.188	120	1	-0.340	0.052	6.359	684	WS0087_B09
6	69.073	121	4	-0.190	0.064	4.921	684	WS0087_B09
6	89.401	125	1	-0.207	0.041	4.938	684	WS0087_B09
6	95.249	126	1	0.332	0.047	4.006	684	WS0087_B09
9	0.000	163	1	-0.404	0.046	3.855	684	WS0087_B09
9	28.608	170	3	0.435	0.092	8.744	684	WS0087_B09
9	55.358	175	3	0.522	0.096	5.467	684	WS0087_B09
10	80.088	194	1	-0.199	0.052	6.215	684	WS0087_B09
3	83.563	56	3	-0.251	0.070	6.921	685	WS0087_E06
3	116.958	63	4	-0.248	0.045	4.047	685	WS0087_E06
4	92.039	79	2	0.175	0.042	3.986	685	WS0087_E06
5	57.017	102	2	0.238	0.050	4.629	685	WS0087_E06
6	81.996	123	4	-0.362	0.133	5.398	685	WS0087_E06
9	69.046	181	2	0.338	0.129	7.428	685	WS0087_E06
10	70.246	190	4	-0.279	0.044	3.929	685	WS0087_E06
10	78.479	193	4	-0.298	0.070	5.888	685	WS0087_E06
10	95.795	197	4	0.192	0.045	4.055	685	WS0087_E06
3	83.563	56	3	-0.224	0.042	5.112	686	WS0087_E15
5	31.191	95	1	-0.197	0.043	3.891	686	WS0087_E15
5	56.008	101	1	-0.208	0.050	4.509	686	WS0087_E15
6	81.996	123	3	0.316	0.107	6.481	686	WS0087_E15
6	106.702	128	3	-0.244	0.036	4.114	686	WS0087_E15
7	70.278	141	3	-0.242	0.056	6.896	686	WS0087_E15
7	74.560	143	1	0.250	0.044	3.970	686	WS0087_E15
8	87.463	158	2	-0.227	0.056	5.142	686	WS0087_E15
10	35.204	189	1	0.227	0.049	4.396	686	WS0087_E15
12	38.543	232	4	0.334	0.178	5.269	686	WS0087_E15
14	22.402	248	4	0.293	0.063	4.005	686	WS0087_E15
1	62.128	11	1	0.575	0.139	6.447	687	WS0085_N19
1	62.128	11	2	0.372	0.120	4.766	687	WS0085_N19
1	107.333	19	3	-0.163	0.049	4.152	687	WS0085_N19
1	127.805	21	3	-0.392	0.180	15.382	687	WS0085_N19
1	133.992	24	2	0.534	0.325	31.413	687	WS0085_N19
1	141.021	25	2	-0.427	0.226	21.791	687	WS0085_N19
2	102.814	36	2	0.246	0.053	4.186	687	WS0085_N19
2	126.181	39	2	-0.301	0.061	6.056	687	WS0085_N19
4	142.917	85	1	-0.218	0.062	5.761	687	WS0085_N19
4	142.917	85	3	0.276	0.079	6.709	687	WS0085_N19
9	49.013	173	2	-0.211	0.065	6.198	687	WS0085_N19
11	64.014	222	4	0.160	0.040	4.144	687	WS0085_N19
14	0.000	246	1	0.165	0.043	3.869	687	WS0085_N19
2	63.261	30	3	0.234	0.054	5.414	688	WS0086_F12
4	173.929	88	2	-0.256	0.064	7.538	688	WS0086_F12
9	36.227	165	3	-0.251	0.054	5.325	688	WS0086_F12
10	70.246	190	2	-0.237	0.033	3.884	688	WS0086_F12
10	78.479	193	3	0.356	0.063	6.278	688	WS0086_F12
11	27.405	204	1	0.272	0.073	5.221	688	WS0086_F12
11	27.405	204	4	-0.214	0.046	4.025	688	WS0086_F12
11	41.984	212	2	-0.219	0.046	5.462	688	WS0086_F12
11	45.989	216	1	-0.212	0.063	4.412	688	WS0086_F12

11	45.989	216	2	-0.228	0.048	5.680	688	WS0086_F12
12	6.242	234	2	-0.255	0.032	4.542	688	WS0086_F12
4	173.929	88	2	-0.261	0.086	9.109	689	IS0014_L02
14	44.898	251	3	0.190	0.035	4.126	689	IS0014_L02
1	95.572	17	2	-0.297	0.054	5.569	690	WS0041_D05
1	95.572	17	3	0.473	0.139	17.614	690	WS0041_D05
1	107.333	19	3	0.161	0.034	4.183	690	WS0041_D05
3	47.928	49	1	0.238	0.055	4.764	690	WS0041_D05
7	70.278	141	3	0.234	0.041	5.032	690	WS0041_D05
12	6.242	234	2	0.189	0.046	5.909	690	WS0041_D05
1	15.589	3	2	-0.266	0.071	8.421	691	WS00926_D10
4	173.929	88	2	-0.269	0.067	7.885	691	WS00926_D10
11	27.405	204	1	0.212	0.058	4.014	691	WS00926_D10
11	40.960	210	2	0.375	0.046	4.037	691	WS00926_D10
1	2.788	2	4	-0.251	0.052	4.411	692	WS00926_D22
1	82.301	16	4	0.230	0.046	4.180	692	WS00926_D22
2	48.839	29	4	0.235	0.045	4.022	692	WS00926_D22
2	63.261	30	3	0.225	0.045	4.370	692	WS00926_D22
3	47.928	49	1	-0.237	0.038	4.114	692	WS00926_D22
3	69.071	52	1	-0.279	0.061	4.122	692	WS00926_D22
5	84.347	105	1	-0.253	0.042	4.648	692	WS00926_D22
9	3.127	168	1	-0.416	0.136	5.609	692	WS00926_D22
9	59.963	177	4	0.241	0.046	4.215	692	WS00926_D22
10	70.246	190	2	-0.198	0.049	3.866	692	WS00926_D22
11	27.405	204	1	0.269	0.063	6.955	692	WS00926_D22
11	29.069	205	1	0.227	0.049	5.279	692	WS00926_D22
11	45.989	216	1	-0.320	0.081	8.854	692	WS00926_D22
1	95.572	17	3	-0.239	0.042	4.003	693	WS00926_F07
3	9.953	42	3	0.251	0.054	6.004	693	WS00926_F07
3	137.891	64	3	0.267	0.041	4.701	693	WS00926_F07
4	0.000	71	4	0.257	0.083	6.385	693	WS00926_F07
4	140.962	84	3	-0.229	0.044	4.918	693	WS00926_F07
4	142.917	85	3	-0.328	0.076	7.012	693	WS00926_F07
9	55.358	175	1	-0.259	0.052	4.506	693	WS00926_F07
10	78.479	193	3	0.295	0.051	5.600	693	WS00926_F07
11	27.405	204	1	0.223	0.054	4.718	693	WS00926_F07
11	29.069	205	1	0.192	0.047	4.010	693	WS00926_F07
11	45.989	216	1	-0.214	0.045	3.864	693	WS00926_F07
14	43.392	250	1	-0.300	0.092	4.848	693	WS00926_F07
2	48.839	29	4	0.203	0.050	3.980	694	WS00926_F19
3	144.593	66	4	0.164	0.055	5.768	694	WS00926_F19
3	148.745	68	4	-0.240	0.064	6.669	694	WS00926_F19
3	154.505	69	4	0.189	0.045	4.203	694	WS00926_F19
4	82.462	78	1	-0.133	0.057	4.523	694	WS00926_F19
4	103.719	81	4	-0.308	0.054	6.178	694	WS00926_F19
4	116.928	82	4	0.353	0.099	9.958	694	WS00926_F19
6	49.100	118	4	0.236	0.105	4.138	694	WS00926_F19
7	62.787	139	2	0.273	0.053	4.947	694	WS00926_F19
8	0.000	148	4	-0.267	0.040	4.546	694	WS00926_F19
13	0.000	243	4	0.254	0.111	4.344	694	WS00926_F19
5	16.219	93	2	-0.306	0.061	5.794	695	WS00926_H07
12	14.406	230	3	-0.257	0.051	5.088	695	WS00926_H07
14	9.923	247	1	-0.241	0.050	4.138	695	WS00926_H07
14	44.898	251	1	-0.226	0.067	5.575	695	WS00926_H07

14	46.637	252	3	-0.288	0.049	4.935	695	WS00926_H07
3	69.071	52	1	-0.328	0.072	4.584	696	WS00926_H19
3	137.891	64	3	0.334	0.063	6.767	696	WS00926_H19
4	0.000	71	4	0.202	0.058	4.715	696	WS00926_H19
4	173.929	88	2	-0.249	0.058	5.972	696	WS00926_H19
9	0.000	163	1	0.486	0.082	5.200	696	WS00926_H19
10	35.204	189	1	0.331	0.075	6.439	696	WS00926_H19
11	27.405	204	1	0.314	0.109	9.509	696	WS00926_H19
11	27.405	204	4	-0.201	0.056	4.404	696	WS00926_H19
11	29.069	205	1	0.190	0.060	5.137	696	WS00926_H19
11	45.989	216	1	-0.335	0.109	9.395	696	WS00926_H19
12	6.242	234	2	-0.272	0.041	5.116	696	WS00926_H19
3	9.953	42	3	0.255	0.072	6.526	697	WS00926_J02
4	173.929	88	2	-0.267	0.081	9.011	697	WS00926_J02
5	54.072	100	4	0.219	0.048	4.611	697	WS00926_J02
11	27.405	204	1	0.228	0.049	3.906	697	WS00926_J02
11	27.405	204	4	-0.326	0.071	6.848	697	WS00926_J02
11	45.989	216	1	-0.188	0.054	4.156	697	WS00926_J02
3	9.953	42	3	0.243	0.063	6.668	698	WS00926_J08
3	137.891	64	3	0.394	0.113	10.590	698	WS00926_J08
5	7.672	92	4	-0.247	0.055	4.280	698	WS00926_J08
6	87.891	124	2	-0.204	0.042	5.024	698	WS00926_J08
6	89.401	125	2	-0.218	0.036	4.204	698	WS00926_J08
6	95.249	126	1	0.310	0.102	4.167	698	WS00926_J08
11	9.237	202	4	0.190	0.053	4.336	698	WS00926_J08
11	27.405	204	4	-0.338	0.101	7.531	698	WS00926_J08
12	14.406	230	3	-0.259	0.042	4.510	698	WS00926_J08
14	44.898	251	3	0.278	0.058	4.265	698	WS00926_J08
3	137.891	64	3	0.334	0.068	3.949	699	WS01028_H06
4	82.462	78	1	-0.212	0.063	4.609	699	WS01028_H06
4	140.962	84	3	-0.198	0.049	4.054	699	WS01028_H06
4	173.929	88	2	-0.230	0.050	5.769	699	WS01028_H06
7	15.555	130	3	-0.267	0.053	4.271	699	WS01028_H06
9	59.963	177	4	0.254	0.039	4.128	699	WS01028_H06
11	27.405	204	4	-0.255	0.057	5.432	699	WS01028_H06
11	64.014	222	4	-0.230	0.039	4.165	699	WS01028_H06
1	2.788	2	4	-0.179	0.048	4.627	700	WS01028_H13
2	75.550	31	2	-0.201	0.040	3.979	700	WS01028_H13
5	31.191	95	1	-0.256	0.057	4.723	700	WS01028_H13
5	54.072	100	4	0.225	0.043	4.254	700	WS01028_H13
9	36.227	165	3	-0.258	0.052	4.685	700	WS01028_H13
10	70.246	190	2	-0.205	0.037	4.016	700	WS01028_H13
11	37.143	209	3	0.213	0.066	5.892	700	WS01028_H13
11	42.600	213	3	-0.190	0.050	4.472	700	WS01028_H13
12	6.242	234	2	-0.273	0.052	6.269	700	WS01028_H13
4	6.032	72	4	0.178	0.041	4.201	701	WS01028_I21
4	82.462	78	1	-0.192	0.050	3.848	701	WS01028_I21
4	173.929	88	2	-0.255	0.063	7.003	701	WS01028_I21
6	81.996	123	3	0.310	0.106	4.387	701	WS01028_I21
10	78.479	193	3	0.368	0.068	5.855	701	WS01028_I21
11	9.237	202	4	0.257	0.039	4.097	701	WS01028_I21
11	27.405	204	4	-0.255	0.067	6.351	701	WS01028_I21
6	81.996	123	3	0.332	0.083	5.255	702	WS01028_J10
6	87.891	124	2	-0.234	0.048	5.250	702	WS01028_J10

8	95.937	159	3	-0.220	0.028	3.892	702	WS01028_J10
3	137.891	64	3	0.304	0.073	4.086	703	WS01028_L07
4	140.962	84	3	-0.165	0.040	3.864	703	WS01028_L07
4	142.917	85	3	-0.260	0.063	6.360	703	WS01028_L07
4	173.929	88	2	-0.230	0.070	6.714	703	WS01028_L07
6	81.996	123	3	0.258	0.096	5.314	703	WS01028_L07
10	78.479	193	3	0.307	0.050	4.840	703	WS01028_L07
3	112.868	61	2	-0.200	0.058	4.632	704	WS01028_L20
5	16.219	93	1	-0.260	0.044	4.777	704	WS01028_L20
5	31.191	95	1	-0.178	0.046	4.931	704	WS01028_L20
5	54.072	100	4	0.209	0.063	4.808	704	WS01028_L20
6	25.742	113	3	-0.208	0.042	4.669	704	WS01028_L20
6	81.996	123	3	0.328	0.053	4.205	704	WS01028_L20
9	36.227	165	3	-0.190	0.036	4.034	704	WS01028_L20
11	37.143	209	3	0.230	0.051	5.725	704	WS01028_L20
14	44.898	251	3	0.271	0.039	4.327	704	WS01028_L20
3	116.958	63	4	-0.240	0.037	4.129	705	WS01028_N13
3	154.505	69	1	0.249	0.047	4.471	705	WS01028_N13
4	55.925	76	1	-0.206	0.041	3.865	705	WS01028_N13
5	54.072	100	4	0.210	0.054	5.978	705	WS01028_N13
5	57.017	102	4	-0.320	0.061	6.739	705	WS01028_N13
6	54.066	119	4	-0.258	0.070	7.670	705	WS01028_N13
6	95.249	126	1	0.305	0.100	5.651	705	WS01028_N13
12	51.382	242	1	0.317	0.131	5.203	705	WS01028_N13
1	22.419	4	3	0.279	0.103	6.646	706	WS01028_O14
3	9.953	42	3	0.274	0.041	4.494	706	WS01028_O14
3	9.953	42	4	0.249	0.052	4.505	706	WS01028_O14
3	137.891	64	3	0.210	0.039	3.890	706	WS01028_O14
4	0.000	71	4	0.274	0.112	9.276	706	WS01028_O14
4	82.462	78	1	-0.175	0.036	3.869	706	WS01028_O14
6	106.702	128	2	-0.489	0.142	6.358	706	WS01028_O14
8	11.974	150	1	-0.165	0.040	4.287	706	WS01028_O14
9	0.000	163	1	0.633	0.139	9.008	706	WS01028_O14
9	28.608	170	3	-0.294	0.052	5.020	706	WS01028_O14
11	35.604	207	3	-0.205	0.038	3.986	706	WS01028_O14
11	37.143	209	3	0.207	0.039	4.081	706	WS01028_O14
12	6.242	234	2	-0.250	0.065	4.979	706	WS01028_O14
12	6.242	234	4	0.226	0.048	4.527	706	WS01028_O14
1	43.578	8	2	0.303	0.102	10.586	707	WS0042_H13
1	69.854	13	2	-0.473	0.203	21.382	707	WS0042_H13
1	71.592	14	2	-0.478	0.211	22.197	707	WS0042_H13
1	77.177	15	2	-0.505	0.235	24.738	707	WS0042_H13
1	95.572	17	2	-0.490	0.137	14.282	707	WS0042_H13
2	95.880	34	3	0.192	0.050	4.794	707	WS0042_H13
3	116.958	63	4	-0.313	0.059	6.271	707	WS0042_H13
10	35.204	189	2	-0.225	0.044	4.424	707	WS0042_H13
11	17.088	203	1	-0.181	0.058	4.811	707	WS0042_H13
11	29.069	205	1	0.263	0.054	4.492	707	WS0042_H13
11	45.989	216	1	-0.273	0.061	5.133	707	WS0042_H13
14	0.000	246	1	0.193	0.062	5.165	707	WS0042_H13
1	95.572	17	3	0.314	0.084	6.751	708	WS0042_I07
2	95.880	34	3	0.169	0.041	3.903	708	WS0042_I07
2	102.814	36	2	-0.207	0.042	3.881	708	WS0042_I07
3	15.147	44	2	-0.263	0.119	6.846	708	WS0042_I07

4	142.917	85	1	-0.246	0.054	4.811	708	WS0042_I07
5	7.672	92	1	-0.222	0.041	4.054	708	WS0042_I07
6	25.914	114	1	-0.162	0.046	4.055	708	WS0042_I07
6	45.897	117	1	0.205	0.042	3.954	708	WS0042_I07
7	75.883	144	4	-0.316	0.045	4.242	708	WS0042_I07
9	55.358	175	1	0.291	0.046	4.150	708	WS0042_I07
10	70.246	190	2	0.235	0.051	5.103	708	WS0042_I07
11	9.237	202	4	0.223	0.036	3.887	708	WS0042_I07
11	64.014	222	4	0.221	0.050	5.347	708	WS0042_I07
12	22.629	237	1	0.263	0.053	4.745	708	WS0042_I07
3	9.953	42	3	0.296	0.071	7.947	709	WS0042_M03
3	17.158	45	1	0.193	0.047	4.299	709	WS0042_M03
4	0.000	71	4	0.168	0.052	4.014	709	WS0042_M03
4	116.928	82	2	0.220	0.047	4.549	709	WS0042_M03
7	73.169	142	1	0.520	0.115	4.328	709	WS0042_M03
11	37.143	209	3	0.201	0.036	4.034	709	WS0042_M03
13	1.815	244	2	0.236	0.041	3.949	709	WS0042_M03
1	40.464	7	3	0.247	0.035	3.898	710	WS0042_M21
1	43.578	8	3	0.284	0.053	5.470	710	WS0042_M21
1	45.794	9	1	-0.259	0.036	4.047	710	WS0042_M21
2	93.504	32	4	-0.341	0.081	5.940	710	WS0042_M21
1	62.128	11	1	-0.561	0.187	5.981	711	WS0043_A20
3	15.147	44	2	-0.403	0.175	9.385	711	WS0043_A20
3	83.563	56	3	-0.261	0.061	5.990	711	WS0043_A20
6	25.914	114	1	-0.273	0.066	5.796	711	WS0043_A20
6	106.702	128	3	-0.240	0.041	3.867	711	WS0043_A20
7	15.555	130	3	-0.369	0.119	11.114	711	WS0043_A20
10	70.246	190	2	0.213	0.039	3.937	711	WS0043_A20
10	80.088	194	2	-0.276	0.044	4.793	711	WS0043_A20
10	93.347	196	2	0.451	0.113	4.171	711	WS0043_A20
11	0.000	199	2	-0.271	0.046	4.630	711	WS0043_A20
1	2.788	2	4	-0.370	0.094	9.222	712	WS0043_B10
1	38.326	6	2	0.213	0.033	4.260	712	WS0043_B10
3	15.147	44	2	0.314	0.053	4.168	712	WS0043_B10
3	80.211	55	4	0.414	0.142	13.991	712	WS0043_B10
4	142.917	85	1	-0.301	0.097	6.079	712	WS0043_B10
8	50.001	154	1	-0.249	0.060	4.163	712	WS0043_B10
8	50.001	154	2	-0.236	0.066	8.247	712	WS0043_B10
12	0.000	226	3	0.203	0.046	4.144	712	WS0043_B10
12	6.242	234	2	0.297	0.053	6.889	712	WS0043_B10
7	15.555	130	3	-0.232	0.045	5.447	713	WS0043_E08
9	28.608	170	3	0.279	0.041	4.170	713	WS0043_E08
10	78.479	193	4	-0.195	0.056	3.879	713	WS0043_E08
10	93.347	196	2	0.530	0.087	6.350	713	WS0043_E08
12	22.441	227	1	0.260	0.065	4.249	713	WS0043_E08
12	22.441	227	3	-0.245	0.033	4.153	713	WS0043_E08
5	42.979	97	1	-0.226	0.048	4.455	714	WS0043_F14
5	52.820	99	3	-0.224	0.037	3.938	714	WS0043_F14
10	78.479	193	3	-0.260	0.043	5.310	714	WS0043_F14
10	86.151	195	3	0.246	0.039	4.792	714	WS0043_F14
10	93.347	196	2	0.539	0.104	6.332	714	WS0043_F14
4	0.000	71	4	0.288	0.064	6.111	715	WS0041_K21
4	173.929	88	2	-0.230	0.059	6.355	715	WS0041_K21
6	45.897	117	1	-0.230	0.049	3.914	715	WS0041_K21

8	87.463	158	2	-0.229	0.045	3.939	715	WS0041_K21
10	70.246	190	2	-0.270	0.056	6.165	715	WS0041_K21
10	78.479	193	3	0.320	0.065	6.055	715	WS0041_K21
11	27.405	204	1	0.353	0.115	9.566	715	WS0041_K21
11	29.069	205	1	0.217	0.063	5.008	715	WS0041_K21
11	45.989	216	1	-0.289	0.107	8.556	715	WS0041_K21
1	22.419	4	3	0.247	0.047	5.218	716	WS0042_C11
3	69.071	52	1	-0.212	0.036	3.932	716	WS0042_C11
6	25.914	114	1	-0.203	0.031	3.990	716	WS0042_C11
7	15.555	130	3	-0.384	0.100	12.326	716	WS0042_C11
8	95.937	159	3	-0.230	0.042	6.065	716	WS0042_C11
11	45.989	216	1	-0.203	0.033	4.213	716	WS0042_C11
3	15.147	44	2	0.240	0.088	4.385	717	WS00816_C04
3	95.157	59	2	-0.258	0.054	6.475	717	WS00816_C04
3	104.681	60	2	0.223	0.064	7.095	717	WS00816_C04
3	112.868	61	2	-0.282	0.085	9.393	717	WS00816_C04
3	114.747	62	2	-0.276	0.069	7.634	717	WS00816_C04
4	97.576	80	2	0.236	0.049	4.669	717	WS00816_C04
4	124.132	83	2	0.256	0.059	4.417	717	WS00816_C04
9	0.000	166	2	0.232	0.050	5.373	717	WS00816_C04
10	93.347	196	1	0.409	0.074	4.029	717	WS00816_C04
11	27.405	204	1	0.333	0.083	6.475	717	WS00816_C04
14	46.637	252	4	-0.264	0.057	4.175	717	WS00816_C04
1	45.794	9	1	-0.228	0.057	4.691	718	WS00816_I12
1	62.128	11	2	0.332	0.092	4.285	718	WS00816_I12
3	9.953	42	4	0.269	0.051	5.310	718	WS00816_I12
3	15.147	44	2	0.344	0.069	4.650	718	WS00816_I12
3	20.512	46	2	0.211	0.057	6.101	718	WS00816_I12
5	52.820	99	3	-0.269	0.043	3.970	718	WS00816_I12
7	75.883	144	2	-0.423	0.073	4.448	718	WS00816_I12
9	17.951	169	1	-0.278	0.048	3.918	718	WS00816_I12
10	78.479	193	3	-0.337	0.053	5.032	718	WS00816_I12
10	86.151	195	3	0.226	0.040	3.850	718	WS00816_I12
14	43.392	250	2	-0.151	0.045	5.250	718	WS00816_I12
14	46.637	252	4	-0.166	0.030	3.908	718	WS00816_I12
3	9.953	42	3	0.240	0.054	4.820	719	WS0262_L01
3	154.505	69	1	0.202	0.048	4.070	719	WS0262_L01
8	87.463	158	2	-0.229	0.057	5.603	719	WS0262_L01
9	59.963	177	4	0.274	0.051	5.017	719	WS0262_L01
10	70.246	190	2	-0.240	0.043	4.417	719	WS0262_L01
3	9.953	42	3	0.306	0.078	7.826	720	WS0262_P09
3	116.958	63	4	-0.186	0.048	4.199	720	WS0262_P09
4	0.000	71	4	0.232	0.045	3.980	720	WS0262_P09
5	54.072	100	4	0.245	0.056	4.945	720	WS0262_P09
5	57.017	102	4	-0.283	0.068	5.085	720	WS0262_P09
6	81.996	123	3	0.334	0.113	7.125	720	WS0262_P09
7	70.278	141	3	-0.225	0.046	4.640	720	WS0262_P09
9	28.608	170	3	0.223	0.041	3.927	720	WS0262_P09
9	55.358	175	3	0.397	0.068	4.151	720	WS0262_P09
1	22.419	4	3	0.287	0.086	4.307	721	WS0038_G06
3	9.953	42	3	0.337	0.109	11.609	721	WS0038_G06
4	6.032	72	4	0.189	0.048	3.865	721	WS0038_G06
5	16.219	93	2	0.283	0.053	4.807	721	WS0038_G06
11	9.237	202	4	0.257	0.056	4.561	721	WS0038_G06

11	29.069	205	1	0.219	0.048	4.498	721	WS0038_G06
3	9.953	42	3	0.171	0.044	5.220	722	WS0038_O13
4	173.929	88	2	-0.174	0.044	4.676	722	WS0038_O13
5	31.191	95	1	-0.245	0.048	3.966	722	WS0038_O13
6	95.249	126	1	0.320	0.072	4.203	722	WS0038_O13
10	78.479	193	3	0.306	0.040	4.722	722	WS0038_O13
11	27.405	204	1	0.344	0.078	6.612	722	WS0038_O13
11	29.069	205	1	0.238	0.050	4.122	722	WS0038_O13
11	45.989	216	1	-0.236	0.088	7.203	722	WS0038_O13
11	45.989	216	2	-0.227	0.037	3.879	722	WS0038_O13
12	6.242	234	2	-0.301	0.053	7.073	722	WS0038_O13
1	40.464	7	3	0.226	0.047	5.533	723	WS00930_H17
1	43.578	8	3	0.214	0.041	4.254	723	WS00930_H17
2	63.261	30	3	0.265	0.040	4.904	723	WS00930_H17
3	83.563	56	3	-0.212	0.056	6.660	723	WS00930_H17
4	75.958	77	3	-0.316	0.093	4.775	723	WS00930_H17
5	31.191	95	1	-0.222	0.047	4.492	723	WS00930_H17
6	25.914	114	1	-0.237	0.044	4.225	723	WS00930_H17
11	45.989	216	1	-0.210	0.049	4.742	723	WS00930_H17
1	50.955	10	1	-0.243	0.079	4.854	724	WS00930_I06
1	69.854	13	2	-0.201	0.041	5.147	724	WS00930_I06
3	47.928	49	1	0.241	0.081	4.986	724	WS00930_I06
4	75.958	77	3	-0.314	0.103	6.744	724	WS00930_I06
4	140.962	84	3	-0.196	0.048	4.812	724	WS00930_I06
4	142.917	85	3	-0.276	0.063	4.923	724	WS00930_I06
5	0.000	89	1	-0.325	0.127	8.344	724	WS00930_I06
5	16.219	93	1	-0.297	0.076	4.687	724	WS00930_I06
5	31.191	95	1	-0.230	0.076	4.707	724	WS00930_I06
6	26.350	115	2	-0.205	0.035	4.361	724	WS00930_I06
6	69.073	121	3	0.301	0.061	6.152	724	WS00930_I06
9	59.963	177	4	-0.230	0.056	4.413	724	WS00930_I06
3	9.953	42	3	0.241	0.069	7.598	725	WS00930_J10
4	55.925	76	1	-0.284	0.057	5.261	725	WS00930_J10
4	82.462	78	1	-0.173	0.041	3.843	725	WS00930_J10
5	0.000	89	1	-0.337	0.062	5.995	725	WS00930_J10
5	16.219	93	1	-0.243	0.064	6.077	725	WS00930_J10
5	31.191	95	1	-0.330	0.081	7.661	725	WS00930_J10
9	55.358	175	1	-0.317	0.043	4.148	725	WS00930_J10
9	81.073	183	1	0.205	0.043	4.080	725	WS00930_J10
9	81.073	183	3	-0.185	0.035	3.847	725	WS00930_J10
11	27.405	204	1	0.298	0.068	6.591	725	WS00930_J10
14	22.402	248	3	0.284	0.052	5.681	725	WS00930_J10
1	40.464	7	3	0.253	0.060	5.752	726	WS00930_J21
1	43.578	8	3	0.276	0.074	7.218	726	WS00930_J21
1	45.794	9	3	-0.239	0.063	5.526	726	WS00930_J21
3	141.443	65	3	-0.287	0.119	5.777	726	WS00930_J21
4	0.000	71	4	-0.259	0.051	4.797	726	WS00930_J21
4	82.462	78	1	-0.300	0.068	6.170	726	WS00930_J21
4	92.039	79	4	-0.298	0.051	4.477	726	WS00930_J21
5	56.008	101	1	-0.187	0.047	4.225	726	WS00930_J21
6	106.702	128	2	0.627	0.191	9.946	726	WS00930_J21
10	104.214	198	4	-0.338	0.069	4.285	726	WS00930_J21
12	38.543	232	4	0.378	0.168	6.966	726	WS00930_J21
13	3.386	245	1	0.295	0.066	5.959	726	WS00930_J21

14	22.402	248	3	-0.272	0.048	4.620	726	WS00930_J21
14	22.402	248	4	0.717	0.321	29.840	726	WS00930_J21
14	43.392	250	2	-0.220	0.049	4.631	726	WS00930_J21
14	46.637	252	3	0.369	0.099	9.419	726	WS00930_J21
3	9.953	42	3	0.153	0.036	3.949	727	WS00930_L01
3	83.563	56	3	-0.199	0.040	4.428	727	WS00930_L01
6	69.073	121	3	0.263	0.044	4.863	727	WS00930_L01
11	0.000	199	2	0.280	0.044	5.035	727	WS00930_L01
1	107.333	19	4	-0.189	0.052	5.249	728	WS00930_L11
3	104.681	60	2	0.185	0.043	4.442	728	WS00930_L11
4	116.928	82	4	0.303	0.062	4.569	728	WS00930_L11
7	62.787	139	4	0.326	0.085	8.992	728	WS00930_L11
7	73.169	142	1	-0.540	0.128	4.663	728	WS00930_L11
9	81.073	183	3	-0.198	0.044	3.878	728	WS00930_L11
10	80.088	194	1	0.236	0.050	4.253	728	WS00930_L11
14	46.637	252	4	-0.298	0.040	3.955	728	WS00930_L11
2	127.147	40	3	-0.230	0.049	4.325	729	WS00930_M11
4	55.925	76	1	-0.238	0.062	4.386	729	WS00930_M11
4	82.462	78	1	-0.215	0.061	4.920	729	WS00930_M11
4	173.929	88	2	-0.211	0.042	4.508	729	WS00930_M11
5	16.219	93	1	-0.263	0.065	5.261	729	WS00930_M11
5	31.191	95	1	-0.224	0.064	5.136	729	WS00930_M11
9	59.963	177	4	0.276	0.050	4.415	729	WS00930_M11
14	0.000	246	2	-0.211	0.055	4.494	729	WS00930_M11
1	45.794	9	1	0.303	0.052	5.284	730	WS00930_M22
4	140.962	84	3	0.264	0.064	5.171	730	WS00930_M22
5	0.000	89	1	0.237	0.046	4.668	730	WS00930_M22
6	49.100	118	4	-0.475	0.186	8.745	730	WS00930_M22
6	81.996	123	3	-0.395	0.075	4.009	730	WS00930_M22
6	81.996	123	4	0.312	0.093	4.463	730	WS00930_M22
7	74.560	143	1	-0.190	0.044	4.450	730	WS00930_M22
9	36.227	165	4	-0.203	0.030	4.098	730	WS00930_M22
9	49.013	173	1	-0.153	0.040	4.599	730	WS00930_M22
9	55.358	175	1	0.256	0.048	4.781	730	WS00930_M22
14	9.923	247	1	0.197	0.054	5.368	730	WS00930_M22
2	93.504	32	4	-0.251	0.045	4.042	731	WS00735_E17
5	42.979	97	1	-0.279	0.051	5.138	731	WS00735_E17
5	54.072	100	4	0.219	0.046	3.962	731	WS00735_E17
7	15.555	130	3	0.293	0.065	3.914	731	WS00735_E17
8	62.580	155	1	-0.348	0.085	4.360	731	WS00735_E17
12	22.441	227	1	-0.310	0.066	6.821	731	WS00735_E17
3	9.953	42	3	0.220	0.031	4.076	732	WS00735_H22
3	137.891	64	3	0.369	0.062	8.572	732	WS00735_H22
4	92.039	79	2	-0.181	0.043	3.937	732	WS00735_H22
5	52.820	99	3	-0.209	0.029	4.117	732	WS00735_H22
12	32.473	238	1	-0.211	0.041	3.991	732	WS00735_H22
3	9.953	42	3	0.249	0.043	5.285	733	WS00735_N18
3	137.891	64	3	0.261	0.038	4.684	733	WS00735_N18
4	173.929	88	2	-0.210	0.050	4.229	733	WS00735_N18
11	37.143	209	3	0.247	0.038	4.618	733	WS00735_N18
11	45.989	216	1	-0.232	0.046	4.714	733	WS00735_N18
3	15.147	44	2	0.309	0.123	7.681	734	WS00735_O12
3	137.891	64	3	0.173	0.042	3.952	734	WS00735_O12
4	82.462	78	1	-0.226	0.045	4.097	734	WS00735_O12

4	173.929	88	2	-0.230	0.060	5.869	734	WS00735_O12
5	0.000	89	1	-0.252	0.052	4.691	734	WS00735_O12
5	16.219	93	1	-0.335	0.078	7.046	734	WS00735_O12
6	81.996	123	3	0.271	0.081	4.863	734	WS00735_O12
10	70.246	190	2	-0.184	0.041	3.967	734	WS00735_O12
11	27.405	204	1	0.324	0.092	8.658	734	WS00735_O12
11	29.069	205	1	0.233	0.055	4.976	734	WS00735_O12
11	45.989	216	1	-0.242	0.063	5.703	734	WS00735_O12
1	40.464	7	3	-0.174	0.037	3.928	735	WS01039_D05
1	43.578	8	3	-0.178	0.039	4.127	735	WS01039_D05
1	45.794	9	3	0.289	0.057	6.233	735	WS01039_D05
3	154.505	69	1	-0.271	0.041	3.914	735	WS01039_D05
5	0.000	89	3	0.231	0.047	5.339	735	WS01039_D05
6	106.702	128	3	-0.183	0.039	4.066	735	WS01039_D05
9	0.000	166	2	0.164	0.039	4.426	735	WS01039_D05
10	78.479	193	3	-0.288	0.046	4.861	735	WS01039_D05
10	80.088	194	1	-0.296	0.046	4.416	735	WS01039_D05
1	38.326	6	2	0.301	0.052	5.572	736	WS01039_D23
1	95.572	17	2	-0.336	0.045	4.857	736	WS01039_D23
1	131.686	23	1	-0.544	0.151	5.024	736	WS01039_D23
4	142.917	85	3	0.360	0.059	5.143	736	WS01039_D23
6	69.073	121	3	-0.176	0.039	4.133	736	WS01039_D23
7	74.560	143	2	0.171	0.045	4.781	736	WS01039_D23
8	11.974	150	1	-0.296	0.092	7.593	736	WS01039_D23
10	21.864	188	1	0.276	0.053	4.481	736	WS01039_D23
10	78.479	193	3	-0.222	0.050	5.278	736	WS01039_D23
11	40.960	210	4	0.351	0.087	7.181	736	WS01039_D23
11	43.491	214	4	0.223	0.084	6.198	736	WS01039_D23
11	44.884	215	4	-0.365	0.091	7.199	736	WS01039_D23
11	48.979	218	4	0.352	0.097	8.036	736	WS01039_D23
11	64.014	222	4	0.291	0.065	5.173	736	WS01039_D23
14	43.392	250	2	0.223	0.044	4.055	736	WS01039_D23
2	75.550	31	2	-0.201	0.029	4.379	737	WS01039_H08
3	137.891	64	3	0.267	0.027	4.249	737	WS01039_H08
4	142.917	85	1	0.184	0.050	4.005	737	WS01039_H08
4	173.929	88	2	-0.161	0.046	4.872	737	WS01039_H08
6	54.066	119	4	-0.196	0.070	6.421	737	WS01039_H08
7	62.787	139	2	0.240	0.036	4.079	737	WS01039_H08
10	104.214	198	4	0.394	0.048	5.257	737	WS01039_H08
11	27.405	204	1	0.260	0.071	5.843	737	WS01039_H08
11	27.405	204	4	-0.255	0.056	5.091	737	WS01039_H08
11	40.960	210	2	0.351	0.066	4.244	737	WS01039_H08
3	9.953	42	3	0.244	0.051	5.109	738	WS01039_I06
3	116.958	63	4	-0.185	0.052	4.525	738	WS01039_I06
4	173.929	88	2	-0.237	0.061	6.681	738	WS01039_I06
5	54.072	100	4	0.172	0.048	4.219	738	WS01039_I06
6	81.996	123	3	0.326	0.086	4.972	738	WS01039_I06
10	70.246	190	2	-0.233	0.035	3.863	738	WS01039_I06
11	27.405	204	4	-0.313	0.075	5.371	738	WS01039_I06
1	62.128	11	1	-0.638	0.162	8.699	739	WS00930_O03
3	9.953	42	4	0.201	0.044	5.722	739	WS00930_O03
4	124.132	83	1	-0.540	0.110	5.893	739	WS00930_O03
8	11.974	150	1	0.203	0.048	3.965	739	WS00930_O03
11	17.088	203	1	0.205	0.054	4.387	739	WS00930_O03

11	27.405	204	4	-0.281	0.042	5.684	739	WS00930_O03
11	40.960	210	2	0.406	0.062	4.950	739	WS00930_O03
12	28.493	228	2	0.363	0.103	8.231	739	WS00930_O03
3	114.747	62	2	-0.218	0.048	4.698	740	WS00930_O11
4	173.929	88	2	-0.195	0.045	4.415	740	WS00930_O11
6	81.996	123	3	0.382	0.146	8.605	740	WS00930_O11
2	93.504	32	4	-0.371	0.091	9.111	741	WS00930_P19
3	9.953	42	3	0.174	0.047	3.946	741	WS00930_P19
4	6.032	72	4	-0.238	0.052	5.336	741	WS00930_P19
4	92.039	79	4	-0.319	0.055	5.236	741	WS00930_P19
6	69.073	121	4	0.311	0.059	5.740	741	WS00930_P19
6	95.249	126	1	0.353	0.092	5.726	741	WS00930_P19
9	81.073	183	1	0.269	0.035	4.012	741	WS00930_P19
11	9.237	202	4	0.227	0.049	5.046	741	WS00930_P19
11	27.405	204	1	0.322	0.046	5.128	741	WS00930_P19
11	27.405	204	4	-0.314	0.078	8.416	741	WS00930_P19
11	40.960	210	2	0.509	0.123	7.075	741	WS00930_P19
11	40.960	210	4	-0.237	0.045	4.573	741	WS00930_P19
11	45.989	216	1	-0.206	0.043	4.796	741	WS00930_P19
1	22.419	4	4	-0.425	0.155	4.842	742	WS00931_A05
1	38.326	6	4	-0.342	0.056	4.760	742	WS00931_A05
3	47.928	49	1	0.263	0.073	6.445	742	WS00931_A05
3	95.157	59	2	-0.188	0.042	4.275	742	WS00931_A05
4	97.576	80	2	-0.173	0.032	4.169	742	WS00931_A05
4	124.132	83	1	0.445	0.129	3.949	742	WS00931_A05
4	142.917	85	1	-0.335	0.106	9.388	742	WS00931_A05
9	28.608	170	3	0.275	0.045	4.264	742	WS00931_A05
12	22.629	237	1	0.210	0.045	4.007	742	WS00931_A05
1	95.572	17	2	0.258	0.041	5.206	743	WS00931_B11
3	17.158	45	1	0.214	0.069	4.148	743	WS00931_B11
7	75.883	144	2	-0.380	0.039	3.923	743	WS00931_B11
9	59.963	177	4	0.349	0.084	8.779	743	WS00931_B11
10	104.214	198	4	0.299	0.049	7.749	743	WS00931_B11
12	6.242	234	2	-0.171	0.027	5.653	743	WS00931_B11
14	22.402	248	4	-0.289	0.042	4.342	743	WS00931_B11
2	63.261	30	3	0.237	0.049	6.021	744	WS00931_B23
4	173.929	88	2	-0.294	0.060	7.505	744	WS00931_B23
9	36.227	165	3	-0.177	0.034	4.150	744	WS00931_B23
10	70.246	190	2	-0.216	0.043	5.278	744	WS00931_B23
11	45.989	216	1	-0.265	0.068	4.231	744	WS00931_B23
12	22.441	227	3	0.310	0.052	5.070	744	WS00931_B23
12	6.242	234	2	-0.269	0.054	6.860	744	WS00931_B23
3	116.958	63	4	-0.342	0.047	4.149	745	WS00931_D06
4	173.929	88	2	-0.368	0.054	6.606	745	WS00931_D06
5	0.000	89	3	-0.250	0.033	3.999	745	WS00931_D06
9	28.608	170	3	-0.324	0.055	5.908	745	WS00931_D06
10	104.214	198	2	0.303	0.036	4.792	745	WS00931_D06
11	35.604	207	3	-0.179	0.037	4.271	745	WS00931_D06
11	37.143	209	3	0.216	0.054	6.181	745	WS00931_D06
11	42.600	213	3	-0.186	0.036	4.115	745	WS00931_D06
11	45.989	216	1	-0.336	0.048	4.300	745	WS00931_D06
11	60.464	221	1	0.158	0.059	4.481	745	WS00931_D06
11	64.014	222	3	-0.249	0.035	4.239	745	WS00931_D06
12	6.242	234	2	-0.192	0.051	5.573	745	WS00931_D06

3	9.953	42	3	-0.206	0.048	5.255	746	WS00931_D19
6	106.702	128	3	0.302	0.060	4.148	746	WS00931_D19
9	0.000	163	1	0.514	0.100	6.136	746	WS00931_D19
9	55.358	175	1	0.312	0.055	5.240	746	WS00931_D19
9	58.045	176	1	-0.265	0.066	4.060	746	WS00931_D19
9	59.963	177	1	-0.311	0.071	6.855	746	WS00931_D19
9	61.113	178	1	-0.280	0.112	3.973	746	WS00931_D19
9	69.046	181	1	0.354	0.101	6.198	746	WS00931_D19
9	81.073	183	1	-0.214	0.040	3.939	746	WS00931_D19
10	86.151	195	3	0.227	0.047	5.135	746	WS00931_D19
11	40.960	210	2	0.376	0.088	3.910	746	WS00931_D19
12	28.493	228	2	0.282	0.127	5.372	746	WS00931_D19
7	105.477	147	2	-0.384	0.175	10.122	747	WS00931_F16
9	2.011	167	4	0.193	0.048	4.195	747	WS00931_F16
9	28.608	170	2	-0.318	0.067	7.854	747	WS00931_F16
9	32.356	171	2	-0.243	0.049	5.599	747	WS00931_F16
10	70.246	190	4	-0.602	0.268	20.461	747	WS00931_F16
10	78.479	193	3	0.486	0.128	12.751	747	WS00931_F16
10	78.479	193	4	-0.452	0.236	20.713	747	WS00931_F16
10	86.151	195	3	-0.425	0.145	14.590	747	WS00931_F16
10	95.795	197	4	0.332	0.139	12.221	747	WS00931_F16
10	104.214	198	4	-0.281	0.058	4.370	747	WS00931_F16
11	9.237	202	4	0.211	0.062	5.460	747	WS00931_F16
11	40.960	210	2	-0.499	0.117	6.884	747	WS00931_F16
11	43.491	214	3	-0.304	0.073	7.291	747	WS00931_F16
11	44.884	215	3	0.275	0.040	4.035	747	WS00931_F16
12	0.000	226	3	0.157	0.042	4.181	747	WS00931_F16
12	22.441	227	1	0.239	0.050	3.934	747	WS00931_F16
12	22.441	227	3	-0.420	0.125	12.600	747	WS00931_F16
12	28.493	228	2	-0.292	0.108	6.254	747	WS00931_F16
3	9.953	42	3	0.215	0.049	4.431	748	WS00931_J07
3	15.147	44	2	0.322	0.073	4.927	748	WS00931_J07
3	80.211	55	4	0.250	0.043	4.205	748	WS00931_J07
3	137.891	64	3	0.348	0.068	5.078	748	WS00931_J07
4	0.000	71	4	0.225	0.041	3.939	748	WS00931_J07
4	55.925	76	1	-0.262	0.062	5.102	748	WS00931_J07
5	52.820	99	1	0.178	0.053	4.189	748	WS00931_J07
14	22.402	248	3	0.274	0.045	4.174	748	WS00931_J07
1	45.794	9	3	-0.194	0.035	4.075	749	WS01025_L07
3	14.500	43	1	-0.245	0.053	3.917	749	WS01025_L07
3	20.512	46	2	0.164	0.047	5.321	749	WS01025_L07
3	112.868	61	2	-0.259	0.046	5.284	749	WS01025_L07
3	114.747	62	2	-0.154	0.042	4.788	749	WS01025_L07
3	137.891	64	3	0.307	0.060	6.277	749	WS01025_L07
7	17.610	131	3	0.267	0.064	4.059	749	WS01025_L07
11	45.989	216	1	-0.345	0.112	8.334	749	WS01025_L07
1	2.788	2	3	-0.216	0.050	5.478	750	WS01026_A02
1	45.794	9	1	0.224	0.040	3.870	750	WS01026_A02
4	82.462	78	1	-0.190	0.044	4.188	750	WS01026_A02
9	32.356	171	2	-0.208	0.049	4.528	750	WS01026_A02
10	9.580	187	3	-0.375	0.115	7.945	750	WS01026_A02
10	80.088	194	1	-0.265	0.044	4.212	750	WS01026_A02
3	15.147	44	2	0.274	0.074	5.130	751	WS0099_G16
4	82.462	78	1	-0.233	0.064	5.084	751	WS0099_G16

4	173.929	88	2	-0.264	0.059	6.490	751	WS0099_G16
8	11.974	150	1	-0.201	0.053	4.176	751	WS0099_G16
10	70.246	190	2	-0.206	0.045	4.894	751	WS0099_G16
11	9.237	202	4	0.199	0.046	4.301	751	WS0099_G16
11	27.405	204	1	0.232	0.069	5.553	751	WS0099_G16
11	27.405	204	4	-0.382	0.107	10.004	751	WS0099_G16
11	45.989	216	1	-0.253	0.059	4.630	751	WS0099_G16
3	15.147	44	2	0.226	0.065	4.004	752	WS0099_H07
3	137.891	64	3	0.248	0.067	6.523	752	WS0099_H07
3	141.443	65	3	0.292	0.085	5.306	752	WS0099_H07
4	0.000	71	4	0.264	0.059	5.399	752	WS0099_H07
4	173.929	88	2	-0.216	0.052	5.120	752	WS0099_H07
10	70.246	190	2	-0.232	0.054	5.375	752	WS0099_H07
11	27.405	204	1	0.265	0.066	6.065	752	WS0099_H07
11	45.989	216	1	-0.231	0.059	5.248	752	WS0099_H07
1	45.794	9	1	0.340	0.075	5.768	753	WS0099_J19
3	15.147	44	2	0.246	0.061	4.521	753	WS0099_J19
3	20.512	46	2	0.271	0.058	6.532	753	WS0099_J19
3	104.681	60	2	0.211	0.038	4.194	753	WS0099_J19
4	173.929	88	2	-0.236	0.051	5.696	753	WS0099_J19
11	27.405	204	1	0.261	0.062	4.810	753	WS0099_J19
11	27.405	204	4	-0.335	0.076	8.190	753	WS0099_J19
1	69.854	13	2	0.204	0.042	4.206	754	WS0099_K05
1	71.592	14	2	0.226	0.053	5.367	754	WS0099_K05
1	77.177	15	2	0.210	0.044	4.500	754	WS0099_K05
3	14.500	43	1	-0.239	0.055	4.773	754	WS0099_K05
4	82.462	78	1	-0.224	0.046	4.004	754	WS0099_K05
7	17.610	131	3	0.289	0.108	5.178	754	WS0099_K05
7	62.787	139	2	0.231	0.045	4.294	754	WS0099_K05
10	70.246	190	2	-0.277	0.045	4.594	754	WS0099_K05
11	27.405	204	1	0.344	0.093	8.235	754	WS0099_K05
11	27.405	204	4	-0.300	0.087	6.625	754	WS0099_K05
11	45.989	216	1	-0.234	0.072	6.260	754	WS0099_K05
1	50.955	10	1	-0.144	0.044	4.056	755	WS0099_L11
2	101.076	35	4	-0.295	0.048	4.944	755	WS0099_L11
2	102.814	36	1	0.231	0.049	5.508	755	WS0099_L11
4	0.000	71	4	-0.128	0.043	4.362	755	WS0099_L11
4	97.576	80	2	0.234	0.037	3.871	755	WS0099_L11
5	16.219	93	2	-0.277	0.056	5.907	755	WS0099_L11
5	52.820	99	1	0.166	0.047	4.378	755	WS0099_L11
7	91.487	146	3	0.165	0.048	4.169	755	WS0099_L11
9	36.227	165	4	0.217	0.057	5.500	755	WS0099_L11
4	140.962	84	3	-0.232	0.039	4.060	756	WS0099_N01
4	142.917	85	3	-0.266	0.041	4.182	756	WS0099_N01
4	173.929	88	2	-0.296	0.081	8.077	756	WS0099_N01
6	81.996	123	3	0.389	0.144	8.880	756	WS0099_N01
11	45.989	216	1	-0.266	0.060	5.282	756	WS0099_N01
14	44.898	251	3	0.292	0.051	5.174	756	WS0099_N01
2	63.261	30	3	0.260	0.050	6.039	757	WS0099_P15
3	95.157	59	2	-0.186	0.035	4.428	757	WS0099_P15
3	137.891	64	3	0.397	0.119	14.263	757	WS0099_P15
4	0.000	71	4	0.251	0.065	4.378	757	WS0099_P15
8	50.001	154	2	-0.221	0.049	5.695	757	WS0099_P15
10	9.580	187	2	-0.251	0.083	4.409	757	WS0099_P15

10	35.204	189	2	0.291	0.065	6.257	757	WS0099_P15
10	70.246	190	2	-0.204	0.045	5.165	757	WS0099_P15
11	60.464	221	2	0.205	0.034	3.878	757	WS0099_P15
12	22.441	227	3	0.310	0.051	6.192	757	WS0099_P15
14	44.898	251	3	0.331	0.058	6.919	757	WS0099_P15
1	133.992	24	2	0.219	0.038	4.357	758	WS00910_A01
3	14.500	43	2	-0.259	0.062	6.919	758	WS00910_A01
3	137.891	64	3	0.234	0.050	5.298	758	WS00910_A01
7	17.610	131	3	0.390	0.091	6.721	758	WS00910_A01
8	87.463	158	2	-0.250	0.049	4.423	758	WS00910_A01
10	70.246	190	2	-0.276	0.059	6.776	758	WS00910_A01
10	80.088	194	2	0.331	0.064	7.205	758	WS00910_A01
10	104.214	198	2	0.331	0.067	8.798	758	WS00910_A01
11	9.237	202	4	0.239	0.083	6.499	758	WS00910_A01
11	27.405	204	4	-0.396	0.108	6.288	758	WS00910_A01
11	43.491	214	3	-0.222	0.035	3.885	758	WS00910_A01
11	56.921	219	2	-0.260	0.070	5.020	758	WS00910_A01
12	6.242	234	2	-0.261	0.049	6.978	758	WS00910_A01
5	31.191	95	1	-0.210	0.056	4.521	759	WS01026_D17
6	87.891	124	2	-0.210	0.040	4.260	759	WS01026_D17
11	27.405	204	1	0.542	0.205	17.553	759	WS01026_D17
11	27.405	204	4	-0.377	0.088	8.546	759	WS01026_D17
11	29.069	205	1	0.404	0.132	10.975	759	WS01026_D17
11	45.989	216	1	-0.347	0.127	10.276	759	WS01026_D17
11	60.464	221	1	0.190	0.049	3.937	759	WS01026_D17
1	77.177	15	2	0.185	0.047	4.548	760	WS01026_E12
1	95.572	17	2	0.263	0.064	6.280	760	WS01026_E12
4	55.925	76	1	-0.259	0.061	5.392	760	WS01026_E12
5	31.191	95	1	-0.248	0.063	5.692	760	WS01026_E12
6	49.100	118	4	-0.345	0.175	4.331	760	WS01026_E12
6	69.073	121	4	0.262	0.051	4.768	760	WS01026_E12
6	95.249	126	1	0.260	0.064	4.147	760	WS01026_E12
9	59.963	177	4	0.308	0.068	6.681	760	WS01026_E12
11	27.405	204	1	0.259	0.057	5.117	760	WS01026_E12
11	27.405	204	4	-0.251	0.056	5.698	760	WS01026_E12
1	15.589	3	2	0.177	0.044	4.230	761	WS01026_G12
3	80.211	55	4	0.253	0.052	4.143	761	WS01026_G12
8	50.001	154	2	-0.230	0.071	6.725	761	WS01026_G12
14	22.402	248	4	-0.281	0.066	5.128	761	WS01026_G12
1	45.794	9	1	0.339	0.092	7.556	762	WS01026_G22
1	50.955	10	1	-0.326	0.098	8.028	762	WS01026_G22
3	137.891	64	3	0.316	0.068	6.945	762	WS01026_G22
3	148.745	68	1	-0.254	0.047	3.870	762	WS01026_G22
4	43.146	74	3	0.232	0.041	4.390	762	WS01026_G22
5	42.979	97	1	0.249	0.052	4.256	762	WS01026_G22
5	52.820	99	1	0.283	0.071	5.861	762	WS01026_G22
6	49.100	118	4	-0.443	0.264	5.060	762	WS01026_G22
6	54.066	119	4	-0.200	0.062	5.134	762	WS01026_G22
6	89.401	125	1	-0.270	0.071	5.775	762	WS01026_G22
6	95.249	126	1	0.233	0.079	4.421	762	WS01026_G22
7	59.789	137	3	-0.328	0.098	10.364	762	WS01026_G22
8	95.937	159	3	-0.243	0.047	4.936	762	WS01026_G22
11	45.989	216	1	-0.230	0.050	4.077	762	WS01026_G22
12	14.406	230	3	-0.253	0.061	6.456	762	WS01026_G22

14	22.402	248	3	0.349	0.069	7.261	762	WS01026_G22
1	2.788	2	4	-0.214	0.080	3.978	763	WS01026_I18
1	29.800	5	1	0.316	0.051	5.284	763	WS01026_I18
2	95.880	34	1	0.356	0.044	4.557	763	WS01026_I18
2	120.617	38	3	0.245	0.034	3.920	763	WS01026_I18
2	127.147	40	3	-0.295	0.064	7.474	763	WS01026_I18
4	43.146	74	3	0.224	0.035	4.073	763	WS01026_I18
5	0.000	89	3	-0.302	0.076	9.145	763	WS01026_I18
7	105.477	147	2	-0.284	0.099	4.093	763	WS01026_I18
9	36.227	165	3	-0.376	0.108	12.487	763	WS01026_I18
11	35.604	207	3	-0.361	0.099	11.517	763	WS01026_I18
11	37.143	209	3	0.378	0.110	12.766	763	WS01026_I18
11	42.600	213	3	-0.350	0.090	10.400	763	WS01026_I18
1	29.800	5	1	0.243	0.039	5.448	764	WS01026_J12
1	40.464	7	3	0.227	0.042	5.597	764	WS01026_J12
1	43.578	8	3	0.214	0.036	4.510	764	WS01026_J12
3	154.505	69	1	0.235	0.031	4.326	764	WS01026_J12
7	74.560	143	1	0.262	0.036	5.138	764	WS01026_J12
8	95.937	159	3	-0.220	0.039	5.288	764	WS01026_J12
11	0.000	199	3	-0.312	0.056	6.295	764	WS01026_J12
11	27.405	204	1	0.222	0.041	5.774	764	WS01026_J12
11	45.989	216	1	-0.181	0.028	3.956	764	WS01026_J12
14	0.000	246	1	0.240	0.033	4.640	764	WS01026_J12
14	46.637	252	3	0.200	0.029	3.925	764	WS01026_J12
4	173.929	88	2	-0.214	0.053	5.158	765	WS01026_L08
11	27.405	204	1	0.369	0.112	10.274	765	WS01026_L08
11	27.405	204	4	-0.256	0.060	5.697	765	WS01026_L08
11	29.069	205	1	0.230	0.062	5.603	765	WS01026_L08
11	45.989	216	1	-0.263	0.078	7.038	765	WS01026_L08
12	6.242	234	2	-0.235	0.037	4.338	765	WS01026_L08
1	22.419	4	3	0.372	0.093	6.175	766	WS01026_L17
3	15.147	44	2	0.270	0.094	5.141	766	WS01026_L17
3	116.958	63	4	-0.193	0.039	3.867	766	WS01026_L17
6	87.891	124	2	-0.213	0.057	4.465	766	WS01026_L17
7	105.477	147	2	-0.244	0.078	4.444	766	WS01026_L17
9	36.227	165	3	-0.273	0.057	5.071	766	WS01026_L17
11	27.405	204	1	0.252	0.038	4.250	766	WS01026_L17
11	45.989	216	1	-0.224	0.046	4.977	766	WS01026_L17
13	3.386	245	1	-0.202	0.048	5.177	766	WS01026_L17
1	50.955	10	1	-0.274	0.084	7.614	767	WS01026_P02
1	69.854	13	2	-0.183	0.048	4.618	767	WS01026_P02
1	71.592	14	2	-0.182	0.048	4.613	767	WS01026_P02
4	142.917	85	1	0.210	0.052	4.799	767	WS01026_P02
6	26.350	115	2	-0.222	0.059	5.729	767	WS01026_P02
9	32.356	171	2	-0.228	0.054	5.221	767	WS01026_P02
9	49.013	173	2	0.242	0.063	6.091	767	WS01026_P02
1	95.572	17	3	0.281	0.048	4.509	768	WS00912_E24
2	102.814	36	1	0.234	0.043	4.068	768	WS00912_E24
3	47.928	49	1	0.255	0.041	3.893	768	WS00912_E24
8	50.001	154	2	-0.245	0.051	5.007	768	WS00912_E24
14	46.637	252	3	0.268	0.041	5.085	768	WS00912_E24
6	56.188	120	2	0.226	0.030	4.331	769	WS00819_E15
13	1.815	244	4	0.309	0.062	5.287	769	WS00819_E15
3	9.953	42	4	0.196	0.038	4.239	770	WS00912_J20

3	83.563	56	3	0.152	0.054	4.164	770	WS00912_J20
7	105.477	147	2	0.367	0.135	9.615	770	WS00912_J20
10	9.580	187	2	0.242	0.063	4.541	770	WS00912_J20
10	9.580	187	3	0.369	0.080	4.369	770	WS00912_J20
10	104.214	198	4	0.315	0.053	5.016	770	WS00912_J20
11	40.960	210	2	0.346	0.057	4.157	770	WS00912_J20
11	60.464	221	2	-0.178	0.042	4.552	770	WS00912_J20
12	6.242	234	2	0.267	0.074	6.749	770	WS00912_J20
13	1.815	244	4	0.158	0.034	3.949	770	WS00912_J20
14	22.402	248	4	-0.358	0.059	6.742	770	WS00912_J20
14	46.637	252	4	-0.521	0.147	16.626	770	WS00912_J20
3	9.953	42	3	0.236	0.042	4.588	771	WS00829_K19
3	137.891	64	3	0.249	0.045	4.998	771	WS00829_K19
3	141.443	65	3	0.223	0.055	4.592	771	WS00829_K19
4	82.462	78	1	-0.155	0.038	4.066	771	WS00829_K19
4	173.929	88	2	-0.218	0.062	5.032	771	WS00829_K19
11	27.405	204	1	0.168	0.037	3.944	771	WS00829_K19
11	27.405	204	4	-0.241	0.051	3.929	771	WS00829_K19
4	173.929	88	2	-0.224	0.065	5.773	772	WS00830_F02
6	81.996	123	3	0.327	0.065	4.718	772	WS00830_F02
8	11.974	150	1	-0.238	0.043	4.253	772	WS00830_F02
9	59.963	177	4	0.183	0.053	3.921	772	WS00830_F02
9	81.073	183	1	0.190	0.039	3.893	772	WS00830_F02
11	27.405	204	1	0.208	0.060	6.341	772	WS00830_F02
3	9.953	42	3	0.220	0.041	3.944	773	WS00931_N16
5	3.484	90	2	-0.296	0.100	6.846	773	WS00931_N16
9	36.227	165	3	0.195	0.041	3.888	773	WS00931_N16
9	49.013	173	2	0.217	0.045	5.148	773	WS00931_N16
1	2.788	2	4	0.186	0.033	4.022	774	WS01029_C14
1	22.419	4	4	0.358	0.121	4.635	774	WS01029_C14
3	9.953	42	4	-0.195	0.045	5.492	774	WS01029_C14
4	75.958	77	3	0.367	0.116	5.411	774	WS01029_C14
7	62.787	139	2	-0.219	0.049	6.978	774	WS01029_C14
7	73.169	142	1	-0.530	0.115	4.629	774	WS01029_C14
8	95.937	159	3	-0.228	0.061	4.363	774	WS01029_C14
9	59.963	177	4	-0.226	0.042	4.933	774	WS01029_C14
2	48.839	29	4	0.303	0.066	4.009	775	WS00913_K13
3	9.953	42	3	0.225	0.047	5.865	775	WS00913_K13
3	95.157	59	2	-0.201	0.036	4.220	775	WS00913_K13
3	137.891	64	3	0.265	0.040	5.035	775	WS00913_K13
4	140.962	84	3	-0.208	0.040	4.963	775	WS00913_K13
5	0.000	89	1	-0.294	0.054	5.316	775	WS00913_K13
5	16.219	93	1	-0.207	0.042	3.957	775	WS00913_K13
5	54.072	100	4	0.221	0.065	4.120	775	WS00913_K13
6	81.996	123	3	0.294	0.090	6.474	775	WS00913_K13
7	15.555	130	3	-0.236	0.032	4.628	775	WS00913_K13
7	70.278	141	3	-0.228	0.045	5.636	775	WS00913_K13
9	28.608	170	3	0.243	0.038	4.425	775	WS00913_K13
10	77.676	192	1	-0.247	0.049	4.673	775	WS00913_K13
10	80.088	194	1	-0.307	0.064	6.028	775	WS00913_K13
11	0.000	199	3	-0.251	0.039	4.954	775	WS00913_K13
11	27.405	204	1	0.290	0.070	6.795	775	WS00913_K13
11	45.989	216	1	-0.204	0.059	5.544	775	WS00913_K13
11	64.014	222	4	-0.239	0.066	3.951	775	WS00913_K13

14	44.898	251	3	0.220	0.030	3.853	775	WS00913_K13
1	95.572	17	2	0.267	0.061	5.703	776	WS00914_A18
4	0.000	71	4	0.208	0.045	4.064	776	WS00914_A18
11	27.405	204	1	0.333	0.077	7.402	776	WS00914_A18
11	45.989	216	1	-0.205	0.050	4.775	776	WS00914_A18
1	43.578	8	3	0.227	0.056	4.243	777	WS00910_B13
2	75.550	31	2	-0.222	0.050	5.334	777	WS00910_B13
2	127.147	40	4	-0.320	0.055	4.791	777	WS00910_B13
4	173.929	88	2	-0.273	0.055	4.507	777	WS00910_B13
10	80.088	194	1	-0.235	0.041	4.546	777	WS00910_B13
10	93.347	196	1	0.466	0.100	5.216	777	WS00910_B13
11	9.237	202	4	0.273	0.117	12.274	777	WS00910_B13
11	27.405	204	4	-0.290	0.075	7.689	777	WS00910_B13
11	45.989	216	1	-0.309	0.047	5.302	777	WS00910_B13
12	38.543	232	4	-0.508	0.073	4.714	777	WS00910_B13
14	44.898	251	3	0.273	0.038	4.223	777	WS00910_B13
1	95.572	17	2	0.320	0.060	6.615	778	WS00910_C12
5	54.072	100	4	0.239	0.052	5.184	778	WS00910_C12
7	73.169	142	2	0.523	0.081	6.617	778	WS00910_C12
7	74.560	143	2	-0.225	0.053	4.658	778	WS00910_C12
7	75.883	144	2	-0.576	0.103	8.709	778	WS00910_C12
9	36.227	165	3	-0.225	0.046	4.029	778	WS00910_C12
9	59.963	177	4	0.381	0.125	12.531	778	WS00910_C12
11	27.405	204	1	0.301	0.079	6.498	778	WS00910_C12
11	45.989	216	1	-0.200	0.059	4.702	778	WS00910_C12
1	95.572	17	2	0.296	0.046	5.054	779	WS00910_F21
3	14.500	43	1	-0.226	0.066	5.634	779	WS00910_F21
3	17.158	45	1	0.296	0.054	4.779	779	WS00910_F21
4	142.917	85	1	0.293	0.059	5.141	779	WS00910_F21
4	173.929	88	2	-0.200	0.068	6.881	779	WS00910_F21
5	0.000	89	3	-0.292	0.049	4.929	779	WS00910_F21
5	57.017	102	4	-0.202	0.045	4.159	779	WS00910_F21
9	0.000	163	1	-0.492	0.105	5.357	779	WS00910_F21
9	49.013	173	2	0.170	0.048	5.034	779	WS00910_F21
10	70.246	190	2	-0.226	0.049	5.034	779	WS00910_F21
10	70.246	190	4	0.293	0.044	4.149	779	WS00910_F21
10	80.088	194	2	0.285	0.047	4.236	779	WS00910_F21
10	104.214	198	2	0.304	0.047	4.735	779	WS00910_F21
11	42.600	213	3	-0.155	0.045	4.270	779	WS00910_F21
11	43.491	214	4	-0.203	0.067	6.093	779	WS00910_F21
12	6.242	234	2	-0.193	0.032	4.067	779	WS00910_F21
14	43.392	250	2	-0.235	0.041	3.956	779	WS00910_F21
1	29.800	5	1	0.235	0.035	4.185	780	WS00910_G09
1	50.955	10	1	-0.221	0.045	5.369	780	WS00910_G09
1	141.021	25	2	0.195	0.059	3.964	780	WS00910_G09
2	102.814	36	1	0.230	0.034	4.094	780	WS00910_G09
6	56.188	120	1	-0.284	0.043	5.180	780	WS00910_G09
7	15.555	130	3	0.286	0.035	4.082	780	WS00910_G09
11	44.884	215	3	-0.193	0.037	5.021	780	WS00910_G09
12	14.406	230	3	-0.257	0.029	3.959	780	WS00910_G09
1	43.578	8	3	0.187	0.041	4.062	781	WS00910_I08
3	9.953	42	3	0.132	0.036	4.047	781	WS00910_I08
3	9.953	42	4	0.233	0.053	3.972	781	WS00910_I08
4	173.929	88	2	-0.171	0.046	4.344	781	WS00910_I08

6	95.249	126	1	0.404	0.100	7.128	781	WS00910_I08
9	49.013	173	1	0.349	0.103	9.182	781	WS00910_I08
10	93.347	196	1	0.593	0.119	8.462	781	WS00910_I08
11	27.405	204	1	0.330	0.058	5.506	781	WS00910_I08
11	29.069	205	1	0.227	0.041	3.906	781	WS00910_I08
11	37.143	209	3	0.197	0.036	4.009	781	WS00910_I08
11	45.989	216	1	-0.215	0.064	5.959	781	WS00910_I08
13	3.386	245	1	-0.290	0.104	9.703	781	WS00910_I08
1	40.464	7	3	0.293	0.068	7.320	782	WS00910_I23
1	43.578	8	3	0.292	0.067	7.348	782	WS00910_I23
1	45.794	9	3	-0.234	0.041	4.355	782	WS00910_I23
1	95.572	17	2	-0.222	0.046	4.221	782	WS00910_I23
1	123.316	20	4	-0.311	0.102	5.928	782	WS00910_I23
1	127.805	21	4	0.222	0.056	4.430	782	WS00910_I23
3	47.928	49	1	0.247	0.049	4.764	782	WS00910_I23
6	87.891	124	2	-0.238	0.070	6.457	782	WS00910_I23
7	15.555	130	3	0.340	0.068	6.722	782	WS00910_I23
1	95.572	17	2	0.264	0.057	6.018	783	WS00910_L14
1	95.572	17	3	-0.193	0.045	4.030	783	WS00910_L14
2	48.839	29	4	0.381	0.113	8.716	783	WS00910_L14
3	144.593	66	4	0.200	0.059	5.906	783	WS00910_L14
3	148.745	68	4	-0.230	0.046	4.381	783	WS00910_L14
4	55.925	76	1	-0.256	0.064	4.960	783	WS00910_L14
4	82.462	78	1	-0.264	0.111	9.285	783	WS00910_L14
4	116.928	82	4	0.230	0.047	4.367	783	WS00910_L14
4	142.917	85	1	0.239	0.060	5.053	783	WS00910_L14
4	173.929	88	2	-0.243	0.065	6.752	783	WS00910_L14
7	62.787	139	2	0.235	0.052	4.625	783	WS00910_L14
11	27.405	204	4	-0.237	0.045	4.194	783	WS00910_L14
14	43.392	250	2	-0.224	0.049	5.096	783	WS00910_L14
14	46.637	252	4	-0.340	0.048	5.103	783	WS00910_L14
1	123.316	20	4	-0.315	0.068	4.138	784	WS00910_M05
1	133.992	24	2	-0.233	0.040	4.475	784	WS00910_M05
1	141.021	25	2	0.240	0.046	5.055	784	WS00910_M05
2	95.880	34	1	0.280	0.083	6.730	784	WS00910_M05
4	43.146	74	3	0.216	0.047	3.972	784	WS00910_M05
5	42.979	97	2	-0.283	0.043	4.692	784	WS00910_M05
7	74.560	143	1	0.188	0.051	4.004	784	WS00910_M05
8	0.000	148	4	0.176	0.042	4.340	784	WS00910_M05
9	55.358	175	3	0.479	0.151	4.141	784	WS00910_M05
11	60.464	221	1	0.302	0.060	4.760	784	WS00910_M05
12	32.473	238	1	0.238	0.050	3.960	784	WS00910_M05
14	44.898	251	3	-0.270	0.068	5.209	784	WS00910_M05
3	15.147	44	2	0.251	0.049	3.906	785	WS00830_M15
4	0.000	71	4	0.261	0.046	5.327	785	WS00830_M15
4	124.132	83	1	-0.524	0.167	5.511	785	WS00830_M15
4	142.917	85	1	0.276	0.063	4.863	785	WS00830_M15
4	173.929	88	2	-0.249	0.062	6.885	785	WS00830_M15
8	87.463	158	2	-0.219	0.044	4.731	785	WS00830_M15
10	70.246	190	2	-0.254	0.047	5.351	785	WS00830_M15
11	27.405	204	1	0.197	0.058	4.604	785	WS00830_M15
3	114.747	62	2	-0.184	0.045	4.102	786	WS00830_O03
4	0.000	71	4	0.224	0.057	4.784	786	WS00830_O03
4	173.929	88	2	-0.256	0.067	6.161	786	WS00830_O03

11	45.989	216	1	-0.241	0.045	4.344	786	WS00830_O03
1	95.572	17	2	0.302	0.046	4.254	787	WS00831_B22
2	127.147	40	4	-0.250	0.058	4.731	787	WS00831_B22
3	141.443	65	3	0.383	0.115	8.372	787	WS00831_B22
4	0.000	71	4	0.275	0.058	4.890	787	WS00831_B22
4	173.929	88	2	-0.241	0.071	6.599	787	WS00831_B22
9	81.073	183	1	0.360	0.089	8.433	787	WS00831_B22
11	27.405	204	1	0.205	0.043	4.258	787	WS00831_B22
11	27.405	204	4	-0.324	0.053	4.379	787	WS00831_B22
14	44.898	251	3	0.294	0.043	4.522	787	WS00831_B22
1	29.800	5	1	-0.225	0.037	3.873	788	WS00831_D02
4	55.925	76	1	-0.215	0.046	4.580	788	WS00831_D02
4	173.929	88	2	-0.220	0.051	4.202	788	WS00831_D02
8	87.463	158	2	-0.176	0.049	3.948	788	WS00831_D02
9	81.073	183	1	0.266	0.057	6.139	788	WS00831_D02
1	82.301	16	4	0.220	0.038	4.119	789	WS00831_G01
5	56.008	101	2	0.163	0.028	3.841	789	WS00831_G01
8	39.090	152	4	-0.416	0.181	20.413	789	WS00831_G01
8	50.001	154	1	-0.215	0.074	5.021	789	WS00831_G01
8	50.001	154	2	0.231	0.067	7.902	789	WS00831_G01
8	87.463	158	2	-0.318	0.073	7.138	789	WS00831_G01
3	15.147	44	2	0.348	0.102	8.964	790	WS00831_H20
3	80.211	55	4	0.228	0.041	4.615	790	WS00831_H20
3	137.891	64	3	0.224	0.064	4.928	790	WS00831_H20
4	6.032	72	4	0.224	0.043	4.810	790	WS00831_H20
4	173.929	88	2	-0.211	0.046	5.372	790	WS00831_H20
6	87.891	124	2	-0.197	0.035	4.112	790	WS00831_H20
6	89.401	125	2	-0.263	0.043	4.701	790	WS00831_H20
11	27.405	204	1	0.199	0.063	4.647	790	WS00831_H20
3	9.953	42	3	0.236	0.070	5.891	791	WS00831_N08
3	80.211	55	4	0.265	0.058	6.092	791	WS00831_N08
3	95.157	59	2	-0.206	0.029	4.360	791	WS00831_N08
3	104.681	60	2	0.159	0.035	4.283	791	WS00831_N08
3	112.868	61	2	-0.158	0.033	4.079	791	WS00831_N08
3	137.891	64	3	0.269	0.062	5.329	791	WS00831_N08
3	141.443	65	3	0.321	0.109	5.477	791	WS00831_N08
7	75.883	144	2	-0.413	0.054	4.893	791	WS00831_N08
9	17.951	169	1	0.354	0.075	4.973	791	WS00831_N08
10	80.088	194	1	-0.264	0.062	4.065	791	WS00831_N08
10	95.795	197	4	-0.212	0.044	4.620	791	WS00831_N08
1	95.572	17	2	0.198	0.032	4.046	792	WS00831_O17
4	82.462	78	2	-0.193	0.046	5.932	792	WS00831_O17
5	52.820	99	3	-0.239	0.056	5.180	792	WS00831_O17
5	56.008	101	2	0.200	0.046	4.678	792	WS00831_O17
5	57.017	102	2	-0.261	0.041	5.308	792	WS00831_O17
9	28.608	170	2	0.249	0.037	4.707	792	WS00831_O17
11	0.000	199	2	0.259	0.041	5.200	792	WS00831_O17
11	17.088	203	2	-0.221	0.042	4.204	792	WS00831_O17
11	40.960	210	2	-0.366	0.065	4.248	792	WS00831_O17
12	22.441	227	3	0.176	0.042	4.068	792	WS00831_O17
13	3.386	245	1	-0.259	0.065	3.996	792	WS00831_O17
3	137.891	64	3	0.269	0.043	4.382	793	WS01027_J05
4	82.462	78	1	-0.151	0.045	4.026	793	WS01027_J05
4	173.929	88	2	-0.234	0.062	6.094	793	WS01027_J05

9	59.963	177	4	0.267	0.055	4.464	793 WS01027_J05
10	78.479	193	3	0.369	0.062	5.981	793 WS01027_J05
10	86.151	195	3	-0.179	0.051	4.948	793 WS01027_J05
12	6.242	234	2	-0.212	0.033	4.280	793 WS01027_J05
1	62.128	11	1	-0.414	0.087	3.920	794 WS01027_J20
2	48.839	29	4	0.193	0.049	3.968	794 WS01027_J20
8	50.001	154	2	-0.247	0.072	5.656	794 WS01027_J20
9	17.951	169	1	0.376	0.044	4.996	794 WS01027_J20
10	9.580	187	3	-0.226	0.061	3.856	794 WS01027_J20
11	64.014	222	3	-0.293	0.054	5.754	794 WS01027_J20
12	51.382	242	1	0.461	0.116	5.252	794 WS01027_J20
13	1.815	244	4	0.221	0.082	7.570	794 WS01027_J20
13	3.386	245	1	-0.280	0.034	3.911	794 WS01027_J20
3	15.147	44	2	0.277	0.072	4.093	795 WS01027_M03
4	0.000	71	4	0.201	0.044	3.895	795 WS01027_M03
4	173.929	88	2	-0.282	0.070	7.350	795 WS01027_M03
5	57.017	102	4	-0.260	0.056	5.098	795 WS01027_M03
6	81.996	123	3	0.354	0.102	5.530	795 WS01027_M03
8	87.463	158	2	-0.187	0.044	4.271	795 WS01027_M03
10	70.246	190	2	-0.209	0.043	4.458	795 WS01027_M03
1	2.788	2	4	-0.219	0.041	4.088	796 WS01027_M14
2	93.504	32	4	-0.427	0.109	8.279	796 WS01027_M14
2	120.617	38	3	-0.217	0.050	5.246	796 WS01027_M14
3	137.891	64	3	-0.198	0.045	5.172	796 WS01027_M14
3	141.443	65	3	-0.363	0.090	5.688	796 WS01027_M14
4	116.928	82	2	0.302	0.065	6.007	796 WS01027_M14
4	124.132	83	2	0.316	0.105	4.158	796 WS01027_M14
5	84.347	105	1	0.233	0.057	5.454	796 WS01027_M14
6	81.996	123	3	0.319	0.061	3.849	796 WS01027_M14
9	55.358	175	3	0.448	0.085	5.140	796 WS01027_M14
9	59.963	177	4	0.266	0.053	3.919	796 WS01027_M14
11	44.884	215	3	-0.271	0.038	4.062	796 WS01027_M14
13	0.000	243	4	0.395	0.230	7.733	796 WS01027_M14
2	127.147	40	4	-0.278	0.071	4.502	797 WS01027_O24
6	95.249	126	1	0.292	0.069	5.335	797 WS01027_O24
7	74.560	143	1	0.276	0.049	5.291	797 WS01027_O24
8	95.937	159	3	-0.211	0.061	6.044	797 WS01027_O24
11	27.405	204	1	0.244	0.045	4.879	797 WS01027_O24
11	45.989	216	1	-0.278	0.070	7.516	797 WS01027_O24
14	22.402	248	3	0.238	0.045	4.435	797 WS01027_O24
2	63.261	30	3	0.217	0.042	4.006	798 WS01027_P16
2	93.504	32	4	-0.299	0.057	5.178	798 WS01027_P16
2	101.076	35	4	0.256	0.068	7.757	798 WS01027_P16
3	47.928	49	1	-0.217	0.056	4.529	798 WS01027_P16
4	124.132	83	1	0.466	0.111	4.399	798 WS01027_P16
5	7.672	92	1	-0.284	0.066	6.568	798 WS01027_P16
6	26.350	115	2	-0.192	0.038	4.051	798 WS01027_P16
6	56.188	120	1	-0.210	0.053	4.323	798 WS01027_P16
8	78.389	157	1	-0.276	0.080	6.519	798 WS01027_P16
8	87.463	158	1	0.217	0.050	4.097	798 WS01027_P16
12	22.441	227	1	-0.383	0.113	10.095	798 WS01027_P16
12	22.441	227	3	0.407	0.115	11.125	798 WS01027_P16
3	9.953	42	3	0.247	0.060	5.927	799 WS00923_B07
4	55.925	76	1	-0.247	0.053	4.878	799 WS00923_B07

5	31.191	95	1	-0.208	0.045	4.448	799	WS00923_B07
9	36.227	165	3	-0.240	0.045	4.480	799	WS00923_B07
11	27.405	204	1	0.250	0.051	5.079	799	WS00923_B07
11	27.405	204	4	-0.234	0.051	4.537	799	WS00923_B07
11	45.989	216	1	-0.224	0.050	4.885	799	WS00923_B07
3	9.953	42	3	0.270	0.097	9.577	800	WS00923_B18
3	95.157	59	2	-0.241	0.048	5.780	800	WS00923_B18
5	3.484	90	2	-0.271	0.081	5.022	800	WS00923_B18
11	27.405	204	1	0.295	0.059	5.045	800	WS00923_B18
2	127.147	40	3	-0.144	0.037	4.219	801	WS00911_K02
3	83.563	56	3	-0.154	0.038	4.358	801	WS00911_K02
6	95.249	126	1	0.384	0.103	5.206	801	WS00911_K02
7	74.560	143	1	0.355	0.052	4.426	801	WS00911_K02
9	28.608	170	3	0.460	0.105	8.958	801	WS00911_K02
11	17.088	203	1	-0.366	0.116	9.465	801	WS00911_K02
11	17.088	203	2	-0.187	0.042	4.511	801	WS00911_K02
11	27.405	204	1	0.458	0.136	11.697	801	WS00911_K02
11	29.069	205	1	0.355	0.089	7.373	801	WS00911_K02
11	45.989	216	1	-0.260	0.093	7.503	801	WS00911_K02
11	56.921	219	1	-0.246	0.131	4.125	801	WS00911_K02
12	14.406	230	3	-0.254	0.035	4.031	801	WS00911_K02
14	0.000	246	1	0.355	0.106	8.627	801	WS00911_K02
1	22.419	4	4	-0.346	0.135	4.112	802	WS00911_P13
2	48.839	29	1	0.244	0.066	6.030	802	WS00911_P13
2	48.839	29	4	-0.248	0.054	4.807	802	WS00911_P13
3	9.953	42	4	0.190	0.043	3.844	802	WS00911_P13
3	17.158	45	1	0.256	0.056	4.979	802	WS00911_P13
3	47.928	49	1	0.247	0.056	4.971	802	WS00911_P13
3	137.891	64	1	-0.261	0.059	4.753	802	WS00911_P13
3	137.891	64	3	-0.219	0.047	4.119	802	WS00911_P13
3	148.745	68	1	0.414	0.099	8.746	802	WS00911_P13
3	154.505	69	1	0.500	0.162	14.376	802	WS00911_P13
6	95.249	126	1	0.345	0.105	6.127	802	WS00911_P13
7	73.169	142	1	0.514	0.143	4.246	802	WS00911_P13
14	22.402	248	4	-0.309	0.064	5.532	802	WS00911_P13
2	127.147	40	3	-0.202	0.039	4.087	803	WS00828_C24
3	9.953	42	3	0.264	0.063	6.611	803	WS00828_C24
4	55.925	76	1	-0.236	0.058	5.131	803	WS00828_C24
4	82.462	78	1	-0.226	0.044	4.199	803	WS00828_C24
6	49.100	118	2	-0.316	0.095	4.777	803	WS00828_C24
8	87.463	158	2	-0.268	0.087	8.074	803	WS00828_C24
9	59.963	177	4	0.269	0.054	4.432	803	WS00828_C24
9	81.073	183	1	0.289	0.047	4.588	803	WS00828_C24
1	40.464	7	3	0.228	0.037	4.036	804	WS01031_E02
2	95.880	34	1	0.343	0.055	5.183	804	WS01031_E02
3	104.681	60	2	0.182	0.048	4.461	804	WS01031_E02
3	112.868	61	2	-0.243	0.059	5.576	804	WS01031_E02
3	114.747	62	2	-0.232	0.070	6.550	804	WS01031_E02
4	75.958	77	3	-0.222	0.078	3.864	804	WS01031_E02
7	75.883	144	4	0.306	0.054	4.039	804	WS01031_E02
9	36.227	165	4	0.271	0.078	6.253	804	WS01031_E02
9	69.046	181	2	0.321	0.089	4.316	804	WS01031_E02
13	0.000	243	4	0.351	0.189	6.412	804	WS01031_E02
1	62.128	11	1	-0.690	0.181	9.357	805	WS00829_A04

3	148.745	68	4	-0.158	0.038	4.052	805	WS00829_A04
4	55.925	76	1	-0.276	0.064	5.361	805	WS00829_A04
4	82.462	78	1	-0.153	0.050	4.573	805	WS00829_A04
6	45.897	117	1	0.287	0.052	4.994	805	WS00829_A04
6	49.100	118	4	0.196	0.061	4.082	805	WS00829_A04
6	69.073	121	3	0.404	0.078	6.102	805	WS00829_A04
7	75.883	144	2	-0.465	0.092	6.053	805	WS00829_A04
13	1.815	244	4	0.273	0.074	9.190	805	WS00829_A04
13	3.386	245	1	-0.338	0.071	6.618	805	WS00829_A04
1	29.800	5	1	-0.248	0.056	6.718	806	WS01031_I05
2	120.617	38	3	-0.169	0.034	4.341	806	WS01031_I05
3	9.953	42	3	0.254	0.036	4.781	806	WS01031_I05
14	44.898	251	3	0.256	0.036	4.180	806	WS01031_I05
3	9.953	42	3	0.205	0.044	4.979	807	WS0074_J02
3	137.891	64	3	0.386	0.109	12.252	807	WS0074_J02
3	141.443	65	3	0.494	0.143	12.273	807	WS0074_J02
4	0.000	71	4	0.282	0.083	6.222	807	WS0074_J02
4	173.929	88	2	-0.177	0.057	4.088	807	WS0074_J02
9	17.951	169	1	0.247	0.040	4.661	807	WS0074_J02
11	27.405	204	1	0.324	0.068	8.100	807	WS0074_J02
11	29.069	205	1	0.274	0.059	6.840	807	WS0074_J02
11	45.989	216	1	-0.262	0.070	8.149	807	WS0074_J02
14	44.898	251	3	0.251	0.044	4.935	807	WS0074_J02
1	95.572	17	3	0.234	0.040	4.038	808	WS0074_J12
3	14.500	43	2	0.252	0.083	8.475	808	WS0074_J12
3	20.512	46	2	-0.260	0.044	4.639	808	WS0074_J12
5	84.347	105	1	0.304	0.094	8.146	808	WS0074_J12
7	15.555	130	2	0.292	0.048	4.964	808	WS0074_J12
11	43.491	214	3	0.246	0.053	5.495	808	WS0074_J12
11	44.884	215	3	-0.295	0.058	5.921	808	WS0074_J12
11	64.014	222	3	-0.339	0.072	7.464	808	WS0074_J12
12	0.000	226	3	0.280	0.092	9.343	808	WS0074_J12
12	22.441	227	3	-0.259	0.040	4.063	808	WS0074_J12
14	43.392	250	2	0.358	0.123	12.515	808	WS0074_J12
1	107.333	19	3	0.251	0.059	5.404	809	WS0074_M05
5	57.017	102	4	0.217	0.044	4.281	809	WS0074_M05
6	106.702	128	2	0.381	0.102	4.095	809	WS0074_M05
7	15.555	130	3	-0.188	0.038	3.867	809	WS0074_M05
9	0.000	166	2	0.241	0.052	4.490	809	WS0074_M05
9	28.608	170	3	0.297	0.061	5.558	809	WS0074_M05
10	104.214	198	4	0.301	0.065	6.125	809	WS0074_M05
3	137.891	64	3	0.214	0.036	4.191	810	WS0074_N04
3	141.443	65	3	0.247	0.056	4.891	810	WS0074_N04
4	173.929	88	2	-0.234	0.069	5.651	810	WS0074_N04
10	77.676	192	1	-0.233	0.037	3.920	810	WS0074_N04
10	80.088	194	1	-0.290	0.048	5.135	810	WS0074_N04
11	27.405	204	1	0.211	0.047	5.064	810	WS0074_N04
11	45.989	216	1	-0.220	0.045	4.780	810	WS0074_N04
14	44.898	251	3	0.267	0.043	5.156	810	WS0074_N04
3	9.953	42	3	0.196	0.035	4.416	811	WS0074_P14
3	95.157	59	2	-0.215	0.042	4.327	811	WS0074_P14
3	104.681	60	2	0.153	0.047	3.927	811	WS0074_P14
3	112.868	61	2	-0.147	0.049	4.150	811	WS0074_P14
3	114.747	62	2	-0.234	0.047	4.057	811	WS0074_P14

3	141.443	65	3	0.399	0.100	8.513	811	WS0074_P14
9	0.000	166	2	0.285	0.069	5.629	811	WS0074_P14
10	35.204	189	1	-0.308	0.053	5.533	811	WS0074_P14
10	77.676	192	1	-0.363	0.105	10.868	811	WS0074_P14
10	80.088	194	1	-0.341	0.101	10.353	811	WS0074_P14
14	44.898	251	3	0.355	0.076	9.771	811	WS0074_P14
2	126.181	39	4	0.432	0.137	4.916	812	WS0075_A08
2	127.147	40	4	-0.253	0.068	5.267	812	WS0075_A08
3	15.147	44	2	0.305	0.136	5.111	812	WS0075_A08
3	116.958	63	4	-0.225	0.055	4.218	812	WS0075_A08
5	86.669	106	4	-0.382	0.123	4.710	812	WS0075_A08
7	75.883	144	4	-0.330	0.084	4.632	812	WS0075_A08
11	42.600	213	3	-0.171	0.035	3.849	812	WS0075_A08
12	14.406	230	4	0.315	0.061	4.412	812	WS0075_A08
12	6.242	234	2	-0.147	0.051	4.088	812	WS0075_A08
2	93.504	32	4	-0.354	0.050	5.037	813	WS0075_C23
3	137.891	64	3	0.324	0.051	4.901	813	WS0075_C23
6	69.073	121	4	0.273	0.075	7.125	813	WS0075_C23
7	15.555	130	3	-0.284	0.044	4.059	813	WS0075_C23
7	73.169	142	2	0.485	0.091	5.508	813	WS0075_C23
7	74.560	143	2	-0.242	0.034	4.119	813	WS0075_C23
7	75.883	144	2	-0.541	0.130	8.318	813	WS0075_C23
8	78.389	157	1	-0.226	0.056	4.624	813	WS0075_C23
8	87.463	158	1	0.215	0.052	4.265	813	WS0075_C23
9	59.963	177	4	0.358	0.074	7.375	813	WS0075_C23
10	9.580	187	3	-0.165	0.076	3.886	813	WS0075_C23
10	104.214	198	4	0.283	0.034	5.002	813	WS0075_C23
4	82.462	78	1	-0.176	0.034	4.170	814	WS0075_E04
5	0.000	89	1	-0.294	0.037	4.706	814	WS0075_E04
7	15.555	130	3	-0.314	0.055	6.267	814	WS0075_E04
10	9.580	187	3	-0.361	0.083	7.468	814	WS0075_E04
10	80.088	194	1	-0.282	0.049	6.066	814	WS0075_E04
11	27.405	204	1	0.241	0.049	6.085	814	WS0075_E04
1	29.800	5	1	-0.229	0.058	4.877	815	WS00937_H07
2	120.617	38	4	-0.290	0.046	4.777	815	WS00937_H07
2	127.147	40	4	-0.176	0.049	4.777	815	WS00937_H07
4	0.000	71	4	0.186	0.040	3.904	815	WS00937_H07
4	173.929	88	2	-0.274	0.061	6.433	815	WS00937_H07
5	57.017	102	4	-0.220	0.050	4.944	815	WS00937_H07
8	87.463	158	2	-0.186	0.054	5.858	815	WS00937_H07
9	28.608	170	2	0.242	0.047	5.103	815	WS00937_H07
3	69.071	52	1	-0.265	0.061	4.176	816	WS00937_I05
4	103.719	81	4	-0.240	0.070	5.245	816	WS00937_I05
4	124.132	83	1	0.542	0.142	5.956	816	WS00937_I05
4	142.917	85	1	-0.351	0.067	7.480	816	WS00937_I05
6	25.914	114	1	-0.199	0.041	4.560	816	WS00937_I05
6	69.073	121	4	-0.287	0.058	4.131	816	WS00937_I05
9	3.127	168	1	-0.350	0.088	3.950	816	WS00937_I05
9	55.358	175	3	0.435	0.072	5.011	816	WS00937_I05
1	40.464	7	3	0.158	0.039	4.156	817	WS00937_L08
1	45.794	9	3	-0.295	0.070	7.563	817	WS00937_L08
3	9.953	42	3	0.209	0.066	7.075	817	WS00937_L08
3	137.891	64	3	0.270	0.039	4.204	817	WS00937_L08
6	95.249	126	1	0.320	0.059	4.241	817	WS00937_L08

11	27.405	204	1	0.396	0.082	7.896	817	WS00937_L08
11	29.069	205	1	0.279	0.058	5.552	817	WS00937_L08
11	45.989	216	1	-0.243	0.081	7.688	817	WS00937_L08
6	95.249	126	1	0.386	0.110	7.223	818	WS00937_M08
6	106.702	128	3	0.203	0.052	4.288	818	WS00937_M08
7	15.555	130	2	-0.259	0.067	5.980	818	WS00937_M08
11	27.405	204	1	0.351	0.070	7.403	818	WS00937_M08
11	27.405	204	4	-0.262	0.051	4.793	818	WS00937_M08
12	6.242	234	2	-0.244	0.045	4.756	818	WS00937_M08
4	55.925	76	1	-0.268	0.075	6.298	819	WS00937_P01
4	116.928	82	4	0.281	0.080	7.009	819	WS00937_P01
7	62.787	139	2	0.260	0.050	4.511	819	WS00937_P01
12	28.493	228	2	-0.377	0.085	5.377	819	WS00937_P01
1	62.128	11	1	-0.505	0.130	4.866	820	WS00937_P16
1	107.333	19	4	-0.238	0.044	4.521	820	WS00937_P16
3	14.500	43	1	-0.208	0.043	3.893	820	WS00937_P16
4	124.132	83	1	-0.508	0.138	5.169	820	WS00937_P16
4	142.917	85	1	0.400	0.071	6.507	820	WS00937_P16
4	142.917	85	3	-0.244	0.061	5.645	820	WS00937_P16
4	173.929	88	2	-0.240	0.066	6.321	820	WS00937_P16
6	54.066	119	4	-0.260	0.051	5.348	820	WS00937_P16
6	87.891	124	2	-0.228	0.055	5.270	820	WS00937_P16
6	89.401	125	2	-0.286	0.068	6.275	820	WS00937_P16
7	73.169	142	1	0.519	0.115	4.317	820	WS00937_P16
11	0.000	199	2	0.250	0.047	4.521	820	WS00937_P16
11	27.405	204	1	0.319	0.060	5.338	820	WS00937_P16
11	27.405	204	4	-0.269	0.060	6.498	820	WS00937_P16
1	123.316	20	4	0.208	0.067	4.058	821	WS01033_C09
2	48.839	29	1	-0.213	0.063	5.748	821	WS01033_C09
2	48.839	29	4	0.266	0.071	7.121	821	WS01033_C09
2	127.147	40	3	-0.255	0.045	4.261	821	WS01033_C09
4	124.132	83	1	-0.442	0.102	4.029	821	WS01033_C09
7	62.787	139	2	0.295	0.072	6.694	821	WS01033_C09
8	0.000	148	4	-0.257	0.039	4.037	821	WS01033_C09
9	0.000	166	1	0.114	0.043	3.863	821	WS01033_C09
11	40.960	210	2	0.424	0.088	5.868	821	WS01033_C09
12	28.493	228	2	-0.334	0.074	4.611	821	WS01033_C09
13	1.815	244	4	0.155	0.041	4.586	821	WS01033_C09
1	38.326	6	4	-0.167	0.039	4.518	822	WS01033_D22
1	40.464	7	4	0.194	0.047	4.532	822	WS01033_D22
2	95.880	34	1	0.294	0.049	4.108	822	WS01033_D22
2	102.814	36	1	0.300	0.087	7.363	822	WS01033_D22
3	47.928	49	1	0.271	0.058	4.953	822	WS01033_D22
3	148.745	68	4	0.278	0.063	5.829	822	WS01033_D22
9	55.358	175	3	0.394	0.072	4.184	822	WS01033_D22
11	45.989	216	1	0.255	0.053	4.518	822	WS01033_D22
12	6.242	234	2	0.153	0.033	4.352	822	WS01033_D22
14	43.392	250	1	0.310	0.081	4.699	822	WS01033_D22
1	95.572	17	3	-0.259	0.042	4.076	823	WS0076_I10
3	9.953	42	3	0.216	0.066	7.158	823	WS0076_I10
7	59.789	137	3	-0.204	0.038	4.136	823	WS0076_I10
10	9.580	187	3	0.309	0.099	6.771	823	WS0076_I10
10	78.479	193	3	0.338	0.043	4.771	823	WS0076_I10
11	27.405	204	1	0.351	0.087	8.334	823	WS0076_I10

12	14.406	230	3	-0.353	0.098	10.756	823	WS0076_I10
12	6.242	234	2	-0.229	0.035	3.933	823	WS0076_I10
1	40.464	7	3	0.270	0.044	6.461	824	WS0076_J07
1	43.578	8	3	0.250	0.037	4.010	824	WS0076_J07
1	45.794	9	3	-0.199	0.029	4.329	824	WS0076_J07
1	95.572	17	3	0.258	0.040	4.877	824	WS0076_J07
3	47.928	49	1	0.246	0.050	4.492	824	WS0076_J07
6	25.914	114	1	-0.243	0.044	3.993	824	WS0076_J07
6	45.897	117	1	0.227	0.046	4.172	824	WS0076_J07
9	55.358	175	3	0.342	0.051	3.961	824	WS0076_J07
1	43.578	8	2	-0.216	0.049	3.860	825	WS0076_L01
1	43.578	8	3	0.272	0.046	4.139	825	WS0076_L01
1	45.794	9	1	-0.243	0.047	5.217	825	WS0076_L01
2	93.504	32	4	-0.337	0.077	7.093	825	WS0076_L01
3	15.147	44	2	0.273	0.085	4.048	825	WS0076_L01
3	80.211	55	4	0.194	0.045	4.423	825	WS0076_L01
4	55.925	76	1	-0.311	0.095	10.634	825	WS0076_L01
4	124.132	83	1	0.589	0.143	7.421	825	WS0076_L01
4	142.917	85	1	-0.282	0.041	4.584	825	WS0076_L01
6	49.100	118	4	0.406	0.122	5.166	825	WS0076_L01
7	15.555	130	3	0.223	0.059	4.141	825	WS0076_L01
7	74.560	143	1	0.302	0.064	7.151	825	WS0076_L01
8	50.001	154	1	-0.249	0.036	4.048	825	WS0076_L01
9	59.963	177	4	0.239	0.046	4.603	825	WS0076_L01
11	9.237	202	4	0.244	0.062	6.215	825	WS0076_L01
11	27.405	204	4	-0.302	0.067	5.811	825	WS0076_L01
12	22.441	227	1	-0.261	0.054	6.210	825	WS0076_L01
12	22.441	227	3	0.311	0.044	3.867	825	WS0076_L01
1	95.572	17	2	-0.233	0.043	4.457	826	WS0076_L15
1	107.333	19	4	-0.248	0.047	5.144	826	WS0076_L15
2	95.880	34	3	0.236	0.053	3.937	826	WS0076_L15
5	86.669	106	4	0.393	0.122	5.610	826	WS0076_L15
6	69.073	121	4	-0.230	0.044	4.347	826	WS0076_L15
8	50.001	154	2	-0.204	0.039	4.107	826	WS0076_L15
9	36.227	165	4	0.327	0.066	5.675	826	WS0076_L15
12	22.441	227	3	0.216	0.053	3.975	826	WS0076_L15
14	9.923	247	1	-0.308	0.081	6.865	826	WS0076_L15
3	14.500	43	1	-0.275	0.075	6.380	827	WS0076_N06
3	15.147	44	2	0.184	0.077	5.634	827	WS0076_N06
3	20.512	46	2	0.264	0.047	5.026	827	WS0076_N06
4	82.462	78	1	-0.216	0.053	4.531	827	WS0076_N06
4	116.928	82	2	-0.220	0.042	4.227	827	WS0076_N06
4	142.917	85	1	0.314	0.071	6.056	827	WS0076_N06
5	54.072	100	4	0.288	0.067	6.417	827	WS0076_N06
5	57.017	102	4	-0.307	0.087	7.018	827	WS0076_N06
6	45.897	117	1	-0.255	0.056	5.249	827	WS0076_N06
8	95.937	159	3	0.246	0.048	4.469	827	WS0076_N06
11	0.000	199	2	0.437	0.097	10.428	827	WS0076_N06
11	0.000	199	3	-0.382	0.100	9.867	827	WS0076_N06
11	17.088	203	1	-0.311	0.050	4.282	827	WS0076_N06
11	17.088	203	2	-0.310	0.134	13.018	827	WS0076_N06
11	27.405	204	1	0.331	0.084	7.385	827	WS0076_N06
11	29.069	205	1	0.225	0.056	4.747	827	WS0076_N06
11	43.491	214	4	-0.210	0.063	6.181	827	WS0076_N06

13	1.815	244	2	0.233	0.045	4.749	827	WS0076_N06
2	101.076	35	3	-0.208	0.030	3.841	828	WS0076_N18
2	120.617	38	3	-0.345	0.101	13.001	828	WS0076_N18
2	127.147	40	3	0.243	0.068	8.782	828	WS0076_N18
2	127.147	40	4	-0.166	0.074	4.298	828	WS0076_N18
3	9.953	42	3	0.308	0.051	6.765	828	WS0076_N18
5	0.000	89	1	-0.228	0.041	4.401	828	WS0076_N18
9	36.227	165	3	0.290	0.039	5.101	828	WS0076_N18
10	93.347	196	1	-0.439	0.059	5.395	828	WS0076_N18
11	43.491	214	3	0.298	0.065	8.424	828	WS0076_N18
11	44.884	215	3	-0.346	0.058	7.547	828	WS0076_N18
11	64.014	222	3	-0.355	0.060	7.823	828	WS0076_N18
14	44.898	251	3	0.337	0.068	8.638	828	WS0076_N18
1	22.419	4	4	0.411	0.109	4.474	829	WS0076_P07
2	75.550	31	1	-0.302	0.046	5.062	829	WS0076_P07
6	81.996	123	3	0.327	0.061	4.130	829	WS0076_P07
11	17.088	203	1	0.551	0.236	25.736	829	WS0076_P07
11	27.405	204	1	-0.617	0.215	23.695	829	WS0076_P07
11	29.069	205	1	-0.576	0.236	25.711	829	WS0076_P07
11	45.989	216	1	0.333	0.112	12.162	829	WS0076_P07
1	29.800	5	1	-0.235	0.047	5.542	830	WS0076_P18
2	120.617	38	3	-0.142	0.030	3.935	830	WS0076_P18
3	83.563	56	3	0.125	0.038	4.832	830	WS0076_P18
5	0.000	89	3	0.362	0.065	5.023	830	WS0076_P18
6	87.891	124	2	0.178	0.057	3.975	830	WS0076_P18
8	50.001	154	2	-0.217	0.090	4.552	830	WS0076_P18
11	43.491	214	3	0.304	0.056	7.366	830	WS0076_P18
11	44.884	215	3	-0.309	0.043	5.675	830	WS0076_P18
11	64.014	222	3	-0.269	0.034	4.438	830	WS0076_P18
14	9.923	247	1	0.215	0.036	4.216	830	WS0076_P18
4	82.462	78	1	-0.291	0.057	6.043	831	WS00821_O10
5	7.672	92	1	-0.276	0.059	6.460	831	WS00821_O10
5	16.219	93	1	-0.218	0.041	4.279	831	WS00821_O10
8	39.090	152	4	0.196	0.049	4.081	831	WS00821_O10
10	9.580	187	3	-0.343	0.073	4.360	831	WS00821_O10
10	77.676	192	1	-0.261	0.070	7.422	831	WS00821_O10
10	80.088	194	1	-0.247	0.050	5.327	831	WS00821_O10
12	22.629	237	1	0.198	0.049	5.136	831	WS00821_O10
14	22.402	248	4	0.325	0.070	5.882	831	WS00821_O10
1	45.794	9	1	0.400	0.075	7.131	832	WS01027_E10
4	92.039	79	4	-0.372	0.069	5.469	832	WS01027_E10
4	97.576	80	2	0.148	0.043	3.865	832	WS01027_E10
4	103.719	81	4	-0.216	0.035	3.885	832	WS01027_E10
4	124.132	83	1	0.435	0.065	4.012	832	WS01027_E10
5	0.000	89	1	0.335	0.059	5.629	832	WS01027_E10
5	31.191	95	1	0.242	0.041	3.901	832	WS01027_E10
9	0.000	166	1	-0.205	0.045	4.215	832	WS01027_E10
11	9.237	202	4	0.292	0.058	6.582	832	WS01027_E10
11	64.014	222	4	0.289	0.054	5.730	832	WS01027_E10
12	22.441	227	1	-0.289	0.075	7.078	832	WS01027_E10
12	6.242	234	2	0.269	0.055	4.969	832	WS01027_E10
12	6.242	234	4	-0.375	0.065	8.388	832	WS01027_E10
1	45.794	9	1	0.219	0.050	3.901	833	WS00823_J05
3	137.891	64	3	0.369	0.060	7.007	833	WS00823_J05

4	103.719	81	4	-0.242	0.042	3.870	833	WS00823_J05
10	21.864	188	3	0.176	0.039	4.510	833	WS00823_J05
10	70.246	190	2	0.220	0.053	5.854	833	WS00823_J05
2	48.839	29	1	-0.209	0.045	4.287	834	WS00823_O02
3	116.958	63	4	-0.182	0.077	5.495	834	WS00823_O02
4	82.462	78	1	-0.248	0.086	8.020	834	WS00823_O02
5	0.000	89	1	-0.351	0.068	6.481	834	WS00823_O02
3	14.500	43	2	-0.206	0.044	6.310	835	WS00822_N22
3	20.512	46	2	0.312	0.054	6.191	835	WS00822_N22
6	45.897	117	1	-0.393	0.081	7.002	835	WS00822_N22
6	49.100	118	4	-0.550	0.354	8.607	835	WS00822_N22
6	54.066	119	4	-0.271	0.035	4.153	835	WS00822_N22
6	95.249	126	1	0.430	0.078	4.511	835	WS00822_N22
9	28.608	170	3	0.324	0.065	3.997	835	WS00822_N22
9	49.013	173	2	0.129	0.046	5.088	835	WS00822_N22
10	93.347	196	1	0.433	0.085	4.508	835	WS00822_N22
11	0.000	199	2	0.346	0.064	7.327	835	WS00822_N22
11	27.405	204	1	0.323	0.043	3.966	835	WS00822_N22
14	43.392	250	2	-0.205	0.025	4.015	835	WS00822_N22
1	43.578	8	2	-0.283	0.055	7.407	836	WS00823_D04
1	123.316	20	4	0.421	0.120	11.243	836	WS00823_D04
3	80.211	55	4	0.212	0.045	5.052	836	WS00823_D04
10	9.580	187	2	-0.249	0.052	4.893	836	WS00823_D04
10	93.347	196	1	0.403	0.109	3.906	836	WS00823_D04
11	27.405	204	1	0.321	0.103	5.734	836	WS00823_D04
11	27.405	204	4	-0.363	0.083	9.559	836	WS00823_D04
12	6.242	234	2	-0.154	0.016	4.351	836	WS00823_D04
13	3.386	245	1	-0.210	0.073	3.967	836	WS00823_D04
1	29.800	5	1	0.241	0.055	3.876	837	WS00916_J06
2	75.550	31	2	0.265	0.049	5.833	837	WS00916_J06
2	126.181	39	4	-0.567	0.120	10.178	837	WS00916_J06
5	7.672	92	4	-0.206	0.032	3.859	837	WS00916_J06
6	87.891	124	2	0.234	0.041	4.864	837	WS00916_J06
6	89.401	125	2	0.225	0.046	5.437	837	WS00916_J06
11	0.000	199	2	0.261	0.051	6.032	837	WS00916_J06
11	0.000	199	3	-0.275	0.058	4.177	837	WS00916_J06
11	40.960	210	2	0.447	0.066	5.489	837	WS00916_J06
13	0.000	243	4	0.330	0.111	3.890	837	WS00916_J06
14	22.402	248	4	0.211	0.034	3.999	837	WS00916_J06
1	43.578	8	2	0.251	0.043	4.749	838	WS00916_O17
1	45.794	9	1	0.292	0.052	4.264	838	WS00916_O17
1	45.794	9	3	-0.227	0.042	3.845	838	WS00916_O17
2	93.504	32	4	0.418	0.084	8.327	838	WS00916_O17
5	3.484	90	2	0.299	0.079	4.718	838	WS00916_O17
5	56.008	101	1	0.253	0.050	4.067	838	WS00916_O17
6	26.350	115	2	-0.197	0.042	4.564	838	WS00916_O17
6	49.100	118	2	0.356	0.097	4.775	838	WS00916_O17
6	49.100	118	4	-0.408	0.204	7.856	838	WS00916_O17
6	106.702	128	2	0.721	0.249	13.223	838	WS00916_O17
8	39.090	152	4	0.295	0.103	10.119	838	WS00916_O17
9	3.127	168	1	-0.381	0.110	4.434	838	WS00916_O17
9	28.608	170	3	0.281	0.054	4.450	838	WS00916_O17
9	59.963	177	4	-0.328	0.072	6.958	838	WS00916_O17
11	17.088	203	1	-0.326	0.105	8.353	838	WS00916_O17

11	27.405	204	1	0.377	0.092	7.216	838	WS00916_O17
11	29.069	205	1	0.338	0.104	8.253	838	WS00916_O17
11	45.989	216	1	-0.247	0.079	6.283	838	WS00916_O17
1	40.464	7	3	0.220	0.060	6.675	839	WS01029_F10
1	43.578	8	3	0.244	0.070	8.079	839	WS01029_F10
1	45.794	9	3	-0.229	0.052	6.206	839	WS01029_F10
3	47.928	49	1	0.228	0.047	4.558	839	WS01029_F10
5	57.017	102	2	0.179	0.046	4.234	839	WS01029_F10
9	28.608	170	3	0.310	0.043	4.796	839	WS01029_F10
10	93.347	196	1	0.441	0.098	4.661	839	WS01029_F10
12	22.629	237	1	0.239	0.058	5.638	839	WS01029_F10
12	32.473	238	1	0.215	0.045	4.339	839	WS01029_F10
14	0.000	246	1	0.244	0.051	4.921	839	WS01029_F10
14	22.402	248	4	0.329	0.053	4.374	839	WS01029_F10
1	95.572	17	2	-0.352	0.099	9.866	840	WS01029_G14
3	114.747	62	2	0.203	0.039	3.878	840	WS01029_G14
4	55.925	76	1	0.261	0.080	7.228	840	WS01029_G14
6	56.188	120	1	0.213	0.048	4.221	840	WS01029_G14
6	95.249	126	1	-0.234	0.077	4.774	840	WS01029_G14
8	39.090	152	4	0.283	0.078	7.616	840	WS01029_G14
8	87.463	158	2	0.252	0.076	7.108	840	WS01029_G14
10	21.864	188	1	-0.258	0.046	4.041	840	WS01029_G14
11	0.000	199	3	-0.304	0.052	4.632	840	WS01029_G14
11	9.237	202	4	-0.244	0.052	5.132	840	WS01029_G14
12	22.441	227	3	-0.234	0.050	4.496	840	WS01029_G14
1	50.955	10	1	-0.243	0.062	5.013	841	WS01029_I09
4	82.462	78	1	-0.175	0.055	4.408	841	WS01029_I09
4	116.928	82	2	-0.271	0.045	4.794	841	WS01029_I09
5	42.979	97	1	-0.310	0.052	4.249	841	WS01029_I09
6	25.914	114	1	0.285	0.084	6.802	841	WS01029_I09
8	0.000	148	4	-0.218	0.052	4.563	841	WS01029_I09
10	104.214	198	2	-0.162	0.034	3.951	841	WS01029_I09
13	0.000	243	4	0.281	0.160	4.238	841	WS01029_I09
3	137.891	64	3	0.263	0.035	4.321	842	WS01029_I17
3	141.443	65	3	0.372	0.078	7.595	842	WS01029_I17
9	17.951	169	1	0.278	0.046	5.117	842	WS01029_I17
9	17.951	169	2	-0.244	0.064	4.949	842	WS01029_I17
10	77.676	192	1	-0.198	0.038	4.259	842	WS01029_I17
10	80.088	194	1	-0.224	0.043	4.726	842	WS01029_I17
4	124.132	83	1	-0.482	0.093	4.805	843	WS01029_K03
9	28.608	170	2	0.323	0.071	6.925	843	WS01029_K03
3	137.891	64	3	0.251	0.043	4.630	844	WS01029_K15
3	141.443	65	3	0.265	0.053	5.275	844	WS01029_K15
9	81.073	183	1	0.247	0.039	4.736	844	WS01029_K15
11	27.405	204	1	0.237	0.055	6.548	844	WS01029_K15
11	44.884	215	3	-0.223	0.034	4.315	844	WS01029_K15
11	64.014	222	3	-0.251	0.047	5.905	844	WS01029_K15
3	95.157	59	2	-0.237	0.031	3.979	845	WS01029_M07
3	141.443	65	3	0.382	0.072	4.877	845	WS01029_M07
6	89.401	125	2	-0.317	0.058	5.499	845	WS01029_M07
9	81.073	183	1	0.189	0.046	4.510	845	WS01029_M07
11	27.405	204	1	0.435	0.102	10.654	845	WS01029_M07
14	0.000	246	1	-0.141	0.049	4.865	845	WS01029_M07
4	82.462	78	1	-0.221	0.053	6.251	846	WS01029_N02

5	0.000	89	1	-0.274	0.035	4.214	846	WS01029_N02
9	69.046	181	2	0.341	0.226	6.764	846	WS01029_N02
10	9.580	187	3	-0.272	0.053	5.124	846	WS01029_N02
10	77.676	192	1	-0.271	0.045	5.334	846	WS01029_N02
10	80.088	194	1	-0.300	0.058	6.867	846	WS01029_N02
14	44.898	251	3	0.241	0.029	4.043	846	WS01029_N02
4	82.462	78	1	-0.247	0.054	4.980	847	WS01031_L13
4	140.962	84	3	0.249	0.067	6.329	847	WS01031_L13
4	142.917	85	3	0.277	0.046	4.662	847	WS01031_L13
7	75.883	144	2	0.387	0.071	3.946	847	WS01031_L13
8	39.090	152	4	0.258	0.054	5.028	847	WS01031_L13
8	78.389	157	1	0.274	0.061	5.629	847	WS01031_L13
8	87.463	158	1	-0.275	0.065	6.021	847	WS01031_L13
9	0.000	163	2	0.493	0.116	4.858	847	WS01031_L13
10	9.580	187	3	-0.272	0.087	4.504	847	WS01031_L13
12	6.242	234	2	0.238	0.052	5.492	847	WS01031_L13
13	3.386	245	1	0.360	0.118	10.833	847	WS01031_L13
4	43.146	74	3	0.197	0.043	5.062	848	WS01031_L21
4	75.958	77	3	-0.239	0.076	4.248	848	WS01031_L21
5	16.219	93	2	0.285	0.056	5.898	848	WS01031_L21
1	82.301	16	3	0.168	0.045	4.224	849	WS01031_N02
4	140.962	84	3	0.228	0.047	4.418	849	WS01031_N02
5	57.017	102	2	-0.246	0.033	3.891	849	WS01031_N02
7	15.555	130	2	0.218	0.033	3.891	849	WS01031_N02
8	87.463	158	2	0.244	0.061	7.141	849	WS01031_N02
10	70.246	190	2	0.171	0.036	4.055	849	WS01031_N02
12	38.543	232	4	0.438	0.138	5.276	849	WS01031_N02
12	6.242	234	2	0.214	0.047	5.469	849	WS01031_N02
12	6.242	234	4	-0.252	0.065	6.285	849	WS01031_N02
14	43.392	250	2	0.275	0.079	8.978	849	WS01031_N02
3	114.747	62	2	-0.188	0.046	4.804	850	WS01031_N15
4	173.929	88	2	-0.198	0.039	4.084	850	WS01031_N15
5	3.484	90	2	-0.282	0.072	3.977	850	WS01031_N15
6	81.996	123	3	0.392	0.112	6.982	850	WS01031_N15
9	59.963	177	4	0.272	0.061	4.166	850	WS01031_N15
9	69.046	181	2	0.344	0.123	5.567	850	WS01031_N15
11	45.989	216	1	-0.213	0.048	3.939	850	WS01031_N15
3	9.953	42	3	0.273	0.057	5.893	851	WS01031_P13
4	0.000	71	4	0.219	0.047	4.023	851	WS01031_P13
4	173.929	88	2	-0.213	0.041	4.437	851	WS01031_P13
6	81.996	123	3	0.407	0.142	8.414	851	WS01031_P13
7	70.278	141	3	-0.220	0.052	5.349	851	WS01031_P13
8	87.463	158	2	-0.220	0.054	5.751	851	WS01031_P13
2	101.076	35	4	0.153	0.034	4.092	852	WS00825_A07
3	9.953	42	3	0.167	0.063	7.008	852	WS00825_A07
3	112.868	61	2	-0.215	0.036	4.008	852	WS00825_A07
8	50.001	154	2	-0.248	0.043	5.027	852	WS00825_A07
9	0.000	166	1	-0.336	0.139	10.816	852	WS00825_A07
9	17.951	169	1	0.332	0.058	4.675	852	WS00825_A07
1	95.572	17	2	-0.257	0.041	4.450	853	WS00825_B13
2	63.261	30	3	0.272	0.071	6.809	853	WS00825_B13
2	95.880	34	3	0.295	0.061	5.318	853	WS00825_B13
3	15.147	44	2	-0.316	0.104	5.700	853	WS00825_B13
12	22.629	237	1	0.229	0.061	4.878	853	WS00825_B13

13	1.815	244	2	-0.221	0.037	4.109	853	WS00825_B13
3	80.211	55	4	-0.215	0.037	3.960	854	WS00825_C06
3	83.563	56	3	-0.205	0.051	4.158	854	WS00825_C06
4	0.000	71	4	0.201	0.040	4.282	854	WS00825_C06
4	92.039	79	4	0.221	0.041	4.338	854	WS00825_C06
4	124.132	83	1	-0.433	0.088	4.090	854	WS00825_C06
5	54.072	100	4	-0.238	0.059	6.336	854	WS00825_C06
5	57.017	102	4	0.234	0.056	5.068	854	WS00825_C06
6	25.914	114	1	0.205	0.048	4.377	854	WS00825_C06
8	39.090	152	4	0.199	0.039	4.215	854	WS00825_C06
9	0.000	166	2	0.208	0.057	5.224	854	WS00825_C06
11	0.000	199	3	-0.419	0.108	5.525	854	WS00825_C06
11	17.088	203	1	-0.259	0.055	4.970	854	WS00825_C06
11	27.405	204	1	0.312	0.050	4.585	854	WS00825_C06
11	43.491	214	3	0.227	0.074	5.997	854	WS00825_C06
14	22.402	248	4	0.273	0.039	4.205	854	WS00825_C06
14	44.898	251	1	-0.297	0.060	5.400	854	WS00825_C06
2	48.839	29	4	0.354	0.077	9.112	855	WS01012_I21
3	116.958	63	4	-0.214	0.038	4.627	855	WS01012_I21
3	141.443	65	4	0.310	0.100	4.069	855	WS01012_I21
6	26.350	115	2	0.173	0.040	4.386	855	WS01012_I21
6	87.891	124	2	-0.218	0.035	3.939	855	WS01012_I21
7	105.477	147	2	-0.315	0.083	6.639	855	WS01012_I21
9	55.358	175	1	0.291	0.067	5.141	855	WS01012_I21
9	55.358	175	3	-0.478	0.146	5.912	855	WS01012_I21
11	27.405	204	4	-0.270	0.051	6.098	855	WS01012_I21
11	41.984	212	2	-0.161	0.036	3.977	855	WS01012_I21
12	38.543	232	4	-0.465	0.188	7.684	855	WS01012_I21
12	6.242	234	2	-0.151	0.028	3.941	855	WS01012_I21
14	22.402	248	4	-0.432	0.084	9.519	855	WS01012_I21
14	44.898	251	1	-0.287	0.060	4.802	855	WS01012_I21
1	40.464	7	3	0.300	0.056	4.496	856	WS01012_J04
1	43.578	8	3	0.301	0.068	5.750	856	WS01012_J04
3	47.928	49	1	0.220	0.051	5.081	856	WS01012_J04
4	92.039	79	4	-0.221	0.045	4.255	856	WS01012_J04
4	103.719	81	4	-0.294	0.055	6.373	856	WS01012_J04
5	86.669	106	2	0.251	0.130	4.501	856	WS01012_J04
6	25.914	114	1	0.263	0.053	5.373	856	WS01012_J04
6	76.376	122	1	0.416	0.161	8.899	856	WS01012_J04
11	9.237	202	4	0.285	0.048	5.334	856	WS01012_J04
11	27.405	204	4	-0.298	0.081	6.659	856	WS01012_J04
11	40.960	210	2	0.460	0.106	6.183	856	WS01012_J04
11	40.960	210	4	-0.470	0.122	13.803	856	WS01012_J04
11	43.491	214	4	-0.262	0.041	4.437	856	WS01012_J04
11	44.884	215	4	0.354	0.055	6.180	856	WS01012_J04
13	1.815	244	4	0.182	0.034	3.915	856	WS01012_J04
2	63.261	30	3	0.233	0.053	4.235	857	WS01012_K15
3	20.512	46	2	0.243	0.050	5.744	857	WS01012_K15
4	0.000	71	4	0.205	0.039	4.164	857	WS01012_K15
6	87.891	124	2	-0.183	0.034	3.852	857	WS01012_K15
9	59.963	177	4	0.230	0.038	4.305	857	WS01012_K15
11	27.405	204	1	0.367	0.116	9.015	857	WS01012_K15
11	27.405	204	4	-0.322	0.079	8.451	857	WS01012_K15
11	29.069	205	1	0.241	0.058	4.276	857	WS01012_K15

11	45.989	216	1	-0.262	0.093	6.872	857	WS01012_K15
1	2.788	2	4	-0.193	0.064	3.948	858	WS01012_L04
3	112.868	61	2	-0.264	0.072	6.484	858	WS01012_L04
3	114.747	62	2	-0.201	0.059	5.301	858	WS01012_L04
11	27.405	204	1	0.361	0.104	10.338	858	WS01012_L04
11	27.405	204	4	-0.243	0.059	4.049	858	WS01012_L04
11	45.989	216	1	-0.201	0.056	5.424	858	WS01012_L04
1	95.572	17	2	0.231	0.040	4.401	859	WS01012_M24
1	95.572	17	3	-0.221	0.039	3.927	859	WS01012_M24
2	63.261	30	3	0.227	0.051	4.989	859	WS01012_M24
3	9.953	42	3	0.211	0.046	4.547	859	WS01012_M24
3	15.147	44	2	0.260	0.066	4.247	859	WS01012_M24
3	20.512	46	2	0.203	0.041	4.496	859	WS01012_M24
3	104.681	60	2	0.182	0.036	3.968	859	WS01012_M24
3	112.868	61	2	-0.190	0.039	4.223	859	WS01012_M24
3	114.747	62	2	-0.222	0.046	5.071	859	WS01012_M24
4	173.929	88	2	-0.176	0.036	3.922	859	WS01012_M24
10	70.246	190	2	-0.291	0.057	6.316	859	WS01012_M24
11	27.405	204	1	0.359	0.121	9.923	859	WS01012_M24
11	29.069	205	1	0.220	0.068	5.355	859	WS01012_M24
11	45.989	216	1	-0.209	0.061	4.829	859	WS01012_M24
12	22.441	227	3	0.227	0.041	4.028	859	WS01012_M24
12	6.242	234	2	-0.317	0.064	8.456	859	WS01012_M24
1	43.578	8	2	0.284	0.040	4.316	860	WS01012_N13
1	69.854	13	2	-0.144	0.046	4.806	860	WS01012_N13
1	71.592	14	2	-0.130	0.040	4.183	860	WS01012_N13
1	95.572	17	2	-0.176	0.046	4.762	860	WS01012_N13
5	0.000	89	3	-0.225	0.043	4.572	860	WS01012_N13
9	17.951	169	2	0.241	0.074	7.990	860	WS01012_N13
9	28.608	170	2	-0.378	0.090	9.884	860	WS01012_N13
9	28.608	170	3	0.256	0.047	4.799	860	WS01012_N13
9	32.356	171	2	-0.274	0.058	6.207	860	WS01012_N13
11	27.405	204	1	0.362	0.077	6.507	860	WS01012_N13
11	45.989	216	1	-0.178	0.070	5.790	860	WS01012_N13
1	2.788	2	4	-0.189	0.051	3.976	861	WS01013_A01
3	9.953	42	3	0.194	0.044	4.533	861	WS01013_A01
3	137.891	64	3	0.260	0.044	4.497	861	WS01013_A01
8	50.001	154	1	-0.185	0.043	4.249	861	WS01013_A01
10	78.479	193	3	0.343	0.055	5.773	861	WS01013_A01
11	27.405	204	1	0.383	0.108	10.464	861	WS01013_A01
11	27.405	204	4	-0.348	0.101	8.709	861	WS01013_A01
11	29.069	205	1	0.210	0.050	4.757	861	WS01013_A01
11	45.989	216	1	-0.270	0.089	8.523	861	WS01013_A01
12	6.242	234	2	-0.230	0.043	5.047	861	WS01013_A01
1	15.589	3	2	-0.262	0.041	4.165	862	WS01013_A22
6	69.073	121	3	0.221	0.052	5.355	862	WS01013_A22
8	87.463	158	2	-0.099	0.035	4.190	862	WS01013_A22
8	95.937	159	3	-0.225	0.039	4.068	862	WS01013_A22
9	0.000	163	1	0.591	0.122	7.800	862	WS01013_A22
9	28.608	170	2	0.310	0.052	5.226	862	WS01013_A22
9	59.963	177	4	0.351	0.069	5.452	862	WS01013_A22
11	45.989	216	1	-0.344	0.073	6.932	862	WS01013_A22
11	60.464	221	1	0.227	0.060	5.545	862	WS01013_A22
1	2.788	2	4	-0.214	0.044	4.174	863	WS01011_B04

3	15.147	44	2	0.303	0.074	5.160	863	WS01011_B04
4	0.000	71	4	0.231	0.042	4.348	863	WS01011_B04
4	124.132	83	1	-0.496	0.144	4.941	863	WS01011_B04
4	142.917	85	1	0.295	0.050	4.587	863	WS01011_B04
4	173.929	88	2	-0.225	0.060	5.835	863	WS01011_B04
9	59.963	177	4	0.330	0.077	7.460	863	WS01011_B04
10	78.479	193	3	0.313	0.050	4.269	863	WS01011_B04
1	2.788	2	3	-0.390	0.201	25.851	864	WS01011_B16
1	71.592	14	2	0.287	0.058	5.031	864	WS01011_B16
1	77.177	15	2	0.284	0.055	4.729	864	WS01011_B16
3	141.443	65	3	0.422	0.094	7.513	864	WS01011_B16
5	0.000	89	1	-0.298	0.036	4.071	864	WS01011_B16
5	0.000	89	3	0.257	0.033	4.478	864	WS01011_B16
6	69.073	121	3	-0.166	0.033	4.241	864	WS01011_B16
7	54.200	136	2	0.215	0.090	4.506	864	WS01011_B16
7	62.787	139	2	0.270	0.068	5.976	864	WS01011_B16
9	81.073	183	1	0.208	0.056	5.984	864	WS01011_B16
10	77.676	192	1	-0.268	0.052	5.507	864	WS01011_B16
10	80.088	194	1	-0.224	0.053	5.499	864	WS01011_B16
11	27.405	204	1	0.316	0.063	6.865	864	WS01011_B16
1	2.788	2	3	-0.191	0.052	6.334	865	WS01011_D20
4	142.917	85	1	0.183	0.042	4.016	866	WS01011_E16
6	95.249	126	1	-0.277	0.067	5.312	866	WS01011_E16
7	15.555	130	2	0.212	0.046	4.070	866	WS01011_E16
9	0.000	163	1	-0.441	0.054	4.325	866	WS01011_E16
9	36.227	165	3	0.207	0.033	4.341	866	WS01011_E16
10	77.676	192	1	-0.250	0.041	4.111	866	WS01011_E16
10	80.088	194	1	-0.269	0.040	3.994	866	WS01011_E16
11	43.491	214	3	0.325	0.071	9.293	866	WS01011_E16
11	44.884	215	3	-0.256	0.046	6.005	866	WS01011_E16
11	64.014	222	3	-0.292	0.062	8.116	866	WS01011_E16
12	6.242	234	2	0.185	0.035	4.344	866	WS01011_E16
2	48.839	29	1	-0.377	0.107	9.086	867	WS01011_H04
3	20.512	46	2	0.229	0.053	5.478	867	WS01011_H04
6	89.401	125	1	-0.268	0.071	5.954	867	WS01011_H04
10	70.246	190	4	-0.298	0.093	8.331	867	WS01011_H04
10	78.479	193	4	-0.242	0.087	5.176	867	WS01011_H04
2	48.839	29	1	-0.321	0.064	6.531	868	WS01011_H24
2	48.839	29	4	0.311	0.102	7.842	868	WS01011_H24
3	116.958	63	4	-0.289	0.106	7.903	868	WS01011_H24
3	137.891	64	3	0.310	0.052	5.894	868	WS01011_H24
5	7.672	92	1	-0.228	0.038	4.110	868	WS01011_H24
7	74.560	143	1	0.245	0.040	4.010	868	WS01011_H24
10	80.088	194	1	-0.260	0.055	5.457	868	WS01011_H24
1	50.955	10	1	-0.257	0.058	5.900	869	WS01011_J24
3	14.500	43	2	-0.357	0.104	6.869	869	WS01011_J24
3	20.512	46	2	0.275	0.100	8.712	869	WS01011_J24
7	105.477	147	2	-0.300	0.115	4.096	869	WS01011_J24
10	9.580	187	3	-0.253	0.071	5.065	869	WS01011_J24
10	80.088	194	1	-0.299	0.046	4.702	869	WS01011_J24
11	17.088	203	1	0.288	0.079	7.925	869	WS01011_J24
7	15.555	130	3	-0.321	0.068	8.515	870	WS01011_L10
9	36.227	165	3	0.200	0.036	4.825	870	WS01011_L10
12	14.406	230	3	-0.222	0.038	5.091	870	WS01011_L10

1	50.955	10	1	-0.266	0.060	5.687	871	WS0101_F15
1	95.572	17	3	0.281	0.045	4.669	871	WS0101_F15
5	31.191	95	1	0.251	0.060	5.600	871	WS0101_F15
5	52.820	99	3	-0.304	0.076	9.123	871	WS0101_F15
8	50.001	154	2	-0.195	0.052	4.716	871	WS0101_F15
10	78.479	193	3	-0.326	0.065	7.713	871	WS0101_F15
10	78.479	193	4	-0.279	0.097	6.730	871	WS0101_F15
11	43.491	214	3	0.271	0.046	5.568	871	WS0101_F15
11	44.884	215	3	-0.247	0.034	4.099	871	WS0101_F15
1	95.572	17	3	0.285	0.057	5.526	872	WS0101_K24
3	17.158	45	1	-0.268	0.067	7.212	872	WS0101_K24
3	47.928	49	1	0.239	0.045	4.894	872	WS0101_K24
3	148.745	68	4	-0.181	0.044	3.851	872	WS0101_K24
4	116.928	82	4	0.283	0.066	5.993	872	WS0101_K24
5	54.072	100	4	0.144	0.045	3.904	872	WS0101_K24
7	15.555	130	2	0.250	0.051	4.222	872	WS0101_K24
9	28.608	170	3	0.211	0.044	4.482	872	WS0101_K24
9	55.358	175	3	0.527	0.119	7.261	872	WS0101_K24
10	77.676	192	1	-0.270	0.046	5.073	872	WS0101_K24
10	80.088	194	1	-0.351	0.073	7.940	872	WS0101_K24
13	0.000	243	4	0.297	0.104	4.951	872	WS0101_K24
14	44.898	251	1	0.283	0.040	4.481	872	WS0101_K24
3	15.147	44	2	0.355	0.103	6.279	873	WS0079_K09
3	144.593	66	4	0.226	0.056	5.544	873	WS0079_K09
4	116.928	82	2	0.218	0.041	4.237	873	WS0079_K09
5	16.219	93	1	-0.232	0.046	3.928	873	WS0079_K09
6	87.891	124	2	-0.208	0.038	3.918	873	WS0079_K09
9	36.227	165	3	-0.228	0.051	4.847	873	WS0079_K09
9	59.963	177	4	0.255	0.049	4.339	873	WS0079_K09
11	27.405	204	1	0.201	0.046	3.963	873	WS0079_K09
14	46.637	252	4	-0.295	0.070	6.534	873	WS0079_K09
3	9.953	42	3	0.201	0.051	3.864	874	WS00938_K10
3	15.147	44	2	0.194	0.046	4.320	874	WS00938_K10
5	54.072	100	4	0.247	0.053	5.887	874	WS00938_K10
6	95.249	126	1	0.313	0.081	5.102	874	WS00938_K10
9	59.963	177	4	0.251	0.056	6.339	874	WS00938_K10
11	27.405	204	1	0.397	0.119	11.207	874	WS00938_K10
11	27.405	204	4	-0.290	0.047	5.238	874	WS00938_K10
11	29.069	205	1	0.228	0.056	5.029	874	WS00938_K10
12	14.406	230	4	0.239	0.044	4.806	874	WS00938_K10
2	63.261	30	3	0.285	0.061	6.106	875	WS0092_B04
2	75.550	31	1	0.311	0.044	5.165	875	WS0092_B04
3	15.147	44	2	0.282	0.090	4.618	875	WS0092_B04
4	55.925	76	1	-0.210	0.045	3.997	875	WS0092_B04
4	82.462	78	1	-0.218	0.034	3.957	875	WS0092_B04
5	16.219	93	1	-0.324	0.055	6.441	875	WS0092_B04
5	31.191	95	1	-0.223	0.059	6.829	875	WS0092_B04
7	74.560	143	1	0.270	0.049	5.644	875	WS0092_B04
1	43.578	8	2	-0.166	0.040	4.566	876	WS00939_E13
1	95.572	17	2	0.287	0.044	5.261	876	WS00939_E13
3	80.211	55	4	0.197	0.060	6.501	876	WS00939_E13
5	54.072	100	4	0.253	0.040	4.465	876	WS00939_E13
9	28.608	170	2	-0.256	0.035	4.161	876	WS00939_E13
10	9.580	187	2	-0.297	0.108	9.913	876	WS00939_E13

11	27.405	204	1	0.460	0.154	11.769	876	WS00939_E13
11	27.405	204	4	-0.397	0.082	9.366	876	WS00939_E13
11	29.069	205	1	0.226	0.056	4.065	876	WS00939_E13
11	45.989	216	1	-0.222	0.096	6.941	876	WS00939_E13
12	14.406	230	4	0.239	0.046	5.219	876	WS00939_E13
4	92.039	79	2	0.282	0.041	4.311	877	WS0093_I12
7	62.787	139	4	-0.249	0.033	4.397	877	WS0093_I12
10	70.246	190	4	-0.261	0.056	4.539	877	WS0093_I12
10	78.479	193	4	-0.184	0.046	4.691	877	WS0093_I12
3	14.500	43	2	-0.263	0.037	4.280	878	WS0093_J02
7	62.787	139	4	-0.225	0.030	4.277	878	WS0093_J02
10	70.246	190	4	-0.337	0.092	7.901	878	WS0093_J02
4	55.925	76	1	-0.213	0.050	4.556	879	WS0093_L04
5	7.672	92	1	-0.251	0.036	4.336	879	WS0093_L04
9	36.227	165	3	-0.232	0.058	5.290	879	WS0093_L04
9	59.963	177	4	0.289	0.058	5.568	879	WS0093_L04
10	70.246	190	2	-0.206	0.047	4.151	879	WS0093_L04
11	27.405	204	1	0.195	0.039	3.876	879	WS0093_L04
11	27.405	204	4	-0.222	0.042	4.027	879	WS0093_L04
11	45.989	216	1	-0.254	0.052	5.185	879	WS0093_L04
12	6.242	234	2	-0.269	0.065	6.423	879	WS0093_L04
2	63.261	30	3	0.251	0.060	6.052	880	WS0093_M07
2	127.147	40	4	-0.237	0.066	5.283	880	WS0093_M07
3	9.953	42	3	0.237	0.049	4.898	880	WS0093_M07
4	55.925	76	1	-0.236	0.055	5.182	880	WS0093_M07
4	173.929	88	2	-0.206	0.044	3.915	880	WS0093_M07
11	27.405	204	1	0.213	0.042	4.147	880	WS0093_M07
11	45.989	216	1	-0.230	0.045	4.483	880	WS0093_M07
6	81.996	123	3	0.336	0.078	5.116	881	WS0093_N21
9	59.963	177	4	0.253	0.044	4.031	881	WS0093_N21
1	22.419	4	3	-0.329	0.064	4.588	882	WS0093_O15
1	69.854	13	2	-0.177	0.049	3.855	882	WS0093_O15
1	71.592	14	2	-0.188	0.056	4.406	882	WS0093_O15
1	133.992	24	2	-0.207	0.053	4.167	882	WS0093_O15
2	102.814	36	1	0.283	0.041	4.663	882	WS0093_O15
3	144.593	66	4	0.180	0.048	4.686	882	WS0093_O15
4	55.925	76	1	-0.244	0.045	4.929	882	WS0093_O15
5	16.219	93	2	-0.245	0.074	5.930	882	WS0093_O15
6	106.702	128	2	-0.446	0.156	3.895	882	WS0093_O15
10	95.795	197	4	0.250	0.049	4.591	882	WS0093_O15
11	0.000	199	3	-0.317	0.093	8.589	882	WS0093_O15
11	27.405	204	1	0.221	0.039	4.238	882	WS0093_O15
14	9.923	247	1	-0.235	0.040	4.442	882	WS0093_O15
1	62.128	11	2	-0.281	0.051	4.987	883	WS0101_B12
2	101.076	35	3	0.187	0.030	4.125	883	WS0101_B12
2	127.147	40	3	-0.201	0.033	4.410	883	WS0101_B12
4	92.039	79	2	-0.228	0.041	5.957	883	WS0101_B12
4	116.928	82	2	0.247	0.034	4.911	883	WS0101_B12
5	57.017	102	2	-0.231	0.033	4.874	883	WS0101_B12
6	49.100	118	2	0.216	0.040	4.352	883	WS0101_B12
10	80.088	194	2	0.209	0.031	4.539	883	WS0101_B12
11	56.921	219	2	-0.265	0.047	5.312	883	WS0101_B12
13	1.815	244	2	-0.258	0.041	5.879	883	WS0101_B12
1	69.854	13	2	-0.170	0.050	5.124	884	WS0101_C07

1	71.592	14	2	-0.171	0.051	5.212	884	WS0101_C07
1	95.572	17	2	-0.243	0.058	6.025	884	WS0101_C07
2	63.261	30	3	-0.281	0.072	6.626	884	WS0101_C07
2	95.880	34	3	-0.304	0.103	9.713	884	WS0101_C07
2	101.076	35	3	0.235	0.056	5.170	884	WS0101_C07
2	101.076	35	4	-0.303	0.063	5.730	884	WS0101_C07
3	148.745	68	4	0.224	0.053	5.041	884	WS0101_C07
4	82.462	78	2	-0.230	0.060	5.545	884	WS0101_C07
5	7.672	92	1	0.308	0.077	5.483	884	WS0101_C07
5	7.672	92	4	-0.257	0.045	4.382	884	WS0101_C07
6	25.914	114	1	0.202	0.064	5.377	884	WS0101_C07
7	15.555	130	2	-0.260	0.045	4.700	884	WS0101_C07
7	15.555	130	3	0.265	0.060	5.598	884	WS0101_C07
9	0.000	163	1	-0.501	0.101	5.538	884	WS0101_C07
9	36.227	165	3	0.265	0.051	4.663	884	WS0101_C07
10	9.580	187	2	-0.285	0.081	5.409	884	WS0101_C07
11	41.984	212	2	0.155	0.039	4.035	884	WS0101_C07
11	45.989	216	2	0.195	0.055	5.945	884	WS0101_C07
11	60.464	221	2	0.297	0.062	6.659	884	WS0101_C07
12	32.473	238	1	-0.292	0.062	5.344	884	WS0101_C07
2	127.147	40	4	-0.234	0.082	5.534	885	WS01011_N13
3	137.891	64	3	0.221	0.047	5.678	885	WS01011_N13
3	141.443	65	3	0.261	0.069	5.950	885	WS01011_N13
4	82.462	78	1	-0.185	0.038	4.066	885	WS01011_N13
4	173.929	88	2	-0.215	0.047	3.854	885	WS01011_N13
9	59.963	177	4	0.253	0.063	4.046	885	WS01011_N13
1	107.333	19	4	-0.123	0.034	4.382	886	WS01011_O04
2	48.839	29	1	-0.218	0.049	6.056	886	WS01011_O04
2	95.880	34	1	0.350	0.039	4.785	886	WS01011_O04
3	141.443	65	4	0.513	0.099	6.937	886	WS01011_O04
8	0.000	148	4	-0.278	0.045	4.949	886	WS01011_O04
9	0.000	166	1	0.113	0.032	3.847	886	WS01011_O04
13	3.386	245	1	-0.292	0.032	4.089	886	WS01011_O04
14	22.402	248	4	-0.348	0.038	3.971	886	WS01011_O04
14	46.637	252	4	-0.333	0.043	5.047	886	WS01011_O04
3	137.891	64	3	0.286	0.077	7.973	887	WS01012_A08
3	141.443	65	3	0.336	0.090	7.517	887	WS01012_A08
4	0.000	71	4	0.262	0.059	4.927	887	WS01012_A08
4	173.929	88	2	-0.209	0.057	4.841	887	WS01012_A08
8	87.463	158	2	-0.232	0.060	4.506	887	WS01012_A08
9	59.963	177	4	0.226	0.057	4.681	887	WS01012_A08
11	27.405	204	1	0.214	0.049	5.112	887	WS01012_A08
1	2.788	2	4	-0.222	0.054	5.028	888	WS01012_B09
1	69.854	13	2	0.176	0.058	4.560	888	WS01012_B09
1	71.592	14	2	0.174	0.057	4.476	888	WS01012_B09
3	137.891	64	3	0.190	0.040	3.851	888	WS01012_B09
9	17.951	169	1	0.274	0.036	3.983	888	WS01012_B09
4	82.462	78	2	-0.152	0.036	4.272	889	WS01012_D07
4	97.576	80	2	0.195	0.034	4.394	889	WS01012_D07
7	62.787	139	4	-0.280	0.054	7.158	889	WS01012_D07
7	74.560	143	2	-0.254	0.034	3.952	889	WS01012_D07
11	40.960	210	2	-0.473	0.098	7.317	889	WS01012_D07
11	40.960	210	4	0.237	0.046	4.613	889	WS01012_D07
11	57.661	220	4	-0.210	0.041	4.388	889	WS01012_D07

11	64.014	222	4	0.322	0.051	5.376	889	WS01012_D07
1	62.128	11	1	-0.448	0.083	3.955	890	WS01012_E05
3	137.891	64	3	0.363	0.083	6.701	890	WS01012_E05
4	82.462	78	1	-0.231	0.055	5.994	890	WS01012_E05
10	80.088	194	1	-0.272	0.035	3.885	890	WS01012_E05
11	9.237	202	4	0.252	0.044	4.036	890	WS01012_E05
12	38.543	232	4	-0.314	0.104	4.894	890	WS01012_E05
12	6.242	234	2	-0.182	0.034	4.371	890	WS01012_E05
13	0.000	243	4	0.369	0.112	5.276	890	WS01012_E05
6	69.073	121	4	-0.300	0.070	5.673	891	WS01012_F20
6	76.376	122	1	0.294	0.159	6.777	891	WS01012_F20
7	15.555	130	2	0.221	0.055	4.764	891	WS01012_F20
7	15.555	130	3	-0.337	0.060	6.325	891	WS01012_F20
7	73.169	142	1	-0.508	0.097	4.155	891	WS01012_F20
9	17.951	169	2	0.246	0.053	4.519	891	WS01012_F20
9	49.013	173	2	0.192	0.056	4.115	891	WS01012_F20
11	17.088	203	1	0.219	0.055	5.812	891	WS01012_F20
11	56.921	219	2	-0.301	0.087	4.144	891	WS01012_F20
14	0.000	246	1	-0.178	0.037	3.971	891	WS01012_F20
1	62.128	11	1	-0.638	0.135	12.420	892	WS01012_G09
1	107.333	19	4	-0.211	0.033	4.305	892	WS01012_G09
3	137.891	64	1	0.250	0.048	5.781	892	WS01012_G09
3	141.443	65	4	0.310	0.077	6.803	892	WS01012_G09
4	82.462	78	1	-0.278	0.045	5.454	892	WS01012_G09
4	92.039	79	4	-0.295	0.035	5.057	892	WS01012_G09
4	173.929	88	2	-0.262	0.077	5.232	892	WS01012_G09
5	54.072	100	4	0.195	0.031	4.125	892	WS01012_G09
5	57.017	102	4	-0.313	0.051	6.873	892	WS01012_G09
11	9.237	202	4	0.228	0.043	5.751	892	WS01012_G09
11	27.405	204	4	-0.246	0.046	5.967	892	WS01012_G09
4	173.929	88	2	-0.283	0.064	7.063	893	WS02612_E09
6	81.996	123	3	0.308	0.103	4.357	893	WS02612_E09
8	87.463	158	2	-0.207	0.056	6.330	893	WS02612_E09
11	9.237	202	4	0.294	0.066	7.515	893	WS02612_E09
11	27.405	204	4	-0.237	0.053	5.846	893	WS02612_E09
11	45.989	216	1	-0.259	0.059	4.653	893	WS02612_E09
2	127.147	40	3	-0.194	0.059	4.745	894	WS02613_E22
5	31.191	95	1	-0.221	0.054	4.376	894	WS02613_E22
11	27.405	204	1	0.371	0.108	8.915	894	WS02613_E22
11	27.405	204	4	-0.327	0.088	9.405	894	WS02613_E22
11	29.069	205	1	0.238	0.053	4.335	894	WS02613_E22
11	45.989	216	1	-0.205	0.051	4.103	894	WS02613_E22
12	14.406	230	4	0.312	0.054	5.658	894	WS02613_E22
14	43.392	250	2	-0.194	0.040	4.060	894	WS02613_E22
2	48.839	29	4	0.300	0.048	4.974	895	WS0091_B02
4	173.929	88	2	-0.155	0.035	4.219	895	WS0091_B02
9	49.013	173	2	0.211	0.051	6.178	895	WS0091_B02
10	95.795	197	4	0.234	0.060	6.247	895	WS0091_B02
14	0.000	246	2	-0.256	0.051	6.414	895	WS0091_B02
1	29.800	5	1	0.193	0.046	4.308	896	WS00928_A24
1	38.326	6	4	-0.307	0.065	5.327	896	WS00928_A24
1	40.464	7	4	0.264	0.041	4.317	896	WS00928_A24
1	45.794	9	1	0.388	0.098	9.256	896	WS00928_A24
1	50.955	10	1	-0.189	0.055	5.137	896	WS00928_A24

3	20.512	46	2	0.196	0.051	4.824	896	WS00928_A24
7	62.787	139	4	-0.258	0.056	4.082	896	WS00928_A24
10	86.151	195	3	0.177	0.040	3.941	896	WS00928_A24
13	3.386	245	1	0.280	0.046	4.440	896	WS00928_A24
2	93.504	32	4	-0.239	0.046	4.669	897	WS0091_G10
2	101.076	35	4	0.231	0.041	4.976	897	WS0091_G10
2	127.147	40	4	-0.290	0.076	6.997	897	WS0091_G10
3	144.647	67	1	0.374	0.101	5.181	897	WS0091_G10
4	124.132	83	1	0.444	0.085	4.366	897	WS0091_G10
11	27.405	204	1	0.257	0.050	4.888	897	WS0091_G10
11	45.989	216	1	-0.225	0.043	4.107	897	WS0091_G10
14	0.000	246	1	0.206	0.044	4.181	897	WS0091_G10
1	95.572	17	3	0.285	0.071	5.313	898	WS00928_E20
2	126.181	39	4	0.457	0.097	6.128	898	WS00928_E20
11	27.405	204	1	0.237	0.054	3.935	898	WS00928_E20
14	44.898	251	1	0.216	0.056	4.249	898	WS00928_E20
1	22.419	4	3	0.371	0.084	4.867	899	WS00927_O01
3	80.211	55	4	0.200	0.048	4.677	899	WS00927_O01
5	54.072	100	4	0.325	0.094	9.162	899	WS00927_O01
5	57.017	102	4	-0.301	0.071	6.856	899	WS00927_O01
11	27.405	204	1	0.354	0.072	5.743	899	WS00927_O01
11	27.405	204	4	-0.301	0.069	7.022	899	WS00927_O01
12	6.242	234	2	-0.284	0.048	6.738	899	WS00927_O01
13	3.386	245	1	-0.190	0.055	4.437	899	WS00927_O01
14	22.402	248	3	0.219	0.050	4.505	899	WS00927_O01
1	29.800	5	1	0.336	0.057	6.958	900	WS00927_O11
1	131.686	23	1	0.642	0.134	7.120	900	WS00927_O11
2	102.814	36	1	0.211	0.031	4.144	900	WS00927_O11
2	120.617	38	3	0.280	0.052	5.476	900	WS00927_O11
2	127.147	40	3	-0.196	0.042	4.338	900	WS00927_O11
3	20.512	46	2	0.244	0.078	5.209	900	WS00927_O11
4	75.958	77	3	0.297	0.138	4.965	900	WS00927_O11
4	142.917	85	1	0.239	0.037	4.468	900	WS00927_O11
8	62.580	155	1	0.416	0.101	5.380	900	WS00927_O11
10	9.580	187	3	0.350	0.111	7.410	900	WS00927_O11
10	35.204	189	1	0.228	0.035	4.174	900	WS00927_O11
11	0.000	199	3	-0.282	0.051	4.173	900	WS00927_O11
11	27.405	204	1	0.297	0.054	6.553	900	WS00927_O11
11	40.960	210	2	0.386	0.118	3.996	900	WS00927_O11
12	6.242	234	2	-0.251	0.074	5.635	900	WS00927_O11
3	9.953	42	4	0.184	0.052	4.411	901	WS00927_P22
6	81.996	123	3	0.277	0.072	4.444	901	WS00927_P22
10	78.479	193	3	0.292	0.044	4.176	901	WS00927_P22
11	27.405	204	1	0.337	0.091	9.190	901	WS00927_P22
12	6.242	234	2	-0.234	0.050	5.544	901	WS00927_P22
1	69.854	13	2	-0.273	0.095	12.555	902	JF16
1	71.592	14	2	-0.249	0.082	10.857	902	JF16
1	77.177	15	2	-0.230	0.071	9.431	902	JF16
1	95.572	17	2	-0.425	0.084	11.321	902	JF16
6	56.188	120	2	0.358	0.053	6.053	902	JF16
9	0.000	166	1	-0.168	0.072	4.159	902	JF16
10	78.479	193	4	-0.245	0.088	5.033	902	JF16
12	22.441	227	1	0.300	0.103	6.017	902	JF16
12	22.441	227	3	-0.293	0.036	5.150	902	JF16

12	14.406	230	3	-0.304	0.030	4.320	902	JF16
1	43.578	8	2	0.292	0.050	5.842	903	SS1
1	69.854	13	2	-0.192	0.048	5.685	903	SS1
1	71.592	14	2	-0.191	0.048	5.674	903	SS1
1	77.177	15	2	-0.174	0.040	4.761	903	SS1
7	74.560	143	2	0.214	0.040	4.993	903	SS1
7	75.883	144	2	0.399	0.073	4.025	903	SS1
11	27.405	204	1	0.296	0.058	4.033	903	SS1
11	56.921	219	2	-0.312	0.068	4.411	903	SS1
1	29.800	5	1	-0.282	0.075	7.382	904	WS0092_F14
2	127.147	40	4	-0.237	0.064	3.924	904	WS0092_F14
3	137.891	64	3	0.190	0.057	7.299	904	WS0092_F14
11	9.237	202	4	0.271	0.092	6.777	904	WS0092_F14
11	27.405	204	4	-0.211	0.077	6.053	904	WS0092_F14
14	44.898	251	3	0.371	0.066	8.435	904	WS0092_F14
1	2.788	2	4	-0.267	0.050	5.961	905	WS0092_K11
2	95.880	34	1	-0.218	0.033	3.994	905	WS0092_K11
3	80.211	55	4	0.211	0.031	4.144	905	WS0092_K11
3	137.891	64	3	0.238	0.091	5.101	905	WS0092_K11
4	82.462	78	1	-0.221	0.039	4.895	905	WS0092_K11
5	42.979	97	1	-0.247	0.031	3.926	905	WS0092_K11
6	45.897	117	1	-0.216	0.034	4.204	905	WS0092_K11
9	0.000	166	1	-0.241	0.042	5.212	905	WS0092_K11
9	17.951	169	1	0.341	0.067	8.366	905	WS0092_K11
11	9.237	202	4	0.263	0.046	6.099	905	WS0092_K11
11	27.405	204	4	-0.256	0.043	4.385	905	WS0092_K11
14	0.000	246	1	-0.246	0.048	5.955	905	WS0092_K11
1	69.854	13	2	0.173	0.041	4.013	906	WS01013_E10
1	71.592	14	2	0.191	0.050	4.909	906	WS01013_E10
1	77.177	15	2	0.202	0.054	5.334	906	WS01013_E10
1	95.572	17	2	0.190	0.040	3.892	906	WS01013_E10
3	137.891	64	3	0.383	0.097	9.138	906	WS01013_E10
6	106.702	128	2	0.416	0.100	4.202	906	WS01013_E10
8	50.001	154	1	-0.275	0.066	5.942	906	WS01013_E10
8	78.389	157	1	-0.226	0.058	5.200	906	WS01013_E10
8	87.463	158	1	0.223	0.058	5.142	906	WS01013_E10
9	59.963	177	4	0.329	0.075	7.253	906	WS01013_E10
11	27.405	204	1	0.188	0.044	3.948	906	WS01013_E10
11	45.989	216	1	-0.228	0.051	4.597	906	WS01013_E10
14	44.898	251	1	0.288	0.057	5.112	906	WS01013_E10
1	62.128	11	1	-0.581	0.115	8.947	907	WS01038_O14
3	141.443	65	4	0.243	0.058	4.543	907	WS01038_O14
3	148.745	68	4	-0.180	0.036	4.545	907	WS01038_O14
4	82.462	78	1	-0.243	0.037	4.240	907	WS01038_O14
4	92.039	79	4	-0.368	0.067	6.586	907	WS01038_O14
9	28.608	170	3	0.238	0.065	4.535	907	WS01038_O14
10	78.479	193	4	-0.296	0.047	4.982	907	WS01038_O14
10	80.088	194	1	-0.281	0.049	5.554	907	WS01038_O14
11	9.237	202	4	0.247	0.047	5.794	907	WS01038_O14
11	27.405	204	4	-0.206	0.044	5.062	907	WS01038_O14
12	38.543	232	4	0.326	0.100	7.808	907	WS01038_O14
2	63.261	30	3	-0.325	0.127	16.847	908	WS01013_J17
2	75.550	31	2	-0.212	0.070	8.618	908	WS01013_J17
2	95.880	34	3	-0.637	0.167	22.800	908	WS01013_J17

2	101.076	35	3	0.597	0.149	20.130	908	WS01013_J17
3	9.953	42	3	0.406	0.110	14.702	908	WS01013_J17
3	137.891	64	3	0.166	0.034	4.848	908	WS01013_J17
4	82.462	78	2	-0.255	0.038	4.906	908	WS01013_J17
5	86.669	106	2	-0.401	0.165	13.357	908	WS01013_J17
6	87.891	124	2	-0.211	0.038	4.260	908	WS01013_J17
6	89.401	125	2	-0.261	0.039	3.912	908	WS01013_J17
6	106.702	128	2	0.518	0.093	7.725	908	WS01013_J17
7	54.200	136	2	-0.181	0.056	4.422	908	WS01013_J17
7	62.787	139	2	-0.314	0.057	6.527	908	WS01013_J17
9	49.013	173	2	-0.218	0.044	4.837	908	WS01013_J17
9	69.046	181	2	-0.368	0.062	5.296	908	WS01013_J17
12	22.441	227	1	-0.275	0.108	8.800	908	WS01013_J17
12	6.242	234	2	-0.223	0.053	5.797	908	WS01013_J17
4	142.917	85	1	0.302	0.044	4.677	909	WS0269_K14
4	173.929	88	2	-0.196	0.053	4.199	909	WS0269_K14
5	54.072	100	4	0.198	0.047	5.193	909	WS0269_K14
5	57.017	102	4	-0.255	0.051	5.683	909	WS0269_K14
6	81.996	123	3	0.309	0.120	5.093	909	WS0269_K14
13	0.000	243	4	0.297	0.076	4.917	909	WS0269_K14
14	22.402	248	4	0.281	0.050	5.642	909	WS0269_K14
1	15.589	3	2	-0.191	0.040	4.704	910	WS00712_C01
4	173.929	88	2	-0.202	0.044	5.115	910	WS00712_C01
6	106.702	128	2	-0.504	0.085	6.476	910	WS00712_C01
9	55.358	175	1	0.343	0.100	7.297	910	WS00712_C01
9	61.113	178	1	-0.350	0.135	5.277	910	WS00712_C01
12	22.441	227	3	0.220	0.048	4.757	910	WS00712_C01
13	0.000	243	4	0.373	0.113	4.449	910	WS00712_C01
14	44.898	251	3	0.290	0.083	7.234	910	WS00712_C01
3	14.500	43	2	-0.149	0.037	4.176	911	WS00712_I22
3	15.147	44	2	0.195	0.081	4.755	911	WS00712_I22
3	20.512	46	2	0.255	0.038	4.376	911	WS00712_I22
3	137.891	64	3	0.188	0.045	4.465	911	WS00712_I22
4	75.958	77	3	-0.340	0.076	4.006	911	WS00712_I22
4	82.462	78	1	-0.283	0.047	3.977	911	WS00712_I22
5	31.191	95	1	-0.301	0.094	7.396	911	WS00712_I22
5	52.820	99	1	0.267	0.053	4.300	911	WS00712_I22
5	56.008	101	1	-0.159	0.060	4.739	911	WS00712_I22
9	2.011	167	4	-0.237	0.053	4.435	911	WS00712_I22
9	69.046	181	2	0.217	0.093	4.111	911	WS00712_I22
11	43.491	214	3	-0.281	0.048	5.151	911	WS00712_I22
14	44.898	251	3	0.325	0.069	4.970	911	WS00712_I22
1	62.128	11	1	-0.450	0.110	3.855	912	WS0094_M24
1	107.333	19	3	0.174	0.039	4.845	912	WS0094_M24
1	127.805	21	3	0.231	0.037	4.766	912	WS0094_M24
7	15.555	130	2	0.292	0.039	4.289	912	WS0094_M24
7	17.610	131	3	0.299	0.087	7.052	912	WS0094_M24
7	54.200	136	2	0.264	0.072	5.840	912	WS0094_M24
9	36.227	165	3	0.209	0.038	4.769	912	WS0094_M24
9	49.013	173	1	-0.269	0.078	6.142	912	WS0094_M24
9	59.963	177	1	-0.274	0.079	6.374	912	WS0094_M24
13	0.000	243	4	0.373	0.109	3.880	912	WS0094_M24
14	44.898	251	3	0.169	0.035	4.492	912	WS0094_M24
3	137.891	64	3	0.276	0.057	4.448	913	WS0057_D24

4	0.000	71	4	0.294	0.086	8.521	913	WS0057_D24
4	173.929	88	2	-0.231	0.056	6.394	913	WS0057_D24
10	70.246	190	2	-0.271	0.040	4.658	913	WS0057_D24
11	27.405	204	4	-0.289	0.060	4.263	913	WS0057_D24
1	45.794	9	3	-0.187	0.034	3.881	914	WS01017_A14
9	36.227	165	3	0.200	0.040	4.553	914	WS01017_A14
10	35.204	189	1	0.186	0.045	3.883	914	WS01017_A14
11	27.405	204	1	0.263	0.047	4.109	914	WS01017_A14
11	45.989	216	1	-0.196	0.049	4.303	914	WS01017_A14
11	46.026	217	1	-0.208	0.049	4.295	914	WS01017_A14
4	0.000	71	4	0.339	0.135	11.998	915	WS0057_N15
10	70.246	190	2	-0.360	0.082	9.234	915	WS0057_N15
1	15.589	3	2	-0.328	0.030	3.922	916	WS00712_M24
1	29.800	5	1	-0.266	0.123	10.737	916	WS00712_M24
3	9.953	42	4	0.234	0.009	4.245	916	WS00712_M24
5	7.672	92	4	0.296	0.031	4.185	916	WS00712_M24
5	57.017	102	2	0.308	0.041	4.748	916	WS00712_M24
8	87.463	158	2	-0.046	0.024	4.397	916	WS00712_M24
9	59.963	177	4	0.336	0.048	5.463	916	WS00712_M24
10	93.347	196	1	-0.498	0.116	5.958	916	WS00712_M24
14	43.392	250	1	-0.345	0.062	4.213	916	WS00712_M24
1	22.419	4	4	-0.523	0.132	7.503	917	WS00712_O06
1	69.854	13	2	0.173	0.060	4.728	917	WS00712_O06
3	14.500	43	1	-0.162	0.040	4.364	917	WS00712_O06
3	17.158	45	1	0.244	0.063	6.832	917	WS00712_O06
3	47.928	49	1	0.170	0.036	3.848	917	WS00712_O06
3	95.157	59	2	-0.206	0.079	6.263	917	WS00712_O06
8	11.974	150	1	-0.215	0.050	5.369	917	WS00712_O06
9	55.358	175	1	0.328	0.036	4.056	917	WS00712_O06
11	17.088	203	1	-0.186	0.042	4.512	917	WS00712_O06
11	29.069	205	1	0.280	0.039	4.428	917	WS00712_O06
12	6.242	234	4	-0.396	0.084	7.517	917	WS00712_O06
12	22.629	237	1	0.264	0.047	5.133	917	WS00712_O06
12	32.473	238	1	0.244	0.038	4.218	917	WS00712_O06
13	3.386	245	1	0.296	0.033	3.859	917	WS00712_O06
14	44.898	251	3	0.264	0.046	5.381	917	WS00712_O06
1	131.686	23	1	-0.477	0.081	3.993	918	WS00712_P20
2	120.617	38	3	-0.225	0.047	4.117	918	WS00712_P20
3	47.928	49	1	-0.261	0.046	4.266	918	WS00712_P20
4	0.000	71	4	0.117	0.041	4.163	918	WS00712_P20
5	52.820	99	3	-0.235	0.048	4.311	918	WS00712_P20
5	86.669	106	2	-0.257	0.092	4.243	918	WS00712_P20
5	86.669	106	4	-0.433	0.078	4.041	918	WS00712_P20
9	3.127	168	1	-0.439	0.078	4.373	918	WS00712_P20
11	45.989	216	1	-0.314	0.069	6.374	918	WS00712_P20
14	46.637	252	4	0.346	0.040	3.871	918	WS00712_P20
1	62.128	11	2	0.231	0.062	3.918	919	WS00713_A20
1	107.333	19	3	0.176	0.038	4.392	919	WS00713_A20
3	17.158	45	1	0.249	0.069	6.415	919	WS00713_A20
3	95.157	59	2	-0.191	0.055	4.908	919	WS00713_A20
3	137.891	64	3	-0.204	0.043	4.739	919	WS00713_A20
3	141.443	65	3	-0.289	0.098	5.081	919	WS00713_A20
4	55.925	76	1	-0.226	0.056	4.861	919	WS00713_A20
4	82.462	78	1	-0.279	0.063	5.973	919	WS00713_A20

7	17.610	131	3	0.253	0.068	4.367	919	WS00713_A20
7	54.200	136	2	0.349	0.121	7.735	919	WS00713_A20
7	62.787	139	2	0.312	0.067	6.050	919	WS00713_A20
8	95.937	159	3	0.214	0.050	5.813	919	WS00713_A20
9	3.127	168	1	-0.354	0.086	3.846	919	WS00713_A20
10	9.580	187	3	-0.291	0.089	5.146	919	WS00713_A20
12	0.000	226	3	0.188	0.040	4.693	919	WS00713_A20
3	17.158	45	1	0.237	0.049	4.232	920	WS00713_E09
4	43.146	74	3	0.212	0.045	5.366	920	WS00713_E09
4	75.958	77	3	-0.248	0.058	4.023	920	WS00713_E09
7	15.555	130	3	-0.260	0.055	6.480	920	WS00713_E09
7	17.610	131	3	-0.280	0.056	3.848	920	WS00713_E09
11	42.600	213	3	-0.212	0.039	4.692	920	WS00713_E09
11	64.014	222	4	-0.241	0.074	4.966	920	WS00713_E09
12	6.242	234	2	-0.224	0.031	4.177	920	WS00713_E09
1	2.788	2	3	-0.237	0.049	4.918	921	WS00713_F11
1	133.992	24	2	0.257	0.063	7.226	921	WS00713_F11
1	141.021	25	2	-0.202	0.041	4.734	921	WS00713_F11
4	116.928	82	2	-0.252	0.037	4.379	921	WS00713_F11
6	106.702	128	2	-0.508	0.104	6.614	921	WS00713_F11
7	75.883	144	2	0.474	0.098	5.514	921	WS00713_F11
8	50.001	154	1	0.206	0.057	4.196	921	WS00713_F11
8	95.937	159	3	0.217	0.044	4.400	921	WS00713_F11
12	6.242	234	2	-0.193	0.034	4.489	921	WS00713_F11
3	14.500	43	1	-0.244	0.078	4.885	922	WS00713_I21
3	17.158	45	1	0.296	0.113	7.090	922	WS00713_I21
4	103.719	81	4	-0.203	0.035	4.122	922	WS00713_I21
6	25.914	114	1	-0.230	0.068	4.245	922	WS00713_I21
6	89.401	125	1	-0.299	0.078	4.901	922	WS00713_I21
7	15.555	130	2	0.259	0.040	5.048	922	WS00713_I21
9	49.013	173	2	0.263	0.053	6.631	922	WS00713_I21
11	27.405	204	4	0.315	0.048	4.612	922	WS00713_I21
1	29.800	5	1	-0.281	0.051	4.005	923	WS00713_J18
1	45.794	9	3	-0.280	0.074	6.575	923	WS00713_J18
3	14.500	43	1	-0.327	0.089	6.851	923	WS00713_J18
3	17.158	45	1	0.213	0.069	5.366	923	WS00713_J18
7	15.555	130	2	0.460	0.111	12.390	923	WS00713_J18
7	17.610	131	3	0.430	0.204	11.201	923	WS00713_J18
7	91.487	146	3	0.183	0.039	4.021	923	WS00713_J18
8	11.974	150	1	-0.180	0.050	3.854	923	WS00713_J18
10	104.214	198	2	0.216	0.048	6.348	923	WS00713_J18
13	1.815	244	2	0.271	0.063	6.806	923	WS00713_J18
14	44.898	251	3	0.251	0.065	6.321	923	WS00713_J18
14	46.637	252	3	0.244	0.041	4.247	923	WS00713_J18
3	80.211	55	4	0.265	0.039	3.958	924	WS00946_B17
3	154.505	69	4	0.175	0.046	4.719	924	WS00946_B17
4	75.958	77	3	0.361	0.116	5.370	924	WS00946_B17
4	97.576	80	2	0.222	0.055	6.654	924	WS00946_B17
4	116.928	82	2	0.317	0.056	6.661	924	WS00946_B17
4	173.929	88	2	0.193	0.045	5.162	924	WS00946_B17
6	54.066	119	4	-0.215	0.073	7.153	924	WS00946_B17
9	0.000	163	1	0.421	0.090	3.902	924	WS00946_B17
9	59.963	177	1	-0.259	0.053	3.995	924	WS00946_B17
11	42.600	213	3	0.166	0.057	5.122	924	WS00946_B17

11	57.661	220	4	-0.227	0.058	5.731	924	WS00946_B17
11	64.014	222	4	0.350	0.071	7.122	924	WS00946_B17
12	14.406	230	4	0.219	0.049	5.080	924	WS00946_B17
14	22.402	248	4	-0.295	0.040	4.138	924	WS00946_B17
3	14.500	43	1	-0.242	0.054	5.143	925	WS00817_L03
3	17.158	45	1	0.268	0.088	8.328	925	WS00817_L03
6	25.914	114	1	-0.217	0.062	5.877	925	WS00817_L03
10	9.580	187	3	-0.320	0.102	4.990	925	WS00817_L03
10	21.864	188	1	0.335	0.050	4.825	925	WS00817_L03
13	3.386	245	1	0.262	0.045	4.319	925	WS00817_L03
14	22.402	248	3	-0.268	0.052	4.788	925	WS00817_L03
1	131.686	23	2	0.441	0.088	3.890	926	WS00946_K15
2	95.880	34	3	0.235	0.061	4.708	926	WS00946_K15
3	14.500	43	1	-0.153	0.058	4.513	926	WS00946_K15
3	17.158	45	1	0.313	0.103	8.054	926	WS00946_K15
4	97.576	80	2	0.182	0.035	4.308	926	WS00946_K15
6	56.188	120	2	-0.212	0.037	4.402	926	WS00946_K15
6	95.249	126	1	0.370	0.111	4.162	926	WS00946_K15
7	17.610	131	3	0.346	0.092	5.290	926	WS00946_K15
7	105.477	147	2	-0.284	0.098	5.138	926	WS00946_K15
8	95.937	159	3	0.271	0.050	4.792	926	WS00946_K15
10	35.204	189	2	0.216	0.042	4.746	926	WS00946_K15
11	9.237	202	4	0.185	0.061	5.755	926	WS00946_K15
11	46.026	217	1	-0.238	0.058	4.499	926	WS00946_K15
11	60.464	221	2	0.227	0.048	6.258	926	WS00946_K15
12	6.242	234	4	-0.378	0.060	7.179	926	WS00946_K15
14	44.898	251	3	0.202	0.039	3.985	926	WS00946_K15
1	2.788	2	4	0.328	0.074	7.465	927	WS00818_B05
4	92.039	79	4	0.240	0.042	4.512	927	WS00818_B05
11	64.014	222	4	0.331	0.060	6.084	927	WS00818_B05
13	0.000	243	4	-0.371	0.100	4.257	927	WS00818_B05
1	29.800	5	1	0.307	0.088	6.557	928	WS00922_I08
1	40.464	7	4	-0.223	0.051	4.880	928	WS00922_I08
1	133.992	24	2	-0.184	0.035	4.004	928	WS00922_I08
4	173.929	88	2	0.203	0.035	3.972	928	WS00922_I08
7	17.610	131	3	0.286	0.099	4.478	928	WS00922_I08
9	17.951	169	2	0.189	0.037	4.379	928	WS00922_I08
9	28.608	170	2	-0.201	0.036	4.069	928	WS00922_I08
4	75.958	77	3	-0.306	0.078	4.021	929	WS00922_L22
5	57.017	102	2	0.242	0.035	3.878	929	WS00922_L22
14	44.898	251	3	0.257	0.042	4.755	929	WS00922_L22
5	31.191	95	1	-0.192	0.040	4.037	930	WS00814_G05
5	52.820	99	1	0.229	0.037	3.977	930	WS00814_G05
5	56.008	101	1	-0.236	0.057	5.786	930	WS00814_G05
6	25.914	114	1	-0.236	0.049	4.954	930	WS00814_G05
14	44.898	251	3	0.240	0.032	3.931	930	WS00814_G05
3	114.747	62	2	-0.181	0.043	3.937	931	WS00814_H10
4	75.958	77	3	-0.284	0.095	4.258	931	WS00814_H10
4	82.462	78	1	-0.281	0.046	4.529	931	WS00814_H10
12	22.441	227	1	-0.224	0.042	4.035	931	WS00814_H10
1	29.800	5	1	-0.282	0.049	4.188	932	WS00814_J16
1	62.128	11	2	0.250	0.076	4.781	932	WS00814_J16
1	69.854	13	2	0.186	0.046	4.763	932	WS00814_J16
1	71.592	14	2	0.180	0.044	4.491	932	WS00814_J16

2	93.504	32	4	-0.252	0.063	4.125	932	WS00814_J16
4	82.462	78	1	-0.251	0.067	5.731	932	WS00814_J16
7	54.200	136	2	0.237	0.067	4.200	932	WS00814_J16
9	49.013	173	1	-0.220	0.048	4.535	932	WS00814_J16
9	58.045	176	1	-0.288	0.078	4.462	932	WS00814_J16
9	59.963	177	1	-0.268	0.058	5.045	932	WS00814_J16
9	81.073	183	3	-0.208	0.046	5.598	932	WS00814_J16
14	22.402	248	3	-0.297	0.056	6.740	932	WS00814_J16
14	46.637	252	3	0.297	0.043	5.176	932	WS00814_J16
1	82.301	16	3	-0.258	0.038	4.726	933	WS00814_K12
2	93.504	32	4	-0.243	0.063	3.963	933	WS00814_K12
3	9.953	42	3	0.168	0.035	4.338	933	WS00814_K12
3	137.891	64	3	0.271	0.036	3.856	933	WS00814_K12
7	15.555	130	3	-0.309	0.071	7.141	933	WS00814_K12
10	21.864	188	1	0.310	0.066	5.452	933	WS00814_K12
10	80.088	194	1	-0.197	0.047	3.941	933	WS00814_K12
11	46.026	217	1	-0.206	0.048	4.055	933	WS00814_K12
1	45.794	9	1	-0.341	0.061	6.206	934	WS00814_N07
1	50.955	10	1	0.233	0.043	4.329	934	WS00814_N07
4	55.925	76	1	-0.232	0.042	4.738	934	WS00814_N07
4	124.132	83	1	0.450	0.090	4.308	934	WS00814_N07
7	17.610	131	3	0.246	0.084	4.826	934	WS00814_N07
7	54.200	136	2	0.293	0.111	6.392	934	WS00814_N07
9	36.227	165	3	0.242	0.052	5.752	934	WS00814_N07
12	38.543	232	4	0.329	0.091	4.373	934	WS00814_N07
13	0.000	243	4	0.309	0.085	4.091	934	WS00814_N07
14	46.637	252	3	0.261	0.037	4.104	934	WS00814_N07
1	69.854	13	2	0.206	0.041	4.734	935	WS00814_O23
1	71.592	14	2	0.196	0.037	4.281	935	WS00814_O23
4	0.000	71	4	0.275	0.082	6.749	935	WS00814_O23
4	82.462	78	1	-0.248	0.056	4.130	935	WS00814_O23
4	173.929	88	2	-0.236	0.060	6.853	935	WS00814_O23
10	78.479	193	3	0.311	0.044	4.714	935	WS00814_O23
10	86.151	195	3	-0.203	0.039	4.084	935	WS00814_O23
11	64.014	222	4	-0.276	0.051	4.264	935	WS00814_O23
3	154.505	69	1	0.222	0.046	4.075	936	WS00815_A14
4	0.000	71	4	0.259	0.073	6.696	936	WS00815_A14
8	87.463	158	2	-0.278	0.075	7.464	936	WS00815_A14
10	70.246	190	2	-0.274	0.061	6.258	936	WS00815_A14
11	43.491	214	3	-0.247	0.058	5.560	936	WS00815_A14
14	44.898	251	3	0.272	0.061	5.898	936	WS00815_A14
4	173.929	88	2	-0.214	0.050	4.498	937	WS00815_B04
11	43.491	214	3	-0.181	0.036	3.947	937	WS00815_B04
12	6.242	234	2	-0.260	0.038	4.205	937	WS00815_B04
1	62.128	11	1	0.448	0.090	3.844	938	WS00815_E22
3	17.158	45	1	0.207	0.042	4.046	938	WS00815_E22
3	137.891	64	3	0.291	0.064	5.372	938	WS00815_E22
6	106.702	128	2	0.484	0.131	6.826	938	WS00815_E22
6	106.702	128	3	-0.408	0.088	8.987	938	WS00815_E22
11	17.088	203	1	-0.320	0.098	9.399	938	WS00815_E22
11	29.069	205	1	0.315	0.046	4.684	938	WS00815_E22
11	40.960	210	2	-0.385	0.094	4.848	938	WS00815_E22
11	46.026	217	1	-0.305	0.073	7.026	938	WS00815_E22
11	64.014	222	4	0.294	0.107	10.367	938	WS00815_E22

12	38.543	232	4	-0.479	0.119	5.261	938	WS00815_E22
13	0.000	243	4	-0.214	0.112	4.740	938	WS00815_E22
14	22.402	248	4	-0.158	0.044	4.083	938	WS00815_E22
14	46.637	252	3	0.198	0.042	4.184	938	WS00815_E22
3	47.928	49	1	0.213	0.065	5.534	939	WS00815_K08
3	80.211	55	4	-0.210	0.067	7.146	939	WS00815_K08
3	148.745	68	1	-0.250	0.056	4.983	939	WS00815_K08
5	7.672	92	4	0.352	0.051	4.839	939	WS00815_K08
6	87.891	124	2	0.213	0.059	6.055	939	WS00815_K08
6	89.401	125	2	0.385	0.077	7.956	939	WS00815_K08
6	106.702	128	2	-0.438	0.107	4.990	939	WS00815_K08
8	50.001	154	1	0.220	0.067	4.934	939	WS00815_K08
9	55.358	175	1	-0.224	0.049	4.147	939	WS00815_K08
9	61.113	178	1	0.428	0.125	5.889	939	WS00815_K08
10	35.204	189	2	-0.159	0.043	3.898	939	WS00815_K08
1	141.021	25	2	0.252	0.043	5.594	940	WS00715_E11
7	54.200	136	2	-0.284	0.070	5.805	940	WS00715_E11
7	62.787	139	2	-0.262	0.046	6.128	940	WS00715_E11
8	50.001	154	2	0.206	0.033	4.049	940	WS00715_E11
10	21.864	188	3	-0.226	0.051	6.204	940	WS00715_E11
11	35.604	207	3	0.244	0.047	5.353	940	WS00715_E11
11	37.143	209	3	-0.272	0.058	6.674	940	WS00715_E11
11	48.979	218	2	-0.260	0.050	6.512	940	WS00715_E11
13	0.000	243	4	-0.249	0.143	3.926	940	WS00715_E11
1	82.301	16	4	-0.226	0.039	4.379	941	WS01021_P19
2	93.504	32	4	0.292	0.055	6.380	941	WS01021_P19
4	103.719	81	4	0.238	0.052	6.070	941	WS01021_P19
9	36.227	165	4	-0.215	0.041	5.088	941	WS01021_P19
11	60.464	221	2	0.274	0.045	4.523	941	WS01021_P19
11	64.014	222	4	0.285	0.054	5.091	941	WS01021_P19
13	1.815	244	4	-0.255	0.054	5.971	941	WS01021_P19
5	84.347	105	1	-0.260	0.054	4.248	942	WS00715_M11
6	25.914	114	1	-0.242	0.091	7.038	942	WS00715_M11
7	59.789	137	3	-0.258	0.088	7.091	942	WS00715_M11
11	41.984	212	2	-0.150	0.036	3.976	942	WS00715_M11
13	0.000	243	4	-0.328	0.176	6.892	942	WS00715_M11
1	45.794	9	1	0.310	0.074	6.232	943	WS00921_E02
3	69.071	52	1	-0.379	0.091	3.946	943	WS00921_E02
4	82.462	78	1	-0.180	0.057	4.682	943	WS00921_E02
4	173.929	88	2	-0.249	0.040	4.319	943	WS00921_E02
5	57.017	102	2	0.336	0.054	5.750	943	WS00921_E02
5	57.017	102	4	-0.397	0.104	11.508	943	WS00921_E02
7	105.477	147	2	-0.340	0.077	4.851	943	WS00921_E02
8	62.580	155	1	0.327	0.123	5.029	943	WS00921_E02
9	59.963	177	4	0.268	0.051	5.175	943	WS00921_E02
10	86.151	195	3	-0.208	0.056	4.724	943	WS00921_E02
10	93.347	196	1	-0.566	0.185	7.677	943	WS00921_E02
10	95.795	197	4	-0.218	0.050	5.113	943	WS00921_E02
12	22.629	237	1	-0.133	0.047	3.842	943	WS00921_E02
1	95.572	17	3	0.328	0.054	4.655	944	WS00945_C18
3	9.953	42	3	-0.160	0.033	3.847	944	WS00945_C18
3	14.500	43	1	-0.376	0.103	11.062	944	WS00945_C18
3	17.158	45	1	0.337	0.102	11.024	944	WS00945_C18
3	141.443	65	3	-0.278	0.064	4.709	944	WS00945_C18

4	82.462	78	1	-0.215	0.044	4.760	944	WS00945_C18
7	105.477	147	2	-0.290	0.121	4.622	944	WS00945_C18
10	9.580	187	3	-0.482	0.183	13.302	944	WS00945_C18
10	21.864	188	1	0.262	0.046	4.852	944	WS00945_C18
13	0.000	243	4	-0.378	0.158	5.371	944	WS00945_C18
13	3.386	245	1	0.226	0.045	4.888	944	WS00945_C18
1	131.686	23	1	0.595	0.100	7.327	945	WS00945_D23
5	0.000	89	1	-0.266	0.033	3.875	945	WS00945_D23
10	77.676	192	1	-0.320	0.053	6.080	945	WS00945_D23
11	56.921	219	1	0.240	0.097	6.991	945	WS00945_D23
12	6.242	234	2	-0.287	0.044	4.135	945	WS00945_D23
12	6.242	234	4	0.504	0.092	12.346	945	WS00945_D23
3	141.443	65	3	-0.387	0.089	6.027	946	WS00945_G21
6	81.996	123	3	-0.211	0.069	4.646	946	WS00945_G21
10	93.347	196	1	-0.604	0.130	8.815	946	WS00945_G21
13	1.815	244	2	0.184	0.046	4.120	946	WS00945_G21
14	22.402	248	4	-0.308	0.053	4.258	946	WS00945_G21
1	82.301	16	3	-0.369	0.094	9.326	947	WS00945_H18
2	63.261	30	3	0.253	0.047	4.700	947	WS00945_H18
2	75.550	31	1	0.314	0.050	4.878	947	WS00945_H18
2	93.504	32	2	-0.193	0.043	4.113	947	WS00945_H18
2	93.504	32	4	0.317	0.047	4.167	947	WS00945_H18
2	126.181	39	4	0.435	0.110	5.181	947	WS00945_H18
3	17.158	45	1	0.324	0.078	7.597	947	WS00945_H18
3	148.745	68	4	0.189	0.040	4.074	947	WS00945_H18
5	54.072	100	4	0.270	0.061	5.460	947	WS00945_H18
6	81.996	123	3	-0.398	0.090	5.071	947	WS00945_H18
6	89.401	125	1	-0.369	0.088	8.624	947	WS00945_H18
6	95.249	126	1	0.364	0.084	4.744	947	WS00945_H18
7	91.487	146	3	0.140	0.039	3.871	947	WS00945_H18
10	77.676	192	1	0.323	0.056	5.664	947	WS00945_H18
10	80.088	194	1	0.279	0.041	4.148	947	WS00945_H18
11	35.604	207	3	-0.162	0.046	4.557	947	WS00945_H18
11	46.026	217	1	-0.174	0.040	3.842	947	WS00945_H18
12	14.406	230	3	-0.386	0.073	7.389	947	WS00945_H18
13	0.000	243	4	-0.296	0.107	4.465	947	WS00945_H18
1	50.955	10	1	0.184	0.043	4.232	948	WS00945_N03
1	77.177	15	2	-0.204	0.044	3.888	948	WS00945_N03
3	15.147	44	2	-0.295	0.101	5.294	948	WS00945_N03
3	141.443	65	3	-0.341	0.089	6.378	948	WS00945_N03
5	57.017	102	2	0.229	0.043	3.861	948	WS00945_N03
9	55.358	175	3	0.507	0.111	5.967	948	WS00945_N03
11	0.000	199	3	-0.248	0.045	4.571	948	WS00945_N03
11	17.088	203	1	-0.240	0.065	6.463	948	WS00945_N03
12	0.000	226	3	0.298	0.068	7.462	948	WS00945_N03
2	95.880	34	1	0.337	0.054	4.964	949	WS00945_O14
3	137.891	64	3	-0.212	0.049	4.915	949	WS00945_O14
3	141.443	65	3	-0.268	0.062	3.921	949	WS00945_O14
7	59.789	137	3	-0.229	0.048	4.681	949	WS00945_O14
7	70.278	141	3	0.214	0.043	4.230	949	WS00945_O14
7	75.883	144	2	-0.394	0.065	4.238	949	WS00945_O14
10	80.088	194	1	0.272	0.057	5.292	949	WS00945_O14
11	0.000	199	3	-0.412	0.122	11.461	949	WS00945_O14
11	17.088	203	1	-0.226	0.042	3.884	949	WS00945_O14

12	0.000	226	3	0.190	0.041	4.001	949	WS00945_O14
3	9.953	42	3	0.204	0.045	4.935	950	WS00728_D17
3	69.071	52	1	-0.270	0.077	4.179	950	WS00728_D17
3	137.891	64	3	0.364	0.077	7.945	950	WS00728_D17
4	0.000	71	4	0.252	0.083	6.549	950	WS00728_D17
4	173.929	88	2	-0.206	0.043	4.876	950	WS00728_D17
5	57.017	102	4	-0.265	0.067	4.908	950	WS00728_D17
11	43.491	214	3	-0.206	0.054	5.845	950	WS00728_D17
14	0.000	246	2	-0.200	0.044	4.887	950	WS00728_D17
1	95.572	17	3	0.216	0.027	3.938	951	WS00728_E23
3	9.953	42	3	0.178	0.030	4.431	951	WS00728_E23
4	140.962	84	3	-0.176	0.031	4.502	951	WS00728_E23
6	25.914	114	1	-0.276	0.064	6.307	951	WS00728_E23
7	15.555	130	3	-0.298	0.055	7.571	951	WS00728_E23
9	55.358	175	3	0.464	0.065	5.491	951	WS00728_E23
11	37.143	209	3	0.193	0.030	4.399	951	WS00728_E23
3	137.891	64	3	0.269	0.037	4.106	952	WS00928_G10
4	116.928	82	2	-0.260	0.054	6.055	952	WS00928_G10
4	124.132	83	2	-0.288	0.108	5.713	952	WS00928_G10
9	49.013	173	2	0.178	0.035	3.955	952	WS00928_G10
10	78.479	193	3	0.315	0.040	4.431	952	WS00928_G10
11	29.069	205	2	-0.250	0.054	6.015	952	WS00928_G10
11	41.984	212	2	-0.211	0.037	4.216	952	WS00928_G10
11	43.491	214	3	-0.235	0.056	6.217	952	WS00928_G10
12	14.406	230	3	-0.212	0.035	3.845	952	WS00928_G10
2	101.076	35	4	-0.176	0.040	4.106	953	WS00928_G19
4	43.146	74	3	0.157	0.039	3.994	953	WS00928_G19
4	82.462	78	2	0.200	0.051	4.020	953	WS00928_G19
4	97.576	80	2	-0.320	0.069	7.756	953	WS00928_G19
5	3.484	90	2	-0.334	0.092	4.484	953	WS00928_G19
5	16.219	93	2	0.443	0.136	15.953	953	WS00928_G19
5	57.017	102	2	0.268	0.043	5.015	953	WS00928_G19
8	50.001	154	2	0.249	0.034	4.852	953	WS00928_G19
8	87.463	158	2	-0.118	0.038	5.729	953	WS00928_G19
9	28.608	170	3	0.230	0.058	5.800	953	WS00928_G19
11	9.237	202	4	0.219	0.068	5.795	953	WS00928_G19
11	43.491	214	3	-0.243	0.050	5.156	953	WS00928_G19
11	44.884	215	3	0.196	0.059	6.134	953	WS00928_G19
11	48.979	218	2	-0.245	0.072	8.120	953	WS00928_G19
3	141.443	65	3	-0.342	0.098	5.791	954	WS00928_H23
4	82.462	78	1	-0.212	0.066	5.707	954	WS00928_H23
6	25.742	113	3	-0.181	0.036	4.474	954	WS00928_H23
6	49.100	118	2	-0.331	0.083	5.468	954	WS00928_H23
9	69.046	181	2	0.310	0.093	6.196	954	WS00928_H23
10	9.580	187	3	-0.317	0.124	7.122	954	WS00928_H23
13	3.386	245	1	0.178	0.050	4.374	954	WS00928_H23
5	3.484	90	2	-0.377	0.104	6.769	955	WS00928_I12
11	35.604	207	3	-0.247	0.056	5.223	955	WS00928_I12
11	37.143	209	3	0.257	0.062	5.711	955	WS00928_I12
11	42.600	213	3	-0.238	0.049	4.535	955	WS00928_I12
14	0.000	246	2	-0.194	0.038	4.414	955	WS00928_I12
1	62.128	11	1	-0.723	0.202	11.628	956	WS00928_J21
3	9.953	42	4	0.333	0.088	11.397	956	WS00928_J21
5	0.000	89	1	-0.288	0.061	5.338	956	WS00928_J21

5	16.219	93	1	-0.246	0.056	4.850	956	WS00928_J21
7	105.477	147	2	0.217	0.056	4.058	956	WS00928_J21
9	0.000	166	1	0.232	0.072	6.179	956	WS00928_J21
12	28.493	228	2	0.221	0.058	4.186	956	WS00928_J21
13	1.815	244	4	0.270	0.057	7.881	956	WS00928_J21
4	142.917	85	3	-0.162	0.029	4.011	957	WS00928_K08
5	7.672	92	1	-0.181	0.034	5.136	957	WS00928_K08
6	106.702	128	3	-0.246	0.033	4.333	957	WS00928_K08
7	105.477	147	2	0.254	0.144	4.311	957	WS00928_K08
10	9.580	187	3	0.240	0.062	5.375	957	WS00928_K08
12	6.242	234	2	0.279	0.093	9.026	957	WS00928_K08
12	22.629	237	1	0.250	0.060	6.724	957	WS00928_K08
12	32.473	238	1	0.184	0.035	3.888	957	WS00928_K08
4	0.000	71	4	0.247	0.094	7.098	958	WS00928_L14
11	43.491	214	3	-0.228	0.043	4.815	958	WS00928_L14
11	56.921	219	1	-0.262	0.108	4.167	958	WS00928_L14
14	44.898	251	3	0.215	0.039	4.475	958	WS00928_L14
5	56.008	101	1	-0.210	0.048	4.418	959	WS00928_M02
6	25.742	113	3	-0.176	0.034	4.276	959	WS00928_M02
6	25.914	114	1	-0.222	0.050	4.562	959	WS00928_M02
6	56.188	120	2	0.266	0.047	4.537	959	WS00928_M02
6	87.891	124	2	0.187	0.042	4.050	959	WS00928_M02
7	54.200	136	2	0.270	0.090	5.083	959	WS00928_M02
9	49.013	173	1	-0.240	0.054	5.141	959	WS00928_M02
13	3.386	245	1	0.277	0.066	6.006	959	WS00928_M02
14	22.402	248	3	-0.271	0.037	4.689	959	WS00928_M02
3	95.157	59	2	-0.213	0.039	3.895	960	WS0089_N09
4	82.462	78	1	-0.220	0.047	4.479	960	WS0089_N09
11	40.960	210	2	0.389	0.099	4.278	960	WS0089_N09
11	40.960	210	4	-0.293	0.056	4.218	960	WS0089_N09
12	14.406	230	3	-0.229	0.038	4.337	960	WS0089_N09
8	50.001	154	2	-0.221	0.034	4.903	961	WS00928_O05
11	27.405	204	1	0.311	0.077	4.403	961	WS00928_O05
11	64.014	222	4	-0.246	0.081	4.767	961	WS00928_O05
12	22.441	227	1	0.231	0.071	4.053	961	WS00928_O05
13	1.815	244	2	0.208	0.053	6.891	961	WS00928_O05
14	0.000	246	2	-0.204	0.028	4.038	961	WS00928_O05
3	17.158	45	1	0.220	0.048	4.781	962	WS00810_C04
4	43.146	74	3	0.163	0.049	4.803	962	WS00810_C04
4	82.462	78	1	-0.296	0.048	4.832	962	WS00810_C04
4	116.928	82	4	-0.317	0.075	6.926	962	WS00810_C04
7	73.169	142	1	0.480	0.080	3.962	962	WS00810_C04
8	11.974	150	1	-0.251	0.058	5.727	962	WS00810_C04
9	3.127	168	1	-0.496	0.125	7.952	962	WS00810_C04
11	43.491	214	3	-0.275	0.057	5.663	962	WS00810_C04
14	0.000	246	2	-0.179	0.048	4.256	962	WS00810_C04
14	44.898	251	3	0.229	0.039	3.898	962	WS00810_C04
6	49.100	118	2	-0.284	0.049	4.653	963	WS00929_B08
7	15.555	130	2	0.258	0.038	4.766	963	WS00929_B08
7	54.200	136	2	0.400	0.098	9.397	963	WS00929_B08
9	36.227	165	3	0.291	0.060	8.095	963	WS00929_B08
9	81.073	183	3	-0.218	0.030	3.995	963	WS00929_B08
11	46.026	217	1	-0.246	0.072	4.604	963	WS00929_B08
14	46.637	252	3	0.254	0.035	4.632	963	WS00929_B08

4	82.462	78	1	-0.319	0.058	5.294	964	WS01021_G18
4	140.962	84	3	-0.149	0.043	4.001	964	WS01021_G18
5	54.072	100	4	0.236	0.050	4.787	964	WS01021_G18
5	57.017	102	4	-0.351	0.071	6.735	964	WS01021_G18
6	25.914	114	1	-0.233	0.052	4.503	964	WS01021_G18
7	75.883	144	2	0.404	0.076	4.207	964	WS01021_G18
11	46.026	217	1	-0.281	0.074	6.455	964	WS01021_G18
14	44.898	251	3	0.311	0.104	10.036	964	WS01021_G18
1	22.419	4	3	0.335	0.106	4.385	965	WS01021_K13
3	69.071	52	1	0.371	0.148	6.091	965	WS01021_K13
4	103.719	81	4	0.238	0.058	5.404	965	WS01021_K13
4	173.929	88	2	0.264	0.059	7.336	965	WS01021_K13
6	69.073	121	3	-0.219	0.049	5.013	965	WS01021_K13
6	81.996	123	3	-0.390	0.160	6.583	965	WS01021_K13
11	17.088	203	2	-0.249	0.056	6.354	965	WS01021_K13
11	40.960	210	2	-0.396	0.065	4.283	965	WS01021_K13
11	56.921	219	2	0.259	0.063	3.849	965	WS01021_K13
12	14.406	230	3	-0.288	0.053	5.455	965	WS01021_K13
4	0.000	71	4	-0.214	0.061	5.218	966	WS0102_H18
5	52.820	99	3	0.266	0.049	4.982	966	WS0102_H18
6	45.897	117	4	-0.240	0.062	5.437	966	WS0102_H18
6	106.702	128	2	0.484	0.124	6.194	966	WS0102_H18
14	22.402	248	4	-0.331	0.051	4.267	966	WS0102_H18
1	45.794	9	1	0.414	0.065	7.821	967	WS0102_I07
2	48.839	29	4	-0.330	0.059	5.345	967	WS0102_I07
4	92.039	79	4	-0.353	0.041	4.737	967	WS0102_I07
5	0.000	89	3	0.161	0.037	3.923	967	WS0102_I07
5	57.017	102	4	-0.419	0.087	6.210	967	WS0102_I07
8	62.580	155	1	0.250	0.096	6.192	967	WS0102_I07
8	87.463	158	2	-0.157	0.067	4.216	967	WS0102_I07
9	3.127	168	1	-0.380	0.031	5.319	967	WS0102_I07
11	17.088	203	1	-0.146	0.036	4.205	967	WS0102_I07
3	137.891	64	3	0.261	0.051	5.265	968	WS0102_K02
4	0.000	71	4	0.259	0.073	6.128	968	WS0102_K02
4	173.929	88	2	-0.250	0.060	6.736	968	WS0102_K02
5	54.072	100	4	0.194	0.054	4.480	968	WS0102_K02
5	57.017	102	4	-0.217	0.047	3.928	968	WS0102_K02
10	70.246	190	2	-0.273	0.047	5.311	968	WS0102_K02
14	44.898	251	3	0.200	0.037	3.852	968	WS0102_K02
3	9.953	42	3	0.192	0.036	4.040	969	WS0102_K15
3	137.891	64	3	0.303	0.056	5.781	969	WS0102_K15
4	140.962	84	3	-0.185	0.034	3.863	969	WS0102_K15
4	173.929	88	2	-0.245	0.067	6.740	969	WS0102_K15
6	81.996	123	3	0.261	0.065	4.071	969	WS0102_K15
9	0.000	166	1	-0.250	0.051	4.502	969	WS0102_K15
10	78.479	193	3	0.335	0.046	5.209	969	WS0102_K15
3	137.891	64	3	0.243	0.051	5.255	970	WS0102_M10
4	0.000	71	4	0.241	0.063	5.366	970	WS0102_M10
4	173.929	88	2	-0.243	0.056	6.351	970	WS0102_M10
5	54.072	100	4	0.213	0.050	4.260	970	WS0102_M10
5	57.017	102	4	-0.280	0.071	5.904	970	WS0102_M10
6	81.996	123	3	0.281	0.091	4.663	970	WS0102_M10
8	87.463	158	2	-0.244	0.053	5.437	970	WS0102_M10
10	70.246	190	2	-0.250	0.044	4.932	970	WS0102_M10

11	27.405	204	4	-0.287	0.063	4.530	970	WS0102_M10
14	44.898	251	3	0.245	0.047	4.800	970	WS0102_M10
7	15.555	130	3	-0.242	0.048	4.414	971	WS0102_M22
7	75.883	144	2	0.410	0.079	4.433	971	WS0102_M22
8	0.000	148	4	-0.189	0.040	3.882	971	WS0102_M22
8	62.580	155	1	-0.323	0.139	5.710	971	WS0102_M22
10	78.479	193	3	0.309	0.059	5.427	971	WS0102_M22
10	86.151	195	3	-0.226	0.051	4.696	971	WS0102_M22
3	9.953	42	3	0.200	0.038	4.078	972	WS0102_O21
8	87.463	158	2	-0.227	0.050	3.944	972	WS0102_O21
3	137.891	64	3	0.214	0.044	4.177	973	WS0102_P09
4	0.000	71	4	0.274	0.076	6.932	973	WS0102_P09
5	54.072	100	4	0.246	0.058	5.344	973	WS0102_P09
5	57.017	102	4	-0.314	0.077	6.907	973	WS0102_P09
8	87.463	158	2	-0.258	0.069	6.645	973	WS0102_P09
10	70.246	190	2	-0.232	0.049	4.827	973	WS0102_P09
3	137.891	64	3	0.292	0.048	5.296	974	WS0264_M01
6	106.702	128	2	0.389	0.067	4.151	974	WS0264_M01
9	36.227	165	3	0.193	0.049	5.400	974	WS0264_M01
9	2.011	167	4	-0.220	0.052	4.047	974	WS0264_M01
9	49.013	173	2	0.213	0.049	5.138	974	WS0264_M01
13	0.000	243	4	0.361	0.145	3.945	974	WS0264_M01
1	62.128	11	1	-0.773	0.219	11.368	975	WS0264_M13
2	120.617	38	4	-0.286	0.053	5.369	975	WS0264_M13
6	45.897	117	1	0.221	0.041	4.082	975	WS0264_M13
8	95.937	159	3	0.194	0.056	4.938	975	WS0264_M13
9	81.073	183	3	-0.286	0.066	5.796	975	WS0264_M13
13	0.000	243	4	0.411	0.088	4.593	975	WS0264_M13
4	0.000	71	4	0.263	0.041	4.560	976	WS0264_O06
8	87.463	158	2	-0.311	0.075	7.825	976	WS0264_O06
9	49.013	173	2	0.192	0.045	5.391	976	WS0264_O06
10	21.864	188	3	0.284	0.063	4.962	976	WS0264_O06
11	64.014	222	4	-0.351	0.069	6.945	976	WS0264_O06
1	45.794	9	1	-0.249	0.050	4.445	977	WS0264_O18
1	95.572	17	3	0.283	0.059	5.544	977	WS0264_O18
4	116.928	82	4	0.312	0.048	4.271	977	WS0264_O18
7	15.555	130	3	-0.230	0.042	3.878	977	WS0264_O18
7	54.200	136	2	0.267	0.085	4.497	977	WS0264_O18
9	2.011	167	4	-0.190	0.055	5.221	977	WS0264_O18
10	78.479	193	3	-0.323	0.076	6.976	977	WS0264_O18
11	46.026	217	1	-0.252	0.053	4.717	977	WS0264_O18
3	9.953	42	3	0.153	0.047	4.291	978	WS0264_P22
3	137.891	64	3	0.433	0.120	9.321	978	WS0264_P22
6	95.249	126	1	0.382	0.129	5.362	978	WS0264_P22
9	36.227	165	4	-0.189	0.035	3.958	978	WS0264_P22
9	49.013	173	2	0.184	0.038	4.779	978	WS0264_P22
1	2.788	2	3	-0.271	0.072	9.629	979	WS00824_A05
1	45.794	9	1	0.302	0.049	4.698	979	WS00824_A05
1	45.794	9	3	-0.297	0.048	6.626	979	WS00824_A05
4	82.462	78	1	-0.334	0.083	7.704	979	WS00824_A05
5	57.017	102	2	0.217	0.046	4.520	979	WS00824_A05
6	26.350	115	2	0.193	0.045	4.269	979	WS00824_A05
7	17.610	131	3	0.347	0.090	5.744	979	WS00824_A05
7	91.487	146	3	0.206	0.038	5.159	979	WS00824_A05

9	36.227	165	3	0.191	0.035	4.685	979	WS00824_A05
10	9.580	187	3	-0.347	0.085	5.660	979	WS00824_A05
10	78.479	193	3	-0.310	0.038	5.172	979	WS00824_A05
12	14.406	230	3	-0.246	0.036	4.866	979	WS00824_A05
3	141.443	65	3	-0.232	0.078	3.877	980	WS00824_B14
4	0.000	71	4	0.155	0.062	4.118	980	WS00824_B14
4	82.462	78	1	-0.287	0.046	3.945	980	WS00824_B14
5	0.000	89	1	-0.160	0.050	4.433	980	WS00824_B14
5	0.000	89	3	0.257	0.036	4.274	980	WS00824_B14
5	57.017	102	2	0.323	0.079	8.386	980	WS00824_B14
7	17.610	131	3	0.314	0.065	4.646	980	WS00824_B14
7	54.200	136	2	0.402	0.109	7.853	980	WS00824_B14
7	70.278	141	3	-0.230	0.044	5.325	980	WS00824_B14
8	87.463	158	2	-0.170	0.038	4.020	980	WS00824_B14
10	9.580	187	3	-0.393	0.140	7.057	980	WS00824_B14
11	17.088	203	1	-0.230	0.071	5.914	980	WS00824_B14
13	1.815	244	2	0.270	0.060	6.169	980	WS00824_B14
14	44.898	251	3	0.247	0.039	4.588	980	WS00824_B14
3	137.891	64	3	0.220	0.038	4.313	981	WS00824_C05
6	87.891	124	2	0.285	0.089	9.487	981	WS00824_C05
6	89.401	125	2	0.285	0.058	5.952	981	WS00824_C05
7	15.555	130	3	-0.273	0.070	7.587	981	WS00824_C05
7	75.883	144	4	-0.386	0.089	4.890	981	WS00824_C05
11	45.989	216	1	-0.240	0.057	4.651	981	WS00824_C05
1	95.572	17	2	-0.195	0.038	3.865	982	WS0104_H03
5	0.000	89	1	0.222	0.051	4.511	982	WS0104_H03
5	86.669	106	2	0.267	0.105	4.042	982	WS0104_H03
6	26.350	115	2	0.210	0.040	4.053	982	WS0104_H03
6	89.401	125	1	-0.247	0.046	3.982	982	WS0104_H03
7	74.560	143	1	0.279	0.050	4.403	982	WS0104_H03
9	0.000	166	1	-0.241	0.053	4.602	982	WS0104_H03
9	17.951	169	2	0.218	0.039	4.419	982	WS0104_H03
9	28.608	170	2	-0.287	0.071	7.295	982	WS0104_H03
10	9.580	187	3	0.353	0.122	5.308	982	WS0104_H03
10	35.204	189	1	0.226	0.045	3.868	982	WS0104_H03
10	77.676	192	1	0.227	0.046	4.026	982	WS0104_H03
10	80.088	194	1	0.369	0.075	6.624	982	WS0104_H03
11	35.604	207	3	-0.227	0.051	5.062	982	WS0104_H03
11	37.143	209	3	0.250	0.062	6.113	982	WS0104_H03
12	22.441	227	3	-0.224	0.044	4.345	982	WS0104_H03
14	22.402	248	3	-0.273	0.059	5.828	982	WS0104_H03
1	22.419	4	4	-0.465	0.161	7.320	983	WS0104_H16
1	38.326	6	4	-0.400	0.102	8.233	983	WS0104_H16
1	40.464	7	4	0.353	0.079	5.037	983	WS0104_H16
2	93.504	32	4	0.344	0.061	3.914	983	WS0104_H16
4	55.925	76	1	-0.232	0.054	4.497	983	WS0104_H16
4	124.132	83	1	-0.473	0.099	4.501	983	WS0104_H16
5	56.008	101	2	-0.201	0.046	4.191	983	WS0104_H16
7	59.789	137	3	0.197	0.039	4.135	983	WS0104_H16
8	39.090	152	4	0.259	0.087	7.192	983	WS0104_H16
8	50.001	154	2	-0.192	0.042	4.358	983	WS0104_H16
9	55.358	175	1	0.337	0.075	6.702	983	WS0104_H16
10	21.864	188	1	0.310	0.062	5.390	983	WS0104_H16
12	14.406	230	4	0.294	0.047	3.943	983	WS0104_H16

14	0.000	246	1	-0.217	0.044	3.849	983	WS0104_H16
14	46.637	252	3	0.373	0.085	8.959	983	WS0104_H16
3	9.953	42	3	-0.296	0.070	6.258	984	WS0104_J11
5	57.017	102	4	0.266	0.064	7.002	984	WS0104_J11
6	76.376	122	1	0.364	0.102	3.926	984	WS0104_J11
9	0.000	166	1	-0.197	0.043	3.916	984	WS0104_J11
12	28.493	228	2	0.279	0.093	5.723	984	WS0104_J11
14	44.898	251	1	-0.334	0.057	5.224	984	WS0104_J11
1	40.464	7	4	0.301	0.038	3.997	985	WS0104_K09
2	75.550	31	1	0.393	0.062	6.827	985	WS0104_K09
3	17.158	45	1	0.261	0.071	7.630	985	WS0104_K09
3	47.928	49	1	0.175	0.037	3.959	985	WS0104_K09
4	92.039	79	4	-0.317	0.044	3.975	985	WS0104_K09
5	57.017	102	2	0.256	0.082	6.641	985	WS0104_K09
6	25.914	114	1	-0.195	0.043	4.661	985	WS0104_K09
6	49.100	118	2	-0.330	0.094	5.110	985	WS0104_K09
7	75.883	144	2	0.409	0.144	4.496	985	WS0104_K09
7	75.883	144	4	-0.319	0.049	4.723	985	WS0104_K09
9	3.127	168	1	-0.401	0.039	4.933	985	WS0104_K09
11	64.014	222	3	0.245	0.040	3.974	985	WS0104_K09
12	51.382	242	1	-0.383	0.064	4.288	985	WS0104_K09
13	3.386	245	1	0.295	0.041	4.672	985	WS0104_K09
14	44.898	251	3	0.180	0.042	4.297	985	WS0104_K09
14	46.637	252	3	0.198	0.045	4.240	985	WS0104_K09
3	80.211	55	4	-0.102	0.028	3.955	986	WS0104_M09
4	103.719	81	4	-0.265	0.024	3.863	986	WS0104_M09
4	116.928	82	2	0.339	0.037	4.637	986	WS0104_M09
4	124.132	83	1	-0.624	0.161	7.813	986	WS0104_M09
5	57.017	102	4	0.222	0.029	5.594	986	WS0104_M09
6	49.100	118	4	-0.448	0.120	7.088	986	WS0104_M09
7	62.787	139	4	-0.320	0.045	5.734	986	WS0104_M09
9	49.013	173	1	-0.352	0.133	9.645	986	WS0104_M09
9	55.358	175	1	0.262	0.093	6.567	986	WS0104_M09
9	59.963	177	1	-0.301	0.058	4.271	986	WS0104_M09
11	45.989	216	1	0.232	0.086	6.010	986	WS0104_M09
12	22.441	227	1	0.215	0.058	4.021	986	WS0104_M09
1	62.128	11	1	0.482	0.107	4.664	987	WS0104_M21
2	75.550	31	2	-0.268	0.066	5.427	987	WS0104_M21
3	17.158	45	1	-0.186	0.040	3.994	987	WS0104_M21
4	82.462	78	1	0.230	0.044	4.422	987	WS0104_M21
4	103.719	81	4	0.233	0.051	4.923	987	WS0104_M21
5	3.484	90	2	0.390	0.153	7.577	987	WS0104_M21
5	16.219	93	2	-0.309	0.075	6.497	987	WS0104_M21
7	15.555	130	3	0.435	0.130	11.299	987	WS0104_M21
8	39.090	152	4	0.266	0.075	6.950	987	WS0104_M21
9	81.073	183	1	-0.318	0.050	5.045	987	WS0104_M21
10	9.580	187	3	0.273	0.074	4.301	987	WS0104_M21
10	21.864	188	1	-0.302	0.049	4.806	987	WS0104_M21
11	0.000	199	3	-0.305	0.064	4.937	987	WS0104_M21
11	17.088	203	1	-0.254	0.048	4.839	987	WS0104_M21
11	35.604	207	3	0.261	0.074	7.013	987	WS0104_M21
11	37.143	209	3	-0.302	0.100	9.516	987	WS0104_M21
11	42.600	213	3	0.217	0.047	4.477	987	WS0104_M21
14	46.637	252	3	-0.272	0.047	4.484	987	WS0104_M21

6	25.914	114	1	-0.256	0.050	4.889	988	WS0104_O19
7	67.154	140	4	-0.263	0.103	4.384	988	WS0104_O19
7	75.883	144	2	0.415	0.104	4.901	988	WS0104_O19
7	75.883	144	4	-0.379	0.084	7.394	988	WS0104_O19
9	3.127	168	1	-0.400	0.084	5.958	988	WS0104_O19
11	17.088	203	1	-0.231	0.045	4.334	988	WS0104_O19
12	14.406	230	3	-0.258	0.042	4.438	988	WS0104_O19
1	43.578	8	2	0.265	0.035	4.236	989	WS0104_P11
1	95.572	17	3	0.248	0.038	4.010	989	WS0104_P11
1	133.992	24	2	0.187	0.039	4.642	989	WS0104_P11
1	141.021	25	2	-0.203	0.046	5.463	989	WS0104_P11
4	0.000	71	4	-0.221	0.055	4.621	989	WS0104_P11
5	57.017	102	2	0.280	0.048	5.950	989	WS0104_P11
6	26.350	115	2	0.197	0.041	4.982	989	WS0104_P11
7	15.555	130	3	0.274	0.071	7.672	989	WS0104_P11
7	17.610	131	3	0.436	0.121	7.219	989	WS0104_P11
11	48.979	218	4	-0.228	0.054	4.457	989	WS0104_P11
2	127.147	40	3	0.231	0.049	5.147	990	WS00731_D20
3	17.158	45	1	0.242	0.041	4.033	990	WS00731_D20
3	148.745	68	4	0.186	0.051	3.942	990	WS00731_D20
4	75.958	77	3	-0.236	0.089	4.138	990	WS00731_D20
4	82.462	78	1	0.270	0.072	6.922	990	WS00731_D20
5	0.000	89	1	-0.311	0.077	7.864	990	WS00731_D20
5	16.219	93	1	-0.269	0.070	6.687	990	WS00731_D20
5	52.820	99	1	0.262	0.051	4.945	990	WS00731_D20
7	73.169	142	2	-0.417	0.093	3.938	990	WS00731_D20
8	0.000	148	4	-0.236	0.069	5.705	990	WS00731_D20
11	44.884	215	3	0.276	0.053	5.602	990	WS00731_D20
3	95.157	59	2	-0.179	0.032	4.156	991	WS00112_G24
3	104.681	60	2	0.198	0.033	4.210	991	WS00112_G24
3	114.747	62	2	-0.201	0.036	4.597	991	WS00112_G24
4	116.928	82	2	-0.260	0.041	5.143	991	WS00112_G24
4	116.928	82	4	0.241	0.080	4.367	991	WS00112_G24
4	124.132	83	2	-0.318	0.077	5.963	991	WS00112_G24
5	3.484	90	2	-0.269	0.127	4.047	991	WS00112_G24
5	86.669	106	2	-0.245	0.046	4.313	991	WS00112_G24
7	17.610	131	3	0.362	0.078	7.378	991	WS00112_G24
9	36.227	165	3	0.271	0.058	7.907	991	WS00112_G24
2	95.880	34	1	0.366	0.063	6.377	992	WS00731_O11
3	9.953	42	3	0.252	0.046	5.703	992	WS00731_O11
3	95.157	59	2	-0.197	0.043	4.107	992	WS00731_O11
4	140.962	84	3	-0.207	0.033	4.066	992	WS00731_O11
5	42.979	97	2	-0.201	0.059	5.275	992	WS00731_O11
11	37.143	209	3	0.226	0.038	4.733	992	WS00731_O11
1	50.955	10	1	-0.231	0.058	4.855	993	WS00113_A19
1	95.572	17	2	-0.273	0.069	7.305	993	WS00113_A19
1	95.572	17	3	0.237	0.047	5.434	993	WS00113_A19
2	93.504	32	2	0.286	0.068	7.268	993	WS00113_A19
2	126.181	39	2	-0.229	0.043	4.437	993	WS00113_A19
3	14.500	43	1	-0.245	0.063	5.255	993	WS00113_A19
3	14.500	43	2	-0.262	0.046	4.785	993	WS00113_A19
3	47.928	49	1	0.228	0.050	4.172	993	WS00113_A19
4	82.462	78	1	-0.264	0.047	4.038	993	WS00113_A19
4	92.039	79	4	-0.380	0.094	5.784	993	WS00113_A19

5	52.820	99	3	0.350	0.092	10.512	993	WS00113_A19
5	57.017	102	2	0.213	0.045	4.772	993	WS00113_A19
7	15.555	130	3	-0.226	0.045	5.164	993	WS00113_A19
7	17.610	131	3	-0.277	0.064	3.997	993	WS00113_A19
7	59.789	137	3	-0.170	0.040	4.547	993	WS00113_A19
10	86.151	195	3	-0.248	0.043	5.005	993	WS00113_A19
11	42.600	213	3	-0.181	0.040	4.597	993	WS00113_A19
11	46.026	217	1	-0.279	0.079	6.599	993	WS00113_A19
12	14.406	230	3	-0.275	0.043	4.938	993	WS00113_A19
1	62.128	11	1	-0.487	0.104	4.496	994	WS00941_M04
2	102.814	36	2	-0.222	0.070	5.446	994	WS00941_M04
4	124.132	83	1	-0.595	0.164	7.103	994	WS00941_M04
4	142.917	85	1	0.224	0.046	4.093	994	WS00941_M04
5	86.669	106	4	-0.192	0.073	3.990	994	WS00941_M04
9	36.227	165	3	0.180	0.039	3.934	994	WS00941_M04
9	81.073	183	3	-0.246	0.077	7.565	994	WS00941_M04
11	27.405	204	1	0.290	0.050	4.590	994	WS00941_M04
11	64.014	222	4	-0.240	0.063	4.420	994	WS00941_M04
1	50.955	10	1	0.246	0.069	5.457	995	WS01040_E21
3	154.505	69	4	-0.244	0.047	4.750	995	WS01040_E21
6	76.376	122	1	-0.339	0.138	5.144	995	WS01040_E21
8	39.090	152	4	0.237	0.053	5.656	995	WS01040_E21
10	35.204	189	1	-0.250	0.058	4.573	995	WS01040_E21
14	0.000	246	2	0.190	0.038	4.043	995	WS01040_E21
8	87.463	158	2	-0.238	0.071	5.779	996	WS0014_I11
11	46.026	217	1	-0.235	0.041	4.039	996	WS0014_I11
14	44.898	251	3	0.240	0.053	4.479	996	WS0014_I11
1	95.572	17	3	0.267	0.049	4.195	997	WS0109_O13
2	48.839	29	1	-0.240	0.040	4.185	997	WS0109_O13
2	95.880	34	3	0.254	0.054	5.713	997	WS0109_O13
2	101.076	35	3	-0.224	0.039	4.425	997	WS0109_O13
2	120.617	38	3	-0.215	0.045	5.083	997	WS0109_O13
2	127.147	40	3	0.246	0.049	5.554	997	WS0109_O13
6	26.350	115	2	0.180	0.047	4.007	997	WS0109_O13
7	62.787	139	2	0.170	0.048	4.623	997	WS0109_O13
7	62.787	139	4	-0.348	0.078	7.084	997	WS0109_O13
7	67.154	140	4	-0.387	0.172	6.125	997	WS0109_O13
7	75.883	144	2	0.671	0.220	10.993	997	WS0109_O13
7	75.883	144	4	-0.464	0.157	12.526	997	WS0109_O13
8	50.001	154	1	0.193	0.040	4.157	997	WS0109_O13
8	62.580	155	1	-0.381	0.126	4.517	997	WS0109_O13
10	104.214	198	2	-0.217	0.056	4.521	997	WS0109_O13
11	17.088	203	1	0.253	0.052	5.346	997	WS0109_O13
13	3.386	245	1	0.357	0.108	11.056	997	WS0109_O13
4	6.032	72	4	0.208	0.052	4.428	998	WS0015_H11
4	75.958	77	3	-0.212	0.085	4.470	998	WS0015_H11
4	82.462	78	1	-0.296	0.082	7.276	998	WS0015_H11
5	0.000	89	1	-0.245	0.049	4.516	998	WS0015_H11
5	16.219	93	1	-0.207	0.048	4.181	998	WS0015_H11
5	31.191	95	1	-0.259	0.048	4.241	998	WS0015_H11
6	25.914	114	1	-0.276	0.051	4.538	998	WS0015_H11
11	27.405	204	1	0.324	0.065	5.864	998	WS0015_H11
11	27.405	204	4	-0.276	0.062	5.364	998	WS0015_H11
11	40.960	210	2	0.493	0.133	6.926	998	WS0015_H11

11	64.014	222	4	-0.360	0.092	7.718	998	WS0015_H11
3	69.071	52	1	-0.311	0.097	4.444	999	WS0081_G08
4	0.000	71	4	0.172	0.042	4.362	999	WS0081_G08
10	35.204	189	2	-0.197	0.039	4.113	999	WS0081_G08
11	64.014	222	4	-0.283	0.040	4.181	999	WS0081_G08
13	0.000	243	4	0.346	0.111	4.217	999	WS0081_G08
1	40.464	7	4	0.271	0.042	4.069	1000	WS00929_E14
6	25.914	114	1	-0.233	0.048	3.904	1000	WS00929_E14
6	49.100	118	2	-0.275	0.082	4.604	1000	WS00929_E14
8	87.463	158	2	-0.219	0.040	4.352	1000	WS00929_E14
9	3.127	168	1	-0.290	0.070	4.385	1000	WS00929_E14
11	43.491	214	3	-0.251	0.051	5.120	1000	WS00929_E14
11	46.026	217	1	-0.207	0.053	4.255	1000	WS00929_E14
14	44.898	251	3	0.324	0.078	6.430	1000	WS00929_E14
1	107.333	19	4	0.239	0.039	4.012	1001	WS00929_H20
2	126.181	39	4	0.475	0.077	6.348	1001	WS00929_H20
3	9.953	42	4	-0.162	0.039	4.602	1001	WS00929_H20
3	14.500	43	1	-0.274	0.090	5.705	1001	WS00929_H20
3	17.158	45	1	0.210	0.075	4.738	1001	WS00929_H20
3	144.593	66	4	-0.239	0.042	4.667	1001	WS00929_H20
3	148.745	68	4	0.297	0.046	5.460	1001	WS00929_H20
4	116.928	82	4	-0.357	0.063	7.087	1001	WS00929_H20
6	25.914	114	1	-0.241	0.094	5.979	1001	WS00929_H20
11	64.014	222	4	0.284	0.050	5.463	1001	WS00929_H20
14	43.392	250	2	-0.169	0.032	4.022	1001	WS00929_H20
1	50.955	10	1	-0.238	0.057	4.144	1002	WS0019_F03
2	126.181	39	2	-0.221	0.038	4.064	1002	WS0019_F03
3	14.500	43	1	-0.300	0.090	6.535	1002	WS0019_F03
3	17.158	45	1	0.213	0.060	4.378	1002	WS0019_F03
5	52.820	99	3	0.254	0.048	5.000	1002	WS0019_F03
5	57.017	102	2	0.325	0.078	9.097	1002	WS0019_F03
7	59.789	137	3	-0.180	0.043	4.645	1002	WS0019_F03
8	95.937	159	3	0.223	0.040	4.348	1002	WS0019_F03
10	86.151	195	3	-0.230	0.042	4.516	1002	WS0019_F03
11	46.026	217	1	-0.238	0.055	4.046	1002	WS0019_F03
12	14.406	230	3	-0.243	0.039	4.223	1002	WS0019_F03
14	9.923	247	1	0.255	0.065	4.777	1002	WS0019_F03
1	2.788	2	3	-0.207	0.083	8.738	1003	WS0019_G21
1	22.419	4	4	0.222	0.076	3.854	1003	WS0019_G21
6	49.100	118	4	0.368	0.134	4.330	1003	WS0019_G21
8	95.937	159	3	0.395	0.092	9.911	1003	WS0019_G21
9	0.000	163	1	-0.564	0.106	7.010	1003	WS0019_G21
9	28.608	170	2	-0.349	0.060	5.655	1003	WS0019_G21
9	28.608	170	3	0.256	0.042	4.904	1003	WS0019_G21
11	46.026	217	1	-0.238	0.092	9.222	1003	WS0019_G21
11	64.014	222	4	-0.216	0.047	4.918	1003	WS0019_G21
12	14.406	230	3	-0.306	0.036	3.996	1003	WS0019_G21
2	127.147	40	3	-0.222	0.039	5.017	1004	WS0019_M10
3	14.500	43	1	-0.222	0.055	4.407	1005	WS0019_N20
3	17.158	45	1	0.268	0.074	5.935	1005	WS0019_N20
8	95.937	159	3	0.236	0.040	3.950	1005	WS0019_N20
9	2.011	167	4	-0.245	0.057	5.195	1005	WS0019_N20
10	9.580	187	3	-0.355	0.120	4.472	1005	WS0019_N20
11	27.405	204	4	0.204	0.046	4.225	1005	WS0019_N20

11	64.014	222	4	-0.297	0.047	4.310	1005	WS0019_N20
14	0.000	246	2	-0.206	0.048	4.584	1005	WS0019_N20
1	40.464	7	4	0.137	0.042	5.332	1006	WS00110_C01
2	102.814	36	2	0.247	0.024	4.006	1006	WS00110_C01
2	127.147	40	4	0.293	0.091	8.098	1006	WS00110_C01
11	0.000	199	2	0.261	0.033	3.942	1006	WS00110_C01
11	64.014	222	4	-0.200	0.061	6.075	1006	WS00110_C01
12	22.441	227	1	0.173	0.052	4.250	1006	WS00110_C01
14	0.000	246	2	-0.220	0.024	3.990	1006	WS00110_C01
2	75.550	31	2	0.204	0.054	4.147	1007	WS00110_C19
8	50.001	154	1	0.197	0.041	4.891	1007	WS00110_C19
8	62.580	155	1	0.246	0.082	5.177	1007	WS00110_C19
9	3.127	168	1	-0.392	0.055	5.390	1007	WS00110_C19
10	35.204	189	2	0.323	0.090	7.007	1007	WS00110_C19
10	78.479	193	3	-0.306	0.056	4.931	1007	WS00110_C19
14	0.000	246	1	0.305	0.093	10.017	1007	WS00110_C19
1	107.333	19	3	0.202	0.045	4.753	1008	WS00110_F15
4	92.039	79	2	-0.218	0.043	4.725	1008	WS00110_F15
11	40.960	210	2	0.420	0.079	4.884	1008	WS00110_F15
11	48.979	218	2	0.195	0.038	4.120	1008	WS00110_F15
3	9.953	42	3	0.268	0.069	8.271	1009	WS00110_H21
3	15.147	44	2	-0.298	0.149	3.863	1009	WS00110_H21
7	15.555	130	3	-0.195	0.038	3.991	1009	WS00110_H21
8	50.001	154	1	0.215	0.036	4.449	1009	WS00110_H21
8	62.580	155	1	-0.332	0.097	4.083	1009	WS00110_H21
9	28.608	170	2	-0.227	0.060	4.615	1009	WS00110_H21
9	61.113	178	1	-0.362	0.120	5.017	1009	WS00110_H21
10	9.580	187	3	-0.293	0.061	4.068	1009	WS00110_H21
10	21.864	188	3	0.202	0.039	4.807	1009	WS00110_H21
11	37.143	209	3	0.239	0.060	7.196	1009	WS00110_H21
6	81.996	123	3	0.267	0.086	5.627	1010	WS0016_D07
6	87.891	124	2	-0.256	0.073	6.463	1010	WS0016_D07
8	87.463	158	2	-0.302	0.099	8.410	1010	WS0016_D07
11	43.491	214	3	-0.241	0.045	4.712	1010	WS0016_D07
11	64.014	222	4	-0.362	0.084	6.045	1010	WS0016_D07
14	0.000	246	2	-0.189	0.049	4.146	1010	WS0016_D07
4	75.958	77	3	-0.246	0.075	4.152	1011	WS0016_E10
6	25.914	114	1	-0.304	0.066	6.472	1011	WS0016_E10
9	55.358	175	3	0.443	0.064	5.124	1011	WS0016_E10
9	81.073	183	3	-0.243	0.039	5.404	1011	WS0016_E10
10	35.204	189	1	0.216	0.050	4.821	1011	WS0016_E10
5	31.191	95	1	-0.215	0.037	4.227	1012	WS0016_J12
6	87.891	124	2	0.212	0.055	4.048	1012	WS0016_J12
7	15.555	130	3	-0.357	0.079	10.338	1012	WS0016_J12
9	55.358	175	3	0.415	0.057	4.894	1012	WS0016_J12
10	35.204	189	1	0.212	0.037	4.151	1012	WS0016_J12
11	37.143	209	3	0.253	0.036	4.963	1012	WS0016_J12
6	25.914	114	1	-0.307	0.078	7.573	1013	WS0016_L02
7	15.555	130	3	-0.421	0.115	14.625	1013	WS0016_L02
11	0.000	199	2	-0.292	0.046	4.253	1013	WS0016_L02
11	35.604	207	3	-0.211	0.031	4.042	1013	WS0016_L02
11	37.143	209	3	0.227	0.037	4.746	1013	WS0016_L02
11	46.026	217	1	-0.212	0.040	3.868	1013	WS0016_L02
2	127.147	40	3	-0.240	0.052	6.629	1014	WS0017_B03

5	56.008	101	1	-0.202	0.036	3.894	1014	WS0017_B03
6	49.100	118	2	-0.303	0.082	4.425	1014	WS0017_B03
6	49.100	118	4	0.360	0.136	4.480	1014	WS0017_B03
6	56.188	120	1	-0.210	0.043	4.600	1014	WS0017_B03
8	78.389	157	1	-0.193	0.038	4.076	1014	WS0017_B03
8	87.463	158	2	-0.255	0.087	4.604	1014	WS0017_B03
10	9.580	187	3	-0.316	0.083	6.143	1014	WS0017_B03
12	14.406	230	3	-0.250	0.056	7.164	1014	WS0017_B03
14	44.898	251	3	0.242	0.036	4.559	1014	WS0017_B03
1	107.333	19	3	0.219	0.039	3.954	1015	WS0017_C21
3	47.928	49	1	0.215	0.054	5.505	1015	WS0017_C21
6	25.914	114	1	-0.198	0.046	4.613	1015	WS0017_C21
7	54.200	136	2	0.347	0.075	4.416	1015	WS0017_C21
7	73.169	142	1	0.513	0.074	4.442	1015	WS0017_C21
8	50.001	154	1	0.225	0.051	5.489	1015	WS0017_C21
1	133.992	24	2	0.219	0.038	4.469	1016	WS0017_F11
7	15.555	130	3	-0.334	0.115	7.153	1016	WS0017_F11
7	73.169	142	2	-0.520	0.090	7.342	1016	WS0017_F11
7	74.560	143	2	0.260	0.056	6.496	1016	WS0017_F11
10	78.479	193	4	-0.210	0.043	4.741	1016	WS0017_F11
11	0.000	199	2	-0.235	0.045	5.183	1016	WS0017_F11
4	124.132	83	1	-0.484	0.133	4.750	1017	WS0017_H07
6	25.914	114	1	-0.297	0.068	6.520	1017	WS0017_H07
6	95.249	126	1	-0.294	0.076	4.579	1017	WS0017_H07
10	86.151	195	3	-0.172	0.051	5.592	1017	WS0017_H07
1	2.788	2	3	-0.194	0.040	3.996	1018	WS00728_M24
4	0.000	71	4	0.244	0.055	4.826	1018	WS00728_M24
4	82.462	78	1	-0.218	0.045	4.329	1018	WS00728_M24
5	42.979	97	1	0.268	0.042	4.128	1018	WS00728_M24
6	81.996	123	3	0.306	0.095	5.398	1018	WS00728_M24
8	87.463	158	2	-0.210	0.046	4.028	1018	WS00728_M24
11	56.921	219	1	-0.336	0.101	4.065	1018	WS00728_M24
11	57.661	220	4	0.230	0.055	4.847	1018	WS00728_M24
11	64.014	222	4	-0.351	0.086	7.445	1018	WS00728_M24
7	15.555	130	2	0.277	0.047	5.005	1019	WS01041_O07
7	105.477	147	2	0.358	0.114	8.372	1019	WS01041_O07
11	40.960	210	2	-0.380	0.052	3.971	1019	WS01041_O07
11	44.884	215	3	0.250	0.040	3.906	1019	WS01041_O07
14	43.392	250	1	-0.263	0.092	5.860	1019	WS01041_O07
3	69.071	52	1	-0.246	0.076	4.096	1020	WS00729_M03
7	62.787	139	2	0.215	0.050	4.598	1020	WS00729_M03
7	67.154	140	4	-0.278	0.102	4.360	1020	WS00729_M03
7	73.169	142	1	0.557	0.117	4.978	1020	WS00729_M03
7	75.883	144	2	0.562	0.154	8.286	1020	WS00729_M03
7	75.883	144	4	-0.420	0.113	10.340	1020	WS00729_M03
8	39.090	152	4	-0.232	0.052	4.637	1020	WS00729_M03
8	50.001	154	2	-0.204	0.064	5.733	1020	WS00729_M03
11	45.989	216	1	-0.256	0.053	5.117	1020	WS00729_M03
12	51.382	242	1	-0.361	0.096	4.107	1020	WS00729_M03
2	120.617	38	3	-0.198	0.041	4.457	1021	WS00834_I21
3	95.157	59	2	0.208	0.041	3.900	1021	WS00834_I21
4	92.039	79	2	-0.289	0.058	6.421	1021	WS00834_I21
6	106.702	128	2	-0.533	0.145	7.595	1021	WS00834_I21
9	36.227	165	3	0.301	0.051	5.600	1021	WS00834_I21

9	0.000	166	1	0.196	0.072	5.691	1021	WS00834_I21
11	57.661	220	4	0.268	0.076	5.987	1021	WS00834_I21
1	40.464	7	4	0.318	0.076	8.031	1022	WS0094_E13
2	95.880	34	1	0.381	0.093	8.921	1022	WS0094_E13
2	102.814	36	1	0.315	0.062	5.818	1022	WS0094_E13
4	116.928	82	2	0.221	0.043	4.154	1022	WS0094_E13
5	0.000	89	1	0.253	0.064	6.104	1022	WS0094_E13
5	16.219	93	1	0.369	0.071	6.806	1022	WS0094_E13
6	25.742	113	3	0.300	0.091	7.334	1022	WS0094_E13
8	39.090	152	4	0.258	0.045	4.854	1022	WS0094_E13
11	27.405	204	1	0.229	0.042	3.975	1022	WS0094_E13
12	51.382	242	1	-0.250	0.102	4.223	1022	WS0094_E13
9	3.127	168	1	-0.256	0.073	4.273	1023	WS0094_J01
10	78.479	193	3	0.285	0.041	4.217	1023	WS0094_J01
11	60.464	221	2	-0.213	0.038	4.143	1023	WS0094_J01
1	95.572	17	3	0.208	0.060	5.498	1024	WS0017_P03
2	93.504	32	4	0.281	0.045	3.932	1024	WS0017_P03
4	142.917	85	1	-0.365	0.096	8.097	1024	WS0017_P03
7	54.200	136	2	0.287	0.098	4.584	1024	WS0017_P03
8	62.580	155	1	0.302	0.119	4.432	1024	WS0017_P03
9	69.046	181	2	0.293	0.099	4.595	1024	WS0017_P03
1	38.326	6	4	-0.287	0.101	6.750	1025	WS00940_J01
1	40.464	7	4	0.280	0.093	6.404	1025	WS00940_J01
1	43.578	8	2	0.221	0.029	4.234	1025	WS00940_J01
1	77.177	15	2	-0.156	0.036	5.022	1025	WS00940_J01
6	87.891	124	2	0.222	0.027	3.945	1025	WS00940_J01
8	50.001	154	2	-0.286	0.052	7.656	1025	WS00940_J01
1	43.578	8	2	0.320	0.074	8.617	1026	WS0018_N04
1	107.333	19	4	-0.212	0.043	3.996	1026	WS0018_N04
1	123.316	20	4	-0.423	0.118	8.268	1026	WS0018_N04
2	75.550	31	2	0.193	0.033	3.930	1026	WS0018_N04
3	14.500	43	1	-0.362	0.126	8.967	1026	WS0018_N04
3	17.158	45	1	0.203	0.063	4.453	1026	WS0018_N04
3	80.211	55	4	-0.264	0.067	6.177	1026	WS0018_N04
5	7.672	92	1	-0.251	0.066	5.194	1026	WS0018_N04
5	52.820	99	3	0.334	0.075	6.472	1026	WS0018_N04
6	69.073	121	3	0.276	0.045	4.362	1026	WS0018_N04
6	87.891	124	2	0.247	0.064	7.445	1026	WS0018_N04
6	89.401	125	2	0.379	0.096	11.547	1026	WS0018_N04
7	59.789	137	3	-0.232	0.063	6.075	1026	WS0018_N04
8	95.937	159	3	0.198	0.042	4.073	1026	WS0018_N04
9	0.000	166	2	-0.217	0.038	4.458	1026	WS0018_N04
9	55.358	175	3	-0.518	0.133	4.899	1026	WS0018_N04
10	86.151	195	3	-0.220	0.047	4.502	1026	WS0018_N04
11	46.026	217	1	-0.270	0.071	5.052	1026	WS0018_N04
14	9.923	247	1	0.273	0.068	4.835	1026	WS0018_N04
3	154.505	69	1	0.181	0.039	4.136	1027	WS00941_C23
6	25.914	114	1	-0.260	0.080	8.593	1027	WS00941_C23
6	45.897	117	1	0.366	0.068	7.412	1027	WS00941_C23
6	56.188	120	1	0.322	0.059	6.323	1027	WS00941_C23
11	56.921	219	1	-0.375	0.157	6.369	1027	WS00941_C23
11	60.464	221	1	0.260	0.044	4.747	1027	WS00941_C23
12	22.441	227	1	0.349	0.048	5.161	1027	WS00941_C23
14	0.000	246	1	0.183	0.046	4.866	1027	WS00941_C23

1	38.326	6	2	0.248	0.038	4.266	1028	WS00923_D09
1	123.316	20	4	-0.263	0.066	4.481	1028	WS00923_D09
6	81.996	123	4	0.406	0.189	4.686	1028	WS00923_D09
7	17.610	131	3	0.388	0.173	7.666	1028	WS00923_D09
7	73.169	142	2	-0.418	0.062	3.873	1028	WS00923_D09
9	17.951	169	2	0.273	0.055	5.599	1028	WS00923_D09
9	81.073	183	1	-0.266	0.051	3.846	1028	WS00923_D09
1	107.333	19	3	0.197	0.029	4.292	1029	WS00923_G14
5	56.008	101	1	-0.266	0.059	6.818	1029	WS00923_G14
6	25.914	114	1	-0.244	0.041	4.760	1029	WS00923_G14
7	15.555	130	3	-0.249	0.040	3.989	1029	WS00923_G14
9	36.227	165	3	0.194	0.030	4.347	1029	WS00923_G14
9	55.358	175	3	0.469	0.065	6.250	1029	WS00923_G14
4	6.032	72	4	0.221	0.053	4.185	1030	WS0021_P05
5	52.820	99	1	0.263	0.040	4.110	1030	WS0021_P05
7	74.560	143	1	-0.205	0.043	4.279	1030	WS0021_P05
8	50.001	154	2	-0.243	0.074	6.354	1030	WS0021_P05
8	62.580	155	1	-0.413	0.158	5.662	1030	WS0021_P05
8	78.389	157	1	-0.225	0.043	4.343	1030	WS0021_P05
9	2.011	167	4	0.235	0.054	4.303	1030	WS0021_P05
11	9.237	202	4	0.252	0.067	5.322	1030	WS0021_P05
11	27.405	204	4	-0.277	0.083	6.655	1030	WS0021_P05
11	46.026	217	1	-0.203	0.038	3.860	1030	WS0021_P05
14	44.898	251	3	0.213	0.050	5.470	1030	WS0021_P05
1	107.333	19	3	0.190	0.042	4.484	1031	WS0022_A07
4	0.000	71	4	0.276	0.100	8.037	1031	WS0022_A07
4	82.462	78	1	-0.237	0.053	5.617	1031	WS0022_A07
5	7.672	92	1	-0.234	0.029	4.101	1031	WS0022_A07
5	16.219	93	1	-0.256	0.047	5.019	1031	WS0022_A07
5	31.191	95	1	-0.174	0.037	3.958	1031	WS0022_A07
6	25.742	113	3	-0.193	0.041	4.398	1031	WS0022_A07
6	25.914	114	1	-0.184	0.036	3.843	1031	WS0022_A07
6	81.996	123	3	0.321	0.082	5.441	1031	WS0022_A07
11	43.491	214	3	-0.217	0.053	5.694	1031	WS0022_A07
11	44.884	215	3	0.226	0.042	4.485	1031	WS0022_A07
11	57.661	220	4	0.245	0.053	4.312	1031	WS0022_A07
11	64.014	222	4	-0.362	0.078	5.302	1031	WS0022_A07
14	44.898	251	3	0.216	0.039	4.197	1031	WS0022_A07
4	82.462	78	2	0.218	0.035	4.068	1032	WS0022_D18
7	15.555	130	3	-0.319	0.057	6.455	1032	WS0022_D18
7	75.883	144	2	0.401	0.090	3.984	1032	WS0022_D18
9	81.073	183	3	-0.282	0.057	6.258	1032	WS0022_D18
10	93.347	196	1	-0.510	0.123	6.290	1032	WS0022_D18
11	42.600	213	3	-0.240	0.033	3.965	1032	WS0022_D18
12	22.441	227	3	0.220	0.039	4.305	1032	WS0022_D18
14	0.000	246	2	-0.174	0.043	4.667	1032	WS0022_D18
7	75.883	144	2	0.384	0.061	4.633	1033	WS0022_E23
9	36.227	165	3	0.208	0.049	4.100	1033	WS0022_E23
9	28.608	170	2	-0.225	0.034	3.848	1033	WS0022_E23
9	49.013	173	2	0.194	0.037	4.034	1033	WS0022_E23
9	59.963	177	4	-0.292	0.046	4.814	1033	WS0022_E23
12	6.242	234	4	0.304	0.042	4.002	1033	WS0022_E23
1	2.788	2	4	0.183	0.058	5.228	1034	WS0022_G23
6	25.914	114	1	-0.192	0.039	3.990	1034	WS0022_G23

11	9.237	202	4	0.203	0.044	3.880	1034	WS0022_G23
13	3.386	245	1	0.270	0.046	4.774	1034	WS0022_G23
1	29.800	5	2	0.275	0.037	4.871	1035	WS0022_H19
1	38.326	6	2	-0.217	0.041	4.970	1035	WS0022_H19
4	82.462	78	2	0.212	0.044	5.121	1035	WS0022_H19
5	52.820	99	3	0.259	0.035	4.691	1035	WS0022_H19
6	25.914	114	1	-0.282	0.059	4.077	1035	WS0022_H19
10	78.479	193	3	0.339	0.058	7.810	1035	WS0022_H19
10	86.151	195	3	-0.261	0.047	6.529	1035	WS0022_H19
11	64.014	222	4	-0.249	0.098	4.987	1035	WS0022_H19
12	22.441	227	1	0.286	0.075	5.017	1035	WS0022_H19
1	107.333	19	3	0.271	0.059	6.145	1036	WS0022_K05
4	75.958	77	3	-0.392	0.120	6.928	1036	WS0022_K05
4	116.928	82	2	-0.241	0.033	3.888	1036	WS0022_K05
5	84.347	105	1	-0.212	0.060	4.181	1036	WS0022_K05
6	25.914	114	1	-0.281	0.082	5.735	1036	WS0022_K05
7	15.555	130	3	-0.234	0.047	5.011	1036	WS0022_K05
8	95.937	159	3	0.257	0.050	5.203	1036	WS0022_K05
11	46.026	217	1	-0.221	0.063	4.436	1036	WS0022_K05
2	93.504	32	4	0.293	0.052	6.820	1037	WS0022_L04
2	120.617	38	4	0.301	0.054	7.099	1037	WS0022_L04
3	137.891	64	1	0.230	0.041	5.492	1037	WS0022_L04
4	55.925	76	1	0.226	0.048	6.586	1037	WS0022_L04
5	86.669	106	4	-0.284	0.053	5.437	1037	WS0022_L04
6	81.996	123	4	-0.261	0.052	5.887	1037	WS0022_L04
9	55.358	175	1	-0.196	0.036	4.735	1037	WS0022_L04
11	29.069	205	1	0.284	0.042	5.658	1037	WS0022_L04
11	45.989	216	1	-0.200	0.041	5.395	1037	WS0022_L04
12	38.543	232	4	-0.304	0.067	6.762	1037	WS0022_L04
12	0.000	233	1	-0.284	0.057	7.736	1037	WS0022_L04
12	51.382	242	1	-0.320	0.075	7.593	1037	WS0022_L04
14	0.000	246	1	0.250	0.038	5.167	1037	WS0022_L04
14	44.898	251	1	0.211	0.034	4.546	1037	WS0022_L04
2	48.839	29	4	-0.263	0.056	5.260	1038	WS00821_D04
2	75.550	31	1	-0.244	0.050	4.093	1038	WS00821_D04
2	93.504	32	4	-0.329	0.052	4.976	1038	WS00821_D04
4	124.132	83	2	-0.285	0.147	4.516	1038	WS00821_D04
5	3.484	90	2	0.283	0.064	4.780	1038	WS00821_D04
6	81.996	123	3	-0.331	0.087	4.697	1038	WS00821_D04
9	36.227	165	3	0.224	0.066	5.627	1038	WS00821_D04
9	17.951	169	2	0.280	0.072	5.518	1038	WS00821_D04
9	28.608	170	2	-0.282	0.067	7.268	1038	WS00821_D04
10	78.479	193	4	-0.208	0.049	5.045	1038	WS00821_D04
11	40.960	210	2	-0.408	0.065	5.053	1038	WS00821_D04
11	45.989	216	1	0.230	0.049	4.028	1038	WS00821_D04
11	48.979	218	2	-0.262	0.053	5.819	1038	WS00821_D04
14	0.000	246	2	0.202	0.054	4.953	1038	WS00821_D04
4	92.039	79	4	-0.299	0.074	4.182	1039	WS00821_D16
4	103.719	81	4	-0.225	0.044	3.923	1039	WS00821_D16
4	116.928	82	4	0.396	0.097	7.625	1039	WS00821_D16
6	81.996	123	3	0.309	0.087	4.880	1039	WS00821_D16
7	54.200	136	2	0.345	0.139	7.368	1039	WS00821_D16
7	62.787	139	2	0.275	0.052	4.481	1039	WS00821_D16
9	2.011	167	4	-0.175	0.050	3.922	1039	WS00821_D16

6	25.914	114	1	-0.307	0.084	6.220	1040	WS00821_F15
7	70.278	141	3	-0.219	0.039	4.313	1040	WS00821_F15
10	9.580	187	3	-0.421	0.140	6.705	1040	WS00821_F15
10	80.088	194	1	-0.196	0.052	3.860	1040	WS00821_F15
1	40.464	7	3	0.180	0.034	4.092	1041	WS00821_G01
1	43.578	8	3	0.193	0.038	4.230	1041	WS00821_G01
1	45.794	9	3	-0.250	0.039	4.647	1041	WS00821_G01
1	82.301	16	3	-0.239	0.042	5.178	1041	WS00821_G01
2	127.147	40	4	-0.270	0.072	4.944	1041	WS00821_G01
4	116.928	82	2	-0.300	0.062	7.021	1041	WS00821_G01
6	25.914	114	1	-0.228	0.054	4.042	1041	WS00821_G01
6	69.073	121	3	-0.240	0.032	3.983	1041	WS00821_G01
10	70.246	190	2	0.270	0.048	5.527	1041	WS00821_G01
11	27.405	204	4	-0.279	0.074	3.982	1041	WS00821_G01
11	40.960	210	2	-0.397	0.095	4.455	1041	WS00821_G01
11	40.960	210	4	0.362	0.095	6.581	1041	WS00821_G01
1	2.788	2	4	-0.190	0.038	3.879	1042	WS00821_H16
4	0.000	71	4	0.222	0.047	4.390	1042	WS00821_H16
5	54.072	100	4	0.170	0.045	4.228	1042	WS00821_H16
8	87.463	158	2	-0.248	0.046	5.184	1042	WS00821_H16
8	95.937	159	3	0.185	0.052	4.887	1042	WS00821_H16
9	49.013	173	1	0.284	0.085	5.668	1042	WS00821_H16
9	55.358	175	1	-0.272	0.056	3.938	1042	WS00821_H16
9	69.046	181	1	-0.280	0.099	3.995	1042	WS00821_H16
9	81.073	183	1	0.251	0.058	4.206	1042	WS00821_H16
11	64.014	222	4	-0.313	0.069	6.532	1042	WS00821_H16
10	78.479	193	3	0.407	0.075	7.494	1043	WS00821_I10
11	17.088	203	1	-0.235	0.062	3.846	1043	WS00821_I10
11	27.405	204	1	0.295	0.066	4.121	1043	WS00821_I10
11	64.014	222	4	-0.318	0.065	5.952	1043	WS00821_I10
12	14.406	230	3	-0.238	0.040	3.964	1043	WS00821_I10
1	29.800	5	2	-0.274	0.037	4.022	1044	WS00821_K02
1	107.333	19	3	-0.238	0.045	4.672	1044	WS00821_K02
1	127.805	21	3	-0.269	0.056	5.770	1044	WS00821_K02
2	102.814	36	2	-0.234	0.036	4.094	1044	WS00821_K02
3	80.211	55	4	-0.179	0.052	4.403	1044	WS00821_K02
5	57.017	102	4	0.254	0.046	3.926	1044	WS00821_K02
5	84.347	105	1	0.177	0.048	3.845	1044	WS00821_K02
5	86.669	106	2	-0.378	0.125	6.730	1044	WS00821_K02
6	26.350	115	2	-0.255	0.055	5.878	1044	WS00821_K02
6	89.401	125	2	0.259	0.052	5.187	1044	WS00821_K02
6	106.702	128	2	-0.611	0.179	10.128	1044	WS00821_K02
9	3.127	168	1	0.385	0.152	4.615	1044	WS00821_K02
9	17.951	169	2	0.302	0.047	5.020	1044	WS00821_K02
9	55.358	175	3	-0.474	0.113	4.066	1044	WS00821_K02
12	22.441	227	3	0.283	0.058	4.155	1044	WS00821_K02
12	28.493	228	2	-0.338	0.089	4.826	1044	WS00821_K02
4	116.928	82	2	-0.176	0.037	4.606	1045	WS00821_K17
6	56.188	120	2	0.359	0.051	5.463	1045	WS00821_K17
6	87.891	124	2	0.304	0.067	8.527	1045	WS00821_K17
6	89.401	125	2	0.274	0.046	5.776	1045	WS00821_K17
8	95.937	159	3	0.217	0.061	6.944	1045	WS00821_K17
10	93.347	196	2	0.445	0.064	4.569	1045	WS00821_K17
11	57.661	220	4	0.271	0.070	5.239	1045	WS00821_K17

11	64.014	222	4	-0.285	0.074	5.414	1045	WS00821_K17
12	14.406	230	4	0.241	0.055	3.920	1045	WS00821_K17
2	93.504	32	4	-0.242	0.039	4.031	1046	WS01016_C17
3	14.500	43	1	-0.311	0.053	4.053	1046	WS01016_C17
3	17.158	45	1	0.204	0.050	3.879	1046	WS01016_C17
13	0.000	243	4	0.331	0.116	5.154	1046	WS01016_C17
1	127.805	21	4	0.228	0.043	4.695	1047	WS01016_H18
2	48.839	29	4	-0.345	0.105	9.024	1047	WS01016_H18
2	126.181	39	4	-0.328	0.075	4.076	1047	WS01016_H18
4	0.000	71	4	0.296	0.067	6.238	1047	WS01016_H18
4	82.462	78	1	0.241	0.049	4.433	1047	WS01016_H18
4	142.917	85	1	-0.353	0.067	6.298	1047	WS01016_H18
7	59.789	137	3	-0.252	0.052	5.010	1047	WS01016_H18
7	70.278	141	3	0.244	0.051	4.903	1047	WS01016_H18
9	3.127	168	1	0.377	0.086	3.946	1047	WS01016_H18
9	49.013	173	1	0.296	0.060	5.236	1047	WS01016_H18
9	55.358	175	1	-0.353	0.062	5.704	1047	WS01016_H18
9	81.073	183	1	0.345	0.098	8.752	1047	WS01016_H18
10	70.246	190	4	-0.211	0.038	5.257	1047	WS01016_H18
11	0.000	199	3	0.240	0.064	6.082	1047	WS01016_H18
11	40.960	210	2	0.380	0.086	3.949	1047	WS01016_H18
11	43.491	214	4	0.154	0.041	3.987	1047	WS01016_H18
11	48.979	218	4	0.186	0.056	5.717	1047	WS01016_H18
12	28.493	228	2	0.283	0.096	4.540	1047	WS01016_H18
13	3.386	245	1	-0.276	0.064	5.771	1047	WS01016_H18
5	57.017	102	4	-0.294	0.057	4.359	1048	WS0084_N14
6	81.996	123	3	0.353	0.122	6.271	1048	WS0084_N14
6	81.996	123	4	-0.358	0.118	5.814	1048	WS0084_N14
7	74.560	143	1	0.267	0.064	6.439	1048	WS0084_N14
8	87.463	158	2	-0.219	0.066	5.706	1048	WS0084_N14
11	64.014	222	4	-0.424	0.091	6.968	1048	WS0084_N14
13	0.000	243	4	0.395	0.104	5.132	1048	WS0084_N14
2	75.550	31	1	-0.332	0.090	7.873	1049	WS0022_P17
3	9.953	42	3	0.305	0.066	7.952	1049	WS0022_P17
3	137.891	64	3	0.299	0.059	6.037	1049	WS0022_P17
5	0.000	89	3	0.255	0.044	5.250	1049	WS0022_P17
5	3.484	90	2	-0.276	0.088	4.109	1049	WS0022_P17
5	16.219	93	2	0.279	0.064	5.298	1049	WS0022_P17
7	15.555	130	2	0.197	0.040	4.144	1049	WS0022_P17
8	62.580	155	1	-0.338	0.201	4.432	1049	WS0022_P17
14	43.392	250	1	-0.343	0.094	6.110	1049	WS0022_P17
14	44.898	251	3	0.228	0.036	4.350	1049	WS0022_P17
5	56.008	101	1	-0.282	0.053	5.549	1050	WS0085_D22
6	25.914	114	1	-0.254	0.055	5.752	1050	WS0085_D22
6	69.073	121	3	0.231	0.031	4.068	1050	WS0085_D22
8	50.001	154	1	0.235	0.049	5.431	1050	WS0085_D22
9	55.358	175	3	0.440	0.067	4.172	1050	WS0085_D22
10	78.479	193	3	-0.285	0.035	4.616	1050	WS0085_D22
10	93.347	196	1	-0.463	0.067	5.420	1050	WS0085_D22
11	35.604	207	3	-0.266	0.053	6.911	1050	WS0085_D22
11	37.143	209	3	0.275	0.057	7.415	1050	WS0085_D22
11	42.600	213	3	-0.256	0.044	5.815	1050	WS0085_D22
14	43.392	250	1	-0.295	0.060	4.848	1050	WS0085_D22
2	120.617	38	4	-0.242	0.046	4.073	1051	WS0023_G21

3	137.891	64	3	0.233	0.058	5.874	1051	WS0023_G21
4	6.032	72	4	0.268	0.057	5.061	1051	WS0023_G21
4	92.039	79	4	-0.388	0.077	7.013	1051	WS0023_G21
4	173.929	88	2	-0.235	0.045	4.950	1051	WS0023_G21
5	57.017	102	4	-0.254	0.056	4.972	1051	WS0023_G21
6	49.100	118	2	-0.227	0.077	4.224	1051	WS0023_G21
8	87.463	158	2	-0.167	0.038	4.253	1051	WS0023_G21
11	27.405	204	4	-0.276	0.053	4.269	1051	WS0023_G21
11	64.014	222	4	-0.372	0.069	5.107	1051	WS0023_G21
1	45.794	9	1	-0.269	0.052	3.950	1052	WS0105_M16
2	102.814	36	2	-0.144	0.027	4.308	1052	WS0105_M16
2	120.617	38	3	-0.147	0.066	4.840	1052	WS0105_M16
2	120.617	38	4	-0.332	0.056	6.435	1052	WS0105_M16
2	126.181	39	2	0.296	0.041	4.452	1052	WS0105_M16
2	127.147	40	4	-0.273	0.129	14.764	1052	WS0105_M16
4	82.462	78	1	-0.127	0.052	4.148	1052	WS0105_M16
4	103.719	81	4	-0.273	0.038	4.120	1052	WS0105_M16
6	87.891	124	2	0.159	0.047	5.330	1052	WS0105_M16
1	69.854	13	2	0.148	0.033	3.903	1053	WS0106_C17
1	77.177	15	2	0.204	0.059	6.881	1053	WS0106_C17
1	82.301	16	4	0.316	0.063	4.854	1053	WS0106_C17
4	0.000	71	4	0.183	0.059	4.638	1053	WS0106_C17
4	82.462	78	1	-0.225	0.080	5.681	1053	WS0106_C17
4	124.132	83	1	-0.453	0.115	4.109	1053	WS0106_C17
6	49.100	118	2	-0.437	0.120	9.035	1053	WS0106_C17
7	17.610	131	3	0.302	0.067	4.999	1053	WS0106_C17
8	87.463	158	2	-0.185	0.041	4.953	1053	WS0106_C17
9	81.073	183	1	0.358	0.090	6.494	1053	WS0106_C17
9	81.073	183	3	-0.231	0.044	4.980	1053	WS0106_C17
12	22.441	227	1	0.266	0.055	3.897	1053	WS0106_C17
1	62.128	11	1	-0.552	0.123	6.439	1054	WS00923_K03
2	126.181	39	4	-0.496	0.124	6.257	1054	WS00923_K03
3	14.500	43	1	-0.199	0.042	3.938	1054	WS00923_K03
4	124.132	83	1	-0.572	0.137	7.061	1054	WS00923_K03
5	0.000	89	1	-0.302	0.053	5.376	1054	WS00923_K03
6	56.188	120	2	0.356	0.060	3.957	1054	WS00923_K03
7	59.789	137	3	-0.225	0.054	4.171	1054	WS00923_K03
7	105.477	147	2	0.367	0.092	5.477	1054	WS00923_K03
9	55.358	175	1	-0.441	0.089	8.622	1054	WS00923_K03
9	81.073	183	1	0.201	0.051	4.716	1054	WS00923_K03
10	104.214	198	2	-0.250	0.039	3.910	1054	WS00923_K03
11	64.014	222	3	-0.204	0.060	4.618	1054	WS00923_K03
11	64.014	222	4	-0.171	0.036	3.956	1054	WS00923_K03
12	22.441	227	1	0.261	0.079	7.329	1054	WS00923_K03
12	6.242	234	2	-0.251	0.024	3.963	1054	WS00923_K03
12	6.242	234	4	0.337	0.052	4.746	1054	WS00923_K03
3	14.500	43	1	-0.241	0.059	4.269	1055	WS00923_N11
5	86.669	106	4	-0.408	0.217	6.867	1055	WS00923_N11
6	25.914	114	1	-0.246	0.058	4.114	1055	WS00923_N11
6	45.897	117	1	0.203	0.055	4.018	1055	WS00923_N11
6	87.891	124	2	0.257	0.053	6.236	1055	WS00923_N11
6	89.401	125	2	0.313	0.064	7.069	1055	WS00923_N11
3	14.500	43	1	-0.329	0.051	5.855	1056	WS00929_K01
3	17.158	45	1	0.213	0.049	5.574	1056	WS00929_K01

3	137.891	64	1	-0.245	0.048	5.529	1056	WS00929_K01
3	141.443	65	3	-0.289	0.048	3.942	1056	WS00929_K01
8	11.974	150	1	-0.191	0.040	4.537	1056	WS00929_K01
10	9.580	187	3	-0.362	0.132	10.628	1056	WS00929_K01
11	35.604	207	3	-0.209	0.035	3.964	1056	WS00929_K01
11	42.600	213	3	-0.224	0.038	4.240	1056	WS00929_K01
9	0.000	166	1	-0.202	0.052	4.636	1057	WS00929_K10
11	57.661	220	4	0.232	0.049	4.386	1057	WS00929_K10
11	64.014	222	4	-0.248	0.047	4.264	1057	WS00929_K10
14	46.637	252	3	0.247	0.061	5.955	1057	WS00929_K10
1	2.788	2	4	-0.209	0.054	5.438	1058	WS00929_L11
1	131.686	23	2	0.434	0.077	4.475	1058	WS00929_L11
3	14.500	43	1	-0.252	0.067	5.632	1058	WS00929_L11
3	17.158	45	1	0.339	0.087	7.451	1058	WS00929_L11
5	54.072	100	4	0.293	0.089	8.274	1058	WS00929_L11
5	57.017	102	4	-0.286	0.079	6.853	1058	WS00929_L11
8	87.463	158	2	-0.252	0.046	4.971	1058	WS00929_L11
8	95.937	159	3	0.298	0.092	8.717	1058	WS00929_L11
9	81.073	183	1	0.281	0.058	4.987	1058	WS00929_L11
10	78.479	193	3	0.422	0.077	7.328	1058	WS00929_L11
11	56.921	219	1	-0.380	0.144	4.859	1058	WS00929_L11
11	60.464	221	2	-0.247	0.041	4.081	1058	WS00929_L11
11	64.014	222	4	-0.297	0.068	6.281	1058	WS00929_L11
3	14.500	43	1	-0.197	0.055	5.417	1059	WS00929_M01
3	17.158	45	1	0.268	0.055	5.507	1059	WS00929_M01
4	0.000	71	4	0.216	0.066	5.954	1059	WS00929_M01
7	75.883	144	2	0.437	0.102	5.368	1059	WS00929_M01
8	87.463	158	2	-0.218	0.048	3.930	1059	WS00929_M01
9	3.127	168	1	-0.490	0.124	7.885	1059	WS00929_M01
11	17.088	203	1	-0.303	0.075	7.533	1059	WS00929_M01
11	29.069	205	1	0.303	0.051	5.215	1059	WS00929_M01
11	43.491	214	4	-0.253	0.092	6.842	1059	WS00929_M01
11	46.026	217	1	-0.201	0.039	3.909	1059	WS00929_M01
11	57.661	220	4	0.260	0.050	4.573	1059	WS00929_M01
11	64.014	222	4	-0.322	0.051	5.131	1059	WS00929_M01
2	102.814	36	2	-0.177	0.038	4.481	1060	WS00929_N07
4	55.925	76	1	0.210	0.051	3.856	1060	WS00929_N07
4	173.929	88	2	0.209	0.043	4.886	1060	WS00929_N07
5	86.669	106	2	0.292	0.096	5.380	1060	WS00929_N07
6	106.702	128	2	-0.415	0.076	4.459	1060	WS00929_N07
9	0.000	166	2	0.189	0.051	5.732	1060	WS00929_N07
10	9.580	187	2	0.261	0.076	4.397	1060	WS00929_N07
11	40.960	210	2	0.419	0.083	5.193	1060	WS00929_N07
11	40.960	210	4	-0.390	0.108	9.756	1060	WS00929_N07
11	44.884	215	4	0.293	0.052	4.238	1060	WS00929_N07
12	0.000	226	3	0.173	0.042	4.160	1060	WS00929_N07
12	0.000	233	1	-0.226	0.061	4.486	1060	WS00929_N07
14	0.000	246	1	0.235	0.055	4.078	1060	WS00929_N07
1	2.788	2	3	-0.167	0.040	4.262	1061	WS00929_N14
5	56.008	101	2	-0.224	0.037	4.967	1061	WS00929_N14
5	57.017	102	2	0.260	0.049	6.304	1061	WS00929_N14
7	75.883	144	2	0.438	0.084	5.221	1061	WS00929_N14
7	75.883	144	4	-0.324	0.056	4.637	1061	WS00929_N14
10	78.479	193	4	-0.265	0.056	4.436	1061	WS00929_N14

1	95.572	17	2	0.275	0.049	5.351	1062	WS00929_O16
2	48.839	29	1	0.227	0.062	4.874	1062	WS00929_O16
3	148.745	68	1	0.287	0.056	4.487	1062	WS00929_O16
4	82.462	78	2	0.251	0.070	7.558	1062	WS00929_O16
4	92.039	79	2	0.274	0.046	5.028	1062	WS00929_O16
5	84.347	105	2	-0.237	0.052	5.465	1062	WS00929_O16
6	49.100	118	4	-0.301	0.145	3.905	1062	WS00929_O16
9	17.951	169	1	0.237	0.055	4.365	1062	WS00929_O16
9	58.045	176	1	0.225	0.079	4.067	1062	WS00929_O16
10	9.580	187	3	-0.392	0.110	5.191	1062	WS00929_O16
11	17.088	203	1	-0.245	0.052	4.121	1062	WS00929_O16
13	0.000	243	4	-0.309	0.143	3.875	1062	WS00929_O16
14	46.637	252	3	0.191	0.042	3.912	1062	WS00929_O16
1	38.326	6	4	-0.329	0.049	4.844	1063	WS00929_O24
1	40.464	7	4	0.313	0.042	4.381	1063	WS00929_O24
4	0.000	71	4	0.139	0.042	4.284	1063	WS00929_O24
7	73.169	142	1	0.448	0.062	4.118	1063	WS00929_O24
9	3.127	168	1	-0.361	0.036	5.419	1063	WS00929_O24
11	46.026	217	1	-0.275	0.051	5.685	1063	WS00929_O24
11	57.661	220	4	0.281	0.049	5.107	1063	WS00929_O24
11	64.014	222	4	-0.400	0.074	6.683	1063	WS00929_O24
6	25.914	114	1	-0.249	0.059	5.858	1064	WS0082_G23
9	28.608	170	2	0.326	0.051	4.546	1064	WS0082_G23
10	86.151	195	3	-0.146	0.039	4.563	1064	WS0082_G23
11	35.604	207	3	-0.281	0.052	6.165	1064	WS0082_G23
11	37.143	209	3	0.326	0.066	7.834	1064	WS0082_G23
11	40.960	210	2	0.379	0.078	3.974	1064	WS0082_G23
11	42.600	213	3	-0.257	0.035	4.283	1064	WS0082_G23
14	43.392	250	1	-0.293	0.054	4.190	1064	WS0082_G23
4	0.000	71	4	0.294	0.163	8.016	1065	WS0082_H15
6	25.914	114	1	-0.271	0.051	5.668	1065	WS0082_H15
6	81.996	123	3	0.263	0.070	6.568	1065	WS0082_H15
7	15.555	130	3	-0.305	0.064	8.909	1065	WS0082_H15
9	0.000	166	1	-0.235	0.041	4.620	1065	WS0082_H15
9	28.608	170	3	0.252	0.043	6.074	1065	WS0082_H15
9	55.358	175	3	0.376	0.044	4.095	1065	WS0082_H15
10	35.204	189	1	0.199	0.041	4.434	1065	WS0082_H15
10	86.151	195	3	-0.201	0.035	4.788	1065	WS0082_H15
11	37.143	209	3	0.189	0.030	4.089	1065	WS0082_H15
11	41.984	212	2	-0.206	0.054	4.147	1065	WS0082_H15
11	43.491	214	3	-0.214	0.042	5.873	1065	WS0082_H15
11	45.989	216	2	-0.238	0.069	4.316	1065	WS0082_H15
2	93.504	32	4	-0.302	0.048	4.275	1066	WS0082_K06
4	0.000	71	4	0.235	0.044	4.276	1066	WS0082_K06
4	43.146	74	3	0.188	0.043	3.950	1066	WS0082_K06
5	56.008	101	2	-0.194	0.050	4.910	1066	WS0082_K06
5	57.017	102	2	0.274	0.049	4.754	1066	WS0082_K06
6	106.702	128	2	-0.458	0.105	5.340	1066	WS0082_K06
10	35.204	189	1	0.257	0.056	5.052	1066	WS0082_K06
10	70.246	190	2	-0.307	0.083	8.270	1066	WS0082_K06
11	35.604	207	3	-0.245	0.073	6.640	1066	WS0082_K06
11	37.143	209	3	0.258	0.081	7.392	1066	WS0082_K06
11	42.600	213	3	-0.211	0.051	4.645	1066	WS0082_K06
11	64.014	222	4	-0.285	0.062	5.928	1066	WS0082_K06

1	43.578	8	2	-0.217	0.046	4.205	1067	WS0082_K20
1	43.578	8	3	0.264	0.035	4.393	1067	WS0082_K20
4	75.958	77	3	-0.332	0.094	5.004	1067	WS0082_K20
5	56.008	101	1	-0.243	0.042	4.052	1067	WS0082_K20
5	84.347	105	1	-0.245	0.056	5.382	1067	WS0082_K20
6	25.914	114	1	-0.323	0.090	8.737	1067	WS0082_K20
7	54.200	136	2	0.316	0.073	3.935	1067	WS0082_K20
9	55.358	175	3	0.428	0.066	4.248	1067	WS0082_K20
10	35.204	189	1	0.262	0.054	5.171	1067	WS0082_K20
10	78.479	193	4	-0.186	0.069	4.534	1067	WS0082_K20
14	44.898	251	3	0.270	0.046	5.494	1067	WS0082_K20
3	69.071	52	1	-0.588	0.161	13.564	1068	WS0082_M24
3	80.211	55	4	-0.162	0.055	4.114	1068	WS0082_M24
4	0.000	71	4	0.170	0.089	6.608	1068	WS0082_M24
4	82.462	78	1	-0.198	0.058	6.653	1068	WS0082_M24
6	56.188	120	1	0.351	0.046	5.484	1068	WS0082_M24
6	69.073	121	3	0.155	0.037	4.205	1068	WS0082_M24
6	81.996	123	3	0.334	0.045	3.879	1068	WS0082_M24
6	89.401	125	1	0.214	0.047	5.411	1068	WS0082_M24
6	95.249	126	1	-0.512	0.118	9.968	1068	WS0082_M24
9	59.963	177	1	-0.381	0.064	7.628	1068	WS0082_M24
10	21.864	188	3	0.293	0.035	4.041	1068	WS0082_M24
10	35.204	189	1	0.424	0.070	8.304	1068	WS0082_M24
11	37.143	209	3	0.276	0.057	6.497	1068	WS0082_M24
11	56.921	219	1	-0.272	0.143	4.595	1068	WS0082_M24
13	3.386	245	1	0.139	0.034	3.880	1068	WS0082_M24
1	95.572	17	2	-0.332	0.077	10.012	1069	WS0082_N20
1	95.572	17	3	0.246	0.046	4.246	1069	WS0082_N20
1	123.316	20	4	0.282	0.071	4.908	1069	WS0082_N20
3	47.928	49	1	0.265	0.084	5.086	1069	WS0082_N20
3	95.157	59	2	-0.220	0.042	5.333	1069	WS0082_N20
4	6.032	72	4	0.234	0.058	5.574	1069	WS0082_N20
4	43.146	74	3	0.284	0.082	7.526	1069	WS0082_N20
4	75.958	77	3	-0.262	0.083	4.791	1069	WS0082_N20
7	105.477	147	2	0.311	0.086	5.963	1069	WS0082_N20
9	69.046	181	2	0.358	0.104	5.245	1069	WS0082_N20
11	37.143	209	3	0.216	0.047	4.297	1069	WS0082_N20
13	1.815	244	2	0.208	0.038	4.691	1069	WS0082_N20
1	95.572	17	3	0.309	0.078	6.756	1070	WS0082_P11
1	107.333	19	3	0.287	0.082	8.564	1070	WS0082_P11
3	15.147	44	2	-0.297	0.091	4.061	1070	WS0082_P11
3	17.158	45	1	-0.213	0.056	4.288	1070	WS0082_P11
4	0.000	71	4	0.305	0.089	7.445	1070	WS0082_P11
4	55.925	76	1	0.199	0.046	3.953	1070	WS0082_P11
7	75.883	144	2	0.413	0.082	4.446	1070	WS0082_P11
8	87.463	158	2	0.208	0.043	4.585	1070	WS0082_P11
9	59.963	177	4	-0.287	0.061	4.980	1070	WS0082_P11
10	78.479	193	4	-0.196	0.052	4.342	1070	WS0082_P11
10	95.795	197	4	0.247	0.069	5.746	1070	WS0082_P11
1	69.854	13	2	0.179	0.039	4.121	1071	WS0083_A08
4	173.929	88	2	-0.246	0.062	6.554	1071	WS0083_A08
11	43.491	214	3	-0.259	0.059	5.589	1071	WS0083_A08
11	57.661	220	4	0.224	0.041	3.870	1071	WS0083_A08
11	64.014	222	4	-0.341	0.057	4.806	1071	WS0083_A08

12	6.242	234	2	-0.218	0.036	4.151	1071	WS0083_A08
14	0.000	246	2	-0.217	0.055	5.775	1071	WS0083_A08
1	2.788	2	4	0.270	0.049	4.364	1072	WS00824_N01
1	38.326	6	4	0.359	0.058	4.414	1072	WS00824_N01
1	45.794	9	3	-0.258	0.043	4.688	1072	WS00824_N01
4	82.462	78	2	-0.227	0.055	4.750	1072	WS00824_N01
4	124.132	83	1	-0.479	0.132	4.604	1072	WS00824_N01
4	142.917	85	1	0.242	0.049	5.113	1072	WS00824_N01
6	81.996	123	3	-0.291	0.094	6.438	1072	WS00824_N01
9	69.046	181	2	-0.322	0.146	4.644	1072	WS00824_N01
11	45.989	216	1	0.272	0.062	6.449	1072	WS00824_N01
4	0.000	71	4	0.269	0.062	6.297	1073	WS00824_N19
4	82.462	78	1	-0.204	0.044	3.987	1073	WS00824_N19
8	87.463	158	2	-0.197	0.040	3.884	1073	WS00824_N19
11	57.661	220	4	0.288	0.071	7.197	1073	WS00824_N19
11	64.014	222	4	-0.390	0.089	8.752	1073	WS00824_N19
1	95.572	17	3	0.295	0.050	5.606	1074	WS00824_P11
4	0.000	71	4	0.174	0.062	4.215	1074	WS00824_P11
6	25.914	114	1	-0.317	0.109	9.306	1074	WS00824_P11
6	49.100	118	2	-0.328	0.073	4.798	1074	WS00824_P11
6	81.996	123	3	0.327	0.116	6.207	1074	WS00824_P11
7	54.200	136	2	0.270	0.064	4.172	1074	WS00824_P11
7	75.883	144	2	0.476	0.141	5.789	1074	WS00824_P11
9	81.073	183	3	-0.203	0.033	4.090	1074	WS00824_P11
10	9.580	187	3	-0.299	0.092	4.468	1074	WS00824_P11
11	46.026	217	1	-0.232	0.046	3.914	1074	WS00824_P11
1	62.128	11	1	-0.530	0.123	5.349	1075	WS01028_A03
2	126.181	39	4	-0.390	0.086	4.196	1075	WS01028_A03
4	124.132	83	1	-0.453	0.099	4.332	1075	WS01028_A03
6	25.914	114	1	-0.238	0.053	4.427	1075	WS01028_A03
7	15.555	130	2	0.258	0.042	4.430	1075	WS01028_A03
7	15.555	130	3	-0.318	0.066	6.582	1075	WS01028_A03
7	67.154	140	4	-0.438	0.106	4.769	1075	WS01028_A03
12	22.441	227	1	0.266	0.063	5.243	1075	WS01028_A03
12	6.242	234	2	-0.248	0.037	5.663	1075	WS01028_A03
3	69.071	52	1	-0.308	0.114	4.290	1076	WS01028_B15
4	6.032	72	4	0.179	0.057	4.382	1076	WS01028_B15
8	87.463	158	2	-0.191	0.030	3.890	1076	WS01028_B15
1	2.788	2	4	-0.286	0.068	8.040	1077	WS01028_C05
1	38.326	6	4	-0.236	0.057	5.770	1077	WS01028_C05
1	40.464	7	4	0.266	0.065	6.218	1077	WS01028_C05
7	15.555	130	2	0.246	0.036	4.482	1077	WS01028_C05
7	15.555	130	3	-0.347	0.080	7.125	1077	WS01028_C05
7	54.200	136	2	0.245	0.115	5.519	1077	WS01028_C05
9	36.227	165	3	0.321	0.078	6.668	1077	WS01028_C05
10	78.479	193	4	-0.201	0.038	4.383	1077	WS01028_C05
11	57.661	220	4	0.242	0.045	4.687	1077	WS01028_C05
11	64.014	222	4	-0.219	0.044	4.798	1077	WS01028_C05
13	1.815	244	4	0.192	0.042	4.896	1077	WS01028_C05
1	29.800	5	1	-0.324	0.043	4.207	1078	WS01028_E03
4	82.462	78	1	-0.269	0.071	6.822	1078	WS01028_E03
6	56.188	120	1	-0.221	0.049	4.682	1078	WS01028_E03
7	75.883	144	2	0.403	0.079	4.063	1078	WS01028_E03
11	27.405	204	1	0.253	0.052	4.992	1078	WS01028_E03

11	57.661	220	4	0.242	0.057	4.965	1078	WS01028_E03
11	64.014	222	4	-0.361	0.090	7.682	1078	WS01028_E03
3	137.891	64	3	-0.245	0.055	4.491	1079	WS01028_E13
3	154.505	69	4	-0.327	0.076	4.319	1079	WS01028_E13
6	69.073	121	3	-0.232	0.049	4.563	1079	WS01028_E13
6	81.996	123	3	-0.251	0.072	3.870	1079	WS01028_E13
6	87.891	124	2	-0.227	0.050	5.142	1079	WS01028_E13
9	69.046	181	2	-0.291	0.124	4.344	1079	WS01028_E13
10	80.088	194	1	0.277	0.054	4.567	1079	WS01028_E13
10	80.088	194	2	-0.197	0.036	3.857	1079	WS01028_E13
1	69.854	13	2	0.199	0.035	4.118	1080	WS0058_G14
1	71.592	14	2	0.220	0.044	5.192	1080	WS0058_G14
1	131.686	23	2	0.462	0.077	5.114	1080	WS0058_G14
3	17.158	45	1	0.277	0.080	5.526	1080	WS0058_G14
4	173.929	88	2	-0.184	0.040	4.741	1080	WS0058_G14
5	54.072	100	4	0.313	0.066	6.451	1080	WS0058_G14
5	57.017	102	4	-0.220	0.042	4.366	1080	WS0058_G14
7	105.477	147	2	-0.290	0.123	8.102	1080	WS0058_G14
11	57.661	220	4	0.281	0.089	8.569	1080	WS0058_G14
11	64.014	222	4	-0.349	0.076	7.262	1080	WS0058_G14
12	6.242	234	2	-0.172	0.027	4.046	1080	WS0058_G14
1	50.955	10	1	-0.214	0.037	3.916	1081	WS0058_O17
1	77.177	15	2	-0.216	0.062	5.064	1081	WS0058_O17
4	92.039	79	4	-0.382	0.082	7.531	1081	WS0058_O17
4	124.132	83	1	0.429	0.071	4.467	1081	WS0058_O17
9	49.013	173	1	0.225	0.042	5.048	1081	WS0058_O17
11	57.661	220	4	0.241	0.039	4.097	1081	WS0058_O17
11	64.014	222	4	-0.330	0.054	5.614	1081	WS0058_O17
13	0.000	243	4	0.346	0.068	4.287	1081	WS0058_O17
1	82.301	16	4	0.325	0.061	6.840	1082	WS00924_A14
2	95.880	34	1	-0.334	0.037	4.588	1082	WS00924_A14
5	86.669	106	4	-0.571	0.174	13.700	1082	WS00924_A14
7	67.154	140	4	-0.185	0.053	4.167	1082	WS00924_A14
7	75.883	144	4	-0.393	0.086	6.738	1082	WS00924_A14
9	3.127	168	1	-0.389	0.031	5.974	1082	WS00924_A14
11	9.237	202	4	0.184	0.038	4.237	1082	WS00924_A14
11	45.989	216	1	-0.248	0.035	4.368	1082	WS00924_A14
12	14.406	230	4	0.472	0.087	9.693	1082	WS00924_A14
12	38.543	232	4	-0.462	0.099	7.880	1082	WS00924_A14
12	6.242	234	2	-0.236	0.061	3.845	1082	WS00924_A14
12	51.382	242	1	-0.494	0.141	11.127	1082	WS00924_A14
3	116.958	63	4	-0.264	0.066	6.688	1083	WS00924_D09
4	173.929	88	2	-0.220	0.050	5.079	1083	WS00924_D09
5	54.072	100	4	0.305	0.086	8.758	1083	WS00924_D09
5	57.017	102	4	-0.322	0.076	7.850	1083	WS00924_D09
6	81.996	123	3	0.275	0.090	4.625	1083	WS00924_D09
7	73.169	142	1	0.509	0.120	4.154	1083	WS00924_D09
11	27.405	204	4	-0.344	0.074	5.699	1083	WS00924_D09
11	43.491	214	3	-0.240	0.057	4.926	1083	WS00924_D09
5	54.072	100	4	0.215	0.045	4.298	1084	WS00924_G02
11	27.405	204	4	-0.325	0.060	5.197	1084	WS00924_G02
11	64.014	222	4	-0.325	0.060	4.953	1084	WS00924_G02
13	1.815	244	4	0.237	0.043	4.087	1084	WS00924_G02
3	9.953	42	3	0.293	0.070	8.707	1085	WS00924_G11

3	114.747	62	2	-0.130	0.039	3.931	1085	WS00924_G11
4	0.000	71	4	0.192	0.076	4.864	1085	WS00924_G11
6	81.996	123	3	0.311	0.071	4.420	1085	WS00924_G11
11	37.143	209	3	0.240	0.038	4.717	1085	WS00924_G11
1	50.955	10	1	-0.205	0.054	4.488	1086	WS00924_H19
1	107.333	19	3	-0.231	0.059	5.666	1086	WS00924_H19
1	141.021	25	2	0.181	0.038	4.002	1086	WS00924_H19
4	6.032	72	4	0.226	0.047	4.357	1086	WS00924_H19
4	142.917	85	1	0.276	0.050	4.126	1086	WS00924_H19
6	76.376	122	1	-0.406	0.139	4.677	1086	WS00924_H19
7	75.883	144	2	-0.391	0.069	4.235	1086	WS00924_H19
9	3.127	168	1	0.432	0.177	5.821	1086	WS00924_H19
12	14.406	230	4	0.293	0.060	5.427	1086	WS00924_H19
12	51.382	242	1	0.336	0.133	4.416	1086	WS00924_H19
14	43.392	250	2	-0.279	0.067	6.573	1086	WS00924_H19
4	173.929	88	2	-0.264	0.070	7.969	1087	WS00924_I07
6	81.996	123	3	0.383	0.139	6.575	1087	WS00924_I07
9	81.073	183	1	0.253	0.060	4.506	1087	WS00924_I07
9	81.073	183	3	-0.241	0.055	5.482	1087	WS00924_I07
10	78.479	193	3	0.410	0.080	7.750	1087	WS00924_I07
11	64.014	222	4	-0.280	0.058	5.163	1087	WS00924_I07
12	6.242	234	2	-0.241	0.034	4.554	1087	WS00924_I07
1	133.992	24	2	-0.202	0.036	4.013	1088	WS00924_J09
4	124.132	83	1	-0.492	0.132	4.837	1088	WS00924_J09
4	142.917	85	1	0.231	0.053	4.064	1088	WS00924_J09
4	142.917	85	3	-0.245	0.064	5.418	1088	WS00924_J09
4	173.929	88	2	-0.293	0.088	9.723	1088	WS00924_J09
7	17.610	131	3	-0.329	0.106	4.553	1088	WS00924_J09
9	2.011	167	4	-0.270	0.062	6.469	1088	WS00924_J09
10	21.864	188	3	0.223	0.051	4.188	1088	WS00924_J09
3	47.928	49	1	0.395	0.145	11.893	1089	WS00924_J18
4	116.928	82	2	-0.306	0.059	6.607	1089	WS00924_J18
4	116.928	82	4	0.306	0.040	4.035	1089	WS00924_J18
5	54.072	100	4	0.393	0.106	10.853	1089	WS00924_J18
5	57.017	102	4	-0.355	0.080	6.838	1089	WS00924_J18
6	81.996	123	4	-0.337	0.120	5.277	1089	WS00924_J18
6	95.249	126	1	0.368	0.137	5.526	1089	WS00924_J18
11	17.088	203	1	-0.247	0.052	4.257	1089	WS00924_J18
11	48.979	218	4	-0.239	0.047	5.245	1089	WS00924_J18
14	22.402	248	3	0.245	0.067	5.773	1089	WS00924_J18
6	25.914	114	1	-0.260	0.048	4.610	1090	WS00924_L04
7	15.555	130	3	-0.246	0.034	3.999	1090	WS00924_L04
10	9.580	187	3	-0.469	0.185	13.142	1090	WS00924_L04
10	77.676	192	1	-0.291	0.055	5.315	1090	WS00924_L04
10	80.088	194	1	-0.303	0.061	5.863	1090	WS00924_L04
4	6.032	72	4	0.211	0.044	4.392	1091	WS00924_L15
4	173.929	88	2	-0.342	0.114	11.247	1091	WS00924_L15
6	81.996	123	3	0.324	0.090	4.019	1091	WS00924_L15
11	64.014	222	4	-0.336	0.055	5.253	1091	WS00924_L15
13	0.000	243	4	0.360	0.113	5.085	1091	WS00924_L15
14	0.000	246	2	-0.173	0.046	4.123	1091	WS00924_L15
1	50.955	10	1	-0.241	0.051	4.247	1092	WS00925_A13
2	48.839	29	4	-0.482	0.144	13.987	1092	WS00925_A13
2	93.504	32	2	-0.208	0.043	4.535	1092	WS00925_A13

2	102.814	36	2	-0.277	0.060	6.174	1092	WS00925_A13
2	126.181	39	2	0.302	0.055	5.843	1092	WS00925_A13
2	126.181	39	4	-0.381	0.072	4.588	1092	WS00925_A13
3	9.953	42	4	0.285	0.063	6.047	1092	WS00925_A13
5	52.820	99	3	-0.229	0.062	5.017	1092	WS00925_A13
9	17.951	169	1	-0.283	0.056	4.703	1092	WS00925_A13
11	0.000	199	3	0.254	0.047	4.336	1092	WS00925_A13
11	48.979	218	4	0.256	0.049	4.900	1092	WS00925_A13
11	57.661	220	4	-0.186	0.051	4.983	1092	WS00925_A13
11	64.014	222	4	0.289	0.057	4.422	1092	WS00925_A13
1	69.854	13	2	0.192	0.043	4.746	1093	WS00925_D15
1	71.592	14	2	0.184	0.040	4.442	1093	WS00925_D15
4	0.000	71	4	0.267	0.053	5.465	1093	WS00925_D15
4	173.929	88	2	-0.272	0.073	8.149	1093	WS00925_D15
10	70.246	190	2	-0.246	0.051	5.785	1093	WS00925_D15
11	64.014	222	4	-0.291	0.055	5.568	1093	WS00925_D15
2	75.550	31	1	0.311	0.040	4.231	1094	WS0031_C06
7	70.278	141	3	-0.269	0.056	6.319	1094	WS0031_C06
8	95.937	159	3	0.264	0.048	5.380	1094	WS0031_C06
10	9.580	187	3	-0.510	0.197	11.186	1094	WS0031_C06
10	80.088	194	1	-0.242	0.048	4.983	1094	WS0031_C06
13	0.000	243	4	0.400	0.089	4.192	1094	WS0031_C06
1	50.955	10	1	-0.218	0.072	5.281	1095	WS01019_A22
3	14.500	43	2	-0.265	0.044	5.024	1095	WS01019_A22
3	20.512	46	2	0.248	0.051	5.874	1095	WS01019_A22
5	86.669	106	2	-0.345	0.101	5.453	1095	WS01019_A22
9	36.227	165	3	0.319	0.103	9.409	1095	WS01019_A22
9	28.608	170	2	-0.271	0.045	5.134	1095	WS01019_A22
9	32.356	171	2	-0.327	0.095	10.879	1095	WS01019_A22
9	49.013	173	2	0.305	0.092	10.265	1095	WS01019_A22
10	9.580	187	3	-0.321	0.127	4.471	1095	WS01019_A22
10	78.479	193	4	-0.171	0.048	4.188	1095	WS01019_A22
1	22.419	4	3	0.377	0.165	5.386	1096	WS0031_P09
1	131.686	23	2	0.475	0.100	5.406	1096	WS0031_P09
1	133.992	24	2	0.187	0.031	3.948	1096	WS0031_P09
2	101.076	35	4	-0.223	0.058	4.278	1096	WS0031_P09
3	15.147	44	2	-0.231	0.075	4.227	1096	WS0031_P09
3	69.071	52	1	0.312	0.124	4.056	1096	WS0031_P09
8	11.974	150	1	0.246	0.079	4.605	1096	WS0031_P09
9	49.013	173	2	-0.205	0.033	3.859	1096	WS0031_P09
10	21.864	188	3	-0.251	0.040	4.454	1096	WS0031_P09
11	40.960	210	4	-0.229	0.054	4.163	1096	WS0031_P09
3	83.563	56	3	-0.211	0.052	5.197	1097	WS01019_I11
3	154.505	69	1	0.282	0.048	4.593	1097	WS01019_I11
4	0.000	71	4	0.266	0.069	6.027	1097	WS01019_I11
4	75.958	77	3	-0.289	0.089	3.893	1097	WS01019_I11
6	49.100	118	2	-0.300	0.105	4.600	1097	WS01019_I11
6	106.702	128	3	-0.311	0.064	5.556	1097	WS01019_I11
7	15.555	130	3	-0.234	0.041	4.152	1097	WS01019_I11
7	59.789	137	3	0.213	0.041	4.098	1097	WS01019_I11
7	70.278	141	3	-0.229	0.049	4.871	1097	WS01019_I11
11	46.026	217	1	-0.233	0.048	4.601	1097	WS01019_I11
3	83.563	56	3	-0.175	0.041	3.868	1098	WS00925_M10
4	0.000	71	4	0.249	0.065	6.166	1098	WS00925_M10

4	173.929	88	2	-0.202	0.047	4.472	1098	WS00925_M10
8	95.937	159	3	0.230	0.047	4.406	1098	WS00925_M10
11	46.026	217	1	-0.234	0.052	4.860	1098	WS00925_M10
11	57.661	220	4	0.239	0.048	4.540	1098	WS00925_M10
11	64.014	222	4	-0.318	0.059	5.408	1098	WS00925_M10
3	104.681	60	2	0.237	0.046	4.921	1099	WS00925_M18
3	112.868	61	2	-0.227	0.039	4.193	1099	WS00925_M18
3	114.747	62	2	-0.163	0.046	4.910	1099	WS00925_M18
3	137.891	64	1	-0.226	0.054	4.359	1099	WS00925_M18
3	154.505	69	1	0.282	0.084	6.913	1099	WS00925_M18
4	124.132	83	2	-0.274	0.099	4.100	1099	WS00925_M18
5	42.979	97	2	-0.284	0.045	4.667	1099	WS00925_M18
5	86.669	106	2	-0.310	0.071	3.928	1099	WS00925_M18
6	45.897	117	1	0.270	0.049	4.016	1099	WS00925_M18
6	56.188	120	1	0.269	0.053	4.324	1099	WS00925_M18
10	78.479	193	3	0.283	0.039	3.921	1099	WS00925_M18
10	86.151	195	3	-0.184	0.048	4.817	1099	WS00925_M18
1	62.128	11	1	-0.671	0.165	8.765	1100	WS00925_O03
3	141.443	65	4	0.416	0.094	5.165	1100	WS00925_O03
3	144.593	66	4	0.139	0.042	4.719	1100	WS00925_O03
3	148.745	68	4	-0.194	0.047	5.889	1100	WS00925_O03
4	43.146	74	3	0.271	0.053	4.192	1100	WS00925_O03
4	82.462	78	1	-0.150	0.059	5.485	1100	WS00925_O03
4	103.719	81	4	-0.281	0.043	5.239	1100	WS00925_O03
4	116.928	82	4	0.327	0.089	10.104	1100	WS00925_O03
6	106.702	128	2	-0.446	0.156	5.760	1100	WS00925_O03
7	15.555	130	3	-0.333	0.071	4.982	1100	WS00925_O03
9	81.073	183	3	-0.207	0.062	4.698	1100	WS00925_O03
11	64.014	222	4	-0.224	0.045	5.937	1100	WS00925_O03
12	14.406	230	4	0.158	0.027	4.336	1100	WS00925_O03
12	6.242	234	2	-0.263	0.050	6.841	1100	WS00925_O03
13	1.815	244	4	0.242	0.057	6.616	1100	WS00925_O03
14	22.402	248	4	-0.363	0.055	6.584	1100	WS00925_O03
1	62.128	11	1	-0.536	0.132	5.783	1101	WS00925_O11
3	9.953	42	4	0.248	0.044	4.258	1101	WS00925_O11
5	86.669	106	4	-0.278	0.104	4.993	1101	WS00925_O11
6	49.100	118	4	0.330	0.115	5.850	1101	WS00925_O11
6	87.891	124	2	0.225	0.043	5.266	1101	WS00925_O11
6	89.401	125	2	0.299	0.040	4.637	1101	WS00925_O11
10	70.246	190	2	0.173	0.042	5.039	1101	WS00925_O11
11	17.088	203	2	0.203	0.031	4.098	1101	WS00925_O11
13	1.815	244	4	0.260	0.062	4.426	1101	WS00925_O11
1	43.578	8	2	0.192	0.033	4.023	1102	WS00925_P15
3	137.891	64	3	-0.205	0.049	4.776	1102	WS00925_P15
4	116.928	82	4	0.290	0.073	6.336	1102	WS00925_P15
4	142.917	85	1	0.293	0.108	6.826	1102	WS00925_P15
7	17.610	131	3	0.257	0.081	4.932	1102	WS00925_P15
7	91.487	146	3	0.254	0.059	5.692	1102	WS00925_P15
9	3.127	168	1	0.419	0.133	4.135	1102	WS00925_P15
11	48.979	218	2	0.184	0.035	4.470	1102	WS00925_P15
12	22.441	227	3	0.380	0.140	12.196	1102	WS00925_P15
1	69.854	13	2	-0.217	0.042	4.960	1103	WS00925_P24
1	71.592	14	2	-0.222	0.045	5.308	1103	WS00925_P24
1	77.177	15	2	-0.261	0.063	7.394	1103	WS00925_P24

1	95.572	17	3	0.207	0.046	4.257	1103	WS00925_P24
1	107.333	19	3	0.144	0.045	4.263	1103	WS00925_P24
1	133.992	24	2	-0.216	0.033	3.926	1103	WS00925_P24
1	141.021	25	2	0.234	0.041	4.862	1103	WS00925_P24
4	0.000	71	4	0.190	0.075	7.018	1103	WS00925_P24
4	116.928	82	2	0.361	0.059	6.877	1103	WS00925_P24
4	124.132	83	2	0.374	0.093	4.389	1103	WS00925_P24
11	48.979	218	2	0.159	0.035	4.138	1103	WS00925_P24
12	6.242	234	2	0.210	0.051	7.012	1103	WS00925_P24
3	95.157	59	2	-0.229	0.037	4.787	1104	WS00926_B05
3	154.505	69	1	0.272	0.052	3.972	1104	WS00926_B05
4	173.929	88	2	-0.188	0.054	6.012	1104	WS00926_B05
6	25.742	113	3	-0.302	0.081	10.057	1104	WS00926_B05
1	40.464	7	3	0.217	0.036	4.482	1105	WS00926_B13
1	43.578	8	3	0.217	0.036	4.682	1105	WS00926_B13
3	95.157	59	2	-0.222	0.050	5.619	1105	WS00926_B13
4	0.000	71	4	0.296	0.120	7.412	1105	WS00926_B13
4	75.958	77	3	-0.253	0.085	5.121	1105	WS00926_B13
6	25.914	114	1	-0.256	0.055	4.718	1105	WS00926_B13
7	54.200	136	2	0.267	0.086	5.175	1105	WS00926_B13
12	14.406	230	4	0.320	0.119	6.785	1105	WS00926_B13
4	0.000	71	4	0.281	0.083	6.501	1106	WS0263_B07
4	173.929	88	2	-0.224	0.067	6.181	1106	WS0263_B07
6	81.996	123	3	0.320	0.100	6.955	1106	WS0263_B07
11	45.989	216	1	-0.188	0.042	3.985	1106	WS0263_B07
12	6.242	234	2	-0.248	0.038	4.280	1106	WS0263_B07
1	50.955	10	1	0.196	0.048	3.983	1107	WS0263_B15
9	36.227	165	4	0.272	0.046	4.788	1107	WS0263_B15
9	55.358	175	1	-0.252	0.052	4.283	1107	WS0263_B15
10	9.580	187	2	0.227	0.057	4.268	1107	WS0263_B15
11	40.960	210	2	0.554	0.109	8.576	1107	WS0263_B15
12	32.473	238	1	0.257	0.061	4.998	1107	WS0263_B15
14	9.923	247	1	-0.200	0.049	3.994	1107	WS0263_B15
14	46.637	252	4	-0.303	0.058	6.420	1107	WS0263_B15
4	173.929	88	2	-0.211	0.044	4.113	1108	WS0263_D15
5	57.017	102	4	-0.201	0.042	3.871	1108	WS0263_D15
6	81.996	123	3	0.378	0.109	6.660	1108	WS0263_D15
11	64.014	222	4	-0.305	0.062	5.652	1108	WS0263_D15
13	0.000	243	4	0.384	0.169	5.659	1108	WS0263_D15
1	62.128	11	1	-0.491	0.155	4.572	1109	WS0263_E04
4	0.000	71	4	0.240	0.053	4.861	1109	WS0263_E04
6	81.996	123	3	0.348	0.104	5.613	1109	WS0263_E04
9	81.073	183	1	0.240	0.049	4.164	1109	WS0263_E04
11	64.014	222	4	-0.284	0.058	5.228	1109	WS0263_E04
13	1.815	244	4	0.214	0.044	4.053	1109	WS0263_E04
14	43.392	250	1	-0.354	0.110	5.960	1109	WS0263_E04
1	69.854	13	2	0.186	0.040	4.278	1110	WS0263_F13
1	71.592	14	2	0.199	0.046	4.927	1110	WS0263_F13
2	93.504	32	4	-0.327	0.045	4.666	1110	WS0263_F13
7	15.555	130	2	0.254	0.049	5.439	1110	WS0263_F13
11	57.661	220	4	0.189	0.036	4.023	1110	WS0263_F13
11	64.014	222	4	-0.281	0.062	6.938	1110	WS0263_F13
12	6.242	234	4	0.418	0.083	5.026	1110	WS0263_F13
13	0.000	243	4	0.400	0.208	8.869	1110	WS0263_F13

4	75.958	77	3	-0.399	0.185	7.648	1111	WS0263_F24
4	124.132	83	1	0.442	0.063	4.342	1111	WS0263_F24
4	142.917	85	1	-0.286	0.057	6.703	1111	WS0263_F24
6	25.914	114	1	-0.174	0.035	4.035	1111	WS0263_F24
6	81.996	123	4	0.385	0.070	4.375	1111	WS0263_F24
7	15.555	130	3	-0.279	0.069	6.312	1111	WS0263_F24
9	17.951	169	1	-0.353	0.057	6.737	1111	WS0263_F24
9	55.358	175	1	0.284	0.036	4.225	1111	WS0263_F24
9	81.073	183	1	-0.201	0.034	4.018	1111	WS0263_F24
11	0.000	199	2	-0.299	0.076	5.304	1111	WS0263_F24
11	46.026	217	1	-0.262	0.039	4.629	1111	WS0263_F24
14	44.898	251	3	0.250	0.061	5.829	1111	WS0263_F24
3	14.500	43	1	-0.284	0.043	3.968	1112	WS0263_H18
4	0.000	71	4	0.211	0.066	7.070	1112	WS0263_H18
4	173.929	88	2	-0.292	0.061	6.053	1112	WS0263_H18
5	57.017	102	4	-0.285	0.049	5.241	1112	WS0263_H18
6	81.996	123	3	0.363	0.107	3.876	1112	WS0263_H18
6	81.996	123	4	-0.270	0.092	4.879	1112	WS0263_H18
8	87.463	158	2	-0.133	0.039	4.403	1112	WS0263_H18
9	59.963	177	4	0.292	0.051	5.510	1112	WS0263_H18
1	82.301	16	4	0.275	0.043	4.966	1113	WS0263_I09
4	0.000	71	4	0.288	0.077	8.980	1113	WS0263_I09
5	57.017	102	4	-0.215	0.035	4.111	1113	WS0263_I09
6	81.996	123	3	0.314	0.122	4.591	1113	WS0263_I09
7	73.169	142	1	0.492	0.072	4.112	1113	WS0263_I09
10	70.246	190	2	-0.252	0.057	5.435	1113	WS0263_I09
11	57.661	220	4	0.252	0.042	4.894	1113	WS0263_I09
11	64.014	222	4	-0.338	0.052	6.024	1113	WS0263_I09
1	2.788	2	4	-0.252	0.048	4.524	1114	WS0084_B19
4	0.000	71	4	0.273	0.081	7.999	1114	WS0084_B19
4	173.929	88	2	-0.213	0.043	4.191	1114	WS0084_B19
6	81.996	123	3	0.321	0.097	4.316	1114	WS0084_B19
11	64.014	222	4	-0.331	0.059	5.770	1114	WS0084_B19
1	82.301	16	4	0.287	0.061	5.676	1115	WS0084_I04
3	69.071	52	1	-0.330	0.082	4.710	1115	WS0084_I04
3	137.891	64	3	0.230	0.056	4.327	1115	WS0084_I04
4	0.000	71	4	0.211	0.058	5.395	1115	WS0084_I04
4	142.917	85	3	-0.267	0.048	4.042	1115	WS0084_I04
5	54.072	100	4	0.240	0.063	5.817	1115	WS0084_I04
5	57.017	102	4	-0.344	0.095	9.343	1115	WS0084_I04
6	81.996	123	3	0.302	0.067	3.847	1115	WS0084_I04
7	15.555	130	3	-0.309	0.054	4.468	1115	WS0084_I04
11	0.000	199	2	-0.229	0.047	4.480	1115	WS0084_I04
12	0.000	233	1	-0.168	0.045	4.330	1115	WS0084_I04
12	22.629	237	1	-0.161	0.041	3.912	1115	WS0084_I04
14	44.898	251	3	0.265	0.049	4.688	1115	WS0084_I04
1	123.316	20	4	-0.197	0.059	4.096	1116	WS01015_I21
10	35.204	189	2	0.167	0.032	3.936	1116	WS01015_I21
1	2.788	2	4	-0.265	0.082	7.166	1117	WS01015_N11
1	38.326	6	4	-0.277	0.066	5.980	1117	WS01015_N11
1	40.464	7	4	0.246	0.050	4.458	1117	WS01015_N11
6	87.891	124	2	0.232	0.045	4.236	1117	WS01015_N11
7	15.555	130	2	0.262	0.053	4.947	1117	WS01015_N11
7	74.560	143	1	-0.406	0.126	11.997	1117	WS01015_N11

7	91.487	146	3	-0.262	0.045	4.556	1117	WS01015_N11
10	9.580	187	3	-0.421	0.143	7.273	1117	WS01015_N11
10	35.204	189	1	-0.307	0.043	4.345	1117	WS01015_N11
14	0.000	246	1	-0.287	0.104	9.786	1117	WS01015_N11
2	48.839	29	4	-0.383	0.100	9.542	1118	WS0035_M19
2	93.504	32	4	0.328	0.058	4.460	1118	WS0035_M19
9	69.046	181	2	-0.420	0.136	6.765	1118	WS0035_M19
11	48.979	218	2	0.266	0.033	3.988	1118	WS0035_M19
12	6.242	234	2	0.220	0.028	4.076	1118	WS0035_M19
12	6.242	234	4	-0.416	0.083	7.553	1118	WS0035_M19
13	1.815	244	4	-0.209	0.049	4.545	1118	WS0035_M19
14	44.898	251	3	0.328	0.074	6.590	1118	WS0035_M19
1	82.301	16	3	-0.287	0.039	4.056	1119	IS0011_L13
2	93.504	32	2	-0.194	0.038	4.988	1119	IS0011_L13
5	86.669	106	2	0.330	0.117	7.719	1119	IS0011_L13
6	26.350	115	2	0.238	0.038	5.045	1119	IS0011_L13
7	17.610	131	3	0.224	0.073	4.755	1119	IS0011_L13
7	75.883	144	2	-0.403	0.067	4.669	1119	IS0011_L13
9	36.227	165	3	-0.294	0.076	7.791	1119	IS0011_L13
9	59.963	177	4	0.292	0.069	5.701	1119	IS0011_L13
11	48.979	218	2	0.170	0.033	4.355	1119	IS0011_L13
1	29.800	5	1	-0.308	0.071	8.590	1120	WS0043_I19
1	45.794	9	1	-0.255	0.035	4.299	1120	WS0043_I19
4	97.576	80	2	-0.271	0.080	4.691	1120	WS0043_I19
4	116.928	82	2	-0.319	0.067	4.575	1120	WS0043_I19
6	45.897	117	1	0.274	0.034	4.148	1120	WS0043_I19
7	70.278	141	3	-0.332	0.110	13.597	1120	WS0043_I19
8	87.463	158	2	-0.143	0.066	4.829	1120	WS0043_I19
9	59.963	177	1	-0.277	0.031	3.848	1120	WS0043_I19
10	9.580	187	3	-0.247	0.084	6.680	1120	WS0043_I19
11	35.604	207	3	-0.190	0.033	4.046	1120	WS0043_I19
11	37.143	209	3	0.193	0.034	4.158	1120	WS0043_I19
11	48.979	218	4	-0.199	0.057	4.034	1120	WS0043_I19
12	22.441	227	1	-0.214	0.034	4.162	1120	WS0043_I19
12	22.441	227	3	0.302	0.035	4.448	1120	WS0043_I19
14	43.392	250	1	-0.326	0.052	4.186	1120	WS0043_I19
1	77.177	15	2	-0.208	0.038	4.505	1121	WS00927_H17
1	107.333	19	3	0.201	0.049	4.837	1121	WS00927_H17
3	47.928	49	1	-0.237	0.058	3.999	1121	WS00927_H17
3	116.958	63	4	-0.234	0.043	3.882	1121	WS00927_H17
10	35.204	189	2	-0.342	0.085	9.415	1121	WS00927_H17
11	41.984	212	2	-0.181	0.040	4.745	1121	WS00927_H17
11	45.989	216	2	-0.220	0.055	6.147	1121	WS00927_H17
1	45.794	9	1	0.223	0.056	4.307	1122	WS0044_B15
1	45.794	9	3	-0.328	0.071	8.024	1122	WS0044_B15
1	127.805	21	4	-0.240	0.069	5.552	1122	WS0044_B15
4	0.000	71	4	0.271	0.089	6.836	1122	WS0044_B15
8	87.463	158	2	-0.212	0.052	5.279	1122	WS0044_B15
9	49.013	173	1	-0.254	0.056	4.064	1122	WS0044_B15
9	49.013	173	2	0.248	0.048	5.420	1122	WS0044_B15
13	1.815	244	2	0.268	0.054	6.113	1122	WS0044_B15
1	69.854	13	2	-0.201	0.043	4.146	1123	WS00927_K22
1	71.592	14	2	-0.231	0.060	5.698	1123	WS00927_K22
1	77.177	15	2	-0.221	0.054	5.138	1123	WS00927_K22

1	82.301	16	4	0.269	0.051	4.904	1123	WS00927_K22
1	107.333	19	3	0.172	0.045	4.156	1123	WS00927_K22
2	48.839	29	1	-0.254	0.048	4.412	1123	WS00927_K22
3	17.158	45	1	-0.283	0.066	6.146	1123	WS00927_K22
3	20.512	46	2	-0.243	0.047	4.575	1123	WS00927_K22
3	47.928	49	1	-0.315	0.084	7.735	1123	WS00927_K22
4	0.000	71	4	0.180	0.049	4.584	1123	WS00927_K22
4	82.462	78	1	0.244	0.056	5.190	1123	WS00927_K22
7	15.555	130	3	-0.303	0.063	5.398	1123	WS00927_K22
10	93.347	196	1	-0.418	0.075	4.200	1123	WS00927_K22
11	0.000	199	2	-0.286	0.043	4.161	1123	WS00927_K22
1	2.788	2	4	-0.182	0.084	5.605	1124	WS01024_F05
3	137.891	64	3	0.272	0.049	5.908	1124	WS01024_F05
4	0.000	71	4	0.303	0.079	5.129	1124	WS01024_F05
5	0.000	89	3	0.208	0.033	3.976	1124	WS01024_F05
6	69.073	121	3	0.244	0.034	4.215	1124	WS01024_F05
8	50.001	154	2	-0.214	0.037	4.550	1124	WS01024_F05
9	69.046	181	2	0.342	0.099	7.436	1124	WS01024_F05
9	81.073	183	1	0.190	0.060	4.196	1124	WS01024_F05
10	70.246	190	2	-0.322	0.079	9.507	1124	WS01024_F05
12	0.000	226	3	-0.248	0.054	6.793	1124	WS01024_F05
2	126.181	39	4	-0.439	0.097	5.578	1125	WS01024_F15
4	92.039	79	2	-0.181	0.044	4.253	1125	WS01024_F15
5	42.979	97	1	0.208	0.055	5.050	1125	WS01024_F15
5	52.820	99	1	0.234	0.070	6.551	1125	WS01024_F15
6	45.897	117	4	0.294	0.059	4.293	1125	WS01024_F15
11	9.237	202	4	-0.202	0.056	5.772	1125	WS01024_F15
12	6.242	234	4	0.315	0.040	4.867	1125	WS01024_F15
1	123.316	20	4	0.310	0.073	4.904	1126	WS01024_H11
2	95.880	34	1	0.351	0.064	5.451	1126	WS01024_H11
3	80.211	55	4	0.255	0.046	4.963	1126	WS01024_H11
4	97.576	80	2	0.233	0.040	5.273	1126	WS01024_H11
4	116.928	82	2	0.253	0.046	4.728	1126	WS01024_H11
5	7.672	92	1	0.236	0.039	3.904	1126	WS01024_H11
5	54.072	100	4	0.244	0.068	7.345	1126	WS01024_H11
6	69.073	121	3	0.303	0.060	4.830	1126	WS01024_H11
8	50.001	154	2	-0.224	0.044	4.517	1126	WS01024_H11
9	0.000	166	1	0.197	0.051	4.330	1126	WS01024_H11
1	22.419	4	4	0.329	0.069	4.247	1127	WS01024_H21
1	107.333	19	4	-0.232	0.044	5.179	1127	WS01024_H21
1	131.686	23	1	0.461	0.061	3.841	1127	WS01024_H21
3	20.512	46	2	0.226	0.055	4.616	1127	WS01024_H21
4	103.719	81	4	0.222	0.046	5.834	1127	WS01024_H21
5	7.672	92	4	-0.219	0.034	3.918	1127	WS01024_H21
8	78.389	157	1	0.239	0.045	4.744	1127	WS01024_H21
8	87.463	158	1	-0.242	0.048	5.022	1127	WS01024_H21
9	2.011	167	4	0.226	0.050	6.113	1127	WS01024_H21
9	3.127	168	1	0.299	0.065	4.195	1127	WS01024_H21
11	17.088	203	1	0.345	0.088	9.280	1127	WS01024_H21
11	27.405	204	1	-0.265	0.040	4.245	1127	WS01024_H21
11	29.069	205	1	-0.251	0.053	5.512	1127	WS01024_H21
11	45.989	216	1	0.192	0.044	4.522	1127	WS01024_H21
14	46.637	252	4	-0.309	0.055	6.791	1127	WS01024_H21
3	14.500	43	1	-0.278	0.053	4.285	1128	WS01024_J16

3	15.147	44	2	0.255	0.063	4.247	1128	WS01024_J16
3	17.158	45	1	0.265	0.075	5.975	1128	WS01024_J16
4	173.929	88	2	-0.244	0.059	6.333	1128	WS01024_J16
5	54.072	100	4	0.221	0.051	5.081	1128	WS01024_J16
6	69.073	121	3	0.217	0.047	4.114	1128	WS01024_J16
6	81.996	123	3	0.334	0.103	5.138	1128	WS01024_J16
9	81.073	183	1	0.266	0.055	4.463	1128	WS01024_J16
11	27.405	204	4	-0.310	0.059	5.033	1128	WS01024_J16
11	57.661	220	4	0.201	0.042	4.186	1128	WS01024_J16
11	64.014	222	4	-0.310	0.061	5.028	1128	WS01024_J16
12	6.242	234	2	-0.220	0.036	4.358	1128	WS01024_J16
3	15.147	44	2	0.376	0.077	6.499	1129	WS01024_K09
3	20.512	46	2	0.174	0.042	4.693	1129	WS01024_K09
8	78.389	157	1	0.350	0.099	7.676	1129	WS01024_K09
8	87.463	158	1	-0.302	0.074	5.717	1129	WS01024_K09
9	61.113	178	1	0.437	0.185	5.802	1129	WS01024_K09
10	70.246	190	2	-0.277	0.051	5.650	1129	WS01024_K09
5	54.072	100	4	0.340	0.077	8.646	1130	WS01024_L24
5	57.017	102	4	-0.276	0.063	7.308	1130	WS01024_L24
10	35.204	189	2	0.234	0.053	6.233	1130	WS01024_L24
10	70.246	190	2	-0.315	0.083	9.253	1130	WS01024_L24
11	27.405	204	1	0.258	0.055	4.742	1130	WS01024_L24
11	27.405	204	4	-0.350	0.049	5.567	1130	WS01024_L24
11	64.014	222	4	-0.297	0.061	6.347	1130	WS01024_L24
12	6.242	234	2	-0.178	0.026	3.897	1130	WS01024_L24
3	9.953	42	4	0.182	0.047	4.957	1131	WS01024_M08
4	173.929	88	2	-0.216	0.035	4.404	1131	WS01024_M08
5	54.072	100	4	0.258	0.065	5.860	1131	WS01024_M08
5	57.017	102	4	-0.223	0.054	5.424	1131	WS01024_M08
11	27.405	204	4	-0.370	0.081	7.686	1131	WS01024_M08
5	54.072	100	4	0.263	0.062	5.660	1132	WS01016_L11
5	57.017	102	4	-0.206	0.053	4.831	1132	WS01016_L11
8	95.937	159	3	0.279	0.068	6.672	1132	WS01016_L11
9	49.013	173	1	0.267	0.058	4.937	1132	WS01016_L11
9	58.045	176	1	0.268	0.079	4.519	1132	WS01016_L11
9	69.046	181	1	-0.336	0.086	4.948	1132	WS01016_L11
10	21.864	188	3	0.226	0.042	4.208	1132	WS01016_L11
11	57.661	220	4	0.164	0.047	4.278	1132	WS01016_L11
11	64.014	222	4	-0.358	0.102	9.350	1132	WS01016_L11
12	22.441	227	3	0.300	0.061	4.718	1132	WS01016_L11
12	6.242	234	2	-0.277	0.052	8.186	1132	WS01016_L11
12	32.473	238	1	-0.238	0.047	3.855	1132	WS01016_L11
14	0.000	246	1	-0.185	0.051	4.153	1132	WS01016_L11
1	82.301	16	4	0.279	0.053	4.511	1133	WS01016_M05
9	55.358	175	1	-0.360	0.060	5.842	1133	WS01016_M05
10	78.479	193	4	-0.214	0.063	4.160	1133	WS01016_M05
11	17.088	203	1	0.268	0.066	6.342	1133	WS01016_M05
1	50.955	10	1	-0.320	0.081	7.066	1134	WS01016_O14
2	48.839	29	4	-0.193	0.035	3.854	1134	WS01016_O14
4	75.958	77	3	-0.333	0.118	4.397	1134	WS01016_O14
5	57.017	102	4	-0.289	0.069	6.431	1134	WS01016_O14
6	69.073	121	3	0.230	0.053	4.760	1134	WS01016_O14
8	95.937	159	3	0.212	0.053	4.755	1134	WS01016_O14
2	93.504	32	4	-0.230	0.050	3.961	1135	WS01016_P09

3	14.500	43	2	-0.324	0.072	6.935	1135	WS01016_P09
3	20.512	46	2	0.218	0.068	7.097	1135	WS01016_P09
6	45.897	117	1	0.239	0.046	3.844	1135	WS01016_P09
7	91.487	146	3	-0.231	0.044	4.772	1135	WS01016_P09
9	0.000	166	1	0.254	0.060	4.953	1135	WS01016_P09
9	17.951	169	1	-0.315	0.076	6.240	1135	WS01016_P09
9	81.073	183	1	-0.283	0.054	4.433	1135	WS01016_P09
11	46.026	217	1	-0.242	0.055	4.523	1135	WS01016_P09
1	82.301	16	4	0.257	0.042	3.888	1136	WS01018_A22
3	154.505	69	1	0.193	0.059	4.628	1136	WS01018_A22
4	0.000	71	4	0.242	0.078	7.169	1136	WS01018_A22
5	57.017	102	4	-0.304	0.077	5.459	1136	WS01018_A22
6	49.100	118	2	-0.286	0.074	4.052	1136	WS01018_A22
8	87.463	158	2	-0.240	0.067	7.626	1136	WS01018_A22
1	50.955	10	1	-0.220	0.052	4.209	1137	WS01018_B08
2	48.839	29	1	-0.229	0.062	5.065	1137	WS01018_B08
5	56.008	101	1	-0.264	0.049	4.099	1137	WS01018_B08
5	84.347	105	1	-0.230	0.056	4.537	1137	WS01018_B08
7	54.200	136	2	0.260	0.106	4.485	1137	WS01018_B08
7	105.477	147	2	-0.459	0.156	10.030	1137	WS01018_B08
8	39.090	152	4	-0.166	0.044	4.142	1137	WS01018_B08
9	36.227	165	3	0.312	0.066	6.230	1137	WS01018_B08
9	59.963	177	4	0.332	0.069	5.497	1137	WS01018_B08
11	17.088	203	1	0.216	0.051	4.170	1137	WS01018_B08
11	29.069	205	1	-0.247	0.050	4.097	1137	WS01018_B08
11	56.921	219	1	-0.375	0.127	3.901	1137	WS01018_B08
12	22.441	227	1	0.233	0.053	4.214	1137	WS01018_B08
12	22.629	237	1	-0.299	0.093	7.544	1137	WS01018_B08
12	32.473	238	1	-0.293	0.086	7.050	1137	WS01018_B08
14	22.402	248	4	-0.308	0.044	4.163	1137	WS01018_B08
2	48.839	29	4	-0.229	0.059	5.263	1138	WS01018_D01
2	126.181	39	2	0.278	0.051	5.838	1138	WS01018_D01
4	75.958	77	3	0.414	0.151	8.027	1138	WS01018_D01
4	116.928	82	4	-0.354	0.063	5.874	1138	WS01018_D01
6	45.897	117	4	0.301	0.055	4.716	1138	WS01018_D01
10	35.204	189	1	-0.281	0.052	3.864	1138	WS01018_D01
14	22.402	248	4	-0.375	0.074	6.047	1138	WS01018_D01
3	14.500	43	1	-0.307	0.072	5.963	1139	WS01018_D18
3	17.158	45	1	0.252	0.058	4.769	1139	WS01018_D18
4	103.719	81	4	-0.271	0.051	6.189	1139	WS01018_D18
4	116.928	82	4	0.322	0.064	7.124	1139	WS01018_D18
6	87.891	124	2	0.237	0.050	5.236	1139	WS01018_D18
6	89.401	125	2	0.234	0.045	4.599	1139	WS01018_D18
7	105.477	147	2	-0.368	0.094	6.665	1139	WS01018_D18
9	2.011	167	4	-0.283	0.069	7.571	1139	WS01018_D18
9	59.963	177	4	0.296	0.047	5.211	1139	WS01018_D18
11	57.661	220	4	0.216	0.042	4.523	1139	WS01018_D18
11	64.014	222	4	-0.282	0.060	6.610	1139	WS01018_D18
12	38.543	232	4	0.425	0.148	5.723	1139	WS01018_D18
12	51.382	242	1	0.350	0.104	4.001	1139	WS01018_D18
1	45.794	9	1	-0.253	0.044	4.004	1140	WS0085_L08
1	82.301	16	4	0.261	0.058	5.612	1140	WS0085_L08
1	141.021	25	2	0.198	0.063	6.284	1140	WS0085_L08
3	137.891	64	3	-0.150	0.040	4.334	1140	WS0085_L08

7	105.477	147	2	-0.320	0.102	4.635	1140	WS0085_L08
9	49.013	173	1	0.281	0.057	4.592	1140	WS0085_L08
9	55.358	175	1	-0.289	0.047	4.167	1140	WS0085_L08
11	43.491	214	4	-0.287	0.065	5.521	1140	WS0085_L08
11	57.661	220	4	0.228	0.058	4.748	1140	WS0085_L08
11	64.014	222	4	-0.195	0.044	3.970	1140	WS0085_L08
12	22.441	227	1	0.209	0.046	4.133	1140	WS0085_L08
12	14.406	230	3	0.189	0.038	3.981	1140	WS0085_L08
4	0.000	71	4	0.217	0.048	4.330	1141	WS0086_C19
4	173.929	88	2	-0.283	0.073	7.746	1141	WS0086_C19
6	81.996	123	3	0.409	0.143	7.719	1141	WS0086_C19
9	59.963	177	4	0.258	0.054	4.837	1141	WS0086_C19
1	133.992	24	2	0.116	0.047	4.416	1142	IS0014_I14
2	120.617	38	4	-0.218	0.065	6.077	1142	IS0014_I14
2	127.147	40	4	-0.198	0.051	3.987	1142	IS0014_I14
3	95.157	59	2	-0.239	0.138	13.563	1142	IS0014_I14
3	104.681	60	2	0.428	0.127	12.482	1142	IS0014_I14
3	112.868	61	2	-0.352	0.135	12.799	1142	IS0014_I14
3	114.747	62	2	-0.370	0.154	14.573	1142	IS0014_I14
3	148.745	68	1	-0.271	0.041	3.906	1142	IS0014_I14
4	82.462	78	1	-0.218	0.049	4.572	1142	IS0014_I14
6	49.100	118	2	-0.199	0.091	4.635	1142	IS0014_I14
6	81.996	123	3	0.406	0.099	5.783	1142	IS0014_I14
7	15.555	130	2	0.262	0.044	4.676	1142	IS0014_I14
11	43.491	214	3	-0.194	0.041	4.367	1142	IS0014_I14
14	43.392	250	1	-0.398	0.102	5.966	1142	IS0014_I14
14	44.898	251	3	0.271	0.040	4.686	1142	IS0014_I14
3	9.953	42	3	0.217	0.054	6.489	1143	WS0041_A22
4	6.032	72	4	0.200	0.071	4.845	1143	WS0041_A22
5	0.000	89	3	0.351	0.059	4.488	1143	WS0041_A22
5	52.820	99	3	-0.215	0.041	4.514	1143	WS0041_A22
6	81.996	123	3	0.300	0.129	4.666	1143	WS0041_A22
7	17.610	131	3	0.218	0.049	4.067	1143	WS0041_A22
7	54.200	136	2	0.198	0.047	3.859	1143	WS0041_A22
9	3.127	168	1	-0.408	0.136	6.541	1143	WS0041_A22
3	137.891	64	3	0.244	0.053	4.533	1144	WS01018_F03
4	0.000	71	4	0.256	0.085	7.759	1144	WS01018_F03
4	173.929	88	2	-0.244	0.045	5.155	1144	WS01018_F03
5	54.072	100	4	0.232	0.060	5.541	1144	WS01018_F03
5	57.017	102	4	-0.286	0.061	5.368	1144	WS01018_F03
8	50.001	154	2	-0.172	0.045	4.446	1144	WS01018_F03
10	78.479	193	3	0.286	0.061	5.808	1144	WS01018_F03
3	15.147	44	2	-0.319	0.120	8.154	1145	WS01018_F17
7	15.555	130	2	0.308	0.054	5.805	1145	WS01018_F17
9	0.000	163	1	-0.492	0.093	5.372	1145	WS01018_F17
9	36.227	165	4	0.222	0.048	4.903	1145	WS01018_F17
10	35.204	189	1	-0.247	0.050	3.948	1145	WS01018_F17
3	69.071	52	1	0.251	0.076	4.570	1146	WS01018_H19
5	7.672	92	1	-0.255	0.049	4.725	1146	WS01018_H19
10	21.864	188	3	-0.381	0.104	9.155	1146	WS01018_H19
11	37.143	209	3	-0.188	0.053	4.967	1146	WS01018_H19
11	42.600	213	3	0.220	0.066	6.138	1146	WS01018_H19
1	2.788	2	4	-0.256	0.067	4.294	1147	WS01018_I24
3	14.500	43	1	-0.246	0.064	4.881	1147	WS01018_I24

3	17.158	45	1	0.248	0.059	4.406	1147	WS01018_I24
3	95.157	59	2	-0.199	0.039	4.459	1147	WS01018_I24
6	95.249	126	1	0.240	0.086	3.975	1147	WS01018_I24
7	17.610	131	3	0.214	0.071	4.674	1147	WS01018_I24
10	9.580	187	3	-0.292	0.108	5.215	1147	WS01018_I24
3	14.500	43	1	-0.394	0.091	10.424	1148	WS01018_K10
3	17.158	45	1	0.279	0.075	8.583	1148	WS01018_K10
3	137.891	64	1	-0.205	0.034	3.885	1148	WS01018_K10
4	140.962	84	3	-0.277	0.043	5.956	1148	WS01018_K10
6	45.897	117	1	0.321	0.051	5.813	1148	WS01018_K10
8	50.001	154	2	-0.172	0.064	4.013	1148	WS01018_K10
8	95.937	159	3	0.202	0.041	5.700	1148	WS01018_K10
14	43.392	250	1	-0.270	0.040	3.912	1148	WS01018_K10
4	142.917	85	1	0.305	0.059	5.318	1149	WS01018_K22
4	173.929	88	2	-0.220	0.046	4.612	1149	WS01018_K22
5	52.820	99	1	0.246	0.052	4.772	1149	WS01018_K22
5	52.820	99	3	-0.336	0.066	5.534	1149	WS01018_K22
5	54.072	100	4	0.224	0.047	4.925	1149	WS01018_K22
5	57.017	102	4	-0.233	0.046	5.158	1149	WS01018_K22
5	86.669	106	4	-0.319	0.102	4.859	1149	WS01018_K22
10	70.246	190	2	-0.170	0.038	3.881	1149	WS01018_K22
12	38.543	232	4	-0.306	0.104	4.794	1149	WS01018_K22
4	173.929	88	2	-0.212	0.043	4.219	1150	WS01018_M16
5	54.072	100	4	0.249	0.054	5.155	1150	WS01018_M16
5	57.017	102	4	-0.265	0.058	5.947	1150	WS01018_M16
8	87.463	158	2	-0.209	0.052	4.697	1150	WS01018_M16
10	70.246	190	2	-0.190	0.041	3.964	1150	WS01018_M16
12	6.242	234	2	-0.202	0.042	4.101	1150	WS01018_M16
12	6.242	234	4	0.242	0.047	4.810	1150	WS01018_M16
1	69.854	13	2	0.205	0.054	5.200	1151	WS01018_N12
1	71.592	14	2	0.201	0.052	5.030	1151	WS01018_N12
1	77.177	15	2	0.204	0.053	5.072	1151	WS01018_N12
7	15.555	130	2	0.225	0.050	5.057	1151	WS01018_N12
11	64.014	222	4	-0.277	0.096	6.936	1151	WS01018_N12
1	50.955	10	1	-0.232	0.041	4.054	1152	WS00936_A14
3	17.158	45	1	0.207	0.046	4.534	1152	WS00936_A14
3	154.505	69	1	0.301	0.058	5.696	1152	WS00936_A14
5	0.000	89	3	0.327	0.086	8.269	1152	WS00936_A14
7	15.555	130	2	0.273	0.074	6.978	1152	WS00936_A14
7	15.555	130	3	-0.255	0.051	5.032	1152	WS00936_A14
8	78.389	157	1	0.219	0.040	3.886	1152	WS00936_A14
10	78.479	193	4	-0.233	0.046	3.887	1152	WS00936_A14
1	131.686	23	1	-0.536	0.062	5.666	1153	WS00936_B19
2	120.617	38	4	-0.245	0.065	7.282	1153	WS00936_B19
2	127.147	40	4	-0.251	0.040	5.107	1153	WS00936_B19
4	124.132	83	1	0.546	0.082	7.398	1153	WS00936_B19
4	142.917	85	1	-0.218	0.049	6.237	1153	WS00936_B19
5	52.820	99	3	-0.325	0.058	4.602	1153	WS00936_B19
5	86.669	106	4	-0.390	0.049	4.579	1153	WS00936_B19
6	49.100	118	4	0.442	0.085	7.855	1153	WS00936_B19
6	54.066	119	4	0.157	0.041	4.590	1153	WS00936_B19
7	70.278	141	3	-0.299	0.085	6.541	1153	WS00936_B19
10	35.204	189	1	-0.248	0.032	4.151	1153	WS00936_B19
11	40.960	210	2	-0.429	0.201	5.260	1153	WS00936_B19

11	40.960	210	4	0.376	0.069	7.938	1153	WS00936_B19
14	0.000	246	1	-0.262	0.030	3.981	1153	WS00936_B19
14	46.637	252	4	0.340	0.043	4.836	1153	WS00936_B19
1	2.788	2	4	0.258	0.067	5.917	1154	WS00936_G08
1	50.955	10	1	-0.231	0.041	3.905	1154	WS00936_G08
1	69.854	13	2	-0.250	0.065	6.064	1154	WS00936_G08
1	71.592	14	2	-0.251	0.066	6.146	1154	WS00936_G08
1	95.572	17	2	-0.344	0.087	8.419	1154	WS00936_G08
1	95.572	17	3	0.302	0.063	5.843	1154	WS00936_G08
1	127.805	21	3	0.160	0.043	3.865	1154	WS00936_G08
2	48.839	29	4	-0.283	0.047	4.593	1154	WS00936_G08
2	93.504	32	2	-0.257	0.044	4.120	1154	WS00936_G08
2	93.504	32	4	0.229	0.045	4.510	1154	WS00936_G08
4	6.032	72	4	0.212	0.054	5.289	1154	WS00936_G08
4	82.462	78	1	0.260	0.043	4.097	1154	WS00936_G08
4	116.928	82	2	-0.260	0.048	4.498	1154	WS00936_G08
4	124.132	83	2	-0.277	0.112	3.979	1154	WS00936_G08
9	36.227	165	4	0.293	0.071	5.849	1154	WS00936_G08
9	17.951	169	1	-0.241	0.048	4.484	1154	WS00936_G08
11	0.000	199	2	-0.341	0.068	6.415	1154	WS00936_G08
1	131.686	23	2	0.506	0.101	6.154	1155	WS00936_I04
2	126.181	39	4	-0.484	0.106	6.485	1155	WS00936_I04
3	14.500	43	1	-0.243	0.059	4.715	1155	WS00936_I04
3	17.158	45	1	0.305	0.106	8.510	1155	WS00936_I04
5	57.017	102	4	-0.272	0.057	4.822	1155	WS00936_I04
6	25.914	114	1	-0.245	0.069	5.549	1155	WS00936_I04
6	45.897	117	1	0.233	0.049	3.988	1155	WS00936_I04
7	73.169	142	1	0.566	0.129	5.121	1155	WS00936_I04
8	39.090	152	4	-0.237	0.051	5.107	1155	WS00936_I04
9	36.227	165	4	0.269	0.043	3.991	1155	WS00936_I04
10	78.479	193	4	-0.234	0.041	4.115	1155	WS00936_I04
1	45.794	9	1	0.375	0.073	6.129	1156	WS00936_M09
1	50.955	10	1	-0.332	0.099	8.269	1156	WS00936_M09
6	81.996	123	4	0.371	0.116	4.112	1156	WS00936_M09
7	67.154	140	4	-0.336	0.130	4.789	1156	WS00936_M09
7	75.883	144	2	0.422	0.079	4.777	1156	WS00936_M09
7	75.883	144	4	-0.374	0.090	8.597	1156	WS00936_M09
8	95.937	159	3	0.190	0.047	4.432	1156	WS00936_M09
9	55.358	175	3	-0.598	0.172	5.928	1156	WS00936_M09
10	9.580	187	2	-0.328	0.070	4.052	1156	WS00936_M09
11	17.088	203	1	0.238	0.050	4.155	1156	WS00936_M09
11	27.405	204	1	-0.223	0.051	4.417	1156	WS00936_M09
1	2.788	2	4	-0.371	0.089	10.141	1157	WS00936_O06
1	38.326	6	4	-0.350	0.068	6.968	1157	WS00936_O06
1	40.464	7	4	0.296	0.043	4.794	1157	WS00936_O06
3	14.500	43	1	-0.351	0.070	6.995	1157	WS00936_O06
3	17.158	45	1	0.223	0.063	6.266	1157	WS00936_O06
3	137.891	64	1	-0.265	0.041	4.213	1157	WS00936_O06
3	154.505	69	1	0.180	0.041	4.103	1157	WS00936_O06
4	0.000	71	4	0.151	0.037	3.974	1157	WS00936_O06
6	45.897	117	1	0.305	0.054	5.490	1157	WS00936_O06
7	59.789	137	3	-0.236	0.061	4.838	1157	WS00936_O06
7	105.477	147	2	-0.323	0.064	3.880	1157	WS00936_O06
10	70.246	190	4	-0.358	0.065	6.969	1157	WS00936_O06

10	77.676	192	1	0.243	0.054	5.382	1157	WS00936_O06
10	78.479	193	4	-0.484	0.166	17.687	1157	WS00936_O06
10	80.088	194	2	-0.211	0.049	3.977	1157	WS00936_O06
10	95.795	197	4	0.251	0.045	4.958	1157	WS00936_O06
1	69.854	13	2	0.260	0.045	4.742	1158	WS00937_C12
1	71.592	14	2	0.281	0.044	4.703	1158	WS00937_C12
2	75.550	31	2	-0.176	0.046	5.791	1158	WS00937_C12
3	15.147	44	2	0.191	0.068	4.803	1158	WS00937_C12
3	20.512	46	2	0.213	0.048	4.868	1158	WS00937_C12
3	80.211	55	4	0.227	0.041	4.412	1158	WS00937_C12
3	112.868	61	2	-0.214	0.052	5.283	1158	WS00937_C12
3	114.747	62	2	-0.242	0.058	5.921	1158	WS00937_C12
3	141.443	65	3	0.362	0.105	5.059	1158	WS00937_C12
4	142.917	85	1	0.201	0.055	4.682	1158	WS00937_C12
5	54.072	100	4	0.215	0.049	5.254	1158	WS00937_C12
6	45.897	117	1	-0.290	0.052	4.672	1158	WS00937_C12
8	0.000	148	4	-0.209	0.038	4.156	1158	WS00937_C12
8	50.001	154	2	-0.211	0.030	4.284	1158	WS00937_C12
9	81.073	183	1	0.210	0.066	5.656	1158	WS00937_C12
11	0.000	199	2	0.286	0.044	4.709	1158	WS00937_C12
11	64.014	222	4	-0.174	0.047	5.917	1158	WS00937_C12
1	2.788	2	4	-0.234	0.070	5.589	1159	WS00937_D17
3	154.505	69	4	-0.238	0.064	4.780	1159	WS00937_D17
7	74.560	143	1	0.250	0.046	3.900	1159	WS00937_D17
9	28.608	170	3	0.301	0.049	4.135	1159	WS00937_D17
12	22.629	237	1	-0.241	0.051	4.266	1159	WS00937_D17
4	0.000	71	4	0.262	0.070	6.362	1160	WS00826_M02
4	173.929	88	2	-0.229	0.049	5.579	1160	WS00826_M02
5	54.072	100	4	0.176	0.043	3.862	1160	WS00826_M02
5	57.017	102	4	-0.253	0.062	4.712	1160	WS00826_M02
6	81.996	123	3	0.354	0.125	5.844	1160	WS00826_M02
8	87.463	158	2	-0.197	0.036	4.062	1160	WS00826_M02
9	59.963	177	4	0.309	0.072	6.221	1160	WS00826_M02
12	6.242	234	2	-0.249	0.036	4.741	1160	WS00826_M02
3	112.868	61	2	-0.213	0.046	4.552	1161	WS00826_N10
3	114.747	62	2	-0.196	0.045	4.450	1161	WS00826_N10
4	55.925	76	1	-0.214	0.051	4.259	1161	WS00826_N10
9	28.608	170	2	0.212	0.042	4.312	1161	WS00826_N10
12	22.441	227	3	0.240	0.046	4.527	1161	WS00826_N10
13	0.000	243	4	0.255	0.101	4.190	1161	WS00826_N10
14	43.392	250	1	-0.366	0.097	4.589	1161	WS00826_N10
14	43.392	250	2	-0.182	0.040	4.064	1161	WS00826_N10
14	44.898	251	3	0.227	0.044	4.287	1161	WS00826_N10
14	46.637	252	4	-0.328	0.047	4.794	1161	WS00826_N10
3	69.071	52	1	-0.439	0.137	8.807	1162	WS00720_D08
4	82.462	78	1	-0.201	0.059	5.180	1162	WS00720_D08
4	140.962	84	3	-0.291	0.051	5.928	1162	WS00720_D08
4	173.929	88	2	-0.252	0.052	5.253	1162	WS00720_D08
5	54.072	100	4	0.235	0.067	5.055	1162	WS00720_D08
5	57.017	102	4	-0.331	0.116	8.771	1162	WS00720_D08
5	84.347	105	1	-0.273	0.055	4.939	1162	WS00720_D08
6	69.073	121	3	0.189	0.038	4.257	1162	WS00720_D08
7	62.787	139	4	0.313	0.087	5.252	1162	WS00720_D08
7	74.560	143	1	0.193	0.059	5.200	1162	WS00720_D08

10	35.204	189	1	0.322	0.063	5.584	1162	WS00720_D08
10	35.204	189	2	0.168	0.039	3.912	1162	WS00720_D08
11	27.405	204	4	-0.316	0.073	4.704	1162	WS00720_D08
11	64.014	222	4	-0.373	0.095	6.814	1162	WS00720_D08
12	22.441	227	3	0.321	0.045	4.744	1162	WS00720_D08
3	137.891	64	3	0.323	0.074	8.450	1163	WS00720_E07
4	173.929	88	2	-0.236	0.047	4.888	1163	WS00720_E07
6	25.914	114	1	-0.181	0.047	3.941	1163	WS00720_E07
6	81.996	123	3	0.414	0.108	6.954	1163	WS00720_E07
3	15.147	44	2	0.301	0.076	4.198	1164	WS00720_I21
4	0.000	71	4	0.211	0.051	4.571	1164	WS00720_I21
4	173.929	88	2	-0.280	0.065	7.530	1164	WS00720_I21
5	54.072	100	4	0.198	0.048	4.305	1164	WS00720_I21
5	57.017	102	4	-0.250	0.054	4.875	1164	WS00720_I21
6	69.073	121	3	0.208	0.039	3.850	1164	WS00720_I21
6	81.996	123	3	0.343	0.116	4.845	1164	WS00720_I21
8	87.463	158	2	-0.189	0.040	4.632	1164	WS00720_I21
10	70.246	190	2	-0.261	0.052	5.932	1164	WS00720_I21
11	27.405	204	4	-0.241	0.045	3.925	1164	WS00720_I21
1	69.854	13	2	0.204	0.044	4.035	1165	WS00720_J13
1	71.592	14	2	0.218	0.050	4.605	1165	WS00720_J13
1	95.572	17	2	0.381	0.097	9.152	1165	WS00720_J13
4	82.462	78	1	-0.240	0.081	7.716	1165	WS00720_J13
4	142.917	85	1	0.270	0.065	6.297	1165	WS00720_J13
4	173.929	88	2	-0.239	0.081	7.529	1165	WS00720_J13
5	54.072	100	4	0.219	0.057	5.744	1165	WS00720_J13
11	64.014	222	4	-0.364	0.115	11.665	1165	WS00720_J13
12	28.493	228	2	-0.226	0.068	4.021	1165	WS00720_J13
14	0.000	246	1	-0.206	0.049	4.689	1165	WS00720_J13
14	43.392	250	2	-0.219	0.046	4.304	1165	WS00720_J13
1	95.572	17	3	0.227	0.035	4.050	1166	WS00720_O13
4	103.719	81	4	-0.242	0.047	3.977	1166	WS00720_O13
6	25.914	114	1	-0.241	0.045	4.386	1166	WS00720_O13
9	0.000	166	1	-0.276	0.060	5.935	1166	WS00720_O13
9	28.608	170	3	0.231	0.037	4.240	1166	WS00720_O13
9	55.358	175	3	0.447	0.082	4.891	1166	WS00720_O13
10	9.580	187	3	-0.327	0.104	6.205	1166	WS00720_O13
10	78.479	193	4	-0.237	0.056	4.728	1166	WS00720_O13
10	80.088	194	1	-0.261	0.042	4.093	1166	WS00720_O13
11	57.661	220	4	0.246	0.053	4.103	1166	WS00720_O13
13	0.000	243	4	0.412	0.191	7.130	1166	WS00720_O13
3	95.157	59	2	-0.163	0.038	4.310	1167	WS00720_P15
3	104.681	60	2	0.224	0.044	4.640	1167	WS00720_P15
3	114.747	62	2	-0.166	0.048	5.107	1167	WS00720_P15
3	148.745	68	1	-0.254	0.051	4.173	1167	WS00720_P15
3	154.505	69	1	-0.314	0.074	6.015	1167	WS00720_P15
4	6.032	72	4	0.230	0.075	5.986	1167	WS00720_P15
5	31.191	95	1	0.227	0.048	3.965	1167	WS00720_P15
6	26.350	115	2	0.213	0.054	5.753	1167	WS00720_P15
8	95.937	159	3	0.169	0.039	4.168	1167	WS00720_P15
11	43.491	214	3	-0.199	0.053	5.698	1167	WS00720_P15
11	43.491	214	4	0.208	0.067	5.285	1167	WS00720_P15
11	44.884	215	3	0.231	0.038	4.133	1167	WS00720_P15
11	44.884	215	4	-0.295	0.069	5.479	1167	WS00720_P15

4	75.958	77	3	-0.280	0.099	4.623	1168	WS0041_I13
6	25.914	114	1	-0.319	0.091	8.985	1168	WS0041_I13
6	45.897	117	1	0.266	0.071	6.909	1168	WS0041_I13
6	49.100	118	4	0.393	0.096	4.323	1168	WS0041_I13
6	54.066	119	4	0.213	0.048	4.424	1168	WS0041_I13
6	69.073	121	4	-0.258	0.047	4.246	1168	WS0041_I13
7	54.200	136	2	0.273	0.082	3.850	1168	WS0041_I13
7	73.169	142	2	-0.458	0.119	4.248	1168	WS0041_I13
7	75.883	144	2	0.405	0.101	4.319	1168	WS0041_I13
9	28.608	170	3	0.248	0.043	3.940	1168	WS0041_I13
9	55.358	175	3	0.430	0.093	4.636	1168	WS0041_I13
11	57.661	220	4	0.289	0.061	5.665	1168	WS0041_I13
1	62.128	11	2	0.292	0.062	3.906	1169	WS0042_B01
1	95.572	17	3	0.307	0.067	3.908	1169	WS0042_B01
2	63.261	30	3	-0.202	0.041	4.039	1169	WS0042_B01
3	14.500	43	2	0.229	0.045	5.210	1169	WS0042_B01
5	16.219	93	2	0.253	0.039	4.518	1169	WS0042_B01
7	17.610	131	3	0.424	0.144	9.299	1169	WS0042_B01
8	78.389	157	1	-0.184	0.062	4.451	1169	WS0042_B01
9	81.073	183	1	-0.303	0.096	7.094	1169	WS0042_B01
11	46.026	217	1	-0.270	0.059	4.379	1169	WS0042_B01
12	28.493	228	2	0.280	0.111	5.626	1169	WS0042_B01
12	6.242	234	4	0.219	0.052	5.289	1169	WS0042_B01
1	71.592	14	2	0.148	0.045	3.975	1170	WS00816_A04
3	17.158	45	1	0.184	0.045	4.492	1170	WS00816_A04
5	0.000	89	3	0.240	0.060	5.661	1170	WS00816_A04
5	57.017	102	4	-0.311	0.054	4.454	1170	WS00816_A04
6	81.996	123	4	-0.235	0.076	3.954	1170	WS00816_A04
2	48.839	29	4	-0.234	0.052	5.482	1171	WS00816_G10
3	15.147	44	2	0.319	0.098	7.109	1171	WS00816_G10
3	154.505	69	1	0.236	0.074	5.657	1171	WS00816_G10
5	52.820	99	1	0.285	0.068	5.543	1171	WS00816_G10
7	105.477	147	2	-0.322	0.094	7.143	1171	WS00816_G10
8	87.463	158	2	-0.298	0.108	11.930	1171	WS00816_G10
9	3.127	168	1	-0.376	0.086	4.491	1171	WS00816_G10
10	70.246	190	2	-0.171	0.042	4.709	1171	WS00816_G10
11	43.491	214	4	-0.160	0.036	4.350	1171	WS00816_G10
12	22.629	237	1	-0.179	0.061	4.633	1171	WS00816_G10
14	44.898	251	3	0.408	0.097	4.844	1171	WS00816_G10
2	48.839	29	1	0.277	0.040	4.122	1172	WS0262_K04
3	154.505	69	1	0.241	0.059	5.794	1172	WS0262_K04
5	0.000	89	3	0.234	0.067	6.188	1172	WS0262_K04
5	16.219	93	2	0.271	0.058	5.566	1172	WS0262_K04
7	73.169	142	1	0.623	0.117	6.301	1172	WS0262_K04
9	3.127	168	1	-0.455	0.079	6.420	1172	WS0262_K04
10	9.580	187	2	0.202	0.078	3.866	1172	WS0262_K04
10	9.580	187	3	-0.224	0.091	4.131	1172	WS0262_K04
11	17.088	203	2	0.101	0.032	3.947	1172	WS0262_K04
1	22.419	4	4	-0.338	0.089	4.364	1173	WS0262_N22
3	17.158	45	1	0.229	0.073	5.891	1173	WS0262_N22
3	80.211	55	4	-0.186	0.045	4.272	1173	WS0262_N22
6	25.914	114	1	-0.179	0.048	3.859	1173	WS0262_N22
6	56.188	120	2	0.255	0.046	4.036	1173	WS0262_N22
7	54.200	136	2	0.261	0.068	4.156	1173	WS0262_N22

8	50.001	154	2	-0.174	0.046	4.044	1173	WS0262_N22
8	87.463	158	2	0.296	0.068	7.391	1173	WS0262_N22
9	36.227	165	3	0.298	0.082	7.587	1173	WS0262_N22
9	17.951	169	1	-0.312	0.065	5.234	1173	WS0262_N22
9	17.951	169	2	0.304	0.036	4.545	1173	WS0262_N22
9	81.073	183	3	-0.268	0.067	6.303	1173	WS0262_N22
10	77.676	192	1	0.237	0.054	4.362	1173	WS0262_N22
10	80.088	194	1	0.185	0.052	4.202	1173	WS0262_N22
11	46.026	217	1	-0.241	0.047	3.848	1173	WS0262_N22
3	9.953	42	3	0.266	0.064	7.119	1174	WS0038_D17
3	116.958	63	4	-0.215	0.059	4.555	1174	WS0038_D17
4	140.962	84	3	-0.206	0.039	4.329	1174	WS0038_D17
6	81.996	123	3	0.291	0.086	6.008	1174	WS0038_D17
7	15.555	130	3	-0.269	0.043	4.876	1174	WS0038_D17
9	28.608	170	3	0.228	0.040	4.428	1174	WS0038_D17
11	45.989	216	1	-0.211	0.045	4.384	1174	WS0038_D17
11	57.661	220	4	0.232	0.060	4.600	1174	WS0038_D17
11	64.014	222	4	-0.327	0.071	5.479	1174	WS0038_D17
12	6.242	234	4	0.315	0.075	5.448	1174	WS0038_D17
4	173.929	88	2	-0.225	0.054	4.814	1175	WS0038_M08
5	16.219	93	1	-0.263	0.040	4.075	1175	WS0038_M08
5	31.191	95	1	-0.207	0.051	5.013	1175	WS0038_M08
5	57.017	102	4	-0.249	0.053	4.686	1175	WS0038_M08
6	25.914	114	1	-0.198	0.041	4.029	1175	WS0038_M08
6	81.996	123	3	0.304	0.101	6.628	1175	WS0038_M08
7	70.278	141	3	-0.271	0.050	5.622	1175	WS0038_M08
7	73.169	142	1	0.493	0.120	3.943	1175	WS0038_M08
7	75.883	144	2	0.470	0.120	5.700	1175	WS0038_M08
10	80.088	194	1	-0.247	0.042	4.188	1175	WS0038_M08
11	57.661	220	4	0.200	0.054	4.134	1175	WS0038_M08
14	44.898	251	3	0.390	0.069	7.808	1175	WS0038_M08
1	123.316	20	4	0.267	0.091	4.675	1176	WS00812_A16
6	25.914	114	1	-0.296	0.074	5.821	1176	WS00812_A16
7	15.555	130	3	-0.361	0.094	9.799	1176	WS00812_A16
8	50.001	154	2	-0.261	0.062	6.834	1176	WS00812_A16
10	104.214	198	4	0.239	0.035	3.859	1176	WS00812_A16
1	45.794	9	1	0.240	0.059	5.192	1177	WS00812_B19
1	82.301	16	4	0.210	0.050	5.261	1177	WS00812_B19
7	73.169	142	2	-0.485	0.103	5.112	1177	WS00812_B19
7	75.883	144	2	0.613	0.164	9.227	1177	WS00812_B19
9	0.000	166	1	-0.206	0.048	4.012	1177	WS00812_B19
9	17.951	169	1	0.327	0.070	5.827	1177	WS00812_B19
10	9.580	187	3	-0.368	0.156	5.046	1177	WS00812_B19
10	21.864	188	1	0.281	0.061	5.090	1177	WS00812_B19
11	27.405	204	4	-0.282	0.049	3.867	1177	WS00812_B19
11	45.989	216	1	-0.252	0.081	6.704	1177	WS00812_B19
4	173.929	88	2	-0.238	0.059	6.228	1178	WS00812_F05
5	54.072	100	4	0.272	0.073	7.147	1178	WS00812_F05
5	57.017	102	4	-0.334	0.076	7.152	1178	WS00812_F05
6	69.073	121	3	0.269	0.052	4.700	1178	WS00812_F05
1	123.316	20	4	0.371	0.138	6.954	1179	WS00812_F23
1	131.686	23	2	-0.470	0.106	3.910	1179	WS00812_F23
3	137.891	64	3	-0.272	0.053	5.665	1179	WS00812_F23
5	56.008	101	2	-0.145	0.034	4.042	1179	WS00812_F23

9	0.000	166	2	0.259	0.081	8.258	1179	WS00812_F23
9	17.951	169	2	-0.301	0.082	7.781	1179	WS00812_F23
11	40.960	210	4	-0.367	0.061	5.033	1179	WS00812_F23
11	43.491	214	4	-0.269	0.055	4.820	1179	WS00812_F23
11	44.884	215	4	0.352	0.050	4.394	1179	WS00812_F23
11	48.979	218	4	-0.405	0.078	5.395	1179	WS00812_F23
3	14.500	43	1	-0.211	0.044	4.228	1180	WS00812_I12
3	17.158	45	1	0.236	0.051	4.942	1180	WS00812_I12
6	81.996	123	3	0.277	0.106	5.260	1180	WS00812_I12
8	87.463	158	2	-0.209	0.048	3.895	1180	WS00812_I12
11	9.237	202	4	0.229	0.056	5.607	1180	WS00812_I12
11	27.405	204	4	-0.273	0.052	4.724	1180	WS00812_I12
4	0.000	71	4	0.260	0.061	6.558	1181	WS00812_J14
4	173.929	88	2	-0.217	0.040	4.210	1181	WS00812_J14
5	54.072	100	4	0.193	0.041	4.400	1181	WS00812_J14
5	57.017	102	4	-0.250	0.049	5.521	1181	WS00812_J14
6	81.996	123	3	0.328	0.095	4.350	1181	WS00812_J14
8	87.463	158	2	-0.220	0.057	5.968	1181	WS00812_J14
4	0.000	71	4	0.185	0.045	4.578	1182	WS00812_L24
8	87.463	158	2	-0.169	0.040	4.227	1182	WS00812_L24
9	59.963	177	4	0.259	0.039	3.898	1182	WS00812_L24
14	0.000	246	2	-0.155	0.041	4.107	1182	WS00812_L24
1	45.794	9	1	0.258	0.060	4.784	1183	WS00812_N05
1	45.794	9	3	-0.354	0.084	8.443	1183	WS00812_N05
7	75.883	144	2	0.520	0.117	6.774	1183	WS00812_N05
10	78.479	193	3	0.298	0.055	5.401	1183	WS00812_N05
11	45.989	216	1	-0.207	0.069	5.228	1183	WS00812_N05
4	173.929	88	2	-0.233	0.055	5.300	1184	WS0097_K23
6	81.996	123	3	0.319	0.126	6.026	1184	WS0097_K23
7	73.169	142	1	0.490	0.090	3.949	1184	WS0097_K23
11	57.661	220	4	0.244	0.043	4.444	1184	WS0097_K23
11	64.014	222	4	-0.320	0.052	5.060	1184	WS0097_K23
5	52.820	99	3	-0.271	0.048	4.792	1185	WS0097_L24
5	54.072	100	4	0.230	0.055	4.858	1185	WS0097_L24
6	81.996	123	3	0.332	0.112	6.351	1185	WS0097_L24
11	57.661	220	4	0.239	0.050	4.453	1185	WS0097_L24
11	64.014	222	4	-0.314	0.063	5.210	1185	WS0097_L24
14	0.000	246	2	-0.182	0.051	4.394	1185	WS0097_L24
3	154.505	69	1	0.232	0.046	4.065	1186	WS0097_N24
5	54.072	100	4	0.245	0.055	5.666	1186	WS0097_N24
5	57.017	102	4	-0.271	0.042	4.299	1186	WS0097_N24
1	2.788	2	4	-0.296	0.048	5.875	1187	WS0097_O12
1	38.326	6	4	-0.344	0.035	5.571	1187	WS0097_O12
3	14.500	43	1	-0.195	0.038	4.210	1187	WS0097_O12
3	141.443	65	4	0.256	0.089	6.506	1187	WS0097_O12
4	0.000	71	4	0.206	0.059	6.429	1187	WS0097_O12
4	55.925	76	1	0.243	0.023	4.540	1187	WS0097_O12
5	54.072	100	4	0.273	0.043	4.863	1187	WS0097_O12
5	57.017	102	4	-0.358	0.077	9.426	1187	WS0097_O12
7	62.787	139	4	-0.178	0.027	6.231	1187	WS0097_O12
7	67.154	140	4	-0.372	0.151	11.075	1187	WS0097_O12
7	73.169	142	1	0.738	0.147	10.808	1187	WS0097_O12
7	75.883	144	2	0.676	0.267	12.541	1187	WS0097_O12
7	75.883	144	4	-0.542	0.155	17.677	1187	WS0097_O12

9	3.127	168	1	-0.339	0.021	4.174	1187	WS0097_O12
12	38.543	232	4	-0.431	0.070	5.240	1187	WS0097_O12
1	95.572	17	3	0.219	0.054	4.965	1188	WS0099_A05
1	107.333	19	3	0.252	0.045	4.148	1188	WS0099_A05
3	15.147	44	2	-0.318	0.084	3.884	1188	WS0099_A05
3	17.158	45	1	-0.237	0.053	5.057	1188	WS0099_A05
3	137.891	64	3	0.279	0.081	7.215	1188	WS0099_A05
3	141.443	65	4	0.234	0.077	4.071	1188	WS0099_A05
4	55.925	76	1	0.204	0.039	4.330	1188	WS0099_A05
5	86.669	106	4	-0.466	0.130	6.280	1188	WS0099_A05
7	67.154	140	4	-0.300	0.110	5.411	1188	WS0099_A05
7	75.883	144	2	0.506	0.130	7.355	1188	WS0099_A05
7	75.883	144	4	-0.374	0.080	8.106	1188	WS0099_A05
9	28.608	170	3	0.346	0.086	7.386	1188	WS0099_A05
9	59.963	177	4	-0.358	0.075	7.212	1188	WS0099_A05
9	81.073	183	1	-0.385	0.114	10.887	1188	WS0099_A05
10	70.246	190	2	0.294	0.058	5.598	1188	WS0099_A05
10	77.676	192	1	-0.300	0.125	11.848	1188	WS0099_A05
10	80.088	194	1	-0.322	0.108	10.215	1188	WS0099_A05
10	80.088	194	2	-0.209	0.059	5.664	1188	WS0099_A05
11	45.989	216	1	-0.207	0.046	4.376	1188	WS0099_A05
11	46.026	217	1	-0.264	0.064	6.031	1188	WS0099_A05
12	51.382	242	1	-0.404	0.104	5.154	1188	WS0099_A05
1	2.788	2	4	-0.298	0.066	5.762	1189	WS0099_A17
1	38.326	6	4	-0.361	0.063	6.351	1189	WS0099_A17
1	40.464	7	4	0.371	0.066	7.052	1189	WS0099_A17
6	25.914	114	1	-0.233	0.038	4.026	1189	WS0099_A17
7	75.883	144	2	0.485	0.122	6.241	1189	WS0099_A17
9	3.127	168	1	-0.343	0.079	6.067	1189	WS0099_A17
9	28.608	170	3	0.280	0.049	4.115	1189	WS0099_A17
1	62.128	11	1	-0.688	0.200	9.134	1190	WS0099_C09
1	95.572	17	3	0.437	0.145	12.799	1190	WS0099_C09
3	47.928	49	1	0.247	0.078	7.289	1190	WS0099_C09
5	42.979	97	1	-0.276	0.061	5.782	1190	WS0099_C09
7	59.789	137	3	-0.282	0.087	7.799	1190	WS0099_C09
8	50.001	154	1	0.228	0.062	5.786	1190	WS0099_C09
8	95.937	159	3	0.222	0.064	5.688	1190	WS0099_C09
9	55.358	175	1	0.339	0.061	5.890	1190	WS0099_C09
10	21.864	188	1	0.327	0.055	5.320	1190	WS0099_C09
11	43.491	214	3	-0.245	0.082	7.336	1190	WS0099_C09
12	22.629	237	1	0.300	0.070	6.635	1190	WS0099_C09
14	0.000	246	1	-0.289	0.060	5.724	1190	WS0099_C09
1	2.788	2	3	0.287	0.102	9.150	1191	WS0099_D13
1	2.788	2	4	-0.282	0.044	4.231	1191	WS0099_D13
1	22.419	4	3	-0.358	0.102	5.992	1191	WS0099_D13
1	38.326	6	4	-0.311	0.049	4.135	1191	WS0099_D13
1	40.464	7	4	0.347	0.061	5.977	1191	WS0099_D13
7	15.555	130	2	0.281	0.055	5.171	1191	WS0099_D13
9	3.127	168	1	-0.331	0.095	5.159	1191	WS0099_D13
11	17.088	203	2	0.213	0.062	5.457	1191	WS0099_D13
12	22.441	227	1	0.276	0.044	4.305	1191	WS0099_D13
1	50.955	10	1	-0.238	0.048	4.444	1192	WS00932_M18
1	131.686	23	2	0.472	0.089	5.226	1192	WS00932_M18
6	25.914	114	1	-0.227	0.049	4.561	1192	WS00932_M18

6	87.891	124	2	0.210	0.051	4.855	1192	WS00932_M18
6	89.401	125	1	-0.370	0.063	5.840	1192	WS00932_M18
6	89.401	125	2	0.236	0.054	5.225	1192	WS00932_M18
7	15.555	130	2	0.238	0.054	5.307	1192	WS00932_M18
9	36.227	165	4	0.330	0.051	6.474	1192	WS00932_M18
11	27.405	204	1	-0.190	0.041	3.859	1192	WS00932_M18
12	0.000	233	1	-0.246	0.068	6.235	1192	WS00932_M18
12	32.473	238	1	-0.212	0.045	4.195	1192	WS00932_M18
14	22.402	248	4	-0.297	0.063	6.220	1192	WS00932_M18
14	46.637	252	4	-0.234	0.045	3.926	1192	WS00932_M18
2	48.839	29	4	-0.412	0.106	8.185	1193	WS00932_O18
3	83.563	56	3	0.208	0.072	8.078	1193	WS00932_O18
6	25.742	113	3	-0.314	0.144	16.119	1193	WS00932_O18
6	25.914	114	1	-0.313	0.142	11.159	1193	WS00932_O18
6	26.350	115	2	-0.516	0.213	23.292	1193	WS00932_O18
6	45.897	117	1	0.397	0.099	8.124	1193	WS00932_O18
6	49.100	118	2	0.386	0.175	9.373	1193	WS00932_O18
6	56.188	120	1	0.319	0.057	4.587	1193	WS00932_O18
6	56.188	120	2	0.423	0.107	11.772	1193	WS00932_O18
6	87.891	124	2	0.153	0.038	4.104	1193	WS00932_O18
7	59.789	137	3	-0.335	0.072	8.236	1193	WS00932_O18
7	70.278	141	3	0.306	0.062	7.103	1193	WS00932_O18
9	55.358	175	3	0.419	0.085	4.607	1193	WS00932_O18
2	126.181	39	2	0.230	0.037	4.140	1194	WS00933_B23
3	15.147	44	2	0.247	0.068	4.572	1194	WS00933_B23
7	75.883	144	2	-0.584	0.131	9.139	1194	WS00933_B23
9	0.000	166	2	0.191	0.034	3.894	1194	WS00933_B23
1	107.333	19	3	0.167	0.036	4.037	1195	WS00933_D12
2	127.147	40	3	-0.222	0.039	4.413	1195	WS00933_D12
6	49.100	118	2	0.279	0.152	6.812	1195	WS00933_D12
4	173.929	88	2	0.221	0.047	5.003	1196	WS00933_H18
6	106.702	128	2	0.438	0.092	5.231	1196	WS00933_H18
10	78.479	193	3	-0.223	0.042	4.672	1196	WS00933_H18
10	86.151	195	3	0.275	0.054	6.053	1196	WS00933_H18
11	40.960	210	2	0.378	0.075	3.998	1196	WS00933_H18
11	64.014	222	3	0.219	0.041	4.537	1196	WS00933_H18
14	22.402	248	3	-0.295	0.056	6.251	1196	WS00933_H18
1	29.800	5	1	-0.261	0.044	4.015	1197	WS00933_J02
4	43.146	74	3	0.231	0.040	4.017	1197	WS00933_J02
5	16.219	93	1	-0.224	0.057	5.152	1197	WS00933_J02
5	52.820	99	1	-0.305	0.057	5.321	1197	WS00933_J02
5	56.008	101	2	-0.257	0.046	4.105	1197	WS00933_J02
5	57.017	102	2	0.209	0.042	4.070	1197	WS00933_J02
5	84.347	105	2	-0.232	0.060	5.619	1197	WS00933_J02
6	49.100	118	4	0.357	0.193	4.831	1197	WS00933_J02
7	75.883	144	2	-0.436	0.093	5.108	1197	WS00933_J02
11	27.405	204	4	-0.221	0.055	4.529	1197	WS00933_J02
11	40.960	210	2	0.423	0.092	5.212	1197	WS00933_J02
11	40.960	210	4	-0.325	0.058	5.182	1197	WS00933_J02
11	43.491	214	3	-0.204	0.069	6.900	1197	WS00933_J02
13	1.815	244	4	0.313	0.103	7.005	1197	WS00933_J02
1	95.572	17	2	-0.292	0.074	7.788	1198	WS00933_M02
1	95.572	17	3	0.289	0.089	9.369	1198	WS00933_M02
5	0.000	89	1	-0.193	0.048	3.860	1198	WS00933_M02

6	95.249	126	1	0.314	0.065	4.082	1198	WS00933_M02
9	28.608	170	3	0.340	0.072	6.183	1198	WS00933_M02
9	55.358	175	3	0.426	0.076	4.745	1198	WS00933_M02
11	17.088	203	1	-0.226	0.048	3.845	1198	WS00933_M02
12	6.242	234	2	0.239	0.040	5.457	1198	WS00933_M02
4	173.929	88	2	-0.191	0.049	4.681	1199	WS00933_N08
5	54.072	100	4	0.240	0.062	5.752	1199	WS00933_N08
5	57.017	102	4	-0.213	0.048	4.457	1199	WS00933_N08
6	81.996	123	3	0.319	0.110	6.280	1199	WS00933_N08
1	107.333	19	3	0.249	0.044	4.633	1200	WS00931_F02
3	137.891	64	1	0.251	0.069	4.898	1200	WS00931_F02
4	0.000	71	4	0.249	0.057	4.965	1200	WS00931_F02
4	55.925	76	1	0.211	0.057	4.275	1200	WS00931_F02
4	75.958	77	3	-0.302	0.064	3.986	1200	WS00931_F02
5	3.484	90	2	0.329	0.082	4.577	1200	WS00931_F02
9	28.608	170	3	0.300	0.055	5.457	1200	WS00931_F02
10	9.580	187	3	0.273	0.151	5.694	1200	WS00931_F02
12	6.242	234	2	0.265	0.037	4.699	1200	WS00931_F02
6	25.914	114	1	-0.265	0.075	6.509	1201	WS00931_I04
7	73.169	142	1	0.556	0.134	4.991	1201	WS00931_I04
8	39.090	152	4	-0.240	0.071	5.630	1201	WS00931_I04
9	0.000	163	2	-0.443	0.066	3.913	1201	WS00931_I04
9	36.227	165	3	0.171	0.038	4.154	1201	WS00931_I04
10	9.580	187	3	-0.334	0.107	5.473	1201	WS00931_I04
6	81.996	123	3	0.334	0.064	4.581	1202	WS01025_J24
10	9.580	187	3	-0.237	0.066	4.652	1202	WS01025_J24
3	9.953	42	3	-0.230	0.046	4.204	1203	WS01025_P03
3	9.953	42	4	0.242	0.057	5.556	1203	WS01025_P03
3	14.500	43	1	0.266	0.092	5.145	1203	WS01025_P03
8	95.937	159	3	0.187	0.049	4.446	1203	WS01025_P03
10	86.151	195	3	0.250	0.056	5.060	1203	WS01025_P03
10	93.347	196	1	0.445	0.143	4.744	1203	WS01025_P03
11	17.088	203	2	-0.251	0.050	6.526	1203	WS01025_P03
12	28.493	228	2	0.314	0.086	6.390	1203	WS01025_P03
12	6.242	234	4	0.251	0.040	5.567	1203	WS01025_P03
3	9.953	42	4	0.171	0.034	4.117	1204	WS0076_A13
5	16.219	93	1	-0.247	0.042	3.984	1204	WS0076_A13
5	54.072	100	4	0.221	0.047	5.016	1204	WS0076_A13
5	57.017	102	4	-0.248	0.050	5.661	1204	WS0076_A13
6	25.914	114	1	-0.254	0.071	6.693	1204	WS0076_A13
9	2.011	167	4	-0.213	0.037	3.946	1204	WS0076_A13
5	52.820	99	3	-0.321	0.066	4.061	1205	WS0076_A23
5	54.072	100	4	0.226	0.053	5.318	1205	WS0076_A23
11	27.405	204	4	-0.242	0.047	4.236	1205	WS0076_A23
1	2.788	2	4	-0.271	0.060	6.288	1206	WS0076_C09
1	45.794	9	1	0.280	0.043	4.737	1206	WS0076_C09
3	14.500	43	1	-0.338	0.095	10.318	1206	WS0076_C09
3	17.158	45	1	0.370	0.096	10.509	1206	WS0076_C09
8	50.001	154	2	-0.228	0.067	5.072	1206	WS0076_C09
11	35.604	207	3	-0.220	0.048	4.386	1206	WS0076_C09
11	37.143	209	3	0.225	0.050	4.582	1206	WS0076_C09
13	3.386	245	1	0.236	0.039	4.262	1206	WS0076_C09
14	0.000	246	1	-0.198	0.038	4.127	1206	WS0076_C09
14	0.000	246	2	-0.208	0.055	4.281	1206	WS0076_C09

1	2.788	2	4	-0.309	0.052	5.755	1207	WS0076_C18
2	48.839	29	1	0.292	0.053	5.452	1207	WS0076_C18
3	154.505	69	1	0.274	0.056	5.896	1207	WS0076_C18
8	11.974	150	1	-0.177	0.044	4.525	1207	WS0076_C18
8	78.389	157	1	0.253	0.038	4.023	1207	WS0076_C18
1	95.572	17	2	0.328	0.061	6.981	1208	WS0076_D23
5	54.072	100	4	0.283	0.070	6.794	1208	WS0076_D23
9	59.963	177	4	0.277	0.053	4.680	1208	WS0076_D23
11	43.491	214	4	-0.182	0.040	3.905	1208	WS0076_D23
12	6.242	234	2	-0.241	0.055	8.589	1208	WS0076_D23
14	0.000	246	1	-0.255	0.057	4.177	1208	WS0076_D23
1	45.794	9	1	0.280	0.036	4.336	1209	WS0076_E13
1	82.301	16	4	0.400	0.084	5.793	1209	WS0076_E13
4	103.719	81	4	-0.261	0.038	3.975	1209	WS0076_E13
5	0.000	89	1	0.494	0.218	20.454	1209	WS0076_E13
5	0.000	89	3	0.361	0.176	14.664	1209	WS0076_E13
5	7.672	92	1	0.446	0.175	22.297	1209	WS0076_E13
5	16.219	93	1	0.473	0.175	20.657	1209	WS0076_E13
6	45.897	117	1	0.252	0.031	3.875	1209	WS0076_E13
6	56.188	120	1	0.403	0.073	8.725	1209	WS0076_E13
12	14.406	230	4	0.225	0.037	3.928	1209	WS0076_E13
12	0.000	233	1	-0.193	0.068	8.028	1209	WS0076_E13
12	6.242	234	2	-0.187	0.048	4.078	1209	WS0076_E13
12	22.629	237	1	-0.240	0.091	10.669	1209	WS0076_E13
12	32.473	238	1	-0.280	0.112	13.185	1209	WS0076_E13
14	43.392	250	1	-0.361	0.052	4.854	1209	WS0076_E13
1	131.686	23	2	0.483	0.079	5.520	1210	WS0076_G03
4	103.719	81	4	-0.256	0.036	3.902	1210	WS0076_G03
6	69.073	121	3	0.291	0.044	4.132	1210	WS0076_G03
6	87.891	124	2	0.178	0.042	4.381	1210	WS0076_G03
6	89.401	125	2	0.345	0.074	7.612	1210	WS0076_G03
8	50.001	154	2	-0.181	0.049	5.035	1210	WS0076_G03
9	59.963	177	4	0.337	0.062	5.597	1210	WS0076_G03
10	93.347	196	1	0.415	0.070	4.141	1210	WS0076_G03
12	0.000	233	1	-0.256	0.091	7.625	1210	WS0076_G03
12	22.629	237	1	-0.213	0.063	5.246	1210	WS0076_G03
3	14.500	43	1	-0.363	0.073	7.431	1211	WS0076_G10
3	17.158	45	1	0.234	0.064	6.486	1211	WS0076_G10
7	59.789	137	3	-0.238	0.050	5.169	1211	WS0076_G10
11	35.604	207	3	-0.284	0.071	7.320	1211	WS0076_G10
11	37.143	209	3	0.281	0.069	7.108	1211	WS0076_G10
12	6.242	234	2	-0.287	0.066	6.508	1211	WS0076_G10
14	9.923	247	1	0.251	0.040	4.078	1211	WS0076_G10
1	107.333	19	4	-0.092	0.024	3.904	1212	WS0073_M07
2	48.839	29	4	0.206	0.051	6.527	1212	WS0073_M07
3	144.593	66	4	0.161	0.051	5.616	1212	WS0073_M07
3	148.745	68	4	-0.262	0.071	8.352	1212	WS0073_M07
3	154.505	69	4	0.191	0.043	4.485	1212	WS0073_M07
4	116.928	82	4	0.312	0.078	9.135	1212	WS0073_M07
5	57.017	102	2	-0.237	0.041	4.673	1212	WS0073_M07
8	87.463	158	1	-0.233	0.052	4.072	1212	WS0073_M07
9	3.127	168	1	0.437	0.118	5.294	1212	WS0073_M07
10	80.088	194	2	0.271	0.066	8.196	1212	WS0073_M07
10	93.347	196	2	-0.492	0.131	4.843	1212	WS0073_M07

14	22.402	248	4	-0.288	0.029	4.304	1212	WS0073_M07
14	46.637	252	4	-0.322	0.044	5.990	1212	WS0073_M07
4	75.958	77	3	-0.314	0.100	4.496	1213	WS0073_N06
5	7.672	92	4	-0.357	0.075	6.759	1213	WS0073_N06
6	54.066	119	4	0.136	0.043	3.948	1213	WS0073_N06
6	69.073	121	4	-0.250	0.089	7.033	1213	WS0073_N06
9	28.608	170	2	-0.302	0.051	6.287	1213	WS0073_N06
9	28.608	170	3	0.603	0.231	23.782	1213	WS0073_N06
9	55.358	175	3	0.478	0.104	4.756	1213	WS0073_N06
11	0.000	199	3	-0.383	0.074	7.270	1213	WS0073_N06
11	40.960	210	2	0.366	0.056	3.929	1213	WS0073_N06
11	43.491	214	3	-0.234	0.045	4.535	1213	WS0073_N06
11	44.884	215	3	0.259	0.042	4.125	1213	WS0073_N06
4	0.000	71	4	0.308	0.069	7.369	1214	WS0073_P19
5	54.072	100	4	0.285	0.065	6.930	1214	WS0073_P19
5	57.017	102	4	-0.300	0.069	7.609	1214	WS0073_P19
6	81.996	123	3	0.275	0.081	3.993	1214	WS0073_P19
8	87.463	158	2	-0.210	0.042	4.323	1214	WS0073_P19
10	70.246	190	2	-0.193	0.038	3.934	1214	WS0073_P19
11	64.014	222	4	-0.308	0.056	5.252	1214	WS0073_P19
12	6.242	234	2	-0.186	0.035	4.232	1214	WS0073_P19
1	71.592	14	2	0.189	0.035	3.958	1215	WS0074_A16
3	14.500	43	2	-0.311	0.064	7.602	1215	WS0074_A16
3	20.512	46	2	0.261	0.053	6.100	1215	WS0074_A16
6	25.914	114	1	0.223	0.065	4.811	1215	WS0074_A16
8	50.001	154	2	-0.193	0.034	3.857	1215	WS0074_A16
12	6.242	234	2	-0.252	0.048	6.428	1215	WS0074_A16
12	6.242	234	4	0.475	0.090	8.563	1215	WS0074_A16
13	0.000	243	4	0.251	0.119	4.685	1215	WS0074_A16
1	50.955	10	1	-0.260	0.082	5.400	1216	WS0074_C12
1	69.854	13	2	-0.185	0.036	4.371	1216	WS0074_C12
1	71.592	14	2	-0.195	0.040	4.906	1216	WS0074_C12
1	95.572	17	2	-0.336	0.074	9.209	1216	WS0074_C12
2	75.550	31	2	0.230	0.032	3.962	1216	WS0074_C12
2	93.504	32	2	0.271	0.051	6.341	1216	WS0074_C12
4	92.039	79	4	-0.261	0.047	3.878	1216	WS0074_C12
5	57.017	102	2	0.209	0.038	4.652	1216	WS0074_C12
6	69.073	121	3	0.247	0.037	3.865	1216	WS0074_C12
7	105.477	147	2	0.346	0.134	7.553	1216	WS0074_C12
3	137.891	64	3	0.425	0.134	12.782	1217	WS0074_D18
5	3.484	90	2	-0.274	0.091	5.500	1217	WS0074_D18
7	75.883	144	2	0.448	0.088	5.364	1217	WS0074_D18
9	49.013	173	2	0.198	0.037	4.925	1217	WS0074_D18
14	0.000	246	2	-0.259	0.058	7.109	1217	WS0074_D18
1	123.316	20	4	-0.243	0.069	5.321	1218	WS0074_F13
3	137.891	64	1	-0.230	0.067	5.056	1218	WS0074_F13
5	31.191	95	1	0.173	0.052	3.895	1218	WS0074_F13
11	41.984	212	2	0.192	0.047	5.281	1218	WS0074_F13
11	45.989	216	2	0.230	0.062	7.049	1218	WS0074_F13
1	69.854	13	2	0.225	0.062	7.061	1219	WS0074_F24
1	71.592	14	2	0.212	0.056	6.361	1219	WS0074_F24
1	77.177	15	2	0.214	0.056	6.327	1219	WS0074_F24
1	95.572	17	2	0.272	0.039	4.561	1219	WS0074_F24
3	116.958	63	4	-0.210	0.050	5.234	1219	WS0074_F24

4	173.929	88	2	-0.209	0.039	4.450	1219	WS0074_F24
5	54.072	100	4	0.303	0.063	6.616	1219	WS0074_F24
6	81.996	123	3	0.289	0.118	5.000	1219	WS0074_F24
9	2.011	167	4	-0.229	0.041	4.277	1219	WS0074_F24
11	64.014	222	4	-0.382	0.075	7.347	1219	WS0074_F24
14	0.000	246	2	-0.189	0.043	4.600	1219	WS0074_F24
1	127.805	21	3	0.173	0.037	4.738	1220	WS01026_O03
2	93.504	32	2	-0.245	0.041	4.194	1220	WS01026_O03
2	120.617	38	3	-0.280	0.051	6.582	1220	WS01026_O03
2	127.147	40	3	0.233	0.041	5.280	1220	WS01026_O03
3	14.500	43	2	-0.256	0.081	7.700	1220	WS01026_O03
3	15.147	44	2	0.313	0.167	6.417	1220	WS01026_O03
3	20.512	46	2	0.357	0.106	10.584	1220	WS01026_O03
4	43.146	74	3	-0.276	0.062	8.025	1220	WS01026_O03
4	82.462	78	1	-0.207	0.043	3.855	1220	WS01026_O03
8	50.001	154	2	-0.211	0.058	5.792	1220	WS01026_O03
9	36.227	165	3	0.262	0.041	5.385	1220	WS01026_O03
9	81.073	183	3	-0.293	0.045	5.731	1220	WS01026_O03
11	40.960	210	2	0.448	0.139	5.289	1220	WS01026_O03
11	48.979	218	2	0.264	0.051	5.043	1220	WS01026_O03
11	60.464	221	2	0.229	0.043	4.355	1220	WS01026_O03
12	0.000	226	3	-0.255	0.071	9.206	1220	WS01026_O03
12	22.441	227	3	0.287	0.041	5.329	1220	WS01026_O03
1	15.589	3	2	0.223	0.058	5.813	1221	WS00912_D09
1	95.572	17	3	0.384	0.087	8.460	1221	WS00912_D09
1	107.333	19	3	0.212	0.064	6.843	1221	WS00912_D09
1	127.805	21	3	0.212	0.045	4.878	1221	WS00912_D09
6	25.914	114	1	-0.226	0.045	3.943	1221	WS00912_D09
6	69.073	121	4	-0.271	0.054	4.256	1221	WS00912_D09
9	55.358	175	3	0.488	0.099	5.358	1221	WS00912_D09
10	9.580	187	3	-0.312	0.114	6.330	1221	WS00912_D09
11	40.960	210	2	0.432	0.097	5.663	1221	WS00912_D09
11	40.960	210	4	-0.326	0.064	5.260	1221	WS00912_D09
1	15.589	3	2	0.201	0.041	3.990	1222	WS00819_D01
1	69.854	13	2	0.223	0.048	4.768	1222	WS00819_D01
1	71.592	14	2	0.227	0.051	5.011	1222	WS00819_D01
1	82.301	16	3	0.242	0.034	3.862	1222	WS00819_D01
1	95.572	17	3	-0.368	0.078	8.904	1222	WS00819_D01
1	107.333	19	3	-0.313	0.127	14.149	1222	WS00819_D01
1	127.805	21	3	-0.261	0.063	6.991	1222	WS00819_D01
2	95.880	34	3	0.219	0.050	4.662	1222	WS00819_D01
2	101.076	35	3	-0.248	0.058	6.420	1222	WS00819_D01
2	120.617	38	3	-0.327	0.061	6.892	1222	WS00819_D01
2	127.147	40	3	0.376	0.106	11.824	1222	WS00819_D01
3	95.157	59	2	-0.224	0.047	4.804	1222	WS00819_D01
6	69.073	121	4	-0.197	0.064	4.999	1222	WS00819_D01
6	89.401	125	1	-0.241	0.044	4.074	1222	WS00819_D01
7	75.883	144	2	0.414	0.082	4.504	1222	WS00819_D01
14	43.392	250	1	0.358	0.094	6.288	1222	WS00819_D01
1	15.589	3	2	-0.222	0.038	3.979	1223	WS00912_I20
3	47.928	49	1	-0.279	0.057	4.864	1223	WS00912_I20
4	0.000	71	4	0.142	0.039	4.075	1223	WS00912_I20
5	57.017	102	4	-0.236	0.038	3.931	1223	WS00912_I20
6	89.401	125	1	0.216	0.051	4.310	1223	WS00912_I20

10	78.479	193	3	0.293	0.057	4.849	1223	WS00912_I20
4	55.925	76	1	0.196	0.044	4.237	1224	WS00829_H17
7	15.555	130	3	-0.237	0.060	5.871	1224	WS00829_H17
7	91.487	146	3	-0.244	0.038	4.210	1224	WS00829_H17
9	28.608	170	2	-0.227	0.057	5.692	1224	WS00829_H17
9	28.608	170	3	0.268	0.037	4.015	1224	WS00829_H17
10	78.479	193	4	-0.215	0.049	3.859	1224	WS00829_H17
10	80.088	194	1	-0.226	0.052	4.843	1224	WS00829_H17
12	0.000	233	1	-0.307	0.083	7.704	1224	WS00829_H17
1	2.788	2	4	-0.228	0.060	7.332	1225	WS00830_C15
1	45.794	9	1	0.317	0.086	6.772	1225	WS00830_C15
4	0.000	71	4	0.249	0.037	4.207	1225	WS00830_C15
4	173.929	88	2	-0.223	0.058	6.389	1225	WS00830_C15
5	54.072	100	4	0.252	0.051	5.772	1225	WS00830_C15
11	27.405	204	4	-0.356	0.061	5.800	1225	WS00830_C15
1	69.854	13	2	0.263	0.064	6.487	1226	WS00931_M13
1	71.592	14	2	0.272	0.066	6.757	1226	WS00931_M13
1	77.177	15	2	0.229	0.046	4.715	1226	WS00931_M13
1	82.301	16	4	-0.276	0.060	4.213	1226	WS00931_M13
1	95.572	17	3	0.200	0.041	4.919	1226	WS00931_M13
3	14.500	43	1	-0.191	0.053	4.521	1226	WS00931_M13
3	17.158	45	1	0.303	0.063	5.547	1226	WS00931_M13
6	26.350	115	2	0.259	0.056	5.724	1226	WS00931_M13
7	15.555	130	2	0.207	0.049	4.929	1226	WS00931_M13
7	17.610	131	3	0.280	0.069	4.290	1226	WS00931_M13
8	95.937	159	3	0.234	0.039	4.540	1226	WS00931_M13
10	9.580	187	3	-0.470	0.138	4.866	1226	WS00931_M13
12	38.543	232	4	0.329	0.141	4.432	1226	WS00931_M13
14	0.000	246	2	-0.211	0.040	4.360	1226	WS00931_M13
1	15.589	3	2	-0.268	0.051	6.033	1227	WS01029_B01
1	45.794	9	1	0.398	0.087	6.427	1227	WS01029_B01
2	95.880	34	1	-0.326	0.058	4.220	1227	WS01029_B01
3	20.512	46	2	-0.310	0.062	7.434	1227	WS01029_B01
7	15.555	130	2	0.437	0.104	12.464	1227	WS01029_B01
7	67.154	140	4	-0.201	0.088	3.898	1227	WS01029_B01
7	75.883	144	4	-0.401	0.055	4.367	1227	WS01029_B01
14	44.898	251	3	0.179	0.049	4.107	1227	WS01029_B01
1	45.794	9	1	0.327	0.052	5.597	1228	WS00913_I24
2	48.839	29	4	-0.353	0.087	6.919	1228	WS00913_I24
2	75.550	31	1	0.262	0.042	4.480	1228	WS00913_I24
3	137.891	64	1	-0.253	0.039	4.262	1228	WS00913_I24
3	154.505	69	1	0.147	0.048	5.161	1228	WS00913_I24
6	25.914	114	1	-0.142	0.042	4.529	1228	WS00913_I24
6	45.897	117	1	0.315	0.046	5.176	1228	WS00913_I24
6	56.188	120	1	0.349	0.052	5.731	1228	WS00913_I24
8	62.580	155	1	0.318	0.127	3.941	1228	WS00913_I24
11	17.088	203	1	0.280	0.036	4.058	1228	WS00913_I24
11	27.405	204	1	-0.348	0.054	5.980	1228	WS00913_I24
11	29.069	205	1	-0.255	0.036	3.951	1228	WS00913_I24
11	56.921	219	1	-0.343	0.154	4.773	1228	WS00913_I24
2	126.181	39	4	0.479	0.130	4.187	1229	WS00913_O20
3	9.953	42	3	0.221	0.037	4.398	1229	WS00913_O20
3	83.563	56	3	-0.212	0.044	5.216	1229	WS00913_O20
6	69.073	121	3	0.226	0.039	4.669	1229	WS00913_O20

6	81.996	123	3	0.331	0.101	8.070	1229	WS00913_O20
7	15.555	130	3	-0.268	0.044	5.473	1229	WS00913_O20
7	70.278	141	3	-0.213	0.039	4.680	1229	WS00913_O20
9	28.608	170	3	0.322	0.076	8.872	1229	WS00913_O20
9	55.358	175	3	0.426	0.061	4.752	1229	WS00913_O20
11	45.989	216	1	-0.262	0.063	6.520	1229	WS00913_O20
14	44.898	251	3	0.256	0.037	4.386	1229	WS00913_O20
3	9.953	42	3	0.240	0.037	4.300	1230	WS0052_E13
3	137.891	64	3	0.260	0.057	6.550	1230	WS0052_E13
4	116.928	82	2	-0.317	0.069	6.986	1230	WS0052_E13
5	57.017	102	4	-0.271	0.079	5.828	1230	WS0052_E13
7	70.278	141	3	-0.222	0.048	5.528	1230	WS0052_E13
8	87.463	158	2	-0.186	0.045	4.331	1230	WS0052_E13
9	69.046	181	2	0.216	0.076	4.138	1230	WS0052_E13
11	56.921	219	1	-0.318	0.134	3.991	1230	WS0052_E13
12	22.441	227	3	0.283	0.040	4.612	1230	WS0052_E13
4	75.958	77	3	-0.383	0.129	5.399	1231	WS0052_F17
4	124.132	83	1	0.410	0.107	3.843	1231	WS0052_F17
6	54.066	119	4	0.195	0.047	4.293	1231	WS0052_F17
7	15.555	130	3	-0.288	0.050	4.203	1231	WS0052_F17
10	70.246	190	4	-0.329	0.067	5.719	1231	WS0052_F17
10	78.479	193	4	-0.312	0.096	9.047	1231	WS0052_F17
10	80.088	194	1	-0.276	0.044	3.972	1231	WS0052_F17
11	56.921	219	1	-0.324	0.150	5.284	1231	WS0052_F17
11	57.661	220	4	0.233	0.047	4.330	1231	WS0052_F17
13	0.000	243	4	0.422	0.152	5.394	1231	WS0052_F17
13	3.386	245	1	-0.244	0.058	5.244	1231	WS0052_F17
3	137.891	64	1	-0.175	0.064	4.015	1232	WS0052_M06
3	137.891	64	3	0.352	0.071	7.038	1232	WS0052_M06
3	154.505	69	4	0.265	0.050	4.228	1232	WS0052_M06
4	75.958	77	3	-0.246	0.066	4.636	1232	WS0052_M06
5	57.017	102	2	-0.191	0.030	3.888	1232	WS0052_M06
10	21.864	188	3	0.186	0.040	4.425	1232	WS0052_M06
11	0.000	199	2	-0.244	0.042	5.399	1232	WS0052_M06
12	22.441	227	1	0.230	0.078	4.735	1232	WS0052_M06
3	116.958	63	4	-0.183	0.046	4.965	1233	WS0052_N22
4	116.928	82	4	0.339	0.090	7.268	1233	WS0052_N22
5	54.072	100	4	0.172	0.046	4.952	1233	WS0052_N22
7	15.555	130	3	-0.348	0.076	5.842	1233	WS0052_N22
9	36.227	165	4	0.178	0.035	3.917	1233	WS0052_N22
10	70.246	190	4	-0.287	0.072	7.644	1233	WS0052_N22
11	0.000	199	2	-0.236	0.043	4.341	1233	WS0052_N22
1	95.572	17	2	-0.202	0.032	3.901	1234	WS0053_B20
1	107.333	19	3	0.170	0.051	4.471	1234	WS0053_B20
4	55.925	76	1	0.234	0.061	4.458	1234	WS0053_B20
4	75.958	77	3	-0.320	0.141	5.509	1234	WS0053_B20
9	59.963	177	4	-0.214	0.033	3.943	1234	WS0053_B20
11	0.000	199	2	-0.268	0.047	5.680	1234	WS0053_B20
12	6.242	234	4	0.327	0.051	5.208	1234	WS0053_B20
6	49.100	118	2	-0.296	0.090	5.475	1235	WS0053_C14
6	81.996	123	3	0.324	0.122	4.821	1235	WS0053_C14
7	62.787	139	4	0.313	0.072	5.834	1235	WS0053_C14
7	70.278	141	3	-0.206	0.046	4.687	1235	WS0053_C14
8	87.463	158	2	-0.237	0.064	6.907	1235	WS0053_C14

10	9.580	187	3	-0.321	0.146	4.233	1235	WS0053_C14
13	0.000	243	4	0.453	0.185	7.372	1235	WS0053_C14
3	15.147	44	2	0.257	0.054	3.943	1236	WS0053_F12
4	173.929	88	2	-0.223	0.042	4.788	1236	WS0053_F12
6	69.073	121	3	0.202	0.053	4.348	1236	WS0053_F12
6	81.996	123	3	0.331	0.115	5.041	1236	WS0053_F12
8	87.463	158	2	-0.237	0.057	6.431	1236	WS0053_F12
11	27.405	204	4	-0.307	0.051	5.281	1236	WS0053_F12
4	116.928	82	2	-0.248	0.036	4.251	1237	WS0053_H11
6	69.073	121	4	-0.219	0.062	4.177	1237	WS0053_H11
7	17.610	131	3	-0.244	0.070	4.063	1237	WS0053_H11
10	35.204	189	2	-0.273	0.042	4.709	1237	WS0053_H11
10	104.214	198	2	-0.167	0.031	4.596	1237	WS0053_H11
11	46.026	217	1	-0.236	0.083	5.634	1237	WS0053_H11
12	6.242	234	4	0.299	0.053	5.518	1237	WS0053_H11
4	142.917	85	1	0.218	0.048	5.221	1238	WS01033_E21
5	7.672	92	1	0.268	0.055	6.724	1238	WS01033_E21
12	22.441	227	1	0.189	0.040	4.349	1238	WS01033_E21
12	14.406	230	3	0.248	0.049	4.598	1238	WS01033_E21
12	22.629	237	1	-0.275	0.050	5.456	1238	WS01033_E21
12	32.473	238	1	-0.255	0.043	4.663	1238	WS01033_E21
4	0.000	71	4	0.244	0.054	5.685	1239	WS01033_F16
4	173.929	88	2	-0.213	0.046	4.611	1239	WS01033_F16
5	54.072	100	4	0.210	0.049	5.130	1239	WS01033_F16
5	57.017	102	4	-0.211	0.041	4.311	1239	WS01033_F16
4	140.962	84	3	-0.221	0.038	4.570	1240	WS01033_I05
5	52.820	99	3	-0.323	0.062	7.310	1240	WS01033_I05
6	81.996	123	3	0.349	0.104	7.032	1240	WS01033_I05
7	15.555	130	3	-0.273	0.052	6.153	1240	WS01033_I05
7	70.278	141	3	-0.218	0.050	5.963	1240	WS01033_I05
7	73.169	142	1	0.503	0.151	4.053	1240	WS01033_I05
7	91.487	146	3	-0.171	0.033	3.878	1240	WS01033_I05
10	80.088	194	1	-0.312	0.057	5.478	1240	WS01033_I05
10	93.347	196	1	0.537	0.101	6.914	1240	WS01033_I05
12	6.242	234	2	-0.206	0.039	4.442	1240	WS01033_I05
1	38.326	6	4	-0.191	0.039	3.913	1241	WS01033_I24
1	62.128	11	1	-0.583	0.222	6.501	1241	WS01033_I24
3	9.953	42	4	0.317	0.089	9.091	1241	WS01033_I24
9	0.000	163	1	0.489	0.095	5.278	1241	WS01033_I24
9	0.000	163	2	0.447	0.117	3.989	1241	WS01033_I24
9	28.608	170	2	0.242	0.041	4.403	1241	WS01033_I24
11	27.405	204	4	-0.176	0.042	4.737	1241	WS01033_I24
13	1.815	244	4	0.223	0.066	6.739	1241	WS01033_I24
14	43.392	250	1	-0.413	0.080	4.696	1241	WS01033_I24
14	46.637	252	4	-0.284	0.031	4.188	1241	WS01033_I24
1	127.805	21	3	0.148	0.044	4.104	1242	WS01033_M20
5	86.669	106	2	-0.288	0.087	4.301	1242	WS01033_M20
6	49.100	118	2	-0.300	0.093	4.569	1242	WS01033_M20
6	49.100	118	4	0.307	0.137	5.999	1242	WS01033_M20
6	54.066	119	4	0.239	0.043	4.075	1242	WS01033_M20
6	69.073	121	3	0.253	0.060	5.605	1242	WS01033_M20
11	0.000	199	2	-0.192	0.039	3.999	1242	WS01033_M20
1	2.788	2	4	-0.235	0.046	4.244	1243	WS01033_O06
2	93.504	32	2	-0.194	0.032	3.876	1243	WS01033_O06

3	14.500	43	1	-0.257	0.066	4.405	1243	WS01033_O06
3	17.158	45	1	0.243	0.076	5.102	1243	WS01033_O06
5	54.072	100	4	0.255	0.076	6.456	1243	WS01033_O06
5	57.017	102	4	-0.254	0.064	5.542	1243	WS01033_O06
12	6.242	234	2	-0.236	0.030	4.291	1243	WS01033_O06
3	95.157	59	2	0.228	0.044	4.641	1244	WS01034_B03
5	84.347	105	1	0.162	0.047	4.213	1244	WS01034_B03
7	15.555	130	2	0.284	0.039	4.056	1244	WS01034_B03
1	2.788	2	4	-0.320	0.050	4.600	1245	WS01034_C12
4	103.719	81	4	-0.281	0.057	6.715	1245	WS01034_C12
4	124.132	83	1	0.472	0.091	4.518	1245	WS01034_C12
6	87.891	124	2	0.166	0.045	4.062	1245	WS01034_C12
9	3.127	168	1	-0.366	0.079	5.072	1245	WS01034_C12
10	9.580	187	3	-0.327	0.122	5.891	1245	WS01034_C12
10	21.864	188	1	0.300	0.047	4.695	1245	WS01034_C12
1	2.788	2	4	-0.284	0.050	4.364	1246	WS0078_A07
1	82.301	16	4	0.304	0.046	4.665	1246	WS0078_A07
4	103.719	81	4	-0.223	0.033	4.412	1246	WS0078_A07
4	173.929	88	2	-0.244	0.050	4.686	1246	WS0078_A07
5	52.820	99	3	-0.299	0.087	7.495	1246	WS0078_A07
1	82.301	16	3	-0.253	0.050	4.896	1247	WS0078_A17
1	95.572	17	3	0.225	0.047	4.551	1247	WS0078_A17
6	25.914	114	1	-0.273	0.066	4.904	1247	WS0078_A17
7	17.610	131	3	-0.300	0.081	4.989	1247	WS0078_A17
11	56.921	219	1	-0.368	0.122	4.536	1247	WS0078_A17
11	57.661	220	4	0.200	0.053	4.772	1247	WS0078_A17
11	64.014	222	4	-0.272	0.057	4.019	1247	WS0078_A17
12	22.441	227	1	0.293	0.059	4.312	1247	WS0078_A17
1	15.589	3	2	-0.238	0.047	4.676	1248	WS0078_C11
2	48.839	29	4	-0.325	0.048	4.616	1248	WS0078_C11
6	69.073	121	3	0.297	0.059	5.347	1248	WS0078_C11
6	89.401	125	2	0.309	0.044	4.087	1248	WS0078_C11
7	74.560	143	2	-0.236	0.038	4.319	1248	WS0078_C11
9	28.608	170	2	0.348	0.077	7.897	1248	WS0078_C11
11	0.000	199	3	0.278	0.042	4.047	1248	WS0078_C11
3	154.505	69	1	0.294	0.057	5.935	1249	WS0078_C21
5	54.072	100	4	0.234	0.038	4.142	1249	WS0078_C21
10	93.347	196	1	0.436	0.083	4.555	1249	WS0078_C21
11	46.026	217	1	-0.210	0.040	4.024	1249	WS0078_C21
11	64.014	222	4	-0.326	0.047	5.176	1249	WS0078_C21
2	120.617	38	4	-0.232	0.041	3.956	1250	WS0078_E16
4	173.929	88	2	-0.253	0.049	4.611	1250	WS0078_E16
6	81.996	123	3	0.435	0.108	7.023	1250	WS0078_E16
9	36.227	165	3	-0.150	0.039	3.910	1250	WS0078_E16
9	59.963	177	4	0.292	0.061	5.324	1250	WS0078_E16
11	37.143	209	3	0.226	0.046	4.600	1250	WS0078_E16
3	9.953	42	3	0.224	0.045	4.421	1251	WS0078_F05
3	137.891	64	3	0.203	0.040	3.992	1251	WS0078_F05
5	54.072	100	4	0.219	0.043	3.887	1251	WS0078_F05
5	57.017	102	4	-0.224	0.051	4.810	1251	WS0078_F05
6	81.996	123	3	0.305	0.103	6.497	1251	WS0078_F05
8	87.463	158	2	-0.241	0.056	5.039	1251	WS0078_F05
11	45.989	216	1	-0.192	0.043	4.046	1251	WS0078_F05
4	116.928	82	4	0.186	0.056	4.518	1252	WS0078_H18

6	81.996	123	3	0.382	0.111	6.623	1252	WS0078_H18
3	137.891	64	3	0.283	0.068	5.347	1253	WS0078_I17
4	0.000	71	4	0.223	0.045	4.157	1253	WS0078_I17
4	173.929	88	2	-0.205	0.041	4.100	1253	WS0078_I17
5	54.072	100	4	0.187	0.042	3.891	1253	WS0078_I17
6	81.996	123	3	0.431	0.163	9.533	1253	WS0078_I17
11	27.405	204	4	-0.297	0.056	5.296	1253	WS0078_I17
3	116.958	63	4	-0.188	0.049	4.528	1254	WS00911_I13
4	103.719	81	4	-0.267	0.052	5.181	1254	WS00911_I13
4	116.928	82	4	0.337	0.092	8.260	1254	WS00911_I13
6	25.914	114	1	-0.293	0.068	6.485	1254	WS00911_I13
8	50.001	154	2	-0.203	0.047	3.880	1254	WS00911_I13
10	21.864	188	3	0.168	0.037	3.952	1254	WS00911_I13
10	93.347	196	2	0.428	0.083	3.955	1254	WS00911_I13
12	38.543	232	4	0.383	0.082	4.149	1254	WS00911_I13
12	51.382	242	1	0.372	0.091	4.568	1254	WS00911_I13
1	62.128	11	1	-0.465	0.086	4.143	1255	WS00911_N22
4	116.928	82	4	0.251	0.045	4.558	1255	WS00911_N22
13	0.000	243	4	0.386	0.148	7.053	1255	WS00911_N22
1	62.128	11	1	-0.463	0.128	4.100	1256	WS00828_A07
8	50.001	154	2	-0.293	0.094	9.960	1256	WS00828_A07
9	55.358	175	1	-0.337	0.056	4.695	1256	WS00828_A07
10	70.246	190	4	-0.327	0.091	8.810	1256	WS00828_A07
10	78.479	193	4	-0.183	0.044	4.531	1256	WS00828_A07
11	17.088	203	1	0.219	0.053	4.266	1256	WS00828_A07
11	27.405	204	4	-0.234	0.035	4.171	1256	WS00828_A07
1	29.800	5	1	-0.367	0.089	7.819	1257	WS01031_C23
1	50.955	10	1	0.167	0.055	4.793	1257	WS01031_C23
8	87.463	158	2	-0.148	0.043	4.460	1257	WS01031_C23
1	15.589	3	2	0.210	0.035	3.938	1258	WS00828_M08
2	101.076	35	4	-0.268	0.049	5.728	1258	WS00828_M08
8	50.001	154	2	-0.287	0.070	7.865	1258	WS00828_M08
11	27.405	204	4	-0.265	0.050	4.986	1258	WS00828_M08
1	82.301	16	4	0.450	0.106	10.714	1259	WS01031_H11
1	95.572	17	3	-0.152	0.049	4.209	1259	WS01031_H11
1	107.333	19	3	-0.360	0.091	7.050	1259	WS01031_H11
1	107.333	19	4	0.282	0.054	6.304	1259	WS01031_H11
3	95.157	59	2	0.229	0.040	4.748	1259	WS01031_H11
5	57.017	102	4	-0.229	0.043	4.828	1259	WS01031_H11
9	55.358	175	1	-0.292	0.063	4.342	1259	WS01031_H11
10	9.580	187	2	0.173	0.052	4.315	1259	WS01031_H11
10	35.204	189	2	-0.161	0.033	4.007	1259	WS01031_H11
3	14.500	43	1	-0.261	0.054	4.602	1260	WS00825_C16
3	17.158	45	1	0.286	0.087	7.390	1260	WS00825_C16
7	73.169	142	1	0.619	0.127	6.284	1260	WS00825_C16
9	81.073	183	1	0.284	0.049	4.161	1260	WS00825_C16
8	95.937	159	3	0.205	0.047	5.093	1261	WS00825_D07
9	32.356	171	2	-0.206	0.033	4.103	1261	WS00825_D07
6	25.914	114	1	-0.257	0.077	6.999	1262	WS00825_F12
7	70.278	141	3	-0.296	0.075	7.551	1262	WS00825_F12
10	9.580	187	3	-0.450	0.163	7.540	1262	WS00825_F12
10	80.088	194	1	-0.288	0.062	5.578	1262	WS00825_F12
10	93.347	196	1	0.527	0.126	6.680	1262	WS00825_F12
11	45.989	216	1	-0.239	0.046	4.159	1262	WS00825_F12

11	57.661	220	4	0.269	0.046	4.183	1262	WS00825_F12
12	0.000	233	1	-0.199	0.055	4.927	1262	WS00825_F12
5	52.820	99	3	-0.244	0.058	4.678	1263	WS00825_G03
5	54.072	100	4	0.258	0.056	5.960	1263	WS00825_G03
6	49.100	118	2	-0.349	0.147	5.495	1263	WS00825_G03
6	69.073	121	3	0.239	0.048	3.967	1263	WS00825_G03
7	70.278	141	3	-0.236	0.063	5.149	1263	WS00825_G03
8	39.090	152	4	-0.226	0.040	4.288	1263	WS00825_G03
10	9.580	187	3	-0.436	0.175	6.485	1263	WS00825_G03
10	77.676	192	1	-0.212	0.068	4.840	1263	WS00825_G03
10	80.088	194	1	-0.360	0.130	9.273	1263	WS00825_G03
10	93.347	196	1	0.463	0.116	5.150	1263	WS00825_G03
11	64.014	222	4	-0.252	0.042	3.942	1263	WS00825_G03
13	0.000	243	4	0.353	0.142	3.842	1263	WS00825_G03
14	9.923	247	1	0.200	0.054	3.866	1263	WS00825_G03
2	127.147	40	4	0.141	0.036	4.053	1264	WS00825_I10
3	141.443	65	4	0.299	0.082	5.464	1264	WS00825_I10
6	89.401	125	1	-0.322	0.054	5.857	1264	WS00825_I10
7	75.883	144	2	0.518	0.136	6.999	1264	WS00825_I10
9	0.000	166	1	-0.143	0.041	4.416	1264	WS00825_I10
9	28.608	170	3	0.470	0.129	9.223	1264	WS00825_I10
9	59.963	177	4	-0.275	0.050	3.883	1264	WS00825_I10
10	9.580	187	3	-0.408	0.094	5.440	1264	WS00825_I10
10	21.864	188	1	0.305	0.051	5.631	1264	WS00825_I10
10	93.347	196	1	0.476	0.100	5.432	1264	WS00825_I10
11	40.960	210	2	-0.404	0.090	4.527	1264	WS00825_I10
12	6.242	234	4	0.307	0.062	6.077	1264	WS00825_I10
1	38.326	6	4	-0.373	0.085	7.365	1265	WS00825_I23
1	40.464	7	4	0.383	0.083	6.826	1265	WS00825_I23
3	83.563	56	3	-0.218	0.039	3.958	1265	WS00825_I23
7	75.883	144	2	0.432	0.114	4.679	1265	WS00825_I23
9	3.127	168	1	-0.315	0.077	5.256	1265	WS00825_I23
10	9.580	187	3	-0.394	0.130	6.851	1265	WS00825_I23
10	93.347	196	1	0.475	0.105	5.424	1265	WS00825_I23
1	62.128	11	1	-0.579	0.105	6.573	1266	WS00825_K20
1	107.333	19	4	-0.284	0.073	6.725	1266	WS00825_K20
6	25.914	114	1	-0.275	0.073	7.660	1266	WS00825_K20
7	75.883	144	2	0.448	0.118	5.138	1266	WS00825_K20
10	70.246	190	4	-0.325	0.053	4.894	1266	WS00825_K20
10	78.479	193	4	-0.301	0.064	4.916	1266	WS00825_K20
10	80.088	194	1	-0.250	0.037	3.867	1266	WS00825_K20
11	43.491	214	3	-0.216	0.052	4.394	1266	WS00825_K20
1	45.794	9	3	-0.179	0.038	4.227	1267	WS00825_M02
6	26.350	115	2	0.181	0.035	3.876	1267	WS00825_M02
6	106.702	128	2	0.412	0.064	4.916	1267	WS00825_M02
9	3.127	168	1	-0.326	0.065	4.647	1267	WS00825_M02
1	38.326	6	4	-0.191	0.044	5.282	1268	WS0262_A18
1	40.464	7	4	0.177	0.037	3.929	1268	WS0262_A18
4	0.000	71	4	-0.317	0.083	8.417	1268	WS0262_A18
4	116.928	82	2	0.290	0.041	5.158	1268	WS0262_A18
6	76.376	122	1	0.297	0.133	5.080	1268	WS0262_A18
6	87.891	124	2	0.238	0.047	5.799	1268	WS0262_A18
6	89.401	125	2	0.349	0.070	4.914	1268	WS0262_A18
6	106.702	128	2	-0.456	0.085	5.603	1268	WS0262_A18

8	50.001	154	2	-0.276	0.072	9.020	1268	WS0262_A18
8	87.463	158	2	0.352	0.105	12.530	1268	WS0262_A18
11	27.405	204	1	-0.237	0.058	3.964	1268	WS0262_A18
13	1.815	244	2	-0.305	0.038	4.761	1268	WS0262_A18
14	22.402	248	3	-0.197	0.057	5.003	1268	WS0262_A18
1	29.800	5	1	0.181	0.037	4.028	1269	WS0262_B09
3	14.500	43	2	0.206	0.062	5.478	1269	WS0262_B09
3	15.147	44	2	-0.333	0.118	5.771	1269	WS0262_B09
3	20.512	46	2	-0.242	0.064	5.094	1269	WS0262_B09
4	6.032	72	4	-0.181	0.058	4.470	1269	WS0262_B09
4	142.917	85	1	-0.250	0.054	5.890	1269	WS0262_B09
5	0.000	89	3	-0.160	0.034	4.587	1269	WS0262_B09
9	28.608	170	3	0.320	0.048	5.020	1269	WS0262_B09
10	80.088	194	1	-0.155	0.036	3.879	1269	WS0262_B09
10	93.347	196	1	0.634	0.132	10.703	1269	WS0262_B09
11	17.088	203	1	-0.266	0.051	5.520	1269	WS0262_B09
11	45.989	216	1	-0.225	0.053	5.669	1269	WS0262_B09
11	64.014	222	4	0.274	0.099	7.460	1269	WS0262_B09
12	22.441	227	3	-0.289	0.050	5.641	1269	WS0262_B09
12	22.629	237	1	0.278	0.074	8.017	1269	WS0262_B09
12	32.473	238	1	0.335	0.102	10.987	1269	WS0262_B09
12	51.382	242	1	-0.460	0.196	5.345	1269	WS0262_B09
14	43.392	250	1	0.330	0.054	4.411	1269	WS0262_B09
14	44.898	251	3	-0.246	0.037	4.240	1269	WS0262_B09
3	9.953	42	3	0.283	0.084	8.091	1270	WS0262_D07
3	83.563	56	3	-0.233	0.051	4.909	1270	WS0262_D07
4	173.929	88	2	-0.215	0.060	7.296	1270	WS0262_D07
6	81.996	123	3	0.336	0.138	6.494	1270	WS0262_D07
9	28.608	170	3	0.236	0.049	4.824	1270	WS0262_D07
11	27.405	204	4	-0.370	0.076	4.697	1270	WS0262_D07
11	37.143	209	3	0.219	0.053	5.142	1270	WS0262_D07
11	60.464	221	2	0.263	0.052	6.239	1270	WS0262_D07
1	38.326	6	2	-0.200	0.041	4.149	1271	WS0262_D19
4	103.719	81	4	0.199	0.066	6.335	1271	WS0262_D19
4	124.132	83	1	-0.578	0.203	6.686	1271	WS0262_D19
5	0.000	89	1	-0.317	0.041	3.891	1271	WS0262_D19
5	7.672	92	4	0.199	0.041	4.095	1271	WS0262_D19
5	16.219	93	1	-0.291	0.113	10.196	1271	WS0262_D19
5	31.191	95	1	-0.271	0.064	5.778	1271	WS0262_D19
5	42.979	97	2	-0.219	0.041	4.193	1271	WS0262_D19
6	25.742	113	3	-0.179	0.054	4.937	1271	WS0262_D19
6	69.073	121	3	0.316	0.082	7.480	1271	WS0262_D19
6	81.996	123	3	0.453	0.150	8.750	1271	WS0262_D19
11	17.088	203	1	0.259	0.075	6.799	1271	WS0262_D19
11	29.069	205	1	-0.298	0.054	5.071	1271	WS0262_D19
5	0.000	89	1	-0.341	0.082	6.540	1272	WS0262_F02
6	87.891	124	2	0.214	0.035	3.947	1272	WS0262_F02
6	89.401	125	1	0.367	0.085	6.591	1272	WS0262_F02
7	105.477	147	2	0.320	0.094	6.438	1272	WS0262_F02
8	11.974	150	1	0.209	0.056	4.266	1272	WS0262_F02
9	0.000	166	1	0.183	0.060	4.586	1272	WS0262_F02
11	64.014	222	4	-0.279	0.061	5.020	1272	WS0262_F02
13	0.000	243	4	0.292	0.138	5.310	1272	WS0262_F02
3	9.953	42	3	0.200	0.039	4.098	1273	WS0262_F12

5	57.017	102	4	-0.173	0.050	3.958	1273	WS0262_F12
9	55.358	175	3	0.405	0.088	5.021	1273	WS0262_F12
1	133.992	24	2	-0.229	0.045	5.693	1274	WS0262_H04
3	80.211	55	4	-0.195	0.038	4.052	1274	WS0262_H04
3	137.891	64	3	0.373	0.105	8.664	1274	WS0262_H04
4	173.929	88	2	-0.193	0.040	5.110	1274	WS0262_H04
9	49.013	173	2	0.277	0.064	7.854	1274	WS0262_H04
10	78.479	193	3	0.326	0.067	5.459	1274	WS0262_H04
12	6.242	234	4	0.327	0.053	5.747	1274	WS0262_H04
3	137.891	64	3	0.228	0.051	4.166	1275	WS0262_H18
4	0.000	71	4	0.234	0.041	4.165	1275	WS0262_H18
4	173.929	88	2	-0.254	0.058	6.982	1275	WS0262_H18
5	57.017	102	4	-0.195	0.038	3.982	1275	WS0262_H18
10	70.246	190	2	-0.210	0.039	4.633	1275	WS0262_H18
12	6.242	234	2	-0.248	0.045	5.602	1275	WS0262_H18
12	6.242	234	4	0.270	0.038	4.022	1275	WS0262_H18
14	0.000	246	2	-0.222	0.057	6.755	1275	WS0262_H18
3	9.953	42	4	0.302	0.119	10.976	1276	WS01030_H10
4	116.928	82	4	0.272	0.098	8.749	1276	WS01030_H10
9	81.073	183	3	-0.246	0.055	5.780	1276	WS01030_H10
14	46.637	252	4	-0.257	0.032	3.895	1276	WS01030_H10
1	29.800	5	1	-0.189	0.056	4.115	1277	WS01030_H20
1	77.177	15	2	0.158	0.035	4.022	1277	WS01030_H20
3	154.505	69	4	0.150	0.038	4.623	1277	WS01030_H20
4	82.462	78	2	0.267	0.045	6.040	1277	WS01030_H20
5	0.000	89	3	0.317	0.083	6.079	1277	WS01030_H20
11	0.000	199	2	-0.275	0.041	4.871	1277	WS01030_H20
12	6.242	234	4	0.243	0.018	4.111	1277	WS01030_H20
4	173.929	88	2	-0.323	0.104	10.835	1278	WS01030_J18
7	73.169	142	1	0.604	0.146	5.850	1278	WS01030_J18
7	75.883	144	2	0.403	0.064	4.468	1278	WS01030_J18
8	50.001	154	2	-0.262	0.086	5.679	1278	WS01030_J18
10	78.479	193	3	0.286	0.048	3.905	1278	WS01030_J18
11	27.405	204	4	-0.278	0.066	5.597	1278	WS01030_J18
13	1.815	244	4	0.282	0.043	4.659	1278	WS01030_J18
14	0.000	246	2	-0.174	0.039	3.973	1278	WS01030_J18
6	69.073	121	4	-0.247	0.056	4.645	1279	WS01030_K09
7	73.169	142	2	-0.442	0.063	4.086	1279	WS01030_K09
8	50.001	154	2	-0.326	0.096	10.332	1279	WS01030_K09
11	27.405	204	4	-0.328	0.062	6.299	1279	WS01030_K09
11	60.464	221	2	0.293	0.059	7.535	1279	WS01030_K09
12	0.000	233	1	-0.273	0.092	5.640	1279	WS01030_K09
3	14.500	43	1	-0.300	0.056	5.935	1280	WS01030_M02
5	54.072	100	4	0.218	0.040	4.257	1280	WS01030_M02
9	36.227	165	3	-0.221	0.062	4.951	1280	WS01030_M02
11	9.237	202	4	0.198	0.039	4.171	1280	WS01030_M02
1	2.788	2	4	-0.250	0.050	6.337	1281	WS01030_M16
6	26.350	115	2	0.200	0.041	5.573	1281	WS01030_M16
11	0.000	199	2	-0.202	0.032	4.412	1281	WS01030_M16
11	9.237	202	4	0.234	0.044	5.653	1281	WS01030_M16
11	27.405	204	4	-0.349	0.081	10.328	1281	WS01030_M16
11	60.464	221	2	0.303	0.060	8.055	1281	WS01030_M16
1	2.788	2	4	-0.157	0.034	4.147	1282	WS01030_O20
4	124.132	83	1	-0.499	0.123	4.987	1282	WS01030_O20

4	142.917	85	1	0.237	0.047	4.096	1282	WS01030_O20
5	54.072	100	4	0.185	0.043	4.466	1282	WS01030_O20
1	2.788	2	4	-0.183	0.039	4.077	1283	WS01030_P18
4	0.000	71	4	0.229	0.041	3.960	1283	WS01030_P18
5	54.072	100	4	0.248	0.054	5.240	1283	WS01030_P18
6	81.996	123	3	0.257	0.082	4.121	1283	WS01030_P18
10	93.347	196	1	0.463	0.101	5.135	1283	WS01030_P18
2	95.880	34	1	-0.279	0.052	4.287	1284	WS00821_N09
2	126.181	39	2	0.323	0.089	9.723	1284	WS00821_N09
2	126.181	39	4	-0.380	0.079	3.997	1284	WS00821_N09
3	9.953	42	3	-0.187	0.036	4.141	1284	WS00821_N09
7	15.555	130	3	0.347	0.074	7.976	1284	WS00821_N09
9	81.073	183	1	-0.217	0.050	4.039	1284	WS00821_N09
10	9.580	187	3	0.347	0.122	5.804	1284	WS00821_N09
11	40.960	210	4	0.329	0.070	4.135	1284	WS00821_N09
11	43.491	214	3	0.194	0.043	4.881	1284	WS00821_N09
11	43.491	214	4	0.249	0.061	4.410	1284	WS00821_N09
11	44.884	215	3	-0.233	0.041	4.663	1284	WS00821_N09
1	45.794	9	1	0.315	0.035	4.002	1285	WS01027_C15
3	14.500	43	1	-0.267	0.051	5.471	1285	WS01027_C15
3	17.158	45	1	0.170	0.050	5.332	1285	WS01027_C15
3	154.505	69	1	0.278	0.057	6.264	1285	WS01027_C15
4	92.039	79	4	-0.369	0.041	5.125	1285	WS01027_C15
5	52.820	99	3	-0.256	0.043	3.935	1285	WS01027_C15
11	9.237	202	4	0.212	0.049	5.293	1285	WS01027_C15
2	101.076	35	4	0.223	0.029	3.892	1286	WS00823_H07
3	14.500	43	1	0.311	0.079	5.787	1286	WS00823_H07
3	17.158	45	1	-0.297	0.128	9.238	1286	WS00823_H07
3	144.593	66	4	0.215	0.053	5.656	1286	WS00823_H07
6	87.891	124	2	0.279	0.062	7.164	1286	WS00823_H07
6	89.401	125	2	0.319	0.085	9.546	1286	WS00823_H07
6	106.702	128	2	-0.474	0.121	5.464	1286	WS00823_H07
7	15.555	130	3	0.311	0.055	4.756	1286	WS00823_H07
7	74.560	143	1	-0.210	0.062	4.455	1286	WS00823_H07
11	0.000	199	2	0.194	0.036	4.170	1286	WS00823_H07
11	9.237	202	4	-0.210	0.042	4.405	1286	WS00823_H07
11	40.960	210	2	-0.391	0.060	4.596	1286	WS00823_H07
11	40.960	210	4	0.245	0.041	4.230	1286	WS00823_H07
3	14.500	43	1	-0.209	0.038	4.078	1287	WS00823_M23
3	17.158	45	1	0.220	0.067	7.160	1287	WS00823_M23
4	97.576	80	2	-0.248	0.048	5.218	1287	WS00823_M23
6	89.401	125	2	0.302	0.064	4.723	1287	WS00823_M23
7	67.154	140	4	-0.197	0.076	4.445	1287	WS00823_M23
7	73.169	142	1	0.495	0.077	4.538	1287	WS00823_M23
7	74.560	143	1	-0.271	0.050	5.345	1287	WS00823_M23
7	75.883	144	2	0.430	0.096	5.501	1287	WS00823_M23
7	75.883	144	4	-0.515	0.110	12.760	1287	WS00823_M23
8	87.463	158	1	-0.256	0.036	3.897	1287	WS00823_M23
8	87.463	158	2	-0.188	0.075	6.126	1287	WS00823_M23
9	3.127	168	1	-0.470	0.080	6.120	1287	WS00823_M23
10	9.580	187	3	-0.345	0.117	5.132	1287	WS00823_M23
10	21.864	188	1	0.364	0.046	4.938	1287	WS00823_M23
11	9.237	202	4	0.215	0.051	5.594	1287	WS00823_M23
3	137.891	64	3	0.316	0.091	6.704	1288	WS00822_M24

6	25.914	114	1	-0.237	0.054	5.243	1288	WS00822_M24
7	15.555	130	3	-0.405	0.124	9.368	1288	WS00822_M24
7	75.883	144	2	0.424	0.070	4.624	1288	WS00822_M24
9	36.227	165	4	0.316	0.058	5.595	1288	WS00822_M24
9	0.000	166	1	-0.226	0.045	4.429	1288	WS00822_M24
11	0.000	199	2	-0.303	0.075	6.848	1288	WS00822_M24
11	37.143	209	3	0.189	0.049	4.093	1288	WS00822_M24
11	45.989	216	1	-0.235	0.050	4.842	1288	WS00822_M24
11	56.921	219	1	-0.354	0.145	5.598	1288	WS00822_M24
12	0.000	233	1	-0.278	0.084	8.127	1288	WS00822_M24
14	9.923	247	1	-0.215	0.045	4.364	1288	WS00822_M24
1	77.177	15	2	0.208	0.045	4.128	1289	WS00823_B07
1	123.316	20	4	0.305	0.082	4.357	1289	WS00823_B07
1	131.686	23	2	-0.535	0.130	5.904	1289	WS00823_B07
6	95.249	126	1	-0.299	0.067	4.758	1289	WS00823_B07
7	54.200	136	2	0.272	0.107	4.190	1289	WS00823_B07
8	78.389	157	1	-0.274	0.057	5.470	1289	WS00823_B07
8	87.463	158	1	0.249	0.047	4.524	1289	WS00823_B07
10	21.864	188	3	0.252	0.065	7.725	1289	WS00823_B07
10	80.088	194	1	-0.321	0.057	5.517	1289	WS00823_B07
1	62.128	11	2	-0.281	0.089	5.008	1290	WS00916_I05
3	69.071	52	1	-0.334	0.083	4.618	1290	WS00916_I05
3	137.891	64	3	0.266	0.050	5.470	1290	WS00916_I05
4	140.962	84	3	-0.260	0.049	5.570	1290	WS00916_I05
4	142.917	85	3	-0.324	0.069	7.315	1290	WS00916_I05
5	3.484	90	2	-0.299	0.091	4.677	1290	WS00916_I05
6	81.996	123	3	0.344	0.085	4.767	1290	WS00916_I05
7	70.278	141	3	-0.214	0.055	6.125	1290	WS00916_I05
10	78.479	193	3	0.385	0.089	9.958	1290	WS00916_I05
10	86.151	195	3	-0.275	0.070	7.892	1290	WS00916_I05
3	9.953	42	4	0.172	0.038	3.874	1291	WS00916_N06
4	0.000	71	4	0.222	0.036	3.924	1291	WS00916_N06
5	54.072	100	4	0.206	0.056	5.970	1291	WS00916_N06
6	81.996	123	3	0.376	0.105	5.040	1291	WS00916_N06
11	27.405	204	4	-0.343	0.079	9.513	1291	WS00916_N06
3	80.211	55	4	-0.185	0.043	4.272	1292	WS00917_M04
4	6.032	72	4	0.208	0.050	4.981	1292	WS00917_M04
5	16.219	93	1	-0.306	0.065	5.578	1292	WS00917_M04
6	49.100	118	2	-0.273	0.117	5.064	1292	WS00917_M04
6	69.073	121	3	0.256	0.073	6.515	1292	WS00917_M04
11	37.143	209	3	0.233	0.054	4.765	1292	WS00917_M04
14	0.000	246	2	-0.165	0.052	5.880	1292	WS00917_M04
1	95.572	17	2	-0.296	0.058	6.618	1293	WS00917_N02
5	42.979	97	1	0.237	0.058	4.334	1293	WS00917_N02
5	56.008	101	1	-0.369	0.117	8.826	1293	WS00917_N02
6	25.914	114	1	-0.234	0.064	4.746	1293	WS00917_N02
7	54.200	136	2	0.293	0.075	4.123	1293	WS00917_N02
11	0.000	199	2	-0.324	0.058	6.504	1293	WS00917_N02
11	0.000	199	3	0.242	0.040	4.132	1293	WS00917_N02
12	6.242	234	4	0.347	0.064	5.905	1293	WS00917_N02
14	43.392	250	1	-0.334	0.113	4.961	1293	WS00917_N02
14	44.898	251	1	0.284	0.061	4.458	1293	WS00917_N02
3	137.891	64	3	0.219	0.049	4.125	1294	WS00917_P09
6	49.100	118	2	-0.252	0.074	4.812	1294	WS00917_P09

3	148.745	68	4	-0.166	0.043	4.571	1295	WS00918_A07
4	116.928	82	4	0.270	0.073	6.327	1295	WS00918_A07
6	49.100	118	4	0.198	0.070	3.891	1295	WS00918_A07
9	3.127	168	1	0.465	0.129	5.972	1295	WS00918_A07
12	22.441	227	1	0.197	0.040	4.561	1295	WS00918_A07
13	0.000	243	4	0.270	0.101	5.155	1295	WS00918_A07
1	29.800	5	1	-0.230	0.076	6.936	1296	WS00918_C04
1	62.128	11	1	-0.605	0.200	6.968	1296	WS00918_C04
3	141.443	65	3	-0.465	0.081	4.974	1296	WS00918_C04
3	141.443	65	4	0.363	0.122	4.584	1296	WS00918_C04
4	103.719	81	4	-0.282	0.042	4.563	1296	WS00918_C04
4	116.928	82	4	0.441	0.143	13.907	1296	WS00918_C04
6	45.897	117	1	0.309	0.040	4.453	1296	WS00918_C04
6	49.100	118	4	0.312	0.141	5.029	1296	WS00918_C04
6	89.401	125	2	0.340	0.057	5.166	1296	WS00918_C04
6	106.702	128	2	-0.408	0.154	4.950	1296	WS00918_C04
7	15.555	130	3	-0.366	0.070	5.620	1296	WS00918_C04
9	81.073	183	3	-0.195	0.052	4.206	1296	WS00918_C04
13	1.815	244	4	0.287	0.068	6.810	1296	WS00918_C04
1	43.578	8	2	0.274	0.042	4.562	1297	WS00918_C24
1	107.333	19	3	0.233	0.049	5.040	1297	WS00918_C24
2	93.504	32	4	0.414	0.127	9.646	1297	WS00918_C24
2	120.617	38	3	0.216	0.045	4.655	1297	WS00918_C24
2	127.147	40	3	-0.233	0.064	6.571	1297	WS00918_C24
2	127.147	40	4	0.210	0.060	5.056	1297	WS00918_C24
3	14.500	43	2	0.218	0.058	6.357	1297	WS00918_C24
3	15.147	44	2	-0.414	0.152	7.906	1297	WS00918_C24
3	20.512	46	2	-0.231	0.054	5.808	1297	WS00918_C24
3	80.211	55	4	-0.256	0.060	5.104	1297	WS00918_C24
3	104.681	60	2	-0.222	0.037	4.056	1297	WS00918_C24
3	114.747	62	2	0.191	0.038	4.060	1297	WS00918_C24
4	0.000	71	4	-0.208	0.059	4.989	1297	WS00918_C24
6	56.188	120	2	0.319	0.072	7.873	1297	WS00918_C24
7	75.883	144	4	-0.265	0.056	4.198	1297	WS00918_C24
9	59.963	177	4	-0.311	0.070	5.345	1297	WS00918_C24
11	17.088	203	1	-0.250	0.051	4.197	1297	WS00918_C24
11	41.984	212	2	-0.228	0.040	4.295	1297	WS00918_C24
11	45.989	216	1	-0.179	0.056	4.529	1297	WS00918_C24
11	45.989	216	2	-0.258	0.045	4.854	1297	WS00918_C24
14	43.392	250	2	0.192	0.052	5.023	1297	WS00918_C24
1	107.333	19	4	-0.251	0.057	4.353	1298	WS00918_F20
3	17.158	45	1	-0.300	0.090	7.575	1298	WS00918_F20
3	137.891	64	1	0.307	0.081	6.730	1298	WS00918_F20
3	137.891	64	3	-0.303	0.066	7.689	1298	WS00918_F20
5	52.820	99	3	-0.250	0.037	4.051	1298	WS00918_F20
9	17.951	169	1	-0.302	0.050	4.225	1298	WS00918_F20
10	70.246	190	2	0.255	0.046	4.842	1298	WS00918_F20
10	93.347	196	2	0.488	0.086	5.334	1298	WS00918_F20
12	22.441	227	1	0.327	0.049	4.172	1298	WS00918_F20
12	6.242	234	2	0.273	0.050	5.207	1298	WS00918_F20
14	43.392	250	2	0.195	0.039	3.976	1298	WS00918_F20
5	7.672	92	1	-0.280	0.070	6.313	1299	WS00918_G17
8	50.001	154	2	-0.243	0.067	6.778	1299	WS00918_G17
10	78.479	193	3	0.337	0.038	5.012	1299	WS00918_G17

13	1.815	244	4	0.259	0.071	4.132	1299	WS00918_G17
1	133.992	24	2	0.127	0.034	4.031	1300	WS00914_D23
3	148.745	68	1	-0.317	0.078	5.728	1300	WS00914_D23
4	0.000	71	4	0.141	0.050	5.059	1300	WS00914_D23
4	173.929	88	2	-0.255	0.038	4.627	1300	WS00914_D23
8	50.001	154	2	-0.139	0.042	3.858	1300	WS00914_D23
9	28.608	170	3	-0.279	0.066	5.533	1300	WS00914_D23
10	78.479	193	3	0.263	0.053	4.769	1300	WS00914_D23
12	14.406	230	4	0.300	0.038	4.489	1300	WS00914_D23
1	45.794	9	1	0.383	0.088	8.470	1301	WS00914_E19
1	45.794	9	3	-0.278	0.068	7.232	1301	WS00914_E19
3	137.891	64	1	-0.316	0.076	7.708	1301	WS00914_E19
3	137.891	64	3	0.278	0.040	3.955	1301	WS00914_E19
4	142.917	85	3	-0.198	0.042	3.924	1301	WS00914_E19
7	74.560	143	1	-0.274	0.075	7.163	1301	WS00914_E19
7	75.883	144	2	0.448	0.107	5.169	1301	WS00914_E19
7	75.883	144	4	-0.292	0.073	6.336	1301	WS00914_E19
12	28.493	228	2	0.413	0.170	7.628	1301	WS00914_E19
14	0.000	246	1	-0.227	0.064	6.036	1301	WS00914_E19
14	22.402	248	3	-0.267	0.044	4.444	1301	WS00914_E19
1	45.794	9	1	0.335	0.057	5.191	1302	WS00914_H05
2	95.880	34	1	-0.299	0.044	3.966	1302	WS00914_H05
5	52.820	99	3	-0.292	0.055	4.798	1302	WS00914_H05
9	36.227	165	3	-0.208	0.040	3.939	1302	WS00914_H05
1	45.794	9	1	0.326	0.047	5.084	1303	WS00914_H21
3	14.500	43	1	-0.310	0.061	6.495	1303	WS00914_H21
3	17.158	45	1	0.243	0.067	7.055	1303	WS00914_H21
4	82.462	78	1	-0.243	0.040	4.253	1303	WS00914_H21
4	173.929	88	2	-0.260	0.068	5.634	1303	WS00914_H21
5	54.072	100	4	0.252	0.046	5.073	1303	WS00914_H21
5	57.017	102	4	-0.274	0.046	5.445	1303	WS00914_H21
10	93.347	196	1	0.454	0.108	4.952	1303	WS00914_H21
11	64.014	222	4	-0.279	0.037	4.120	1303	WS00914_H21
1	45.794	9	1	0.314	0.044	4.036	1304	WS00914_K15
4	173.929	88	2	-0.250	0.069	6.573	1304	WS00914_K15
14	0.000	246	1	-0.235	0.046	4.291	1304	WS00914_K15
1	38.326	6	4	-0.312	0.042	4.504	1305	WS00914_L08
1	40.464	7	4	0.310	0.047	4.537	1305	WS00914_L08
2	120.617	38	4	-0.234	0.049	4.265	1305	WS00914_L08
3	141.443	65	4	0.284	0.085	4.372	1305	WS00914_L08
3	154.505	69	1	0.222	0.042	4.214	1305	WS00914_L08
6	25.914	114	1	-0.219	0.050	4.906	1305	WS00914_L08
8	11.974	150	1	-0.205	0.044	4.315	1305	WS00914_L08
14	0.000	246	1	-0.241	0.042	4.157	1305	WS00914_L08
1	50.955	10	1	-0.156	0.067	3.968	1306	WS00914_N13
3	20.512	46	2	0.257	0.039	5.120	1306	WS00914_N13
4	82.462	78	2	-0.240	0.035	4.348	1306	WS00914_N13
5	16.219	93	2	-0.254	0.032	4.043	1306	WS00914_N13
6	89.401	125	1	-0.184	0.079	4.756	1306	WS00914_N13
11	35.604	207	3	-0.185	0.046	4.091	1306	WS00914_N13
11	37.143	209	3	0.184	0.045	4.021	1306	WS00914_N13
11	43.491	214	4	-0.248	0.058	5.362	1306	WS00914_N13
12	22.441	227	3	0.239	0.052	4.797	1306	WS00914_N13
2	63.261	30	3	0.180	0.054	5.020	1307	WS00914_O02

2	95.880	34	3	0.341	0.073	6.815	1307	WS00914_O02
2	101.076	35	3	-0.334	0.075	7.025	1307	WS00914_O02
2	102.814	36	2	-0.195	0.040	3.955	1307	WS00914_O02
4	173.929	88	2	0.223	0.044	5.139	1307	WS00914_O02
5	57.017	102	2	-0.265	0.043	5.167	1307	WS00914_O02
6	45.897	117	1	-0.229	0.068	4.845	1307	WS00914_O02
6	49.100	118	2	0.422	0.200	10.049	1307	WS00914_O02
6	56.188	120	2	0.265	0.039	4.542	1307	WS00914_O02
9	69.046	181	2	-0.234	0.086	4.427	1307	WS00914_O02
10	80.088	194	2	0.232	0.047	5.246	1307	WS00914_O02
10	93.347	196	2	-0.463	0.098	4.425	1307	WS00914_O02
11	37.143	209	3	-0.163	0.048	4.536	1307	WS00914_O02
11	46.026	217	1	0.236	0.058	4.141	1307	WS00914_O02
11	48.979	218	4	0.186	0.047	4.170	1307	WS00914_O02
12	22.441	227	1	0.303	0.113	8.498	1307	WS00914_O02
12	6.242	234	4	0.324	0.049	4.986	1307	WS00914_O02
14	22.402	248	4	-0.361	0.057	5.348	1307	WS00914_O02
2	102.814	36	2	-0.196	0.040	5.518	1308	WS00919_I12
2	120.617	38	4	-0.396	0.069	6.750	1308	WS00919_I12
2	126.181	39	2	0.305	0.054	5.828	1308	WS00919_I12
2	127.147	40	4	-0.209	0.081	11.487	1308	WS00919_I12
3	15.147	44	2	0.418	0.057	5.656	1308	WS00919_I12
5	7.672	92	4	0.248	0.035	6.054	1308	WS00919_I12
6	45.897	117	4	-0.185	0.019	4.000	1308	WS00919_I12
6	76.376	122	1	0.294	0.108	4.537	1308	WS00919_I12
9	36.227	165	4	0.192	0.033	4.400	1308	WS00919_I12
10	70.246	190	4	-0.260	0.047	5.864	1308	WS00919_I12
11	46.026	217	1	0.230	0.098	7.458	1308	WS00919_I12
11	48.979	218	2	0.198	0.042	4.637	1308	WS00919_I12
6	81.996	123	3	0.373	0.088	5.223	1309	WS00919_J05
11	27.405	204	4	-0.233	0.045	4.577	1309	WS00919_J05
3	137.891	64	3	0.232	0.053	4.512	1310	WS00919_L11
4	0.000	71	4	0.253	0.050	4.962	1310	WS00919_L11
4	173.929	88	2	-0.233	0.055	6.233	1310	WS00919_L11
5	54.072	100	4	0.247	0.062	6.082	1310	WS00919_L11
6	81.996	123	3	0.316	0.107	5.217	1310	WS00919_L11
11	27.405	204	4	-0.378	0.088	6.625	1310	WS00919_L11
1	69.854	13	2	0.258	0.061	6.627	1311	WS00919_L24
1	71.592	14	2	0.250	0.057	6.211	1311	WS00919_L24
1	77.177	15	2	0.263	0.063	6.902	1311	WS00919_L24
1	131.686	23	2	-0.455	0.074	5.421	1311	WS00919_L24
5	54.072	100	4	0.255	0.051	5.452	1311	WS00919_L24
11	27.405	204	4	-0.389	0.083	7.042	1311	WS00919_L24
1	95.572	17	2	-0.226	0.053	6.083	1312	WS00919_O12
2	48.839	29	1	-0.213	0.053	3.973	1312	WS00919_O12
3	137.891	64	3	0.304	0.056	3.977	1312	WS00919_O12
11	43.491	214	3	-0.166	0.049	3.920	1312	WS00919_O12
12	6.242	234	4	0.326	0.043	4.263	1312	WS00919_O12
1	22.419	4	3	-0.368	0.083	4.877	1313	WS00919_P16
6	69.073	121	3	0.279	0.044	4.288	1313	WS00919_P16
11	17.088	203	1	0.194	0.039	3.878	1313	WS00919_P16
5	54.072	100	4	0.212	0.045	5.369	1314	WS00920_C09
5	57.017	102	4	-0.219	0.038	4.685	1314	WS00920_C09
7	75.883	144	2	0.371	0.039	3.899	1314	WS00920_C09

11	27.405	204	4	-0.305	0.063	7.731	1314	WS00920_C09
3	17.158	45	1	0.196	0.056	4.019	1315	WS00920_D03
3	144.593	66	4	0.255	0.060	6.726	1315	WS00920_D03
3	148.745	68	4	-0.306	0.061	5.487	1315	WS00920_D03
3	154.505	69	4	0.252	0.039	4.037	1315	WS00920_D03
7	75.883	144	2	0.418	0.055	4.576	1315	WS00920_D03
11	27.405	204	4	-0.334	0.069	7.865	1315	WS00920_D03
7	75.883	144	2	0.450	0.074	5.477	1316	WS0054_N13
1	69.854	13	2	0.200	0.038	4.012	1317	WS0054_O07
3	137.891	64	1	-0.224	0.050	4.120	1317	WS0054_O07
3	137.891	64	3	0.248	0.074	7.093	1317	WS0054_O07
4	116.928	82	2	-0.376	0.061	6.591	1317	WS0054_O07
4	124.132	83	2	-0.372	0.102	4.418	1317	WS0054_O07
5	16.219	93	1	-0.195	0.046	3.882	1317	WS0054_O07
5	54.072	100	4	0.250	0.043	4.009	1317	WS0054_O07
7	70.278	141	3	-0.309	0.077	7.498	1317	WS0054_O07
8	87.463	158	2	-0.224	0.039	4.268	1317	WS0054_O07
10	104.214	198	4	-0.200	0.044	5.361	1317	WS0054_O07
3	154.505	69	1	0.277	0.045	3.912	1318	WS0261_A24
5	42.979	97	1	0.213	0.046	4.151	1318	WS0261_A24
5	56.008	101	1	-0.187	0.062	5.287	1318	WS0261_A24
5	57.017	102	4	-0.214	0.046	4.532	1318	WS0261_A24
5	84.347	105	1	-0.370	0.211	17.862	1318	WS0261_A24
6	95.249	126	1	0.347	0.081	4.383	1318	WS0261_A24
10	70.246	190	2	-0.294	0.088	9.252	1318	WS0261_A24
11	64.014	222	4	-0.223	0.050	4.759	1318	WS0261_A24
12	0.000	233	1	-0.299	0.057	5.037	1318	WS0261_A24
12	22.629	237	1	-0.297	0.051	4.566	1318	WS0261_A24
2	127.147	40	3	-0.241	0.053	5.505	1319	WS0261_B16
3	47.928	49	1	-0.238	0.053	4.852	1319	WS0261_B16
3	137.891	64	3	0.387	0.086	5.793	1319	WS0261_B16
6	106.702	128	2	0.471	0.116	5.483	1319	WS0261_B16
10	21.864	188	1	0.343	0.077	7.105	1319	WS0261_B16
1	45.794	9	1	0.287	0.065	5.192	1320	WS0261_D10
5	52.820	99	1	0.327	0.065	5.235	1320	WS0261_D10
5	57.017	102	4	-0.222	0.035	4.759	1320	WS0261_D10
5	84.347	105	1	-0.233	0.060	4.631	1320	WS0261_D10
7	75.883	144	2	0.507	0.094	7.535	1320	WS0261_D10
8	87.463	158	2	-0.252	0.057	6.338	1320	WS0261_D10
1	45.794	9	1	0.210	0.054	4.288	1321	WS0261_D18
4	82.462	78	2	0.174	0.037	4.148	1321	WS0261_D18
11	64.014	222	3	0.188	0.040	3.924	1321	WS0261_D18
4	82.462	78	2	0.187	0.039	4.476	1322	WS0261_E24
6	49.100	118	4	0.511	0.264	9.438	1322	WS0261_E24
6	54.066	119	4	0.235	0.037	4.193	1322	WS0261_E24
9	59.963	177	4	-0.228	0.040	5.020	1322	WS0261_E24
11	27.405	204	1	-0.320	0.062	4.994	1322	WS0261_E24
11	27.405	204	4	0.356	0.076	8.743	1322	WS0261_E24
2	126.181	39	4	-0.364	0.060	4.368	1323	WS0261_F13
4	173.929	88	2	-0.191	0.034	3.888	1323	WS0261_F13
6	81.996	123	4	-0.326	0.127	4.966	1323	WS0261_F13
13	1.815	244	4	0.256	0.040	4.142	1323	WS0261_F13
1	45.794	9	1	0.307	0.045	4.074	1324	WS0101_E10
1	50.955	10	1	-0.245	0.047	4.203	1324	WS0101_E10

1	82.301	16	3	-0.304	0.044	4.814	1324	WS0101_E10
1	95.572	17	3	0.278	0.061	6.615	1324	WS0101_E10
2	93.504	32	2	-0.150	0.040	3.989	1324	WS0101_E10
3	154.505	69	1	0.214	0.055	5.104	1324	WS0101_E10
4	0.000	71	4	-0.314	0.068	6.095	1324	WS0101_E10
7	15.555	130	2	0.268	0.038	3.874	1324	WS0101_E10
11	35.604	207	3	-0.237	0.042	4.292	1324	WS0101_E10
11	43.491	214	3	0.244	0.053	5.421	1324	WS0101_E10
11	44.884	215	3	-0.224	0.040	4.106	1324	WS0101_E10
12	14.406	230	3	0.285	0.057	5.833	1324	WS0101_E10
1	22.419	4	3	-0.309	0.087	5.613	1325	WS0101_J12
1	38.326	6	4	-0.302	0.049	4.884	1325	WS0101_J12
1	40.464	7	4	0.319	0.053	5.252	1325	WS0101_J12
5	54.072	100	4	0.211	0.048	4.764	1325	WS0101_J12
9	49.013	173	2	-0.191	0.052	4.232	1325	WS0101_J12
3	9.953	42	3	0.242	0.061	5.150	1326	WS0079_I19
4	173.929	88	2	-0.202	0.051	5.610	1326	WS0079_I19
5	54.072	100	4	0.240	0.042	4.333	1326	WS0079_I19
6	81.996	123	3	0.236	0.088	3.884	1326	WS0079_I19
8	50.001	154	1	-0.173	0.043	3.891	1326	WS0079_I19
10	78.479	193	3	0.249	0.052	4.450	1326	WS0079_I19
11	64.014	222	4	-0.275	0.047	5.036	1326	WS0079_I19
12	6.242	234	2	-0.259	0.051	5.975	1326	WS0079_I19
12	6.242	234	4	0.257	0.038	3.972	1326	WS0079_I19
14	0.000	246	2	-0.224	0.045	4.665	1326	WS0079_I19
6	69.073	121	3	0.219	0.052	4.709	1327	WS00938_H22
6	81.996	123	3	0.389	0.148	6.468	1327	WS00938_H22
10	78.479	193	3	0.235	0.046	4.120	1327	WS00938_H22
12	6.242	234	2	-0.313	0.074	8.471	1327	WS00938_H22
14	0.000	246	2	-0.175	0.043	4.983	1327	WS00938_H22
3	9.953	42	3	0.357	0.087	9.867	1328	WS0079_P06
3	144.593	66	4	0.200	0.052	3.929	1328	WS0079_P06
6	81.996	123	3	0.440	0.152	8.336	1328	WS0079_P06
8	95.937	159	3	-0.188	0.034	3.843	1328	WS0079_P06
2	127.147	40	4	-0.233	0.059	5.318	1329	WS00939_B16
4	0.000	71	4	0.249	0.063	6.406	1329	WS00939_B16
4	173.929	88	2	-0.266	0.050	5.578	1329	WS00939_B16
5	54.072	100	4	0.252	0.070	7.149	1329	WS00939_B16
5	57.017	102	4	-0.251	0.052	5.106	1329	WS00939_B16
6	69.073	121	3	0.175	0.051	4.362	1329	WS00939_B16
6	81.996	123	3	0.459	0.148	7.593	1329	WS00939_B16
6	89.401	125	1	0.225	0.050	3.880	1329	WS00939_B16
10	70.246	190	2	-0.173	0.035	3.906	1329	WS00939_B16
11	27.405	204	4	-0.311	0.065	7.865	1329	WS00939_B16
3	15.147	44	2	0.222	0.053	3.948	1330	WS0261_G24
3	137.891	64	3	0.212	0.052	4.272	1330	WS0261_G24
4	0.000	71	4	0.269	0.054	5.470	1330	WS0261_G24
6	81.996	123	3	0.370	0.143	6.752	1330	WS0261_G24
11	27.405	204	4	-0.353	0.064	5.233	1330	WS0261_G24
1	45.794	9	3	0.296	0.084	4.669	1331	WS0261_H12
4	0.000	71	4	0.245	0.043	4.478	1331	WS0261_H12
7	17.610	131	3	-0.312	0.093	4.473	1331	WS0261_H12
10	104.214	198	2	-0.172	0.031	4.836	1331	WS0261_H12
1	131.686	23	2	-0.400	0.060	3.899	1332	WS0261_J08

6	69.073	121	4	-0.231	0.045	4.552	1332	WS0261_J08
11	0.000	199	2	-0.214	0.043	4.231	1332	WS0261_J08
11	27.405	204	4	-0.342	0.067	5.616	1332	WS0261_J08
1	95.572	17	3	0.233	0.048	4.205	1333	WS0261_J16
1	131.686	23	2	-0.504	0.088	8.118	1333	WS0261_J16
10	80.088	194	1	-0.249	0.055	3.935	1333	WS0261_J16
11	0.000	199	2	-0.220	0.059	6.786	1333	WS0261_J16
12	0.000	233	1	-0.254	0.065	4.706	1333	WS0261_J16
4	173.929	88	2	-0.194	0.037	4.038	1334	WS0261_K22
5	54.072	100	4	0.200	0.037	3.881	1334	WS0261_K22
6	81.996	123	3	0.347	0.127	6.281	1334	WS0261_K22
11	27.405	204	4	-0.345	0.067	7.071	1334	WS0261_K22
5	54.072	100	4	0.228	0.048	4.751	1335	WS0261_L18
5	57.017	102	4	-0.223	0.043	4.248	1335	WS0261_L18
6	81.996	123	3	0.386	0.117	6.973	1335	WS0261_L18
11	27.405	204	4	-0.294	0.051	4.796	1335	WS0261_L18
4	173.929	88	2	-0.213	0.045	4.358	1336	WS0261_N03
6	81.996	123	3	0.339	0.092	5.254	1336	WS0261_N03
11	27.405	204	4	-0.248	0.041	4.299	1336	WS0261_N03
8	50.001	154	2	-0.187	0.045	4.232	1337	WS0261_N17
10	80.088	194	1	-0.270	0.047	4.102	1337	WS0261_N17
11	0.000	199	2	-0.254	0.055	5.616	1337	WS0261_N17
11	0.000	199	3	0.268	0.044	4.054	1337	WS0261_N17
5	54.072	100	4	0.307	0.087	8.902	1338	WS00920_F10
10	80.088	194	1	-0.291	0.053	4.964	1338	WS00920_F10
12	0.000	233	1	-0.226	0.057	5.302	1338	WS00920_F10
14	0.000	246	1	-0.225	0.042	3.926	1338	WS00920_F10
1	2.788	2	3	0.158	0.041	4.546	1339	WS00920_G05
1	22.419	4	3	-0.365	0.061	4.694	1339	WS00920_G05
5	52.820	99	3	-0.220	0.044	4.864	1339	WS00920_G05
6	81.996	123	3	0.337	0.058	4.436	1339	WS00920_G05
7	75.883	144	2	-0.450	0.111	5.783	1339	WS00920_G05
8	50.001	154	2	-0.215	0.073	6.096	1339	WS00920_G05
10	21.864	188	3	0.212	0.040	4.865	1339	WS00920_G05
11	17.088	203	1	0.270	0.056	5.689	1339	WS00920_G05
12	22.441	227	3	0.191	0.037	4.658	1339	WS00920_G05
12	22.629	237	1	-0.184	0.038	3.871	1339	WS00920_G05
1	62.128	11	1	-0.498	0.148	4.720	1340	WS00920_I14
3	80.211	55	4	-0.162	0.050	4.240	1340	WS00920_I14
3	141.443	65	3	-0.333	0.079	5.090	1340	WS00920_I14
3	144.647	67	1	0.264	0.124	3.942	1340	WS00920_I14
6	45.897	117	1	0.251	0.039	3.903	1340	WS00920_I14
8	50.001	154	2	-0.284	0.110	9.985	1340	WS00920_I14
8	87.463	158	2	0.289	0.082	7.511	1340	WS00920_I14
9	3.127	168	1	0.382	0.128	4.232	1340	WS00920_I14
9	17.951	169	1	-0.237	0.050	4.849	1340	WS00920_I14
9	17.951	169	2	0.344	0.074	7.391	1340	WS00920_I14
11	0.000	199	3	0.302	0.052	3.893	1340	WS00920_I14
11	35.604	207	3	-0.269	0.055	5.739	1340	WS00920_I14
11	37.143	209	3	0.280	0.061	6.294	1340	WS00920_I14
12	14.406	230	3	0.314	0.057	5.928	1340	WS00920_I14
14	22.402	248	3	-0.215	0.047	4.834	1340	WS00920_I14
1	2.788	2	4	-0.118	0.030	4.152	1341	WS00920_J02
1	62.128	11	1	-0.535	0.136	5.468	1341	WS00920_J02

5	3.484	90	2	0.286	0.048	4.159	1341	WS00920_J02
5	42.979	97	2	-0.182	0.041	5.018	1341	WS00920_J02
8	50.001	154	2	-0.263	0.051	5.287	1341	WS00920_J02
8	87.463	158	2	0.182	0.048	5.631	1341	WS00920_J02
11	46.026	217	1	-0.217	0.085	6.420	1341	WS00920_J02
7	54.200	136	2	0.336	0.155	5.619	1342	WS00920_L01
9	0.000	163	1	-0.554	0.119	7.277	1342	WS00920_L01
10	77.676	192	1	-0.256	0.054	4.482	1342	WS00920_L01
10	80.088	194	1	-0.367	0.092	7.534	1342	WS00920_L01
12	14.406	230	3	0.345	0.093	9.040	1342	WS00920_L01
14	0.000	246	1	-0.235	0.076	6.190	1342	WS00920_L01
1	43.578	8	3	-0.198	0.043	4.205	1343	WS00920_L12
4	82.462	78	2	-0.306	0.092	8.263	1343	WS00920_L12
4	92.039	79	2	-0.307	0.052	5.375	1343	WS00920_L12
4	97.576	80	2	0.185	0.049	5.461	1343	WS00920_L12
4	116.928	82	2	0.183	0.041	4.250	1343	WS00920_L12
1	2.788	2	4	-0.214	0.044	4.265	1344	WS00920_N12
10	93.347	196	1	0.401	0.075	3.867	1344	WS00920_N12
14	0.000	246	1	-0.311	0.080	6.798	1344	WS00920_N12
2	95.880	34	1	-0.306	0.051	4.967	1345	WS00920_O06
3	14.500	43	1	-0.264	0.057	5.579	1345	WS00920_O06
3	17.158	45	1	0.185	0.042	4.121	1345	WS00920_O06
5	54.072	100	4	0.248	0.050	5.032	1345	WS00920_O06
5	57.017	102	4	-0.327	0.060	4.685	1345	WS00920_O06
9	3.127	168	1	-0.341	0.047	3.877	1345	WS00920_O06
11	9.237	202	4	0.199	0.047	4.748	1345	WS00920_O06
14	0.000	246	1	-0.276	0.053	5.273	1345	WS00920_O06
5	54.072	100	4	0.274	0.061	6.319	1346	WS02611_P21
10	93.347	196	1	0.523	0.119	6.575	1346	WS02611_P21
6	81.996	123	3	0.377	0.139	7.832	1347	WS02612_O22
11	27.405	204	4	-0.244	0.047	4.017	1347	WS02612_O22
2	93.504	32	4	0.278	0.054	6.520	1348	WS0261_P24
4	173.929	88	2	-0.146	0.038	3.937	1348	WS0261_P24
8	11.974	150	1	0.211	0.070	5.765	1348	WS0261_P24
9	28.608	170	3	0.307	0.082	4.746	1348	WS0261_P24
9	61.113	178	1	-0.363	0.103	4.098	1348	WS0261_P24
5	52.820	99	1	0.298	0.051	5.435	1349	WS00928_A05
5	52.820	99	3	-0.295	0.054	3.991	1349	WS00928_A05
5	54.072	100	4	0.214	0.039	4.565	1349	WS00928_A05
5	57.017	102	4	-0.282	0.049	6.161	1349	WS00928_A05
8	62.580	155	1	-0.400	0.121	7.290	1349	WS00928_A05
1	107.333	19	3	0.258	0.058	5.919	1350	WS0091_E24
2	48.839	29	4	0.224	0.049	3.981	1350	WS0091_E24
5	42.979	97	1	-0.242	0.047	4.682	1350	WS0091_E24
5	86.669	106	2	-0.224	0.108	4.101	1350	WS0091_E24
6	81.996	123	3	0.289	0.070	4.417	1350	WS0091_E24
10	9.580	187	3	-0.317	0.103	6.585	1350	WS0091_E24
11	9.237	202	4	0.191	0.047	4.009	1350	WS0091_E24
11	27.405	204	4	-0.300	0.065	5.674	1350	WS0091_E24
2	102.814	36	2	0.223	0.046	3.849	1351	WS00928_D23
2	126.181	39	2	-0.274	0.049	4.922	1351	WS00928_D23
3	9.953	42	3	0.308	0.064	6.363	1351	WS00928_D23
6	81.996	123	3	0.341	0.083	4.192	1351	WS00928_D23
9	28.608	170	2	0.243	0.039	3.865	1351	WS00928_D23

4	0.000	71	4	0.286	0.057	6.444	1352	WS0092_M11
4	173.929	88	2	-0.193	0.038	4.165	1352	WS0092_M11
5	54.072	100	4	0.238	0.054	6.097	1352	WS0092_M11
6	81.996	123	3	0.338	0.126	5.712	1352	WS0092_M11
11	27.405	204	4	-0.344	0.064	6.492	1352	WS0092_M11
6	81.996	123	3	0.462	0.174	8.751	1353	WS0092_N02
11	27.405	204	4	-0.299	0.055	6.425	1353	WS0092_N02
3	17.158	45	1	0.218	0.055	4.694	1354	WS0092_O13
3	148.745	68	4	-0.225	0.040	4.756	1354	WS0092_O13
4	92.039	79	2	-0.273	0.057	6.088	1354	WS0092_O13
7	62.787	139	2	0.220	0.046	3.960	1354	WS0092_O13
8	87.463	158	1	-0.256	0.063	5.386	1354	WS0092_O13
11	27.405	204	4	-0.331	0.082	9.178	1354	WS0092_O13
12	6.242	234	2	-0.231	0.038	5.046	1354	WS0092_O13
14	0.000	246	1	-0.247	0.048	4.072	1354	WS0092_O13
14	0.000	246	2	-0.168	0.038	3.959	1354	WS0092_O13
4	92.039	79	4	-0.293	0.036	3.897	1355	WS0092_P08
6	81.996	123	3	0.284	0.060	3.849	1355	WS0092_P08
6	81.996	123	4	-0.248	0.066	4.038	1355	WS0092_P08
13	0.000	243	4	0.417	0.105	6.389	1355	WS0092_P08
2	48.839	29	4	0.275	0.042	4.781	1356	WS0093_C01
5	54.072	100	4	0.194	0.048	5.165	1356	WS0093_C01
6	45.897	117	1	-0.222	0.054	4.784	1356	WS0093_C01
6	45.897	117	4	-0.191	0.037	3.865	1356	WS0093_C01
6	56.188	120	1	-0.197	0.048	4.422	1356	WS0093_C01
6	76.376	122	1	0.282	0.149	4.922	1356	WS0093_C01
6	81.996	123	3	0.327	0.086	5.075	1356	WS0093_C01
7	15.555	130	3	-0.299	0.061	4.781	1356	WS0093_C01
9	36.227	165	4	0.205	0.039	3.986	1356	WS0093_C01
3	14.500	43	1	-0.246	0.043	4.311	1357	WS0093_C09
3	17.158	45	1	0.198	0.047	4.732	1357	WS0093_C09
5	54.072	100	4	0.343	0.116	11.198	1357	WS0093_C09
6	25.914	114	1	-0.256	0.070	7.079	1357	WS0093_C09
8	11.974	150	1	0.263	0.046	4.626	1357	WS0093_C09
8	39.090	152	4	-0.290	0.072	6.925	1357	WS0093_C09
8	50.001	154	2	-0.256	0.108	8.803	1357	WS0093_C09
9	3.127	168	1	0.294	0.110	4.284	1357	WS0093_C09
11	35.604	207	3	-0.202	0.057	5.195	1357	WS0093_C09
11	37.143	209	3	0.208	0.060	5.486	1357	WS0093_C09
11	46.026	217	1	-0.295	0.060	6.090	1357	WS0093_C09
1	29.800	5	1	-0.186	0.046	3.845	1358	WS0093_E04
1	82.301	16	4	0.290	0.042	4.610	1358	WS0093_E04
2	102.814	36	2	0.262	0.035	4.169	1358	WS0093_E04
2	126.181	39	2	-0.166	0.038	3.855	1358	WS0093_E04
3	9.953	42	4	0.220	0.051	4.944	1358	WS0093_E04
3	154.505	69	4	0.226	0.074	6.773	1358	WS0093_E04
7	59.789	137	3	-0.336	0.064	6.101	1358	WS0093_E04
9	28.608	170	2	0.226	0.039	4.269	1358	WS0093_E04
3	15.147	44	2	0.279	0.117	6.572	1359	WS0093_F07
3	95.157	59	2	-0.197	0.032	4.399	1359	WS0093_F07
8	50.001	154	2	-0.262	0.059	6.794	1359	WS0093_F07
1	45.794	9	1	0.341	0.064	6.424	1360	WS0092_E10
1	50.955	10	1	-0.262	0.048	4.882	1360	WS0092_E10
11	17.088	203	2	0.201	0.065	4.086	1360	WS0092_E10

11	56.921	219	2	-0.343	0.131	5.526	1360	WS0092_E10
14	44.898	251	3	0.243	0.047	4.375	1360	WS0092_E10
1	29.800	5	2	0.208	0.036	4.441	1361	WS0092_J05
4	43.146	74	3	-0.246	0.052	3.978	1361	WS0092_J05
7	15.555	130	2	0.185	0.040	4.687	1361	WS0092_J05
7	15.555	130	3	-0.176	0.057	4.167	1361	WS0092_J05
10	80.088	194	1	-0.222	0.073	5.170	1361	WS0092_J05
10	93.347	196	1	0.481	0.111	5.556	1361	WS0092_J05
1	2.788	2	4	-0.232	0.049	5.674	1362	WS01013_C16
1	38.326	6	4	-0.195	0.042	4.650	1362	WS01013_C16
3	14.500	43	1	-0.287	0.061	5.254	1362	WS01013_C16
4	116.928	82	2	0.292	0.049	5.276	1362	WS01013_C16
7	15.555	130	2	0.269	0.046	4.796	1362	WS01013_C16
9	2.011	167	4	0.264	0.067	6.537	1362	WS01013_C16
10	78.479	193	3	0.429	0.065	6.063	1362	WS01013_C16
14	0.000	246	1	-0.219	0.059	5.042	1362	WS01013_C16
3	17.158	45	1	0.260	0.054	4.868	1363	WS01038_M08
6	95.249	126	1	0.408	0.097	5.271	1363	WS01038_M08
7	73.169	142	1	0.562	0.136	5.064	1363	WS01038_M08
8	0.000	148	4	0.280	0.037	4.108	1363	WS01038_M08
9	0.000	166	1	-0.187	0.089	7.998	1363	WS01038_M08
9	28.608	170	2	-0.263	0.064	6.770	1363	WS01038_M08
9	28.608	170	3	0.520	0.168	14.092	1363	WS01038_M08
9	49.013	173	1	0.288	0.069	5.819	1363	WS01038_M08
9	49.013	173	2	0.237	0.063	6.181	1363	WS01038_M08
9	59.963	177	4	0.261	0.068	7.482	1363	WS01038_M08
10	9.580	187	2	-0.186	0.061	4.327	1363	WS01038_M08
10	35.204	189	2	0.191	0.042	4.150	1363	WS01038_M08
11	57.661	220	4	0.257	0.052	5.573	1363	WS01038_M08
11	64.014	222	4	-0.233	0.036	4.337	1363	WS01038_M08
1	38.326	6	4	-0.361	0.065	6.151	1364	WS01013_I08
1	45.794	9	1	0.368	0.061	4.798	1364	WS01013_I08
2	126.181	39	2	-0.209	0.042	4.656	1364	WS01013_I08
4	0.000	71	4	-0.262	0.041	4.531	1364	WS01013_I08
4	75.958	77	3	0.323	0.095	4.421	1364	WS01013_I08
4	82.462	78	1	-0.254	0.061	4.877	1364	WS01013_I08
6	45.897	117	1	0.238	0.053	4.193	1364	WS01013_I08
6	89.401	125	2	0.326	0.069	7.726	1364	WS01013_I08
8	62.580	155	1	0.284	0.099	4.349	1364	WS01013_I08
1	38.326	6	4	-0.326	0.037	3.972	1365	WS0269_H20
1	77.177	15	2	-0.210	0.045	4.184	1365	WS0269_H20
1	95.572	17	2	-0.327	0.062	5.840	1365	WS0269_H20
1	95.572	17	3	0.260	0.093	7.928	1365	WS0269_H20
4	142.917	85	1	-0.160	0.045	4.217	1365	WS0269_H20
5	16.219	93	2	0.269	0.042	4.609	1365	WS0269_H20
6	25.914	114	1	-0.291	0.092	8.711	1365	WS0269_H20
9	28.608	170	3	0.306	0.060	5.349	1365	WS0269_H20
10	78.479	193	4	-0.346	0.061	6.203	1365	WS0269_H20
10	80.088	194	1	-0.213	0.056	5.354	1365	WS0269_H20
10	80.088	194	2	-0.238	0.044	4.323	1365	WS0269_H20
2	126.181	39	2	-0.245	0.039	4.409	1366	WS00712_A23
2	127.147	40	3	0.274	0.058	5.245	1366	WS00712_A23
3	14.500	43	1	-0.205	0.069	5.085	1366	WS00712_A23
4	92.039	79	2	0.254	0.056	6.401	1366	WS00712_A23

4	97.576	80	2	-0.194	0.040	4.013	1366	WS00712_A23
4	103.719	81	4	0.268	0.056	5.648	1366	WS00712_A23
5	57.017	102	2	0.195	0.033	3.912	1366	WS00712_A23
6	26.350	115	2	0.203	0.040	4.488	1366	WS00712_A23
13	3.386	245	1	0.372	0.102	7.784	1366	WS00712_A23
1	95.572	17	3	0.245	0.053	4.947	1367	WS00712_H04
3	14.500	43	1	-0.174	0.052	4.397	1367	WS00712_H04
7	17.610	131	3	0.261	0.068	4.167	1367	WS00712_H04
7	74.560	143	1	-0.220	0.057	4.835	1367	WS00712_H04
7	75.883	144	2	0.372	0.090	4.088	1367	WS00712_H04
9	0.000	163	1	-0.434	0.109	4.140	1367	WS00712_H04
9	55.358	175	1	0.339	0.050	4.435	1367	WS00712_H04
10	9.580	187	3	-0.375	0.124	4.823	1367	WS00712_H04
14	44.898	251	3	0.190	0.041	4.896	1367	WS00712_H04
5	0.000	89	3	0.389	0.055	4.471	1368	WS0094_M01
5	16.219	93	2	0.284	0.041	4.896	1368	WS0094_M01
5	57.017	102	2	0.209	0.051	6.332	1368	WS0094_M01
7	17.610	131	3	0.366	0.120	8.861	1368	WS0094_M01
8	0.000	148	4	0.297	0.128	9.460	1368	WS0094_M01
8	50.001	154	2	-0.160	0.034	4.120	1368	WS0094_M01
10	78.479	193	3	-0.201	0.036	4.017	1368	WS0094_M01
10	86.151	195	3	0.250	0.048	5.482	1368	WS0094_M01
3	17.158	45	1	0.251	0.048	4.639	1369	WS0057_B23
4	82.462	78	1	-0.283	0.047	4.629	1369	WS0057_B23
5	54.072	100	4	0.253	0.046	4.736	1369	WS0057_B23
8	95.937	159	3	0.247	0.054	4.718	1369	WS0057_B23
10	9.580	187	2	-0.303	0.112	5.875	1369	WS0057_B23
11	46.026	217	1	-0.282	0.090	8.626	1369	WS0057_B23
11	57.661	220	4	0.257	0.056	5.732	1369	WS0057_B23
11	64.014	222	4	-0.379	0.080	8.477	1369	WS0057_B23
1	95.572	17	3	0.321	0.095	9.640	1370	WS0094_P22
1	107.333	19	3	0.249	0.051	5.698	1370	WS0094_P22
3	15.147	44	2	-0.337	0.116	5.541	1370	WS0094_P22
4	116.928	82	2	-0.211	0.036	3.841	1370	WS0094_P22
6	25.914	114	1	-0.236	0.053	4.274	1370	WS0094_P22
6	106.702	128	2	0.448	0.094	5.606	1370	WS0094_P22
7	75.883	144	2	0.396	0.078	4.178	1370	WS0094_P22
11	46.026	217	1	-0.282	0.086	6.895	1370	WS0094_P22
12	0.000	233	1	-0.249	0.066	5.290	1370	WS0094_P22
2	126.181	39	4	-0.402	0.089	4.191	1371	WS0057_K19
4	75.958	77	3	-0.387	0.142	8.301	1371	WS0057_K19
4	124.132	83	1	0.733	0.229	10.991	1371	WS0057_K19
4	142.917	85	1	-0.204	0.064	4.951	1371	WS0057_K19
6	45.897	117	1	0.196	0.051	4.030	1371	WS0057_K19
6	95.249	126	1	0.318	0.135	4.157	1371	WS0057_K19
10	78.479	193	3	-0.392	0.102	9.240	1371	WS0057_K19
10	86.151	195	3	0.243	0.083	7.374	1371	WS0057_K19
14	44.898	251	3	0.247	0.057	5.434	1371	WS0057_K19
3	14.500	43	1	-0.263	0.052	4.050	1372	WS00922_A24
3	83.563	56	3	0.255	0.071	6.012	1372	WS00922_A24
7	62.787	139	2	0.217	0.041	4.044	1372	WS00922_A24
8	50.001	154	1	0.178	0.062	4.802	1372	WS00922_A24
10	21.864	188	1	0.330	0.061	4.740	1372	WS00922_A24
11	27.405	204	1	-0.231	0.051	4.034	1372	WS00922_A24

1	82.301	16	3	-0.253	0.036	4.143	1373	WS00922_B08
6	25.914	114	1	-0.216	0.046	3.845	1373	WS00922_B08
6	45.897	117	1	0.193	0.050	4.103	1373	WS00922_B08
8	39.090	152	4	-0.237	0.061	4.565	1373	WS00922_B08
8	95.937	159	3	0.216	0.039	4.409	1373	WS00922_B08
11	35.604	207	3	-0.200	0.043	4.928	1373	WS00922_B08
11	42.600	213	3	-0.198	0.040	4.488	1373	WS00922_B08
11	46.026	217	1	-0.222	0.047	3.933	1373	WS00922_B08
12	0.000	233	1	-0.203	0.048	3.942	1373	WS00922_B08
13	3.386	245	1	-0.246	0.098	8.112	1373	WS00922_B08
4	75.958	77	3	-0.308	0.120	4.553	1374	WS00922_C18
4	124.132	83	1	0.433	0.075	4.352	1374	WS00922_C18
8	0.000	148	4	0.244	0.036	4.377	1374	WS00922_C18
9	59.963	177	4	-0.289	0.067	8.696	1374	WS00922_C18
10	78.479	193	3	-0.381	0.102	6.990	1374	WS00922_C18
10	78.479	193	4	-0.244	0.036	4.366	1374	WS00922_C18
10	86.151	195	3	0.198	0.088	6.030	1374	WS00922_C18
11	27.405	204	4	0.279	0.042	5.706	1374	WS00922_C18
13	0.000	243	4	0.460	0.099	5.838	1374	WS00922_C18
1	107.333	19	4	-0.235	0.033	3.867	1375	WS00922_D05
1	123.316	20	4	-0.294	0.101	4.500	1375	WS00922_D05
1	131.686	23	2	0.431	0.100	4.378	1375	WS00922_D05
3	47.928	49	1	0.189	0.044	4.439	1375	WS00922_D05
3	148.745	68	4	-0.140	0.028	3.953	1375	WS00922_D05
4	124.132	83	1	0.578	0.129	7.987	1375	WS00922_D05
4	142.917	85	1	-0.256	0.070	7.027	1375	WS00922_D05
6	45.897	117	1	0.173	0.041	4.148	1375	WS00922_D05
6	95.249	126	1	0.326	0.097	3.843	1375	WS00922_D05
8	0.000	148	4	0.269	0.044	4.801	1375	WS00922_D05
9	28.608	170	3	0.268	0.050	4.045	1375	WS00922_D05
10	78.479	193	3	-0.352	0.066	5.469	1375	WS00922_D05
10	86.151	195	3	0.157	0.056	4.584	1375	WS00922_D05
10	93.347	196	1	0.452	0.124	4.902	1375	WS00922_D05
10	93.347	196	2	0.420	0.097	4.054	1375	WS00922_D05
1	123.316	20	4	-0.297	0.072	3.980	1376	WS00922_E10
4	124.132	83	1	0.767	0.227	11.986	1376	WS00922_E10
4	142.917	85	1	-0.194	0.065	4.874	1376	WS00922_E10
6	49.100	118	4	0.448	0.072	3.885	1376	WS00922_E10
8	0.000	148	4	0.283	0.052	5.636	1376	WS00922_E10
9	59.963	177	4	-0.281	0.075	6.561	1376	WS00922_E10
10	78.479	193	3	-0.477	0.143	11.643	1376	WS00922_E10
10	86.151	195	3	0.185	0.071	5.742	1376	WS00922_E10
11	9.237	202	4	0.153	0.040	4.261	1376	WS00922_E10
1	50.955	10	1	-0.209	0.066	4.718	1377	WS00922_E17
4	75.958	77	3	-0.329	0.101	4.611	1377	WS00922_E17
4	103.719	81	4	0.299	0.059	6.083	1377	WS00922_E17
4	116.928	82	2	-0.323	0.060	6.875	1377	WS00922_E17
9	17.951	169	2	-0.260	0.040	4.865	1377	WS00922_E17
10	9.580	187	3	-0.460	0.151	5.556	1377	WS00922_E17
10	86.151	195	3	0.145	0.048	4.277	1377	WS00922_E17
10	93.347	196	1	0.455	0.112	4.980	1377	WS00922_E17
10	104.214	198	4	-0.288	0.049	7.089	1377	WS00922_E17
13	1.815	244	2	0.286	0.040	4.667	1377	WS00922_E17
14	0.000	246	1	-0.212	0.065	4.657	1377	WS00922_E17

14	43.392	250	2	-0.208	0.037	4.787	1377	WS00922_E17
14	44.898	251	3	0.275	0.059	5.328	1377	WS00922_E17
1	131.686	23	2	0.402	0.056	3.862	1378	WS00922_F17
8	0.000	148	4	0.186	0.037	3.859	1378	WS00922_F17
1	15.589	3	2	0.206	0.070	6.247	1379	WS00922_G07
4	124.132	83	1	0.491	0.101	5.074	1379	WS00922_G07
4	124.132	83	2	-0.305	0.116	3.898	1379	WS00922_G07
6	49.100	118	4	0.398	0.083	4.074	1379	WS00922_G07
7	62.787	139	4	-0.178	0.037	4.486	1379	WS00922_G07
7	75.883	144	2	0.448	0.088	5.392	1379	WS00922_G07
7	75.883	144	4	-0.373	0.064	7.008	1379	WS00922_G07
11	9.237	202	4	0.178	0.040	4.269	1379	WS00922_G07
11	46.026	217	1	-0.281	0.056	5.589	1379	WS00922_G07
12	22.629	237	1	0.237	0.043	4.234	1379	WS00922_G07
14	0.000	246	2	0.248	0.067	5.615	1379	WS00922_G07
1	107.333	19	3	0.229	0.042	5.032	1380	WS00946_A07
3	95.157	59	2	-0.195	0.045	3.848	1380	WS00946_A07
4	55.925	76	1	0.196	0.038	4.022	1380	WS00946_A07
6	25.914	114	1	-0.239	0.037	4.155	1380	WS00946_A07
6	81.996	123	3	-0.332	0.064	5.065	1380	WS00946_A07
6	106.702	128	2	-0.409	0.098	4.093	1380	WS00946_A07
11	0.000	199	3	-0.298	0.039	4.627	1380	WS00946_A07
13	0.000	243	4	-0.334	0.145	4.830	1380	WS00946_A07
1	40.464	7	4	-0.252	0.044	4.426	1381	WS00817_J08
3	80.211	55	4	0.244	0.071	7.086	1381	WS00817_J08
3	116.958	63	4	-0.187	0.043	4.269	1381	WS00817_J08
5	84.347	105	1	-0.232	0.057	4.995	1381	WS00817_J08
5	86.669	106	4	0.452	0.218	7.880	1381	WS00817_J08
9	28.608	170	3	-0.229	0.049	4.658	1381	WS00817_J08
9	55.358	175	1	0.283	0.057	5.026	1381	WS00817_J08
9	55.358	175	3	-0.550	0.132	7.498	1381	WS00817_J08
14	9.923	247	1	0.212	0.047	4.148	1381	WS00817_J08
14	43.392	250	2	-0.225	0.049	4.889	1381	WS00817_J08
1	22.419	4	3	0.345	0.077	6.266	1382	WS00946_J07
1	40.464	7	3	0.233	0.034	4.090	1382	WS00946_J07
1	40.464	7	4	-0.252	0.061	4.123	1382	WS00946_J07
1	43.578	8	2	-0.216	0.049	4.304	1382	WS00946_J07
1	43.578	8	3	0.269	0.053	6.292	1382	WS00946_J07
1	133.992	24	2	-0.194	0.044	3.880	1382	WS00946_J07
1	141.021	25	2	0.191	0.044	3.848	1382	WS00946_J07
9	0.000	166	1	0.235	0.045	4.483	1382	WS00946_J07
12	0.000	226	3	-0.195	0.036	4.259	1382	WS00946_J07
1	38.326	6	4	-0.259	0.041	4.311	1383	WS00817_P07
1	40.464	7	4	0.330	0.054	5.834	1383	WS00817_P07
1	50.955	10	1	-0.187	0.078	4.566	1383	WS00817_P07
3	47.928	49	1	0.285	0.082	4.822	1383	WS00817_P07
6	89.401	125	1	-0.294	0.082	4.787	1383	WS00817_P07
6	95.249	126	1	0.470	0.217	6.664	1383	WS00817_P07
8	62.580	155	1	0.289	0.156	4.430	1383	WS00817_P07
9	0.000	163	1	-0.424	0.132	3.956	1383	WS00817_P07
9	28.608	170	3	0.296	0.046	4.252	1383	WS00817_P07
10	9.580	187	2	-0.301	0.069	4.783	1383	WS00817_P07
11	29.069	205	2	0.155	0.034	5.083	1383	WS00817_P07
11	41.984	212	2	0.152	0.031	3.942	1383	WS00817_P07

11	45.989	216	2	0.163	0.033	4.489	1383	WS00817_P07
11	60.464	221	2	0.312	0.053	5.827	1383	WS00817_P07
1	40.464	7	4	-0.307	0.043	4.634	1384	WS00922_H12
1	43.578	8	2	-0.250	0.043	5.126	1384	WS00922_H12
4	97.576	80	2	0.255	0.041	6.314	1384	WS00922_H12
4	103.719	81	4	0.244	0.060	7.769	1384	WS00922_H12
4	124.132	83	2	0.390	0.164	6.103	1384	WS00922_H12
7	75.883	144	2	-0.470	0.073	6.240	1384	WS00922_H12
12	51.382	242	1	0.388	0.170	5.047	1384	WS00922_H12
3	14.500	43	1	-0.329	0.080	6.968	1385	WS00922_L03
3	17.158	45	1	0.197	0.078	6.605	1385	WS00922_L03
8	11.974	150	1	-0.191	0.071	6.068	1385	WS00922_L03
8	95.937	159	3	0.291	0.056	6.103	1385	WS00922_L03
9	3.127	168	1	-0.461	0.110	5.427	1385	WS00922_L03
14	44.898	251	3	0.344	0.049	5.549	1385	WS00922_L03
1	62.128	11	1	0.449	0.194	3.862	1386	WS01017_M20
2	120.617	38	3	0.538	0.214	21.940	1386	WS01017_M20
2	127.147	40	3	-0.428	0.206	21.094	1386	WS01017_M20
4	43.146	74	3	0.160	0.039	3.921	1386	WS01017_M20
6	25.742	113	3	-0.224	0.038	3.882	1386	WS01017_M20
7	59.789	137	3	-0.187	0.050	5.121	1386	WS01017_M20
8	87.463	158	2	-0.197	0.034	4.198	1386	WS01017_M20
10	80.088	194	2	0.308	0.036	4.025	1386	WS01017_M20
13	1.815	244	2	0.324	0.058	7.360	1386	WS01017_M20
1	38.326	6	2	0.262	0.040	4.577	1387	WS01017_N05
1	95.572	17	3	0.373	0.100	9.046	1387	WS01017_N05
1	107.333	19	3	0.258	0.090	8.150	1387	WS01017_N05
1	107.333	19	4	-0.191	0.045	4.336	1387	WS01017_N05
2	75.550	31	2	0.252	0.056	6.413	1387	WS01017_N05
3	14.500	43	2	-0.329	0.062	6.121	1387	WS01017_N05
3	20.512	46	2	0.211	0.059	6.744	1387	WS01017_N05
3	141.443	65	3	-0.276	0.089	4.424	1387	WS01017_N05
3	144.593	66	4	0.229	0.066	6.102	1387	WS01017_N05
3	148.745	68	4	-0.219	0.039	3.935	1387	WS01017_N05
3	154.505	69	4	0.346	0.126	11.721	1387	WS01017_N05
7	62.787	139	2	0.227	0.038	4.069	1387	WS01017_N05
9	55.358	175	3	0.640	0.193	9.478	1387	WS01017_N05
9	69.046	181	1	0.278	0.080	4.013	1387	WS01017_N05
11	44.884	215	3	0.232	0.047	4.235	1387	WS01017_N05
14	43.392	250	2	-0.231	0.050	4.846	1387	WS01017_N05
14	46.637	252	4	-0.269	0.059	5.812	1387	WS01017_N05
1	29.800	5	2	-0.211	0.036	4.416	1388	WS01017_O18
1	38.326	6	2	0.248	0.038	4.566	1388	WS01017_O18
1	40.464	7	4	-0.314	0.064	6.506	1388	WS01017_O18
2	102.814	36	2	-0.291	0.058	6.885	1388	WS01017_O18
3	116.958	63	4	-0.270	0.063	6.420	1388	WS01017_O18
3	137.891	64	3	-0.279	0.068	5.779	1388	WS01017_O18
4	92.039	79	2	-0.222	0.038	4.768	1388	WS01017_O18
4	92.039	79	4	0.265	0.043	4.425	1388	WS01017_O18
4	103.719	81	4	0.230	0.065	7.296	1388	WS01017_O18
5	86.669	106	4	0.377	0.195	4.473	1388	WS01017_O18
6	25.742	113	3	-0.196	0.050	4.283	1388	WS01017_O18
6	26.350	115	2	0.246	0.056	7.017	1388	WS01017_O18
12	0.000	226	3	0.215	0.062	5.286	1388	WS01017_O18

12	22.441	227	3	-0.253	0.062	5.262	1388	WS01017_O18
2	101.076	35	4	-0.221	0.062	5.646	1389	WS01017_P04
5	0.000	89	3	0.273	0.049	4.687	1389	WS01017_P04
5	52.820	99	3	-0.247	0.046	4.156	1389	WS01017_P04
6	26.350	115	2	0.162	0.038	4.551	1389	WS01017_P04
8	87.463	158	2	-0.197	0.047	4.677	1389	WS01017_P04
10	86.151	195	3	0.265	0.050	4.958	1389	WS01017_P04
11	57.661	220	4	-0.239	0.072	6.560	1389	WS01017_P04
11	64.014	222	4	0.400	0.117	8.548	1389	WS01017_P04
13	1.815	244	2	0.234	0.041	5.070	1389	WS01017_P04
1	95.572	17	3	0.253	0.057	5.332	1390	WS00814_A19
4	103.719	81	4	-0.296	0.039	5.827	1390	WS00814_A19
7	15.555	130	2	0.386	0.065	6.772	1390	WS00814_A19
8	11.974	150	1	-0.184	0.045	3.998	1390	WS00814_A19
1	38.326	6	4	-0.302	0.056	5.579	1391	WS00814_B06
1	40.464	7	4	0.332	0.057	5.222	1391	WS00814_B06
2	75.550	31	1	0.282	0.044	4.561	1391	WS00814_B06
6	89.401	125	1	-0.315	0.053	5.541	1391	WS00814_B06
7	15.555	130	2	0.324	0.057	4.878	1391	WS00814_B06
9	0.000	163	1	-0.455	0.083	4.563	1391	WS00814_B06
11	46.026	217	1	-0.222	0.043	4.390	1391	WS00814_B06
1	131.686	23	2	0.409	0.106	3.913	1392	WS00814_C23
5	86.669	106	2	0.261	0.090	3.921	1392	WS00814_C23
8	95.937	159	3	0.252	0.054	5.326	1392	WS00814_C23
9	0.000	163	1	-0.487	0.096	5.245	1392	WS00814_C23
11	46.026	217	1	-0.206	0.043	4.516	1392	WS00814_C23
1	107.333	19	4	-0.318	0.066	5.865	1393	WS00814_D18
3	95.157	59	2	-0.234	0.059	5.253	1393	WS00814_D18
3	104.681	60	2	0.183	0.045	4.252	1393	WS00814_D18
3	112.868	61	2	-0.162	0.048	4.573	1393	WS00814_D18
3	114.747	62	2	-0.242	0.051	4.841	1393	WS00814_D18
5	31.191	95	1	-0.241	0.047	4.422	1393	WS00814_D18
9	2.011	167	4	0.238	0.055	6.360	1393	WS00814_D18
1	131.686	23	1	-0.483	0.070	4.142	1394	WS00815_D05
9	59.963	177	4	-0.295	0.074	7.965	1394	WS00815_D05
10	78.479	193	3	-0.347	0.052	4.712	1394	WS00815_D05
12	22.629	237	1	0.234	0.041	4.181	1394	WS00815_D05
13	0.000	243	4	0.458	0.067	4.217	1394	WS00815_D05
11	35.604	207	3	-0.234	0.081	6.486	1395	WS00815_I11
11	37.143	209	3	0.205	0.064	5.090	1395	WS00815_I11
11	42.600	213	3	-0.232	0.074	5.939	1395	WS00815_I11
3	154.505	69	1	0.220	0.061	6.732	1396	WS00715_C11
4	124.132	83	1	0.460	0.099	4.329	1396	WS00715_C11
4	124.132	83	2	-0.340	0.168	4.138	1396	WS00715_C11
4	142.917	85	1	-0.248	0.043	4.519	1396	WS00715_C11
7	15.555	130	2	0.363	0.097	8.280	1396	WS00715_C11
4	103.719	81	4	-0.249	0.055	4.775	1397	WS01021_O11
6	26.350	115	2	-0.176	0.036	3.954	1397	WS01021_O11
8	50.001	154	2	-0.205	0.038	4.002	1397	WS01021_O11
12	0.000	226	3	-0.277	0.058	6.145	1397	WS01021_O11
1	40.464	7	3	0.193	0.060	4.697	1398	WS00715_J19
1	43.578	8	3	0.189	0.058	4.512	1398	WS00715_J19
2	48.839	29	1	-0.316	0.066	5.037	1398	WS00715_J19
6	26.350	115	2	-0.217	0.061	6.960	1398	WS00715_J19

6	89.401	125	1	-0.290	0.063	4.635	1398	WS00715_J19
10	9.580	187	2	-0.276	0.075	5.722	1398	WS00715_J19
10	78.479	193	4	-0.172	0.036	3.868	1398	WS00715_J19
12	0.000	226	3	-0.262	0.058	4.619	1398	WS00715_J19
1	40.464	7	3	0.244	0.066	5.537	1399	WS00921_D05
1	43.578	8	3	0.256	0.072	6.023	1399	WS00921_D05
4	97.576	80	2	-0.200	0.040	4.664	1399	WS00921_D05
4	103.719	81	4	-0.161	0.030	4.073	1399	WS00921_D05
1	123.316	20	4	0.274	0.052	3.847	1400	WS00716_C07
3	148.745	68	4	0.225	0.039	4.089	1400	WS00716_C07
6	69.073	121	3	-0.250	0.068	6.248	1400	WS00716_C07
7	15.555	130	2	0.188	0.032	3.985	1400	WS00716_C07
11	57.661	220	4	0.190	0.045	4.311	1400	WS00716_C07
14	0.000	246	2	0.199	0.052	6.111	1400	WS00716_C07
4	103.719	81	4	-0.205	0.040	5.521	1401	WS00716_C22
8	50.001	154	2	-0.231	0.054	6.145	1401	WS00716_C22
9	0.000	163	1	-0.420	0.118	3.882	1401	WS00716_C22
10	104.214	198	4	-0.273	0.049	4.714	1401	WS00716_C22
11	35.604	207	3	-0.195	0.058	4.484	1401	WS00716_C22
11	46.026	217	1	-0.220	0.063	4.538	1401	WS00716_C22
11	64.014	222	4	-0.329	0.058	6.183	1401	WS00716_C22
1	15.589	3	2	0.180	0.036	3.926	1402	WS00716_E20
2	48.839	29	4	-0.203	0.041	4.619	1402	WS00716_E20
8	62.580	155	1	0.314	0.135	4.797	1402	WS00716_E20
10	78.479	193	3	-0.398	0.096	7.389	1402	WS00716_E20
10	104.214	198	4	-0.361	0.073	7.767	1402	WS00716_E20
11	46.026	217	1	-0.251	0.072	5.598	1402	WS00716_E20
14	0.000	246	2	0.194	0.044	4.845	1402	WS00716_E20
1	2.788	2	4	-0.207	0.034	3.852	1403	WS00716_F15
1	123.316	20	4	0.303	0.062	4.889	1403	WS00716_F15
8	50.001	154	2	-0.211	0.040	4.613	1403	WS00716_F15
8	87.463	158	2	0.180	0.034	4.067	1403	WS00716_F15
11	35.604	207	3	-0.208	0.085	6.508	1403	WS00716_F15
11	37.143	209	3	0.194	0.076	5.797	1403	WS00716_F15
11	46.026	217	1	-0.352	0.163	11.244	1403	WS00716_F15
11	64.014	222	4	-0.327	0.054	5.004	1403	WS00716_F15
3	14.500	43	1	-0.346	0.066	7.230	1404	WS00716_H08
3	17.158	45	1	0.302	0.091	9.895	1404	WS00716_H08
7	73.169	142	1	0.519	0.096	4.649	1404	WS00716_H08
8	11.974	150	1	-0.184	0.036	3.968	1404	WS00716_H08
9	3.127	168	1	-0.284	0.056	4.565	1404	WS00716_H08
12	22.629	237	1	-0.182	0.040	4.333	1404	WS00716_H08
12	32.473	238	1	-0.229	0.058	6.260	1404	WS00716_H08
1	2.788	2	3	-0.197	0.041	4.172	1405	WS00716_I01
3	14.500	43	1	-0.355	0.080	5.630	1405	WS00716_I01
3	15.147	44	2	0.362	0.068	5.047	1405	WS00716_I01
3	17.158	45	1	0.240	0.094	6.416	1405	WS00716_I01
4	82.462	78	1	-0.182	0.065	4.399	1405	WS00716_I01
5	84.347	105	2	0.201	0.032	3.944	1405	WS00716_I01
8	11.974	150	1	-0.214	0.072	4.952	1405	WS00716_I01
9	28.608	170	3	-0.393	0.105	9.725	1405	WS00716_I01
11	46.026	217	1	-0.264	0.061	4.230	1405	WS00716_I01
12	22.629	237	1	-0.181	0.061	4.154	1405	WS00716_I01
12	32.473	238	1	-0.236	0.093	6.364	1405	WS00716_I01

7	75.883	144	2	0.522	0.128	7.354	1406	WS00716_K08
8	50.001	154	1	0.263	0.061	6.184	1406	WS00716_K08
9	59.963	177	4	-0.243	0.053	4.305	1406	WS00716_K08
10	78.479	193	4	-0.232	0.048	4.376	1406	WS00716_K08
10	95.795	197	4	0.203	0.047	4.352	1406	WS00716_K08
11	0.000	199	3	-0.349	0.067	5.520	1406	WS00716_K08
12	51.382	242	1	-0.334	0.103	3.964	1406	WS00716_K08
3	17.158	45	1	0.173	0.051	3.871	1407	WS00716_L01
3	141.443	65	4	0.334	0.094	4.160	1407	WS00716_L01
3	144.647	67	1	-0.311	0.103	4.276	1407	WS00716_L01
9	3.127	168	1	-0.292	0.079	4.617	1407	WS00716_L01
14	46.637	252	4	-0.250	0.042	5.220	1407	WS00716_L01
1	40.464	7	4	-0.231	0.040	3.908	1408	WS00716_N10
1	62.128	11	1	0.530	0.152	5.328	1408	WS00716_N10
1	62.128	11	2	0.315	0.095	5.715	1408	WS00716_N10
3	95.157	59	2	-0.346	0.123	13.701	1408	WS00716_N10
3	104.681	60	2	0.314	0.086	10.452	1408	WS00716_N10
3	112.868	61	2	-0.328	0.084	10.208	1408	WS00716_N10
3	114.747	62	2	-0.287	0.091	11.097	1408	WS00716_N10
6	25.914	114	1	-0.256	0.084	5.561	1408	WS00716_N10
8	0.000	148	4	0.208	0.043	4.180	1408	WS00716_N10
8	50.001	154	2	-0.179	0.035	4.157	1408	WS00716_N10
3	104.681	60	2	0.244	0.049	5.907	1409	WS00716_N20
3	112.868	61	2	-0.247	0.040	4.879	1409	WS00716_N20
3	114.747	62	2	-0.162	0.042	4.980	1409	WS00716_N20
5	57.017	102	2	0.163	0.032	3.906	1409	WS00716_N20
6	26.350	115	2	0.230	0.053	6.268	1409	WS00716_N20
7	15.555	130	3	0.269	0.061	5.015	1409	WS00716_N20
8	39.090	152	4	0.236	0.046	4.889	1409	WS00716_N20
11	17.088	203	1	-0.245	0.078	5.333	1409	WS00716_N20
11	64.014	222	4	0.231	0.044	4.550	1409	WS00716_N20
13	0.000	243	4	-0.436	0.229	8.375	1409	WS00716_N20
1	45.794	9	1	0.376	0.059	5.911	1410	WS0089_A22
1	45.794	9	3	-0.351	0.115	11.589	1410	WS0089_A22
3	14.500	43	1	-0.147	0.040	3.867	1410	WS0089_A22
3	17.158	45	1	0.320	0.082	7.971	1410	WS0089_A22
3	47.928	49	1	0.231	0.044	4.310	1410	WS0089_A22
6	25.914	114	1	-0.292	0.069	6.684	1410	WS0089_A22
6	69.073	121	3	-0.241	0.059	5.639	1410	WS0089_A22
6	69.073	121	4	0.223	0.045	4.166	1410	WS0089_A22
7	73.169	142	1	0.634	0.137	7.439	1410	WS0089_A22
7	91.487	146	3	0.147	0.050	4.765	1410	WS0089_A22
9	3.127	168	1	-0.369	0.047	4.468	1410	WS0089_A22
11	46.026	217	1	-0.258	0.063	6.100	1410	WS0089_A22
1	45.794	9	3	-0.367	0.072	6.484	1411	WS0089_C03
2	120.617	38	3	0.225	0.053	5.911	1411	WS0089_C03
6	106.702	128	2	0.379	0.061	3.985	1411	WS0089_C03
7	75.883	144	2	0.440	0.148	5.395	1411	WS0089_C03
7	75.883	144	4	-0.353	0.066	5.311	1411	WS0089_C03
8	87.463	158	2	-0.267	0.086	7.782	1411	WS0089_C03
9	3.127	168	1	-0.556	0.126	8.583	1411	WS0089_C03
9	28.608	170	3	0.302	0.060	4.201	1411	WS0089_C03
10	80.088	194	1	-0.234	0.053	4.903	1411	WS0089_C03
10	93.347	196	1	0.613	0.192	9.023	1411	WS0089_C03

12	22.441	227	3	-0.247	0.081	9.912	1411	WS0089_C03
14	44.898	251	3	0.393	0.090	10.384	1411	WS0089_C03
5	16.219	93	2	-0.322	0.050	5.928	1412	WS0089_F14
7	75.883	144	2	0.477	0.088	5.845	1412	WS0089_F14
1	95.572	17	3	0.234	0.061	4.058	1413	WS0089_G06
2	126.181	39	4	-0.409	0.102	4.838	1413	WS0089_G06
3	17.158	45	1	0.258	0.051	5.172	1413	WS0089_G06
6	89.401	125	1	-0.303	0.054	5.527	1413	WS0089_G06
7	73.169	142	1	0.547	0.109	5.176	1413	WS0089_G06
9	0.000	163	1	-0.516	0.108	5.865	1413	WS0089_G06
9	58.045	176	1	0.235	0.074	4.011	1413	WS0089_G06
3	154.505	69	1	0.269	0.064	6.390	1414	WS0089_J02
4	75.958	77	3	-0.384	0.123	5.461	1414	WS0089_J02
4	82.462	78	1	-0.282	0.050	4.777	1414	WS0089_J02
5	0.000	89	3	0.205	0.056	4.996	1414	WS0089_J02
6	95.249	126	1	0.307	0.141	5.257	1414	WS0089_J02
7	15.555	130	2	0.262	0.057	5.347	1414	WS0089_J02
8	62.580	155	1	0.209	0.072	4.099	1414	WS0089_J02
9	3.127	168	1	-0.371	0.050	4.891	1414	WS0089_J02
14	44.898	251	3	0.281	0.055	4.232	1414	WS0089_J02
1	45.794	9	3	-0.240	0.043	4.373	1415	WS0089_J16
1	50.955	10	1	-0.210	0.040	3.989	1415	WS0089_J16
4	0.000	71	4	-0.303	0.085	5.842	1415	WS0089_J16
4	124.132	83	1	0.707	0.278	10.007	1415	WS0089_J16
6	89.401	125	1	-0.387	0.073	7.340	1415	WS0089_J16
9	0.000	163	1	-0.652	0.148	9.440	1415	WS0089_J16
9	58.045	176	1	0.226	0.076	4.801	1415	WS0089_J16
9	59.963	177	1	0.320	0.050	5.059	1415	WS0089_J16
10	70.246	190	2	0.238	0.063	5.735	1415	WS0089_J16
2	48.839	29	4	-0.292	0.058	5.652	1416	WS0089_M04
3	116.958	63	4	0.152	0.037	3.965	1416	WS0089_M04
3	154.505	69	1	0.268	0.039	3.980	1416	WS0089_M04
6	89.401	125	1	-0.413	0.090	8.937	1416	WS0089_M04
6	95.249	126	1	0.449	0.177	8.650	1416	WS0089_M04
7	74.560	143	1	-0.265	0.069	6.769	1416	WS0089_M04
9	3.127	168	1	-0.503	0.127	8.710	1416	WS0089_M04
10	104.214	198	4	-0.325	0.040	4.092	1416	WS0089_M04
11	35.604	207	3	-0.164	0.050	3.969	1416	WS0089_M04
12	0.000	233	1	-0.229	0.051	5.030	1416	WS0089_M04
14	0.000	246	1	-0.212	0.039	3.891	1416	WS0089_M04
2	48.839	29	1	0.226	0.031	4.189	1417	WS00928_N13
3	9.953	42	3	-0.249	0.055	7.101	1417	WS00928_N13
3	17.158	45	1	0.174	0.031	4.257	1417	WS00928_N13
3	141.443	65	3	-0.237	0.052	5.452	1417	WS00928_N13
5	84.347	105	1	0.257	0.058	8.030	1417	WS00928_N13
9	0.000	166	1	-0.212	0.045	6.201	1417	WS00928_N13
9	58.045	176	1	0.237	0.041	4.404	1417	WS00928_N13
14	46.637	252	3	0.218	0.031	3.964	1417	WS00928_N13
1	95.572	17	3	0.216	0.053	4.301	1418	WS00810_A20
2	93.504	32	4	-0.253	0.055	5.438	1418	WS00810_A20
6	49.100	118	2	0.262	0.115	4.361	1418	WS00810_A20
6	49.100	118	4	0.423	0.122	4.915	1418	WS00810_A20
7	15.555	130	2	0.273	0.056	5.523	1418	WS00810_A20
10	9.580	187	3	-0.401	0.120	4.960	1418	WS00810_A20

11	17.088	203	2	0.240	0.055	4.392	1418	WS00810_A20
11	64.014	222	4	-0.361	0.061	5.839	1418	WS00810_A20
13	0.000	243	4	0.433	0.106	4.357	1418	WS00810_A20
1	127.805	21	3	0.223	0.038	5.166	1419	WS00929_A11
3	9.953	42	3	0.188	0.037	5.021	1419	WS00929_A11
3	14.500	43	1	0.299	0.065	7.894	1419	WS00929_A11
3	17.158	45	1	-0.266	0.065	7.994	1419	WS00929_A11
3	83.563	56	3	-0.208	0.032	4.354	1419	WS00929_A11
3	141.443	65	3	0.227	0.042	4.366	1419	WS00929_A11
7	59.789	137	3	0.186	0.033	4.429	1419	WS00929_A11
8	95.937	159	3	-0.192	0.034	4.634	1419	WS00929_A11
9	36.227	165	3	0.238	0.036	5.008	1419	WS00929_A11
10	78.479	193	3	-0.293	0.039	5.360	1419	WS00929_A11
11	43.491	214	3	0.244	0.056	7.665	1419	WS00929_A11
11	44.884	215	3	-0.198	0.038	5.160	1419	WS00929_A11
14	0.000	246	1	0.246	0.040	4.908	1419	WS00929_A11
1	45.794	9	3	-0.181	0.044	4.086	1420	WS01021_F23
1	82.301	16	3	-0.322	0.060	5.677	1420	WS01021_F23
3	47.928	49	1	0.241	0.066	4.423	1420	WS01021_F23
4	6.032	72	4	-0.300	0.068	6.526	1420	WS01021_F23
4	103.719	81	4	-0.209	0.035	3.915	1420	WS01021_F23
8	50.001	154	2	-0.199	0.036	3.914	1420	WS01021_F23
9	81.073	183	3	-0.233	0.067	6.109	1420	WS01021_F23
14	22.402	248	3	-0.255	0.044	4.128	1420	WS01021_F23
14	22.402	248	4	-0.269	0.042	4.036	1420	WS01021_F23
1	2.788	2	4	-0.209	0.038	4.085	1421	WS01021_J14
1	15.589	3	2	0.185	0.032	3.902	1421	WS01021_J14
11	57.661	220	4	0.223	0.044	5.264	1421	WS01021_J14
11	64.014	222	4	-0.291	0.054	5.666	1421	WS01021_J14
1	43.578	8	2	0.181	0.040	3.894	1422	WS0063_M21
1	69.854	13	2	-0.166	0.041	3.906	1422	WS0063_M21
1	71.592	14	2	-0.171	0.044	4.189	1422	WS0063_M21
4	82.462	78	1	0.166	0.045	4.152	1422	WS0063_M21
7	15.555	130	3	-0.207	0.053	5.259	1422	WS0063_M21
9	0.000	163	1	-0.566	0.116	7.141	1422	WS0063_M21
9	28.608	170	2	-0.165	0.039	3.905	1422	WS0063_M21
9	28.608	170	3	0.363	0.068	7.295	1422	WS0063_M21
10	95.795	197	4	0.216	0.053	4.268	1422	WS0063_M21
14	46.637	252	4	0.301	0.063	5.339	1422	WS0063_M21
3	47.928	49	1	0.231	0.069	5.954	1423	WS0063_N13
3	104.681	60	2	-0.176	0.040	4.074	1423	WS0063_N13
4	103.719	81	4	-0.250	0.051	4.718	1423	WS0063_N13
6	76.376	122	1	0.439	0.142	5.380	1423	WS0063_N13
6	89.401	125	1	-0.286	0.057	5.091	1423	WS0063_N13
8	62.580	155	1	0.283	0.104	3.911	1423	WS0063_N13
10	21.864	188	1	0.293	0.052	4.622	1423	WS0063_N13
1	82.301	16	4	0.366	0.082	6.819	1424	WS0064_B19
3	95.157	59	2	-0.200	0.063	8.551	1424	WS0064_B19
3	104.681	60	2	0.297	0.045	6.928	1424	WS0064_B19
3	114.747	62	2	-0.225	0.038	5.745	1424	WS0064_B19
4	103.719	81	4	-0.213	0.045	4.501	1424	WS0064_B19
5	3.484	90	2	-0.361	0.076	5.967	1424	WS0064_B19
5	42.979	97	2	-0.437	0.082	12.709	1424	WS0064_B19
8	39.090	152	4	-0.202	0.043	4.082	1424	WS0064_B19

9	17.951	169	2	0.299	0.041	5.644	1424	WS0064_B19
9	28.608	170	2	-0.246	0.057	8.237	1424	WS0064_B19
9	32.356	171	2	-0.170	0.051	7.537	1424	WS0064_B19
9	59.963	177	4	0.252	0.045	4.762	1424	WS0064_B19
10	80.088	194	2	0.273	0.027	4.171	1424	WS0064_B19
10	104.214	198	2	0.267	0.037	5.388	1424	WS0064_B19
11	9.237	202	4	0.283	0.074	6.915	1424	WS0064_B19
11	17.088	203	2	0.085	0.017	4.352	1424	WS0064_B19
12	6.242	234	2	-0.183	0.035	4.253	1424	WS0064_B19
14	0.000	246	2	-0.134	0.036	6.538	1424	WS0064_B19
1	40.464	7	3	0.222	0.039	4.368	1425	WS0064_C18
1	43.578	8	3	0.229	0.042	4.554	1425	WS0064_C18
1	45.794	9	3	-0.193	0.034	3.999	1425	WS0064_C18
4	140.962	84	3	-0.171	0.039	4.389	1425	WS0064_C18
6	25.914	114	1	-0.258	0.076	5.435	1425	WS0064_C18
9	36.227	165	3	0.195	0.049	5.436	1425	WS0064_C18
11	46.026	217	1	-0.230	0.058	4.138	1425	WS0064_C18
12	14.406	230	3	-0.224	0.035	3.923	1425	WS0064_C18
1	40.464	7	3	-0.279	0.079	6.266	1426	WS0064_G03
1	43.578	8	3	-0.278	0.079	5.969	1426	WS0064_G03
3	9.953	42	4	0.290	0.057	6.146	1426	WS0064_G03
6	25.742	113	3	-0.165	0.067	5.325	1426	WS0064_G03
6	45.897	117	1	0.267	0.072	5.646	1426	WS0064_G03
6	56.188	120	1	0.251	0.053	3.947	1426	WS0064_G03
6	56.188	120	2	0.420	0.086	9.950	1426	WS0064_G03
6	69.073	121	4	0.190	0.050	5.863	1426	WS0064_G03
7	15.555	130	3	-0.247	0.047	4.041	1426	WS0064_G03
7	59.789	137	3	-0.320	0.090	7.269	1426	WS0064_G03
7	70.278	141	3	0.316	0.094	7.576	1426	WS0064_G03
7	105.477	147	2	0.321	0.136	10.712	1426	WS0064_G03
9	49.013	173	2	-0.241	0.054	6.000	1426	WS0064_G03
10	9.580	187	3	0.358	0.108	4.282	1426	WS0064_G03
10	93.347	196	1	-0.481	0.127	5.554	1426	WS0064_G03
11	17.088	203	2	0.197	0.039	4.718	1426	WS0064_G03
11	40.960	210	2	0.525	0.100	7.936	1426	WS0064_G03
12	6.242	234	4	0.223	0.034	5.058	1426	WS0064_G03
13	1.815	244	4	0.191	0.035	4.345	1426	WS0064_G03
3	141.443	65	4	0.213	0.086	3.848	1427	WS0064_H04
4	140.962	84	3	-0.166	0.045	4.816	1427	WS0064_H04
5	31.191	95	1	-0.231	0.058	5.339	1427	WS0064_H04
6	25.914	114	1	-0.280	0.073	6.725	1427	WS0064_H04
11	17.088	203	1	-0.225	0.049	4.484	1427	WS0064_H04
11	46.026	217	1	-0.324	0.091	8.379	1427	WS0064_H04
11	57.661	220	4	0.284	0.053	4.511	1427	WS0064_H04
11	64.014	222	4	-0.353	0.062	4.149	1427	WS0064_H04
12	22.629	237	1	0.289	0.080	7.408	1427	WS0064_H04
12	32.473	238	1	0.221	0.045	4.189	1427	WS0064_H04
12	51.382	242	1	-0.393	0.107	4.517	1427	WS0064_H04
1	69.854	13	2	-0.251	0.050	4.455	1428	WS0064_K01
1	71.592	14	2	-0.244	0.047	4.176	1428	WS0064_K01
1	131.686	23	2	0.428	0.072	4.112	1428	WS0064_K01
2	75.550	31	1	0.219	0.037	3.899	1428	WS0064_K01
4	43.146	74	3	0.283	0.063	5.330	1428	WS0064_K01
4	103.719	81	4	-0.299	0.046	6.234	1428	WS0064_K01

5	7.672	92	1	-0.292	0.051	5.421	1428	WS0064_K01
6	45.897	117	1	0.357	0.078	8.612	1428	WS0064_K01
6	87.891	124	2	0.217	0.100	8.633	1428	WS0064_K01
6	89.401	125	2	0.417	0.106	9.242	1428	WS0064_K01
6	106.702	128	2	-0.398	0.148	4.351	1428	WS0064_K01
8	50.001	154	1	0.143	0.043	4.891	1428	WS0064_K01
11	17.088	203	1	0.280	0.041	4.318	1428	WS0064_K01
11	27.405	204	1	-0.380	0.088	9.401	1428	WS0064_K01
11	29.069	205	1	-0.240	0.043	4.401	1428	WS0064_K01
3	95.157	59	2	0.242	0.056	4.884	1429	WS0064_L12
3	104.681	60	2	-0.149	0.048	3.960	1429	WS0064_L12
3	141.443	65	4	0.202	0.067	4.324	1429	WS0064_L12
4	103.719	81	4	-0.162	0.029	4.734	1429	WS0064_L12
7	75.883	144	2	0.560	0.154	8.489	1429	WS0064_L12
7	75.883	144	4	-0.312	0.040	4.490	1429	WS0064_L12
11	46.026	217	1	-0.280	0.049	5.207	1429	WS0064_L12
1	141.021	25	2	0.208	0.033	3.933	1430	WS0091_I07
7	91.487	146	3	-0.218	0.081	6.474	1430	WS0091_I07
12	6.242	234	4	0.284	0.036	5.640	1430	WS0091_I07
14	22.402	248	4	-0.358	0.060	6.524	1430	WS0091_I07
14	46.637	252	4	-0.321	0.056	6.369	1430	WS0091_I07
1	69.854	13	2	0.196	0.039	4.537	1431	WS0091_I17
1	71.592	14	2	0.197	0.040	4.642	1431	WS0091_I17
1	77.177	15	2	0.253	0.066	7.691	1431	WS0091_I17
3	69.071	52	1	0.320	0.112	5.278	1431	WS0091_I17
5	3.484	90	2	-0.310	0.085	5.889	1431	WS0091_I17
5	16.219	93	2	0.225	0.036	3.868	1431	WS0091_I17
5	86.669	106	2	-0.406	0.143	6.849	1431	WS0091_I17
6	45.897	117	1	-0.289	0.074	5.270	1431	WS0091_I17
6	81.996	123	3	-0.300	0.093	4.378	1431	WS0091_I17
6	89.401	125	1	-0.317	0.110	7.825	1431	WS0091_I17
1	95.572	17	3	0.350	0.082	10.906	1432	WS0091_K14
3	14.500	43	1	-0.293	0.058	5.753	1432	WS0091_K14
3	17.158	45	1	0.254	0.070	6.920	1432	WS0091_K14
7	91.487	146	3	0.169	0.038	4.493	1432	WS0091_K14
11	35.604	207	3	-0.257	0.037	4.541	1432	WS0091_K14
11	42.600	213	3	-0.244	0.035	4.339	1432	WS0091_K14
11	56.921	219	2	0.207	0.099	4.697	1432	WS0091_K14
1	45.794	9	3	-0.232	0.041	4.956	1433	WS0091_L09
2	48.839	29	1	-0.246	0.056	5.506	1433	WS0091_L09
2	126.181	39	4	-0.482	0.177	4.496	1433	WS0091_L09
3	17.158	45	1	0.230	0.056	5.504	1433	WS0091_L09
3	141.443	65	3	-0.377	0.104	6.645	1433	WS0091_L09
5	7.672	92	1	-0.362	0.071	6.631	1433	WS0091_L09
5	16.219	93	2	0.206	0.045	4.181	1433	WS0091_L09
6	45.897	117	1	0.322	0.062	6.244	1433	WS0091_L09
7	54.200	136	2	0.370	0.136	7.661	1433	WS0091_L09
7	62.787	139	2	0.356	0.107	10.188	1433	WS0091_L09
7	62.787	139	4	-0.296	0.065	5.176	1433	WS0091_L09
9	0.000	166	1	0.205	0.055	5.380	1433	WS0091_L09
11	46.026	217	1	-0.258	0.051	5.058	1433	WS0091_L09
13	1.815	244	2	0.296	0.092	8.344	1433	WS0091_L09
7	75.883	144	2	0.486	0.097	6.424	1434	WS0091_N17
9	55.358	175	1	0.313	0.061	4.388	1434	WS0091_N17

10	35.204	189	2	0.192	0.058	6.308	1434	WS0091_N17
10	70.246	190	4	-0.313	0.062	6.310	1434	WS0091_N17
12	6.242	234	4	-0.296	0.042	5.083	1434	WS0091_N17
6	95.249	126	1	0.354	0.109	4.684	1435	WS0091_O15
7	74.560	143	1	-0.190	0.049	4.244	1435	WS0091_O15
7	75.883	144	4	-0.346	0.050	3.919	1435	WS0091_O15
8	50.001	154	2	-0.185	0.042	4.163	1435	WS0091_O15
9	0.000	163	1	-0.458	0.108	4.610	1435	WS0091_O15
9	58.045	176	1	0.210	0.093	3.968	1435	WS0091_O15
11	46.026	217	1	-0.251	0.058	5.055	1435	WS0091_O15
14	0.000	246	1	-0.233	0.050	4.339	1435	WS0091_O15
1	127.805	21	3	-0.190	0.039	4.129	1436	WS0094_A22
4	116.928	82	2	-0.279	0.050	4.515	1436	WS0094_A22
4	124.132	83	2	-0.327	0.152	5.204	1436	WS0094_A22
7	15.555	130	2	0.238	0.052	4.690	1436	WS0094_A22
9	0.000	163	1	-0.588	0.109	7.595	1436	WS0094_A22
9	58.045	176	1	0.351	0.110	7.612	1436	WS0094_A22
9	69.046	181	1	-0.362	0.096	6.653	1436	WS0094_A22
14	0.000	246	1	-0.280	0.071	6.900	1436	WS0094_A22
1	40.464	7	4	0.267	0.037	4.107	1437	WS0094_B09
3	95.157	59	2	0.240	0.075	6.813	1437	WS0094_B09
3	104.681	60	2	-0.326	0.098	9.806	1437	WS0094_B09
3	112.868	61	2	0.232	0.062	6.130	1437	WS0094_B09
3	114.747	62	2	0.250	0.063	6.320	1437	WS0094_B09
3	154.505	69	4	-0.274	0.060	5.170	1437	WS0094_B09
6	89.401	125	1	-0.308	0.061	5.369	1437	WS0094_B09
6	95.249	126	1	0.339	0.081	4.693	1437	WS0094_B09
9	0.000	163	1	-0.512	0.100	5.768	1437	WS0094_B09
10	78.479	193	4	-0.224	0.044	4.418	1437	WS0094_B09
11	64.014	222	4	-0.234	0.037	3.891	1437	WS0094_B09
14	0.000	246	1	-0.223	0.058	5.100	1437	WS0094_B09
3	14.500	43	1	-0.216	0.059	4.906	1438	WS0012_G19
3	17.158	45	1	0.315	0.085	7.060	1438	WS0012_G19
6	106.702	128	2	0.392	0.085	4.409	1438	WS0012_G19
9	0.000	163	1	-0.502	0.106	5.533	1438	WS0012_G19
9	58.045	176	1	0.217	0.079	4.082	1438	WS0012_G19
10	86.151	195	3	0.190	0.038	3.883	1438	WS0012_G19
11	46.026	217	1	-0.255	0.080	6.652	1438	WS0012_G19
1	62.128	11	1	-0.516	0.108	5.404	1439	WS0012_I14
2	93.504	32	4	-0.217	0.034	4.141	1439	WS0012_I14
3	9.953	42	4	0.259	0.042	5.085	1439	WS0012_I14
6	49.100	118	4	0.451	0.136	7.025	1439	WS0012_I14
13	0.000	243	4	0.361	0.079	3.984	1439	WS0012_I14
9	0.000	163	1	-0.457	0.104	4.598	1440	WS0012_N20
10	78.479	193	3	-0.297	0.061	4.508	1440	WS0012_N20
10	104.214	198	4	-0.251	0.040	4.083	1440	WS0012_N20
11	35.604	207	3	-0.182	0.057	4.173	1440	WS0012_N20
11	46.026	217	1	-0.263	0.075	6.382	1440	WS0012_N20
11	57.661	220	4	0.245	0.046	5.284	1440	WS0012_N20
11	64.014	222	4	-0.293	0.052	5.689	1440	WS0012_N20
1	15.589	3	2	0.184	0.032	3.868	1441	WS0012_P06
4	103.719	81	4	-0.191	0.039	6.033	1441	WS0012_P06
8	50.001	154	2	-0.245	0.057	7.048	1441	WS0012_P06
11	0.000	199	2	0.231	0.038	4.660	1441	WS0012_P06

11	64.014	222	4	-0.255	0.040	5.494	1441	WS0012_P06
14	0.000	246	2	0.188	0.041	4.759	1441	WS0012_P06
1	133.992	24	2	0.190	0.043	4.920	1442	WS0013_E11
2	126.181	39	4	-0.389	0.056	3.910	1442	WS0013_E11
6	45.897	117	1	0.225	0.059	4.312	1442	WS0013_E11
6	89.401	125	2	0.255	0.039	4.306	1442	WS0013_E11
7	15.555	130	2	0.321	0.070	8.127	1442	WS0013_E11
10	78.479	193	3	-0.358	0.074	5.857	1442	WS0013_E11
10	104.214	198	4	-0.360	0.059	4.874	1442	WS0013_E11
14	0.000	246	2	0.275	0.068	5.213	1442	WS0013_E11
4	103.719	81	4	-0.194	0.039	5.397	1443	WS0013_F13
7	15.555	130	2	0.211	0.042	4.978	1443	WS0013_F13
10	104.214	198	4	-0.280	0.051	3.963	1443	WS0013_F13
1	123.316	20	4	0.225	0.052	4.329	1444	WS0013_M05
4	103.719	81	4	-0.198	0.029	4.720	1444	WS0013_M05
6	69.073	121	3	-0.250	0.087	5.534	1444	WS0013_M05
7	15.555	130	2	0.239	0.041	4.942	1444	WS0013_M05
8	50.001	154	2	-0.179	0.034	4.051	1444	WS0013_M05
10	104.214	198	4	-0.265	0.041	3.915	1444	WS0013_M05
11	46.026	217	1	-0.286	0.098	6.738	1444	WS0013_M05
14	0.000	246	2	0.204	0.042	4.743	1444	WS0013_M05
8	50.001	154	2	-0.236	0.050	5.685	1445	WS0013_O17
9	0.000	163	1	-0.450	0.131	4.462	1445	WS0013_O17
10	78.479	193	3	-0.328	0.076	6.053	1445	WS0013_O17
11	46.026	217	1	-0.197	0.059	4.185	1445	WS0013_O17
11	64.014	222	4	-0.269	0.046	3.934	1445	WS0013_O17
14	0.000	246	2	0.175	0.040	4.461	1445	WS0013_O17
1	95.572	17	2	0.258	0.048	4.056	1446	WS00731_A19
2	102.814	36	1	0.247	0.039	4.370	1446	WS00731_A19
3	14.500	43	1	-0.303	0.070	7.345	1446	WS00731_A19
3	15.147	44	2	0.257	0.109	6.186	1446	WS00731_A19
3	17.158	45	1	0.349	0.084	8.916	1446	WS00731_A19
4	82.462	78	1	-0.219	0.039	4.099	1446	WS00731_A19
4	142.917	85	1	0.406	0.097	10.374	1446	WS00731_A19
5	57.017	102	4	-0.217	0.054	4.058	1446	WS00731_A19
8	95.937	159	3	0.222	0.043	4.356	1446	WS00731_A19
9	0.000	163	1	-0.633	0.130	9.069	1446	WS00731_A19
9	17.951	169	1	0.297	0.060	6.265	1446	WS00731_A19
9	55.358	175	3	-0.451	0.073	4.809	1446	WS00731_A19
10	70.246	190	2	-0.231	0.050	4.136	1446	WS00731_A19
10	70.246	190	4	0.298	0.056	5.021	1446	WS00731_A19
11	64.014	222	4	-0.237	0.052	4.058	1446	WS00731_A19
12	32.473	238	1	-0.246	0.041	4.340	1446	WS00731_A19
14	0.000	246	1	-0.233	0.058	6.052	1446	WS00731_A19
1	107.333	19	3	0.233	0.061	5.433	1447	WS00112_F14
2	48.839	29	4	0.298	0.052	5.036	1447	WS00112_F14
3	154.505	69	4	0.264	0.048	4.784	1447	WS00112_F14
14	0.000	246	2	-0.251	0.060	5.454	1447	WS00112_F14
1	38.326	6	2	0.303	0.043	4.205	1448	WS00731_L02
1	95.572	17	3	0.234	0.051	4.862	1448	WS00731_L02
1	141.021	25	2	0.198	0.058	5.568	1448	WS00731_L02
5	31.191	95	1	-0.276	0.058	5.348	1448	WS00731_L02
6	45.897	117	1	0.204	0.044	4.072	1448	WS00731_L02
6	106.702	128	2	-0.520	0.140	6.854	1448	WS00731_L02

7	59.789	137	3	-0.220	0.043	4.144	1448	WS00731_L02
8	50.001	154	1	0.262	0.055	4.942	1448	WS00731_L02
8	95.937	159	3	0.200	0.057	5.479	1448	WS00731_L02
9	36.227	165	3	-0.254	0.044	4.248	1448	WS00731_L02
9	81.073	183	1	0.284	0.060	5.476	1448	WS00731_L02
11	43.491	214	3	-0.254	0.089	8.563	1448	WS00731_L02
11	44.884	215	3	0.292	0.059	5.762	1448	WS00731_L02
11	57.661	220	4	0.282	0.072	6.647	1448	WS00731_L02
11	64.014	222	4	-0.337	0.079	6.580	1448	WS00731_L02
1	43.578	8	2	0.207	0.050	5.284	1449	WS00112_O10
4	103.719	81	4	-0.221	0.054	4.575	1449	WS00112_O10
4	124.132	83	1	0.515	0.151	5.306	1449	WS00112_O10
6	87.891	124	2	0.225	0.064	6.793	1449	WS00112_O10
6	89.401	125	2	0.319	0.058	4.367	1449	WS00112_O10
1	2.788	2	3	-0.156	0.047	5.073	1450	WS00941_J14
1	40.464	7	4	0.272	0.047	4.365	1450	WS00941_J14
1	69.854	13	2	0.240	0.048	4.266	1450	WS00941_J14
1	71.592	14	2	0.251	0.055	4.899	1450	WS00941_J14
1	77.177	15	2	0.265	0.063	5.590	1450	WS00941_J14
5	7.672	92	4	-0.339	0.072	5.880	1450	WS00941_J14
5	57.017	102	2	0.264	0.048	4.180	1450	WS00941_J14
5	57.017	102	4	-0.245	0.052	4.409	1450	WS00941_J14
5	84.347	105	2	-0.255	0.059	5.608	1450	WS00941_J14
6	95.249	126	1	0.359	0.064	4.627	1450	WS00941_J14
7	54.200	136	2	0.374	0.158	5.969	1450	WS00941_J14
7	62.787	139	2	0.271	0.082	7.130	1450	WS00941_J14
8	95.937	159	3	0.348	0.076	8.503	1450	WS00941_J14
9	0.000	163	1	-0.613	0.121	8.667	1450	WS00941_J14
9	58.045	176	1	0.158	0.056	3.915	1450	WS00941_J14
9	59.963	177	1	0.345	0.069	7.213	1450	WS00941_J14
9	81.073	183	1	0.344	0.067	6.975	1450	WS00941_J14
11	46.026	217	1	-0.187	0.055	5.513	1450	WS00941_J14
12	0.000	233	1	-0.282	0.050	5.061	1450	WS00941_J14
3	104.681	60	2	0.271	0.060	5.851	1451	WS01040_C20
3	141.443	65	3	0.232	0.074	4.898	1451	WS01040_C20
4	43.146	74	3	0.272	0.066	7.668	1451	WS01040_C20
4	116.928	82	2	-0.269	0.056	5.503	1451	WS01040_C20
5	0.000	89	3	0.254	0.046	5.397	1451	WS01040_C20
5	42.979	97	1	0.351	0.068	6.166	1451	WS01040_C20
5	52.820	99	1	0.379	0.094	9.235	1451	WS01040_C20
6	81.996	123	3	-0.249	0.074	4.948	1451	WS01040_C20
6	106.702	128	2	-0.486	0.127	6.726	1451	WS01040_C20
6	106.702	128	3	0.271	0.043	4.927	1451	WS01040_C20
7	17.610	131	3	0.315	0.086	4.163	1451	WS01040_C20
8	87.463	158	2	-0.161	0.042	4.058	1451	WS01040_C20
11	40.960	210	2	0.606	0.181	9.743	1451	WS01040_C20
11	60.464	221	1	0.252	0.050	4.540	1451	WS01040_C20
4	43.146	74	3	0.248	0.072	6.357	1452	WS0014_E19
6	89.401	125	2	-0.214	0.033	3.850	1452	WS0014_E19
7	74.560	143	1	0.264	0.052	3.843	1452	WS0014_E19
8	39.090	152	4	0.225	0.046	4.617	1452	WS0014_E19
9	28.608	170	3	0.282	0.079	6.268	1452	WS0014_E19
9	55.358	175	3	0.538	0.182	5.272	1452	WS0014_E19
2	120.617	38	3	0.180	0.045	3.996	1453	WS0109_N04

4	103.719	81	4	-0.162	0.034	4.298	1453	WS0109_N04
6	95.249	126	1	0.319	0.068	4.101	1453	WS0109_N04
7	15.555	130	2	0.309	0.082	6.690	1453	WS0109_N04
9	0.000	163	1	-0.565	0.117	7.028	1453	WS0109_N04
10	9.580	187	3	-0.337	0.074	4.328	1453	WS0109_N04
10	93.347	196	1	0.418	0.072	4.298	1453	WS0109_N04
11	64.014	222	4	-0.344	0.051	5.297	1453	WS0109_N04
1	2.788	2	4	0.331	0.087	9.551	1454	WS0015_F01
1	22.419	4	3	0.275	0.091	4.463	1454	WS0015_F01
1	29.800	5	2	-0.264	0.047	4.961	1454	WS0015_F01
1	38.326	6	4	0.314	0.067	5.460	1454	WS0015_F01
1	40.464	7	4	-0.372	0.087	7.242	1454	WS0015_F01
1	43.578	8	2	-0.282	0.065	7.068	1454	WS0015_F01
1	43.578	8	3	0.308	0.062	4.820	1454	WS0015_F01
1	50.955	10	1	0.195	0.048	3.999	1454	WS0015_F01
1	95.572	17	2	-0.251	0.049	5.097	1454	WS0015_F01
7	15.555	130	3	0.207	0.058	4.603	1454	WS0015_F01
8	39.090	152	4	0.197	0.038	4.122	1454	WS0015_F01
9	0.000	163	1	0.508	0.117	5.710	1454	WS0015_F01
1	45.794	9	3	-0.171	0.047	4.325	1455	WS0081_D23
3	17.158	45	1	0.255	0.047	4.147	1455	WS0081_D23
6	25.914	114	1	-0.287	0.063	5.500	1455	WS0081_D23
7	62.787	139	4	-0.234	0.036	4.495	1455	WS0081_D23
7	67.154	140	4	-0.382	0.112	5.742	1455	WS0081_D23
7	73.169	142	1	0.623	0.131	6.722	1455	WS0081_D23
7	75.883	144	4	-0.359	0.092	7.003	1455	WS0081_D23
8	50.001	154	2	-0.221	0.045	4.561	1455	WS0081_D23
11	46.026	217	1	-0.215	0.064	5.527	1455	WS0081_D23
12	22.441	227	1	0.282	0.052	4.547	1455	WS0081_D23
1	43.578	8	2	0.231	0.058	4.722	1456	WS00929_D22
3	9.953	42	4	-0.184	0.059	5.548	1456	WS00929_D22
3	80.211	55	4	-0.284	0.078	6.626	1456	WS00929_D22
11	35.604	207	3	-0.200	0.047	4.846	1456	WS00929_D22
11	37.143	209	3	0.185	0.041	4.195	1456	WS00929_D22
11	42.600	213	3	-0.194	0.042	4.294	1456	WS00929_D22
12	6.242	234	4	-0.381	0.075	4.036	1456	WS00929_D22
3	141.443	65	4	0.269	0.116	5.599	1457	WS00929_G21
5	56.008	101	2	-0.172	0.031	4.001	1457	WS00929_G21
7	17.610	131	3	0.345	0.147	7.097	1457	WS00929_G21
9	0.000	163	1	-0.517	0.113	5.882	1457	WS00929_G21
11	46.026	217	1	-0.240	0.060	5.675	1457	WS00929_G21
1	15.589	3	2	0.224	0.042	4.898	1458	WS00732_I06
8	50.001	154	2	-0.272	0.063	7.399	1458	WS00732_I06
8	87.463	158	2	0.198	0.044	5.096	1458	WS00732_I06
14	22.402	248	4	-0.298	0.049	5.165	1458	WS00732_I06
2	120.617	38	3	0.220	0.059	3.876	1459	WS00732_I20
2	127.147	40	3	-0.182	0.059	3.864	1459	WS00732_I20
4	103.719	81	4	-0.232	0.050	6.954	1459	WS00732_I20
8	62.580	155	1	0.311	0.124	4.801	1459	WS00732_I20
10	78.479	193	3	-0.314	0.076	4.990	1459	WS00732_I20
10	104.214	198	4	-0.330	0.055	6.686	1459	WS00732_I20
11	46.026	217	1	-0.241	0.062	4.560	1459	WS00732_I20
11	64.014	222	4	-0.259	0.032	3.946	1459	WS00732_I20
4	97.576	80	2	-0.205	0.038	4.915	1460	WS00732_O24

4	103.719	81	4	-0.231	0.056	7.647	1460	WS00732_O24
6	26.350	115	2	-0.247	0.053	6.463	1460	WS00732_O24
9	0.000	163	1	-0.527	0.173	6.120	1460	WS00732_O24
10	78.479	193	4	-0.331	0.068	7.508	1460	WS00732_O24
10	80.088	194	2	-0.246	0.037	4.158	1460	WS00732_O24
11	41.984	212	2	0.213	0.041	5.048	1460	WS00732_O24
3	144.593	66	4	-0.169	0.042	4.327	1461	WS00733_B21
3	148.745	68	4	0.209	0.036	5.123	1461	WS00733_B21
3	154.505	69	4	-0.244	0.056	6.133	1461	WS00733_B21
4	142.917	85	1	-0.258	0.059	4.623	1461	WS00733_B21
6	69.073	121	3	-0.198	0.059	5.120	1461	WS00733_B21
6	89.401	125	1	-0.390	0.117	9.069	1461	WS00733_B21
6	95.249	126	1	0.376	0.087	4.534	1461	WS00733_B21
9	0.000	163	1	-0.637	0.182	9.178	1461	WS00733_B21
10	80.088	194	2	-0.338	0.054	6.456	1461	WS00733_B21
11	46.026	217	1	-0.206	0.075	5.841	1461	WS00733_B21
3	104.681	60	2	-0.190	0.037	3.915	1462	WS00733_H01
3	112.868	61	2	0.176	0.041	4.268	1462	WS00733_H01
3	114.747	62	2	0.236	0.048	5.111	1462	WS00733_H01
12	6.242	234	2	0.250	0.044	4.341	1462	WS00733_H01
14	46.637	252	3	0.265	0.044	5.321	1462	WS00733_H01
2	48.839	29	4	-0.211	0.070	5.238	1463	WS00733_I09
2	120.617	38	3	0.271	0.067	7.068	1463	WS00733_I09
2	127.147	40	3	-0.169	0.047	4.995	1463	WS00733_I09
3	116.958	63	4	0.265	0.055	4.596	1463	WS00733_I09
5	3.484	90	2	0.325	0.065	4.238	1463	WS00733_I09
6	95.249	126	1	0.322	0.085	4.284	1463	WS00733_I09
9	0.000	163	1	-0.524	0.121	6.091	1463	WS00733_I09
9	58.045	176	1	0.241	0.090	4.537	1463	WS00733_I09
9	59.963	177	1	0.305	0.077	5.515	1463	WS00733_I09
10	78.479	193	3	-0.426	0.079	8.343	1463	WS00733_I09
10	86.151	195	3	0.181	0.052	5.434	1463	WS00733_I09
1	2.788	2	3	-0.271	0.065	7.864	1464	WS00733_M24
1	127.805	21	3	-0.260	0.054	6.506	1464	WS00733_M24
2	95.880	34	3	0.273	0.051	5.572	1464	WS00733_M24
2	101.076	35	3	-0.293	0.054	6.458	1464	WS00733_M24
3	47.928	49	1	0.192	0.052	4.256	1464	WS00733_M24
7	15.555	130	3	-0.272	0.049	5.557	1464	WS00733_M24
10	9.580	187	3	-0.256	0.072	4.909	1464	WS00733_M24
10	70.246	190	2	0.247	0.049	5.225	1464	WS00733_M24
11	41.984	212	2	-0.184	0.044	4.641	1464	WS00733_M24
12	22.629	237	1	0.278	0.071	5.856	1464	WS00733_M24
3	154.505	69	1	0.220	0.040	4.111	1465	WS00733_P06
8	95.937	159	3	0.158	0.032	3.972	1465	WS00733_P06
10	9.580	187	3	-0.283	0.066	4.833	1465	WS00733_P06
10	35.204	189	1	-0.273	0.047	4.704	1465	WS00733_P06
10	93.347	196	1	0.400	0.054	3.846	1465	WS00733_P06
11	64.014	222	4	-0.300	0.080	5.037	1465	WS00733_P06
14	0.000	246	1	-0.251	0.059	5.895	1465	WS00733_P06
1	38.326	6	4	-0.307	0.062	5.063	1466	WS0105_B11
1	40.464	7	4	0.297	0.060	5.700	1466	WS0105_B11
1	95.572	17	3	0.182	0.044	4.576	1466	WS0105_B11
2	75.550	31	1	0.287	0.046	4.571	1466	WS0105_B11
4	75.958	77	3	-0.299	0.095	5.047	1466	WS0105_B11

5	56.008	101	2	-0.147	0.043	4.475	1466 WS0105_B11
5	57.017	102	2	0.309	0.093	8.449	1466 WS0105_B11
5	57.017	102	4	-0.363	0.089	7.940	1466 WS0105_B11
7	73.169	142	1	0.491	0.085	4.270	1466 WS0105_B11
7	75.883	144	2	0.458	0.138	5.382	1466 WS0105_B11
9	0.000	163	1	-0.438	0.085	4.229	1466 WS0105_B11
10	9.580	187	3	-0.389	0.086	4.217	1466 WS0105_B11
11	46.026	217	1	-0.210	0.057	5.599	1466 WS0105_B11
11	64.014	222	4	-0.292	0.061	5.134	1466 WS0105_B11
2	127.147	40	3	-0.178	0.036	4.023	1467 WS0105_C04
4	124.132	83	1	0.428	0.086	4.045	1467 WS0105_C04
5	57.017	102	2	0.278	0.074	7.018	1467 WS0105_C04
7	54.200	136	2	0.312	0.093	6.261	1467 WS0105_C04
7	73.169	142	1	0.485	0.086	4.071	1467 WS0105_C04
1	15.589	3	2	0.241	0.089	9.762	1468 WS0105_E01
1	29.800	5	2	-0.369	0.090	9.331	1468 WS0105_E01
1	38.326	6	2	0.428	0.113	12.678	1468 WS0105_E01
1	95.572	17	3	0.326	0.073	7.481	1468 WS0105_E01
4	103.719	81	4	-0.217	0.035	3.849	1468 WS0105_E01
5	57.017	102	2	0.216	0.041	4.391	1468 WS0105_E01
5	57.017	102	4	-0.355	0.081	7.651	1468 WS0105_E01
6	25.914	114	1	-0.197	0.069	5.333	1468 WS0105_E01
7	15.555	130	3	-0.275	0.062	5.457	1468 WS0105_E01
11	43.491	214	3	-0.156	0.040	3.906	1468 WS0105_E01
2	75.550	31	1	0.284	0.051	4.398	1469 WS0105_E23
3	47.928	49	1	0.237	0.063	5.378	1469 WS0105_E23
5	42.979	97	1	0.318	0.045	3.892	1469 WS0105_E23
9	0.000	163	1	-0.430	0.108	4.063	1469 WS0105_E23
11	46.026	217	1	-0.243	0.052	4.465	1469 WS0105_E23
3	80.211	55	4	-0.369	0.093	10.178	1470 WS0105_G17
6	87.891	124	2	0.226	0.048	6.510	1470 WS0105_G17
6	89.401	125	2	0.251	0.049	6.511	1470 WS0105_G17
7	75.883	144	2	0.364	0.055	4.278	1470 WS0105_G17
10	78.479	193	3	-0.353	0.070	5.591	1470 WS0105_G17
11	27.405	204	4	0.395	0.099	10.817	1470 WS0105_G17
13	1.815	244	4	-0.248	0.073	7.075	1470 WS0105_G17
1	141.021	25	2	-0.246	0.053	5.337	1471 WS0105_H12
3	83.563	56	3	-0.211	0.050	5.538	1471 WS0105_H12
4	124.132	83	1	-0.479	0.140	4.594	1471 WS0105_H12
6	81.996	123	3	-0.368	0.088	4.905	1471 WS0105_H12
7	62.787	139	4	-0.224	0.053	4.983	1471 WS0105_H12
7	75.883	144	2	0.701	0.230	12.277	1471 WS0105_H12
7	75.883	144	4	-0.370	0.094	7.263	1471 WS0105_H12
8	50.001	154	1	0.277	0.066	5.942	1471 WS0105_H12
9	0.000	163	1	-0.474	0.094	5.150	1471 WS0105_H12
13	3.386	245	1	0.338	0.072	6.493	1471 WS0105_H12
3	141.443	65	4	0.295	0.098	5.251	1472 WS0105_J04
4	82.462	78	1	-0.261	0.041	4.373	1472 WS0105_J04
6	25.914	114	1	-0.195	0.038	3.946	1472 WS0105_J04
6	45.897	117	1	0.203	0.042	4.318	1472 WS0105_J04
11	35.604	207	3	-0.195	0.051	4.363	1472 WS0105_J04
11	46.026	217	1	-0.348	0.091	9.415	1472 WS0105_J04
12	38.543	232	4	-0.360	0.076	4.053	1472 WS0105_J04
1	62.128	11	1	-0.568	0.139	6.402	1473 WS0105_J16

2	127.147	40	3	0.176	0.053	4.523	1473	WS0105_J16
4	103.719	81	4	0.231	0.074	7.727	1473	WS0105_J16
5	0.000	89	1	-0.274	0.047	4.425	1473	WS0105_J16
6	95.249	126	1	-0.352	0.142	6.621	1473	WS0105_J16
9	0.000	166	1	0.306	0.106	9.841	1473	WS0105_J16
9	2.011	167	4	0.210	0.055	5.599	1473	WS0105_J16
9	3.127	168	1	0.438	0.121	5.976	1473	WS0105_J16
9	69.046	181	1	0.348	0.121	5.624	1473	WS0105_J16
11	29.069	205	1	-0.291	0.064	5.971	1473	WS0105_J16
11	45.989	216	1	0.372	0.129	11.926	1473	WS0105_J16
11	60.464	221	1	-0.253	0.060	5.573	1473	WS0105_J16
3	116.958	63	4	0.175	0.047	4.369	1474	WS00728_K13
4	0.000	71	4	0.172	0.043	3.990	1474	WS00728_K13
6	87.891	124	2	0.266	0.057	6.754	1474	WS00728_K13
6	89.401	125	2	0.362	0.079	8.659	1474	WS00728_K13
11	35.604	207	3	-0.252	0.062	5.843	1474	WS00728_K13
11	37.143	209	3	0.218	0.045	4.295	1474	WS00728_K13
11	42.600	213	3	-0.233	0.051	4.827	1474	WS00728_K13
11	46.026	217	1	-0.215	0.065	4.536	1474	WS00728_K13
14	9.923	247	1	0.208	0.058	4.105	1474	WS00728_K13
1	40.464	7	4	-0.321	0.043	4.261	1475	WS01041_M11
1	43.578	8	2	-0.190	0.035	4.382	1475	WS01041_M11
4	103.719	81	4	0.248	0.067	7.290	1475	WS01041_M11
5	0.000	89	3	0.245	0.052	4.401	1475	WS01041_M11
7	75.883	144	2	-0.485	0.084	6.337	1475	WS01041_M11
10	77.676	192	1	0.190	0.067	4.095	1475	WS01041_M11
10	80.088	194	1	0.264	0.065	3.992	1475	WS01041_M11
11	42.600	213	3	0.219	0.054	4.586	1475	WS01041_M11
11	45.989	216	1	0.270	0.071	4.345	1475	WS01041_M11
1	40.464	7	4	-0.260	0.053	5.073	1476	WS00729_I04
1	62.128	11	1	0.683	0.270	8.827	1476	WS00729_I04
1	107.333	19	3	0.255	0.074	6.286	1476	WS00729_I04
9	36.227	165	3	0.182	0.046	3.895	1476	WS00729_I04
11	57.661	220	4	-0.217	0.042	4.281	1476	WS00729_I04
11	64.014	222	4	0.308	0.075	7.767	1476	WS00729_I04
1	50.955	10	1	0.271	0.041	4.778	1477	WS00834_G13
1	107.333	19	3	0.284	0.085	8.365	1477	WS00834_G13
1	127.805	21	3	0.251	0.044	4.416	1477	WS00834_G13
2	126.181	39	4	0.416	0.105	4.649	1477	WS00834_G13
3	14.500	43	1	0.293	0.040	4.675	1477	WS00834_G13
3	17.158	45	1	-0.259	0.077	8.828	1477	WS00834_G13
3	47.928	49	1	-0.181	0.046	5.256	1477	WS00834_G13
10	86.151	195	3	-0.225	0.046	4.566	1477	WS00834_G13
5	7.672	92	4	-0.179	0.054	3.998	1478	WS0094_D05
7	105.477	147	2	0.219	0.076	4.511	1478	WS0094_D05
9	36.227	165	3	0.275	0.054	5.744	1478	WS0094_D05
10	35.204	189	1	0.263	0.059	5.782	1478	WS0094_D05
10	104.214	198	4	-0.296	0.057	5.890	1478	WS0094_D05
11	17.088	203	2	0.242	0.055	4.047	1478	WS0094_D05
13	1.815	244	4	0.218	0.050	4.671	1478	WS0094_D05
1	82.301	16	3	-0.287	0.052	6.021	1479	WS0094_H16
6	56.188	120	2	0.237	0.048	4.345	1479	WS0094_H16
7	15.555	130	2	0.230	0.045	4.030	1479	WS0094_H16
9	0.000	163	1	-0.589	0.111	7.636	1479	WS0094_H16

9	17.951	169	2	0.310	0.079	5.033	1479	WS0094_H16
9	58.045	176	1	0.420	0.150	10.207	1479	WS0094_H16
9	59.963	177	1	0.377	0.091	9.008	1479	WS0094_H16
9	59.963	177	4	-0.229	0.068	5.201	1479	WS0094_H16
9	69.046	181	1	-0.370	0.097	6.616	1479	WS0094_H16
1	62.128	11	1	0.502	0.145	4.789	1480	WS0017_K17
3	14.500	43	1	0.283	0.055	4.456	1480	WS0017_K17
3	17.158	45	1	-0.260	0.058	4.612	1480	WS0017_K17
4	55.925	76	1	0.296	0.071	6.023	1480	WS0017_K17
12	14.406	230	3	-0.306	0.080	6.748	1480	WS0017_K17
13	1.815	244	2	0.332	0.058	6.335	1480	WS0017_K17
14	0.000	246	1	0.202	0.056	4.477	1480	WS0017_K17
1	95.572	17	3	0.264	0.053	6.010	1481	WS00940_E23
2	93.504	32	4	-0.387	0.153	7.729	1481	WS00940_E23
2	95.880	34	1	-0.461	0.130	10.169	1481	WS00940_E23
2	101.076	35	4	0.382	0.192	16.062	1481	WS00940_E23
2	102.814	36	1	-0.332	0.093	7.842	1481	WS00940_E23
2	126.181	39	2	0.219	0.053	5.770	1481	WS00940_E23
4	82.462	78	2	-0.249	0.047	5.300	1481	WS00940_E23
6	69.073	121	3	-0.258	0.038	4.145	1481	WS00940_E23
6	76.376	122	1	-0.281	0.145	4.447	1481	WS00940_E23
7	59.789	137	3	-0.147	0.043	4.564	1481	WS00940_E23
7	70.278	141	3	0.163	0.052	5.506	1481	WS00940_E23
7	91.487	146	3	0.210	0.040	4.272	1481	WS00940_E23
10	78.479	193	3	-0.399	0.060	6.461	1481	WS00940_E23
11	35.604	207	3	-0.196	0.064	6.820	1481	WS00940_E23
11	37.143	209	3	0.149	0.042	4.469	1481	WS00940_E23
11	42.600	213	3	-0.141	0.037	3.934	1481	WS00940_E23
11	46.026	217	1	-0.252	0.063	4.895	1481	WS00940_E23
1	15.589	3	2	0.211	0.046	5.352	1482	WS0018_J06
1	38.326	6	4	-0.227	0.031	3.858	1482	WS0018_J06
1	40.464	7	4	0.280	0.037	4.059	1482	WS0018_J06
4	103.719	81	4	-0.260	0.039	6.058	1482	WS0018_J06
6	87.891	124	2	0.225	0.044	5.111	1482	WS0018_J06
8	50.001	154	2	-0.245	0.060	6.898	1482	WS0018_J06
1	69.854	13	2	-0.168	0.037	4.927	1483	WS00941_A22
1	71.592	14	2	-0.152	0.032	4.187	1483	WS00941_A22
1	77.177	15	2	-0.153	0.032	4.215	1483	WS00941_A22
1	82.301	16	3	-0.263	0.052	5.137	1483	WS00941_A22
2	48.839	29	4	-0.398	0.142	12.497	1483	WS00941_A22
2	126.181	39	4	-0.465	0.089	5.661	1483	WS00941_A22
4	43.146	74	3	-0.231	0.062	6.141	1483	WS00941_A22
6	26.350	115	2	-0.171	0.035	4.642	1483	WS00941_A22
7	15.555	130	2	0.193	0.031	4.080	1483	WS00941_A22
10	78.479	193	3	-0.336	0.081	7.933	1483	WS00941_A22
10	86.151	195	3	0.263	0.065	6.399	1483	WS00941_A22
1	38.326	6	4	-0.299	0.050	4.700	1484	WS00923_C03
1	40.464	7	4	0.316	0.051	4.896	1484	WS00923_C03
1	95.572	17	3	0.255	0.058	5.326	1484	WS00923_C03
3	17.158	45	1	0.203	0.037	4.637	1484	WS00923_C03
3	154.505	69	1	0.276	0.042	5.301	1484	WS00923_C03
6	25.914	114	1	-0.228	0.043	5.385	1484	WS00923_C03
6	45.897	117	1	0.196	0.034	4.271	1484	WS00923_C03
7	73.169	142	1	0.449	0.068	4.337	1484	WS00923_C03

8	95.937	159	3	0.268	0.066	6.109	1484	WS00923_C03
9	28.608	170	3	0.263	0.051	4.585	1484	WS00923_C03
11	46.026	217	1	-0.271	0.050	6.362	1484	WS00923_C03
11	57.661	220	4	0.318	0.075	7.227	1484	WS00923_C03
11	64.014	222	4	-0.409	0.092	8.786	1484	WS00923_C03
4	140.962	84	3	0.501	0.235	23.589	1485	WS00923_F16
4	142.917	85	3	0.489	0.141	14.155	1485	WS00923_F16
4	173.929	88	2	0.188	0.047	4.941	1485	WS00923_F16
5	54.072	100	4	0.293	0.124	10.888	1485	WS00923_F16
5	57.017	102	4	-0.291	0.068	5.001	1485	WS00923_F16
9	2.011	167	4	-0.227	0.091	7.911	1485	WS00923_F16
9	55.358	175	3	-0.563	0.151	7.646	1485	WS00923_F16
9	58.045	176	1	0.394	0.122	6.632	1485	WS00923_F16
9	59.963	177	1	0.243	0.051	4.196	1485	WS00923_F16
9	69.046	181	1	-0.259	0.076	4.132	1485	WS00923_F16
14	43.392	250	2	0.260	0.057	5.513	1485	WS00923_F16
1	69.854	13	2	-0.250	0.052	5.335	1486	WS00820_K04
1	71.592	14	2	-0.255	0.055	5.672	1486	WS00820_K04
1	77.177	15	2	-0.273	0.065	6.619	1486	WS00820_K04
1	82.301	16	4	0.397	0.071	7.772	1486	WS00820_K04
4	0.000	71	4	0.186	0.058	6.963	1486	WS00820_K04
4	92.039	79	4	-0.338	0.054	5.804	1486	WS00820_K04
4	103.719	81	4	-0.254	0.036	5.655	1486	WS00820_K04
6	106.702	128	2	-0.392	0.130	4.046	1486	WS00820_K04
7	15.555	130	3	-0.285	0.077	5.568	1486	WS00820_K04
7	75.883	144	2	0.457	0.076	5.855	1486	WS00820_K04
7	75.883	144	4	-0.350	0.060	7.317	1486	WS00820_K04
9	17.951	169	1	-0.299	0.054	4.680	1486	WS00820_K04
10	9.580	187	3	0.326	0.114	4.843	1486	WS00820_K04
10	104.214	198	4	0.230	0.044	5.209	1486	WS00820_K04
1	38.326	6	4	-0.372	0.050	5.410	1487	WS00820_K14
1	40.464	7	4	0.394	0.065	6.803	1487	WS00820_K14
1	45.794	9	1	0.310	0.042	4.652	1487	WS00820_K14
1	50.955	10	1	-0.268	0.051	5.539	1487	WS00820_K14
3	17.158	45	1	0.198	0.042	4.546	1487	WS00820_K14
3	154.505	69	1	0.295	0.039	4.289	1487	WS00820_K14
4	92.039	79	4	-0.387	0.050	4.914	1487	WS00820_K14
7	73.169	142	1	0.624	0.134	7.460	1487	WS00820_K14
8	11.974	150	1	-0.193	0.037	4.010	1487	WS00820_K14
9	58.045	176	1	0.272	0.103	5.408	1487	WS00820_K14
9	59.963	177	1	0.311	0.046	5.065	1487	WS00820_K14
11	46.026	217	1	-0.300	0.063	6.913	1487	WS00820_K14
1	95.572	17	3	0.260	0.051	4.577	1488	WS00820_M13
2	120.617	38	3	0.266	0.049	4.353	1488	WS00820_M13
4	103.719	81	4	-0.193	0.028	4.153	1488	WS00820_M13
6	25.914	114	1	-0.265	0.081	9.112	1488	WS00820_M13
6	45.897	117	1	0.302	0.057	6.601	1488	WS00820_M13
8	62.580	155	1	0.238	0.079	4.304	1488	WS00820_M13
8	78.389	157	1	0.279	0.048	5.452	1488	WS00820_M13
10	93.347	196	1	0.498	0.102	5.998	1488	WS00820_M13
11	35.604	207	3	-0.207	0.045	3.996	1488	WS00820_M13
11	46.026	217	1	-0.276	0.040	4.609	1488	WS00820_M13
12	0.000	233	1	-0.148	0.036	4.061	1488	WS00820_M13
3	17.158	45	1	0.249	0.058	5.575	1489	WS00820_N01

7	73.169	142	1	0.497	0.086	4.463	1489	WS00820_N01
7	75.883	144	2	0.504	0.108	7.025	1489	WS00820_N01
9	3.127	168	1	-0.296	0.048	4.378	1489	WS00820_N01
9	58.045	176	1	0.302	0.128	5.610	1489	WS00820_N01
3	14.500	43	2	-0.217	0.042	3.843	1490	WS00820_O10
3	15.147	44	2	0.232	0.052	4.547	1490	WS00820_O10
3	116.958	63	4	0.211	0.053	5.230	1490	WS00820_O10
6	95.249	126	1	0.328	0.113	5.430	1490	WS00820_O10
8	39.090	152	4	-0.252	0.048	4.713	1490	WS00820_O10
8	50.001	154	2	-0.200	0.044	4.117	1490	WS00820_O10
9	0.000	163	1	-0.507	0.119	5.702	1490	WS00820_O10
11	35.604	207	3	-0.176	0.049	4.362	1490	WS00820_O10
11	46.026	217	1	-0.233	0.057	4.131	1490	WS00820_O10
1	95.572	17	2	-0.281	0.043	4.409	1491	WS00820_O17
1	107.333	19	4	-0.245	0.066	6.388	1491	WS00820_O17
1	127.805	21	4	0.224	0.058	5.338	1491	WS00820_O17
1	131.686	23	1	0.479	0.124	3.898	1491	WS00820_O17
4	82.462	78	2	-0.224	0.059	6.079	1491	WS00820_O17
4	97.576	80	2	0.181	0.042	4.069	1491	WS00820_O17
4	142.917	85	1	0.281	0.064	5.675	1491	WS00820_O17
5	84.347	105	1	0.252	0.075	6.255	1491	WS00820_O17
5	84.347	105	2	0.220	0.049	5.097	1491	WS00820_O17
5	86.669	106	2	-0.298	0.116	4.417	1491	WS00820_O17
7	15.555	130	3	0.299	0.085	4.255	1491	WS00820_O17
9	2.011	167	4	0.254	0.057	5.555	1491	WS00820_O17
9	28.608	170	3	-0.277	0.054	4.358	1491	WS00820_O17
9	55.358	175	3	-0.394	0.077	3.914	1491	WS00820_O17
9	59.963	177	4	-0.258	0.066	6.371	1491	WS00820_O17
13	3.386	245	1	0.220	0.059	4.976	1491	WS00820_O17
1	107.333	19	4	-0.225	0.044	3.917	1492	WS00821_A20
1	131.686	23	2	0.484	0.099	5.266	1492	WS00821_A20
1	133.992	24	2	0.211	0.044	4.768	1492	WS00821_A20
1	141.021	25	2	-0.221	0.050	5.375	1492	WS00821_A20
3	17.158	45	1	0.234	0.062	4.966	1492	WS00821_A20
6	89.401	125	1	-0.267	0.061	4.945	1492	WS00821_A20
6	95.249	126	1	0.279	0.101	5.072	1492	WS00821_A20
11	60.464	221	2	0.164	0.037	4.084	1492	WS00821_A20
1	123.316	20	4	-0.331	0.107	5.999	1493	WS00821_B17
4	142.917	85	3	0.270	0.053	4.716	1493	WS00821_B17
6	95.249	126	1	0.342	0.100	3.909	1493	WS00821_B17
7	15.555	130	2	0.263	0.052	5.682	1493	WS00821_B17
7	75.883	144	2	0.598	0.166	10.483	1493	WS00821_B17
7	105.477	147	2	-0.272	0.074	4.148	1493	WS00821_B17
9	0.000	163	1	-0.481	0.134	5.085	1493	WS00821_B17
9	28.608	170	3	0.281	0.050	4.264	1493	WS00821_B17
9	59.963	177	4	-0.181	0.042	4.543	1493	WS00821_B17
14	46.637	252	4	0.349	0.054	4.979	1493	WS00821_B17
1	15.589	3	2	-0.220	0.037	4.111	1494	WS00110_M07
1	29.800	5	2	0.249	0.045	5.080	1494	WS00110_M07
1	43.578	8	2	0.289	0.080	8.823	1494	WS00110_M07
1	45.794	9	3	0.307	0.063	4.808	1494	WS00110_M07
1	71.592	14	2	-0.230	0.042	4.647	1494	WS00110_M07
1	107.333	19	3	0.248	0.082	7.161	1494	WS00110_M07
3	80.211	55	4	-0.283	0.077	7.790	1494	WS00110_M07

4	75.958	77	3	-0.298	0.111	5.216	1494	WS00110_M07
5	7.672	92	4	0.307	0.051	4.851	1494	WS00110_M07
6	25.914	114	1	-0.191	0.069	5.266	1494	WS00110_M07
6	45.897	117	1	0.272	0.069	5.465	1494	WS00110_M07
6	56.188	120	1	0.240	0.057	4.383	1494	WS00110_M07
6	87.891	124	2	0.269	0.096	10.636	1494	WS00110_M07
6	89.401	125	2	0.368	0.080	8.567	1494	WS00110_M07
6	106.702	128	2	-0.443	0.099	4.970	1494	WS00110_M07
7	75.883	144	2	0.484	0.097	6.699	1494	WS00110_M07
9	17.951	169	1	-0.334	0.046	3.845	1494	WS00110_M07
10	93.347	196	1	-0.401	0.097	3.872	1494	WS00110_M07
12	0.000	233	1	-0.164	0.058	4.442	1494	WS00110_M07
1	107.333	19	3	0.265	0.059	5.426	1495	WS00110_O04
2	48.839	29	4	0.244	0.045	4.922	1495	WS00110_O04
4	55.925	76	1	0.190	0.049	4.201	1495	WS00110_O04
4	75.958	77	3	-0.296	0.213	4.780	1495	WS00110_O04
5	3.484	90	2	-0.220	0.064	4.743	1495	WS00110_O04
5	16.219	93	2	0.269	0.074	6.664	1495	WS00110_O04
12	0.000	233	1	-0.241	0.045	4.143	1495	WS00110_O04
3	20.512	46	2	-0.273	0.050	4.875	1496	WS00111_D09
3	80.211	55	4	-0.206	0.064	4.377	1496	WS00111_D09
3	80.211	55	4	-0.212	0.059	4.827	1497	WS00111_F06
5	0.000	89	3	0.335	0.043	4.144	1497	WS00111_F06
6	87.891	124	2	0.226	0.046	5.012	1497	WS00111_F06
6	89.401	125	2	0.310	0.041	4.487	1497	WS00111_F06
7	15.555	130	2	0.340	0.055	6.095	1497	WS00111_F06
9	0.000	163	1	-0.436	0.094	4.180	1497	WS00111_F06
9	28.608	170	3	0.253	0.034	3.881	1497	WS00111_F06
10	78.479	193	3	-0.212	0.050	5.236	1497	WS00111_F06
10	86.151	195	3	0.220	0.039	4.163	1497	WS00111_F06
12	6.242	234	2	0.212	0.049	5.936	1497	WS00111_F06
14	43.392	250	2	0.213	0.048	4.670	1497	WS00111_F06
3	80.211	55	4	-0.178	0.045	4.456	1498	WS00111_K05
6	87.891	124	2	0.344	0.114	13.860	1498	WS00111_K05
6	89.401	125	2	0.439	0.107	12.987	1498	WS00111_K05
8	50.001	154	2	-0.190	0.043	4.185	1498	WS00111_K05
1	131.686	23	1	-0.528	0.093	4.737	1499	WS00111_L22
2	120.617	38	4	-0.184	0.055	3.883	1499	WS00111_L22
4	103.719	81	4	-0.187	0.049	4.530	1499	WS00111_L22
4	124.132	83	1	0.601	0.146	7.429	1499	WS00111_L22
4	142.917	85	1	-0.252	0.079	6.663	1499	WS00111_L22
5	0.000	89	3	0.309	0.052	7.011	1499	WS00111_L22
5	16.219	93	2	0.248	0.038	4.452	1499	WS00111_L22
6	49.100	118	4	0.444	0.102	5.088	1499	WS00111_L22
6	54.066	119	4	0.214	0.056	4.812	1499	WS00111_L22
9	55.358	175	1	0.335	0.051	4.518	1499	WS00111_L22
10	78.479	193	3	-0.472	0.132	13.417	1499	WS00111_L22
10	86.151	195	3	0.187	0.041	4.160	1499	WS00111_L22
1	127.805	21	4	-0.240	0.044	5.148	1500	WS00111_P24
3	137.891	64	3	0.306	0.063	4.794	1500	WS00111_P24
6	26.350	115	2	-0.193	0.043	4.799	1500	WS00111_P24
7	62.787	139	4	-0.211	0.035	4.454	1500	WS00111_P24
11	46.026	217	1	-0.308	0.114	8.558	1500	WS00111_P24
14	46.637	252	4	0.272	0.038	4.061	1500	WS00111_P24

4	103.719	81	4	-0.250	0.065	7.033	1501	WS00112_A24
6	49.100	118	4	0.406	0.126	4.597	1501	WS00112_A24
6	87.891	124	2	0.193	0.040	4.832	1501	WS00112_A24
6	89.401	125	2	0.289	0.043	4.434	1501	WS00112_A24
8	50.001	154	2	-0.247	0.071	8.189	1501	WS00112_A24
8	87.463	158	2	0.214	0.035	4.163	1501	WS00112_A24
9	81.073	183	1	-0.229	0.059	3.937	1501	WS00112_A24
10	78.479	193	3	-0.329	0.081	7.105	1501	WS00112_A24
10	93.347	196	2	0.487	0.094	5.367	1501	WS00112_A24
11	41.984	212	2	0.205	0.033	4.067	1501	WS00112_A24
8	62.580	155	1	0.358	0.166	5.066	1502	WS01016_B05
10	78.479	193	3	-0.304	0.056	4.662	1502	WS01016_B05
11	57.661	220	4	0.209	0.037	3.865	1502	WS01016_B05
11	64.014	222	4	-0.284	0.053	4.386	1502	WS01016_B05
1	45.794	9	1	0.291	0.043	4.463	1503	WS01016_G22
1	123.316	20	4	-0.230	0.078	4.415	1503	WS01016_G22
2	75.550	31	1	0.278	0.046	4.774	1503	WS01016_G22
3	17.158	45	1	0.272	0.055	5.640	1503	WS01016_G22
9	3.127	168	1	-0.333	0.071	6.478	1503	WS01016_G22
11	46.026	217	1	-0.292	0.086	8.865	1503	WS01016_G22
11	56.921	219	1	-0.276	0.081	4.634	1503	WS01016_G22
11	57.661	220	4	0.225	0.045	5.191	1503	WS01016_G22
11	64.014	222	4	-0.342	0.058	7.025	1503	WS01016_G22
3	14.500	43	1	-0.278	0.053	6.383	1504	WS0084_L23
3	17.158	45	1	0.312	0.073	8.774	1504	WS0084_L23
4	0.000	71	4	0.243	0.064	6.744	1504	WS0084_L23
5	86.669	106	4	-0.304	0.073	4.991	1504	WS0084_L23
7	73.169	142	1	0.563	0.094	6.259	1504	WS0084_L23
11	46.026	217	1	-0.193	0.032	3.891	1504	WS0084_L23
11	57.661	220	4	0.234	0.040	4.202	1504	WS0084_L23
11	64.014	222	4	-0.347	0.067	6.978	1504	WS0084_L23
1	62.128	11	2	-0.428	0.117	7.408	1505	WS0022_N03
1	77.177	15	2	-0.192	0.048	4.842	1505	WS0022_N03
2	127.147	40	3	-0.230	0.054	5.527	1505	WS0022_N03
3	104.681	60	2	-0.215	0.044	4.482	1505	WS0022_N03
4	82.462	78	1	0.177	0.053	4.558	1505	WS0022_N03
5	86.669	106	4	-0.389	0.114	4.815	1505	WS0022_N03
6	26.350	115	2	-0.253	0.069	7.045	1505	WS0022_N03
7	75.883	144	2	0.436	0.119	4.993	1505	WS0022_N03
7	75.883	144	4	-0.282	0.055	4.732	1505	WS0022_N03
9	49.013	173	1	0.218	0.047	4.434	1505	WS0022_N03
9	49.013	173	2	0.205	0.042	4.299	1505	WS0022_N03
1	95.572	17	3	0.272	0.048	4.736	1506	WS0085_B24
1	131.686	23	2	0.455	0.152	4.608	1506	WS0085_B24
1	133.992	24	2	0.147	0.051	4.165	1506	WS0085_B24
3	14.500	43	1	-0.294	0.048	5.147	1506	WS0085_B24
3	17.158	45	1	0.234	0.076	7.985	1506	WS0085_B24
3	137.891	64	3	-0.321	0.051	4.935	1506	WS0085_B24
10	9.580	187	3	-0.243	0.079	3.979	1506	WS0085_B24
1	38.326	6	2	0.213	0.034	4.047	1507	WS0023_F11
2	48.839	29	1	0.237	0.065	4.792	1507	WS0023_F11
5	0.000	89	3	0.233	0.024	3.898	1507	WS0023_F11
5	16.219	93	2	0.368	0.073	8.929	1507	WS0023_F11
6	56.188	120	2	0.269	0.038	4.455	1507	WS0023_F11

7	54.200	136	2	0.269	0.071	5.488	1507	WS0023_F11
10	70.246	190	2	0.247	0.063	7.405	1507	WS0023_F11
10	78.479	193	3	-0.179	0.037	4.173	1507	WS0023_F11
10	86.151	195	3	0.214	0.040	4.415	1507	WS0023_F11
3	141.443	65	4	0.282	0.098	5.405	1508	WS0105_L14
3	154.505	69	1	0.256	0.044	4.920	1508	WS0105_L14
4	103.719	81	4	-0.180	0.033	4.947	1508	WS0105_L14
5	0.000	89	3	0.244	0.044	4.003	1508	WS0105_L14
7	15.555	130	2	0.281	0.055	4.276	1508	WS0105_L14
7	67.154	140	4	-0.245	0.082	4.598	1508	WS0105_L14
7	73.169	142	1	0.515	0.095	5.333	1508	WS0105_L14
7	74.560	143	1	-0.295	0.058	6.460	1508	WS0105_L14
7	75.883	144	2	0.413	0.094	5.025	1508	WS0105_L14
7	75.883	144	4	-0.357	0.067	7.159	1508	WS0105_L14
9	3.127	168	1	-0.367	0.048	5.631	1508	WS0105_L14
10	35.204	189	1	-0.394	0.074	8.262	1508	WS0105_L14
10	93.347	196	1	0.442	0.087	4.699	1508	WS0105_L14
11	46.026	217	1	-0.253	0.043	4.753	1508	WS0105_L14
6	76.376	122	1	-0.333	0.178	4.210	1509	WS0106_A19
9	0.000	163	1	-0.451	0.069	4.481	1509	WS0106_A19
9	36.227	165	4	-0.322	0.087	7.399	1509	WS0106_A19
10	9.580	187	3	-0.373	0.075	4.516	1509	WS0106_A19
14	0.000	246	1	-0.190	0.047	4.143	1509	WS0106_A19
2	48.839	29	1	0.210	0.033	3.859	1510	WS00923_J06
3	9.953	42	3	-0.263	0.052	6.157	1510	WS00923_J06
7	62.787	139	4	-0.191	0.036	4.537	1510	WS00923_J06
7	67.154	140	4	-0.300	0.152	3.991	1510	WS00923_J06
7	75.883	144	2	0.531	0.142	7.210	1510	WS00923_J06
7	75.883	144	4	-0.338	0.086	6.239	1510	WS00923_J06
9	0.000	166	1	-0.180	0.034	4.052	1510	WS00923_J06
9	28.608	170	3	0.283	0.054	4.749	1510	WS00923_J06
10	78.479	193	4	-0.316	0.091	6.497	1510	WS00923_J06
12	22.441	227	3	-0.212	0.041	5.277	1510	WS00923_J06
14	46.637	252	3	0.265	0.055	6.374	1510	WS00923_J06
1	40.464	7	4	0.270	0.037	3.982	1511	WS00923_M16
1	95.572	17	3	0.188	0.048	4.041	1511	WS00923_M16
3	80.211	55	4	-0.244	0.043	4.366	1511	WS00923_M16
4	55.925	76	1	0.197	0.038	4.862	1511	WS00923_M16
7	15.555	130	2	0.244	0.051	4.391	1511	WS00923_M16
9	0.000	163	1	-0.496	0.091	5.473	1511	WS00923_M16
9	59.963	177	4	-0.216	0.042	3.888	1511	WS00923_M16
11	46.026	217	1	-0.358	0.113	11.464	1511	WS00923_M16
1	40.464	7	4	0.294	0.057	5.275	1512	WS0107_K10
1	50.955	10	1	-0.261	0.032	3.951	1512	WS0107_K10
1	62.128	11	1	-0.502	0.109	4.792	1512	WS0107_K10
1	69.854	13	2	-0.302	0.088	6.167	1512	WS0107_K10
1	71.592	14	2	-0.280	0.074	5.167	1512	WS0107_K10
1	77.177	15	2	-0.241	0.054	3.861	1512	WS0107_K10
4	92.039	79	4	-0.315	0.055	4.833	1512	WS0107_K10
4	103.719	81	4	-0.235	0.029	4.100	1512	WS0107_K10
6	45.897	117	1	0.333	0.060	7.402	1512	WS0107_K10
3	14.500	43	1	0.167	0.054	4.538	1513	WS0107_K17
4	0.000	71	4	-0.309	0.072	7.013	1513	WS0107_K17
6	25.742	113	3	0.327	0.071	6.683	1513	WS0107_K17

7	15.555	130	2	0.274	0.041	4.430	1513	WS0107_K17
9	69.046	181	1	0.359	0.075	4.406	1513	WS0107_K17
10	93.347	196	1	-0.439	0.082	4.673	1513	WS0107_K17
12	28.493	228	2	0.174	0.060	4.064	1513	WS0107_K17
12	32.473	238	1	-0.197	0.057	4.827	1513	WS0107_K17
1	40.464	7	3	0.226	0.063	6.558	1514	WS0107_M20
1	43.578	8	3	0.205	0.053	5.292	1514	WS0107_M20
1	45.794	9	3	-0.355	0.088	9.306	1514	WS0107_M20
4	97.576	80	2	-0.198	0.043	4.691	1514	WS0107_M20
4	116.928	82	2	-0.279	0.056	5.831	1514	WS0107_M20
4	124.132	83	1	0.508	0.140	5.161	1514	WS0107_M20
4	142.917	85	1	-0.370	0.129	10.855	1514	WS0107_M20
6	69.073	121	3	-0.298	0.056	5.837	1514	WS0107_M20
6	106.702	128	2	0.420	0.086	5.000	1514	WS0107_M20
7	75.883	144	2	0.412	0.096	5.036	1514	WS0107_M20
7	75.883	144	4	-0.317	0.045	3.886	1514	WS0107_M20
9	0.000	163	1	-0.520	0.126	5.944	1514	WS0107_M20
10	93.347	196	1	0.442	0.100	4.694	1514	WS0107_M20
14	44.898	251	3	0.236	0.043	4.407	1514	WS0107_M20
14	46.637	252	3	0.177	0.047	4.828	1514	WS0107_M20
1	38.326	6	4	-0.205	0.039	4.098	1515	WS0107_O03
1	40.464	7	4	0.260	0.051	4.558	1515	WS0107_O03
1	95.572	17	3	0.263	0.060	5.695	1515	WS0107_O03
6	69.073	121	3	-0.205	0.053	5.172	1515	WS0107_O03
7	15.555	130	2	0.315	0.056	5.657	1515	WS0107_O03
9	0.000	163	1	-0.497	0.101	5.492	1515	WS0107_O03
9	59.963	177	4	-0.201	0.050	4.022	1515	WS0107_O03
2	126.181	39	4	-0.375	0.057	5.028	1516	WS0107_P19
4	103.719	81	4	-0.298	0.047	6.109	1516	WS0107_P19
7	15.555	130	2	0.422	0.089	10.867	1516	WS0107_P19
7	15.555	130	3	-0.235	0.054	4.231	1516	WS0107_P19
8	50.001	154	2	-0.229	0.056	6.543	1516	WS0107_P19
8	87.463	158	2	0.222	0.040	3.870	1516	WS0107_P19
9	36.227	165	4	0.220	0.044	5.702	1516	WS0107_P19
11	35.604	207	3	-0.274	0.060	4.485	1516	WS0107_P19
3	9.953	42	3	-0.271	0.047	4.860	1517	WS0108_A14
4	0.000	71	4	-0.233	0.059	5.079	1517	WS0108_A14
6	89.401	125	1	-0.278	0.046	5.147	1517	WS0108_A14
9	0.000	163	1	-0.506	0.078	5.733	1517	WS0108_A14
9	58.045	176	1	0.228	0.056	4.074	1517	WS0108_A14
10	21.864	188	1	0.238	0.035	4.008	1517	WS0108_A14
10	70.246	190	4	-0.364	0.097	5.234	1517	WS0108_A14
10	78.479	193	4	-0.311	0.109	8.393	1517	WS0108_A14
10	93.347	196	1	0.445	0.065	4.770	1517	WS0108_A14
11	9.237	202	4	0.216	0.045	3.906	1517	WS0108_A14
1	40.464	7	4	0.247	0.049	4.422	1518	WS0108_C23
1	95.572	17	3	0.311	0.068	4.803	1518	WS0108_C23
2	126.181	39	4	-0.501	0.108	3.888	1518	WS0108_C23
3	144.593	66	4	-0.240	0.043	4.478	1518	WS0108_C23
6	69.073	121	3	-0.196	0.053	4.637	1518	WS0108_C23
7	15.555	130	2	0.362	0.092	9.628	1518	WS0108_C23
9	0.000	163	1	-0.437	0.087	4.217	1518	WS0108_C23
1	95.572	17	3	0.374	0.097	9.124	1519	WS0108_D15
3	141.443	65	3	-0.239	0.066	4.415	1519	WS0108_D15

10	93.347	196	1	0.437	0.071	4.781	1519	WS0108_D15
14	0.000	246	2	0.221	0.066	5.364	1519	WS0108_D15
1	62.128	11	2	0.305	0.112	4.559	1520	WS0109_B12
2	127.147	40	3	0.205	0.055	4.531	1520	WS0109_B12
3	95.157	59	2	-0.194	0.036	3.929	1520	WS0109_B12
3	104.681	60	2	0.187	0.034	4.179	1520	WS0109_B12
7	105.477	147	2	0.350	0.091	7.198	1520	WS0109_B12
8	50.001	154	2	-0.199	0.036	3.992	1520	WS0109_B12
14	0.000	246	2	-0.205	0.041	4.809	1520	WS0109_B12
14	46.637	252	3	0.293	0.056	4.623	1520	WS0109_B12
1	62.128	11	1	-0.459	0.069	4.096	1521	WS0109_C04
3	14.500	43	1	-0.296	0.068	6.552	1521	WS0109_C04
3	17.158	45	1	0.307	0.099	9.483	1521	WS0109_C04
7	73.169	142	1	0.553	0.089	5.327	1521	WS0109_C04
7	75.883	144	2	0.478	0.185	5.742	1521	WS0109_C04
7	75.883	144	4	-0.250	0.051	4.356	1521	WS0109_C04
10	9.580	187	3	-0.271	0.113	4.539	1521	WS0109_C04
10	77.676	192	1	0.251	0.053	5.064	1521	WS0109_C04
11	35.604	207	3	-0.211	0.038	3.844	1521	WS0109_C04
11	46.026	217	1	-0.282	0.060	5.782	1521	WS0109_C04
1	95.572	17	2	-0.275	0.041	4.290	1522	WS0109_E07
4	82.462	78	2	-0.195	0.062	5.712	1522	WS0109_E07
4	92.039	79	2	-0.268	0.073	7.771	1522	WS0109_E07
4	97.576	80	2	0.252	0.066	6.210	1522	WS0109_E07
7	73.169	142	1	0.493	0.107	3.891	1522	WS0109_E07
7	75.883	144	2	0.644	0.164	10.141	1522	WS0109_E07
1	38.326	6	4	-0.392	0.083	6.466	1523	WS0109_F03
1	40.464	7	4	0.364	0.063	6.762	1523	WS0109_F03
1	95.572	17	3	0.249	0.063	5.048	1523	WS0109_F03
2	75.550	31	1	0.277	0.042	4.345	1523	WS0109_F03
3	14.500	43	1	-0.262	0.063	6.574	1523	WS0109_F03
3	17.158	45	1	0.325	0.087	9.038	1523	WS0109_F03
3	47.928	49	1	0.304	0.078	8.096	1523	WS0109_F03
4	82.462	78	1	-0.269	0.048	5.068	1523	WS0109_F03
6	89.401	125	1	-0.321	0.053	5.548	1523	WS0109_F03
7	75.883	144	2	0.458	0.127	5.389	1523	WS0109_F03
8	11.974	150	1	-0.231	0.040	4.179	1523	WS0109_F03
9	3.127	168	1	-0.353	0.078	7.047	1523	WS0109_F03
1	29.800	5	2	-0.226	0.036	3.848	1524	WS0109_H20
1	38.326	6	2	0.256	0.053	5.854	1524	WS0109_H20
1	40.464	7	4	-0.235	0.053	4.112	1524	WS0109_H20
1	43.578	8	2	-0.294	0.049	5.573	1524	WS0109_H20
2	127.147	40	3	0.242	0.066	6.870	1524	WS0109_H20
3	69.071	52	1	0.273	0.098	4.981	1524	WS0109_H20
10	104.214	198	4	0.215	0.045	4.668	1524	WS0109_H20
11	64.014	222	4	0.240	0.053	4.188	1524	WS0109_H20
6	56.188	120	2	0.304	0.051	5.174	1525	WS0109_I12
6	87.891	124	2	0.239	0.053	5.759	1525	WS0109_I12
6	95.249	126	1	0.327	0.083	4.683	1525	WS0109_I12
7	17.610	131	3	0.255	0.065	4.255	1525	WS0109_I12
8	50.001	154	2	-0.240	0.061	6.361	1525	WS0109_I12
10	9.580	187	3	-0.410	0.118	6.104	1525	WS0109_I12
10	80.088	194	2	0.224	0.038	4.495	1525	WS0109_I12
12	6.242	234	2	0.193	0.032	4.195	1525	WS0109_I12

14	43.392	250	2	-0.245	0.054	5.967	1525	WS0109_I12
1	22.419	4	3	-0.402	0.087	4.997	1526	WS0109_K01
1	38.326	6	4	-0.264	0.044	4.235	1526	WS0109_K01
1	95.572	17	3	0.324	0.059	5.993	1526	WS0109_K01
1	131.686	23	2	0.422	0.072	4.109	1526	WS0109_K01
4	103.719	81	4	-0.261	0.034	4.694	1526	WS0109_K01
5	57.017	102	2	0.204	0.049	4.688	1526	WS0109_K01
6	56.188	120	1	0.306	0.052	5.021	1526	WS0109_K01
6	87.891	124	2	0.132	0.042	3.871	1526	WS0109_K01
6	89.401	125	2	0.377	0.067	5.719	1526	WS0109_K01
9	3.127	168	1	-0.302	0.077	4.491	1526	WS0109_K01
11	56.921	219	1	-0.272	0.098	3.854	1526	WS0109_K01
14	46.637	252	4	0.249	0.046	4.740	1526	WS0109_K01
1	95.572	17	3	0.196	0.045	3.916	1527	WS0109_L04
2	75.550	31	1	0.273	0.039	3.942	1527	WS0109_L04
3	17.158	45	1	0.225	0.047	4.777	1527	WS0109_L04
6	87.891	124	2	0.206	0.052	4.516	1527	WS0109_L04
11	35.604	207	3	-0.250	0.075	6.270	1527	WS0109_L04
11	37.143	209	3	0.202	0.049	4.087	1527	WS0109_L04
11	42.600	213	3	-0.251	0.071	5.894	1527	WS0109_L04
11	46.026	217	1	-0.332	0.093	9.401	1527	WS0109_L04
2	126.181	39	4	0.404	0.061	3.966	1528	WS0265_B20
3	47.928	49	1	-0.236	0.055	4.282	1528	WS0265_B20
6	45.897	117	4	-0.233	0.037	4.264	1528	WS0265_B20
6	76.376	122	1	0.387	0.135	5.218	1528	WS0265_B20
7	73.169	142	1	-0.502	0.104	4.026	1528	WS0265_B20
9	32.356	171	2	0.208	0.042	4.641	1528	WS0265_B20
10	9.580	187	2	0.278	0.071	5.215	1528	WS0265_B20
10	35.204	189	2	-0.299	0.072	8.165	1528	WS0265_B20
11	17.088	203	2	0.196	0.039	4.243	1528	WS0265_B20
11	43.491	214	3	-0.175	0.056	4.242	1528	WS0265_B20
12	28.493	228	2	0.240	0.062	4.508	1528	WS0265_B20
1	95.572	17	3	0.341	0.090	7.629	1529	WS0265_D19
3	116.958	63	4	0.136	0.038	3.909	1529	WS0265_D19
4	124.132	83	1	0.437	0.086	3.928	1529	WS0265_D19
6	49.100	118	4	0.394	0.099	4.511	1529	WS0265_D19
6	106.702	128	3	0.254	0.056	4.203	1529	WS0265_D19
11	0.000	199	3	0.237	0.056	4.706	1529	WS0265_D19
14	22.402	248	3	-0.208	0.049	4.133	1529	WS0265_D19
4	124.132	83	1	0.372	0.055	4.235	1530	WS0265_L03
7	15.555	130	2	0.319	0.073	5.346	1530	WS0265_L03
7	73.169	142	1	0.475	0.063	4.903	1530	WS0265_L03
9	3.127	168	1	-0.395	0.072	8.196	1530	WS0265_L03
11	46.026	217	1	-0.307	0.064	7.323	1530	WS0265_L03
1	38.326	6	4	-0.290	0.037	3.935	1531	WS0265_M04
1	40.464	7	4	0.329	0.045	4.653	1531	WS0265_M04
1	95.572	17	3	0.328	0.107	8.718	1531	WS0265_M04
5	86.669	106	4	-0.402	0.089	4.077	1531	WS0265_M04
7	15.555	130	2	0.341	0.071	6.341	1531	WS0265_M04
9	3.127	168	1	-0.353	0.054	5.291	1531	WS0265_M04
2	48.839	29	4	-0.303	0.044	4.102	1532	WS0266_C11
2	127.147	40	3	-0.221	0.042	4.042	1532	WS0266_C11
3	80.211	55	4	-0.185	0.049	4.556	1532	WS0266_C11
7	15.555	130	2	0.332	0.048	4.932	1532	WS0266_C11

8	78.389	157	1	0.231	0.044	3.915	1532	WS0266_C11
11	56.921	219	1	-0.252	0.111	4.432	1532	WS0266_C11
11	64.014	222	3	-0.190	0.041	4.026	1532	WS0266_C11
7	15.555	130	2	0.298	0.051	4.870	1533	WS0266_E19
9	0.000	163	1	-0.421	0.108	3.903	1533	WS0266_E19
11	46.026	217	1	-0.261	0.056	5.176	1533	WS0266_E19
13	0.000	243	4	0.458	0.097	5.514	1533	WS0266_E19
5	52.820	99	3	-0.270	0.059	5.366	1534	WS0266_K14
6	89.401	125	2	0.208	0.036	4.097	1534	WS0266_K14
9	0.000	163	1	-0.482	0.114	5.110	1534	WS0266_K14
11	35.604	207	3	-0.278	0.078	7.875	1534	WS0266_K14
11	37.143	209	3	0.244	0.060	6.084	1534	WS0266_K14
11	46.026	217	1	-0.250	0.080	5.479	1534	WS0266_K14
11	64.014	222	3	-0.217	0.060	6.061	1534	WS0266_K14
3	80.211	55	4	-0.271	0.062	6.752	1535	WS0266_M17
4	103.719	81	4	-0.182	0.031	4.043	1535	WS0266_M17
6	89.401	125	2	0.321	0.066	6.749	1535	WS0266_M17
7	15.555	130	2	0.368	0.092	9.509	1535	WS0266_M17
7	15.555	130	3	-0.226	0.060	4.974	1535	WS0266_M17
8	62.580	155	1	0.363	0.190	6.247	1535	WS0266_M17
9	0.000	163	1	-0.437	0.080	4.218	1535	WS0266_M17
11	27.405	204	4	0.258	0.046	4.081	1535	WS0266_M17
14	9.923	247	1	0.242	0.050	4.304	1535	WS0266_M17
1	95.572	17	3	0.265	0.059	5.367	1536	WS0058_F06
9	0.000	163	1	-0.465	0.092	4.779	1536	WS0058_F06
11	46.026	217	1	-0.265	0.062	5.957	1536	WS0058_F06
3	15.147	44	2	0.257	0.067	5.209	1537	WS0058_N08
8	62.580	155	1	0.286	0.124	4.826	1537	WS0058_N08
8	78.389	157	1	0.227	0.052	4.053	1537	WS0058_N08
9	0.000	163	1	-0.548	0.168	6.619	1537	WS0058_N08
10	104.214	198	4	-0.259	0.038	4.203	1537	WS0058_N08
11	64.014	222	4	-0.325	0.054	5.742	1537	WS0058_N08
1	40.464	7	4	0.291	0.038	4.434	1538	WS00923_P23
1	95.572	17	3	0.213	0.059	4.606	1538	WS00923_P23
3	154.505	69	4	-0.221	0.040	4.410	1538	WS00923_P23
9	0.000	163	1	-0.576	0.176	7.300	1538	WS00923_P23
2	75.550	31	1	0.283	0.042	4.938	1539	WS00924_C11
3	14.500	43	1	-0.289	0.054	6.314	1539	WS00924_C11
3	17.158	45	1	0.280	0.070	8.176	1539	WS00924_C11
3	137.891	64	1	-0.249	0.034	3.949	1539	WS00924_C11
3	154.505	69	1	0.316	0.062	7.251	1539	WS00924_C11
9	3.127	168	1	-0.359	0.074	7.117	1539	WS00924_C11
11	46.026	217	1	-0.283	0.058	6.810	1539	WS00924_C11
11	64.014	222	4	-0.293	0.044	4.168	1539	WS00924_C11
1	29.800	5	1	-0.203	0.045	4.673	1540	WS0032_O12
1	40.464	7	4	0.280	0.037	3.855	1540	WS0032_O12
1	69.854	13	2	-0.301	0.059	5.198	1540	WS0032_O12
1	71.592	14	2	-0.300	0.059	5.222	1540	WS0032_O12
1	77.177	15	2	-0.330	0.078	6.766	1540	WS0032_O12
5	31.191	95	1	0.333	0.061	6.532	1540	WS0032_O12
5	86.669	106	4	-0.500	0.134	6.515	1540	WS0032_O12
7	62.787	139	4	-0.242	0.044	5.607	1540	WS0032_O12
7	67.154	140	4	-0.344	0.134	7.097	1540	WS0032_O12
7	73.169	142	1	0.521	0.084	4.437	1540	WS0032_O12

7	75.883	144	2	0.491	0.130	6.613	1540	WS0032_O12
7	75.883	144	4	-0.500	0.145	14.834	1540	WS0032_O12
8	50.001	154	2	-0.103	0.046	4.304	1540	WS0032_O12
9	17.951	169	1	-0.254	0.037	4.000	1540	WS0032_O12
12	51.382	242	1	-0.367	0.077	4.100	1540	WS0032_O12
1	82.301	16	4	0.276	0.036	3.918	1541	WS0033_B23
3	17.158	45	1	0.191	0.048	5.247	1541	WS0033_B23
3	154.505	69	1	0.223	0.039	4.397	1541	WS0033_B23
4	0.000	71	4	0.134	0.035	4.391	1541	WS0033_B23
4	82.462	78	1	-0.264	0.041	4.606	1541	WS0033_B23
5	54.072	100	4	0.237	0.044	5.494	1541	WS0033_B23
5	57.017	102	4	-0.329	0.048	6.042	1541	WS0033_B23
7	73.169	142	1	0.530	0.085	5.846	1541	WS0033_B23
7	75.883	144	2	0.364	0.066	3.884	1541	WS0033_B23
9	3.127	168	1	-0.322	0.033	4.854	1541	WS0033_B23
11	64.014	222	4	-0.324	0.043	5.404	1541	WS0033_B23
9	59.963	177	1	0.302	0.078	4.834	1542	WS0033_K04
10	80.088	194	1	-0.221	0.065	4.103	1542	WS0033_K04
11	64.014	222	4	-0.248	0.057	4.987	1542	WS0033_K04
5	86.669	106	4	-0.437	0.109	6.310	1543	WS0033_N21
6	49.100	118	4	0.436	0.102	5.184	1543	WS0033_N21
7	67.154	140	4	-0.348	0.129	7.319	1543	WS0033_N21
7	73.169	142	1	0.491	0.068	3.866	1543	WS0033_N21
7	75.883	144	2	0.475	0.143	6.455	1543	WS0033_N21
7	75.883	144	4	-0.428	0.115	11.288	1543	WS0033_N21
9	55.358	175	1	0.338	0.059	6.181	1543	WS0033_N21
9	55.358	175	3	-0.449	0.110	5.773	1543	WS0033_N21
11	46.026	217	1	-0.253	0.042	4.381	1543	WS0033_N21
14	0.000	246	1	-0.255	0.042	4.385	1543	WS0033_N21
1	38.326	6	4	-0.291	0.047	4.734	1544	WS0034_D23
1	40.464	7	4	0.293	0.041	4.527	1544	WS0034_D23
1	45.794	9	1	0.250	0.036	3.882	1544	WS0034_D23
3	14.500	43	1	-0.359	0.063	6.905	1544	WS0034_D23
3	17.158	45	1	0.250	0.072	7.743	1544	WS0034_D23
4	92.039	79	4	-0.268	0.041	4.638	1544	WS0034_D23
4	103.719	81	4	-0.221	0.035	5.650	1544	WS0034_D23
6	106.702	128	3	0.323	0.080	4.661	1544	WS0034_D23
9	58.045	176	1	0.330	0.074	3.872	1544	WS0034_D23
11	35.604	207	3	-0.254	0.077	6.080	1544	WS0034_D23
11	37.143	209	3	0.229	0.062	4.909	1544	WS0034_D23
11	42.600	213	3	-0.230	0.060	4.721	1544	WS0034_D23
11	46.026	217	1	-0.280	0.048	5.225	1544	WS0034_D23
1	40.464	7	4	0.283	0.040	4.139	1545	WS0034_F05
1	62.128	11	1	-0.520	0.096	5.199	1545	WS0034_F05
1	131.686	23	2	0.410	0.086	3.912	1545	WS0034_F05
3	141.443	65	4	0.294	0.086	4.774	1545	WS0034_F05
4	103.719	81	4	-0.244	0.053	7.019	1545	WS0034_F05
6	45.897	117	1	0.183	0.041	4.197	1545	WS0034_F05
6	87.891	124	2	0.265	0.082	7.150	1545	WS0034_F05
6	89.401	125	2	0.341	0.087	4.758	1545	WS0034_F05
7	67.154	140	4	-0.298	0.089	4.776	1545	WS0034_F05
11	46.026	217	1	-0.268	0.061	6.108	1545	WS0034_F05
3	17.158	45	1	0.193	0.048	4.531	1546	WS0034_N23
9	58.045	176	1	0.321	0.110	4.108	1546	WS0034_N23

11	46.026	217	1	-0.238	0.041	3.935	1546	WS0034_N23
1	69.854	13	2	0.219	0.051	5.279	1547	WS0034_O18
1	71.592	14	2	0.229	0.057	5.857	1547	WS0034_O18
1	77.177	15	2	0.222	0.053	5.412	1547	WS0034_O18
1	95.572	17	3	0.237	0.065	3.844	1547	WS0034_O18
5	84.347	105	2	0.243	0.075	5.867	1547	WS0034_O18
5	86.669	106	2	-0.305	0.114	5.421	1547	WS0034_O18
7	75.883	144	2	0.598	0.146	9.283	1547	WS0034_O18
7	75.883	144	4	-0.375	0.076	6.773	1547	WS0034_O18
9	55.358	175	1	0.363	0.070	6.163	1547	WS0034_O18
9	55.358	175	3	-0.474	0.102	6.056	1547	WS0034_O18
9	58.045	176	1	0.215	0.080	4.285	1547	WS0034_O18
11	46.026	217	1	-0.209	0.049	4.149	1547	WS0034_O18
14	0.000	246	1	-0.231	0.058	4.960	1547	WS0034_O18
2	95.880	34	3	0.219	0.064	4.072	1548	WS00924_O22
3	141.443	65	4	0.216	0.092	4.996	1548	WS00924_O22
6	87.891	124	2	0.204	0.045	4.332	1548	WS00924_O22
6	89.401	125	1	-0.357	0.055	5.102	1548	WS00924_O22
6	89.401	125	2	0.239	0.055	5.497	1548	WS00924_O22
7	15.555	130	2	0.300	0.078	7.521	1548	WS00924_O22
7	62.787	139	4	-0.130	0.023	3.880	1548	WS00924_O22
7	67.154	140	4	-0.225	0.100	4.639	1548	WS00924_O22
7	74.560	143	1	-0.151	0.044	3.957	1548	WS00924_O22
7	75.883	144	2	0.452	0.091	6.539	1548	WS00924_O22
7	75.883	144	4	-0.428	0.070	8.055	1548	WS00924_O22
9	0.000	163	1	-0.634	0.200	8.865	1548	WS00924_O22
10	35.204	189	1	-0.371	0.062	5.788	1548	WS00924_O22
11	46.026	217	1	-0.217	0.043	3.890	1548	WS00924_O22
1	95.572	17	3	0.197	0.051	4.126	1549	WS00925_C17
3	141.443	65	4	0.277	0.079	4.404	1549	WS00925_C17
11	46.026	217	1	-0.311	0.092	9.385	1549	WS00925_C17
1	40.464	7	4	0.321	0.045	4.279	1550	WS0024_P05
1	43.578	8	2	0.221	0.047	4.532	1550	WS0024_P05
1	77.177	15	2	-0.196	0.053	5.039	1550	WS0024_P05
1	107.333	19	4	-0.256	0.046	5.115	1550	WS0024_P05
3	15.147	44	2	-0.256	0.071	4.186	1550	WS0024_P05
3	141.443	65	4	0.255	0.084	4.160	1550	WS0024_P05
4	142.917	85	1	-0.294	0.071	6.512	1550	WS0024_P05
7	62.787	139	4	-0.216	0.040	6.502	1550	WS0024_P05
7	67.154	140	4	-0.249	0.085	4.260	1550	WS0024_P05
7	75.883	144	2	0.526	0.119	8.459	1550	WS0024_P05
7	75.883	144	4	-0.338	0.053	6.023	1550	WS0024_P05
9	59.963	177	4	-0.286	0.064	6.334	1550	WS0024_P05
10	78.479	193	4	-0.297	0.057	6.054	1550	WS0024_P05
11	46.026	217	1	-0.275	0.072	6.604	1550	WS0024_P05
1	38.326	6	4	-0.328	0.045	5.343	1551	WS01018_P09
1	40.464	7	4	0.384	0.059	8.214	1551	WS01018_P09
1	43.578	8	2	0.203	0.040	4.017	1551	WS01018_P09
7	15.555	130	2	0.239	0.044	4.337	1551	WS01018_P09
7	75.883	144	2	0.383	0.059	4.496	1551	WS01018_P09
7	75.883	144	4	-0.342	0.042	5.486	1551	WS01018_P09
9	0.000	163	1	-0.537	0.195	6.356	1551	WS01018_P09
9	59.963	177	4	-0.309	0.082	10.961	1551	WS01018_P09
11	46.026	217	1	-0.292	0.078	6.952	1551	WS01018_P09

1	43.578	8	2	-0.222	0.038	4.073	1552	WS0031_M18
3	95.157	59	2	-0.221	0.041	3.989	1552	WS0031_M18
4	55.925	76	1	0.189	0.056	4.296	1552	WS0031_M18
6	81.996	123	3	-0.341	0.089	4.002	1552	WS0031_M18
8	50.001	154	2	-0.211	0.036	3.939	1552	WS0031_M18
9	2.011	167	4	0.222	0.058	5.633	1552	WS0031_M18
11	9.237	202	4	0.201	0.043	4.126	1552	WS0031_M18
11	46.026	217	1	-0.248	0.090	7.329	1552	WS0031_M18
1	45.794	9	3	-0.239	0.051	4.587	1553	WS01019_H09
4	75.958	77	3	-0.244	0.090	4.651	1553	WS01019_H09
5	54.072	100	4	-0.180	0.041	3.863	1553	WS01019_H09
5	86.669	106	4	-0.401	0.131	5.131	1553	WS01019_H09
6	49.100	118	4	0.400	0.133	5.472	1553	WS01019_H09
6	54.066	119	4	0.252	0.067	6.278	1553	WS01019_H09
6	69.073	121	4	-0.259	0.051	4.375	1553	WS01019_H09
7	75.883	144	2	0.415	0.082	4.644	1553	WS01019_H09
9	0.000	163	1	-0.493	0.126	5.349	1553	WS01019_H09
10	78.479	193	3	-0.227	0.045	4.215	1553	WS01019_H09
11	35.604	207	3	-0.175	0.041	3.841	1553	WS01019_H09
11	37.143	209	3	0.179	0.042	3.992	1553	WS01019_H09
14	46.637	252	4	0.357	0.085	8.488	1553	WS01019_H09
1	95.572	17	3	0.300	0.069	6.638	1554	WS01014_N08
1	107.333	19	3	0.151	0.039	4.088	1554	WS01014_N08
3	83.563	56	3	0.162	0.038	4.032	1554	WS01014_N08
3	154.505	69	1	-0.377	0.110	9.415	1554	WS01014_N08
5	16.219	93	2	-0.222	0.057	5.990	1554	WS01014_N08
6	81.996	123	3	-0.319	0.095	6.047	1554	WS01014_N08
7	15.555	130	3	0.304	0.063	6.783	1554	WS01014_N08
7	17.610	131	3	0.248	0.132	5.570	1554	WS01014_N08
7	70.278	141	3	0.256	0.052	5.576	1554	WS01014_N08
7	91.487	146	3	0.156	0.041	4.325	1554	WS01014_N08
8	87.463	158	2	0.221	0.061	6.280	1554	WS01014_N08
9	17.951	169	2	0.311	0.068	5.242	1554	WS01014_N08
9	69.046	181	1	0.318	0.093	5.948	1554	WS01014_N08
12	14.406	230	3	-0.289	0.051	5.412	1554	WS01014_N08
1	40.464	7	4	0.312	0.042	4.554	1555	WS01014_N23
2	95.880	34	3	0.205	0.045	4.268	1555	WS01014_N23
2	101.076	35	3	-0.254	0.063	5.455	1555	WS01014_N23
3	47.928	49	1	0.236	0.060	5.447	1555	WS01014_N23
4	103.719	81	4	-0.186	0.044	5.297	1555	WS01014_N23
6	49.100	118	4	0.431	0.109	4.334	1555	WS01014_N23
7	15.555	130	2	0.281	0.059	5.761	1555	WS01014_N23
9	0.000	163	1	-0.632	0.183	8.901	1555	WS01014_N23
9	59.963	177	4	-0.252	0.059	5.554	1555	WS01014_N23
10	70.246	190	2	0.230	0.045	4.446	1555	WS01014_N23
10	80.088	194	2	-0.326	0.067	5.753	1555	WS01014_N23
10	93.347	196	2	0.442	0.120	4.421	1555	WS01014_N23
11	46.026	217	1	-0.248	0.052	4.727	1555	WS01014_N23
12	51.382	242	1	-0.356	0.092	3.854	1555	WS01014_N23
3	148.745	68	4	0.221	0.034	3.904	1556	WS01014_P20
3	154.505	69	4	-0.229	0.042	4.991	1556	WS01014_P20
9	0.000	163	1	-0.506	0.133	5.649	1556	WS01014_P20
9	28.608	170	3	0.264	0.062	3.887	1556	WS01014_P20
11	46.026	217	1	-0.220	0.077	6.012	1556	WS01014_P20

11	57.661	220	4	0.200	0.044	5.221	1556	WS01014_P20
11	64.014	222	4	-0.275	0.059	7.193	1556	WS01014_P20
9	59.963	177	4	-0.220	0.040	4.158	1557	WS01015_A04
10	78.479	193	3	-0.493	0.127	10.524	1557	WS01015_A04
14	0.000	246	2	0.254	0.056	6.329	1557	WS01015_A04
3	15.147	44	2	0.291	0.091	5.534	1558	WS01015_C01
3	17.158	45	1	0.204	0.047	4.116	1558	WS01015_C01
3	154.505	69	1	0.258	0.053	4.646	1558	WS01015_C01
7	105.477	147	2	-0.246	0.067	4.114	1558	WS01015_C01
9	3.127	168	1	-0.341	0.081	6.370	1558	WS01015_C01
10	35.204	189	2	0.244	0.059	4.888	1558	WS01015_C01
3	15.147	44	2	0.408	0.107	8.788	1559	WS01015_C18
4	124.132	83	1	0.599	0.237	7.244	1559	WS01015_C18
5	42.979	97	2	-0.238	0.037	4.419	1559	WS01015_C18
11	35.604	207	3	0.247	0.067	5.093	1559	WS01015_C18
11	64.014	222	4	-0.295	0.040	4.464	1559	WS01015_C18
2	75.550	31	1	0.287	0.043	4.678	1560	WS01015_E24
3	17.158	45	1	0.199	0.037	4.061	1560	WS01015_E24
5	54.072	100	4	0.228	0.045	4.145	1560	WS01015_E24
5	57.017	102	4	-0.288	0.064	5.713	1560	WS01015_E24
6	106.702	128	3	0.227	0.045	4.381	1560	WS01015_E24
7	59.789	137	3	-0.227	0.074	7.122	1560	WS01015_E24
11	35.604	207	3	-0.195	0.054	5.224	1560	WS01015_E24
11	37.143	209	3	0.167	0.041	3.982	1560	WS01015_E24
11	42.600	213	3	-0.169	0.040	3.842	1560	WS01015_E24
11	46.026	217	1	-0.301	0.068	7.408	1560	WS01015_E24
11	57.661	220	4	0.265	0.058	5.366	1560	WS01015_E24
11	64.014	222	4	-0.430	0.113	10.466	1560	WS01015_E24
12	0.000	233	1	-0.202	0.043	4.626	1560	WS01015_E24
12	6.242	234	2	-0.211	0.048	4.282	1560	WS01015_E24
1	107.333	19	4	-0.241	0.039	4.396	1561	WS01015_F12
1	123.316	20	4	-0.303	0.078	5.552	1561	WS01015_F12
1	127.805	21	3	-0.230	0.053	4.085	1561	WS01015_F12
3	144.593	66	4	0.256	0.044	5.224	1561	WS01015_F12
3	148.745	68	4	-0.249	0.050	5.578	1561	WS01015_F12
6	25.914	114	1	-0.192	0.056	4.049	1561	WS01015_F12
11	35.604	207	3	-0.169	0.055	4.224	1561	WS01015_F12
11	37.143	209	3	0.161	0.050	3.885	1561	WS01015_F12
11	46.026	217	1	-0.286	0.079	5.782	1561	WS01015_F12
12	38.543	232	4	-0.367	0.099	3.904	1561	WS01015_F12
1	82.301	16	4	0.323	0.045	4.177	1562	WS0055_L21
4	142.917	85	1	-0.206	0.065	4.302	1562	WS0055_L21
5	7.672	92	4	0.337	0.058	5.618	1562	WS0055_L21
7	15.555	130	3	-0.285	0.060	5.429	1562	WS0055_L21
9	36.227	165	4	0.370	0.059	6.292	1562	WS0055_L21
12	38.543	232	4	-0.399	0.106	3.892	1562	WS0055_L21
1	38.326	6	4	-0.349	0.041	5.120	1563	WS0055_N07
1	40.464	7	4	0.346	0.046	4.842	1563	WS0055_N07
1	45.794	9	1	0.327	0.060	5.750	1563	WS0055_N07
1	50.955	10	1	-0.251	0.041	3.894	1563	WS0055_N07
2	75.550	31	1	0.288	0.041	3.932	1563	WS0055_N07
3	17.158	45	1	0.215	0.072	6.729	1563	WS0055_N07
6	25.914	114	1	-0.197	0.061	5.677	1563	WS0055_N07
7	73.169	142	1	0.448	0.073	4.117	1563	WS0055_N07

7	75.883	144	2	0.387	0.071	4.396	1563	WS0055_N07
9	3.127	168	1	-0.338	0.036	4.810	1563	WS0055_N07
11	46.026	217	1	-0.274	0.046	4.374	1563	WS0055_N07
6	25.914	114	1	-0.202	0.061	5.989	1564	WS0045_C04
6	81.996	123	4	0.438	0.091	4.939	1564	WS0045_C04
7	75.883	144	4	-0.392	0.077	5.080	1564	WS0045_C04
9	0.000	166	2	-0.239	0.045	3.859	1564	WS0045_C04
10	35.204	189	1	-0.236	0.042	4.138	1564	WS0045_C04
14	0.000	246	1	-0.291	0.054	5.439	1564	WS0045_C04
1	40.464	7	4	0.296	0.037	3.974	1565	WS0045_D02
2	75.550	31	1	0.307	0.042	4.340	1565	WS0045_D02
3	17.158	45	1	0.205	0.049	4.906	1565	WS0045_D02
4	103.719	81	4	-0.150	0.030	4.340	1565	WS0045_D02
6	45.897	117	1	0.204	0.042	4.284	1565	WS0045_D02
6	106.702	128	3	0.226	0.058	4.659	1565	WS0045_D02
9	58.045	176	1	0.305	0.108	5.521	1565	WS0045_D02
11	46.026	217	1	-0.236	0.039	3.972	1565	WS0045_D02
1	2.788	2	4	-0.167	0.045	4.159	1566	WS0045_F20
4	55.925	76	1	-0.231	0.053	4.502	1566	WS0045_F20
4	103.719	81	4	0.224	0.064	5.546	1566	WS0045_F20
12	38.543	232	4	-0.292	0.183	4.783	1566	WS0045_F20
1	95.572	17	3	0.266	0.078	4.424	1567	WS0045_H07
1	107.333	19	4	-0.264	0.065	7.446	1567	WS0045_H07
1	123.316	20	4	-0.543	0.175	15.916	1567	WS0045_H07
1	127.805	21	4	0.226	0.063	7.503	1567	WS0045_H07
1	131.686	23	2	0.695	0.127	11.070	1567	WS0045_H07
6	45.897	117	1	0.239	0.077	4.621	1567	WS0045_H07
9	81.073	183	1	0.298	0.066	3.930	1567	WS0045_H07
11	17.088	203	2	0.239	0.046	5.223	1567	WS0045_H07
11	41.984	212	2	0.195	0.033	4.276	1567	WS0045_H07
11	43.491	214	4	0.196	0.038	4.533	1567	WS0045_H07
1	2.788	2	4	0.200	0.044	4.017	1568	WS0045_M18
1	40.464	7	3	-0.157	0.049	3.968	1568	WS0045_M18
1	43.578	8	3	-0.154	0.047	3.861	1568	WS0045_M18
2	75.550	31	2	0.441	0.132	14.166	1568	WS0045_M18
2	93.504	32	2	0.184	0.049	5.494	1568	WS0045_M18
2	93.504	32	4	-0.273	0.056	5.722	1568	WS0045_M18
2	101.076	35	4	0.195	0.033	4.100	1568	WS0045_M18
2	126.181	39	2	-0.323	0.053	5.887	1568	WS0045_M18
4	0.000	71	4	-0.245	0.040	4.300	1568	WS0045_M18
6	45.897	117	1	0.388	0.124	9.817	1568	WS0045_M18
6	49.100	118	4	0.457	0.123	4.939	1568	WS0045_M18
8	39.090	152	4	-0.301	0.072	7.695	1568	WS0045_M18
2	48.839	29	1	-0.295	0.060	6.625	1569	WS0045_N09
2	127.147	40	3	0.195	0.042	4.843	1569	WS0045_N09
5	7.672	92	1	-0.266	0.057	6.165	1569	WS0045_N09
5	16.219	93	1	-0.204	0.039	4.344	1569	WS0045_N09
6	45.897	117	4	0.267	0.088	6.320	1569	WS0045_N09
6	106.702	128	3	-0.223	0.038	4.106	1569	WS0045_N09
8	50.001	154	2	-0.264	0.094	4.054	1569	WS0045_N09
9	0.000	166	1	0.224	0.048	5.310	1569	WS0045_N09
9	58.045	176	1	-0.243	0.049	4.218	1569	WS0045_N09
9	69.046	181	1	0.320	0.068	5.916	1569	WS0045_N09
14	22.402	248	4	-0.380	0.107	7.018	1569	WS0045_N09

14	46.637	252	4	-0.361	0.106	7.420	1569	WS0045_N09
1	95.572	17	3	0.259	0.044	4.127	1570	WS0084_A13
3	17.158	45	1	0.218	0.051	5.369	1570	WS0084_A13
6	25.914	114	1	-0.243	0.057	6.085	1570	WS0084_A13
6	45.897	117	1	0.285	0.057	6.059	1570	WS0084_A13
7	59.789	137	3	-0.189	0.043	4.062	1570	WS0084_A13
7	91.487	146	3	0.228	0.046	4.305	1570	WS0084_A13
11	35.604	207	3	-0.264	0.083	7.732	1570	WS0084_A13
11	37.143	209	3	0.226	0.061	5.748	1570	WS0084_A13
11	42.600	213	3	-0.237	0.062	5.813	1570	WS0084_A13
11	46.026	217	1	-0.303	0.063	6.757	1570	WS0084_A13
6	25.742	113	3	0.207	0.040	3.908	1571	WS0084_F24
7	15.555	130	2	0.291	0.062	5.463	1571	WS0084_F24
9	0.000	163	1	-0.500	0.085	5.505	1571	WS0084_F24
2	48.839	29	4	-0.286	0.064	4.040	1572	WS01015_H13
5	7.672	92	1	-0.301	0.078	5.876	1572	WS01015_H13
7	67.154	140	4	0.341	0.193	4.917	1572	WS01015_H13
7	75.883	144	2	-0.396	0.074	3.949	1572	WS01015_H13
7	75.883	144	4	0.353	0.087	6.736	1572	WS01015_H13
9	28.608	170	3	-0.288	0.065	7.227	1572	WS01015_H13
10	70.246	190	2	-0.239	0.037	4.070	1572	WS01015_H13
11	43.491	214	3	0.239	0.045	5.092	1572	WS01015_H13
1	62.128	11	1	-0.578	0.163	6.724	1573	WS01015_M16
2	48.839	29	4	-0.324	0.044	5.628	1573	WS01015_M16
2	126.181	39	4	-0.368	0.050	4.502	1573	WS01015_M16
3	9.953	42	4	0.291	0.047	5.842	1573	WS01015_M16
4	82.462	78	1	-0.173	0.065	4.505	1573	WS01015_M16
6	56.188	120	2	0.271	0.039	4.384	1573	WS01015_M16
6	89.401	125	2	0.297	0.043	4.520	1573	WS01015_M16
7	70.278	141	3	0.256	0.064	3.855	1573	WS01015_M16
8	62.580	155	1	0.383	0.132	5.415	1573	WS01015_M16
8	78.389	157	1	0.272	0.069	4.759	1573	WS01015_M16
9	0.000	166	1	0.180	0.066	4.545	1573	WS01015_M16
9	28.608	170	2	0.357	0.080	10.045	1573	WS01015_M16
9	32.356	171	2	0.218	0.036	4.344	1573	WS01015_M16
9	49.013	173	2	-0.194	0.040	4.899	1573	WS01015_M16
10	70.246	190	2	0.226	0.059	7.077	1573	WS01015_M16
11	40.960	210	2	0.415	0.054	4.830	1573	WS01015_M16
11	43.491	214	4	0.144	0.034	4.591	1573	WS01015_M16
13	1.815	244	4	0.253	0.037	4.791	1573	WS01015_M16
14	0.000	246	2	0.230	0.043	5.035	1573	WS01015_M16
3	47.928	49	1	0.257	0.082	5.643	1574	WS0035_E24
3	148.745	68	1	-0.173	0.056	4.071	1574	WS0035_E24
4	75.958	77	3	-0.229	0.085	4.524	1574	WS0035_E24
4	103.719	81	4	-0.349	0.112	12.012	1574	WS0035_E24
4	116.928	82	4	0.345	0.045	4.070	1574	WS0035_E24
8	39.090	152	4	0.231	0.045	4.459	1574	WS0035_E24
9	2.011	167	4	-0.179	0.055	5.330	1574	WS0035_E24
10	9.580	187	2	-0.352	0.064	4.326	1574	WS0035_E24
10	78.479	193	4	-0.296	0.062	6.092	1574	WS0035_E24
11	17.088	203	1	-0.231	0.065	4.490	1574	WS0035_E24
11	57.661	220	4	0.288	0.046	4.615	1574	WS0035_E24
11	64.014	222	4	-0.362	0.073	6.144	1574	WS0035_E24
1	77.177	15	2	0.166	0.045	4.518	1575	IS0011_I22

4	116.928	82	2	-0.292	0.045	4.731	1575	IS0011_I22
4	124.132	83	1	0.640	0.170	8.826	1575	IS0011_I22
4	142.917	85	1	-0.303	0.109	9.305	1575	IS0011_I22
5	57.017	102	2	0.329	0.067	7.051	1575	IS0011_I22
6	49.100	118	2	-0.199	0.114	3.994	1575	IS0011_I22
7	70.278	141	3	-0.349	0.117	8.147	1575	IS0011_I22
7	91.487	146	3	-0.192	0.065	4.414	1575	IS0011_I22
9	36.227	165	4	0.342	0.032	8.063	1575	IS0011_I22
9	3.127	168	1	-0.456	0.084	5.871	1575	IS0011_I22
3	80.211	55	4	-0.214	0.049	4.100	1576	WS0043_G09
4	75.958	77	3	-0.350	0.110	7.311	1576	WS0043_G09
4	173.929	88	2	-0.242	0.048	5.413	1576	WS0043_G09
6	25.914	114	1	-0.240	0.081	6.028	1576	WS0043_G09
6	87.891	124	2	0.186	0.042	4.794	1576	WS0043_G09
6	89.401	125	2	0.238	0.038	3.981	1576	WS0043_G09
6	106.702	128	2	-0.478	0.089	5.990	1576	WS0043_G09
7	17.610	131	3	-0.300	0.065	4.308	1576	WS0043_G09
10	78.479	193	3	0.259	0.038	3.934	1576	WS0043_G09
11	35.604	207	3	-0.199	0.041	4.240	1576	WS0043_G09
11	42.600	213	3	-0.304	0.086	8.951	1576	WS0043_G09
11	46.026	217	1	-0.378	0.130	9.747	1576	WS0043_G09
1	43.578	8	2	0.243	0.060	5.372	1577	WS00927_H05
3	14.500	43	1	-0.249	0.046	4.561	1577	WS00927_H05
3	47.928	49	1	0.262	0.077	7.591	1577	WS00927_H05
5	7.672	92	1	-0.306	0.059	5.661	1577	WS00927_H05
5	7.672	92	4	0.382	0.110	9.559	1577	WS00927_H05
5	84.347	105	1	0.184	0.041	4.016	1577	WS00927_H05
9	49.013	173	1	-0.228	0.045	3.950	1577	WS00927_H05
9	59.963	177	1	-0.352	0.105	10.717	1577	WS00927_H05
9	61.113	178	1	-0.309	0.130	3.870	1577	WS00927_H05
9	81.073	183	1	-0.275	0.049	4.906	1577	WS00927_H05
10	70.246	190	4	-0.297	0.070	5.656	1577	WS00927_H05
11	17.088	203	1	0.244	0.046	4.591	1577	WS00927_H05
12	6.242	234	2	0.250	0.053	5.260	1577	WS00927_H05
13	0.000	243	4	0.380	0.146	4.377	1577	WS00927_H05
14	44.898	251	3	0.182	0.039	4.333	1577	WS00927_H05
14	46.637	252	3	0.284	0.044	4.700	1577	WS00927_H05
6	87.891	124	2	0.212	0.065	6.804	1578	WS0043_O15
6	89.401	125	2	0.411	0.102	10.294	1578	WS0043_O15
7	59.789	137	3	-0.240	0.051	4.439	1578	WS0043_O15
9	81.073	183	3	-0.255	0.055	4.391	1578	WS0043_O15
11	17.088	203	2	0.177	0.044	4.670	1578	WS0043_O15
11	35.604	207	3	-0.262	0.061	5.289	1578	WS0043_O15
11	37.143	209	3	0.231	0.045	3.921	1578	WS0043_O15
11	42.600	213	3	-0.236	0.045	3.938	1578	WS0043_O15
14	22.402	248	4	-0.217	0.044	4.706	1578	WS0043_O15
14	43.392	250	1	-0.320	0.091	4.428	1578	WS0043_O15
1	62.128	11	1	-0.457	0.071	4.072	1579	WS00927_J20
2	75.550	31	1	0.300	0.042	4.719	1579	WS00927_J20
3	14.500	43	1	-0.258	0.041	4.546	1579	WS00927_J20
3	17.158	45	1	0.212	0.055	6.128	1579	WS00927_J20
4	92.039	79	4	-0.302	0.034	3.866	1579	WS00927_J20
9	3.127	168	1	-0.363	0.044	5.068	1579	WS00927_J20
1	43.578	8	2	0.219	0.047	6.154	1580	WS00910_M24

1	82.301	16	4	0.245	0.035	4.097	1580	WS00910_M24
3	14.500	43	2	0.219	0.038	3.892	1580	WS00910_M24
4	82.462	78	2	0.212	0.030	4.205	1580	WS00910_M24
4	97.576	80	2	-0.184	0.023	3.973	1580	WS00910_M24
5	56.008	101	2	-0.273	0.046	5.960	1580	WS00910_M24
5	57.017	102	2	0.283	0.049	6.526	1580	WS00910_M24
6	26.350	115	2	0.204	0.049	6.421	1580	WS00910_M24
11	45.989	216	2	-0.168	0.034	4.642	1580	WS00910_M24
1	50.955	10	1	-0.249	0.058	5.108	1581	WS00910_N15
1	95.572	17	2	-0.224	0.050	4.953	1581	WS00910_N15
1	95.572	17	3	0.244	0.057	6.023	1581	WS00910_N15
3	14.500	43	1	-0.283	0.072	6.422	1581	WS00910_N15
3	17.158	45	1	0.232	0.055	4.874	1581	WS00910_N15
5	52.820	99	3	0.197	0.040	4.289	1581	WS00910_N15
7	59.789	137	3	-0.176	0.049	5.153	1581	WS00910_N15
9	55.358	175	3	-0.526	0.109	7.404	1581	WS00910_N15
11	46.026	217	1	-0.320	0.090	8.003	1581	WS00910_N15
14	0.000	246	1	-0.245	0.052	4.575	1581	WS00910_N15
1	50.955	10	1	-0.265	0.052	4.982	1582	WS00910_P19
1	131.686	23	2	0.463	0.082	5.243	1582	WS00910_P19
5	57.017	102	4	-0.272	0.045	5.217	1582	WS00910_P19
9	58.045	176	1	0.316	0.103	4.567	1582	WS00910_P19
10	86.151	195	3	0.174	0.057	4.204	1582	WS00910_P19
11	64.014	222	4	-0.347	0.053	5.342	1582	WS00910_P19
14	22.402	248	3	0.275	0.075	5.509	1582	WS00910_P19
5	3.484	90	2	-0.319	0.089	3.858	1583	WS00911_A09
5	16.219	93	2	0.321	0.076	5.587	1583	WS00911_A09
7	75.883	144	2	0.469	0.125	6.080	1583	WS00911_A09
7	75.883	144	4	-0.399	0.077	6.772	1583	WS00911_A09
9	58.045	176	1	0.238	0.070	5.202	1583	WS00911_A09
9	69.046	181	1	-0.341	0.059	4.481	1583	WS00911_A09
10	35.204	189	1	-0.296	0.040	4.744	1583	WS00911_A09
10	93.347	196	1	0.498	0.080	6.083	1583	WS00911_A09
11	45.989	216	1	-0.237	0.037	4.317	1583	WS00911_A09
4	82.462	78	2	-0.263	0.075	3.976	1584	WS00911_C10
6	25.914	114	1	0.217	0.033	4.097	1584	WS00911_C10
9	58.045	176	1	-0.213	0.044	4.582	1584	WS00911_C10
9	59.963	177	1	-0.318	0.058	7.160	1584	WS00911_C10
9	69.046	181	1	0.319	0.051	5.457	1584	WS00911_C10
10	35.204	189	1	0.258	0.034	4.168	1584	WS00911_C10
11	40.960	210	2	-0.411	0.110	4.514	1584	WS00911_C10
1	29.800	5	1	0.301	0.069	6.649	1585	WS00911_C19
1	127.805	21	3	0.290	0.061	7.299	1585	WS00911_C19
3	20.512	46	2	0.183	0.043	3.906	1585	WS00911_C19
3	95.157	59	2	-0.268	0.078	7.377	1585	WS00911_C19
3	104.681	60	2	0.184	0.046	4.207	1585	WS00911_C19
4	43.146	74	3	-0.212	0.041	4.874	1585	WS00911_C19
7	15.555	130	3	0.335	0.073	8.284	1585	WS00911_C19
8	0.000	148	4	0.237	0.066	4.581	1585	WS00911_C19
9	0.000	166	1	0.278	0.051	4.948	1585	WS00911_C19
9	69.046	181	1	0.230	0.056	4.448	1585	WS00911_C19
10	35.204	189	1	0.197	0.041	3.956	1585	WS00911_C19
11	0.000	199	2	0.229	0.043	3.926	1585	WS00911_C19
11	40.960	210	2	-0.546	0.142	8.149	1585	WS00911_C19

1	107.333	19	3	0.223	0.046	4.462	1586	WS00911_E24
5	57.017	102	2	-0.285	0.047	4.378	1586	WS00911_E24
5	86.669	106	2	-0.326	0.135	3.951	1586	WS00911_E24
7	75.883	144	2	-0.508	0.098	6.688	1586	WS00911_E24
11	9.237	202	4	0.172	0.044	4.049	1586	WS00911_E24
11	27.405	204	4	-0.270	0.056	5.261	1586	WS00911_E24
11	42.600	213	3	0.292	0.067	6.513	1586	WS00911_E24
11	64.014	222	4	0.281	0.058	5.549	1586	WS00911_E24
3	17.158	45	1	0.177	0.042	3.986	1587	WS00911_F18
6	45.897	117	1	0.254	0.048	4.532	1587	WS00911_F18
7	75.883	144	4	-0.300	0.053	4.067	1587	WS00911_F18
11	64.014	222	3	-0.285	0.071	6.740	1587	WS00911_F18
1	15.589	3	2	-0.182	0.036	4.219	1588	WS0086_I13
1	133.992	24	2	-0.206	0.035	4.088	1588	WS0086_I13
3	15.147	44	2	-0.233	0.050	3.909	1588	WS0086_I13
4	116.928	82	2	0.295	0.053	6.184	1588	WS0086_I13
11	41.984	212	2	-0.182	0.033	3.883	1588	WS0086_I13
11	45.989	216	2	-0.234	0.052	5.114	1588	WS0086_I13
11	60.464	221	2	-0.261	0.050	5.865	1588	WS0086_I13
1	40.464	7	4	0.306	0.056	5.555	1589	WS0086_J08
1	95.572	17	3	0.226	0.062	5.453	1589	WS0086_J08
3	80.211	55	4	-0.223	0.041	4.104	1589	WS0086_J08
7	15.555	130	2	0.215	0.040	4.300	1589	WS0086_J08
7	74.560	143	1	-0.197	0.058	4.535	1589	WS0086_J08
7	75.883	144	2	0.372	0.054	3.863	1589	WS0086_J08
9	59.963	177	4	-0.263	0.058	5.873	1589	WS0086_J08
10	78.479	193	3	-0.218	0.045	3.984	1589	WS0086_J08
14	43.392	250	2	0.180	0.043	4.750	1589	WS0086_J08
14	46.637	252	4	0.244	0.043	4.481	1589	WS0086_J08
2	120.617	38	3	0.246	0.055	6.123	1590	WS0086_L12
2	127.147	40	3	-0.171	0.046	5.129	1590	WS0086_L12
4	75.958	77	3	-0.338	0.076	4.624	1590	WS0086_L12
4	124.132	83	1	0.437	0.126	3.864	1590	WS0086_L12
6	45.897	117	1	0.226	0.057	4.502	1590	WS0086_L12
6	49.100	118	4	0.496	0.232	7.419	1590	WS0086_L12
6	54.066	119	4	0.200	0.076	5.817	1590	WS0086_L12
7	17.610	131	3	0.366	0.081	4.987	1590	WS0086_L12
9	0.000	163	1	-0.556	0.134	6.863	1590	WS0086_L12
10	78.479	193	3	-0.478	0.086	9.583	1590	WS0086_L12
11	35.604	207	3	-0.156	0.040	4.444	1590	WS0086_L12
11	42.600	213	3	-0.165	0.042	4.623	1590	WS0086_L12
4	0.000	71	4	-0.182	0.038	4.116	1591	WS0086_M12
5	7.672	92	4	-0.258	0.059	4.359	1591	WS0086_M12
5	54.072	100	4	-0.259	0.045	4.923	1591	WS0086_M12
5	57.017	102	4	0.311	0.049	5.158	1591	WS0086_M12
6	49.100	118	4	0.381	0.201	8.771	1591	WS0086_M12
6	54.066	119	4	0.298	0.052	5.716	1591	WS0086_M12
9	59.963	177	4	-0.283	0.069	7.458	1591	WS0086_M12
11	60.464	221	1	-0.233	0.051	4.552	1591	WS0086_M12
13	1.815	244	2	-0.269	0.046	4.723	1591	WS0086_M12
1	43.578	8	2	0.217	0.044	5.490	1592	WS0086_O23
3	95.157	59	2	0.248	0.061	8.524	1592	WS0086_O23
7	75.883	144	2	0.483	0.068	6.453	1592	WS0086_O23
9	0.000	163	1	-0.449	0.198	4.427	1592	WS0086_O23

9	59.963	177	4	-0.223	0.052	7.064	1592	WS0086_O23
14	43.392	250	2	0.221	0.044	5.336	1592	WS0086_O23
14	46.637	252	4	0.328	0.044	5.863	1592	WS0086_O23
1	38.326	6	4	-0.265	0.045	3.854	1593	WS0086_P18
1	95.572	17	3	0.191	0.044	4.699	1593	WS0086_P18
3	47.928	49	1	0.241	0.051	4.352	1593	WS0086_P18
6	56.188	120	2	0.251	0.038	4.199	1593	WS0086_P18
6	87.891	124	2	0.242	0.054	5.520	1593	WS0086_P18
9	0.000	163	1	-0.469	0.119	4.848	1593	WS0086_P18
9	59.963	177	4	-0.245	0.084	6.983	1593	WS0086_P18
4	124.132	83	1	0.542	0.124	5.906	1594	WS0087_C18
5	86.669	106	4	-0.454	0.128	6.194	1594	WS0087_C18
6	49.100	118	4	0.540	0.189	8.985	1594	WS0087_C18
9	0.000	163	1	-0.515	0.131	5.828	1594	WS0087_C18
9	2.011	167	4	0.220	0.043	4.039	1594	WS0087_C18
10	78.479	193	3	-0.363	0.065	6.127	1594	WS0087_C18
3	14.500	43	2	-0.298	0.046	6.109	1595	WS0087_D10
3	20.512	46	2	0.204	0.056	6.736	1595	WS0087_D10
3	116.958	63	4	0.250	0.053	4.517	1595	WS0087_D10
6	26.350	115	2	-0.146	0.037	4.492	1595	WS0087_D10
7	17.610	131	3	0.194	0.076	4.237	1595	WS0087_D10
11	0.000	199	2	0.286	0.049	5.978	1595	WS0087_D10
12	6.242	234	2	0.197	0.042	4.755	1595	WS0087_D10
2	75.550	31	1	0.299	0.042	5.428	1596	WS0085_I24
3	14.500	43	1	-0.200	0.036	4.574	1596	WS0085_I24
3	17.158	45	1	0.270	0.050	6.469	1596	WS0085_I24
3	154.505	69	1	0.287	0.039	5.045	1596	WS0085_I24
6	89.401	125	1	-0.268	0.034	4.437	1596	WS0085_I24
6	95.249	126	1	0.384	0.094	7.218	1596	WS0085_I24
6	106.702	128	3	0.206	0.048	4.586	1596	WS0085_I24
7	73.169	142	1	0.506	0.085	4.428	1596	WS0085_I24
10	35.204	189	1	-0.272	0.030	3.884	1596	WS0085_I24
11	35.604	207	3	-0.187	0.051	5.350	1596	WS0085_I24
11	37.143	209	3	0.169	0.043	4.504	1596	WS0085_I24
11	45.989	216	1	-0.183	0.030	3.863	1596	WS0085_I24
11	46.026	217	1	-0.256	0.053	6.808	1596	WS0085_I24
11	57.661	220	4	0.296	0.081	6.697	1596	WS0085_I24
11	64.014	222	4	-0.428	0.122	10.226	1596	WS0085_I24
1	22.419	4	3	-0.273	0.091	6.473	1597	WS0086_B05
1	40.464	7	3	-0.407	0.069	9.789	1597	WS0086_B05
1	43.578	8	3	-0.437	0.087	12.672	1597	WS0086_B05
2	120.617	38	3	-0.329	0.053	7.501	1597	WS0086_B05
2	127.147	40	3	0.323	0.052	7.462	1597	WS0086_B05
3	14.500	43	1	-0.211	0.057	5.545	1597	WS0086_B05
3	17.158	45	1	0.265	0.079	7.855	1597	WS0086_B05
3	137.891	64	3	-0.372	0.044	6.902	1597	WS0086_B05
6	26.350	115	2	0.214	0.069	6.116	1597	WS0086_B05
6	49.100	118	2	-0.368	0.179	12.193	1597	WS0086_B05
6	56.188	120	2	-0.348	0.057	5.168	1597	WS0086_B05
6	81.996	123	3	0.521	0.274	19.551	1597	WS0086_B05
6	106.702	128	3	-0.573	0.168	22.563	1597	WS0086_B05
8	50.001	154	1	-0.284	0.056	6.299	1597	WS0086_B05
3	47.928	49	1	0.217	0.056	6.165	1598	IS0014_F19
4	103.719	81	4	-0.232	0.066	6.173	1598	IS0014_F19

4	124.132	83	1	0.541	0.118	6.535	1598	IS0014_F19
4	142.917	85	1	-0.180	0.040	4.332	1598	IS0014_F19
10	9.580	187	3	-0.313	0.076	4.755	1598	IS0014_F19
10	70.246	190	2	0.188	0.049	3.927	1598	IS0014_F19
10	78.479	193	3	-0.261	0.047	4.852	1598	IS0014_F19
11	0.000	199	3	0.204	0.042	4.093	1598	IS0014_F19
1	123.316	20	4	-0.297	0.046	4.711	1599	IS0014_P01
1	131.686	23	2	0.446	0.052	5.390	1599	IS0014_P01
2	48.839	29	4	-0.259	0.041	5.420	1599	IS0014_P01
3	116.958	63	4	0.157	0.031	4.091	1599	IS0014_P01
3	154.505	69	4	-0.285	0.039	4.232	1599	IS0014_P01
6	95.249	126	1	0.307	0.119	4.785	1599	IS0014_P01
9	36.227	165	4	0.194	0.024	4.747	1599	IS0014_P01
9	59.963	177	4	-0.226	0.051	5.582	1599	IS0014_P01
1	38.326	6	4	-0.278	0.038	4.838	1600	WS00926_C17
1	40.464	7	4	0.303	0.040	4.259	1600	WS00926_C17
1	43.578	8	2	0.187	0.056	5.782	1600	WS00926_C17
4	142.917	85	1	-0.222	0.048	4.048	1600	WS00926_C17
5	86.669	106	4	-0.433	0.102	4.179	1600	WS00926_C17
6	25.914	114	1	-0.191	0.056	4.750	1600	WS00926_C17
7	15.555	130	3	-0.258	0.077	5.646	1600	WS00926_C17
7	67.154	140	4	-0.319	0.134	6.075	1600	WS00926_C17
7	75.883	144	4	-0.405	0.070	5.302	1600	WS00926_C17
9	17.951	169	1	-0.364	0.069	6.103	1600	WS00926_C17
9	17.951	169	2	0.294	0.031	4.587	1600	WS00926_C17
11	9.237	202	4	0.256	0.041	4.982	1600	WS00926_C17
3	17.158	45	1	0.239	0.036	4.778	1601	WS00926_D01
9	0.000	163	1	-0.511	0.060	5.748	1601	WS00926_D01
9	28.608	170	3	0.316	0.057	4.805	1601	WS00926_D01
11	46.026	217	1	-0.261	0.045	5.982	1601	WS00926_D01
11	57.661	220	4	0.244	0.060	4.562	1601	WS00926_D01
11	64.014	222	4	-0.333	0.080	6.096	1601	WS00926_D01
14	46.637	252	3	0.214	0.047	5.093	1601	WS00926_D01
1	40.464	7	4	0.307	0.035	4.602	1602	WS00926_E09
3	15.147	44	2	-0.236	0.063	3.946	1602	WS00926_E09
4	103.719	81	4	-0.159	0.035	4.840	1602	WS00926_E09
7	15.555	130	2	0.315	0.074	6.658	1602	WS00926_E09
7	62.787	139	4	-0.121	0.021	3.850	1602	WS00926_E09
7	74.560	143	1	-0.219	0.064	6.228	1602	WS00926_E09
7	75.883	144	2	0.370	0.067	4.368	1602	WS00926_E09
7	75.883	144	4	-0.359	0.052	5.863	1602	WS00926_E09
9	0.000	163	1	-0.512	0.125	5.785	1602	WS00926_E09
11	46.026	217	1	-0.259	0.059	5.783	1602	WS00926_E09
1	40.464	7	4	0.338	0.043	5.083	1603	WS00926_E20
1	95.572	17	3	0.234	0.071	5.322	1603	WS00926_E20
4	92.039	79	4	-0.326	0.034	3.924	1603	WS00926_E20
4	103.719	81	4	-0.166	0.035	5.414	1603	WS00926_E20
6	49.100	118	4	0.428	0.059	4.181	1603	WS00926_E20
7	15.555	130	2	0.267	0.059	4.004	1603	WS00926_E20
11	46.026	217	1	-0.291	0.049	5.901	1603	WS00926_E20
3	95.157	59	2	0.218	0.046	4.896	1604	WS00926_G05
7	15.555	130	2	0.322	0.061	6.417	1604	WS00926_G05
5	86.669	106	2	0.334	0.080	4.285	1605	WS00926_G21
6	45.897	117	1	0.261	0.051	4.246	1605	WS00926_G21

7	59.789	137	3	-0.188	0.040	4.103	1605	WS00926_G21
11	46.026	217	1	-0.266	0.065	5.332	1605	WS00926_G21
1	82.301	16	4	0.209	0.038	4.128	1606	WS00926_I05
3	104.681	60	2	-0.233	0.056	5.258	1606	WS00926_I05
9	0.000	163	1	-0.439	0.071	4.248	1606	WS00926_I05
9	17.951	169	2	0.244	0.050	4.469	1606	WS00926_I05
9	28.608	170	3	0.292	0.076	4.131	1606	WS00926_I05
9	58.045	176	1	0.286	0.091	5.386	1606	WS00926_I05
9	69.046	181	1	-0.324	0.076	4.556	1606	WS00926_I05
14	0.000	246	1	-0.251	0.069	6.474	1606	WS00926_I05
1	38.326	6	4	-0.282	0.048	4.269	1607	WS00926_I18
1	40.464	7	4	0.329	0.060	5.005	1607	WS00926_I18
4	103.719	81	4	-0.195	0.036	4.635	1607	WS00926_I18
7	15.555	130	2	0.330	0.096	7.427	1607	WS00926_I18
7	75.883	144	4	-0.287	0.054	4.122	1607	WS00926_I18
9	0.000	163	1	-0.485	0.078	5.183	1607	WS00926_I18
9	58.045	176	1	0.284	0.074	4.886	1607	WS00926_I18
10	78.479	193	3	-0.259	0.041	4.023	1607	WS00926_I18
14	0.000	246	1	-0.223	0.034	3.847	1607	WS00926_I18
1	2.788	2	4	0.228	0.067	7.358	1608	WS01028_G06
1	43.578	8	2	0.296	0.081	8.528	1608	WS01028_G06
1	107.333	19	3	0.177	0.047	4.454	1608	WS01028_G06
2	120.617	38	3	0.254	0.055	5.241	1608	WS01028_G06
2	127.147	40	3	-0.258	0.057	5.443	1608	WS01028_G06
3	80.211	55	4	-0.203	0.053	4.938	1608	WS01028_G06
3	137.891	64	1	0.266	0.062	5.330	1608	WS01028_G06
4	103.719	81	4	-0.229	0.047	4.099	1608	WS01028_G06
8	50.001	154	1	0.245	0.054	4.408	1608	WS01028_G06
8	87.463	158	2	0.242	0.066	4.746	1608	WS01028_G06
9	3.127	168	1	0.306	0.126	4.310	1608	WS01028_G06
9	28.608	170	2	-0.186	0.046	4.764	1608	WS01028_G06
9	32.356	171	2	-0.204	0.039	4.099	1608	WS01028_G06
1	95.572	17	3	0.203	0.040	3.979	1609	WS01028_G19
2	48.839	29	4	-0.224	0.048	4.338	1609	WS01028_G19
5	0.000	89	3	0.310	0.065	5.897	1609	WS01028_G19
7	15.555	130	2	0.363	0.104	11.896	1609	WS01028_G19
10	78.479	193	3	-0.390	0.081	7.953	1609	WS01028_G19
10	104.214	198	4	-0.335	0.070	5.848	1609	WS01028_G19
11	57.661	220	4	0.226	0.044	3.981	1609	WS01028_G19
11	64.014	222	4	-0.337	0.072	5.826	1609	WS01028_G19
1	38.326	6	4	-0.243	0.040	3.909	1610	WS01028_H20
1	40.464	7	4	0.290	0.047	4.308	1610	WS01028_H20
2	48.839	29	4	-0.274	0.061	5.824	1610	WS01028_H20
2	126.181	39	4	-0.402	0.073	4.332	1610	WS01028_H20
5	52.820	99	3	-0.249	0.059	5.206	1610	WS01028_H20
6	89.401	125	1	-0.290	0.064	4.643	1610	WS01028_H20
7	15.555	130	2	0.303	0.071	8.106	1610	WS01028_H20
7	17.610	131	3	0.254	0.091	4.657	1610	WS01028_H20
9	3.127	168	1	-0.304	0.093	3.902	1610	WS01028_H20
1	38.326	6	4	-0.309	0.051	4.737	1611	WS01028_I08
1	40.464	7	4	0.340	0.059	4.816	1611	WS01028_I08
1	95.572	17	3	0.278	0.070	6.208	1611	WS01028_I08
6	69.073	121	3	-0.234	0.045	4.242	1611	WS01028_I08
7	15.555	130	2	0.306	0.071	6.932	1611	WS01028_I08

9	0.000	163	1	-0.415	0.074	3.854	1611	WS01028_I08
9	59.963	177	4	-0.209	0.044	3.937	1611	WS01028_I08
10	78.479	193	3	-0.400	0.088	8.204	1611	WS01028_I08
1	38.326	6	4	-0.293	0.044	4.435	1612	WS01028_J22
1	40.464	7	4	0.289	0.038	4.114	1612	WS01028_J22
2	126.181	39	4	-0.434	0.091	5.133	1612	WS01028_J22
7	73.169	142	1	0.456	0.070	3.852	1612	WS01028_J22
8	62.580	155	1	0.291	0.091	4.991	1612	WS01028_J22
10	78.479	193	3	-0.345	0.083	6.243	1612	WS01028_J22
11	46.026	217	1	-0.285	0.070	6.359	1612	WS01028_J22
1	40.464	7	4	0.300	0.077	4.577	1613	WS01028_K09
1	95.572	17	3	0.290	0.050	4.591	1613	WS01028_K09
2	102.814	36	1	0.241	0.041	3.999	1613	WS01028_K09
2	120.617	38	3	0.301	0.058	6.191	1613	WS01028_K09
2	127.147	40	3	-0.218	0.040	4.254	1613	WS01028_K09
3	47.928	49	1	0.266	0.063	5.549	1613	WS01028_K09
3	144.593	66	4	-0.234	0.053	4.665	1613	WS01028_K09
7	15.555	130	2	0.298	0.071	6.985	1613	WS01028_K09
10	78.479	193	3	-0.257	0.046	4.890	1613	WS01028_K09
13	1.815	244	4	0.310	0.090	7.447	1613	WS01028_K09
3	14.500	43	1	-0.290	0.046	4.657	1614	WS01028_M08
3	17.158	45	1	0.220	0.051	5.152	1614	WS01028_M08
3	154.505	69	1	0.209	0.039	3.997	1614	WS01028_M08
5	0.000	89	3	0.306	0.067	7.348	1614	WS01028_M08
6	25.914	114	1	-0.218	0.050	5.050	1614	WS01028_M08
6	45.897	117	1	0.313	0.073	7.517	1614	WS01028_M08
6	89.401	125	2	0.239	0.049	4.041	1614	WS01028_M08
7	59.789	137	3	-0.190	0.037	4.106	1614	WS01028_M08
9	36.227	165	3	-0.210	0.036	3.933	1614	WS01028_M08
11	46.026	217	1	-0.246	0.043	4.424	1614	WS01028_M08
11	64.014	222	4	-0.304	0.061	3.884	1614	WS01028_M08
1	2.788	2	3	-0.253	0.054	6.600	1615	WS01028_M19
1	62.128	11	2	0.226	0.076	3.900	1615	WS01028_M19
5	0.000	89	3	0.292	0.049	5.021	1615	WS01028_M19
6	45.897	117	1	0.384	0.089	7.724	1615	WS01028_M19
7	59.789	137	3	-0.265	0.043	5.307	1615	WS01028_M19
9	28.608	170	3	-0.271	0.042	3.942	1615	WS01028_M19
12	32.473	238	1	-0.170	0.048	4.029	1615	WS01028_M19
14	43.392	250	1	-0.352	0.068	4.743	1615	WS01028_M19
1	38.326	6	4	-0.350	0.059	4.710	1616	WS0042_G10
1	40.464	7	4	0.356	0.066	4.582	1616	WS0042_G10
1	45.794	9	1	0.303	0.041	4.762	1616	WS0042_G10
1	45.794	9	3	-0.331	0.061	6.853	1616	WS0042_G10
1	95.572	17	3	0.216	0.045	4.948	1616	WS0042_G10
3	141.443	65	4	0.218	0.073	3.985	1616	WS0042_G10
5	57.017	102	2	0.276	0.054	3.982	1616	WS0042_G10
5	57.017	102	4	-0.310	0.077	6.683	1616	WS0042_G10
6	25.914	114	1	-0.196	0.035	3.856	1616	WS0042_G10
7	75.883	144	2	0.603	0.269	9.230	1616	WS0042_G10
7	75.883	144	4	-0.330	0.068	5.380	1616	WS0042_G10
9	3.127	168	1	-0.330	0.032	4.450	1616	WS0042_G10
9	58.045	176	1	0.272	0.092	5.471	1616	WS0042_G10
10	86.151	195	3	0.178	0.052	5.608	1616	WS0042_G10
11	46.026	217	1	-0.223	0.039	4.331	1616	WS0042_G10

14	44.898	251	3	0.300	0.062	6.886	1616	WS0042_G10
1	62.128	11	1	-0.579	0.166	6.375	1617	WS0042_G21
1	95.572	17	3	0.238	0.051	4.948	1617	WS0042_G21
2	75.550	31	1	0.224	0.044	4.755	1617	WS0042_G21
3	14.500	43	1	-0.291	0.072	7.740	1617	WS0042_G21
3	17.158	45	1	0.284	0.051	5.564	1617	WS0042_G21
7	74.560	143	1	-0.214	0.041	4.405	1617	WS0042_G21
7	75.883	144	2	0.421	0.084	4.577	1617	WS0042_G21
7	75.883	144	4	-0.217	0.044	3.925	1617	WS0042_G21
9	81.073	183	1	0.261	0.048	5.264	1617	WS0042_G21
14	46.637	252	3	0.215	0.042	4.089	1617	WS0042_G21
1	40.464	7	4	0.332	0.043	3.857	1618	WS0042_J10
4	124.132	83	1	0.452	0.105	4.255	1618	WS0042_J10
4	142.917	85	1	-0.246	0.057	5.784	1618	WS0042_J10
6	45.897	117	1	0.200	0.040	4.142	1618	WS0042_J10
6	95.249	126	1	0.268	0.062	3.929	1618	WS0042_J10
7	75.883	144	2	0.421	0.072	4.608	1618	WS0042_J10
9	36.227	165	4	0.235	0.026	5.400	1618	WS0042_J10
10	35.204	189	1	-0.313	0.053	5.572	1618	WS0042_J10
6	25.914	114	1	-0.218	0.049	4.038	1619	WS0042_L05
1	43.578	8	2	-0.275	0.051	5.962	1620	WS0042_O23
1	77.177	15	2	0.160	0.034	3.944	1620	WS0042_O23
1	123.316	20	4	-0.290	0.064	5.132	1620	WS0042_O23
3	95.157	59	2	-0.239	0.068	7.565	1620	WS0042_O23
3	104.681	60	2	0.267	0.059	6.786	1620	WS0042_O23
3	112.868	61	2	-0.232	0.061	7.039	1620	WS0042_O23
3	114.747	62	2	-0.249	0.051	5.934	1620	WS0042_O23
4	55.925	76	1	-0.211	0.059	4.369	1620	WS0042_O23
6	69.073	121	4	0.274	0.054	5.954	1620	WS0042_O23
6	76.376	122	1	-0.380	0.137	4.170	1620	WS0042_O23
7	62.787	139	2	0.218	0.047	5.203	1620	WS0042_O23
8	39.090	152	4	-0.215	0.044	4.849	1620	WS0042_O23
8	50.001	154	1	-0.218	0.043	4.317	1620	WS0042_O23
9	81.073	183	1	0.291	0.067	4.955	1620	WS0042_O23
10	35.204	189	2	0.247	0.051	4.941	1620	WS0042_O23
11	29.069	205	2	0.243	0.048	5.390	1620	WS0042_O23
11	41.984	212	2	0.202	0.035	4.095	1620	WS0042_O23
11	57.661	220	4	-0.268	0.053	5.860	1620	WS0042_O23
11	64.014	222	4	0.323	0.057	5.452	1620	WS0042_O23
1	29.800	5	1	-0.340	0.074	6.554	1621	WS0042_P22
1	43.578	8	2	0.201	0.044	4.432	1621	WS0042_P22
2	101.076	35	3	-0.241	0.047	5.005	1621	WS0042_P22
4	0.000	71	4	-0.276	0.063	5.223	1621	WS0042_P22
4	75.958	77	3	-0.286	0.086	3.871	1621	WS0042_P22
7	15.555	130	2	0.271	0.055	5.551	1621	WS0042_P22
7	75.883	144	2	0.423	0.084	4.875	1621	WS0042_P22
12	22.629	237	1	0.232	0.044	3.872	1621	WS0042_P22
14	44.898	251	3	0.248	0.049	5.061	1621	WS0042_P22
14	46.637	252	3	0.284	0.046	4.912	1621	WS0042_P22
1	45.794	9	1	0.390	0.070	7.919	1622	WS0043_C07
1	50.955	10	1	-0.305	0.054	5.967	1622	WS0043_C07
3	154.505	69	1	0.209	0.044	4.795	1622	WS0043_C07
4	82.462	78	1	-0.256	0.038	4.172	1622	WS0043_C07
4	92.039	79	4	-0.314	0.038	4.896	1622	WS0043_C07

5	57.017	102	4	-0.361	0.065	4.775	1622	WS0043_C07
6	95.249	126	1	0.352	0.187	8.014	1622	WS0043_C07
8	0.000	148	4	0.234	0.043	4.272	1622	WS0043_C07
8	62.580	155	1	0.212	0.078	5.016	1622	WS0043_C07
10	78.479	193	3	-0.332	0.045	4.361	1622	WS0043_C07
10	86.151	195	3	0.185	0.051	4.543	1622	WS0043_C07
11	45.989	216	1	-0.245	0.035	3.920	1622	WS0043_C07
1	29.800	5	1	-0.297	0.040	4.772	1623	WS0043_D13
3	14.500	43	1	-0.216	0.036	4.214	1623	WS0043_D13
3	83.563	56	3	0.214	0.036	5.052	1623	WS0043_D13
3	137.891	64	3	-0.275	0.053	6.293	1623	WS0043_D13
3	154.505	69	1	0.213	0.035	4.183	1623	WS0043_D13
5	16.219	93	1	0.208	0.036	4.337	1623	WS0043_D13
7	15.555	130	2	0.333	0.110	7.991	1623	WS0043_D13
7	17.610	131	3	0.310	0.119	5.125	1623	WS0043_D13
7	74.560	143	1	-0.293	0.054	6.413	1623	WS0043_D13
10	35.204	189	1	-0.379	0.082	9.795	1623	WS0043_D13
14	0.000	246	1	-0.353	0.104	12.268	1623	WS0043_D13
1	95.572	17	3	0.180	0.048	4.848	1624	WS0041_H04
3	154.505	69	4	-0.145	0.032	3.942	1624	WS0041_H04
4	55.925	76	1	0.165	0.046	4.216	1624	WS0041_H04
4	103.719	81	4	-0.148	0.044	4.100	1624	WS0041_H04
9	0.000	163	1	-0.536	0.111	6.393	1624	WS0041_H04
10	70.246	190	2	0.240	0.062	6.355	1624	WS0041_H04
13	0.000	243	4	0.473	0.157	4.987	1624	WS0041_H04
1	43.578	8	2	0.218	0.056	4.222	1625	WS0041_O07
3	17.158	45	1	0.212	0.042	4.737	1625	WS0041_O07
6	49.100	118	4	0.457	0.120	4.650	1625	WS0041_O07
9	0.000	163	1	-0.543	0.090	6.499	1625	WS0041_O07
9	28.608	170	3	0.281	0.045	4.623	1625	WS0041_O07
9	58.045	176	1	0.224	0.058	4.132	1625	WS0041_O07
11	35.604	207	3	-0.168	0.041	4.305	1625	WS0041_O07
1	82.301	16	4	-0.185	0.044	4.055	1626	WS01019_O16
2	102.814	36	1	0.288	0.060	5.099	1626	WS01019_O16
7	17.610	131	3	0.224	0.076	4.390	1626	WS01019_O16
7	62.787	139	2	0.256	0.043	4.872	1626	WS01019_O16
13	0.000	243	4	0.286	0.218	5.849	1626	WS01019_O16
1	95.572	17	3	0.307	0.062	5.994	1627	WS00816_E24
6	25.914	114	1	-0.227	0.052	5.137	1627	WS00816_E24
9	0.000	163	1	-0.444	0.074	4.330	1627	WS00816_E24
9	59.963	177	1	0.244	0.043	4.240	1627	WS00816_E24
13	1.815	244	2	0.246	0.066	5.829	1627	WS00816_E24
1	69.854	13	2	-0.205	0.040	4.323	1628	WS0262_J11
1	71.592	14	2	-0.195	0.036	3.924	1628	WS0262_J11
4	103.719	81	4	-0.314	0.060	6.869	1628	WS0262_J11
5	86.669	106	2	0.199	0.090	4.449	1628	WS0262_J11
8	87.463	158	2	0.268	0.062	6.414	1628	WS0262_J11
10	35.204	189	2	-0.162	0.037	3.961	1628	WS0262_J11
14	43.392	250	2	0.231	0.046	4.978	1628	WS0262_J11
4	75.958	77	3	-0.351	0.122	6.696	1629	WS0262_N02
7	75.883	144	4	-0.304	0.071	5.318	1629	WS0262_N02
9	0.000	163	1	-0.420	0.073	3.918	1629	WS0262_N02
9	59.963	177	4	-0.390	0.125	9.352	1629	WS0262_N02
10	78.479	193	3	-0.410	0.086	9.348	1629	WS0262_N02

2	120.617	38	3	-0.166	0.039	4.335	1630	WS0038_B20
2	127.147	40	3	0.272	0.059	6.607	1630	WS0038_B20
6	106.702	128	2	-0.492	0.112	6.472	1630	WS0038_B20
7	15.555	130	2	0.227	0.043	4.544	1630	WS0038_B20
7	70.278	141	3	-0.263	0.061	6.798	1630	WS0038_B20
8	87.463	158	2	-0.194	0.066	7.679	1630	WS0038_B20
10	35.204	189	1	-0.283	0.091	7.474	1630	WS0038_B20
10	70.246	190	4	-0.375	0.078	5.611	1630	WS0038_B20
10	77.676	192	1	-0.322	0.098	8.106	1630	WS0038_B20
10	78.479	193	4	-0.251	0.063	5.401	1630	WS0038_B20
10	80.088	194	1	-0.469	0.136	11.573	1630	WS0038_B20
10	93.347	196	1	0.527	0.113	6.668	1630	WS0038_B20
11	35.604	207	3	0.226	0.041	4.604	1630	WS0038_B20
14	0.000	246	2	0.281	0.061	7.120	1630	WS0038_B20
14	44.898	251	3	0.430	0.099	11.307	1630	WS0038_B20
4	92.039	79	4	-0.349	0.046	4.921	1631	WS0038_K15
5	54.072	100	4	0.237	0.041	4.002	1631	WS0038_K15
5	57.017	102	4	-0.311	0.058	5.742	1631	WS0038_K15
6	89.401	125	1	-0.319	0.033	4.523	1631	WS0038_K15
6	95.249	126	1	0.353	0.107	6.726	1631	WS0038_K15
6	106.702	128	3	0.212	0.046	3.873	1631	WS0038_K15
7	67.154	140	4	-0.214	0.062	4.310	1631	WS0038_K15
9	3.127	168	1	-0.393	0.049	6.296	1631	WS0038_K15
10	35.204	189	1	-0.274	0.031	4.191	1631	WS0038_K15
11	57.661	220	4	0.282	0.046	4.666	1631	WS0038_K15
11	64.014	222	4	-0.351	0.053	4.885	1631	WS0038_K15
1	43.578	8	2	0.192	0.050	4.356	1632	WS00930_G22
2	127.147	40	3	-0.195	0.054	4.636	1632	WS00930_G22
4	124.132	83	1	0.469	0.086	4.775	1632	WS00930_G22
5	56.008	101	2	-0.224	0.060	3.871	1632	WS00930_G22
5	57.017	102	2	0.349	0.086	7.890	1632	WS00930_G22
10	104.214	198	2	0.318	0.062	5.454	1632	WS00930_G22
11	29.069	205	1	0.244	0.044	4.462	1632	WS00930_G22
11	45.989	216	1	-0.399	0.092	9.421	1632	WS00930_G22
12	51.382	242	1	-0.405	0.088	4.938	1632	WS00930_G22
1	123.316	20	4	-0.364	0.101	6.053	1633	WS00930_H09
1	131.686	23	2	0.525	0.111	6.404	1633	WS00930_H09
2	75.550	31	2	0.224	0.049	5.033	1633	WS00930_H09
4	43.146	74	3	0.255	0.082	8.007	1633	WS00930_H09
5	7.672	92	1	-0.233	0.043	4.614	1633	WS00930_H09
6	87.891	124	2	0.177	0.047	4.674	1633	WS00930_H09
6	89.401	125	2	0.258	0.057	5.872	1633	WS00930_H09
6	106.702	128	2	-0.397	0.096	3.849	1633	WS00930_H09
7	75.883	144	2	0.425	0.078	5.035	1633	WS00930_H09
10	70.246	190	2	-0.216	0.048	4.797	1633	WS00930_H09
10	104.214	198	2	0.276	0.060	5.912	1633	WS00930_H09
11	37.143	209	3	0.177	0.040	3.947	1633	WS00930_H09
11	45.989	216	1	-0.263	0.051	4.629	1633	WS00930_H09
11	46.026	217	1	-0.321	0.069	6.235	1633	WS00930_H09
11	60.464	221	1	0.200	0.048	4.282	1633	WS00930_H09
2	127.147	40	3	-0.184	0.060	4.523	1634	WS00930_I16
3	17.158	45	1	0.170	0.036	4.087	1634	WS00930_I16
3	141.443	65	4	0.284	0.094	5.720	1634	WS00930_I16
6	25.914	114	1	-0.236	0.059	6.680	1634	WS00930_I16

6	95.249	126	1	0.304	0.093	4.870	1634	WS00930_I16
7	75.883	144	2	0.373	0.076	4.199	1634	WS00930_I16
7	75.883	144	4	-0.329	0.053	6.014	1634	WS00930_I16
9	3.127	168	1	-0.331	0.037	4.697	1634	WS00930_I16
11	35.604	207	3	-0.216	0.082	6.204	1634	WS00930_I16
11	37.143	209	3	0.199	0.071	5.374	1634	WS00930_I16
11	42.600	213	3	-0.237	0.091	6.829	1634	WS00930_I16
11	46.026	217	1	-0.272	0.045	5.119	1634	WS00930_I16
1	38.326	6	4	-0.380	0.061	5.820	1635	WS00930_I23
1	40.464	7	4	0.438	0.074	6.757	1635	WS00930_I23
3	17.158	45	1	0.194	0.039	4.296	1635	WS00930_I23
3	47.928	49	1	0.180	0.038	4.156	1635	WS00930_I23
6	95.249	126	1	0.444	0.138	8.003	1635	WS00930_I23
7	67.154	140	4	-0.190	0.077	4.125	1635	WS00930_I23
7	75.883	144	2	0.526	0.156	7.868	1635	WS00930_I23
7	75.883	144	4	-0.439	0.085	8.583	1635	WS00930_I23
9	3.127	168	1	-0.391	0.034	4.015	1635	WS00930_I23
9	59.963	177	4	-0.212	0.061	6.340	1635	WS00930_I23
10	35.204	189	1	-0.288	0.037	4.232	1635	WS00930_I23
11	45.989	216	1	-0.230	0.040	4.417	1635	WS00930_I23
12	22.629	237	1	0.259	0.048	5.356	1635	WS00930_I23
2	48.839	29	4	-0.315	0.053	6.071	1636	WS00930_K04
2	95.880	34	1	-0.319	0.039	4.237	1636	WS00930_K04
3	14.500	43	1	-0.249	0.037	4.005	1636	WS00930_K04
3	17.158	45	1	0.174	0.044	4.711	1636	WS00930_K04
4	92.039	79	4	-0.365	0.047	5.906	1636	WS00930_K04
4	103.719	81	4	-0.160	0.028	4.682	1636	WS00930_K04
5	54.072	100	4	0.234	0.038	4.520	1636	WS00930_K04
5	57.017	102	4	-0.349	0.057	6.804	1636	WS00930_K04
9	3.127	168	1	-0.325	0.022	4.124	1636	WS00930_K04
11	46.026	217	1	-0.358	0.082	8.853	1636	WS00930_K04
1	38.326	6	4	-0.328	0.049	4.745	1637	WS00930_K12
1	40.464	7	4	0.406	0.072	6.278	1637	WS00930_K12
1	43.578	8	2	0.208	0.067	5.447	1637	WS00930_K12
1	95.572	17	3	0.233	0.045	3.850	1637	WS00930_K12
3	14.500	43	1	-0.226	0.037	3.853	1637	WS00930_K12
7	15.555	130	2	0.305	0.059	4.978	1637	WS00930_K12
7	15.555	130	3	-0.227	0.050	4.627	1637	WS00930_K12
7	67.154	140	4	-0.260	0.113	4.579	1637	WS00930_K12
7	75.883	144	2	0.514	0.121	7.706	1637	WS00930_K12
7	75.883	144	4	-0.411	0.085	8.399	1637	WS00930_K12
9	58.045	176	1	0.309	0.084	5.453	1637	WS00930_K12
9	69.046	181	1	-0.288	0.070	4.542	1637	WS00930_K12
10	35.204	189	1	-0.262	0.049	5.233	1637	WS00930_K12
12	51.382	242	1	-0.426	0.130	5.324	1637	WS00930_K12
14	0.000	246	1	-0.278	0.050	5.302	1637	WS00930_K12
3	9.953	42	3	0.231	0.039	4.287	1638	WS00930_L20
4	55.925	76	1	0.268	0.040	4.620	1638	WS00930_L20
5	42.979	97	1	-0.197	0.044	4.441	1638	WS00930_L20
7	74.560	143	1	-0.324	0.060	6.139	1638	WS00930_L20
9	0.000	166	1	0.342	0.082	8.371	1638	WS00930_L20
9	49.013	173	1	-0.512	0.188	18.983	1638	WS00930_L20
9	49.013	173	2	-0.188	0.063	5.460	1638	WS00930_L20
9	55.358	175	1	0.402	0.073	7.751	1638	WS00930_L20

9	58.045	176	1	-0.370	0.117	8.263	1638	WS00930_L20
9	59.963	177	1	-0.428	0.134	13.696	1638	WS00930_L20
9	69.046	181	1	0.345	0.084	5.918	1638	WS00930_L20
9	81.073	183	1	-0.260	0.052	5.261	1638	WS00930_L20
11	17.088	203	1	0.326	0.066	6.868	1638	WS00930_L20
11	27.405	204	1	-0.467	0.148	15.226	1638	WS00930_L20
11	29.069	205	1	-0.241	0.070	7.151	1638	WS00930_L20
13	1.815	244	4	0.306	0.054	4.402	1638	WS00930_L20
1	69.854	13	2	0.203	0.053	5.119	1639	WS00930_M04
1	71.592	14	2	0.193	0.049	4.716	1639	WS00930_M04
1	77.177	15	2	0.192	0.048	4.605	1639	WS00930_M04
3	104.681	60	2	0.199	0.043	4.158	1639	WS00930_M04
4	92.039	79	4	0.250	0.052	4.317	1639	WS00930_M04
7	15.555	130	3	0.285	0.078	8.133	1639	WS00930_M04
7	17.610	131	3	0.344	0.192	5.659	1639	WS00930_M04
7	59.789	137	3	0.175	0.038	3.952	1639	WS00930_M04
9	36.227	165	3	0.269	0.046	4.823	1639	WS00930_M04
9	49.013	173	1	-0.235	0.055	5.125	1639	WS00930_M04
9	59.963	177	1	-0.277	0.052	4.720	1639	WS00930_M04
1	29.800	5	2	0.321	0.066	7.941	1640	WS00735_C22
1	38.326	6	2	-0.269	0.053	6.133	1640	WS00735_C22
1	40.464	7	4	0.355	0.073	6.721	1640	WS00735_C22
1	43.578	8	2	0.361	0.103	12.534	1640	WS00735_C22
1	69.854	13	2	-0.262	0.052	6.301	1640	WS00735_C22
1	71.592	14	2	-0.243	0.044	5.371	1640	WS00735_C22
1	127.805	21	3	0.185	0.047	4.414	1640	WS00735_C22
3	83.563	56	3	0.190	0.044	4.159	1640	WS00735_C22
3	137.891	64	3	-0.322	0.073	7.003	1640	WS00735_C22
4	0.000	71	4	0.155	0.051	4.705	1640	WS00735_C22
4	116.928	82	2	0.286	0.056	6.642	1640	WS00735_C22
5	86.669	106	2	0.281	0.069	4.169	1640	WS00735_C22
9	17.951	169	1	-0.430	0.143	9.466	1640	WS00735_C22
11	35.604	207	3	-0.227	0.048	4.505	1640	WS00735_C22
11	37.143	209	3	0.216	0.043	4.061	1640	WS00735_C22
11	42.600	213	3	-0.219	0.043	4.099	1640	WS00735_C22
1	69.854	13	2	-0.231	0.043	4.367	1641	WS00735_D18
1	131.686	23	2	0.433	0.096	6.355	1641	WS00735_D18
2	48.839	29	1	0.295	0.062	5.640	1641	WS00735_D18
3	141.443	65	3	-0.339	0.069	4.954	1641	WS00735_D18
9	28.608	170	2	0.289	0.038	4.059	1641	WS00735_D18
11	9.237	202	4	-0.226	0.120	8.044	1641	WS00735_D18
11	27.405	204	4	0.440	0.115	5.355	1641	WS00735_D18
11	35.604	207	3	-0.296	0.056	6.825	1641	WS00735_D18
11	37.143	209	3	0.274	0.045	5.580	1641	WS00735_D18
11	42.600	213	3	-0.306	0.055	6.792	1641	WS00735_D18
1	40.464	7	4	0.310	0.063	6.546	1642	WS00735_J14
1	43.578	8	2	0.288	0.036	4.678	1642	WS00735_J14
1	127.805	21	4	0.236	0.038	4.759	1642	WS00735_J14
2	48.839	29	4	-0.201	0.038	4.298	1642	WS00735_J14
6	26.350	115	2	-0.207	0.031	4.020	1642	WS00735_J14
10	70.246	190	4	-0.310	0.058	6.701	1642	WS00735_J14
10	78.479	193	4	-0.223	0.055	6.765	1642	WS00735_J14
10	104.214	198	4	-0.206	0.028	4.458	1642	WS00735_J14
1	15.589	3	2	0.235	0.044	4.870	1643	WS00735_L11

1	22.419	4	3	-0.219	0.081	3.871	1643	WS00735_L11
1	38.326	6	4	-0.216	0.053	4.171	1643	WS00735_L11
1	40.464	7	4	0.289	0.080	7.335	1643	WS00735_L11
1	95.572	17	3	0.213	0.047	4.734	1643	WS00735_L11
1	107.333	19	4	-0.259	0.063	4.443	1643	WS00735_L11
9	0.000	163	1	-0.457	0.096	4.607	1643	WS00735_L11
9	59.963	177	1	0.274	0.049	3.930	1643	WS00735_L11
1	40.464	7	4	0.288	0.050	4.308	1644	WS01039_A02
1	95.572	17	3	0.336	0.083	8.559	1644	WS01039_A02
3	116.958	63	4	0.178	0.052	4.462	1644	WS01039_A02
7	15.555	130	2	0.269	0.054	4.978	1644	WS01039_A02
9	0.000	163	1	-0.511	0.093	5.754	1644	WS01039_A02
11	35.604	207	3	-0.232	0.056	5.671	1644	WS01039_A02
11	42.600	213	3	-0.213	0.043	4.371	1644	WS01039_A02
1	43.578	8	2	0.237	0.063	6.009	1645	WS01039_B13
1	95.572	17	3	0.280	0.058	5.774	1645	WS01039_B13
2	120.617	38	3	0.245	0.043	4.260	1645	WS01039_B13
3	9.953	42	3	-0.242	0.042	4.215	1645	WS01039_B13
4	124.132	83	1	0.576	0.178	6.825	1645	WS01039_B13
4	142.917	85	1	-0.331	0.056	5.241	1645	WS01039_B13
4	142.917	85	3	0.273	0.046	4.897	1645	WS01039_B13
6	49.100	118	4	0.304	0.101	4.338	1645	WS01039_B13
6	89.401	125	1	-0.281	0.061	5.681	1645	WS01039_B13
8	11.974	150	1	-0.322	0.083	7.716	1645	WS01039_B13
11	17.088	203	1	0.207	0.044	4.049	1645	WS01039_B13
11	40.960	210	2	0.446	0.101	5.611	1645	WS01039_B13
12	38.543	232	4	0.369	0.135	5.126	1645	WS01039_B13
14	22.402	248	3	-0.235	0.044	4.327	1645	WS01039_B13
1	62.128	11	1	-0.447	0.087	3.842	1646	WS01039_E19
1	133.992	24	2	0.215	0.042	4.471	1646	WS01039_E19
1	141.021	25	2	-0.237	0.052	5.551	1646	WS01039_E19
2	48.839	29	4	-0.216	0.055	5.235	1646	WS01039_E19
4	116.928	82	2	-0.285	0.041	4.390	1646	WS01039_E19
5	0.000	89	3	0.237	0.064	4.769	1646	WS01039_E19
9	3.127	168	1	-0.334	0.131	5.877	1646	WS01039_E19
9	59.963	177	1	-0.299	0.075	5.975	1646	WS01039_E19
11	17.088	203	1	0.221	0.051	4.079	1646	WS01039_E19
1	38.326	6	4	-0.302	0.076	6.925	1647	WS01039_G12
1	40.464	7	4	0.365	0.097	7.608	1647	WS01039_G12
1	95.572	17	3	0.324	0.091	8.661	1647	WS01039_G12
6	69.073	121	3	-0.211	0.048	4.842	1647	WS01039_G12
6	106.702	128	2	0.412	0.109	4.403	1647	WS01039_G12
9	0.000	163	1	-0.510	0.096	5.891	1647	WS01039_G12
9	59.963	177	4	-0.245	0.058	4.644	1647	WS01039_G12
1	77.177	15	2	-0.200	0.046	5.191	1648	WS00930_N09
4	0.000	71	4	0.222	0.048	4.107	1648	WS00930_N09
4	82.462	78	1	0.243	0.056	4.242	1648	WS00930_N09
8	0.000	148	4	-0.254	0.072	6.113	1648	WS00930_N09
9	28.608	170	3	0.275	0.063	6.428	1648	WS00930_N09
9	55.358	175	3	0.427	0.090	4.071	1648	WS00930_N09
9	59.963	177	1	-0.239	0.052	3.919	1648	WS00930_N09
1	38.326	6	4	-0.263	0.048	4.790	1649	WS00930_N18
1	40.464	7	4	0.316	0.060	5.165	1649	WS00930_N18
1	95.572	17	3	0.334	0.096	6.332	1649	WS00930_N18

6	69.073	121	3	-0.247	0.058	5.140	1649	WS00930_N18
9	59.963	177	4	-0.296	0.068	6.305	1649	WS00930_N18
11	46.026	217	1	-0.245	0.060	5.553	1649	WS00930_N18
3	14.500	43	1	-0.344	0.057	7.160	1650	WS00930_O20
3	17.158	45	1	0.250	0.061	7.515	1650	WS00930_O20
4	0.000	71	4	0.191	0.052	4.417	1650	WS00930_O20
7	59.789	137	3	-0.241	0.060	6.197	1650	WS00930_O20
9	36.227	165	3	-0.190	0.040	4.115	1650	WS00930_O20
1	45.794	9	1	0.289	0.064	5.473	1651	WS00930_P09
6	106.702	128	2	-0.432	0.086	4.761	1651	WS00930_P09
7	74.560	143	1	-0.230	0.060	5.022	1651	WS00930_P09
7	75.883	144	2	0.395	0.079	4.323	1651	WS00930_P09
7	75.883	144	4	-0.249	0.054	4.398	1651	WS00930_P09
9	58.045	176	1	0.280	0.075	4.006	1651	WS00930_P09
11	56.921	219	2	-0.310	0.110	5.389	1651	WS00930_P09
3	141.443	65	4	0.293	0.093	4.252	1652	WS00931_A14
6	25.914	114	1	-0.182	0.041	4.157	1652	WS00931_A14
8	62.580	155	1	0.326	0.123	5.923	1652	WS00931_A14
12	0.000	233	1	-0.165	0.040	3.973	1652	WS00931_A14
1	40.464	7	4	0.300	0.046	4.116	1653	WS00931_A22
3	14.500	43	1	-0.183	0.036	3.886	1653	WS00931_A22
3	17.158	45	1	0.249	0.052	5.787	1653	WS00931_A22
4	103.719	81	4	-0.177	0.040	4.218	1653	WS00931_A22
6	95.249	126	1	0.402	0.105	7.615	1653	WS00931_A22
7	67.154	140	4	-0.253	0.106	4.075	1653	WS00931_A22
7	75.883	144	2	0.415	0.098	4.932	1653	WS00931_A22
7	75.883	144	4	-0.315	0.054	4.644	1653	WS00931_A22
9	3.127	168	1	-0.374	0.076	4.722	1653	WS00931_A22
1	38.326	6	4	-0.313	0.069	5.639	1654	WS00931_C10
1	40.464	7	4	0.303	0.056	4.521	1654	WS00931_C10
1	62.128	11	1	-0.479	0.100	4.356	1654	WS00931_C10
6	25.914	114	1	-0.244	0.061	6.390	1654	WS00931_C10
6	45.897	117	1	0.298	0.058	6.232	1654	WS00931_C10
1	82.301	16	4	0.273	0.032	4.427	1655	WS00931_C22
1	131.686	23	1	-0.470	0.049	5.049	1655	WS00931_C22
6	81.996	123	4	0.427	0.109	11.415	1655	WS00931_C22
7	73.169	142	1	0.674	0.081	8.406	1655	WS00931_C22
8	39.090	152	4	-0.203	0.035	4.830	1655	WS00931_C22
8	50.001	154	1	-0.225	0.020	4.504	1655	WS00931_C22
9	3.127	168	1	-0.270	0.030	3.851	1655	WS00931_C22
10	21.864	188	1	0.334	0.057	7.371	1655	WS00931_C22
10	70.246	190	4	0.185	0.028	3.906	1655	WS00931_C22
11	45.989	216	1	-0.283	0.050	6.485	1655	WS00931_C22
11	56.921	219	1	0.288	0.040	4.117	1655	WS00931_C22
6	95.249	126	1	0.404	0.163	6.724	1656	WS00931_E06
9	59.963	177	4	-0.210	0.048	5.706	1656	WS00931_E06
12	22.629	237	1	0.240	0.038	3.912	1656	WS00931_E06
14	46.637	252	4	0.438	0.074	7.704	1656	WS00931_E06
2	75.550	31	2	0.173	0.042	4.371	1657	WS00931_H10
2	95.880	34	1	-0.498	0.079	9.124	1657	WS00931_H10
2	102.814	36	1	-0.296	0.038	4.372	1657	WS00931_H10
3	148.745	68	4	-0.203	0.054	6.916	1657	WS00931_H10
8	0.000	148	4	0.255	0.037	3.893	1657	WS00931_H10
10	70.246	190	2	0.199	0.046	3.858	1657	WS00931_H10

11	40.960	210	2	-0.315	0.079	3.974	1657	WS00931_H10
12	22.629	237	1	0.280	0.053	5.994	1657	WS00931_H10
12	32.473	238	1	0.246	0.040	4.518	1657	WS00931_H10
1	43.578	8	2	0.284	0.085	6.656	1658	WS01025_I12
3	137.891	64	1	0.240	0.036	3.974	1658	WS01025_I12
3	141.443	65	3	0.345	0.093	7.304	1658	WS01025_I12
4	55.925	76	1	0.218	0.045	4.964	1658	WS01025_I12
4	82.462	78	1	0.195	0.042	4.723	1658	WS01025_I12
6	56.188	120	2	0.232	0.055	4.034	1658	WS01025_I12
10	86.151	195	3	-0.264	0.065	7.147	1658	WS01025_I12
10	93.347	196	1	-0.406	0.052	4.147	1658	WS01025_I12
12	22.441	227	1	0.266	0.043	4.926	1658	WS01025_I12
14	46.637	252	3	-0.319	0.045	5.050	1658	WS01025_I12
1	45.794	9	1	0.350	0.098	5.673	1659	WS01025_O06
1	50.955	10	1	-0.346	0.157	9.009	1659	WS01025_O06
1	82.301	16	3	-0.188	0.040	4.143	1659	WS01025_O06
2	63.261	30	3	0.238	0.045	4.631	1659	WS01025_O06
3	15.147	44	2	0.284	0.088	5.469	1659	WS01025_O06
3	20.512	46	2	0.308	0.062	8.092	1659	WS01025_O06
9	0.000	163	2	0.556	0.097	5.397	1659	WS01025_O06
9	36.227	165	3	-0.200	0.043	4.465	1659	WS01025_O06
10	70.246	190	2	-0.309	0.070	9.076	1659	WS01025_O06
11	64.014	222	3	-0.343	0.100	10.376	1659	WS01025_O06
4	173.929	88	2	0.235	0.052	5.043	1660	WS0099_F07
7	17.610	131	3	0.275	0.092	4.259	1660	WS0099_F07
10	70.246	190	2	0.202	0.043	4.158	1660	WS0099_F07
10	78.479	193	3	-0.338	0.064	6.901	1660	WS0099_F07
12	22.629	237	1	0.181	0.044	4.005	1660	WS0099_F07
1	43.578	8	2	0.218	0.038	3.901	1661	WS0099_G03
1	95.572	17	3	0.273	0.056	5.531	1661	WS0099_G03
7	15.555	130	2	0.307	0.064	6.816	1661	WS0099_G03
9	0.000	163	1	-0.547	0.128	6.768	1661	WS0099_G03
10	78.479	193	3	-0.304	0.046	4.629	1661	WS0099_G03
11	64.014	222	3	-0.215	0.043	4.265	1661	WS0099_G03
14	43.392	250	2	0.249	0.047	4.612	1661	WS0099_G03
1	95.572	17	3	0.250	0.047	4.151	1662	WS0099_H24
2	48.839	29	4	-0.229	0.052	4.415	1662	WS0099_H24
5	0.000	89	3	0.275	0.061	5.744	1662	WS0099_H24
7	15.555	130	2	0.313	0.086	8.928	1662	WS0099_H24
8	62.580	155	1	0.293	0.160	4.434	1662	WS0099_H24
1	40.464	7	4	0.235	0.064	5.435	1663	WS0099_I15
1	43.578	8	2	0.242	0.036	4.146	1663	WS0099_I15
1	95.572	17	3	0.229	0.043	4.405	1663	WS0099_I15
2	48.839	29	4	-0.264	0.059	4.434	1663	WS0099_I15
3	116.958	63	4	0.236	0.055	4.593	1663	WS0099_I15
9	0.000	163	1	-0.461	0.085	4.705	1663	WS0099_I15
10	78.479	193	3	-0.224	0.041	4.235	1663	WS0099_I15
1	38.326	6	4	-0.249	0.037	4.003	1664	WS0099_K16
1	40.464	7	4	0.291	0.047	4.253	1664	WS0099_K16
2	48.839	29	4	-0.318	0.059	6.917	1664	WS0099_K16
4	173.929	88	2	0.153	0.046	3.861	1664	WS0099_K16
7	73.169	142	1	0.462	0.073	4.668	1664	WS0099_K16
7	75.883	144	4	-0.312	0.056	6.520	1664	WS0099_K16
8	62.580	155	1	0.269	0.069	4.436	1664	WS0099_K16

9	3.127	168	1	-0.294	0.049	5.537	1664	WS0099_K16
9	59.963	177	4	-0.217	0.041	4.591	1664	WS0099_K16
11	46.026	217	1	-0.346	0.080	8.392	1664	WS0099_K16
1	38.326	6	4	-0.338	0.071	6.482	1665	WS0099_L04
1	40.464	7	4	0.386	0.085	6.732	1665	WS0099_L04
1	95.572	17	3	0.266	0.059	5.515	1665	WS0099_L04
7	75.883	144	4	-0.296	0.056	4.273	1665	WS0099_L04
8	62.580	155	1	0.286	0.100	4.277	1665	WS0099_L04
9	0.000	163	1	-0.432	0.074	4.140	1665	WS0099_L04
10	78.479	193	3	-0.382	0.065	6.172	1665	WS0099_L04
11	46.026	217	1	-0.247	0.049	4.814	1665	WS0099_L04
1	38.326	6	4	-0.285	0.056	5.635	1666	WS0099_N21
1	40.464	7	4	0.351	0.074	6.668	1666	WS0099_N21
1	95.572	17	3	0.294	0.084	6.924	1666	WS0099_N21
6	106.702	128	2	0.415	0.118	4.355	1666	WS0099_N21
8	62.580	155	1	0.354	0.107	5.644	1666	WS0099_N21
9	3.127	168	1	-0.352	0.095	7.453	1666	WS0099_N21
9	59.963	177	4	-0.269	0.051	5.190	1666	WS0099_N21
11	46.026	217	1	-0.256	0.062	6.340	1666	WS0099_N21
3	69.071	52	1	0.243	0.081	4.353	1667	WS0099_O19
4	75.958	77	3	-0.334	0.112	5.055	1667	WS0099_O19
5	0.000	89	3	0.257	0.046	4.361	1667	WS0099_O19
5	16.219	93	2	0.264	0.054	5.080	1667	WS0099_O19
8	62.580	155	1	0.324	0.119	4.506	1667	WS0099_O19
10	78.479	193	3	-0.330	0.049	4.822	1667	WS0099_O19
10	78.479	193	4	0.173	0.044	3.874	1667	WS0099_O19
11	35.604	207	3	-0.236	0.059	5.813	1667	WS0099_O19
11	37.143	209	3	0.228	0.055	5.440	1667	WS0099_O19
11	42.600	213	3	-0.216	0.044	4.409	1667	WS0099_O19
13	0.000	243	4	-0.310	0.104	3.917	1667	WS0099_O19
13	1.815	244	2	0.328	0.085	8.587	1667	WS0099_O19
13	1.815	244	4	-0.278	0.058	5.118	1667	WS0099_O19
3	14.500	43	1	-0.289	0.042	5.314	1668	WS01026_C11
3	17.158	45	1	0.257	0.067	8.448	1668	WS01026_C11
6	95.249	126	1	0.224	0.051	4.564	1668	WS01026_C11
6	106.702	128	3	0.234	0.039	4.106	1668	WS01026_C11
7	59.789	137	3	-0.205	0.049	5.694	1668	WS01026_C11
9	36.227	165	3	-0.209	0.044	5.068	1668	WS01026_C11
11	35.604	207	3	-0.178	0.037	4.214	1668	WS01026_C11
11	37.143	209	3	0.178	0.037	4.238	1668	WS01026_C11
11	42.600	213	3	-0.184	0.037	4.280	1668	WS01026_C11
11	64.014	222	3	-0.224	0.034	4.027	1668	WS01026_C11
14	43.392	250	1	-0.274	0.058	5.091	1668	WS01026_C11
1	2.788	2	3	-0.210	0.046	4.510	1669	WS01026_D02
3	14.500	43	1	-0.341	0.057	6.593	1669	WS01026_D02
3	17.158	45	1	0.215	0.053	6.159	1669	WS01026_D02
4	82.462	78	1	-0.208	0.042	4.857	1669	WS01026_D02
6	106.702	128	3	0.306	0.061	5.516	1669	WS01026_D02
7	74.560	143	1	-0.277	0.033	3.903	1669	WS01026_D02
1	40.464	7	4	0.168	0.046	4.073	1670	WS01026_F02
1	43.578	8	2	0.202	0.079	7.436	1670	WS01026_F02
1	69.854	13	2	-0.235	0.057	5.354	1670	WS01026_F02
1	77.177	15	2	-0.209	0.044	4.178	1670	WS01026_F02
3	14.500	43	1	-0.378	0.058	5.735	1670	WS01026_F02

3	17.158	45	1	0.216	0.055	5.140	1670	WS01026_F02
11	35.604	207	3	-0.329	0.055	6.170	1670	WS01026_F02
11	37.143	209	3	0.306	0.042	4.845	1670	WS01026_F02
1	40.464	7	4	0.204	0.051	4.472	1671	WS01026_F16
3	141.443	65	3	-0.375	0.074	3.999	1671	WS01026_F16
6	87.891	124	2	0.168	0.040	4.266	1671	WS01026_F16
8	50.001	154	2	-0.164	0.042	4.053	1671	WS01026_F16
9	28.608	170	2	0.300	0.035	4.184	1671	WS01026_F16
9	28.608	170	3	-0.265	0.040	4.423	1671	WS01026_F16
10	35.204	189	2	-0.265	0.100	11.250	1671	WS01026_F16
10	70.246	190	2	0.155	0.042	4.429	1671	WS01026_F16
11	35.604	207	3	-0.338	0.048	5.162	1671	WS01026_F16
11	37.143	209	3	0.319	0.035	3.935	1671	WS01026_F16
1	2.788	2	3	-0.270	0.047	4.893	1672	WS01026_H11
2	93.504	32	4	-0.267	0.063	5.907	1672	WS01026_H11
3	83.563	56	3	0.265	0.079	7.609	1672	WS01026_H11
4	103.719	81	4	-0.239	0.046	5.151	1672	WS01026_H11
4	124.132	83	1	0.530	0.135	5.802	1672	WS01026_H11
5	0.000	89	3	0.243	0.058	5.768	1672	WS01026_H11
6	45.897	117	1	0.241	0.040	3.977	1672	WS01026_H11
6	49.100	118	4	0.395	0.109	4.906	1672	WS01026_H11
1	40.464	7	4	0.206	0.038	3.846	1673	WS01026_I01
3	9.953	42	4	0.291	0.056	4.441	1673	WS01026_I01
3	141.443	65	3	-0.416	0.121	6.620	1673	WS01026_I01
3	144.647	67	1	0.253	0.068	3.905	1673	WS01026_I01
4	55.925	76	1	-0.235	0.060	6.239	1673	WS01026_I01
4	142.917	85	1	-0.266	0.038	4.279	1673	WS01026_I01
5	57.017	102	4	0.280	0.044	4.325	1673	WS01026_I01
6	49.100	118	4	0.326	0.098	4.930	1673	WS01026_I01
7	15.555	130	2	0.295	0.053	4.540	1673	WS01026_I01
8	62.580	155	1	0.390	0.088	5.105	1673	WS01026_I01
11	17.088	203	1	0.273	0.044	4.927	1673	WS01026_I01
1	38.326	6	4	-0.334	0.045	4.015	1674	WS01026_K02
1	40.464	7	4	0.341	0.043	4.556	1674	WS01026_K02
1	45.794	9	1	0.342	0.042	5.120	1674	WS01026_K02
1	82.301	16	4	0.330	0.052	5.403	1674	WS01026_K02
1	95.572	17	3	0.276	0.075	6.103	1674	WS01026_K02
2	75.550	31	1	0.264	0.034	4.194	1674	WS01026_K02
3	14.500	43	1	-0.246	0.039	4.721	1674	WS01026_K02
3	17.158	45	1	0.212	0.053	6.364	1674	WS01026_K02
4	103.719	81	4	-0.207	0.038	5.509	1674	WS01026_K02
6	25.914	114	1	-0.164	0.035	4.230	1674	WS01026_K02
6	89.401	125	1	-0.272	0.036	4.507	1674	WS01026_K02
6	95.249	126	1	0.309	0.088	5.624	1674	WS01026_K02
7	15.555	130	2	0.354	0.099	6.854	1674	WS01026_K02
7	67.154	140	4	-0.208	0.072	4.030	1674	WS01026_K02
7	74.560	143	1	-0.287	0.050	6.025	1674	WS01026_K02
7	75.883	144	2	0.524	0.137	7.634	1674	WS01026_K02
7	75.883	144	4	-0.402	0.079	8.518	1674	WS01026_K02
9	3.127	168	1	-0.505	0.101	9.397	1674	WS01026_K02
11	46.026	217	1	-0.260	0.034	4.161	1674	WS01026_K02
14	0.000	246	1	-0.243	0.032	3.907	1674	WS01026_K02
1	38.326	6	4	-0.330	0.058	4.228	1675	WS01026_K20
1	40.464	7	4	0.339	0.065	5.029	1675	WS01026_K20

1	43.578	8	2	0.206	0.076	4.612	1675	WS01026_K20
1	95.572	17	3	0.237	0.037	4.088	1675	WS01026_K20
2	75.550	31	1	0.304	0.039	5.000	1675	WS01026_K20
3	14.500	43	1	-0.238	0.032	4.057	1675	WS01026_K20
4	103.719	81	4	-0.214	0.047	5.328	1675	WS01026_K20
6	95.249	126	1	0.284	0.070	6.030	1675	WS01026_K20
6	106.702	128	3	0.309	0.077	7.555	1675	WS01026_K20
7	75.883	144	2	0.440	0.121	5.164	1675	WS01026_K20
7	75.883	144	4	-0.332	0.069	5.502	1675	WS01026_K20
9	36.227	165	3	-0.213	0.035	3.862	1675	WS01026_K20
9	3.127	168	1	-0.362	0.063	5.266	1675	WS01026_K20
10	35.204	189	1	-0.257	0.046	5.796	1675	WS01026_K20
11	35.604	207	3	-0.191	0.038	4.132	1675	WS01026_K20
11	42.600	213	3	-0.202	0.039	4.300	1675	WS01026_K20
1	95.572	17	2	0.165	0.038	4.179	1676	WS01026_M12
1	141.021	25	2	-0.226	0.048	5.456	1676	WS01026_M12
4	103.719	81	4	-0.241	0.053	4.899	1676	WS01026_M12
6	106.702	128	3	0.228	0.040	4.083	1676	WS01026_M12
1	43.578	8	2	0.215	0.055	4.285	1677	WS00912_B21
1	95.572	17	3	0.233	0.072	5.965	1677	WS00912_B21
2	95.880	34	1	-0.382	0.046	5.218	1677	WS00912_B21
6	25.914	114	1	-0.201	0.047	5.116	1677	WS00912_B21
7	75.883	144	2	0.476	0.121	7.108	1677	WS00912_B21
9	28.608	170	3	0.314	0.066	5.238	1677	WS00912_B21
10	35.204	189	1	-0.295	0.039	4.365	1677	WS00912_B21
10	80.088	194	1	-0.204	0.045	4.923	1677	WS00912_B21
1	40.464	7	4	0.330	0.045	5.016	1678	WS00819_B23
1	95.572	17	3	0.241	0.052	3.923	1678	WS00819_B23
2	48.839	29	4	-0.288	0.052	5.498	1678	WS00819_B23
5	86.669	106	4	-0.486	0.135	8.671	1678	WS00819_B23
7	62.787	139	4	-0.186	0.028	4.406	1678	WS00819_B23
7	67.154	140	4	-0.320	0.098	6.530	1678	WS00819_B23
7	75.883	144	4	-0.378	0.085	6.026	1678	WS00819_B23
9	3.127	168	1	-0.284	0.027	4.041	1678	WS00819_B23
11	46.026	217	1	-0.245	0.034	4.043	1678	WS00819_B23
14	0.000	246	1	-0.329	0.064	7.528	1678	WS00819_B23
1	40.464	7	4	0.329	0.047	4.920	1679	WS00912_H24
1	95.572	17	3	0.250	0.074	6.355	1679	WS00912_H24
4	55.925	76	1	0.229	0.045	6.153	1679	WS00912_H24
5	86.669	106	4	-0.452	0.114	5.003	1679	WS00912_H24
7	67.154	140	4	-0.278	0.116	5.384	1679	WS00912_H24
7	73.169	142	1	0.530	0.104	4.829	1679	WS00912_H24
7	74.560	143	1	-0.227	0.054	5.478	1679	WS00912_H24
7	75.883	144	2	0.448	0.099	6.143	1679	WS00912_H24
7	75.883	144	4	-0.397	0.077	8.136	1679	WS00912_H24
9	0.000	163	1	-0.478	0.092	5.124	1679	WS00912_H24
10	93.347	196	1	0.431	0.080	4.482	1679	WS00912_H24
11	57.661	220	4	0.227	0.041	4.199	1679	WS00912_H24
11	64.014	222	4	-0.262	0.035	3.970	1679	WS00912_H24
1	62.128	11	1	-0.556	0.169	5.907	1680	WS00829_E13
2	93.504	32	4	-0.250	0.041	3.867	1680	WS00829_E13
3	104.681	60	2	-0.211	0.040	4.027	1680	WS00829_E13
7	15.555	130	2	0.282	0.057	5.681	1680	WS00829_E13
9	0.000	163	1	-0.460	0.086	4.649	1680	WS00829_E13

9	55.358	175	3	-0.419	0.081	4.271	1680	WS00829_E13
9	58.045	176	1	0.293	0.099	5.303	1680	WS00829_E13
9	59.963	177	1	0.315	0.060	5.309	1680	WS00829_E13
10	70.246	190	2	0.236	0.056	5.632	1680	WS00829_E13
12	28.493	228	2	-0.267	0.067	3.919	1680	WS00829_E13
13	0.000	243	4	0.429	0.186	6.456	1680	WS00829_E13
13	3.386	245	1	-0.193	0.046	4.093	1680	WS00829_E13
14	0.000	246	1	-0.217	0.056	4.934	1680	WS00829_E13
1	131.686	23	2	-0.487	0.081	5.033	1681	WS00829_P23
2	93.504	32	2	0.236	0.036	4.168	1681	WS00829_P23
2	126.181	39	4	0.486	0.095	6.447	1681	WS00829_P23
6	87.891	124	2	-0.170	0.034	3.888	1681	WS00829_P23
6	106.702	128	2	0.615	0.192	9.551	1681	WS00829_P23
7	15.555	130	3	0.294	0.068	6.529	1681	WS00829_P23
10	21.864	188	3	-0.234	0.043	4.078	1681	WS00829_P23
11	0.000	199	3	-0.230	0.044	4.053	1681	WS00829_P23
11	43.491	214	4	-0.231	0.056	5.101	1681	WS00829_P23
14	43.392	250	1	0.272	0.074	3.861	1681	WS00829_P23
2	127.147	40	3	-0.171	0.041	4.039	1682	WS00931_L05
3	14.500	43	2	0.228	0.038	4.351	1682	WS00931_L05
3	20.512	46	2	-0.206	0.047	5.311	1682	WS00931_L05
3	104.681	60	2	-0.218	0.045	5.033	1682	WS00931_L05
4	55.925	76	1	0.232	0.058	4.773	1682	WS00931_L05
7	15.555	130	2	0.255	0.045	5.010	1682	WS00931_L05
7	75.883	144	2	0.443	0.074	5.257	1682	WS00931_L05
7	75.883	144	4	-0.306	0.052	4.744	1682	WS00931_L05
11	27.405	204	4	0.283	0.068	5.895	1682	WS00931_L05
11	42.600	213	3	-0.177	0.042	4.163	1682	WS00931_L05
1	95.572	17	3	0.286	0.069	5.728	1683	WS00931_P14
6	56.188	120	2	0.277	0.040	3.949	1683	WS00931_P14
7	74.560	143	1	-0.259	0.074	5.479	1683	WS00931_P14
9	2.011	167	4	0.224	0.060	5.243	1683	WS00931_P14
9	69.046	181	2	0.326	0.080	3.847	1683	WS00931_P14
1	95.572	17	3	0.253	0.063	6.268	1684	WS00913_H19
5	3.484	90	2	0.298	0.070	3.987	1684	WS00913_H19
6	69.073	121	3	-0.244	0.046	4.533	1684	WS00913_H19
6	106.702	128	2	0.433	0.116	4.782	1684	WS00913_H19
8	78.389	157	1	0.184	0.048	4.305	1684	WS00913_H19
9	0.000	163	1	-0.443	0.076	4.412	1684	WS00913_H19
9	59.963	177	4	-0.317	0.063	4.698	1684	WS00913_H19
10	78.479	193	3	-0.286	0.044	4.329	1684	WS00913_H19
1	40.464	7	4	0.271	0.035	3.857	1685	WS00913_N01
4	55.925	76	1	0.243	0.060	6.243	1685	WS00913_N01
6	25.914	114	1	-0.306	0.088	7.469	1685	WS00913_N01
9	0.000	166	1	-0.213	0.074	6.250	1685	WS00913_N01
9	28.608	170	3	0.265	0.050	4.180	1685	WS00913_N01
3	15.147	44	2	0.215	0.065	4.063	1686	WS00910_A11
6	25.914	114	1	-0.308	0.093	6.951	1686	WS00910_A11
8	39.090	152	4	-0.290	0.075	6.379	1686	WS00910_A11
8	95.937	159	3	0.224	0.039	4.019	1686	WS00910_A11
10	9.580	187	2	-0.243	0.072	4.496	1686	WS00910_A11
11	35.604	207	3	-0.180	0.046	4.699	1686	WS00910_A11
12	0.000	233	1	-0.235	0.058	4.329	1686	WS00910_A11
13	3.386	245	1	-0.228	0.068	5.076	1686	WS00910_A11

1	38.326	6	4	-0.348	0.071	5.885	1687	WS00910_A24
1	40.464	7	4	0.348	0.068	4.871	1687	WS00910_A24
2	75.550	31	1	0.291	0.045	4.672	1687	WS00910_A24
5	86.669	106	4	-0.359	0.088	4.204	1687	WS00910_A24
7	74.560	143	1	-0.270	0.048	4.971	1687	WS00910_A24
7	75.883	144	4	-0.329	0.078	5.005	1687	WS00910_A24
9	3.127	168	1	-0.307	0.058	4.629	1687	WS00910_A24
9	49.013	173	1	0.192	0.045	4.960	1687	WS00910_A24
9	55.358	175	3	-0.394	0.071	3.912	1687	WS00910_A24
10	93.347	196	1	0.404	0.070	3.921	1687	WS00910_A24
14	0.000	246	1	-0.243	0.040	4.162	1687	WS00910_A24
1	38.326	6	4	-0.294	0.045	4.839	1688	WS00910_D06
1	40.464	7	4	0.324	0.050	5.387	1688	WS00910_D06
2	75.550	31	1	0.260	0.036	4.319	1688	WS00910_D06
9	3.127	168	1	-0.333	0.061	6.807	1688	WS00910_D06
10	93.347	196	1	0.465	0.089	5.238	1688	WS00910_D06
11	46.026	217	1	-0.279	0.052	6.327	1688	WS00910_D06
1	95.572	17	3	0.331	0.069	7.533	1689	WS00910_E08
3	14.500	43	1	-0.313	0.054	6.069	1689	WS00910_E08
3	17.158	45	1	0.204	0.048	5.399	1689	WS00910_E08
11	0.000	199	3	0.230	0.036	4.076	1689	WS00910_E08
11	46.026	217	1	-0.260	0.041	4.676	1689	WS00910_E08
3	15.147	44	2	0.342	0.101	6.941	1690	WS00910_H05
3	20.512	46	2	0.272	0.062	6.550	1690	WS00910_H05
4	82.462	78	2	-0.276	0.075	8.697	1690	WS00910_H05
5	7.672	92	4	0.297	0.074	6.984	1690	WS00910_H05
6	45.897	117	4	0.262	0.050	4.462	1690	WS00910_H05
11	17.088	203	1	0.327	0.095	7.938	1690	WS00910_H05
1	38.326	6	4	-0.305	0.046	6.075	1691	WS00910_H21
1	40.464	7	4	0.358	0.058	7.583	1691	WS00910_H21
1	95.572	17	3	0.281	0.092	5.183	1691	WS00910_H21
1	131.686	23	2	0.382	0.043	3.905	1691	WS00910_H21
1	38.326	6	4	-0.311	0.060	4.640	1692	WS00910_J14
1	40.464	7	4	0.335	0.068	5.046	1692	WS00910_J14
3	14.500	43	1	-0.238	0.030	4.105	1692	WS00910_J14
3	17.158	45	1	0.178	0.034	4.656	1692	WS00910_J14
10	93.347	196	1	0.458	0.056	5.289	1692	WS00910_J14
14	0.000	246	1	-0.265	0.042	5.769	1692	WS00910_J14
1	38.326	6	4	-0.261	0.071	6.775	1693	WS00910_K21
1	40.464	7	4	0.313	0.088	7.787	1693	WS00910_K21
1	43.578	8	2	0.267	0.042	3.917	1693	WS00910_K21
4	0.000	71	4	-0.200	0.049	4.659	1693	WS00910_K21
13	3.386	245	1	-0.290	0.058	5.427	1693	WS00910_K21
14	43.392	250	2	0.205	0.045	4.135	1693	WS00910_K21
3	95.157	59	2	0.184	0.042	4.455	1694	WS00830_K02
11	35.604	207	3	-0.214	0.064	5.166	1694	WS00830_K02
11	46.026	217	1	-0.300	0.094	8.126	1694	WS00830_K02
11	64.014	222	4	-0.266	0.040	4.364	1694	WS00830_K02
1	38.326	6	4	-0.222	0.041	4.240	1695	WS00830_L14
1	40.464	7	4	0.277	0.055	5.651	1695	WS00830_L14
2	48.839	29	4	-0.268	0.046	4.241	1695	WS00830_L14
4	92.039	79	4	-0.269	0.049	4.832	1695	WS00830_L14
7	15.555	130	2	0.269	0.056	4.890	1695	WS00830_L14
3	83.563	56	3	0.209	0.054	4.031	1696	WS00830_P05

4	103.719	81	4	-0.236	0.051	6.427	1696	WS00830_P05
6	25.742	113	3	0.281	0.099	7.404	1696	WS00830_P05
7	15.555	130	2	0.439	0.099	12.009	1696	WS00830_P05
8	0.000	148	4	0.186	0.036	4.012	1696	WS00830_P05
9	55.358	175	3	-0.413	0.128	5.067	1696	WS00830_P05
1	40.464	7	4	0.294	0.045	4.704	1697	WS00831_A12
2	48.839	29	4	-0.304	0.066	6.882	1697	WS00831_A12
3	104.681	60	2	-0.177	0.035	4.077	1697	WS00831_A12
3	116.958	63	4	0.200	0.048	5.068	1697	WS00831_A12
4	55.925	76	1	0.208	0.040	3.926	1697	WS00831_A12
6	25.742	113	3	0.275	0.087	7.093	1697	WS00831_A12
7	74.560	143	1	-0.258	0.056	4.065	1697	WS00831_A12
9	59.963	177	4	-0.233	0.055	4.331	1697	WS00831_A12
14	43.392	250	2	0.224	0.041	4.604	1697	WS00831_A12
3	116.958	63	4	0.233	0.064	5.152	1698	WS00831_E01
6	25.742	113	3	0.239	0.042	4.592	1698	WS00831_E01
7	15.555	130	2	0.311	0.061	7.103	1698	WS00831_E01
9	0.000	163	1	-0.438	0.079	4.252	1698	WS00831_E01
9	0.000	163	2	0.478	0.085	4.592	1698	WS00831_E01
4	103.719	81	4	-0.244	0.038	4.484	1699	WS00831_F12
5	57.017	102	2	-0.237	0.031	4.074	1699	WS00831_F12
6	45.897	117	1	0.268	0.075	4.681	1699	WS00831_F12
7	15.555	130	2	0.252	0.033	4.221	1699	WS00831_F12
11	17.088	203	2	0.233	0.042	4.576	1699	WS00831_F12
13	1.815	244	4	0.269	0.064	4.685	1699	WS00831_F12
1	62.128	11	1	-0.647	0.242	8.054	1700	WS00831_J16
3	15.147	44	2	-0.255	0.063	4.177	1700	WS00831_J16
3	95.157	59	2	0.268	0.097	10.502	1700	WS00831_J16
3	104.681	60	2	-0.375	0.119	12.574	1700	WS00831_J16
3	112.868	61	2	0.248	0.087	9.166	1700	WS00831_J16
3	114.747	62	2	0.346	0.099	10.474	1700	WS00831_J16
3	116.958	63	4	0.226	0.037	3.965	1700	WS00831_J16
4	0.000	71	4	-0.222	0.056	5.869	1700	WS00831_J16
6	45.897	117	1	0.214	0.050	4.148	1700	WS00831_J16
6	87.891	124	2	0.259	0.052	5.503	1700	WS00831_J16
6	89.401	125	2	0.260	0.055	6.147	1700	WS00831_J16
7	15.555	130	2	0.231	0.039	3.983	1700	WS00831_J16
7	15.555	130	3	-0.292	0.091	7.872	1700	WS00831_J16
14	43.392	250	2	0.227	0.066	6.969	1700	WS00831_J16
1	95.572	17	3	0.266	0.060	4.460	1701	WS00831_K23
10	78.479	193	3	-0.341	0.071	6.738	1701	WS00831_K23
11	35.604	207	3	-0.210	0.049	4.619	1701	WS00831_K23
11	46.026	217	1	-0.241	0.044	4.397	1701	WS00831_K23
2	48.839	29	1	-0.277	0.042	4.452	1702	WS01027_I04
5	57.017	102	2	-0.269	0.048	4.186	1702	WS01027_I04
5	57.017	102	4	0.372	0.074	8.423	1702	WS01027_I04
5	86.669	106	4	-0.249	0.090	4.755	1702	WS01027_I04
6	89.401	125	1	-0.196	0.046	4.865	1702	WS01027_I04
8	0.000	148	4	-0.244	0.046	5.110	1702	WS01027_I04
8	87.463	158	2	0.152	0.053	4.069	1702	WS01027_I04
9	17.951	169	2	0.198	0.040	4.194	1702	WS01027_I04
9	59.963	177	4	-0.327	0.063	6.684	1702	WS01027_I04
10	93.347	196	1	-0.410	0.085	4.066	1702	WS01027_I04
12	6.242	234	4	0.300	0.034	4.283	1702	WS01027_I04

13	0.000	243	4	0.284	0.105	5.997	1702	WS01027_I04
1	95.572	17	2	-0.218	0.046	4.601	1703	WS01027_I17
2	126.181	39	4	-0.742	0.253	12.654	1703	WS01027_I17
5	57.017	102	2	-0.282	0.051	5.466	1703	WS01027_I17
5	57.017	102	4	0.338	0.071	6.116	1703	WS01027_I17
5	86.669	106	4	-0.244	0.116	3.908	1703	WS01027_I17
6	49.100	118	2	0.256	0.115	5.387	1703	WS01027_I17
7	15.555	130	2	0.229	0.049	4.947	1703	WS01027_I17
9	0.000	163	1	-0.755	0.230	13.123	1703	WS01027_I17
11	64.014	222	4	-0.228	0.050	4.347	1703	WS01027_I17
12	28.493	228	2	0.333	0.093	5.030	1703	WS01027_I17
13	1.815	244	4	0.210	0.050	4.393	1703	WS01027_I17
1	95.572	17	3	0.288	0.058	4.878	1704	WS01027_K10
7	15.555	130	2	0.335	0.081	8.054	1704	WS01027_K10
10	78.479	193	3	-0.397	0.092	8.387	1704	WS01027_K10
10	93.347	196	2	0.478	0.115	4.220	1704	WS01027_K10
1	95.572	17	3	0.273	0.050	4.886	1705	WS01027_L09
2	93.504	32	2	-0.217	0.041	4.156	1706	WS01027_N22
2	120.617	38	3	-0.188	0.037	4.370	1706	WS01027_N22
2	127.147	40	3	0.182	0.038	4.561	1706	WS01027_N22
4	140.962	84	3	0.201	0.043	5.115	1706	WS01027_N22
10	78.479	193	3	-0.413	0.086	10.355	1706	WS01027_N22
10	86.151	195	3	0.307	0.067	8.075	1706	WS01027_N22
10	93.347	196	2	0.483	0.090	4.705	1706	WS01027_N22
10	104.214	198	2	-0.229	0.039	4.690	1706	WS01027_N22
11	17.088	203	1	0.220	0.052	4.424	1706	WS01027_N22
11	44.884	215	3	-0.245	0.034	4.171	1706	WS01027_N22
12	0.000	226	3	-0.199	0.040	4.742	1706	WS01027_N22
12	22.441	227	3	0.372	0.094	11.164	1706	WS01027_N22
12	22.629	237	1	-0.224	0.050	4.297	1706	WS01027_N22
12	32.473	238	1	-0.232	0.052	4.485	1706	WS01027_N22
14	0.000	246	2	0.187	0.042	4.278	1706	WS01027_N22
1	62.128	11	1	-0.471	0.074	4.266	1707	WS01027_O10
4	103.719	81	4	-0.289	0.066	6.805	1707	WS01027_O10
4	116.928	82	4	0.318	0.058	5.038	1707	WS01027_O10
4	124.132	83	1	0.477	0.087	5.028	1707	WS01027_O10
6	49.100	118	4	0.349	0.093	5.390	1707	WS01027_O10
10	93.347	196	2	0.477	0.121	4.971	1707	WS01027_O10
12	22.441	227	3	-0.229	0.045	4.629	1707	WS01027_O10
13	0.000	243	4	0.415	0.114	6.582	1707	WS01027_O10
1	62.128	11	1	-0.568	0.099	6.722	1708	WS00923_A07
2	93.504	32	4	-0.187	0.035	3.928	1708	WS00923_A07
4	92.039	79	4	-0.295	0.033	4.003	1708	WS00923_A07
4	124.132	83	1	0.394	0.058	3.959	1708	WS00923_A07
8	11.974	150	1	-0.173	0.036	4.201	1708	WS00923_A07
11	57.661	220	4	0.268	0.043	5.043	1708	WS00923_A07
11	64.014	222	4	-0.411	0.082	9.476	1708	WS00923_A07
13	0.000	243	4	0.534	0.139	9.499	1708	WS00923_A07
1	95.572	17	3	0.274	0.050	4.385	1709	WS00923_A19
2	126.181	39	2	-0.244	0.056	5.194	1709	WS00923_A19
3	137.891	64	1	-0.282	0.044	4.341	1709	WS00923_A19
3	154.505	69	1	0.182	0.042	3.951	1709	WS00923_A19
6	25.914	114	1	-0.179	0.045	4.301	1709	WS00923_A19
6	45.897	117	1	0.361	0.092	9.194	1709	WS00923_A19

6	49.100	118	4	0.356	0.079	3.976	1709	WS00923_A19
6	69.073	121	3	0.280	0.063	5.700	1709	WS00923_A19
11	46.026	217	1	-0.284	0.053	5.239	1709	WS00923_A19
3	112.868	61	2	-0.215	0.026	3.893	1710	WS00911_H14
5	3.484	90	2	-0.316	0.041	4.489	1710	WS00911_H14
5	52.820	99	3	-0.314	0.112	6.650	1710	WS00911_H14
7	62.787	139	2	0.229	0.027	4.042	1710	WS00911_H14
3	154.505	69	1	0.229	0.054	5.649	1711	WS00911_M19
4	92.039	79	4	-0.327	0.041	4.310	1711	WS00911_M19
4	140.962	84	3	-0.237	0.042	3.853	1711	WS00911_M19
6	45.897	117	1	0.247	0.038	4.016	1711	WS00911_M19
7	75.883	144	4	-0.311	0.050	4.265	1711	WS00911_M19
11	29.069	205	2	0.209	0.049	4.148	1711	WS00911_M19
11	46.026	217	1	-0.379	0.089	9.366	1711	WS00911_M19
1	131.686	23	1	-0.472	0.069	4.177	1712	WS00827_M21
2	95.880	34	1	-0.372	0.051	6.021	1712	WS00827_M21
6	106.702	128	3	0.254	0.058	4.470	1712	WS00827_M21
7	75.883	144	2	0.399	0.085	4.548	1712	WS00827_M21
7	75.883	144	4	-0.335	0.045	5.089	1712	WS00827_M21
9	3.127	168	1	-0.373	0.030	3.852	1712	WS00827_M21
14	0.000	246	1	-0.251	0.037	4.426	1712	WS00827_M21
1	40.464	7	4	0.320	0.060	4.609	1713	WS01031_B16
1	95.572	17	3	0.259	0.042	4.032	1713	WS01031_B16
2	48.839	29	1	0.238	0.032	4.167	1713	WS01031_B16
2	48.839	29	4	-0.273	0.055	4.425	1713	WS01031_B16
3	14.500	43	1	-0.243	0.037	4.898	1713	WS01031_B16
3	17.158	45	1	0.179	0.035	4.702	1713	WS01031_B16
7	15.555	130	2	0.278	0.083	4.605	1713	WS01031_B16
9	3.127	168	1	-0.288	0.055	4.082	1713	WS01031_B16
9	28.608	170	3	0.213	0.042	4.981	1713	WS01031_B16
10	35.204	189	1	-0.222	0.030	3.999	1713	WS01031_B16
10	86.151	195	3	0.215	0.043	4.786	1713	WS01031_B16
10	93.347	196	1	0.476	0.061	5.801	1713	WS01031_B16
14	0.000	246	1	-0.243	0.035	4.675	1713	WS01031_B16
1	40.464	7	4	0.325	0.059	5.740	1714	WS00828_I11
1	45.794	9	1	0.307	0.042	4.950	1714	WS00828_I11
2	48.839	29	4	-0.273	0.060	5.171	1714	WS00828_I11
3	17.158	45	1	0.205	0.045	5.320	1714	WS00828_I11
4	92.039	79	4	-0.301	0.053	5.196	1714	WS00828_I11
6	25.914	114	1	-0.179	0.035	4.092	1714	WS00828_I11
7	59.789	137	3	-0.207	0.049	4.506	1714	WS00828_I11
7	70.278	141	3	0.190	0.043	3.987	1714	WS00828_I11
10	93.347	196	1	0.455	0.089	4.983	1714	WS00828_I11
11	46.026	217	1	-0.292	0.052	6.207	1714	WS00828_I11
1	22.419	4	3	-0.310	0.082	3.950	1715	WS01031_G10
2	48.839	29	4	-0.267	0.051	5.693	1715	WS01031_G10
2	75.550	31	1	0.261	0.041	3.943	1715	WS01031_G10
4	55.925	76	1	0.201	0.027	3.918	1715	WS01031_G10
4	103.719	81	4	-0.189	0.032	4.798	1715	WS01031_G10
5	86.669	106	4	-0.340	0.085	4.137	1715	WS01031_G10
6	45.897	117	1	0.256	0.047	4.563	1715	WS01031_G10
7	15.555	130	2	0.320	0.081	7.486	1715	WS01031_G10
11	46.026	217	1	-0.258	0.046	4.484	1715	WS01031_G10
13	0.000	243	4	0.426	0.116	5.587	1715	WS01031_G10

14	9.923	247	1	0.293	0.063	6.099	1715	WS01031_G10
1	40.464	7	4	0.289	0.038	4.067	1716	WS0074_H18
1	95.572	17	3	0.262	0.060	4.855	1716	WS0074_H18
3	95.157	59	2	0.214	0.041	4.503	1716	WS0074_H18
6	106.702	128	2	0.407	0.119	3.982	1716	WS0074_H18
7	15.555	130	2	0.402	0.104	10.445	1716	WS0074_H18
1	95.572	17	3	0.261	0.053	4.525	1717	WS0074_I05
3	95.157	59	2	0.219	0.045	4.429	1717	WS0074_I05
3	104.681	60	2	-0.175	0.048	4.448	1717	WS0074_I05
5	86.669	106	4	-0.431	0.136	4.623	1717	WS0074_I05
7	15.555	130	2	0.365	0.101	9.439	1717	WS0074_I05
7	15.555	130	3	-0.253	0.061	5.363	1717	WS0074_I05
9	0.000	163	1	-0.505	0.097	5.635	1717	WS0074_I05
1	43.578	8	2	0.298	0.078	6.867	1718	WS0074_K05
1	69.854	13	2	-0.190	0.054	4.790	1718	WS0074_K05
1	71.592	14	2	-0.175	0.047	4.116	1718	WS0074_K05
1	77.177	15	2	-0.194	0.056	4.956	1718	WS0074_K05
5	86.669	106	4	-0.427	0.106	4.215	1718	WS0074_K05
7	62.787	139	4	-0.181	0.031	4.192	1718	WS0074_K05
7	67.154	140	4	-0.304	0.113	5.582	1718	WS0074_K05
7	75.883	144	2	0.550	0.146	8.902	1718	WS0074_K05
7	75.883	144	4	-0.360	0.070	7.232	1718	WS0074_K05
9	28.608	170	3	0.281	0.050	4.146	1718	WS0074_K05
9	59.963	177	4	-0.303	0.077	6.205	1718	WS0074_K05
10	78.479	193	4	-0.334	0.087	9.033	1718	WS0074_K05
10	95.795	197	4	0.170	0.040	4.096	1718	WS0074_K05
11	46.026	217	1	-0.225	0.048	4.754	1718	WS0074_K05
1	95.572	17	3	0.197	0.044	3.845	1719	WS0074_L12
4	55.925	76	1	0.195	0.034	4.992	1719	WS0074_L12
6	25.914	114	1	-0.224	0.040	4.203	1719	WS0074_L12
7	15.555	130	2	0.302	0.083	6.780	1719	WS0074_L12
7	67.154	140	4	-0.369	0.139	7.283	1719	WS0074_L12
7	73.169	142	1	0.572	0.115	6.007	1719	WS0074_L12
7	75.883	144	4	-0.440	0.117	11.460	1719	WS0074_L12
9	0.000	163	1	-0.556	0.127	6.839	1719	WS0074_L12
11	46.026	217	1	-0.237	0.049	5.228	1719	WS0074_L12
1	29.800	5	1	-0.250	0.040	3.928	1720	WS0074_N18
2	48.839	29	4	-0.261	0.059	4.740	1720	WS0074_N18
3	141.443	65	3	-0.272	0.061	4.229	1720	WS0074_N18
7	15.555	130	2	0.365	0.110	10.103	1720	WS0074_N18
9	0.000	163	1	-0.474	0.072	4.981	1720	WS0074_N18
10	104.214	198	2	0.232	0.039	4.225	1720	WS0074_N18
1	95.572	17	3	0.241	0.064	6.196	1721	WS0074_O24
3	154.505	69	4	-0.184	0.050	4.320	1721	WS0074_O24
4	55.925	76	1	0.163	0.047	4.797	1721	WS0074_O24
6	69.073	121	3	-0.259	0.060	6.428	1721	WS0074_O24
9	0.000	163	1	-0.612	0.136	8.393	1721	WS0074_O24
10	78.479	193	3	-0.269	0.045	4.803	1721	WS0074_O24
9	0.000	163	1	-0.502	0.108	5.666	1722	WS0075_A19
10	78.479	193	3	-0.322	0.055	5.516	1722	WS0075_A19
12	0.000	233	1	-0.231	0.055	4.083	1722	WS0075_A19
13	1.815	244	2	0.208	0.037	4.231	1722	WS0075_A19
1	38.326	6	4	-0.203	0.038	4.847	1723	WS0075_B22
1	40.464	7	4	0.272	0.051	5.397	1723	WS0075_B22

1	43.578	8	2	0.276	0.075	8.707	1723	WS0075_B22
3	47.928	49	1	0.238	0.073	5.203	1723	WS0075_B22
5	86.669	106	4	-0.387	0.228	5.413	1723	WS0075_B22
6	89.401	125	2	0.283	0.036	3.870	1723	WS0075_B22
7	15.555	130	2	0.334	0.061	7.358	1723	WS0075_B22
11	35.604	207	3	-0.284	0.068	5.841	1723	WS0075_B22
3	141.443	65	3	-0.289	0.084	3.855	1724	WS00937_G05
5	57.017	102	2	-0.239	0.055	6.245	1724	WS00937_G05
10	9.580	187	2	-0.306	0.076	6.038	1724	WS00937_G05
11	17.088	203	1	0.203	0.055	4.330	1724	WS00937_G05
1	95.572	17	3	0.234	0.049	4.013	1725	WS00937_G15
2	48.839	29	4	-0.231	0.045	4.232	1725	WS00937_G15
7	15.555	130	2	0.214	0.042	4.246	1725	WS00937_G15
8	62.580	155	1	0.390	0.123	5.123	1725	WS00937_G15
10	78.479	193	3	-0.338	0.077	6.484	1725	WS00937_G15
11	46.026	217	1	-0.224	0.056	4.811	1725	WS00937_G15
1	38.326	6	4	-0.233	0.043	4.776	1726	WS00937_I22
1	40.464	7	4	0.266	0.051	5.205	1726	WS00937_I22
1	133.992	24	2	0.224	0.036	4.205	1726	WS00937_I22
3	14.500	43	2	-0.277	0.046	4.918	1726	WS00937_I22
3	20.512	46	2	0.173	0.041	4.647	1726	WS00937_I22
6	25.742	113	3	-0.216	0.061	5.089	1726	WS00937_I22
6	89.401	125	1	-0.332	0.088	6.462	1726	WS00937_I22
8	50.001	154	2	-0.280	0.070	7.723	1726	WS00937_I22
9	49.013	173	2	0.180	0.036	4.096	1726	WS00937_I22
11	46.026	217	1	-0.271	0.099	7.264	1726	WS00937_I22
1	62.128	11	1	-0.477	0.194	4.344	1727	WS00937_K02
5	54.072	100	4	0.146	0.037	4.154	1727	WS00937_K02
14	22.402	248	3	-0.251	0.074	5.673	1727	WS00937_K02
1	2.788	2	4	-0.216	0.049	3.883	1728	WS00937_N03
1	62.128	11	2	0.228	0.073	4.307	1728	WS00937_N03
1	69.854	13	2	0.191	0.040	4.467	1728	WS00937_N03
1	71.592	14	2	0.197	0.042	4.779	1728	WS00937_N03
1	77.177	15	2	0.202	0.044	4.968	1728	WS00937_N03
1	95.572	17	2	0.204	0.045	5.000	1728	WS00937_N03
2	95.880	34	1	0.274	0.053	3.970	1728	WS00937_N03
4	97.576	80	2	0.182	0.034	4.376	1728	WS00937_N03
8	87.463	158	2	0.206	0.039	4.412	1728	WS00937_N03
11	17.088	203	1	0.234	0.066	4.922	1728	WS00937_N03
14	22.402	248	3	-0.258	0.046	5.556	1728	WS00937_N03
1	45.794	9	1	0.241	0.069	4.489	1729	WS00937_N14
2	120.617	38	3	-0.224	0.054	5.462	1729	WS00937_N14
2	127.147	40	3	0.138	0.039	4.001	1729	WS00937_N14
4	75.958	77	3	0.334	0.083	4.728	1729	WS00937_N14
6	56.188	120	2	0.329	0.058	7.305	1729	WS00937_N14
6	87.891	124	2	0.195	0.044	5.424	1729	WS00937_N14
9	28.608	170	2	0.243	0.033	4.204	1729	WS00937_N14
1	40.464	7	4	0.311	0.043	4.730	1730	WS01033_A11
1	45.794	9	1	0.381	0.068	7.050	1730	WS01033_A11
1	50.955	10	1	-0.290	0.048	5.018	1730	WS01033_A11
4	92.039	79	4	-0.267	0.032	3.988	1730	WS01033_A11
5	54.072	100	4	0.228	0.047	4.932	1730	WS01033_A11
6	89.401	125	1	-0.315	0.041	4.330	1730	WS01033_A11
8	11.974	150	1	-0.150	0.039	3.968	1730	WS01033_A11

9	3.127	168	1	-0.558	0.160	12.526	1730	WS01033_A11
10	35.204	189	2	0.262	0.075	6.367	1730	WS01033_A11
12	32.473	238	1	-0.164	0.047	4.778	1730	WS01033_A11
1	38.326	6	4	-0.321	0.060	5.843	1731	WS01033_B14
1	40.464	7	4	0.295	0.047	4.521	1731	WS01033_B14
1	43.578	8	2	0.292	0.075	6.657	1731	WS01033_B14
1	62.128	11	1	-0.486	0.111	4.522	1731	WS01033_B14
3	14.500	43	1	-0.331	0.076	7.571	1731	WS01033_B14
3	17.158	45	1	0.320	0.100	9.908	1731	WS01033_B14
3	47.928	49	1	0.211	0.050	4.933	1731	WS01033_B14
4	82.462	78	1	-0.261	0.056	5.560	1731	WS01033_B14
6	45.897	117	1	0.234	0.041	4.096	1731	WS01033_B14
8	95.937	159	3	0.189	0.047	4.271	1731	WS01033_B14
14	0.000	246	1	-0.265	0.052	5.160	1731	WS01033_B14
1	131.686	23	2	0.414	0.087	4.123	1732	WS0076_H16
2	127.147	40	3	0.212	0.047	5.483	1732	WS0076_H16
3	137.891	64	1	-0.297	0.063	6.360	1732	WS0076_H16
3	141.443	65	3	-0.253	0.056	4.405	1732	WS0076_H16
5	7.672	92	1	-0.344	0.065	6.759	1732	WS0076_H16
9	55.358	175	3	-0.377	0.051	3.909	1732	WS0076_H16
14	22.402	248	4	-0.367	0.092	5.239	1732	WS0076_H16
1	45.794	9	1	0.278	0.055	4.376	1733	WS0076_I02
1	50.955	10	1	-0.208	0.054	4.295	1733	WS0076_I02
2	48.839	29	4	-0.196	0.049	5.150	1733	WS0076_I02
3	137.891	64	1	-0.280	0.068	5.339	1733	WS0076_I02
5	3.484	90	2	0.295	0.050	4.206	1733	WS0076_I02
9	0.000	163	1	-0.423	0.070	3.941	1733	WS0076_I02
11	35.604	207	3	-0.209	0.080	6.403	1733	WS0076_I02
11	42.600	213	3	-0.202	0.071	5.732	1733	WS0076_I02
11	46.026	217	1	-0.234	0.064	5.094	1733	WS0076_I02
12	6.242	234	2	-0.209	0.031	3.911	1733	WS0076_I02
1	45.794	9	1	0.312	0.029	4.151	1734	WS0076_K04
3	141.443	65	3	-0.295	0.064	6.265	1734	WS0076_K04
9	28.608	170	3	0.173	0.033	3.876	1734	WS0076_K04
10	93.347	196	1	0.404	0.044	4.357	1734	WS0076_K04
1	38.326	6	4	-0.305	0.054	4.778	1735	WS0076_K13
1	40.464	7	4	0.365	0.077	7.112	1735	WS0076_K13
1	95.572	17	3	0.280	0.047	4.425	1735	WS0076_K13
3	14.500	43	1	-0.303	0.046	4.877	1735	WS0076_K13
3	17.158	45	1	0.197	0.052	5.467	1735	WS0076_K13
3	83.563	56	3	0.196	0.042	4.090	1735	WS0076_K13
7	15.555	130	2	0.242	0.049	4.257	1735	WS0076_K13
9	36.227	165	3	-0.203	0.046	4.492	1735	WS0076_K13
10	9.580	187	3	-0.252	0.077	5.278	1735	WS0076_K13
10	78.479	193	3	-0.345	0.054	5.374	1735	WS0076_K13
11	35.604	207	3	-0.207	0.039	3.884	1735	WS0076_K13
14	0.000	246	1	-0.245	0.039	4.205	1735	WS0076_K13
1	38.326	6	4	-0.393	0.084	9.149	1736	WS0076_M04
1	40.464	7	4	0.422	0.085	8.727	1736	WS0076_M04
1	62.128	11	1	-0.611	0.143	7.173	1736	WS0076_M04
3	17.158	45	1	0.217	0.041	4.324	1736	WS0076_M04
3	95.157	59	2	0.186	0.045	4.383	1736	WS0076_M04
5	86.669	106	4	-0.369	0.107	5.352	1736	WS0076_M04
7	15.555	130	2	0.277	0.070	5.940	1736	WS0076_M04

7	74.560	143	1	-0.295	0.051	5.359	1736	WS0076_M04
9	58.045	176	1	0.274	0.072	3.922	1736	WS0076_M04
10	21.864	188	1	0.266	0.037	3.908	1736	WS0076_M04
11	42.600	213	3	-0.212	0.052	4.061	1736	WS0076_M04
11	46.026	217	1	-0.227	0.042	4.410	1736	WS0076_M04
13	1.815	244	4	0.250	0.036	4.043	1736	WS0076_M04
14	0.000	246	1	-0.285	0.065	6.832	1736	WS0076_M04
1	82.301	16	4	0.301	0.038	4.553	1737	WS0076_M18
2	95.880	34	1	-0.344	0.050	5.292	1737	WS0076_M18
5	86.669	106	4	-0.581	0.147	10.573	1737	WS0076_M18
6	56.188	120	2	0.298	0.090	6.612	1737	WS0076_M18
7	62.787	139	4	-0.192	0.038	6.085	1737	WS0076_M18
7	67.154	140	4	-0.309	0.120	8.899	1737	WS0076_M18
7	75.883	144	4	-0.531	0.130	13.100	1737	WS0076_M18
8	39.090	152	4	-0.201	0.033	4.001	1737	WS0076_M18
9	3.127	168	1	-0.393	0.030	4.150	1737	WS0076_M18
9	49.013	173	2	-0.156	0.061	4.888	1737	WS0076_M18
10	21.864	188	1	0.443	0.082	8.794	1737	WS0076_M18
11	9.237	202	4	0.216	0.037	4.446	1737	WS0076_M18
11	40.960	210	4	0.351	0.048	4.565	1737	WS0076_M18
11	48.979	218	4	0.324	0.033	3.893	1737	WS0076_M18
12	51.382	242	1	-0.397	0.068	5.112	1737	WS0076_M18
1	40.464	7	4	0.289	0.044	4.350	1738	WS0076_O10
3	9.953	42	3	-0.193	0.046	4.190	1738	WS0076_O10
3	95.157	59	2	0.228	0.060	5.149	1738	WS0076_O10
7	15.555	130	2	0.248	0.056	4.333	1738	WS0076_O10
10	93.347	196	1	0.406	0.060	4.030	1738	WS0076_O10
14	0.000	246	1	-0.254	0.043	4.888	1738	WS0076_O10
1	38.326	6	4	-0.264	0.042	4.371	1739	WS0076_O19
1	40.464	7	4	0.330	0.057	5.816	1739	WS0076_O19
2	48.839	29	4	-0.287	0.053	5.492	1739	WS0076_O19
3	95.157	59	2	0.209	0.049	4.217	1739	WS0076_O19
5	86.669	106	4	-0.371	0.095	3.987	1739	WS0076_O19
9	3.127	168	1	-0.257	0.041	4.132	1739	WS0076_O19
11	46.026	217	1	-0.268	0.049	5.263	1739	WS0076_O19
1	38.326	6	4	-0.279	0.039	4.182	1740	WS00821_M04
1	40.464	7	4	0.338	0.053	5.394	1740	WS00821_M04
1	43.578	8	2	0.217	0.054	4.297	1740	WS00821_M04
1	95.572	17	3	0.242	0.058	4.223	1740	WS00821_M04
3	47.928	49	1	0.186	0.036	3.901	1740	WS00821_M04
3	95.157	59	2	0.199	0.048	4.136	1740	WS00821_M04
3	104.681	60	2	-0.182	0.050	3.944	1740	WS00821_M04
6	45.897	117	1	0.182	0.038	4.066	1740	WS00821_M04
7	15.555	130	2	0.328	0.084	6.730	1740	WS00821_M04
9	0.000	163	1	-0.426	0.078	4.018	1740	WS00821_M04
9	59.963	177	4	-0.238	0.059	5.284	1740	WS00821_M04
11	46.026	217	1	-0.279	0.059	6.429	1740	WS00821_M04
1	45.794	9	3	-0.226	0.050	5.031	1741	WS01027_A24
1	95.572	17	3	0.237	0.077	5.201	1741	WS01027_A24
1	107.333	19	3	0.312	0.084	8.749	1741	WS01027_A24
2	93.504	32	4	0.403	0.089	7.658	1741	WS01027_A24
2	120.617	38	4	0.328	0.058	4.367	1741	WS01027_A24
3	9.953	42	3	-0.262	0.036	3.952	1741	WS01027_A24
3	15.147	44	2	-0.289	0.064	3.896	1741	WS01027_A24

6	56.188	120	1	-0.276	0.037	3.910	1741	WS01027_A24
6	69.073	121	3	-0.209	0.049	5.100	1741	WS01027_A24
6	76.376	122	1	0.450	0.151	5.082	1741	WS01027_A24
6	81.996	123	3	-0.374	0.062	4.508	1741	WS01027_A24
6	89.401	125	1	-0.349	0.065	6.750	1741	WS01027_A24
9	0.000	166	1	-0.168	0.056	5.734	1741	WS01027_A24
9	2.011	167	4	0.240	0.069	5.769	1741	WS01027_A24
9	59.963	177	4	-0.279	0.066	5.725	1741	WS01027_A24
11	29.069	205	1	0.294	0.043	4.570	1741	WS01027_A24
11	48.979	218	2	-0.267	0.048	4.123	1741	WS01027_A24
11	64.014	222	4	0.239	0.054	4.154	1741	WS01027_A24
1	95.572	17	3	0.296	0.107	7.478	1742	WS00823_F20
3	95.157	59	2	0.194	0.052	5.386	1742	WS00823_F20
3	104.681	60	2	-0.216	0.036	3.899	1742	WS00823_F20
6	25.914	114	1	-0.325	0.083	6.730	1742	WS00823_F20
6	54.066	119	4	0.184	0.041	4.421	1742	WS00823_F20
6	106.702	128	2	0.434	0.127	5.003	1742	WS00823_F20
7	73.169	142	1	0.604	0.165	5.946	1742	WS00823_F20
7	73.169	142	2	-0.397	0.054	3.927	1742	WS00823_F20
7	75.883	144	2	0.505	0.094	6.967	1742	WS00823_F20
8	95.937	159	3	0.259	0.066	5.295	1742	WS00823_F20
9	0.000	163	1	-0.522	0.136	5.995	1742	WS00823_F20
9	28.608	170	3	0.233	0.048	4.015	1742	WS00823_F20
9	81.073	183	1	0.266	0.052	4.250	1742	WS00823_F20
10	80.088	194	2	-0.250	0.036	4.412	1742	WS00823_F20
10	93.347	196	2	0.427	0.118	3.906	1742	WS00823_F20
11	46.026	217	1	-0.222	0.072	5.771	1742	WS00823_F20
13	1.815	244	4	-0.425	0.125	13.623	1742	WS00823_F20
13	3.386	245	1	-0.377	0.182	14.531	1742	WS00823_F20
14	43.392	250	2	0.238	0.072	7.277	1742	WS00823_F20
1	69.854	13	2	-0.216	0.052	5.972	1743	WS00823_L15
1	71.592	14	2	-0.201	0.046	5.226	1743	WS00823_L15
1	77.177	15	2	-0.230	0.059	6.787	1743	WS00823_L15
3	95.157	59	2	0.232	0.057	7.115	1743	WS00823_L15
5	86.669	106	2	0.300	0.097	5.281	1743	WS00823_L15
7	15.555	130	2	0.240	0.034	3.937	1743	WS00823_L15
9	59.963	177	4	-0.278	0.079	6.748	1743	WS00823_L15
3	17.158	45	1	0.195	0.038	4.286	1744	WS00822_L20
7	73.169	142	1	0.511	0.093	4.877	1744	WS00822_L20
9	3.127	168	1	-0.396	0.077	6.850	1744	WS00822_L20
1	38.326	6	4	-0.336	0.048	5.689	1745	WS00822_P17
1	40.464	7	4	0.368	0.058	6.432	1745	WS00822_P17
1	95.572	17	3	0.216	0.056	3.923	1745	WS00822_P17
1	107.333	19	4	-0.303	0.059	6.933	1745	WS00822_P17
3	141.443	65	4	0.275	0.077	4.384	1745	WS00822_P17
6	25.914	114	1	-0.197	0.047	4.215	1745	WS00822_P17
9	3.127	168	1	-0.283	0.031	3.954	1745	WS00822_P17
1	40.464	7	3	0.459	0.154	15.922	1746	WS00916_G04
1	43.578	8	3	0.453	0.149	15.397	1746	WS00916_G04
1	45.794	9	3	-0.509	0.213	19.564	1746	WS00916_G04
1	127.805	21	3	-0.168	0.037	3.861	1746	WS00916_G04
4	82.462	78	1	-0.249	0.048	4.153	1746	WS00916_G04
5	0.000	89	3	0.317	0.066	5.582	1746	WS00916_G04
10	93.347	196	1	0.567	0.117	7.729	1746	WS00916_G04

14	46.637	252	3	0.279	0.055	5.644	1746	WS00916_G04
1	45.794	9	1	0.278	0.039	3.842	1747	WS00916_M05
1	50.955	10	1	-0.325	0.082	8.169	1747	WS00916_M05
2	120.617	38	3	0.341	0.063	6.369	1747	WS00916_M05
14	22.402	248	4	-0.282	0.047	3.854	1747	WS00916_M05
3	80.211	55	4	-0.254	0.085	8.992	1748	WS01029_E10
6	89.401	125	2	0.264	0.045	4.352	1748	WS01029_E10
7	15.555	130	2	0.318	0.081	8.259	1748	WS01029_E10
1	38.326	6	4	-0.394	0.058	6.718	1749	WS01029_E22
1	40.464	7	4	0.412	0.063	7.484	1749	WS01029_E22
1	45.794	9	1	0.381	0.065	7.359	1749	WS01029_E22
1	50.955	10	1	-0.322	0.063	7.090	1749	WS01029_E22
3	14.500	43	1	-0.238	0.039	4.314	1749	WS01029_E22
3	17.158	45	1	0.208	0.058	6.333	1749	WS01029_E22
3	47.928	49	1	0.198	0.054	5.999	1749	WS01029_E22
4	82.462	78	1	-0.292	0.049	5.475	1749	WS01029_E22
7	75.883	144	2	0.420	0.086	4.933	1749	WS01029_E22
7	75.883	144	4	-0.333	0.047	5.625	1749	WS01029_E22
9	0.000	163	1	-0.450	0.098	4.455	1749	WS01029_E22
1	43.578	8	2	0.211	0.036	4.226	1750	WS01029_H08
1	95.572	17	3	0.241	0.058	4.384	1750	WS01029_H08
3	137.891	64	3	-0.219	0.055	4.075	1750	WS01029_H08
4	103.719	81	4	-0.196	0.042	5.415	1750	WS01029_H08
6	87.891	124	2	0.307	0.097	11.212	1750	WS01029_H08
6	89.401	125	2	0.481	0.144	16.616	1750	WS01029_H08
7	15.555	130	2	0.271	0.040	4.598	1750	WS01029_H08
7	17.610	131	3	0.310	0.126	4.906	1750	WS01029_H08
9	0.000	163	1	-0.444	0.110	4.345	1750	WS01029_H08
10	9.580	187	3	-0.343	0.116	4.499	1750	WS01029_H08
10	78.479	193	3	-0.313	0.074	5.852	1750	WS01029_H08
11	27.405	204	4	0.312	0.047	4.478	1750	WS01029_H08
1	38.326	6	4	-0.311	0.034	4.080	1751	WS01029_I01
1	40.464	7	4	0.405	0.059	7.592	1751	WS01029_I01
1	62.128	11	1	-0.561	0.079	6.701	1751	WS01029_I01
4	92.039	79	4	-0.411	0.075	8.259	1751	WS01029_I01
4	124.132	83	1	0.408	0.057	4.842	1751	WS01029_I01
4	142.917	85	1	-0.219	0.053	6.267	1751	WS01029_I01
6	49.100	118	4	0.380	0.046	3.871	1751	WS01029_I01
6	106.702	128	2	0.394	0.165	4.217	1751	WS01029_I01
7	15.555	130	2	0.252	0.059	4.011	1751	WS01029_I01
9	59.963	177	4	-0.225	0.043	6.276	1751	WS01029_I01
10	78.479	193	4	-0.301	0.046	5.896	1751	WS01029_I01
11	46.026	217	1	-0.272	0.048	5.723	1751	WS01029_I01
13	0.000	243	4	0.446	0.064	5.546	1751	WS01029_I01
1	38.326	6	4	-0.171	0.038	4.264	1752	WS01029_J04
1	40.464	7	4	0.252	0.057	5.004	1752	WS01029_J04
1	43.578	8	2	0.210	0.047	5.962	1752	WS01029_J04
1	69.854	13	2	-0.144	0.035	4.308	1752	WS01029_J04
3	95.157	59	2	0.150	0.036	4.451	1752	WS01029_J04
4	92.039	79	4	-0.193	0.046	4.928	1752	WS01029_J04
8	62.580	155	1	0.382	0.152	4.559	1752	WS01029_J04
11	64.014	222	4	-0.325	0.079	6.601	1752	WS01029_J04
1	62.128	11	1	-0.462	0.091	4.065	1753	WS01029_J15
2	75.550	31	1	0.255	0.038	4.504	1753	WS01029_J15

3	47.928	49	1	0.280	0.067	7.983	1753	WS01029_J15
4	82.462	78	1	-0.292	0.052	6.267	1753	WS01029_J15
9	59.963	177	4	-0.251	0.070	5.375	1753	WS01029_J15
10	70.246	190	2	0.234	0.073	5.047	1753	WS01029_J15
11	60.464	221	1	-0.283	0.032	3.933	1753	WS01029_J15
4	92.039	79	4	-0.410	0.079	5.690	1754	WS01029_L01
5	0.000	89	3	0.379	0.084	6.923	1754	WS01029_L01
6	45.897	117	1	0.226	0.050	4.858	1754	WS01029_L01
10	78.479	193	3	-0.343	0.076	6.786	1754	WS01029_L01
1	95.572	17	3	0.237	0.047	4.456	1755	WS01029_L15
6	49.100	118	4	0.373	0.085	4.152	1755	WS01029_L15
9	3.127	168	1	-0.301	0.053	3.910	1755	WS01029_L15
9	59.963	177	4	-0.224	0.067	5.758	1755	WS01029_L15
10	70.246	190	2	0.291	0.114	8.610	1755	WS01029_L15
10	78.479	193	3	-0.263	0.046	4.833	1755	WS01029_L15
1	2.788	2	4	0.174	0.039	3.846	1756	WS01031_K05
1	69.854	13	2	-0.194	0.038	4.247	1756	WS01031_K05
2	48.839	29	4	-0.319	0.060	5.215	1756	WS01031_K05
3	80.211	55	4	-0.187	0.042	4.673	1756	WS01031_K05
3	116.958	63	4	0.154	0.035	3.887	1756	WS01031_K05
6	26.350	115	2	-0.288	0.073	8.031	1756	WS01031_K05
7	75.883	144	4	-0.250	0.042	4.225	1756	WS01031_K05
8	0.000	148	4	0.168	0.039	4.302	1756	WS01031_K05
9	59.963	177	4	-0.172	0.041	4.420	1756	WS01031_K05
14	43.392	250	2	0.205	0.036	3.989	1756	WS01031_K05
6	45.897	117	1	0.253	0.063	6.480	1757	WS01031_K20
6	49.100	118	4	0.418	0.121	4.979	1757	WS01031_K20
9	0.000	163	1	-0.512	0.095	5.905	1757	WS01031_K20
9	59.963	177	4	-0.299	0.085	6.982	1757	WS01031_K20
10	70.246	190	2	0.246	0.066	5.662	1757	WS01031_K20
1	40.464	7	4	0.278	0.058	4.162	1758	WS01031_M06
1	95.572	17	3	0.247	0.062	5.407	1758	WS01031_M06
3	116.958	63	4	0.214	0.054	4.978	1758	WS01031_M06
6	69.073	121	3	-0.160	0.041	3.851	1758	WS01031_M06
9	0.000	163	1	-0.472	0.109	4.914	1758	WS01031_M06
9	59.963	177	4	-0.261	0.071	6.781	1758	WS01031_M06
1	40.464	7	4	0.327	0.048	4.221	1759	WS01031_M16
3	47.928	49	1	0.295	0.072	7.441	1759	WS01031_M16
4	124.132	83	1	0.543	0.106	6.449	1759	WS01031_M16
4	142.917	85	1	-0.173	0.044	4.535	1759	WS01031_M16
6	106.702	128	2	0.527	0.144	8.024	1759	WS01031_M16
7	17.610	131	3	0.336	0.134	7.092	1759	WS01031_M16
9	0.000	163	1	-0.497	0.129	5.449	1759	WS01031_M16
10	78.479	193	3	-0.232	0.046	4.379	1759	WS01031_M16
12	6.242	234	2	0.219	0.055	4.986	1759	WS01031_M16
1	95.572	17	3	0.294	0.061	5.558	1760	WS01031_N22
2	102.814	36	1	0.251	0.043	5.119	1760	WS01031_N22
3	137.891	64	3	-0.166	0.038	4.449	1760	WS01031_N22
3	141.443	65	3	-0.236	0.062	4.132	1760	WS01031_N22
6	49.100	118	4	0.330	0.135	4.093	1760	WS01031_N22
7	15.555	130	2	0.331	0.082	7.104	1760	WS01031_N22
7	74.560	143	1	-0.245	0.043	4.376	1760	WS01031_N22
9	0.000	163	1	-0.520	0.090	6.128	1760	WS01031_N22
9	59.963	177	4	-0.248	0.068	5.109	1760	WS01031_N22

1	62.128	11	1	-0.454	0.092	4.196	1761	WS01031_O07
1	69.854	13	2	-0.183	0.040	4.068	1761	WS01031_O07
3	15.147	44	2	-0.170	0.069	4.267	1761	WS01031_O07
3	47.928	49	1	0.206	0.047	4.027	1761	WS01031_O07
3	80.211	55	4	-0.143	0.042	4.398	1761	WS01031_O07
4	82.462	78	1	-0.250	0.085	7.299	1761	WS01031_O07
6	49.100	118	4	0.271	0.077	4.134	1761	WS01031_O07
6	87.891	124	2	0.200	0.042	4.347	1761	WS01031_O07
6	89.401	125	2	0.332	0.057	5.753	1761	WS01031_O07
7	74.560	143	1	-0.298	0.057	5.073	1761	WS01031_O07
8	62.580	155	1	0.388	0.123	5.579	1761	WS01031_O07
8	78.389	157	1	0.279	0.077	6.593	1761	WS01031_O07
8	87.463	158	1	-0.261	0.068	5.821	1761	WS01031_O07
13	1.815	244	4	0.217	0.041	4.959	1761	WS01031_O07
3	154.505	69	1	0.206	0.049	4.730	1762	WS00825_A15
9	0.000	166	1	-0.236	0.048	4.465	1762	WS00825_A15
9	49.013	173	1	0.217	0.055	5.658	1762	WS00825_A15
10	9.580	187	3	-0.213	0.078	3.871	1762	WS00825_A15
10	93.347	196	1	0.448	0.119	4.817	1762	WS00825_A15
11	64.014	222	4	-0.302	0.045	4.701	1762	WS00825_A15
1	22.419	4	3	-0.281	0.068	4.190	1763	WS00825_B03
2	95.880	34	3	0.298	0.046	5.505	1763	WS00825_B03
2	101.076	35	3	-0.302	0.045	5.392	1763	WS00825_B03
3	80.211	55	4	-0.176	0.056	3.853	1763	WS00825_B03
3	104.681	60	2	0.265	0.071	7.769	1763	WS00825_B03
3	112.868	61	2	-0.297	0.071	7.779	1763	WS00825_B03
3	114.747	62	2	-0.218	0.069	7.482	1763	WS00825_B03
5	52.820	99	3	-0.303	0.060	7.406	1763	WS00825_B03
8	39.090	152	4	-0.195	0.068	4.667	1763	WS00825_B03
9	0.000	163	1	-0.683	0.177	10.883	1763	WS00825_B03
9	0.000	166	2	0.184	0.053	5.740	1763	WS00825_B03
11	9.237	202	4	-0.216	0.078	5.294	1763	WS00825_B03
11	27.405	204	1	-0.342	0.107	8.720	1763	WS00825_B03
11	27.405	204	4	0.359	0.103	7.125	1763	WS00825_B03
11	29.069	205	1	-0.259	0.074	5.799	1763	WS00825_B03
14	0.000	246	2	-0.212	0.048	4.770	1763	WS00825_B03
3	17.158	45	1	0.209	0.054	4.698	1764	WS01012_H09
5	54.072	100	4	0.243	0.044	4.930	1764	WS01012_H09
9	49.013	173	1	0.237	0.070	5.969	1764	WS01012_H09
11	57.661	220	4	0.281	0.048	5.515	1764	WS01012_H09
11	64.014	222	4	-0.383	0.065	6.802	1764	WS01012_H09
1	38.326	6	4	-0.197	0.036	4.252	1765	WS01012_I14
1	62.128	11	1	-0.476	0.104	4.384	1765	WS01012_I14
3	148.745	68	4	-0.152	0.035	4.077	1765	WS01012_I14
4	103.719	81	4	-0.351	0.070	9.084	1765	WS01012_I14
6	45.897	117	1	0.272	0.047	4.637	1765	WS01012_I14
6	49.100	118	4	0.270	0.106	3.979	1765	WS01012_I14
8	87.463	158	2	0.225	0.048	4.251	1765	WS01012_I14
11	35.604	207	3	-0.263	0.048	3.865	1765	WS01012_I14
13	1.815	244	4	0.253	0.054	6.250	1765	WS01012_I14
14	0.000	246	1	-0.248	0.048	4.479	1765	WS01012_I14
5	54.072	100	4	0.340	0.094	9.790	1766	WS01012_J13
5	57.017	102	4	-0.234	0.045	4.552	1766	WS01012_J13
8	95.937	159	3	0.234	0.054	4.551	1766	WS01012_J13

10	93.347	196	1	0.428	0.085	4.394	1766	WS01012_J13
10	104.214	198	4	-0.233	0.033	3.888	1766	WS01012_J13
11	35.604	207	3	-0.170	0.048	4.002	1766	WS01012_J13
11	64.014	222	4	-0.315	0.057	5.395	1766	WS01012_J13
12	0.000	233	1	-0.223	0.048	4.401	1766	WS01012_J13
1	22.419	4	4	-0.360	0.070	5.284	1767	WS01012_K03
2	93.504	32	4	-0.213	0.038	4.857	1767	WS01012_K03
3	9.953	42	4	0.304	0.051	5.137	1767	WS01012_K03
3	17.158	45	1	0.172	0.051	4.935	1767	WS01012_K03
4	124.132	83	1	0.567	0.111	8.007	1767	WS01012_K03
4	142.917	85	1	-0.288	0.058	5.648	1767	WS01012_K03
6	49.100	118	4	0.489	0.132	9.664	1767	WS01012_K03
7	73.169	142	1	0.565	0.082	5.887	1767	WS01012_K03
10	21.864	188	1	0.315	0.048	4.729	1767	WS01012_K03
11	17.088	203	2	0.155	0.049	4.933	1767	WS01012_K03
11	46.026	217	1	-0.269	0.039	3.963	1767	WS01012_K03
1	38.326	6	4	-0.300	0.048	4.770	1768	WS01012_L24
1	40.464	7	4	0.311	0.047	4.120	1768	WS01012_L24
1	95.572	17	3	0.248	0.055	4.678	1768	WS01012_L24
7	15.555	130	2	0.269	0.065	4.661	1768	WS01012_L24
7	75.883	144	4	-0.251	0.040	3.879	1768	WS01012_L24
9	3.127	168	1	-0.330	0.071	6.336	1768	WS01012_L24
1	38.326	6	4	-0.298	0.052	4.651	1769	WS01012_M13
1	40.464	7	4	0.340	0.059	5.771	1769	WS01012_M13
1	45.794	9	1	0.280	0.040	3.944	1769	WS01012_M13
1	95.572	17	3	0.251	0.065	5.584	1769	WS01012_M13
3	9.953	42	3	-0.241	0.052	4.406	1769	WS01012_M13
4	0.000	71	4	-0.280	0.046	4.908	1769	WS01012_M13
6	76.376	122	1	0.447	0.120	5.587	1769	WS01012_M13
6	89.401	125	1	-0.335	0.057	5.778	1769	WS01012_M13
7	15.555	130	2	0.392	0.106	9.646	1769	WS01012_M13
7	17.610	131	3	0.262	0.155	4.766	1769	WS01012_M13
9	0.000	163	1	-0.470	0.093	4.876	1769	WS01012_M13
10	9.580	187	3	-0.349	0.106	5.464	1769	WS01012_M13
10	21.864	188	1	0.350	0.071	7.221	1769	WS01012_M13
7	15.555	130	2	0.299	0.075	7.634	1770	WS01012_O04
9	3.127	168	1	-0.365	0.080	4.808	1770	WS01012_O04
12	32.473	238	1	-0.175	0.050	4.425	1770	WS01012_O04
1	95.572	17	3	0.266	0.078	4.561	1771	WS01012_O22
2	48.839	29	4	-0.226	0.041	3.947	1771	WS01012_O22
3	69.071	52	1	0.342	0.093	4.886	1771	WS01012_O22
4	82.462	78	1	-0.256	0.048	4.039	1771	WS01012_O22
7	15.555	130	2	0.364	0.100	10.528	1771	WS01012_O22
7	17.610	131	3	0.256	0.119	4.862	1771	WS01012_O22
12	22.629	237	1	0.232	0.070	5.742	1771	WS01012_O22
1	38.326	6	4	-0.487	0.095	11.069	1772	WS01010_P24
1	40.464	7	4	0.543	0.112	12.743	1772	WS01010_P24
1	43.578	8	2	0.214	0.055	5.045	1772	WS01010_P24
1	45.794	9	1	0.393	0.064	6.316	1772	WS01010_P24
1	45.794	9	3	-0.246	0.078	5.941	1772	WS01010_P24
1	50.955	10	1	-0.228	0.042	4.081	1772	WS01010_P24
3	47.928	49	1	0.241	0.063	6.024	1772	WS01010_P24
4	0.000	71	4	-0.274	0.036	4.299	1772	WS01010_P24
6	89.401	125	1	-0.339	0.052	5.196	1772	WS01010_P24

6	95.249	126	1	0.361	0.132	4.827	1772	WS01010_P24
7	15.555	130	2	0.256	0.047	4.317	1772	WS01010_P24
9	3.127	168	1	-0.402	0.054	4.569	1772	WS01010_P24
9	59.963	177	4	-0.232	0.058	7.570	1772	WS01010_P24
10	21.864	188	1	0.319	0.049	4.883	1772	WS01010_P24
11	9.237	202	4	0.178	0.035	4.007	1772	WS01010_P24
12	22.629	237	1	0.277	0.068	6.545	1772	WS01010_P24
1	131.686	23	2	0.436	0.102	4.547	1773	WS01011_A16
4	43.146	74	3	0.242	0.047	4.922	1773	WS01011_A16
4	124.132	83	1	0.573	0.166	6.598	1773	WS01011_A16
9	0.000	166	1	0.220	0.065	6.688	1773	WS01011_A16
9	81.073	183	1	-0.292	0.038	4.010	1773	WS01011_A16
1	40.464	7	4	0.242	0.071	4.171	1774	WS01011_C05
1	43.578	8	2	0.250	0.061	4.847	1774	WS01011_C05
4	43.146	74	3	-0.178	0.033	4.404	1774	WS01011_C05
6	49.100	118	2	0.248	0.118	5.204	1774	WS01011_C05
6	106.702	128	2	0.458	0.118	5.612	1774	WS01011_C05
1	127.805	21	3	0.214	0.026	4.023	1775	WS01011_D03
6	49.100	118	2	0.242	0.077	3.963	1775	WS01011_D03
7	15.555	130	3	0.283	0.049	7.476	1775	WS01011_D03
9	36.227	165	3	0.270	0.051	8.010	1775	WS01011_D03
14	46.637	252	3	-0.246	0.029	4.458	1775	WS01011_D03
1	29.800	5	1	0.223	0.036	5.157	1776	WS01011_F11
3	14.500	43	1	0.267	0.037	5.278	1776	WS01011_F11
3	17.158	45	1	-0.197	0.036	5.255	1776	WS01011_F11
3	83.563	56	3	-0.180	0.028	4.367	1776	WS01011_F11
4	43.146	74	3	-0.189	0.028	4.252	1776	WS01011_F11
4	55.925	76	1	0.229	0.045	6.526	1776	WS01011_F11
7	59.789	137	3	0.190	0.029	4.429	1776	WS01011_F11
9	81.073	183	1	-0.238	0.032	4.730	1776	WS01011_F11
12	0.000	226	3	-0.271	0.036	5.639	1776	WS01011_F11
1	40.464	7	4	0.282	0.048	4.886	1777	WS01011_G08
1	107.333	19	3	0.239	0.080	6.892	1777	WS01011_G08
5	3.484	90	2	0.295	0.087	7.052	1777	WS01011_G08
5	16.219	93	2	-0.207	0.034	4.260	1777	WS01011_G08
7	15.555	130	2	0.250	0.042	5.099	1777	WS01011_G08
7	17.610	131	3	0.300	0.114	4.861	1777	WS01011_G08
7	105.477	147	2	0.222	0.051	4.099	1777	WS01011_G08
9	36.227	165	4	0.277	0.051	6.136	1777	WS01011_G08
1	95.572	17	3	0.238	0.044	4.782	1778	WS01011_I09
3	116.958	63	4	0.230	0.068	5.485	1778	WS01011_I09
7	74.560	143	1	-0.257	0.052	4.297	1778	WS01011_I09
9	0.000	163	1	-0.429	0.068	4.122	1778	WS01011_I09
9	59.963	177	4	-0.229	0.057	4.579	1778	WS01011_I09
1	62.128	11	1	-0.470	0.108	4.232	1779	WS01011_J03
4	82.462	78	1	-0.237	0.053	4.666	1779	WS01011_J03
7	15.555	130	2	0.310	0.081	8.143	1779	WS01011_J03
7	73.169	142	1	0.509	0.107	4.202	1779	WS01011_J03
12	6.242	234	2	-0.144	0.023	3.895	1779	WS01011_J03
14	9.923	247	1	0.228	0.049	4.345	1779	WS01011_J03
3	47.928	49	1	0.293	0.070	5.877	1780	WS0101_C24
4	103.719	81	4	-0.241	0.062	7.180	1780	WS0101_C24
7	15.555	130	2	0.317	0.066	6.808	1780	WS0101_C24
7	67.154	140	4	-0.278	0.093	3.878	1780	WS0101_C24

9	0.000	163	1	-0.509	0.138	5.705	1780	WS0101_C24
9	17.951	169	2	0.272	0.043	4.681	1780	WS0101_C24
9	59.963	177	4	-0.221	0.054	5.129	1780	WS0101_C24
11	46.026	217	1	-0.179	0.049	4.052	1780	WS0101_C24
13	0.000	243	4	0.503	0.168	7.120	1780	WS0101_C24
1	95.572	17	3	0.252	0.055	4.586	1781	WS0101_I03
6	69.073	121	3	-0.239	0.057	5.262	1781	WS0101_I03
7	15.555	130	2	0.241	0.046	4.468	1781	WS0101_I03
9	59.963	177	4	-0.262	0.054	4.860	1781	WS0101_I03
11	46.026	217	1	-0.212	0.050	4.558	1781	WS0101_I03
3	154.505	69	1	0.263	0.049	5.592	1782	WS0079_G19
4	82.462	78	1	-0.257	0.040	4.576	1782	WS0079_G19
5	54.072	100	4	0.235	0.044	4.387	1782	WS0079_G19
9	49.013	173	1	0.205	0.036	4.083	1782	WS0079_G19
10	93.347	196	1	0.523	0.102	6.569	1782	WS0079_G19
11	46.026	217	1	-0.224	0.043	4.799	1782	WS0079_G19
11	57.661	220	4	0.232	0.045	4.461	1782	WS0079_G19
11	64.014	222	4	-0.336	0.063	6.446	1782	WS0079_G19
1	22.419	4	3	-0.275	0.059	3.994	1783	WS00938_F19
1	95.572	17	2	0.233	0.048	4.778	1783	WS00938_F19
3	14.500	43	1	-0.262	0.052	4.528	1783	WS00938_F19
3	17.158	45	1	0.223	0.059	5.125	1783	WS00938_F19
5	54.072	100	4	0.231	0.062	5.407	1783	WS00938_F19
8	50.001	154	2	-0.164	0.041	3.876	1783	WS00938_F19
9	49.013	173	1	0.229	0.067	5.525	1783	WS00938_F19
9	58.045	176	1	0.341	0.078	5.320	1783	WS00938_F19
9	59.963	177	1	0.244	0.049	4.216	1783	WS00938_F19
9	69.046	181	1	-0.254	0.064	4.297	1783	WS00938_F19
12	0.000	233	1	-0.205	0.057	4.932	1783	WS00938_F19
5	54.072	100	4	0.187	0.040	4.390	1784	WS0079_N23
6	89.401	125	2	0.286	0.062	4.959	1784	WS0079_N23
7	67.154	140	4	-0.303	0.095	5.377	1784	WS0079_N23
7	73.169	142	1	0.682	0.157	8.825	1784	WS0079_N23
7	75.883	144	4	-0.371	0.077	7.882	1784	WS0079_N23
9	3.127	168	1	-0.283	0.035	3.995	1784	WS0079_N23
7	75.883	144	2	-0.403	0.044	4.172	1785	WS00938_O17
7	105.477	147	2	0.235	0.058	5.305	1785	WS00938_O17
10	21.864	188	1	0.321	0.089	5.668	1785	WS00938_O17
12	6.242	234	4	0.361	0.073	12.909	1785	WS00938_O17
13	1.815	244	4	0.300	0.071	9.536	1785	WS00938_O17
14	22.402	248	4	-0.353	0.059	7.808	1785	WS00938_O17
14	46.637	252	4	-0.414	0.092	12.530	1785	WS00938_O17
6	25.914	114	1	-0.280	0.082	6.325	1786	WS0093_H06
8	50.001	154	2	-0.269	0.079	6.543	1786	WS0093_H06
10	70.246	190	2	0.217	0.043	4.775	1786	WS0093_H06
12	22.441	227	3	0.237	0.033	4.143	1786	WS0093_H06
12	0.000	233	1	-0.209	0.053	4.086	1786	WS0093_H06
13	3.386	245	1	-0.219	0.066	5.129	1786	WS0093_H06
1	38.326	6	4	-0.282	0.039	4.447	1787	WS0093_H17
1	40.464	7	4	0.330	0.047	4.919	1787	WS0093_H17
2	75.550	31	1	0.268	0.032	3.946	1787	WS0093_H17
4	92.039	79	4	-0.364	0.059	6.696	1787	WS0093_H17
4	103.719	81	4	-0.211	0.041	6.155	1787	WS0093_H17
9	49.013	173	1	0.192	0.034	4.635	1787	WS0093_H17

10	93.347	196	1	0.421	0.077	4.254	1787	WS0093_H17
11	57.661	220	4	0.286	0.052	5.935	1787	WS0093_H17
11	64.014	222	4	-0.398	0.075	8.501	1787	WS0093_H17
13	0.000	243	4	0.369	0.058	3.904	1787	WS0093_H17
5	54.072	100	4	0.226	0.038	4.020	1788	WS0093_J21
7	59.789	137	3	-0.161	0.047	3.853	1788	WS0093_J21
9	3.127	168	1	-0.342	0.064	5.553	1788	WS0093_J21
10	21.864	188	1	0.314	0.044	4.482	1788	WS0093_J21
11	46.026	217	1	-0.249	0.043	4.605	1788	WS0093_J21
11	64.014	222	4	-0.321	0.050	5.194	1788	WS0093_J21
12	0.000	233	1	-0.202	0.046	4.810	1788	WS0093_J21
12	32.473	238	1	-0.216	0.048	5.050	1788	WS0093_J21
1	38.326	6	4	-0.307	0.034	4.051	1789	WS0093_K17
1	40.464	7	4	0.360	0.048	4.675	1789	WS0093_K17
1	62.128	11	1	-0.641	0.111	8.071	1789	WS0093_K17
1	95.572	17	3	0.247	0.080	5.042	1789	WS0093_K17
4	103.719	81	4	-0.178	0.031	4.846	1789	WS0093_K17
7	15.555	130	2	0.297	0.072	5.737	1789	WS0093_K17
7	67.154	140	4	-0.202	0.054	3.867	1789	WS0093_K17
9	3.127	168	1	-0.321	0.030	4.316	1789	WS0093_K17
10	93.347	196	1	0.491	0.163	5.789	1789	WS0093_K17
11	64.014	222	4	-0.310	0.041	4.646	1789	WS0093_K17
13	0.000	243	4	0.446	0.071	5.281	1789	WS0093_K17
2	48.839	29	4	-0.309	0.053	5.759	1790	WS0093_M20
3	95.157	59	2	0.220	0.043	4.000	1790	WS0093_M20
7	15.555	130	2	0.285	0.054	5.053	1790	WS0093_M20
7	74.560	143	1	-0.311	0.041	4.128	1790	WS0093_M20
9	3.127	168	1	-0.290	0.075	4.671	1790	WS0093_M20
11	0.000	199	3	0.272	0.056	3.858	1790	WS0093_M20
1	82.301	16	4	0.261	0.041	3.902	1791	WS0093_N09
1	95.572	17	3	0.285	0.074	6.024	1791	WS0093_N09
2	48.839	29	4	-0.227	0.041	4.138	1791	WS0093_N09
7	15.555	130	2	0.319	0.085	7.643	1791	WS0093_N09
7	74.560	143	1	-0.248	0.041	4.093	1791	WS0093_N09
10	104.214	198	4	-0.286	0.037	3.936	1791	WS0093_N09
11	46.026	217	1	-0.246	0.053	5.309	1791	WS0093_N09
14	0.000	246	1	-0.209	0.039	3.850	1791	WS0093_N09
1	40.464	7	4	0.223	0.045	4.443	1792	WS0093_P14
2	48.839	29	1	-0.267	0.044	4.113	1792	WS0093_P14
2	102.814	36	1	0.204	0.039	3.882	1792	WS0093_P14
5	7.672	92	4	-0.221	0.043	4.419	1792	WS0093_P14
6	87.891	124	2	0.244	0.053	5.030	1792	WS0093_P14
6	89.401	125	2	0.250	0.056	4.932	1792	WS0093_P14
7	15.555	130	2	0.238	0.047	4.407	1792	WS0093_P14
7	17.610	131	3	0.215	0.130	4.255	1792	WS0093_P14
7	74.560	143	1	-0.255	0.058	5.375	1792	WS0093_P14
9	0.000	163	1	-0.422	0.078	3.913	1792	WS0093_P14
12	0.000	226	3	-0.268	0.063	5.274	1792	WS0093_P14
12	22.441	227	3	0.256	0.096	7.584	1792	WS0093_P14
13	1.815	244	4	0.281	0.065	6.723	1792	WS0093_P14
1	69.854	13	2	-0.289	0.090	7.299	1793	WS0101_A09
1	71.592	14	2	-0.270	0.078	6.313	1793	WS0101_A09
3	17.158	45	1	0.162	0.037	3.934	1793	WS0101_A09
7	15.555	130	2	0.316	0.081	6.807	1793	WS0101_A09

9	28.608	170	3	0.261	0.051	5.839	1793	WS0101_A09
9	58.045	176	1	0.238	0.049	3.962	1793	WS0101_A09
9	59.963	177	1	0.241	0.040	4.261	1793	WS0101_A09
10	86.151	195	3	0.224	0.050	5.722	1793	WS0101_A09
10	93.347	196	1	0.396	0.048	3.990	1793	WS0101_A09
11	56.921	219	2	-0.298	0.165	5.541	1793	WS0101_A09
2	48.839	29	4	-0.251	0.055	5.189	1794	WS01011_M07
4	43.146	74	3	-0.228	0.053	4.997	1794	WS01011_M07
8	78.389	157	1	0.216	0.044	4.012	1794	WS01011_M07
11	46.026	217	1	-0.254	0.056	5.043	1794	WS01011_M07
2	48.839	29	1	-0.250	0.048	4.631	1795	WS01011_M22
3	116.958	63	4	0.157	0.049	4.042	1795	WS01011_M22
4	55.925	76	1	0.286	0.062	6.705	1795	WS01011_M22
9	0.000	166	1	0.252	0.050	4.906	1795	WS01011_M22
9	59.963	177	1	-0.230	0.042	4.018	1795	WS01011_M22
9	69.046	181	1	0.275	0.056	4.253	1795	WS01011_M22
11	46.026	217	1	-0.263	0.053	5.192	1795	WS01011_M22
14	43.392	250	2	0.238	0.060	5.438	1795	WS01011_M22
1	29.800	5	1	0.282	0.048	6.621	1796	WS01011_P04
3	14.500	43	1	0.223	0.029	3.993	1796	WS01011_P04
3	141.443	65	3	0.287	0.056	6.766	1796	WS01011_P04
4	55.925	76	1	0.285	0.072	10.055	1796	WS01011_P04
6	49.100	118	2	0.249	0.170	4.817	1796	WS01011_P04
7	15.555	130	3	0.220	0.027	4.097	1796	WS01011_P04
9	36.227	165	3	0.195	0.034	5.105	1796	WS01011_P04
9	58.045	176	1	-0.279	0.049	5.931	1796	WS01011_P04
9	59.963	177	1	-0.198	0.034	4.689	1796	WS01011_P04
9	69.046	181	1	0.201	0.037	4.530	1796	WS01011_P04
9	81.073	183	1	-0.256	0.033	4.773	1796	WS01011_P04
14	0.000	246	1	0.224	0.028	3.892	1796	WS01011_P04
2	95.880	34	1	0.296	0.061	4.611	1797	WS01011_P21
4	103.719	81	4	0.320	0.076	8.581	1797	WS01011_P21
5	56.008	101	1	-0.189	0.051	3.865	1797	WS01011_P21
7	75.883	144	2	-0.465	0.073	5.981	1797	WS01011_P21
10	78.479	193	4	0.339	0.070	6.953	1797	WS01011_P21
10	93.347	196	1	0.465	0.098	5.336	1797	WS01011_P21
1	77.177	15	2	0.256	0.065	6.438	1798	WS01012_C03
1	133.992	24	2	0.220	0.040	4.002	1798	WS01012_C03
1	141.021	25	2	-0.241	0.050	4.987	1798	WS01012_C03
2	48.839	29	1	-0.263	0.055	4.954	1798	WS01012_C03
3	104.681	60	2	-0.217	0.039	3.921	1798	WS01012_C03
3	114.747	62	2	0.180	0.041	4.088	1798	WS01012_C03
4	0.000	71	4	-0.165	0.041	4.146	1798	WS01012_C03
5	57.017	102	2	-0.211	0.042	4.213	1798	WS01012_C03
6	49.100	118	2	0.377	0.160	6.347	1798	WS01012_C03
7	15.555	130	3	0.409	0.137	11.801	1798	WS01012_C03
9	0.000	166	1	0.201	0.046	4.122	1798	WS01012_C03
10	21.864	188	3	-0.237	0.046	3.995	1798	WS01012_C03
12	38.543	232	4	-0.264	0.107	4.443	1798	WS01012_C03
14	22.402	248	4	-0.404	0.077	7.756	1798	WS01012_C03
1	95.572	17	3	0.271	0.055	4.999	1799	WS01012_C16
4	124.132	83	1	0.462	0.087	5.164	1799	WS01012_C16
6	49.100	118	4	0.353	0.086	4.352	1799	WS01012_C16
13	0.000	243	4	0.334	0.067	4.019	1799	WS01012_C16

1	29.800	5	1	0.300	0.080	5.064	1800	WS01012_E22
1	38.326	6	4	-0.181	0.038	4.369	1800	WS01012_E22
5	3.484	90	2	0.246	0.050	3.937	1800	WS01012_E22
6	26.350	115	2	-0.156	0.032	3.968	1800	WS01012_E22
9	17.951	169	2	0.295	0.054	6.565	1800	WS01012_E22
10	35.204	189	1	0.207	0.065	4.132	1800	WS01012_E22
1	40.464	7	4	0.233	0.040	4.080	1801	WS01012_F09
2	48.839	29	4	-0.204	0.040	4.051	1801	WS01012_F09
3	95.157	59	2	0.175	0.043	4.398	1801	WS01012_F09
5	0.000	89	3	0.264	0.047	3.860	1801	WS01012_F09
7	15.555	130	2	0.279	0.073	6.944	1801	WS01012_F09
8	62.580	155	1	0.372	0.116	5.079	1801	WS01012_F09
10	78.479	193	3	-0.338	0.074	6.258	1801	WS01012_F09
11	46.026	217	1	-0.240	0.062	5.736	1801	WS01012_F09
1	38.326	6	4	-0.318	0.065	6.503	1802	WS02611_M07
1	40.464	7	4	0.371	0.077	7.198	1802	WS02611_M07
1	95.572	17	3	0.270	0.062	3.986	1802	WS02611_M07
7	15.555	130	2	0.302	0.071	6.517	1802	WS02611_M07
8	62.580	155	1	0.348	0.115	5.387	1802	WS02611_M07
8	78.389	157	1	0.217	0.045	4.341	1802	WS02611_M07
9	3.127	168	1	-0.346	0.096	6.482	1802	WS02611_M07
10	78.479	193	3	-0.327	0.073	6.357	1802	WS02611_M07
3	14.500	43	1	-0.224	0.041	3.885	1803	WS02612_K24
3	17.158	45	1	0.226	0.064	6.040	1803	WS02612_K24
3	116.958	63	4	0.128	0.040	3.899	1803	WS02612_K24
4	6.032	72	4	0.223	0.053	5.175	1803	WS02612_K24
5	52.820	99	3	-0.266	0.071	6.509	1803	WS02612_K24
9	3.127	168	1	-0.388	0.090	4.611	1803	WS02612_K24
10	9.580	187	3	-0.304	0.093	5.309	1803	WS02612_K24
11	0.000	199	2	-0.275	0.044	4.329	1803	WS02612_K24
14	0.000	246	1	-0.265	0.045	4.301	1803	WS02612_K24
1	45.794	9	1	0.333	0.041	4.331	1804	WS0261_P07
3	141.443	65	4	0.297	0.107	6.045	1804	WS0261_P07
7	75.883	144	2	0.522	0.138	7.516	1804	WS0261_P07
7	75.883	144	4	-0.358	0.068	7.130	1804	WS0261_P07
10	93.347	196	1	0.415	0.089	4.141	1804	WS0261_P07
11	46.026	217	1	-0.335	0.076	7.841	1804	WS0261_P07
1	38.326	6	4	-0.299	0.049	5.333	1805	WS01028_P02
1	40.464	7	4	0.290	0.043	4.502	1805	WS01028_P02
1	45.794	9	1	0.268	0.036	3.984	1805	WS01028_P02
7	73.169	142	1	0.580	0.115	6.253	1805	WS01028_P02
9	3.127	168	1	-0.324	0.081	6.622	1805	WS01028_P02
14	0.000	246	1	-0.226	0.046	5.080	1805	WS01028_P02
3	95.157	59	2	0.184	0.038	4.262	1806	WS0091_D06
9	0.000	163	1	-0.429	0.114	4.055	1806	WS0091_D06
9	55.358	175	1	0.303	0.045	3.926	1806	WS0091_D06
9	81.073	183	1	-0.254	0.073	6.072	1806	WS0091_D06
11	40.960	210	2	-0.454	0.093	6.139	1806	WS0091_D06
1	45.794	9	1	0.371	0.047	5.518	1807	WS00928_C16
1	45.794	9	3	-0.230	0.050	4.427	1807	WS00928_C16
1	95.572	17	3	0.210	0.048	3.870	1807	WS00928_C16
7	73.169	142	1	0.461	0.071	4.200	1807	WS00928_C16
8	62.580	155	1	0.184	0.066	3.856	1807	WS00928_C16
9	3.127	168	1	-0.558	0.109	10.090	1807	WS00928_C16

10	21.864	188	1	0.345	0.041	4.769	1807	WS00928_C16
10	93.347	196	1	0.454	0.089	4.950	1807	WS00928_C16
11	46.026	217	1	-0.285	0.046	5.346	1807	WS00928_C16
1	95.572	17	2	0.319	0.042	4.791	1808	WS00927_N09
1	133.992	24	2	0.204	0.045	5.081	1808	WS00927_N09
1	141.021	25	2	-0.220	0.053	5.959	1808	WS00927_N09
2	75.550	31	1	0.284	0.050	4.017	1808	WS00927_N09
3	17.158	45	1	0.215	0.065	4.953	1808	WS00927_N09
6	89.401	125	1	-0.299	0.054	4.394	1808	WS00927_N09
9	0.000	163	1	-0.496	0.130	5.404	1808	WS00927_N09
9	28.608	170	3	0.190	0.036	4.372	1808	WS00927_N09
10	78.479	193	3	-0.395	0.037	4.103	1808	WS00927_N09
10	93.347	196	1	0.445	0.114	4.750	1808	WS00927_N09
2	48.839	29	4	-0.344	0.079	6.278	1809	WS00927_N16
3	14.500	43	1	-0.289	0.053	4.439	1809	WS00927_N16
3	116.958	63	4	0.165	0.058	4.850	1809	WS00927_N16
7	59.789	137	3	-0.233	0.040	4.188	1809	WS00927_N16
7	70.278	141	3	0.254	0.049	5.148	1809	WS00927_N16
9	0.000	166	2	-0.256	0.045	4.935	1809	WS00927_N16
9	17.951	169	2	0.291	0.039	4.290	1809	WS00927_N16
11	46.026	217	1	-0.251	0.049	4.041	1809	WS00927_N16
14	0.000	246	1	-0.266	0.053	4.443	1809	WS00927_N16
1	40.464	7	4	0.312	0.042	3.888	1810	WS00927_O19
1	95.572	17	3	0.263	0.052	4.773	1810	WS00927_O19
5	0.000	89	3	0.217	0.048	4.540	1810	WS00927_O19
7	15.555	130	2	0.222	0.052	4.258	1810	WS00927_O19
10	9.580	187	3	-0.269	0.072	4.508	1810	WS00927_O19
10	78.479	193	3	-0.334	0.047	4.433	1810	WS00927_O19
10	93.347	196	1	0.498	0.097	5.984	1810	WS00927_O19
11	46.026	217	1	-0.275	0.046	5.084	1810	WS00927_O19
1	38.326	6	4	-0.333	0.063	4.837	1811	WS00927_P12
1	40.464	7	4	0.340	0.066	5.423	1811	WS00927_P12
3	9.953	42	3	-0.209	0.060	6.305	1811	WS00927_P12
3	17.158	45	1	0.193	0.044	5.447	1811	WS00927_P12
5	0.000	89	3	0.235	0.047	4.141	1811	WS00927_P12
7	15.555	130	2	0.286	0.082	5.492	1811	WS00927_P12
9	3.127	168	1	-0.317	0.070	4.831	1811	WS00927_P12
9	58.045	176	1	0.298	0.051	4.291	1811	WS00927_P12
14	0.000	246	1	-0.304	0.051	6.359	1811	WS00927_P12
4	55.925	76	1	0.217	0.057	6.786	1812	M19
11	46.026	217	1	-0.293	0.091	9.779	1812	M19
12	22.441	227	1	0.263	0.036	4.043	1812	M19
1	29.800	5	1	0.196	0.051	5.204	1813	Pa2420
1	131.686	23	1	-0.532	0.094	4.811	1813	Pa2420
3	83.563	56	3	-0.259	0.080	7.565	1813	Pa2420
9	0.000	163	1	-0.616	0.163	8.361	1813	Pa2420
9	17.951	169	1	0.193	0.045	4.638	1813	Pa2420
12	32.473	238	1	0.231	0.063	6.396	1813	Pa2420
14	43.392	250	2	0.252	0.097	8.151	1813	Pa2420
14	46.637	252	4	0.469	0.129	13.603	1813	Pa2420
3	14.500	43	1	0.281	0.037	3.959	1814	JF71
3	17.158	45	1	-0.239	0.067	7.120	1814	JF71
3	141.443	65	3	0.291	0.045	3.894	1814	JF71
6	26.350	115	2	-0.175	0.060	4.885	1814	JF71

9	2.011	167	4	0.238	0.077	4.843	1814	JF71
14	22.402	248	4	0.331	0.092	5.825	1814	JF71
1	95.572	17	3	0.227	0.051	4.131	1815	WS0092_D11
2	48.839	29	4	-0.279	0.060	5.886	1815	WS0092_D11
6	106.702	128	2	0.396	0.107	3.908	1815	WS0092_D11
7	15.555	130	2	0.235	0.043	4.124	1815	WS0092_D11
7	74.560	143	1	-0.257	0.059	5.461	1815	WS0092_D11
9	0.000	163	1	-0.425	0.080	3.992	1815	WS0092_D11
10	78.479	193	3	-0.306	0.054	4.741	1815	WS0092_D11
1	95.572	17	3	0.331	0.075	5.890	1816	WS0092_H19
3	9.953	42	3	-0.227	0.051	4.708	1816	WS0092_H19
3	95.157	59	2	0.201	0.038	4.138	1816	WS0092_H19
3	104.681	60	2	-0.200	0.049	4.957	1816	WS0092_H19
5	86.669	106	4	-0.367	0.160	4.418	1816	WS0092_H19
6	49.100	118	2	0.250	0.121	3.887	1816	WS0092_H19
6	56.188	120	2	0.262	0.043	4.421	1816	WS0092_H19
7	15.555	130	2	0.332	0.064	6.577	1816	WS0092_H19
10	21.864	188	1	0.306	0.065	5.723	1816	WS0092_H19
11	35.604	207	3	-0.255	0.060	5.506	1816	WS0092_H19
2	126.181	39	4	-0.372	0.061	3.864	1817	WS01013_B11
5	84.347	105	2	-0.212	0.039	4.399	1817	WS01013_B11
6	25.914	114	1	-0.287	0.064	5.166	1817	WS01013_B11
6	26.350	115	2	-0.157	0.037	3.991	1817	WS01013_B11
8	50.001	154	1	0.259	0.046	4.605	1817	WS01013_B11
9	17.951	169	2	0.242	0.037	3.995	1817	WS01013_B11
10	70.246	190	2	0.198	0.039	4.210	1817	WS01013_B11
1	45.794	9	3	-0.228	0.050	4.009	1818	WS01038_K19
1	69.854	13	2	-0.180	0.044	4.925	1818	WS01038_K19
1	71.592	14	2	-0.157	0.035	3.911	1818	WS01038_K19
1	77.177	15	2	-0.171	0.040	4.499	1818	WS01038_K19
2	120.617	38	3	0.188	0.055	4.802	1818	WS01038_K19
2	127.147	40	3	-0.157	0.049	4.201	1818	WS01038_K19
3	104.681	60	2	-0.214	0.036	4.127	1818	WS01038_K19
3	112.868	61	2	0.199	0.037	4.228	1818	WS01038_K19
3	114.747	62	2	0.205	0.043	4.808	1818	WS01038_K19
6	26.350	115	2	-0.206	0.051	5.720	1818	WS01038_K19
6	49.100	118	2	0.268	0.082	3.982	1818	WS01038_K19
7	75.883	144	2	0.429	0.076	5.285	1818	WS01038_K19
9	0.000	163	1	-0.428	0.104	4.034	1818	WS01038_K19
9	17.951	169	2	0.257	0.043	5.414	1818	WS01038_K19
9	59.963	177	4	-0.311	0.077	6.096	1818	WS01038_K19
10	78.479	193	4	-0.272	0.061	6.039	1818	WS01038_K19
10	93.347	196	1	0.447	0.124	4.795	1818	WS01038_K19
11	56.921	219	2	-0.263	0.073	3.937	1818	WS01038_K19
2	48.839	29	4	-0.224	0.055	4.518	1819	WS01013_G19
2	120.617	38	3	0.255	0.047	5.025	1819	WS01013_G19
6	26.350	115	2	-0.197	0.036	4.268	1819	WS01013_G19
6	95.249	126	1	0.326	0.099	4.649	1819	WS01013_G19
10	78.479	193	3	-0.334	0.052	5.563	1819	WS01013_G19
10	104.214	198	4	-0.309	0.053	6.554	1819	WS01013_G19
11	46.026	217	1	-0.255	0.074	5.213	1819	WS01013_G19
14	44.898	251	3	0.247	0.044	4.700	1819	WS01013_G19
1	40.464	7	3	0.183	0.044	4.462	1820	WS0269_D24
1	45.794	9	3	-0.262	0.074	8.100	1820	WS0269_D24

1	95.572	17	3	0.253	0.067	5.516	1820	WS0269_D24
2	120.617	38	3	0.185	0.042	4.262	1820	WS0269_D24
2	127.147	40	3	-0.198	0.047	4.776	1820	WS0269_D24
3	47.928	49	1	0.201	0.042	4.314	1820	WS0269_D24
6	25.914	114	1	-0.292	0.073	7.468	1820	WS0269_D24
6	26.350	115	2	-0.232	0.061	5.200	1820	WS0269_D24
6	56.188	120	2	0.243	0.048	4.203	1820	WS0269_D24
7	75.883	144	2	0.437	0.117	5.219	1820	WS0269_D24
7	75.883	144	4	-0.359	0.065	5.835	1820	WS0269_D24
9	59.963	177	4	-0.223	0.065	4.497	1820	WS0269_D24
9	81.073	183	1	-0.207	0.044	4.503	1820	WS0269_D24
10	93.347	196	1	0.545	0.131	7.263	1820	WS0269_D24
11	46.026	217	1	-0.254	0.051	5.182	1820	WS0269_D24
12	6.242	234	2	0.233	0.059	5.154	1820	WS0269_D24
3	47.928	49	1	0.343	0.129	11.868	1821	WS00712_F06
4	0.000	71	4	-0.300	0.058	7.029	1821	WS00712_F06
6	87.891	124	2	0.185	0.047	4.464	1821	WS00712_F06
6	106.702	128	2	-0.430	0.119	4.898	1821	WS00712_F06
9	0.000	166	1	0.228	0.062	5.705	1821	WS00712_F06
12	14.406	230	4	-0.388	0.081	9.770	1821	WS00712_F06
12	38.543	232	4	0.385	0.118	7.175	1821	WS00712_F06
2	126.181	39	4	-0.392	0.070	3.982	1822	WS00712_L15
4	116.928	82	2	-0.219	0.034	4.431	1822	WS00712_L15
5	7.672	92	4	0.271	0.063	4.802	1822	WS00712_L15
6	81.996	123	3	-0.350	0.138	4.710	1822	WS00712_L15
7	17.610	131	3	0.313	0.067	4.625	1822	WS00712_L15
12	28.493	228	2	0.285	0.066	3.949	1822	WS00712_L15
3	47.928	49	1	0.199	0.046	4.703	1823	WS0094_O23
10	86.151	195	3	0.168	0.051	4.875	1823	WS0094_O23
1	71.592	14	2	-0.203	0.038	4.364	1824	WS0057_H23
1	77.177	15	2	-0.232	0.050	5.751	1824	WS0057_H23
1	82.301	16	4	-0.246	0.045	4.572	1824	WS0057_H23
1	95.572	17	2	-0.260	0.050	5.664	1824	WS0057_H23
3	47.928	49	1	0.264	0.093	6.861	1824	WS0057_H23
3	144.593	66	4	-0.222	0.069	6.930	1824	WS0057_H23
3	154.505	69	4	-0.282	0.073	7.938	1824	WS0057_H23
4	75.958	77	3	-0.379	0.117	6.359	1824	WS0057_H23
4	82.462	78	2	-0.229	0.045	5.562	1824	WS0057_H23
4	97.576	80	2	0.203	0.047	5.676	1824	WS0057_H23
9	59.963	177	4	-0.238	0.042	4.458	1824	WS0057_H23
9	81.073	183	1	-0.251	0.077	5.730	1824	WS0057_H23
11	29.069	205	1	0.245	0.052	3.919	1824	WS0057_H23
3	144.593	66	4	-0.181	0.036	3.890	1825	WS01017_D04
3	154.505	69	4	-0.234	0.051	4.622	1825	WS01017_D04
11	35.604	207	3	-0.185	0.045	3.940	1825	WS01017_D04
3	137.891	64	3	0.219	0.047	3.876	1826	WS0058_C05
4	75.958	77	3	-0.381	0.121	6.314	1826	WS0058_C05
4	124.132	83	1	0.591	0.196	7.074	1826	WS0058_C05
4	142.917	85	1	-0.320	0.083	6.958	1826	WS0058_C05
9	49.013	173	2	0.234	0.037	3.982	1826	WS0058_C05
9	81.073	183	1	-0.205	0.053	4.451	1826	WS0058_C05
14	0.000	246	1	0.179	0.051	4.267	1826	WS0058_C05
3	14.500	43	2	0.192	0.037	4.627	1827	WS00816_M06
3	47.928	49	1	0.227	0.075	4.506	1827	WS00816_M06

4	116.928	82	2	0.267	0.046	5.712	1827	WS00816_M06
4	142.917	85	1	-0.234	0.067	4.213	1827	WS00816_M06
5	0.000	89	3	0.291	0.040	4.840	1827	WS00816_M06
6	106.702	128	2	0.525	0.074	7.891	1827	WS00816_M06
11	43.491	214	3	0.236	0.050	6.372	1827	WS00816_M06
12	22.441	227	3	-0.283	0.043	5.441	1827	WS00816_M06
12	14.406	230	4	-0.264	0.070	4.072	1827	WS00816_M06
12	6.242	234	2	0.276	0.053	6.953	1827	WS00816_M06
13	1.815	244	2	0.254	0.046	5.883	1827	WS00816_M06
1	127.805	21	3	-0.162	0.031	4.231	1828	WS00816_N11
3	9.953	42	3	0.167	0.032	4.447	1828	WS00816_N11
9	0.000	166	2	0.222	0.052	6.008	1828	WS00816_N11
10	78.479	193	3	-0.288	0.041	5.717	1828	WS00816_N11
14	44.898	251	3	0.229	0.032	4.472	1828	WS00816_N11
1	45.794	9	1	-0.294	0.050	3.844	1829	WS00817_A02
1	45.794	9	3	0.313	0.061	6.825	1829	WS00817_A02
4	43.146	74	3	-0.218	0.054	5.771	1829	WS00817_A02
10	104.214	198	2	-0.240	0.031	4.994	1829	WS00817_A02
11	42.600	213	3	0.188	0.037	3.935	1829	WS00817_A02
12	6.242	234	2	0.309	0.060	6.375	1829	WS00817_A02
14	0.000	246	1	0.269	0.053	4.115	1829	WS00817_A02
3	47.928	49	1	0.230	0.072	7.889	1830	WS00817_B07
3	154.505	69	1	0.270	0.050	5.545	1830	WS00817_B07
4	55.925	76	1	-0.238	0.072	7.950	1830	WS00817_B07
4	75.958	77	3	-0.372	0.077	4.150	1830	WS00817_B07
4	82.462	78	1	-0.336	0.066	7.409	1830	WS00817_B07
5	86.669	106	4	0.248	0.127	5.846	1830	WS00817_B07
6	95.249	126	1	0.251	0.064	4.318	1830	WS00817_B07
10	78.479	193	3	-0.503	0.074	9.134	1830	WS00817_B07
10	86.151	195	3	0.230	0.069	8.215	1830	WS00817_B07
12	22.441	227	1	-0.235	0.051	5.607	1830	WS00817_B07
2	95.880	34	3	-0.275	0.060	5.715	1831	WS00817_D12
2	101.076	35	3	0.284	0.064	6.134	1831	WS00817_D12
5	54.072	100	4	0.212	0.046	4.274	1831	WS00817_D12
5	84.347	105	1	-0.254	0.061	5.622	1831	WS00817_D12
5	84.347	105	2	0.195	0.048	4.555	1831	WS00817_D12
10	35.204	189	1	0.214	0.044	4.065	1831	WS00817_D12
10	95.795	197	4	-0.239	0.055	5.100	1831	WS00817_D12
11	43.491	214	3	-0.198	0.042	4.020	1831	WS00817_D12
11	44.884	215	3	0.228	0.052	4.935	1831	WS00817_D12
11	64.014	222	3	0.222	0.046	4.376	1831	WS00817_D12
12	22.441	227	1	0.245	0.044	4.055	1831	WS00817_D12
14	0.000	246	1	0.218	0.043	3.910	1831	WS00817_D12
14	43.392	250	1	-0.299	0.087	4.842	1831	WS00817_D12
14	46.637	252	3	-0.225	0.041	3.912	1831	WS00817_D12
6	95.249	126	1	0.348	0.144	5.464	1832	WS00817_E08
10	78.479	193	3	-0.389	0.074	8.917	1832	WS00817_E08
10	86.151	195	3	0.194	0.041	4.867	1832	WS00817_E08
3	137.891	64	1	-0.347	0.083	7.621	1833	WS00817_H16
3	144.593	66	4	0.203	0.076	5.101	1833	WS00817_H16
3	148.745	68	1	0.442	0.146	13.340	1833	WS00817_H16
3	148.745	68	4	-0.255	0.100	7.545	1833	WS00817_H16
3	154.505	69	1	0.434	0.144	12.878	1833	WS00817_H16
4	55.925	76	1	-0.247	0.059	5.380	1833	WS00817_H16

4	82.462	78	1	-0.329	0.102	9.338	1833	WS00817_H16
6	87.891	124	2	0.187	0.042	4.050	1833	WS00817_H16
6	89.401	125	2	0.274	0.044	4.098	1833	WS00817_H16
11	43.491	214	4	0.200	0.070	4.477	1833	WS00817_H16
11	60.464	221	1	-0.286	0.044	4.009	1833	WS00817_H16
12	0.000	233	1	0.230	0.048	4.377	1833	WS00817_H16
13	3.386	245	1	0.264	0.066	6.010	1833	WS00817_H16
1	43.578	8	3	0.267	0.047	5.693	1834	WS00817_I19
3	95.157	59	2	-0.182	0.042	4.390	1834	WS00817_I19
5	0.000	89	3	0.344	0.041	5.296	1834	WS00817_I19
6	95.249	126	1	0.354	0.092	6.294	1834	WS00817_I19
10	78.479	193	3	-0.307	0.049	6.696	1834	WS00817_I19
10	86.151	195	3	0.209	0.039	5.350	1834	WS00817_I19
2	102.814	36	2	-0.253	0.052	5.667	1835	WS00946_G15
2	126.181	39	2	0.233	0.048	4.836	1835	WS00946_G15
3	14.500	43	1	-0.483	0.128	11.447	1835	WS00946_G15
6	69.073	121	3	-0.253	0.044	4.138	1835	WS00946_G15
7	15.555	130	2	0.337	0.043	4.955	1835	WS00946_G15
7	70.278	141	3	0.267	0.056	5.386	1835	WS00946_G15
9	28.608	170	3	-0.398	0.115	10.488	1835	WS00946_G15
9	69.046	181	2	-0.319	0.117	6.378	1835	WS00946_G15
10	77.676	192	1	0.235	0.067	5.924	1835	WS00946_G15
10	80.088	194	1	0.201	0.062	5.470	1835	WS00946_G15
10	93.347	196	1	-0.561	0.157	7.554	1835	WS00946_G15
11	43.491	214	3	0.260	0.046	4.365	1835	WS00946_G15
11	64.014	222	3	-0.389	0.079	7.705	1835	WS00946_G15
13	3.386	245	1	0.247	0.078	6.850	1835	WS00946_G15
14	22.402	248	4	0.315	0.036	4.461	1835	WS00946_G15
14	46.637	252	4	0.332	0.043	4.619	1835	WS00946_G15
1	2.788	2	4	-0.301	0.084	7.450	1836	WS00817_N22
2	48.839	29	4	0.226	0.059	5.014	1836	WS00817_N22
2	127.147	40	3	0.242	0.059	6.131	1836	WS00817_N22
3	148.745	68	1	-0.251	0.079	5.293	1836	WS00817_N22
9	32.356	171	2	0.213	0.048	5.830	1836	WS00817_N22
9	49.013	173	2	-0.229	0.041	4.522	1836	WS00817_N22
10	80.088	194	2	-0.257	0.036	4.190	1836	WS00817_N22
14	22.402	248	4	0.304	0.069	4.471	1836	WS00817_N22
3	144.647	67	1	0.257	0.096	4.118	1837	WS00945_A23
8	50.001	154	2	-0.219	0.039	4.236	1837	WS00945_A23
11	27.405	204	1	0.334	0.074	6.187	1837	WS00945_A23
13	3.386	245	1	-0.274	0.071	5.875	1837	WS00945_A23
4	55.925	76	1	-0.239	0.063	6.249	1838	WS00818_E06
4	75.958	77	3	-0.314	0.103	3.967	1838	WS00818_E06
4	82.462	78	1	-0.261	0.052	4.976	1838	WS00818_E06
5	31.191	95	1	-0.177	0.041	3.890	1838	WS00818_E06
5	57.017	102	4	-0.336	0.065	6.393	1838	WS00818_E06
5	84.347	105	1	-0.254	0.048	4.599	1838	WS00818_E06
5	86.669	106	4	0.299	0.100	5.081	1838	WS00818_E06
6	49.100	118	4	-0.289	0.094	4.766	1838	WS00818_E06
6	54.066	119	4	-0.229	0.038	4.003	1838	WS00818_E06
6	95.249	126	1	0.318	0.134	5.885	1838	WS00818_E06
7	105.477	147	2	-0.291	0.081	4.338	1838	WS00818_E06
8	87.463	158	2	-0.274	0.098	9.110	1838	WS00818_E06
12	28.493	228	2	-0.303	0.086	4.630	1838	WS00818_E06

3	15.147	44	2	-0.321	0.092	5.289	1839	WS00922_K07
3	144.593	66	4	-0.163	0.037	4.194	1839	WS00922_K07
4	173.929	88	2	0.258	0.054	6.282	1839	WS00922_K07
9	28.608	170	3	0.314	0.053	4.945	1839	WS00922_K07
9	49.013	173	1	0.283	0.069	5.830	1839	WS00922_K07
9	49.013	173	2	-0.263	0.047	5.535	1839	WS00922_K07
10	70.246	190	2	0.233	0.053	6.312	1839	WS00922_K07
10	70.246	190	4	-0.357	0.058	5.775	1839	WS00922_K07
10	78.479	193	4	-0.266	0.060	5.896	1839	WS00922_K07
13	0.000	243	4	0.466	0.179	6.202	1839	WS00922_K07
1	15.589	3	2	0.223	0.041	4.638	1840	WS00922_N09
6	89.401	125	1	-0.293	0.066	4.979	1840	WS00922_N09
9	59.963	177	4	-0.403	0.119	9.646	1840	WS00922_N09
1	82.301	16	3	-0.243	0.051	5.896	1841	WS00921_J07
6	69.073	121	3	-0.246	0.035	3.998	1841	WS00921_J07
6	89.401	125	1	-0.349	0.066	6.262	1841	WS00921_J07
6	95.249	126	1	0.345	0.060	3.952	1841	WS00921_J07
7	17.610	131	3	-0.231	0.076	3.888	1841	WS00921_J07
9	0.000	163	1	-0.558	0.108	6.901	1841	WS00921_J07
9	59.963	177	4	-0.452	0.143	7.812	1841	WS00921_J07
10	9.580	187	3	-0.507	0.133	9.120	1841	WS00921_J07
10	78.479	193	3	-0.359	0.046	5.367	1841	WS00921_J07
11	17.088	203	1	-0.260	0.044	4.107	1841	WS00921_J07
11	17.088	203	2	-0.171	0.043	4.191	1841	WS00921_J07
11	43.491	214	3	0.169	0.057	6.487	1841	WS00921_J07
11	44.884	215	3	-0.163	0.043	4.902	1841	WS00921_J07
11	64.014	222	3	-0.267	0.089	10.203	1841	WS00921_J07
2	127.147	40	3	-0.218	0.054	4.041	1842	WS00921_J17
3	112.868	61	2	0.171	0.037	3.999	1842	WS00921_J17
4	173.929	88	2	-0.182	0.054	5.743	1842	WS00921_J17
5	16.219	93	2	-0.219	0.034	4.017	1842	WS00921_J17
6	76.376	122	1	0.389	0.154	7.972	1842	WS00921_J17
6	89.401	125	1	-0.296	0.088	7.068	1842	WS00921_J17
6	106.702	128	2	-0.418	0.093	4.321	1842	WS00921_J17
9	36.227	165	4	0.192	0.039	4.559	1842	WS00921_J17
9	61.113	178	1	-0.445	0.109	5.707	1842	WS00921_J17
12	28.493	228	2	-0.252	0.068	4.214	1842	WS00921_J17
13	1.815	244	2	-0.315	0.044	4.630	1842	WS00921_J17
13	1.815	244	4	0.257	0.066	7.885	1842	WS00921_J17
1	15.589	3	2	-0.218	0.045	4.804	1843	WS00921_L06
1	50.955	10	1	-0.234	0.048	3.951	1843	WS00921_L06
4	103.719	81	4	0.212	0.058	5.625	1843	WS00921_L06
5	7.672	92	4	0.274	0.056	5.038	1843	WS00921_L06
5	84.347	105	2	0.244	0.057	5.468	1843	WS00921_L06
12	0.000	226	3	-0.240	0.075	7.311	1843	WS00921_L06
12	22.441	227	3	0.267	0.048	4.394	1843	WS00921_L06
14	22.402	248	4	-0.254	0.047	3.892	1843	WS00921_L06
1	15.589	3	2	0.310	0.089	10.302	1844	WS00921_L21
1	29.800	5	2	-0.349	0.082	9.719	1844	WS00921_L21
1	38.326	6	2	0.385	0.105	12.220	1844	WS00921_L21
1	43.578	8	2	-0.317	0.084	9.877	1844	WS00921_L21
3	80.211	55	4	0.212	0.053	4.409	1844	WS00921_L21
5	31.191	95	1	-0.235	0.055	4.011	1844	WS00921_L21
6	25.914	114	1	-0.259	0.064	4.714	1844	WS00921_L21

8	62.580	155	1	0.427	0.236	5.811	1844	WS00921_L21
8	78.389	157	1	0.223	0.077	5.582	1844	WS00921_L21
8	87.463	158	1	-0.189	0.058	4.214	1844	WS00921_L21
9	61.113	178	1	0.345	0.200	4.919	1844	WS00921_L21
9	69.046	181	2	-0.333	0.111	5.567	1844	WS00921_L21
9	81.073	183	1	0.295	0.058	4.188	1844	WS00921_L21
12	32.473	238	1	0.186	0.055	3.965	1844	WS00921_L21
2	120.617	38	3	0.231	0.043	4.424	1845	WS00921_N19
4	55.925	76	1	-0.237	0.047	4.221	1845	WS00921_N19
4	97.576	80	2	0.233	0.057	6.926	1845	WS00921_N19
6	89.401	125	1	-0.301	0.061	5.396	1845	WS00921_N19
6	95.249	126	1	0.366	0.126	6.366	1845	WS00921_N19
8	11.974	150	1	-0.316	0.083	7.366	1845	WS00921_N19
8	62.580	155	1	0.317	0.112	4.267	1845	WS00921_N19
9	59.963	177	4	-0.292	0.066	5.606	1845	WS00921_N19
10	78.479	193	3	-0.292	0.038	3.872	1845	WS00921_N19
11	17.088	203	2	-0.217	0.049	4.673	1845	WS00921_N19
11	35.604	207	3	-0.184	0.051	5.194	1845	WS00921_N19
12	14.406	230	4	-0.288	0.068	5.126	1845	WS00921_N19
1	45.794	9	1	0.267	0.056	4.662	1846	WS00921_O07
1	50.955	10	1	-0.216	0.055	4.576	1846	WS00921_O07
1	82.301	16	4	-0.265	0.050	4.511	1846	WS00921_O07
5	52.820	99	1	0.411	0.142	12.585	1846	WS00921_O07
5	54.072	100	4	0.240	0.055	4.787	1846	WS00921_O07
5	56.008	101	1	-0.283	0.080	6.707	1846	WS00921_O07
5	57.017	102	4	-0.221	0.051	4.588	1846	WS00921_O07
5	84.347	105	1	-0.270	0.081	6.796	1846	WS00921_O07
6	89.401	125	2	-0.258	0.041	4.217	1846	WS00921_O07
7	17.610	131	3	0.314	0.087	3.954	1846	WS00921_O07
12	0.000	233	1	-0.231	0.052	4.388	1846	WS00921_O07
12	22.629	237	1	-0.226	0.047	3.962	1846	WS00921_O07
1	82.301	16	4	-0.223	0.058	4.300	1847	WS00921_P18
1	127.805	21	3	-0.268	0.062	6.116	1847	WS00921_P18
3	47.928	49	1	0.209	0.062	4.852	1847	WS00921_P18
3	144.647	67	1	0.321	0.123	3.987	1847	WS00921_P18
7	59.789	137	3	-0.278	0.079	7.821	1847	WS00921_P18
7	70.278	141	3	0.248	0.066	6.457	1847	WS00921_P18
8	11.974	150	1	-0.216	0.058	4.495	1847	WS00921_P18
8	95.937	159	3	0.181	0.041	4.045	1847	WS00921_P18
11	17.088	203	2	-0.248	0.047	4.625	1847	WS00921_P18
11	35.604	207	3	-0.265	0.067	6.646	1847	WS00921_P18
11	37.143	209	3	0.209	0.041	4.029	1847	WS00921_P18
11	42.600	213	3	-0.216	0.041	4.006	1847	WS00921_P18
11	64.014	222	3	-0.232	0.040	3.959	1847	WS00921_P18
14	22.402	248	4	-0.324	0.072	6.040	1847	WS00921_P18
14	43.392	250	2	0.217	0.039	4.318	1847	WS00921_P18
1	69.854	13	2	0.222	0.032	3.844	1848	WS00922_A10
1	95.572	17	2	0.246	0.031	4.000	1848	WS00922_A10
5	31.191	95	1	-0.302	0.060	4.375	1848	WS00922_A10
6	49.100	118	4	-0.359	0.183	5.554	1848	WS00922_A10
6	87.891	124	2	-0.138	0.035	4.139	1848	WS00922_A10
6	95.249	126	1	0.453	0.118	5.049	1848	WS00922_A10
11	27.405	204	1	0.464	0.109	8.273	1848	WS00922_A10
11	29.069	205	1	0.302	0.061	4.434	1848	WS00922_A10

12	22.441	227	3	-0.245	0.052	4.723	1848	WS00922_A10
1	2.788	2	4	-0.230	0.044	5.043	1849	WS00815_H10
1	123.316	20	4	-0.223	0.051	4.071	1849	WS00815_H10
4	82.462	78	2	0.187	0.044	6.096	1849	WS00815_H10
6	26.350	115	2	-0.241	0.052	6.761	1849	WS00815_H10
7	62.787	139	4	-0.188	0.033	4.171	1849	WS00815_H10
7	73.169	142	2	-0.408	0.050	3.955	1849	WS00815_H10
7	75.883	144	2	0.436	0.060	4.940	1849	WS00815_H10
10	78.479	193	4	-0.203	0.038	4.262	1849	WS00815_H10
10	95.795	197	4	0.219	0.059	6.469	1849	WS00815_H10
14	44.898	251	3	0.254	0.066	4.747	1849	WS00815_H10
1	82.301	16	3	-0.208	0.039	4.987	1850	WS00815_N12
1	95.572	17	3	0.245	0.039	4.481	1850	WS00815_N12
9	36.227	165	3	0.177	0.035	4.505	1850	WS00815_N12
9	49.013	173	2	0.224	0.039	5.091	1850	WS00815_N12
10	78.479	193	3	-0.268	0.032	4.129	1850	WS00815_N12
11	60.464	221	2	0.195	0.028	3.970	1850	WS00815_N12
14	22.402	248	3	0.247	0.043	5.688	1850	WS00815_N12
11	27.405	204	1	0.466	0.124	11.161	1851	WS00715_I08
11	29.069	205	1	0.320	0.089	7.945	1851	WS00715_I08
11	45.989	216	1	-0.192	0.066	5.864	1851	WS00715_I08
12	28.493	228	2	-0.268	0.129	5.073	1851	WS00715_I08
1	29.800	5	2	0.236	0.039	4.099	1852	WS00921_C01
1	38.326	6	2	-0.262	0.046	5.222	1852	WS00921_C01
3	154.505	69	4	-0.298	0.052	4.816	1852	WS00921_C01
11	44.884	215	4	-0.294	0.045	4.742	1852	WS00921_C01
12	28.493	228	2	0.250	0.054	3.953	1852	WS00921_C01
1	45.794	9	1	0.243	0.049	5.435	1853	WS00716_A11
4	142.917	85	1	0.218	0.044	4.989	1853	WS00716_A11
7	59.789	137	3	-0.200	0.050	5.956	1853	WS00716_A11
10	9.580	187	3	0.433	0.139	10.805	1853	WS00716_A11
10	21.864	188	1	-0.346	0.062	7.052	1853	WS00716_A11
10	80.088	194	1	0.255	0.043	4.796	1853	WS00716_A11
12	14.406	230	3	-0.195	0.033	3.923	1853	WS00716_A11
12	32.473	238	1	-0.252	0.046	5.048	1853	WS00716_A11
3	14.500	43	1	-0.260	0.047	4.162	1854	WS00921_G13
3	15.147	44	2	0.287	0.087	5.360	1854	WS00921_G13
3	17.158	45	1	0.178	0.045	3.964	1854	WS00921_G13
3	95.157	59	2	-0.189	0.056	6.052	1854	WS00921_G13
4	97.576	80	2	0.148	0.036	3.937	1854	WS00921_G13
4	124.132	83	1	0.467	0.164	4.367	1854	WS00921_G13
4	142.917	85	1	-0.303	0.065	5.706	1854	WS00921_G13
6	87.891	124	2	-0.227	0.043	4.318	1854	WS00921_G13
3	47.928	49	1	0.234	0.039	4.949	1855	WS00815_P10
3	154.505	69	1	0.276	0.040	5.077	1855	WS00815_P10
3	154.505	69	4	-0.240	0.085	6.229	1855	WS00815_P10
4	6.032	72	4	-0.205	0.065	4.611	1855	WS00815_P10
6	95.249	126	1	0.311	0.064	5.980	1855	WS00815_P10
11	27.405	204	1	0.282	0.049	6.279	1855	WS00815_P10
11	43.491	214	3	0.188	0.046	5.287	1855	WS00815_P10
11	44.884	215	3	-0.258	0.053	6.141	1855	WS00815_P10
11	64.014	222	3	-0.205	0.039	4.482	1855	WS00815_P10
2	75.550	31	2	-0.359	0.087	8.190	1856	WS00815_P20
3	9.953	42	3	0.217	0.053	4.689	1856	WS00815_P20

4	82.462	78	1	-0.304	0.090	6.598	1856	WS00815_P20
5	54.072	100	4	0.233	0.051	5.093	1856	WS00815_P20
5	86.669	106	4	0.444	0.202	6.722	1856	WS00815_P20
6	49.100	118	4	-0.393	0.158	5.438	1856	WS00815_P20
6	54.066	119	4	-0.231	0.043	4.278	1856	WS00815_P20
6	106.702	128	2	-0.395	0.071	3.991	1856	WS00815_P20
9	0.000	166	2	0.232	0.049	5.477	1856	WS00815_P20
9	81.073	183	1	0.239	0.051	3.871	1856	WS00815_P20
1	107.333	19	3	0.254	0.067	6.736	1857	WS0061_C21
1	127.805	21	3	0.265	0.070	7.107	1857	WS0061_C21
3	154.505	69	1	0.191	0.043	3.971	1857	WS0061_C21
3	154.505	69	4	-0.374	0.102	8.764	1857	WS0061_C21
5	3.484	90	2	0.300	0.108	5.815	1857	WS0061_C21
9	69.046	181	2	0.316	0.122	3.918	1857	WS0061_C21
10	78.479	193	3	-0.296	0.047	4.718	1857	WS0061_C21
2	63.261	30	3	-0.293	0.090	6.671	1858	WS0061_E09
2	93.504	32	2	0.200	0.038	3.920	1858	WS0061_E09
2	95.880	34	3	-0.224	0.059	4.193	1858	WS0061_E09
3	137.891	64	1	-0.267	0.064	5.583	1858	WS0061_E09
3	144.593	66	4	0.198	0.048	5.847	1858	WS0061_E09
3	154.505	69	4	0.231	0.044	4.094	1858	WS0061_E09
5	86.669	106	4	0.575	0.209	10.385	1858	WS0061_E09
6	106.702	128	2	0.453	0.138	4.947	1858	WS0061_E09
9	55.358	175	1	-0.351	0.076	6.832	1858	WS0061_E09
9	55.358	175	3	0.502	0.147	6.461	1858	WS0061_E09
9	61.113	178	1	0.356	0.145	7.169	1858	WS0061_E09
9	81.073	183	1	0.425	0.122	10.872	1858	WS0061_E09
5	57.017	102	2	0.240	0.046	4.223	1859	WS0061_L11
7	15.555	130	2	0.272	0.059	5.005	1859	WS0061_L11
8	0.000	148	4	0.238	0.041	3.988	1859	WS0061_L11
8	39.090	152	4	0.152	0.043	4.157	1859	WS0061_L11
8	50.001	154	1	0.209	0.065	7.025	1859	WS0061_L11
10	78.479	193	3	-0.445	0.085	7.966	1859	WS0061_L11
12	6.242	234	4	0.148	0.036	4.325	1859	WS0061_L11
12	51.382	242	1	-0.449	0.092	5.540	1859	WS0061_L11
1	107.333	19	3	0.259	0.052	6.369	1860	WS0061_N18
2	102.814	36	1	0.218	0.056	4.864	1860	WS0061_N18
3	148.745	68	1	0.275	0.048	4.113	1860	WS0061_N18
6	95.249	126	1	0.330	0.063	4.401	1860	WS0061_N18
7	74.560	143	1	-0.234	0.056	4.725	1860	WS0061_N18
9	59.963	177	4	-0.321	0.094	5.255	1860	WS0061_N18
10	9.580	187	3	-0.390	0.086	5.673	1860	WS0061_N18
11	35.604	207	3	-0.162	0.034	4.073	1860	WS0061_N18
4	116.928	82	2	0.260	0.044	4.986	1861	WS0062_C04
4	124.132	83	2	0.305	0.088	5.170	1861	WS0062_C04
6	95.249	126	1	0.289	0.083	4.608	1861	WS0062_C04
8	11.974	150	1	-0.241	0.061	4.560	1861	WS0062_C04
11	35.604	207	3	-0.206	0.041	4.822	1861	WS0062_C04
11	43.491	214	3	0.215	0.044	5.235	1861	WS0062_C04
11	44.884	215	3	-0.213	0.037	4.395	1861	WS0062_C04
11	64.014	222	3	-0.414	0.126	15.017	1861	WS0062_C04
3	47.928	49	1	0.214	0.051	4.340	1862	WS0062_D11
3	154.505	69	4	-0.213	0.041	4.294	1862	WS0062_D11
4	124.132	83	1	0.623	0.198	7.847	1862	WS0062_D11

4	140.962	84	3	-0.203	0.064	5.822	1862	WS0062_D11
4	142.917	85	1	-0.274	0.075	6.410	1862	WS0062_D11
7	17.610	131	3	-0.295	0.095	4.241	1862	WS0062_D11
10	78.479	193	4	-0.307	0.075	7.109	1862	WS0062_D11
12	22.441	227	3	-0.190	0.039	3.978	1862	WS0062_D11
1	15.589	3	2	0.245	0.042	4.814	1863	WS0062_K03
1	95.572	17	2	-0.240	0.061	6.945	1863	WS0062_K03
3	47.928	49	1	0.315	0.066	5.117	1863	WS0062_K03
3	80.211	55	4	0.257	0.081	8.018	1863	WS0062_K03
3	144.593	66	4	-0.198	0.055	5.348	1863	WS0062_K03
3	154.505	69	4	-0.195	0.053	5.652	1863	WS0062_K03
4	0.000	71	4	-0.157	0.043	4.211	1863	WS0062_K03
6	69.073	121	4	-0.208	0.049	4.133	1863	WS0062_K03
6	87.891	124	2	-0.270	0.109	12.394	1863	WS0062_K03
6	89.401	125	1	-0.263	0.078	5.772	1863	WS0062_K03
6	89.401	125	2	-0.439	0.123	14.068	1863	WS0062_K03
6	95.249	126	1	0.385	0.099	4.507	1863	WS0062_K03
6	106.702	128	2	0.381	0.088	4.131	1863	WS0062_K03
7	74.560	143	2	0.144	0.026	4.010	1863	WS0062_K03
9	0.000	166	1	-0.157	0.052	3.866	1863	WS0062_K03
9	28.608	170	3	0.327	0.071	5.006	1863	WS0062_K03
9	59.963	177	4	-0.319	0.078	7.309	1863	WS0062_K03
11	27.405	204	1	0.360	0.060	4.656	1863	WS0062_K03
11	44.884	215	4	0.220	0.034	4.237	1863	WS0062_K03
12	6.242	234	4	0.270	0.042	4.532	1863	WS0062_K03
3	47.928	49	1	0.326	0.095	7.697	1864	WS0062_M14
6	25.914	114	1	-0.298	0.070	5.729	1864	WS0062_M14
6	95.249	126	1	0.397	0.105	5.857	1864	WS0062_M14
10	70.246	190	2	0.292	0.051	5.378	1864	WS0062_M14
10	70.246	190	4	-0.303	0.076	6.308	1864	WS0062_M14
10	80.088	194	2	-0.292	0.052	5.629	1864	WS0062_M14
12	22.441	227	3	-0.272	0.058	4.116	1864	WS0062_M14
5	31.191	95	1	-0.294	0.057	4.331	1865	WS0063_B03
6	56.188	120	2	0.273	0.045	5.307	1865	WS0063_B03
6	95.249	126	1	0.368	0.079	4.274	1865	WS0063_B03
9	17.951	169	2	0.213	0.044	4.928	1865	WS0063_B03
9	28.608	170	2	-0.333	0.072	8.693	1865	WS0063_B03
1	45.794	9	3	-0.214	0.037	4.051	1866	WS0063_C08
3	20.512	46	2	-0.207	0.039	4.249	1866	WS0063_C08
3	47.928	49	1	0.240	0.050	4.053	1866	WS0063_C08
6	89.401	125	1	-0.278	0.059	4.709	1866	WS0063_C08
8	50.001	154	1	0.238	0.043	4.119	1866	WS0063_C08
9	2.011	167	4	0.225	0.062	4.693	1866	WS0063_C08
9	59.963	177	4	-0.240	0.065	5.496	1866	WS0063_C08
10	70.246	190	2	0.309	0.065	6.960	1866	WS0063_C08
11	41.984	212	2	-0.221	0.038	4.168	1866	WS0063_C08
1	2.788	2	3	-0.202	0.047	4.820	1867	WS0063_G02
1	29.800	5	1	0.307	0.057	4.995	1867	WS0063_G02
2	120.617	38	3	0.261	0.043	4.353	1867	WS0063_G02
4	142.917	85	1	-0.222	0.046	3.985	1867	WS0063_G02
6	26.350	115	2	0.176	0.040	3.983	1867	WS0063_G02
10	70.246	190	4	-0.338	0.061	5.221	1867	WS0063_G02
10	78.479	193	4	-0.311	0.083	7.214	1867	WS0063_G02
10	95.795	197	4	0.230	0.058	5.067	1867	WS0063_G02

14	46.637	252	3	0.347	0.077	7.732	1867	WS0063_G02
1	22.419	4	4	-0.287	0.100	4.056	1868	WS0063_G17
1	62.128	11	1	-0.484	0.108	4.433	1868	WS0063_G17
2	102.814	36	2	-0.180	0.039	4.334	1868	WS0063_G17
3	80.211	55	4	-0.173	0.049	4.767	1868	WS0063_G17
3	144.647	67	1	0.348	0.129	5.270	1868	WS0063_G17
4	6.032	72	4	0.198	0.059	5.692	1868	WS0063_G17
6	56.188	120	2	0.388	0.085	9.406	1868	WS0063_G17
6	87.891	124	2	0.193	0.034	3.935	1868	WS0063_G17
8	50.001	154	2	-0.292	0.091	9.866	1868	WS0063_G17
8	87.463	158	2	0.301	0.078	8.781	1868	WS0063_G17
9	17.951	169	1	-0.251	0.064	4.717	1868	WS0063_G17
9	17.951	169	2	0.461	0.097	12.605	1868	WS0063_G17
10	77.676	192	1	0.238	0.069	5.015	1868	WS0063_G17
10	80.088	194	1	0.239	0.071	5.197	1868	WS0063_G17
10	104.214	198	4	0.330	0.063	7.459	1868	WS0063_G17
11	48.979	218	4	0.196	0.036	4.005	1868	WS0063_G17
12	14.406	230	4	0.312	0.057	5.564	1868	WS0063_G17
1	82.301	16	4	0.423	0.073	6.825	1869	WS0063_K02
2	75.550	31	2	-0.685	0.189	23.195	1869	WS0063_K02
2	93.504	32	2	-0.451	0.139	17.594	1869	WS0063_K02
2	102.814	36	2	-0.509	0.182	25.683	1869	WS0063_K02
2	120.617	38	3	-0.125	0.056	4.492	1869	WS0063_K02
2	126.181	39	2	0.405	0.092	11.960	1869	WS0063_K02
3	83.563	56	3	0.128	0.055	4.385	1869	WS0063_K02
6	56.188	120	2	0.185	0.033	5.752	1869	WS0063_K02
7	17.610	131	3	-0.343	0.115	4.851	1869	WS0063_K02
9	28.608	170	2	0.252	0.028	4.069	1869	WS0063_K02
10	35.204	189	2	-0.131	0.020	4.080	1869	WS0063_K02
10	104.214	198	4	0.225	0.043	6.100	1869	WS0063_K02
11	45.989	216	2	-0.130	0.039	6.677	1869	WS0063_K02
1	38.326	6	2	-0.188	0.037	4.189	1870	WS0063_L14
5	0.000	89	3	-0.370	0.104	11.427	1870	WS0063_L14
5	16.219	93	2	-0.316	0.057	6.806	1870	WS0063_L14
9	28.608	170	2	-0.235	0.034	3.974	1870	WS0063_L14
9	61.113	178	1	-0.444	0.141	4.914	1870	WS0063_L14
10	35.204	189	2	-0.250	0.042	4.826	1870	WS0063_L14
10	78.479	193	3	0.330	0.040	4.445	1870	WS0063_L14
3	15.147	44	2	-0.394	0.106	5.999	1871	WS00810_A04
3	83.563	56	3	-0.156	0.044	3.855	1871	WS00810_A04
5	31.191	95	1	-0.303	0.111	7.023	1871	WS00810_A04
11	57.661	220	4	0.215	0.052	5.213	1871	WS00810_A04
1	2.788	2	3	0.279	0.059	6.022	1872	WS00928_P19
1	131.686	23	1	0.561	0.142	5.353	1872	WS00928_P19
2	101.076	35	4	-0.211	0.043	3.888	1872	WS00928_P19
6	49.100	118	4	-0.345	0.128	5.111	1872	WS00928_P19
8	39.090	152	4	0.262	0.064	5.602	1872	WS00928_P19
10	93.347	196	1	0.413	0.071	4.126	1872	WS00928_P19
11	27.405	204	1	0.266	0.040	3.852	1872	WS00928_P19
11	42.600	213	3	0.182	0.039	3.939	1872	WS00928_P19
1	45.794	9	3	-0.242	0.075	6.650	1873	WS00810_F07
5	0.000	89	1	-0.233	0.045	4.298	1873	WS00810_F07
6	49.100	118	2	0.234	0.105	4.918	1873	WS00810_F07
7	75.883	144	2	0.416	0.089	4.368	1873	WS00810_F07

8	0.000	148	4	-0.258	0.053	5.366	1873	WS00810_F07
8	39.090	152	4	0.226	0.038	3.920	1873	WS00810_F07
10	35.204	189	2	-0.198	0.051	4.093	1873	WS00810_F07
10	95.795	197	4	0.240	0.042	4.265	1873	WS00810_F07
12	0.000	226	3	0.191	0.076	6.576	1873	WS00810_F07
12	22.441	227	3	-0.341	0.058	3.984	1873	WS00810_F07
13	3.386	245	1	0.284	0.077	7.201	1873	WS00810_F07
6	25.914	114	1	0.172	0.066	5.945	1874	WS00929_C24
6	69.073	121	3	-0.246	0.050	6.379	1874	WS00929_C24
6	95.249	126	1	0.436	0.099	6.426	1874	WS00929_C24
11	17.088	203	2	-0.113	0.037	4.258	1874	WS00929_C24
11	27.405	204	1	0.446	0.093	8.678	1874	WS00929_C24
11	27.405	204	4	-0.259	0.054	3.895	1874	WS00929_C24
11	64.014	222	3	-0.241	0.074	9.362	1874	WS00929_C24
12	6.242	234	2	-0.308	0.052	7.224	1874	WS00929_C24
1	123.316	20	4	-0.227	0.081	5.407	1875	WS01021_I17
1	131.686	23	2	0.438	0.069	4.564	1875	WS01021_I17
6	87.891	124	2	-0.285	0.066	7.696	1875	WS01021_I17
7	105.477	147	2	-0.266	0.106	7.050	1875	WS01021_I17
11	60.464	221	2	0.209	0.052	4.649	1875	WS01021_I17
14	9.923	247	1	-0.205	0.058	4.114	1875	WS01021_I17
2	48.839	29	1	0.316	0.059	5.455	1876	WS01021_N11
2	126.181	39	2	-0.247	0.043	4.043	1876	WS01021_N11
2	126.181	39	4	0.618	0.160	10.773	1876	WS01021_N11
2	127.147	40	4	-0.216	0.039	4.049	1876	WS01021_N11
4	43.146	74	3	0.199	0.055	4.594	1876	WS01021_N11
4	173.929	88	2	0.266	0.087	8.328	1876	WS01021_N11
5	0.000	89	1	0.244	0.054	5.108	1876	WS01021_N11
5	16.219	93	1	0.240	0.048	4.405	1876	WS01021_N11
6	81.996	123	3	-0.282	0.098	4.729	1876	WS01021_N11
9	3.127	168	1	0.457	0.187	7.871	1876	WS01021_N11
9	17.951	169	1	-0.522	0.159	14.652	1876	WS01021_N11
1	43.578	8	2	-0.172	0.055	4.448	1877	WS00818_G22
1	43.578	8	3	0.331	0.047	4.800	1877	WS00818_G22
1	95.572	17	2	0.293	0.063	4.710	1877	WS00818_G22
2	95.880	34	1	0.437	0.090	10.803	1877	WS00818_G22
2	95.880	34	3	-0.343	0.072	7.482	1877	WS00818_G22
2	101.076	35	3	0.359	0.073	7.520	1877	WS00818_G22
2	102.814	36	1	0.335	0.049	6.237	1877	WS00818_G22
2	102.814	36	2	0.321	0.108	8.005	1877	WS00818_G22
2	120.617	38	3	0.316	0.077	7.872	1877	WS00818_G22
2	126.181	39	2	-0.481	0.205	16.379	1877	WS00818_G22
2	127.147	40	3	-0.273	0.067	6.926	1877	WS00818_G22
3	14.500	43	2	-0.207	0.069	5.243	1877	WS00818_G22
3	15.147	44	2	0.314	0.185	7.260	1877	WS00818_G22
3	20.512	46	2	0.298	0.095	7.123	1877	WS00818_G22
4	55.925	76	1	-0.247	0.041	4.642	1877	WS00818_G22
5	84.347	105	1	-0.217	0.043	4.865	1877	WS00818_G22
6	106.702	128	2	0.416	0.127	3.942	1877	WS00818_G22
7	15.555	130	3	0.243	0.063	4.628	1877	WS00818_G22
9	0.000	163	1	0.425	0.059	4.117	1877	WS00818_G22
9	81.073	183	1	0.225	0.038	4.279	1877	WS00818_G22
10	21.864	188	1	-0.275	0.033	3.916	1877	WS00818_G22
10	86.151	195	3	0.224	0.043	4.452	1877	WS00818_G22

11	0.000	199	2	0.323	0.065	4.779	1877	WS00818_G22
14	0.000	246	1	0.266	0.047	5.316	1877	WS00818_G22
2	127.147	40	4	-0.197	0.043	4.578	1878	WS00818_H14
3	95.157	59	2	-0.192	0.029	4.346	1878	WS00818_H14
4	103.719	81	4	-0.217	0.040	4.578	1878	WS00818_H14
12	28.493	228	2	-0.292	0.055	3.968	1878	WS00818_H14
13	1.815	244	4	0.295	0.047	5.070	1878	WS00818_H14
5	16.219	93	2	0.376	0.076	9.461	1879	WS00818_J10
5	31.191	95	1	-0.220	0.063	3.864	1879	WS00818_J10
9	2.011	167	4	0.335	0.089	8.372	1879	WS00818_J10
9	49.013	173	2	-0.215	0.033	3.852	1879	WS00818_J10
10	70.246	190	4	0.214	0.043	4.115	1879	WS00818_J10
1	69.854	13	2	-0.229	0.053	6.561	1880	WS00818_J20
1	71.592	14	2	-0.234	0.056	6.905	1880	WS00818_J20
1	77.177	15	2	-0.226	0.053	6.590	1880	WS00818_J20
1	95.572	17	2	-0.217	0.034	4.249	1880	WS00818_J20
1	107.333	19	4	-0.161	0.030	4.271	1880	WS00818_J20
2	75.550	31	2	0.231	0.036	4.227	1880	WS00818_J20
2	126.181	39	4	-0.404	0.048	4.891	1880	WS00818_J20
3	104.681	60	2	0.230	0.045	5.605	1880	WS00818_J20
3	112.868	61	2	-0.225	0.049	6.054	1880	WS00818_J20
3	114.747	62	2	-0.225	0.045	5.598	1880	WS00818_J20
5	57.017	102	2	-0.228	0.041	5.077	1880	WS00818_J20
7	62.787	139	2	0.217	0.042	6.068	1880	WS00818_J20
9	0.000	166	2	0.219	0.047	6.677	1880	WS00818_J20
9	2.011	167	4	0.202	0.044	6.030	1880	WS00818_J20
11	40.960	210	2	0.386	0.040	4.378	1880	WS00818_J20
2	120.617	38	3	0.214	0.037	4.175	1881	WS00818_L13
4	97.576	80	2	0.142	0.031	3.867	1881	WS00818_L13
5	31.191	95	1	-0.196	0.057	4.852	1881	WS00818_L13
6	81.996	123	3	-0.306	0.092	5.147	1881	WS00818_L13
6	89.401	125	1	-0.352	0.066	5.694	1881	WS00818_L13
9	59.963	177	4	-0.263	0.069	5.289	1881	WS00818_L13
10	78.479	193	3	-0.377	0.049	5.511	1881	WS00818_L13
11	35.604	207	3	-0.169	0.042	4.703	1881	WS00818_L13
13	3.386	245	1	0.264	0.056	4.791	1881	WS00818_L13
2	120.617	38	3	0.189	0.047	4.172	1882	WS00818_M03
2	127.147	40	3	-0.220	0.075	6.587	1882	WS00818_M03
3	116.958	63	4	0.301	0.091	9.142	1882	WS00818_M03
4	97.576	80	2	0.199	0.031	4.560	1882	WS00818_M03
5	7.672	92	4	-0.216	0.037	3.900	1882	WS00818_M03
5	31.191	95	1	-0.229	0.060	3.924	1882	WS00818_M03
6	56.188	120	2	0.229	0.046	5.284	1882	WS00818_M03
8	50.001	154	1	0.281	0.086	5.914	1882	WS00818_M03
9	59.963	177	4	-0.235	0.044	4.491	1882	WS00818_M03
10	70.246	190	2	0.240	0.040	4.909	1882	WS00818_M03
10	78.479	193	3	-0.307	0.060	5.295	1882	WS00818_M03
11	29.069	205	1	0.270	0.066	4.393	1882	WS00818_M03
1	38.326	6	2	0.216	0.066	5.983	1883	WS00818_O09
1	38.326	6	4	-0.379	0.055	5.771	1883	WS00818_O09
1	40.464	7	4	0.355	0.051	5.420	1883	WS00818_O09
3	47.928	49	1	0.247	0.049	4.863	1883	WS00818_O09
4	0.000	71	4	-0.216	0.051	5.264	1883	WS00818_O09
4	116.928	82	2	-0.286	0.061	5.509	1883	WS00818_O09

4	124.132	83	1	0.464	0.092	4.750	1883	WS00818_O09
5	42.979	97	1	-0.220	0.040	3.957	1883	WS00818_O09
6	89.401	125	1	-0.274	0.038	3.863	1883	WS00818_O09
8	11.974	150	1	-0.258	0.047	4.757	1883	WS00818_O09
8	50.001	154	1	0.224	0.036	3.934	1883	WS00818_O09
8	62.580	155	1	0.294	0.109	5.560	1883	WS00818_O09
2	127.147	40	3	-0.251	0.069	7.398	1884	WS00818_P03
4	0.000	71	4	-0.234	0.059	4.731	1884	WS00818_P03
8	50.001	154	1	0.260	0.050	4.017	1884	WS00818_P03
12	14.406	230	3	-0.305	0.040	4.467	1884	WS00818_P03
2	126.181	39	4	0.414	0.064	5.393	1885	WS0106_H03
3	116.958	63	4	-0.169	0.039	3.944	1885	WS0106_H03
7	74.560	143	1	0.296	0.097	6.709	1885	WS0106_H03
8	39.090	152	4	-0.252	0.047	4.779	1885	WS0106_H03
8	87.463	158	2	-0.234	0.050	5.364	1885	WS0106_H03
9	28.608	170	2	-0.290	0.053	6.265	1885	WS0106_H03
10	35.204	189	2	0.258	0.066	7.035	1885	WS0106_H03
11	0.000	199	3	0.287	0.053	4.194	1885	WS0106_H03
4	6.032	72	4	-0.160	0.038	3.880	1886	WS0106_H14
6	95.249	126	1	0.342	0.129	5.265	1886	WS0106_H14
9	32.356	171	2	-0.201	0.051	5.334	1886	WS0106_H14
1	62.128	11	1	-0.457	0.101	4.615	1887	WS0106_J08
1	127.805	21	4	0.230	0.040	4.692	1887	WS0106_J08
1	131.686	23	1	0.602	0.142	6.310	1887	WS0106_J08
8	50.001	154	2	-0.228	0.043	4.168	1887	WS0106_J08
9	55.358	175	1	-0.318	0.055	4.445	1887	WS0106_J08
11	40.960	210	2	0.404	0.078	5.076	1887	WS0106_J08
3	14.500	43	1	-0.265	0.080	5.920	1888	WS0106_J22
3	17.158	45	1	0.242	0.064	4.791	1888	WS0106_J22
4	43.146	74	3	0.287	0.056	6.626	1888	WS0106_J22
13	3.386	245	1	0.299	0.086	6.449	1888	WS0106_J22
2	102.814	36	2	-0.262	0.050	5.343	1889	WS0106_M09
2	127.147	40	4	-0.202	0.048	4.803	1889	WS0106_M09
3	80.211	55	4	-0.179	0.040	3.999	1889	WS0106_M09
3	104.681	60	2	-0.156	0.035	4.649	1889	WS0106_M09
3	144.593	66	4	0.161	0.035	4.040	1889	WS0106_M09
3	154.505	69	4	0.176	0.048	3.893	1889	WS0106_M09
4	173.929	88	2	-0.251	0.039	5.184	1889	WS0106_M09
5	42.979	97	2	-0.422	0.084	10.840	1889	WS0106_M09
9	17.951	169	2	0.278	0.038	4.040	1889	WS0106_M09
3	47.928	49	1	0.265	0.078	5.716	1890	WS0106_N03
5	42.979	97	2	-0.193	0.038	4.352	1890	WS0106_N03
5	56.008	101	2	-0.249	0.055	7.133	1890	WS0106_N03
5	57.017	102	2	0.203	0.039	4.474	1890	WS0106_N03
11	56.921	219	2	0.269	0.082	5.057	1890	WS0106_N03
1	45.794	9	1	0.289	0.048	4.039	1891	WS0106_O13
3	137.891	64	3	0.301	0.051	5.115	1891	WS0106_O13
3	141.443	65	3	0.314	0.079	3.870	1891	WS0106_O13
6	81.996	123	4	-0.319	0.150	5.024	1891	WS0106_O13
6	106.702	128	2	0.392	0.073	3.976	1891	WS0106_O13
8	87.463	158	2	0.196	0.039	4.023	1891	WS0106_O13
10	78.479	193	4	-0.265	0.065	5.599	1891	WS0106_O13
14	22.402	248	4	0.277	0.048	4.101	1891	WS0106_O13
1	82.301	16	3	-0.328	0.063	6.422	1892	WS0106_P02

3	14.500	43	1	-0.313	0.124	7.130	1892	WS0106_P02
6	26.350	115	2	0.197	0.042	5.403	1892	WS0106_P02
6	49.100	118	2	-0.471	0.139	9.496	1892	WS0106_P02
7	15.555	130	3	-0.208	0.046	4.130	1892	WS0106_P02
9	0.000	163	2	0.481	0.070	4.359	1892	WS0106_P02
9	59.963	177	1	0.285	0.068	3.931	1892	WS0106_P02
11	46.026	217	1	-0.263	0.072	4.174	1892	WS0106_P02
14	22.402	248	4	0.386	0.074	6.539	1892	WS0106_P02
14	46.637	252	4	0.359	0.059	4.685	1892	WS0106_P02
1	2.788	2	4	-0.222	0.072	5.110	1893	WS00824_E15
2	48.839	29	4	0.303	0.084	6.294	1893	WS00824_E15
11	27.405	204	1	0.446	0.107	9.616	1893	WS00824_E15
11	29.069	205	1	0.290	0.065	5.803	1893	WS00824_E15
1	2.788	2	4	-0.317	0.096	9.620	1894	WS00824_F05
1	15.589	3	2	0.234	0.046	5.267	1894	WS00824_F05
1	82.301	16	3	-0.400	0.092	8.847	1894	WS00824_F05
1	95.572	17	3	0.260	0.077	6.873	1894	WS00824_F05
3	141.443	65	3	-0.200	0.090	3.921	1894	WS00824_F05
4	92.039	79	2	-0.266	0.066	7.507	1894	WS00824_F05
6	89.401	125	1	-0.266	0.053	4.002	1894	WS00824_F05
7	17.610	131	3	-0.315	0.076	4.016	1894	WS00824_F05
8	87.463	158	2	0.216	0.055	5.917	1894	WS00824_F05
10	70.246	190	2	0.184	0.037	4.187	1894	WS00824_F05
11	27.405	204	1	0.396	0.096	7.161	1894	WS00824_F05
12	22.441	227	3	-0.334	0.086	7.877	1894	WS00824_F05
7	62.787	139	4	-0.310	0.082	6.240	1895	WS00824_G20
9	28.608	170	2	-0.226	0.039	4.660	1895	WS00824_G20
9	55.358	175	1	0.322	0.079	5.938	1895	WS00824_G20
11	35.604	207	3	0.244	0.039	4.039	1895	WS00824_G20
12	22.629	237	1	0.221	0.091	6.803	1895	WS00824_G20
12	32.473	238	1	0.219	0.088	6.574	1895	WS00824_G20
1	141.021	25	2	0.196	0.035	4.020	1896	WS00824_H12
2	75.550	31	2	-0.377	0.063	5.623	1896	WS00824_H12
8	11.974	150	1	-0.273	0.077	5.710	1896	WS00824_H12
9	17.951	169	1	0.409	0.099	7.517	1896	WS00824_H12
10	35.204	189	2	-0.244	0.076	8.738	1896	WS00824_H12
11	40.960	210	2	0.635	0.198	11.118	1896	WS00824_H12
11	40.960	210	4	-0.409	0.095	8.350	1896	WS00824_H12
1	2.788	2	4	-0.203	0.066	5.054	1897	WS00824_J18
1	22.419	4	3	0.316	0.070	3.878	1897	WS00824_J18
3	15.147	44	2	0.168	0.056	4.065	1897	WS00824_J18
4	55.925	76	1	-0.264	0.052	4.009	1897	WS00824_J18
6	69.073	121	4	-0.192	0.050	4.416	1897	WS00824_J18
6	87.891	124	2	-0.256	0.085	8.580	1897	WS00824_J18
6	89.401	125	2	-0.376	0.086	5.846	1897	WS00824_J18
9	81.073	183	1	0.218	0.044	3.876	1897	WS00824_J18
11	27.405	204	1	0.385	0.099	8.717	1897	WS00824_J18
13	3.386	245	1	-0.211	0.045	3.920	1897	WS00824_J18
4	82.462	78	1	-0.244	0.032	4.053	1898	WS00824_K04
8	87.463	158	2	-0.219	0.067	4.304	1898	WS00824_K04
11	0.000	199	3	-0.409	0.096	7.840	1898	WS00824_K04
11	27.405	204	1	0.378	0.083	10.238	1898	WS00824_K04
11	29.069	205	1	0.281	0.055	6.833	1898	WS00824_K04
11	45.989	216	1	-0.215	0.044	5.341	1898	WS00824_K04

11	64.014	222	3	-0.222	0.049	5.514	1898	WS00824_K04
14	9.923	247	1	-0.242	0.039	4.771	1898	WS00824_K04
14	22.402	248	3	0.207	0.040	4.513	1898	WS00824_K04
1	141.021	25	2	0.147	0.039	4.066	1899	WS00824_L19
6	106.702	128	2	0.422	0.110	4.459	1899	WS00824_L19
6	106.702	128	3	-0.238	0.063	4.528	1899	WS00824_L19
9	59.963	177	4	0.314	0.049	5.730	1899	WS00824_L19
11	43.491	214	4	-0.283	0.045	4.855	1899	WS00824_L19
11	45.989	216	1	0.275	0.059	5.063	1899	WS00824_L19
14	0.000	246	1	-0.188	0.064	5.354	1899	WS00824_L19
14	22.402	248	4	-0.470	0.083	9.439	1899	WS00824_L19
14	43.392	250	1	0.311	0.169	5.842	1899	WS00824_L19
14	46.637	252	4	-0.374	0.053	6.130	1899	WS00824_L19
4	124.132	83	1	-0.506	0.158	5.552	1900	WS00824_M06
4	142.917	85	1	0.312	0.076	5.741	1900	WS00824_M06
5	52.820	99	3	-0.145	0.049	4.302	1900	WS00824_M06
6	49.100	118	4	-0.448	0.148	5.325	1900	WS00824_M06
6	54.066	119	4	-0.182	0.048	4.725	1900	WS00824_M06
6	69.073	121	4	0.156	0.029	3.870	1900	WS00824_M06
9	36.227	165	3	-0.237	0.043	4.029	1900	WS00824_M06
10	78.479	193	4	0.215	0.045	5.004	1900	WS00824_M06
10	95.795	197	4	-0.189	0.052	5.089	1900	WS00824_M06
11	0.000	199	2	0.213	0.043	4.981	1900	WS00824_M06
11	27.405	204	1	0.285	0.056	4.269	1900	WS00824_M06
11	27.405	204	4	-0.232	0.055	5.498	1900	WS00824_M06
11	40.960	210	2	0.393	0.065	4.463	1900	WS00824_M06
1	29.800	5	2	-0.282	0.066	6.168	1901	WS00731_I18
1	38.326	6	2	0.264	0.051	4.947	1901	WS00731_I18
3	95.157	59	2	0.178	0.050	5.054	1901	WS00731_I18
4	0.000	71	4	-0.307	0.101	8.557	1901	WS00731_I18
8	50.001	154	1	0.285	0.062	5.593	1901	WS00731_I18
9	17.951	169	1	0.320	0.073	6.776	1901	WS00731_I18
10	70.246	190	2	0.241	0.047	4.516	1901	WS00731_I18
1	62.128	11	1	-0.527	0.102	5.697	1902	WS00112_M17
1	123.316	20	4	-0.214	0.086	5.000	1902	WS00112_M17
1	127.805	21	4	0.408	0.106	11.608	1902	WS00112_M17
1	131.686	23	1	0.505	0.084	4.686	1902	WS00112_M17
3	47.928	49	1	0.222	0.041	3.964	1902	WS00112_M17
4	0.000	71	4	-0.304	0.117	13.290	1902	WS00112_M17
9	0.000	166	1	0.188	0.041	3.980	1902	WS00112_M17
9	81.073	183	1	0.229	0.045	4.357	1902	WS00112_M17
12	14.406	230	4	-0.289	0.044	4.684	1902	WS00112_M17
3	47.928	49	1	0.256	0.068	5.752	1903	WS00732_E24
4	75.958	77	3	-0.333	0.068	4.404	1903	WS00732_E24
6	95.249	126	1	0.324	0.079	4.084	1903	WS00732_E24
7	74.560	143	2	0.269	0.037	4.060	1903	WS00732_E24
9	59.963	177	4	-0.321	0.077	5.624	1903	WS00732_E24
10	78.479	193	3	-0.319	0.036	4.159	1903	WS00732_E24
11	29.069	205	1	0.287	0.058	5.038	1903	WS00732_E24
1	15.589	3	2	-0.175	0.048	4.351	1904	WS00113_H06
8	87.463	158	1	0.255	0.054	5.239	1904	WS00113_H06
11	27.405	204	4	0.291	0.044	4.476	1904	WS00113_H06
11	57.661	220	4	-0.228	0.033	3.881	1904	WS00113_H06
12	14.406	230	4	-0.356	0.048	5.465	1904	WS00113_H06

12	22.629	237	1	-0.228	0.041	3.987	1904	WS00113_H06
14	46.637	252	4	0.227	0.039	4.316	1904	WS00113_H06
2	120.617	38	3	0.244	0.032	4.137	1905	WS01040_A18
3	14.500	43	1	-0.263	0.054	5.161	1905	WS01040_A18
3	47.928	49	1	0.203	0.048	4.602	1905	WS01040_A18
8	11.974	150	1	-0.324	0.091	8.769	1905	WS01040_A18
9	0.000	163	1	-0.461	0.072	5.006	1905	WS01040_A18
9	36.227	165	3	0.216	0.036	4.613	1905	WS01040_A18
1	62.128	11	1	0.458	0.066	4.779	1906	WS01040_J02
4	116.928	82	4	-0.233	0.053	4.066	1906	WS01040_J02
9	3.127	168	1	-0.621	0.200	15.035	1906	WS01040_J02
1	95.572	17	2	-0.298	0.045	5.664	1907	WS0015_D02
1	95.572	17	3	0.291	0.072	6.696	1907	WS0015_D02
9	81.073	183	1	-0.203	0.066	4.314	1907	WS0015_D02
11	40.960	210	2	-0.405	0.066	5.246	1907	WS0015_D02
12	0.000	226	3	0.300	0.093	8.198	1907	WS0015_D02
2	127.147	40	3	-0.168	0.038	4.218	1908	WS0081_C15
4	124.132	83	1	0.441	0.125	3.891	1908	WS0081_C15
4	142.917	85	1	-0.237	0.041	3.913	1908	WS0081_C15
6	89.401	125	1	-0.291	0.047	4.532	1908	WS0081_C15
6	95.249	126	1	0.334	0.075	4.837	1908	WS0081_C15
10	70.246	190	2	0.274	0.055	5.094	1908	WS0081_C15
11	27.405	204	1	0.294	0.042	4.057	1908	WS0081_C15
11	29.069	205	1	0.275	0.053	5.083	1908	WS0081_C15
11	64.014	222	3	-0.273	0.069	7.727	1908	WS0081_C15
3	47.928	49	1	0.240	0.042	3.879	1909	WS0015_P23
4	142.917	85	1	0.276	0.050	4.563	1909	WS0015_P23
5	86.669	106	2	-0.378	0.140	6.249	1909	WS0015_P23
6	45.897	117	4	-0.195	0.046	4.007	1909	WS0015_P23
9	55.358	175	3	-0.451	0.091	5.275	1909	WS0015_P23
10	21.864	188	3	0.299	0.059	5.903	1909	WS0015_P23
11	64.014	222	4	-0.274	0.071	6.535	1909	WS0015_P23
12	14.406	230	4	0.222	0.050	4.319	1909	WS0015_P23
12	6.242	234	2	-0.196	0.030	3.865	1909	WS0015_P23
13	3.386	245	1	-0.185	0.043	3.925	1909	WS0015_P23
6	89.401	125	1	-0.313	0.073	5.948	1910	WS0081_K01
7	17.610	131	3	-0.336	0.064	4.263	1910	WS0081_K01
8	11.974	150	1	-0.284	0.046	4.021	1910	WS0081_K01
9	3.127	168	1	-0.311	0.118	4.977	1910	WS0081_K01
9	59.963	177	4	-0.267	0.068	5.655	1910	WS0081_K01
6	95.249	126	1	0.435	0.188	5.949	1911	WS00929_G05
3	80.211	55	4	-0.274	0.055	5.290	1912	WS00929_J06
6	87.891	124	2	0.210	0.048	5.091	1912	WS00929_J06
6	89.401	125	2	0.262	0.060	5.905	1912	WS00929_J06
9	59.963	177	4	-0.219	0.053	5.667	1912	WS00929_J06
11	64.014	222	4	0.171	0.047	4.205	1912	WS00929_J06
2	127.147	40	3	-0.229	0.048	6.397	1913	WS00819_I01
5	31.191	95	1	-0.261	0.053	4.878	1913	WS00819_I01
6	95.249	126	1	0.296	0.051	3.988	1913	WS00819_I01
11	27.405	204	1	0.378	0.089	8.198	1913	WS00819_I01
11	29.069	205	1	0.249	0.058	5.330	1913	WS00819_I01
1	22.419	4	3	-0.288	0.118	6.036	1914	WS00819_I11
3	154.505	69	1	0.306	0.072	5.778	1914	WS00819_I11
3	154.505	69	4	-0.255	0.055	4.904	1914	WS00819_I11

6	69.073	121	3	0.295	0.048	4.828	1914	WS00819_I11
9	55.358	175	1	-0.359	0.083	6.650	1914	WS00819_I11
9	55.358	175	3	0.396	0.079	4.375	1914	WS00819_I11
9	59.963	177	4	0.235	0.057	4.193	1914	WS00819_I11
9	69.046	181	2	0.398	0.121	5.507	1914	WS00819_I11
9	81.073	183	1	0.296	0.070	5.436	1914	WS00819_I11
11	29.069	205	2	-0.234	0.039	4.098	1914	WS00819_I11
11	60.464	221	2	-0.210	0.036	4.018	1914	WS00819_I11
11	64.014	222	4	-0.326	0.079	7.010	1914	WS00819_I11
14	43.392	250	1	0.332	0.095	4.882	1914	WS00819_I11
8	87.463	158	2	-0.462	0.200	20.622	1915	WS00819_J23
8	95.937	159	3	-0.222	0.068	7.334	1915	WS00819_J23
9	0.000	166	2	0.248	0.046	4.604	1915	WS00819_J23
11	27.405	204	1	0.407	0.117	9.929	1915	WS00819_J23
11	29.069	205	1	0.317	0.087	7.306	1915	WS00819_J23
11	45.989	216	1	-0.224	0.058	4.832	1915	WS00819_J23
1	40.464	7	4	0.198	0.082	4.447	1916	WS00819_K12
1	95.572	17	2	-0.246	0.037	4.367	1916	WS00819_K12
1	127.805	21	3	0.235	0.036	4.510	1916	WS00819_K12
11	0.000	199	3	-0.376	0.080	9.989	1916	WS00819_K12
12	22.441	227	3	0.226	0.050	6.225	1916	WS00819_K12
2	75.550	31	1	0.287	0.061	5.623	1917	WS00819_N04
3	47.928	49	1	0.214	0.047	4.337	1917	WS00819_N04
4	55.925	76	1	-0.261	0.062	5.090	1917	WS00819_N04
4	82.462	78	1	-0.318	0.099	9.130	1917	WS00819_N04
8	39.090	152	4	0.262	0.055	5.155	1917	WS00819_N04
8	87.463	158	2	-0.271	0.075	5.877	1917	WS00819_N04
11	27.405	204	1	0.226	0.043	4.003	1917	WS00819_N04
11	40.960	210	4	-0.293	0.060	3.891	1917	WS00819_N04
12	0.000	233	1	0.239	0.060	5.516	1917	WS00819_N04
1	2.788	2	4	-0.231	0.077	7.060	1918	WS00819_N14
1	40.464	7	3	0.262	0.051	4.946	1918	WS00819_N14
1	43.578	8	3	0.252	0.047	4.123	1918	WS00819_N14
3	112.868	61	2	-0.224	0.045	4.392	1918	WS00819_N14
3	114.747	62	2	-0.168	0.043	4.176	1918	WS00819_N14
4	82.462	78	2	0.229	0.051	5.478	1918	WS00819_N14
4	97.576	80	2	-0.174	0.044	4.836	1918	WS00819_N14
5	56.008	101	2	-0.222	0.044	4.537	1918	WS00819_N14
6	45.897	117	4	-0.232	0.047	3.925	1918	WS00819_N14
6	49.100	118	4	-0.390	0.098	4.082	1918	WS00819_N14
6	81.996	123	3	0.315	0.091	4.630	1918	WS00819_N14
10	21.864	188	1	-0.330	0.042	3.910	1918	WS00819_N14
10	35.204	189	2	-0.215	0.045	3.999	1918	WS00819_N14
3	47.928	49	1	0.212	0.049	3.928	1919	WS00820_A08
3	95.157	59	2	0.188	0.040	4.387	1919	WS00820_A08
8	50.001	154	1	0.230	0.056	4.797	1919	WS00820_A08
9	59.963	177	4	-0.298	0.084	7.441	1919	WS00820_A08
10	9.580	187	3	-0.368	0.109	5.329	1919	WS00820_A08
10	70.246	190	2	0.293	0.061	6.722	1919	WS00820_A08
10	78.479	193	3	-0.256	0.039	3.956	1919	WS00820_A08
10	80.088	194	2	-0.262	0.042	4.066	1919	WS00820_A08
13	3.386	245	1	0.259	0.049	4.022	1919	WS00820_A08
1	82.301	16	3	-0.254	0.044	4.906	1920	WS00820_A14
2	120.617	38	3	0.232	0.048	5.384	1920	WS00820_A14

2	127.147	40	3	-0.233	0.057	6.331	1920	WS00820_A14
3	95.157	59	2	0.296	0.086	8.592	1920	WS00820_A14
7	17.610	131	3	-0.382	0.117	7.258	1920	WS00820_A14
7	73.169	142	1	0.507	0.102	4.120	1920	WS00820_A14
8	11.974	150	1	-0.215	0.057	5.047	1920	WS00820_A14
9	3.127	168	1	-0.459	0.120	5.991	1920	WS00820_A14
10	70.246	190	2	0.256	0.054	5.358	1920	WS00820_A14
11	17.088	203	1	-0.225	0.066	5.890	1920	WS00820_A14
11	29.069	205	1	0.313	0.077	6.934	1920	WS00820_A14
13	0.000	243	4	-0.240	0.112	4.519	1920	WS00820_A14
3	95.157	59	2	0.264	0.059	5.346	1921	WS01040_N03
4	0.000	71	4	-0.480	0.149	14.809	1921	WS01040_N03
9	36.227	165	3	0.253	0.041	3.862	1921	WS01040_N03
9	81.073	183	1	-0.189	0.048	4.030	1921	WS01040_N03
9	81.073	183	3	-0.380	0.099	8.521	1921	WS01040_N03
10	70.246	190	2	0.301	0.059	5.998	1921	WS01040_N03
10	78.479	193	3	-0.369	0.062	5.767	1921	WS01040_N03
10	80.088	194	2	-0.214	0.051	4.188	1921	WS01040_N03
12	28.493	228	2	-0.326	0.082	4.186	1921	WS01040_N03
2	120.617	38	3	0.262	0.081	8.172	1922	WS01040_N16
2	127.147	40	3	-0.178	0.066	6.620	1922	WS01040_N16
3	47.928	49	1	0.222	0.056	4.889	1922	WS01040_N16
5	31.191	95	1	-0.215	0.056	4.814	1922	WS01040_N16
6	54.066	119	4	0.151	0.056	4.861	1922	WS01040_N16
10	78.479	193	3	-0.434	0.078	7.943	1922	WS01040_N16
11	29.069	205	1	0.347	0.076	6.833	1922	WS01040_N16
1	107.333	19	4	-0.287	0.070	4.897	1923	WS01041_A22
4	75.958	77	3	-0.283	0.068	4.006	1923	WS01041_A22
6	25.914	114	1	0.192	0.058	4.082	1923	WS01041_A22
6	106.702	128	2	0.431	0.084	5.181	1923	WS01041_A22
7	105.477	147	2	-0.218	0.081	4.758	1923	WS01041_A22
10	78.479	193	3	-0.349	0.055	5.471	1923	WS01041_A22
10	93.347	196	2	0.411	0.067	3.861	1923	WS01041_A22
1	82.301	16	4	-0.227	0.045	3.957	1924	WS01041_B23
6	87.891	124	2	0.246	0.037	3.890	1924	WS01041_B23
9	36.227	165	3	0.256	0.063	6.622	1924	WS01041_B23
9	32.356	171	2	-0.310	0.074	7.512	1924	WS01041_B23
10	70.246	190	4	-0.181	0.050	4.720	1924	WS01041_B23
11	42.600	213	3	0.183	0.047	4.888	1924	WS01041_B23
12	28.493	228	2	-0.239	0.090	4.035	1924	WS01041_B23
1	123.316	20	4	0.313	0.097	5.535	1925	WS01041_E15
6	25.742	113	3	0.237	0.053	5.240	1925	WS01041_E15
8	50.001	154	1	0.197	0.047	4.047	1925	WS01041_E15
10	70.246	190	2	0.261	0.038	4.240	1925	WS01041_E15
13	3.386	245	1	0.203	0.051	3.888	1925	WS01041_E15
1	127.805	21	4	0.232	0.052	4.312	1926	WS01041_F14
1	131.686	23	1	0.651	0.136	7.795	1926	WS01041_F14
2	75.550	31	1	0.163	0.045	4.965	1926	WS01041_F14
2	95.880	34	1	0.339	0.040	4.449	1926	WS01041_F14
4	43.146	74	3	0.288	0.045	4.472	1926	WS01041_F14
4	116.928	82	4	0.372	0.120	11.565	1926	WS01041_F14
5	56.008	101	2	-0.268	0.036	4.021	1926	WS01041_F14
6	45.897	117	1	0.431	0.071	8.223	1926	WS01041_F14
6	45.897	117	4	-0.283	0.046	4.971	1926	WS01041_F14

6	56.188	120	1	0.385	0.063	7.301	1926	WS01041_F14
7	73.169	142	1	-0.488	0.072	4.104	1926	WS01041_F14
7	75.883	144	2	-0.447	0.131	5.731	1926	WS01041_F14
10	86.151	195	3	-0.108	0.044	4.046	1926	WS01041_F14
12	0.000	226	3	0.216	0.043	3.996	1926	WS01041_F14
12	14.406	230	4	-0.284	0.035	4.585	1926	WS01041_F14
14	46.637	252	4	-0.292	0.036	4.069	1926	WS01041_F14
1	29.800	5	2	-0.340	0.050	4.315	1927	WS01041_J12
1	43.578	8	2	-0.251	0.087	7.736	1927	WS01041_J12
3	47.928	49	1	0.194	0.047	4.657	1927	WS01041_J12
6	87.891	124	2	-0.270	0.074	6.545	1927	WS01041_J12
6	89.401	125	1	-0.332	0.056	5.731	1927	WS01041_J12
6	95.249	126	1	0.408	0.133	8.174	1927	WS01041_J12
7	75.883	144	2	0.406	0.087	4.499	1927	WS01041_J12
8	50.001	154	1	0.189	0.042	4.238	1927	WS01041_J12
8	78.389	157	1	0.255	0.049	4.939	1927	WS01041_J12
8	87.463	158	1	-0.241	0.044	4.430	1927	WS01041_J12
11	64.014	222	3	-0.302	0.077	7.734	1927	WS01041_J12
1	82.301	16	4	-0.232	0.045	3.996	1928	WS01041_K15
3	9.953	42	3	-0.209	0.056	4.783	1928	WS01041_K15
4	140.962	84	3	-0.222	0.052	4.442	1928	WS01041_K15
5	52.820	99	3	0.221	0.045	3.878	1928	WS01041_K15
5	54.072	100	4	0.217	0.051	5.232	1928	WS01041_K15
6	45.897	117	4	0.308	0.071	5.547	1928	WS01041_K15
10	80.088	194	2	0.326	0.053	6.017	1928	WS01041_K15
10	93.347	196	2	-0.572	0.160	5.327	1928	WS01041_K15
11	27.405	204	1	-0.284	0.071	3.999	1928	WS01041_K15
12	28.493	228	2	-0.273	0.059	4.938	1928	WS01041_K15
14	22.402	248	4	-0.354	0.075	7.489	1928	WS01041_K15
1	133.992	24	2	-0.214	0.058	6.653	1929	WS00729_E24
1	141.021	25	2	0.171	0.042	4.760	1929	WS00729_E24
4	0.000	71	4	-0.236	0.093	9.106	1929	WS00729_E24
4	116.928	82	4	0.264	0.054	4.956	1929	WS00729_E24
6	95.249	126	1	0.341	0.144	5.498	1929	WS00729_E24
7	15.555	130	3	-0.194	0.048	4.220	1929	WS00729_E24
7	17.610	131	3	-0.395	0.137	7.063	1929	WS00729_E24
10	80.088	194	2	-0.274	0.037	4.140	1929	WS00729_E24
9	0.000	163	2	-0.482	0.135	4.556	1930	WS00834_D13
10	9.580	187	3	-0.271	0.074	4.252	1930	WS00834_D13
11	29.069	205	2	-0.190	0.050	4.378	1930	WS00834_D13
11	64.014	222	3	-0.332	0.080	8.005	1930	WS00834_D13
2	48.839	29	1	-0.342	0.123	10.975	1931	WS00940_C16
3	83.563	56	3	0.259	0.089	7.615	1931	WS00940_C16
3	112.868	61	2	0.186	0.039	3.956	1931	WS00940_C16
3	148.745	68	4	-0.238	0.040	4.208	1931	WS00940_C16
6	45.897	117	1	0.331	0.065	5.827	1931	WS00940_C16
6	81.996	123	3	-0.206	0.073	3.944	1931	WS00940_C16
6	106.702	128	3	0.349	0.082	6.781	1931	WS00940_C16
7	105.477	147	2	0.303	0.083	5.811	1931	WS00940_C16
8	0.000	148	4	-0.284	0.064	6.543	1931	WS00940_C16
9	0.000	166	1	0.174	0.051	4.465	1931	WS00940_C16
9	59.963	177	4	-0.278	0.055	5.948	1931	WS00940_C16
9	81.073	183	3	-0.240	0.051	4.289	1931	WS00940_C16
11	40.960	210	2	0.397	0.064	4.727	1931	WS00940_C16

14	46.637	252	4	-0.314	0.053	5.205	1931	WS00940_C16
1	95.572	17	3	0.323	0.058	4.387	1932	WS00834_N11
6	49.100	118	2	-0.400	0.101	5.802	1932	WS00834_N11
8	0.000	148	4	-0.264	0.046	4.094	1932	WS00834_N11
8	95.937	159	3	0.139	0.040	4.088	1932	WS00834_N11
10	21.864	188	3	0.147	0.034	4.117	1932	WS00834_N11
11	29.069	205	1	-0.263	0.052	4.222	1932	WS00834_N11
11	45.989	216	1	0.276	0.075	5.964	1932	WS00834_N11
12	28.493	228	2	0.259	0.087	4.717	1932	WS00834_N11
3	47.928	49	1	0.344	0.073	8.726	1933	WS0094_G24
4	0.000	71	4	-0.208	0.051	4.743	1933	WS0094_G24
4	82.462	78	1	-0.292	0.043	5.227	1933	WS0094_G24
1	22.419	4	3	0.323	0.062	4.287	1934	WS0094_L02
1	131.686	23	1	0.494	0.083	4.185	1934	WS0094_L02
7	73.169	142	1	0.519	0.092	4.672	1934	WS0094_L02
11	27.405	204	1	0.388	0.072	8.564	1934	WS0094_L02
11	29.069	205	1	0.269	0.044	5.298	1934	WS0094_L02
11	64.014	222	3	-0.188	0.045	4.104	1934	WS0094_L02
12	38.543	232	4	-0.334	0.090	4.526	1934	WS0094_L02
2	95.880	34	3	0.185	0.033	4.012	1935	WS0018_E04
7	15.555	130	3	-0.287	0.056	6.258	1935	WS0018_E04
7	17.610	131	3	-0.255	0.075	4.357	1935	WS0018_E04
9	49.013	173	1	0.177	0.054	4.330	1935	WS0018_E04
11	45.989	216	1	-0.250	0.049	3.907	1935	WS0018_E04
12	22.441	227	3	-0.185	0.040	4.996	1935	WS0018_E04
1	29.800	5	1	0.219	0.033	3.998	1936	WS00940_O21
2	48.839	29	4	0.303	0.059	4.465	1936	WS00940_O21
5	56.008	101	1	-0.186	0.032	3.901	1936	WS00940_O21
5	57.017	102	2	-0.230	0.075	4.898	1936	WS00940_O21
6	45.897	117	1	-0.254	0.035	4.324	1936	WS00940_O21
8	95.937	159	3	-0.171	0.038	4.258	1936	WS00940_O21
11	46.026	217	1	0.302	0.046	5.681	1936	WS00940_O21
12	14.406	230	3	-0.312	0.052	5.951	1936	WS00940_O21
3	47.928	49	1	0.226	0.052	4.168	1937	WS0019_C14
6	95.249	126	1	0.313	0.108	5.159	1937	WS0019_C14
8	87.463	158	2	-0.221	0.042	4.486	1937	WS0019_C14
10	9.580	187	2	-0.254	0.059	4.128	1937	WS0019_C14
11	60.464	221	2	0.399	0.114	12.062	1937	WS0019_C14
13	3.386	245	1	0.307	0.089	7.085	1937	WS0019_C14
1	123.316	20	4	-0.374	0.120	9.204	1938	WS00941_H11
1	127.805	21	4	0.220	0.045	4.056	1938	WS00941_H11
2	48.839	29	4	-0.219	0.046	4.219	1938	WS00941_H11
3	14.500	43	2	-0.270	0.055	6.747	1938	WS00941_H11
3	15.147	44	2	0.336	0.095	7.163	1938	WS00941_H11
3	20.512	46	2	0.280	0.060	7.860	1938	WS00941_H11
3	95.157	59	2	-0.178	0.031	4.427	1938	WS00941_H11
3	104.681	60	2	0.189	0.032	4.215	1938	WS00941_H11
3	112.868	61	2	-0.180	0.033	4.298	1938	WS00941_H11
3	114.747	62	2	-0.209	0.035	4.554	1938	WS00941_H11
9	3.127	168	1	0.351	0.285	4.073	1938	WS00941_H11
10	35.204	189	2	0.253	0.040	5.363	1938	WS00941_H11
10	93.347	196	1	0.420	0.099	4.235	1938	WS00941_H11
11	0.000	199	2	0.248	0.033	4.271	1938	WS00941_H11
11	35.604	207	3	0.236	0.056	5.423	1938	WS00941_H11

11	37.143	209	3	-0.250	0.063	6.074	1938	WS00941_H11
11	41.984	212	2	0.178	0.030	3.927	1938	WS00941_H11
11	42.600	213	3	0.247	0.057	5.534	1938	WS00941_H11
11	45.989	216	2	0.211	0.040	4.972	1938	WS00941_H11
11	56.921	219	2	-0.266	0.076	4.077	1938	WS00941_H11
1	69.854	13	2	-0.181	0.037	4.142	1939	WS00923_E19
1	71.592	14	2	-0.176	0.035	3.934	1939	WS00923_E19
1	77.177	15	2	-0.232	0.059	6.582	1939	WS00923_E19
1	82.301	16	4	-0.223	0.036	4.123	1939	WS00923_E19
1	95.572	17	2	-0.251	0.060	6.933	1939	WS00923_E19
1	107.333	19	4	-0.229	0.050	5.456	1939	WS00923_E19
2	48.839	29	4	-0.220	0.048	5.384	1939	WS00923_E19
2	101.076	35	4	0.258	0.036	4.767	1939	WS00923_E19
4	55.925	76	1	0.213	0.048	3.955	1939	WS00923_E19
6	45.897	117	4	-0.236	0.036	3.962	1939	WS00923_E19
6	89.401	125	2	-0.226	0.039	3.956	1939	WS00923_E19
7	105.477	147	2	0.341	0.079	6.274	1939	WS00923_E19
10	21.864	188	1	0.233	0.052	3.994	1939	WS00923_E19
11	27.405	204	1	-0.294	0.059	4.423	1939	WS00923_E19
14	0.000	246	2	0.306	0.099	11.008	1939	WS00923_E19
14	44.898	251	3	0.299	0.084	6.479	1939	WS00923_E19
3	47.928	49	1	0.276	0.059	5.316	1940	WS00923_I06
3	154.505	69	4	-0.186	0.051	4.669	1940	WS00923_I06
8	87.463	158	2	0.216	0.062	6.161	1940	WS00923_I06
9	59.963	177	4	-0.464	0.182	13.695	1940	WS00923_I06
12	22.629	237	1	0.176	0.046	4.086	1940	WS00923_I06
4	75.958	77	3	-0.226	0.078	3.952	1941	WS0108_H23
4	142.917	85	3	0.240	0.049	4.645	1941	WS0108_H23
5	54.072	100	4	-0.244	0.062	5.924	1941	WS0108_H23
6	56.188	120	2	0.296	0.052	6.054	1941	WS0108_H23
9	0.000	166	2	0.248	0.070	8.290	1941	WS0108_H23
9	59.963	177	4	-0.239	0.058	5.432	1941	WS0108_H23
9	59.963	177	4	-0.315	0.076	6.680	1942	WS0108_I16
11	29.069	205	1	0.265	0.065	5.275	1942	WS0108_I16
1	40.464	7	4	0.319	0.049	3.863	1943	WS0108_K15
3	47.928	49	1	0.181	0.056	4.550	1943	WS0108_K15
4	75.958	77	3	-0.383	0.109	6.820	1943	WS0108_K15
8	50.001	154	1	0.178	0.053	4.266	1943	WS0108_K15
9	0.000	166	2	0.194	0.038	4.425	1943	WS0108_K15
9	59.963	177	4	-0.321	0.114	7.763	1943	WS0108_K15
10	9.580	187	3	-0.309	0.102	4.748	1943	WS0108_K15
10	78.479	193	3	-0.414	0.077	8.511	1943	WS0108_K15
12	22.441	227	3	-0.225	0.052	5.891	1943	WS0108_K15
12	6.242	234	2	0.245	0.036	4.018	1943	WS0108_K15
1	77.177	15	2	-0.225	0.047	4.769	1944	WS0108_L13
8	50.001	154	1	0.175	0.045	4.317	1944	WS0108_L13
9	59.963	177	4	-0.279	0.066	6.175	1944	WS0108_L13
12	22.629	237	1	0.300	0.074	6.440	1944	WS0108_L13
12	51.382	242	1	-0.414	0.121	4.617	1944	WS0108_L13
2	126.181	39	4	0.470	0.084	4.980	1945	WS0108_N17
9	17.951	169	1	-0.228	0.054	3.887	1945	WS0108_N17
9	55.358	175	3	0.438	0.099	4.907	1945	WS0108_N17
9	69.046	181	1	0.320	0.089	4.454	1945	WS0108_N17
10	70.246	190	2	-0.307	0.048	5.702	1945	WS0108_N17

10	104.214	198	2	0.199	0.040	4.501	1945	WS0108_N17
11	27.405	204	4	0.324	0.058	5.079	1945	WS0108_N17
1	22.419	4	4	-0.337	0.074	5.374	1946	WS0108_O15
1	45.794	9	1	-0.325	0.078	8.208	1946	WS0108_O15
1	50.955	10	1	0.234	0.038	4.022	1946	WS0108_O15
4	124.132	83	1	0.543	0.114	7.457	1946	WS0108_O15
4	142.917	85	1	-0.257	0.050	5.252	1946	WS0108_O15
6	49.100	118	4	0.318	0.063	4.084	1946	WS0108_O15
7	73.169	142	1	0.467	0.059	3.865	1946	WS0108_O15
9	3.127	168	1	-0.380	0.093	5.856	1946	WS0108_O15
9	17.951	169	1	0.258	0.052	5.442	1946	WS0108_O15
11	17.088	203	1	-0.277	0.055	5.724	1946	WS0108_O15
11	27.405	204	1	0.322	0.041	4.368	1946	WS0108_O15
11	29.069	205	1	0.377	0.095	9.992	1946	WS0108_O15
11	45.989	216	1	-0.212	0.053	5.547	1946	WS0108_O15
14	0.000	246	1	0.247	0.042	4.421	1946	WS0108_O15
3	17.158	45	1	0.226	0.083	6.671	1947	WS0109_A08
3	47.928	49	1	0.172	0.057	4.539	1947	WS0109_A08
4	97.576	80	2	0.164	0.047	5.427	1947	WS0109_A08
9	2.011	167	4	0.257	0.042	4.215	1947	WS0109_A08
9	3.127	168	1	-0.422	0.112	5.135	1947	WS0109_A08
9	17.951	169	2	0.369	0.082	6.557	1947	WS0109_A08
9	28.608	170	2	-0.200	0.036	3.857	1947	WS0109_A08
9	32.356	171	2	-0.163	0.046	4.924	1947	WS0109_A08
1	22.419	4	3	0.289	0.106	4.627	1948	WS0109_A18
1	43.578	8	2	-0.239	0.041	5.031	1948	WS0109_A18
1	43.578	8	3	0.288	0.051	4.366	1948	WS0109_A18
1	123.316	20	4	0.249	0.057	4.200	1948	WS0109_A18
3	9.953	42	3	0.224	0.044	3.898	1948	WS0109_A18
3	47.928	49	1	0.249	0.098	6.931	1948	WS0109_A18
4	82.462	78	1	-0.364	0.135	9.625	1948	WS0109_A18
5	31.191	95	1	-0.189	0.062	4.381	1948	WS0109_A18
9	0.000	166	1	0.252	0.056	4.017	1948	WS0109_A18
11	40.960	210	2	0.373	0.055	3.930	1948	WS0109_A18
14	22.402	248	3	0.321	0.083	7.202	1948	WS0109_A18
14	43.392	250	1	-0.306	0.111	4.829	1948	WS0109_A18
2	75.550	31	2	-0.289	0.051	5.845	1949	WS00925_G06
2	127.147	40	3	-0.203	0.046	4.884	1949	WS00925_G06
4	75.958	77	3	-0.284	0.079	4.607	1949	WS00925_G06
6	95.249	126	1	0.329	0.121	5.708	1949	WS00925_G06
7	59.789	137	3	-0.235	0.068	7.123	1949	WS00925_G06
7	70.278	141	3	0.193	0.048	5.069	1949	WS00925_G06
8	11.974	150	1	-0.231	0.060	4.496	1949	WS00925_G06
10	78.479	193	3	-0.285	0.042	4.460	1949	WS00925_G06
10	86.151	195	3	0.224	0.052	5.447	1949	WS00925_G06
14	22.402	248	3	0.210	0.039	4.092	1949	WS00925_G06
5	84.347	105	1	-0.097	0.039	4.278	1950	WS00925_G15
6	25.742	113	3	-0.220	0.049	6.196	1950	WS00925_G15
6	95.249	126	1	0.438	0.101	7.045	1950	WS00925_G15
7	75.883	144	2	-0.599	0.343	9.282	1950	WS00925_G15
7	75.883	144	4	0.305	0.064	3.903	1950	WS00925_G15
11	27.405	204	1	0.326	0.043	4.820	1950	WS00925_G15
11	46.026	217	1	-0.357	0.200	21.676	1950	WS00925_G15
11	60.464	221	1	-0.210	0.049	5.348	1950	WS00925_G15

11	64.014	222	3	0.339	0.050	6.635	1950	WS00925_G15
12	51.382	242	1	0.444	0.142	5.788	1950	WS00925_G15
14	44.898	251	1	0.304	0.051	6.155	1950	WS00925_G15
2	101.076	35	3	0.198	0.035	4.022	1951	WS00925_I07
3	9.953	42	3	0.184	0.051	5.739	1951	WS00925_I07
5	31.191	95	1	-0.286	0.066	4.716	1951	WS00925_I07
6	95.249	126	1	0.394	0.109	5.770	1951	WS00925_I07
7	15.555	130	2	-0.298	0.043	5.358	1951	WS00925_I07
7	17.610	131	3	-0.276	0.066	4.045	1951	WS00925_I07
9	0.000	163	2	-0.471	0.073	4.247	1951	WS00925_I07
10	9.580	187	2	-0.189	0.081	4.630	1951	WS00925_I07
11	17.088	203	2	-0.216	0.068	6.879	1951	WS00925_I07
11	27.405	204	1	0.426	0.119	8.632	1951	WS00925_I07
11	29.069	205	1	0.279	0.065	4.529	1951	WS00925_I07
11	60.464	221	2	0.204	0.048	5.581	1951	WS00925_I07
2	127.147	40	3	-0.249	0.048	4.427	1952	WS00925_I20
4	97.576	80	2	0.169	0.042	4.897	1952	WS00925_I20
7	70.278	141	3	0.304	0.087	7.960	1952	WS00925_I20
13	0.000	243	4	0.457	0.154	7.066	1952	WS00925_I20
2	120.617	38	3	0.143	0.047	4.207	1953	WS00925_J20
2	127.147	40	3	-0.134	0.052	4.611	1953	WS00925_J20
4	55.925	76	1	-0.293	0.068	4.508	1953	WS00925_J20
5	31.191	95	1	-0.309	0.085	6.449	1953	WS00925_J20
6	87.891	124	2	-0.165	0.040	4.501	1953	WS00925_J20
6	95.249	126	1	0.464	0.140	7.307	1953	WS00925_J20
7	15.555	130	2	-0.270	0.043	5.140	1953	WS00925_J20
11	0.000	199	2	0.254	0.044	5.421	1953	WS00925_J20
11	0.000	199	3	-0.354	0.049	4.548	1953	WS00925_J20
11	27.405	204	1	0.473	0.139	10.920	1953	WS00925_J20
11	29.069	205	1	0.385	0.101	7.726	1953	WS00925_J20
11	60.464	221	2	0.242	0.061	6.209	1953	WS00925_J20
5	86.669	106	4	-0.386	0.076	4.034	1954	WS00925_K05
6	81.996	123	4	0.449	0.094	4.890	1954	WS00925_K05
9	0.000	166	1	-0.170	0.049	4.990	1954	WS00925_K05
11	45.989	216	1	-0.229	0.044	4.539	1954	WS00925_K05
3	80.211	55	4	0.227	0.045	4.874	1955	WS00925_L14
5	16.219	93	1	-0.291	0.049	4.107	1955	WS00925_L14
5	16.219	93	2	0.231	0.041	4.362	1955	WS00925_L14
5	54.072	100	4	0.194	0.036	3.853	1955	WS00925_L14
5	57.017	102	2	0.216	0.039	4.025	1955	WS00925_L14
5	57.017	102	4	-0.413	0.096	10.726	1955	WS00925_L14
6	25.914	114	1	-0.204	0.049	4.080	1955	WS00925_L14
7	73.169	142	1	0.640	0.152	6.559	1955	WS00925_L14
9	28.608	170	2	0.259	0.044	4.676	1955	WS00925_L14
11	0.000	199	2	0.289	0.088	9.411	1955	WS00925_L14
11	27.405	204	1	0.279	0.058	4.775	1955	WS00925_L14
11	60.464	221	1	0.291	0.068	5.623	1955	WS00925_L14
14	0.000	246	1	0.223	0.058	4.748	1955	WS00925_L14
14	44.898	251	1	0.254	0.058	4.820	1955	WS00925_L14
3	144.593	66	4	-0.148	0.042	4.015	1956	WS00925_L23
3	154.505	69	4	-0.202	0.059	5.350	1956	WS00925_L23
7	17.610	131	3	-0.340	0.115	6.722	1956	WS00925_L23
12	14.406	230	3	-0.252	0.038	3.884	1956	WS00925_L23
14	0.000	246	1	0.264	0.064	4.664	1956	WS00925_L23

2	126.181	39	4	-0.422	0.057	4.032	1957	WS01016_F11
6	89.401	125	1	-0.298	0.089	6.378	1957	WS01016_F11
7	17.610	131	3	-0.294	0.137	3.856	1957	WS01016_F11
9	28.608	170	2	-0.362	0.083	10.378	1957	WS01016_F11
9	28.608	170	3	0.348	0.069	4.967	1957	WS01016_F11
10	93.347	196	1	0.484	0.131	5.632	1957	WS01016_F11
10	104.214	198	2	-0.253	0.056	6.365	1957	WS01016_F11
11	27.405	204	1	0.420	0.113	8.352	1957	WS01016_F11
11	29.069	205	1	0.272	0.069	4.918	1957	WS01016_F11
11	45.989	216	1	-0.161	0.075	5.287	1957	WS01016_F11
12	14.406	230	3	-0.328	0.052	3.986	1957	WS01016_F11
12	6.242	234	2	-0.175	0.022	3.903	1957	WS01016_F11
2	102.814	36	1	0.290	0.048	4.764	1958	WS01016_K14
3	9.953	42	4	0.293	0.088	6.858	1958	WS01016_K14
3	14.500	43	2	0.231	0.073	5.175	1958	WS01016_K14
4	173.929	88	2	0.183	0.042	3.917	1958	WS01016_K14
5	56.008	101	2	-0.224	0.043	3.884	1958	WS01016_K14
8	50.001	154	1	0.267	0.072	6.813	1958	WS01016_K14
9	55.358	175	3	0.401	0.067	3.893	1958	WS01016_K14
10	93.347	196	1	-0.483	0.081	6.097	1958	WS01016_K14
11	35.604	207	3	-0.267	0.060	6.473	1958	WS01016_K14
11	37.143	209	3	0.238	0.047	5.134	1958	WS01016_K14
11	40.960	210	2	0.447	0.099	6.076	1958	WS01016_K14
11	42.600	213	3	-0.237	0.046	4.940	1958	WS01016_K14
11	48.979	218	2	0.276	0.081	7.616	1958	WS01016_K14
5	52.820	99	3	0.246	0.035	3.991	1959	WS0085_B03
6	49.100	118	2	0.304	0.114	4.576	1959	WS0085_B03
6	87.891	124	2	-0.176	0.046	4.242	1959	WS0085_B03
6	89.401	125	2	-0.220	0.046	3.951	1959	WS0085_B03
6	106.702	128	2	0.535	0.177	7.441	1959	WS0085_B03
10	35.204	189	1	-0.374	0.073	6.966	1959	WS0085_B03
11	40.960	210	2	-0.521	0.130	7.601	1959	WS0085_B03
11	48.979	218	2	-0.227	0.050	4.781	1959	WS0085_B03
11	64.014	222	3	-0.193	0.038	4.270	1959	WS0085_B03
1	15.589	3	2	-0.263	0.053	6.517	1960	WS0023_D24
2	75.550	31	1	-0.235	0.076	5.016	1960	WS0023_D24
3	95.157	59	2	0.212	0.033	4.010	1960	WS0023_D24
3	104.681	60	2	-0.183	0.043	5.263	1960	WS0023_D24
3	112.868	61	2	0.194	0.050	6.055	1960	WS0023_D24
3	114.747	62	2	0.262	0.047	5.814	1960	WS0023_D24
6	56.188	120	2	0.255	0.039	4.096	1960	WS0023_D24
8	39.090	152	4	0.259	0.059	5.511	1960	WS0023_D24
9	59.963	177	1	-0.267	0.061	3.983	1960	WS0023_D24
10	70.246	190	2	0.230	0.033	4.088	1960	WS0023_D24
4	116.928	82	2	0.274	0.055	6.570	1961	WS0085_H02
4	124.132	83	1	0.493	0.162	4.755	1961	WS0085_H02
4	124.132	83	2	0.348	0.135	9.423	1961	WS0085_H02
6	95.249	126	1	0.321	0.125	4.579	1961	WS0085_H02
7	62.787	139	2	-0.251	0.034	4.103	1961	WS0085_H02
9	36.227	165	3	-0.252	0.049	5.447	1961	WS0085_H02
1	82.301	16	3	-0.242	0.044	5.034	1962	WS0023_L10
2	127.147	40	3	-0.166	0.038	4.296	1962	WS0023_L10
8	95.937	159	3	0.243	0.040	4.653	1962	WS0023_L10
9	28.608	170	2	-0.235	0.029	3.958	1962	WS0023_L10

9	32.356	171	2	-0.244	0.039	5.396	1962	WS0023_L10
9	49.013	173	2	0.273	0.072	10.018	1962	WS0023_L10
11	35.604	207	3	-0.261	0.091	10.391	1962	WS0023_L10
11	37.143	209	3	0.240	0.079	9.003	1962	WS0023_L10
11	42.600	213	3	-0.202	0.054	6.153	1962	WS0023_L10
6	89.401	125	1	-0.245	0.051	4.390	1963	WS0105_P10
10	70.246	190	2	0.291	0.080	8.151	1963	WS0105_P10
11	64.014	222	3	-0.244	0.057	6.138	1963	WS0105_P10
4	75.958	77	3	-0.234	0.069	3.987	1964	WS0106_E17
9	0.000	166	2	0.186	0.049	5.327	1964	WS0106_E17
10	78.479	193	3	-0.330	0.067	7.210	1964	WS0106_E17
12	22.441	227	3	-0.344	0.080	7.977	1964	WS0106_E17
13	3.386	245	1	0.244	0.061	4.840	1964	WS0106_E17
14	22.402	248	3	-0.252	0.042	4.577	1964	WS0106_E17
1	82.301	16	4	0.231	0.049	4.133	1965	WS00923_L22
4	0.000	71	4	0.176	0.063	5.732	1965	WS00923_L22
7	75.883	144	2	0.571	0.176	8.785	1965	WS00923_L22
7	75.883	144	4	-0.363	0.060	5.670	1965	WS00923_L22
12	32.473	238	1	0.248	0.038	4.142	1965	WS00923_L22
12	51.382	242	1	-0.407	0.091	4.593	1965	WS00923_L22
1	43.578	8	2	-0.194	0.032	4.029	1966	WS00923_P06
11	60.464	221	2	0.251	0.042	5.387	1966	WS00923_P06
11	64.014	222	4	0.289	0.050	5.481	1966	WS00923_P06
4	140.962	84	3	-0.295	0.086	8.153	1967	WS0037_B09
4	142.917	85	1	-0.276	0.060	4.814	1967	WS0037_B09
6	106.702	128	3	-0.326	0.087	6.583	1967	WS0037_B09
7	15.555	130	3	-0.261	0.054	5.126	1967	WS0037_B09
7	17.610	131	3	-0.339	0.144	5.983	1967	WS0037_B09
7	105.477	147	2	-0.283	0.067	4.269	1967	WS0037_B09
8	39.090	152	4	0.212	0.046	4.293	1967	WS0037_B09
9	55.358	175	3	0.685	0.206	10.925	1967	WS0037_B09
10	21.864	188	1	-0.275	0.049	4.042	1967	WS0037_B09
1	82.301	16	3	-0.231	0.054	5.593	1968	WS0037_C11
2	93.504	32	4	0.287	0.057	4.636	1968	WS0037_C11
3	83.563	56	3	-0.221	0.069	7.226	1968	WS0037_C11
4	0.000	71	4	0.376	0.121	10.193	1968	WS0037_C11
5	86.669	106	4	-0.362	0.126	4.224	1968	WS0037_C11
9	59.963	177	4	-0.360	0.095	7.828	1968	WS0037_C11
11	0.000	199	3	-0.430	0.104	10.828	1968	WS0037_C11
11	27.405	204	1	0.327	0.062	6.397	1968	WS0037_C11
11	29.069	205	1	0.246	0.045	4.472	1968	WS0037_C11
14	46.637	252	4	0.276	0.057	4.958	1968	WS0037_C11
1	22.419	4	4	-0.441	0.112	3.904	1969	WS0037_F09
1	62.128	11	1	-0.533	0.111	5.496	1969	WS0037_F09
3	15.147	44	2	-0.231	0.087	5.255	1969	WS0037_F09
3	47.928	49	1	0.227	0.071	6.517	1969	WS0037_F09
3	148.745	68	1	-0.263	0.062	5.850	1969	WS0037_F09
7	67.154	140	4	-0.382	0.159	7.782	1969	WS0037_F09
7	73.169	142	1	0.508	0.083	4.122	1969	WS0037_F09
7	75.883	144	2	0.576	0.135	9.005	1969	WS0037_F09
7	75.883	144	4	-0.368	0.067	7.360	1969	WS0037_F09
10	93.347	196	2	0.455	0.128	4.502	1969	WS0037_F09
10	104.214	198	2	-0.294	0.069	6.622	1969	WS0037_F09
11	45.989	216	2	-0.169	0.044	3.875	1969	WS0037_F09

11	56.921	219	1	0.398	0.091	4.534	1969	WS0037_F09
12	0.000	233	1	0.275	0.059	5.421	1969	WS0037_F09
12	22.629	237	1	0.329	0.085	7.814	1969	WS0037_F09
12	32.473	238	1	0.300	0.068	6.304	1969	WS0037_F09
12	51.382	242	1	-0.446	0.144	7.128	1969	WS0037_F09
3	83.563	56	3	-0.217	0.054	4.992	1970	WS0037_G09
4	116.928	82	2	-0.258	0.049	6.638	1970	WS0037_G09
9	2.011	167	4	-0.240	0.040	3.931	1970	WS0037_G09
9	28.608	170	2	-0.221	0.032	4.427	1970	WS0037_G09
9	32.356	171	2	-0.217	0.030	4.050	1970	WS0037_G09
10	70.246	190	4	-0.200	0.043	3.963	1970	WS0037_G09
10	78.479	193	4	-0.209	0.069	6.042	1970	WS0037_G09
11	46.026	217	1	-0.182	0.095	4.994	1970	WS0037_G09
1	22.419	4	3	0.393	0.094	6.452	1971	WS0037_L10
1	40.464	7	3	0.198	0.047	4.815	1971	WS0037_L10
1	43.578	8	3	0.208	0.052	5.198	1971	WS0037_L10
1	45.794	9	3	-0.264	0.051	5.235	1971	WS0037_L10
6	87.891	124	2	-0.187	0.061	5.397	1971	WS0037_L10
6	95.249	126	1	0.303	0.058	3.966	1971	WS0037_L10
11	27.405	204	1	0.415	0.102	10.277	1971	WS0037_L10
11	27.405	204	4	-0.294	0.057	5.056	1971	WS0037_L10
11	29.069	205	1	0.219	0.043	4.266	1971	WS0037_L10
11	41.984	212	2	-0.228	0.047	4.240	1971	WS0037_L10
12	0.000	233	1	0.162	0.043	4.194	1971	WS0037_L10
7	17.610	131	3	-0.266	0.096	3.925	1972	WS0037_M05
10	9.580	187	2	-0.203	0.058	3.945	1972	WS0037_M05
11	0.000	199	3	-0.314	0.043	3.912	1972	WS0037_M05
11	17.088	203	1	-0.253	0.053	4.164	1972	WS0037_M05
11	27.405	204	1	0.414	0.110	8.944	1972	WS0037_M05
11	27.405	204	4	-0.293	0.061	5.644	1972	WS0037_M05
11	29.069	205	1	0.310	0.068	5.367	1972	WS0037_M05
11	57.661	220	4	0.240	0.065	5.802	1972	WS0037_M05
14	46.637	252	3	0.193	0.044	4.278	1972	WS0037_M05
2	120.617	38	3	0.276	0.050	4.697	1973	WS0037_P12
2	126.181	39	2	-0.417	0.106	11.450	1973	WS0037_P12
3	14.500	43	2	-0.182	0.041	4.274	1973	WS0037_P12
4	55.925	76	1	-0.293	0.120	9.185	1973	WS0037_P12
4	82.462	78	1	-0.310	0.121	10.040	1973	WS0037_P12
4	92.039	79	2	0.175	0.039	4.154	1973	WS0037_P12
4	97.576	80	2	-0.267	0.053	6.381	1973	WS0037_P12
6	69.073	121	3	0.296	0.084	7.777	1973	WS0037_P12
6	69.073	121	4	0.205	0.042	3.976	1973	WS0037_P12
9	36.227	165	4	-0.300	0.051	4.907	1973	WS0037_P12
9	17.951	169	2	-0.200	0.047	5.455	1973	WS0037_P12
9	28.608	170	2	0.220	0.048	5.233	1973	WS0037_P12
9	59.963	177	4	0.292	0.055	5.123	1973	WS0037_P12
1	40.464	7	3	0.216	0.073	6.822	1974	WS0038_A23
1	43.578	8	3	0.233	0.084	7.454	1974	WS0038_A23
1	45.794	9	3	-0.351	0.075	7.221	1974	WS0038_A23
4	142.917	85	3	-0.253	0.044	4.056	1974	WS0038_A23
6	87.891	124	2	-0.238	0.065	7.082	1974	WS0038_A23
7	62.787	139	2	-0.297	0.052	5.475	1974	WS0038_A23
8	87.463	158	2	-0.221	0.041	4.541	1974	WS0038_A23
10	93.347	196	1	0.442	0.100	4.684	1974	WS0038_A23

11	27.405	204	1	0.352	0.071	5.758	1974	WS0038_A23
11	43.491	214	4	-0.104	0.034	4.017	1974	WS0038_A23
2	127.147	40	3	-0.152	0.034	3.888	1975	WS00927_B12
8	50.001	154	1	0.256	0.058	5.317	1975	WS00927_B12
9	59.963	177	4	-0.335	0.114	8.583	1975	WS00927_B12
10	9.580	187	3	-0.380	0.084	4.540	1975	WS00927_B12
10	70.246	190	2	0.320	0.077	8.219	1975	WS00927_B12
1	29.800	5	2	-0.250	0.047	5.288	1976	WS00927_B20
1	43.578	8	2	-0.251	0.037	4.148	1976	WS00927_B20
1	45.794	9	1	0.340	0.088	6.839	1976	WS00927_B20
1	50.955	10	1	-0.288	0.073	5.699	1976	WS00927_B20
3	148.745	68	4	0.266	0.044	4.457	1976	WS00927_B20
6	54.066	119	4	-0.179	0.040	4.048	1976	WS00927_B20
8	11.974	150	1	-0.188	0.050	3.906	1976	WS00927_B20
2	127.147	40	3	-0.261	0.044	5.049	1977	WS00927_C24
3	95.157	59	2	0.186	0.034	4.111	1977	WS00927_C24
4	75.958	77	3	-0.223	0.069	4.299	1977	WS00927_C24
6	45.897	117	1	0.284	0.061	4.843	1977	WS00927_C24
8	50.001	154	1	0.200	0.052	4.670	1977	WS00927_C24
10	9.580	187	3	-0.249	0.091	5.010	1977	WS00927_C24
10	70.246	190	2	0.198	0.040	4.475	1977	WS00927_C24
11	42.600	213	3	-0.235	0.034	3.943	1977	WS00927_C24
11	43.491	214	3	0.226	0.034	3.851	1977	WS00927_C24
1	22.419	4	3	0.376	0.102	4.016	1978	WS00927_D07
2	120.617	38	3	0.268	0.111	8.187	1978	WS00927_D07
2	127.147	40	3	-0.238	0.120	8.920	1978	WS00927_D07
4	142.917	85	1	-0.228	0.042	4.252	1978	WS00927_D07
5	86.669	106	4	-0.546	0.180	11.238	1978	WS00927_D07
6	89.401	125	1	-0.358	0.070	7.193	1978	WS00927_D07
6	95.249	126	1	0.404	0.161	6.263	1978	WS00927_D07
7	67.154	140	4	-0.277	0.079	5.000	1978	WS00927_D07
7	75.883	144	2	0.674	0.207	12.058	1978	WS00927_D07
7	75.883	144	4	-0.315	0.058	6.404	1978	WS00927_D07
8	50.001	154	1	0.352	0.100	10.738	1978	WS00927_D07
9	59.963	177	4	-0.249	0.042	5.192	1978	WS00927_D07
10	70.246	190	2	0.267	0.047	4.132	1978	WS00927_D07
10	80.088	194	2	-0.222	0.051	4.597	1978	WS00927_D07
12	22.629	237	1	0.237	0.056	5.702	1978	WS00927_D07
12	51.382	242	1	-0.427	0.133	8.386	1978	WS00927_D07
2	127.147	40	3	-0.223	0.049	5.587	1979	WS00927_E18
5	52.820	99	3	0.261	0.048	4.205	1979	WS00927_E18
6	95.249	126	1	0.324	0.114	5.783	1979	WS00927_E18
8	62.580	155	1	0.383	0.184	7.441	1979	WS00927_E18
8	78.389	157	1	0.284	0.070	6.415	1979	WS00927_E18
8	87.463	158	1	-0.223	0.043	3.899	1979	WS00927_E18
9	69.046	181	2	0.281	0.074	4.457	1979	WS00927_E18
12	0.000	226	3	-0.206	0.038	4.309	1979	WS00927_E18
1	123.316	20	4	0.307	0.102	5.402	1980	WS00927_F02
2	120.617	38	3	0.200	0.039	4.014	1980	WS00927_F02
2	127.147	40	3	-0.220	0.061	6.205	1980	WS00927_F02
3	47.928	49	1	0.216	0.054	4.246	1980	WS00927_F02
3	95.157	59	2	0.188	0.039	4.017	1980	WS00927_F02
3	112.868	61	2	0.186	0.037	4.052	1980	WS00927_F02
8	50.001	154	1	0.228	0.055	4.689	1980	WS00927_F02

9	59.963	177	4	-0.272	0.075	6.667	1980	WS00927_F02
10	70.246	190	2	0.237	0.043	4.847	1980	WS00927_F02
11	27.405	204	1	0.343	0.070	5.479	1980	WS00927_F02
11	29.069	205	1	0.302	0.075	5.899	1980	WS00927_F02
11	64.014	222	3	-0.254	0.061	6.209	1980	WS00927_F02
2	120.617	38	3	0.206	0.039	4.191	1981	WS00927_G14
2	127.147	40	3	-0.173	0.044	4.712	1981	WS00927_G14
3	80.211	55	4	0.210	0.072	5.750	1981	WS00927_G14
6	89.401	125	1	-0.248	0.051	4.130	1981	WS00927_G14
6	95.249	126	1	0.328	0.084	4.519	1981	WS00927_G14
8	11.974	150	1	-0.248	0.051	4.171	1981	WS00927_G14
8	50.001	154	1	0.228	0.044	3.928	1981	WS00927_G14
10	78.479	193	3	-0.352	0.057	6.128	1981	WS00927_G14
11	17.088	203	2	-0.202	0.044	4.192	1981	WS00927_G14
11	35.604	207	3	-0.154	0.037	3.934	1981	WS00927_G14
11	64.014	222	3	-0.235	0.056	6.025	1981	WS00927_G14
1	43.578	8	3	0.190	0.034	4.130	1982	WS00927_G22
1	45.794	9	3	-0.246	0.047	5.552	1982	WS00927_G22
2	127.147	40	3	-0.195	0.037	4.325	1982	WS00927_G22
3	9.953	42	3	0.187	0.045	5.315	1982	WS00927_G22
3	95.157	59	2	0.217	0.052	6.356	1982	WS00927_G22
4	75.958	77	3	-0.261	0.066	5.363	1982	WS00927_G22
8	50.001	154	1	0.243	0.070	4.283	1982	WS00927_G22
9	49.013	173	2	0.217	0.040	4.701	1982	WS00927_G22
1	107.333	19	4	0.242	0.046	4.006	1983	WS0264_E17
1	141.021	25	2	0.218	0.037	5.126	1983	WS0264_E17
3	15.147	44	2	-0.294	0.097	6.716	1983	WS0264_E17
3	20.512	46	2	-0.246	0.035	4.966	1983	WS0264_E17
3	144.593	66	4	-0.239	0.047	5.068	1983	WS0264_E17
6	87.891	124	2	0.201	0.034	4.709	1983	WS0264_E17
9	49.013	173	1	-0.214	0.092	4.706	1983	WS0264_E17
11	27.405	204	1	-0.302	0.133	6.818	1983	WS0264_E17
11	27.405	204	4	0.322	0.064	5.970	1983	WS0264_E17
1	45.794	9	1	-0.360	0.103	8.662	1984	WS0264_F02
3	144.593	66	4	-0.179	0.029	4.307	1984	WS0264_F02
4	124.132	83	1	0.516	0.104	5.703	1984	WS0264_F02
9	59.963	177	4	-0.255	0.053	6.129	1984	WS0264_F02
10	78.479	193	4	-0.316	0.074	8.986	1984	WS0264_F02
13	1.815	244	4	0.289	0.043	4.018	1984	WS0264_F02
3	47.928	49	1	0.335	0.095	7.470	1985	WS0264_G10
5	31.191	95	1	-0.275	0.064	5.053	1985	WS0264_G10
9	0.000	163	1	-0.439	0.085	4.315	1985	WS0264_G10
9	59.963	177	4	-0.358	0.102	10.540	1985	WS0264_G10
1	107.333	19	4	0.242	0.074	6.119	1986	WS0264_H01
3	47.928	49	1	0.242	0.056	4.590	1986	WS0264_H01
3	144.593	66	4	-0.175	0.045	4.633	1986	WS0264_H01
3	154.505	69	4	-0.271	0.070	6.798	1986	WS0264_H01
9	3.127	168	1	-0.414	0.102	5.184	1986	WS0264_H01
10	9.580	187	2	-0.281	0.083	4.169	1986	WS0264_H01
10	80.088	194	2	-0.247	0.038	4.267	1986	WS0264_H01
11	29.069	205	1	0.283	0.057	4.761	1986	WS0264_H01
4	55.925	76	1	-0.248	0.061	4.908	1987	WS0264_I22
4	82.462	78	1	-0.310	0.059	5.431	1987	WS0264_I22
5	31.191	95	1	-0.197	0.045	3.954	1987	WS0264_I22

9	3.127	168	1	-0.387	0.085	4.157	1987	WS0264_I22
10	86.151	195	3	0.191	0.037	4.358	1987	WS0264_I22
11	17.088	203	1	-0.248	0.066	5.805	1987	WS0264_I22
11	29.069	205	1	0.247	0.050	4.453	1987	WS0264_I22
11	56.921	219	2	0.306	0.084	6.411	1987	WS0264_I22
2	127.147	40	3	-0.169	0.026	3.848	1988	WS0264_J05
4	43.146	74	3	-0.255	0.047	6.943	1988	WS0264_J05
6	56.188	120	2	0.229	0.026	4.117	1988	WS0264_J05
7	59.789	137	3	0.194	0.030	4.376	1988	WS0264_J05
9	69.046	181	2	0.290	0.052	6.685	1988	WS0264_J05
10	35.204	189	2	-0.189	0.028	3.920	1988	WS0264_J05
10	70.246	190	2	0.215	0.039	5.377	1988	WS0264_J05
11	56.921	219	2	0.280	0.065	7.405	1988	WS0264_J05
12	6.242	234	2	0.219	0.037	5.003	1988	WS0264_J05
3	47.928	49	1	0.242	0.065	5.414	1989	WS0264_K15
3	69.071	52	1	0.238	0.070	4.070	1989	WS0264_K15
4	140.962	84	3	0.267	0.069	7.940	1989	WS0264_K15
4	142.917	85	3	0.305	0.053	6.127	1989	WS0264_K15
6	54.066	119	4	-0.177	0.054	3.949	1989	WS0264_K15
8	62.580	155	1	0.370	0.164	4.081	1989	WS0264_K15
10	77.676	192	1	0.193	0.049	4.078	1989	WS0264_K15
10	80.088	194	1	0.234	0.054	4.506	1989	WS0264_K15
10	104.214	198	2	-0.244	0.043	5.511	1989	WS0264_K15
11	35.604	207	3	0.175	0.039	4.477	1989	WS0264_K15
11	37.143	209	3	-0.196	0.047	5.450	1989	WS0264_K15
11	40.960	210	2	0.554	0.160	8.652	1989	WS0264_K15
11	40.960	210	4	-0.279	0.062	4.452	1989	WS0264_K15
11	60.464	221	2	-0.240	0.053	5.707	1989	WS0264_K15
2	120.617	38	3	0.221	0.042	4.562	1990	WS0264_L07
2	127.147	40	3	-0.224	0.045	4.855	1990	WS0264_L07
3	14.500	43	1	-0.248	0.064	4.940	1990	WS0264_L07
7	15.555	130	3	-0.301	0.065	7.155	1990	WS0264_L07
11	56.921	219	2	0.267	0.080	4.019	1990	WS0264_L07
13	3.386	245	1	0.272	0.069	5.370	1990	WS0264_L07
1	22.419	4	3	0.350	0.058	4.553	1991	WS0058_K17
3	9.953	42	3	0.163	0.044	4.847	1991	WS0058_K17
6	95.249	126	1	0.310	0.054	4.149	1991	WS0058_K17
11	27.405	204	1	0.445	0.108	10.185	1991	WS0058_K17
11	27.405	204	4	-0.194	0.049	3.852	1991	WS0058_K17
11	29.069	205	1	0.295	0.066	6.248	1991	WS0058_K17
11	64.014	222	3	-0.188	0.043	4.730	1991	WS0058_K17
2	102.814	36	2	0.178	0.038	4.224	1992	WS01014_C08
2	127.147	40	3	-0.165	0.042	4.768	1992	WS01014_C08
4	82.462	78	2	-0.316	0.076	7.173	1992	WS01014_C08
4	97.576	80	2	0.234	0.037	4.480	1992	WS01014_C08
6	95.249	126	1	0.354	0.078	5.075	1992	WS01014_C08
7	15.555	130	2	-0.332	0.058	5.949	1992	WS01014_C08
10	9.580	187	2	-0.221	0.114	5.568	1992	WS01014_C08
11	27.405	204	1	0.538	0.181	17.035	1992	WS01014_C08
11	29.069	205	1	0.291	0.076	6.821	1992	WS01014_C08
11	35.604	207	3	-0.147	0.036	4.042	1992	WS01014_C08
11	42.600	213	3	-0.148	0.034	3.855	1992	WS01014_C08
2	48.839	29	4	0.243	0.040	4.697	1993	WS00924_B23
3	9.953	42	3	0.213	0.063	4.850	1993	WS00924_B23

3	144.647	67	1	0.297	0.065	4.541	1993	WS00924_B23
7	105.477	147	2	-0.275	0.130	5.126	1993	WS00924_B23
11	27.405	204	1	0.304	0.048	5.729	1993	WS00924_B23
11	57.661	220	4	0.251	0.055	6.143	1993	WS00924_B23
14	0.000	246	1	0.240	0.038	4.515	1993	WS00924_B23
14	44.898	251	1	0.316	0.055	6.508	1993	WS00924_B23
2	120.617	38	3	0.194	0.041	4.038	1994	WS00924_F10
2	127.147	40	3	-0.158	0.045	4.357	1994	WS00924_F10
3	47.928	49	1	0.196	0.045	4.254	1994	WS00924_F10
4	124.132	83	1	0.448	0.099	4.005	1994	WS00924_F10
6	89.401	125	1	-0.326	0.052	4.971	1994	WS00924_F10
8	50.001	154	1	0.227	0.056	5.838	1994	WS00924_F10
9	59.963	177	4	-0.284	0.070	6.556	1994	WS00924_F10
10	9.580	187	3	-0.360	0.095	5.116	1994	WS00924_F10
10	70.246	190	2	0.280	0.062	6.062	1994	WS00924_F10
10	78.479	193	3	-0.365	0.061	5.928	1994	WS00924_F10
11	64.014	222	3	-0.257	0.069	6.721	1994	WS00924_F10
3	14.500	43	2	0.206	0.036	3.869	1995	WS0083_F02
3	20.512	46	2	-0.211	0.042	4.323	1995	WS0083_F02
9	59.963	177	4	-0.456	0.156	14.913	1995	WS0083_F02
4	75.958	77	3	-0.422	0.132	9.733	1996	WS0083_F12
6	87.891	124	2	0.322	0.109	12.490	1996	WS0083_F12
6	89.401	125	2	0.313	0.084	9.727	1996	WS0083_F12
6	106.702	128	2	-0.415	0.059	4.363	1996	WS0083_F12
8	95.937	159	3	0.244	0.043	4.848	1996	WS0083_F12
9	59.963	177	4	-0.294	0.093	7.263	1996	WS0083_F12
11	42.600	213	3	-0.169	0.037	4.083	1996	WS0083_F12
1	95.572	17	3	0.248	0.035	4.107	1997	WS0083_H16
5	7.672	92	1	-0.285	0.050	5.550	1997	WS0083_H16
5	16.219	93	1	-0.269	0.080	8.530	1997	WS0083_H16
8	50.001	154	1	0.211	0.033	4.416	1997	WS0083_H16
9	69.046	181	2	0.279	0.086	4.623	1997	WS0083_H16
10	9.580	187	3	-0.274	0.061	4.821	1997	WS0083_H16
11	35.604	207	3	-0.203	0.035	4.920	1997	WS0083_H16
12	22.441	227	3	-0.250	0.045	5.782	1997	WS0083_H16
1	107.333	19	3	0.283	0.051	5.790	1998	WS0083_I11
2	120.617	38	3	0.206	0.055	6.182	1998	WS0083_I11
2	127.147	40	3	-0.236	0.083	9.201	1998	WS0083_I11
3	47.928	49	1	0.289	0.056	5.837	1998	WS0083_I11
4	0.000	71	4	-0.224	0.071	5.428	1998	WS0083_I11
5	52.820	99	3	0.336	0.076	8.448	1998	WS0083_I11
7	62.787	139	2	-0.238	0.059	4.701	1998	WS0083_I11
8	11.974	150	1	-0.268	0.042	4.381	1998	WS0083_I11
8	50.001	154	1	0.254	0.034	4.102	1998	WS0083_I11
9	0.000	163	1	-0.578	0.109	7.350	1998	WS0083_I11
9	59.963	177	4	-0.308	0.072	3.968	1998	WS0083_I11
11	0.000	199	3	-0.313	0.037	4.035	1998	WS0083_I11
11	64.014	222	3	-0.142	0.035	3.865	1998	WS0083_I11
12	14.406	230	4	-0.283	0.083	6.657	1998	WS0083_I11
14	22.402	248	3	0.202	0.055	6.036	1998	WS0083_I11
6	87.891	124	2	-0.184	0.040	4.364	1999	WS0083_L10
6	89.401	125	1	-0.389	0.094	7.493	1999	WS0083_L10
6	95.249	126	1	0.453	0.166	7.717	1999	WS0083_L10
7	75.883	144	2	0.422	0.071	5.011	1999	WS0083_L10

8	39.090	152	4	0.134	0.040	4.055	1999	WS0083_L10
9	0.000	166	2	0.284	0.058	6.161	1999	WS0083_L10
9	3.127	168	1	-0.448	0.150	4.869	1999	WS0083_L10
11	27.405	204	4	0.196	0.044	5.032	1999	WS0083_L10
1	69.854	13	2	-0.267	0.070	9.190	2000	WS0083_M02
1	71.592	14	2	-0.268	0.071	9.349	2000	WS0083_M02
1	77.177	15	2	-0.300	0.088	11.563	2000	WS0083_M02
1	95.572	17	2	-0.356	0.080	10.659	2000	WS0083_M02
2	63.261	30	3	0.305	0.088	9.260	2000	WS0083_M02
4	116.928	82	2	0.229	0.031	4.148	2000	WS0083_M02
9	2.011	167	4	0.279	0.079	6.515	2000	WS0083_M02
9	59.963	177	4	-0.269	0.075	6.229	2000	WS0083_M02
11	64.014	222	4	0.275	0.079	6.561	2000	WS0083_M02
14	44.898	251	3	0.235	0.051	4.617	2000	WS0083_M02
1	43.578	8	2	0.276	0.061	7.171	2001	WS0083_O23
1	95.572	17	2	-0.329	0.063	7.543	2001	WS0083_O23
4	43.146	74	3	0.287	0.082	7.043	2001	WS0083_O23
7	15.555	130	2	0.287	0.053	6.399	2001	WS0083_O23
9	17.951	169	2	0.258	0.035	4.197	2001	WS0083_O23
10	95.795	197	4	0.339	0.083	8.527	2001	WS0083_O23
11	64.014	222	4	0.298	0.049	5.358	2001	WS0083_O23
1	82.301	16	3	-0.248	0.042	4.268	2002	WS0083_P17
3	9.953	42	3	0.247	0.082	8.262	2002	WS0083_P17
3	20.512	46	2	-0.155	0.035	4.011	2002	WS0083_P17
4	142.917	85	1	-0.198	0.056	4.222	2002	WS0083_P17
5	42.979	97	1	0.334	0.053	4.023	2002	WS0083_P17
6	69.073	121	3	-0.235	0.041	4.097	2002	WS0083_P17
7	15.555	130	2	0.474	0.174	19.891	2002	WS0083_P17
7	15.555	130	3	-0.223	0.071	7.494	2002	WS0083_P17
7	17.610	131	3	-0.373	0.134	7.602	2002	WS0083_P17
7	62.787	139	2	-0.285	0.059	6.628	2002	WS0083_P17
10	80.088	194	2	-0.235	0.040	4.808	2002	WS0083_P17
11	29.069	205	1	0.297	0.064	4.961	2002	WS0083_P17
11	45.989	216	1	-0.186	0.056	4.139	2002	WS0083_P17
11	64.014	222	3	-0.168	0.041	4.117	2002	WS0083_P17
4	92.039	79	2	-0.196	0.046	4.627	2003	WS00925_C02
4	97.576	80	2	0.271	0.063	6.853	2003	WS00925_C02
4	124.132	83	2	0.408	0.105	7.122	2003	WS00925_C02
6	89.401	125	1	-0.321	0.074	6.511	2003	WS00925_C02
6	95.249	126	1	0.335	0.072	4.691	2003	WS00925_C02
7	70.278	141	3	0.181	0.033	4.096	2003	WS00925_C02
10	78.479	193	4	0.282	0.127	8.181	2003	WS00925_C02
11	27.405	204	1	0.363	0.070	6.209	2003	WS00925_C02
11	29.069	205	1	0.279	0.069	6.096	2003	WS00925_C02
11	41.984	212	2	-0.251	0.055	5.497	2003	WS00925_C02
11	64.014	222	3	-0.214	0.049	6.150	2003	WS00925_C02
12	22.441	227	3	-0.313	0.037	3.896	2003	WS00925_C02
1	43.578	8	2	-0.284	0.044	5.460	2004	WS00925_F03
2	93.504	32	2	-0.230	0.039	4.653	2004	WS00925_F03
2	95.880	34	3	0.280	0.049	5.110	2004	WS00925_F03
2	101.076	35	3	-0.249	0.040	4.160	2004	WS00925_F03
2	127.147	40	3	0.184	0.039	4.041	2004	WS00925_F03
3	137.891	64	3	-0.264	0.044	4.302	2004	WS00925_F03
5	0.000	89	3	-0.237	0.044	4.346	2004	WS00925_F03

5	57.017	102	4	-0.268	0.068	4.737	2004	WS00925_F03
6	56.188	120	1	0.309	0.110	7.238	2004	WS00925_F03
6	56.188	120	2	-0.259	0.052	6.355	2004	WS00925_F03
6	76.376	122	1	-0.381	0.180	4.209	2004	WS00925_F03
6	89.401	125	1	0.311	0.078	5.137	2004	WS00925_F03
6	106.702	128	3	-0.235	0.046	4.362	2004	WS00925_F03
9	55.358	175	3	0.421	0.101	4.323	2004	WS00925_F03
12	28.493	228	2	0.461	0.210	12.900	2004	WS00925_F03
4	124.132	83	1	-0.467	0.138	4.395	2005	WS0031_K15
6	26.350	115	2	-0.214	0.039	5.002	2005	WS0031_K15
6	106.702	128	2	-0.475	0.070	5.900	2005	WS0031_K15
7	15.555	130	3	-0.212	0.052	6.660	2005	WS0031_K15
7	74.560	143	1	-0.287	0.108	6.397	2005	WS0031_K15
10	70.246	190	2	0.290	0.066	8.198	2006	WS01019_E24
10	70.246	190	4	-0.262	0.067	5.491	2006	WS01019_E24
10	78.479	193	3	-0.179	0.035	3.854	2006	WS01019_E24
10	93.347	196	2	0.585	0.099	7.815	2006	WS01019_E24
12	6.242	234	2	0.188	0.040	5.720	2006	WS01019_E24
1	2.788	2	4	-0.194	0.041	4.301	2007	WS0032_K02
5	54.072	100	4	0.287	0.061	6.632	2007	WS0032_K02
9	2.011	167	4	0.234	0.060	6.445	2007	WS0032_K02
11	0.000	199	2	0.228	0.031	4.192	2007	WS0032_K02
11	27.405	204	1	0.350	0.106	6.217	2007	WS0032_K02
11	29.069	205	1	0.252	0.074	4.284	2007	WS0032_K02
14	22.402	248	4	0.229	0.036	3.887	2007	WS0032_K02
6	45.897	117	1	0.241	0.066	5.369	2008	WS01019_N10
7	75.883	144	4	-0.299	0.059	3.930	2008	WS01019_N10
11	0.000	199	3	0.231	0.046	6.824	2008	WS01019_N10
12	22.441	227	3	-0.319	0.055	5.725	2008	WS01019_N10
4	82.462	78	1	-0.226	0.051	5.021	2009	WS00810_H12
7	15.555	130	2	-0.235	0.048	4.259	2009	WS00810_H12
11	27.405	204	1	0.403	0.120	12.053	2009	WS00810_H12
11	29.069	205	1	0.191	0.045	4.496	2009	WS00810_H12
12	22.441	227	3	-0.231	0.056	5.453	2009	WS00810_H12
14	46.637	252	3	0.274	0.045	4.576	2009	WS00810_H12
1	82.301	16	3	-0.238	0.054	5.975	2010	WS00810_I10
10	21.864	188	1	-0.231	0.044	4.631	2010	WS00810_I10
11	0.000	199	3	-0.501	0.155	16.933	2010	WS00810_I10
11	27.405	204	1	0.395	0.104	11.115	2010	WS00810_I10
11	29.069	205	1	0.255	0.052	5.528	2010	WS00810_I10
11	45.989	216	1	-0.212	0.043	4.528	2010	WS00810_I10
12	22.629	237	1	0.207	0.037	3.897	2010	WS00810_I10
12	32.473	238	1	0.233	0.046	4.847	2010	WS00810_I10
14	0.000	246	1	0.283	0.065	6.821	2010	WS00810_I10
1	62.128	11	2	0.296	0.089	4.328	2011	WS00810_K17
1	127.805	21	3	-0.243	0.049	4.877	2011	WS00810_K17
2	101.076	35	4	-0.198	0.057	5.663	2011	WS00810_K17
3	95.157	59	2	-0.152	0.037	3.931	2011	WS00810_K17
4	0.000	71	4	0.209	0.058	5.197	2011	WS00810_K17
6	49.100	118	4	-0.389	0.161	5.073	2011	WS00810_K17
6	54.066	119	4	-0.178	0.049	4.396	2011	WS00810_K17
9	49.013	173	2	-0.174	0.039	4.103	2011	WS00810_K17
11	46.026	217	1	0.263	0.091	7.408	2011	WS00810_K17
12	38.543	232	4	-0.309	0.133	4.245	2011	WS00810_K17

1	2.788	2	4	-0.177	0.048	4.585	2012	WS00810_L08
1	40.464	7	3	0.230	0.057	5.194	2012	WS00810_L08
1	43.578	8	3	0.248	0.068	5.610	2012	WS00810_L08
2	120.617	38	3	0.219	0.061	5.569	2012	WS00810_L08
2	127.147	40	3	-0.246	0.086	7.847	2012	WS00810_L08
5	31.191	95	1	-0.240	0.040	3.927	2012	WS00810_L08
11	0.000	199	3	-0.319	0.065	4.287	2012	WS00810_L08
11	27.405	204	1	0.415	0.104	10.241	2012	WS00810_L08
11	29.069	205	1	0.229	0.045	4.360	2012	WS00810_L08
11	45.989	216	1	-0.160	0.040	3.884	2012	WS00810_L08
13	3.386	245	1	-0.308	0.099	9.582	2012	WS00810_L08
3	9.953	42	3	-0.217	0.034	4.392	2013	WS00810_N01
3	15.147	44	2	-0.268	0.117	3.998	2013	WS00810_N01
3	104.681	60	2	-0.218	0.062	4.483	2013	WS00810_N01
3	112.868	61	2	0.185	0.059	4.234	2013	WS00810_N01
3	114.747	62	2	0.223	0.063	4.501	2013	WS00810_N01
3	141.443	65	3	-0.259	0.061	5.533	2013	WS00810_N01
6	25.914	114	1	-0.247	0.040	4.635	2013	WS00810_N01
8	50.001	154	1	0.223	0.033	4.696	2013	WS00810_N01
10	78.479	193	4	-0.283	0.139	7.256	2013	WS00810_N01
3	95.157	59	2	0.181	0.038	3.879	2014	WS00810_N19
4	0.000	71	4	-0.222	0.048	3.955	2014	WS00810_N19
8	50.001	154	1	0.246	0.062	5.969	2014	WS00810_N19
9	59.963	177	4	-0.300	0.075	6.137	2014	WS00810_N19
10	9.580	187	3	-0.346	0.089	4.929	2014	WS00810_N19
10	70.246	190	2	0.313	0.069	7.385	2014	WS00810_N19
10	78.479	193	3	-0.317	0.043	4.548	2014	WS00810_N19
11	29.069	205	1	0.285	0.060	5.013	2014	WS00810_N19
11	64.014	222	3	-0.257	0.059	6.215	2014	WS00810_N19
4	0.000	71	4	-0.236	0.059	4.842	2015	WS00811_A08
7	74.560	143	1	-0.196	0.049	4.401	2015	WS00811_A08
8	50.001	154	1	0.268	0.060	6.356	2015	WS00811_A08
9	59.963	177	4	-0.288	0.067	5.631	2015	WS00811_A08
10	9.580	187	3	-0.359	0.084	5.072	2015	WS00811_A08
10	70.246	190	2	0.296	0.065	6.457	2015	WS00811_A08
11	27.405	204	1	0.303	0.043	4.030	2015	WS00811_A08
11	29.069	205	1	0.279	0.049	4.537	2015	WS00811_A08
11	43.491	214	3	0.161	0.044	4.638	2015	WS00811_A08
11	64.014	222	3	-0.292	0.083	8.805	2015	WS00811_A08
1	40.464	7	4	0.310	0.050	4.716	2016	WS00811_B14
2	93.504	32	2	-0.245	0.039	4.847	2016	WS00811_B14
4	75.958	77	3	-0.364	0.082	4.301	2016	WS00811_B14
5	52.820	99	1	0.269	0.059	4.483	2016	WS00811_B14
10	70.246	190	4	-0.309	0.057	3.854	2016	WS00811_B14
10	78.479	193	3	-0.384	0.054	4.789	2016	WS00811_B14
11	48.979	218	4	-0.180	0.039	4.498	2016	WS00811_B14
11	64.014	222	3	-0.210	0.047	4.092	2016	WS00811_B14
13	3.386	245	1	0.327	0.090	6.540	2016	WS00811_B14
14	0.000	246	2	0.208	0.042	4.716	2016	WS00811_B14
1	82.301	16	3	-0.202	0.045	5.020	2017	WS00930_B04
3	17.158	45	1	0.232	0.050	5.048	2017	WS00930_B04
3	47.928	49	1	0.241	0.056	5.670	2017	WS00930_B04
8	11.974	150	1	-0.275	0.061	6.189	2017	WS00930_B04
8	50.001	154	1	0.201	0.039	4.153	2017	WS00930_B04

9	59.963	177	4	-0.341	0.091	6.147	2017	WS00930_B04
10	78.479	193	3	-0.355	0.043	4.885	2017	WS00930_B04
11	27.405	204	1	0.287	0.048	4.818	2017	WS00930_B04
11	64.014	222	3	-0.311	0.082	9.203	2017	WS00930_B04
2	101.076	35	3	-0.223	0.035	4.035	2018	WS00930_B11
6	45.897	117	1	0.321	0.056	6.525	2018	WS00930_B11
6	49.100	118	4	0.404	0.239	5.537	2018	WS00930_B11
7	15.555	130	3	-0.404	0.098	10.280	2018	WS00930_B11
10	70.246	190	2	0.225	0.061	4.573	2018	WS00930_B11
10	86.151	195	3	-0.151	0.035	4.065	2018	WS00930_B11
14	9.923	247	1	-0.171	0.038	4.250	2018	WS00930_B11
2	120.617	38	3	0.160	0.041	4.404	2019	WS00930_C14
2	127.147	40	3	-0.137	0.042	4.443	2019	WS00930_C14
4	6.032	72	4	-0.144	0.053	4.273	2019	WS00930_C14
4	124.132	83	1	0.513	0.170	5.259	2019	WS00930_C14
6	89.401	125	1	-0.299	0.049	4.551	2019	WS00930_C14
6	95.249	126	1	0.395	0.095	5.702	2019	WS00930_C14
8	50.001	154	1	0.258	0.044	4.859	2019	WS00930_C14
11	0.000	199	3	-0.321	0.041	4.424	2019	WS00930_C14
11	27.405	204	1	0.347	0.056	5.287	2019	WS00930_C14
11	29.069	205	1	0.372	0.079	7.351	2019	WS00930_C14
11	45.989	216	1	-0.193	0.055	4.911	2019	WS00930_C14
1	43.578	8	3	0.205	0.045	4.511	2020	WS00930_C24
1	131.686	23	1	0.482	0.107	4.174	2020	WS00930_C24
3	15.147	44	2	-0.323	0.098	4.753	2020	WS00930_C24
3	47.928	49	1	0.315	0.087	6.958	2020	WS00930_C24
3	144.593	66	4	-0.219	0.064	6.239	2020	WS00930_C24
3	154.505	69	4	-0.211	0.053	4.181	2020	WS00930_C24
7	17.610	131	3	-0.320	0.077	4.667	2020	WS00930_C24
8	50.001	154	1	0.280	0.058	5.949	2020	WS00930_C24
9	55.358	175	1	-0.259	0.057	4.517	2020	WS00930_C24
9	55.358	175	3	0.414	0.102	4.514	2020	WS00930_C24
12	14.406	230	4	-0.398	0.103	8.705	2020	WS00930_C24
12	38.543	232	4	0.313	0.099	3.879	2020	WS00930_C24
12	22.629	237	1	0.264	0.103	8.236	2020	WS00930_C24
12	32.473	238	1	0.247	0.090	7.174	2020	WS00930_C24
3	148.745	68	1	0.289	0.057	4.306	2021	WS00930_E02
4	0.000	71	4	-0.158	0.040	4.029	2021	WS00930_E02
6	89.401	125	1	-0.364	0.109	7.882	2021	WS00930_E02
6	95.249	126	1	0.471	0.186	8.831	2021	WS00930_E02
9	59.963	177	4	-0.327	0.068	6.200	2021	WS00930_E02
11	64.014	222	3	-0.239	0.076	6.654	2021	WS00930_E02
1	131.686	23	1	0.556	0.154	5.281	2022	WS00930_E19
6	89.401	125	1	-0.226	0.050	4.875	2022	WS00930_E19
6	95.249	126	1	0.351	0.077	4.922	2022	WS00930_E19
9	49.013	173	2	0.162	0.050	4.736	2022	WS00930_E19
11	27.405	204	1	0.344	0.063	6.270	2022	WS00930_E19
11	64.014	222	3	-0.247	0.073	7.818	2022	WS00930_E19
1	50.955	10	1	-0.275	0.062	5.986	2023	WS00930_G02
3	69.071	52	1	-0.285	0.091	5.280	2023	WS00930_G02
8	78.389	157	1	0.250	0.059	5.642	2023	WS00930_G02
11	40.960	210	4	-0.357	0.121	6.668	2023	WS00930_G02
11	43.491	214	4	-0.183	0.060	3.916	2023	WS00930_G02
11	48.979	218	4	-0.319	0.094	5.320	2023	WS00930_G02

11	64.014	222	3	-0.269	0.057	6.499	2023	WS00930_G02
1	123.316	20	4	0.321	0.087	6.262	2024	WS00930_G11
3	69.071	52	1	0.340	0.077	4.765	2024	WS00930_G11
5	31.191	95	1	-0.364	0.125	9.879	2024	WS00930_G11
8	50.001	154	1	0.289	0.072	4.700	2024	WS00930_G11
9	36.227	165	4	-0.321	0.067	4.495	2024	WS00930_G11
10	70.246	190	2	0.269	0.055	6.019	2024	WS00930_G11
1	29.800	5	2	-0.273	0.033	4.462	2025	WS0084_E13
1	38.326	6	2	0.223	0.041	5.409	2025	WS0084_E13
1	127.805	21	3	-0.166	0.033	3.850	2025	WS0084_E13
4	92.039	79	2	0.158	0.029	3.873	2025	WS0084_E13
4	116.928	82	2	-0.243	0.035	4.635	2025	WS0084_E13
7	75.883	144	2	0.452	0.118	5.693	2025	WS0084_E13
7	75.883	144	4	-0.348	0.075	5.503	2025	WS0084_E13
8	50.001	154	2	0.303	0.082	9.427	2025	WS0084_E13
8	87.463	158	2	-0.264	0.059	7.778	2025	WS0084_E13
9	36.227	165	3	-0.305	0.040	4.726	2025	WS0084_E13
9	0.000	166	2	0.221	0.036	5.361	2025	WS0084_E13
11	17.088	203	2	-0.244	0.045	4.902	2025	WS0084_E13
11	29.069	205	2	-0.235	0.058	7.899	2025	WS0084_E13
11	41.984	212	2	-0.221	0.049	6.417	2025	WS0084_E13
11	56.921	219	2	0.405	0.096	8.299	2025	WS0084_E13
12	22.629	237	1	0.233	0.084	4.670	2025	WS0084_E13
1	43.578	8	2	-0.248	0.051	4.893	2026	WS0084_L01
1	43.578	8	3	0.267	0.042	5.030	2026	WS0084_L01
10	70.246	190	2	0.254	0.055	5.313	2026	WS0084_L01
1	107.333	19	3	-0.219	0.039	3.988	2027	WS01015_L18
4	0.000	71	4	0.220	0.095	8.257	2027	WS01015_L18
5	16.219	93	2	0.282	0.035	4.185	2027	WS01015_L18
7	70.278	141	3	0.250	0.054	5.472	2027	WS01015_L18
9	36.227	165	3	-0.239	0.046	4.680	2027	WS01015_L18
9	36.227	165	4	-0.234	0.051	4.699	2027	WS01015_L18
9	69.046	181	2	-0.277	0.108	6.101	2027	WS01015_L18
10	9.580	187	2	0.197	0.076	4.508	2027	WS01015_L18
10	104.214	198	4	-0.269	0.035	5.258	2027	WS01015_L18
11	46.026	217	1	-0.254	0.056	3.996	2027	WS01015_L18
13	0.000	243	4	-0.313	0.179	4.901	2027	WS01015_L18
1	22.419	4	3	0.497	0.142	9.764	2028	WS01015_P21
1	43.578	8	2	-0.179	0.060	5.224	2028	WS01015_P21
3	9.953	42	3	0.240	0.054	5.206	2028	WS01015_P21
6	87.891	124	2	-0.205	0.063	5.495	2028	WS01015_P21
6	95.249	126	1	0.332	0.066	4.546	2028	WS01015_P21
11	27.405	204	1	0.453	0.118	12.318	2028	WS01015_P21
11	29.069	205	1	0.355	0.092	9.313	2028	WS01015_P21
11	45.989	216	1	-0.213	0.059	5.897	2028	WS01015_P21
1	133.992	24	2	-0.156	0.034	4.723	2029	IS0011_G14
3	95.157	59	2	0.231	0.072	9.980	2029	IS0011_G14
3	104.681	60	2	-0.302	0.052	7.400	2029	IS0011_G14
3	112.868	61	2	0.336	0.081	11.520	2029	IS0011_G14
3	114.747	62	2	0.255	0.065	9.116	2029	IS0011_G14
3	137.891	64	3	0.355	0.087	7.430	2029	IS0011_G14
3	144.593	66	4	0.578	0.284	30.999	2029	IS0011_G14
3	148.745	68	4	-0.658	0.273	28.622	2029	IS0011_G14
3	154.505	69	4	0.536	0.164	16.921	2029	IS0011_G14

5	84.347	105	2	-0.251	0.040	5.910	2029	IS0011_G14
7	15.555	130	2	-0.203	0.028	4.158	2029	IS0011_G14
8	39.090	152	4	0.213	0.062	6.277	2029	IS0011_G14
9	2.011	167	4	-0.250	0.041	4.170	2029	IS0011_G14
9	28.608	170	2	-0.239	0.033	4.771	2029	IS0011_G14
9	32.356	171	2	-0.237	0.032	4.584	2029	IS0011_G14
9	49.013	173	2	0.320	0.105	15.260	2029	IS0011_G14
13	1.815	244	2	0.207	0.033	4.652	2029	IS0011_G14
1	107.333	19	4	0.267	0.066	6.265	2030	IS0012_A13
1	123.316	20	4	0.290	0.098	4.316	2030	IS0012_A13
3	144.593	66	4	-0.240	0.059	5.752	2030	IS0012_A13
3	148.745	68	4	0.223	0.051	4.677	2030	IS0012_A13
5	42.979	97	1	0.261	0.055	4.721	2030	IS0012_A13
8	78.389	157	1	-0.242	0.046	3.988	2030	IS0012_A13
9	61.113	178	1	-0.379	0.103	5.146	2030	IS0012_A13
10	95.795	197	4	0.243	0.055	5.191	2030	IS0012_A13
13	1.815	244	2	-0.248	0.040	4.088	2030	IS0012_A13
14	22.402	248	4	0.303	0.063	5.815	2030	IS0012_A13
14	46.637	252	4	0.360	0.091	8.786	2030	IS0012_A13
1	133.992	24	2	0.219	0.046	3.928	2031	WS0043_N10
2	95.880	34	1	0.244	0.040	4.158	2031	WS0043_N10
3	9.953	42	3	-0.217	0.045	4.088	2031	WS0043_N10
4	0.000	71	4	-0.293	0.107	10.376	2031	WS0043_N10
7	74.560	143	1	-0.250	0.041	4.306	2031	WS0043_N10
9	81.073	183	3	0.318	0.101	9.117	2031	WS0043_N10
10	9.580	187	3	-0.387	0.138	8.092	2031	WS0043_N10
12	14.406	230	4	-0.290	0.044	4.102	2031	WS0043_N10
2	75.550	31	2	0.297	0.058	5.652	2032	WS00927_J04
3	9.953	42	3	-0.343	0.100	10.218	2032	WS00927_J04
3	95.157	59	2	-0.208	0.041	4.297	2032	WS00927_J04
3	154.505	69	1	-0.256	0.062	5.028	2032	WS00927_J04
5	56.008	101	1	-0.239	0.057	4.618	2032	WS00927_J04
6	81.996	123	3	-0.371	0.131	7.624	2032	WS00927_J04
8	39.090	152	4	-0.244	0.061	5.303	2032	WS00927_J04
9	28.608	170	3	-0.217	0.047	4.319	2032	WS00927_J04
2	120.617	38	3	0.227	0.054	5.592	2033	WS0044_G14
3	9.953	42	3	0.180	0.038	3.956	2033	WS0044_G14
9	49.013	173	2	0.248	0.054	5.343	2033	WS0044_G14
10	78.479	193	3	-0.275	0.039	4.062	2033	WS0044_G14
11	29.069	205	1	0.276	0.053	4.706	2033	WS0044_G14
3	9.953	42	4	-0.186	0.053	4.593	2034	WS00927_M17
3	95.157	59	2	0.231	0.055	6.127	2034	WS00927_M17
3	104.681	60	2	-0.188	0.040	4.321	2034	WS00927_M17
3	112.868	61	2	0.204	0.040	4.275	2034	WS00927_M17
3	114.747	62	2	0.215	0.049	5.332	2034	WS00927_M17
4	0.000	71	4	-0.274	0.061	5.358	2034	WS00927_M17
4	124.132	83	1	0.564	0.178	6.346	2034	WS00927_M17
4	142.917	85	1	-0.245	0.054	4.335	2034	WS00927_M17
6	26.350	115	2	-0.249	0.056	6.055	2034	WS00927_M17
10	70.246	190	2	0.239	0.040	4.410	2034	WS00927_M17
11	43.491	214	3	0.221	0.066	6.623	2034	WS00927_M17
11	64.014	222	3	-0.273	0.062	6.263	2034	WS00927_M17
2	120.617	38	3	0.195	0.048	4.878	2035	WS00722_N08
2	127.147	40	3	-0.185	0.055	5.606	2035	WS00722_N08

3	14.500	43	2	0.212	0.033	4.035	2035	WS00722_N08
3	95.157	59	2	0.155	0.037	4.089	2035	WS00722_N08
4	0.000	71	4	-0.179	0.046	3.908	2035	WS00722_N08
5	86.669	106	2	0.295	0.073	4.079	2035	WS00722_N08
6	89.401	125	1	-0.323	0.088	6.824	2035	WS00722_N08
6	95.249	126	1	0.415	0.147	6.911	2035	WS00722_N08
8	11.974	150	1	-0.265	0.051	4.040	2035	WS00722_N08
9	49.013	173	2	0.168	0.035	3.948	2035	WS00722_N08
9	59.963	177	4	-0.326	0.078	6.660	2035	WS00722_N08
6	25.742	113	3	0.189	0.039	4.653	2036	WS00722_O21
11	17.088	203	1	-0.246	0.053	5.666	2036	WS00722_O21
11	27.405	204	1	0.287	0.042	4.440	2036	WS00722_O21
11	29.069	205	1	0.253	0.046	4.894	2036	WS00722_O21
11	43.491	214	3	0.176	0.035	4.208	2036	WS00722_O21
11	64.014	222	3	-0.242	0.043	5.204	2036	WS00722_O21
13	0.000	243	4	-0.304	0.119	4.287	2036	WS00722_O21
13	1.815	244	2	0.224	0.048	4.024	2036	WS00722_O21
13	1.815	244	4	-0.230	0.062	4.213	2036	WS00722_O21
1	15.589	3	2	0.250	0.065	6.499	2037	WS00723_C08
1	29.800	5	2	-0.222	0.049	4.755	2037	WS00723_C08
1	38.326	6	2	0.310	0.060	6.068	2037	WS00723_C08
1	82.301	16	3	-0.299	0.047	5.254	2037	WS00723_C08
6	95.249	126	1	0.291	0.078	3.940	2037	WS00723_C08
7	105.477	147	2	-0.335	0.142	5.618	2037	WS00723_C08
9	0.000	166	2	0.268	0.097	9.689	2037	WS00723_C08
9	17.951	169	2	-0.252	0.057	4.132	2037	WS00723_C08
11	64.014	222	3	-0.190	0.040	4.449	2037	WS00723_C08
13	0.000	243	4	-0.454	0.179	6.792	2037	WS00723_C08
2	101.076	35	4	0.218	0.026	3.972	2038	WS00723_E14
5	54.072	100	4	0.196	0.034	3.878	2038	WS00723_E14
6	25.914	114	1	-0.237	0.050	4.466	2038	WS00723_E14
6	26.350	115	2	-0.235	0.061	6.037	2038	WS00723_E14
6	54.066	119	4	-0.240	0.044	5.133	2038	WS00723_E14
6	56.188	120	1	0.210	0.066	5.846	2038	WS00723_E14
6	56.188	120	2	0.279	0.062	6.095	2038	WS00723_E14
6	69.073	121	4	0.301	0.070	7.980	2038	WS00723_E14
6	76.376	122	1	-0.362	0.106	5.608	2038	WS00723_E14
8	0.000	148	4	0.211	0.039	4.499	2038	WS00723_E14
8	39.090	152	4	-0.242	0.046	5.352	2038	WS00723_E14
9	49.013	173	2	-0.285	0.077	7.690	2038	WS00723_E14
10	70.246	190	2	0.204	0.041	4.058	2038	WS00723_E14
10	104.214	198	4	-0.345	0.059	5.640	2038	WS00723_E14
12	22.441	227	1	0.272	0.042	3.848	2038	WS00723_E14
3	47.928	49	1	0.287	0.052	6.739	2039	WS00723_J13
3	80.211	55	4	0.185	0.045	5.596	2039	WS00723_J13
4	0.000	71	4	-0.228	0.043	5.295	2039	WS00723_J13
4	103.719	81	4	-0.147	0.033	4.187	2039	WS00723_J13
4	124.132	83	1	0.503	0.094	8.515	2039	WS00723_J13
6	81.996	123	4	-0.296	0.067	5.930	2039	WS00723_J13
6	95.249	126	1	0.415	0.175	6.445	2039	WS00723_J13
11	27.405	204	1	0.331	0.034	4.627	2039	WS00723_J13
11	64.014	222	4	-0.275	0.047	3.935	2039	WS00723_J13
1	127.805	21	3	-0.260	0.052	4.888	2040	WS00723_L15
1	133.992	24	2	0.175	0.051	4.792	2040	WS00723_L15

2	120.617	38	3	-0.240	0.043	4.105	2040	WS00723_L15
2	127.147	40	3	0.248	0.050	4.751	2040	WS00723_L15
6	49.100	118	4	-0.273	0.138	5.283	2040	WS00723_L15
7	75.883	144	4	-0.358	0.057	4.702	2040	WS00723_L15
8	95.937	159	3	-0.168	0.048	4.433	2040	WS00723_L15
1	43.578	8	2	-0.216	0.047	5.705	2041	WS00934_B23
3	69.071	52	1	0.352	0.102	4.252	2041	WS00934_B23
3	154.505	69	4	-0.194	0.048	5.609	2041	WS00934_B23
6	95.249	126	1	0.349	0.098	4.059	2041	WS00934_B23
11	29.069	205	1	0.292	0.070	4.778	2041	WS00934_B23
2	120.617	38	3	-0.226	0.041	4.110	2042	WS00934_D12
3	154.505	69	1	0.245	0.061	4.952	2042	WS00934_D12
3	154.505	69	4	-0.244	0.053	4.768	2042	WS00934_D12
10	9.580	187	3	-0.314	0.099	4.845	2042	WS00934_D12
10	35.204	189	1	-0.357	0.108	8.630	2042	WS00934_D12
10	93.347	196	1	0.440	0.083	4.657	2042	WS00934_D12
11	64.014	222	3	-0.245	0.046	4.569	2042	WS00934_D12
12	6.242	234	4	0.456	0.111	9.845	2042	WS00934_D12
1	82.301	16	3	-0.279	0.047	5.391	2043	WS0039_G12
3	80.211	55	4	0.253	0.087	6.460	2043	WS0039_G12
5	57.017	102	2	-0.244	0.061	6.525	2043	WS0039_G12
6	95.249	126	1	0.310	0.087	4.555	2043	WS0039_G12
7	15.555	130	2	-0.375	0.085	9.009	2043	WS0039_G12
7	17.610	131	3	-0.323	0.099	6.051	2043	WS0039_G12
11	27.405	204	1	0.317	0.078	6.762	2043	WS0039_G12
11	27.405	204	4	-0.353	0.078	6.053	2043	WS0039_G12
11	64.014	222	3	-0.191	0.039	4.451	2043	WS0039_G12
2	120.617	38	3	0.230	0.043	5.122	2044	WS0039_H18
2	127.147	40	3	-0.278	0.068	8.139	2044	WS0039_H18
8	50.001	154	1	0.291	0.079	8.910	2044	WS0039_H18
8	62.580	155	1	0.388	0.167	8.176	2044	WS0039_H18
11	41.984	212	2	0.238	0.063	5.245	2044	WS0039_H18
11	45.989	216	2	0.233	0.057	4.453	2044	WS0039_H18
3	9.953	42	3	0.312	0.111	9.264	2045	WS0039_M05
4	75.958	77	3	-0.320	0.088	4.249	2045	WS0039_M05
9	49.013	173	2	0.206	0.036	4.353	2045	WS0039_M05
6	87.891	124	2	-0.198	0.047	4.892	2046	WS0039_P04
11	27.405	204	1	0.325	0.084	7.157	2046	WS0039_P04
2	127.147	40	3	0.240	0.054	5.721	2047	WS0055_D11
3	9.953	42	3	0.265	0.045	4.727	2047	WS0055_D11
3	9.953	42	4	0.163	0.051	4.190	2047	WS0055_D11
7	15.555	130	3	-0.564	0.193	20.199	2047	WS0055_D11
7	17.610	131	3	-0.539	0.288	16.675	2047	WS0055_D11
10	104.214	198	2	-0.276	0.066	6.999	2047	WS0055_D11
11	64.014	222	3	-0.286	0.066	6.946	2047	WS0055_D11
14	22.402	248	4	-0.338	0.065	4.891	2047	WS0055_D11
1	22.419	4	3	0.300	0.071	4.582	2048	WS0055_E10
1	40.464	7	3	0.268	0.062	6.994	2048	WS0055_E10
1	43.578	8	3	0.296	0.070	7.521	2048	WS0055_E10
1	45.794	9	3	-0.332	0.073	8.320	2048	WS0055_E10
3	9.953	42	3	0.225	0.039	4.322	2048	WS0055_E10
3	137.891	64	3	0.185	0.039	4.851	2048	WS0055_E10
5	7.672	92	1	-0.240	0.041	4.190	2048	WS0055_E10
8	95.937	159	3	-0.195	0.059	6.480	2048	WS0055_E10

9	3.127	168	1	-0.400	0.084	4.039	2048	WS0055_E10
12	0.000	233	1	0.257	0.046	4.753	2048	WS0055_E10
14	44.898	251	3	0.247	0.038	4.668	2048	WS0055_E10
7	15.555	130	2	-0.308	0.048	5.553	2049	WS0055_I18
10	21.864	188	1	-0.335	0.062	4.971	2049	WS0055_I18
11	17.088	203	2	-0.180	0.050	5.254	2049	WS0055_I18
11	27.405	204	1	0.369	0.099	8.059	2049	WS0055_I18
11	60.464	221	2	0.254	0.081	8.876	2049	WS0055_I18
1	43.578	8	2	-0.221	0.041	4.208	2050	WS0055_K11
3	15.147	44	2	0.317	0.087	5.957	2050	WS0055_K11
4	55.925	76	1	-0.274	0.073	5.652	2050	WS0055_K11
4	82.462	78	1	-0.319	0.095	8.261	2050	WS0055_K11
5	31.191	95	1	-0.218	0.053	4.553	2050	WS0055_K11
5	52.820	99	3	0.258	0.058	5.073	2050	WS0055_K11
7	15.555	130	2	-0.208	0.039	3.956	2050	WS0055_K11
8	87.463	158	2	-0.305	0.091	9.230	2050	WS0055_K11
10	9.580	187	3	0.298	0.089	3.940	2050	WS0055_K11
10	21.864	188	1	-0.254	0.056	4.892	2050	WS0055_K11
11	27.405	204	1	0.260	0.059	5.139	2050	WS0055_K11
11	56.921	219	2	0.301	0.135	3.976	2050	WS0055_K11
3	47.928	49	1	0.231	0.054	4.898	2051	WS0086_A04
3	144.593	66	4	-0.167	0.056	4.934	2051	WS0086_A04
3	154.505	69	4	-0.192	0.044	4.320	2051	WS0086_A04
6	69.073	121	3	-0.310	0.065	6.419	2051	WS0086_A04
7	75.883	144	2	0.416	0.096	5.231	2051	WS0086_A04
9	59.963	177	4	-0.397	0.129	11.655	2051	WS0086_A04
10	9.580	187	3	-0.381	0.082	4.336	2051	WS0086_A04
10	70.246	190	2	0.230	0.043	4.244	2051	WS0086_A04
11	60.464	221	2	0.183	0.036	3.885	2051	WS0086_A04
12	14.406	230	4	-0.172	0.045	4.324	2051	WS0086_A04
3	104.681	60	2	-0.201	0.043	4.389	2052	WS0086_H03
5	31.191	95	1	-0.201	0.048	4.167	2052	WS0086_H03
6	25.914	114	1	-0.246	0.063	5.441	2052	WS0086_H03
6	89.401	125	1	-0.275	0.051	4.486	2052	WS0086_H03
7	15.555	130	3	-0.293	0.076	5.578	2052	WS0086_H03
8	50.001	154	1	0.344	0.119	11.287	2052	WS0086_H03
9	59.963	177	4	-0.334	0.092	7.706	2052	WS0086_H03
10	70.246	190	2	0.277	0.053	5.348	2052	WS0086_H03
10	70.246	190	4	-0.307	0.062	5.755	2052	WS0086_H03
10	95.795	197	4	0.211	0.048	4.299	2052	WS0086_H03
12	22.441	227	1	0.285	0.044	3.904	2052	WS0086_H03
12	22.441	227	3	-0.222	0.045	4.513	2052	WS0086_H03
12	0.000	233	1	-0.217	0.054	4.710	2052	WS0086_H03
1	40.464	7	4	0.355	0.061	4.452	2053	IS0014_M21
5	31.191	95	1	-0.195	0.047	4.129	2053	IS0014_M21
6	69.073	121	4	-0.230	0.048	4.636	2053	IS0014_M21
9	59.963	177	4	-0.385	0.114	7.584	2053	IS0014_M21
10	78.479	193	4	-0.273	0.068	4.870	2053	IS0014_M21
11	27.405	204	1	0.275	0.045	4.038	2053	IS0014_M21
11	29.069	205	1	0.252	0.044	3.947	2053	IS0014_M21
3	47.928	49	1	0.242	0.047	4.308	2054	WS0041_E24
6	89.401	125	1	-0.308	0.056	5.182	2054	WS0041_E24
8	11.974	150	1	-0.260	0.050	4.652	2054	WS0041_E24
8	50.001	154	1	0.223	0.035	3.846	2054	WS0041_E24

9	59.963	177	4	-0.375	0.123	9.797	2054	WS0041_E24
10	70.246	190	2	0.282	0.058	5.532	2054	WS0041_E24
11	29.069	205	1	0.281	0.053	4.917	2054	WS0041_E24
11	64.014	222	3	-0.230	0.060	6.430	2054	WS0041_E24
2	95.880	34	1	0.425	0.076	7.072	2055	WS01039_M07
6	56.188	120	1	0.274	0.040	4.104	2055	WS01039_M07
6	87.891	124	2	0.254	0.062	5.851	2055	WS01039_M07
6	89.401	125	2	0.269	0.044	4.646	2055	WS01039_M07
7	15.555	130	3	-0.208	0.040	4.627	2055	WS01039_M07
8	62.580	155	1	0.498	0.111	5.122	2055	WS01039_M07
9	0.000	166	1	0.158	0.059	5.479	2055	WS01039_M07
9	28.608	170	3	-0.255	0.050	4.750	2055	WS01039_M07
12	6.242	234	4	0.230	0.025	3.943	2055	WS01039_M07
1	95.572	17	2	-0.260	0.051	5.481	2056	WS01039_N02
1	95.572	17	3	0.301	0.058	5.439	2056	WS01039_N02
2	75.550	31	2	0.188	0.026	3.859	2056	WS01039_N02
2	127.147	40	4	0.347	0.089	8.786	2056	WS01039_N02
3	95.157	59	2	-0.221	0.039	4.399	2056	WS01039_N02
4	55.925	76	1	0.185	0.054	4.009	2056	WS01039_N02
10	80.088	194	2	0.213	0.045	5.537	2056	WS01039_N02
11	45.989	216	1	0.238	0.051	4.156	2056	WS01039_N02
3	47.928	49	1	0.246	0.057	5.490	2057	WS00932_B05
6	95.249	126	1	0.415	0.135	8.151	2057	WS00932_B05
8	11.974	150	1	-0.262	0.055	5.347	2057	WS00932_B05
11	27.405	204	1	0.357	0.069	6.737	2057	WS00932_B05
11	29.069	205	1	0.308	0.068	6.619	2057	WS00932_B05
11	64.014	222	3	-0.283	0.074	7.441	2057	WS00932_B05
3	112.868	61	2	0.217	0.041	4.034	2058	WS00932_C15
5	52.820	99	3	0.284	0.053	6.000	2058	WS00932_C15
6	81.996	123	3	-0.362	0.065	4.136	2058	WS00932_C15
6	89.401	125	1	-0.297	0.089	7.935	2058	WS00932_C15
6	95.249	126	1	0.424	0.098	6.134	2058	WS00932_C15
7	59.789	137	3	-0.237	0.080	8.765	2058	WS00932_C15
7	62.787	139	4	0.249	0.066	5.134	2058	WS00932_C15
7	70.278	141	3	0.243	0.087	9.500	2058	WS00932_C15
7	74.560	143	1	-0.171	0.044	3.899	2058	WS00932_C15
7	75.883	144	4	0.283	0.054	4.169	2058	WS00932_C15
8	0.000	148	4	-0.199	0.074	5.746	2058	WS00932_C15
9	28.608	170	2	-0.250	0.038	3.992	2058	WS00932_C15
11	27.405	204	1	0.491	0.115	10.627	2058	WS00932_C15
11	29.069	205	1	0.319	0.072	6.483	2058	WS00932_C15
11	64.014	222	3	-0.139	0.038	4.169	2058	WS00932_C15
2	48.839	29	4	0.312	0.053	5.902	2059	WS00932_G03
3	47.928	49	1	0.212	0.064	4.323	2059	WS00932_G03
4	116.928	82	2	-0.256	0.033	3.927	2059	WS00932_G03
8	50.001	154	1	0.229	0.070	5.225	2059	WS00932_G03
9	36.227	165	4	-0.277	0.062	6.454	2059	WS00932_G03
10	9.580	187	2	-0.263	0.072	5.638	2059	WS00932_G03
11	29.069	205	2	-0.151	0.046	5.803	2059	WS00932_G03
11	41.984	212	2	-0.188	0.038	4.591	2059	WS00932_G03
11	56.921	219	2	0.349	0.093	4.022	2059	WS00932_G03
2	48.839	29	4	0.354	0.104	10.835	2060	WS00932_H02
3	17.158	45	1	0.221	0.074	5.891	2060	WS00932_H02
3	83.563	56	3	0.265	0.064	5.457	2060	WS00932_H02

4	55.925	76	1	-0.243	0.081	6.223	2060	WS00932_H02
7	73.169	142	1	0.584	0.162	5.472	2060	WS00932_H02
9	36.227	165	4	-0.184	0.037	4.166	2060	WS00932_H02
11	57.661	220	4	0.246	0.036	3.859	2060	WS00932_H02
1	43.578	8	2	0.250	0.033	3.893	2061	WS00932_K15
2	101.076	35	4	-0.286	0.057	4.993	2061	WS00932_K15
5	7.672	92	1	0.197	0.058	3.855	2061	WS00932_K15
5	54.072	100	4	0.196	0.056	5.415	2061	WS00932_K15
6	81.996	123	3	0.390	0.136	6.709	2061	WS00932_K15
8	0.000	148	4	-0.313	0.079	7.814	2061	WS00932_K15
1	62.128	11	1	0.470	0.074	4.217	2062	WS00932_L14
4	55.925	76	1	-0.184	0.045	5.214	2062	WS00932_L14
5	31.191	95	1	-0.257	0.083	9.631	2062	WS00932_L14
6	81.996	123	4	0.415	0.072	4.095	2062	WS00932_L14
9	36.227	165	3	-0.280	0.052	4.814	2062	WS00932_L14
3	9.953	42	4	-0.242	0.053	4.136	2063	WS0263_K21
3	144.647	67	1	-0.372	0.115	4.152	2063	WS0263_K21
6	49.100	118	2	0.350	0.099	6.841	2063	WS0263_K21
6	49.100	118	4	-0.462	0.174	6.294	2063	WS0263_K21
7	74.560	143	1	-0.206	0.053	3.994	2063	WS0263_K21
11	35.604	207	3	-0.232	0.060	6.463	2063	WS0263_K21
11	37.143	209	3	0.196	0.043	4.665	2063	WS0263_K21
12	6.242	234	2	-0.242	0.033	4.436	2063	WS0263_K21
13	3.386	245	1	0.245	0.062	4.675	2063	WS0263_K21
2	93.504	32	4	-0.231	0.051	5.147	2064	WS0263_L14
3	80.211	55	4	-0.225	0.040	4.060	2064	WS0263_L14
9	0.000	166	2	0.228	0.077	7.128	2064	WS0263_L14
10	104.214	198	2	0.249	0.064	5.773	2064	WS0263_L14
10	104.214	198	4	-0.333	0.051	5.611	2064	WS0263_L14
12	22.441	227	1	-0.312	0.058	5.846	2064	WS0263_L14
14	22.402	248	3	0.225	0.059	5.194	2064	WS0263_L14
14	46.637	252	4	0.375	0.077	8.037	2064	WS0263_L14
1	62.128	11	1	-0.698	0.157	10.941	2065	WS0263_N07
1	69.854	13	2	-0.228	0.071	6.471	2065	WS0263_N07
1	71.592	14	2	-0.196	0.054	4.921	2065	WS0263_N07
2	127.147	40	3	-0.186	0.054	4.069	2065	WS0263_N07
3	17.158	45	1	-0.211	0.040	3.869	2065	WS0263_N07
4	116.928	82	2	-0.295	0.079	7.392	2065	WS0263_N07
4	116.928	82	4	0.311	0.036	4.112	2065	WS0263_N07
4	173.929	88	2	0.262	0.081	7.306	2065	WS0263_N07
6	45.897	117	1	0.196	0.043	4.046	2065	WS0263_N07
6	76.376	122	1	-0.325	0.090	6.181	2065	WS0263_N07
7	70.278	141	3	-0.224	0.066	4.980	2065	WS0263_N07
8	50.001	154	1	0.239	0.051	5.153	2065	WS0263_N07
9	0.000	166	1	0.256	0.043	4.181	2065	WS0263_N07
9	17.951	169	1	-0.305	0.041	3.994	2065	WS0263_N07
10	21.864	188	1	-0.243	0.049	4.663	2065	WS0263_N07
10	104.214	198	4	-0.343	0.064	3.991	2065	WS0263_N07
11	41.984	212	2	0.190	0.045	4.069	2065	WS0263_N07
11	45.989	216	2	0.200	0.047	4.191	2065	WS0263_N07
4	82.462	78	1	-0.231	0.047	4.044	2066	WS0263_N17
5	0.000	89	3	-0.233	0.070	6.781	2066	WS0263_N17
1	2.788	2	4	-0.212	0.055	5.600	2067	WS0263_P14
1	22.419	4	3	0.253	0.085	4.017	2067	WS0263_P14

4	55.925	76	1	-0.248	0.064	4.661	2067	WS0263_P14
4	173.929	88	2	-0.179	0.034	3.890	2067	WS0263_P14
9	0.000	163	1	0.467	0.102	4.803	2067	WS0263_P14
11	0.000	199	2	0.284	0.064	7.520	2067	WS0263_P14
11	17.088	203	2	-0.204	0.038	4.190	2067	WS0263_P14
11	60.464	221	2	0.254	0.044	4.585	2067	WS0263_P14
12	6.242	234	2	-0.216	0.039	6.227	2067	WS0263_P14
3	9.953	42	3	0.207	0.069	5.521	2068	WS0264_A16
3	80.211	55	4	0.239	0.045	4.855	2068	WS0264_A16
3	137.891	64	3	0.268	0.050	4.024	2068	WS0264_A16
4	55.925	76	1	-0.261	0.056	4.044	2068	WS0264_A16
7	15.555	130	2	-0.217	0.045	5.096	2068	WS0264_A16
7	105.477	147	2	-0.271	0.064	4.851	2068	WS0264_A16
9	81.073	183	1	0.323	0.085	6.731	2068	WS0264_A16
9	81.073	183	3	-0.253	0.071	5.832	2068	WS0264_A16
11	0.000	199	2	0.248	0.054	6.060	2068	WS0264_A16
11	17.088	203	2	-0.182	0.040	3.902	2068	WS0264_A16
11	27.405	204	1	0.308	0.068	5.214	2068	WS0264_A16
11	27.405	204	4	-0.273	0.060	6.541	2068	WS0264_A16
12	6.242	234	2	-0.252	0.035	5.374	2068	WS0264_A16
4	124.132	83	1	0.488	0.167	4.795	2069	WS0264_C12
4	142.917	85	1	-0.281	0.071	5.384	2069	WS0264_C12
9	59.963	177	4	-0.356	0.109	8.678	2069	WS0264_C12
11	42.600	213	3	-0.155	0.041	4.242	2069	WS0264_C12
11	60.464	221	2	0.211	0.044	5.102	2069	WS0264_C12
11	64.014	222	3	-0.220	0.058	5.961	2069	WS0264_C12
2	120.617	38	3	0.199	0.048	4.360	2070	WS0264_D08
3	47.928	49	1	0.194	0.050	4.243	2070	WS0264_D08
5	31.191	95	1	-0.197	0.053	4.463	2070	WS0264_D08
6	106.702	128	2	0.410	0.095	4.492	2070	WS0264_D08
8	50.001	154	1	0.217	0.059	5.295	2070	WS0264_D08
9	55.358	175	1	0.370	0.064	5.564	2070	WS0264_D08
9	59.963	177	4	-0.363	0.102	10.253	2070	WS0264_D08
11	35.604	207	3	-0.125	0.044	4.030	2070	WS0264_D08
12	22.629	237	1	0.228	0.046	3.897	2070	WS0264_D08
1	95.572	17	3	0.294	0.052	3.900	2071	WS01020_B16
3	95.157	59	2	0.239	0.056	6.386	2071	WS01020_B16
3	104.681	60	2	-0.211	0.051	5.520	2071	WS01020_B16
3	112.868	61	2	0.263	0.060	6.473	2071	WS01020_B16
3	114.747	62	2	0.194	0.048	5.120	2071	WS01020_B16
4	0.000	71	4	-0.210	0.049	4.475	2071	WS01020_B16
8	50.001	154	1	0.198	0.050	4.487	2071	WS01020_B16
9	59.963	177	4	-0.269	0.055	5.135	2071	WS01020_B16
11	29.069	205	2	-0.177	0.042	4.242	2071	WS01020_B16
11	35.604	207	3	-0.227	0.055	5.331	2071	WS01020_B16
11	42.600	213	3	-0.260	0.066	6.467	2071	WS01020_B16
11	64.014	222	3	-0.269	0.050	4.883	2071	WS01020_B16
1	29.800	5	2	-0.253	0.050	4.442	2072	WS01020_D04
1	38.326	6	2	0.219	0.044	3.971	2072	WS01020_D04
3	47.928	49	1	0.261	0.059	5.882	2072	WS01020_D04
5	54.072	100	4	-0.174	0.049	4.645	2072	WS01020_D04
5	57.017	102	4	0.193	0.041	3.913	2072	WS01020_D04
6	89.401	125	1	-0.321	0.055	5.499	2072	WS01020_D04
8	11.974	150	1	-0.238	0.043	4.271	2072	WS01020_D04

9	59.963	177	4	-0.328	0.086	8.246	2072	WS01020_D04
10	78.479	193	3	-0.290	0.046	4.207	2072	WS01020_D04
11	29.069	205	1	0.277	0.048	4.790	2072	WS01020_D04
11	64.014	222	3	-0.208	0.054	4.976	2072	WS01020_D04
1	45.794	9	1	0.332	0.086	8.856	2073	WS01020_F16
1	127.805	21	4	0.213	0.042	3.862	2073	WS01020_F16
1	131.686	23	1	0.530	0.133	4.782	2073	WS01020_F16
7	59.789	137	3	-0.193	0.041	4.162	2073	WS01020_F16
7	70.278	141	3	0.206	0.049	5.013	2073	WS01020_F16
10	35.204	189	1	0.254	0.039	3.915	2073	WS01020_F16
12	22.629	237	1	-0.206	0.041	4.092	2073	WS01020_F16
12	32.473	238	1	-0.234	0.051	5.104	2073	WS01020_F16
14	46.637	252	4	-0.328	0.066	4.014	2073	WS01020_F16
2	93.504	32	4	0.201	0.051	5.507	2074	WS01020_G09
2	102.814	36	1	0.334	0.071	8.992	2074	WS01020_G09
6	56.188	120	2	0.321	0.065	4.919	2074	WS01020_G09
6	76.376	122	1	0.239	0.092	5.086	2074	WS01020_G09
8	11.974	150	1	-0.263	0.035	4.038	2074	WS01020_G09
1	2.788	2	3	0.231	0.048	5.500	2075	WS01020_I07
1	45.794	9	3	0.253	0.051	5.727	2075	WS01020_I07
5	57.017	102	2	-0.224	0.033	4.198	2075	WS01020_I07
6	89.401	125	1	-0.346	0.143	8.660	2075	WS01020_I07
6	106.702	128	2	-0.423	0.062	4.827	2075	WS01020_I07
7	70.278	141	3	0.189	0.047	5.325	2075	WS01020_I07
7	91.487	146	3	0.183	0.039	4.395	2075	WS01020_I07
10	78.479	193	3	-0.361	0.067	7.656	2075	WS01020_I07
10	86.151	195	3	0.179	0.036	4.052	2075	WS01020_I07
11	0.000	199	3	-0.259	0.036	4.248	2075	WS01020_I07
12	6.242	234	2	0.295	0.047	5.676	2075	WS01020_I07
1	45.794	9	1	0.260	0.075	5.190	2076	WS01020_J03
2	48.839	29	1	-0.269	0.065	4.529	2076	WS01020_J03
4	116.928	82	2	-0.316	0.054	6.497	2076	WS01020_J03
4	124.132	83	2	-0.340	0.103	5.172	2076	WS01020_J03
5	16.219	93	2	-0.285	0.040	4.907	2076	WS01020_J03
7	75.883	144	2	0.445	0.081	4.951	2076	WS01020_J03
9	32.356	171	2	-0.208	0.034	4.134	2076	WS01020_J03
10	9.580	187	2	-0.248	0.089	5.189	2076	WS01020_J03
1	123.316	20	4	0.285	0.091	5.304	2077	WS01020_L08
3	47.928	49	1	0.202	0.055	4.741	2077	WS01020_L08
9	0.000	166	1	0.246	0.047	4.139	2077	WS01020_L08
9	59.963	177	4	-0.210	0.047	3.889	2077	WS01020_L08
11	27.405	204	1	0.277	0.050	4.368	2077	WS01020_L08
11	29.069	205	1	0.237	0.053	4.654	2077	WS01020_L08
11	56.921	219	1	0.364	0.123	3.997	2077	WS01020_L08
1	2.788	2	3	0.264	0.063	5.765	2078	WS01020_M04
1	45.794	9	1	0.359	0.056	5.220	2078	WS01020_M04
1	45.794	9	3	-0.196	0.051	4.952	2078	WS01020_M04
1	82.301	16	4	-0.219	0.043	4.359	2078	WS01020_M04
2	75.550	31	1	0.285	0.042	3.982	2078	WS01020_M04
5	52.820	99	1	0.293	0.042	3.994	2078	WS01020_M04
5	52.820	99	3	-0.255	0.069	6.515	2078	WS01020_M04
9	0.000	166	2	0.182	0.050	4.111	2078	WS01020_M04
9	49.013	173	2	0.208	0.049	4.466	2078	WS01020_M04
12	22.629	237	1	0.206	0.048	4.452	2078	WS01020_M04

3	154.505	69	4	-0.215	0.048	5.198	2079	WS0041_M21
4	82.462	78	1	-0.390	0.105	8.260	2079	WS0041_M21
4	82.462	78	2	-0.401	0.172	18.973	2079	WS0041_M21
4	92.039	79	2	-0.441	0.161	16.199	2079	WS0041_M21
4	92.039	79	4	-0.313	0.066	5.798	2079	WS0041_M21
4	97.576	80	2	0.427	0.154	18.077	2079	WS0041_M21
4	103.719	81	4	-0.270	0.091	9.654	2079	WS0041_M21
6	69.073	121	3	-0.249	0.069	6.096	2079	WS0041_M21
6	89.401	125	1	-0.243	0.051	4.017	2079	WS0041_M21
8	39.090	152	4	0.237	0.065	6.445	2079	WS0041_M21
9	59.963	177	4	-0.279	0.057	5.992	2079	WS0041_M21
10	70.246	190	2	0.223	0.042	4.627	2079	WS0041_M21
11	27.405	204	1	0.386	0.087	6.948	2079	WS0041_M21
11	29.069	205	1	0.284	0.066	5.192	2079	WS0041_M21
11	57.661	220	4	0.206	0.039	3.933	2079	WS0041_M21
1	40.464	7	4	0.281	0.047	3.933	2080	WS0042_E16
1	82.301	16	3	-0.227	0.038	4.079	2080	WS0042_E16
4	75.958	77	3	-0.243	0.058	4.071	2080	WS0042_E16
6	56.188	120	2	0.214	0.030	4.193	2080	WS0042_E16
7	15.555	130	3	-0.209	0.057	5.740	2080	WS0042_E16
7	17.610	131	3	-0.257	0.059	4.143	2080	WS0042_E16
9	59.963	177	4	-0.348	0.107	7.192	2080	WS0042_E16
10	78.479	193	3	-0.287	0.044	4.703	2080	WS0042_E16
10	78.479	193	4	-0.273	0.079	6.319	2080	WS0042_E16
10	86.151	195	3	0.153	0.037	3.984	2080	WS0042_E16
11	35.604	207	3	-0.149	0.036	3.900	2080	WS0042_E16
11	37.143	209	3	0.167	0.044	4.706	2080	WS0042_E16
2	75.550	31	1	0.332	0.080	6.867	2081	WS00816_D14
3	9.953	42	3	0.200	0.042	4.307	2081	WS00816_D14
3	95.157	59	2	0.180	0.050	4.570	2081	WS00816_D14
3	137.891	64	3	0.235	0.056	5.739	2081	WS00816_D14
5	16.219	93	2	-0.290	0.044	4.092	2081	WS00816_D14
6	87.891	124	2	-0.207	0.057	5.820	2081	WS00816_D14
6	89.401	125	2	-0.371	0.080	6.757	2081	WS00816_D14
6	95.249	126	1	0.324	0.083	4.348	2081	WS00816_D14
7	17.610	131	3	-0.358	0.164	8.245	2081	WS00816_D14
9	49.013	173	2	0.220	0.057	4.327	2081	WS00816_D14
11	27.405	204	1	0.320	0.061	5.351	2081	WS00816_D14
11	43.491	214	4	-0.189	0.066	4.982	2081	WS00816_D14
11	64.014	222	4	-0.302	0.073	5.936	2081	WS00816_D14
3	148.745	68	4	0.192	0.034	3.855	2082	WS00816_J15
5	3.484	90	2	-0.300	0.097	5.645	2082	WS00816_J15
5	16.219	93	2	0.264	0.062	3.936	2082	WS00816_J15
7	15.555	130	2	-0.246	0.048	3.918	2082	WS00816_J15
8	39.090	152	4	-0.222	0.064	7.096	2082	WS00816_J15
9	17.951	169	1	0.368	0.074	7.826	2082	WS00816_J15
9	55.358	175	3	-0.462	0.105	5.628	2082	WS00816_J15
12	38.543	232	4	-0.268	0.103	5.599	2082	WS00816_J15
4	82.462	78	1	-0.259	0.059	5.429	2083	WS0262_M08
6	106.702	128	2	-0.507	0.141	6.244	2083	WS0262_M08
10	21.864	188	1	-0.324	0.054	4.854	2083	WS0262_M08
11	0.000	199	3	-0.354	0.073	7.980	2083	WS0262_M08
11	27.405	204	1	0.355	0.078	7.163	2083	WS0262_M08
3	15.147	44	2	-0.283	0.096	4.798	2084	WS0263_A09

3	47.928	49	1	0.191	0.042	4.036	2084	WS0263_A09
4	124.132	83	1	0.561	0.154	6.476	2084	WS0263_A09
4	142.917	85	1	-0.322	0.061	5.932	2084	WS0263_A09
5	54.072	100	4	-0.184	0.042	3.963	2084	WS0263_A09
5	57.017	102	4	0.271	0.062	5.052	2084	WS0263_A09
10	86.151	195	3	0.192	0.043	4.066	2084	WS0263_A09
12	28.493	228	2	0.272	0.087	4.497	2084	WS0263_A09
1	133.992	24	2	0.254	0.049	4.861	2085	WS0038_I11
1	141.021	25	2	-0.290	0.061	6.041	2085	WS0038_I11
2	63.261	30	3	-0.145	0.041	4.402	2085	WS0038_I11
2	93.504	32	4	0.306	0.068	5.471	2085	WS0038_I11
4	116.928	82	2	-0.267	0.045	4.393	2085	WS0038_I11
5	16.219	93	2	-0.336	0.054	5.056	2085	WS0038_I11
7	15.555	130	2	-0.277	0.038	3.942	2085	WS0038_I11
8	50.001	154	1	0.306	0.070	6.710	2085	WS0038_I11
9	58.045	176	1	0.228	0.095	4.916	2085	WS0038_I11
9	69.046	181	1	-0.362	0.085	4.489	2085	WS0038_I11
10	70.246	190	2	0.275	0.040	3.915	2085	WS0038_I11
11	0.000	199	3	-0.282	0.045	5.672	2085	WS0038_I11
13	1.815	244	2	-0.197	0.040	3.894	2085	WS0038_I11
13	3.386	245	1	0.273	0.042	4.010	2085	WS0038_I11
1	38.326	6	2	0.249	0.050	4.226	2086	WS0039_C04
2	101.076	35	3	0.260	0.040	4.015	2086	WS0039_C04
2	120.617	38	3	0.177	0.041	4.061	2086	WS0039_C04
2	126.181	39	2	-0.250	0.059	5.077	2086	WS0039_C04
2	127.147	40	3	-0.216	0.053	5.204	2086	WS0039_C04
3	47.928	49	1	0.276	0.082	8.470	2086	WS0039_C04
3	95.157	59	2	0.221	0.056	4.692	2086	WS0039_C04
3	154.505	69	1	0.250	0.049	5.128	2086	WS0039_C04
4	0.000	71	4	-0.338	0.077	6.997	2086	WS0039_C04
6	45.897	117	1	0.255	0.053	5.538	2086	WS0039_C04
9	0.000	166	2	0.171	0.049	4.262	2086	WS0039_C04
9	17.951	169	1	0.212	0.047	4.785	2086	WS0039_C04
10	104.214	198	2	0.262	0.063	5.727	2086	WS0039_C04
10	104.214	198	4	-0.375	0.078	7.197	2086	WS0039_C04
12	38.543	232	4	0.223	0.082	3.973	2086	WS0039_C04
1	2.788	2	4	0.237	0.045	4.496	2087	WS0046_D01
1	95.572	17	3	0.331	0.081	5.554	2087	WS0046_D01
3	137.891	64	3	-0.282	0.077	7.649	2087	WS0046_D01
7	91.487	146	3	0.240	0.070	6.952	2087	WS0046_D01
8	95.937	159	3	0.203	0.061	6.057	2087	WS0046_D01
11	0.000	199	3	-0.302	0.060	5.389	2087	WS0046_D01
2	95.880	34	1	0.296	0.040	3.919	2088	WS0046_E20
2	102.814	36	1	0.252	0.050	5.459	2088	WS0046_E20
2	120.617	38	3	0.204	0.033	4.054	2089	WS0046_I14
4	75.958	77	3	-0.365	0.098	8.183	2089	WS0046_I14
6	95.249	126	1	0.314	0.106	4.037	2089	WS0046_I14
9	49.013	173	2	0.218	0.038	4.804	2089	WS0046_I14
10	70.246	190	2	0.249	0.039	5.078	2089	WS0046_I14
10	78.479	193	3	-0.249	0.035	4.222	2089	WS0046_I14
11	27.405	204	1	0.286	0.067	3.906	2089	WS0046_I14
11	29.069	205	1	0.263	0.074	4.307	2089	WS0046_I14
1	45.794	9	3	-0.265	0.054	4.492	2090	WS0046_J19
3	104.681	60	2	-0.242	0.033	4.339	2090	WS0046_J19

3	112.868	61	2	0.227	0.035	4.521	2090	WS0046_J19
3	137.891	64	3	0.262	0.047	4.484	2090	WS0046_J19
5	0.000	89	3	-0.239	0.048	4.146	2090	WS0046_J19
5	16.219	93	2	-0.295	0.040	4.996	2090	WS0046_J19
7	17.610	131	3	-0.341	0.090	5.580	2090	WS0046_J19
9	28.608	170	2	-0.227	0.029	3.902	2090	WS0046_J19
9	49.013	173	2	0.217	0.061	6.289	2090	WS0046_J19
1	29.800	5	1	-0.150	0.040	3.978	2091	WS0046_N18
1	29.800	5	2	-0.217	0.050	4.238	2091	WS0046_N18
1	82.301	16	4	0.384	0.070	4.594	2091	WS0046_N18
3	144.647	67	1	-0.424	0.125	5.179	2091	WS0046_N18
4	82.462	78	1	-0.176	0.067	6.758	2091	WS0046_N18
4	103.719	81	4	-0.337	0.067	6.279	2091	WS0046_N18
4	116.928	82	4	0.334	0.064	5.562	2091	WS0046_N18
4	124.132	83	1	0.481	0.113	4.650	2091	WS0046_N18
9	28.608	170	3	-0.337	0.060	4.963	2091	WS0046_N18
13	3.386	245	1	0.170	0.064	6.418	2091	WS0046_N18
1	123.316	20	4	0.241	0.082	4.855	2092	WS0046_O11
1	131.686	23	1	0.480	0.102	3.914	2092	WS0046_O11
3	15.147	44	2	-0.218	0.070	4.410	2092	WS0046_O11
3	83.563	56	3	0.175	0.053	4.817	2092	WS0046_O11
4	116.928	82	4	0.242	0.043	4.250	2092	WS0046_O11
7	74.560	143	1	-0.300	0.056	4.869	2092	WS0046_O11
11	17.088	203	1	0.238	0.053	4.638	2092	WS0046_O11
11	56.921	219	1	0.262	0.109	4.178	2092	WS0046_O11
13	3.386	245	1	0.160	0.048	4.105	2092	WS0046_O11
14	0.000	246	1	-0.315	0.090	7.802	2092	WS0046_O11
3	95.157	59	2	0.211	0.048	5.170	2093	WS0047_B13
3	112.868	61	2	0.269	0.043	5.148	2093	WS0047_B13
6	95.249	126	1	0.373	0.121	5.611	2093	WS0047_B13
10	104.214	198	4	-0.326	0.061	6.098	2093	WS0047_B13
11	27.405	204	1	0.336	0.082	6.107	2093	WS0047_B13
11	29.069	205	1	0.302	0.076	5.730	2093	WS0047_B13
3	47.928	49	1	0.233	0.060	5.510	2094	WS0047_B21
4	124.132	83	1	0.437	0.116	3.851	2094	WS0047_B21
4	142.917	85	1	-0.250	0.048	4.391	2094	WS0047_B21
6	95.249	126	1	0.344	0.109	6.352	2094	WS0047_B21
8	50.001	154	1	0.187	0.037	4.223	2094	WS0047_B21
9	3.127	168	1	-0.374	0.112	4.576	2094	WS0047_B21
9	59.963	177	4	-0.255	0.084	6.368	2094	WS0047_B21
3	148.745	68	1	0.233	0.030	3.898	2095	WS0071_D13
4	43.146	74	3	-0.258	0.052	6.527	2095	WS0071_D13
4	142.917	85	1	-0.293	0.050	6.398	2095	WS0071_D13
7	74.560	143	1	-0.245	0.034	4.411	2095	WS0071_D13
10	35.204	189	1	-0.267	0.047	6.151	2095	WS0071_D13
11	44.884	215	3	-0.232	0.041	5.166	2095	WS0071_D13
11	64.014	222	3	-0.253	0.052	6.511	2095	WS0071_D13
1	62.128	11	1	-0.579	0.169	6.675	2096	WS0071_E03
3	148.745	68	4	-0.161	0.047	3.913	2096	WS0071_E03
4	55.925	76	1	-0.268	0.060	4.882	2096	WS0071_E03
4	82.462	78	1	-0.224	0.069	6.004	2096	WS0071_E03
5	52.820	99	1	-0.254	0.045	4.085	2096	WS0071_E03
6	45.897	117	1	0.275	0.068	6.075	2096	WS0071_E03
6	87.891	124	2	0.269	0.064	6.505	2096	WS0071_E03

6	89.401	125	2	0.278	0.054	5.583	2096	WS0071_E03
7	15.555	130	3	-0.230	0.050	4.237	2096	WS0071_E03
7	59.789	137	3	-0.263	0.054	5.587	2096	WS0071_E03
9	81.073	183	1	0.241	0.046	3.973	2096	WS0071_E03
1	82.301	16	3	-0.276	0.040	6.054	2097	WS0071_G19
3	9.953	42	3	0.208	0.039	5.862	2097	WS0071_G19
3	95.157	59	2	0.209	0.049	5.110	2097	WS0071_G19
3	112.868	61	2	0.190	0.037	4.294	2097	WS0071_G19
5	57.017	102	2	-0.234	0.046	5.250	2097	WS0071_G19
7	17.610	131	3	-0.246	0.050	4.683	2097	WS0071_G19
1	95.572	17	2	0.284	0.045	3.891	2098	WS0071_H08
6	95.249	126	1	0.428	0.083	7.231	2098	WS0071_H08
11	27.405	204	1	0.468	0.104	11.650	2098	WS0071_H08
11	29.069	205	1	0.367	0.074	8.341	2098	WS0071_H08
11	45.989	216	1	-0.211	0.060	6.535	2098	WS0071_H08
12	28.493	228	2	-0.224	0.114	6.086	2098	WS0071_H08
1	43.578	8	2	-0.237	0.060	3.979	2099	WS0071_K07
1	43.578	8	3	0.299	0.063	6.000	2099	WS0071_K07
3	154.505	69	1	0.200	0.034	4.220	2099	WS0071_K07
4	82.462	78	1	-0.334	0.058	7.333	2099	WS0071_K07
6	95.249	126	1	0.260	0.066	4.453	2099	WS0071_K07
7	73.169	142	1	0.598	0.126	7.057	2099	WS0071_K07
9	17.951	169	1	-0.325	0.031	3.925	2099	WS0071_K07
11	0.000	199	3	-0.325	0.082	6.769	2099	WS0071_K07
11	27.405	204	1	0.398	0.088	10.998	2099	WS0071_K07
11	29.069	205	1	0.335	0.063	7.877	2099	WS0071_K07
11	45.989	216	1	-0.268	0.039	4.893	2099	WS0071_K07
1	95.572	17	2	0.261	0.050	4.555	2100	WS0071_K24
4	55.925	76	1	-0.247	0.060	5.476	2100	WS0071_K24
6	69.073	121	3	-0.326	0.048	5.318	2100	WS0071_K24
6	87.891	124	2	-0.216	0.046	4.089	2100	WS0071_K24
6	89.401	125	1	-0.327	0.051	5.223	2100	WS0071_K24
6	89.401	125	2	-0.235	0.043	3.902	2100	WS0071_K24
6	95.249	126	1	0.434	0.119	8.343	2100	WS0071_K24
11	0.000	199	3	-0.302	0.051	4.599	2100	WS0071_K24
11	17.088	203	1	-0.227	0.058	5.726	2100	WS0071_K24
11	27.405	204	1	0.526	0.153	15.726	2100	WS0071_K24
11	27.405	204	4	-0.266	0.050	4.215	2100	WS0071_K24
11	29.069	205	1	0.483	0.144	14.547	2100	WS0071_K24
11	45.989	216	1	-0.342	0.105	10.484	2100	WS0071_K24
12	28.493	228	2	-0.210	0.079	3.971	2100	WS0071_K24
3	9.953	42	3	0.232	0.055	5.372	2101	WS0071_N11
3	15.147	44	2	0.241	0.070	4.450	2101	WS0071_N11
3	83.563	56	3	-0.204	0.058	5.669	2101	WS0071_N11
6	95.249	126	1	0.296	0.057	3.955	2101	WS0071_N11
7	74.560	143	1	0.271	0.042	4.063	2101	WS0071_N11
8	11.974	150	1	-0.247	0.048	4.628	2101	WS0071_N11
10	9.580	187	2	-0.261	0.072	4.608	2101	WS0071_N11
11	0.000	199	3	-0.342	0.070	5.676	2101	WS0071_N11
11	27.405	204	1	0.387	0.097	9.478	2101	WS0071_N11
11	29.069	205	1	0.299	0.069	6.580	2101	WS0071_N11
11	45.989	216	1	-0.195	0.042	3.984	2101	WS0071_N11
14	0.000	246	1	0.287	0.067	6.445	2101	WS0071_N11
3	9.953	42	3	0.235	0.064	5.224	2102	WS0071_O05

4	0.000	71	4	-0.222	0.044	4.698	2102	WS0071_O05
6	95.249	126	1	0.406	0.129	7.191	2102	WS0071_O05
7	73.169	142	2	0.502	0.074	6.526	2102	WS0071_O05
7	75.883	144	2	-0.442	0.063	5.475	2102	WS0071_O05
9	59.963	177	4	0.202	0.039	4.053	2102	WS0071_O05
2	75.550	31	1	0.204	0.040	3.892	2103	WS0072_A12
4	173.929	88	2	-0.148	0.046	4.215	2103	WS0072_A12
7	59.789	137	3	-0.192	0.040	4.499	2103	WS0072_A12
8	39.090	152	4	0.216	0.053	3.982	2103	WS0072_A12
11	27.405	204	1	0.517	0.134	12.973	2103	WS0072_A12
11	27.405	204	4	-0.212	0.050	4.168	2103	WS0072_A12
11	29.069	205	1	0.314	0.088	8.519	2103	WS0072_A12
11	35.604	207	3	-0.192	0.038	4.267	2103	WS0072_A12
11	45.989	216	1	-0.205	0.069	6.607	2103	WS0072_A12
11	64.014	222	3	-0.176	0.039	4.332	2103	WS0072_A12
12	6.242	234	2	-0.295	0.045	5.974	2103	WS0072_A12
1	95.572	17	2	0.301	0.059	4.819	2104	WS0072_B18
4	55.925	76	1	-0.281	0.052	5.367	2104	WS0072_B18
4	142.917	85	3	-0.235	0.035	3.847	2104	WS0072_B18
11	27.405	204	1	0.424	0.111	12.241	2104	WS0072_B18
11	27.405	204	4	-0.303	0.051	4.131	2104	WS0072_B18
11	44.884	215	3	-0.238	0.047	5.139	2104	WS0072_B18
11	64.014	222	3	-0.269	0.066	7.171	2104	WS0072_B18
12	0.000	233	1	0.216	0.057	6.077	2104	WS0072_B18
12	6.242	234	2	-0.159	0.041	5.068	2104	WS0072_B18
1	29.800	5	2	-0.257	0.045	4.332	2105	WS0072_E02
1	38.326	6	2	0.207	0.040	4.190	2105	WS0072_E02
3	95.157	59	2	0.212	0.049	4.983	2105	WS0072_E02
6	69.073	121	3	-0.251	0.038	3.945	2105	WS0072_E02
7	75.883	144	2	0.394	0.072	4.113	2105	WS0072_E02
9	59.963	177	4	-0.293	0.079	6.659	2105	WS0072_E02
10	9.580	187	3	-0.391	0.121	6.336	2105	WS0072_E02
10	70.246	190	2	0.296	0.062	6.549	2105	WS0072_E02
11	29.069	205	1	0.270	0.048	4.108	2105	WS0072_E02
11	43.491	214	3	0.167	0.041	4.204	2105	WS0072_E02
11	64.014	222	3	-0.322	0.087	8.901	2105	WS0072_E02
1	95.572	17	3	0.286	0.072	7.552	2106	WS0072_E13
3	14.500	43	2	0.202	0.056	5.344	2106	WS0072_E13
4	142.917	85	1	-0.277	0.069	6.495	2106	WS0072_E13
5	42.979	97	1	-0.246	0.052	4.813	2106	WS0072_E13
5	56.008	101	1	0.291	0.064	5.979	2106	WS0072_E13
8	95.937	159	3	0.209	0.047	5.154	2106	WS0072_E13
12	0.000	226	3	0.155	0.043	4.668	2106	WS0072_E13
12	22.441	227	3	-0.293	0.052	5.815	2106	WS0072_E13
1	82.301	16	3	-0.241	0.060	6.589	2107	WS0072_H13
2	120.617	38	3	0.273	0.045	4.976	2107	WS0072_H13
2	127.147	40	3	-0.187	0.042	4.637	2107	WS0072_H13
6	69.073	121	4	-0.230	0.052	4.145	2107	WS0072_H13
6	89.401	125	1	-0.283	0.053	4.825	2107	WS0072_H13
8	11.974	150	1	-0.386	0.142	12.753	2107	WS0072_H13
8	50.001	154	1	0.197	0.044	4.247	2107	WS0072_H13
8	95.937	159	3	0.230	0.042	4.646	2107	WS0072_H13
9	59.963	177	4	-0.266	0.056	4.188	2107	WS0072_H13
10	78.479	193	3	-0.367	0.050	5.572	2107	WS0072_H13

11	35.604	207	3	-0.237	0.068	7.472	2107	WS0072_H13
11	37.143	209	3	0.200	0.050	5.526	2107	WS0072_H13
11	42.600	213	3	-0.197	0.046	5.019	2107	WS0072_H13
11	64.014	222	3	-0.302	0.061	6.716	2107	WS0072_H13
1	82.301	16	3	-0.259	0.043	5.227	2108	WS0072_I08
1	95.572	17	3	0.398	0.100	12.214	2108	WS0072_I08
1	107.333	19	3	0.240	0.055	6.664	2108	WS0072_I08
2	102.814	36	1	0.234	0.038	4.362	2108	WS0072_I08
3	141.443	65	3	-0.285	0.079	5.810	2108	WS0072_I08
4	6.032	72	4	-0.244	0.075	4.975	2108	WS0072_I08
6	25.914	114	1	-0.323	0.078	8.070	2108	WS0072_I08
6	45.897	117	1	0.239	0.047	4.829	2108	WS0072_I08
6	95.249	126	1	-0.253	0.068	4.984	2108	WS0072_I08
6	106.702	128	2	0.414	0.085	4.207	2108	WS0072_I08
7	59.789	137	3	-0.187	0.035	4.234	2108	WS0072_I08
7	70.278	141	3	0.209	0.045	5.484	2108	WS0072_I08
7	91.487	146	3	0.218	0.048	5.803	2108	WS0072_I08
8	87.463	158	2	0.193	0.051	4.210	2108	WS0072_I08
8	95.937	159	3	0.193	0.033	4.017	2108	WS0072_I08
10	78.479	193	3	-0.384	0.068	8.281	2108	WS0072_I08
11	35.604	207	3	-0.185	0.033	3.969	2108	WS0072_I08
11	56.921	219	2	-0.319	0.102	4.859	2108	WS0072_I08
14	44.898	251	1	0.277	0.041	4.329	2108	WS0072_I08
6	45.897	117	1	0.330	0.074	8.670	2109	WS0072_L02
6	49.100	118	4	0.330	0.079	3.883	2109	WS0072_L02
7	74.560	143	1	-0.396	0.092	10.790	2109	WS0072_L02
7	75.883	144	2	0.442	0.121	5.024	2109	WS0072_L02
8	50.001	154	1	0.237	0.060	7.577	2109	WS0072_L02
11	35.604	207	3	-0.180	0.046	5.043	2109	WS0072_L02
11	42.600	213	3	-0.163	0.037	4.039	2109	WS0072_L02
11	64.014	222	3	-0.267	0.046	5.041	2109	WS0072_L02
4	142.917	85	1	-0.251	0.036	3.850	2110	WS0072_L14
6	25.914	114	1	-0.196	0.037	3.901	2110	WS0072_L14
6	45.897	117	1	0.258	0.052	5.454	2110	WS0072_L14
7	15.555	130	3	-0.275	0.057	6.125	2110	WS0072_L14
8	50.001	154	1	0.253	0.058	7.062	2110	WS0072_L14
4	75.958	77	3	-0.358	0.077	3.953	2111	WS00931_G10
4	124.132	83	1	0.444	0.076	4.583	2111	WS00931_G10
6	106.702	128	3	0.248	0.049	4.498	2111	WS00931_G10
7	75.883	144	2	0.388	0.108	4.313	2111	WS00931_G10
9	3.127	168	1	-0.604	0.150	8.878	2111	WS00931_G10
12	22.441	227	1	-0.160	0.042	4.306	2111	WS00931_G10
12	14.406	230	4	-0.217	0.063	4.963	2111	WS00931_G10
13	3.386	245	1	0.303	0.047	4.990	2111	WS00931_G10
1	22.419	4	4	-0.428	0.096	4.623	2112	WS00931_K10
1	133.992	24	2	0.180	0.058	4.802	2112	WS00931_K10
3	144.593	66	4	-0.205	0.054	5.127	2112	WS00931_K10
4	124.132	83	1	0.563	0.123	6.528	2112	WS00931_K10
4	142.917	85	1	-0.263	0.059	6.128	2112	WS00931_K10
6	49.100	118	4	0.395	0.085	4.279	2112	WS00931_K10
7	74.560	143	1	-0.263	0.039	4.138	2112	WS00931_K10
9	3.127	168	1	-0.540	0.169	8.465	2112	WS00931_K10
10	9.580	187	3	-0.278	0.080	4.094	2112	WS00931_K10
10	78.479	193	3	-0.316	0.047	4.678	2112	WS00931_K10

1	38.326	6	2	0.239	0.052	5.230	2113	WS01025_M21
1	45.794	9	3	-0.270	0.071	5.967	2113	WS01025_M21
3	47.928	49	1	0.252	0.059	5.314	2113	WS01025_M21
4	0.000	71	4	-0.295	0.068	6.681	2113	WS01025_M21
6	69.073	121	3	-0.219	0.043	3.882	2113	WS01025_M21
6	89.401	125	1	-0.308	0.055	5.022	2113	WS01025_M21
6	95.249	126	1	0.394	0.167	6.381	2113	WS01025_M21
8	39.090	152	4	0.225	0.058	5.599	2113	WS01025_M21
11	56.921	219	1	0.377	0.096	5.015	2113	WS01025_M21
14	0.000	246	1	-0.208	0.045	4.048	2113	WS01025_M21
1	22.419	4	3	0.369	0.121	5.649	2114	WS01026_B11
1	38.326	6	2	0.258	0.058	5.823	2114	WS01026_B11
1	43.578	8	2	-0.284	0.045	4.481	2114	WS01026_B11
1	45.794	9	3	-0.229	0.041	4.146	2114	WS01026_B11
4	124.132	83	1	0.507	0.121	5.177	2114	WS01026_B11
4	142.917	85	1	-0.268	0.057	5.059	2114	WS01026_B11
5	57.017	102	4	0.205	0.048	3.973	2114	WS01026_B11
9	3.127	168	1	-0.472	0.165	7.348	2114	WS01026_B11
11	64.014	222	3	-0.197	0.043	4.257	2114	WS01026_B11
1	15.589	3	2	-0.157	0.034	3.940	2115	WS01035_H24
1	141.021	25	2	-0.270	0.055	6.463	2115	WS01035_H24
3	69.071	52	1	-0.329	0.109	4.776	2115	WS01035_H24
3	137.891	64	3	0.284	0.098	8.018	2115	WS01035_H24
3	154.505	69	1	-0.297	0.056	4.096	2115	WS01035_H24
5	3.484	90	2	-0.184	0.052	4.009	2115	WS01035_H24
6	89.401	125	2	-0.225	0.031	3.898	2115	WS01035_H24
7	15.555	130	2	-0.345	0.051	5.898	2115	WS01035_H24
7	15.555	130	3	0.176	0.052	4.687	2115	WS01035_H24
8	0.000	148	4	-0.269	0.066	6.992	2115	WS01035_H24
11	0.000	199	2	0.317	0.037	4.409	2115	WS01035_H24
1	95.572	17	3	0.320	0.069	6.059	2116	WS01035_J02
4	82.462	78	2	-0.147	0.044	3.860	2116	WS01035_J02
5	7.672	92	1	-0.308	0.042	5.462	2116	WS01035_J02
5	7.672	92	4	0.253	0.051	4.396	2116	WS01035_J02
6	25.742	113	3	-0.206	0.063	6.559	2116	WS01035_J02
9	2.011	167	4	0.326	0.118	9.827	2116	WS01035_J02
9	49.013	173	1	-0.237	0.055	4.721	2116	WS01035_J02
10	77.676	192	1	-0.278	0.044	4.441	2116	WS01035_J02
10	86.151	195	3	-0.210	0.065	6.732	2116	WS01035_J02
10	104.214	198	2	-0.267	0.051	5.599	2116	WS01035_J02
11	17.088	203	1	0.246	0.054	5.274	2116	WS01035_J02
11	56.921	219	1	0.282	0.129	4.674	2116	WS01035_J02
13	3.386	245	1	0.211	0.067	6.524	2116	WS01035_J02
14	0.000	246	1	-0.272	0.061	5.936	2116	WS01035_J02
14	0.000	246	2	0.195	0.046	4.159	2116	WS01035_J02
1	43.578	8	2	0.255	0.057	6.655	2117	WS01035_M15
1	77.177	15	2	-0.181	0.035	4.085	2117	WS01035_M15
1	95.572	17	2	-0.273	0.048	5.588	2117	WS01035_M15
1	141.021	25	2	0.218	0.040	4.664	2117	WS01035_M15
3	80.211	55	4	-0.193	0.041	4.580	2117	WS01035_M15
4	0.000	71	4	0.245	0.060	6.821	2117	WS01035_M15
10	78.479	193	4	-0.242	0.046	4.507	2117	WS01035_M15
1	22.419	4	4	0.390	0.101	4.616	2118	WS01035_N22
1	69.854	13	2	-0.169	0.039	4.294	2118	WS01035_N22

1	71.592	14	2	-0.160	0.036	3.918	2118	WS01035_N22
1	95.572	17	2	-0.416	0.120	13.267	2118	WS01035_N22
3	148.745	68	4	0.309	0.057	7.166	2118	WS01035_N22
4	82.462	78	2	-0.316	0.124	12.252	2118	WS01035_N22
4	92.039	79	2	-0.190	0.041	4.269	2118	WS01035_N22
4	92.039	79	4	-0.241	0.046	5.726	2118	WS01035_N22
4	97.576	80	2	0.379	0.131	15.010	2118	WS01035_N22
4	116.928	82	2	0.347	0.073	7.971	2118	WS01035_N22
5	52.820	99	1	0.258	0.064	5.031	2118	WS01035_N22
6	49.100	118	4	-0.415	0.116	4.185	2118	WS01035_N22
11	9.237	202	4	-0.172	0.031	3.911	2118	WS01035_N22
11	41.984	212	2	0.190	0.045	4.897	2118	WS01035_N22
11	43.491	214	4	0.202	0.040	5.222	2118	WS01035_N22
11	45.989	216	2	0.201	0.047	4.762	2118	WS01035_N22
11	57.661	220	4	-0.254	0.039	5.031	2118	WS01035_N22
12	28.493	228	2	0.293	0.069	5.522	2118	WS01035_N22
13	0.000	243	4	-0.407	0.105	5.061	2118	WS01035_N22
1	62.128	11	1	-0.492	0.098	4.780	2119	WS01036_A09
4	103.719	81	4	0.202	0.059	6.314	2119	WS01036_A09
4	116.928	82	2	-0.215	0.047	4.246	2119	WS01036_A09
5	42.979	97	1	0.172	0.041	4.026	2119	WS01036_A09
9	2.011	167	4	-0.268	0.040	4.437	2119	WS01036_A09
9	3.127	168	1	0.418	0.085	4.266	2119	WS01036_A09
10	70.246	190	2	-0.222	0.053	4.996	2119	WS01036_A09
11	9.237	202	4	-0.162	0.037	3.948	2119	WS01036_A09
12	32.473	238	1	-0.237	0.048	4.681	2119	WS01036_A09
3	148.745	68	4	0.276	0.060	4.608	2120	WS01036_B13
6	95.249	126	1	-0.388	0.120	6.579	2120	WS01036_B13
6	106.702	128	2	-0.390	0.089	4.218	2120	WS01036_B13
7	75.883	144	2	-0.386	0.067	3.933	2120	WS01036_B13
9	28.608	170	3	-0.317	0.071	4.999	2120	WS01036_B13
11	64.014	222	3	0.225	0.058	5.823	2120	WS01036_B13
12	22.629	237	1	-0.188	0.046	3.920	2120	WS01036_B13
1	95.572	17	2	0.228	0.042	4.857	2121	WS01036_E19
4	82.462	78	2	-0.249	0.053	6.137	2121	WS01036_E19
4	92.039	79	2	-0.289	0.051	5.894	2121	WS01036_E19
4	97.576	80	2	0.218	0.046	6.078	2121	WS01036_E19
6	25.914	114	1	0.274	0.087	6.329	2121	WS01036_E19
8	87.463	158	2	-0.205	0.036	3.948	2121	WS01036_E19
9	69.046	181	2	-0.304	0.064	4.912	2121	WS01036_E19
11	17.088	203	2	-0.245	0.056	5.184	2121	WS01036_E19
11	29.069	205	2	-0.161	0.033	4.042	2121	WS01036_E19
11	41.984	212	2	-0.230	0.051	5.849	2121	WS01036_E19
11	56.921	219	2	0.517	0.179	13.818	2121	WS01036_E19
3	15.147	44	2	0.259	0.072	4.498	2122	WS01036_F17
3	104.681	60	2	0.234	0.064	6.357	2122	WS01036_F17
3	112.868	61	2	-0.243	0.061	6.121	2122	WS01036_F17
3	114.747	62	2	-0.237	0.063	6.279	2122	WS01036_F17
3	148.745	68	4	0.211	0.045	4.031	2122	WS01036_F17
5	7.672	92	1	0.177	0.042	4.103	2122	WS01036_F17
5	31.191	95	1	0.206	0.051	4.485	2122	WS01036_F17
5	52.820	99	1	0.201	0.046	4.037	2122	WS01036_F17
5	86.669	106	4	0.430	0.113	4.004	2122	WS01036_F17
6	49.100	118	4	-0.414	0.112	4.122	2122	WS01036_F17

7	73.169	142	2	0.497	0.101	5.026	2122	WS01036_F17
7	74.560	143	2	-0.235	0.046	4.405	2122	WS01036_F17
7	75.883	144	2	-0.574	0.137	8.540	2122	WS01036_F17
9	3.127	168	1	0.461	0.103	7.031	2122	WS01036_F17
11	57.661	220	4	-0.305	0.070	6.784	2122	WS01036_F17
11	64.014	222	4	0.320	0.043	4.003	2122	WS01036_F17
12	51.382	242	1	0.427	0.140	5.338	2122	WS01036_F17
2	127.147	40	3	-0.224	0.049	5.102	2123	WS00725_C02
3	95.157	59	2	0.260	0.064	5.514	2123	WS00725_C02
6	89.401	125	1	-0.271	0.039	3.979	2123	WS00725_C02
8	50.001	154	1	0.231	0.074	7.810	2123	WS00725_C02
9	59.963	177	4	-0.265	0.082	6.956	2123	WS00725_C02
10	80.088	194	1	0.234	0.040	4.048	2123	WS00725_C02
11	35.604	207	3	-0.257	0.065	6.798	2123	WS00725_C02
11	37.143	209	3	0.198	0.038	4.035	2123	WS00725_C02
11	42.600	213	3	-0.244	0.054	5.676	2123	WS00725_C02
11	64.014	222	3	-0.294	0.050	5.361	2123	WS00725_C02
1	15.589	3	2	0.254	0.048	4.816	2124	WS00725_D20
2	126.181	39	4	-0.339	0.065	4.171	2124	WS00725_D20
3	112.868	61	2	0.155	0.045	4.409	2124	WS00725_D20
3	114.747	62	2	0.244	0.045	4.471	2124	WS00725_D20
3	116.958	63	4	0.286	0.054	5.175	2124	WS00725_D20
6	54.066	119	4	0.308	0.074	7.033	2124	WS00725_D20
6	69.073	121	4	-0.227	0.048	3.979	2124	WS00725_D20
6	89.401	125	1	-0.194	0.056	4.990	2124	WS00725_D20
6	95.249	126	1	0.316	0.076	4.065	2124	WS00725_D20
8	0.000	148	4	-0.227	0.041	3.878	2124	WS00725_D20
8	50.001	154	1	0.296	0.045	4.645	2124	WS00725_D20
8	50.001	154	2	-0.259	0.048	4.940	2124	WS00725_D20
11	64.014	222	3	-0.206	0.051	4.764	2124	WS00725_D20
2	102.814	36	1	0.307	0.083	8.103	2125	WS00725_J04
2	120.617	38	4	0.214	0.053	4.297	2125	WS00725_J04
4	92.039	79	4	0.337	0.049	4.648	2125	WS00725_J04
4	124.132	83	1	-0.442	0.097	4.068	2125	WS00725_J04
8	39.090	152	4	-0.208	0.042	4.272	2125	WS00725_J04
9	0.000	166	1	0.240	0.060	5.676	2125	WS00725_J04
11	40.960	210	4	-0.307	0.042	3.895	2125	WS00725_J04
11	43.491	214	4	-0.272	0.062	5.724	2125	WS00725_J04
11	48.979	218	4	-0.377	0.067	6.846	2125	WS00725_J04
2	101.076	35	4	0.243	0.070	6.236	2126	WS00725_L09
3	47.928	49	1	0.248	0.065	5.437	2126	WS00725_L09
5	31.191	95	1	-0.207	0.048	3.991	2126	WS00725_L09
6	95.249	126	1	0.336	0.085	4.719	2126	WS00725_L09
8	50.001	154	1	0.283	0.078	7.189	2126	WS00725_L09
10	9.580	187	3	-0.397	0.121	7.048	2126	WS00725_L09
10	70.246	190	2	0.258	0.041	4.589	2126	WS00725_L09
10	78.479	193	3	-0.370	0.047	4.724	2126	WS00725_L09
11	64.014	222	3	-0.213	0.049	4.826	2126	WS00725_L09
1	123.316	20	4	-0.276	0.078	4.439	2127	WS00725_N24
1	127.805	21	4	0.295	0.075	6.723	2127	WS00725_N24
7	74.560	143	1	-0.290	0.054	4.197	2127	WS00725_N24
10	70.246	190	4	-0.279	0.040	4.020	2127	WS00725_N24
1	95.572	17	3	0.287	0.091	6.120	2128	WS00725_O24
1	107.333	19	3	0.264	0.074	6.546	2128	WS00725_O24

5	86.669	106	4	-0.437	0.163	5.911	2128	WS00725_O24
7	17.610	131	3	-0.342	0.131	5.647	2128	WS00725_O24
7	62.787	139	2	-0.225	0.045	4.407	2128	WS00725_O24
7	74.560	143	2	0.336	0.082	8.285	2128	WS00725_O24
10	78.479	193	4	-0.302	0.076	7.434	2128	WS00725_O24
10	80.088	194	2	-0.241	0.050	5.286	2128	WS00725_O24
11	17.088	203	1	-0.205	0.048	3.928	2128	WS00725_O24
11	27.405	204	1	0.281	0.055	4.503	2128	WS00725_O24
11	29.069	205	1	0.243	0.050	4.084	2128	WS00725_O24
11	45.989	216	1	-0.249	0.066	5.415	2128	WS00725_O24
12	51.382	242	1	-0.357	0.118	4.290	2128	WS00725_O24
14	43.392	250	2	0.231	0.049	5.024	2128	WS00725_O24
14	46.637	252	4	0.346	0.070	6.767	2128	WS00725_O24
2	75.550	31	2	0.380	0.064	7.398	2129	WS00727_C11
2	95.880	34	1	-0.306	0.054	4.114	2129	WS00727_C11
2	102.814	36	2	0.119	0.032	4.249	2129	WS00727_C11
4	55.925	76	1	0.196	0.055	4.235	2129	WS00727_C11
4	75.958	77	3	-0.410	0.131	5.563	2129	WS00727_C11
4	116.928	82	4	-0.193	0.044	4.713	2129	WS00727_C11
8	87.463	158	2	-0.265	0.052	6.000	2129	WS00727_C11
14	44.898	251	3	0.298	0.055	4.275	2129	WS00727_C11
1	133.992	24	2	0.193	0.046	5.519	2130	WS00727_D21
4	75.958	77	3	-0.386	0.091	5.276	2130	WS00727_D21
6	25.742	113	3	0.196	0.048	4.784	2130	WS00727_D21
6	106.702	128	2	-0.405	0.073	4.299	2130	WS00727_D21
8	50.001	154	1	0.172	0.057	3.948	2130	WS00727_D21
11	42.600	213	3	-0.194	0.049	4.878	2130	WS00727_D21
1	123.316	20	4	0.307	0.113	7.226	2131	WS00819_A07
2	127.147	40	3	-0.210	0.044	4.456	2131	WS00819_A07
3	95.157	59	2	0.307	0.086	8.845	2131	WS00819_A07
3	104.681	60	2	-0.170	0.043	4.629	2131	WS00819_A07
3	112.868	61	2	0.217	0.057	6.189	2131	WS00819_A07
3	114.747	62	2	0.218	0.040	4.373	2131	WS00819_A07
8	11.974	150	1	-0.224	0.072	5.702	2131	WS00819_A07
10	70.246	190	2	0.246	0.042	4.612	2131	WS00819_A07
11	29.069	205	1	0.233	0.060	4.819	2131	WS00819_A07
11	64.014	222	3	-0.251	0.041	4.123	2131	WS00819_A07
1	45.794	9	1	-0.246	0.060	4.670	2132	WS00912_G08
3	15.147	44	2	-0.323	0.093	4.296	2132	WS00912_G08
3	20.512	46	2	-0.189	0.038	4.218	2132	WS00912_G08
3	47.928	49	1	0.283	0.071	5.535	2132	WS00912_G08
4	75.958	77	3	-0.244	0.076	4.088	2132	WS00912_G08
5	86.669	106	4	-0.474	0.164	5.980	2132	WS00912_G08
9	59.963	177	4	-0.310	0.078	8.059	2132	WS00912_G08
2	93.504	32	4	0.324	0.058	4.655	2133	WS00819_F19
2	101.076	35	3	-0.189	0.044	4.242	2133	WS00819_F19
2	102.814	36	1	0.262	0.068	4.827	2133	WS00819_F19
3	95.157	59	2	-0.185	0.041	4.667	2133	WS00819_F19
3	112.868	61	2	-0.172	0.034	4.027	2133	WS00819_F19
3	114.747	62	2	-0.204	0.034	4.030	2133	WS00819_F19
4	97.576	80	2	-0.198	0.031	3.946	2133	WS00819_F19
11	41.984	212	2	-0.197	0.042	4.962	2133	WS00819_F19
11	45.989	216	2	-0.243	0.060	6.238	2133	WS00819_F19
11	60.464	221	2	-0.253	0.044	5.049	2133	WS00819_F19

1	123.316	20	4	0.339	0.085	5.282	2134	WS00912_L02
3	20.512	46	2	-0.191	0.041	4.481	2134	WS00912_L02
3	47.928	49	1	0.271	0.064	5.109	2134	WS00912_L02
6	69.073	121	3	-0.225	0.045	4.477	2134	WS00912_L02
6	95.249	126	1	0.382	0.093	4.909	2134	WS00912_L02
9	59.963	177	4	-0.242	0.052	5.390	2134	WS00912_L02
10	70.246	190	2	0.256	0.047	5.198	2134	WS00912_L02
11	27.405	204	1	0.437	0.098	8.044	2134	WS00912_L02
11	29.069	205	1	0.404	0.118	9.506	2134	WS00912_L02
12	51.382	242	1	-0.387	0.157	4.378	2134	WS00912_L02
3	95.157	59	2	0.195	0.045	4.077	2135	WS00829_M19
8	11.974	150	1	-0.266	0.062	5.382	2135	WS00829_M19
11	64.014	222	3	-0.244	0.053	5.546	2135	WS00829_M19
1	82.301	16	3	-0.272	0.058	6.228	2136	WS00830_H17
6	89.401	125	1	-0.225	0.077	5.482	2136	WS00830_H17
7	15.555	130	2	-0.317	0.053	6.969	2136	WS00830_H17
7	17.610	131	3	-0.288	0.075	4.788	2136	WS00830_H17
9	28.608	170	2	-0.277	0.029	3.907	2136	WS00830_H17
9	49.013	173	2	0.114	0.033	5.138	2136	WS00830_H17
11	0.000	199	2	0.428	0.087	11.295	2136	WS00830_H17
11	17.088	203	2	-0.167	0.055	7.748	2136	WS00830_H17
11	27.405	204	1	0.370	0.086	6.485	2136	WS00830_H17
3	83.563	56	3	-0.157	0.043	4.016	2137	WS00931_O18
4	75.958	77	3	-0.330	0.091	5.849	2137	WS00931_O18
6	87.891	124	2	0.233	0.042	5.040	2137	WS00931_O18
9	59.963	177	4	-0.252	0.053	5.784	2137	WS00931_O18
9	61.113	178	1	-0.300	0.112	4.274	2137	WS00931_O18
10	78.479	193	3	-0.299	0.051	4.766	2137	WS00931_O18
11	27.405	204	4	0.211	0.047	4.400	2137	WS00931_O18
3	9.953	42	3	0.209	0.060	5.637	2138	WS01029_D11
4	103.719	81	4	0.301	0.064	6.347	2138	WS01029_D11
7	59.789	137	3	0.256	0.052	4.934	2138	WS01029_D11
7	70.278	141	3	-0.274	0.063	6.001	2138	WS01029_D11
12	6.242	234	4	-0.262	0.061	8.611	2138	WS01029_D11
1	45.794	9	1	-0.230	0.055	4.321	2139	WS00913_M01
3	69.071	52	1	0.235	0.087	3.964	2139	WS00913_M01
4	116.928	82	2	0.272	0.064	7.052	2139	WS00913_M01
4	142.917	85	1	-0.250	0.060	4.546	2139	WS00913_M01
7	15.555	130	2	-0.211	0.038	4.164	2139	WS00913_M01
7	73.169	142	2	0.541	0.094	6.121	2139	WS00913_M01
7	75.883	144	2	-0.584	0.115	8.774	2139	WS00913_M01
10	70.246	190	4	0.215	0.039	4.000	2139	WS00913_M01
10	95.795	197	4	-0.219	0.046	4.861	2139	WS00913_M01
11	37.143	209	3	0.219	0.055	4.511	2139	WS00913_M01
2	48.839	29	4	-0.216	0.045	4.073	2140	WS00914_C09
7	70.278	141	3	0.209	0.043	4.097	2140	WS00914_C09
9	36.227	165	4	0.304	0.092	8.459	2140	WS00914_C09
9	28.608	170	3	-0.268	0.048	4.605	2140	WS00914_C09
9	55.358	175	1	-0.337	0.063	5.257	2140	WS00914_C09
9	55.358	175	3	0.488	0.117	6.387	2140	WS00914_C09
9	69.046	181	1	0.333	0.080	3.892	2140	WS00914_C09
10	35.204	189	1	0.271	0.048	3.903	2140	WS00914_C09
12	22.629	237	1	-0.244	0.061	4.986	2140	WS00914_C09
12	32.473	238	1	-0.242	0.058	4.755	2140	WS00914_C09

2	120.617	38	3	0.170	0.043	4.077	2141	WS01029_O18
3	95.157	59	2	0.211	0.047	5.540	2141	WS01029_O18
3	112.868	61	2	0.272	0.048	5.952	2141	WS01029_O18
3	114.747	62	2	0.128	0.032	3.890	2141	WS01029_O18
6	95.249	126	1	0.359	0.121	4.631	2141	WS01029_O18
9	49.013	173	2	0.299	0.070	8.558	2141	WS01029_O18
9	59.963	177	4	-0.425	0.126	11.748	2141	WS01029_O18
11	64.014	222	3	-0.185	0.050	4.650	2141	WS01029_O18
9	59.963	177	4	-0.254	0.073	5.932	2142	WS01029_P12
11	17.088	203	1	-0.316	0.114	7.584	2142	WS01029_P12
11	29.069	205	1	0.350	0.072	5.191	2142	WS01029_P12
11	45.989	216	1	-0.230	0.081	5.376	2142	WS01029_P12
12	6.242	234	2	0.225	0.043	5.020	2142	WS01029_P12
3	15.147	44	2	-0.253	0.076	4.424	2143	WS01030_B04
3	104.681	60	2	-0.214	0.037	4.301	2143	WS01030_B04
10	70.246	190	2	0.278	0.066	7.827	2143	WS01030_B04
1	62.128	11	2	0.344	0.118	5.414	2144	WS01030_B18
1	77.177	15	2	0.212	0.062	5.955	2144	WS01030_B18
3	80.211	55	4	-0.265	0.068	6.597	2144	WS01030_B18
3	137.891	64	3	-0.345	0.094	7.930	2144	WS01030_B18
4	55.925	76	1	0.283	0.066	5.844	2144	WS01030_B18
5	42.979	97	2	-0.318	0.073	6.924	2144	WS01030_B18
9	0.000	163	2	0.449	0.086	4.013	2144	WS01030_B18
9	55.358	175	3	0.410	0.093	3.879	2144	WS01030_B18
11	56.921	219	1	-0.320	0.105	4.905	2144	WS01030_B18
12	0.000	233	1	-0.178	0.043	3.901	2144	WS01030_B18
13	0.000	243	4	0.422	0.129	6.105	2144	WS01030_B18
1	43.578	8	2	0.302	0.056	6.692	2145	WS01030_D24
4	173.929	88	2	-0.204	0.035	4.038	2145	WS01030_D24
6	106.702	128	2	0.495	0.111	6.200	2145	WS01030_D24
8	50.001	154	1	0.211	0.062	5.131	2145	WS01030_D24
10	9.580	187	3	-0.321	0.119	4.701	2145	WS01030_D24
11	43.491	214	3	-0.240	0.045	4.688	2145	WS01030_D24
11	44.884	215	3	0.218	0.041	4.261	2145	WS01030_D24
12	51.382	242	1	-0.363	0.209	4.998	2145	WS01030_D24
1	22.419	4	4	0.401	0.132	5.247	2146	WS01030_E11
1	62.128	11	1	0.472	0.091	4.240	2146	WS01030_E11
3	17.158	45	1	-0.261	0.063	6.706	2146	WS01030_E11
5	42.979	97	2	-0.277	0.079	5.805	2146	WS01030_E11
5	56.008	101	2	-0.275	0.083	6.640	2146	WS01030_E11
5	57.017	102	2	0.210	0.065	5.360	2146	WS01030_E11
6	26.350	115	2	-0.228	0.071	5.726	2146	WS01030_E11
6	56.188	120	2	0.324	0.112	8.707	2146	WS01030_E11
9	81.073	183	1	-0.334	0.053	5.723	2146	WS01030_E11
10	21.864	188	1	0.218	0.036	3.869	2146	WS01030_E11
10	93.347	196	2	0.456	0.141	4.335	2146	WS01030_E11
11	46.026	217	1	-0.269	0.061	6.572	2146	WS01030_E11
11	57.661	220	4	-0.232	0.050	4.643	2146	WS01030_E11
11	64.014	222	4	0.293	0.051	4.204	2146	WS01030_E11
13	1.815	244	4	-0.272	0.050	4.294	2146	WS01030_E11
14	44.898	251	1	0.260	0.037	3.985	2146	WS01030_E11
3	137.891	64	3	0.251	0.052	5.005	2147	WS01030_G01
4	116.928	82	2	-0.350	0.067	7.875	2147	WS01030_G01
4	124.132	83	2	-0.370	0.133	5.818	2147	WS01030_G01

5	0.000	89	3	-0.251	0.044	3.988	2147	WS01030_G01
5	16.219	93	2	-0.360	0.059	7.019	2147	WS01030_G01
7	75.883	144	2	0.484	0.093	5.905	2147	WS01030_G01
9	28.608	170	2	-0.287	0.042	5.208	2147	WS01030_G01
9	49.013	173	2	0.187	0.052	4.781	2147	WS01030_G01
9	61.113	178	1	-0.377	0.146	4.413	2147	WS01030_G01
11	45.989	216	1	-0.145	0.057	4.043	2147	WS01030_G01
3	14.500	43	2	0.234	0.038	4.055	2148	WS01030_G24
4	55.925	76	1	-0.262	0.097	8.016	2148	WS01030_G24
8	50.001	154	2	0.288	0.073	8.153	2148	WS01030_G24
8	87.463	158	2	-0.158	0.034	3.869	2148	WS01030_G24
12	0.000	233	1	0.263	0.061	4.802	2148	WS01030_G24
1	131.686	23	1	0.494	0.108	4.148	2149	WS00915_B13
6	95.249	126	1	0.365	0.141	6.693	2149	WS00915_B13
9	59.963	177	4	-0.327	0.070	5.137	2149	WS00915_B13
13	1.815	244	4	-0.182	0.039	3.941	2149	WS00915_B13
1	45.794	9	1	0.217	0.051	4.611	2150	WS00915_C01
2	102.814	36	1	0.209	0.046	4.179	2150	WS00915_C01
5	3.484	90	2	0.285	0.094	3.876	2150	WS00915_C01
6	25.742	113	3	0.244	0.053	6.741	2150	WS00915_C01
7	17.610	131	3	0.275	0.089	5.131	2150	WS00915_C01
10	35.204	189	1	-0.316	0.057	5.100	2150	WS00915_C01
11	27.405	204	1	0.326	0.072	6.409	2150	WS00915_C01
11	29.069	205	1	0.219	0.049	4.374	2150	WS00915_C01
12	14.406	230	3	0.214	0.038	4.782	2150	WS00915_C01
13	1.815	244	2	0.209	0.051	5.231	2150	WS00915_C01
13	3.386	245	1	0.281	0.070	6.192	2150	WS00915_C01
1	69.854	13	2	-0.215	0.055	6.419	2151	WS00915_E05
1	71.592	14	2	-0.215	0.055	6.489	2151	WS00915_E05
1	77.177	15	2	-0.256	0.076	8.894	2151	WS00915_E05
1	95.572	17	2	-0.377	0.084	9.961	2151	WS00915_E05
1	95.572	17	3	0.150	0.043	4.221	2151	WS00915_E05
3	15.147	44	2	-0.313	0.106	6.375	2151	WS00915_E05
4	142.917	85	1	-0.213	0.063	4.420	2151	WS00915_E05
7	15.555	130	3	-0.175	0.059	4.579	2151	WS00915_E05
7	17.610	131	3	-0.446	0.160	8.783	2151	WS00915_E05
7	74.560	143	2	0.178	0.028	4.019	2151	WS00915_E05
9	0.000	163	2	-0.507	0.094	5.040	2151	WS00915_E05
9	59.963	177	4	-0.344	0.122	11.466	2151	WS00915_E05
10	70.246	190	2	0.227	0.045	5.332	2151	WS00915_E05
10	70.246	190	4	-0.360	0.052	4.911	2151	WS00915_E05
10	78.479	193	4	-0.310	0.076	6.744	2151	WS00915_E05
11	60.464	221	2	0.253	0.036	4.543	2151	WS00915_E05
12	22.441	227	3	-0.226	0.048	4.302	2151	WS00915_E05
12	14.406	230	3	-0.293	0.042	4.286	2151	WS00915_E05
5	16.219	93	2	0.281	0.060	6.112	2152	WS00915_E18
9	36.227	165	4	-0.265	0.081	6.770	2152	WS00915_E18
14	22.402	248	3	0.262	0.071	7.336	2152	WS00915_E18
3	9.953	42	3	0.208	0.051	6.694	2153	WS00915_G12
3	80.211	55	4	0.223	0.071	4.059	2153	WS00915_G12
3	95.157	59	2	0.208	0.055	6.468	2153	WS00915_G12
6	95.249	126	1	0.337	0.127	4.774	2153	WS00915_G12
7	17.610	131	3	-0.284	0.053	4.907	2153	WS00915_G12
9	0.000	163	2	-0.421	0.045	4.155	2153	WS00915_G12

10	70.246	190	2	0.212	0.040	5.061	2153	WS00915_G12
1	45.794	9	1	-0.220	0.052	4.706	2154	WS00915_H03
4	124.132	83	1	0.516	0.138	5.386	2154	WS00915_H03
8	11.974	150	1	-0.175	0.066	5.910	2154	WS00915_H03
9	55.358	175	1	0.329	0.052	4.783	2154	WS00915_H03
9	55.358	175	3	-0.364	0.070	3.879	2154	WS00915_H03
3	15.147	44	2	0.244	0.091	5.119	2155	WS00915_J06
3	148.745	68	4	0.205	0.047	4.114	2155	WS00915_J06
5	57.017	102	4	-0.244	0.037	4.044	2155	WS00915_J06
8	87.463	158	2	-0.165	0.041	3.917	2155	WS00915_J06
9	17.951	169	2	-0.268	0.046	4.074	2155	WS00915_J06
9	32.356	171	2	0.227	0.038	4.363	2155	WS00915_J06
10	104.214	198	4	-0.212	0.027	4.123	2155	WS00915_J06
13	3.386	245	1	0.304	0.087	6.525	2155	WS00915_J06
14	9.923	247	1	-0.216	0.081	6.065	2155	WS00915_J06
1	2.788	2	4	-0.252	0.052	5.867	2156	WS00915_J24
2	120.617	38	3	-0.249	0.060	5.513	2156	WS00915_J24
2	127.147	40	3	0.242	0.058	5.335	2156	WS00915_J24
6	49.100	118	2	-0.347	0.148	7.914	2156	WS00915_J24
8	50.001	154	2	0.206	0.039	4.251	2156	WS00915_J24
8	87.463	158	2	-0.275	0.085	9.729	2156	WS00915_J24
9	17.951	169	2	-0.263	0.048	3.875	2156	WS00915_J24
9	32.356	171	2	0.240	0.046	5.298	2156	WS00915_J24
10	35.204	189	1	-0.202	0.060	4.426	2156	WS00915_J24
11	0.000	199	2	0.211	0.039	4.414	2156	WS00915_J24
14	0.000	246	1	0.192	0.062	4.569	2156	WS00915_J24
14	0.000	246	2	0.273	0.059	6.781	2156	WS00915_J24
4	75.958	77	3	-0.375	0.094	4.730	2157	WS0053_N21
4	124.132	83	1	0.509	0.129	5.338	2157	WS0053_N21
7	74.560	143	1	0.241	0.041	4.199	2157	WS0053_N21
8	87.463	158	2	-0.196	0.062	5.141	2157	WS0053_N21
11	27.405	204	1	0.261	0.039	3.964	2157	WS0053_N21
11	29.069	205	1	0.266	0.042	4.344	2157	WS0053_N21
11	45.989	216	1	-0.246	0.043	4.318	2157	WS0053_N21
12	22.441	227	1	-0.216	0.047	4.649	2157	WS0053_N21
1	40.464	7	3	0.155	0.036	4.076	2158	WS0053_O15
1	43.578	8	3	0.133	0.027	4.468	2158	WS0053_O15
3	9.953	42	3	0.247	0.063	7.157	2158	WS0053_O15
3	14.500	43	1	0.305	0.038	4.434	2158	WS0053_O15
3	17.158	45	1	-0.152	0.037	4.103	2158	WS0053_O15
10	104.214	198	4	-0.281	0.071	6.320	2158	WS0053_O15
2	63.261	30	3	0.232	0.039	4.025	2159	WS0054_D03
3	15.147	44	2	0.326	0.146	5.877	2159	WS0054_D03
3	20.512	46	2	0.276	0.075	6.441	2159	WS0054_D03
3	47.928	49	1	0.179	0.038	3.884	2159	WS0054_D03
4	55.925	76	1	-0.232	0.053	5.162	2159	WS0054_D03
5	31.191	95	1	-0.210	0.050	5.150	2159	WS0054_D03
6	95.249	126	1	0.288	0.079	4.401	2159	WS0054_D03
10	78.479	193	3	-0.339	0.043	4.356	2159	WS0054_D03
10	86.151	195	3	0.180	0.046	4.588	2159	WS0054_D03
11	35.604	207	3	-0.173	0.047	4.648	2159	WS0054_D03
11	37.143	209	3	0.204	0.061	6.133	2159	WS0054_D03
11	60.464	221	2	0.288	0.079	6.937	2159	WS0054_D03
1	22.419	4	3	0.298	0.060	4.949	2160	WS0054_E09

3	9.953	42	3	0.230	0.045	6.308	2160	WS0054_E09
3	14.500	43	1	0.322	0.051	5.416	2160	WS0054_E09
3	17.158	45	1	-0.242	0.060	6.313	2160	WS0054_E09
3	137.891	64	1	0.249	0.049	5.262	2160	WS0054_E09
3	148.745	68	1	-0.269	0.072	7.550	2160	WS0054_E09
8	95.937	159	3	-0.226	0.060	8.313	2160	WS0054_E09
9	58.045	176	1	-0.351	0.060	5.034	2160	WS0054_E09
9	69.046	181	1	0.204	0.050	4.036	2160	WS0054_E09
11	43.491	214	3	-0.234	0.032	4.556	2160	WS0054_E09
1	29.800	5	2	-0.290	0.044	4.283	2161	WS0054_H12
1	38.326	6	2	0.194	0.041	4.159	2161	WS0054_H12
2	120.617	38	3	0.198	0.036	3.895	2161	WS0054_H12
3	47.928	49	1	0.216	0.058	5.106	2161	WS0054_H12
8	50.001	154	1	0.248	0.072	6.757	2161	WS0054_H12
9	59.963	177	4	-0.269	0.068	5.437	2161	WS0054_H12
10	9.580	187	3	-0.360	0.101	5.543	2161	WS0054_H12
10	70.246	190	2	0.290	0.064	6.588	2161	WS0054_H12
10	78.479	193	3	-0.456	0.082	8.930	2161	WS0054_H12
11	64.014	222	3	-0.272	0.060	6.569	2161	WS0054_H12
3	15.147	44	2	-0.306	0.078	4.666	2162	WS0054_I12
6	56.188	120	2	0.214	0.036	3.939	2162	WS0054_I12
9	59.963	177	4	-0.355	0.107	9.741	2162	WS0054_I12
10	70.246	190	2	0.310	0.063	7.136	2162	WS0054_I12
10	78.479	193	3	-0.293	0.051	5.034	2162	WS0054_I12
11	64.014	222	3	-0.172	0.039	3.843	2162	WS0054_I12
1	107.333	19	3	0.222	0.053	4.775	2163	WS0054_L14
2	102.814	36	1	0.192	0.050	4.536	2163	WS0054_L14
9	49.013	173	2	0.144	0.037	3.932	2163	WS0054_L14
9	55.358	175	3	0.377	0.077	3.953	2163	WS0054_L14
1	15.589	3	2	0.196	0.049	4.819	2164	WS0054_M11
4	0.000	71	4	-0.226	0.047	4.305	2164	WS0054_M11
4	75.958	77	3	-0.324	0.107	4.709	2164	WS0054_M11
4	124.132	83	1	0.459	0.118	4.300	2164	WS0054_M11
8	50.001	154	1	0.264	0.065	6.860	2164	WS0054_M11
8	62.580	155	1	0.313	0.140	5.068	2164	WS0054_M11
10	9.580	187	3	-0.379	0.115	5.956	2164	WS0054_M11
10	78.479	193	3	-0.328	0.051	4.899	2164	WS0054_M11
1	43.578	8	2	-0.205	0.040	4.394	2165	WS00911_L20
3	148.745	68	1	0.259	0.056	4.387	2165	WS00911_L20
6	56.188	120	1	0.276	0.073	5.728	2165	WS00911_L20
8	87.463	158	2	-0.220	0.051	4.424	2165	WS00911_L20
11	35.604	207	3	-0.180	0.054	4.713	2165	WS00911_L20
11	42.600	213	3	-0.201	0.062	5.415	2165	WS00911_L20
11	64.014	222	3	-0.246	0.047	4.117	2165	WS00911_L20
1	69.854	13	2	-0.197	0.038	4.966	2166	WS00912_A20
1	71.592	14	2	-0.192	0.036	4.791	2166	WS00912_A20
1	77.177	15	2	-0.218	0.046	6.104	2166	WS00912_A20
6	26.350	115	2	-0.203	0.035	4.587	2166	WS00912_A20
7	15.555	130	3	-0.226	0.052	5.614	2166	WS00912_A20
7	105.477	147	2	0.200	0.066	3.958	2166	WS00912_A20
8	50.001	154	2	-0.211	0.043	5.534	2166	WS00912_A20
8	87.463	158	2	0.242	0.044	5.793	2166	WS00912_A20
9	59.963	177	4	-0.212	0.067	5.781	2166	WS00912_A20
10	70.246	190	2	0.290	0.060	7.882	2166	WS00912_A20

10	78.479	193	4	-0.262	0.049	4.200	2166	WS00912_A20
10	80.088	194	2	-0.379	0.062	8.902	2166	WS00912_A20
10	93.347	196	2	0.489	0.076	5.371	2166	WS00912_A20
10	104.214	198	2	-0.246	0.021	3.916	2166	WS00912_A20
12	6.242	234	2	0.272	0.062	8.565	2166	WS00912_A20
3	148.745	68	1	0.290	0.045	4.502	2167	WS00828_F21
4	43.146	74	3	-0.231	0.036	4.800	2167	WS00828_F21
6	95.249	126	1	0.258	0.056	4.135	2167	WS00828_F21
7	74.560	143	1	-0.290	0.069	6.821	2167	WS00828_F21
10	9.580	187	3	-0.351	0.080	5.920	2167	WS00828_F21
10	35.204	189	1	-0.295	0.065	6.451	2167	WS00828_F21
10	78.479	193	3	-0.369	0.046	6.218	2167	WS00828_F21
11	43.491	214	3	0.218	0.056	7.361	2167	WS00828_F21
11	44.884	215	3	-0.268	0.048	6.387	2167	WS00828_F21
13	3.386	245	1	0.268	0.047	4.700	2167	WS00828_F21
14	0.000	246	1	-0.230	0.040	4.045	2167	WS00828_F21
3	47.928	49	1	0.232	0.043	4.032	2168	WS01031_F12
6	89.401	125	1	-0.289	0.048	4.550	2168	WS01031_F12
6	95.249	126	1	0.326	0.069	3.942	2168	WS01031_F12
7	75.883	144	2	0.390	0.068	3.963	2168	WS01031_F12
8	50.001	154	1	0.258	0.046	4.778	2168	WS01031_F12
9	59.963	177	4	-0.295	0.069	6.342	2168	WS01031_F12
10	70.246	190	2	0.265	0.044	4.159	2168	WS01031_F12
11	27.405	204	1	0.312	0.044	4.240	2168	WS01031_F12
11	29.069	205	1	0.312	0.058	5.538	2168	WS01031_F12
11	64.014	222	3	-0.165	0.041	3.944	2168	WS01031_F12
4	6.032	72	4	-0.234	0.050	3.963	2169	WS00829_C02
5	0.000	89	1	0.300	0.062	5.379	2169	WS00829_C02
5	16.219	93	1	0.337	0.092	7.988	2169	WS00829_C02
6	25.742	113	3	0.220	0.042	4.577	2169	WS00829_C02
7	74.560	143	1	-0.308	0.060	5.309	2169	WS00829_C02
10	78.479	193	3	-0.359	0.046	5.009	2169	WS00829_C02
3	95.157	59	2	0.266	0.069	6.273	2170	WS01031_J05
4	55.925	76	1	-0.208	0.056	4.838	2170	WS01031_J05
4	82.462	78	1	-0.253	0.043	4.104	2170	WS01031_J05
6	95.249	126	1	0.264	0.069	3.910	2170	WS01031_J05
8	11.974	150	1	-0.226	0.061	5.669	2170	WS01031_J05
11	64.014	222	3	-0.231	0.045	4.257	2170	WS01031_J05
1	131.686	23	1	0.745	0.222	9.444	2171	WS00935_H14
5	84.347	105	1	0.234	0.073	7.814	2171	WS00935_H14
8	11.974	150	1	-0.233	0.066	7.075	2171	WS00935_H14
8	39.090	152	4	0.258	0.043	4.030	2171	WS00935_H14
9	28.608	170	3	-0.255	0.040	4.000	2171	WS00935_H14
11	56.921	219	1	0.315	0.099	4.192	2171	WS00935_H14
11	64.014	222	3	-0.296	0.046	4.620	2171	WS00935_H14
12	0.000	233	1	0.287	0.051	5.579	2171	WS00935_H14
12	51.382	242	1	-0.273	0.091	3.844	2171	WS00935_H14
1	131.686	23	1	0.522	0.082	6.131	2172	WS00935_I07
3	15.147	44	2	-0.284	0.158	7.084	2172	WS00935_I07
3	20.512	46	2	-0.283	0.049	4.447	2172	WS00935_I07
3	144.647	67	1	0.371	0.103	7.701	2172	WS00935_I07
5	0.000	89	1	-0.218	0.043	4.472	2172	WS00935_I07
5	16.219	93	1	-0.223	0.039	4.035	2172	WS00935_I07
6	76.376	122	1	-0.372	0.075	5.669	2172	WS00935_I07

6	89.401	125	1	0.271	0.042	4.402	2172	WS00935_I07
7	67.154	140	4	-0.367	0.074	5.583	2172	WS00935_I07
9	0.000	166	1	0.184	0.038	3.987	2172	WS00935_I07
9	59.963	177	1	0.213	0.038	3.915	2172	WS00935_I07
9	61.113	178	1	0.444	0.122	9.159	2172	WS00935_I07
9	81.073	183	1	0.325	0.056	5.711	2172	WS00935_I07
10	21.864	188	1	-0.324	0.059	6.038	2172	WS00935_I07
12	28.493	228	2	0.272	0.142	6.523	2172	WS00935_I07
12	0.000	233	1	0.238	0.058	6.030	2172	WS00935_I07
12	32.473	238	1	0.196	0.037	3.861	2172	WS00935_I07
14	43.392	250	2	0.212	0.060	4.299	2172	WS00935_I07
14	44.898	251	1	0.227	0.039	4.245	2172	WS00935_I07
1	62.128	11	2	0.242	0.055	4.330	2173	WS00935_M09
1	82.301	16	3	-0.263	0.039	5.232	2173	WS00935_M09
1	107.333	19	3	0.208	0.029	3.975	2173	WS00935_M09
3	9.953	42	3	0.132	0.029	3.951	2173	WS00935_M09
4	6.032	72	4	-0.216	0.090	4.654	2173	WS00935_M09
6	95.249	126	1	0.384	0.098	5.262	2173	WS00935_M09
7	17.610	131	3	-0.288	0.055	4.574	2173	WS00935_M09
1	22.419	4	3	0.337	0.066	4.318	2174	WS00935_N23
3	95.157	59	2	0.164	0.046	4.473	2174	WS00935_N23
3	137.891	64	3	0.277	0.048	5.369	2174	WS00935_N23
3	141.443	65	3	0.357	0.082	5.349	2174	WS00935_N23
4	6.032	72	4	-0.225	0.076	5.323	2174	WS00935_N23
5	0.000	89	3	-0.285	0.057	6.732	2174	WS00935_N23
6	95.249	126	1	0.326	0.066	4.300	2174	WS00935_N23
7	17.610	131	3	-0.427	0.153	8.123	2174	WS00935_N23
7	73.169	142	2	0.446	0.093	4.685	2174	WS00935_N23
7	75.883	144	2	-0.404	0.080	4.090	2174	WS00935_N23
1	45.794	9	1	-0.239	0.055	5.626	2175	WS00827_C15
1	82.301	16	3	-0.233	0.047	4.409	2175	WS00827_C15
5	31.191	95	1	-0.306	0.085	8.769	2175	WS00827_C15
7	73.169	142	1	0.450	0.061	3.860	2175	WS00827_C15
9	61.113	178	1	0.350	0.065	4.124	2175	WS00827_C15
9	69.046	181	2	-0.313	0.083	4.495	2175	WS00827_C15
11	56.921	219	2	0.311	0.079	4.115	2175	WS00827_C15
11	60.464	221	1	0.311	0.074	7.612	2175	WS00827_C15
1	29.800	5	1	-0.191	0.083	8.401	2176	WS00827_E20
1	82.301	16	4	0.477	0.069	8.506	2176	WS00827_E20
3	148.745	68	1	-0.327	0.039	5.161	2176	WS00827_E20
4	82.462	78	1	-0.232	0.064	6.438	2176	WS00827_E20
6	56.188	120	1	0.369	0.031	4.312	2176	WS00827_E20
6	95.249	126	1	-0.531	0.095	6.430	2176	WS00827_E20
9	0.000	166	1	0.214	0.055	5.517	2176	WS00827_E20
9	28.608	170	2	0.341	0.048	5.611	2176	WS00827_E20
9	28.608	170	3	-0.434	0.086	8.875	2176	WS00827_E20
9	59.963	177	1	-0.358	0.053	6.573	2176	WS00827_E20
9	81.073	183	1	-0.156	0.055	5.380	2176	WS00827_E20
11	9.237	202	4	0.287	0.058	5.831	2176	WS00827_E20
12	51.382	242	1	-0.550	0.145	6.483	2176	WS00827_E20
1	2.788	2	4	-0.265	0.069	4.698	2177	WS00827_K24
8	50.001	154	1	-0.236	0.045	5.014	2177	WS00827_K24
9	81.073	183	3	-0.235	0.046	4.741	2177	WS00827_K24
10	9.580	187	3	0.342	0.098	7.236	2177	WS00827_K24

10	77.676	192	1	0.244	0.051	5.147	2177	WS00827_K24
10	80.088	194	1	0.359	0.089	8.973	2177	WS00827_K24
11	27.405	204	1	0.301	0.069	6.969	2177	WS00827_K24
11	27.405	204	4	-0.227	0.055	4.794	2177	WS00827_K24
12	0.000	233	1	0.322	0.102	10.236	2177	WS00827_K24
12	6.242	234	4	-0.268	0.059	4.382	2177	WS00827_K24
13	1.815	244	2	0.226	0.046	4.033	2177	WS00827_K24
14	22.402	248	3	-0.260	0.055	5.684	2177	WS00827_K24
1	131.686	23	1	0.574	0.115	6.375	2178	WS00827_L22
3	17.158	45	1	-0.151	0.035	4.436	2178	WS00827_L22
4	116.928	82	4	0.224	0.080	7.726	2178	WS00827_L22
6	81.996	123	3	0.243	0.062	4.745	2178	WS00827_L22
11	17.088	203	1	0.262	0.087	11.086	2178	WS00827_L22
11	29.069	205	1	-0.318	0.043	5.758	2178	WS00827_L22
14	43.392	250	1	-0.296	0.060	4.422	2178	WS00827_L22
3	137.891	64	1	-0.257	0.091	7.289	2179	WS00915_M20
3	144.647	67	1	-0.420	0.102	4.140	2179	WS00915_M20
5	54.072	100	4	0.201	0.050	5.499	2179	WS00915_M20
6	106.702	128	2	-0.650	0.260	10.508	2179	WS00915_M20
7	15.555	130	2	-0.247	0.038	4.207	2179	WS00915_M20
8	0.000	148	4	-0.243	0.043	4.859	2179	WS00915_M20
10	9.580	187	3	0.302	0.108	4.454	2179	WS00915_M20
10	93.347	196	2	-0.434	0.122	4.172	2179	WS00915_M20
11	17.088	203	1	0.167	0.052	4.087	2179	WS00915_M20
14	22.402	248	4	-0.403	0.051	5.240	2179	WS00915_M20
14	46.637	252	4	-0.355	0.047	5.308	2179	WS00915_M20
4	116.928	82	2	-0.315	0.050	7.066	2180	WS00915_N16
4	124.132	83	2	-0.352	0.103	11.483	2180	WS00915_N16
4	173.929	88	2	0.190	0.031	4.334	2180	WS00915_N16
5	86.669	106	2	-0.261	0.060	5.482	2180	WS00915_N16
9	69.046	181	2	-0.346	0.075	7.055	2180	WS00915_N16
1	82.301	16	3	-0.227	0.055	5.502	2181	WS00916_A08
1	95.572	17	3	0.215	0.040	4.073	2181	WS00916_A08
2	127.147	40	3	-0.167	0.044	4.428	2181	WS00916_A08
3	47.928	49	1	0.341	0.108	9.601	2181	WS00916_A08
4	0.000	71	4	-0.243	0.071	6.205	2181	WS00916_A08
5	52.820	99	3	0.221	0.046	4.651	2181	WS00916_A08
6	89.401	125	1	-0.257	0.046	4.071	2181	WS00916_A08
7	91.487	146	3	0.217	0.049	4.907	2181	WS00916_A08
11	29.069	205	2	-0.162	0.047	4.087	2181	WS00916_A08
11	35.604	207	3	-0.160	0.042	4.190	2181	WS00916_A08
11	41.984	212	2	-0.215	0.046	4.531	2181	WS00916_A08
11	42.600	213	3	-0.175	0.046	4.596	2181	WS00916_A08
11	56.921	219	2	0.413	0.148	7.613	2181	WS00916_A08
11	64.014	222	3	-0.195	0.046	4.627	2181	WS00916_A08
14	22.402	248	4	-0.410	0.111	9.242	2181	WS00916_A08
1	107.333	19	3	0.229	0.043	4.619	2182	WS00916_A21
2	120.617	38	3	0.195	0.043	4.621	2182	WS00916_A21
2	127.147	40	3	-0.178	0.039	4.171	2182	WS00916_A21
3	15.147	44	2	-0.293	0.068	3.969	2182	WS00916_A21
3	47.928	49	1	0.249	0.061	4.283	2182	WS00916_A21
3	104.681	60	2	-0.243	0.044	5.247	2182	WS00916_A21
3	112.868	61	2	0.208	0.038	4.456	2182	WS00916_A21
3	114.747	62	2	0.223	0.050	5.932	2182	WS00916_A21

4	0.000	71	4	-0.263	0.090	7.262	2182	WS00916_A21
6	56.188	120	2	0.240	0.036	4.346	2182	WS00916_A21
9	59.963	177	4	-0.283	0.063	5.143	2182	WS00916_A21
10	78.479	193	3	-0.312	0.060	6.352	2182	WS00916_A21
1	2.788	2	4	0.309	0.089	9.303	2183	WS00916_C09
1	133.992	24	2	0.193	0.048	4.971	2183	WS00916_C09
1	141.021	25	2	-0.222	0.062	6.459	2183	WS00916_C09
2	48.839	29	1	-0.275	0.070	5.847	2183	WS00916_C09
4	103.719	81	4	-0.178	0.045	4.556	2183	WS00916_C09
5	16.219	93	1	-0.283	0.048	4.066	2183	WS00916_C09
5	84.347	105	2	-0.163	0.042	4.596	2183	WS00916_C09
6	45.897	117	1	0.324	0.085	7.008	2183	WS00916_C09
6	49.100	118	4	0.572	0.240	6.787	2183	WS00916_C09
6	54.066	119	4	0.224	0.062	5.949	2183	WS00916_C09
9	0.000	166	1	0.244	0.058	4.845	2183	WS00916_C09
10	70.246	190	2	0.379	0.091	9.345	2183	WS00916_C09
10	80.088	194	2	-0.231	0.058	6.113	2183	WS00916_C09
11	40.960	210	2	0.484	0.109	6.615	2183	WS00916_C09
11	40.960	210	4	-0.254	0.060	5.737	2183	WS00916_C09
11	42.600	213	3	-0.179	0.046	4.163	2183	WS00916_C09
13	1.815	244	4	0.279	0.044	4.386	2183	WS00916_C09
3	47.928	49	1	0.232	0.051	4.764	2184	WS00916_D02
5	31.191	95	1	-0.247	0.057	5.358	2184	WS00916_D02
10	78.479	193	3	-0.342	0.056	5.866	2184	WS00916_D02
10	86.151	195	3	0.200	0.038	3.942	2184	WS00916_D02
3	47.928	49	1	0.221	0.070	5.785	2185	WS00916_E16
3	116.958	63	4	0.214	0.061	5.535	2185	WS00916_E16
8	11.974	150	1	-0.239	0.070	5.832	2185	WS00916_E16
8	78.389	157	1	0.234	0.048	3.959	2185	WS00916_E16
9	59.963	177	4	-0.250	0.054	4.183	2185	WS00916_E16
10	78.479	193	3	-0.486	0.089	8.677	2185	WS00916_E16
10	86.151	195	3	0.204	0.058	5.587	2185	WS00916_E16
12	22.441	227	1	-0.206	0.051	4.320	2185	WS00916_E16
13	3.386	245	1	0.274	0.061	5.128	2185	WS00916_E16
2	48.839	29	4	-0.304	0.068	7.323	2186	WS00916_F14
2	101.076	35	4	0.219	0.043	4.107	2186	WS00916_F14
3	9.953	42	4	0.274	0.070	7.457	2186	WS00916_F14
4	82.462	78	1	-0.345	0.144	10.142	2186	WS00916_F14
4	142.917	85	1	0.368	0.100	7.154	2186	WS00916_F14
5	54.072	100	4	0.227	0.041	4.494	2186	WS00916_F14
7	15.555	130	2	0.233	0.041	4.848	2186	WS00916_F14
10	78.479	193	3	-0.356	0.058	4.562	2186	WS00916_F14
11	40.960	210	2	0.556	0.109	8.305	2186	WS00916_F14
1	45.794	9	3	-0.274	0.065	4.746	2187	WS00727_G22
3	15.147	44	2	-0.304	0.073	5.221	2187	WS00727_G22
4	103.719	81	4	-0.200	0.048	4.542	2187	WS00727_G22
6	45.897	117	1	0.210	0.053	4.467	2187	WS00727_G22
7	15.555	130	2	0.286	0.077	8.095	2187	WS00727_G22
11	0.000	199	2	-0.240	0.045	4.615	2187	WS00727_G22
2	120.617	38	3	0.245	0.048	5.714	2188	WS00727_H24
3	47.928	49	1	0.284	0.080	6.784	2188	WS00727_H24
4	6.032	72	4	-0.178	0.062	4.216	2188	WS00727_H24
5	31.191	95	1	-0.234	0.056	4.728	2188	WS00727_H24
6	95.249	126	1	0.417	0.142	7.751	2188	WS00727_H24

8	50.001	154	1	0.285	0.075	7.088	2188	WS00727_H24
9	59.963	177	4	-0.246	0.061	4.205	2188	WS00727_H24
10	70.246	190	2	0.263	0.047	4.919	2188	WS00727_H24
1	38.326	6	2	0.246	0.042	4.016	2189	WS00727_M21
1	62.128	11	1	-0.488	0.096	5.191	2189	WS00727_M21
1	131.686	23	1	0.666	0.140	7.578	2189	WS00727_M21
2	48.839	29	4	0.240	0.051	6.533	2189	WS00727_M21
3	15.147	44	2	-0.275	0.106	6.183	2189	WS00727_M21
3	47.928	49	1	0.225	0.064	5.893	2189	WS00727_M21
3	112.868	61	2	0.162	0.044	4.157	2189	WS00727_M21
3	114.747	62	2	0.220	0.041	3.921	2189	WS00727_M21
3	148.745	68	4	-0.282	0.067	8.053	2189	WS00727_M21
5	0.000	89	3	0.237	0.052	4.144	2189	WS00727_M21
5	57.017	102	2	-0.214	0.044	4.365	2189	WS00727_M21
6	106.702	128	2	-0.467	0.144	6.177	2189	WS00727_M21
8	0.000	148	4	-0.335	0.072	8.017	2189	WS00727_M21
8	87.463	158	1	-0.242	0.052	4.812	2189	WS00727_M21
9	81.073	183	3	-0.202	0.056	4.376	2189	WS00727_M21
11	17.088	203	1	0.318	0.078	7.185	2189	WS00727_M21
11	17.088	203	2	0.181	0.042	4.195	2189	WS00727_M21
11	27.405	204	1	-0.315	0.041	3.932	2189	WS00727_M21
11	29.069	205	1	-0.326	0.068	6.422	2189	WS00727_M21
11	45.989	216	1	0.259	0.086	7.870	2189	WS00727_M21
11	46.026	217	1	0.245	0.069	6.307	2189	WS00727_M21
12	0.000	233	1	0.247	0.050	4.625	2189	WS00727_M21
6	95.249	126	1	0.201	0.068	4.300	2190	WS00727_N23
7	59.789	137	3	-0.276	0.059	5.870	2190	WS00727_N23
7	70.278	141	3	0.271	0.059	5.888	2190	WS00727_N23
8	11.974	150	1	-0.262	0.051	5.868	2190	WS00727_N23
10	78.479	193	3	-0.313	0.067	6.617	2190	WS00727_N23
1	38.326	6	2	0.184	0.039	4.059	2191	WS00832_C15
2	120.617	38	3	0.180	0.037	4.346	2191	WS00832_C15
2	127.147	40	3	-0.133	0.034	3.916	2191	WS00832_C15
3	47.928	49	1	0.229	0.050	4.270	2191	WS00832_C15
6	89.401	125	1	-0.305	0.053	4.664	2191	WS00832_C15
6	95.249	126	1	0.482	0.173	10.037	2191	WS00832_C15
8	11.974	150	1	-0.256	0.057	4.897	2191	WS00832_C15
8	50.001	154	1	0.290	0.076	7.111	2191	WS00832_C15
10	35.204	189	2	0.248	0.059	4.922	2191	WS00832_C15
10	78.479	193	3	-0.514	0.100	11.836	2191	WS00832_C15
10	86.151	195	3	0.294	0.113	13.214	2191	WS00832_C15
2	126.181	39	2	0.241	0.055	5.533	2192	WS00832_D18
2	126.181	39	4	-0.409	0.094	4.766	2192	WS00832_D18
2	127.147	40	3	0.283	0.054	5.923	2192	WS00832_D18
5	3.484	90	2	0.326	0.093	4.430	2192	WS00832_D18
9	28.608	170	3	0.349	0.070	7.769	2192	WS00832_D18
10	35.204	189	1	-0.302	0.062	5.363	2192	WS00832_D18
10	70.246	190	2	0.307	0.067	6.988	2192	WS00832_D18
10	78.479	193	3	-0.660	0.192	21.000	2192	WS00832_D18
10	80.088	194	2	-0.227	0.041	4.776	2192	WS00832_D18
10	86.151	195	3	0.359	0.155	16.889	2192	WS00832_D18
10	93.347	196	2	0.606	0.156	6.730	2192	WS00832_D18
11	44.884	215	3	-0.239	0.066	7.187	2192	WS00832_D18
3	148.745	68	1	-0.247	0.031	4.191	2193	WS00832_I21

3	154.505	69	1	-0.247	0.030	4.143	2193	WS00832_I21
4	142.917	85	1	0.204	0.032	4.375	2193	WS00832_I21
12	22.629	237	1	-0.241	0.030	4.159	2193	WS00832_I21
12	32.473	238	1	-0.246	0.031	4.231	2193	WS00832_I21
3	112.868	61	2	0.188	0.036	4.610	2194	WS00832_L19
5	54.072	100	4	-0.193	0.057	4.313	2194	WS00832_L19
5	56.008	101	2	0.289	0.072	9.632	2194	WS00832_L19
5	57.017	102	2	-0.320	0.065	8.529	2194	WS00832_L19
9	59.963	177	4	-0.298	0.090	7.253	2194	WS00832_L19
10	78.479	193	3	-0.335	0.068	7.605	2194	WS00832_L19
10	86.151	195	3	0.185	0.040	4.499	2194	WS00832_L19
1	43.578	8	2	0.233	0.034	4.220	2195	WS00821_P11
3	47.928	49	1	0.301	0.082	5.284	2195	WS00821_P11
3	154.505	69	1	0.351	0.074	4.910	2195	WS00821_P11
4	6.032	72	4	-0.159	0.035	4.009	2195	WS00821_P11
6	106.702	128	2	0.433	0.164	5.407	2195	WS00821_P11
7	75.883	144	2	0.382	0.047	4.564	2195	WS00821_P11
8	39.090	152	4	0.183	0.045	5.257	2195	WS00821_P11
9	28.608	170	2	-0.252	0.048	6.072	2195	WS00821_P11
9	49.013	173	2	0.166	0.034	4.379	2195	WS00821_P11
10	104.214	198	4	-0.278	0.034	4.936	2195	WS00821_P11
11	0.000	199	2	0.232	0.037	4.720	2195	WS00821_P11
14	43.392	250	2	0.194	0.049	6.300	2195	WS00821_P11
1	131.686	23	1	0.481	0.072	4.289	2196	WS01027_G13
9	28.608	170	3	-0.297	0.086	6.062	2196	WS01027_G13
10	21.864	188	1	-0.332	0.038	3.993	2196	WS01027_G13
10	80.088	194	2	0.167	0.044	4.202	2196	WS01027_G13
10	93.347	196	2	-0.437	0.159	4.026	2196	WS01027_G13
12	22.629	237	1	-0.259	0.047	4.863	2196	WS01027_G13
14	0.000	246	2	-0.219	0.052	4.541	2196	WS01027_G13
14	22.402	248	4	-0.424	0.053	6.375	2196	WS01027_G13
2	48.839	29	1	-0.343	0.073	9.069	2197	WS00823_K18
3	69.071	52	1	-0.250	0.045	4.768	2197	WS00823_K18
9	36.227	165	3	0.227	0.031	4.262	2197	WS00823_K18
10	9.580	187	3	0.341	0.083	8.916	2197	WS00823_K18
10	35.204	189	1	0.253	0.036	4.489	2197	WS00823_K18
10	93.347	196	1	-0.426	0.045	4.864	2197	WS00823_K18
12	0.000	233	1	0.229	0.045	5.577	2197	WS00823_K18
1	69.854	13	2	-0.227	0.045	4.355	2198	WS00823_P16
1	71.592	14	2	-0.233	0.049	4.696	2198	WS00823_P16
1	131.686	23	2	-0.485	0.139	5.293	2198	WS00823_P16
4	75.958	77	3	-0.323	0.092	5.044	2198	WS00823_P16
7	73.169	142	1	0.475	0.094	3.940	2198	WS00823_P16
8	50.001	154	1	0.278	0.097	9.360	2198	WS00823_P16
11	56.921	219	1	-0.282	0.098	4.079	2198	WS00823_P16
14	0.000	246	1	0.188	0.050	4.650	2198	WS00823_P16
1	29.800	5	2	-0.289	0.045	4.994	2199	WS00822_O24
1	38.326	6	2	0.280	0.046	5.121	2199	WS00822_O24
2	120.617	38	3	0.235	0.056	5.993	2199	WS00822_O24
4	0.000	71	4	-0.240	0.051	4.167	2199	WS00822_O24
8	50.001	154	1	0.282	0.081	7.432	2199	WS00822_O24
9	59.963	177	4	-0.288	0.076	5.679	2199	WS00822_O24
10	70.246	190	2	0.280	0.057	6.481	2199	WS00822_O24
10	78.479	193	3	-0.344	0.065	6.947	2199	WS00822_O24

1	2.788	2	4	0.249	0.070	5.549	2200	WS00823_E14
4	6.032	72	4	-0.216	0.077	6.394	2200	WS00823_E14
8	50.001	154	1	0.300	0.075	6.151	2200	WS00823_E14
10	70.246	190	2	0.370	0.094	10.815	2200	WS00823_E14
11	35.604	207	3	-0.161	0.045	4.726	2200	WS00823_E14
11	37.143	209	3	0.140	0.037	3.842	2200	WS00823_E14
3	95.157	59	2	0.182	0.035	4.141	2201	WS00916_K22
8	11.974	150	1	-0.248	0.069	4.547	2201	WS00916_K22
9	49.013	173	2	0.268	0.057	6.626	2201	WS00916_K22
9	59.963	177	4	-0.376	0.089	7.112	2201	WS00916_K22
1	45.794	9	1	-0.390	0.081	6.793	2202	WS00917_B03
1	50.955	10	1	0.224	0.062	5.184	2202	WS00917_B03
1	127.805	21	3	-0.235	0.050	4.839	2202	WS00917_B03
4	103.719	81	4	-0.273	0.051	4.282	2202	WS00917_B03
4	116.928	82	2	0.237	0.042	4.309	2202	WS00917_B03
5	0.000	89	3	0.293	0.057	4.529	2202	WS00917_B03
5	57.017	102	4	0.284	0.050	4.225	2202	WS00917_B03
6	45.897	117	1	0.298	0.051	4.483	2202	WS00917_B03
6	49.100	118	2	0.281	0.115	5.080	2202	WS00917_B03
6	106.702	128	3	0.386	0.083	7.946	2202	WS00917_B03
8	50.001	154	2	-0.180	0.043	3.997	2202	WS00917_B03
8	87.463	158	2	0.226	0.044	4.571	2202	WS00917_B03
11	40.960	210	2	0.451	0.098	6.267	2202	WS00917_B03
4	75.958	77	3	-0.356	0.090	4.909	2203	WS00825_O21
4	142.917	85	1	-0.189	0.047	4.138	2203	WS00825_O21
8	11.974	150	1	-0.218	0.055	4.861	2203	WS00825_O21
10	78.479	193	3	-0.351	0.046	4.762	2203	WS00825_O21
12	22.441	227	1	-0.248	0.062	5.501	2203	WS00825_O21
12	14.406	230	4	-0.234	0.062	3.923	2203	WS00825_O21
3	47.928	49	1	0.252	0.077	7.188	2204	WS00825_P13
4	116.928	82	2	-0.217	0.050	4.921	2204	WS00825_P13
6	25.742	113	3	-0.266	0.059	5.626	2204	WS00825_P13
6	25.914	114	1	-0.286	0.088	8.273	2204	WS00825_P13
8	50.001	154	1	0.202	0.048	4.586	2204	WS00825_P13
9	59.963	177	4	-0.284	0.088	7.691	2204	WS00825_P13
14	46.637	252	3	0.219	0.048	4.631	2204	WS00825_P13
1	43.578	8	2	-0.162	0.043	4.506	2205	WS0051_C19
3	47.928	49	1	0.177	0.052	4.382	2205	WS0051_C19
4	75.958	77	3	-0.448	0.084	5.304	2205	WS0051_C19
4	116.928	82	2	-0.308	0.034	4.007	2205	WS0051_C19
4	124.132	83	1	0.411	0.074	4.152	2205	WS0051_C19
6	95.249	126	1	0.375	0.177	5.770	2205	WS0051_C19
7	70.278	141	3	-0.323	0.066	6.256	2205	WS0051_C19
8	87.463	158	2	-0.234	0.072	7.664	2205	WS0051_C19
10	104.214	198	4	-0.273	0.040	5.312	2205	WS0051_C19
11	0.000	199	3	-0.286	0.051	5.078	2205	WS0051_C19
3	137.891	64	3	0.194	0.055	5.636	2206	WS0051_E06
4	116.928	82	2	-0.364	0.062	6.422	2206	WS0051_E06
7	70.278	141	3	-0.331	0.073	6.984	2206	WS0051_E06
7	75.883	144	2	0.475	0.101	6.747	2206	WS0051_E06
10	35.204	189	1	-0.339	0.070	6.266	2206	WS0051_E06
10	78.479	193	4	-0.192	0.042	4.809	2206	WS0051_E06
11	0.000	199	3	-0.313	0.054	5.477	2206	WS0051_E06
11	17.088	203	1	-0.226	0.046	4.067	2206	WS0051_E06

11	40.960	210	2	-0.345	0.062	3.966	2206	WS0051_E06
12	51.382	242	1	-0.436	0.111	4.071	2206	WS0051_E06
13	1.815	244	4	-0.199	0.067	7.075	2206	WS0051_E06
3	47.928	49	1	0.243	0.077	6.427	2207	WS0051_J13
5	31.191	95	1	-0.275	0.100	8.315	2207	WS0051_J13
10	78.479	193	3	-0.373	0.049	5.676	2207	WS0051_J13
3	47.928	49	1	0.305	0.076	7.734	2208	WS0051_L03
4	124.132	83	1	0.461	0.094	4.660	2208	WS0051_L03
10	78.479	193	3	-0.248	0.040	4.259	2208	WS0051_L03
10	86.151	195	3	0.220	0.050	5.370	2208	WS0051_L03
1	45.794	9	3	0.219	0.052	4.295	2209	WS0051_P20
1	62.128	11	2	0.399	0.068	6.762	2209	WS0051_P20
5	31.191	95	1	-0.222	0.145	5.531	2209	WS0051_P20
10	78.479	193	3	-0.390	0.054	6.667	2209	WS0051_P20
1	95.572	17	2	0.256	0.043	5.474	2210	WS0052_B17
2	48.839	29	4	0.307	0.073	8.887	2210	WS0052_B17
4	0.000	71	4	-0.167	0.033	3.927	2210	WS0052_B17
7	15.555	130	2	-0.201	0.030	3.882	2210	WS0052_B17
7	75.883	144	2	0.387	0.040	4.178	2210	WS0052_B17
7	105.477	147	2	-0.205	0.039	4.052	2210	WS0052_B17
11	0.000	199	2	0.249	0.045	5.728	2210	WS0052_B17
3	80.211	55	4	0.226	0.054	5.417	2211	WS0075_G14
4	55.925	76	1	-0.361	0.106	11.598	2211	WS0075_G14
4	82.462	78	1	-0.238	0.052	5.855	2211	WS0075_G14
4	82.462	78	2	-0.253	0.059	4.247	2211	WS0075_G14
4	97.576	80	2	0.200	0.067	5.791	2211	WS0075_G14
8	11.974	150	1	-0.322	0.085	9.644	2211	WS0075_G14
11	27.405	204	1	0.241	0.037	4.173	2211	WS0075_G14
12	22.441	227	1	-0.266	0.039	4.555	2211	WS0075_G14
12	28.493	228	2	-0.260	0.091	4.255	2211	WS0075_G14
3	14.500	43	2	-0.263	0.077	5.585	2212	WS0075_H02
4	6.032	72	4	-0.249	0.091	6.211	2212	WS0075_H02
4	142.917	85	1	-0.232	0.034	3.900	2212	WS0075_H02
6	95.249	126	1	0.319	0.073	5.942	2212	WS0075_H02
7	74.560	143	1	-0.294	0.068	7.782	2212	WS0075_H02
7	75.883	144	4	-0.277	0.062	4.242	2212	WS0075_H02
10	35.204	189	1	-0.292	0.054	6.190	2212	WS0075_H02
14	0.000	246	1	-0.239	0.050	5.771	2212	WS0075_H02
3	15.147	44	2	0.231	0.117	7.480	2213	WS0075_J14
3	20.512	46	2	0.373	0.049	6.169	2213	WS0075_J14
6	87.891	124	2	-0.231	0.047	5.164	2213	WS0075_J14
8	50.001	154	2	0.230	0.053	5.501	2213	WS0075_J14
8	87.463	158	2	-0.228	0.052	5.600	2213	WS0075_J14
10	35.204	189	2	0.136	0.044	4.867	2213	WS0075_J14
10	104.214	198	4	-0.284	0.046	6.507	2213	WS0075_J14
11	27.405	204	1	0.268	0.049	4.029	2213	WS0075_J14
11	29.069	205	1	0.231	0.045	4.145	2213	WS0075_J14
12	22.441	227	1	-0.163	0.049	4.070	2213	WS0075_J14
1	95.572	17	2	0.222	0.040	3.968	2214	WS0075_K05
4	55.925	76	1	-0.208	0.049	4.216	2214	WS0075_K05
4	142.917	85	1	-0.287	0.064	5.507	2214	WS0075_K05
11	42.600	213	3	-0.209	0.045	4.796	2214	WS0075_K05
11	60.464	221	2	0.303	0.070	6.590	2214	WS0075_K05
1	22.419	4	3	0.277	0.068	4.973	2215	WS0075_M01

3	9.953	42	3	0.237	0.037	3.909	2215	WS0075_M01
3	15.147	44	2	0.227	0.072	4.588	2215	WS0075_M01
4	55.925	76	1	-0.303	0.081	7.089	2215	WS0075_M01
7	15.555	130	2	-0.333	0.073	6.766	2215	WS0075_M01
9	36.227	165	3	-0.241	0.058	6.003	2215	WS0075_M01
10	21.864	188	1	-0.232	0.041	4.061	2215	WS0075_M01
11	27.405	204	1	0.249	0.061	6.002	2215	WS0075_M01
1	77.177	15	2	0.204	0.041	4.491	2216	WS0075_M16
2	127.147	40	3	0.207	0.037	4.339	2216	WS0075_M16
5	52.820	99	3	0.263	0.042	4.770	2216	WS0075_M16
12	22.629	237	1	-0.416	0.167	13.068	2216	WS0075_M16
12	32.473	238	1	-0.470	0.213	16.748	2216	WS0075_M16
14	46.637	252	4	-0.330	0.086	6.139	2216	WS0075_M16
1	50.955	10	1	-0.246	0.053	5.402	2217	WS0075_O12
2	120.617	38	3	0.256	0.040	4.891	2217	WS0075_O12
2	127.147	40	3	-0.170	0.046	5.608	2217	WS0075_O12
5	56.008	101	1	-0.169	0.039	3.980	2217	WS0075_O12
6	89.401	125	1	-0.295	0.054	5.602	2217	WS0075_O12
6	95.249	126	1	0.301	0.063	5.272	2217	WS0075_O12
8	50.001	154	1	0.288	0.074	8.276	2217	WS0075_O12
9	0.000	163	1	-0.454	0.067	5.607	2217	WS0075_O12
9	0.000	166	2	0.273	0.051	4.131	2217	WS0075_O12
9	49.013	173	2	0.248	0.062	4.621	2217	WS0075_O12
10	9.580	187	3	-0.330	0.065	4.443	2217	WS0075_O12
10	78.479	193	3	-0.470	0.061	7.620	2217	WS0075_O12
10	86.151	195	3	0.222	0.069	8.351	2217	WS0075_O12
11	64.014	222	3	-0.270	0.066	8.024	2217	WS0075_O12
14	22.402	248	3	0.184	0.033	4.032	2217	WS0075_O12
1	2.788	2	3	-0.236	0.042	4.779	2218	WS0075_P21
2	120.617	38	3	0.259	0.040	4.606	2218	WS0075_P21
3	47.928	49	1	0.227	0.064	6.606	2218	WS0075_P21
6	45.897	117	1	0.261	0.043	4.480	2218	WS0075_P21
6	95.249	126	1	0.231	0.059	4.012	2218	WS0075_P21
8	11.974	150	1	-0.247	0.067	6.852	2218	WS0075_P21
9	55.358	175	3	0.419	0.065	4.618	2218	WS0075_P21
10	9.580	187	3	-0.286	0.073	4.899	2218	WS0075_P21
10	78.479	193	3	-0.507	0.098	11.274	2218	WS0075_P21
11	35.604	207	3	-0.258	0.074	8.445	2218	WS0075_P21
11	37.143	209	3	0.220	0.054	6.187	2218	WS0075_P21
11	42.600	213	3	-0.238	0.060	6.792	2218	WS0075_P21
1	15.589	3	2	0.188	0.039	4.617	2219	WS00820_C09
2	102.814	36	2	-0.253	0.042	4.365	2219	WS00820_C09
3	47.928	49	1	0.249	0.075	5.100	2219	WS00820_C09
4	116.928	82	2	-0.292	0.060	7.162	2219	WS00820_C09
6	89.401	125	1	-0.281	0.059	4.084	2219	WS00820_C09
9	59.963	177	4	-0.273	0.085	6.439	2219	WS00820_C09
9	69.046	181	2	-0.292	0.075	5.367	2219	WS00820_C09
3	47.928	49	1	0.232	0.060	5.012	2220	WS00820_D04
3	95.157	59	2	0.192	0.041	4.104	2220	WS00820_D04
8	11.974	150	1	-0.248	0.058	4.851	2220	WS00820_D04
8	50.001	154	1	0.209	0.047	4.225	2220	WS00820_D04
9	59.963	177	4	-0.237	0.048	4.158	2220	WS00820_D04
10	70.246	190	2	0.259	0.046	4.832	2220	WS00820_D04
10	78.479	193	3	-0.332	0.050	4.956	2220	WS00820_D04

11	43.491	214	3	0.160	0.040	3.999	2220	WS00820_D04
11	64.014	222	3	-0.305	0.083	8.220	2220	WS00820_D04
2	48.839	29	1	0.193	0.047	4.217	2221	WS00820_E18
3	47.928	49	1	0.228	0.059	5.287	2221	WS00820_E18
6	25.914	114	1	-0.235	0.061	5.421	2221	WS00820_E18
6	89.401	125	1	-0.319	0.045	4.166	2221	WS00820_E18
9	0.000	166	1	-0.140	0.049	4.383	2221	WS00820_E18
9	59.963	177	4	-0.310	0.076	7.757	2221	WS00820_E18
10	70.246	190	2	0.260	0.049	4.943	2221	WS00820_E18
10	78.479	193	3	-0.405	0.086	7.647	2221	WS00820_E18
10	86.151	195	3	0.171	0.057	4.988	2221	WS00820_E18
10	93.347	196	2	0.503	0.119	5.811	2221	WS00820_E18
11	9.237	202	4	0.152	0.040	3.995	2221	WS00820_E18
14	0.000	246	2	0.239	0.061	6.051	2221	WS00820_E18
1	50.955	10	1	-0.249	0.051	4.039	2222	WS00820_F06
1	95.572	17	2	-0.245	0.037	4.028	2222	WS00820_F06
3	9.953	42	4	0.281	0.064	5.432	2222	WS00820_F06
3	80.211	55	4	0.202	0.050	4.736	2222	WS00820_F06
4	75.958	77	3	-0.330	0.086	4.371	2222	WS00820_F06
4	124.132	83	1	0.528	0.160	5.826	2222	WS00820_F06
7	59.789	137	3	-0.168	0.043	4.007	2222	WS00820_F06
8	50.001	154	1	0.173	0.048	4.377	2222	WS00820_F06
10	78.479	193	3	-0.318	0.042	3.947	2222	WS00820_F06
10	86.151	195	3	0.177	0.043	3.952	2222	WS00820_F06
13	1.815	244	2	0.289	0.043	4.719	2222	WS00820_F06
3	47.928	49	1	0.262	0.062	6.631	2223	WS00820_H04
4	142.917	85	1	0.297	0.049	5.302	2223	WS00820_H04
5	31.191	95	1	-0.232	0.047	5.051	2223	WS00820_H04
5	86.669	106	4	0.341	0.097	4.216	2223	WS00820_H04
9	49.013	173	2	0.205	0.049	3.930	2223	WS00820_H04
10	78.479	193	3	-0.365	0.054	6.115	2223	WS00820_H04
10	86.151	195	3	0.230	0.053	5.943	2223	WS00820_H04
14	22.402	248	3	0.229	0.038	4.263	2223	WS00820_H04
14	22.402	248	4	-0.268	0.052	3.875	2223	WS00820_H04
14	44.898	251	3	0.269	0.046	5.145	2223	WS00820_H04
1	123.316	20	4	0.295	0.069	4.121	2224	WS00820_H16
2	120.617	38	3	0.182	0.039	3.861	2224	WS00820_H16
2	127.147	40	3	-0.160	0.047	4.673	2224	WS00820_H16
3	47.928	49	1	0.205	0.049	4.085	2224	WS00820_H16
3	95.157	59	2	0.245	0.065	6.392	2224	WS00820_H16
6	95.249	126	1	0.369	0.106	5.789	2224	WS00820_H16
8	50.001	154	1	0.224	0.055	4.012	2224	WS00820_H16
11	64.014	222	3	-0.264	0.069	6.867	2224	WS00820_H16
1	29.800	5	1	0.264	0.041	5.006	2225	WS00820_J07
2	63.261	30	3	-0.249	0.093	6.295	2225	WS00820_J07
4	124.132	83	1	-0.472	0.071	5.353	2225	WS00820_J07
5	86.669	106	4	0.424	0.084	5.940	2225	WS00820_J07
6	76.376	122	1	-0.411	0.072	5.476	2225	WS00820_J07
9	0.000	166	1	0.297	0.060	7.287	2225	WS00820_J07
9	81.073	183	1	0.376	0.086	10.372	2225	WS00820_J07
13	0.000	243	4	-0.557	0.155	11.781	2225	WS00820_J07
14	22.402	248	4	-0.321	0.049	5.531	2225	WS00820_J07
1	29.800	5	1	0.245	0.047	4.494	2226	WS00820_J19
3	47.928	49	1	0.271	0.056	5.458	2226	WS00820_J19

4	124.132	83	1	0.445	0.104	3.968	2226	WS00820_J19
5	31.191	95	1	-0.291	0.069	6.662	2226	WS00820_J19
6	89.401	125	1	-0.337	0.071	6.906	2226	WS00820_J19
6	95.249	126	1	0.383	0.108	6.331	2226	WS00820_J19
8	11.974	150	1	-0.331	0.079	7.710	2226	WS00820_J19
9	59.963	177	4	-0.204	0.052	4.534	2226	WS00820_J19
10	78.479	193	3	-0.234	0.042	4.517	2226	WS00820_J19
10	86.151	195	3	0.219	0.047	4.953	2226	WS00820_J19
11	35.604	207	3	-0.196	0.040	4.299	2226	WS00820_J19
14	46.637	252	3	0.207	0.040	4.252	2226	WS00820_J19
1	38.326	6	2	0.223	0.060	5.255	2227	WS00833_B16
2	120.617	38	3	0.171	0.042	4.090	2227	WS00833_B16
3	47.928	49	1	0.273	0.059	6.048	2227	WS00833_B16
6	69.073	121	3	-0.253	0.044	4.368	2227	WS00833_B16
6	95.249	126	1	0.545	0.205	12.003	2227	WS00833_B16
8	11.974	150	1	-0.333	0.082	8.382	2227	WS00833_B16
9	59.963	177	4	-0.274	0.060	4.756	2227	WS00833_B16
10	78.479	193	3	-0.322	0.048	4.738	2227	WS00833_B16
11	29.069	205	1	0.288	0.037	3.902	2227	WS00833_B16
1	71.592	14	2	-0.207	0.035	4.035	2228	WS00833_C19
1	82.301	16	3	-0.260	0.051	6.102	2228	WS00833_C19
3	141.443	65	3	-0.369	0.104	5.387	2228	WS00833_C19
6	54.066	119	4	-0.186	0.076	5.091	2228	WS00833_C19
7	105.477	147	2	0.217	0.094	4.332	2228	WS00833_C19
10	78.479	193	3	-0.353	0.059	7.078	2228	WS00833_C19
1	45.794	9	1	-0.214	0.039	4.454	2229	WS00833_I22
4	124.132	83	1	0.548	0.108	8.160	2229	WS00833_I22
4	142.917	85	1	-0.299	0.064	7.298	2229	WS00833_I22
8	11.974	150	1	-0.217	0.039	4.509	2229	WS00833_I22
12	22.441	227	1	-0.240	0.040	4.622	2229	WS00833_I22
12	14.406	230	4	-0.247	0.043	4.993	2229	WS00833_I22
13	0.000	243	4	0.340	0.063	4.786	2229	WS00833_I22
1	82.301	16	3	-0.236	0.045	4.789	2230	WS00833_L03
3	69.071	52	1	0.362	0.081	4.397	2230	WS00833_L03
7	15.555	130	2	-0.282	0.059	6.061	2230	WS00833_L03
9	17.951	169	1	0.247	0.049	4.499	2230	WS00833_L03
11	0.000	199	3	-0.499	0.126	11.252	2230	WS00833_L03
11	27.405	204	1	0.353	0.059	5.699	2230	WS00833_L03
11	29.069	205	1	0.311	0.068	6.353	2230	WS00833_L03
12	22.441	227	1	-0.259	0.043	4.012	2230	WS00833_L03
1	50.955	10	1	-0.152	0.041	4.168	2231	WS00833_O09
1	62.128	11	1	-0.621	0.106	7.410	2231	WS00833_O09
3	47.928	49	1	0.262	0.043	4.400	2231	WS00833_O09
3	80.211	55	4	0.207	0.045	4.376	2231	WS00833_O09
4	0.000	71	4	-0.145	0.046	4.447	2231	WS00833_O09
10	70.246	190	4	-0.303	0.076	3.951	2231	WS00833_O09
1	95.572	17	2	0.238	0.048	4.515	2232	WS00833_P05
3	148.745	68	1	0.213	0.051	4.679	2232	WS00833_P05
7	73.169	142	2	0.499	0.149	5.971	2232	WS00833_P05
7	75.883	144	2	-0.466	0.140	5.633	2232	WS00833_P05
9	0.000	166	1	0.164	0.042	3.902	2232	WS00833_P05
9	59.963	177	1	-0.268	0.043	3.990	2232	WS00833_P05
10	78.479	193	4	0.232	0.063	4.235	2232	WS00833_P05
11	43.491	214	3	0.214	0.036	4.355	2232	WS00833_P05

11	44.884	215	3	-0.285	0.043	5.186	2232	WS00833_P05
11	45.989	216	2	-0.188	0.042	4.000	2232	WS00833_P05
11	64.014	222	3	-0.336	0.066	7.917	2232	WS00833_P05
1	62.128	11	1	-0.406	0.059	4.191	2233	WS00938_D15
2	93.504	32	4	-0.341	0.047	4.576	2233	WS00938_D15
3	9.953	42	4	0.259	0.075	9.430	2233	WS00938_D15
4	55.925	76	1	-0.236	0.046	5.322	2233	WS00938_D15
6	45.897	117	1	0.289	0.040	4.619	2233	WS00938_D15
6	49.100	118	4	0.213	0.067	4.923	2233	WS00938_D15
7	74.560	143	2	-0.247	0.052	4.075	2233	WS00938_D15
7	75.883	144	2	-0.630	0.168	10.401	2233	WS00938_D15
7	75.883	144	4	0.335	0.046	5.842	2233	WS00938_D15
9	36.227	165	4	-0.254	0.027	4.001	2233	WS00938_D15
9	0.000	166	1	0.188	0.068	7.585	2233	WS00938_D15
10	78.479	193	4	0.296	0.055	7.035	2233	WS00938_D15
11	56.921	219	1	0.222	0.072	5.136	2233	WS00938_D15
14	46.637	252	4	-0.280	0.029	4.519	2233	WS00938_D15
1	2.788	2	3	-0.221	0.036	3.846	2234	WS00938_E23
9	0.000	166	1	0.209	0.067	5.718	2234	WS00938_E23
11	35.604	207	3	-0.189	0.039	4.009	2234	WS00938_E23
12	22.441	227	3	0.299	0.048	4.936	2234	WS00938_E23
14	44.898	251	3	0.241	0.038	3.937	2234	WS00938_E23
4	55.925	76	1	-0.237	0.057	5.862	2235	WS0101_G24
5	0.000	89	1	-0.320	0.060	6.224	2235	WS0101_G24
5	16.219	93	1	-0.226	0.054	5.481	2235	WS0101_G24
6	45.897	117	1	0.287	0.043	4.697	2235	WS0101_G24
6	49.100	118	4	0.248	0.111	5.001	2235	WS0101_G24
6	81.996	123	3	0.342	0.070	4.325	2235	WS0101_G24
7	75.883	144	2	-0.430	0.086	5.016	2235	WS0101_G24
11	40.960	210	2	0.370	0.069	4.022	2235	WS0101_G24
11	40.960	210	4	-0.327	0.052	5.093	2235	WS0101_G24
12	38.543	232	4	0.453	0.139	5.798	2235	WS0101_G24
1	22.419	4	3	0.408	0.077	5.576	2236	WS0101_M04
1	45.794	9	1	-0.250	0.053	5.177	2236	WS0101_M04
1	77.177	15	2	0.225	0.048	4.406	2236	WS0101_M04
3	9.953	42	3	0.220	0.071	8.166	2236	WS0101_M04
3	141.443	65	3	0.384	0.084	5.961	2236	WS0101_M04
8	39.090	152	4	0.169	0.058	4.171	2236	WS0101_M04
8	95.937	159	3	-0.148	0.034	3.932	2236	WS0101_M04
11	0.000	199	3	-0.281	0.035	3.915	2236	WS0101_M04
11	27.405	204	1	0.304	0.042	4.125	2236	WS0101_M04
13	1.815	244	2	-0.230	0.044	4.061	2236	WS0101_M04
1	127.805	21	4	0.190	0.053	4.765	2237	WS0079_M05
3	17.158	45	1	0.241	0.059	5.100	2237	WS0079_M05
3	104.681	60	2	-0.204	0.044	4.466	2237	WS0079_M05
3	114.747	62	2	0.254	0.036	3.873	2237	WS0079_M05
4	124.132	83	1	0.462	0.111	4.306	2237	WS0079_M05
4	142.917	85	1	-0.342	0.070	6.290	2237	WS0079_M05
6	49.100	118	4	0.437	0.207	7.968	2237	WS0079_M05
6	87.891	124	2	0.252	0.066	6.772	2237	WS0079_M05
6	89.401	125	2	0.269	0.050	4.959	2237	WS0079_M05
7	59.789	137	3	-0.334	0.099	9.086	2237	WS0079_M05
8	95.937	159	3	0.155	0.048	4.363	2237	WS0079_M05
10	21.864	188	1	0.312	0.086	7.631	2237	WS0079_M05

11	46.026	217	1	-0.245	0.054	4.670	2237	WS0079_M05
14	43.392	250	1	-0.334	0.110	5.185	2237	WS0079_M05
1	29.800	5	2	-0.192	0.045	4.494	2238	WS00938_M19
1	123.316	20	4	0.250	0.079	4.834	2238	WS00938_M19
3	80.211	55	4	0.252	0.050	4.814	2238	WS00938_M19
4	0.000	71	4	-0.263	0.056	5.388	2238	WS00938_M19
8	11.974	150	1	-0.270	0.062	5.420	2238	WS00938_M19
9	59.963	177	4	-0.219	0.050	4.491	2238	WS00938_M19
10	70.246	190	2	0.280	0.087	9.010	2238	WS00938_M19
11	64.014	222	3	-0.262	0.068	6.222	2238	WS00938_M19
1	123.316	20	4	0.301	0.083	5.984	2239	WS0092_C13
2	93.504	32	2	-0.299	0.059	6.274	2239	WS0092_C13
3	47.928	49	1	0.195	0.055	4.680	2239	WS0092_C13
8	0.000	148	4	-0.179	0.031	3.877	2239	WS0092_C13
8	50.001	154	1	0.240	0.073	6.322	2239	WS0092_C13
8	62.580	155	1	0.342	0.103	5.343	2239	WS0092_C13
8	78.389	157	1	0.264	0.059	5.074	2239	WS0092_C13
8	87.463	158	1	-0.228	0.049	4.233	2239	WS0092_C13
8	87.463	158	2	0.285	0.058	5.985	2239	WS0092_C13
9	59.963	177	4	-0.286	0.046	5.825	2239	WS0092_C13
14	0.000	246	2	0.213	0.041	3.999	2239	WS0092_C13
14	44.898	251	1	0.235	0.046	3.957	2239	WS0092_C13
1	127.805	21	4	0.308	0.079	9.409	2240	WS00939_G01
1	131.686	23	1	0.517	0.069	5.096	2240	WS00939_G01
5	42.979	97	1	-0.294	0.035	4.121	2240	WS00939_G01
6	45.897	117	1	0.393	0.066	7.849	2240	WS00939_G01
8	11.974	150	1	-0.208	0.038	4.367	2240	WS00939_G01
9	0.000	166	1	0.141	0.036	4.196	2240	WS00939_G01
11	43.491	214	4	0.174	0.044	5.626	2240	WS00939_G01
11	44.884	215	4	-0.164	0.031	4.059	2240	WS00939_G01
11	56.921	219	1	0.421	0.168	12.152	2240	WS00939_G01
11	57.661	220	4	-0.322	0.073	8.904	2240	WS00939_G01
11	64.014	222	4	0.350	0.051	5.694	2240	WS00939_G01
12	14.406	230	4	-0.379	0.061	5.400	2240	WS00939_G01
14	0.000	246	1	-0.251	0.045	5.238	2240	WS00939_G01
14	22.402	248	4	-0.328	0.043	5.223	2240	WS00939_G01
14	44.898	251	1	0.365	0.057	6.868	2240	WS00939_G01
3	141.443	65	4	-0.385	0.115	4.418	2241	WS00813_C17
3	144.593	66	4	-0.210	0.053	5.069	2241	WS00813_C17
4	0.000	71	4	-0.218	0.040	4.008	2241	WS00813_C17
4	124.132	83	1	0.456	0.118	4.251	2241	WS00813_C17
10	70.246	190	2	0.234	0.039	4.307	2241	WS00813_C17
10	78.479	193	3	-0.277	0.058	5.032	2241	WS00813_C17
1	43.578	8	2	0.257	0.058	5.723	2242	WS00813_D17
3	137.891	64	3	-0.293	0.076	6.178	2242	WS00813_D17
4	75.958	77	3	0.284	0.105	4.229	2242	WS00813_D17
7	91.487	146	3	0.228	0.054	4.703	2242	WS00813_D17
11	43.491	214	4	0.215	0.049	4.226	2242	WS00813_D17
2	120.617	38	3	0.196	0.047	5.254	2243	WS00813_F13
3	47.928	49	1	0.206	0.054	4.639	2243	WS00813_F13
3	112.868	61	2	0.247	0.038	3.961	2243	WS00813_F13
4	75.958	77	3	-0.381	0.093	5.603	2243	WS00813_F13
10	104.214	198	4	-0.349	0.059	4.258	2243	WS00813_F13
11	17.088	203	1	-0.249	0.072	6.245	2243	WS00813_F13

11	29.069	205	1	0.383	0.090	8.015	2243	WS00813_F13
11	45.989	216	1	-0.263	0.063	5.465	2243	WS00813_F13
1	45.794	9	1	-0.331	0.089	5.741	2244	WS00813_G11
2	102.814	36	2	-0.373	0.091	11.512	2244	WS00813_G11
2	126.181	39	2	0.236	0.034	4.159	2244	WS00813_G11
4	116.928	82	4	0.264	0.049	4.783	2244	WS00813_G11
6	87.891	124	2	0.195	0.046	5.670	2244	WS00813_G11
6	89.401	125	2	0.322	0.049	6.057	2244	WS00813_G11
6	106.702	128	2	-0.463	0.084	5.928	2244	WS00813_G11
8	87.463	158	2	0.290	0.057	6.763	2244	WS00813_G11
9	17.951	169	2	0.301	0.048	5.650	2244	WS00813_G11
10	78.479	193	3	0.303	0.055	4.815	2244	WS00813_G11
13	0.000	243	4	0.347	0.092	4.089	2244	WS00813_G11
1	2.788	2	4	0.174	0.034	3.879	2245	WS00813_K03
3	95.157	59	2	0.228	0.048	4.975	2245	WS00813_K03
5	31.191	95	1	-0.217	0.068	5.276	2245	WS00813_K03
6	95.249	126	1	0.291	0.119	4.725	2245	WS00813_K03
8	50.001	154	1	0.189	0.051	4.447	2245	WS00813_K03
9	59.963	177	4	-0.212	0.036	3.999	2245	WS00813_K03
13	3.386	245	1	0.240	0.051	3.997	2245	WS00813_K03
1	45.794	9	1	-0.235	0.059	5.049	2246	WS00813_L21
2	120.617	38	3	0.274	0.069	5.989	2246	WS00813_L21
2	127.147	40	3	-0.195	0.058	5.040	2246	WS00813_L21
3	95.157	59	2	0.209	0.040	4.356	2246	WS00813_L21
4	55.925	76	1	-0.246	0.072	6.185	2246	WS00813_L21
4	82.462	78	1	-0.301	0.061	5.220	2246	WS00813_L21
4	124.132	83	1	0.490	0.124	4.950	2246	WS00813_L21
8	11.974	150	1	-0.197	0.046	3.926	2246	WS00813_L21
9	59.963	177	4	-0.241	0.043	4.002	2246	WS00813_L21
11	0.000	199	3	-0.399	0.130	9.890	2246	WS00813_L21
12	22.441	227	1	-0.349	0.104	8.848	2246	WS00813_L21
12	38.543	232	4	0.238	0.102	4.068	2246	WS00813_L21
1	22.419	4	4	0.263	0.102	5.368	2247	WS00813_O17
1	45.794	9	3	-0.199	0.056	4.314	2247	WS00813_O17
1	95.572	17	2	-0.239	0.043	4.540	2247	WS00813_O17
2	95.880	34	1	-0.371	0.078	6.547	2247	WS00813_O17
3	15.147	44	2	-0.328	0.083	5.219	2247	WS00813_O17
3	20.512	46	2	-0.240	0.055	5.772	2247	WS00813_O17
3	148.745	68	4	0.270	0.034	5.482	2247	WS00813_O17
3	154.505	69	4	-0.308	0.071	4.582	2247	WS00813_O17
4	92.039	79	2	-0.249	0.045	4.698	2247	WS00813_O17
4	97.576	80	2	0.220	0.045	5.087	2247	WS00813_O17
6	69.073	121	3	-0.223	0.052	3.875	2247	WS00813_O17
7	59.789	137	3	0.238	0.056	4.183	2247	WS00813_O17
7	70.278	141	3	-0.293	0.097	7.197	2247	WS00813_O17
7	74.560	143	2	0.295	0.071	8.827	2247	WS00813_O17
7	91.487	146	3	-0.343	0.095	7.223	2247	WS00813_O17
9	59.963	177	4	-0.294	0.067	8.727	2247	WS00813_O17
9	81.073	183	1	-0.305	0.095	7.910	2247	WS00813_O17
12	22.441	227	3	-0.236	0.061	4.780	2247	WS00813_O17
13	3.386	245	1	0.296	0.043	3.884	2247	WS00813_O17
14	43.392	250	2	0.198	0.046	5.754	2247	WS00813_O17
14	46.637	252	3	0.256	0.111	8.194	2247	WS00813_O17
2	48.839	29	4	-0.280	0.049	5.298	2248	WS0097_A04

3	69.071	52	1	0.355	0.180	5.235	2248	WS0097_A04
3	154.505	69	1	0.282	0.076	5.165	2248	WS0097_A04
5	86.669	106	2	0.288	0.097	4.287	2248	WS0097_A04
7	105.477	147	2	0.275	0.078	5.936	2248	WS0097_A04
9	59.963	177	4	-0.310	0.076	8.533	2248	WS0097_A04
10	80.088	194	2	-0.229	0.033	4.040	2248	WS0097_A04
14	0.000	246	2	0.222	0.046	5.625	2248	WS0097_A04
1	133.992	24	2	-0.246	0.050	5.619	2249	WS00918_L10
2	93.504	32	2	-0.204	0.040	4.422	2249	WS00918_L10
2	101.076	35	4	0.300	0.040	4.506	2249	WS00918_L10
2	102.814	36	2	-0.361	0.126	14.232	2249	WS00918_L10
3	148.745	68	1	-0.357	0.097	7.279	2249	WS00918_L10
3	154.505	69	1	-0.367	0.100	7.696	2249	WS00918_L10
4	124.132	83	1	-0.473	0.132	4.483	2249	WS00918_L10
6	45.897	117	4	-0.306	0.054	4.787	2249	WS00918_L10
6	76.376	122	1	-0.393	0.115	3.937	2249	WS00918_L10
8	39.090	152	4	0.270	0.046	4.889	2249	WS00918_L10
9	55.358	175	1	0.280	0.085	6.318	2249	WS00918_L10
9	55.358	175	3	-0.501	0.141	6.560	2249	WS00918_L10
9	61.113	178	1	-0.418	0.149	5.050	2249	WS00918_L10
10	95.795	197	4	0.296	0.057	6.030	2249	WS00918_L10
10	104.214	198	4	-0.259	0.052	6.018	2249	WS00918_L10
11	43.491	214	4	0.162	0.040	3.864	2249	WS00918_L10
14	22.402	248	4	0.287	0.055	5.198	2249	WS00918_L10
3	47.928	49	1	0.285	0.067	6.056	2250	WS00918_M03
3	83.563	56	3	-0.181	0.048	4.722	2250	WS00918_M03
4	75.958	77	3	-0.268	0.091	4.173	2250	WS00918_M03
4	124.132	83	1	0.477	0.123	4.568	2250	WS00918_M03
4	142.917	85	1	-0.246	0.050	4.588	2250	WS00918_M03
9	59.963	177	4	-0.353	0.107	9.118	2250	WS00918_M03
10	80.088	194	2	-0.194	0.031	3.935	2250	WS00918_M03
12	0.000	233	1	0.182	0.048	4.295	2250	WS00918_M03
12	22.629	237	1	0.182	0.046	4.115	2250	WS00918_M03
3	95.157	59	2	0.273	0.072	8.117	2251	WS00918_N10
11	29.069	205	1	0.295	0.073	5.281	2251	WS00918_N10
12	22.441	227	1	-0.404	0.151	11.154	2251	WS00918_N10
1	45.794	9	1	-0.266	0.039	4.668	2252	WS00918_N21
3	9.953	42	3	0.229	0.064	6.815	2252	WS00918_N21
3	137.891	64	1	0.254	0.040	4.811	2252	WS00918_N21
4	124.132	83	1	0.473	0.094	4.684	2252	WS00918_N21
4	142.917	85	1	-0.381	0.074	9.097	2252	WS00918_N21
5	54.072	100	4	-0.318	0.117	9.503	2252	WS00918_N21
5	57.017	102	4	0.409	0.153	12.611	2252	WS00918_N21
6	81.996	123	3	0.217	0.056	3.943	2252	WS00918_N21
9	61.113	178	1	-0.316	0.085	4.211	2252	WS00918_N21
11	46.026	217	1	0.242	0.037	4.522	2252	WS00918_N21
1	22.419	4	3	0.315	0.090	4.380	2253	WS00918_O19
1	62.128	11	2	-0.282	0.085	4.982	2253	WS00918_O19
4	43.146	74	3	0.171	0.038	4.188	2253	WS00918_O19
9	49.013	173	2	0.216	0.045	5.036	2253	WS00918_O19
10	78.479	193	3	0.356	0.074	8.101	2253	WS00918_O19
12	14.406	230	3	-0.315	0.062	6.862	2253	WS00918_O19
13	3.386	245	1	0.223	0.055	3.990	2253	WS00918_O19
2	126.181	39	4	-0.449	0.122	4.800	2254	WS00918_P16

4	142.917	85	1	0.280	0.047	4.077	2254	WS00918_P16
9	0.000	163	2	-0.526	0.097	5.585	2254	WS00918_P16
11	27.405	204	1	0.542	0.161	14.435	2254	WS00918_P16
11	29.069	205	1	0.339	0.091	7.934	2254	WS00918_P16
1	2.788	2	3	0.239	0.059	4.839	2255	WS01010_B20
1	107.333	19	3	-0.278	0.080	6.463	2255	WS01010_B20
1	127.805	21	3	-0.180	0.058	4.634	2255	WS01010_B20
2	48.839	29	1	-0.247	0.040	4.151	2255	WS01010_B20
2	120.617	38	3	-0.344	0.091	7.364	2255	WS01010_B20
2	120.617	38	4	0.194	0.041	4.433	2255	WS01010_B20
2	127.147	40	3	0.169	0.051	4.160	2255	WS01010_B20
3	9.953	42	3	0.292	0.127	10.267	2255	WS01010_B20
3	47.928	49	1	-0.156	0.040	4.093	2255	WS01010_B20
5	0.000	89	1	-0.363	0.079	8.401	2255	WS01010_B20
5	52.820	99	3	-0.168	0.048	3.939	2255	WS01010_B20
5	54.072	100	4	-0.410	0.142	15.163	2255	WS01010_B20
5	57.017	102	4	0.471	0.115	12.012	2255	WS01010_B20
5	86.669	106	4	-0.388	0.206	9.860	2255	WS01010_B20
6	81.996	123	4	0.271	0.118	5.676	2255	WS01010_B20
7	73.169	142	1	-0.533	0.095	4.566	2255	WS01010_B20
8	11.974	150	1	0.159	0.038	3.854	2255	WS01010_B20
9	0.000	163	1	-0.416	0.070	3.847	2255	WS01010_B20
9	17.951	169	1	0.481	0.101	10.466	2255	WS01010_B20
9	61.113	178	1	-0.484	0.117	5.762	2255	WS01010_B20
11	46.026	217	1	0.298	0.056	5.877	2255	WS01010_B20
12	14.406	230	3	-0.235	0.050	4.039	2255	WS01010_B20
13	1.815	244	4	0.240	0.063	4.938	2255	WS01010_B20
14	43.392	250	1	0.266	0.074	4.078	2255	WS01010_B20
14	44.898	251	1	-0.325	0.102	10.257	2255	WS01010_B20
14	46.637	252	4	0.264	0.063	5.989	2255	WS01010_B20
1	2.788	2	4	-0.231	0.054	4.272	2256	WS01010_C22
4	82.462	78	1	-0.215	0.041	3.880	2256	WS01010_C22
6	26.350	115	2	-0.202	0.052	4.949	2256	WS01010_C22
10	35.204	189	2	-0.180	0.043	4.169	2256	WS01010_C22
2	126.181	39	4	0.357	0.068	4.386	2257	WS02612_H08
3	15.147	44	2	0.252	0.108	6.646	2257	WS02612_H08
6	87.891	124	2	-0.224	0.047	4.801	2257	WS02612_H08
6	89.401	125	2	-0.246	0.040	4.232	2257	WS02612_H08
6	95.249	126	1	0.354	0.101	4.716	2257	WS02612_H08
8	50.001	154	2	0.207	0.045	3.841	2257	WS02612_H08
9	2.011	167	4	-0.244	0.065	6.268	2257	WS02612_H08
10	104.214	198	4	-0.323	0.048	5.817	2257	WS02612_H08
11	17.088	203	1	-0.247	0.069	5.891	2257	WS02612_H08
11	27.405	204	1	0.394	0.083	7.214	2257	WS02612_H08
11	29.069	205	1	0.362	0.103	9.060	2257	WS02612_H08
11	45.989	216	1	-0.309	0.095	8.103	2257	WS02612_H08
5	16.219	93	2	-0.260	0.035	4.097	2258	WS02613_H18
6	56.188	120	2	0.218	0.040	4.573	2258	WS02613_H18
9	28.608	170	2	-0.240	0.034	3.980	2258	WS02613_H18
9	49.013	173	2	0.177	0.039	4.456	2258	WS02613_H18
9	59.963	177	4	-0.280	0.055	5.151	2258	WS02613_H18
10	70.246	190	2	0.279	0.047	5.454	2258	WS02613_H18
7	74.560	143	1	-0.238	0.049	5.012	2259	WS0091_C06
9	49.013	173	1	-0.226	0.050	5.189	2259	WS0091_C06

9	59.963	177	1	-0.216	0.059	5.842	2259	WS0091_C06
10	9.580	187	3	-0.453	0.134	8.644	2259	WS0091_C06
1	15.589	3	2	0.217	0.049	4.545	2260	WS00928_B23
3	47.928	49	1	0.295	0.070	6.695	2260	WS00928_B23
4	6.032	72	4	-0.203	0.039	3.942	2260	WS00928_B23
7	74.560	143	1	-0.173	0.044	4.176	2260	WS00928_B23
8	62.580	155	1	-0.307	0.069	3.937	2260	WS00928_B23
8	87.463	158	2	0.233	0.069	6.479	2260	WS00928_B23
9	28.608	170	2	-0.206	0.043	4.111	2260	WS00928_B23
9	28.608	170	3	0.271	0.049	4.328	2260	WS00928_B23
9	81.073	183	1	-0.281	0.048	4.586	2260	WS00928_B23
3	144.593	66	4	-0.217	0.060	5.290	2261	WS0091_H11
8	50.001	154	1	0.254	0.058	5.822	2261	WS0091_H11
9	0.000	163	2	-0.494	0.100	4.046	2261	WS0091_H11
9	59.963	177	4	-0.194	0.046	4.494	2261	WS0091_H11
10	70.246	190	2	0.285	0.068	6.940	2261	WS0091_H11
10	78.479	193	3	-0.268	0.050	4.920	2261	WS0091_H11
11	64.014	222	3	-0.205	0.048	4.677	2261	WS0091_H11
4	124.132	83	1	0.619	0.170	8.189	2262	WS00928_F18
4	142.917	85	1	-0.190	0.045	4.113	2262	WS00928_F18
5	54.072	100	4	-0.159	0.048	4.501	2262	WS00928_F18
9	3.127	168	1	-0.518	0.143	7.499	2262	WS00928_F18
12	22.441	227	1	-0.214	0.061	5.654	2262	WS00928_F18
1	95.572	17	2	-0.339	0.075	8.638	2263	WS0101_P04
3	15.147	44	2	-0.319	0.134	9.873	2263	WS0101_P04
4	103.719	81	4	-0.310	0.058	5.797	2263	WS0101_P04
6	45.897	117	1	0.308	0.066	5.363	2263	WS0101_P04
6	56.188	120	2	0.294	0.036	4.463	2263	WS0101_P04
6	87.891	124	2	0.200	0.036	3.962	2263	WS0101_P04
7	15.555	130	2	0.351	0.070	7.742	2263	WS0101_P04
7	15.555	130	3	-0.258	0.060	4.978	2263	WS0101_P04
7	73.169	142	1	-0.536	0.114	4.603	2263	WS0101_P04
10	35.204	189	2	-0.156	0.039	4.728	2263	WS0101_P04
10	70.246	190	2	0.194	0.050	5.454	2263	WS0101_P04
10	104.214	198	4	0.202	0.023	5.107	2263	WS0101_P04
11	0.000	199	2	-0.347	0.058	6.497	2263	WS0101_P04
12	14.406	230	4	-0.418	0.080	9.138	2263	WS0101_P04
14	22.402	248	4	-0.293	0.034	4.032	2263	WS0101_P04
3	14.500	43	2	0.227	0.031	4.352	2264	WS0101_P18
3	104.681	60	2	-0.217	0.031	4.132	2264	WS0101_P18
3	112.868	61	2	0.266	0.043	5.776	2264	WS0101_P18
9	49.013	173	2	0.183	0.034	4.506	2264	WS0101_P18
9	59.963	177	4	-0.299	0.052	5.734	2264	WS0101_P18
11	17.088	203	2	-0.218	0.046	4.889	2264	WS0101_P18
11	27.405	204	1	0.358	0.104	5.788	2264	WS0101_P18
11	35.604	207	3	-0.154	0.052	4.036	2264	WS0101_P18
11	64.014	222	3	-0.198	0.053	4.135	2264	WS0101_P18
2	126.181	39	2	0.335	0.073	7.216	2265	WS0102_B13
3	154.505	69	4	-0.214	0.041	4.025	2265	WS0102_B13
4	116.928	82	2	0.247	0.043	4.144	2265	WS0102_B13
6	49.100	118	2	0.237	0.090	4.058	2265	WS0102_B13
7	59.789	137	3	-0.220	0.050	4.450	2265	WS0102_B13
7	70.278	141	3	0.244	0.064	5.729	2265	WS0102_B13
7	91.487	146	3	0.185	0.057	5.098	2265	WS0102_B13

9	17.951	169	2	0.334	0.092	9.626	2265	WS0102_B13
1	82.301	16	3	-0.221	0.039	4.204	2266	WS0102_C06
3	47.928	49	1	0.220	0.043	4.186	2266	WS0102_C06
6	95.249	126	1	0.283	0.064	4.027	2266	WS0102_C06
8	11.974	150	1	-0.313	0.074	7.305	2266	WS0102_C06
9	59.963	177	4	-0.261	0.054	4.326	2266	WS0102_C06
11	64.014	222	3	-0.271	0.060	6.511	2266	WS0102_C06
8	11.974	150	1	-0.220	0.051	4.704	2267	WS0102_E07
9	0.000	166	1	0.206	0.056	5.115	2267	WS0102_E07
1	82.301	16	3	-0.245	0.057	5.895	2268	WS0102_E21
3	47.928	49	1	0.266	0.059	5.577	2268	WS0102_E21
4	124.132	83	1	0.568	0.149	6.447	2268	WS0102_E21
4	142.917	85	1	-0.307	0.063	5.988	2268	WS0102_E21
6	89.401	125	1	-0.290	0.045	4.334	2268	WS0102_E21
6	95.249	126	1	0.353	0.095	4.929	2268	WS0102_E21
8	39.090	152	4	0.173	0.065	5.469	2268	WS0102_E21
9	0.000	166	2	0.266	0.055	5.253	2268	WS0102_E21
12	14.406	230	4	-0.247	0.052	4.578	2268	WS0102_E21
1	2.788	2	4	-0.295	0.099	8.725	2269	WS0102_G21
1	40.464	7	4	0.282	0.045	4.121	2269	WS0102_G21
11	27.405	204	1	0.335	0.076	6.139	2269	WS0102_G21
11	29.069	205	1	0.304	0.086	6.886	2269	WS0102_G21
3	95.157	59	2	0.186	0.050	5.174	2270	WS0102_H07
4	0.000	71	4	-0.242	0.061	5.809	2270	WS0102_H07
7	75.883	144	2	-0.392	0.066	4.165	2270	WS0102_H07
11	45.989	216	2	-0.231	0.045	4.932	2270	WS0102_H07
2	95.880	34	1	0.265	0.040	4.369	2271	WS0092_G13
3	47.928	49	1	0.300	0.077	8.374	2271	WS0092_G13
4	55.925	76	1	-0.269	0.053	5.786	2271	WS0092_G13
8	0.000	148	4	-0.224	0.041	3.993	2271	WS0092_G13
11	27.405	204	1	0.267	0.048	5.065	2271	WS0092_G13
11	27.405	204	4	-0.196	0.041	4.041	2271	WS0092_G13
11	64.014	222	3	-0.339	0.089	8.144	2271	WS0092_G13
12	0.000	233	1	0.211	0.043	4.678	2271	WS0092_G13
1	15.589	3	2	0.226	0.061	7.182	2272	WS0092_L07
1	29.800	5	2	-0.244	0.038	4.029	2272	WS0092_L07
1	38.326	6	2	0.252	0.045	5.429	2272	WS0092_L07
1	43.578	8	2	-0.258	0.033	3.855	2272	WS0092_L07
1	82.301	16	4	0.273	0.059	6.249	2272	WS0092_L07
3	47.928	49	1	0.200	0.058	4.086	2272	WS0092_L07
1	45.794	9	1	0.355	0.092	8.466	2273	WS01013_F11
1	123.316	20	4	-0.345	0.078	4.971	2273	WS01013_F11
3	9.953	42	3	-0.220	0.052	4.294	2273	WS01013_F11
3	14.500	43	2	-0.290	0.067	5.852	2273	WS01013_F11
3	20.512	46	2	0.173	0.053	5.063	2273	WS01013_F11
3	137.891	64	1	-0.329	0.079	7.463	2273	WS01013_F11
3	144.647	67	1	-0.363	0.098	4.212	2273	WS01013_F11
7	74.560	143	1	-0.322	0.062	5.753	2273	WS01013_F11
9	28.608	170	2	0.258	0.056	5.661	2273	WS01013_F11
9	28.608	170	3	-0.293	0.065	5.331	2273	WS01013_F11
11	17.088	203	1	0.233	0.048	4.375	2273	WS01013_F11
11	27.405	204	1	-0.251	0.043	4.070	2273	WS01013_F11
1	22.419	4	3	0.415	0.091	4.441	2274	WS0269_B20
3	15.147	44	2	-0.284	0.084	3.923	2274	WS0269_B20

4	116.928	82	2	-0.250	0.062	6.090	2274	WS0269_B20
4	124.132	83	1	0.781	0.245	12.411	2274	WS0269_B20
4	124.132	83	2	-0.296	0.121	5.006	2274	WS0269_B20
4	142.917	85	1	-0.304	0.105	9.689	2274	WS0269_B20
4	173.929	88	2	0.191	0.041	3.929	2274	WS0269_B20
10	35.204	189	1	-0.457	0.087	8.261	2274	WS0269_B20
10	80.088	194	1	-0.165	0.054	4.954	2274	WS0269_B20
10	93.347	196	1	0.473	0.130	5.367	2274	WS0269_B20
12	22.441	227	3	-0.198	0.041	4.113	2274	WS0269_B20
1	22.419	4	3	0.371	0.094	5.579	2275	WS01013_K23
1	95.572	17	2	0.355	0.040	4.506	2275	WS01013_K23
1	95.572	17	3	-0.248	0.041	5.740	2275	WS01013_K23
3	9.953	42	3	0.246	0.039	5.204	2275	WS01013_K23
3	141.443	65	3	0.483	0.179	10.457	2275	WS01013_K23
4	116.928	82	4	-0.215	0.069	4.150	2275	WS01013_K23
7	15.555	130	3	0.230	0.035	3.924	2275	WS01013_K23
9	28.608	170	3	-0.160	0.024	4.184	2275	WS01013_K23
11	64.014	222	3	-0.189	0.040	5.243	2275	WS01013_K23
1	15.589	3	2	0.193	0.044	5.026	2276	WS0269_O03
1	40.464	7	3	0.274	0.065	5.959	2276	WS0269_O03
1	43.578	8	3	0.288	0.078	7.210	2276	WS0269_O03
1	45.794	9	3	-0.261	0.077	6.931	2276	WS0269_O03
1	95.572	17	3	0.270	0.064	6.093	2276	WS0269_O03
3	137.891	64	1	0.282	0.059	4.364	2276	WS0269_O03
4	55.925	76	1	0.240	0.060	4.476	2276	WS0269_O03
4	103.719	81	4	-0.232	0.044	4.240	2276	WS0269_O03
6	49.100	118	2	0.272	0.109	5.211	2276	WS0269_O03
6	69.073	121	3	-0.216	0.054	5.006	2276	WS0269_O03
9	0.000	163	2	0.464	0.091	4.270	2276	WS0269_O03
6	95.249	126	1	0.411	0.091	5.000	2277	WS00712_E04
9	69.046	181	1	-0.385	0.080	4.392	2277	WS00712_E04
11	0.000	199	3	-0.428	0.081	6.571	2277	WS00712_E04
11	27.405	204	1	0.361	0.054	5.127	2277	WS00712_E04
11	45.989	216	1	-0.169	0.053	4.781	2277	WS00712_E04
12	14.406	230	3	-0.371	0.057	6.391	2277	WS00712_E04
3	9.953	42	3	0.192	0.057	5.199	2278	WS00712_K08
3	137.891	64	1	0.214	0.048	4.104	2278	WS00712_K08
4	75.958	77	3	-0.310	0.145	7.057	2278	WS00712_K08
5	3.484	90	2	-0.379	0.089	4.918	2278	WS00712_K08
5	16.219	93	2	0.281	0.036	3.947	2278	WS00712_K08
5	57.017	102	2	0.166	0.037	3.909	2278	WS00712_K08
9	69.046	181	2	0.357	0.109	4.054	2278	WS00712_K08
11	35.604	207	3	0.189	0.058	5.262	2278	WS00712_K08
11	37.143	209	3	-0.157	0.043	3.844	2278	WS00712_K08
11	42.600	213	3	0.181	0.051	4.567	2278	WS00712_K08
12	6.242	234	2	0.295	0.077	9.381	2278	WS00712_K08
1	38.326	6	2	0.268	0.035	4.158	2279	WS0094_N23
3	47.928	49	1	0.277	0.069	4.913	2279	WS0094_N23
5	0.000	89	3	0.342	0.051	4.871	2279	WS0094_N23
6	106.702	128	2	0.587	0.109	9.160	2279	WS0094_N23
7	17.610	131	3	0.272	0.077	6.267	2279	WS0094_N23
7	62.787	139	2	0.267	0.036	3.871	2279	WS0094_N23
11	42.600	213	3	0.241	0.050	6.025	2279	WS0094_N23
12	22.441	227	1	0.303	0.082	6.013	2279	WS0094_N23

12	22.441	227	3	-0.279	0.043	5.236	2279	WS0094_N23
2	95.880	34	3	0.320	0.062	7.024	2280	WS0057_F16
3	95.157	59	2	-0.230	0.046	5.033	2280	WS0057_F16
3	104.681	60	2	0.264	0.089	9.023	2280	WS0057_F16
3	112.868	61	2	-0.285	0.091	9.187	2280	WS0057_F16
3	114.747	62	2	-0.286	0.083	8.377	2280	WS0057_F16
4	43.146	74	3	-0.218	0.032	3.939	2280	WS0057_F16
4	173.929	88	2	0.261	0.063	6.414	2280	WS0057_F16
5	42.979	97	2	-0.230	0.052	4.492	2280	WS0057_F16
7	59.789	137	3	0.359	0.103	12.629	2280	WS0057_F16
7	70.278	141	3	-0.266	0.061	7.432	2280	WS0057_F16
11	56.921	219	2	0.305	0.103	4.938	2280	WS0057_F16
12	22.441	227	1	0.251	0.043	3.868	2280	WS0057_F16
12	6.242	234	2	0.329	0.078	7.683	2280	WS0057_F16
12	6.242	234	4	-0.344	0.088	6.985	2280	WS0057_F16
4	124.132	83	1	0.521	0.110	5.424	2281	WS01017_C01
4	142.917	85	1	-0.315	0.077	7.596	2281	WS01017_C01
6	106.702	128	2	0.394	0.072	4.029	2281	WS01017_C01
7	75.883	144	2	0.463	0.147	5.549	2281	WS01017_C01
7	75.883	144	4	-0.292	0.046	3.875	2281	WS01017_C01
9	55.358	175	1	0.336	0.048	4.965	2281	WS01017_C01
9	59.963	177	4	-0.262	0.065	5.216	2281	WS01017_C01
10	95.795	197	4	0.169	0.056	4.592	2281	WS01017_C01
11	64.014	222	4	0.185	0.044	3.963	2281	WS01017_C01
12	14.406	230	4	-0.216	0.049	4.160	2281	WS01017_C01
14	0.000	246	1	-0.278	0.062	6.196	2281	WS01017_C01
3	17.158	45	1	0.198	0.042	4.118	2282	WS0058_A06
9	17.951	169	1	0.253	0.049	4.835	2282	WS0058_A06
12	38.543	232	4	0.285	0.073	4.718	2282	WS0058_A06
1	2.788	2	4	-0.294	0.085	6.133	2283	WS01017_F19
2	48.839	29	4	0.228	0.053	4.297	2283	WS01017_F19
2	63.261	30	3	0.188	0.037	3.991	2283	WS01017_F19
2	93.504	32	4	-0.259	0.055	4.060	2283	WS01017_F19
3	69.071	52	1	-0.331	0.082	5.288	2283	WS01017_F19
3	95.157	59	2	-0.195	0.059	5.617	2283	WS01017_F19
3	112.868	61	2	-0.226	0.059	5.054	2283	WS01017_F19
3	114.747	62	2	-0.220	0.064	5.442	2283	WS01017_F19
4	55.925	76	1	-0.251	0.059	5.902	2283	WS01017_F19
6	106.702	128	2	0.457	0.112	5.127	2283	WS01017_F19
7	74.560	143	1	0.358	0.102	10.401	2283	WS01017_F19
9	0.000	163	1	0.435	0.068	4.350	2283	WS01017_F19
11	0.000	199	3	-0.277	0.058	6.101	2283	WS01017_F19
11	27.405	204	1	0.324	0.085	8.793	2283	WS01017_F19
11	29.069	205	1	0.182	0.039	3.978	2283	WS01017_F19
12	0.000	226	3	0.228	0.043	4.663	2283	WS01017_F19
13	0.000	243	4	0.320	0.102	3.897	2283	WS01017_F19
13	3.386	245	1	-0.376	0.106	10.926	2283	WS01017_F19
14	0.000	246	1	0.230	0.050	5.088	2283	WS01017_F19
1	40.464	7	3	0.201	0.047	4.881	2284	WS01017_G06
1	43.578	8	3	0.185	0.040	4.201	2284	WS01017_G06
2	48.839	29	4	0.469	0.121	6.569	2284	WS01017_G06
2	95.880	34	1	0.477	0.060	6.106	2284	WS01017_G06
2	102.814	36	1	0.320	0.116	10.894	2284	WS01017_G06
4	124.132	83	1	-0.401	0.088	4.026	2284	WS01017_G06

6	89.401	125	2	-0.250	0.041	4.730	2284	WS01017_G06
11	0.000	199	2	0.274	0.045	4.669	2284	WS01017_G06
11	43.491	214	3	0.242	0.051	5.320	2284	WS01017_G06
11	44.884	215	3	-0.154	0.039	4.263	2284	WS01017_G06
12	22.441	227	1	0.205	0.084	7.921	2284	WS01017_G06
1	107.333	19	3	-0.236	0.047	5.647	2285	WS01017_H13
2	75.550	31	2	-0.260	0.028	3.910	2285	WS01017_H13
3	9.953	42	3	0.198	0.054	6.392	2285	WS01017_H13
3	9.953	42	4	-0.164	0.059	3.973	2285	WS01017_H13
6	106.702	128	2	0.416	0.046	4.624	2285	WS01017_H13
7	15.555	130	2	0.290	0.043	6.309	2285	WS01017_H13
7	17.610	131	3	0.272	0.071	6.957	2285	WS01017_H13
9	69.046	181	2	-0.291	0.078	7.888	2285	WS01017_H13
2	48.839	29	4	0.216	0.050	4.199	2286	WS01017_I01
4	92.039	79	2	-0.169	0.036	4.403	2286	WS01017_I01
8	0.000	148	4	-0.285	0.056	4.564	2286	WS01017_I01
9	17.951	169	1	0.370	0.057	4.048	2286	WS01017_I01
9	69.046	181	2	-0.266	0.092	6.859	2286	WS01017_I01
11	43.491	214	3	0.226	0.038	4.258	2286	WS01017_I01
11	64.014	222	3	-0.271	0.045	4.933	2286	WS01017_I01
12	0.000	226	3	0.225	0.057	6.202	2286	WS01017_I01
7	105.477	147	2	-0.240	0.091	4.475	2287	WS01017_J08
9	0.000	166	2	0.243	0.040	4.220	2287	WS01017_J08
9	69.046	181	1	-0.266	0.088	4.369	2287	WS01017_J08
10	78.479	193	3	-0.330	0.041	4.833	2287	WS01017_J08
10	86.151	195	3	0.226	0.050	5.810	2287	WS01017_J08
11	0.000	199	3	-0.454	0.137	15.663	2287	WS01017_J08
11	64.014	222	3	-0.254	0.042	4.951	2287	WS01017_J08
14	22.402	248	3	0.377	0.082	9.635	2287	WS01017_J08
14	44.898	251	1	-0.263	0.081	6.146	2287	WS01017_J08
1	82.301	16	3	-0.239	0.052	4.266	2288	WS01017_K02
1	95.572	17	3	0.335	0.093	5.190	2288	WS01017_K02
3	14.500	43	2	0.329	0.104	7.917	2288	WS01017_K02
3	69.071	52	1	0.262	0.115	4.843	2288	WS01017_K02
4	116.928	82	2	0.283	0.067	6.440	2288	WS01017_K02
6	81.996	123	4	-0.350	0.104	5.329	2288	WS01017_K02
12	28.493	228	2	0.309	0.129	7.374	2288	WS01017_K02
12	38.543	232	4	-0.302	0.081	4.052	2288	WS01017_K02
12	22.629	237	1	0.295	0.069	6.371	2288	WS01017_K02
12	32.473	238	1	0.313	0.078	7.220	2288	WS01017_K02
12	51.382	242	1	-0.292	0.081	4.057	2288	WS01017_K02
1	123.316	20	4	-0.240	0.054	4.337	2289	WS01017_L18
1	131.686	23	2	0.411	0.051	3.934	2289	WS01017_L18
3	9.953	42	3	0.328	0.101	8.173	2289	WS01017_L18
3	95.157	59	2	-0.186	0.036	4.718	2289	WS01017_L18
3	114.747	62	2	-0.223	0.040	4.535	2289	WS01017_L18
4	173.929	88	2	-0.186	0.037	4.161	2289	WS01017_L18
5	3.484	90	2	-0.226	0.051	4.099	2289	WS01017_L18
7	105.477	147	2	-0.400	0.129	10.374	2289	WS01017_L18
9	59.963	177	4	0.317	0.078	8.761	2289	WS01017_L18
9	81.073	183	1	0.244	0.076	5.735	2289	WS01017_L18
11	0.000	199	2	0.282	0.055	6.164	2289	WS01017_L18
11	64.014	222	3	-0.320	0.080	6.447	2289	WS01017_L18
1	2.788	2	3	-0.193	0.037	3.967	2290	WS01017_M11

3	9.953	42	3	0.260	0.057	6.104	2290	WS01017_M11
3	137.891	64	1	0.222	0.044	3.988	2290	WS01017_M11
3	137.891	64	3	0.234	0.052	5.400	2290	WS01017_M11
3	141.443	65	3	0.319	0.083	4.411	2290	WS01017_M11
6	89.401	125	2	-0.289	0.059	5.617	2290	WS01017_M11
11	45.989	216	1	-0.270	0.072	6.721	2290	WS01017_M11
1	29.800	5	1	-0.282	0.051	5.346	2291	WS00946_E12
4	82.462	78	1	-0.229	0.060	6.187	2291	WS00946_E12
4	142.917	85	1	0.317	0.050	5.338	2291	WS00946_E12
5	86.669	106	4	0.385	0.111	6.747	2291	WS00946_E12
6	45.897	117	4	-0.268	0.050	5.271	2291	WS00946_E12
6	54.066	119	4	-0.224	0.055	5.403	2291	WS00946_E12
9	49.013	173	1	-0.248	0.036	4.907	2291	WS00946_E12
11	0.000	199	2	-0.277	0.067	5.816	2291	WS00946_E12
13	3.386	245	1	0.179	0.041	4.242	2291	WS00946_E12
14	0.000	246	1	-0.274	0.045	4.759	2291	WS00946_E12
1	77.177	15	2	0.205	0.045	4.749	2292	WS00817_M14
2	120.617	38	3	-0.194	0.044	4.771	2292	WS00817_M14
3	80.211	55	4	0.249	0.049	3.901	2292	WS00817_M14
4	116.928	82	2	-0.296	0.070	7.381	2292	WS00817_M14
4	124.132	83	2	-0.320	0.120	5.227	2292	WS00817_M14
7	62.787	139	4	0.195	0.062	4.205	2292	WS00817_M14
7	75.883	144	4	0.380	0.071	5.355	2292	WS00817_M14
9	36.227	165	3	0.287	0.053	5.797	2292	WS00817_M14
12	51.382	242	1	0.402	0.145	3.910	2292	WS00817_M14
3	14.500	43	1	-0.203	0.037	3.895	2293	WS00946_N14
3	80.211	55	4	-0.262	0.074	5.474	2293	WS00946_N14
3	137.891	64	1	0.266	0.059	6.049	2293	WS00946_N14
3	141.443	65	3	0.290	0.059	4.271	2293	WS00946_N14
7	15.555	130	2	0.247	0.063	5.377	2293	WS00946_N14
7	17.610	131	3	0.344	0.144	5.849	2293	WS00946_N14
7	91.487	146	3	0.234	0.061	6.941	2293	WS00946_N14
10	104.214	198	2	0.191	0.040	4.031	2293	WS00946_N14
11	45.989	216	1	-0.257	0.053	5.609	2293	WS00946_N14
1	29.800	5	1	-0.285	0.075	7.544	2294	WS00818_D01
4	82.462	78	1	-0.228	0.051	5.206	2294	WS00818_D01
5	16.219	93	2	0.351	0.051	4.936	2294	WS00818_D01
9	28.608	170	2	0.344	0.065	6.157	2294	WS00818_D01
9	32.356	171	2	0.318	0.059	5.471	2294	WS00818_D01
11	9.237	202	4	0.287	0.054	5.321	2294	WS00818_D01
14	22.402	248	4	0.398	0.045	4.703	2294	WS00818_D01
14	46.637	252	4	0.392	0.054	5.271	2294	WS00818_D01
3	9.953	42	3	0.309	0.051	6.134	2295	WS00922_J07
4	55.925	76	1	-0.197	0.041	3.962	2295	WS00922_J07
4	82.462	78	1	-0.219	0.055	5.678	2295	WS00922_J07
9	49.013	173	1	0.168	0.037	4.102	2295	WS00922_J07
1	22.419	4	3	0.253	0.085	5.319	2296	WS00922_M19
1	133.992	24	2	0.189	0.045	4.516	2296	WS00922_M19
4	55.925	76	1	-0.297	0.101	8.537	2296	WS00922_M19
4	82.462	78	1	-0.161	0.045	3.900	2296	WS00922_M19
5	86.669	106	4	0.315	0.139	3.948	2296	WS00922_M19
10	78.479	193	4	0.224	0.044	4.516	2296	WS00922_M19
11	27.405	204	1	0.231	0.074	6.645	2296	WS00922_M19
11	27.405	204	4	-0.380	0.087	7.399	2296	WS00922_M19

11	37.143	209	3	0.204	0.055	5.627	2296	WS00922_M19
11	42.600	213	3	-0.219	0.059	5.976	2296	WS00922_M19
1	40.464	7	3	0.153	0.032	4.085	2297	WS0087_H06
1	45.794	9	3	-0.226	0.046	5.576	2297	WS0087_H06
1	69.854	13	2	-0.128	0.032	4.096	2297	WS0087_H06
1	71.592	14	2	-0.128	0.032	4.105	2297	WS0087_H06
1	95.572	17	2	-0.263	0.062	8.015	2297	WS0087_H06
3	83.563	56	3	-0.268	0.065	8.235	2297	WS0087_H06
4	116.928	82	2	-0.232	0.050	6.422	2297	WS0087_H06
4	124.132	83	2	-0.226	0.063	4.096	2297	WS0087_H06
9	28.608	170	2	-0.218	0.029	3.926	2297	WS0087_H06
9	28.608	170	3	0.310	0.043	5.575	2297	WS0087_H06
9	32.356	171	2	-0.226	0.032	4.135	2297	WS0087_H06
1	40.464	7	3	0.333	0.081	7.538	2298	WS0087_I02
1	43.578	8	3	0.326	0.079	6.916	2298	WS0087_I02
1	45.794	9	1	-0.252	0.050	4.724	2298	WS0087_I02
4	124.132	83	1	0.512	0.119	5.268	2298	WS0087_I02
6	54.066	119	4	0.246	0.050	4.787	2298	WS0087_I02
9	81.073	183	1	-0.263	0.058	5.717	2298	WS0087_I02
10	93.347	196	1	0.459	0.097	5.072	2298	WS0087_I02
11	9.237	202	4	0.275	0.062	5.906	2298	WS0087_I02
13	0.000	243	4	0.417	0.127	5.609	2298	WS0087_I02
13	3.386	245	1	-0.261	0.059	5.724	2298	WS0087_I02
3	144.593	66	4	0.224	0.061	5.337	2299	WS0087_K24
3	154.505	69	4	0.208	0.046	4.077	2299	WS0087_K24
4	0.000	71	4	0.249	0.066	5.617	2299	WS0087_K24
5	31.191	95	1	-0.279	0.078	8.895	2299	WS0087_K24
5	52.820	99	1	0.250	0.038	4.483	2299	WS0087_K24
9	36.227	165	3	-0.211	0.041	4.270	2299	WS0087_K24
12	38.543	232	4	-0.314	0.104	4.605	2299	WS0087_K24
1	43.578	8	2	0.259	0.039	3.870	2300	WS0087_L13
2	75.550	31	2	-0.317	0.076	7.206	2300	WS0087_L13
3	104.681	60	2	-0.221	0.056	5.408	2300	WS0087_L13
3	112.868	61	2	0.226	0.047	4.629	2300	WS0087_L13
3	114.747	62	2	0.176	0.044	4.328	2300	WS0087_L13
4	75.958	77	3	-0.314	0.082	4.427	2300	WS0087_L13
5	0.000	89	3	0.252	0.047	5.410	2300	WS0087_L13
7	75.883	144	2	0.536	0.148	7.135	2300	WS0087_L13
9	59.963	177	1	0.232	0.051	4.699	2300	WS0087_L13
10	80.088	194	2	-0.285	0.066	6.275	2300	WS0087_L13
10	86.151	195	3	0.201	0.046	5.211	2300	WS0087_L13
14	44.898	251	3	0.233	0.038	4.099	2300	WS0087_L13
14	46.637	252	3	0.300	0.063	7.177	2300	WS0087_L13
1	22.419	4	3	0.262	0.075	4.270	2301	WS0087_N14
9	0.000	163	1	0.556	0.120	6.813	2301	WS0087_N14
9	36.227	165	3	-0.251	0.060	6.496	2301	WS0087_N14
10	9.580	187	3	0.333	0.087	4.789	2301	WS0087_N14
11	40.960	210	2	0.384	0.062	4.076	2301	WS0087_N14
2	120.617	38	3	-0.320	0.059	6.408	2302	WS0087_O09
2	127.147	40	3	0.257	0.057	6.146	2302	WS0087_O09
3	47.928	49	1	0.268	0.050	5.288	2302	WS0087_O09
3	83.563	56	3	-0.202	0.043	4.606	2302	WS0087_O09
3	95.157	59	2	-0.288	0.090	8.097	2302	WS0087_O09
3	104.681	60	2	0.175	0.054	4.445	2302	WS0087_O09

3	112.868	61	2	-0.211	0.065	5.338	2302	WS0087_O09
3	114.747	62	2	-0.216	0.053	4.369	2302	WS0087_O09
12	6.242	234	2	-0.252	0.047	4.079	2302	WS0087_O09
12	22.629	237	1	0.176	0.039	4.150	2302	WS0087_O09
12	32.473	238	1	0.170	0.037	3.864	2302	WS0087_O09
1	43.578	8	2	0.340	0.082	8.961	2303	WS00715_A24
1	71.592	14	2	-0.178	0.037	3.928	2303	WS00715_A24
3	9.953	42	3	-0.212	0.046	4.722	2303	WS00715_A24
3	14.500	43	1	0.284	0.065	5.278	2303	WS00715_A24
3	17.158	45	1	-0.250	0.063	5.143	2303	WS00715_A24
8	0.000	148	4	-0.222	0.059	5.043	2303	WS00715_A24
9	17.951	169	1	-0.370	0.095	7.743	2303	WS00715_A24
9	17.951	169	2	0.250	0.045	5.377	2303	WS00715_A24
9	81.073	183	1	-0.339	0.078	6.490	2303	WS00715_A24
10	9.580	187	2	0.295	0.075	4.156	2303	WS00715_A24
10	35.204	189	2	-0.260	0.041	4.468	2303	WS00715_A24
11	17.088	203	1	-0.226	0.054	4.380	2303	WS00715_A24
11	40.960	210	2	0.409	0.082	4.533	2303	WS00715_A24
1	107.333	19	4	-0.162	0.035	4.525	2304	WS00715_B19
1	133.992	24	2	0.227	0.044	4.877	2304	WS00715_B19
4	173.929	88	2	0.218	0.045	4.990	2304	WS00715_B19
5	3.484	90	2	-0.287	0.050	4.012	2304	WS00715_B19
6	25.914	114	1	-0.228	0.054	4.127	2304	WS00715_B19
6	106.702	128	2	0.410	0.120	4.103	2304	WS00715_B19
9	55.358	175	1	-0.293	0.073	5.454	2304	WS00715_B19
9	55.358	175	3	0.664	0.264	10.923	2304	WS00715_B19
9	61.113	178	1	0.426	0.178	7.507	2304	WS00715_B19
11	43.491	214	4	0.170	0.037	4.824	2304	WS00715_B19
11	44.884	215	4	-0.224	0.035	4.300	2304	WS00715_B19
12	22.629	237	1	0.221	0.059	4.483	2304	WS00715_B19
12	32.473	238	1	0.226	0.060	4.540	2304	WS00715_B19
14	43.392	250	2	0.189	0.036	3.943	2304	WS00715_B19
14	44.898	251	1	0.324	0.077	6.127	2304	WS00715_B19
14	44.898	251	3	-0.311	0.078	5.440	2304	WS00715_B19
2	93.504	32	4	-0.267	0.089	5.549	2305	WS00815_G03
2	127.147	40	3	-0.192	0.048	5.980	2305	WS00815_G03
3	95.157	59	2	0.185	0.033	4.068	2305	WS00815_G03
3	104.681	60	2	-0.182	0.032	4.112	2305	WS00815_G03
3	112.868	61	2	0.200	0.030	3.881	2305	WS00815_G03
6	26.350	115	2	-0.190	0.030	3.885	2305	WS00815_G03
6	49.100	118	2	0.352	0.057	5.627	2305	WS00815_G03
11	56.921	219	2	-0.211	0.046	4.426	2305	WS00815_G03
13	1.815	244	2	-0.170	0.037	4.764	2305	WS00815_G03
14	0.000	246	1	0.239	0.071	4.219	2305	WS00815_G03
3	104.681	60	2	0.196	0.038	4.060	2306	WS00815_M12
4	116.928	82	2	-0.334	0.065	7.201	2306	WS00815_M12
5	16.219	93	1	-0.265	0.063	5.012	2306	WS00815_M12
9	49.013	173	2	0.262	0.061	6.554	2306	WS00815_M12
12	14.406	230	3	-0.227	0.053	5.450	2306	WS00815_M12
3	9.953	42	3	0.272	0.096	8.301	2307	WS00715_G04
4	142.917	85	1	-0.183	0.055	4.170	2307	WS00715_G04
5	52.820	99	3	-0.189	0.039	3.948	2307	WS00715_G04
7	17.610	131	3	0.400	0.125	7.572	2307	WS00715_G04
11	41.984	212	2	0.207	0.036	4.022	2307	WS00715_G04

11	60.464	221	2	0.244	0.040	4.907	2307	WS00715_G04
11	64.014	222	4	0.227	0.069	6.756	2307	WS00715_G04
12	14.406	230	4	-0.291	0.082	8.352	2307	WS00715_G04
1	62.128	11	1	-0.449	0.087	3.887	2308	WS00921_A18
2	93.504	32	4	0.237	0.041	4.230	2308	WS00921_A18
3	144.593	66	4	0.152	0.040	4.128	2308	WS00921_A18
3	148.745	68	4	-0.243	0.059	6.658	2308	WS00921_A18
4	6.032	72	4	0.290	0.102	10.574	2308	WS00921_A18
4	116.928	82	4	0.304	0.077	8.021	2308	WS00921_A18
4	142.917	85	1	0.316	0.090	8.790	2308	WS00921_A18
7	62.787	139	2	0.252	0.067	5.943	2308	WS00921_A18
9	49.013	173	1	-0.232	0.033	3.897	2308	WS00921_A18
12	14.406	230	4	0.312	0.071	6.983	2308	WS00921_A18
1	2.788	2	3	-0.264	0.055	6.713	2309	WS00715_O01
5	31.191	95	1	0.216	0.044	4.143	2309	WS00715_O01
7	62.787	139	2	0.168	0.043	4.031	2309	WS00715_O01
7	74.560	143	1	-0.263	0.052	4.876	2309	WS00715_O01
10	9.580	187	3	-0.387	0.126	7.865	2309	WS00715_O01
10	35.204	189	1	-0.248	0.056	5.236	2309	WS00715_O01
10	77.676	192	1	-0.256	0.053	4.955	2309	WS00715_O01
10	80.088	194	1	-0.277	0.045	4.265	2309	WS00715_O01
2	126.181	39	4	0.412	0.091	4.411	2310	WS00921_F05
3	116.958	63	4	-0.178	0.053	4.216	2310	WS00921_F05
3	137.891	64	3	0.309	0.100	10.757	2310	WS00921_F05
4	142.917	85	1	0.259	0.053	4.594	2310	WS00921_F05
4	142.917	85	3	-0.329	0.055	6.095	2310	WS00921_F05
6	87.891	124	2	-0.311	0.087	8.966	2310	WS00921_F05
6	89.401	125	2	-0.355	0.094	9.567	2310	WS00921_F05
9	0.000	166	2	0.262	0.050	4.813	2310	WS00921_F05
9	49.013	173	2	0.280	0.063	6.465	2310	WS00921_F05
9	59.963	177	4	0.199	0.052	4.390	2310	WS00921_F05
1	2.788	2	4	-0.212	0.045	5.124	2311	WS01013_N05
2	48.839	29	4	0.282	0.054	5.765	2311	WS01013_N05
3	20.512	46	2	0.228	0.033	3.957	2311	WS01013_N05
3	137.891	64	3	0.231	0.073	4.322	2311	WS01013_N05
5	54.072	100	4	0.208	0.038	3.943	2311	WS01013_N05
7	17.610	131	3	-0.273	0.147	6.608	2311	WS01013_N05
9	17.951	169	2	-0.299	0.065	7.248	2311	WS01013_N05
11	42.600	213	3	-0.186	0.047	4.029	2311	WS01013_N05
12	6.242	234	2	-0.187	0.040	4.488	2311	WS01013_N05
14	0.000	246	2	-0.182	0.050	5.767	2311	WS01013_N05
1	29.800	5	1	-0.284	0.096	7.962	2312	WS01013_O03
3	9.953	42	3	0.256	0.035	4.297	2312	WS01013_O03
5	3.484	90	2	-0.379	0.083	4.595	2312	WS01013_O03
5	16.219	93	2	0.351	0.064	5.116	2312	WS01013_O03
6	95.249	126	1	-0.352	0.064	3.940	2312	WS01013_O03
9	28.608	170	2	0.256	0.037	4.185	2312	WS01013_O03
13	0.000	243	4	0.422	0.225	5.139	2312	WS01013_O03
3	15.147	44	2	0.223	0.058	4.193	2313	WS00919_B09
4	82.462	78	1	-0.168	0.049	3.914	2313	WS00919_B09
4	173.929	88	2	-0.296	0.084	9.170	2313	WS00919_B09
6	81.996	123	3	0.321	0.079	4.401	2313	WS00919_B09
7	74.560	143	1	0.205	0.049	3.880	2313	WS00919_B09
11	60.464	221	2	0.206	0.037	4.104	2313	WS00919_B09

13	3.386	245	1	-0.260	0.049	3.927	2313	WS00919_B09
1	95.572	17	2	0.272	0.042	5.166	2314	WS00919_C08
2	48.839	29	4	0.300	0.046	3.918	2314	WS00919_C08
3	9.953	42	3	0.281	0.054	5.064	2314	WS00919_C08
3	116.958	63	4	-0.186	0.048	4.615	2314	WS00919_C08
4	173.929	88	2	-0.271	0.063	7.663	2314	WS00919_C08
11	27.405	204	4	-0.327	0.057	5.702	2314	WS00919_C08
14	0.000	246	1	0.206	0.061	4.071	2314	WS00919_C08
2	95.880	34	1	-0.417	0.105	10.751	2315	WS00919_E19
2	95.880	34	3	0.188	0.066	6.099	2315	WS00919_E19
2	102.814	36	1	-0.251	0.068	7.154	2315	WS00919_E19
4	116.928	82	2	-0.316	0.043	3.956	2315	WS00919_E19
7	75.883	144	2	0.504	0.133	6.654	2315	WS00919_E19
7	105.477	147	2	-0.334	0.136	6.205	2315	WS00919_E19
8	95.937	159	3	-0.207	0.062	5.780	2315	WS00919_E19
9	61.113	178	1	-0.267	0.080	3.940	2315	WS00919_E19
11	40.960	210	4	0.269	0.047	4.155	2315	WS00919_E19
11	45.989	216	1	-0.283	0.049	5.079	2315	WS00919_E19
2	48.839	29	4	0.252	0.056	5.396	2316	WS00919_F17
3	148.745	68	4	-0.206	0.037	4.403	2316	WS00919_F17
4	55.925	76	1	-0.210	0.056	4.157	2316	WS00919_F17
4	82.462	78	1	-0.281	0.128	9.771	2316	WS00919_F17
4	103.719	81	4	-0.241	0.036	4.298	2316	WS00919_F17
4	116.928	82	4	0.276	0.051	6.017	2316	WS00919_F17
7	75.883	144	2	-0.388	0.052	3.916	2316	WS00919_F17
10	104.214	198	2	0.193	0.024	4.098	2316	WS00919_F17
12	28.493	228	2	-0.334	0.067	5.001	2316	WS00919_F17
1	50.955	10	1	-0.232	0.057	5.016	2317	WS00919_H13
11	27.405	204	1	0.220	0.050	4.534	2317	WS00919_H13
12	6.242	234	2	-0.166	0.036	4.369	2317	WS00919_H13
1	45.794	9	3	-0.235	0.041	4.036	2318	WS00919_H23
2	48.839	29	4	0.213	0.050	3.921	2318	WS00919_H23
2	102.814	36	2	-0.242	0.037	4.914	2318	WS00919_H23
9	49.013	173	1	0.223	0.085	4.238	2318	WS00919_H23
11	27.405	204	1	0.264	0.079	4.449	2318	WS00919_H23
12	6.242	234	2	-0.239	0.029	4.067	2318	WS00919_H23
1	107.333	19	4	-0.277	0.104	9.481	2319	WS01020_O06
2	95.880	34	1	0.265	0.047	4.109	2319	WS01020_O06
2	102.814	36	1	0.310	0.056	5.327	2319	WS01020_O06
2	127.147	40	3	-0.245	0.051	4.856	2319	WS01020_O06
5	52.820	99	3	0.281	0.060	5.352	2319	WS01020_O06
6	56.188	120	2	0.286	0.049	4.142	2319	WS01020_O06
6	76.376	122	1	0.273	0.105	4.162	2319	WS01020_O06
10	86.151	195	3	0.240	0.041	3.910	2319	WS01020_O06
11	40.960	210	2	0.362	0.070	3.873	2319	WS01020_O06
1	127.805	21	3	-0.319	0.067	6.975	2320	WS01020_O23
2	93.504	32	4	0.284	0.051	4.045	2320	WS01020_O23
3	14.500	43	1	-0.291	0.052	5.229	2320	WS01020_O23
3	17.158	45	1	0.179	0.042	4.142	2320	WS01020_O23
3	144.593	66	4	0.251	0.050	4.570	2320	WS01020_O23
3	148.745	68	4	-0.288	0.056	4.748	2320	WS01020_O23
3	154.505	69	4	0.267	0.052	4.471	2320	WS01020_O23
4	75.958	77	3	-0.401	0.121	5.273	2320	WS01020_O23
5	31.191	95	1	-0.176	0.043	4.265	2320	WS01020_O23

6	106.702	128	2	-0.420	0.104	4.327	2320	WS01020_O23
9	36.227	165	3	-0.215	0.038	3.904	2320	WS01020_O23
11	0.000	199	3	-0.384	0.125	13.020	2320	WS01020_O23
11	60.464	221	1	0.276	0.053	5.283	2320	WS01020_O23
1	22.419	4	4	0.292	0.075	4.151	2321	WS01021_A21
1	127.805	21	3	-0.214	0.048	4.085	2321	WS01021_A21
2	48.839	29	1	0.296	0.059	6.767	2321	WS01021_A21
2	75.550	31	1	-0.329	0.062	7.137	2321	WS01021_A21
3	137.891	64	1	0.310	0.049	5.734	2321	WS01021_A21
3	141.443	65	3	0.281	0.098	5.631	2321	WS01021_A21
4	43.146	74	3	0.229	0.056	4.850	2321	WS01021_A21
4	124.132	83	1	-0.606	0.129	7.447	2321	WS01021_A21
4	142.917	85	1	0.402	0.081	9.254	2321	WS01021_A21
6	45.897	117	4	-0.242	0.039	4.026	2321	WS01021_A21
6	54.066	119	4	-0.228	0.056	5.662	2321	WS01021_A21
6	81.996	123	3	-0.261	0.083	4.733	2321	WS01021_A21
6	81.996	123	4	-0.460	0.141	8.126	2321	WS01021_A21
6	106.702	128	2	-0.497	0.181	6.142	2321	WS01021_A21
6	106.702	128	3	0.405	0.129	11.494	2321	WS01021_A21
8	50.001	154	1	0.290	0.075	8.264	2321	WS01021_A21
8	78.389	157	1	0.385	0.117	13.524	2321	WS01021_A21
8	87.463	158	1	-0.310	0.078	9.011	2321	WS01021_A21
9	2.011	167	4	0.224	0.039	4.008	2321	WS01021_A21
9	58.045	176	1	0.320	0.075	4.354	2321	WS01021_A21
11	41.984	212	2	-0.193	0.067	4.827	2321	WS01021_A21
11	46.026	217	1	0.267	0.061	7.092	2321	WS01021_A21
12	22.441	227	1	0.228	0.035	4.011	2321	WS01021_A21
13	3.386	245	1	0.305	0.082	9.444	2321	WS01021_A21
1	2.788	2	4	0.217	0.038	3.856	2322	WS01021_B16
3	9.953	42	3	-0.247	0.051	4.898	2322	WS01021_B16
3	148.745	68	1	0.260	0.050	4.753	2322	WS01021_B16
4	142.917	85	1	0.390	0.099	9.377	2322	WS01021_B16
5	57.017	102	4	-0.287	0.060	4.972	2322	WS01021_B16
10	9.580	187	3	-0.315	0.082	3.931	2322	WS01021_B16
12	28.493	228	2	-0.262	0.072	3.977	2322	WS01021_B16
1	40.464	7	3	0.296	0.047	4.202	2323	WS01021_D06
1	43.578	8	3	0.312	0.050	4.092	2323	WS01021_D06
4	55.925	76	1	-0.245	0.056	4.368	2323	WS01021_D06
4	173.929	88	2	-0.208	0.051	5.209	2323	WS01021_D06
8	11.974	150	1	-0.248	0.055	4.699	2323	WS01021_D06
3	15.147	44	2	0.332	0.104	7.283	2324	WS01021_D12
3	104.681	60	2	0.243	0.048	5.696	2324	WS01021_D12
3	112.868	61	2	-0.158	0.034	3.988	2324	WS01021_D12
3	114.747	62	2	-0.248	0.046	5.483	2324	WS01021_D12
6	87.891	124	2	-0.325	0.086	10.268	2324	WS01021_D12
6	89.401	125	2	-0.271	0.060	6.178	2324	WS01021_D12
6	106.702	128	2	0.461	0.110	5.213	2324	WS01021_D12
9	17.951	169	2	-0.243	0.040	4.100	2324	WS01021_D12
9	59.963	177	4	0.320	0.093	8.210	2324	WS01021_D12
11	0.000	199	2	0.281	0.058	7.035	2324	WS01021_D12
1	133.992	24	2	-0.203	0.037	3.908	2325	WS01021_E18
2	93.504	32	4	0.291	0.098	6.070	2325	WS01021_E18
3	69.071	52	1	-0.365	0.110	5.268	2325	WS01021_E18
6	26.350	115	2	0.241	0.058	6.159	2325	WS01021_E18

8	50.001	154	2	-0.191	0.041	4.310	2325	WS01021_E18
11	0.000	199	3	0.307	0.058	5.742	2325	WS01021_E18
11	40.960	210	2	0.407	0.097	5.040	2325	WS01021_E18
11	48.979	218	2	0.231	0.053	5.619	2325	WS01021_E18
13	1.815	244	2	0.243	0.056	6.129	2325	WS01021_E18
1	2.788	2	4	-0.160	0.046	4.393	2326	WS01021_F15
5	57.017	102	2	-0.315	0.056	8.006	2326	WS01021_F15
6	106.702	128	2	-0.460	0.075	5.747	2326	WS01021_F15
1	43.578	8	2	0.225	0.040	4.554	2327	WS0089_O23
1	95.572	17	2	-0.229	0.038	4.316	2327	WS0089_O23
1	95.572	17	3	0.284	0.047	5.096	2327	WS0089_O23
1	127.805	21	4	0.234	0.053	4.259	2327	WS0089_O23
4	55.925	76	1	0.189	0.049	3.861	2327	WS0089_O23
5	42.979	97	2	-0.262	0.057	6.485	2327	WS0089_O23
5	56.008	101	2	-0.202	0.034	4.169	2327	WS0089_O23
12	22.441	227	1	0.260	0.061	4.584	2327	WS0089_O23
12	14.406	230	3	-0.305	0.081	8.588	2327	WS0089_O23
12	0.000	233	1	-0.258	0.071	5.306	2327	WS0089_O23
1	95.572	17	2	0.264	0.037	4.169	2328	WS00928_P02
5	54.072	100	4	0.305	0.087	7.945	2328	WS00928_P02
6	87.891	124	2	-0.232	0.042	4.757	2328	WS00928_P02
9	36.227	165	4	-0.188	0.039	4.111	2328	WS00928_P02
9	59.963	177	4	0.321	0.096	8.174	2328	WS00928_P02
11	64.014	222	4	-0.338	0.070	5.189	2328	WS00928_P02
1	95.572	17	3	0.271	0.048	5.230	2329	WS00810_D18
5	7.672	92	1	-0.251	0.049	4.795	2329	WS00810_D18
6	25.914	114	1	-0.269	0.072	6.022	2329	WS00810_D18
6	45.897	117	1	0.246	0.055	4.574	2329	WS00810_D18
9	28.608	170	3	0.253	0.050	4.479	2329	WS00810_D18
11	35.604	207	3	-0.143	0.038	4.011	2329	WS00810_D18
11	42.600	213	3	-0.175	0.051	5.309	2329	WS00810_D18
12	32.473	238	1	0.213	0.046	3.881	2329	WS00810_D18
13	1.815	244	2	0.299	0.042	4.415	2329	WS00810_D18
13	1.815	244	4	-0.312	0.097	8.561	2329	WS00810_D18
1	2.788	2	4	-0.260	0.049	4.126	2330	WS00929_C03
3	137.891	64	3	0.285	0.072	5.268	2330	WS00929_C03
5	54.072	100	4	0.217	0.042	4.834	2330	WS00929_C03
7	105.477	147	2	-0.242	0.040	3.844	2330	WS00929_C03
11	9.237	202	4	0.225	0.040	4.619	2330	WS00929_C03
11	27.405	204	1	0.260	0.067	4.310	2330	WS00929_C03
11	27.405	204	4	-0.254	0.057	6.519	2330	WS00929_C03
11	45.989	216	1	-0.259	0.089	5.802	2330	WS00929_C03
4	82.462	78	1	-0.162	0.049	4.867	2331	WS01021_H16
1	38.326	6	2	0.238	0.035	4.026	2332	WS01021_M06
1	62.128	11	2	0.362	0.116	5.716	2332	WS01021_M06
1	69.854	13	2	0.152	0.034	3.860	2332	WS01021_M06
1	71.592	14	2	0.169	0.041	4.690	2332	WS01021_M06
3	47.928	49	1	-0.270	0.071	5.300	2332	WS01021_M06
3	112.868	61	2	0.207	0.051	5.766	2332	WS01021_M06
3	114.747	62	2	0.240	0.047	5.419	2332	WS01021_M06
4	82.462	78	2	-0.177	0.039	4.336	2332	WS01021_M06
4	92.039	79	2	-0.254	0.047	5.499	2332	WS01021_M06
11	40.960	210	4	0.367	0.092	7.440	2332	WS01021_M06
11	44.884	215	4	-0.307	0.058	4.378	2332	WS01021_M06

11	48.979	218	4	0.355	0.083	6.110	2332	WS01021_M06
3	112.868	61	2	-0.241	0.055	5.621	2333	WS0011_A04
3	114.747	62	2	-0.221	0.057	5.870	2333	WS0011_A04
3	116.958	63	4	-0.181	0.057	4.812	2333	WS0011_A04
3	141.443	65	3	0.287	0.069	4.337	2333	WS0011_A04
4	173.929	88	2	-0.158	0.041	4.240	2333	WS0011_A04
6	81.996	123	3	0.256	0.073	4.514	2333	WS0011_A04
9	17.951	169	2	-0.287	0.061	4.717	2333	WS0011_A04
9	49.013	173	1	0.257	0.050	4.148	2333	WS0011_A04
9	81.073	183	1	0.219	0.048	4.080	2333	WS0011_A04
10	77.676	192	1	-0.262	0.057	4.887	2333	WS0011_A04
10	80.088	194	1	-0.272	0.061	5.180	2333	WS0011_A04
2	48.839	29	4	0.315	0.053	3.975	2334	WS0011_B24
3	116.958	63	4	-0.214	0.067	5.629	2334	WS0011_B24
5	0.000	89	1	-0.279	0.056	5.095	2334	WS0011_B24
5	16.219	93	1	-0.302	0.070	6.316	2334	WS0011_B24
5	52.820	99	3	-0.289	0.039	3.869	2334	WS0011_B24
6	81.996	123	3	0.386	0.108	6.537	2334	WS0011_B24
7	74.560	143	1	0.223	0.058	5.267	2334	WS0011_B24
10	80.088	194	1	-0.257	0.041	3.865	2334	WS0011_B24
11	45.989	216	1	-0.252	0.054	4.925	2334	WS0011_B24
11	57.661	220	4	0.210	0.058	4.897	2334	WS0011_B24
11	64.014	222	4	-0.307	0.048	4.055	2334	WS0011_B24
13	0.000	243	4	0.336	0.140	4.223	2334	WS0011_B24
14	9.923	247	1	-0.204	0.048	4.354	2334	WS0011_B24
1	40.464	7	3	0.292	0.059	5.472	2335	WS0011_H22
1	43.578	8	3	0.283	0.053	4.871	2335	WS0011_H22
2	126.181	39	4	0.403	0.075	4.459	2335	WS0011_H22
5	0.000	89	1	-0.270	0.059	5.355	2335	WS0011_H22
5	16.219	93	1	-0.260	0.054	4.918	2335	WS0011_H22
5	31.191	95	1	-0.217	0.046	4.191	2335	WS0011_H22
7	62.787	139	2	-0.284	0.056	5.649	2335	WS0011_H22
7	105.477	147	2	-0.289	0.106	6.444	2335	WS0011_H22
8	62.580	155	1	0.337	0.109	3.845	2335	WS0011_H22
9	2.011	167	4	-0.285	0.065	6.289	2335	WS0011_H22
10	9.580	187	2	-0.240	0.073	4.422	2335	WS0011_H22
14	0.000	246	2	-0.255	0.076	6.244	2335	WS0011_H22
14	22.402	248	3	0.289	0.049	4.582	2335	WS0011_H22
14	43.392	250	2	0.345	0.126	11.484	2335	WS0011_H22
14	46.637	252	4	0.267	0.074	6.642	2335	WS0011_H22
1	29.800	5	1	0.200	0.046	4.039	2336	WS0011_I18
3	9.953	42	3	0.157	0.048	4.575	2336	WS0011_I18
3	137.891	64	3	0.294	0.057	5.454	2336	WS0011_I18
5	57.017	102	4	-0.266	0.050	4.495	2336	WS0011_I18
6	45.897	117	1	-0.277	0.042	3.907	2336	WS0011_I18
7	105.477	147	2	-0.266	0.091	5.375	2336	WS0011_I18
9	0.000	163	2	-0.516	0.132	5.285	2336	WS0011_I18
9	3.127	168	1	-0.357	0.123	4.820	2336	WS0011_I18
11	27.405	204	1	0.383	0.081	7.413	2336	WS0011_I18
11	27.405	204	4	-0.303	0.067	6.292	2336	WS0011_I18
11	29.069	205	1	0.257	0.049	4.419	2336	WS0011_I18
11	45.989	216	1	-0.198	0.062	5.464	2336	WS0011_I18
12	14.406	230	3	-0.282	0.049	4.745	2336	WS0011_I18
12	6.242	234	2	-0.289	0.050	6.866	2336	WS0011_I18

2	127.147	40	3	-0.238	0.047	4.282	2337	WS0011_O24
3	15.147	44	2	0.291	0.094	6.364	2337	WS0011_O24
3	20.512	46	2	0.259	0.073	8.177	2337	WS0011_O24
7	15.555	130	2	-0.293	0.056	6.328	2337	WS0011_O24
7	15.555	130	3	0.238	0.042	3.886	2337	WS0011_O24
9	81.073	183	1	0.191	0.060	4.554	2337	WS0011_O24
10	21.864	188	1	-0.339	0.055	4.237	2337	WS0011_O24
11	0.000	199	2	0.361	0.089	10.328	2337	WS0011_O24
11	27.405	204	1	0.259	0.054	4.212	2337	WS0011_O24
11	27.405	204	4	-0.253	0.039	3.846	2337	WS0011_O24
14	22.402	248	3	0.339	0.108	9.825	2337	WS0011_O24
14	43.392	250	2	-0.235	0.038	4.270	2337	WS0011_O24
1	29.800	5	1	0.168	0.056	3.997	2338	WS0012_A09
1	40.464	7	3	0.171	0.052	5.720	2338	WS0012_A09
1	43.578	8	3	0.171	0.053	6.262	2338	WS0012_A09
3	95.157	59	2	-0.269	0.037	5.283	2338	WS0012_A09
4	75.958	77	3	-0.222	0.068	4.218	2338	WS0012_A09
7	70.278	141	3	-0.267	0.055	6.061	2338	WS0012_A09
8	78.389	157	1	-0.378	0.101	7.474	2338	WS0012_A09
8	87.463	158	1	0.329	0.071	5.592	2338	WS0012_A09
9	28.608	170	2	-0.266	0.042	5.357	2338	WS0012_A09
11	27.405	204	1	0.407	0.111	8.414	2338	WS0012_A09
11	29.069	205	1	0.264	0.070	4.959	2338	WS0012_A09
1	71.592	14	2	0.274	0.043	4.570	2339	WS0012_D17
1	127.805	21	4	0.181	0.027	4.466	2339	WS0012_D17
4	124.132	83	1	-0.617	0.173	8.352	2339	WS0012_D17
4	142.917	85	1	0.274	0.049	4.266	2339	WS0012_D17
5	16.219	93	2	0.154	0.040	3.887	2339	WS0012_D17
6	45.897	117	1	-0.427	0.092	8.411	2339	WS0012_D17
6	49.100	118	4	-0.581	0.184	7.633	2339	WS0012_D17
9	17.951	169	2	-0.289	0.047	4.277	2339	WS0012_D17
11	27.405	204	1	0.360	0.051	4.808	2339	WS0012_D17
12	22.441	227	1	0.279	0.086	7.684	2339	WS0012_D17
12	51.382	242	1	0.444	0.124	5.924	2339	WS0012_D17
13	1.815	244	4	-0.358	0.047	5.292	2339	WS0012_D17
1	2.788	2	4	-0.245	0.056	5.992	2340	WS0012_F02
3	80.211	55	4	0.291	0.059	6.497	2340	WS0012_F02
7	105.477	147	2	-0.261	0.043	3.869	2340	WS0012_F02
11	0.000	199	2	0.186	0.035	4.321	2340	WS0012_F02
11	27.405	204	1	0.337	0.111	7.231	2340	WS0012_F02
11	27.405	204	4	-0.232	0.055	5.919	2340	WS0012_F02
12	14.406	230	4	0.261	0.060	5.798	2340	WS0012_F02
12	6.242	234	2	-0.229	0.025	4.798	2340	WS0012_F02
1	2.788	2	4	-0.198	0.042	4.156	2341	WS00943_M18
3	104.681	60	2	0.205	0.047	4.369	2341	WS00943_M18
3	114.747	62	2	-0.238	0.048	4.505	2341	WS00943_M18
4	55.925	76	1	-0.243	0.050	4.275	2341	WS00943_M18
4	82.462	78	1	-0.221	0.043	4.112	2341	WS00943_M18
5	16.219	93	1	-0.236	0.043	4.050	2341	WS00943_M18
5	57.017	102	4	-0.224	0.038	4.134	2341	WS00943_M18
6	87.891	124	2	-0.212	0.049	4.596	2341	WS00943_M18
11	27.405	204	1	0.303	0.072	6.870	2341	WS00943_M18
11	29.069	205	1	0.223	0.050	4.758	2341	WS00943_M18
11	45.989	216	1	-0.216	0.050	4.730	2341	WS00943_M18

12	6.242	234	2	-0.221	0.049	5.410	2341	WS00943_M18
1	2.788	2	4	-0.251	0.063	5.688	2342	WS00943_N06
3	9.953	42	3	0.190	0.052	4.755	2342	WS00943_N06
3	15.147	44	2	0.271	0.061	4.140	2342	WS00943_N06
3	104.681	60	2	0.181	0.043	4.193	2342	WS00943_N06
3	112.868	61	2	-0.206	0.040	3.869	2342	WS00943_N06
3	114.747	62	2	-0.201	0.054	5.210	2342	WS00943_N06
9	49.013	173	1	0.227	0.045	3.903	2342	WS00943_N06
9	59.963	177	4	0.248	0.044	4.136	2342	WS00943_N06
11	27.405	204	1	0.325	0.084	7.745	2342	WS00943_N06
11	27.405	204	4	-0.265	0.061	5.857	2342	WS00943_N06
11	45.989	216	1	-0.190	0.048	4.381	2342	WS00943_N06
1	45.794	9	1	0.277	0.062	4.406	2343	WS00730_B24
1	50.955	10	1	-0.245	0.056	4.021	2343	WS00730_B24
1	107.333	19	4	-0.197	0.041	4.351	2343	WS00730_B24
3	47.928	49	1	0.231	0.083	5.876	2343	WS00730_B24
3	144.593	66	4	0.199	0.041	4.788	2343	WS00730_B24
3	154.505	69	4	0.188	0.035	3.918	2343	WS00730_B24
4	173.929	88	2	-0.210	0.035	4.096	2343	WS00730_B24
5	54.072	100	4	-0.266	0.056	6.317	2343	WS00730_B24
5	84.347	105	2	0.200	0.034	3.929	2343	WS00730_B24
6	45.897	117	4	-0.268	0.043	4.211	2343	WS00730_B24
6	54.066	119	4	-0.289	0.076	8.573	2343	WS00730_B24
6	69.073	121	3	0.271	0.071	5.311	2343	WS00730_B24
6	69.073	121	4	0.381	0.092	10.330	2343	WS00730_B24
7	75.883	144	4	-0.249	0.035	3.956	2343	WS00730_B24
9	55.358	175	1	0.336	0.100	7.309	2343	WS00730_B24
9	61.113	178	1	-0.327	0.115	4.036	2343	WS00730_B24
11	0.000	199	2	0.245	0.044	5.115	2343	WS00730_B24
11	64.014	222	4	0.251	0.042	4.844	2343	WS00730_B24
12	0.000	233	1	0.233	0.057	4.035	2343	WS00730_B24
13	3.386	245	1	0.221	0.069	4.906	2343	WS00730_B24
14	43.392	250	2	-0.197	0.044	4.568	2343	WS00730_B24
4	97.576	80	2	0.176	0.025	4.064	2344	WS00730_D22
5	54.072	100	4	0.229	0.037	4.254	2344	WS00730_D22
6	87.891	124	2	-0.211	0.040	5.115	2344	WS00730_D22
6	89.401	125	2	-0.254	0.035	4.491	2344	WS00730_D22
8	50.001	154	1	-0.205	0.063	3.944	2344	WS00730_D22
9	59.963	177	4	0.196	0.038	4.430	2344	WS00730_D22
11	0.000	199	2	0.342	0.055	7.083	2344	WS00730_D22
11	27.405	204	1	0.229	0.065	3.980	2344	WS00730_D22
1	43.578	8	2	0.232	0.031	4.004	2345	WS00730_J03
1	45.794	9	1	0.225	0.066	4.336	2345	WS00730_J03
1	45.794	9	3	-0.312	0.046	4.336	2345	WS00730_J03
2	101.076	35	4	0.263	0.070	7.301	2345	WS00730_J03
3	80.211	55	4	0.178	0.046	4.345	2345	WS00730_J03
3	95.157	59	2	0.207	0.040	4.387	2345	WS00730_J03
3	137.891	64	3	0.350	0.125	8.076	2345	WS00730_J03
4	6.032	72	4	-0.221	0.062	5.853	2345	WS00730_J03
4	82.462	78	2	-0.323	0.064	6.835	2345	WS00730_J03
4	92.039	79	2	-0.322	0.041	5.284	2345	WS00730_J03
4	97.576	80	2	0.233	0.060	7.205	2345	WS00730_J03
5	52.820	99	1	0.290	0.088	5.418	2345	WS00730_J03
5	54.072	100	4	0.297	0.074	7.019	2345	WS00730_J03

5	57.017	102	4	-0.275	0.049	4.409	2345	WS00730_J03
5	86.669	106	4	0.308	0.155	4.267	2345	WS00730_J03
7	105.477	147	2	-0.240	0.073	5.010	2345	WS00730_J03
9	0.000	163	2	-0.451	0.069	4.061	2345	WS00730_J03
11	0.000	199	2	0.337	0.051	6.562	2345	WS00730_J03
11	60.464	221	1	0.273	0.080	4.836	2345	WS00730_J03
13	1.815	244	4	-0.261	0.064	4.966	2345	WS00730_J03
4	92.039	79	2	-0.228	0.039	5.129	2346	WS00730_K05
9	0.000	163	2	-0.492	0.055	4.516	2346	WS00730_K05
9	36.227	165	3	0.152	0.032	4.423	2346	WS00730_K05
11	41.984	212	2	0.176	0.032	4.258	2346	WS00730_K05
12	22.441	227	3	-0.262	0.049	4.356	2346	WS00730_K05
1	95.572	17	2	0.240	0.033	4.417	2347	WS00730_N19
3	9.953	42	3	0.156	0.046	4.477	2347	WS00730_N19
3	137.891	64	3	0.513	0.188	17.755	2347	WS00730_N19
3	141.443	65	3	0.578	0.280	13.668	2347	WS00730_N19
4	82.462	78	2	-0.250	0.042	5.464	2347	WS00730_N19
4	97.576	80	2	0.259	0.036	5.303	2347	WS00730_N19
4	142.917	85	1	0.253	0.066	4.218	2347	WS00730_N19
4	142.917	85	3	-0.272	0.061	6.084	2347	WS00730_N19
4	173.929	88	2	-0.154	0.043	5.275	2347	WS00730_N19
6	87.891	124	2	-0.197	0.059	7.303	2347	WS00730_N19
6	89.401	125	2	-0.393	0.076	9.687	2347	WS00730_N19
10	78.479	193	3	0.332	0.062	6.104	2347	WS00730_N19
11	0.000	199	2	0.315	0.047	6.090	2347	WS00730_N19
11	27.405	204	1	0.334	0.081	5.768	2347	WS00730_N19
11	27.405	204	4	-0.412	0.094	8.731	2347	WS00730_N19
12	6.242	234	2	-0.214	0.025	4.720	2347	WS00730_N19
3	137.891	64	3	0.222	0.065	4.002	2348	WS00730_P04
4	0.000	71	4	0.234	0.034	3.897	2348	WS00730_P04
4	142.917	85	3	-0.333	0.099	6.426	2348	WS00730_P04
4	173.929	88	2	-0.178	0.036	4.182	2348	WS00730_P04
8	87.463	158	2	-0.287	0.065	7.114	2348	WS00730_P04
9	59.963	177	4	0.252	0.062	6.618	2348	WS00730_P04
1	69.854	13	2	-0.211	0.035	4.270	2349	WS00113_M07
1	71.592	14	2	-0.203	0.032	3.939	2349	WS00113_M07
1	77.177	15	2	-0.239	0.046	5.678	2349	WS00113_M07
1	95.572	17	2	-0.274	0.032	3.897	2349	WS00113_M07
9	81.073	183	1	-0.221	0.077	5.176	2349	WS00113_M07
11	17.088	203	2	0.236	0.063	5.090	2349	WS00113_M07
11	40.960	210	2	0.448	0.075	5.400	2349	WS00113_M07
11	43.491	214	4	0.321	0.088	8.349	2349	WS00113_M07
11	45.989	216	2	-0.152	0.030	3.896	2349	WS00113_M07
12	32.473	238	1	-0.181	0.063	4.157	2349	WS00113_M07
14	44.898	251	3	0.218	0.054	5.157	2349	WS00113_M07
1	107.333	19	4	0.167	0.039	3.968	2350	WS00113_N21
1	127.805	21	4	-0.211	0.052	4.939	2350	WS00113_N21
1	131.686	23	1	-0.530	0.104	4.816	2350	WS00113_N21
2	127.147	40	3	-0.165	0.046	4.151	2350	WS00113_N21
8	78.389	157	1	-0.240	0.058	4.208	2350	WS00113_N21
12	14.406	230	4	0.412	0.092	7.996	2350	WS00113_N21
1	82.301	16	4	-0.234	0.063	6.738	2351	WS0021_B17
2	48.839	29	4	-0.480	0.127	13.477	2351	WS0021_B17
5	0.000	89	1	-0.232	0.061	3.940	2351	WS0021_B17

10	21.864	188	1	0.239	0.068	4.343	2351	WS0021_B17
11	57.661	220	4	-0.201	0.038	4.210	2351	WS0021_B17
13	1.815	244	2	-0.253	0.038	4.892	2351	WS0021_B17
3	95.157	59	2	-0.223	0.054	6.675	2352	WS0021_D12
8	87.463	158	2	-0.216	0.041	4.559	2352	WS0021_D12
10	9.580	187	3	-0.286	0.062	4.227	2353	WS0021_H05
10	80.088	194	1	-0.281	0.047	4.987	2353	WS0021_H05
11	27.405	204	1	0.187	0.039	4.124	2353	WS0021_H05
11	45.989	216	1	-0.218	0.046	4.797	2353	WS0021_H05
1	131.686	23	1	-0.663	0.196	7.467	2354	WS0021_I08
3	104.681	60	2	0.250	0.049	5.198	2354	WS0021_I08
3	112.868	61	2	-0.217	0.039	4.146	2354	WS0021_I08
3	137.891	64	3	0.360	0.092	9.193	2354	WS0021_I08
4	116.928	82	2	-0.246	0.047	4.938	2354	WS0021_I08
6	81.996	123	3	0.279	0.085	3.914	2354	WS0021_I08
8	78.389	157	1	-0.173	0.054	4.455	2354	WS0021_I08
8	87.463	158	1	0.204	0.070	5.822	2354	WS0021_I08
9	0.000	163	1	-0.472	0.108	4.911	2354	WS0021_I08
9	36.227	165	3	0.234	0.045	4.499	2354	WS0021_I08
11	9.237	202	4	0.290	0.080	7.021	2354	WS0021_I08
1	29.800	5	2	0.202	0.035	4.245	2355	WS0021_M05
3	137.891	64	3	0.371	0.113	12.722	2355	WS0021_M05
4	75.958	77	3	-0.470	0.135	8.778	2355	WS0021_M05
4	116.928	82	2	-0.339	0.064	7.997	2355	WS0021_M05
9	36.227	165	3	0.225	0.037	4.204	2355	WS0021_M05
10	21.864	188	1	0.281	0.069	4.275	2355	WS0021_M05
11	42.600	213	3	-0.208	0.053	5.990	2355	WS0021_M05
12	6.242	234	2	-0.202	0.035	5.243	2355	WS0021_M05
14	44.898	251	3	0.268	0.040	3.961	2355	WS0021_M05
1	131.686	23	2	-0.446	0.081	4.760	2356	WS0021_N12
2	48.839	29	4	0.252	0.051	3.928	2356	WS0021_N12
8	87.463	158	2	-0.224	0.041	4.344	2356	WS0021_N12
2	63.261	30	3	0.207	0.062	6.407	2357	WS00731_G21
4	55.925	76	1	-0.270	0.082	8.308	2357	WS00731_G21
6	81.996	123	4	0.432	0.141	7.506	2357	WS00731_G21
8	62.580	155	1	-0.318	0.070	3.945	2357	WS00731_G21
8	78.389	157	1	-0.191	0.049	5.009	2357	WS00731_G21
10	70.246	190	4	0.265	0.053	4.126	2357	WS00731_G21
10	86.151	195	3	0.279	0.053	5.610	2357	WS00731_G21
10	95.795	197	4	-0.165	0.052	4.354	2357	WS00731_G21
11	45.989	216	1	-0.316	0.075	7.746	2357	WS00731_G21
1	82.301	16	3	0.268	0.042	4.461	2358	WS00112_J15
3	83.563	56	3	-0.164	0.037	3.859	2358	WS00112_J15
3	95.157	59	2	-0.171	0.034	4.189	2358	WS00112_J15
4	116.928	82	2	-0.283	0.059	6.659	2358	WS00112_J15
5	31.191	95	1	-0.209	0.054	4.130	2358	WS00112_J15
5	86.669	106	2	-0.271	0.059	4.215	2358	WS00112_J15
13	1.815	244	2	-0.223	0.043	4.786	2358	WS00112_J15
13	1.815	244	4	0.384	0.082	7.146	2358	WS00112_J15
1	29.800	5	1	0.307	0.077	7.853	2359	WS00732_B03
2	95.880	34	3	-0.115	0.046	4.618	2359	WS00732_B03
2	120.617	38	3	0.225	0.062	4.898	2359	WS00732_B03
2	126.181	39	4	-0.351	0.070	4.014	2359	WS00732_B03
2	127.147	40	3	-0.224	0.049	3.954	2359	WS00732_B03

3	141.443	65	3	0.400	0.180	8.948	2359	WS00732_B03
6	56.188	120	1	-0.293	0.062	6.329	2359	WS00732_B03
6	69.073	121	4	-0.281	0.086	9.905	2359	WS00732_B03
6	76.376	122	1	0.219	0.113	5.855	2359	WS00732_B03
6	89.401	125	1	-0.166	0.053	5.327	2359	WS00732_B03
7	73.169	142	2	-0.595	0.154	6.016	2359	WS00732_B03
8	0.000	148	4	-0.716	0.402	43.426	2359	WS00732_B03
8	11.974	150	1	-0.649	0.213	22.464	2359	WS00732_B03
8	39.090	152	4	0.255	0.045	4.845	2359	WS00732_B03
11	17.088	203	2	-0.134	0.059	6.551	2359	WS00732_B03
11	46.026	217	1	0.322	0.044	4.833	2359	WS00732_B03
12	0.000	233	1	-0.279	0.039	4.126	2359	WS00732_B03
12	51.382	242	1	0.503	0.101	5.570	2359	WS00732_B03
2	126.181	39	2	-0.259	0.040	4.945	2360	WS00113_D16
4	0.000	71	4	-0.229	0.039	4.412	2360	WS00113_D16
4	103.719	81	4	-0.154	0.033	4.117	2360	WS00113_D16
5	0.000	89	1	-0.186	0.059	3.950	2360	WS00113_D16
5	54.072	100	4	0.244	0.044	4.997	2360	WS00113_D16
6	69.073	121	3	0.244	0.056	4.186	2360	WS00113_D16
9	36.227	165	4	-0.229	0.041	5.230	2360	WS00113_D16
5	0.000	89	3	-0.234	0.068	6.606	2361	WS00941_P01
8	62.580	155	1	-0.339	0.122	5.650	2361	WS00941_P01
9	36.227	165	3	-0.269	0.047	4.715	2361	WS00941_P01
9	49.013	173	2	0.203	0.061	4.309	2361	WS00941_P01
11	64.014	222	3	-0.197	0.053	5.214	2361	WS00941_P01
1	43.578	8	2	0.192	0.045	4.000	2362	WS01040_H02
1	45.794	9	1	0.293	0.038	3.851	2362	WS01040_H02
1	50.955	10	1	-0.252	0.055	5.455	2362	WS01040_H02
1	82.301	16	3	-0.216	0.041	4.098	2362	WS01040_H02
2	102.814	36	1	0.208	0.049	4.769	2362	WS01040_H02
3	14.500	43	2	0.243	0.071	6.756	2362	WS01040_H02
3	15.147	44	2	-0.248	0.081	4.553	2362	WS01040_H02
3	20.512	46	2	-0.249	0.060	5.340	2362	WS01040_H02
4	55.925	76	1	0.227	0.043	4.186	2362	WS01040_H02
4	92.039	79	4	-0.295	0.048	3.963	2362	WS01040_H02
5	3.484	90	2	0.278	0.109	5.460	2362	WS01040_H02
6	69.073	121	3	-0.270	0.053	5.283	2362	WS01040_H02
8	87.463	158	2	0.255	0.072	6.314	2362	WS01040_H02
10	70.246	190	2	0.182	0.043	3.931	2362	WS01040_H02
12	14.406	230	4	-0.225	0.049	4.702	2362	WS01040_H02
13	1.815	244	2	0.231	0.055	4.841	2362	WS01040_H02
14	9.923	247	1	-0.259	0.057	5.739	2362	WS01040_H02
4	103.719	81	4	-0.289	0.075	6.651	2363	WS0014_N09
5	7.672	92	1	-0.263	0.070	4.798	2363	WS0014_N09
5	42.979	97	2	-0.271	0.031	4.267	2363	WS0014_N09
6	25.742	113	3	-0.168	0.043	4.222	2363	WS0014_N09
6	87.891	124	2	0.177	0.040	4.869	2363	WS0014_N09
8	50.001	154	2	-0.171	0.043	5.350	2363	WS0014_N09
8	87.463	158	2	0.297	0.061	7.549	2363	WS0014_N09
11	17.088	203	2	0.192	0.043	4.718	2363	WS0014_N09
1	77.177	15	2	0.215	0.050	4.099	2364	WS0081_A10
1	95.572	17	2	0.326	0.072	6.227	2364	WS0081_A10
3	9.953	42	3	0.226	0.072	6.214	2364	WS0081_A10
3	14.500	43	2	-0.201	0.067	5.653	2364	WS0081_A10

3	15.147	44	2	0.245	0.117	7.063	2364	WS0081_A10
3	20.512	46	2	0.278	0.047	4.061	2364	WS0081_A10
3	141.443	65	3	0.370	0.082	5.420	2364	WS0081_A10
9	49.013	173	1	0.275	0.049	5.034	2364	WS0081_A10
9	59.963	177	4	0.196	0.054	4.476	2364	WS0081_A10
11	0.000	199	2	0.305	0.049	4.118	2364	WS0081_A10
11	0.000	199	3	-0.274	0.053	5.048	2364	WS0081_A10
11	27.405	204	1	0.312	0.049	5.506	2364	WS0081_A10
11	64.014	222	4	-0.325	0.058	6.203	2364	WS0081_A10
14	43.392	250	2	-0.219	0.045	3.961	2364	WS0081_A10
3	9.953	42	3	0.253	0.057	4.765	2365	WS0015_L15
3	137.891	64	3	0.301	0.105	7.670	2365	WS0015_L15
3	154.505	69	4	-0.207	0.046	4.540	2365	WS0015_L15
4	173.929	88	2	-0.194	0.034	4.380	2365	WS0015_L15
7	15.555	130	2	-0.382	0.084	10.949	2365	WS0015_L15
7	105.477	147	2	-0.301	0.088	7.111	2365	WS0015_L15
8	87.463	158	2	-0.202	0.038	4.641	2365	WS0015_L15
9	0.000	163	2	-0.463	0.088	4.041	2365	WS0015_L15
11	0.000	199	2	0.329	0.060	7.963	2365	WS0015_L15
11	17.088	203	2	-0.311	0.076	8.251	2365	WS0015_L15
12	14.406	230	4	0.287	0.043	3.980	2365	WS0015_L15
12	6.242	234	2	-0.271	0.054	7.544	2365	WS0015_L15
1	22.419	4	3	0.235	0.116	3.993	2366	WS0081_I07
1	43.578	8	3	0.267	0.044	3.956	2366	WS0081_I07
1	62.128	11	1	-0.461	0.121	4.159	2366	WS0081_I07
2	127.147	40	4	-0.157	0.054	5.661	2366	WS0081_I07
9	0.000	163	1	0.454	0.132	4.535	2366	WS0081_I07
10	9.580	187	3	0.338	0.127	5.044	2366	WS0081_I07
12	22.441	227	1	0.256	0.076	5.304	2366	WS0081_I07
2	48.839	29	4	0.268	0.056	4.643	2367	WS00929_F10
5	56.008	101	2	-0.208	0.047	5.624	2367	WS00929_F10
5	57.017	102	2	0.219	0.036	4.521	2367	WS00929_F10
6	25.914	114	1	-0.190	0.057	3.867	2367	WS00929_F10
10	21.864	188	1	0.338	0.093	6.270	2367	WS00929_F10
10	78.479	193	4	-0.212	0.037	3.868	2367	WS00929_F10
11	42.600	213	3	-0.248	0.075	6.543	2367	WS00929_F10
3	9.953	42	3	0.194	0.057	6.654	2368	WS00929_I15
3	14.500	43	2	-0.239	0.037	4.499	2368	WS00929_I15
3	83.563	56	3	-0.316	0.089	10.404	2368	WS00929_I15
5	54.072	100	4	0.264	0.083	5.956	2368	WS00929_I15
9	0.000	163	2	0.439	0.060	3.888	2368	WS00929_I15
9	55.358	175	1	-0.324	0.065	4.942	2368	WS00929_I15
9	69.046	181	2	0.349	0.093	4.131	2368	WS00929_I15
10	9.580	187	2	-0.340	0.187	9.034	2368	WS00929_I15
11	64.014	222	3	-0.176	0.042	4.915	2368	WS00929_I15
14	0.000	246	2	-0.226	0.036	4.626	2368	WS00929_I15
4	142.917	85	1	0.267	0.051	4.348	2369	WS00713_O14
6	69.073	121	3	0.299	0.069	6.742	2369	WS00713_O14
6	81.996	123	3	0.306	0.091	5.560	2369	WS00713_O14
10	70.246	190	2	-0.207	0.044	4.577	2369	WS00713_O14
11	27.405	204	1	0.259	0.070	6.068	2369	WS00713_O14
12	14.406	230	4	0.248	0.063	4.662	2369	WS00713_O14
5	3.484	90	2	-0.355	0.104	4.977	2370	WS00713_P14
5	7.672	92	1	-0.290	0.052	6.747	2370	WS00713_P14

6	81.996	123	3	0.342	0.090	6.917	2370	WS00713_P14
8	78.389	157	1	-0.242	0.058	5.727	2370	WS00713_P14
10	21.864	188	1	0.220	0.042	4.189	2370	WS00713_P14
10	78.479	193	3	0.375	0.042	5.239	2370	WS00713_P14
11	0.000	199	3	-0.230	0.043	5.443	2370	WS00713_P14
11	45.989	216	1	-0.286	0.076	7.462	2370	WS00713_P14
12	14.406	230	4	0.295	0.097	5.610	2370	WS00713_P14
14	46.637	252	4	0.249	0.062	4.191	2370	WS00713_P14
12	22.441	227	1	0.195	0.044	4.261	2371	WS0104_B19
14	0.000	246	2	-0.244	0.058	4.830	2371	WS0104_B19
1	15.589	3	2	-0.173	0.046	4.752	2372	WS0104_C09
1	45.794	9	3	-0.214	0.052	5.768	2372	WS0104_C09
2	95.880	34	1	0.355	0.050	4.281	2372	WS0104_C09
3	69.071	52	1	-0.299	0.101	3.881	2372	WS0104_C09
10	35.204	189	1	0.349	0.099	8.351	2372	WS0104_C09
10	78.479	193	3	0.448	0.074	7.037	2372	WS0104_C09
13	0.000	243	4	0.275	0.122	5.618	2372	WS0104_C09
1	45.794	9	1	-0.318	0.069	5.723	2373	WS0104_E04
2	48.839	29	4	0.171	0.034	4.876	2373	WS0104_E04
2	75.550	31	2	-0.364	0.067	4.731	2373	WS0104_E04
2	102.814	36	2	-0.199	0.061	6.640	2373	WS0104_E04
3	116.958	63	4	-0.255	0.038	5.322	2373	WS0104_E04
3	144.647	67	1	0.396	0.130	8.270	2373	WS0104_E04
3	148.745	68	4	-0.241	0.050	7.430	2373	WS0104_E04
5	57.017	102	4	0.282	0.037	5.152	2373	WS0104_E04
6	49.100	118	4	0.341	0.107	5.495	2373	WS0104_E04
7	15.555	130	2	0.256	0.038	4.267	2373	WS0104_E04
7	62.787	139	4	-0.273	0.038	6.398	2373	WS0104_E04
7	67.154	140	4	-0.347	0.077	4.847	2373	WS0104_E04
7	73.169	142	1	0.480	0.063	3.973	2373	WS0104_E04
7	75.883	144	4	-0.229	0.035	4.908	2373	WS0104_E04
8	0.000	148	4	-0.275	0.035	5.078	2373	WS0104_E04
8	87.463	158	2	0.259	0.060	4.255	2373	WS0104_E04
9	0.000	166	1	0.150	0.052	4.384	2373	WS0104_E04
13	1.815	244	4	0.332	0.075	10.858	2373	WS0104_E04
1	133.992	24	2	-0.181	0.039	4.301	2374	WS0104_E13
1	141.021	25	2	0.202	0.049	5.344	2374	WS0104_E13
3	116.958	63	4	-0.195	0.054	5.758	2374	WS0104_E13
5	16.219	93	2	0.155	0.035	3.854	2374	WS0104_E13
8	78.389	157	1	-0.253	0.055	4.325	2374	WS0104_E13
9	0.000	166	1	-0.282	0.098	7.670	2374	WS0104_E13
11	0.000	199	3	-0.300	0.065	4.285	2374	WS0104_E13
13	3.386	245	1	-0.287	0.104	8.119	2374	WS0104_E13
14	0.000	246	1	0.292	0.066	5.211	2374	WS0104_E13
3	95.157	59	2	-0.170	0.040	4.219	2375	WS0104_G04
4	6.032	72	4	0.224	0.045	4.294	2375	WS0104_G04
4	116.928	82	2	-0.244	0.039	4.158	2375	WS0104_G04
6	95.249	126	1	0.380	0.194	7.576	2375	WS0104_G04
7	17.610	131	3	0.362	0.126	6.744	2375	WS0104_G04
7	75.883	144	4	-0.299	0.047	4.058	2375	WS0104_G04
8	11.974	150	1	-0.203	0.047	3.909	2375	WS0104_G04
9	55.358	175	1	0.298	0.053	4.520	2375	WS0104_G04
10	78.479	193	3	-0.313	0.063	5.865	2375	WS0104_G04
12	0.000	233	1	-0.193	0.051	4.220	2375	WS0104_G04

12	22.629	237	1	-0.211	0.057	4.715	2375	WS0104_G04
12	32.473	238	1	-0.277	0.092	7.601	2375	WS0104_G04
13	3.386	245	1	0.295	0.070	5.851	2375	WS0104_G04
3	14.500	43	2	-0.260	0.046	4.783	2376	WS0104_G11
3	20.512	46	2	0.238	0.070	7.322	2376	WS0104_G11
3	80.211	55	4	0.241	0.049	5.321	2376	WS0104_G11
7	15.555	130	2	-0.260	0.055	5.783	2376	WS0104_G11
9	55.358	175	1	0.245	0.049	4.075	2376	WS0104_G11
9	69.046	181	2	0.308	0.112	3.853	2376	WS0104_G11
5	0.000	89	1	-0.329	0.053	4.203	2377	WS00837_J24
5	31.191	95	1	-0.309	0.050	4.109	2377	WS00837_J24
5	54.072	100	4	0.203	0.038	4.109	2377	WS00837_J24
7	15.555	130	2	-0.371	0.079	8.960	2377	WS00837_J24
7	17.610	131	3	-0.377	0.115	5.498	2377	WS00837_J24
9	81.073	183	1	0.245	0.087	6.627	2377	WS00837_J24
10	104.214	198	2	-0.189	0.032	5.507	2377	WS00837_J24
11	27.405	204	1	0.393	0.121	9.738	2377	WS00837_J24
11	35.604	207	3	-0.200	0.060	4.816	2377	WS00837_J24
11	37.143	209	3	0.214	0.068	5.457	2377	WS00837_J24
11	43.491	214	4	-0.249	0.058	6.032	2377	WS00837_J24
11	57.661	220	4	0.121	0.047	5.022	2377	WS00837_J24
11	64.014	222	4	-0.257	0.075	6.771	2377	WS00837_J24
2	48.839	29	4	0.382	0.084	9.032	2378	WS00837_L09
9	81.073	183	3	0.200	0.062	4.288	2378	WS00837_L09
14	9.923	247	1	-0.275	0.076	5.977	2378	WS00837_L09
3	95.157	59	2	-0.206	0.028	4.443	2379	WS00837_P21
3	114.747	62	2	-0.219	0.036	4.564	2379	WS00837_P21
3	137.891	64	3	0.280	0.054	6.572	2379	WS00837_P21
6	56.188	120	2	-0.184	0.029	4.396	2379	WS00837_P21
11	27.405	204	1	0.401	0.134	8.390	2379	WS00837_P21
11	29.069	205	1	0.266	0.082	4.906	2379	WS00837_P21
1	2.788	2	4	-0.238	0.096	7.522	2380	WS00943_A14
2	63.261	30	3	0.220	0.039	4.225	2380	WS00943_A14
2	93.504	32	4	0.216	0.051	4.009	2380	WS00943_A14
3	83.563	56	3	-0.200	0.039	4.236	2380	WS00943_A14
7	105.477	147	2	-0.308	0.139	7.296	2380	WS00943_A14
9	28.608	170	3	0.319	0.068	4.310	2380	WS00943_A14
9	55.358	175	3	0.612	0.149	10.190	2380	WS00943_A14
9	81.073	183	3	0.245	0.038	4.118	2380	WS00943_A14
11	43.491	214	4	-0.317	0.115	9.082	2380	WS00943_A14
11	44.884	215	4	0.338	0.076	6.094	2380	WS00943_A14
11	45.989	216	1	-0.162	0.041	3.874	2380	WS00943_A14
11	57.661	220	4	0.226	0.085	6.860	2380	WS00943_A14
11	64.014	222	4	-0.240	0.056	4.296	2380	WS00943_A14
12	0.000	226	3	0.194	0.044	4.680	2380	WS00943_A14
14	0.000	246	1	0.250	0.042	4.133	2380	WS00943_A14
4	116.928	82	2	0.317	0.048	5.729	2381	WS00943_D18
5	86.669	106	2	0.174	0.079	4.085	2381	WS00943_D18
6	26.350	115	2	0.203	0.036	4.154	2381	WS00943_D18
6	81.996	123	3	-0.329	0.125	5.850	2381	WS00943_D18
6	106.702	128	2	-0.586	0.154	8.522	2381	WS00943_D18
9	55.358	175	1	-0.346	0.095	7.028	2381	WS00943_D18
9	61.113	178	1	0.422	0.279	6.893	2381	WS00943_D18
10	104.214	198	4	0.184	0.035	4.985	2381	WS00943_D18

13	0.000	243	4	-0.408	0.211	5.225	2381	WS00943_D18
1	2.788	2	4	-0.161	0.057	5.211	2382	WS00943_F05
1	29.800	5	1	0.205	0.053	4.628	2382	WS00943_F05
1	43.578	8	2	0.171	0.039	4.283	2382	WS00943_F05
1	107.333	19	3	0.144	0.035	4.074	2382	WS00943_F05
1	133.992	24	2	-0.161	0.056	5.876	2382	WS00943_F05
1	141.021	25	2	0.155	0.054	5.657	2382	WS00943_F05
2	126.181	39	2	0.328	0.047	5.205	2382	WS00943_F05
10	35.204	189	2	-0.117	0.039	4.384	2382	WS00943_F05
11	27.405	204	1	0.312	0.055	4.557	2382	WS00943_F05
12	22.441	227	1	0.204	0.052	4.273	2382	WS00943_F05
3	47.928	49	1	0.223	0.049	3.993	2383	WS00943_I09
3	141.443	65	3	-0.298	0.092	3.868	2383	WS00943_I09
4	0.000	71	4	-0.229	0.044	4.589	2383	WS00943_I09
4	116.928	82	4	0.213	0.050	5.158	2383	WS00943_I09
6	76.376	122	1	0.351	0.173	7.013	2383	WS00943_I09
7	62.787	139	2	0.270	0.045	4.465	2383	WS00943_I09
11	40.960	210	2	0.442	0.094	6.550	2383	WS00943_I09
11	40.960	210	4	-0.311	0.042	4.450	2383	WS00943_I09
11	64.014	222	4	-0.222	0.043	4.622	2383	WS00943_I09
1	95.572	17	2	0.225	0.040	4.258	2384	WS00943_K10
3	137.891	64	3	0.239	0.048	4.640	2384	WS00943_K10
7	73.169	142	2	0.427	0.067	4.131	2384	WS00943_K10
8	62.580	155	1	-0.420	0.207	5.627	2384	WS00943_K10
8	78.389	157	1	-0.314	0.094	7.771	2384	WS00943_K10
9	59.963	177	4	0.329	0.077	7.040	2384	WS00943_K10
11	45.989	216	1	-0.228	0.071	5.805	2384	WS00943_K10
1	43.578	8	2	-0.207	0.033	3.967	2385	WS00729_B13
4	55.925	76	1	-0.291	0.089	5.759	2385	WS00729_B13
4	173.929	88	2	-0.175	0.035	4.267	2385	WS00729_B13
5	54.072	100	4	0.193	0.045	4.978	2385	WS00729_B13
6	81.996	123	3	0.344	0.101	4.779	2385	WS00729_B13
9	59.963	177	4	0.298	0.075	8.194	2385	WS00729_B13
9	81.073	183	1	0.274	0.104	7.186	2385	WS00729_B13
11	0.000	199	2	0.249	0.035	4.197	2385	WS00729_B13
11	40.960	210	2	0.365	0.045	4.362	2385	WS00729_B13
1	62.128	11	2	-0.261	0.065	5.049	2386	WS00834_A17
2	127.147	40	3	-0.188	0.041	4.421	2386	WS00834_A17
7	73.169	142	1	0.501	0.147	4.019	2386	WS00834_A17
1	15.589	3	2	-0.216	0.055	4.414	2387	WS00940_A06
4	116.928	82	4	0.237	0.060	4.126	2387	WS00940_A06
4	142.917	85	1	0.259	0.042	4.554	2387	WS00940_A06
4	173.929	88	2	-0.237	0.062	4.952	2387	WS00940_A06
6	69.073	121	3	0.228	0.033	3.925	2387	WS00940_A06
7	59.789	137	3	-0.313	0.070	8.305	2387	WS00940_A06
7	70.278	141	3	0.258	0.049	5.771	2387	WS00940_A06
7	91.487	146	3	0.227	0.038	4.455	2387	WS00940_A06
9	28.608	170	3	-0.274	0.052	5.435	2387	WS00940_A06
10	78.479	193	3	0.350	0.047	5.521	2387	WS00940_A06
11	27.405	204	1	0.219	0.060	6.400	2387	WS00940_A06
11	45.989	216	1	-0.276	0.062	6.669	2387	WS00940_A06
11	60.464	221	1	0.303	0.057	6.182	2387	WS00940_A06
12	6.242	234	2	-0.236	0.054	5.468	2387	WS00940_A06
2	93.504	32	4	0.258	0.051	6.500	2388	WS00834_L14

2	120.617	38	4	0.242	0.045	5.382	2388	WS00834_L14
2	127.147	40	4	0.236	0.033	4.334	2388	WS00834_L14
3	80.211	55	4	-0.150	0.035	4.091	2388	WS00834_L14
3	95.157	59	2	-0.210	0.051	3.937	2388	WS00834_L14
3	104.681	60	2	0.157	0.057	3.847	2388	WS00834_L14
3	112.868	61	2	-0.262	0.058	4.072	2388	WS00834_L14
4	55.925	76	1	0.181	0.038	4.227	2388	WS00834_L14
4	116.928	82	4	0.260	0.043	5.902	2388	WS00834_L14
4	124.132	83	1	-0.500	0.086	6.346	2388	WS00834_L14
5	3.484	90	2	0.288	0.139	6.131	2388	WS00834_L14
6	76.376	122	1	0.295	0.080	5.920	2388	WS00834_L14
8	78.389	157	1	0.248	0.066	7.890	2388	WS00834_L14
8	87.463	158	1	-0.197	0.045	5.415	2388	WS00834_L14
8	87.463	158	2	0.175	0.063	4.263	2388	WS00834_L14
9	49.013	173	1	-0.232	0.057	6.736	2388	WS00834_L14
9	59.963	177	1	-0.260	0.050	5.643	2388	WS00834_L14
9	59.963	177	4	-0.237	0.054	6.028	2388	WS00834_L14
10	35.204	189	1	0.258	0.044	5.300	2388	WS00834_L14
11	27.405	204	1	-0.269	0.037	4.513	2388	WS00834_L14
11	40.960	210	4	-0.346	0.063	6.659	2388	WS00834_L14
11	48.979	218	4	-0.324	0.041	3.970	2388	WS00834_L14
12	22.441	227	1	0.220	0.043	5.178	2388	WS00834_L14
3	9.953	42	3	0.210	0.070	5.354	2389	WS0094_F22
5	54.072	100	4	0.216	0.038	4.204	2389	WS0094_F22
7	73.169	142	2	0.560	0.080	8.198	2389	WS0094_F22
7	75.883	144	2	-0.568	0.090	9.394	2389	WS0094_F22
9	59.963	177	4	0.165	0.034	4.029	2389	WS0094_F22
11	60.464	221	2	-0.252	0.028	3.844	2389	WS0094_F22
1	2.788	2	4	-0.284	0.077	7.640	2390	WS0094_K06
4	173.929	88	2	-0.230	0.062	6.069	2390	WS0094_K06
5	56.008	101	1	0.204	0.043	3.853	2390	WS0094_K06
6	81.996	123	3	0.298	0.091	5.214	2390	WS0094_K06
7	105.477	147	2	-0.312	0.087	6.434	2390	WS0094_K06
11	27.405	204	1	0.274	0.065	5.947	2390	WS0094_K06
11	27.405	204	4	-0.258	0.047	4.975	2390	WS0094_K06
12	6.242	234	2	-0.194	0.033	4.104	2390	WS0094_K06
3	9.953	42	4	-0.322	0.073	5.929	2391	WS0018_B24
3	14.500	43	2	-0.300	0.050	5.357	2391	WS0018_B24
4	124.132	83	1	-0.519	0.159	5.559	2391	WS0018_B24
12	51.382	242	1	0.397	0.135	4.676	2391	WS0018_B24
3	9.953	42	4	0.299	0.076	6.434	2392	WS00940_L24
3	80.211	55	4	0.206	0.033	3.858	2392	WS00940_L24
4	173.929	88	2	-0.227	0.042	4.990	2392	WS00940_L24
7	74.560	143	1	0.275	0.089	6.156	2392	WS00940_L24
9	59.963	177	4	0.309	0.062	7.161	2392	WS00940_L24
10	78.479	193	3	0.277	0.062	4.468	2392	WS00940_L24
10	95.795	197	4	-0.200	0.037	4.230	2392	WS00940_L24
11	27.405	204	4	-0.260	0.051	5.955	2392	WS00940_L24
12	6.242	234	2	-0.262	0.047	6.509	2392	WS00940_L24
12	6.242	234	4	0.302	0.044	4.590	2392	WS00940_L24
14	0.000	246	2	-0.218	0.057	4.574	2392	WS00940_L24
14	22.402	248	4	-0.338	0.056	6.711	2392	WS00940_L24
3	148.745	68	1	-0.280	0.043	3.941	2393	WS0018_P12
4	142.917	85	1	0.276	0.047	4.090	2393	WS0018_P12

4	142.917	85	3	-0.264	0.064	6.462	2393	WS0018_P12
4	173.929	88	2	-0.252	0.062	6.060	2393	WS0018_P12
5	3.484	90	2	-0.369	0.102	7.109	2393	WS0018_P12
5	57.017	102	4	-0.223	0.047	4.305	2393	WS0018_P12
6	81.996	123	3	0.483	0.156	10.418	2393	WS0018_P12
6	89.401	125	1	0.277	0.049	4.385	2393	WS0018_P12
6	106.702	128	3	-0.188	0.047	3.929	2393	WS0018_P12
8	50.001	154	1	-0.235	0.042	4.612	2393	WS0018_P12
11	0.000	199	3	-0.190	0.050	4.426	2393	WS0018_P12
14	0.000	246	2	-0.176	0.045	4.510	2393	WS0018_P12
1	69.854	13	2	0.218	0.045	6.019	2394	WS00941_E21
1	71.592	14	2	0.214	0.044	5.819	2394	WS00941_E21
1	77.177	15	2	0.186	0.032	4.283	2394	WS00941_E21
3	137.891	64	3	0.209	0.078	4.933	2394	WS00941_E21
4	0.000	71	4	-0.159	0.044	5.483	2394	WS00941_E21
5	54.072	100	4	0.243	0.047	5.871	2394	WS00941_E21
6	87.891	124	2	-0.250	0.059	7.845	2394	WS00941_E21
6	89.401	125	2	-0.324	0.051	6.801	2394	WS00941_E21
7	62.787	139	4	0.152	0.026	3.921	2394	WS00941_E21
9	59.963	177	4	0.176	0.041	5.223	2394	WS00941_E21
10	9.580	187	2	-0.332	0.091	9.992	2394	WS00941_E21
11	27.405	204	1	0.218	0.075	4.169	2394	WS00941_E21
11	64.014	222	4	-0.276	0.070	7.909	2394	WS00941_E21
12	6.242	234	2	-0.234	0.047	9.041	2394	WS00941_E21
14	0.000	246	2	-0.230	0.047	6.330	2394	WS00941_E21
1	40.464	7	4	0.218	0.048	4.366	2395	WS00923_E03
1	43.578	8	2	0.274	0.054	5.688	2395	WS00923_E03
1	45.794	9	1	0.246	0.048	4.025	2395	WS00923_E03
1	77.177	15	2	-0.154	0.038	3.981	2395	WS00923_E03
2	63.261	30	3	-0.224	0.042	4.105	2395	WS00923_E03
2	75.550	31	2	-0.279	0.048	4.973	2395	WS00923_E03
2	101.076	35	4	-0.265	0.063	4.827	2395	WS00923_E03
3	137.891	64	1	-0.358	0.122	11.035	2395	WS00923_E03
3	141.443	65	3	-0.261	0.108	4.914	2395	WS00923_E03
3	144.647	67	1	-0.385	0.122	4.681	2395	WS00923_E03
10	70.246	190	4	-0.322	0.075	6.046	2395	WS00923_E03
11	64.014	222	4	0.322	0.058	4.067	2395	WS00923_E03
12	6.242	234	4	0.213	0.038	5.306	2395	WS00923_E03
1	29.800	5	1	0.188	0.055	4.077	2396	WS00923_H14
1	43.578	8	3	0.146	0.038	4.305	2396	WS00923_H14
1	45.794	9	3	-0.223	0.048	4.772	2396	WS00923_H14
4	116.928	82	2	-0.208	0.039	4.356	2396	WS00923_H14
9	28.608	170	2	-0.261	0.037	4.566	2396	WS00923_H14
9	28.608	170	3	0.322	0.065	6.269	2396	WS00923_H14
11	0.000	199	3	-0.328	0.053	5.182	2396	WS00923_H14
11	27.405	204	1	0.360	0.086	6.720	2396	WS00923_H14
3	14.500	43	2	-0.313	0.045	4.752	2397	WS00836_C24
3	20.512	46	2	0.285	0.076	7.747	2397	WS00836_C24
3	83.563	56	3	-0.209	0.041	4.736	2397	WS00836_C24
3	141.443	65	3	0.346	0.073	4.624	2397	WS00836_C24
5	3.484	90	2	0.365	0.102	5.043	2397	WS00836_C24
9	69.046	181	1	-0.409	0.093	5.916	2397	WS00836_C24
11	35.604	207	3	0.220	0.039	4.465	2397	WS00836_C24
14	22.402	248	3	0.178	0.044	4.974	2397	WS00836_C24

3	137.891	64	3	0.240	0.042	3.867	2398	WS00836_E12
9	36.227	165	3	-0.250	0.060	5.573	2398	WS00836_E12
9	59.963	177	4	0.235	0.043	4.105	2398	WS00836_E12
11	27.405	204	1	0.245	0.056	4.623	2398	WS00836_E12
12	6.242	234	2	-0.226	0.033	4.225	2398	WS00836_E12
3	69.071	52	1	-0.319	0.093	3.880	2399	WS00836_H16
4	142.917	85	1	0.200	0.063	4.427	2399	WS00836_H16
6	69.073	121	3	0.309	0.050	5.232	2399	WS00836_H16
9	17.951	169	2	0.295	0.054	6.721	2399	WS00836_H16
9	32.356	171	2	-0.268	0.074	8.575	2399	WS00836_H16
1	43.578	8	2	0.413	0.090	12.046	2400	WS00836_J15
1	95.572	17	2	-0.196	0.031	4.156	2400	WS00836_J15
1	95.572	17	3	0.208	0.069	4.834	2400	WS00836_J15
2	126.181	39	4	-0.321	0.039	4.242	2400	WS00836_J15
2	127.147	40	4	0.235	0.045	5.248	2400	WS00836_J15
6	69.073	121	4	-0.263	0.057	6.798	2400	WS00836_J15
7	73.169	142	2	-0.639	0.124	12.198	2400	WS00836_J15
7	74.560	143	2	0.131	0.029	4.304	2400	WS00836_J15
7	75.883	144	2	0.514	0.087	8.239	2400	WS00836_J15
8	0.000	148	4	-0.231	0.046	5.356	2400	WS00836_J15
10	78.479	193	4	-0.189	0.042	5.429	2400	WS00836_J15
10	95.795	197	4	0.205	0.035	4.088	2400	WS00836_J15
14	43.392	250	2	0.167	0.039	4.847	2400	WS00836_J15
1	62.128	11	1	-0.461	0.077	4.107	2401	WS00836_P19
2	102.814	36	2	-0.314	0.076	7.129	2401	WS00836_P19
2	120.617	38	4	-0.320	0.056	5.673	2401	WS00836_P19
2	127.147	40	4	-0.240	0.056	6.528	2401	WS00836_P19
3	148.745	68	1	-0.273	0.046	4.137	2401	WS00836_P19
4	92.039	79	2	-0.228	0.043	4.281	2401	WS00836_P19
6	25.914	114	1	-0.206	0.047	4.295	2401	WS00836_P19
6	95.249	126	1	-0.305	0.103	3.910	2401	WS00836_P19
7	67.154	140	4	-0.468	0.133	7.147	2401	WS00836_P19
7	75.883	144	4	-0.335	0.080	6.535	2401	WS00836_P19
8	87.463	158	2	0.253	0.059	5.662	2401	WS00836_P19
9	17.951	169	1	-0.248	0.042	3.857	2401	WS00836_P19
9	17.951	169	2	0.214	0.037	4.393	2401	WS00836_P19
10	80.088	194	1	0.235	0.061	5.566	2401	WS00836_P19
11	27.405	204	1	0.230	0.058	5.397	2401	WS00836_P19
11	35.604	207	3	-0.212	0.059	4.221	2401	WS00836_P19
11	37.143	209	3	0.228	0.068	4.886	2401	WS00836_P19
11	43.491	214	4	0.237	0.053	6.175	2401	WS00836_P19
11	44.884	215	4	-0.240	0.043	5.045	2401	WS00836_P19
11	48.979	218	4	0.234	0.039	4.409	2401	WS00836_P19
12	14.406	230	4	0.315	0.048	5.481	2401	WS00836_P19
12	0.000	233	1	0.226	0.047	4.300	2401	WS00836_P19
1	22.419	4	4	-0.235	0.097	4.348	2402	WS00837_A15
2	75.550	31	1	0.189	0.060	4.264	2402	WS00837_A15
2	102.814	36	2	-0.260	0.052	5.518	2402	WS00837_A15
3	95.157	59	2	-0.210	0.048	5.573	2402	WS00837_A15
3	104.681	60	2	0.203	0.037	4.303	2402	WS00837_A15
3	112.868	61	2	-0.219	0.033	3.893	2402	WS00837_A15
3	114.747	62	2	-0.171	0.042	4.813	2402	WS00837_A15
5	3.484	90	2	0.310	0.113	7.612	2402	WS00837_A15
6	54.066	119	4	-0.167	0.043	4.633	2402	WS00837_A15

8	87.463	158	2	0.244	0.052	5.450	2402	WS00837_A15
9	3.127	168	1	0.389	0.092	4.050	2402	WS00837_A15
9	49.013	173	2	0.261	0.057	6.317	2402	WS00837_A15
11	35.604	207	3	-0.249	0.051	4.134	2402	WS00837_A15
12	14.406	230	4	0.236	0.053	5.142	2402	WS00837_A15
3	9.953	42	3	0.266	0.062	5.853	2403	WS00837_F01
3	15.147	44	2	0.315	0.082	4.856	2403	WS00837_F01
8	87.463	158	2	-0.219	0.050	3.915	2403	WS00837_F01
9	59.963	177	4	0.264	0.049	4.397	2403	WS00837_F01
10	70.246	190	2	-0.221	0.039	4.027	2403	WS00837_F01
1	45.794	9	3	-0.233	0.077	5.323	2404	WS00837_G15
1	69.854	13	2	-0.185	0.043	4.513	2404	WS00837_G15
1	71.592	14	2	-0.170	0.037	3.880	2404	WS00837_G15
3	9.953	42	4	0.169	0.039	4.013	2404	WS00837_G15
4	97.576	80	2	0.236	0.054	5.538	2404	WS00837_G15
6	56.188	120	2	0.328	0.057	6.419	2404	WS00837_G15
6	106.702	128	2	-0.545	0.155	8.497	2404	WS00837_G15
6	106.702	128	3	0.321	0.051	4.401	2404	WS00837_G15
7	75.883	144	2	-0.459	0.103	6.239	2404	WS00837_G15
10	80.088	194	2	0.204	0.041	5.330	2404	WS00837_G15
12	22.441	227	1	0.176	0.049	4.107	2404	WS00837_G15
2	127.147	40	4	-0.245	0.060	5.506	2405	WS0107_B02
3	15.147	44	2	0.348	0.119	7.210	2405	WS0107_B02
6	81.996	123	3	0.360	0.095	5.413	2405	WS0107_B02
9	59.963	177	4	0.312	0.086	8.253	2405	WS0107_B02
11	27.405	204	1	0.181	0.046	4.278	2405	WS0107_B02
14	22.402	248	4	0.263	0.054	4.877	2405	WS0107_B02
1	29.800	5	1	-0.285	0.074	5.431	2406	WS0107_B17
2	126.181	39	2	0.248	0.043	4.839	2406	WS0107_B17
3	9.953	42	4	-0.143	0.039	3.881	2406	WS0107_B17
4	75.958	77	3	0.367	0.142	7.137	2406	WS0107_B17
10	77.676	192	1	-0.243	0.071	5.240	2406	WS0107_B17
10	80.088	194	1	-0.279	0.067	4.895	2406	WS0107_B17
11	0.000	199	2	0.180	0.039	4.384	2406	WS0107_B17
11	17.088	203	1	0.241	0.053	3.975	2406	WS0107_B17
11	27.405	204	1	-0.296	0.087	6.648	2406	WS0107_B17
11	64.014	222	4	0.210	0.040	4.008	2406	WS0107_B17
13	3.386	245	1	0.253	0.064	4.689	2406	WS0107_B17
2	93.504	32	4	-0.300	0.053	5.069	2407	WS0107_D13
6	95.249	126	1	-0.310	0.084	4.090	2407	WS0107_D13
8	78.389	157	1	-0.211	0.054	5.419	2407	WS0107_D13
8	87.463	158	1	0.241	0.070	6.997	2407	WS0107_D13
1	2.788	2	4	-0.217	0.044	3.971	2408	WS0107_E05
3	9.953	42	3	0.239	0.053	4.899	2408	WS0107_E05
3	137.891	64	3	0.252	0.042	3.930	2408	WS0107_E05
4	173.929	88	2	-0.271	0.077	7.502	2408	WS0107_E05
6	81.996	123	3	0.363	0.098	6.396	2408	WS0107_E05
8	50.001	154	1	-0.212	0.043	4.207	2408	WS0107_E05
8	95.937	159	3	-0.224	0.043	3.999	2408	WS0107_E05
9	59.963	177	4	0.308	0.065	6.076	2408	WS0107_E05
12	6.242	234	2	-0.224	0.039	4.918	2408	WS0107_E05
4	0.000	71	4	0.199	0.044	4.119	2409	WS0107_G21
4	173.929	88	2	-0.283	0.071	7.284	2409	WS0107_G21
6	81.996	123	3	0.361	0.083	5.141	2409	WS0107_G21

8	95.937	159	3	-0.224	0.042	3.974	2409	WS0107_G21
9	0.000	163	1	0.465	0.079	4.867	2409	WS0107_G21
9	17.951	169	2	-0.193	0.041	3.860	2409	WS0107_G21
9	59.963	177	4	0.278	0.051	4.870	2409	WS0107_G21
12	6.242	234	2	-0.256	0.049	5.917	2409	WS0107_G21
1	40.464	7	4	0.176	0.038	4.062	2410	WS0107_H09
3	14.500	43	1	0.259	0.061	4.645	2410	WS0107_H09
5	42.979	97	1	0.234	0.059	4.512	2410	WS0107_H09
9	36.227	165	4	0.265	0.071	7.300	2410	WS0107_H09
12	6.242	234	4	0.374	0.063	7.429	2410	WS0107_H09
4	173.929	88	2	-0.208	0.048	5.811	2411	WS0107_J10
8	50.001	154	1	-0.211	0.067	4.841	2411	WS0107_J10
10	78.479	193	3	0.290	0.053	4.212	2411	WS0107_J10
11	27.405	204	4	-0.354	0.082	5.417	2411	WS0107_J10
1	29.800	5	2	-0.235	0.070	5.907	2412	WS0107_J22
4	6.032	72	4	0.200	0.059	6.049	2412	WS0107_J22
4	82.462	78	2	-0.169	0.050	4.916	2412	WS0107_J22
4	116.928	82	4	0.199	0.048	4.885	2412	WS0107_J22
4	142.917	85	1	0.317	0.103	10.985	2412	WS0107_J22
5	54.072	100	4	0.151	0.042	4.278	2412	WS0107_J22
5	57.017	102	4	-0.159	0.045	4.596	2412	WS0107_J22
9	28.608	170	3	-0.280	0.048	4.293	2412	WS0107_J22
14	46.637	252	4	-0.399	0.055	5.396	2412	WS0107_J22
3	9.953	42	3	0.266	0.061	5.138	2413	WS01016_E04
3	116.958	63	4	-0.171	0.046	4.842	2413	WS01016_E04
6	56.188	120	2	-0.250	0.043	5.316	2413	WS01016_E04
6	81.996	123	3	0.421	0.142	7.427	2413	WS01016_E04
6	87.891	124	2	-0.196	0.035	4.124	2413	WS01016_E04
8	87.463	158	2	-0.217	0.041	4.349	2413	WS01016_E04
11	64.014	222	4	-0.221	0.045	4.810	2413	WS01016_E04
12	6.242	234	2	-0.224	0.040	5.537	2413	WS01016_E04
14	0.000	246	2	-0.185	0.036	4.052	2413	WS01016_E04
3	69.071	52	1	-0.306	0.088	4.631	2414	WS01016_J12
4	82.462	78	1	-0.195	0.054	4.173	2414	WS01016_J12
9	49.013	173	2	0.267	0.063	6.426	2414	WS01016_J12
9	81.073	183	1	-0.280	0.057	4.496	2414	WS01016_J12
10	86.151	195	3	0.247	0.046	4.665	2414	WS01016_J12
11	17.088	203	2	-0.240	0.056	4.353	2414	WS01016_J12
2	63.261	30	3	0.235	0.033	3.893	2415	WS0084_P12
3	9.953	42	3	0.183	0.054	6.279	2415	WS0084_P12
3	14.500	43	1	0.307	0.047	4.118	2415	WS0084_P12
3	17.158	45	1	-0.167	0.051	4.396	2415	WS0084_P12
3	141.443	65	3	0.343	0.067	4.093	2415	WS0084_P12
3	148.745	68	4	-0.301	0.069	5.374	2415	WS0084_P12
4	43.146	74	3	-0.228	0.033	3.928	2415	WS0084_P12
4	142.917	85	3	-0.251	0.053	5.960	2415	WS0084_P12
5	31.191	95	1	-0.256	0.048	4.234	2415	WS0084_P12
6	106.702	128	2	0.482	0.115	6.981	2415	WS0084_P12
7	15.555	130	3	0.261	0.035	4.168	2415	WS0084_P12
8	95.937	159	3	-0.181	0.051	5.901	2415	WS0084_P12
9	3.127	168	1	0.361	0.194	4.498	2415	WS0084_P12
10	86.151	195	3	0.155	0.037	4.320	2415	WS0084_P12
10	93.347	196	1	0.423	0.072	4.294	2415	WS0084_P12
11	27.405	204	1	0.274	0.045	4.181	2415	WS0084_P12

11	27.405	204	4	-0.353	0.086	6.377	2415	WS0084_P12
12	14.406	230	3	0.324	0.110	12.888	2415	WS0084_P12
12	0.000	233	1	0.171	0.056	4.808	2415	WS0084_P12
13	3.386	245	1	-0.152	0.051	4.364	2415	WS0084_P12
1	69.854	13	2	0.172	0.036	3.963	2416	WS0023_B24
3	9.953	42	3	0.292	0.067	6.755	2416	WS0023_B24
4	75.958	77	3	-0.370	0.107	7.908	2416	WS0023_B24
5	86.669	106	4	0.314	0.083	4.216	2416	WS0023_B24
7	75.883	144	4	0.290	0.064	4.787	2416	WS0023_B24
9	2.011	167	4	-0.211	0.044	3.932	2416	WS0023_B24
10	78.479	193	3	-0.307	0.053	5.212	2416	WS0023_B24
12	51.382	242	1	0.289	0.084	4.283	2416	WS0023_B24
14	0.000	246	1	0.210	0.051	3.917	2416	WS0023_B24
1	2.788	2	4	-0.224	0.079	4.970	2417	WS0085_F04
3	15.147	44	2	0.249	0.080	4.034	2417	WS0085_F04
3	20.512	46	2	0.235	0.055	6.441	2417	WS0085_F04
3	95.157	59	2	-0.220	0.039	5.413	2417	WS0085_F04
3	114.747	62	2	-0.191	0.037	4.347	2417	WS0085_F04
3	137.891	64	3	0.247	0.039	4.135	2417	WS0085_F04
4	55.925	76	1	-0.317	0.093	6.058	2417	WS0085_F04
9	36.227	165	3	-0.200	0.036	3.955	2417	WS0085_F04
9	59.963	177	4	0.253	0.063	5.004	2417	WS0085_F04
11	27.405	204	1	0.311	0.095	6.828	2417	WS0085_F04
11	48.979	218	2	0.213	0.040	4.724	2417	WS0085_F04
1	95.572	17	2	-0.273	0.042	4.310	2418	WS0023_I21
2	93.504	32	2	0.315	0.070	7.025	2418	WS0023_I21
2	126.181	39	2	-0.242	0.052	5.173	2418	WS0023_I21
3	14.500	43	1	-0.283	0.066	5.804	2418	WS0023_I21
5	0.000	89	3	-0.171	0.038	4.834	2418	WS0023_I21
5	7.672	92	1	-0.330	0.086	8.428	2418	WS0023_I21
5	52.820	99	3	0.323	0.078	7.935	2418	WS0023_I21
5	56.008	101	2	-0.249	0.054	5.149	2418	WS0023_I21
5	57.017	102	2	0.257	0.068	6.978	2418	WS0023_I21
9	2.011	167	4	-0.138	0.049	4.124	2418	WS0023_I21
9	55.358	175	1	0.344	0.058	5.202	2418	WS0023_I21
9	55.358	175	3	-0.549	0.139	8.274	2418	WS0023_I21
12	28.493	228	2	0.233	0.091	4.478	2418	WS0023_I21
14	9.923	247	1	0.250	0.048	4.308	2418	WS0023_I21
1	95.572	17	2	0.261	0.060	5.198	2419	WS0105_O04
2	63.261	30	3	0.252	0.047	4.622	2419	WS0105_O04
3	9.953	42	3	0.331	0.096	9.223	2419	WS0105_O04
3	15.147	44	2	0.307	0.131	5.835	2419	WS0105_O04
4	82.462	78	1	-0.295	0.053	5.537	2419	WS0105_O04
4	124.132	83	2	0.225	0.107	4.015	2419	WS0105_O04
6	106.702	128	2	0.409	0.097	4.250	2419	WS0105_O04
7	15.555	130	3	0.284	0.064	5.585	2419	WS0105_O04
9	59.963	177	4	0.240	0.061	5.285	2419	WS0105_O04
11	17.088	203	1	-0.221	0.053	5.445	2419	WS0105_O04
11	27.405	204	1	0.279	0.053	5.535	2419	WS0105_O04
11	29.069	205	1	0.289	0.059	6.072	2419	WS0105_O04
11	45.989	216	1	-0.259	0.055	5.602	2419	WS0105_O04
1	2.788	2	4	-0.162	0.043	3.858	2420	WS0106_D19
1	95.572	17	2	0.251	0.040	4.100	2420	WS0106_D19
2	127.147	40	4	-0.189	0.041	4.049	2420	WS0106_D19

3	9.953	42	3	0.244	0.069	6.178	2420	WS0106_D19
6	81.996	123	3	0.271	0.071	4.661	2420	WS0106_D19
8	95.937	159	3	-0.245	0.067	5.985	2420	WS0106_D19
9	59.963	177	4	0.256	0.063	5.441	2420	WS0106_D19
11	0.000	199	2	0.317	0.070	7.259	2420	WS0106_D19
11	27.405	204	1	0.269	0.067	5.734	2420	WS0106_D19
12	28.493	228	2	-0.264	0.067	5.332	2420	WS0106_D19
1	43.578	8	2	-0.263	0.050	4.329	2421	WS00923_L05
2	127.147	40	4	-0.184	0.038	3.875	2421	WS00923_L05
4	0.000	71	4	0.310	0.093	9.542	2421	WS00923_L05
4	55.925	76	1	-0.265	0.063	5.431	2421	WS00923_L05
7	74.560	143	1	0.282	0.059	6.033	2421	WS00923_L05
8	50.001	154	1	-0.289	0.067	7.049	2421	WS00923_L05
10	9.580	187	3	0.407	0.142	7.475	2421	WS00923_L05
11	27.405	204	1	0.247	0.054	5.599	2421	WS00923_L05
11	27.405	204	4	-0.235	0.043	4.340	2421	WS00923_L05
11	29.069	205	1	0.208	0.049	4.913	2421	WS00923_L05
2	48.839	29	1	0.454	0.135	14.710	2422	WS00923_O07
2	75.550	31	1	-0.247	0.037	3.879	2422	WS00923_O07
3	14.500	43	1	-0.244	0.042	4.458	2422	WS00923_O07
3	17.158	45	1	0.298	0.062	6.556	2422	WS00923_O07
4	92.039	79	4	0.296	0.037	4.317	2422	WS00923_O07
4	124.132	83	1	-0.642	0.173	8.397	2422	WS00923_O07
5	57.017	102	4	0.348	0.087	8.370	2422	WS00923_O07
6	81.996	123	4	0.284	0.093	4.490	2422	WS00923_O07
6	89.401	125	2	0.248	0.054	4.606	2422	WS00923_O07
7	70.278	141	3	0.274	0.061	5.950	2422	WS00923_O07
8	78.389	157	1	0.176	0.037	3.898	2422	WS00923_O07
9	0.000	163	1	0.466	0.088	5.080	2422	WS00923_O07
9	28.608	170	3	-0.242	0.047	4.624	2422	WS00923_O07
10	80.088	194	1	0.226	0.038	4.008	2422	WS00923_O07
11	29.069	205	1	-0.244	0.046	4.854	2422	WS00923_O07
11	46.026	217	1	0.251	0.043	4.597	2422	WS00923_O07
6	81.996	123	3	0.318	0.106	5.856	2423	WS0081_M20
9	59.963	177	4	0.286	0.060	6.380	2423	WS0081_M20
1	82.301	16	4	0.189	0.060	4.378	2424	WS0081_N18
2	63.261	30	3	0.242	0.042	4.827	2424	WS0081_N18
3	14.500	43	2	-0.312	0.064	7.213	2424	WS0081_N18
3	137.891	64	3	0.248	0.037	4.223	2424	WS0081_N18
4	43.146	74	3	-0.219	0.036	4.095	2424	WS0081_N18
4	55.925	76	1	0.189	0.059	4.381	2424	WS0081_N18
6	81.996	123	3	0.269	0.106	6.170	2424	WS0081_N18
7	105.477	147	2	-0.231	0.084	4.819	2424	WS0081_N18
13	1.815	244	4	-0.266	0.068	4.441	2424	WS0081_N18
3	137.891	64	3	0.317	0.076	7.247	2425	WS0082_A03
6	81.996	123	3	0.318	0.109	6.299	2425	WS0082_A03
9	28.608	170	3	0.249	0.052	4.801	2425	WS0082_A03
10	80.088	194	1	-0.247	0.055	3.932	2425	WS0082_A03
10	93.347	196	1	0.453	0.085	4.941	2425	WS0082_A03
1	43.578	8	2	0.297	0.053	5.901	2426	WS0082_A10
2	126.181	39	4	-0.484	0.099	7.271	2426	WS0082_A10
3	15.147	44	2	-0.385	0.072	4.882	2426	WS0082_A10
3	144.647	67	1	0.295	0.151	5.185	2426	WS0082_A10
5	16.219	93	1	-0.226	0.061	5.437	2426	WS0082_A10

7	73.169	142	2	-0.406	0.054	4.025	2426	WS0082_A10
7	75.883	144	2	0.413	0.059	4.358	2426	WS0082_A10
8	0.000	148	4	-0.159	0.045	4.862	2426	WS0082_A10
8	11.974	150	1	0.122	0.045	3.873	2426	WS0082_A10
8	87.463	158	2	0.126	0.043	5.750	2426	WS0082_A10
9	3.127	168	1	0.399	0.126	4.036	2426	WS0082_A10
9	28.608	170	2	-0.249	0.035	4.077	2426	WS0082_A10
9	28.608	170	3	0.349	0.073	5.980	2426	WS0082_A10
11	9.237	202	4	-0.258	0.035	3.964	2426	WS0082_A10
11	57.661	220	4	0.121	0.043	4.643	2426	WS0082_A10
11	64.014	222	4	-0.244	0.049	6.234	2426	WS0082_A10
14	22.402	248	3	-0.331	0.060	4.953	2426	WS0082_A10
14	43.392	250	1	0.373	0.081	4.363	2426	WS0082_A10
2	102.814	36	1	-0.229	0.067	5.241	2427	WS0082_C11
3	15.147	44	2	0.348	0.077	5.240	2427	WS0082_C11
3	20.512	46	2	0.209	0.046	5.187	2427	WS0082_C11
3	144.593	66	4	0.201	0.042	4.855	2427	WS0082_C11
6	69.073	121	3	0.239	0.061	5.275	2427	WS0082_C11
7	59.789	137	3	-0.394	0.188	16.369	2427	WS0082_C11
7	70.278	141	3	0.346	0.154	13.360	2427	WS0082_C11
7	91.487	146	3	0.256	0.056	4.939	2427	WS0082_C11
9	59.963	177	4	0.304	0.059	5.852	2427	WS0082_C11
12	14.406	230	3	-0.190	0.047	4.114	2427	WS0082_C11
3	9.953	42	3	0.189	0.036	4.022	2428	WS0082_D12
3	95.157	59	2	-0.253	0.059	6.964	2428	WS0082_D12
5	54.072	100	4	0.216	0.073	5.501	2428	WS0082_D12
6	81.996	123	3	0.336	0.103	5.880	2428	WS0082_D12
7	17.610	131	3	0.265	0.075	4.142	2428	WS0082_D12
11	0.000	199	3	-0.254	0.054	6.094	2428	WS0082_D12
14	22.402	248	3	0.284	0.050	5.607	2428	WS0082_D12
4	173.929	88	2	-0.220	0.049	5.244	2429	WS0082_F15
6	81.996	123	3	0.375	0.107	5.733	2429	WS0082_F15
8	50.001	154	1	-0.220	0.050	4.355	2429	WS0082_F15
9	59.963	177	4	0.239	0.043	4.140	2429	WS0082_F15
10	78.479	193	3	0.309	0.049	4.451	2429	WS0082_F15
11	27.405	204	4	-0.267	0.059	4.354	2429	WS0082_F15
2	120.617	38	4	-0.259	0.041	4.777	2430	WS0082_G06
2	126.181	39	4	0.466	0.096	5.450	2430	WS0082_G06
3	116.958	63	4	-0.220	0.049	5.698	2430	WS0082_G06
6	56.188	120	2	-0.269	0.050	4.643	2430	WS0082_G06
6	81.996	123	3	0.354	0.137	6.016	2430	WS0082_G06
6	81.996	123	4	-0.393	0.118	6.121	2430	WS0082_G06
7	62.787	139	4	0.272	0.046	4.306	2430	WS0082_G06
7	74.560	143	1	0.317	0.086	8.176	2430	WS0082_G06
9	2.011	167	4	-0.248	0.046	5.363	2430	WS0082_G06
11	57.661	220	4	0.239	0.053	6.157	2430	WS0082_G06
11	64.014	222	4	-0.431	0.096	9.727	2430	WS0082_G06
12	6.242	234	2	-0.192	0.047	4.309	2430	WS0082_G06
13	0.000	243	4	0.316	0.077	3.973	2430	WS0082_G06
14	0.000	246	2	-0.215	0.069	3.986	2430	WS0082_G06
1	131.686	23	1	-0.533	0.113	4.911	2431	WS0023_O20
3	14.500	43	1	-0.276	0.068	5.676	2431	WS0023_O20
3	17.158	45	1	0.180	0.056	4.619	2431	WS0023_O20
4	82.462	78	1	-0.285	0.055	4.583	2431	WS0023_O20

4	97.576	80	2	-0.333	0.086	9.910	2431	WS0023_O20
4	124.132	83	2	-0.316	0.056	3.942	2431	WS0023_O20
5	16.219	93	2	0.244	0.055	5.312	2431	WS0023_O20
8	0.000	148	4	0.236	0.039	3.929	2431	WS0023_O20
11	17.088	203	1	-0.212	0.073	6.024	2431	WS0023_O20
11	29.069	205	1	0.269	0.061	5.106	2431	WS0023_O20
11	45.989	216	1	-0.261	0.054	4.499	2431	WS0023_O20
1	29.800	5	1	0.184	0.061	4.300	2432	WS0023_P13
1	43.578	8	3	0.164	0.048	5.066	2432	WS0023_P13
1	45.794	9	3	-0.178	0.035	4.006	2432	WS0023_P13
3	137.891	64	3	0.277	0.042	4.354	2432	WS0023_P13
4	0.000	71	4	0.213	0.052	4.307	2432	WS0023_P13
4	55.925	76	1	0.177	0.062	4.847	2432	WS0023_P13
4	116.928	82	2	-0.206	0.037	4.331	2432	WS0023_P13
4	124.132	83	2	-0.239	0.081	4.172	2432	WS0023_P13
10	78.479	193	4	-0.282	0.087	7.086	2432	WS0023_P13
1	40.464	7	4	-0.186	0.053	4.659	2433	WS0024_D23
1	95.572	17	2	-0.279	0.065	6.016	2433	WS0024_D23
1	95.572	17	3	0.180	0.040	4.102	2433	WS0024_D23
3	14.500	43	1	-0.237	0.058	5.589	2433	WS0024_D23
4	92.039	79	4	-0.468	0.108	8.001	2433	WS0024_D23
4	103.719	81	4	-0.159	0.050	4.379	2433	WS0024_D23
5	7.672	92	1	-0.246	0.057	5.686	2433	WS0024_D23
5	57.017	102	2	0.195	0.046	4.284	2433	WS0024_D23
6	81.996	123	4	-0.203	0.097	4.506	2433	WS0024_D23
6	87.891	124	2	0.190	0.043	3.955	2433	WS0024_D23
6	89.401	125	2	0.192	0.043	4.522	2433	WS0024_D23
11	46.026	217	1	-0.239	0.044	4.318	2433	WS0024_D23
14	9.923	247	1	0.278	0.062	5.986	2433	WS0024_D23
1	107.333	19	4	0.198	0.059	5.109	2434	WS0024_F03
3	137.891	64	3	0.218	0.040	4.212	2434	WS0024_F03
5	0.000	89	3	-0.260	0.068	7.655	2434	WS0024_F03
5	7.672	92	1	-0.235	0.052	5.478	2434	WS0024_F03
11	0.000	199	3	-0.332	0.057	5.069	2434	WS0024_F03
14	22.402	248	4	0.339	0.058	4.815	2434	WS0024_F03
14	46.637	252	4	0.316	0.050	4.309	2434	WS0024_F03
1	141.021	25	2	0.183	0.041	4.959	2435	WS0024_I21
4	6.032	72	4	0.128	0.042	3.941	2435	WS0024_I21
4	124.132	83	1	-0.451	0.116	4.463	2435	WS0024_I21
4	142.917	85	1	0.223	0.060	3.967	2435	WS0024_I21
5	16.219	93	2	0.257	0.038	4.611	2435	WS0024_I21
8	50.001	154	2	-0.259	0.051	6.371	2435	WS0024_I21
11	43.491	214	3	0.282	0.058	5.733	2435	WS0024_I21
12	51.382	242	1	0.397	0.113	4.127	2435	WS0024_I21
2	48.839	29	1	-0.403	0.123	8.435	2436	WS0024_J16
4	173.929	88	2	0.252	0.055	6.501	2436	WS0024_J16
5	0.000	89	1	-0.252	0.091	6.945	2436	WS0024_J16
7	17.610	131	3	0.376	0.134	10.743	2436	WS0024_J16
7	54.200	136	2	0.201	0.055	4.368	2436	WS0024_J16
9	36.227	165	3	0.176	0.039	4.663	2436	WS0024_J16
9	32.356	171	2	-0.196	0.033	3.910	2436	WS0024_J16
10	78.479	193	3	-0.222	0.046	5.335	2436	WS0024_J16
11	46.026	217	1	-0.212	0.064	4.429	2436	WS0024_J16
1	29.800	5	1	0.257	0.059	5.186	2437	WS0024_M18

1	40.464	7	3	0.225	0.059	5.666	2437	WS0024_M18
1	45.794	9	3	-0.264	0.073	7.186	2437	WS0024_M18
6	25.914	114	1	-0.373	0.124	10.956	2437	WS0024_M18
6	45.897	117	1	0.198	0.047	4.166	2437	WS0024_M18
10	78.479	193	4	-0.294	0.069	4.597	2437	WS0024_M18
10	95.795	197	4	0.260	0.072	6.647	2437	WS0024_M18
1	127.805	21	4	-0.228	0.065	5.387	2438	WS0024_O01
3	14.500	43	2	-0.236	0.036	4.378	2438	WS0024_O01
3	83.563	56	3	-0.204	0.038	4.183	2438	WS0024_O01
6	81.996	123	4	0.369	0.152	4.612	2438	WS0024_O01
9	17.951	169	2	-0.277	0.054	5.431	2438	WS0024_O01
10	9.580	187	3	0.313	0.173	6.977	2438	WS0024_O01
10	86.151	195	3	0.193	0.038	4.117	2438	WS0024_O01
11	0.000	199	2	0.266	0.055	6.579	2438	WS0024_O01
12	14.406	230	4	0.329	0.076	4.452	2438	WS0024_O01
3	20.512	46	2	-0.231	0.044	5.724	2439	WS0044_K07
4	43.146	74	3	0.272	0.065	7.188	2439	WS0044_K07
4	75.958	77	3	-0.355	0.120	8.924	2439	WS0044_K07
6	49.100	118	4	-0.491	0.242	5.483	2439	WS0044_K07
3	9.953	42	3	0.152	0.054	6.265	2440	WS0044_L11
5	3.484	90	2	-0.309	0.080	4.070	2440	WS0044_L11
10	35.204	189	2	-0.135	0.030	3.946	2440	WS0044_L11
1	69.854	13	2	0.212	0.031	3.946	2441	WS0044_O13
1	77.177	15	2	0.215	0.032	4.069	2441	WS0044_O13
1	95.572	17	2	0.228	0.032	4.019	2441	WS0044_O13
3	9.953	42	3	0.187	0.064	6.133	2441	WS0044_O13
4	116.928	82	2	0.276	0.035	4.574	2441	WS0044_O13
5	3.484	90	2	-0.322	0.096	6.183	2441	WS0044_O13
5	16.219	93	2	0.289	0.053	5.349	2441	WS0044_O13
9	81.073	183	1	0.248	0.076	4.796	2441	WS0044_O13
3	137.891	64	3	0.315	0.058	5.580	2442	WS0044_P14
6	81.996	123	4	0.421	0.129	4.970	2442	WS0044_P14
8	11.974	150	1	0.228	0.055	3.973	2442	WS0044_P14
8	78.389	157	1	-0.249	0.077	5.553	2442	WS0044_P14
9	0.000	163	1	-0.423	0.113	3.928	2442	WS0044_P14
9	69.046	181	2	0.319	0.080	5.624	2442	WS0044_P14
10	93.347	196	2	0.480	0.070	5.037	2442	WS0044_P14
11	17.088	203	1	-0.219	0.056	4.061	2442	WS0044_P14
11	45.989	216	1	-0.243	0.073	5.266	2442	WS0044_P14
14	46.637	252	4	0.323	0.060	4.317	2442	WS0044_P14
1	62.128	11	2	0.379	0.115	7.456	2443	WS0036_E09
3	95.157	59	2	-0.201	0.063	6.317	2443	WS0036_E09
3	137.891	64	3	-0.234	0.042	5.102	2443	WS0036_E09
4	82.462	78	1	-0.267	0.077	6.246	2443	WS0036_E09
5	7.672	92	1	-0.277	0.059	4.592	2443	WS0036_E09
6	25.742	113	3	-0.174	0.037	4.362	2443	WS0036_E09
6	26.350	115	2	0.169	0.045	4.807	2443	WS0036_E09
7	17.610	131	3	0.366	0.110	7.935	2443	WS0036_E09
7	54.200	136	2	0.251	0.056	3.995	2443	WS0036_E09
7	62.787	139	2	0.235	0.044	5.030	2443	WS0036_E09
7	75.883	144	4	-0.346	0.089	5.969	2443	WS0036_E09
9	49.013	173	1	-0.262	0.043	4.265	2443	WS0036_E09
10	70.246	190	4	0.251	0.075	5.062	2443	WS0036_E09
12	6.242	234	2	0.238	0.036	4.474	2443	WS0036_E09

2	93.504	32	4	0.270	0.076	7.150	2444	WS0036_H02
7	74.560	143	1	-0.184	0.049	3.884	2444	WS0036_H02
9	28.608	170	2	-0.374	0.085	9.360	2444	WS0036_H02
9	32.356	171	2	-0.367	0.125	13.602	2444	WS0036_H02
9	49.013	173	2	0.263	0.076	7.942	2444	WS0036_H02
10	70.246	190	2	-0.196	0.043	4.582	2444	WS0036_H02
11	43.491	214	3	-0.226	0.044	4.103	2444	WS0036_H02
13	3.386	245	1	0.241	0.065	5.085	2444	WS0036_H02
3	80.211	55	4	0.279	0.081	6.413	2445	WS0036_L14
4	124.132	83	1	0.440	0.140	3.871	2445	WS0036_L14
4	142.917	85	1	-0.265	0.076	5.704	2445	WS0036_L14
6	26.350	115	2	0.272	0.071	8.096	2445	WS0036_L14
7	17.610	131	3	0.220	0.070	4.395	2445	WS0036_L14
9	0.000	166	1	-0.203	0.080	5.798	2445	WS0036_L14
12	14.406	230	3	-0.292	0.050	5.467	2445	WS0036_L14
12	32.473	238	1	0.227	0.059	4.324	2445	WS0036_L14
1	82.301	16	4	0.299	0.057	5.064	2446	WS0036_M18
2	48.839	29	1	-0.231	0.057	5.359	2446	WS0036_M18
2	95.880	34	3	0.342	0.060	4.872	2446	WS0036_M18
2	101.076	35	3	-0.330	0.057	6.140	2446	WS0036_M18
5	0.000	89	1	-0.367	0.071	7.040	2446	WS0036_M18
5	16.219	93	1	-0.209	0.057	5.424	2446	WS0036_M18
5	84.347	105	1	0.233	0.044	4.179	2446	WS0036_M18
6	95.249	126	1	-0.307	0.065	4.672	2446	WS0036_M18
6	106.702	128	2	-0.414	0.125	4.485	2446	WS0036_M18
9	0.000	163	1	-0.522	0.084	6.009	2446	WS0036_M18
11	64.014	222	4	-0.196	0.049	3.965	2446	WS0036_M18
12	22.629	237	1	0.199	0.045	4.301	2446	WS0036_M18
2	63.261	30	3	0.234	0.061	3.979	2447	WS0058_I08
2	126.181	39	4	-0.480	0.077	7.797	2447	WS0058_I08
5	57.017	102	2	-0.183	0.032	4.796	2447	WS0058_I08
7	17.610	131	3	-0.318	0.122	5.722	2447	WS0058_I08
11	27.405	204	1	0.247	0.098	4.243	2447	WS0058_I08
11	27.405	204	4	-0.312	0.038	4.792	2447	WS0058_I08
1	2.788	2	4	-0.170	0.037	5.023	2448	WS01014_A24
1	82.301	16	4	0.268	0.048	4.329	2448	WS01014_A24
3	69.071	52	1	-0.426	0.088	5.717	2448	WS01014_A24
4	55.925	76	1	-0.195	0.061	4.953	2448	WS01014_A24
9	17.951	169	2	-0.203	0.043	4.563	2448	WS01014_A24
9	28.608	170	2	0.191	0.039	4.217	2448	WS01014_A24
9	59.963	177	4	0.262	0.056	6.010	2448	WS01014_A24
12	6.242	234	2	-0.186	0.041	4.812	2448	WS01014_A24
2	120.617	38	4	-0.240	0.037	3.998	2449	WS00924_B08
3	80.211	55	4	0.246	0.048	5.242	2449	WS00924_B08
3	104.681	60	2	0.237	0.045	4.298	2449	WS00924_B08
3	114.747	62	2	-0.192	0.043	4.031	2449	WS00924_B08
6	56.188	120	2	-0.259	0.048	4.813	2449	WS00924_B08
6	81.996	123	3	0.324	0.093	3.854	2449	WS00924_B08
7	62.787	139	4	0.391	0.092	8.796	2449	WS00924_B08
7	74.560	143	1	0.234	0.061	5.645	2449	WS00924_B08
8	87.463	158	2	-0.198	0.061	5.853	2449	WS00924_B08
11	27.405	204	4	-0.208	0.037	4.185	2449	WS00924_B08
13	0.000	243	4	0.387	0.090	4.681	2449	WS00924_B08
1	95.572	17	2	0.215	0.039	4.071	2450	WS00924_E09

9	0.000	163	1	0.368	0.061	4.158	2450	WS00924_E09
9	36.227	165	3	-0.252	0.055	4.385	2450	WS00924_E09
9	59.963	177	4	0.245	0.044	4.831	2450	WS00924_E09
11	0.000	199	2	0.220	0.042	4.367	2450	WS00924_E09
11	27.405	204	1	0.310	0.076	6.499	2450	WS00924_E09
11	27.405	204	4	-0.259	0.056	6.019	2450	WS00924_E09
1	22.419	4	4	0.400	0.198	7.505	2451	WS01014_E21
1	40.464	7	3	-0.347	0.059	5.050	2451	WS01014_E21
1	43.578	8	3	-0.342	0.057	4.649	2451	WS01014_E21
1	62.128	11	1	0.560	0.169	6.112	2451	WS01014_E21
1	95.572	17	2	-0.277	0.050	4.856	2451	WS01014_E21
1	133.992	24	2	-0.224	0.071	6.969	2451	WS01014_E21
1	141.021	25	2	0.199	0.060	5.878	2451	WS01014_E21
9	2.011	167	4	0.280	0.060	6.355	2451	WS01014_E21
9	81.073	183	1	-0.251	0.050	4.490	2451	WS01014_E21
10	9.580	187	2	0.275	0.065	4.552	2451	WS01014_E21
10	95.795	197	4	0.199	0.036	3.854	2451	WS01014_E21
2	93.504	32	4	0.369	0.074	7.397	2452	WS01014_F15
6	56.188	120	2	0.291	0.037	4.769	2452	WS01014_F15
10	9.580	187	2	0.228	0.079	4.274	2452	WS01014_F15
4	92.039	79	4	0.249	0.053	4.392	2453	WS01014_I06
6	56.188	120	1	-0.273	0.048	4.557	2453	WS01014_I06
6	69.073	121	3	-0.232	0.052	4.731	2453	WS01014_I06
9	2.011	167	4	0.252	0.049	4.798	2453	WS01014_I06
9	49.013	173	1	-0.186	0.037	3.987	2453	WS01014_I06
9	55.358	175	1	0.356	0.072	7.012	2453	WS01014_I06
11	27.405	204	4	0.303	0.047	4.772	2453	WS01014_I06
1	62.128	11	1	0.453	0.122	3.902	2454	WS01014_I24
3	9.953	42	3	-0.228	0.051	4.497	2454	WS01014_I24
4	82.462	78	1	0.199	0.059	4.469	2454	WS01014_I24
4	142.917	85	1	-0.254	0.053	4.058	2454	WS01014_I24
7	73.169	142	2	-0.424	0.063	4.112	2454	WS01014_I24
7	75.883	144	2	0.510	0.096	6.765	2454	WS01014_I24
9	17.951	169	2	0.232	0.035	3.934	2454	WS01014_I24
11	64.014	222	4	0.269	0.064	6.216	2454	WS01014_I24
12	14.406	230	4	-0.323	0.087	8.155	2454	WS01014_I24
1	62.128	11	1	0.533	0.149	5.426	2455	WS01014_K08
3	148.745	68	4	0.275	0.047	4.517	2455	WS01014_K08
5	52.820	99	3	0.272	0.049	4.911	2455	WS01014_K08
7	75.883	144	2	0.424	0.082	4.724	2455	WS01014_K08
7	75.883	144	4	-0.310	0.048	4.381	2455	WS01014_K08
9	55.358	175	3	-0.589	0.163	6.752	2455	WS01014_K08
9	58.045	176	1	0.275	0.102	5.168	2455	WS01014_K08
10	93.347	196	2	-0.456	0.100	4.559	2455	WS01014_K08
1	62.128	11	1	0.583	0.144	6.533	2456	WS01014_K23
2	63.261	30	3	-0.182	0.052	4.490	2456	WS01014_K23
2	126.181	39	4	-0.394	0.080	4.284	2456	WS01014_K23
5	0.000	89	3	0.218	0.041	3.999	2456	WS01014_K23
5	57.017	102	4	-0.329	0.046	4.598	2456	WS01014_K23
6	69.073	121	3	-0.247	0.045	3.906	2456	WS01014_K23
7	105.477	147	2	0.317	0.110	6.085	2456	WS01014_K23
9	28.608	170	2	0.279	0.073	7.427	2456	WS01014_K23
9	32.356	171	2	0.345	0.118	11.965	2456	WS01014_K23
9	49.013	173	2	-0.237	0.065	5.745	2456	WS01014_K23

9	59.963	177	4	-0.269	0.052	4.119	2456	WS01014_K23
9	61.113	178	1	0.493	0.150	6.893	2456	WS01014_K23
11	17.088	203	1	-0.235	0.067	5.773	2456	WS01014_K23
11	57.661	220	4	-0.200	0.059	5.942	2456	WS01014_K23
11	60.464	221	1	0.324	0.089	7.762	2456	WS01014_K23
13	0.000	243	4	-0.227	0.093	4.170	2456	WS01014_K23
13	1.815	244	2	0.236	0.045	4.570	2456	WS01014_K23
14	44.898	251	1	0.260	0.077	6.731	2456	WS01014_K23
2	48.839	29	4	-0.244	0.056	5.551	2457	WS01014_M10
3	116.958	63	4	0.178	0.044	4.349	2457	WS01014_M10
4	55.925	76	1	0.337	0.092	9.044	2457	WS01014_M10
4	82.462	78	1	0.159	0.051	4.541	2457	WS01014_M10
5	84.347	105	1	-0.238	0.048	4.320	2457	WS01014_M10
9	3.127	168	1	-0.461	0.114	6.124	2457	WS01014_M10
9	17.951	169	2	0.262	0.066	6.347	2457	WS01014_M10
9	59.963	177	4	-0.285	0.056	5.471	2457	WS01014_M10
10	104.214	198	2	0.240	0.043	3.918	2457	WS01014_M10
10	104.214	198	4	-0.440	0.089	10.668	2457	WS01014_M10
11	57.661	220	4	-0.223	0.057	5.672	2457	WS01014_M10
11	64.014	222	4	0.257	0.059	6.448	2457	WS01014_M10
12	14.406	230	4	-0.236	0.052	5.276	2457	WS01014_M10
13	3.386	245	1	0.282	0.042	3.970	2457	WS01014_M10
1	95.572	17	3	0.253	0.050	4.354	2458	WS01014_M21
1	127.805	21	3	0.205	0.055	5.217	2458	WS01014_M21
2	93.504	32	2	-0.240	0.043	4.700	2458	WS01014_M21
2	93.504	32	4	0.346	0.054	5.194	2458	WS01014_M21
4	116.928	82	2	-0.252	0.040	4.135	2458	WS01014_M21
4	140.962	84	3	-0.248	0.058	5.443	2458	WS01014_M21
4	142.917	85	1	0.320	0.049	4.466	2458	WS01014_M21
4	142.917	85	3	-0.326	0.076	7.406	2458	WS01014_M21
6	49.100	118	4	-0.309	0.156	4.737	2458	WS01014_M21
7	15.555	130	3	0.395	0.103	9.817	2458	WS01014_M21
7	73.169	142	2	-0.467	0.095	4.987	2458	WS01014_M21
7	74.560	143	2	0.262	0.045	4.323	2458	WS01014_M21
7	75.883	144	2	0.690	0.201	12.241	2458	WS01014_M21
9	49.013	173	1	-0.229	0.037	3.909	2458	WS01014_M21
9	59.963	177	1	-0.240	0.048	4.077	2458	WS01014_M21
9	69.046	181	1	0.274	0.090	4.177	2458	WS01014_M21
9	81.073	183	3	-0.231	0.046	4.167	2458	WS01014_M21
13	3.386	245	1	0.253	0.050	4.284	2458	WS01014_M21
3	14.500	43	2	-0.239	0.043	4.760	2459	WS00925_B05
3	20.512	46	2	0.185	0.037	4.029	2459	WS00925_B05
3	69.071	52	1	-0.285	0.134	5.471	2459	WS00925_B05
4	124.132	83	1	-0.497	0.135	4.939	2459	WS00925_B05
5	56.008	101	2	-0.245	0.055	5.007	2459	WS00925_B05
7	75.883	144	4	-0.299	0.060	5.188	2459	WS00925_B05
9	36.227	165	3	0.232	0.051	4.642	2459	WS00925_B05
9	36.227	165	4	-0.305	0.050	4.279	2459	WS00925_B05
9	17.951	169	1	-0.255	0.053	4.056	2459	WS00925_B05
9	59.963	177	1	-0.204	0.056	4.384	2459	WS00925_B05
12	32.473	238	1	-0.280	0.077	5.931	2459	WS00925_B05
3	80.211	55	4	0.234	0.039	4.056	2460	WS00925_E07
3	137.891	64	3	0.300	0.072	4.714	2460	WS00925_E07
4	55.925	76	1	-0.284	0.086	6.110	2460	WS00925_E07

4	142.917	85	3	-0.224	0.057	4.338	2460	WS00925_E07
4	173.929	88	2	-0.239	0.056	6.367	2460	WS00925_E07
7	15.555	130	2	-0.185	0.036	4.045	2460	WS00925_E07
7	17.610	131	3	-0.343	0.145	4.993	2460	WS00925_E07
8	50.001	154	1	-0.227	0.056	4.237	2460	WS00925_E07
11	27.405	204	1	0.263	0.081	6.029	2460	WS00925_E07
11	27.405	204	4	-0.222	0.047	4.840	2460	WS00925_E07
12	6.242	234	2	-0.216	0.030	4.006	2460	WS00925_E07
1	2.788	2	4	0.180	0.049	4.271	2461	WS0031_F07
3	15.147	44	2	-0.258	0.087	4.032	2461	WS0031_F07
3	112.868	61	2	0.176	0.044	4.091	2461	WS0031_F07
3	114.747	62	2	0.213	0.045	4.134	2461	WS0031_F07
6	81.996	123	3	-0.480	0.165	9.357	2461	WS0031_F07
12	38.543	232	4	-0.361	0.097	3.870	2461	WS0031_F07
3	14.500	43	2	-0.258	0.050	6.320	2462	WS01019_D10
3	137.891	64	3	0.334	0.086	5.507	2462	WS01019_D10
4	82.462	78	1	-0.148	0.059	4.247	2462	WS01019_D10
4	116.928	82	2	-0.252	0.040	4.611	2462	WS01019_D10
4	124.132	83	2	-0.273	0.078	4.284	2462	WS01019_D10
10	9.580	187	2	-0.292	0.101	6.304	2462	WS01019_D10
12	22.441	227	3	0.307	0.075	7.175	2462	WS01019_D10
12	28.493	228	2	-0.293	0.107	6.685	2462	WS01019_D10
13	3.386	245	1	-0.312	0.057	4.326	2462	WS01019_D10
14	43.392	250	2	-0.207	0.035	4.134	2462	WS01019_D10
6	81.996	123	3	0.229	0.082	3.999	2463	WS0032_G03
3	69.071	52	1	-0.411	0.079	7.465	2464	WS01019_K18
3	137.891	64	3	0.283	0.040	4.789	2464	WS01019_K18
6	81.996	123	3	0.362	0.049	4.723	2464	WS01019_K18
7	74.560	143	1	0.235	0.066	7.297	2464	WS01019_K18
9	81.073	183	3	-0.234	0.043	5.158	2464	WS01019_K18
10	35.204	189	1	0.324	0.047	5.210	2464	WS01019_K18
12	6.242	234	2	-0.222	0.050	4.424	2464	WS01019_K18
2	95.880	34	3	-0.228	0.044	3.889	2465	IS0012_D24
2	101.076	35	3	0.250	0.049	4.660	2465	IS0012_D24
4	173.929	88	2	-0.184	0.032	4.063	2465	IS0012_D24
5	3.484	90	2	-0.279	0.079	5.051	2465	IS0012_D24
1	15.589	3	2	0.185	0.040	4.214	2466	IS0012_E20
7	74.560	143	2	0.247	0.047	4.632	2466	IS0012_E20
7	75.883	144	2	0.547	0.119	7.958	2466	IS0012_E20
9	28.608	170	3	0.273	0.060	5.657	2466	IS0012_E20
11	60.464	221	2	0.312	0.079	7.350	2466	IS0012_E20
12	22.629	237	1	0.235	0.059	4.960	2466	IS0012_E20
4	82.462	78	2	0.280	0.066	6.009	2467	IS0012_I04
7	74.560	143	1	0.221	0.054	4.951	2467	IS0012_I04
7	74.560	143	2	-0.277	0.042	4.013	2467	IS0012_I04
7	75.883	144	2	-0.478	0.122	6.312	2467	IS0012_I04
9	81.073	183	1	0.182	0.041	3.892	2467	IS0012_I04
9	81.073	183	3	-0.223	0.043	4.727	2467	IS0012_I04
11	40.960	210	2	0.484	0.120	6.657	2467	IS0012_I04
11	40.960	210	4	-0.304	0.052	4.031	2467	IS0012_I04
13	3.386	245	1	-0.326	0.066	6.277	2467	IS0012_I04
14	0.000	246	1	0.245	0.051	4.681	2467	IS0012_I04
14	0.000	246	2	-0.276	0.090	8.581	2467	IS0012_I04
14	9.923	247	1	-0.236	0.047	4.349	2467	IS0012_I04

3	137.891	64	1	0.308	0.103	8.832	2468	IS0012_J01
3	148.745	68	1	-0.326	0.096	8.111	2468	IS0012_J01
3	154.505	69	1	-0.297	0.069	5.779	2468	IS0012_J01
6	95.249	126	1	-0.314	0.113	6.528	2468	IS0012_J01
7	105.477	147	2	0.301	0.067	4.789	2468	IS0012_J01
11	60.464	221	1	0.253	0.059	4.927	2468	IS0012_J01
12	28.493	228	2	0.349	0.088	6.273	2468	IS0012_J01
12	22.629	237	1	0.286	0.073	6.131	2468	IS0012_J01
3	9.953	42	4	-0.207	0.041	4.288	2469	IS0012_L21
3	154.505	69	4	-0.190	0.046	5.046	2469	IS0012_L21
9	55.358	175	1	0.370	0.090	6.592	2469	IS0012_L21
9	55.358	175	3	-0.469	0.165	5.791	2469	IS0012_L21
9	59.963	177	4	-0.210	0.033	3.885	2469	IS0012_L21
11	40.960	210	2	-0.369	0.064	4.297	2469	IS0012_L21
12	6.242	234	4	-0.272	0.027	4.092	2469	IS0012_L21
1	69.854	13	2	0.201	0.034	3.884	2470	IS0012_M19
1	71.592	14	2	0.207	0.036	4.174	2470	IS0012_M19
3	9.953	42	3	0.168	0.048	4.546	2470	IS0012_M19
4	75.958	77	3	-0.253	0.109	5.014	2470	IS0012_M19
5	3.484	90	2	-0.354	0.110	7.612	2470	IS0012_M19
9	0.000	166	2	0.232	0.068	6.497	2470	IS0012_M19
9	49.013	173	2	0.196	0.040	4.637	2470	IS0012_M19
10	78.479	193	3	-0.258	0.055	5.221	2470	IS0012_M19
10	86.151	195	3	0.243	0.057	5.415	2470	IS0012_M19
1	40.464	7	3	0.292	0.090	9.993	2472	IS0013_C07
1	43.578	8	3	0.287	0.088	9.476	2472	IS0013_C07
3	83.563	56	3	-0.164	0.035	3.904	2472	IS0013_C07
4	116.928	82	2	-0.203	0.041	3.841	2472	IS0013_C07
5	16.219	93	1	-0.240	0.043	4.069	2472	IS0013_C07
6	69.073	121	4	-0.212	0.063	4.798	2472	IS0013_C07
7	70.278	141	3	-0.233	0.043	4.852	2472	IS0013_C07
8	62.580	155	1	-0.356	0.148	4.494	2472	IS0013_C07
8	78.389	157	1	-0.245	0.042	3.964	2472	IS0013_C07
10	77.676	192	1	-0.183	0.048	4.505	2472	IS0013_C07
10	80.088	194	1	-0.209	0.052	4.846	2472	IS0013_C07
10	93.347	196	1	0.403	0.062	3.931	2472	IS0013_C07
11	45.989	216	1	-0.215	0.069	6.483	2472	IS0013_C07
14	0.000	246	1	0.260	0.042	3.982	2472	IS0013_C07
1	2.788	2	4	-0.198	0.067	4.692	2473	IS0013_G09
5	16.219	93	1	-0.197	0.050	4.451	2473	IS0013_G09
11	27.405	204	1	0.379	0.099	8.887	2473	IS0013_G09
11	29.069	205	1	0.194	0.052	4.588	2473	IS0013_G09
11	45.989	216	1	-0.202	0.068	6.080	2473	IS0013_G09
12	6.242	234	2	-0.275	0.043	5.473	2473	IS0013_G09
14	0.000	246	1	0.291	0.055	5.043	2473	IS0013_G09
1	40.464	7	3	0.159	0.042	4.194	2474	IS0013_H09
4	116.928	82	2	-0.235	0.046	5.194	2474	IS0013_H09
6	25.914	114	1	-0.288	0.070	5.234	2474	IS0013_H09
10	78.479	193	4	-0.249	0.062	5.349	2474	IS0013_H09
1	50.955	10	1	0.232	0.079	6.243	2475	IS0013_M06
1	62.128	11	1	-0.526	0.150	5.514	2475	IS0013_M06
3	144.647	67	1	0.362	0.191	6.984	2475	IS0013_M06
4	92.039	79	2	-0.174	0.041	4.585	2475	IS0013_M06
5	52.820	99	1	-0.283	0.060	4.961	2475	IS0013_M06

5	56.008	101	1	0.130	0.051	4.099	2475	IS0013_M06
6	89.401	125	1	0.297	0.060	4.905	2475	IS0013_M06
7	74.560	143	1	0.180	0.051	4.068	2475	IS0013_M06
9	3.127	168	1	0.378	0.106	4.080	2475	IS0013_M06
9	17.951	169	1	-0.194	0.048	3.866	2475	IS0013_M06
10	80.088	194	1	0.153	0.049	3.860	2475	IS0013_M06
11	37.143	209	3	0.308	0.065	5.183	2475	IS0013_M06
11	43.491	214	4	0.094	0.026	4.070	2475	IS0013_M06
12	0.000	233	1	0.240	0.051	4.041	2475	IS0013_M06
13	0.000	243	4	0.343	0.167	6.101	2475	IS0013_M06
1	2.788	2	4	-0.342	0.118	8.357	2476	IS0013_N19
3	148.745	68	1	-0.259	0.036	4.036	2476	IS0013_N19
4	173.929	88	2	-0.215	0.050	4.106	2476	IS0013_N19
5	7.672	92	1	-0.321	0.045	5.605	2476	IS0013_N19
7	74.560	143	1	0.192	0.046	4.888	2476	IS0013_N19
11	45.989	216	1	-0.249	0.048	5.117	2476	IS0013_N19
1	29.800	5	1	-0.187	0.049	4.287	2477	IS0014_B05
2	120.617	38	3	-0.182	0.037	4.649	2477	IS0014_B05
2	127.147	40	4	-0.165	0.087	5.396	2477	IS0014_B05
5	16.219	93	2	0.351	0.067	6.425	2477	IS0014_B05
10	35.204	189	1	-0.189	0.057	4.958	2477	IS0014_B05
1	123.316	20	4	0.190	0.070	4.000	2478	IS0014_C09
3	144.647	67	1	0.338	0.108	3.845	2478	IS0014_C09
4	92.039	79	2	-0.240	0.043	4.473	2478	IS0014_C09
4	142.917	85	3	-0.220	0.042	3.845	2478	IS0014_C09
5	56.008	101	1	0.219	0.070	5.912	2478	IS0014_C09
7	74.560	143	1	0.237	0.070	5.871	2478	IS0014_C09
8	50.001	154	1	-0.279	0.046	4.591	2478	IS0014_C09
9	81.073	183	3	-0.278	0.066	6.161	2478	IS0014_C09
10	77.676	192	1	0.301	0.113	9.553	2478	IS0014_C09
10	80.088	194	1	0.268	0.101	8.544	2478	IS0014_C09
11	37.143	209	3	0.247	0.053	5.095	2478	IS0014_C09
12	14.406	230	4	0.269	0.044	4.375	2478	IS0014_C09
3	15.147	44	2	0.258	0.052	4.280	2479	IS0014_E09
3	80.211	55	4	0.208	0.037	3.973	2479	IS0014_E09
4	173.929	88	2	-0.209	0.039	4.419	2479	IS0014_E09
9	36.227	165	3	-0.201	0.050	3.998	2479	IS0014_E09
11	27.405	204	1	0.222	0.057	4.269	2479	IS0014_E09
11	27.405	204	4	-0.313	0.081	8.704	2479	IS0014_E09
12	14.406	230	4	0.250	0.041	4.370	2479	IS0014_E09
1	43.578	8	2	-0.229	0.040	4.160	2480	IS0014_F03
1	82.301	16	3	0.246	0.080	9.115	2480	IS0014_F03
3	95.157	59	2	-0.254	0.038	4.462	2480	IS0014_F03
7	17.610	131	3	0.161	0.068	4.737	2480	IS0014_F03
7	62.787	139	2	0.361	0.068	7.413	2480	IS0014_F03
10	93.347	196	1	0.423	0.099	4.299	2480	IS0014_F03
11	60.464	221	1	-0.208	0.072	6.136	2480	IS0014_F03
11	64.014	222	3	0.266	0.043	4.904	2480	IS0014_F03
1	2.788	2	4	-0.154	0.047	4.279	2481	WS0084_D04
3	9.953	42	3	0.249	0.051	4.901	2481	WS0084_D04
3	15.147	44	2	0.218	0.072	4.844	2481	WS0084_D04
3	114.747	62	2	-0.235	0.040	3.938	2481	WS0084_D04
3	137.891	64	3	0.260	0.069	6.287	2481	WS0084_D04
4	173.929	88	2	-0.169	0.041	3.939	2481	WS0084_D04

5	54.072	100	4	0.217	0.046	4.205	2481	WS0084_D04
6	81.996	123	3	0.327	0.099	6.376	2481	WS0084_D04
9	59.963	177	4	0.183	0.044	3.971	2481	WS0084_D04
11	27.405	204	1	0.255	0.062	5.799	2481	WS0084_D04
6	69.073	121	3	0.220	0.051	4.532	2482	WS0084_J21
8	50.001	154	1	-0.261	0.063	5.368	2482	WS0084_J21
8	87.463	158	2	-0.239	0.061	4.517	2482	WS0084_J21
9	17.951	169	2	-0.257	0.053	4.089	2482	WS0084_J21
9	59.963	177	4	0.235	0.043	4.156	2482	WS0084_J21
10	9.580	187	3	0.321	0.071	3.846	2482	WS0084_J21
1	29.800	5	1	0.276	0.054	4.314	2483	WS01015_K16
1	50.955	10	1	-0.231	0.086	6.792	2483	WS01015_K16
1	82.301	16	3	-0.212	0.049	3.962	2483	WS01015_K16
3	14.500	43	2	-0.334	0.081	8.843	2483	WS01015_K16
3	15.147	44	2	0.229	0.078	5.487	2483	WS01015_K16
3	20.512	46	2	0.384	0.118	12.902	2483	WS01015_K16
3	80.211	55	4	0.240	0.045	4.813	2483	WS01015_K16
5	52.820	99	1	0.222	0.074	5.979	2483	WS01015_K16
5	56.008	101	1	-0.273	0.052	4.275	2483	WS01015_K16
7	59.789	137	3	-0.234	0.068	5.510	2483	WS01015_K16
9	49.013	173	2	0.178	0.043	4.566	2483	WS01015_K16
11	27.405	204	4	-0.363	0.061	6.092	2483	WS01015_K16
12	6.242	234	4	0.403	0.062	4.825	2483	WS01015_K16
1	15.589	3	2	-0.228	0.040	4.509	2484	WS01015_O16
2	93.504	32	2	0.165	0.035	3.890	2484	WS01015_O16
7	62.787	139	4	0.238	0.075	7.947	2484	WS01015_O16
9	0.000	166	1	0.135	0.057	4.384	2484	WS01015_O16
10	78.479	193	3	0.195	0.039	3.920	2484	WS01015_O16
12	6.242	234	2	-0.233	0.062	7.466	2484	WS01015_O16
12	6.242	234	4	0.362	0.048	4.142	2484	WS01015_O16
1	2.788	2	4	0.212	0.075	6.420	2485	IS0011_C15
2	126.181	39	2	0.277	0.046	4.682	2485	IS0011_C15
2	126.181	39	4	-0.436	0.086	5.570	2485	IS0011_C15
5	31.191	95	1	-0.220	0.056	4.906	2485	IS0011_C15
6	25.914	114	1	-0.286	0.084	7.379	2485	IS0011_C15
6	87.891	124	2	0.188	0.040	4.008	2485	IS0011_C15
6	89.401	125	2	0.265	0.053	4.525	2485	IS0011_C15
6	106.702	128	2	-0.410	0.104	4.098	2485	IS0011_C15
9	69.046	181	2	-0.224	0.099	4.074	2485	IS0011_C15
11	57.661	220	4	-0.153	0.044	3.991	2485	IS0011_C15
11	64.014	222	4	0.205	0.058	5.232	2485	IS0011_C15
12	28.493	228	2	0.260	0.093	5.539	2485	IS0011_C15
13	1.815	244	2	0.180	0.040	4.062	2485	IS0011_C15
1	77.177	15	2	0.191	0.027	3.850	2486	IS0011_P03
1	82.301	16	3	0.248	0.055	3.958	2486	IS0011_P03
3	154.505	69	4	-0.217	0.043	5.192	2486	IS0011_P03
6	26.350	115	2	-0.189	0.036	5.019	2486	IS0011_P03
9	2.011	167	4	-0.319	0.090	10.484	2486	IS0011_P03
10	78.479	193	4	-0.248	0.060	6.199	2486	IS0011_P03
12	28.493	228	2	-0.226	0.044	4.012	2486	IS0011_P03
13	1.815	244	2	-0.274	0.053	7.571	2486	IS0011_P03
14	22.402	248	3	0.172	0.058	4.128	2486	IS0011_P03
3	14.500	43	1	-0.207	0.045	4.191	2487	WS0043_L10
3	47.928	49	1	0.245	0.064	5.990	2487	WS0043_L10

5	54.072	100	4	0.215	0.043	4.405	2487	WS0043_L10
6	56.188	120	2	0.241	0.045	3.872	2487	WS0043_L10
7	62.787	139	4	-0.318	0.075	7.190	2487	WS0043_L10
7	75.883	144	4	-0.352	0.072	4.057	2487	WS0043_L10
8	50.001	154	1	0.238	0.058	5.107	2487	WS0043_L10
11	43.491	214	3	-0.216	0.052	4.463	2487	WS0043_L10
11	44.884	215	3	0.260	0.048	4.146	2487	WS0043_L10
11	64.014	222	4	0.366	0.089	9.183	2487	WS0043_L10
13	3.386	245	1	0.269	0.051	4.761	2487	WS0043_L10
2	48.839	29	1	-0.251	0.053	4.444	2488	WS00927_I12
2	75.550	31	1	0.307	0.078	6.707	2488	WS00927_I12
10	70.246	190	4	-0.358	0.108	10.563	2488	WS00927_I12
10	78.479	193	4	-0.197	0.062	4.603	2488	WS00927_I12
11	27.405	204	1	0.336	0.103	9.158	2488	WS00927_I12
3	83.563	56	3	-0.140	0.045	4.203	2489	WS0044_E06
4	116.928	82	2	-0.294	0.050	5.540	2489	WS0044_E06
5	57.017	102	2	0.254	0.052	6.225	2489	WS0044_E06
6	81.996	123	4	-0.204	0.086	4.199	2489	WS0044_E06
8	50.001	154	2	0.235	0.060	4.725	2489	WS0044_E06
8	87.463	158	2	-0.251	0.055	6.055	2489	WS0044_E06
11	0.000	199	2	0.259	0.043	5.022	2489	WS0044_E06
11	17.088	203	1	-0.285	0.089	6.801	2489	WS0044_E06
11	17.088	203	2	-0.220	0.040	4.949	2489	WS0044_E06
11	29.069	205	1	0.358	0.087	6.787	2489	WS0044_E06
11	45.989	216	1	-0.204	0.052	3.923	2489	WS0044_E06
14	0.000	246	1	0.258	0.069	5.293	2489	WS0044_E06
14	22.402	248	4	0.412	0.078	6.353	2489	WS0044_E06
1	2.788	2	4	-0.245	0.060	6.168	2490	WS00927_L18
1	95.572	17	2	0.261	0.052	5.603	2490	WS00927_L18
1	95.572	17	3	-0.221	0.050	4.192	2490	WS00927_L18
2	48.839	29	4	0.231	0.038	3.991	2490	WS00927_L18
4	55.925	76	1	-0.234	0.055	4.118	2490	WS00927_L18
7	105.477	147	2	-0.305	0.076	6.339	2490	WS00927_L18
9	59.963	177	4	0.265	0.057	5.998	2490	WS00927_L18
11	27.405	204	1	0.202	0.048	3.971	2490	WS00927_L18
12	6.242	234	2	-0.293	0.070	9.191	2490	WS00927_L18
1	2.788	2	4	-0.292	0.097	10.175	2491	WS0268_F23
1	2.788	2	4	-0.169	0.063	6.576	2492	WS0268_I15
1	29.800	5	1	0.221	0.040	4.000	2492	WS0268_I15
2	126.181	39	4	-0.443	0.098	5.538	2492	WS0268_I15
3	9.953	42	3	0.328	0.149	14.533	2492	WS0268_I15
3	141.443	65	3	0.284	0.058	3.961	2492	WS0268_I15
5	0.000	89	1	-0.359	0.069	7.306	2492	WS0268_I15
8	0.000	148	4	-0.294	0.090	8.103	2492	WS0268_I15
8	95.937	159	3	-0.234	0.055	5.345	2492	WS0268_I15
11	64.014	222	4	-0.276	0.081	7.580	2492	WS0268_I15
12	6.242	234	2	-0.254	0.048	5.483	2492	WS0268_I15
13	0.000	243	4	0.374	0.261	8.423	2492	WS0268_I15
1	29.800	5	2	0.212	0.041	4.194	2493	WS0268_P07
3	14.500	43	2	-0.233	0.056	5.903	2493	WS0268_P07
5	16.219	93	1	-0.216	0.043	3.858	2493	WS0268_P07
10	93.347	196	1	0.425	0.066	4.341	2493	WS0268_P07
11	27.405	204	1	0.408	0.123	11.477	2493	WS0268_P07
11	29.069	205	1	0.244	0.050	4.420	2493	WS0268_P07

11	45.989	216	1	-0.197	0.066	5.711	2493	WS0268_P07
1	95.572	17	2	0.264	0.040	5.394	2494	WS01032_B02
3	112.868	61	2	-0.173	0.039	5.217	2494	WS01032_B02
3	137.891	64	3	0.236	0.070	4.226	2494	WS01032_B02
7	73.169	142	2	0.594	0.088	9.685	2494	WS01032_B02
7	75.883	144	2	-0.589	0.095	10.611	2494	WS01032_B02
9	59.963	177	4	0.234	0.044	5.663	2494	WS01032_B02
10	95.795	197	4	-0.215	0.035	4.406	2494	WS01032_B02
10	104.214	198	4	0.268	0.022	4.192	2494	WS01032_B02
12	6.242	234	2	-0.270	0.047	10.133	2494	WS01032_B02
14	0.000	246	2	-0.202	0.033	4.118	2494	WS01032_B02
2	126.181	39	4	0.357	0.051	4.349	2495	WS01032_E10
4	92.039	79	4	0.272	0.040	4.663	2495	WS01032_E10
4	116.928	82	2	-0.338	0.058	6.748	2495	WS01032_E10
4	142.917	85	1	0.225	0.069	5.127	2495	WS01032_E10
3	9.953	42	3	0.256	0.054	4.889	2496	WS01032_F19
3	137.891	64	3	0.248	0.059	4.219	2496	WS01032_F19
4	173.929	88	2	-0.228	0.059	5.675	2496	WS01032_F19
6	69.073	121	3	0.220	0.047	4.256	2496	WS01032_F19
6	81.996	123	3	0.369	0.100	6.521	2496	WS01032_F19
8	95.937	159	3	-0.225	0.050	4.561	2496	WS01032_F19
11	0.000	199	2	0.226	0.042	4.026	2496	WS01032_F19
12	6.242	234	2	-0.228	0.051	6.361	2496	WS01032_F19
1	40.464	7	4	-0.317	0.034	3.871	2497	WS01032_I18
1	77.177	15	2	0.222	0.047	5.570	2497	WS01032_I18
1	95.572	17	2	0.290	0.046	5.449	2497	WS01032_I18
2	126.181	39	2	-0.249	0.040	4.770	2497	WS01032_I18
4	92.039	79	2	-0.226	0.037	4.584	2497	WS01032_I18
4	97.576	80	2	0.184	0.024	4.288	2497	WS01032_I18
5	7.672	92	4	-0.245	0.033	3.886	2497	WS01032_I18
12	14.406	230	4	0.307	0.081	8.153	2497	WS01032_I18
12	6.242	234	2	-0.157	0.026	3.889	2497	WS01032_I18
1	95.572	17	3	0.400	0.114	9.039	2498	WS01032_J09
1	107.333	19	3	0.198	0.075	7.188	2498	WS01032_J09
3	137.891	64	3	-0.223	0.064	4.931	2498	WS01032_J09
9	36.227	165	4	0.237	0.065	5.769	2498	WS01032_J09
10	104.214	198	2	-0.246	0.031	4.474	2498	WS01032_J09
12	0.000	226	3	0.161	0.044	4.172	2498	WS01032_J09
12	22.441	227	1	0.197	0.048	3.889	2498	WS01032_J09
12	6.242	234	4	0.374	0.074	9.209	2498	WS01032_J09
1	82.301	16	4	0.221	0.037	4.219	2499	WS00926_K17
4	55.925	76	1	-0.277	0.072	6.090	2499	WS00926_K17
4	173.929	88	2	-0.200	0.041	3.953	2499	WS00926_K17
12	14.406	230	4	0.402	0.097	9.196	2499	WS00926_K17
12	6.242	234	2	-0.175	0.033	3.944	2499	WS00926_K17
12	6.242	234	4	0.356	0.055	5.278	2499	WS00926_K17
1	95.572	17	2	0.304	0.072	7.288	2500	WS00926_L04
3	9.953	42	3	0.230	0.066	4.694	2500	WS00926_L04
3	15.147	44	2	0.300	0.074	6.096	2500	WS00926_L04
5	54.072	100	4	0.193	0.035	4.068	2500	WS00926_L04
5	57.017	102	2	-0.211	0.040	3.921	2500	WS00926_L04
6	81.996	123	3	0.277	0.087	4.193	2500	WS00926_L04
9	59.963	177	4	0.250	0.039	4.344	2500	WS00926_L04
11	64.014	222	4	-0.295	0.041	4.845	2500	WS00926_L04

13	1.815	244	4	0.271	0.041	4.292	2500	WS00926_L04
13	3.386	245	1	-0.217	0.044	3.899	2500	WS00926_L04
14	0.000	246	2	-0.191	0.042	4.184	2500	WS00926_L04
1	95.572	17	2	0.267	0.045	4.743	2501	WS00926_M08
3	9.953	42	3	0.219	0.047	4.100	2501	WS00926_M08
3	15.147	44	2	0.266	0.077	5.552	2501	WS00926_M08
3	137.891	64	3	0.227	0.058	4.808	2501	WS00926_M08
4	173.929	88	2	-0.219	0.054	5.604	2501	WS00926_M08
8	50.001	154	1	-0.289	0.089	8.195	2501	WS00926_M08
14	22.402	248	4	0.241	0.043	4.255	2501	WS00926_M08
3	47.928	49	1	-0.235	0.067	4.729	2502	WS00926_M22
10	78.479	193	3	0.361	0.063	7.460	2502	WS00926_M22
11	45.989	216	1	-0.261	0.086	6.067	2502	WS00926_M22
1	95.572	17	2	0.236	0.042	4.701	2503	WS00926_O05
1	95.572	17	3	-0.221	0.056	5.032	2503	WS00926_O05
3	15.147	44	2	0.336	0.097	7.104	2503	WS00926_O05
3	137.891	64	3	0.188	0.055	4.978	2503	WS00926_O05
4	0.000	71	4	0.228	0.042	4.147	2503	WS00926_O05
4	173.929	88	2	-0.217	0.039	4.312	2503	WS00926_O05
6	56.188	120	2	-0.286	0.045	5.094	2503	WS00926_O05
7	15.555	130	2	-0.254	0.041	4.533	2503	WS00926_O05
8	50.001	154	1	-0.245	0.052	4.453	2503	WS00926_O05
1	77.177	15	2	0.203	0.044	3.859	2504	WS00926_O11
1	107.333	19	4	-0.172	0.036	3.950	2504	WS00926_O11
1	133.992	24	2	0.231	0.049	4.408	2504	WS00926_O11
3	144.647	67	1	0.320	0.099	5.915	2504	WS00926_O11
3	154.505	69	1	-0.225	0.045	4.367	2504	WS00926_O11
5	0.000	89	1	-0.293	0.044	4.374	2504	WS00926_O11
5	16.219	93	1	-0.242	0.039	3.897	2504	WS00926_O11
6	49.100	118	4	0.462	0.182	7.989	2504	WS00926_O11
6	81.996	123	3	0.305	0.118	4.677	2504	WS00926_O11
6	89.401	125	1	0.327	0.063	6.314	2504	WS00926_O11
6	106.702	128	2	0.412	0.099	4.141	2504	WS00926_O11
7	59.789	137	3	-0.223	0.054	4.307	2504	WS00926_O11
7	62.787	139	4	0.274	0.051	5.513	2504	WS00926_O11
7	70.278	141	3	0.245	0.069	5.514	2504	WS00926_O11
7	91.487	146	3	0.202	0.064	5.129	2504	WS00926_O11
9	0.000	166	1	0.254	0.067	6.691	2504	WS00926_O11
9	55.358	175	1	-0.261	0.042	4.194	2504	WS00926_O11
13	3.386	245	1	-0.361	0.095	9.519	2504	WS00926_O11
14	22.402	248	4	0.257	0.053	5.568	2504	WS00926_O11
14	44.898	251	3	-0.332	0.088	4.593	2504	WS00926_O11
1	95.572	17	2	0.250	0.039	3.957	2505	WS00926_P18
3	114.747	62	2	-0.217	0.044	4.400	2505	WS00926_P18
4	173.929	88	2	-0.219	0.065	6.495	2505	WS00926_P18
9	59.963	177	4	0.219	0.048	4.621	2505	WS00926_P18
11	64.014	222	4	-0.231	0.050	4.791	2505	WS00926_P18
12	6.242	234	2	-0.242	0.047	5.861	2505	WS00926_P18
14	0.000	246	2	-0.226	0.050	5.007	2505	WS00926_P18
3	9.953	42	3	0.275	0.063	6.385	2506	WS00927_A11
3	114.747	62	2	-0.228	0.044	4.032	2506	WS00927_A11
3	116.958	63	4	-0.217	0.044	3.871	2506	WS00927_A11
3	137.891	64	3	0.214	0.059	5.236	2506	WS00927_A11
3	148.745	68	1	-0.312	0.074	7.261	2506	WS00927_A11

5	52.820	99	3	-0.340	0.067	5.731	2506	WS00927_A11
5	86.669	106	2	-0.233	0.122	4.029	2506	WS00927_A11
8	50.001	154	1	-0.250	0.049	5.294	2506	WS00927_A11
8	95.937	159	3	-0.228	0.072	7.231	2506	WS00927_A11
9	59.963	177	4	0.233	0.044	3.872	2506	WS00927_A11
11	45.989	216	1	-0.306	0.080	7.819	2506	WS00927_A11
1	95.572	17	2	0.217	0.060	4.807	2507	WS0085_N21
2	63.261	30	3	0.207	0.039	4.029	2507	WS0085_N21
2	127.147	40	4	-0.250	0.072	5.985	2507	WS0085_N21
3	9.953	42	3	0.247	0.043	4.547	2507	WS0085_N21
8	95.937	159	3	-0.254	0.068	7.071	2507	WS0085_N21
9	0.000	166	2	0.230	0.065	4.922	2507	WS0085_N21
11	43.491	214	3	0.213	0.048	5.031	2507	WS0085_N21
1	62.128	11	1	-0.481	0.126	4.698	2508	WS0086_F13
4	55.925	76	1	-0.244	0.044	3.842	2508	WS0086_F13
4	82.462	78	1	-0.140	0.043	3.870	2508	WS0086_F13
6	69.073	121	3	0.338	0.040	4.474	2508	WS0086_F13
6	95.249	126	1	-0.310	0.103	5.211	2508	WS0086_F13
9	28.608	170	2	0.274	0.045	4.570	2508	WS0086_F13
9	81.073	183	1	0.229	0.050	4.325	2508	WS0086_F13
12	22.629	237	1	0.222	0.069	6.004	2508	WS0086_F13
12	32.473	238	1	0.235	0.074	6.478	2508	WS0086_F13
13	1.815	244	4	0.189	0.066	6.682	2508	WS0086_F13
1	2.788	2	4	-0.299	0.072	4.793	2509	IS0014_L04
1	43.578	8	3	0.271	0.062	6.081	2509	IS0014_L04
2	63.261	30	3	0.173	0.064	5.696	2509	IS0014_L04
7	74.560	143	1	0.314	0.090	7.703	2509	IS0014_L04
11	27.405	204	1	0.219	0.059	5.007	2509	IS0014_L04
1	22.419	4	3	0.376	0.069	5.533	2510	WS0041_D06
1	40.464	7	3	0.362	0.126	13.925	2510	WS0041_D06
1	43.578	8	3	0.377	0.138	15.003	2510	WS0041_D06
1	45.794	9	3	-0.299	0.075	8.427	2510	WS0041_D06
6	25.914	114	1	-0.272	0.050	5.458	2510	WS0041_D06
10	93.347	196	1	0.576	0.105	8.339	2510	WS0041_D06
2	95.880	34	1	0.219	0.043	5.389	2511	WS00811_E15
3	116.958	63	4	-0.301	0.067	8.489	2511	WS00811_E15
3	137.891	64	1	0.219	0.033	4.167	2511	WS00811_E15
3	141.443	65	4	0.247	0.049	4.213	2511	WS00811_E15
4	0.000	71	4	-0.231	0.037	4.658	2511	WS00811_E15
4	124.132	83	1	0.602	0.096	8.143	2511	WS00811_E15
4	142.917	85	1	-0.243	0.036	4.537	2511	WS00811_E15
11	0.000	199	3	-0.302	0.088	4.918	2511	WS00811_E15
11	45.989	216	1	-0.231	0.047	5.983	2511	WS00811_E15
13	0.000	243	4	0.387	0.099	8.179	2511	WS00811_E15
13	1.815	244	2	-0.187	0.072	4.665	2511	WS00811_E15
13	1.815	244	4	0.275	0.033	4.087	2511	WS00811_E15
13	3.386	245	1	-0.205	0.045	5.762	2511	WS00811_E15
14	0.000	246	1	0.299	0.065	8.252	2511	WS00811_E15
3	104.681	60	2	0.238	0.040	3.960	2512	WS00811_F09
5	54.072	100	4	-0.241	0.098	10.014	2512	WS00811_F09
5	57.017	102	4	0.225	0.062	7.381	2512	WS00811_F09
5	86.669	106	4	-0.792	0.351	18.012	2512	WS00811_F09
7	75.883	144	4	-0.389	0.065	4.202	2512	WS00811_F09
8	50.001	154	2	0.270	0.064	6.583	2512	WS00811_F09

9	0.000	166	1	-0.156	0.043	4.003	2512	WS00811_F09
9	17.951	169	1	0.162	0.043	3.954	2512	WS00811_F09
9	49.013	173	1	0.181	0.063	5.639	2512	WS00811_F09
11	0.000	199	3	-0.381	0.130	10.772	2512	WS00811_F09
12	51.382	242	1	-0.466	0.136	6.838	2512	WS00811_F09
1	95.572	17	2	0.288	0.061	6.524	2513	WS00811_H18
1	127.805	21	3	-0.231	0.050	4.532	2513	WS00811_H18
2	120.617	38	4	-0.337	0.073	5.608	2513	WS00811_H18
3	9.953	42	3	0.239	0.047	4.214	2513	WS00811_H18
4	173.929	88	2	-0.259	0.067	6.985	2513	WS00811_H18
5	3.484	90	2	-0.273	0.096	5.720	2513	WS00811_H18
13	0.000	243	4	0.400	0.130	5.060	2513	WS00811_H18
13	1.815	244	2	-0.156	0.037	3.979	2513	WS00811_H18
14	0.000	246	2	-0.175	0.039	3.915	2513	WS00811_H18
3	9.953	42	3	0.259	0.064	4.828	2514	WS00811_I03
4	55.925	76	1	-0.239	0.060	5.775	2514	WS00811_I03
4	116.928	82	4	-0.227	0.034	4.436	2514	WS00811_I03
5	3.484	90	2	-0.212	0.068	4.027	2514	WS00811_I03
6	87.891	124	2	-0.254	0.062	5.665	2514	WS00811_I03
6	106.702	128	2	0.404	0.119	4.001	2514	WS00811_I03
8	50.001	154	2	0.274	0.080	4.079	2514	WS00811_I03
8	87.463	158	2	-0.219	0.062	5.732	2514	WS00811_I03
9	0.000	166	1	-0.170	0.044	4.308	2514	WS00811_I03
9	17.951	169	1	0.261	0.056	5.435	2514	WS00811_I03
11	0.000	199	2	0.269	0.053	4.995	2514	WS00811_I03
11	45.989	216	1	-0.289	0.069	6.700	2514	WS00811_I03
12	0.000	233	1	0.221	0.040	3.853	2514	WS00811_I03
12	22.629	237	1	0.246	0.047	4.576	2514	WS00811_I03
14	22.402	248	4	0.329	0.047	4.883	2514	WS00811_I03
14	46.637	252	4	0.371	0.062	7.078	2514	WS00811_I03
1	82.301	16	3	0.371	0.082	8.592	2515	WS00811_K15
1	127.805	21	3	-0.226	0.041	4.263	2515	WS00811_K15
3	80.211	55	4	-0.220	0.048	4.007	2515	WS00811_K15
4	55.925	76	1	0.256	0.045	5.072	2515	WS00811_K15
4	116.928	82	2	-0.240	0.048	3.943	2515	WS00811_K15
4	116.928	82	4	0.317	0.058	4.947	2515	WS00811_K15
4	140.962	84	3	0.208	0.058	6.059	2515	WS00811_K15
5	57.017	102	2	0.209	0.048	3.870	2515	WS00811_K15
8	50.001	154	1	0.281	0.083	9.312	2515	WS00811_K15
8	78.389	157	1	0.258	0.050	5.357	2515	WS00811_K15
8	87.463	158	1	-0.235	0.042	4.463	2515	WS00811_K15
11	46.026	217	1	0.231	0.054	5.750	2515	WS00811_K15
13	3.386	245	1	0.244	0.054	5.727	2515	WS00811_K15
1	29.800	5	1	-0.306	0.075	6.454	2516	WS00811_L11
10	21.864	188	1	-0.219	0.048	4.065	2516	WS00811_L11
14	46.637	252	4	0.324	0.053	3.987	2516	WS00811_L11
3	9.953	42	3	0.236	0.078	6.787	2517	WS00811_O01
6	81.996	123	3	0.348	0.118	6.143	2517	WS00811_O01
8	50.001	154	1	-0.179	0.043	4.234	2517	WS00811_O01
9	59.963	177	4	0.314	0.063	6.301	2517	WS00811_O01
1	2.788	2	4	-0.200	0.064	6.158	2518	WS00811_O20
1	22.419	4	4	-0.389	0.148	8.135	2518	WS00811_O20
1	38.326	6	4	-0.261	0.058	5.395	2518	WS00811_O20
1	40.464	7	4	0.278	0.066	6.061	2518	WS00811_O20

2	75.550	31	1	0.237	0.045	5.482	2518	WS00811_O20
2	127.147	40	3	0.198	0.042	4.058	2518	WS00811_O20
4	92.039	79	4	0.388	0.079	7.538	2518	WS00811_O20
4	103.719	81	4	0.213	0.049	4.207	2518	WS00811_O20
4	116.928	82	4	-0.329	0.061	5.777	2518	WS00811_O20
9	0.000	163	1	-0.767	0.197	13.096	2518	WS00811_O20
9	81.073	183	1	0.296	0.055	6.688	2518	WS00811_O20
10	9.580	187	3	-0.331	0.068	4.504	2518	WS00811_O20
11	9.237	202	4	-0.292	0.077	7.106	2518	WS00811_O20
2	63.261	30	3	0.282	0.063	5.299	2519	WS00912_N16
2	127.147	40	4	-0.230	0.037	4.133	2519	WS00912_N16
3	80.211	55	4	0.180	0.044	4.668	2519	WS00912_N16
4	0.000	71	4	-0.247	0.059	6.202	2519	WS00912_N16
4	140.962	84	3	-0.176	0.047	3.922	2519	WS00912_N16
4	142.917	85	3	-0.268	0.064	5.312	2519	WS00912_N16
5	42.979	97	1	-0.320	0.064	6.519	2519	WS00912_N16
5	54.072	100	4	0.320	0.075	7.905	2519	WS00912_N16
11	27.405	204	4	-0.234	0.037	4.086	2519	WS00912_N16
14	46.637	252	3	0.217	0.052	4.295	2519	WS00912_N16
3	9.953	42	3	0.176	0.034	4.194	2520	WS00912_O03
3	137.891	64	3	0.249	0.038	4.664	2520	WS00912_O03
5	57.017	102	2	-0.238	0.039	4.986	2520	WS00912_O03
8	87.463	158	2	-0.207	0.036	4.297	2520	WS00912_O03
1	62.128	11	1	-0.535	0.136	5.519	2521	WS00913_A07
4	82.462	78	1	-0.166	0.073	5.289	2521	WS00913_A07
4	116.928	82	4	0.280	0.054	4.874	2521	WS00913_A07
11	27.405	204	4	-0.191	0.049	5.848	2521	WS00913_A07
1	95.572	17	3	0.316	0.047	5.876	2522	WS00913_A21
3	83.563	56	3	0.191	0.036	4.481	2522	WS00913_A21
3	141.443	65	3	-0.377	0.112	5.797	2522	WS00913_A21
4	124.132	83	1	0.466	0.141	4.344	2522	WS00913_A21
5	0.000	89	3	0.311	0.058	4.862	2522	WS00913_A21
6	106.702	128	2	0.397	0.055	3.850	2522	WS00913_A21
7	15.555	130	2	0.335	0.085	8.990	2522	WS00913_A21
7	17.610	131	3	0.351	0.076	5.461	2522	WS00913_A21
9	0.000	166	2	0.232	0.063	7.037	2522	WS00913_A21
10	78.479	193	3	-0.394	0.068	8.362	2522	WS00913_A21
10	93.347	196	2	0.453	0.066	4.548	2522	WS00913_A21
12	0.000	226	3	0.242	0.036	4.540	2522	WS00913_A21
12	14.406	230	3	0.265	0.031	3.911	2522	WS00913_A21
13	0.000	243	4	0.313	0.129	3.985	2522	WS00913_A21
1	22.419	4	3	-0.286	0.107	4.951	2523	WS00913_C18
1	50.955	10	1	-0.211	0.045	4.076	2523	WS00913_C18
5	86.669	106	4	0.317	0.104	4.517	2523	WS00913_C18
7	105.477	147	2	0.265	0.073	3.943	2523	WS00913_C18
8	95.937	159	3	0.228	0.053	4.816	2523	WS00913_C18
10	21.864	188	1	0.316	0.056	5.004	2523	WS00913_C18
10	35.204	189	2	0.286	0.054	5.137	2523	WS00913_C18
10	78.479	193	3	-0.280	0.048	4.305	2523	WS00913_C18
10	86.151	195	3	0.279	0.089	8.004	2523	WS00913_C18
11	56.921	219	1	0.343	0.101	4.395	2523	WS00913_C18
12	6.242	234	4	0.181	0.031	4.169	2523	WS00913_C18
14	0.000	246	1	-0.231	0.051	4.550	2523	WS00913_C18
3	15.147	44	2	0.321	0.080	4.899	2524	WS00913_D20

3	20.512	46	2	0.191	0.034	3.967	2524 WS00913_D20
3	80.211	55	4	0.257	0.053	5.215	2524 WS00913_D20
3	112.868	61	2	-0.197	0.040	4.559	2524 WS00913_D20
3	114.747	62	2	-0.221	0.044	5.135	2524 WS00913_D20
5	52.820	99	3	-0.291	0.047	4.416	2524 WS00913_D20
5	57.017	102	2	-0.265	0.073	8.868	2524 WS00913_D20
5	86.669	106	2	-0.282	0.088	4.491	2524 WS00913_D20
6	56.188	120	2	-0.267	0.050	6.188	2524 WS00913_D20
6	69.073	121	3	0.269	0.074	6.678	2524 WS00913_D20
6	81.996	123	3	0.346	0.103	4.030	2524 WS00913_D20
11	40.960	210	4	0.244	0.043	4.222	2524 WS00913_D20
13	3.386	245	1	-0.265	0.058	4.225	2524 WS00913_D20
3	9.953	42	3	0.308	0.097	7.669	2525 WS00913_G11
3	15.147	44	2	0.303	0.099	7.002	2525 WS00913_G11
3	20.512	46	2	0.283	0.052	5.584	2525 WS00913_G11
3	137.891	64	3	0.224	0.081	6.895	2525 WS00913_G11
5	52.820	99	1	0.368	0.079	6.937	2525 WS00913_G11
8	87.463	158	2	-0.258	0.072	6.636	2525 WS00913_G11
8	95.937	159	3	-0.192	0.084	6.578	2525 WS00913_G11
9	59.963	177	4	0.231	0.047	5.342	2525 WS00913_G11
11	27.405	204	1	0.301	0.064	5.456	2525 WS00913_G11
11	29.069	205	1	0.325	0.084	7.031	2525 WS00913_G11
11	45.989	216	1	-0.302	0.091	7.542	2525 WS00913_G11
1	43.578	8	2	-0.196	0.044	4.003	2526 WS00913_H05
1	95.572	17	2	0.263	0.045	4.222	2526 WS00913_H05
1	95.572	17	3	-0.256	0.055	5.146	2526 WS00913_H05
3	9.953	42	3	0.237	0.053	4.760	2526 WS00913_H05
3	137.891	64	3	0.207	0.057	5.210	2526 WS00913_H05
5	7.672	92	1	-0.220	0.036	4.074	2526 WS00913_H05
11	27.405	204	1	0.308	0.067	6.544	2526 WS00913_H05
11	45.989	216	1	-0.200	0.043	4.113	2526 WS00913_H05
12	6.242	234	2	-0.201	0.050	6.632	2526 WS00913_H05
1	2.788	2	4	-0.156	0.040	4.412	2527 WS0097_B24
1	40.464	7	3	0.293	0.058	5.155	2527 WS0097_B24
1	43.578	8	2	-0.249	0.054	4.349	2527 WS0097_B24
1	43.578	8	3	0.375	0.096	8.820	2527 WS0097_B24
2	63.261	30	3	0.205	0.064	5.587	2527 WS0097_B24
3	80.211	55	4	0.243	0.038	3.882	2527 WS0097_B24
3	144.593	66	4	-0.243	0.043	4.342	2527 WS0097_B24
3	148.745	68	4	0.203	0.038	3.861	2527 WS0097_B24
11	40.960	210	4	-0.357	0.061	5.273	2527 WS0097_B24
11	43.491	214	4	-0.414	0.128	12.968	2527 WS0097_B24
11	44.884	215	4	0.376	0.053	4.361	2527 WS0097_B24
11	46.026	217	1	0.535	0.206	21.817	2527 WS0097_B24
11	57.661	220	4	0.266	0.088	8.824	2527 WS0097_B24
11	64.014	222	4	-0.251	0.066	6.295	2527 WS0097_B24
1	2.788	2	4	-0.257	0.065	7.702	2528 WS0097_C21
1	22.419	4	3	0.355	0.098	5.277	2528 WS0097_C21
2	48.839	29	4	0.288	0.043	4.459	2528 WS0097_C21
3	80.211	55	4	0.304	0.082	9.282	2528 WS0097_C21
10	104.214	198	2	-0.174	0.029	3.947	2528 WS0097_C21
11	27.405	204	1	0.276	0.051	4.238	2528 WS0097_C21
11	27.405	204	4	-0.271	0.053	6.049	2528 WS0097_C21
12	14.406	230	4	0.336	0.080	7.221	2528 WS0097_C21

1	43.578	8	2	-0.291	0.069	6.107	2529	WS0097_E16
1	45.794	9	1	-0.300	0.077	7.727	2529	WS0097_E16
1	69.854	13	2	0.161	0.045	3.943	2529	WS0097_E16
1	95.572	17	2	0.315	0.083	7.369	2529	WS0097_E16
1	107.333	19	4	0.151	0.029	3.964	2529	WS0097_E16
2	48.839	29	4	0.253	0.039	4.887	2529	WS0097_E16
2	93.504	32	4	-0.301	0.063	8.148	2529	WS0097_E16
4	55.925	76	1	-0.228	0.052	5.277	2529	WS0097_E16
7	73.169	142	1	0.500	0.068	4.046	2529	WS0097_E16
9	59.963	177	4	0.306	0.058	7.320	2529	WS0097_E16
11	9.237	202	4	0.209	0.047	5.966	2529	WS0097_E16
12	22.441	227	1	-0.322	0.082	8.310	2529	WS0097_E16
2	102.814	36	1	0.303	0.086	7.071	2530	WS0097_F04
3	47.928	49	1	0.245	0.044	4.057	2530	WS0097_F04
5	57.017	102	2	-0.238	0.041	3.994	2530	WS0097_F04
9	28.608	170	3	0.324	0.044	3.905	2530	WS0097_F04
9	55.358	175	3	0.563	0.114	8.238	2530	WS0097_F04
10	77.676	192	1	-0.196	0.048	4.387	2530	WS0097_F04
10	80.088	194	1	-0.225	0.049	4.508	2530	WS0097_F04
10	104.214	198	2	-0.235	0.030	4.148	2530	WS0097_F04
14	46.637	252	3	0.211	0.038	4.467	2530	WS0097_F04
1	2.788	2	4	-0.189	0.084	4.156	2531	WS0097_H01
1	40.464	7	3	0.212	0.035	4.482	2531	WS0097_H01
2	63.261	30	3	0.247	0.036	4.724	2531	WS0097_H01
5	7.672	92	1	-0.257	0.042	5.998	2531	WS0097_H01
5	56.008	101	1	0.259	0.048	5.538	2531	WS0097_H01
10	93.347	196	1	0.488	0.059	5.857	2531	WS0097_H01
11	17.088	203	1	-0.314	0.068	7.959	2531	WS0097_H01
11	27.405	204	1	0.432	0.116	13.549	2531	WS0097_H01
11	29.069	205	1	0.274	0.065	7.579	2531	WS0097_H01
11	45.989	216	1	-0.282	0.078	9.052	2531	WS0097_H01
13	3.386	245	1	-0.198	0.038	4.401	2531	WS0097_H01
6	25.914	114	1	-0.224	0.065	4.666	2532	WS0097_H14
7	15.555	130	2	0.233	0.039	4.567	2532	WS0097_H14
7	59.789	137	3	-0.217	0.045	4.226	2532	WS0097_H14
9	17.951	169	2	0.276	0.040	4.339	2532	WS0097_H14
13	3.386	245	1	-0.248	0.064	4.601	2532	WS0097_H14
3	148.745	68	1	-0.242	0.038	4.047	2533	WS0097_J20
4	173.929	88	2	-0.251	0.057	4.809	2533	WS0097_J20
6	81.996	123	3	0.285	0.064	3.934	2533	WS0097_J20
11	9.237	202	4	0.234	0.047	5.215	2533	WS0097_J20
11	45.989	216	1	-0.257	0.041	4.359	2533	WS0097_J20
12	0.000	233	1	0.278	0.054	5.708	2533	WS0097_J20
1	29.800	5	2	0.215	0.045	4.308	2534	WS0097_K11
1	95.572	17	2	0.276	0.047	4.628	2534	WS0097_K11
2	63.261	30	3	0.226	0.045	4.055	2534	WS0097_K11
3	9.953	42	3	0.249	0.061	5.484	2534	WS0097_K11
4	173.929	88	2	-0.171	0.043	4.099	2534	WS0097_K11
9	59.963	177	4	0.170	0.042	4.100	2534	WS0097_K11
11	27.405	204	1	0.272	0.067	6.302	2534	WS0097_K11
11	45.989	216	1	-0.215	0.061	5.573	2534	WS0097_K11
12	6.242	234	2	-0.183	0.039	5.189	2534	WS0097_K11
13	3.386	245	1	-0.239	0.048	4.397	2534	WS0097_K11
14	0.000	246	1	0.243	0.044	4.038	2534	WS0097_K11

1	43.578	8	2	0.260	0.058	6.207	2535	WS0041_L03
1	107.333	19	4	-0.225	0.074	6.141	2535	WS0041_L03
3	137.891	64	3	-0.214	0.048	4.440	2535	WS0041_L03
3	141.443	65	3	-0.335	0.086	5.113	2535	WS0041_L03
7	15.555	130	2	-0.243	0.051	5.400	2535	WS0041_L03
9	32.356	171	2	0.182	0.038	4.074	2535	WS0041_L03
11	27.405	204	1	0.317	0.110	8.649	2535	WS0041_L03
11	29.069	205	1	0.167	0.053	4.242	2535	WS0041_L03
1	2.788	2	4	-0.231	0.044	4.693	2536	WS0042_C17
3	104.681	60	2	0.265	0.050	4.724	2536	WS0042_C17
3	112.868	61	2	-0.224	0.046	4.248	2536	WS0042_C17
3	114.747	62	2	-0.200	0.061	5.557	2536	WS0042_C17
3	148.745	68	1	-0.333	0.052	5.381	2536	WS0042_C17
5	42.979	97	1	-0.328	0.066	6.474	2536	WS0042_C17
5	84.347	105	1	0.157	0.045	4.338	2536	WS0042_C17
8	95.937	159	3	-0.247	0.053	4.559	2536	WS0042_C17
11	64.014	222	4	-0.259	0.039	4.653	2536	WS0042_C17
13	0.000	243	4	0.333	0.110	4.293	2536	WS0042_C17
14	9.923	247	1	-0.148	0.040	3.841	2536	WS0042_C17
14	22.402	248	4	0.183	0.028	3.879	2536	WS0042_C17
4	142.917	85	3	-0.260	0.063	4.510	2537	WS00816_C06
4	173.929	88	2	-0.267	0.079	8.136	2537	WS00816_C06
5	3.484	90	2	-0.332	0.108	7.479	2537	WS00816_C06
9	36.227	165	3	-0.223	0.067	5.988	2537	WS00816_C06
1	95.572	17	2	0.272	0.056	5.731	2538	WS00816_I13
4	173.929	88	2	-0.188	0.039	3.990	2538	WS00816_I13
9	59.963	177	4	0.363	0.100	7.313	2538	WS00816_I13
10	70.246	190	2	-0.353	0.077	8.198	2538	WS00816_I13
10	80.088	194	2	0.239	0.044	5.037	2538	WS00816_I13
12	6.242	234	2	-0.243	0.042	5.837	2538	WS00816_I13
14	0.000	246	2	-0.308	0.108	11.040	2538	WS00816_I13
7	59.789	137	3	0.228	0.066	7.143	2539	WS0262_L03
7	70.278	141	3	-0.282	0.101	10.986	2539	WS0262_L03
11	27.405	204	4	-0.243	0.055	4.522	2539	WS0262_L03
14	22.402	248	3	-0.182	0.037	4.038	2539	WS0262_L03
1	2.788	2	4	-0.208	0.046	6.108	2540	WS0262_P13
1	69.854	13	2	0.204	0.047	5.640	2540	WS0262_P13
1	71.592	14	2	0.193	0.043	5.118	2540	WS0262_P13
1	77.177	15	2	0.209	0.049	5.911	2540	WS0262_P13
1	107.333	19	4	0.171	0.034	4.339	2540	WS0262_P13
3	116.958	63	4	-0.160	0.032	4.071	2540	WS0262_P13
7	105.477	147	2	-0.219	0.051	5.125	2540	WS0262_P13
8	50.001	154	1	-0.211	0.058	4.324	2540	WS0262_P13
11	27.405	204	1	0.314	0.107	7.466	2540	WS0262_P13
13	3.386	245	1	-0.236	0.067	4.472	2540	WS0262_P13
14	0.000	246	2	-0.202	0.041	4.420	2540	WS0262_P13
2	75.550	31	1	0.188	0.036	3.859	2541	WS0038_G11
7	15.555	130	3	0.306	0.074	4.488	2541	WS0038_G11
10	21.864	188	1	-0.298	0.042	4.608	2541	WS0038_G11
11	27.405	204	1	0.307	0.065	7.284	2541	WS0038_G11
7	74.560	143	1	0.236	0.058	4.485	2542	WS0038_O21
13	3.386	245	1	-0.310	0.087	6.812	2542	WS0038_O21
1	2.788	2	4	-0.181	0.034	4.071	2543	WS00922_O19
1	22.419	4	4	-0.319	0.075	4.137	2543	WS00922_O19

1	38.326	6	4	-0.328	0.060	5.274	2543	WS00922_O19
1	40.464	7	4	0.360	0.073	7.135	2543	WS00922_O19
1	95.572	17	3	0.182	0.058	5.670	2543	WS00922_O19
1	107.333	19	3	0.256	0.056	5.523	2543	WS00922_O19
1	131.686	23	1	0.547	0.121	5.119	2543	WS00922_O19
2	75.550	31	1	0.280	0.039	3.893	2543	WS00922_O19
2	127.147	40	4	0.175	0.052	4.211	2543	WS00922_O19
5	0.000	89	3	-0.282	0.079	4.686	2543	WS00922_O19
6	25.914	114	1	-0.260	0.047	4.680	2543	WS00922_O19
7	62.787	139	4	-0.285	0.075	6.507	2543	WS00922_O19
9	28.608	170	3	0.314	0.047	4.530	2543	WS00922_O19
11	17.088	203	1	-0.366	0.094	9.416	2543	WS00922_O19
11	27.405	204	1	0.420	0.085	8.569	2543	WS00922_O19
11	29.069	205	1	0.398	0.100	10.028	2543	WS00922_O19
11	45.989	216	1	-0.230	0.074	7.272	2543	WS00922_O19
12	22.441	227	3	-0.322	0.085	7.039	2543	WS00922_O19
12	32.473	238	1	0.215	0.060	5.964	2543	WS00922_O19
12	51.382	242	1	-0.505	0.200	8.523	2543	WS00922_O19
1	95.572	17	2	0.251	0.052	5.190	2544	WS00922_P05
7	15.555	130	2	-0.246	0.050	4.983	2544	WS00922_P05
11	27.405	204	1	0.255	0.067	5.991	2544	WS00922_P05
11	27.405	204	4	-0.255	0.048	4.864	2544	WS00922_P05
11	45.989	216	1	-0.214	0.046	4.025	2544	WS00922_P05
12	6.242	234	2	-0.223	0.050	6.340	2544	WS00922_P05
7	15.555	130	3	0.218	0.056	4.196	2545	WS01024_A10
10	70.246	190	2	-0.171	0.044	3.905	2545	WS01024_A10
10	93.347	196	1	0.396	0.046	3.859	2545	WS01024_A10
11	27.405	204	1	0.185	0.050	5.046	2545	WS01024_A10
11	27.405	204	4	-0.280	0.053	4.158	2545	WS01024_A10
13	3.386	245	1	-0.311	0.069	6.921	2545	WS01024_A10
1	2.788	2	4	-0.232	0.046	4.443	2546	WS01024_B01
1	15.589	3	2	-0.255	0.047	4.680	2546	WS01024_B01
2	102.814	36	1	-0.277	0.099	8.931	2546	WS01024_B01
4	173.929	88	2	-0.254	0.043	4.345	2546	WS01024_B01
5	56.008	101	1	0.180	0.050	4.532	2546	WS01024_B01
6	69.073	121	3	0.192	0.052	5.611	2546	WS01024_B01
7	74.560	143	1	0.198	0.062	5.590	2546	WS01024_B01
9	59.963	177	4	0.271	0.056	4.182	2546	WS01024_B01
1	95.572	17	2	0.288	0.066	6.449	2547	WS01024_C06
2	127.147	40	4	-0.255	0.041	4.683	2547	WS01024_C06
5	3.484	90	2	-0.340	0.102	7.074	2547	WS01024_C06
5	86.669	106	4	-0.546	0.153	7.816	2547	WS01024_C06
6	76.376	122	1	-0.232	0.104	4.831	2547	WS01024_C06
7	67.154	140	4	-0.210	0.092	4.280	2547	WS01024_C06
7	73.169	142	1	0.560	0.107	5.014	2547	WS01024_C06
9	55.358	175	3	-0.411	0.108	4.857	2547	WS01024_C06
11	40.960	210	2	-0.455	0.084	6.422	2547	WS01024_C06
11	40.960	210	4	0.337	0.038	4.601	2547	WS01024_C06
11	45.989	216	1	-0.355	0.090	8.119	2547	WS01024_C06
12	38.543	232	4	-0.416	0.086	4.169	2547	WS01024_C06
12	51.382	242	1	-0.523	0.175	8.262	2547	WS01024_C06
1	95.572	17	2	0.269	0.059	5.037	2548	WS01024_C17
3	15.147	44	2	0.232	0.072	4.885	2548	WS01024_C17
4	173.929	88	2	-0.202	0.061	5.068	2548	WS01024_C17

9	59.963	177	4	0.263	0.059	7.101	2548	WS01024_C17
11	45.989	216	1	-0.198	0.049	5.027	2548	WS01024_C17
11	60.464	221	1	0.258	0.037	3.851	2548	WS01024_C17
12	38.543	232	4	-0.291	0.078	4.623	2548	WS01024_C17
12	6.242	234	2	-0.166	0.038	4.246	2548	WS01024_C17
12	6.242	234	4	0.357	0.057	5.682	2548	WS01024_C17
13	0.000	243	4	0.324	0.083	4.979	2548	WS01024_C17
13	3.386	245	1	-0.289	0.088	9.180	2548	WS01024_C17
1	62.128	11	1	-0.717	0.198	9.836	2549	WS01024_E06
2	95.880	34	1	0.394	0.056	5.716	2549	WS01024_E06
3	69.071	52	1	-0.369	0.078	4.211	2549	WS01024_E06
3	141.443	65	4	0.466	0.113	4.778	2549	WS01024_E06
4	75.958	77	3	0.408	0.135	4.646	2549	WS01024_E06
4	82.462	78	1	-0.115	0.042	4.250	2549	WS01024_E06
4	124.132	83	1	-0.434	0.084	4.262	2549	WS01024_E06
6	45.897	117	1	0.300	0.036	3.940	2549	WS01024_E06
8	62.580	155	1	0.480	0.099	5.100	2549	WS01024_E06
8	78.389	157	1	0.274	0.065	6.615	2549	WS01024_E06
8	87.463	158	1	-0.288	0.073	7.447	2549	WS01024_E06
1	82.301	16	4	0.282	0.047	4.617	2550	WS01024_E15
4	116.928	82	2	0.258	0.034	3.952	2550	WS01024_E15
5	52.820	99	3	-0.224	0.053	5.705	2550	WS01024_E15
6	45.897	117	4	-0.273	0.051	5.126	2550	WS01024_E15
6	69.073	121	3	0.304	0.040	3.929	2550	WS01024_E15
7	105.477	147	2	-0.346	0.072	4.051	2550	WS01024_E15
9	0.000	163	1	0.484	0.136	5.148	2550	WS01024_E15
9	58.045	176	1	-0.263	0.165	6.260	2550	WS01024_E15
9	59.963	177	1	-0.381	0.101	7.839	2550	WS01024_E15
9	69.046	181	1	0.427	0.127	4.956	2550	WS01024_E15
9	69.046	181	2	0.297	0.065	3.983	2550	WS01024_E15
9	81.073	183	1	-0.306	0.052	4.552	2550	WS01024_E15
10	21.864	188	3	0.171	0.043	4.705	2550	WS01024_E15
14	0.000	246	1	-0.259	0.071	5.332	2550	WS01024_E15
4	55.925	76	1	-0.207	0.057	4.935	2551	WS0047_F12
6	81.996	123	3	0.431	0.115	8.225	2551	WS0047_F12
8	50.001	154	1	-0.284	0.058	5.631	2551	WS0047_F12
9	0.000	163	1	0.408	0.057	4.036	2551	WS0047_F12
9	59.963	177	4	0.258	0.052	4.523	2551	WS0047_F12
10	70.246	190	2	-0.199	0.045	4.184	2551	WS0047_F12
10	93.347	196	1	0.428	0.063	4.508	2551	WS0047_F12
1	29.800	5	2	0.248	0.050	4.199	2552	WS0047_G03
1	38.326	6	2	-0.241	0.070	5.883	2552	WS0047_G03
5	7.672	92	1	-0.244	0.034	4.548	2552	WS0047_G03
6	25.914	114	1	-0.232	0.047	4.844	2552	WS0047_G03
6	76.376	122	1	-0.422	0.179	5.202	2552	WS0047_G03
6	89.401	125	1	0.321	0.049	5.122	2552	WS0047_G03
11	17.088	203	1	-0.273	0.064	6.655	2552	WS0047_G03
11	27.405	204	1	0.297	0.077	7.934	2552	WS0047_G03
11	29.069	205	1	0.202	0.048	4.902	2552	WS0047_G03
11	45.989	216	1	-0.268	0.071	7.319	2552	WS0047_G03
1	2.788	2	4	-0.223	0.043	4.865	2553	WS0047_J08
3	137.891	64	3	0.288	0.078	4.732	2553	WS0047_J08
4	173.929	88	2	-0.218	0.059	5.031	2553	WS0047_J08
6	81.996	123	3	0.295	0.084	4.029	2553	WS0047_J08

8	50.001	154	1	-0.224	0.044	4.563	2553	WS0047_J08
8	62.580	155	1	-0.281	0.091	4.881	2553	WS0047_J08
9	59.963	177	4	0.239	0.035	3.993	2553	WS0047_J08
1	107.333	19	4	-0.247	0.087	6.603	2554	WS0047_K03
1	123.316	20	4	-0.376	0.116	7.502	2554	WS0047_K03
1	127.805	21	4	0.325	0.096	8.601	2554	WS0047_K03
3	17.158	45	1	0.264	0.075	5.809	2554	WS0047_K03
5	57.017	102	2	-0.237	0.041	4.674	2554	WS0047_K03
11	27.405	204	4	-0.245	0.054	5.307	2554	WS0047_K03
1	95.572	17	2	0.199	0.044	4.233	2555	WS0047_M21
3	137.891	64	3	0.262	0.057	4.852	2555	WS0047_M21
4	0.000	71	4	0.212	0.045	4.216	2555	WS0047_M21
4	173.929	88	2	-0.237	0.057	5.479	2555	WS0047_M21
8	50.001	154	1	-0.284	0.068	6.979	2555	WS0047_M21
8	95.937	159	3	-0.217	0.043	4.084	2555	WS0047_M21
12	6.242	234	2	-0.211	0.038	4.292	2555	WS0047_M21
3	137.891	64	3	0.357	0.108	9.475	2556	WS0047_N18
4	173.929	88	2	-0.247	0.058	6.201	2556	WS0047_N18
5	57.017	102	4	-0.212	0.053	4.997	2556	WS0047_N18
6	81.996	123	3	0.320	0.068	4.148	2556	WS0047_N18
8	50.001	154	1	-0.257	0.061	5.546	2556	WS0047_N18
11	27.405	204	4	-0.330	0.078	7.559	2556	WS0047_N18
12	0.000	233	1	0.215	0.049	3.894	2556	WS0047_N18
12	6.242	234	2	-0.235	0.045	5.696	2556	WS0047_N18
1	38.326	6	4	0.236	0.030	4.142	2557	WS0048_A20
1	40.464	7	4	-0.259	0.031	4.154	2557	WS0048_A20
1	131.686	23	2	-0.406	0.042	4.713	2557	WS0048_A20
2	127.147	40	4	-0.196	0.038	4.942	2557	WS0048_A20
3	80.211	55	4	0.282	0.056	7.424	2557	WS0048_A20
3	104.681	60	2	0.197	0.028	3.882	2557	WS0048_A20
3	112.868	61	2	-0.141	0.028	3.858	2557	WS0048_A20
3	114.747	62	2	-0.235	0.033	4.612	2557	WS0048_A20
3	116.958	63	4	-0.194	0.041	5.454	2557	WS0048_A20
8	50.001	154	1	-0.250	0.091	4.918	2557	WS0048_A20
8	95.937	159	3	-0.198	0.075	4.097	2557	WS0048_A20
9	17.951	169	2	-0.277	0.055	7.109	2557	WS0048_A20
11	27.405	204	4	-0.331	0.082	10.614	2557	WS0048_A20
1	2.788	2	4	-0.185	0.037	3.946	2558	WS0048_B24
1	69.854	13	2	0.244	0.067	7.494	2558	WS0048_B24
1	71.592	14	2	0.246	0.069	7.672	2558	WS0048_B24
1	77.177	15	2	0.232	0.060	6.742	2558	WS0048_B24
1	95.572	17	2	0.290	0.056	6.222	2558	WS0048_B24
2	48.839	29	4	0.256	0.043	4.288	2558	WS0048_B24
3	69.071	52	1	-0.331	0.108	5.017	2558	WS0048_B24
4	0.000	71	4	-0.194	0.043	4.638	2558	WS0048_B24
6	69.073	121	4	-0.288	0.055	5.314	2558	WS0048_B24
8	50.001	154	1	-0.265	0.079	6.001	2558	WS0048_B24
14	0.000	246	2	-0.215	0.049	5.446	2558	WS0048_B24
1	77.177	15	2	0.161	0.031	3.899	2559	WS01024_O10
3	15.147	44	2	0.257	0.074	7.117	2559	WS01024_O10
4	142.917	85	1	0.261	0.066	4.075	2559	WS01024_O10
5	54.072	100	4	0.237	0.036	4.460	2559	WS01024_O10
5	57.017	102	4	-0.229	0.041	5.109	2559	WS01024_O10
6	56.188	120	2	-0.339	0.052	6.201	2559	WS01024_O10

8	87.463	158	2	-0.194	0.034	3.985	2559	WS01024_O10
9	17.951	169	2	-0.307	0.047	4.930	2559	WS01024_O10
10	70.246	190	2	-0.207	0.050	6.414	2559	WS01024_O10
10	104.214	198	4	-0.122	0.017	4.311	2559	WS01024_O10
11	43.491	214	4	-0.201	0.042	4.599	2559	WS01024_O10
11	64.014	222	4	-0.259	0.048	4.641	2559	WS01024_O10
1	69.854	13	2	0.187	0.036	4.526	2560	WS01024_O23
1	71.592	14	2	0.190	0.037	4.684	2560	WS01024_O23
1	77.177	15	2	0.193	0.038	4.803	2560	WS01024_O23
3	116.958	63	4	-0.161	0.038	4.691	2560	WS01024_O23
3	137.891	64	3	0.192	0.069	4.088	2560	WS01024_O23
6	87.891	124	2	-0.190	0.034	4.264	2560	WS01024_O23
8	50.001	154	1	-0.190	0.058	3.841	2560	WS01024_O23
10	70.246	190	2	-0.185	0.033	4.188	2560	WS01024_O23
11	64.014	222	4	-0.303	0.062	6.688	2560	WS01024_O23
12	6.242	234	2	-0.211	0.038	5.313	2560	WS01024_O23
14	0.000	246	2	-0.234	0.054	6.828	2560	WS01024_O23
4	55.925	76	1	-0.239	0.042	3.989	2561	WS01025_A12
4	173.929	88	2	-0.259	0.093	8.053	2561	WS01025_A12
5	7.672	92	1	-0.250	0.038	4.272	2561	WS01025_A12
5	16.219	93	1	-0.197	0.042	4.267	2561	WS01025_A12
11	27.405	204	1	0.307	0.069	7.077	2561	WS01025_A12
1	107.333	19	3	-0.226	0.041	4.572	2562	WS01025_B01
1	127.805	21	3	-0.172	0.042	4.642	2562	WS01025_B01
8	50.001	154	2	-0.314	0.084	11.227	2562	WS01025_B01
10	104.214	198	2	-0.274	0.044	6.593	2562	WS01025_B01
1	2.788	2	4	-0.189	0.042	4.198	2563	WS01025_D12
3	9.953	42	3	0.227	0.055	4.861	2563	WS01025_D12
3	80.211	55	4	0.179	0.040	3.932	2563	WS01025_D12
9	36.227	165	3	-0.245	0.048	4.302	2563	WS01025_D12
11	27.405	204	1	0.261	0.051	4.528	2563	WS01025_D12
11	27.405	204	4	-0.291	0.054	5.416	2563	WS01025_D12
3	15.147	44	2	0.279	0.070	3.951	2564	WS01025_E04
4	55.925	76	1	-0.305	0.078	6.771	2564	WS01025_E04
4	82.462	78	1	-0.172	0.049	4.482	2564	WS01025_E04
4	82.462	78	2	-0.232	0.070	6.656	2564	WS01025_E04
4	92.039	79	2	-0.305	0.082	7.852	2564	WS01025_E04
4	97.576	80	2	0.282	0.081	9.001	2564	WS01025_E04
6	25.914	114	1	0.210	0.046	4.232	2564	WS01025_E04
7	15.555	130	2	-0.293	0.074	7.086	2564	WS01025_E04
1	107.333	19	3	-0.223	0.045	4.798	2565	WS01025_G02
1	127.805	21	3	-0.185	0.041	4.375	2565	WS01025_G02
3	83.563	56	3	-0.222	0.038	4.066	2565	WS01025_G02
3	137.891	64	3	0.247	0.038	4.020	2565	WS01025_G02
4	142.917	85	3	-0.213	0.046	4.601	2565	WS01025_G02
4	173.929	88	2	-0.174	0.034	4.029	2565	WS01025_G02
7	15.555	130	2	-0.241	0.042	5.049	2565	WS01025_G02
9	36.227	165	3	-0.232	0.047	4.937	2565	WS01025_G02
1	45.794	9	1	0.383	0.072	6.457	2566	WS01025_G22
1	131.686	23	2	0.422	0.141	4.006	2566	WS01025_G22
1	133.992	24	2	0.257	0.094	9.310	2566	WS01025_G22
1	141.021	25	2	-0.163	0.045	4.458	2566	WS01025_G22
2	75.550	31	1	0.305	0.049	4.432	2566	WS01025_G22
4	6.032	72	4	0.220	0.043	3.932	2566	WS01025_G22

4	75.958	77	3	-0.300	0.061	4.249	2566	WS01025_G22
4	82.462	78	1	-0.315	0.071	6.379	2566	WS01025_G22
4	142.917	85	3	0.208	0.041	4.708	2566	WS01025_G22
6	54.066	119	4	-0.219	0.042	3.890	2566	WS01025_G22
6	76.376	122	1	-0.218	0.064	3.952	2566	WS01025_G22
6	87.891	124	2	0.149	0.042	4.113	2566	WS01025_G22
6	89.401	125	2	0.244	0.037	3.884	2566	WS01025_G22
9	3.127	168	1	-0.364	0.041	5.359	2566	WS01025_G22
11	41.984	212	2	0.250	0.045	4.580	2566	WS01025_G22
11	56.921	219	2	-0.224	0.056	4.544	2566	WS01025_G22
10	9.580	187	2	-0.238	0.057	4.998	2567	WS00931_F17
11	0.000	199	2	0.322	0.053	6.557	2567	WS00931_F17
11	0.000	199	3	-0.283	0.071	5.650	2567	WS00931_F17
11	27.405	204	1	0.261	0.068	4.667	2567	WS00931_F17
3	69.071	52	1	-0.268	0.051	4.866	2568	WS00931_J08
4	173.929	88	2	-0.232	0.058	4.503	2568	WS00931_J08
6	81.996	123	3	0.317	0.078	7.457	2568	WS00931_J08
8	50.001	154	1	-0.237	0.039	4.632	2568	WS00931_J08
11	64.014	222	3	0.176	0.034	3.943	2568	WS00931_J08
1	2.788	2	4	0.204	0.042	4.883	2569	WS01025_L12
1	95.572	17	2	-0.251	0.042	4.060	2569	WS01025_L12
4	55.925	76	1	0.207	0.050	4.456	2569	WS01025_L12
4	92.039	79	4	-0.360	0.079	8.118	2569	WS01025_L12
4	103.719	81	4	-0.245	0.051	5.117	2569	WS01025_L12
7	17.610	131	3	0.201	0.102	3.897	2569	WS01025_L12
7	62.787	139	4	-0.343	0.086	7.796	2569	WS01025_L12
10	95.795	197	4	0.228	0.039	4.078	2569	WS01025_L12
14	44.898	251	1	0.244	0.049	4.670	2569	WS01025_L12
1	2.788	2	4	-0.268	0.055	5.871	2570	WS01026_A06
3	137.891	64	3	0.256	0.070	5.213	2570	WS01026_A06
4	142.917	85	3	-0.221	0.059	4.764	2570	WS01026_A06
4	173.929	88	2	-0.249	0.056	6.332	2570	WS01026_A06
6	81.996	123	3	0.288	0.075	4.139	2570	WS01026_A06
8	50.001	154	1	-0.283	0.080	6.420	2570	WS01026_A06
1	2.788	2	4	-0.270	0.066	5.661	2571	WS0048_E10
4	55.925	76	1	-0.257	0.061	4.980	2571	WS0048_E10
4	142.917	85	3	-0.219	0.059	4.887	2571	WS0048_E10
6	81.996	123	3	0.304	0.083	4.115	2571	WS0048_E10
8	50.001	154	1	-0.254	0.067	5.898	2571	WS0048_E10
10	78.479	193	3	0.353	0.057	5.218	2571	WS0048_E10
1	45.794	9	1	0.265	0.041	3.966	2572	WS0048_F08
1	45.794	9	3	-0.266	0.080	7.857	2572	WS0048_F08
1	127.805	21	3	-0.138	0.047	4.484	2572	WS0048_F08
3	9.953	42	4	-0.264	0.075	7.907	2572	WS0048_F08
3	116.958	63	4	0.227	0.055	5.137	2572	WS0048_F08
5	3.484	90	2	0.291	0.102	5.286	2572	WS0048_F08
7	74.560	143	1	-0.194	0.055	5.347	2572	WS0048_F08
9	28.608	170	3	0.279	0.043	4.181	2572	WS0048_F08
9	69.046	181	2	-0.192	0.095	4.098	2572	WS0048_F08
10	35.204	189	1	-0.313	0.049	4.863	2572	WS0048_F08
1	82.301	16	4	0.206	0.035	4.673	2573	WS0048_J05
4	0.000	71	4	0.271	0.042	5.544	2573	WS0048_J05
9	28.608	170	3	0.230	0.070	4.039	2573	WS0048_J05
11	27.405	204	4	-0.340	0.082	7.765	2573	WS0048_J05

12	6.242	234	2	-0.184	0.025	3.921	2573	WS0048_J05
1	40.464	7	3	0.319	0.045	5.468	2574	WS0048_J24
1	43.578	8	3	0.330	0.053	5.956	2574	WS0048_J24
6	81.996	123	3	0.399	0.095	9.815	2574	WS0048_J24
8	50.001	154	1	-0.269	0.055	6.660	2574	WS0048_J24
10	93.347	196	1	0.587	0.102	10.556	2574	WS0048_J24
12	0.000	226	3	0.243	0.033	4.027	2574	WS0048_J24
1	29.800	5	1	-0.290	0.090	10.517	2575	WS0048_N16
3	141.443	65	3	-0.384	0.053	5.496	2575	WS0048_N16
6	81.996	123	3	0.429	0.056	5.859	2575	WS0048_N16
9	0.000	163	1	0.532	0.061	6.264	2575	WS0048_N16
11	17.088	203	1	0.268	0.039	4.757	2575	WS0048_N16
3	141.443	65	3	-0.410	0.108	6.657	2576	WS0048_O09
3	148.745	68	1	-0.293	0.044	3.954	2576	WS0048_O09
3	148.745	68	4	0.252	0.039	4.222	2576	WS0048_O09
4	0.000	71	4	-0.194	0.037	3.881	2576	WS0048_O09
6	69.073	121	4	-0.275	0.040	4.219	2576	WS0048_O09
9	55.358	175	3	0.423	0.077	3.936	2576	WS0048_O09
2	120.617	38	4	0.375	0.083	9.534	2577	WS0071_A22
4	103.719	81	4	0.205	0.038	4.045	2577	WS0071_A22
4	116.928	82	4	-0.278	0.048	5.374	2577	WS0071_A22
6	76.376	122	1	-0.401	0.119	7.330	2577	WS0071_A22
6	89.401	125	1	0.318	0.061	5.942	2577	WS0071_A22
6	95.249	126	1	-0.285	0.115	3.974	2577	WS0071_A22
8	62.580	155	1	-0.379	0.084	5.236	2577	WS0071_A22
8	78.389	157	1	-0.255	0.075	7.183	2577	WS0071_A22
8	87.463	158	1	0.262	0.080	7.671	2577	WS0071_A22
11	0.000	199	3	-0.241	0.067	4.706	2577	WS0071_A22
11	45.989	216	2	-0.210	0.057	5.205	2577	WS0071_A22
11	60.464	221	2	-0.246	0.045	4.065	2577	WS0071_A22
12	0.000	226	3	0.278	0.085	6.355	2577	WS0071_A22
12	22.441	227	1	0.432	0.078	7.548	2577	WS0071_A22
12	14.406	230	4	0.291	0.043	4.488	2577	WS0071_A22
13	0.000	243	4	-0.309	0.069	4.253	2577	WS0071_A22
2	126.181	39	4	0.464	0.059	6.345	2578	WS0071_B13
7	15.555	130	3	-0.263	0.072	5.211	2578	WS0071_B13
7	105.477	147	2	-0.186	0.042	4.469	2578	WS0071_B13
11	43.491	214	4	-0.255	0.057	6.516	2578	WS0071_B13
11	57.661	220	4	0.243	0.070	8.481	2578	WS0071_B13
11	64.014	222	4	-0.290	0.072	8.562	2578	WS0071_B13
1	45.794	9	3	-0.337	0.083	7.904	2579	WS00917_D11
7	67.154	140	4	-0.300	0.183	4.753	2579	WS00917_D11
7	75.883	144	2	0.436	0.077	5.384	2579	WS00917_D11
7	75.883	144	4	-0.365	0.069	6.473	2579	WS00917_D11
9	32.356	171	2	-0.206	0.034	3.974	2579	WS00917_D11
9	49.013	173	2	0.266	0.071	8.426	2579	WS00917_D11
11	60.464	221	2	0.235	0.043	5.106	2579	WS00917_D11
8	78.389	157	1	-0.182	0.034	3.994	2580	WS00917_D24
8	95.937	159	3	-0.202	0.048	5.363	2580	WS00917_D24
10	21.864	188	1	-0.287	0.043	5.000	2580	WS00917_D24
11	0.000	199	3	-0.330	0.077	7.104	2580	WS00917_D24
11	27.405	204	1	0.279	0.066	7.799	2580	WS00917_D24
11	29.069	205	1	0.182	0.035	4.061	2580	WS00917_D24
11	45.989	216	1	-0.241	0.046	5.393	2580	WS00917_D24

11	27.405	204	1	0.219	0.058	4.190	2581	WS00917_G05
11	27.405	204	4	-0.247	0.046	4.642	2581	WS00917_G05
12	0.000	233	1	0.235	0.058	4.134	2581	WS00917_G05
1	62.128	11	1	-0.536	0.145	5.565	2582	WS00917_G15
2	101.076	35	3	0.198	0.039	4.043	2582	WS00917_G15
7	75.883	144	2	-0.394	0.081	3.888	2582	WS00917_G15
11	46.026	217	1	0.204	0.054	4.530	2582	WS00917_G15
12	38.543	232	4	0.283	0.106	4.050	2582	WS00917_G15
13	0.000	243	4	0.400	0.130	4.993	2582	WS00917_G15
1	107.333	19	4	-0.233	0.084	4.943	2583	WS00917_I17
2	126.181	39	4	-0.490	0.144	5.908	2583	WS00917_I17
3	137.891	64	3	-0.426	0.118	10.803	2583	WS00917_I17
5	0.000	89	1	-0.325	0.090	6.402	2583	WS00917_I17
5	16.219	93	1	-0.285	0.096	6.639	2583	WS00917_I17
9	69.046	181	2	-0.260	0.061	4.001	2583	WS00917_I17
10	86.151	195	3	0.281	0.058	6.765	2583	WS00917_I17
11	9.237	202	4	-0.248	0.090	6.466	2583	WS00917_I17
11	43.491	214	3	0.263	0.053	6.135	2583	WS00917_I17
11	44.884	215	3	-0.242	0.039	4.494	2583	WS00917_I17
11	48.979	218	2	0.213	0.037	4.461	2583	WS00917_I17
11	56.921	219	2	-0.280	0.061	4.449	2583	WS00917_I17
11	64.014	222	3	-0.262	0.042	4.862	2583	WS00917_I17
13	1.815	244	4	0.191	0.056	4.015	2583	WS00917_I17
3	20.512	46	2	0.177	0.048	3.951	2584	WS00917_J08
3	47.928	49	1	0.278	0.062	6.485	2584	WS00917_J08
3	95.157	59	2	-0.207	0.051	4.581	2584	WS00917_J08
7	17.610	131	3	0.261	0.108	4.266	2584	WS00917_J08
7	62.787	139	4	0.209	0.042	4.812	2584	WS00917_J08
7	75.883	144	2	-0.407	0.093	4.369	2584	WS00917_J08
7	75.883	144	4	0.272	0.042	4.014	2584	WS00917_J08
11	27.405	204	1	0.218	0.039	4.148	2584	WS00917_J08
11	35.604	207	3	-0.224	0.046	4.333	2584	WS00917_J08
11	40.960	210	2	0.408	0.099	4.646	2584	WS00917_J08
13	1.815	244	4	0.278	0.086	7.852	2584	WS00917_J08
1	40.464	7	3	0.300	0.040	3.863	2585	WS00917_K24
2	126.181	39	2	-0.234	0.039	3.875	2585	WS00917_K24
2	127.147	40	3	-0.233	0.045	4.167	2585	WS00917_K24
3	9.953	42	4	-0.256	0.059	4.667	2585	WS00917_K24
4	43.146	74	3	0.222	0.047	4.380	2585	WS00917_K24
4	124.132	83	1	-0.507	0.147	5.136	2585	WS00917_K24
5	16.219	93	2	0.260	0.058	4.822	2585	WS00917_K24
6	69.073	121	3	0.250	0.061	5.674	2585	WS00917_K24
8	78.389	157	1	-0.196	0.045	3.967	2585	WS00917_K24
11	46.026	217	1	0.237	0.057	5.068	2585	WS00917_K24
1	43.578	8	2	-0.264	0.050	6.100	2586	WS00917_L15
1	43.578	8	3	0.251	0.056	4.789	2586	WS00917_L15
2	48.839	29	4	0.362	0.085	8.993	2586	WS00917_L15
6	54.066	119	4	-0.251	0.044	4.703	2586	WS00917_L15
6	69.073	121	3	0.179	0.050	4.203	2586	WS00917_L15
4	142.917	85	3	-0.257	0.046	4.507	2587	WS01026_P05
7	74.560	143	1	0.237	0.052	4.245	2587	WS01026_P05
8	87.463	158	2	-0.213	0.046	4.585	2587	WS01026_P05
8	95.937	159	3	-0.199	0.045	4.348	2587	WS01026_P05
1	77.177	15	2	0.239	0.055	5.066	2588	WS00912_F02

5	7.672	92	1	-0.236	0.035	3.944	2588	WS00912_F02
8	11.974	150	1	0.182	0.045	4.339	2588	WS00912_F02
9	17.951	169	2	-0.256	0.049	4.215	2588	WS00912_F02
12	22.629	237	1	0.163	0.042	3.983	2588	WS00912_F02
14	22.402	248	4	0.177	0.049	3.894	2588	WS00912_F02
1	29.800	5	2	0.236	0.035	4.899	2589	WS00819_E16
1	38.326	6	2	-0.269	0.038	5.343	2589	WS00819_E16
1	40.464	7	3	0.229	0.035	4.981	2589	WS00819_E16
1	43.578	8	3	0.228	0.035	4.988	2589	WS00819_E16
1	62.128	11	2	-0.210	0.037	3.917	2589	WS00819_E16
6	56.188	120	2	-0.268	0.048	6.442	2589	WS00819_E16
6	106.702	128	2	0.390	0.046	4.881	2589	WS00819_E16
10	78.479	193	3	0.261	0.045	6.188	2589	WS00819_E16
9	0.000	166	1	-0.146	0.040	3.922	2590	WS00912_J24
9	28.608	170	3	0.388	0.086	6.924	2590	WS00912_J24
10	78.479	193	4	-0.182	0.052	4.459	2590	WS00912_J24
11	45.989	216	1	-0.159	0.042	4.179	2590	WS00912_J24
11	56.921	219	1	-0.328	0.149	4.831	2590	WS00912_J24
14	0.000	246	1	0.277	0.052	5.209	2590	WS00912_J24
3	9.953	42	3	0.331	0.064	6.463	2591	WS00829_K23
3	137.891	64	3	0.169	0.043	4.307	2591	WS00829_K23
4	173.929	88	2	-0.232	0.039	4.066	2591	WS00829_K23
6	81.996	123	3	0.356	0.071	4.629	2591	WS00829_K23
8	87.463	158	2	-0.159	0.045	4.562	2591	WS00829_K23
9	59.963	177	4	0.255	0.048	3.992	2591	WS00829_K23
10	9.580	187	3	0.344	0.065	4.225	2591	WS00829_K23
1	95.572	17	2	0.225	0.053	5.184	2592	WS00830_F08
3	15.147	44	2	0.368	0.091	6.040	2592	WS00830_F08
4	55.925	76	1	-0.195	0.053	4.121	2592	WS00830_F08
4	82.462	78	1	-0.184	0.052	4.583	2592	WS00830_F08
6	56.188	120	2	-0.273	0.054	5.046	2592	WS00830_F08
9	59.963	177	4	0.330	0.069	7.322	2592	WS00830_F08
12	14.406	230	4	0.238	0.035	4.143	2592	WS00830_F08
2	126.181	39	4	-0.471	0.080	6.173	2593	WS00931_N21
3	9.953	42	3	0.277	0.091	7.560	2593	WS00931_N21
3	137.891	64	3	0.292	0.055	4.750	2593	WS00931_N21
11	27.405	204	4	-0.273	0.068	6.178	2593	WS00931_N21
12	6.242	234	2	-0.270	0.035	5.876	2593	WS00931_N21
1	29.800	5	1	-0.260	0.046	4.731	2594	WS01029_C16
1	127.805	21	4	-0.235	0.071	5.668	2594	WS01029_C16
3	9.953	42	3	0.289	0.051	5.657	2594	WS01029_C16
7	70.278	141	3	-0.193	0.046	4.970	2594	WS01029_C16
8	50.001	154	1	-0.247	0.038	4.419	2594	WS01029_C16
9	17.951	169	2	-0.254	0.064	5.338	2594	WS01029_C16
11	27.405	204	4	-0.245	0.057	4.277	2594	WS01029_C16
12	14.406	230	4	0.266	0.065	4.542	2594	WS01029_C16
14	44.898	251	1	-0.265	0.046	4.764	2594	WS01029_C16
1	62.128	11	1	-0.547	0.114	7.209	2595	WS00913_K16
4	142.917	85	1	0.279	0.041	3.941	2595	WS00913_K16
4	142.917	85	3	-0.269	0.073	4.322	2595	WS00913_K16
4	173.929	88	2	-0.233	0.062	5.665	2595	WS00913_K16
9	2.011	167	4	-0.266	0.047	6.345	2595	WS00913_K16
12	38.543	232	4	0.335	0.090	5.701	2595	WS00913_K16
14	22.402	248	4	0.404	0.091	11.642	2595	WS00913_K16

1	95.572	17	2	0.355	0.090	9.440	2596	WS00914_A22
1	95.572	17	3	-0.239	0.047	4.534	2596	WS00914_A22
2	127.147	40	4	-0.272	0.065	5.018	2596	WS00914_A22
3	9.953	42	3	0.245	0.049	4.667	2596	WS00914_A22
12	0.000	233	1	0.233	0.055	4.722	2596	WS00914_A22
1	95.572	17	2	0.228	0.055	4.873	2597	WS00934_G15
2	75.550	31	1	0.279	0.045	4.438	2597	WS00934_G15
6	81.996	123	4	-0.267	0.114	3.957	2597	WS00934_G15
11	60.464	221	1	0.291	0.064	6.279	2597	WS00934_G15
12	6.242	234	2	-0.209	0.067	7.257	2597	WS00934_G15
12	22.629	237	1	-0.186	0.060	5.813	2597	WS00934_G15
1	22.419	4	3	0.263	0.061	3.931	2598	WS00934_H02
1	95.572	17	2	0.240	0.042	4.201	2598	WS00934_H02
3	9.953	42	3	0.270	0.061	6.677	2598	WS00934_H02
5	7.672	92	1	-0.244	0.037	4.219	2598	WS00934_H02
7	15.555	130	2	-0.274	0.056	5.644	2598	WS00934_H02
10	9.580	187	3	0.260	0.060	3.965	2598	WS00934_H02
11	27.405	204	1	0.241	0.052	4.577	2598	WS00934_H02
12	0.000	233	1	0.215	0.057	4.948	2598	WS00934_H02
2	48.839	29	1	-0.263	0.057	4.048	2599	WS00934_K08
2	127.147	40	3	0.235	0.059	5.342	2599	WS00934_K08
6	106.702	128	2	-0.500	0.123	6.158	2599	WS00934_K08
10	35.204	189	1	-0.282	0.077	5.540	2599	WS00934_K08
14	22.402	248	4	-0.424	0.095	8.973	2599	WS00934_K08
1	107.333	19	3	-0.235	0.050	5.260	2600	WS00934_L08
1	127.805	21	3	-0.235	0.039	4.073	2600	WS00934_L08
2	127.147	40	4	-0.186	0.049	4.163	2600	WS00934_L08
3	137.891	64	3	0.222	0.044	4.358	2600	WS00934_L08
4	140.962	84	3	-0.268	0.053	5.533	2600	WS00934_L08
5	3.484	90	2	-0.347	0.133	7.468	2600	WS00934_L08
8	62.580	155	1	-0.311	0.190	5.200	2600	WS00934_L08
1	22.419	4	4	0.428	0.127	9.388	2601	WS00934_O14
1	131.686	23	1	0.508	0.061	4.456	2601	WS00934_O14
3	9.953	42	4	0.337	0.098	10.616	2601	WS00934_O14
4	142.917	85	1	0.239	0.039	4.663	2601	WS00934_O14
6	45.897	117	1	0.301	0.046	5.472	2601	WS00934_O14
9	36.227	165	4	-0.259	0.044	4.631	2601	WS00934_O14
10	77.676	192	1	-0.377	0.082	9.933	2601	WS00934_O14
10	80.088	194	1	-0.273	0.032	3.924	2601	WS00934_O14
11	17.088	203	1	0.314	0.066	7.899	2601	WS00934_O14
11	43.491	214	3	-0.224	0.088	6.640	2601	WS00934_O14
11	44.884	215	3	0.216	0.065	4.935	2601	WS00934_O14
11	56.921	219	1	0.588	0.234	17.032	2601	WS00934_O14
12	6.242	234	4	0.436	0.110	9.090	2601	WS00934_O14
14	46.637	252	4	-0.278	0.048	5.701	2601	WS00934_O14
1	2.788	2	4	-0.206	0.042	3.916	2602	WS00934_P08
7	15.555	130	2	-0.248	0.047	4.844	2602	WS00934_P08
9	36.227	165	3	-0.205	0.061	5.082	2602	WS00934_P08
9	59.963	177	4	0.229	0.040	4.265	2602	WS00934_P08
12	6.242	234	2	-0.194	0.042	5.242	2602	WS00934_P08
1	43.578	8	2	-0.327	0.073	9.901	2603	WS00935_D01
1	95.572	17	2	0.319	0.065	8.711	2603	WS00935_D01
2	127.147	40	4	-0.234	0.038	5.129	2603	WS00935_D01
3	15.147	44	2	0.238	0.054	5.714	2603	WS00935_D01

5	54.072	100	4	0.209	0.031	4.061	2603	WS00935_D01
7	73.169	142	2	0.438	0.047	4.918	2603	WS00935_D01
7	75.883	144	2	-0.398	0.042	4.557	2603	WS00935_D01
9	59.963	177	4	0.281	0.062	8.240	2603	WS00935_D01
10	9.580	187	2	-0.287	0.064	6.912	2603	WS00935_D01
10	95.795	197	4	-0.226	0.032	4.190	2603	WS00935_D01
12	28.493	228	2	-0.231	0.046	4.923	2603	WS00935_D01
12	14.406	230	4	0.252	0.036	4.825	2603	WS00935_D01
12	6.242	234	2	-0.170	0.031	6.629	2603	WS00935_D01
14	43.392	250	2	-0.203	0.040	5.256	2603	WS00935_D01
1	15.589	3	2	-0.223	0.036	3.914	2604	WS00935_E12
1	29.800	5	1	-0.195	0.050	3.987	2604	WS00935_E12
1	95.572	17	2	0.270	0.047	5.048	2604	WS00935_E12
7	73.169	142	1	0.517	0.142	4.282	2604	WS00935_E12
9	36.227	165	3	-0.172	0.036	3.933	2604	WS00935_E12
10	70.246	190	2	-0.161	0.050	5.279	2604	WS00935_E12
10	70.246	190	4	0.281	0.089	6.934	2604	WS00935_E12
12	6.242	234	2	-0.136	0.041	5.215	2604	WS00935_E12
12	51.382	242	1	-0.421	0.166	5.063	2604	WS00935_E12
1	29.800	5	2	0.258	0.088	4.390	2605	WS00721_H18
1	38.326	6	2	-0.221	0.073	4.551	2605	WS00721_H18
3	144.593	66	4	-0.203	0.030	3.878	2605	WS00721_H18
3	148.745	68	4	0.141	0.026	3.905	2605	WS00721_H18
4	116.928	82	4	0.358	0.070	9.784	2605	WS00721_H18
6	45.897	117	4	-0.181	0.032	4.682	2605	WS00721_H18
8	78.389	157	1	-0.233	0.041	5.277	2605	WS00721_H18
8	87.463	158	1	0.225	0.039	4.901	2605	WS00721_H18
10	95.795	197	4	0.226	0.034	4.805	2605	WS00721_H18
12	38.543	232	4	0.354	0.071	7.146	2605	WS00721_H18
12	51.382	242	1	0.296	0.045	4.575	2605	WS00721_H18
2	63.261	30	3	0.186	0.045	4.644	2606	WS00721_I17
3	9.953	42	3	0.204	0.048	4.975	2606	WS00721_I17
5	3.484	90	2	-0.268	0.090	4.771	2606	WS00721_I17
7	15.555	130	3	0.289	0.069	7.222	2606	WS00721_I17
11	42.600	213	3	0.165	0.038	3.924	2606	WS00721_I17
12	0.000	233	1	0.163	0.046	3.896	2606	WS00721_I17
1	45.794	9	3	-0.234	0.046	5.166	2607	WS00721_M07
6	106.702	128	2	0.408	0.089	4.279	2607	WS00721_M07
7	17.610	131	3	0.351	0.126	6.078	2607	WS00721_M07
9	28.608	170	3	0.302	0.058	6.326	2607	WS00721_M07
11	9.237	202	4	0.242	0.066	5.367	2607	WS00721_M07
11	45.989	216	1	-0.257	0.057	5.240	2607	WS00721_M07
12	38.543	232	4	0.238	0.114	3.899	2607	WS00721_M07
14	22.402	248	4	0.336	0.054	4.034	2607	WS00721_M07
4	0.000	71	4	-0.205	0.039	3.859	2608	WS00721_O07
4	103.719	81	4	-0.263	0.064	6.506	2608	WS00721_O07
10	70.246	190	2	0.280	0.054	5.714	2608	WS00721_O07
11	64.014	222	4	-0.302	0.056	5.437	2608	WS00721_O07
13	0.000	243	4	0.466	0.192	9.317	2608	WS00721_O07
13	3.386	245	1	-0.243	0.055	4.532	2608	WS00721_O07
1	69.854	13	2	0.158	0.045	4.358	2609	WS00722_C06
1	71.592	14	2	0.151	0.042	4.055	2609	WS00722_C06
1	77.177	15	2	0.150	0.042	4.026	2609	WS00722_C06
1	95.572	17	2	0.351	0.077	7.496	2609	WS00722_C06

3	15.147	44	2	0.216	0.093	4.822	2609	WS00722_C06
4	142.917	85	3	-0.275	0.037	4.099	2609	WS00722_C06
6	87.891	124	2	-0.263	0.057	5.561	2609	WS00722_C06
9	36.227	165	3	-0.279	0.042	4.487	2609	WS00722_C06
9	17.951	169	2	-0.248	0.043	4.178	2609	WS00722_C06
14	22.402	248	3	-0.275	0.044	4.730	2609	WS00722_C06
2	63.261	30	3	0.228	0.047	6.011	2610	WS00722_D13
2	95.880	34	3	0.231	0.035	4.337	2610	WS00722_D13
5	0.000	89	1	-0.282	0.048	5.572	2610	WS00722_D13
5	42.979	97	1	-0.280	0.038	4.340	2610	WS00722_D13
5	52.820	99	3	-0.317	0.049	6.335	2610	WS00722_D13
1	29.800	5	2	-0.220	0.039	4.122	2611	WS00722_H16
2	126.181	39	4	-0.460	0.084	5.632	2611	WS00722_H16
5	42.979	97	2	0.222	0.045	4.797	2611	WS00722_H16
11	27.405	204	1	-0.279	0.077	6.523	2611	WS00722_H16
11	29.069	205	1	-0.201	0.059	4.961	2611	WS00722_H16
11	43.491	214	4	0.350	0.110	9.953	2611	WS00722_H16
11	44.884	215	4	-0.318	0.065	6.048	2611	WS00722_H16
11	45.989	216	1	0.258	0.076	6.379	2611	WS00722_H16
11	48.979	218	4	0.310	0.063	6.141	2611	WS00722_H16
13	1.815	244	2	0.193	0.039	4.155	2611	WS00722_H16
1	62.128	11	1	-0.469	0.112	4.275	2612	WS00722_J06
3	112.868	61	2	-0.227	0.037	3.867	2612	WS00722_J06
3	114.747	62	2	-0.144	0.039	4.041	2612	WS00722_J06
9	17.951	169	2	-0.197	0.041	4.783	2612	WS00722_J06
9	59.963	177	4	0.251	0.044	4.185	2612	WS00722_J06
10	9.580	187	3	0.347	0.097	4.330	2612	WS00722_J06
11	64.014	222	4	-0.271	0.062	5.181	2612	WS00722_J06
13	0.000	243	4	0.235	0.114	4.337	2612	WS00722_J06
1	82.301	16	4	0.253	0.045	4.350	2613	WS00822_B11
2	120.617	38	3	-0.209	0.043	3.896	2613	WS00822_B11
3	47.928	49	1	-0.235	0.050	4.441	2613	WS00822_B11
6	81.996	123	3	0.306	0.071	3.867	2613	WS00822_B11
8	50.001	154	1	-0.237	0.050	4.547	2613	WS00822_B11
9	59.963	177	4	0.285	0.059	5.788	2613	WS00822_B11
10	78.479	193	3	0.302	0.046	4.177	2613	WS00822_B11
12	6.242	234	2	-0.192	0.031	4.121	2613	WS00822_B11
14	22.402	248	4	0.219	0.044	4.584	2613	WS00822_B11
1	95.572	17	2	-0.300	0.062	7.181	2614	WS00822_C12
1	123.316	20	4	-0.328	0.091	5.039	2614	WS00822_C12
1	127.805	21	4	0.362	0.138	12.288	2614	WS00822_C12
1	133.992	24	2	-0.218	0.053	5.955	2614	WS00822_C12
2	101.076	35	4	0.245	0.032	4.047	2614	WS00822_C12
2	102.814	36	2	-0.175	0.035	4.006	2614	WS00822_C12
3	116.958	63	4	-0.204	0.050	4.328	2614	WS00822_C12
5	52.820	99	1	0.207	0.063	4.665	2614	WS00822_C12
5	56.008	101	1	-0.293	0.093	7.001	2614	WS00822_C12
7	15.555	130	2	0.271	0.054	6.253	2614	WS00822_C12
7	17.610	131	3	0.272	0.108	6.279	2614	WS00822_C12
11	46.026	217	1	-0.190	0.055	4.119	2614	WS00822_C12
12	0.000	233	1	-0.249	0.059	4.432	2614	WS00822_C12
14	44.898	251	1	0.314	0.059	4.517	2614	WS00822_C12
1	62.128	11	1	-0.574	0.122	7.164	2615	WS00822_D22
1	69.854	13	2	0.170	0.045	4.280	2615	WS00822_D22

1	77.177	15	2	0.169	0.044	4.225	2615	WS00822_D22
1	95.572	17	2	0.205	0.044	4.168	2615	WS00822_D22
3	9.953	42	3	0.270	0.073	6.294	2615	WS00822_D22
8	50.001	154	1	-0.245	0.049	4.569	2615	WS00822_D22
14	22.402	248	4	0.340	0.071	7.296	2615	WS00822_D22
3	116.958	63	4	-0.235	0.050	5.204	2616	WS00822_E20
6	56.188	120	2	-0.226	0.037	4.846	2616	WS00822_E20
6	81.996	123	3	0.341	0.078	4.017	2616	WS00822_E20
11	64.014	222	4	-0.355	0.081	8.937	2616	WS00822_E20
4	0.000	71	4	0.131	0.052	4.189	2617	WS00822_H12
4	55.925	76	1	-0.278	0.127	13.247	2617	WS00822_H12
4	82.462	78	1	-0.133	0.049	5.260	2617	WS00822_H12
9	0.000	163	1	0.450	0.058	5.107	2617	WS00822_H12
9	28.608	170	2	0.289	0.047	4.269	2617	WS00822_H12
9	28.608	170	3	-0.402	0.094	10.628	2617	WS00822_H12
9	59.963	177	1	-0.285	0.037	4.534	2617	WS00822_H12
10	93.347	196	1	-0.502	0.076	6.687	2617	WS00822_H12
12	0.000	233	1	0.354	0.068	7.559	2617	WS00822_H12
1	133.992	24	2	0.206	0.044	3.959	2618	WS00822_I06
3	9.953	42	3	0.308	0.078	8.646	2618	WS00822_I06
7	91.487	146	3	-0.196	0.039	4.351	2618	WS00822_I06
2	127.147	40	4	-0.178	0.041	4.154	2619	WS00822_K13
4	55.925	76	1	-0.268	0.080	7.341	2619	WS00822_K13
4	82.462	78	1	-0.211	0.058	4.524	2620	WS00822_L06
7	15.555	130	3	0.185	0.050	4.171	2620	WS00822_L06
8	87.463	158	2	-0.205	0.050	5.575	2620	WS00822_L06
12	28.493	228	2	-0.242	0.052	4.433	2620	WS00822_L06
14	22.402	248	3	0.268	0.054	4.373	2620	WS00822_L06
3	14.500	43	1	0.335	0.053	5.681	2621	WS00911_K05
6	25.914	114	1	-0.256	0.047	5.020	2621	WS00911_K05
9	28.608	170	3	0.432	0.125	9.315	2621	WS00911_K05
11	17.088	203	1	-0.367	0.099	10.446	2621	WS00911_K05
11	27.405	204	1	0.352	0.071	7.756	2621	WS00911_K05
11	29.069	205	1	0.333	0.067	7.123	2621	WS00911_K05
11	45.989	216	1	-0.216	0.052	5.459	2621	WS00911_K05
11	46.026	217	1	-0.263	0.063	6.648	2621	WS00911_K05
11	56.921	219	1	-0.284	0.099	5.611	2621	WS00911_K05
11	57.661	220	4	0.228	0.045	4.469	2621	WS00911_K05
11	60.464	221	1	0.206	0.038	3.972	2621	WS00911_K05
11	60.464	221	2	0.240	0.077	6.356	2621	WS00911_K05
12	0.000	233	1	-0.222	0.039	4.117	2621	WS00911_K05
13	1.815	244	4	-0.197	0.055	5.655	2621	WS00911_K05
14	0.000	246	1	0.244	0.041	4.385	2621	WS00911_K05
1	62.128	11	2	0.300	0.092	4.569	2622	WS00911_P14
1	69.854	13	2	0.240	0.051	6.110	2622	WS00911_P14
1	71.592	14	2	0.251	0.057	6.796	2622	WS00911_P14
1	77.177	15	2	0.303	0.084	9.937	2622	WS00911_P14
2	126.181	39	4	0.508	0.109	7.161	2622	WS00911_P14
2	127.147	40	3	0.192	0.042	3.935	2622	WS00911_P14
3	9.953	42	3	-0.207	0.044	4.119	2622	WS00911_P14
3	137.891	64	3	-0.280	0.066	6.238	2622	WS00911_P14
3	144.593	66	4	-0.245	0.061	5.318	2622	WS00911_P14
3	154.505	69	4	-0.244	0.062	4.254	2622	WS00911_P14
6	54.066	119	4	0.276	0.071	6.769	2622	WS00911_P14

7	75.883	144	2	-0.431	0.069	4.864	2622	WS00911_P14
7	75.883	144	4	0.293	0.045	4.331	2622	WS00911_P14
1	22.419	4	3	-0.300	0.065	4.038	2623	WS00828_D01
3	154.505	69	1	0.199	0.046	4.513	2623	WS00828_D01
4	55.925	76	1	0.331	0.093	8.571	2623	WS00828_D01
4	173.929	88	2	0.217	0.055	5.059	2623	WS00828_D01
5	52.820	99	3	-0.175	0.043	4.402	2623	WS00828_D01
6	56.188	120	1	0.256	0.052	4.972	2623	WS00828_D01
9	58.045	176	1	-0.257	0.066	4.104	2623	WS00828_D01
9	59.963	177	1	-0.237	0.040	3.880	2623	WS00828_D01
9	69.046	181	1	0.283	0.063	3.907	2623	WS00828_D01
11	27.405	204	1	-0.292	0.055	5.309	2623	WS00828_D01
12	0.000	233	1	-0.232	0.054	5.134	2623	WS00828_D01
12	6.242	234	2	0.211	0.049	4.692	2623	WS00828_D01
12	22.629	237	1	-0.217	0.044	4.224	2623	WS00828_D01
1	22.419	4	3	-0.430	0.103	7.274	2624	WS01031_E06
2	95.880	34	3	0.320	0.069	7.141	2624	WS01031_E06
2	101.076	35	3	-0.231	0.038	4.403	2624	WS01031_E06
5	52.820	99	3	-0.248	0.039	4.004	2624	WS01031_E06
9	69.046	181	2	0.288	0.105	5.257	2624	WS01031_E06
14	0.000	246	1	0.172	0.038	4.123	2624	WS01031_E06
6	87.891	124	2	-0.221	0.063	6.934	2625	WS00829_A09
6	89.401	125	2	-0.342	0.073	7.343	2625	WS00829_A09
11	17.088	203	2	-0.187	0.042	4.227	2625	WS00829_A09
11	56.921	219	1	0.292	0.117	5.030	2625	WS00829_A09
1	2.788	2	4	-0.233	0.041	3.842	2626	WS01031_I07
1	95.572	17	2	0.258	0.077	5.940	2626	WS01031_I07
2	63.261	30	3	0.203	0.044	3.883	2626	WS01031_I07
3	9.953	42	3	0.311	0.081	7.276	2626	WS01031_I07
4	142.917	85	1	-0.218	0.036	3.943	2626	WS01031_I07
6	81.996	123	3	0.373	0.099	6.523	2626	WS01031_I07
8	50.001	154	1	-0.255	0.043	4.534	2626	WS01031_I07
12	0.000	233	1	0.227	0.035	3.870	2626	WS01031_I07
14	22.402	248	4	0.322	0.057	5.772	2626	WS01031_I07
1	82.301	16	4	0.251	0.034	3.851	2627	WS01036_I16
3	14.500	43	2	-0.286	0.037	4.695	2627	WS01036_I16
3	20.512	46	2	0.174	0.049	5.956	2627	WS01036_I16
4	124.132	83	1	-0.477	0.136	4.647	2627	WS01036_I16
5	16.219	93	2	-0.280	0.075	9.471	2627	WS01036_I16
6	26.350	115	2	-0.190	0.039	4.733	2627	WS01036_I16
6	106.702	128	2	-0.474	0.131	5.755	2627	WS01036_I16
3	9.953	42	3	0.261	0.051	5.444	2628	WS01036_J16
3	15.147	44	2	0.297	0.065	3.997	2628	WS01036_J16
3	137.891	64	3	0.295	0.070	7.212	2628	WS01036_J16
4	142.917	85	3	-0.266	0.054	4.105	2628	WS01036_J16
4	173.929	88	2	-0.223	0.042	4.905	2628	WS01036_J16
6	81.996	123	3	0.335	0.082	4.207	2628	WS01036_J16
7	17.610	131	3	-0.333	0.110	5.903	2628	WS01036_J16
9	59.963	177	4	0.256	0.053	4.127	2628	WS01036_J16
10	78.479	193	3	0.259	0.038	3.922	2628	WS01036_J16
2	120.617	38	3	-0.282	0.064	5.136	2629	WS01036_N03
5	56.008	101	2	0.266	0.060	6.100	2629	WS01036_N03
6	25.914	114	1	0.212	0.048	4.153	2629	WS01036_N03
8	62.580	155	1	-0.339	0.090	4.192	2629	WS01036_N03

9	81.073	183	1	0.310	0.061	5.336	2629	WS01036_N03
10	35.204	189	1	-0.273	0.046	4.001	2629	WS01036_N03
11	0.000	199	2	0.318	0.059	6.067	2629	WS01036_N03
11	43.491	214	3	0.179	0.059	4.690	2629	WS01036_N03
11	44.884	215	3	-0.242	0.088	7.044	2629	WS01036_N03
11	64.014	222	3	-0.251	0.103	8.206	2629	WS01036_N03
14	22.402	248	3	0.206	0.049	3.919	2629	WS01036_N03
1	82.301	16	3	-0.262	0.044	6.440	2630	WS01036_O07
3	47.928	49	1	0.262	0.041	4.416	2630	WS01036_O07
4	75.958	77	3	-0.263	0.070	4.439	2630	WS01036_O07
4	92.039	79	2	-0.301	0.070	5.762	2630	WS01036_O07
5	0.000	89	1	-0.304	0.071	6.587	2630	WS01036_O07
5	0.000	89	3	-0.389	0.091	12.551	2630	WS01036_O07
5	7.672	92	1	-0.336	0.122	12.972	2630	WS01036_O07
5	16.219	93	1	-0.585	0.256	26.911	2630	WS01036_O07
5	31.191	95	1	-0.452	0.141	14.873	2630	WS01036_O07
6	25.742	113	3	-0.255	0.035	5.255	2630	WS01036_O07
6	49.100	118	2	-0.304	0.066	4.207	2630	WS01036_O07
10	78.479	193	3	0.279	0.028	4.220	2630	WS01036_O07
12	22.441	227	3	-0.278	0.036	5.286	2630	WS01036_O07
12	6.242	234	2	0.286	0.065	5.181	2630	WS01036_O07
14	0.000	246	1	0.261	0.043	4.614	2630	WS01036_O07
1	43.578	8	2	-0.245	0.057	6.098	2631	WS01037_B06
1	50.955	10	1	-0.238	0.075	6.092	2631	WS01037_B06
1	69.854	13	2	0.239	0.055	5.838	2631	WS01037_B06
1	71.592	14	2	0.243	0.058	6.159	2631	WS01037_B06
1	77.177	15	2	0.215	0.042	4.502	2631	WS01037_B06
1	95.572	17	2	0.265	0.037	3.980	2631	WS01037_B06
3	14.500	43	2	-0.251	0.043	4.555	2631	WS01037_B06
3	116.958	63	4	-0.160	0.040	4.288	2631	WS01037_B06
5	56.008	101	2	0.251	0.058	6.005	2631	WS01037_B06
9	17.951	169	2	-0.338	0.076	8.027	2631	WS01037_B06
13	3.386	245	1	-0.321	0.090	7.310	2631	WS01037_B06
14	43.392	250	2	-0.210	0.036	3.853	2631	WS01037_B06
3	9.953	42	3	0.286	0.093	8.293	2632	WS01037_C12
8	50.001	154	1	-0.218	0.087	5.331	2632	WS01037_C12
1	22.419	4	3	-0.357	0.100	6.802	2633	WS01037_H04
2	120.617	38	3	-0.195	0.029	4.075	2633	WS01037_H04
3	9.953	42	3	0.171	0.028	3.929	2633	WS01037_H04
3	83.563	56	3	0.157	0.034	4.700	2633	WS01037_H04
3	141.443	65	3	-0.258	0.057	3.862	2633	WS01037_H04
5	0.000	89	3	0.298	0.036	4.845	2633	WS01037_H04
6	56.188	120	1	0.225	0.045	4.063	2633	WS01037_H04
6	87.891	124	2	0.197	0.045	4.448	2633	WS01037_H04
8	11.974	150	1	0.315	0.084	7.549	2633	WS01037_H04
8	50.001	154	2	-0.241	0.074	6.224	2633	WS01037_H04
8	87.463	158	2	0.209	0.041	3.961	2633	WS01037_H04
10	78.479	193	3	-0.215	0.036	4.921	2633	WS01037_H04
11	43.491	214	3	0.264	0.040	5.648	2633	WS01037_H04
12	22.441	227	1	0.296	0.063	5.663	2633	WS01037_H04
1	43.578	8	2	0.227	0.065	5.856	2634	WS01037_H22
3	9.953	42	4	-0.170	0.058	5.126	2634	WS01037_H22
3	80.211	55	4	-0.242	0.064	5.665	2634	WS01037_H22
4	116.928	82	2	0.242	0.049	4.401	2634	WS01037_H22

4	124.132	83	2	0.292	0.130	5.053	2634	WS01037_H22
5	52.820	99	1	-0.256	0.065	6.411	2634	WS01037_H22
5	56.008	101	1	0.247	0.045	4.403	2634	WS01037_H22
6	69.073	121	3	-0.282	0.060	6.009	2634	WS01037_H22
7	75.883	144	2	0.436	0.092	5.068	2634	WS01037_H22
7	75.883	144	4	-0.315	0.054	4.900	2634	WS01037_H22
10	35.204	189	1	0.237	0.041	3.982	2634	WS01037_H22
11	17.088	203	1	-0.183	0.045	4.426	2634	WS01037_H22
14	46.637	252	3	-0.243	0.039	3.858	2634	WS01037_H22
1	107.333	19	4	-0.270	0.072	7.699	2635	WS01032_L24
2	101.076	35	3	-0.277	0.058	4.728	2635	WS01032_L24
3	14.500	43	2	0.229	0.038	3.906	2635	WS01032_L24
3	95.157	59	2	-0.227	0.065	6.911	2635	WS01032_L24
5	0.000	89	3	-0.236	0.057	4.581	2635	WS01032_L24
5	57.017	102	2	0.249	0.056	6.499	2635	WS01032_L24
5	86.669	106	2	0.357	0.136	5.952	2635	WS01032_L24
6	81.996	123	4	-0.325	0.137	4.451	2635	WS01032_L24
12	6.242	234	4	-0.296	0.042	3.921	2635	WS01032_L24
13	0.000	243	4	-0.376	0.170	6.370	2635	WS01032_L24
1	38.326	6	4	-0.270	0.044	4.204	2636	WS01032_M18
4	116.928	82	2	-0.227	0.038	4.365	2636	WS01032_M18
6	25.914	114	1	-0.279	0.058	4.063	2636	WS01032_M18
6	69.073	121	4	-0.214	0.050	4.456	2636	WS01032_M18
7	74.560	143	2	0.181	0.026	3.880	2636	WS01032_M18
9	28.608	170	3	0.393	0.082	3.905	2636	WS01032_M18
10	78.479	193	4	-0.224	0.063	5.126	2636	WS01032_M18
1	62.128	11	1	0.446	0.089	3.851	2637	WS01032_P14
3	9.953	42	3	-0.217	0.057	5.144	2637	WS01032_P14
3	17.158	45	1	-0.268	0.055	4.865	2637	WS01032_P14
4	0.000	71	4	0.209	0.065	6.358	2637	WS01032_P14
14	22.402	248	3	-0.291	0.081	7.243	2637	WS01032_P14
1	82.301	16	4	-0.239	0.045	4.145	2638	WS00826_A05
3	14.500	43	2	-0.312	0.071	7.973	2638	WS00826_A05
3	20.512	46	2	0.241	0.056	6.197	2638	WS00826_A05
4	103.719	81	4	0.234	0.047	4.801	2638	WS00826_A05
6	81.996	123	3	-0.313	0.095	4.137	2638	WS00826_A05
7	15.555	130	2	-0.282	0.061	6.733	2638	WS00826_A05
7	15.555	130	3	0.372	0.096	8.977	2638	WS00826_A05
11	35.604	207	3	0.210	0.044	3.907	2638	WS00826_A05
11	37.143	209	3	-0.258	0.067	6.013	2638	WS00826_A05
11	40.960	210	2	-0.411	0.071	4.830	2638	WS00826_A05
11	42.600	213	3	0.227	0.049	4.409	2638	WS00826_A05
11	45.989	216	2	0.214	0.047	4.362	2638	WS00826_A05
1	29.800	5	1	0.226	0.067	6.703	2639	WS00826_E12
3	80.211	55	4	0.145	0.045	3.997	2639	WS00826_E12
5	42.979	97	1	0.321	0.051	5.149	2639	WS00826_E12
5	86.669	106	4	0.327	0.142	5.978	2639	WS00826_E12
9	28.608	170	3	0.446	0.104	9.913	2639	WS00826_E12
9	49.013	173	1	0.256	0.042	4.897	2639	WS00826_E12
10	21.864	188	1	-0.270	0.054	5.306	2639	WS00826_E12
10	35.204	189	1	-0.396	0.066	6.812	2639	WS00826_E12
11	17.088	203	1	-0.334	0.062	6.402	2639	WS00826_E12
11	27.405	204	1	0.477	0.104	11.095	2639	WS00826_E12
11	27.405	204	4	-0.291	0.049	4.606	2639	WS00826_E12

11	29.069	205	1	0.352	0.072	7.389	2639	WS00826_E12
1	123.316	20	4	0.380	0.190	10.674	2640	WS00826_F20
1	131.686	23	2	-0.612	0.160	7.796	2640	WS00826_F20
4	124.132	83	1	-0.493	0.120	5.144	2640	WS00826_F20
5	57.017	102	4	0.316	0.052	5.032	2640	WS00826_F20
6	56.188	120	2	0.304	0.044	4.574	2640	WS00826_F20
7	105.477	147	2	0.331	0.154	8.588	2640	WS00826_F20
8	0.000	148	4	-0.248	0.040	4.053	2640	WS00826_F20
8	78.389	157	1	0.236	0.065	5.526	2640	WS00826_F20
8	87.463	158	1	-0.245	0.070	6.003	2640	WS00826_F20
9	59.963	177	4	-0.200	0.044	4.675	2640	WS00826_F20
11	29.069	205	1	-0.289	0.060	5.290	2640	WS00826_F20
11	40.960	210	2	0.423	0.093	5.718	2640	WS00826_F20
11	40.960	210	4	-0.322	0.043	4.363	2640	WS00826_F20
11	45.989	216	1	0.239	0.056	4.805	2640	WS00826_F20
13	1.815	244	4	0.292	0.103	10.949	2640	WS00826_F20
14	46.637	252	4	-0.305	0.041	4.269	2640	WS00826_F20
3	14.500	43	1	0.257	0.049	4.126	2641	WS00826_J02
3	17.158	45	1	-0.266	0.073	6.121	2641	WS00826_J02
6	26.350	115	2	-0.199	0.041	4.229	2641	WS00826_J02
8	95.937	159	3	-0.205	0.050	4.674	2641	WS00826_J02
10	35.204	189	2	-0.224	0.050	5.217	2641	WS00826_J02
10	70.246	190	2	0.320	0.077	7.931	2641	WS00826_J02
10	80.088	194	2	-0.268	0.036	4.275	2641	WS00826_J02
1	95.572	17	2	0.233	0.052	3.955	2642	WS00826_K10
3	83.563	56	3	-0.294	0.083	9.098	2642	WS00826_K10
4	82.462	78	1	-0.231	0.035	4.013	2642	WS00826_K10
4	140.962	84	3	-0.197	0.049	5.362	2642	WS00826_K10
5	7.672	92	1	-0.274	0.050	6.629	2642	WS00826_K10
5	16.219	93	1	-0.199	0.038	4.298	2642	WS00826_K10
6	25.742	113	3	-0.216	0.037	4.057	2642	WS00826_K10
6	81.996	123	3	0.296	0.094	7.480	2642	WS00826_K10
9	28.608	170	3	0.251	0.047	5.204	2642	WS00826_K10
10	9.580	187	3	0.260	0.071	5.885	2642	WS00826_K10
10	21.864	188	3	-0.234	0.037	3.912	2642	WS00826_K10
10	93.347	196	1	0.406	0.049	4.009	2642	WS00826_K10
12	0.000	233	1	0.193	0.046	5.135	2642	WS00826_K10
2	127.147	40	4	-0.237	0.069	6.270	2643	WS01010_F09
3	15.147	44	2	0.286	0.081	5.361	2643	WS01010_F09
5	7.672	92	1	-0.293	0.040	4.352	2643	WS01010_F09
7	15.555	130	2	-0.251	0.049	4.655	2643	WS01010_F09
7	15.555	130	3	0.192	0.046	4.508	2643	WS01010_F09
9	36.227	165	3	-0.216	0.058	5.644	2643	WS01010_F09
11	0.000	199	2	0.263	0.046	4.403	2643	WS01010_F09
12	0.000	233	1	0.231	0.054	4.976	2643	WS01010_F09
4	6.032	72	4	-0.282	0.072	4.597	2644	WS01010_F22
1	45.794	9	1	0.254	0.061	5.001	2645	WS01010_J01
1	50.955	10	1	-0.215	0.069	5.668	2645	WS01010_J01
1	95.572	17	2	-0.254	0.050	5.230	2645	WS01010_J01
1	95.572	17	3	0.217	0.047	4.849	2645	WS01010_J01
10	95.795	197	4	0.223	0.048	4.108	2645	WS01010_J01
2	120.617	38	3	-0.231	0.043	4.509	2646	WS01010_J13
2	127.147	40	3	0.260	0.077	8.054	2646	WS01010_J13
4	75.958	77	3	-0.262	0.079	5.314	2646	WS01010_J13

4	92.039	79	2	0.256	0.034	4.154	2646	WS01010_J13
5	0.000	89	1	-0.222	0.063	4.584	2646	WS01010_J13
5	0.000	89	3	0.382	0.077	8.142	2646	WS01010_J13
5	3.484	90	2	0.233	0.073	4.000	2646	WS01010_J13
6	56.188	120	2	0.284	0.044	5.432	2646	WS01010_J13
7	62.787	139	2	0.318	0.054	7.338	2646	WS01010_J13
7	75.883	144	2	-0.460	0.090	5.197	2646	WS01010_J13
9	55.358	175	3	0.515	0.165	5.230	2646	WS01010_J13
12	6.242	234	2	0.246	0.040	5.417	2646	WS01010_J13
13	1.815	244	2	0.248	0.050	6.116	2646	WS01010_J13
1	2.788	2	4	-0.220	0.044	4.052	2647	WS01010_L19
3	9.953	42	3	0.262	0.071	6.097	2647	WS01010_L19
3	137.891	64	3	0.324	0.098	8.326	2647	WS01010_L19
4	0.000	71	4	0.231	0.046	4.673	2647	WS01010_L19
4	173.929	88	2	-0.190	0.039	3.909	2647	WS01010_L19
8	50.001	154	1	-0.291	0.084	7.515	2647	WS01010_L19
8	78.389	157	1	-0.204	0.045	3.875	2647	WS01010_L19
8	95.937	159	3	-0.197	0.049	4.232	2647	WS01010_L19
10	9.580	187	3	0.314	0.088	5.313	2647	WS01010_L19
11	0.000	199	2	0.252	0.055	5.539	2647	WS01010_L19
11	27.405	204	1	0.255	0.062	5.473	2647	WS01010_L19
11	27.405	204	4	-0.244	0.046	4.646	2647	WS01010_L19
11	29.069	205	1	0.211	0.051	4.425	2647	WS01010_L19
11	45.989	216	1	-0.240	0.062	5.375	2647	WS01010_L19
2	120.617	38	3	-0.212	0.042	4.738	2648	WS01010_M14
3	9.953	42	3	0.213	0.040	4.518	2648	WS01010_M14
8	11.974	150	1	0.208	0.045	4.496	2648	WS01010_M14
8	50.001	154	1	-0.193	0.044	4.422	2648	WS01010_M14
8	95.937	159	3	-0.237	0.038	4.332	2648	WS01010_M14
9	59.963	177	4	0.246	0.054	3.905	2648	WS01010_M14
11	45.989	216	1	-0.197	0.048	4.811	2648	WS01010_M14
3	15.147	44	2	0.356	0.085	5.278	2649	WS01010_O12
4	173.929	88	2	-0.229	0.047	4.282	2649	WS01010_O12
6	81.996	123	3	0.343	0.058	4.223	2649	WS01010_O12
8	50.001	154	1	-0.265	0.051	4.948	2649	WS01010_O12
9	59.963	177	4	0.298	0.059	4.018	2649	WS01010_O12
10	70.246	190	2	-0.225	0.047	4.387	2649	WS01010_O12
1	43.578	8	2	-0.253	0.047	4.084	2650	WS01010_P06
1	95.572	17	2	0.336	0.096	8.306	2650	WS01010_P06
1	95.572	17	3	-0.331	0.117	9.321	2650	WS01010_P06
1	107.333	19	3	-0.225	0.077	5.636	2650	WS01010_P06
2	93.504	32	4	-0.310	0.051	5.693	2650	WS01010_P06
2	102.814	36	1	-0.240	0.044	4.291	2650	WS01010_P06
3	9.953	42	3	0.258	0.071	5.156	2650	WS01010_P06
3	137.891	64	3	0.248	0.067	4.779	2650	WS01010_P06
8	50.001	154	1	-0.255	0.061	6.253	2650	WS01010_P06
8	78.389	157	1	-0.201	0.040	4.022	2650	WS01010_P06
12	6.242	234	2	-0.174	0.038	5.125	2650	WS01010_P06
13	3.386	245	1	-0.253	0.052	5.197	2650	WS01010_P06
1	77.177	15	2	0.157	0.037	3.986	2651	WS00821_O13
3	14.500	43	2	-0.168	0.031	3.918	2651	WS00821_O13
4	173.929	88	2	-0.230	0.046	4.982	2651	WS00821_O13
5	3.484	90	2	-0.261	0.065	4.049	2651	WS00821_O13
6	69.073	121	3	0.213	0.042	3.944	2651	WS00821_O13

7	59.789	137	3	-0.251	0.063	5.950	2651	WS00821_O13
10	70.246	190	2	-0.247	0.050	5.359	2651	WS00821_O13
12	6.242	234	2	-0.365	0.104	12.685	2651	WS00821_O13
1	29.800	5	2	-0.292	0.057	4.284	2652	WS01027_E22
1	38.326	6	2	0.293	0.060	6.390	2652	WS01027_E22
1	45.794	9	1	0.445	0.129	10.893	2652	WS01027_E22
1	50.955	10	1	-0.271	0.065	5.344	2652	WS01027_E22
2	48.839	29	4	-0.338	0.067	4.158	2652	WS01027_E22
2	126.181	39	4	-0.512	0.109	4.530	2652	WS01027_E22
4	173.929	88	2	0.170	0.041	4.277	2652	WS01027_E22
5	0.000	89	1	0.252	0.051	4.102	2652	WS01027_E22
5	16.219	93	1	0.223	0.049	4.009	2652	WS01027_E22
10	21.864	188	1	0.352	0.096	7.609	2652	WS01027_E22
11	0.000	199	3	0.320	0.080	5.586	2652	WS01027_E22
11	27.405	204	1	-0.249	0.053	4.476	2652	WS01027_E22
11	43.491	214	4	0.253	0.062	3.936	2652	WS01027_E22
11	56.921	219	1	0.363	0.119	4.953	2652	WS01027_E22
11	57.661	220	4	-0.195	0.047	4.926	2652	WS01027_E22
11	64.014	222	4	0.320	0.061	4.082	2652	WS01027_E22
12	32.473	238	1	-0.181	0.047	3.855	2652	WS01027_E22
13	0.000	243	4	-0.308	0.098	4.095	2652	WS01027_E22
13	1.815	244	2	0.253	0.061	6.550	2652	WS01027_E22
14	22.402	248	4	-0.255	0.043	4.452	2652	WS01027_E22
14	46.637	252	4	-0.262	0.050	4.669	2652	WS01027_E22
1	45.794	9	1	0.290	0.044	4.637	2653	WS00823_J06
3	137.891	64	1	-0.244	0.043	4.678	2653	WS00823_J06
4	92.039	79	4	-0.347	0.085	4.822	2653	WS00823_J06
4	124.132	83	1	-0.492	0.117	4.840	2653	WS00823_J06
4	142.917	85	1	0.350	0.050	5.290	2653	WS00823_J06
6	49.100	118	4	-0.353	0.117	4.795	2653	WS00823_J06
7	70.278	141	3	-0.170	0.041	4.144	2653	WS00823_J06
8	87.463	158	2	-0.229	0.079	6.513	2653	WS00823_J06
9	17.951	169	2	-0.286	0.082	6.044	2653	WS00823_J06
9	59.963	177	1	-0.303	0.068	7.398	2653	WS00823_J06
13	3.386	245	1	0.254	0.067	7.009	2653	WS00823_J06
3	9.953	42	4	-0.246	0.053	4.294	2654	WS00823_O04
4	173.929	88	2	-0.215	0.050	5.861	2654	WS00823_O04
6	25.914	114	1	0.267	0.088	6.159	2654	WS00823_O04
10	70.246	190	2	-0.184	0.033	3.886	2654	WS00823_O04
11	0.000	199	3	-0.301	0.068	7.118	2654	WS00823_O04
13	1.815	244	2	-0.178	0.041	4.772	2654	WS00823_O04
14	22.402	248	4	0.312	0.083	5.296	2654	WS00823_O04
2	75.550	31	1	0.249	0.041	4.266	2655	WS00822_N23
2	127.147	40	4	-0.209	0.040	3.889	2655	WS00822_N23
7	15.555	130	2	-0.217	0.053	4.717	2655	WS00822_N23
7	15.555	130	3	0.288	0.046	4.173	2655	WS00822_N23
10	9.580	187	3	0.214	0.057	3.927	2655	WS00822_N23
11	0.000	199	3	-0.326	0.060	4.146	2655	WS00822_N23
11	27.405	204	1	0.378	0.087	9.246	2655	WS00822_N23
11	29.069	205	1	0.248	0.051	5.274	2655	WS00822_N23
11	45.989	216	1	-0.194	0.047	4.801	2655	WS00822_N23
12	28.493	228	2	-0.246	0.063	3.999	2655	WS00822_N23
1	2.788	2	4	-0.142	0.035	3.866	2656	WS00823_D05
3	80.211	55	4	0.179	0.038	4.274	2656	WS00823_D05

3	144.593	66	4	-0.166	0.037	4.481	2656	WS00823_D05
3	154.505	69	4	-0.189	0.043	4.287	2656	WS00823_D05
5	7.672	92	1	-0.191	0.037	3.888	2656	WS00823_D05
6	89.401	125	2	-0.241	0.033	3.898	2656	WS00823_D05
8	62.580	155	1	-0.289	0.164	3.907	2656	WS00823_D05
10	78.479	193	3	0.265	0.055	4.104	2656	WS00823_D05
11	27.405	204	1	0.437	0.146	10.490	2656	WS00823_D05
11	27.405	204	4	-0.321	0.061	7.058	2656	WS00823_D05
12	6.242	234	2	-0.259	0.051	8.012	2656	WS00823_D05
1	95.572	17	2	0.325	0.066	6.913	2657	WS00916_J09
1	95.572	17	3	-0.263	0.066	5.468	2657	WS00916_J09
2	127.147	40	4	-0.239	0.043	4.104	2657	WS00916_J09
3	15.147	44	2	0.223	0.057	4.589	2657	WS00916_J09
3	137.891	64	3	0.185	0.053	4.274	2657	WS00916_J09
7	15.555	130	2	-0.292	0.062	6.513	2657	WS00916_J09
7	15.555	130	3	0.242	0.056	4.519	2657	WS00916_J09
11	27.405	204	1	0.233	0.049	4.292	2657	WS00916_J09
11	27.405	204	4	-0.272	0.039	4.261	2657	WS00916_J09
12	28.493	228	2	-0.217	0.052	4.161	2657	WS00916_J09
12	14.406	230	4	0.248	0.040	4.379	2657	WS00916_J09
12	6.242	234	2	-0.207	0.046	6.294	2657	WS00916_J09
1	95.572	17	2	0.333	0.095	10.977	2658	WS00916_O18
2	127.147	40	4	-0.202	0.059	5.608	2658	WS00916_O18
3	9.953	42	3	0.205	0.064	5.909	2658	WS00916_O18
4	116.928	82	2	0.301	0.041	4.765	2658	WS00916_O18
4	124.132	83	2	0.425	0.167	8.498	2658	WS00916_O18
9	69.046	181	2	0.395	0.131	5.760	2658	WS00916_O18
11	0.000	199	2	0.201	0.037	4.164	2658	WS00916_O18
13	1.815	244	4	0.250	0.056	4.973	2658	WS00916_O18
3	9.953	42	3	0.241	0.076	7.551	2659	WS0078_L22
4	43.146	74	3	0.207	0.041	4.140	2659	WS0078_L22
5	16.219	93	2	0.214	0.037	3.968	2659	WS0078_L22
2	126.181	39	4	-0.390	0.099	6.723	2660	WS0078_M10
8	0.000	148	4	-0.274	0.054	4.672	2660	WS0078_M10
11	0.000	199	2	0.239	0.041	5.256	2660	WS0078_M10
12	51.382	242	1	0.489	0.155	4.886	2660	WS0078_M10
3	137.891	64	3	0.244	0.056	4.890	2661	WS0078_O04
4	173.929	88	2	-0.217	0.043	4.613	2661	WS0078_O04
5	3.484	90	2	-0.269	0.063	4.626	2661	WS0078_O04
7	15.555	130	2	-0.250	0.052	5.457	2661	WS0078_O04
7	17.610	131	3	-0.272	0.127	4.247	2661	WS0078_O04
10	70.246	190	2	-0.194	0.038	4.149	2661	WS0078_O04
10	78.479	193	3	0.280	0.056	4.901	2661	WS0078_O04
12	6.242	234	2	-0.284	0.058	7.297	2661	WS0078_O04
3	14.500	43	1	-0.258	0.070	5.255	2662	WS0078_O16
3	17.158	45	1	0.285	0.053	4.007	2662	WS0078_O16
4	75.958	77	3	0.423	0.070	5.948	2662	WS0078_O16
5	0.000	89	3	-0.116	0.029	4.438	2662	WS0078_O16
6	54.066	119	4	-0.135	0.063	4.172	2662	WS0078_O16
7	74.560	143	2	-0.330	0.042	5.023	2662	WS0078_O16
9	0.000	163	2	-0.495	0.063	4.919	2662	WS0078_O16
11	17.088	203	1	0.150	0.064	4.471	2662	WS0078_O16
11	37.143	209	3	0.243	0.033	4.178	2662	WS0078_O16
14	22.402	248	3	-0.224	0.034	4.220	2662	WS0078_O16

14	44.898	251	3	-0.343	0.052	6.422	2662	WS0078_O16
3	9.953	42	3	0.249	0.048	4.459	2663	WS0079_B04
4	55.925	76	1	-0.241	0.057	4.613	2663	WS0079_B04
6	25.914	114	1	0.251	0.055	4.981	2663	WS0079_B04
7	74.560	143	1	0.268	0.067	6.056	2663	WS0079_B04
8	50.001	154	1	-0.283	0.075	7.348	2663	WS0079_B04
8	87.463	158	2	-0.263	0.077	7.085	2663	WS0079_B04
10	9.580	187	3	0.399	0.106	7.133	2663	WS0079_B04
11	0.000	199	2	0.235	0.045	4.392	2663	WS0079_B04
11	27.405	204	1	0.200	0.052	4.673	2663	WS0079_B04
11	27.405	204	4	-0.249	0.049	4.619	2663	WS0079_B04
12	0.000	233	1	0.218	0.047	4.243	2663	WS0079_B04
3	9.953	42	3	0.209	0.058	6.005	2664	WS0079_B23
3	137.891	64	3	0.386	0.102	10.638	2664	WS0079_B23
4	142.917	85	3	-0.269	0.065	6.498	2664	WS0079_B23
5	54.072	100	4	0.236	0.064	5.351	2664	WS0079_B23
7	17.610	131	3	-0.360	0.165	5.502	2664	WS0079_B23
8	50.001	154	1	-0.208	0.066	5.814	2664	WS0079_B23
8	95.937	159	3	-0.192	0.043	4.418	2664	WS0079_B23
9	59.963	177	4	0.279	0.076	6.445	2664	WS0079_B23
10	78.479	193	3	0.310	0.043	4.547	2664	WS0079_B23
2	48.839	29	1	-0.230	0.047	4.952	2665	WS0079_E20
3	9.953	42	3	0.183	0.063	4.989	2665	WS0079_E20
3	17.158	45	1	-0.203	0.057	5.936	2665	WS0079_E20
4	55.925	76	1	-0.269	0.041	5.146	2665	WS0079_E20
6	25.914	114	1	0.160	0.040	4.112	2665	WS0079_E20
6	89.401	125	2	0.230	0.050	4.798	2665	WS0079_E20
7	73.169	142	1	-0.592	0.086	5.748	2665	WS0079_E20
9	3.127	168	1	0.478	0.078	6.673	2665	WS0079_E20
11	43.491	214	4	0.115	0.031	4.136	2665	WS0079_E20
11	56.921	219	1	0.237	0.097	6.353	2665	WS0079_E20
12	0.000	233	1	0.148	0.038	3.914	2665	WS0079_E20
14	0.000	246	1	-0.174	0.043	4.472	2665	WS0079_E20
2	127.147	40	4	-0.169	0.056	4.871	2666	WS0079_F17
7	73.169	142	2	0.285	0.038	3.968	2666	WS0079_F17
7	75.883	144	2	-0.464	0.085	5.539	2666	WS0079_F17
10	21.864	188	1	-0.331	0.072	6.821	2666	WS0079_F17
14	22.402	248	4	0.234	0.059	5.758	2666	WS0079_F17
1	40.464	7	4	0.175	0.045	4.461	2667	WS01034_I01
3	144.593	66	4	-0.259	0.057	4.805	2667	WS01034_I01
3	148.745	68	4	0.312	0.082	7.736	2667	WS01034_I01
3	154.505	69	4	-0.365	0.103	8.025	2667	WS01034_I01
4	75.958	77	3	0.413	0.119	6.951	2667	WS01034_I01
5	7.672	92	1	0.323	0.109	6.169	2667	WS01034_I01
6	49.100	118	2	0.237	0.078	4.493	2667	WS01034_I01
6	56.188	120	2	0.277	0.034	4.617	2667	WS01034_I01
6	69.073	121	3	-0.223	0.056	5.078	2667	WS01034_I01
7	15.555	130	2	-0.229	0.039	5.233	2667	WS01034_I01
7	73.169	142	2	0.525	0.089	5.431	2667	WS01034_I01
7	75.883	144	2	-0.416	0.064	4.903	2667	WS01034_I01
8	39.090	152	4	-0.283	0.091	8.852	2667	WS01034_I01
12	22.629	237	1	-0.276	0.077	4.414	2667	WS01034_I01
1	2.788	2	4	-0.257	0.097	9.035	2668	WS01034_I16
3	9.953	42	3	0.208	0.055	4.673	2668	WS01034_I16

3	15.147	44	2	0.234	0.057	4.142	2668	WS01034_I16
4	173.929	88	2	-0.154	0.040	4.123	2668	WS01034_I16
7	15.555	130	2	-0.263	0.048	4.941	2668	WS01034_I16
8	50.001	154	1	-0.209	0.070	6.301	2668	WS01034_I16
9	17.951	169	2	-0.237	0.042	4.456	2668	WS01034_I16
9	59.963	177	4	0.259	0.060	6.241	2668	WS01034_I16
10	9.580	187	3	0.315	0.082	4.152	2668	WS01034_I16
1	2.788	2	4	-0.158	0.035	4.639	2669	WS01034_L22
1	131.686	23	1	0.536	0.099	5.068	2669	WS01034_L22
3	9.953	42	4	0.183	0.047	4.031	2669	WS01034_L22
4	82.462	78	1	-0.196	0.061	5.140	2669	WS01034_L22
4	142.917	85	1	0.215	0.049	4.085	2669	WS01034_L22
5	54.072	100	4	0.218	0.057	6.814	2669	WS01034_L22
7	70.278	141	3	0.239	0.074	4.960	2669	WS01034_L22
11	27.405	204	4	-0.385	0.073	7.771	2669	WS01034_L22
11	43.491	214	4	-0.242	0.035	4.147	2669	WS01034_L22
12	6.242	234	4	0.505	0.095	10.436	2669	WS01034_L22
14	46.637	252	4	-0.460	0.087	10.630	2669	WS01034_L22
1	95.572	17	2	0.238	0.039	4.607	2670	WS01034_M19
3	15.147	44	2	0.345	0.102	7.669	2670	WS01034_M19
3	20.512	46	2	0.232	0.033	3.911	2670	WS01034_M19
3	104.681	60	2	0.218	0.034	4.024	2670	WS01034_M19
3	114.747	62	2	-0.227	0.034	4.024	2670	WS01034_M19
6	56.188	120	2	-0.286	0.042	4.396	2670	WS01034_M19
8	87.463	158	2	-0.194	0.048	4.880	2670	WS01034_M19
9	17.951	169	2	-0.249	0.039	4.267	2670	WS01034_M19
9	59.963	177	4	0.242	0.049	4.865	2670	WS01034_M19
3	15.147	44	2	0.296	0.076	5.388	2671	WS01034_P19
4	75.958	77	3	0.259	0.124	4.068	2671	WS01034_P19
4	173.929	88	2	-0.231	0.056	6.065	2671	WS01034_P19
6	56.188	120	2	-0.221	0.037	3.990	2671	WS01034_P19
7	15.555	130	2	-0.237	0.040	4.309	2671	WS01034_P19
8	50.001	154	1	-0.245	0.067	5.651	2671	WS01034_P19
10	9.580	187	3	0.384	0.112	5.989	2671	WS01034_P19
11	27.405	204	4	-0.274	0.049	4.173	2671	WS01034_P19
12	6.242	234	2	-0.221	0.041	5.510	2671	WS01034_P19
4	124.132	83	1	-0.405	0.063	4.461	2672	WS01035_A10
4	142.917	85	1	0.229	0.077	7.985	2672	WS01035_A10
5	0.000	89	1	-0.301	0.033	3.954	2672	WS01035_A10
9	81.073	183	1	0.152	0.043	4.391	2672	WS01035_A10
12	6.242	234	4	0.360	0.058	6.539	2672	WS01035_A10
12	51.382	242	1	0.481	0.074	4.903	2672	WS01035_A10
1	107.333	19	3	-0.219	0.055	4.774	2673	WS01035_D13
3	15.147	44	2	0.260	0.066	4.000	2673	WS01035_D13
3	80.211	55	4	0.341	0.097	9.831	2673	WS01035_D13
11	27.405	204	4	-0.252	0.043	4.408	2673	WS01035_D13
12	28.493	228	2	-0.325	0.095	6.563	2673	WS01035_D13
3	14.500	43	1	-0.295	0.044	4.628	2674	WS01035_F01
4	43.146	74	3	0.225	0.032	4.107	2674	WS01035_F01
4	55.925	76	1	-0.257	0.061	6.422	2674	WS01035_F01
5	0.000	89	1	-0.345	0.071	7.769	2674	WS01035_F01
5	16.219	93	1	-0.332	0.120	12.443	2674	WS01035_F01
5	31.191	95	1	-0.230	0.052	5.446	2674	WS01035_F01
6	25.742	113	3	-0.192	0.050	6.300	2674	WS01035_F01

7	15.555	130	3	0.168	0.032	3.995	2674	WS01035_F01
7	59.789	137	3	-0.228	0.034	4.354	2674	WS01035_F01
12	0.000	233	1	0.233	0.047	4.899	2674	WS01035_F01
3	154.505	69	1	0.243	0.045	4.131	2675	WS01037_L19
8	11.974	150	1	-0.268	0.052	4.833	2675	WS01037_L19
11	43.491	214	3	0.221	0.061	5.750	2675	WS01037_L19
11	44.884	215	3	-0.216	0.044	4.171	2675	WS01037_L19
11	64.014	222	3	-0.287	0.070	6.614	2675	WS01037_L19
12	22.441	227	1	-0.289	0.055	5.145	2675	WS01037_L19
13	1.815	244	2	0.209	0.047	4.640	2675	WS01037_L19
2	127.147	40	4	-0.220	0.046	4.242	2676	WS01037_M12
3	15.147	44	2	0.270	0.073	4.794	2676	WS01037_M12
7	15.555	130	2	-0.224	0.044	4.393	2676	WS01037_M12
7	15.555	130	3	0.242	0.063	5.646	2676	WS01037_M12
9	36.227	165	3	-0.215	0.065	5.743	2676	WS01037_M12
9	59.963	177	4	0.250	0.050	4.294	2676	WS01037_M12
10	70.246	190	2	-0.181	0.040	4.021	2676	WS01037_M12
11	0.000	199	2	0.246	0.043	4.294	2676	WS01037_M12
12	6.242	234	2	-0.246	0.070	8.695	2676	WS01037_M12
3	15.147	44	2	-0.259	0.103	6.070	2677	WS01037_O20
5	0.000	89	3	-0.158	0.050	4.152	2677	WS01037_O20
6	26.350	115	2	-0.172	0.036	4.596	2677	WS01037_O20
11	29.069	205	1	-0.267	0.080	5.169	2677	WS01037_O20
11	45.989	216	1	0.290	0.104	6.236	2677	WS01037_O20
1	43.578	8	2	-0.267	0.066	6.759	2678	WS00724_A12
1	95.572	17	2	0.323	0.059	6.039	2678	WS00724_A12
1	123.316	20	4	0.347	0.078	6.116	2678	WS00724_A12
2	127.147	40	4	-0.254	0.051	5.373	2678	WS00724_A12
9	36.227	165	4	-0.284	0.048	4.685	2678	WS00724_A12
12	28.493	228	2	-0.309	0.093	7.215	2678	WS00724_A12
13	3.386	245	1	-0.247	0.070	5.998	2678	WS00724_A12
14	43.392	250	2	-0.225	0.050	3.970	2678	WS00724_A12
1	2.788	2	4	-0.246	0.072	4.135	2679	WS00724_E10
1	62.128	11	1	-0.456	0.112	3.966	2679	WS00724_E10
1	62.128	11	2	0.308	0.070	4.288	2679	WS00724_E10
3	47.928	49	1	0.239	0.072	6.776	2679	WS00724_E10
3	95.157	59	2	-0.191	0.060	5.866	2679	WS00724_E10
3	104.681	60	2	0.212	0.041	3.849	2679	WS00724_E10
4	142.917	85	1	-0.291	0.046	4.343	2679	WS00724_E10
5	52.820	99	3	-0.251	0.032	3.875	2679	WS00724_E10
6	25.742	113	3	-0.232	0.055	6.573	2679	WS00724_E10
7	17.610	131	3	0.308	0.066	4.075	2679	WS00724_E10
10	35.204	189	2	0.339	0.093	8.465	2679	WS00724_E10
11	17.088	203	1	0.293	0.063	6.016	2679	WS00724_E10
1	2.788	2	4	-0.317	0.075	7.775	2680	WS00724_G07
3	148.745	68	1	-0.377	0.106	9.471	2680	WS00724_G07
4	43.146	74	3	0.220	0.044	4.019	2680	WS00724_G07
5	3.484	90	2	-0.328	0.079	4.852	2680	WS00724_G07
5	16.219	93	2	0.300	0.053	5.509	2680	WS00724_G07
5	84.347	105	1	0.226	0.070	5.842	2680	WS00724_G07
6	95.249	126	1	-0.342	0.104	4.931	2680	WS00724_G07
6	106.702	128	2	0.394	0.086	3.862	2680	WS00724_G07
11	27.405	204	1	0.214	0.062	5.064	2680	WS00724_G07
14	46.637	252	4	0.253	0.049	4.612	2680	WS00724_G07

2	127.147	40	4	-0.201	0.041	4.558	2681	WS00724_L08
3	15.147	44	2	0.254	0.052	4.173	2681	WS00724_L08
4	173.929	88	2	-0.240	0.047	4.862	2681	WS00724_L08
7	15.555	130	2	-0.233	0.041	4.172	2681	WS00724_L08
12	14.406	230	4	0.289	0.056	5.995	2681	WS00724_L08
14	43.392	250	2	-0.168	0.045	4.302	2681	WS00724_L08
1	69.854	13	2	0.292	0.075	6.603	2682	WS00724_M09
1	71.592	14	2	0.293	0.077	6.753	2682	WS00724_M09
1	77.177	15	2	0.291	0.074	6.517	2682	WS00724_M09
1	95.572	17	2	0.305	0.053	4.849	2682	WS00724_M09
2	120.617	38	3	-0.261	0.035	3.914	2682	WS00724_M09
3	9.953	42	3	0.171	0.039	4.194	2682	WS00724_M09
3	137.891	64	3	0.236	0.038	4.257	2682	WS00724_M09
6	106.702	128	2	0.418	0.120	4.039	2682	WS00724_M09
7	15.555	130	3	0.311	0.067	7.700	2682	WS00724_M09
8	50.001	154	1	-0.122	0.034	3.918	2682	WS00724_M09
9	81.073	183	1	0.228	0.049	4.968	2682	WS00724_M09
10	104.214	198	2	-0.225	0.052	4.932	2682	WS00724_M09
12	6.242	234	2	-0.197	0.043	4.890	2682	WS00724_M09
13	3.386	245	1	-0.221	0.039	3.983	2682	WS00724_M09
1	2.788	2	4	-0.270	0.064	6.885	2683	WS0072_N14
5	7.672	92	1	-0.304	0.074	7.503	2683	WS0072_N14
7	15.555	130	2	-0.249	0.045	4.514	2683	WS0072_N14
7	105.477	147	2	-0.260	0.075	5.305	2683	WS0072_N14
8	62.580	155	1	-0.372	0.111	4.117	2683	WS0072_N14
8	78.389	157	1	-0.201	0.045	3.915	2683	WS0072_N14
9	81.073	183	3	0.196	0.052	3.922	2683	WS0072_N14
11	27.405	204	1	0.254	0.043	3.870	2683	WS0072_N14
11	27.405	204	4	-0.283	0.046	5.069	2683	WS0072_N14
11	45.989	216	1	-0.209	0.045	3.957	2683	WS0072_N14
14	22.402	248	4	0.342	0.068	6.312	2683	WS0072_N14
2	127.147	40	4	-0.182	0.050	4.108	2684	WS0072_O03
3	9.953	42	3	0.254	0.052	5.465	2684	WS0072_O03
3	14.500	43	2	-0.215	0.046	4.361	2684	WS0072_O03
6	89.401	125	1	0.250	0.044	4.282	2684	WS0072_O03
9	69.046	181	2	0.303	0.119	4.900	2684	WS0072_O03
10	9.580	187	3	0.317	0.068	3.981	2684	WS0072_O03
12	0.000	233	1	0.271	0.067	6.502	2684	WS0072_O03
12	22.629	237	1	0.297	0.072	7.001	2684	WS0072_O03
12	32.473	238	1	0.303	0.075	7.274	2684	WS0072_O03
1	45.794	9	1	0.187	0.052	3.898	2685	WS0073_B07
1	45.794	9	3	-0.319	0.092	8.947	2685	WS0073_B07
3	137.891	64	3	0.363	0.074	4.387	2685	WS0073_B07
4	124.132	83	1	-0.547	0.145	5.993	2685	WS0073_B07
6	49.100	118	4	-0.399	0.098	4.034	2685	WS0073_B07
12	22.441	227	1	0.200	0.055	4.166	2685	WS0073_B07
14	22.402	248	3	-0.314	0.085	8.065	2685	WS0073_B07
6	106.702	128	2	-0.482	0.138	6.213	2686	WS0073_C04
10	104.214	198	2	-0.216	0.036	4.167	2686	WS0073_C04
12	6.242	234	2	-0.231	0.043	4.786	2686	WS0073_C04
13	1.815	244	2	-0.308	0.067	6.513	2686	WS0073_C04
1	2.788	2	4	-0.256	0.062	5.121	2687	WS0073_E06
2	63.261	30	3	0.220	0.047	5.411	2687	WS0073_E06
3	9.953	42	3	0.175	0.051	5.826	2687	WS0073_E06

3	95.157	59	2	-0.239	0.055	5.695	2687	WS0073_E06
3	104.681	60	2	0.168	0.039	3.842	2687	WS0073_E06
6	25.742	113	3	-0.187	0.035	4.048	2687	WS0073_E06
8	50.001	154	1	-0.246	0.076	6.389	2687	WS0073_E06
8	50.001	154	2	-0.220	0.046	4.530	2687	WS0073_E06
9	17.951	169	1	0.277	0.056	5.023	2687	WS0073_E06
11	42.600	213	3	0.231	0.051	5.878	2687	WS0073_E06
11	64.014	222	3	0.251	0.037	4.251	2687	WS0073_E06
1	40.464	7	3	0.279	0.091	8.482	2688	WS0073_F15
1	43.578	8	3	0.300	0.106	9.669	2688	WS0073_F15
1	45.794	9	3	-0.273	0.068	6.773	2688	WS0073_F15
1	95.572	17	2	-0.204	0.041	4.076	2688	WS0073_F15
2	93.504	32	4	0.305	0.048	4.585	2688	WS0073_F15
3	15.147	44	2	-0.315	0.070	4.190	2688	WS0073_F15
3	83.563	56	3	-0.157	0.046	4.283	2688	WS0073_F15
4	116.928	82	2	-0.228	0.043	4.524	2688	WS0073_F15
6	25.914	114	1	-0.287	0.070	6.026	2688	WS0073_F15
7	70.278	141	3	-0.234	0.046	4.353	2688	WS0073_F15
7	75.883	144	2	0.363	0.061	3.962	2688	WS0073_F15
7	75.883	144	4	-0.328	0.052	5.067	2688	WS0073_F15
10	77.676	192	1	-0.249	0.108	9.307	2688	WS0073_F15
10	80.088	194	1	-0.303	0.095	8.202	2688	WS0073_F15
10	93.347	196	1	0.687	0.218	11.344	2688	WS0073_F15
11	40.960	210	2	-0.369	0.067	4.204	2688	WS0073_F15
11	45.989	216	1	-0.208	0.056	4.775	2688	WS0073_F15
12	22.441	227	1	0.352	0.051	4.436	2688	WS0073_F15
14	22.402	248	4	0.299	0.043	4.259	2688	WS0073_F15
1	22.419	4	4	0.277	0.101	4.588	2689	WS0073_J17
1	29.800	5	1	-0.132	0.066	4.757	2689	WS0073_J17
1	38.326	6	4	0.388	0.073	6.046	2689	WS0073_J17
1	40.464	7	4	-0.283	0.045	6.688	2689	WS0073_J17
1	82.301	16	4	0.444	0.068	7.052	2689	WS0073_J17
4	103.719	81	4	-0.536	0.157	15.403	2689	WS0073_J17
4	116.928	82	2	0.590	0.219	22.705	2689	WS0073_J17
4	116.928	82	4	0.373	0.100	8.308	2689	WS0073_J17
4	124.132	83	1	0.470	0.154	4.535	2689	WS0073_J17
4	124.132	83	2	0.599	0.354	14.195	2689	WS0073_J17
4	173.929	88	2	-0.304	0.056	6.684	2689	WS0073_J17
5	86.669	106	2	0.236	0.094	4.227	2689	WS0073_J17
7	70.278	141	3	0.317	0.053	5.227	2689	WS0073_J17
9	2.011	167	4	0.364	0.073	7.655	2689	WS0073_J17
9	28.608	170	2	0.242	0.025	4.251	2689	WS0073_J17
9	69.046	181	2	0.188	0.038	3.987	2689	WS0073_J17
1	29.800	5	1	-0.180	0.061	3.930	2690	WS0073_K21
1	43.578	8	2	-0.333	0.041	5.504	2690	WS0073_K21
5	56.008	101	2	0.385	0.154	16.327	2690	WS0073_K21
5	57.017	102	2	-0.276	0.074	8.689	2690	WS0073_K21
5	84.347	105	2	0.621	0.271	30.171	2690	WS0073_K21
5	86.669	106	2	-0.443	0.262	12.175	2690	WS0073_K21
7	17.610	131	3	-0.245	0.103	4.736	2690	WS0073_K21
7	54.200	136	2	-0.252	0.108	4.963	2690	WS0073_K21
7	73.169	142	1	0.583	0.173	5.438	2690	WS0073_K21
7	75.883	144	2	0.597	0.122	10.527	2690	WS0073_K21
7	75.883	144	4	-0.352	0.038	4.161	2690	WS0073_K21

14	0.000	246	1	0.145	0.080	5.178	2690	WS0073_K21
1	95.572	17	2	0.238	0.042	4.043	2691	WS0101_F16
2	48.839	29	4	0.224	0.052	3.977	2691	WS0101_F16
3	137.891	64	3	0.262	0.039	4.016	2691	WS0101_F16
4	142.917	85	3	-0.253	0.059	5.978	2691	WS0101_F16
8	11.974	150	1	0.240	0.048	4.461	2691	WS0101_F16
12	6.242	234	2	-0.163	0.024	3.848	2691	WS0101_F16
13	3.386	245	1	-0.345	0.091	8.418	2691	WS0101_F16
1	69.854	13	2	0.207	0.066	6.845	2692	WS0101_L03
1	71.592	14	2	0.211	0.069	7.153	2692	WS0101_L03
1	77.177	15	2	0.171	0.047	4.868	2692	WS0101_L03
1	95.572	17	2	0.208	0.038	3.881	2692	WS0101_L03
5	0.000	89	3	0.279	0.061	6.358	2692	WS0101_L03
5	3.484	90	2	-0.311	0.094	4.345	2692	WS0101_L03
9	81.073	183	1	0.255	0.055	4.775	2692	WS0101_L03
1	45.794	9	1	-0.311	0.040	3.936	2693	WS0079_K20
1	45.794	9	3	0.259	0.050	4.841	2693	WS0079_K20
1	131.686	23	1	0.460	0.077	3.850	2693	WS0079_K20
1	141.021	25	2	0.176	0.053	4.980	2693	WS0079_K20
2	127.147	40	4	-0.182	0.046	4.127	2693	WS0079_K20
10	35.204	189	1	-0.248	0.041	3.905	2693	WS0079_K20
10	70.246	190	4	-0.350	0.117	11.610	2693	WS0079_K20
10	77.676	192	1	-0.285	0.051	4.859	2693	WS0079_K20
10	78.479	193	4	-0.257	0.112	8.292	2693	WS0079_K20
10	80.088	194	1	-0.302	0.069	6.408	2693	WS0079_K20
10	104.214	198	2	-0.225	0.037	4.453	2693	WS0079_K20
11	43.491	214	3	0.271	0.072	6.546	2693	WS0079_K20
11	44.884	215	3	-0.201	0.045	4.065	2693	WS0079_K20
11	64.014	222	3	-0.262	0.072	6.582	2693	WS0079_K20
12	22.441	227	3	0.232	0.068	6.728	2693	WS0079_K20
1	29.800	5	1	-0.252	0.078	7.417	2694	WS00938_K20
1	45.794	9	1	-0.359	0.072	6.951	2694	WS00938_K20
1	50.955	10	1	0.223	0.082	7.824	2694	WS00938_K20
2	48.839	29	4	0.131	0.039	4.586	2694	WS00938_K20
4	43.146	74	3	0.263	0.042	3.850	2694	WS00938_K20
4	55.925	76	1	-0.140	0.041	3.845	2694	WS00938_K20
4	82.462	78	1	-0.142	0.049	4.677	2694	WS00938_K20
4	116.928	82	4	0.300	0.068	5.974	2694	WS00938_K20
6	106.702	128	2	-0.357	0.107	3.841	2694	WS00938_K20
8	0.000	148	4	-0.276	0.041	4.307	2694	WS00938_K20
9	0.000	163	1	0.481	0.092	5.100	2694	WS00938_K20
9	81.073	183	3	-0.231	0.058	4.974	2694	WS00938_K20
10	70.246	190	4	0.313	0.060	5.449	2694	WS00938_K20
1	38.326	6	2	0.165	0.040	4.292	2695	WS0092_B07
1	40.464	7	4	-0.142	0.029	4.494	2695	WS0092_B07
1	43.578	8	2	-0.331	0.101	10.846	2695	WS0092_B07
1	69.854	13	2	0.242	0.051	5.487	2695	WS0092_B07
1	71.592	14	2	0.242	0.052	5.587	2695	WS0092_B07
2	48.839	29	4	0.288	0.048	7.152	2695	WS0092_B07
3	80.211	55	4	0.156	0.046	5.576	2695	WS0092_B07
4	55.925	76	1	-0.306	0.070	6.016	2695	WS0092_B07
6	69.073	121	4	0.333	0.044	5.434	2695	WS0092_B07
6	87.891	124	2	-0.184	0.047	5.055	2695	WS0092_B07
7	15.555	130	2	-0.271	0.056	6.386	2695	WS0092_B07

9	17.951	169	2	-0.296	0.050	4.806	2695	WS0092_B07
10	70.246	190	4	0.273	0.057	7.008	2695	WS0092_B07
10	78.479	193	4	0.263	0.046	5.346	2695	WS0092_B07
11	41.984	212	2	-0.221	0.038	4.101	2695	WS0092_B07
12	22.441	227	1	-0.360	0.119	9.695	2695	WS0092_B07
12	22.441	227	3	0.311	0.095	6.547	2695	WS0092_B07
12	14.406	230	4	0.278	0.034	4.810	2695	WS0092_B07
12	0.000	233	1	0.182	0.060	4.769	2695	WS0092_B07
14	22.402	248	4	0.221	0.024	3.875	2695	WS0092_B07
1	62.128	11	1	-0.584	0.143	6.959	2696	WS00939_E14
1	107.333	19	4	-0.149	0.029	3.924	2696	WS00939_E14
2	126.181	39	4	-0.410	0.070	5.828	2696	WS00939_E14
3	148.745	68	4	-0.211	0.033	4.286	2696	WS00939_E14
4	0.000	71	4	-0.189	0.036	4.534	2696	WS00939_E14
4	55.925	76	1	-0.279	0.060	4.624	2696	WS00939_E14
8	0.000	148	4	-0.280	0.048	6.123	2696	WS00939_E14
8	50.001	154	2	-0.200	0.035	3.885	2696	WS00939_E14
8	87.463	158	2	0.164	0.040	4.431	2696	WS00939_E14
1	62.128	11	1	-0.536	0.239	5.465	2697	WS02610_E21
1	107.333	19	3	-0.209	0.044	4.883	2697	WS02610_E21
7	15.555	130	3	0.316	0.088	7.267	2697	WS02610_E21
11	37.143	209	3	0.204	0.042	4.727	2697	WS02610_E21
13	3.386	245	1	-0.322	0.067	7.524	2697	WS02610_E21
1	95.572	17	2	0.226	0.050	4.206	2698	WS02610_F20
1	107.333	19	3	-0.195	0.046	3.925	2698	WS02610_F20
2	127.147	40	4	-0.229	0.060	5.982	2698	WS02610_F20
3	9.953	42	3	0.227	0.052	4.454	2698	WS02610_F20
7	74.560	143	1	0.248	0.050	5.221	2698	WS02610_F20
11	37.143	209	3	0.222	0.059	5.037	2698	WS02610_F20
11	42.600	213	3	-0.210	0.049	4.145	2698	WS02610_F20
11	64.014	222	4	-0.328	0.063	6.455	2698	WS02610_F20
12	6.242	234	2	-0.271	0.069	7.375	2698	WS02610_F20
13	0.000	243	4	0.395	0.144	7.271	2698	WS02610_F20
13	3.386	245	1	-0.290	0.061	6.397	2698	WS02610_F20
1	95.572	17	2	0.253	0.055	5.592	2699	WS02610_K20
2	127.147	40	4	-0.209	0.046	4.542	2699	WS02610_K20
3	9.953	42	3	0.239	0.058	5.064	2699	WS02610_K20
4	173.929	88	2	-0.208	0.046	4.631	2699	WS02610_K20
8	50.001	154	1	-0.221	0.046	4.198	2699	WS02610_K20
10	9.580	187	3	0.335	0.102	6.167	2699	WS02610_K20
12	28.493	228	2	-0.321	0.079	5.940	2699	WS02610_K20
12	14.406	230	4	0.249	0.046	4.486	2699	WS02610_K20
1	38.326	6	2	-0.280	0.070	7.314	2700	WS02610_M08
2	126.181	39	2	-0.200	0.041	4.257	2700	WS02610_M08
3	80.211	55	4	-0.171	0.051	4.518	2700	WS02610_M08
4	43.146	74	3	0.262	0.054	5.389	2700	WS02610_M08
4	124.132	83	1	-0.451	0.090	4.163	2700	WS02610_M08
6	26.350	115	2	-0.198	0.037	3.860	2700	WS02610_M08
6	95.249	126	1	-0.326	0.114	4.458	2700	WS02610_M08
1	45.794	9	3	-0.244	0.035	4.512	2701	WS02611_B01
2	63.261	30	3	0.224	0.031	4.151	2701	WS02611_B01
2	95.880	34	3	0.249	0.038	4.583	2701	WS02611_B01
3	9.953	42	3	0.332	0.103	13.687	2701	WS02611_B01
5	52.820	99	3	-0.224	0.030	4.039	2701	WS02611_B01

6	106.702	128	2	0.427	0.085	5.809	2701	WS02611_B01
7	15.555	130	3	0.215	0.031	4.050	2701	WS02611_B01
8	50.001	154	1	-0.189	0.063	5.173	2701	WS02611_B01
8	95.937	159	3	-0.170	0.037	4.901	2701	WS02611_B01
4	0.000	71	4	0.249	0.045	4.379	2702	WS02611_E13
4	43.146	74	3	0.272	0.063	5.721	2702	WS02611_E13
7	59.789	137	3	-0.272	0.067	6.098	2702	WS02611_E13
11	0.000	199	2	0.277	0.063	6.248	2702	WS02611_E13
1	71.592	14	2	0.199	0.034	4.151	2703	WS02611_J22
1	95.572	17	2	0.319	0.062	7.712	2703	WS02611_J22
7	73.169	142	2	0.497	0.082	6.012	2703	WS02611_J22
7	75.883	144	2	-0.604	0.127	9.606	2703	WS02611_J22
9	28.608	170	3	0.255	0.053	5.172	2703	WS02611_J22
3	9.953	42	3	0.210	0.055	6.879	2704	WS02611_K22
3	83.563	56	3	-0.249	0.052	6.583	2704	WS02611_K22
3	148.745	68	1	0.293	0.048	4.939	2704	WS02611_K22
3	154.505	69	1	0.341	0.053	5.409	2704	WS02611_K22
3	154.505	69	4	-0.168	0.072	4.701	2704	WS02611_K22
4	103.719	81	4	0.215	0.066	4.636	2704	WS02611_K22
9	69.046	181	1	-0.286	0.050	4.065	2704	WS02611_K22
9	81.073	183	1	0.297	0.077	8.004	2704	WS02611_K22
3	15.147	44	2	0.351	0.092	6.080	2705	WS00939_J20
3	137.891	64	3	0.267	0.070	7.008	2705	WS00939_J20
4	173.929	88	2	-0.259	0.048	5.925	2705	WS00939_J20
5	54.072	100	4	0.197	0.057	4.580	2705	WS00939_J20
7	17.610	131	3	-0.379	0.157	9.565	2705	WS00939_J20
9	59.963	177	4	0.344	0.091	6.685	2705	WS00939_J20
11	56.921	219	2	0.310	0.065	3.894	2705	WS00939_J20
12	14.406	230	4	0.331	0.088	7.375	2705	WS00939_J20
12	6.242	234	2	-0.186	0.033	4.897	2705	WS00939_J20
6	26.350	115	2	-0.244	0.064	6.708	2706	WS00939_L09
14	22.402	248	4	-0.379	0.057	5.451	2706	WS00939_L09
1	22.419	4	4	0.325	0.080	5.203	2707	WS00939_P12
1	62.128	11	2	-0.292	0.138	3.905	2707	WS00939_P12
7	67.154	140	4	0.375	0.101	5.512	2707	WS00939_P12
8	11.974	150	1	0.231	0.075	7.346	2707	WS00939_P12
9	28.608	170	3	0.396	0.117	6.170	2707	WS00939_P12
10	21.864	188	1	-0.284	0.056	5.540	2707	WS00939_P12
11	0.000	199	3	-0.290	0.059	5.313	2707	WS00939_P12
11	46.026	217	1	-0.147	0.040	3.899	2707	WS00939_P12
1	15.589	3	2	0.195	0.037	4.260	2708	WS01038_A13
2	93.504	32	2	0.243	0.034	3.930	2708	WS01038_A13
8	87.463	158	2	0.263	0.066	6.432	2708	WS01038_A13
9	32.356	171	2	0.185	0.039	4.472	2708	WS01038_A13
11	40.960	210	2	0.511	0.090	7.187	2708	WS01038_A13
1	50.955	10	1	-0.186	0.049	4.383	2709	WS01038_F06
1	82.301	16	3	-0.288	0.050	4.973	2709	WS01038_F06
4	75.958	77	3	0.363	0.110	5.021	2709	WS01038_F06
4	142.917	85	1	0.195	0.049	4.380	2709	WS01038_F06
5	42.979	97	2	-0.257	0.053	5.497	2709	WS01038_F06
5	54.072	100	4	0.200	0.073	6.456	2709	WS01038_F06
6	87.891	124	2	0.222	0.044	4.272	2709	WS01038_F06
6	89.401	125	2	0.203	0.040	4.200	2709	WS01038_F06
9	2.011	167	4	-0.289	0.070	6.315	2709	WS01038_F06

9	3.127	168	1	0.443	0.108	4.327	2709	WS01038_F06
8	50.001	154	1	-0.231	0.056	4.787	2710	WS01038_G04
10	9.580	187	3	0.444	0.137	8.853	2710	WS01038_G04
13	3.386	245	1	-0.320	0.065	5.902	2710	WS01038_G04
1	43.578	8	2	-0.207	0.037	4.191	2711	WS01038_J06
1	95.572	17	2	0.341	0.076	8.442	2711	WS01038_J06
1	95.572	17	3	-0.189	0.046	4.165	2711	WS01038_J06
3	15.147	44	2	0.252	0.063	4.943	2711	WS01038_J06
5	54.072	100	4	0.233	0.063	6.200	2711	WS01038_J06
8	50.001	154	1	-0.253	0.079	6.211	2711	WS01038_J06
9	59.963	177	4	0.281	0.072	7.155	2711	WS01038_J06
12	6.242	234	2	-0.223	0.044	7.593	2711	WS01038_J06
1	95.572	17	2	0.240	0.051	4.866	2712	WS01038_K03
3	15.147	44	2	0.331	0.099	5.633	2712	WS01038_K03
5	54.072	100	4	0.182	0.050	4.234	2712	WS01038_K03
8	50.001	154	1	-0.285	0.078	7.375	2712	WS01038_K03
9	59.963	177	4	0.270	0.063	5.341	2712	WS01038_K03
10	9.580	187	3	0.478	0.158	10.379	2712	WS01038_K03
12	6.242	234	2	-0.256	0.058	6.274	2712	WS01038_K03
12	6.242	234	4	0.342	0.052	4.324	2712	WS01038_K03
1	95.572	17	2	0.242	0.043	4.523	2713	WS02612_E11
3	9.953	42	3	0.226	0.050	4.725	2713	WS02612_E11
3	15.147	44	2	0.290	0.089	4.708	2713	WS02612_E11
3	137.891	64	3	0.345	0.103	9.783	2713	WS02612_E11
7	17.610	131	3	-0.314	0.104	5.063	2713	WS02612_E11
7	75.883	144	2	0.393	0.069	4.048	2713	WS02612_E11
8	62.580	155	1	-0.324	0.118	4.356	2713	WS02612_E11
10	9.580	187	3	0.297	0.101	4.674	2713	WS02612_E11
10	78.479	193	3	0.295	0.053	5.006	2713	WS02612_E11
1	45.794	9	3	-0.326	0.048	5.228	2714	WS02613_F09
1	133.992	24	2	0.216	0.037	4.191	2714	WS02613_F09
3	137.891	64	3	0.208	0.039	4.907	2714	WS02613_F09
4	43.146	74	3	0.164	0.035	4.265	2714	WS02613_F09
4	75.958	77	3	-0.357	0.080	5.287	2714	WS02613_F09
6	106.702	128	2	-0.460	0.091	6.015	2714	WS02613_F09
7	15.555	130	2	-0.286	0.051	5.946	2714	WS02613_F09
9	0.000	163	1	0.424	0.070	4.042	2714	WS02613_F09
11	60.464	221	2	0.170	0.040	4.659	2714	WS02613_F09
1	45.794	9	3	-0.288	0.043	4.600	2715	WS0091_B05
4	55.925	76	1	-0.187	0.045	4.007	2715	WS0091_B05
4	82.462	78	1	-0.290	0.062	6.441	2715	WS0091_B05
4	116.928	82	2	-0.292	0.063	5.407	2715	WS0091_B05
4	124.132	83	2	-0.339	0.109	4.549	2715	WS0091_B05
5	42.979	97	1	-0.227	0.040	4.149	2715	WS0091_B05
5	52.820	99	1	-0.223	0.040	4.092	2715	WS0091_B05
5	56.008	101	1	0.226	0.037	3.882	2715	WS0091_B05
8	95.937	159	3	-0.180	0.038	4.236	2715	WS0091_B05
9	17.951	169	1	-0.407	0.068	7.149	2715	WS0091_B05
11	27.405	204	4	-0.263	0.074	4.450	2715	WS0091_B05
12	0.000	233	1	0.333	0.078	8.158	2715	WS0091_B05
2	75.550	31	1	0.304	0.054	4.585	2716	WS00928_B01
3	137.891	64	3	0.275	0.084	6.836	2716	WS00928_B01
10	9.580	187	3	0.352	0.135	5.512	2716	WS00928_B01
10	21.864	188	1	-0.233	0.056	4.711	2716	WS00928_B01

11	27.405	204	1	0.239	0.057	4.937	2716	WS00928_B01
12	6.242	234	2	-0.202	0.036	4.797	2716	WS00928_B01
1	15.589	3	2	-0.189	0.042	4.508	2717	WS0091_G11
3	9.953	42	3	0.187	0.040	4.274	2717	WS0091_G11
4	124.132	83	2	0.245	0.064	3.960	2717	WS0091_G11
4	173.929	88	2	-0.226	0.056	5.966	2717	WS0091_G11
8	95.937	159	3	-0.194	0.038	4.063	2717	WS0091_G11
9	69.046	181	2	0.306	0.083	4.582	2717	WS0091_G11
10	9.580	187	3	0.271	0.087	4.543	2717	WS0091_G11
1	95.572	17	2	0.181	0.037	4.321	2718	WS00928_E21
4	55.925	76	1	-0.208	0.059	4.039	2718	WS00928_E21
8	87.463	158	2	-0.198	0.041	4.034	2718	WS00928_E21
11	27.405	204	4	-0.225	0.065	4.693	2718	WS00928_E21
12	0.000	233	1	0.254	0.071	5.069	2718	WS00928_E21
1	22.419	4	4	-0.367	0.138	6.533	2719	WS0056_A24
6	45.897	117	4	-0.294	0.042	4.395	2719	WS0056_A24
6	56.188	120	2	0.216	0.046	4.580	2719	WS0056_A24
8	50.001	154	2	-0.220	0.064	5.624	2719	WS0056_A24
8	87.463	158	2	0.364	0.113	10.841	2719	WS0056_A24
9	17.951	169	1	-0.305	0.069	5.862	2719	WS0056_A24
10	70.246	190	4	0.281	0.053	5.256	2719	WS0056_A24
10	77.676	192	1	0.228	0.061	5.227	2719	WS0056_A24
10	78.479	193	4	0.218	0.053	5.300	2719	WS0056_A24
10	80.088	194	1	0.268	0.084	7.177	2719	WS0056_A24
10	104.214	198	4	0.295	0.042	5.160	2719	WS0056_A24
11	48.979	218	4	0.223	0.043	3.942	2719	WS0056_A24
1	2.788	2	4	-0.267	0.067	6.371	2720	WS0056_B11
2	127.147	40	4	-0.178	0.044	4.971	2720	WS0056_B11
5	56.008	101	1	0.162	0.050	4.820	2720	WS0056_B11
5	57.017	102	2	-0.248	0.078	7.429	2720	WS0056_B11
5	84.347	105	1	0.194	0.045	4.344	2720	WS0056_B11
6	25.914	114	1	0.248	0.044	4.338	2720	WS0056_B11
6	89.401	125	1	0.302	0.050	4.928	2720	WS0056_B11
6	95.249	126	1	-0.355	0.090	4.886	2720	WS0056_B11
8	50.001	154	1	-0.288	0.058	6.317	2720	WS0056_B11
9	3.127	168	1	0.482	0.149	6.531	2720	WS0056_B11
11	27.405	204	4	-0.305	0.066	6.281	2720	WS0056_B11
12	0.000	233	1	0.331	0.092	8.973	2720	WS0056_B11
13	3.386	245	1	-0.279	0.044	4.370	2720	WS0056_B11
1	95.572	17	2	0.233	0.040	4.305	2721	WS0056_F17
3	137.891	64	3	0.226	0.053	5.255	2721	WS0056_F17
4	173.929	88	2	-0.254	0.063	6.722	2721	WS0056_F17
7	15.555	130	2	-0.245	0.041	4.391	2721	WS0056_F17
9	28.608	170	3	-0.259	0.046	4.715	2721	WS0056_F17
9	49.013	173	1	0.245	0.074	5.527	2721	WS0056_F17
9	55.358	175	3	-0.508	0.115	4.651	2721	WS0056_F17
9	81.073	183	1	0.242	0.054	4.459	2721	WS0056_F17
10	9.580	187	3	0.382	0.107	6.198	2721	WS0056_F17
10	21.864	188	1	-0.301	0.078	6.475	2721	WS0056_F17
10	70.246	190	2	-0.249	0.049	5.284	2721	WS0056_F17
12	6.242	234	2	-0.257	0.060	7.911	2721	WS0056_F17
3	15.147	44	2	0.274	0.063	5.078	2722	WS0056_G14
4	173.929	88	2	-0.177	0.033	4.443	2722	WS0056_G14
5	57.017	102	2	-0.221	0.033	4.504	2722	WS0056_G14

10	78.479	193	3	0.380	0.093	7.965	2722	WS0056_G14
10	93.347	196	2	-0.498	0.118	4.863	2722	WS0056_G14
11	27.405	204	4	-0.321	0.073	4.082	2722	WS0056_G14
12	28.493	228	2	-0.248	0.050	4.270	2722	WS0056_G14
1	123.316	20	4	0.352	0.093	8.099	2723	WS0056_K06
1	131.686	23	2	-0.473	0.062	4.744	2723	WS0056_K06
2	127.147	40	4	-0.203	0.041	4.345	2723	WS0056_K06
3	80.211	55	4	0.291	0.069	7.704	2723	WS0056_K06
9	59.963	177	4	-0.255	0.050	5.141	2723	WS0056_K06
11	9.237	202	4	0.281	0.062	6.891	2723	WS0056_K06
11	27.405	204	4	-0.363	0.096	11.001	2723	WS0056_K06
11	60.464	221	2	0.242	0.041	4.896	2723	WS0056_K06
6	25.914	114	1	0.198	0.042	4.483	2724	WS0056_L07
8	50.001	154	1	-0.270	0.074	7.534	2724	WS0056_L07
8	78.389	157	1	-0.240	0.041	4.427	2724	WS0056_L07
8	87.463	158	1	0.234	0.039	4.263	2724	WS0056_L07
10	9.580	187	3	0.391	0.092	7.174	2724	WS0056_L07
3	15.147	44	2	0.272	0.061	4.514	2725	WS0056_O22
8	50.001	154	1	-0.271	0.068	5.097	2725	WS0056_O22
8	95.937	159	3	-0.238	0.067	5.983	2725	WS0056_O22
11	27.405	204	4	-0.276	0.054	5.064	2725	WS0056_O22
1	95.572	17	2	0.241	0.043	4.104	2726	WS0057_A11
2	75.550	31	1	0.220	0.044	4.016	2726	WS0057_A11
3	15.147	44	2	0.276	0.072	5.093	2726	WS0057_A11
4	173.929	88	2	-0.198	0.041	3.981	2726	WS0057_A11
6	25.914	114	1	0.226	0.044	3.986	2726	WS0057_A11
8	50.001	154	1	-0.336	0.102	9.205	2726	WS0057_A11
2	127.147	40	4	-0.191	0.044	4.223	2727	WS0092_F16
3	15.147	44	2	0.294	0.067	4.534	2727	WS0092_F16
3	137.891	64	3	0.230	0.054	4.945	2727	WS0092_F16
6	81.996	123	3	0.322	0.067	4.128	2727	WS0092_F16
8	50.001	154	1	-0.266	0.060	5.392	2727	WS0092_F16
10	21.864	188	1	-0.272	0.049	4.340	2727	WS0092_F16
2	48.839	29	4	0.279	0.053	4.879	2728	WS0092_K13
2	127.147	40	4	-0.241	0.052	5.138	2728	WS0092_K13
3	148.745	68	1	-0.254	0.059	5.187	2728	WS0092_K13
8	50.001	154	1	-0.318	0.101	9.090	2728	WS0092_K13
10	21.864	188	1	-0.242	0.049	4.310	2728	WS0092_K13
11	9.237	202	4	0.232	0.054	5.380	2728	WS0092_K13
11	27.405	204	1	0.239	0.056	4.949	2728	WS0092_K13
11	27.405	204	4	-0.355	0.094	9.338	2728	WS0092_K13
12	6.242	234	2	-0.215	0.042	5.899	2728	WS0092_K13
14	22.402	248	4	0.263	0.052	5.055	2728	WS0092_K13
1	15.589	3	2	0.142	0.039	3.855	2729	WS01013_E12
1	131.686	23	2	-0.417	0.072	4.182	2729	WS01013_E12
1	133.992	24	2	-0.273	0.057	5.721	2729	WS01013_E12
1	141.021	25	2	0.279	0.060	6.068	2729	WS01013_E12
3	141.443	65	3	-0.325	0.076	4.210	2729	WS01013_E12
5	54.072	100	4	0.174	0.049	4.494	2729	WS01013_E12
5	56.008	101	2	-0.220	0.043	3.920	2729	WS01013_E12
7	70.278	141	3	0.232	0.050	4.768	2729	WS01013_E12
8	50.001	154	2	-0.198	0.068	6.365	2729	WS01013_E12
12	28.493	228	2	0.327	0.152	8.852	2729	WS01013_E12
5	52.820	99	3	-0.266	0.038	3.973	2730	WS01038_O15

9	28.608	170	3	0.376	0.077	6.730	2730	WS01038_O15
10	78.479	193	3	-0.325	0.043	4.536	2730	WS01038_O15
10	93.347	196	1	0.430	0.073	4.438	2730	WS01038_O15
11	45.989	216	1	-0.216	0.042	4.166	2730	WS01038_O15
1	77.177	15	2	0.168	0.047	5.071	2731	WS01013_J18
1	95.572	17	2	0.351	0.092	10.160	2731	WS01013_J18
2	120.617	38	4	-0.275	0.059	6.792	2731	WS01013_J18
2	126.181	39	4	0.537	0.111	7.531	2731	WS01013_J18
2	127.147	40	4	-0.255	0.044	5.200	2731	WS01013_J18
3	15.147	44	2	0.423	0.136	9.792	2731	WS01013_J18
7	105.477	147	2	-0.300	0.073	5.509	2731	WS01013_J18
10	9.580	187	3	0.332	0.121	4.794	2731	WS01013_J18
10	35.204	189	2	0.201	0.050	5.494	2731	WS01013_J18
1	40.464	7	3	0.245	0.053	5.059	2732	WS0269_K20
1	43.578	8	3	0.260	0.060	6.180	2732	WS0269_K20
1	82.301	16	3	-0.214	0.052	4.951	2732	WS0269_K20
3	148.745	68	1	-0.208	0.054	5.573	2732	WS0269_K20
7	74.560	143	1	0.298	0.049	5.102	2732	WS0269_K20
9	28.608	170	3	0.420	0.122	9.927	2732	WS0269_K20
9	59.963	177	1	0.263	0.049	5.207	2732	WS0269_K20
10	93.347	196	1	0.521	0.103	6.509	2732	WS0269_K20
11	27.405	204	1	0.291	0.060	6.600	2732	WS0269_K20
11	45.989	216	1	-0.198	0.045	4.629	2732	WS0269_K20
11	56.921	219	1	-0.332	0.143	5.765	2732	WS0269_K20
11	60.464	221	2	0.273	0.090	6.847	2732	WS0269_K20
13	3.386	245	1	-0.183	0.050	5.106	2732	WS0269_K20
14	0.000	246	1	0.269	0.051	5.280	2732	WS0269_K20
1	43.578	8	2	0.229	0.037	4.439	2733	WS00712_C14
1	45.794	9	1	-0.264	0.088	6.212	2733	WS00712_C14
1	50.955	10	1	0.280	0.093	6.457	2733	WS00712_C14
1	107.333	19	3	0.225	0.055	5.086	2733	WS00712_C14
2	48.839	29	4	0.216	0.057	5.997	2733	WS00712_C14
5	16.219	93	2	0.285	0.044	5.035	2733	WS00712_C14
6	106.702	128	2	0.400	0.071	4.228	2733	WS00712_C14
10	70.246	190	4	0.272	0.074	6.164	2733	WS00712_C14
12	14.406	230	3	0.183	0.043	3.983	2733	WS00712_C14
1	22.419	4	3	0.229	0.055	3.935	2734	WS00712_I23
3	137.891	64	1	0.257	0.062	6.608	2734	WS00712_I23
11	0.000	199	2	-0.291	0.063	5.019	2734	WS00712_I23
11	45.989	216	1	-0.271	0.047	5.209	2734	WS00712_I23
14	44.898	251	3	0.262	0.048	5.182	2734	WS00712_I23
1	82.301	16	3	-0.330	0.072	7.125	2735	WS0094_N01
1	95.572	17	3	0.209	0.042	4.046	2735	WS0094_N01
1	107.333	19	3	0.237	0.062	6.100	2735	WS0094_N01
2	95.880	34	3	0.262	0.047	4.504	2735	WS0094_N01
6	106.702	128	2	0.448	0.091	5.070	2735	WS0094_N01
9	0.000	166	2	0.201	0.041	4.831	2735	WS0094_N01
9	59.963	177	4	-0.298	0.064	5.260	2735	WS0094_N01
10	9.580	187	2	0.393	0.108	7.159	2735	WS0094_N01
11	41.984	212	2	-0.326	0.088	10.568	2735	WS0094_N01
11	43.491	214	4	0.266	0.068	6.010	2735	WS0094_N01
11	45.989	216	2	-0.406	0.130	15.431	2735	WS0094_N01
11	60.464	221	2	-0.326	0.055	6.295	2735	WS0094_N01
6	89.401	125	2	0.229	0.049	4.590	2736	WS0057_E01

8	39.090	152	4	-0.164	0.043	3.845	2736	WS0057_E01
10	35.204	189	1	0.296	0.054	5.081	2736	WS0057_E01
11	37.143	209	3	0.215	0.040	3.908	2736	WS0057_E01
11	42.600	213	3	-0.236	0.045	4.465	2736	WS0057_E01
12	22.441	227	1	0.252	0.042	3.867	2736	WS0057_E01
2	93.504	32	4	0.297	0.041	4.384	2737	WS01017_A16
3	144.647	67	1	-0.279	0.059	3.846	2737	WS01017_A16
4	124.132	83	1	-0.521	0.094	6.176	2737	WS01017_A16
8	50.001	154	1	0.260	0.063	7.241	2737	WS01017_A16
9	59.963	177	4	-0.285	0.062	4.959	2737	WS01017_A16
11	64.014	222	4	0.279	0.056	4.697	2737	WS01017_A16
13	3.386	245	1	0.449	0.161	16.919	2737	WS01017_A16
3	9.953	42	3	0.261	0.056	4.498	2738	WS0057_N22
3	137.891	64	3	0.200	0.050	3.879	2738	WS0057_N22
4	6.032	72	4	0.231	0.039	4.239	2738	WS0057_N22
4	173.929	88	2	-0.223	0.049	5.884	2738	WS0057_N22
6	69.073	121	3	0.232	0.068	5.466	2738	WS0057_N22
6	81.996	123	3	0.392	0.150	7.084	2738	WS0057_N22
9	49.013	173	2	0.235	0.044	5.264	2738	WS0057_N22
11	60.464	221	2	0.341	0.077	9.172	2738	WS0057_N22
3	15.147	44	2	0.217	0.055	4.656	2739	WS00816_K15
3	116.958	63	4	-0.160	0.041	4.512	2739	WS00816_K15
3	148.745	68	4	-0.290	0.038	4.288	2739	WS00816_K15
6	54.066	119	4	0.183	0.042	4.680	2739	WS00816_K15
6	69.073	121	3	0.188	0.059	4.489	2739	WS00816_K15
7	70.278	141	3	-0.223	0.053	4.091	2739	WS00816_K15
7	75.883	144	2	0.391	0.051	4.142	2739	WS00816_K15
9	49.013	173	2	0.277	0.059	7.258	2739	WS00816_K15
1	131.686	23	2	-0.390	0.058	4.026	2740	WS00816_L04
3	116.958	63	4	-0.214	0.041	4.317	2740	WS00816_L04
5	7.672	92	4	0.329	0.068	5.374	2740	WS00816_L04
8	39.090	152	4	-0.158	0.038	4.025	2740	WS00816_L04
11	0.000	199	2	-0.316	0.072	6.646	2740	WS00816_L04
11	0.000	199	3	0.279	0.054	4.570	2740	WS00816_L04
11	17.088	203	1	0.261	0.050	4.966	2740	WS00816_L04
12	14.406	230	3	0.322	0.067	5.581	2740	WS00816_L04
12	6.242	234	4	0.297	0.036	5.091	2740	WS00816_L04
13	0.000	243	4	0.354	0.166	5.946	2740	WS00816_L04
1	107.333	19	4	-0.240	0.043	4.384	2741	WS00816_O16
3	9.953	42	3	0.276	0.075	6.240	2741	WS00816_O16
3	137.891	64	3	0.219	0.048	3.998	2741	WS00816_O16
8	39.090	152	4	-0.260	0.053	5.557	2741	WS00816_O16
9	36.227	165	4	0.269	0.045	4.240	2741	WS00816_O16
11	0.000	199	3	-0.222	0.045	3.847	2741	WS00816_O16
11	45.989	216	1	-0.204	0.056	4.402	2741	WS00816_O16
11	45.989	216	2	0.201	0.044	4.804	2741	WS00816_O16
11	60.464	221	2	0.284	0.061	6.537	2741	WS00816_O16
12	22.441	227	1	0.312	0.064	5.061	2741	WS00816_O16
1	2.788	2	3	-0.265	0.054	5.104	2742	WS00816_O24
2	63.261	30	3	-0.276	0.087	7.804	2742	WS00816_O24
3	137.891	64	3	0.189	0.045	3.898	2742	WS00816_O24
4	173.929	88	2	-0.317	0.077	6.955	2742	WS00816_O24
7	75.883	144	2	0.565	0.149	9.200	2742	WS00816_O24
7	75.883	144	4	-0.319	0.057	5.469	2742	WS00816_O24

8	62.580	155	1	-0.385	0.098	4.247	2742	WS00816_O24
9	55.358	175	1	0.317	0.047	4.773	2742	WS00816_O24
9	55.358	175	3	-0.391	0.068	4.168	2742	WS00816_O24
11	43.491	214	3	-0.211	0.047	4.285	2742	WS00816_O24
14	46.637	252	4	0.329	0.059	5.333	2742	WS00816_O24
3	144.593	66	4	0.267	0.068	8.379	2743	WS00817_B24
3	148.745	68	4	-0.371	0.066	4.757	2743	WS00817_B24
3	154.505	69	4	0.324	0.061	6.133	2743	WS00817_B24
4	97.576	80	2	0.205	0.034	4.688	2743	WS00817_B24
9	36.227	165	4	0.234	0.045	4.585	2743	WS00817_B24
1	45.794	9	3	-0.193	0.032	3.948	2744	WS00817_C19
6	89.401	125	1	-0.260	0.049	3.999	2744	WS00817_C19
9	28.608	170	2	-0.224	0.044	4.874	2744	WS00817_C19
9	28.608	170	3	0.300	0.038	4.832	2744	WS00817_C19
10	93.347	196	1	0.408	0.069	4.002	2744	WS00817_C19
11	45.989	216	1	-0.226	0.085	6.951	2744	WS00817_C19
3	83.563	56	3	-0.181	0.034	3.960	2745	WS00817_F04
8	62.580	155	1	-0.314	0.117	4.133	2745	WS00817_F04
8	78.389	157	1	-0.300	0.077	6.749	2745	WS00817_F04
8	87.463	158	1	0.278	0.070	6.196	2745	WS00817_F04
9	17.951	169	1	0.354	0.074	6.539	2745	WS00817_F04
10	78.479	193	3	0.247	0.035	3.952	2745	WS00817_F04
11	43.491	214	3	-0.379	0.120	13.857	2745	WS00817_F04
11	44.884	215	3	0.367	0.078	9.031	2745	WS00817_F04
11	64.014	222	3	0.317	0.061	7.057	2745	WS00817_F04
13	1.815	244	2	-0.214	0.042	4.214	2745	WS00817_F04
1	123.316	20	4	-0.395	0.113	7.320	2746	WS00817_G05
3	104.681	60	2	-0.195	0.035	4.114	2746	WS00817_G05
4	43.146	74	3	0.278	0.067	6.191	2746	WS00817_G05
5	3.484	90	2	-0.316	0.076	4.950	2746	WS00817_G05
6	87.891	124	2	0.252	0.057	6.783	2746	WS00817_G05
6	89.401	125	2	0.216	0.033	3.907	2746	WS00817_G05
7	59.789	137	3	-0.252	0.054	4.959	2746	WS00817_G05
7	73.169	142	2	-0.433	0.070	3.897	2746	WS00817_G05
7	75.883	144	2	0.503	0.101	6.352	2746	WS00817_G05
7	91.487	146	3	-0.203	0.050	4.568	2746	WS00817_G05
8	11.974	150	1	-0.238	0.062	4.340	2746	WS00817_G05
10	21.864	188	1	0.218	0.066	4.591	2746	WS00817_G05
10	78.479	193	3	0.316	0.049	4.516	2746	WS00817_G05
1	29.800	5	1	0.243	0.042	4.315	2747	WS00946_B20
1	133.992	24	2	0.235	0.052	4.497	2747	WS00946_B20
1	141.021	25	2	-0.278	0.077	6.658	2747	WS00946_B20
2	63.261	30	3	-0.198	0.047	5.155	2747	WS00946_B20
5	57.017	102	2	-0.242	0.052	4.435	2747	WS00946_B20
6	49.100	118	2	0.358	0.125	5.451	2747	WS00946_B20
6	54.066	119	4	-0.263	0.061	4.778	2747	WS00946_B20
7	74.560	143	1	-0.165	0.039	3.929	2747	WS00946_B20
9	58.045	176	1	0.230	0.061	4.034	2747	WS00946_B20
11	0.000	199	3	-0.298	0.050	4.327	2747	WS00946_B20
1	123.316	20	4	-0.302	0.082	4.460	2748	WS00817_L04
3	14.500	43	1	-0.281	0.052	4.025	2748	WS00817_L04
3	17.158	45	1	0.244	0.062	4.757	2748	WS00817_L04
4	116.928	82	2	0.215	0.039	4.458	2748	WS00817_L04
9	58.045	176	1	0.357	0.080	4.009	2748	WS00817_L04

9	69.046	181	1	-0.225	0.093	4.486	2748	WS00817_L04
1	45.794	9	1	0.383	0.108	9.886	2749	WS00946_L07
1	50.955	10	1	-0.228	0.043	3.970	2749	WS00946_L07
1	107.333	19	4	-0.199	0.049	5.549	2749	WS00946_L07
1	123.316	20	4	-0.394	0.132	7.696	2749	WS00946_L07
1	131.686	23	2	0.644	0.173	7.473	2749	WS00946_L07
3	9.953	42	4	0.225	0.051	5.469	2749	WS00946_L07
3	80.211	55	4	-0.189	0.042	4.639	2749	WS00946_L07
4	92.039	79	2	-0.227	0.043	4.163	2749	WS00946_L07
4	92.039	79	4	0.268	0.038	4.204	2749	WS00946_L07
6	69.073	121	3	0.240	0.051	3.981	2749	WS00946_L07
7	105.477	147	2	-0.414	0.138	8.036	2749	WS00946_L07
8	50.001	154	1	0.248	0.068	6.863	2749	WS00946_L07
8	62.580	155	1	0.527	0.180	9.377	2749	WS00946_L07
8	78.389	157	1	0.312	0.090	8.211	2749	WS00946_L07
8	87.463	158	1	-0.251	0.058	5.318	2749	WS00946_L07
10	9.580	187	2	0.282	0.080	4.643	2749	WS00946_L07
12	0.000	233	1	-0.191	0.045	4.101	2749	WS00946_L07
12	22.629	237	1	-0.255	0.070	6.427	2749	WS00946_L07
12	32.473	238	1	-0.206	0.046	4.208	2749	WS00946_L07
13	1.815	244	4	-0.301	0.050	3.940	2749	WS00946_L07
1	123.316	20	4	-0.326	0.109	5.099	2750	WS00818_B07
2	48.839	29	4	-0.289	0.067	5.241	2750	WS00818_B07
3	154.505	69	1	0.232	0.056	4.780	2750	WS00818_B07
4	82.462	78	1	-0.233	0.062	5.296	2750	WS00818_B07
7	17.610	131	3	-0.306	0.069	3.907	2750	WS00818_B07
12	14.406	230	3	-0.223	0.042	4.006	2750	WS00818_B07
1	43.578	8	2	0.262	0.035	3.922	2751	WS00922_I09
5	54.072	100	4	0.238	0.048	4.617	2751	WS00922_I09
7	17.610	131	3	-0.326	0.155	5.659	2751	WS00922_I09
7	74.560	143	1	-0.218	0.054	4.233	2751	WS00922_I09
7	75.883	144	2	0.460	0.077	5.930	2751	WS00922_I09
1	43.578	8	2	0.276	0.037	3.868	2752	WS00922_L23
1	45.794	9	1	0.333	0.086	7.094	2752	WS00922_L23
4	173.929	88	2	-0.251	0.062	6.575	2752	WS00922_L23
6	106.702	128	2	-0.405	0.073	4.352	2752	WS00922_L23
11	56.921	219	2	-0.285	0.111	5.994	2752	WS00922_L23
3	15.147	44	2	0.298	0.107	5.781	2753	WS00921_I12
4	6.032	72	4	0.230	0.068	5.510	2753	WS00921_I12
6	81.996	123	3	0.299	0.073	4.579	2753	WS00921_I12
10	70.246	190	2	-0.295	0.071	7.338	2753	WS00921_I12
10	93.347	196	2	-0.480	0.123	5.294	2753	WS00921_I12
11	0.000	199	2	0.298	0.043	4.321	2753	WS00921_I12
14	0.000	246	2	-0.242	0.057	5.450	2753	WS00921_I12
3	137.891	64	3	0.298	0.062	4.934	2754	WS00921_I21
5	54.072	100	4	0.285	0.063	7.312	2754	WS00921_I21
7	105.477	147	2	0.335	0.047	4.666	2754	WS00921_I21
10	78.479	193	3	0.331	0.084	6.297	2754	WS00921_I21
13	1.815	244	2	0.249	0.044	6.067	2754	WS00921_I21
1	22.419	4	3	0.282	0.130	5.706	2755	WS00921_K02
4	82.462	78	1	-0.167	0.054	4.251	2755	WS00921_K02
5	7.672	92	4	-0.289	0.068	4.721	2755	WS00921_K02
7	17.610	131	3	0.340	0.185	6.520	2755	WS00921_K02
7	105.477	147	2	0.238	0.060	4.345	2755	WS00921_K02

9	0.000	163	1	0.483	0.117	5.153	2755	WS00921_K02
9	17.951	169	1	-0.299	0.094	7.453	2755	WS00921_K02
9	17.951	169	2	0.301	0.053	5.150	2755	WS00921_K02
9	81.073	183	3	-0.179	0.050	3.995	2755	WS00921_K02
14	22.402	248	4	-0.521	0.108	10.751	2755	WS00921_K02
14	44.898	251	3	0.219	0.068	5.097	2755	WS00921_K02
14	46.637	252	3	0.396	0.095	7.719	2755	WS00921_K02
14	46.637	252	4	-0.315	0.042	4.701	2755	WS00921_K02
1	131.686	23	2	-0.494	0.090	5.083	2756	WS00921_K15
2	127.147	40	4	-0.195	0.041	4.278	2756	WS00921_K15
3	116.958	63	4	-0.184	0.052	5.402	2756	WS00921_K15
6	56.188	120	2	-0.251	0.039	4.067	2756	WS00921_K15
12	14.406	230	4	0.237	0.045	4.905	2756	WS00921_K15
3	9.953	42	3	0.288	0.078	7.006	2757	WS00921_M15
4	173.929	88	2	-0.183	0.041	4.972	2757	WS00921_M15
6	81.996	123	3	0.330	0.131	6.349	2757	WS00921_M15
8	39.090	152	4	-0.245	0.048	4.752	2757	WS00921_M15
9	49.013	173	2	0.199	0.041	4.478	2757	WS00921_M15
11	60.464	221	2	0.204	0.040	4.353	2757	WS00921_M15
6	56.188	120	1	0.245	0.059	4.700	2758	WS00921_N03
6	87.891	124	2	-0.252	0.061	6.619	2758	WS00921_N03
6	89.401	125	2	-0.277	0.056	5.909	2758	WS00921_N03
7	105.477	147	2	0.311	0.075	3.864	2758	WS00921_N03
9	3.127	168	1	0.357	0.099	4.056	2758	WS00921_N03
4	0.000	71	4	0.278	0.052	4.997	2759	WS00921_O18
5	86.669	106	2	-0.248	0.067	4.233	2759	WS00921_O18
10	78.479	193	3	0.231	0.044	4.080	2759	WS00921_O18
11	43.491	214	3	-0.233	0.052	4.889	2759	WS00921_O18
11	60.464	221	2	0.175	0.028	3.880	2759	WS00921_O18
13	1.815	244	2	-0.185	0.031	4.224	2759	WS00921_O18
6	54.066	119	4	0.177	0.053	4.942	2760	WS00921_P06
6	56.188	120	2	-0.293	0.046	4.142	2760	WS00921_P06
9	49.013	173	1	0.265	0.081	6.249	2760	WS00921_P06
11	45.989	216	1	-0.269	0.083	6.397	2760	WS00921_P06
12	22.441	227	3	0.325	0.049	3.933	2760	WS00921_P06
1	127.805	21	4	-0.183	0.034	4.319	2761	WS00815_E23
3	144.593	66	4	0.191	0.035	4.474	2761	WS00815_E23
5	57.017	102	2	-0.228	0.047	5.458	2761	WS00815_E23
6	89.401	125	2	0.226	0.036	3.953	2761	WS00815_E23
7	74.560	143	1	-0.221	0.052	4.023	2761	WS00815_E23
14	0.000	246	2	-0.251	0.056	5.961	2761	WS00815_E23
1	2.788	2	4	-0.145	0.027	4.010	2762	WS00815_K09
4	103.719	81	4	-0.257	0.046	4.976	2762	WS00815_K09
5	42.979	97	1	-0.526	0.139	11.067	2762	WS00815_K09
5	52.820	99	1	-0.463	0.132	10.530	2762	WS00815_K09
5	56.008	101	1	0.322	0.125	9.888	2762	WS00815_K09
6	26.350	115	2	-0.162	0.047	5.147	2762	WS00815_K09
6	56.188	120	1	-0.236	0.046	3.891	2762	WS00815_K09
8	50.001	154	2	-0.208	0.031	4.288	2762	WS00815_K09
10	21.864	188	1	0.241	0.072	5.709	2762	WS00815_K09
11	0.000	199	2	0.239	0.041	4.482	2762	WS00815_K09
12	14.406	230	4	0.186	0.034	4.026	2762	WS00815_K09
1	95.572	17	2	-0.365	0.073	8.514	2763	WS00715_E12
4	6.032	72	4	0.249	0.039	4.330	2763	WS00715_E12

4	103.719	81	4	0.240	0.044	5.095	2763	WS00715_E12
6	81.996	123	3	-0.339	0.129	5.304	2763	WS00715_E12
11	0.000	199	3	-0.299	0.057	4.072	2763	WS00715_E12
11	43.491	214	4	0.279	0.053	5.845	2763	WS00715_E12
11	57.661	220	4	-0.198	0.057	6.208	2763	WS00715_E12
11	64.014	222	4	0.255	0.072	7.352	2763	WS00715_E12
2	120.617	38	4	-0.252	0.051	4.180	2764	WS01021_P20
3	141.443	65	3	0.398	0.144	7.360	2764	WS01021_P20
7	15.555	130	2	0.163	0.038	3.918	2764	WS01021_P20
9	49.013	173	1	0.281	0.084	8.300	2764	WS01021_P20
9	69.046	181	1	-0.312	0.078	4.008	2764	WS01021_P20
11	35.604	207	3	0.229	0.036	3.858	2764	WS01021_P20
11	45.989	216	1	-0.206	0.051	4.341	2764	WS01021_P20
11	56.921	219	2	-0.235	0.085	4.581	2764	WS01021_P20
13	1.815	244	2	-0.152	0.038	3.964	2764	WS01021_P20
14	22.402	248	3	0.287	0.079	8.189	2764	WS01021_P20
5	16.219	93	2	0.263	0.043	4.397	2765	WS00715_M14
7	74.560	143	1	-0.199	0.050	4.305	2765	WS00715_M14
1	107.333	19	4	-0.226	0.042	4.014	2766	WS00921_E04
1	127.805	21	4	0.202	0.045	4.042	2766	WS00921_E04
3	17.158	45	1	0.237	0.059	4.736	2766	WS00921_E04
4	116.928	82	2	0.244	0.049	5.281	2766	WS00921_E04
8	78.389	157	1	0.207	0.048	3.893	2766	WS00921_E04
10	35.204	189	1	-0.295	0.052	4.212	2766	WS00921_E04
10	93.347	196	1	0.457	0.112	5.023	2766	WS00921_E04
1	131.686	23	1	-0.556	0.102	5.312	2767	WS00815_O06
3	47.928	49	1	-0.254	0.047	4.626	2767	WS00815_O06
7	75.883	144	4	-0.324	0.057	4.506	2767	WS00815_O06
8	39.090	152	4	-0.290	0.064	6.435	2767	WS00815_O06
9	36.227	165	3	-0.249	0.055	4.930	2767	WS00815_O06
11	40.960	210	2	-0.408	0.096	4.982	2767	WS00815_O06
11	40.960	210	4	0.276	0.042	4.228	2767	WS00815_O06
1	29.800	5	2	0.204	0.035	3.987	2768	WS00815_O21
1	40.464	7	4	0.255	0.045	4.075	2768	WS00815_O21
1	107.333	19	4	-0.230	0.039	3.912	2768	WS00815_O21
4	0.000	71	4	-0.157	0.035	3.863	2768	WS00815_O21
6	45.897	117	1	0.238	0.073	5.922	2768	WS00815_O21
8	95.937	159	3	0.271	0.078	6.053	2768	WS00815_O21
10	9.580	187	3	-0.311	0.108	3.981	2768	WS00815_O21
11	64.014	222	4	-0.268	0.049	5.480	2768	WS00815_O21
2	120.617	38	3	0.277	0.058	6.648	2769	WS0061_A16
2	127.147	40	3	-0.195	0.047	5.349	2769	WS0061_A16
3	95.157	59	2	0.187	0.042	3.968	2769	WS0061_A16
3	137.891	64	1	-0.298	0.044	4.335	2769	WS0061_A16
3	154.505	69	1	0.329	0.084	8.379	2769	WS0061_A16
6	95.249	126	1	0.289	0.070	5.141	2769	WS0061_A16
9	36.227	165	3	-0.247	0.039	4.499	2769	WS0061_A16
10	9.580	187	3	-0.522	0.183	13.413	2769	WS0061_A16
10	93.347	196	1	0.446	0.067	5.002	2769	WS0061_A16
11	35.604	207	3	-0.165	0.036	4.121	2769	WS0061_A16
11	37.143	209	3	0.182	0.043	4.856	2769	WS0061_A16
11	42.600	213	3	-0.169	0.035	3.979	2769	WS0061_A16
11	56.921	219	1	-0.286	0.207	4.867	2769	WS0061_A16
1	43.578	8	2	0.160	0.031	3.873	2770	WS0061_B13

6	56.188	120	2	0.303	0.051	5.672	2770	WS0061_B13
9	17.951	169	2	0.299	0.045	5.904	2770	WS0061_B13
10	21.864	188	1	0.344	0.070	4.617	2770	WS0061_B13
11	45.989	216	1	-0.261	0.069	4.353	2770	WS0061_B13
14	43.392	250	2	0.265	0.049	5.436	2770	WS0061_B13
1	71.592	14	2	0.160	0.054	4.083	2771	WS0061_G14
1	77.177	15	2	0.193	0.074	5.649	2771	WS0061_G14
4	82.462	78	1	-0.241	0.061	6.751	2771	WS0061_G14
4	124.132	83	1	-0.735	0.160	12.269	2771	WS0061_G14
4	142.917	85	1	0.405	0.089	9.802	2771	WS0061_G14
5	54.072	100	4	0.211	0.045	5.155	2771	WS0061_G14
5	57.017	102	4	-0.327	0.084	7.241	2771	WS0061_G14
6	56.188	120	1	0.258	0.048	5.348	2771	WS0061_G14
6	76.376	122	1	-0.413	0.082	6.314	2771	WS0061_G14
6	89.401	125	1	0.261	0.039	4.338	2771	WS0061_G14
8	0.000	148	4	0.206	0.041	4.633	2771	WS0061_G14
9	36.227	165	4	-0.274	0.040	4.150	2771	WS0061_G14
12	38.543	232	4	-0.281	0.070	5.273	2771	WS0061_G14
2	93.504	32	4	0.277	0.043	4.278	2772	WS0061_I04
4	82.462	78	1	-0.276	0.071	7.177	2772	WS0061_I04
4	124.132	83	1	-0.696	0.172	10.694	2772	WS0061_I04
4	142.917	85	1	0.475	0.120	12.595	2772	WS0061_I04
5	52.820	99	1	0.266	0.039	4.049	2772	WS0061_I04
8	62.580	155	1	-0.332	0.085	5.170	2772	WS0061_I04
8	87.463	158	2	-0.238	0.082	6.404	2772	WS0061_I04
11	43.491	214	3	-0.234	0.058	5.222	2772	WS0061_I04
14	44.898	251	3	0.339	0.085	6.235	2772	WS0061_I04
1	95.572	17	3	0.293	0.080	5.864	2773	WS0061_P08
1	107.333	19	3	0.241	0.052	4.398	2773	WS0061_P08
3	137.891	64	3	0.311	0.086	7.182	2773	WS0061_P08
6	54.066	119	4	0.226	0.058	6.021	2773	WS0061_P08
6	69.073	121	4	-0.317	0.086	8.619	2773	WS0061_P08
7	73.169	142	2	-0.512	0.082	6.563	2773	WS0061_P08
7	75.883	144	2	0.428	0.062	4.775	2773	WS0061_P08
9	28.608	170	3	0.278	0.070	5.524	2773	WS0061_P08
9	55.358	175	3	0.491	0.113	5.931	2773	WS0061_P08
10	70.246	190	4	-0.359	0.074	7.517	2773	WS0061_P08
10	78.479	193	4	-0.313	0.105	10.956	2773	WS0061_P08
13	0.000	243	4	0.415	0.228	5.488	2773	WS0061_P08
1	133.992	24	2	-0.204	0.032	4.085	2774	WS0062_B11
1	141.021	25	2	0.197	0.031	3.946	2774	WS0062_B11
6	106.702	128	2	-0.531	0.136	7.416	2774	WS0062_B11
11	0.000	199	3	-0.183	0.050	3.937	2774	WS0062_B11
11	56.921	219	2	-0.328	0.085	3.898	2774	WS0062_B11
12	6.242	234	4	0.324	0.047	6.064	2774	WS0062_B11
14	22.402	248	3	0.299	0.053	4.372	2774	WS0062_B11
3	9.953	42	3	0.255	0.068	7.743	2775	WS0062_E09
6	69.073	121	4	-0.249	0.054	3.995	2775	WS0062_E09
6	106.702	128	2	0.398	0.110	4.377	2775	WS0062_E09
7	15.555	130	3	-0.286	0.069	6.668	2775	WS0062_E09
9	0.000	166	1	-0.209	0.051	4.880	2775	WS0062_E09
9	28.608	170	3	0.298	0.058	6.645	2775	WS0062_E09
9	55.358	175	3	0.430	0.073	5.130	2775	WS0062_E09
10	9.580	187	3	-0.419	0.103	7.896	2775	WS0062_E09

10	70.246	190	2	0.310	0.069	6.335	2775	WS0062_E09
10	77.676	192	1	-0.206	0.051	4.875	2775	WS0062_E09
10	78.479	193	4	-0.244	0.070	4.604	2775	WS0062_E09
10	80.088	194	1	-0.260	0.064	6.105	2775	WS0062_E09
10	80.088	194	2	-0.326	0.076	7.605	2775	WS0062_E09
10	104.214	198	2	-0.223	0.035	4.684	2775	WS0062_E09
11	45.989	216	1	-0.213	0.051	4.903	2775	WS0062_E09
2	48.839	29	1	-0.311	0.087	8.771	2776	WS0062_G11
2	48.839	29	4	0.348	0.113	10.101	2776	WS0062_G11
3	141.443	65	3	-0.346	0.088	5.907	2776	WS0062_G11
3	144.593	66	4	0.230	0.084	6.877	2776	WS0062_G11
3	148.745	68	1	-0.280	0.041	4.038	2776	WS0062_G11
3	148.745	68	4	-0.276	0.066	5.701	2776	WS0062_G11
3	154.505	69	1	-0.298	0.055	5.537	2776	WS0062_G11
3	154.505	69	4	0.281	0.080	7.162	2776	WS0062_G11
5	0.000	89	1	-0.507	0.143	14.803	2776	WS0062_G11
5	0.000	89	3	0.246	0.047	4.876	2776	WS0062_G11
5	7.672	92	1	-0.291	0.047	5.111	2776	WS0062_G11
5	16.219	93	1	-0.367	0.097	9.637	2776	WS0062_G11
7	67.154	140	4	-0.410	0.124	4.194	2776	WS0062_G11
7	75.883	144	2	0.461	0.092	5.388	2776	WS0062_G11
7	75.883	144	4	-0.218	0.053	4.558	2776	WS0062_G11
14	9.923	247	1	-0.209	0.041	4.036	2776	WS0062_G11
1	82.301	16	4	0.265	0.054	5.045	2777	WS0062_N16
3	95.157	59	2	-0.208	0.061	9.037	2777	WS0062_N16
3	104.681	60	2	0.318	0.051	8.113	2777	WS0062_N16
3	114.747	62	2	-0.221	0.033	5.130	2777	WS0062_N16
5	42.979	97	2	-0.342	0.052	8.731	2777	WS0062_N16
9	28.608	170	2	-0.223	0.046	6.974	2777	WS0062_N16
9	32.356	171	2	-0.165	0.044	6.814	2777	WS0062_N16
10	70.246	190	2	-0.163	0.030	4.631	2777	WS0062_N16
10	80.088	194	2	0.332	0.040	5.687	2777	WS0062_N16
10	104.214	198	2	0.239	0.026	3.997	2777	WS0062_N16
12	28.493	228	2	-0.357	0.069	5.424	2777	WS0062_N16
14	0.000	246	2	-0.121	0.028	4.431	2777	WS0062_N16
1	40.464	7	3	0.220	0.043	3.863	2778	WS0063_A07
1	131.686	23	2	-0.423	0.069	3.841	2778	WS0063_A07
4	92.039	79	4	0.274	0.050	4.171	2778	WS0063_A07
6	25.914	114	1	-0.248	0.066	4.711	2778	WS0063_A07
10	9.580	187	3	-0.316	0.125	4.411	2778	WS0063_A07
11	0.000	199	2	-0.289	0.074	8.551	2778	WS0063_A07
11	60.464	221	2	0.190	0.039	4.452	2778	WS0063_A07
12	6.242	234	4	0.298	0.053	4.073	2778	WS0063_A07
1	43.578	8	2	0.153	0.042	4.973	2779	WS0063_C22
4	82.462	78	2	0.236	0.030	3.959	2779	WS0063_C22
5	0.000	89	1	-0.330	0.103	7.247	2779	WS0063_C22
5	16.219	93	1	-0.323	0.119	8.129	2779	WS0063_C22
5	54.072	100	4	0.122	0.037	4.120	2779	WS0063_C22
6	49.100	118	4	0.404	0.276	7.952	2779	WS0063_C22
9	28.608	170	2	0.350	0.068	8.443	2779	WS0063_C22
9	32.356	171	2	0.213	0.035	4.182	2779	WS0063_C22
9	49.013	173	2	-0.285	0.080	9.736	2779	WS0063_C22
10	86.151	195	3	-0.123	0.054	4.060	2779	WS0063_C22
11	40.960	210	2	0.608	0.129	11.360	2779	WS0063_C22

13	1.815	244	4	0.243	0.047	5.606	2779	WS0063_C22
4	140.962	84	3	-0.342	0.129	9.829	2780	WS0063_E07
4	142.917	85	3	-0.340	0.109	8.135	2780	WS0063_E07
7	15.555	130	3	-0.326	0.077	6.127	2780	WS0063_E07
11	46.026	217	1	-0.219	0.045	4.098	2780	WS0063_E07
11	60.464	221	2	0.222	0.043	4.176	2780	WS0063_E07
3	9.953	42	4	0.262	0.051	4.822	2781	WS0063_I01
3	137.891	64	1	-0.264	0.059	6.064	2781	WS0063_I01
5	52.820	99	3	-0.188	0.042	3.861	2781	WS0063_I01
6	45.897	117	1	0.296	0.052	5.639	2781	WS0063_I01
6	69.073	121	3	0.285	0.045	4.392	2781	WS0063_I01
9	28.608	170	2	0.309	0.067	6.009	2781	WS0063_I01
11	17.088	203	1	0.267	0.046	4.755	2781	WS0063_I01
11	40.960	210	2	0.374	0.069	4.012	2781	WS0063_I01
12	6.242	234	4	0.299	0.051	4.539	2781	WS0063_I01
13	1.815	244	4	0.344	0.109	9.853	2781	WS0063_I01
5	54.072	100	4	0.228	0.043	4.034	2782	WS0063_I19
6	54.066	119	4	0.170	0.043	3.956	2782	WS0063_I19
6	81.996	123	3	0.407	0.130	7.005	2782	WS0063_I19
7	75.883	144	2	0.465	0.093	5.746	2782	WS0063_I19
11	60.464	221	2	0.278	0.063	5.819	2782	WS0063_I19
1	107.333	19	3	0.235	0.058	5.182	2783	WS0089_N10
2	75.550	31	2	-0.206	0.044	4.538	2783	WS0089_N10
3	104.681	60	2	-0.219	0.037	4.239	2783	WS0089_N10
3	112.868	61	2	0.207	0.034	3.908	2783	WS0089_N10
7	62.787	139	2	-0.224	0.040	4.690	2783	WS0089_N10
9	3.127	168	1	0.369	0.144	4.632	2783	WS0089_N10
14	43.392	250	2	0.235	0.063	6.088	2783	WS0089_N10
1	40.464	7	3	0.200	0.043	4.121	2784	WS00928_O06
1	40.464	7	4	0.273	0.051	4.696	2784	WS00928_O06
1	45.794	9	3	-0.206	0.059	5.718	2784	WS00928_O06
1	62.128	11	1	-0.749	0.205	10.859	2784	WS00928_O06
5	42.979	97	1	-0.290	0.043	4.291	2784	WS00928_O06
6	25.742	113	3	-0.187	0.040	3.846	2784	WS00928_O06
6	25.914	114	1	-0.283	0.060	5.900	2784	WS00928_O06
6	69.073	121	4	-0.254	0.058	4.455	2784	WS00928_O06
9	28.608	170	3	0.330	0.071	6.886	2784	WS00928_O06
10	70.246	190	4	-0.312	0.062	4.970	2784	WS00928_O06
10	78.479	193	4	-0.238	0.060	5.716	2784	WS00928_O06
10	93.347	196	1	0.432	0.098	4.479	2784	WS00928_O06
12	22.441	227	1	0.295	0.052	5.121	2784	WS00928_O06
2	93.504	32	4	0.337	0.086	9.033	2785	WS00810_C06
4	75.958	77	3	-0.332	0.088	4.177	2785	WS00810_C06
6	69.073	121	4	-0.292	0.075	7.019	2785	WS00810_C06
9	59.963	177	4	-0.295	0.054	5.253	2785	WS00810_C06
9	61.113	178	1	-0.370	0.096	4.297	2785	WS00810_C06
10	9.580	187	2	-0.263	0.086	5.062	2785	WS00810_C06
11	56.921	219	1	-0.341	0.097	4.337	2785	WS00810_C06
11	60.464	221	2	0.313	0.084	8.955	2785	WS00810_C06
2	120.617	38	4	-0.240	0.060	5.220	2786	WS00929_B12
6	25.742	113	3	-0.217	0.041	4.233	2786	WS00929_B12
6	87.891	124	2	0.177	0.067	5.010	2786	WS00929_B12
6	89.401	125	2	0.287	0.077	5.828	2786	WS00929_B12
10	9.580	187	3	-0.296	0.090	5.989	2786	WS00929_B12

10	77.676	192	1	-0.205	0.036	4.030	2786	WS00929_B12
10	80.088	194	1	-0.372	0.066	7.580	2786	WS00929_B12
10	93.347	196	1	0.473	0.079	5.389	2786	WS00929_B12
1	45.794	9	1	0.290	0.079	7.751	2787	WS01021_G19
1	50.955	10	1	-0.219	0.043	4.234	2787	WS01021_G19
1	62.128	11	1	-0.480	0.097	4.544	2787	WS01021_G19
2	48.839	29	4	-0.323	0.077	8.372	2787	WS01021_G19
7	59.789	137	3	-0.253	0.046	4.167	2787	WS01021_G19
7	70.278	141	3	0.267	0.056	5.041	2787	WS01021_G19
9	28.608	170	3	-0.280	0.061	4.028	2787	WS01021_G19
12	6.242	234	2	-0.285	0.048	4.829	2787	WS01021_G19
4	6.032	72	4	0.169	0.058	4.566	2788	WS01021_K16
10	78.479	193	3	0.278	0.038	4.213	2788	WS01021_K16
11	29.069	205	1	-0.295	0.045	3.998	2788	WS01021_K16
11	45.989	216	1	0.222	0.061	5.097	2788	WS01021_K16
1	107.333	19	4	-0.235	0.038	4.312	2789	WS00818_F05
1	123.316	20	4	-0.264	0.069	5.350	2789	WS00818_F05
8	39.090	152	4	-0.243	0.040	4.650	2789	WS00818_F05
9	32.356	171	2	0.208	0.032	3.954	2789	WS00818_F05
1	2.788	2	4	-0.245	0.038	3.973	2790	WS00818_F21
4	82.462	78	1	-0.253	0.059	5.384	2790	WS00818_F21
5	52.820	99	3	-0.234	0.055	4.467	2790	WS00818_F21
8	62.580	155	1	0.296	0.093	4.040	2790	WS00818_F21
4	97.576	80	2	-0.219	0.040	3.942	2791	WS00818_I10
4	116.928	82	2	-0.359	0.058	6.870	2791	WS00818_I10
4	124.132	83	2	-0.424	0.165	4.888	2791	WS00818_I10
7	75.883	144	2	0.413	0.068	5.318	2791	WS00818_I10
9	28.608	170	2	-0.269	0.047	5.478	2791	WS00818_I10
11	40.960	210	2	-0.479	0.081	6.566	2791	WS00818_I10
14	46.637	252	4	0.284	0.037	4.441	2791	WS00818_I10
3	104.681	60	2	0.254	0.080	7.592	2792	WS00818_I21
3	112.868	61	2	-0.195	0.066	6.292	2792	WS00818_I21
3	114.747	62	2	-0.275	0.063	6.040	2792	WS00818_I21
4	55.925	76	1	-0.313	0.080	6.594	2792	WS00818_I21
4	82.462	78	1	-0.244	0.086	7.929	2792	WS00818_I21
4	142.917	85	1	0.246	0.048	4.438	2792	WS00818_I21
5	0.000	89	1	-0.362	0.061	5.708	2792	WS00818_I21
5	16.219	93	1	-0.304	0.096	8.943	2792	WS00818_I21
5	31.191	95	1	-0.302	0.080	7.417	2792	WS00818_I21
6	106.702	128	2	0.466	0.112	5.305	2792	WS00818_I21
14	44.898	251	1	-0.186	0.045	4.215	2792	WS00818_I21
1	69.854	13	2	0.221	0.061	6.620	2793	WS00818_K13
1	71.592	14	2	0.221	0.062	6.698	2793	WS00818_K13
1	77.177	15	2	0.228	0.065	7.066	2793	WS00818_K13
4	0.000	71	4	-0.154	0.041	4.066	2793	WS00818_K13
6	81.996	123	3	0.374	0.116	5.875	2793	WS00818_K13
7	75.883	144	2	0.402	0.062	4.298	2793	WS00818_K13
14	0.000	246	2	-0.180	0.039	4.206	2793	WS00818_K13
4	97.576	80	2	-0.223	0.034	4.618	2794	WS00818_L04
4	142.917	85	1	-0.240	0.053	4.737	2794	WS00818_L04
5	7.672	92	1	-0.282	0.062	6.106	2794	WS00818_L04
6	54.066	119	4	0.195	0.051	5.167	2794	WS00818_L04
6	69.073	121	4	-0.222	0.038	4.309	2794	WS00818_L04
8	78.389	157	1	-0.175	0.046	4.124	2794	WS00818_L04

10	21.864	188	1	0.380	0.069	6.186	2794	WS00818_L04
11	45.989	216	1	-0.273	0.061	5.573	2794	WS00818_L04
14	46.637	252	4	0.323	0.052	5.224	2794	WS00818_L04
1	69.854	13	2	0.270	0.058	7.770	2795	WS00818_N02
1	71.592	14	2	0.272	0.059	7.948	2795	WS00818_N02
1	77.177	15	2	0.265	0.055	7.452	2795	WS00818_N02
1	82.301	16	4	-0.254	0.046	4.712	2795	WS00818_N02
1	141.021	25	2	0.175	0.036	4.911	2795	WS00818_N02
3	9.953	42	3	0.183	0.060	4.822	2795	WS00818_N02
3	116.958	63	4	-0.193	0.057	6.160	2795	WS00818_N02
7	73.169	142	2	-0.432	0.058	4.724	2795	WS00818_N02
9	49.013	173	2	0.237	0.059	8.205	2795	WS00818_N02
9	81.073	183	3	-0.260	0.065	3.925	2795	WS00818_N02
13	0.000	243	4	0.270	0.157	4.102	2795	WS00818_N02
14	0.000	246	2	-0.211	0.028	4.122	2795	WS00818_N02
1	62.128	11	1	-0.498	0.113	4.712	2796	WS00818_N18
1	95.572	17	2	-0.408	0.108	11.240	2796	WS00818_N18
2	93.504	32	4	-0.236	0.057	4.905	2796	WS00818_N18
6	54.066	119	4	-0.219	0.054	4.850	2796	WS00818_N18
6	69.073	121	4	0.225	0.054	4.848	2796	WS00818_N18
7	59.789	137	3	-0.227	0.054	5.254	2796	WS00818_N18
10	78.479	193	3	0.296	0.055	5.449	2796	WS00818_N18
12	6.242	234	4	-0.304	0.043	4.108	2796	WS00818_N18
14	44.898	251	3	0.274	0.067	5.983	2796	WS00818_N18
2	75.550	31	2	-0.370	0.075	7.961	2797	WS0106_G07
6	81.996	123	3	-0.255	0.089	3.905	2797	WS0106_G07
11	46.026	217	1	0.235	0.078	6.023	2797	WS0106_G07
6	69.073	121	3	0.254	0.045	4.546	2798	WS0106_G17
10	70.246	190	4	-0.304	0.048	3.872	2798	WS0106_G17
11	45.989	216	1	-0.221	0.066	4.505	2798	WS0106_G17
11	45.989	216	2	0.208	0.040	4.757	2798	WS0106_G17
1	43.578	8	3	0.179	0.042	3.912	2799	WS0106_I08
4	75.958	77	3	-0.224	0.092	4.659	2799	WS0106_I08
4	97.576	80	2	0.192	0.034	5.327	2799	WS0106_I08
5	84.347	105	2	-0.210	0.046	4.929	2799	WS0106_I08
6	25.914	114	1	-0.262	0.077	5.292	2799	WS0106_I08
6	87.891	124	2	0.287	0.075	9.015	2799	WS0106_I08
6	89.401	125	2	0.290	0.046	3.965	2799	WS0106_I08
7	17.610	131	3	0.267	0.111	5.639	2799	WS0106_I08
7	62.787	139	4	-0.261	0.064	7.864	2799	WS0106_I08
7	73.169	142	1	0.549	0.180	4.823	2799	WS0106_I08
10	80.088	194	2	0.200	0.042	5.709	2799	WS0106_I08
10	104.214	198	4	0.261	0.073	6.355	2799	WS0106_I08
11	42.600	213	3	-0.223	0.052	4.814	2799	WS0106_I08
11	48.979	218	4	0.297	0.049	4.767	2799	WS0106_I08
14	44.898	251	3	0.233	0.076	5.782	2799	WS0106_I08
1	22.419	4	3	0.349	0.128	6.280	2800	WS0106_I21
1	77.177	15	2	-0.227	0.045	4.969	2800	WS0106_I21
1	95.572	17	2	-0.244	0.036	3.939	2800	WS0106_I21
1	107.333	19	4	-0.254	0.067	4.656	2800	WS0106_I21
2	126.181	39	4	-0.421	0.074	4.202	2800	WS0106_I21
3	80.211	55	4	-0.234	0.053	5.015	2800	WS0106_I21
3	148.745	68	4	-0.271	0.084	7.804	2800	WS0106_I21
4	6.032	72	4	0.263	0.062	5.842	2800	WS0106_I21

9	0.000	166	1	0.257	0.055	4.347	2800	WS0106_I21
3	137.891	64	1	-0.281	0.063	4.606	2801	WS0106_K13
6	81.996	123	3	0.258	0.116	5.006	2801	WS0106_K13
6	106.702	128	3	-0.361	0.090	7.266	2801	WS0106_K13
7	15.555	130	3	-0.209	0.059	4.836	2801	WS0106_K13
8	39.090	152	4	-0.339	0.074	7.699	2801	WS0106_K13
9	28.608	170	2	-0.294	0.048	5.798	2801	WS0106_K13
9	28.608	170	3	0.360	0.107	9.579	2801	WS0106_K13
10	9.580	187	2	-0.224	0.065	4.820	2801	WS0106_K13
14	46.637	252	3	0.248	0.063	5.433	2801	WS0106_K13
2	75.550	31	1	-0.228	0.070	5.388	2802	WS0106_L14
2	102.814	36	1	-0.318	0.062	5.758	2802	WS0106_L14
3	83.563	56	3	0.238	0.041	4.191	2802	WS0106_L14
4	82.462	78	2	-0.275	0.072	8.171	2802	WS0106_L14
4	97.576	80	2	0.179	0.032	4.088	2802	WS0106_L14
7	17.610	131	3	0.372	0.103	6.331	2802	WS0106_L14
7	62.787	139	4	-0.333	0.099	9.090	2802	WS0106_L14
7	73.169	142	1	0.526	0.120	4.449	2802	WS0106_L14
7	75.883	144	4	-0.338	0.065	5.479	2802	WS0106_L14
9	61.113	178	1	0.382	0.108	4.062	2802	WS0106_L14
9	69.046	181	2	-0.370	0.122	7.290	2802	WS0106_L14
11	17.088	203	1	-0.231	0.063	4.843	2802	WS0106_L14
12	14.406	230	3	-0.235	0.043	4.324	2802	WS0106_L14
12	6.242	234	2	0.244	0.043	5.032	2802	WS0106_L14
14	22.402	248	3	0.211	0.045	4.512	2802	WS0106_L14
1	22.419	4	3	0.381	0.137	6.937	2803	WS0106_N15
1	43.578	8	3	0.260	0.052	4.089	2803	WS0106_N15
1	50.955	10	1	0.235	0.071	5.698	2803	WS0106_N15
1	69.854	13	2	-0.256	0.064	6.838	2803	WS0106_N15
1	71.592	14	2	-0.243	0.058	6.173	2803	WS0106_N15
1	77.177	15	2	-0.265	0.069	7.366	2803	WS0106_N15
1	95.572	17	2	-0.344	0.077	8.341	2803	WS0106_N15
5	16.219	93	2	0.271	0.065	4.939	2803	WS0106_N15
6	81.996	123	3	-0.308	0.091	4.626	2803	WS0106_N15
6	87.891	124	2	-0.218	0.040	4.296	2803	WS0106_N15
6	89.401	125	2	-0.209	0.048	5.177	2803	WS0106_N15
6	106.702	128	2	0.407	0.088	4.188	2803	WS0106_N15
8	50.001	154	1	-0.221	0.037	3.907	2803	WS0106_N15
12	28.493	228	2	0.248	0.068	4.068	2803	WS0106_N15
14	0.000	246	2	0.286	0.078	8.450	2803	WS0106_N15
14	44.898	251	3	0.233	0.051	4.988	2803	WS0106_N15
14	46.637	252	3	0.270	0.046	4.488	2803	WS0106_N15
2	63.261	30	3	-0.225	0.038	4.040	2804	WS0106_O01
2	127.147	40	3	-0.221	0.038	4.058	2804	WS0106_O01
3	104.681	60	2	-0.249	0.049	5.353	2804	WS0106_O01
3	114.747	62	2	0.275	0.034	3.948	2804	WS0106_O01
7	15.555	130	3	-0.246	0.066	7.283	2804	WS0106_O01
7	75.883	144	2	0.457	0.126	5.509	2804	WS0106_O01
8	11.974	150	1	0.186	0.049	3.883	2804	WS0106_O01
11	64.014	222	4	0.291	0.069	5.918	2804	WS0106_O01
12	6.242	234	2	0.162	0.038	4.179	2804	WS0106_O01
3	9.953	42	3	0.188	0.038	4.021	2805	WS00824_D13
4	55.925	76	1	0.183	0.033	3.986	2805	WS00824_D13
6	25.914	114	1	-0.227	0.044	4.152	2805	WS00824_D13

6	49.100	118	2	-0.300	0.108	5.105	2805	WS00824_D13
6	81.996	123	3	0.223	0.077	4.479	2805	WS00824_D13
7	59.789	137	3	0.272	0.056	6.009	2805	WS00824_D13
7	67.154	140	4	-0.279	0.127	4.523	2805	WS00824_D13
7	70.278	141	3	-0.298	0.071	7.560	2805	WS00824_D13
7	73.169	142	1	0.509	0.118	4.191	2805	WS00824_D13
7	75.883	144	2	0.667	0.208	11.582	2805	WS00824_D13
7	75.883	144	4	-0.383	0.093	7.651	2805	WS00824_D13
9	28.608	170	3	0.317	0.060	5.888	2805	WS00824_D13
10	80.088	194	1	-0.233	0.049	4.590	2805	WS00824_D13
1	131.686	23	1	-0.473	0.069	4.142	2806	WS00824_E01
2	120.617	38	3	0.193	0.051	4.117	2806	WS00824_E01
2	127.147	40	3	-0.151	0.048	3.859	2806	WS00824_E01
6	56.188	120	2	-0.263	0.049	4.210	2806	WS00824_E01
6	106.702	128	2	0.542	0.169	7.200	2806	WS00824_E01
7	75.883	144	2	0.423	0.091	4.827	2806	WS00824_E01
7	75.883	144	4	-0.399	0.067	7.479	2806	WS00824_E01
10	80.088	194	1	-0.254	0.043	4.368	2806	WS00824_E01
11	45.989	216	1	-0.243	0.044	4.464	2806	WS00824_E01
4	55.925	76	1	0.230	0.047	3.923	2807	WS00824_F18
4	173.929	88	2	-0.214	0.040	4.392	2807	WS00824_F18
6	87.891	124	2	0.203	0.046	5.065	2807	WS00824_F18
6	89.401	125	2	0.261	0.049	5.401	2807	WS00824_F18
6	106.702	128	2	-0.526	0.112	6.997	2807	WS00824_F18
9	17.951	169	2	0.248	0.037	4.471	2807	WS00824_F18
9	58.045	176	1	0.325	0.082	3.845	2807	WS00824_F18
9	69.046	181	1	-0.232	0.086	3.948	2807	WS00824_F18
11	9.237	202	4	0.239	0.055	4.495	2807	WS00824_F18
12	38.543	232	4	-0.304	0.123	3.936	2807	WS00824_F18
13	1.815	244	2	-0.262	0.059	6.428	2807	WS00824_F18
1	2.788	2	4	-0.193	0.059	4.924	2808	WS00824_G02
1	43.578	8	2	0.250	0.039	4.409	2808	WS00824_G02
7	105.477	147	2	-0.215	0.077	4.008	2808	WS00824_G02
9	49.013	173	1	0.272	0.053	4.386	2808	WS00824_G02
11	46.026	217	1	-0.163	0.056	4.355	2808	WS00824_G02
11	56.921	219	1	-0.361	0.194	4.779	2808	WS00824_G02
2	63.261	30	3	-0.228	0.053	5.120	2809	WS00824_I09
3	15.147	44	2	-0.227	0.080	4.047	2809	WS00824_I09
3	20.512	46	2	-0.209	0.041	4.102	2809	WS00824_I09
5	16.219	93	2	0.264	0.052	5.266	2809	WS00824_I09
12	14.406	230	3	-0.334	0.104	10.111	2809	WS00824_I09
1	2.788	2	4	-0.305	0.046	5.109	2810	WS00824_I18
1	82.301	16	4	0.316	0.042	4.863	2810	WS00824_I18
3	141.443	65	4	0.268	0.060	4.074	2810	WS00824_I18
7	73.169	142	1	0.647	0.126	7.845	2810	WS00824_I18
12	14.406	230	4	0.320	0.045	5.282	2810	WS00824_I18
1	82.301	16	3	-0.276	0.046	4.836	2811	WS00824_K16
1	95.572	17	3	0.391	0.118	12.210	2811	WS00824_K16
4	55.925	76	1	0.207	0.043	5.337	2811	WS00824_K16
4	82.462	78	2	-0.198	0.064	5.676	2811	WS00824_K16
4	92.039	79	2	-0.223	0.046	4.079	2811	WS00824_K16
6	25.914	114	1	-0.311	0.077	7.588	2811	WS00824_K16
8	95.937	159	3	0.192	0.042	4.329	2811	WS00824_K16
10	9.580	187	3	-0.294	0.076	4.109	2811	WS00824_K16

11	46.026	217	1	-0.232	0.050	4.927	2811	WS00824_K16
12	0.000	233	1	-0.305	0.087	8.472	2811	WS00824_K16
4	103.719	81	4	-0.228	0.032	4.338	2812	WS00824_L02
5	7.672	92	4	-0.314	0.093	10.148	2812	WS00824_L02
5	86.669	106	4	-0.386	0.222	7.013	2812	WS00824_L02
6	45.897	117	1	0.251	0.078	4.441	2812	WS00824_L02
7	62.787	139	2	0.278	0.046	5.645	2812	WS00824_L02
8	50.001	154	2	-0.182	0.030	3.978	2812	WS00824_L02
9	32.356	171	2	-0.204	0.037	4.913	2812	WS00824_L02
10	93.347	196	2	0.475	0.102	4.867	2812	WS00824_L02
11	17.088	203	2	0.284	0.064	7.347	2812	WS00824_L02
12	22.441	227	1	0.275	0.087	4.884	2812	WS00824_L02
1	2.788	2	3	-0.194	0.050	4.343	2813	WS00731_D22
3	137.891	64	3	0.327	0.086	5.531	2813	WS00731_D22
4	97.576	80	2	-0.190	0.042	5.606	2813	WS00731_D22
4	116.928	82	2	-0.275	0.046	5.808	2813	WS00731_D22
7	73.169	142	2	-0.517	0.086	5.890	2813	WS00731_D22
9	59.963	177	4	-0.323	0.060	5.688	2813	WS00731_D22
10	95.795	197	4	0.186	0.042	4.164	2813	WS00731_D22
2	93.504	32	4	-0.331	0.074	8.718	2814	WS00112_H01
2	120.617	38	4	-0.249	0.047	5.605	2814	WS00112_H01
2	126.181	39	4	0.381	0.065	3.887	2814	WS00112_H01
3	116.958	63	4	-0.262	0.048	5.644	2814	WS00112_H01
3	137.891	64	1	0.244	0.048	3.947	2814	WS00112_H01
6	87.891	124	2	0.206	0.041	4.236	2814	WS00112_H01
7	15.555	130	3	-0.225	0.063	4.026	2814	WS00112_H01
12	38.543	232	4	0.365	0.130	6.393	2814	WS00112_H01
4	43.146	74	3	0.291	0.063	7.306	2815	WS00731_O12
4	103.719	81	4	0.198	0.080	5.980	2815	WS00731_O12
5	42.979	97	1	0.274	0.078	5.447	2815	WS00731_O12
5	42.979	97	2	0.207	0.050	5.876	2815	WS00731_O12
6	49.100	118	2	0.278	0.109	6.916	2815	WS00731_O12
6	106.702	128	2	0.461	0.093	6.035	2815	WS00731_O12
7	70.278	141	3	0.246	0.047	5.402	2815	WS00731_O12
11	40.960	210	2	0.433	0.094	5.280	2815	WS00731_O12
12	28.493	228	2	0.252	0.087	4.633	2815	WS00731_O12
12	6.242	234	4	0.222	0.035	4.612	2815	WS00731_O12
1	15.589	3	2	0.195	0.033	4.128	2816	WS00113_A21
1	45.794	9	3	0.222	0.064	5.864	2816	WS00113_A21
3	116.958	63	4	-0.158	0.053	5.282	2816	WS00113_A21
5	0.000	89	1	-0.294	0.073	5.043	2816	WS00113_A21
5	0.000	89	3	0.282	0.083	7.309	2816	WS00113_A21
5	42.979	97	1	0.232	0.069	4.489	2816	WS00113_A21
6	69.073	121	4	-0.236	0.064	5.437	2816	WS00113_A21
6	81.996	123	3	0.284	0.082	3.962	2816	WS00113_A21
9	0.000	166	2	0.252	0.036	4.659	2816	WS00113_A21
14	22.402	248	3	0.331	0.083	7.301	2816	WS00113_A21
1	40.464	7	4	-0.302	0.068	6.486	2817	WS00941_M08
1	95.572	17	2	-0.291	0.041	4.415	2817	WS00941_M08
4	0.000	71	4	0.257	0.086	8.156	2817	WS00941_M08
5	0.000	89	3	-0.192	0.046	4.495	2817	WS00941_M08
6	69.073	121	4	0.326	0.103	9.516	2817	WS00941_M08
6	81.996	123	3	-0.286	0.080	4.233	2817	WS00941_M08
7	70.278	141	3	0.239	0.067	6.193	2817	WS00941_M08

7	91.487	146	3	0.213	0.061	5.688	2817	WS00941_M08
10	35.204	189	1	0.245	0.051	4.176	2817	WS00941_M08
11	29.069	205	2	-0.272	0.059	5.177	2817	WS00941_M08
11	41.984	212	2	-0.190	0.041	4.406	2817	WS00941_M08
11	43.491	214	4	0.207	0.045	3.994	2817	WS00941_M08
11	44.884	215	4	-0.302	0.054	5.252	2817	WS00941_M08
12	38.543	232	4	-0.412	0.193	5.460	2817	WS00941_M08
12	6.242	234	2	0.226	0.043	4.874	2817	WS00941_M08
3	9.953	42	3	0.236	0.064	6.880	2818	WS01040_E23
3	83.563	56	3	-0.190	0.038	4.100	2818	WS01040_E23
5	56.008	101	2	0.208	0.033	4.089	2818	WS01040_E23
5	57.017	102	2	-0.222	0.035	4.370	2818	WS01040_E23
6	81.996	123	3	0.288	0.158	5.654	2818	WS01040_E23
9	69.046	181	2	0.326	0.095	6.818	2818	WS01040_E23
1	77.177	15	2	0.176	0.031	4.253	2819	WS0014_I16
2	48.839	29	4	0.300	0.050	6.596	2819	WS0014_I16
5	54.072	100	4	0.273	0.060	7.767	2819	WS0014_I16
6	81.996	123	3	0.268	0.117	4.196	2819	WS0014_I16
10	9.580	187	2	-0.226	0.045	5.197	2819	WS0014_I16
11	0.000	199	2	-0.266	0.060	8.319	2819	WS0014_I16
14	0.000	246	2	-0.236	0.051	6.913	2819	WS0014_I16
3	116.958	63	4	-0.363	0.133	13.139	2820	WS0109_O14
3	144.593	66	4	0.339	0.127	13.470	2820	WS0109_O14
3	148.745	68	4	-0.364	0.097	9.753	2820	WS0109_O14
3	154.505	69	4	0.334	0.089	8.560	2820	WS0109_O14
4	173.929	88	2	-0.217	0.042	5.049	2820	WS0109_O14
7	74.560	143	2	-0.223	0.033	4.148	2820	WS0109_O14
8	50.001	154	1	-0.208	0.048	3.892	2820	WS0109_O14
9	0.000	166	2	0.200	0.036	4.334	2820	WS0109_O14
9	17.951	169	2	-0.231	0.037	3.952	2820	WS0109_O14
9	49.013	173	2	0.246	0.045	4.432	2820	WS0109_O14
9	69.046	181	2	0.361	0.158	6.484	2820	WS0109_O14
10	93.347	196	2	-0.469	0.106	4.969	2820	WS0109_O14
14	46.637	252	4	-0.293	0.058	5.883	2820	WS0109_O14
1	62.128	11	1	-0.486	0.125	4.476	2821	WS0015_H15
3	137.891	64	3	0.258	0.057	4.759	2821	WS0015_H15
7	70.278	141	3	-0.251	0.083	7.437	2821	WS0015_H15
7	91.487	146	3	-0.179	0.048	4.318	2821	WS0015_H15
4	0.000	71	4	0.216	0.051	5.126	2822	WS0081_G16
6	81.996	123	3	0.326	0.126	5.978	2822	WS0081_G16
8	87.463	158	2	-0.196	0.048	4.430	2822	WS0081_G16
3	9.953	42	3	0.210	0.050	5.830	2823	WS00929_E15
6	81.996	123	3	0.294	0.100	4.237	2823	WS00929_E15
6	106.702	128	2	0.393	0.058	4.144	2823	WS00929_E15
11	0.000	199	2	0.140	0.036	3.959	2823	WS00929_E15
12	22.441	227	1	0.276	0.054	4.388	2823	WS00929_E15
12	22.441	227	3	-0.251	0.036	4.321	2823	WS00929_E15
4	124.132	83	2	-0.284	0.119	3.914	2824	WS00929_H21
5	54.072	100	4	0.238	0.038	4.695	2824	WS00929_H21
5	57.017	102	4	-0.184	0.034	3.943	2824	WS00929_H21
10	9.580	187	2	-0.292	0.080	7.165	2824	WS00929_H21
11	64.014	222	4	-0.243	0.031	3.963	2824	WS00929_H21
12	28.493	228	2	-0.257	0.068	6.105	2824	WS00929_H21
13	3.386	245	1	-0.215	0.072	4.757	2824	WS00929_H21

1	82.301	16	4	0.284	0.045	3.924	2825	WS00819_G21
5	52.820	99	3	-0.237	0.040	4.047	2825	WS00819_G21
10	70.246	190	2	-0.225	0.048	4.623	2825	WS00819_G21
4	92.039	79	4	-0.263	0.043	4.296	2826	WS00819_H15
7	105.477	147	2	-0.272	0.088	4.128	2826	WS00819_H15
9	49.013	173	1	0.239	0.044	4.514	2826	WS00819_H15
10	93.347	196	1	0.450	0.095	4.854	2826	WS00819_H15
11	57.661	220	4	0.255	0.055	5.491	2826	WS00819_H15
11	64.014	222	4	-0.336	0.065	6.475	2826	WS00819_H15
1	62.128	11	1	-0.735	0.289	10.667	2827	WS00819_J02
1	127.805	21	4	0.213	0.054	4.044	2827	WS00819_J02
4	6.032	72	4	0.141	0.046	4.718	2827	WS00819_J02
5	7.672	92	1	-0.271	0.056	3.930	2827	WS00819_J02
6	87.891	124	2	0.309	0.090	9.829	2827	WS00819_J02
6	89.401	125	2	0.385	0.095	10.602	2827	WS00819_J02
6	106.702	128	2	-0.423	0.109	4.909	2827	WS00819_J02
9	28.608	170	3	-0.396	0.126	10.555	2827	WS00819_J02
9	55.358	175	1	-0.305	0.060	4.537	2827	WS00819_J02
11	17.088	203	1	0.265	0.074	5.775	2827	WS00819_J02
11	27.405	204	1	-0.307	0.069	5.787	2827	WS00819_J02
11	27.405	204	4	0.335	0.049	5.311	2827	WS00819_J02
11	29.069	205	1	-0.257	0.050	4.327	2827	WS00819_J02
11	45.989	216	1	0.227	0.062	4.841	2827	WS00819_J02
13	1.815	244	4	0.280	0.078	8.695	2827	WS00819_J02
1	40.464	7	4	0.337	0.049	4.146	2828	WS00819_J15
2	75.550	31	2	-0.212	0.058	4.038	2828	WS00819_J15
3	141.443	65	4	0.332	0.081	6.178	2828	WS00819_J15
4	142.917	85	1	-0.229	0.037	4.013	2828	WS00819_J15
6	25.914	114	1	-0.335	0.075	8.201	2828	WS00819_J15
7	67.154	140	4	-0.315	0.087	6.097	2828	WS00819_J15
7	74.560	143	1	-0.177	0.040	4.349	2828	WS00819_J15
7	75.883	144	2	0.455	0.096	6.097	2828	WS00819_J15
7	75.883	144	4	-0.373	0.075	9.771	2828	WS00819_J15
9	36.227	165	4	0.251	0.026	4.149	2828	WS00819_J15
10	9.580	187	3	-0.434	0.171	4.450	2828	WS00819_J15
10	93.347	196	1	0.416	0.113	4.151	2828	WS00819_J15
11	45.989	216	1	-0.200	0.047	5.010	2828	WS00819_J15
11	46.026	217	1	-0.282	0.076	8.234	2828	WS00819_J15
12	22.441	227	1	0.366	0.053	5.803	2828	WS00819_J15
12	28.493	228	2	-0.265	0.088	5.192	2828	WS00819_J15
1	2.788	2	3	-0.184	0.030	3.988	2829	WS00819_L02
1	45.794	9	3	-0.314	0.061	6.013	2829	WS00819_L02
1	127.805	21	3	-0.259	0.055	7.283	2829	WS00819_L02
2	93.504	32	2	-0.246	0.039	5.435	2829	WS00819_L02
4	124.132	83	2	-0.253	0.042	4.844	2829	WS00819_L02
6	26.350	115	2	-0.258	0.045	6.346	2829	WS00819_L02
9	0.000	163	2	0.433	0.039	4.121	2829	WS00819_L02
9	36.227	165	3	-0.226	0.038	5.055	2829	WS00819_L02
2	126.181	39	4	-0.375	0.056	4.105	2830	WS00819_M01
3	15.147	44	2	-0.245	0.067	5.038	2830	WS00819_M01
3	95.157	59	2	0.189	0.036	4.001	2830	WS00819_M01
5	7.672	92	4	-0.239	0.046	5.399	2830	WS00819_M01
5	84.347	105	1	-0.226	0.049	3.990	2830	WS00819_M01
6	81.996	123	4	0.518	0.182	8.796	2830	WS00819_M01

9	17.951	169	2	0.252	0.050	3.991	2830	WS00819_M01
9	55.358	175	1	0.266	0.053	4.385	2830	WS00819_M01
10	70.246	190	4	0.341	0.066	7.196	2830	WS00819_M01
12	14.406	230	3	-0.264	0.066	4.444	2830	WS00819_M01
14	9.923	247	1	0.249	0.058	4.715	2830	WS00819_M01
1	95.572	17	2	0.279	0.044	4.968	2831	WS00819_O15
3	9.953	42	3	0.272	0.054	4.673	2831	WS00819_O15
4	82.462	78	1	-0.203	0.050	3.933	2831	WS00819_O15
4	124.132	83	1	-0.458	0.098	4.391	2831	WS00819_O15
4	142.917	85	1	0.304	0.056	4.445	2831	WS00819_O15
6	56.188	120	2	-0.315	0.057	5.268	2831	WS00819_O15
7	105.477	147	2	-0.233	0.080	4.856	2831	WS00819_O15
8	87.463	158	2	-0.257	0.064	6.977	2831	WS00819_O15
9	59.963	177	4	0.277	0.075	7.849	2831	WS00819_O15
9	81.073	183	1	0.287	0.065	5.320	2831	WS00819_O15
10	70.246	190	2	-0.238	0.065	7.176	2831	WS00819_O15
11	43.491	214	4	-0.233	0.065	4.786	2831	WS00819_O15
11	57.661	220	4	0.210	0.053	5.562	2831	WS00819_O15
11	64.014	222	4	-0.356	0.072	6.573	2831	WS00819_O15
12	6.242	234	2	-0.160	0.033	4.378	2831	WS00819_O15
14	0.000	246	2	-0.240	0.069	7.178	2831	WS00819_O15
1	141.021	25	2	0.241	0.061	6.788	2832	WS00819_P16
2	75.550	31	1	0.157	0.051	3.892	2832	WS00819_P16
3	80.211	55	4	0.295	0.047	4.986	2832	WS00819_P16
3	148.745	68	4	0.268	0.051	4.665	2832	WS00819_P16
9	36.227	165	4	0.223	0.070	7.842	2832	WS00819_P16
10	78.479	193	4	-0.175	0.043	4.847	2832	WS00819_P16
1	2.788	2	3	-0.237	0.099	7.770	2833	WS01040_L12
1	15.589	3	2	0.277	0.060	6.947	2833	WS01040_L12
4	55.925	76	1	0.160	0.058	4.198	2833	WS01040_L12
7	73.169	142	2	-0.426	0.063	3.958	2833	WS01040_L12
8	0.000	148	4	-0.321	0.084	9.256	2833	WS01040_L12
9	0.000	166	2	0.257	0.055	6.984	2833	WS01040_L12
13	0.000	243	4	0.239	0.136	5.161	2833	WS01040_L12
1	69.854	13	2	0.265	0.067	7.228	2834	WS01040_M07
1	71.592	14	2	0.260	0.065	7.008	2834	WS01040_M07
1	77.177	15	2	0.247	0.058	6.192	2834	WS01040_M07
6	81.996	123	3	0.328	0.117	5.023	2834	WS01040_M07
9	49.013	173	2	0.194	0.043	4.451	2834	WS01040_M07
11	43.491	214	4	-0.277	0.052	4.279	2834	WS01040_M07
11	64.014	222	4	-0.210	0.048	4.472	2834	WS01040_M07
1	82.301	16	4	-0.339	0.071	5.946	2835	WS01040_O16
1	127.805	21	3	-0.193	0.048	4.538	2835	WS01040_O16
3	137.891	64	1	-0.286	0.058	4.663	2835	WS01040_O16
4	97.576	80	2	0.200	0.031	3.858	2835	WS01040_O16
6	106.702	128	2	-0.444	0.099	4.858	2835	WS01040_O16
8	39.090	152	4	0.194	0.043	4.010	2835	WS01040_O16
11	40.960	210	2	0.396	0.072	4.367	2835	WS01040_O16
1	43.578	8	2	0.221	0.048	4.072	2836	WS01040_P20
2	48.839	29	1	0.252	0.070	7.345	2836	WS01040_P20
3	69.071	52	1	0.397	0.097	7.196	2836	WS01040_P20
4	103.719	81	4	0.254	0.072	5.627	2836	WS01040_P20
6	45.897	117	1	-0.327	0.061	6.311	2836	WS01040_P20
6	69.073	121	3	-0.219	0.052	5.741	2836	WS01040_P20

6	81.996	123	3	-0.320	0.056	4.173	2836	WS01040_P20
6	106.702	128	2	0.391	0.106	3.848	2836	WS01040_P20
8	50.001	154	2	0.174	0.050	3.893	2836	WS01040_P20
11	0.000	199	2	0.205	0.055	4.922	2836	WS01040_P20
11	17.088	203	1	-0.233	0.040	4.158	2836	WS01040_P20
4	124.132	83	1	-0.477	0.110	4.576	2837	WS01041_C21
5	57.017	102	2	-0.290	0.065	6.475	2837	WS01041_C21
6	81.996	123	3	0.301	0.095	4.562	2837	WS01041_C21
7	17.610	131	3	-0.314	0.079	3.979	2837	WS01041_C21
9	59.963	177	4	0.216	0.044	4.077	2837	WS01041_C21
11	17.088	203	1	0.272	0.084	7.480	2837	WS01041_C21
11	29.069	205	1	-0.268	0.059	5.238	2837	WS01041_C21
13	1.815	244	4	0.217	0.053	4.597	2837	WS01041_C21
4	142.917	85	1	0.298	0.096	7.602	2838	WS01041_D19
4	142.917	85	3	-0.219	0.059	4.847	2838	WS01041_D19
6	45.897	117	4	-0.229	0.035	4.530	2838	WS01041_D19
8	62.580	155	1	-0.337	0.189	7.944	2838	WS01041_D19
8	78.389	157	1	-0.260	0.082	6.512	2838	WS01041_D19
8	87.463	158	1	0.269	0.089	7.016	2838	WS01041_D19
9	36.227	165	3	0.256	0.048	3.980	2838	WS01041_D19
11	27.405	204	4	-0.247	0.058	6.679	2838	WS01041_D19
1	77.177	15	2	0.169	0.034	4.313	2839	WS01041_H06
1	95.572	17	2	0.252	0.034	4.392	2839	WS01041_H06
3	15.147	44	2	0.450	0.171	13.379	2839	WS01041_H06
3	20.512	46	2	0.254	0.036	4.718	2839	WS01041_H06
3	144.593	66	4	0.216	0.044	5.535	2839	WS01041_H06
4	173.929	88	2	-0.231	0.043	5.553	2839	WS01041_H06
5	54.072	100	4	0.201	0.039	4.285	2839	WS01041_H06
6	56.188	120	2	-0.251	0.030	4.033	2839	WS01041_H06
11	27.405	204	4	-0.301	0.041	4.920	2839	WS01041_H06
12	6.242	234	2	-0.173	0.034	4.787	2839	WS01041_H06
1	95.572	17	3	0.271	0.046	4.242	2840	WS01041_H24
9	55.358	175	3	0.424	0.088	4.091	2840	WS01041_H24
11	27.405	204	4	-0.303	0.066	6.353	2840	WS01041_H24
11	41.984	212	2	0.220	0.050	5.887	2840	WS01041_H24
11	42.600	213	3	-0.211	0.047	4.230	2840	WS01041_H24
11	45.989	216	2	0.311	0.089	10.505	2840	WS01041_H24
11	60.464	221	2	0.365	0.085	10.039	2840	WS01041_H24
1	2.788	2	3	0.157	0.042	4.181	2841	WS00728_N02
2	93.504	32	4	0.244	0.050	4.471	2841	WS00728_N02
4	55.925	76	1	0.259	0.058	6.634	2841	WS00728_N02
9	55.358	175	3	0.672	0.148	10.529	2841	WS00728_N02
9	58.045	176	1	-0.283	0.066	5.348	2841	WS00728_N02
9	69.046	181	1	0.285	0.059	4.802	2841	WS00728_N02
12	22.441	227	1	0.293	0.050	5.626	2841	WS00728_N02
12	14.406	230	3	-0.283	0.066	6.599	2841	WS00728_N02
4	75.958	77	3	-0.236	0.091	4.853	2842	WS01041_O15
7	91.487	146	3	-0.300	0.062	6.401	2842	WS01041_O15
11	44.884	215	4	0.258	0.049	4.217	2842	WS01041_O15
6	81.996	123	3	0.447	0.169	7.553	2843	WS00729_M04
9	59.963	177	4	-0.234	0.040	4.947	2843	WS00729_M04
11	60.464	221	2	0.304	0.061	6.179	2843	WS00729_M04
4	0.000	71	4	0.233	0.057	5.327	2844	WS00834_J06
6	81.996	123	3	0.393	0.116	5.993	2844	WS00834_J06

8	87.463	158	2	-0.193	0.046	4.602	2844	WS00834_J06
10	78.479	193	3	0.319	0.059	5.545	2844	WS00834_J06
10	86.151	195	3	-0.172	0.048	4.479	2844	WS00834_J06
12	6.242	234	2	-0.211	0.035	4.079	2844	WS00834_J06
1	2.788	2	3	-0.270	0.092	5.927	2845	WS0094_E15
1	2.788	2	4	-0.310	0.054	6.386	2845	WS0094_E15
4	82.462	78	1	-0.210	0.045	4.457	2845	WS0094_E15
7	73.169	142	1	0.565	0.084	5.668	2845	WS0094_E15
9	59.963	177	4	0.245	0.029	3.851	2845	WS0094_E15
10	78.479	193	3	0.306	0.078	5.022	2845	WS0094_E15
6	81.996	123	3	0.269	0.072	4.038	2846	WS0094_J07
10	9.580	187	3	-0.308	0.088	4.721	2846	WS0094_J07
10	35.204	189	2	0.251	0.061	4.542	2846	WS0094_J07
10	93.347	196	1	0.508	0.110	6.201	2846	WS0094_J07
14	44.898	251	1	-0.347	0.068	6.942	2846	WS0094_J07
1	2.788	2	4	-0.188	0.049	6.074	2847	WS0017_P04
4	92.039	79	2	-0.122	0.033	4.111	2847	WS0017_P04
4	116.928	82	2	0.388	0.059	7.796	2847	WS0017_P04
4	124.132	83	2	0.491	0.124	7.142	2847	WS0017_P04
4	173.929	88	2	-0.155	0.040	5.022	2847	WS0017_P04
6	56.188	120	2	0.338	0.034	3.901	2847	WS0017_P04
6	69.073	121	4	0.193	0.038	4.322	2847	WS0017_P04
6	87.891	124	2	0.511	0.182	22.880	2847	WS0017_P04
6	89.401	125	2	0.395	0.082	9.183	2847	WS0017_P04
7	15.555	130	3	-0.173	0.036	3.867	2847	WS0017_P04
7	70.278	141	3	0.267	0.053	5.465	2847	WS0017_P04
8	50.001	154	2	-0.220	0.031	4.137	2847	WS0017_P04
11	0.000	199	2	0.193	0.035	4.357	2847	WS0017_P04
12	22.441	227	1	0.250	0.100	6.479	2847	WS0017_P04
13	0.000	243	4	0.255	0.155	4.661	2847	WS0017_P04
6	56.188	120	1	0.338	0.069	6.287	2848	WS00940_J10
11	0.000	199	3	0.353	0.055	6.288	2848	WS00940_J10
11	27.405	204	1	-0.403	0.103	9.817	2848	WS00940_J10
1	45.794	9	1	0.337	0.046	4.386	2849	WS0018_N18
1	69.854	13	2	-0.316	0.110	10.420	2849	WS0018_N18
1	71.592	14	2	-0.265	0.078	7.380	2849	WS0018_N18
1	77.177	15	2	-0.279	0.086	8.104	2849	WS0018_N18
4	55.925	76	1	0.245	0.045	5.062	2849	WS0018_N18
5	86.669	106	4	-0.378	0.076	4.295	2849	WS0018_N18
6	87.891	124	2	0.188	0.047	4.411	2849	WS0018_N18
6	89.401	125	2	0.268	0.051	4.220	2849	WS0018_N18
6	106.702	128	2	-0.519	0.127	6.866	2849	WS0018_N18
7	62.787	139	4	-0.181	0.036	3.928	2849	WS0018_N18
7	74.560	143	1	-0.291	0.085	7.921	2849	WS0018_N18
7	75.883	144	4	-0.400	0.092	7.440	2849	WS0018_N18
9	0.000	163	1	-0.446	0.111	4.372	2849	WS0018_N18
11	27.405	204	1	-0.233	0.052	4.885	2849	WS0018_N18
11	60.464	221	1	-0.316	0.051	4.805	2849	WS0018_N18
1	2.788	2	3	-0.172	0.045	3.842	2850	WS00941_D01
1	38.326	6	4	-0.399	0.069	7.020	2850	WS00941_D01
1	40.464	7	4	0.418	0.073	6.038	2850	WS00941_D01
1	45.794	9	3	-0.294	0.064	5.712	2850	WS00941_D01
3	15.147	44	2	-0.328	0.095	5.326	2850	WS00941_D01
4	55.925	76	1	0.216	0.037	4.084	2850	WS00941_D01

5	86.669	106	4	-0.496	0.138	5.834	2850	WS00941_D01
7	62.787	139	4	-0.212	0.043	4.442	2850	WS00941_D01
7	67.154	140	4	-0.213	0.094	3.987	2850	WS00941_D01
7	75.883	144	2	0.380	0.071	4.649	2850	WS00941_D01
7	75.883	144	4	-0.438	0.082	8.767	2850	WS00941_D01
10	35.204	189	1	-0.312	0.067	5.580	2850	WS00941_D01
10	77.676	192	1	-0.137	0.049	4.024	2850	WS00941_D01
10	80.088	194	1	-0.250	0.066	5.487	2850	WS00941_D01
1	69.854	13	2	0.241	0.054	6.202	2851	WS00923_D10
1	71.592	14	2	0.244	0.056	6.429	2851	WS00923_D10
1	77.177	15	2	0.252	0.059	6.794	2851	WS00923_D10
3	9.953	42	3	0.210	0.051	4.018	2851	WS00923_D10
4	6.032	72	4	-0.233	0.037	4.104	2851	WS00923_D10
11	64.014	222	4	-0.196	0.043	4.571	2851	WS00923_D10
12	6.242	234	2	-0.203	0.030	5.377	2851	WS00923_D10
14	0.000	246	2	-0.223	0.044	5.072	2851	WS00923_D10
1	43.578	8	2	0.209	0.044	4.258	2852	WS00923_G15
1	107.333	19	4	-0.286	0.083	9.401	2852	WS00923_G15
1	123.316	20	4	-0.455	0.189	10.279	2852	WS00923_G15
1	127.805	21	4	0.440	0.168	18.891	2852	WS00923_G15
1	131.686	23	1	0.649	0.116	7.211	2852	WS00923_G15
2	48.839	29	4	-0.290	0.055	6.476	2852	WS00923_G15
3	144.647	67	1	0.291	0.078	4.763	2852	WS00923_G15
4	92.039	79	4	0.350	0.053	6.369	2852	WS00923_G15
4	103.719	81	4	0.221	0.056	6.389	2852	WS00923_G15
4	124.132	83	1	-0.478	0.076	4.728	2852	WS00923_G15
5	0.000	89	1	-0.332	0.072	6.715	2852	WS00923_G15
5	16.219	93	1	-0.335	0.087	8.074	2852	WS00923_G15
6	25.914	114	1	-0.225	0.046	4.281	2852	WS00923_G15
7	15.555	130	2	0.196	0.040	3.911	2852	WS00923_G15
9	3.127	168	1	0.426	0.127	7.473	2852	WS00923_G15
9	55.358	175	1	-0.380	0.111	10.288	2852	WS00923_G15
9	55.358	175	3	0.659	0.277	10.779	2852	WS00923_G15
9	61.113	178	1	0.440	0.150	9.241	2852	WS00923_G15
12	38.543	232	4	0.323	0.063	3.916	2852	WS00923_G15
1	45.794	9	1	-0.306	0.072	6.804	2853	WS0108_F15
1	50.955	10	1	0.332	0.080	7.843	2853	WS0108_F15
1	71.592	14	2	-0.154	0.043	3.878	2853	WS0108_F15
1	82.301	16	4	-0.305	0.068	6.205	2853	WS0108_F15
1	95.572	17	2	-0.205	0.049	4.466	2853	WS0108_F15
3	9.953	42	4	-0.207	0.043	3.889	2853	WS0108_F15
4	142.917	85	1	-0.329	0.075	7.389	2853	WS0108_F15
6	69.073	121	3	-0.262	0.057	5.446	2853	WS0108_F15
6	89.401	125	2	-0.256	0.068	4.991	2853	WS0108_F15
9	17.951	169	1	-0.360	0.065	6.340	2853	WS0108_F15
9	17.951	169	2	0.213	0.045	4.331	2853	WS0108_F15
9	58.045	176	1	-0.376	0.081	4.878	2853	WS0108_F15
9	69.046	181	1	0.260	0.103	6.108	2853	WS0108_F15
9	81.073	183	1	-0.214	0.040	3.843	2853	WS0108_F15
12	14.406	230	4	-0.384	0.100	9.861	2853	WS0108_F15
1	95.572	17	3	0.310	0.057	4.801	2854	WS0108_H02
2	95.880	34	3	0.248	0.053	6.493	2854	WS0108_H02
6	25.914	114	1	-0.246	0.093	5.195	2854	WS0108_H02
10	70.246	190	2	0.242	0.029	3.881	2854	WS0108_H02

10	78.479	193	3	-0.304	0.036	4.488	2854	WS0108_H02
11	60.464	221	2	0.297	0.048	5.989	2854	WS0108_H02
12	6.242	234	2	0.247	0.028	4.129	2854	WS0108_H02
3	9.953	42	3	0.196	0.055	4.595	2855	WS0108_J02
3	83.563	56	3	-0.214	0.048	3.971	2855	WS0108_J02
3	148.745	68	4	-0.235	0.038	4.002	2855	WS0108_J02
4	82.462	78	1	-0.205	0.068	4.072	2855	WS0108_J02
5	7.672	92	4	0.259	0.053	4.518	2855	WS0108_J02
5	16.219	93	1	-0.225	0.069	4.093	2855	WS0108_J02
5	56.008	101	2	0.206	0.039	3.966	2855	WS0108_J02
7	70.278	141	3	-0.188	0.057	4.729	2855	WS0108_J02
9	17.951	169	2	-0.299	0.062	6.328	2855	WS0108_J02
9	55.358	175	1	-0.335	0.093	5.757	2855	WS0108_J02
9	55.358	175	3	0.395	0.131	4.054	2855	WS0108_J02
9	69.046	181	2	0.337	0.099	5.527	2855	WS0108_J02
1	50.955	10	1	0.300	0.087	7.802	2856	WS0108_J19
5	7.672	92	1	-0.351	0.059	5.527	2856	WS0108_J19
5	16.219	93	1	-0.159	0.052	4.665	2856	WS0108_J19
6	95.249	126	1	-0.369	0.084	4.602	2856	WS0108_J19
9	58.045	176	1	-0.223	0.107	5.719	2856	WS0108_J19
9	69.046	181	1	0.423	0.108	6.032	2856	WS0108_J19
11	40.960	210	2	0.457	0.126	6.428	2856	WS0108_J19
13	0.000	243	4	0.275	0.184	6.572	2856	WS0108_J19
14	22.402	248	3	-0.238	0.084	8.599	2856	WS0108_J19
2	48.839	29	4	0.249	0.050	6.010	2857	WS0108_L23
11	27.405	204	4	-0.322	0.083	9.526	2857	WS0108_L23
6	54.066	119	4	0.203	0.037	4.115	2858	WS0108_M12
6	69.073	121	4	-0.270	0.059	5.085	2858	WS0108_M12
8	62.580	155	1	-0.284	0.103	3.883	2858	WS0108_M12
9	2.011	167	4	0.170	0.036	4.041	2858	WS0108_M12
9	17.951	169	2	-0.254	0.053	4.206	2858	WS0108_M12
3	116.958	63	4	-0.247	0.040	4.316	2859	WS0108_P02
4	82.462	78	1	-0.245	0.040	3.989	2859	WS0108_P02
5	7.672	92	4	0.301	0.062	4.429	2859	WS0108_P02
10	9.580	187	3	-0.311	0.108	5.250	2859	WS0108_P02
11	60.464	221	2	0.275	0.056	5.148	2859	WS0108_P02
11	64.014	222	3	0.187	0.054	4.531	2859	WS0108_P02
1	38.326	6	4	-0.350	0.051	4.834	2860	WS0108_P14
1	95.572	17	3	0.321	0.123	9.349	2860	WS0108_P14
1	133.992	24	2	0.208	0.048	4.737	2860	WS0108_P14
2	102.814	36	1	-0.290	0.038	3.861	2860	WS0108_P14
4	142.917	85	1	-0.175	0.044	3.927	2860	WS0108_P14
6	49.100	118	4	-0.211	0.095	3.851	2860	WS0108_P14
6	54.066	119	4	-0.313	0.069	7.560	2860	WS0108_P14
6	81.996	123	3	-0.333	0.083	4.237	2860	WS0108_P14
11	9.237	202	4	0.227	0.061	6.582	2860	WS0108_P14
11	27.405	204	4	-0.388	0.121	13.412	2860	WS0108_P14
12	0.000	226	3	0.198	0.068	5.380	2860	WS0108_P14
14	0.000	246	2	0.240	0.060	5.927	2860	WS0108_P14
3	9.953	42	4	-0.241	0.053	5.894	2861	WS00925_F15
4	142.917	85	1	-0.346	0.086	7.003	2861	WS00925_F15
5	3.484	90	2	0.294	0.086	5.756	2861	WS00925_F15
5	52.820	99	1	-0.298	0.067	5.393	2861	WS00925_F15
5	54.072	100	4	-0.200	0.037	3.867	2861	WS00925_F15

6	69.073	121	3	-0.244	0.065	5.403	2861	WS00925_F15
6	76.376	122	1	0.382	0.134	5.030	2861	WS00925_F15
6	89.401	125	1	-0.317	0.066	5.177	2861	WS00925_F15
10	9.580	187	2	0.323	0.086	5.791	2861	WS00925_F15
13	1.815	244	4	0.251	0.048	4.734	2861	WS00925_F15
2	126.181	39	4	-0.667	0.167	13.061	2862	WS00925_F24
5	57.017	102	2	0.199	0.045	5.180	2862	WS00925_F24
7	15.555	130	2	0.341	0.056	6.315	2862	WS00925_F24
10	21.864	188	3	-0.294	0.080	6.552	2862	WS00925_F24
10	70.246	190	4	0.134	0.035	4.959	2862	WS00925_F24
1	50.955	10	1	-0.236	0.069	5.826	2863	WS00925_H02
3	141.443	65	3	0.332	0.113	6.050	2863	WS00925_H02
12	38.543	232	4	-0.272	0.125	3.881	2863	WS00925_H02
3	83.563	56	3	-0.191	0.058	4.268	2864	WS00925_H18
6	25.742	113	3	-0.232	0.058	4.249	2864	WS00925_H18
6	25.914	114	1	-0.220	0.046	4.314	2864	WS00925_H18
6	49.100	118	4	0.407	0.096	3.865	2864	WS00925_H18
6	54.066	119	4	0.183	0.036	4.120	2864	WS00925_H18
7	70.278	141	3	-0.223	0.061	4.447	2864	WS00925_H18
7	74.560	143	1	0.278	0.050	4.715	2864	WS00925_H18
7	91.487	146	3	-0.223	0.058	4.250	2864	WS00925_H18
10	78.479	193	4	-0.260	0.047	4.307	2864	WS00925_H18
11	57.661	220	4	0.240	0.038	4.413	2864	WS00925_H18
13	0.000	243	4	0.413	0.090	4.945	2864	WS00925_H18
1	50.955	10	1	-0.182	0.047	4.151	2865	WS00925_J06
1	107.333	19	3	-0.252	0.071	7.235	2865	WS00925_J06
3	141.443	65	3	0.309	0.079	5.037	2865	WS00925_J06
4	173.929	88	2	-0.165	0.042	4.140	2865	WS00925_J06
8	87.463	158	2	0.173	0.041	4.074	2865	WS00925_J06
9	3.127	168	1	0.393	0.155	4.074	2865	WS00925_J06
9	28.608	170	3	-0.215	0.037	4.021	2865	WS00925_J06
9	55.358	175	3	-0.585	0.143	9.031	2865	WS00925_J06
9	58.045	176	1	0.298	0.082	5.163	2865	WS00925_J06
9	59.963	177	1	0.337	0.083	7.333	2865	WS00925_J06
10	9.580	187	2	-0.303	0.099	5.975	2865	WS00925_J06
11	0.000	199	2	0.256	0.043	4.307	2865	WS00925_J06
11	9.237	202	4	-0.235	0.056	4.764	2865	WS00925_J06
12	0.000	226	3	-0.260	0.061	6.274	2865	WS00925_J06
12	14.406	230	4	0.288	0.073	6.136	2865	WS00925_J06
12	38.543	232	4	-0.255	0.158	3.966	2865	WS00925_J06
13	3.386	245	1	-0.235	0.047	4.203	2865	WS00925_J06
4	0.000	71	4	-0.193	0.034	4.740	2866	WS00925_J12
4	82.462	78	1	0.165	0.075	4.106	2866	WS00925_J12
6	26.350	115	2	-0.172	0.037	4.880	2866	WS00925_J12
8	0.000	148	4	0.221	0.032	4.450	2866	WS00925_J12
9	28.608	170	2	-0.209	0.030	4.012	2866	WS00925_J12
13	1.815	244	2	-0.210	0.029	3.898	2866	WS00925_J12
4	0.000	71	4	0.207	0.052	4.463	2867	WS00925_K17
4	173.929	88	2	-0.213	0.042	4.339	2867	WS00925_K17
6	69.073	121	3	0.254	0.040	4.156	2867	WS00925_K17
2	101.076	35	3	0.194	0.048	3.931	2868	WS00925_L05
3	137.891	64	1	-0.212	0.056	4.104	2868	WS00925_L05
4	173.929	88	2	-0.127	0.033	3.912	2868	WS00925_L05
6	25.914	114	1	-0.297	0.056	4.156	2868	WS00925_L05

10	9.580	187	2	-0.211	0.065	4.506	2868	WS00925_L05
11	40.960	210	2	-0.386	0.061	4.224	2868	WS00925_L05
11	46.026	217	1	-0.206	0.098	6.749	2868	WS00925_L05
1	133.992	24	2	0.211	0.035	3.975	2869	WS01016_C18
2	75.550	31	2	0.197	0.034	3.972	2869	WS01016_C18
2	101.076	35	3	-0.292	0.049	4.515	2869	WS01016_C18
6	106.702	128	2	-0.389	0.077	3.929	2869	WS01016_C18
8	50.001	154	2	-0.264	0.064	6.907	2869	WS01016_C18
8	95.937	159	3	0.224	0.081	7.298	2869	WS01016_C18
10	9.580	187	3	-0.277	0.107	4.370	2869	WS01016_C18
12	22.441	227	3	0.296	0.102	9.239	2869	WS01016_C18
12	0.000	233	1	-0.224	0.052	3.903	2869	WS01016_C18
1	69.854	13	2	0.224	0.041	4.853	2870	WS01016_H21
1	71.592	14	2	0.215	0.037	4.437	2870	WS01016_H21
6	87.891	124	2	-0.157	0.034	3.953	2870	WS01016_H21
9	81.073	183	1	0.235	0.074	5.310	2870	WS01016_H21
11	64.014	222	4	-0.226	0.046	5.064	2870	WS01016_H21
12	6.242	234	2	-0.204	0.031	4.127	2870	WS01016_H21
12	6.242	234	4	0.368	0.043	5.003	2870	WS01016_H21
14	0.000	246	2	-0.230	0.041	5.092	2870	WS01016_H21
3	15.147	44	2	0.222	0.049	4.551	2871	WS0084_N16
3	116.958	63	4	-0.199	0.046	5.576	2871	WS0084_N16
6	56.188	120	2	-0.258	0.033	4.035	2871	WS0084_N16
6	81.996	123	3	0.349	0.137	5.298	2871	WS0084_N16
11	64.014	222	4	-0.289	0.044	4.729	2871	WS0084_N16
12	6.242	234	2	-0.177	0.032	4.116	2871	WS0084_N16
14	0.000	246	2	-0.190	0.042	4.830	2871	WS0084_N16
1	69.854	13	2	0.382	0.133	15.898	2872	WS0022_P18
1	71.592	14	2	0.379	0.133	15.806	2872	WS0022_P18
1	77.177	15	2	0.362	0.119	14.230	2872	WS0022_P18
1	95.572	17	2	0.339	0.082	9.974	2872	WS0022_P18
3	9.953	42	3	0.176	0.041	4.275	2872	WS0022_P18
12	14.406	230	4	0.221	0.052	4.381	2872	WS0022_P18
14	0.000	246	2	-0.205	0.034	4.050	2872	WS0022_P18
1	69.854	13	2	0.245	0.048	5.766	2873	WS0085_E01
1	71.592	14	2	0.242	0.048	5.676	2873	WS0085_E01
1	77.177	15	2	0.228	0.041	4.887	2873	WS0085_E01
2	48.839	29	4	0.308	0.041	3.917	2873	WS0085_E01
2	95.880	34	3	0.242	0.055	5.027	2873	WS0085_E01
3	9.953	42	3	0.200	0.044	4.115	2873	WS0085_E01
10	93.347	196	1	0.430	0.124	4.445	2873	WS0085_E01
11	27.405	204	4	-0.374	0.080	4.747	2873	WS0085_E01
1	69.854	13	2	0.216	0.047	5.476	2874	WS0023_H04
1	71.592	14	2	0.199	0.041	4.701	2874	WS0023_H04
1	77.177	15	2	0.220	0.049	5.706	2874	WS0023_H04
1	95.572	17	2	0.256	0.044	5.074	2874	WS0023_H04
2	48.839	29	4	0.399	0.111	10.546	2874	WS0023_H04
3	137.891	64	3	0.256	0.051	4.356	2874	WS0023_H04
5	52.820	99	3	-0.301	0.070	6.370	2874	WS0023_H04
5	56.008	101	2	0.226	0.040	4.389	2874	WS0023_H04
5	57.017	102	2	-0.234	0.044	4.988	2874	WS0023_H04
2	120.617	38	4	-0.235	0.045	4.730	2875	WS0105_M20
3	116.958	63	4	-0.250	0.036	3.917	2875	WS0105_M20
4	142.917	85	1	-0.223	0.058	4.908	2875	WS0105_M20

6	54.066	119	4	0.218	0.042	4.511	2875	WS0105_M20
12	6.242	234	2	0.258	0.036	4.542	2875	WS0105_M20
13	0.000	243	4	0.600	0.193	9.678	2875	WS0105_M20
2	93.504	32	4	-0.241	0.052	5.951	2876	WS0106_C18
2	101.076	35	4	0.298	0.033	4.854	2876	WS0106_C18
4	124.132	83	1	0.560	0.136	6.515	2876	WS0106_C18
6	49.100	118	4	0.430	0.130	5.983	2876	WS0106_C18
8	50.001	154	2	-0.163	0.055	6.016	2876	WS0106_C18
8	62.580	155	1	-0.381	0.084	4.053	2876	WS0106_C18
9	28.608	170	2	0.289	0.050	5.279	2876	WS0106_C18
9	32.356	171	2	0.272	0.056	5.811	2876	WS0106_C18
9	49.013	173	2	-0.193	0.062	6.853	2876	WS0106_C18
11	43.491	214	4	0.182	0.039	4.489	2876	WS0106_C18
3	14.500	43	1	-0.269	0.045	3.965	2877	WS00923_K06
6	56.188	120	2	-0.283	0.056	5.793	2877	WS00923_K06
7	75.883	144	2	0.427	0.093	4.754	2877	WS00923_K06
7	75.883	144	4	-0.269	0.054	4.786	2877	WS00923_K06
8	62.580	155	1	-0.371	0.122	4.912	2877	WS00923_K06
14	0.000	246	2	-0.207	0.066	5.530	2877	WS00923_K06
1	107.333	19	4	-0.143	0.047	5.045	2878	WS00923_N12
3	9.953	42	3	-0.233	0.089	8.104	2878	WS00923_N12
4	116.928	82	4	0.277	0.054	5.016	2878	WS00923_N12
8	78.389	157	1	0.328	0.068	6.541	2878	WS00923_N12
8	87.463	158	1	-0.338	0.086	8.320	2878	WS00923_N12
9	0.000	166	1	0.191	0.064	5.987	2878	WS00923_N12
11	27.405	204	1	-0.312	0.050	5.091	2878	WS00923_N12
11	40.960	210	2	0.391	0.080	4.338	2878	WS00923_N12
4	173.929	88	2	-0.223	0.051	6.462	2879	WS0036_N20
5	56.008	101	2	0.228	0.048	6.104	2879	WS0036_N20
11	0.000	199	3	-0.260	0.053	5.184	2879	WS0036_N20
11	35.604	207	3	0.252	0.075	7.482	2879	WS0036_N20
11	37.143	209	3	-0.244	0.070	7.015	2879	WS0036_N20
13	1.815	244	2	-0.265	0.048	6.099	2879	WS0036_N20
6	56.188	120	2	0.226	0.048	4.088	2880	WS0037_A14
6	89.401	125	1	-0.271	0.055	4.829	2880	WS0037_A14
12	22.441	227	1	0.262	0.050	4.402	2880	WS0037_A14
12	28.493	228	2	0.359	0.087	5.582	2880	WS0037_A14
12	6.242	234	4	-0.313	0.060	3.940	2880	WS0037_A14
1	133.992	24	2	0.258	0.056	6.401	2881	WS0037_D06
3	9.953	42	4	-0.175	0.052	6.013	2881	WS0037_D06
3	15.147	44	2	-0.301	0.091	5.585	2881	WS0037_D06
4	55.925	76	1	0.213	0.051	4.210	2881	WS0037_D06
8	11.974	150	1	0.226	0.053	3.943	2881	WS0037_D06
9	55.358	175	1	0.304	0.054	4.100	2881	WS0037_D06
9	59.963	177	4	-0.290	0.082	8.905	2881	WS0037_D06
11	27.405	204	4	0.278	0.043	5.122	2881	WS0037_D06
11	45.989	216	1	-0.228	0.064	4.718	2881	WS0037_D06
11	56.921	219	2	-0.290	0.086	4.208	2881	WS0037_D06
14	46.637	252	4	0.282	0.033	3.897	2881	WS0037_D06
1	62.128	11	1	-0.526	0.100	5.727	2882	WS0037_E06
1	107.333	19	4	-0.190	0.044	4.575	2882	WS0037_E06
5	52.820	99	1	-0.363	0.072	8.175	2882	WS0037_E06
5	56.008	101	1	0.223	0.048	5.149	2882	WS0037_E06
8	62.580	155	1	0.507	0.144	8.333	2882	WS0037_E06

8	78.389	157	1	0.196	0.056	6.052	2882	WS0037_E06
8	87.463	158	1	-0.199	0.058	6.222	2882	WS0037_E06
8	87.463	158	2	0.161	0.056	4.401	2882	WS0037_E06
8	95.937	159	3	0.202	0.062	5.276	2882	WS0037_E06
9	3.127	168	1	0.409	0.088	4.844	2882	WS0037_E06
10	93.347	196	1	0.401	0.077	3.861	2882	WS0037_E06
11	9.237	202	4	-0.231	0.058	5.907	2882	WS0037_E06
11	57.661	220	4	0.235	0.067	6.892	2882	WS0037_E06
11	64.014	222	4	-0.378	0.100	9.609	2882	WS0037_E06
1	40.464	7	3	0.209	0.043	4.692	2883	WS0037_I07
1	45.794	9	3	-0.250	0.048	5.006	2883	WS0037_I07
2	95.880	34	1	-0.318	0.044	4.926	2883	WS0037_I07
7	75.883	144	2	0.395	0.087	4.161	2883	WS0037_I07
7	91.487	146	3	-0.280	0.065	7.165	2883	WS0037_I07
10	35.204	189	1	-0.383	0.074	8.211	2883	WS0037_I07
10	77.676	192	1	-0.293	0.100	10.956	2883	WS0037_I07
10	80.088	194	1	-0.480	0.155	17.050	2883	WS0037_I07
10	93.347	196	1	0.654	0.134	10.298	2883	WS0037_I07
10	104.214	198	4	-0.320	0.063	4.814	2883	WS0037_I07
11	48.979	218	2	-0.208	0.058	4.673	2883	WS0037_I07
14	0.000	246	1	-0.214	0.039	4.304	2883	WS0037_I07
1	62.128	11	1	-0.490	0.115	4.571	2884	WS0037_J24
2	93.504	32	4	-0.259	0.050	4.108	2884	WS0037_J24
3	15.147	44	2	-0.380	0.107	6.508	2884	WS0037_J24
3	20.512	46	2	-0.187	0.048	5.351	2884	WS0037_J24
4	116.928	82	2	-0.262	0.037	4.139	2884	WS0037_J24
10	70.246	190	2	0.284	0.044	4.918	2884	WS0037_J24
11	0.000	199	2	-0.219	0.044	4.879	2884	WS0037_J24
11	64.014	222	4	-0.251	0.038	3.931	2884	WS0037_J24
13	3.386	245	1	-0.200	0.065	4.968	2884	WS0037_J24
1	2.788	2	4	-0.256	0.043	4.284	2885	WS0037_N17
3	9.953	42	3	-0.159	0.044	4.263	2885	WS0037_N17
3	14.500	43	1	-0.351	0.051	5.520	2885	WS0037_N17
3	17.158	45	1	0.198	0.053	5.601	2885	WS0037_N17
7	73.169	142	1	0.605	0.129	6.124	2885	WS0037_N17
8	11.974	150	1	-0.213	0.058	6.174	2885	WS0037_N17
11	37.143	209	3	0.246	0.041	4.085	2885	WS0037_N17
1	40.464	7	4	0.280	0.037	4.105	2886	WS0037_O20
3	141.443	65	4	0.364	0.125	3.864	2886	WS0037_O20
4	173.929	88	2	-0.200	0.040	4.623	2886	WS0037_O20
6	87.891	124	2	0.186	0.033	3.871	2886	WS0037_O20
6	89.401	125	2	0.283	0.050	5.832	2886	WS0037_O20
7	75.883	144	4	-0.286	0.046	5.264	2886	WS0037_O20
10	93.347	196	1	0.401	0.108	3.856	2886	WS0037_O20
12	28.493	228	2	-0.369	0.080	6.211	2886	WS0037_O20
1	69.854	13	2	0.268	0.069	9.060	2887	WS00927_A18
1	71.592	14	2	0.264	0.068	8.894	2887	WS00927_A18
1	77.177	15	2	0.260	0.065	8.484	2887	WS00927_A18
3	80.211	55	4	0.215	0.034	4.171	2887	WS00927_A18
3	116.958	63	4	-0.161	0.041	5.073	2887	WS00927_A18
6	87.891	124	2	-0.219	0.046	6.051	2887	WS00927_A18
8	50.001	154	1	-0.230	0.096	5.595	2887	WS00927_A18
9	17.951	169	2	-0.240	0.031	3.937	2887	WS00927_A18
11	41.984	212	2	-0.199	0.031	4.144	2887	WS00927_A18

1	77.177	15	2	0.202	0.032	4.558	2888	WS00927_B01
1	131.686	23	2	-0.477	0.059	6.461	2888	WS00927_B01
3	137.891	64	3	0.259	0.084	4.771	2888	WS00927_B01
3	154.505	69	4	-0.204	0.045	6.132	2888	WS00927_B01
4	82.462	78	2	0.145	0.033	4.799	2888	WS00927_B01
4	116.928	82	2	-0.232	0.028	3.952	2888	WS00927_B01
6	69.073	121	4	-0.193	0.039	4.901	2888	WS00927_B01
6	106.702	128	2	0.421	0.136	4.772	2888	WS00927_B01
9	49.013	173	2	0.180	0.033	4.548	2888	WS00927_B01
9	59.963	177	4	-0.258	0.031	4.066	2888	WS00927_B01
10	78.479	193	4	-0.139	0.030	3.895	2888	WS00927_B01
11	43.491	214	4	-0.149	0.028	3.922	2888	WS00927_B01
11	57.661	220	4	0.190	0.044	5.648	2888	WS00927_B01
11	64.014	222	4	-0.283	0.048	6.088	2888	WS00927_B01
1	2.788	2	4	-0.202	0.037	4.673	2889	WS00927_C05
1	131.686	23	2	-0.547	0.105	7.208	2889	WS00927_C05
3	14.500	43	2	-0.146	0.036	4.327	2889	WS00927_C05
3	15.147	44	2	0.225	0.067	5.559	2889	WS00927_C05
3	116.958	63	4	-0.177	0.035	3.902	2889	WS00927_C05
5	54.072	100	4	0.247	0.034	3.948	2889	WS00927_C05
6	54.066	119	4	0.187	0.052	5.862	2889	WS00927_C05
6	56.188	120	2	-0.282	0.045	4.421	2889	WS00927_C05
9	0.000	166	1	-0.166	0.054	4.135	2889	WS00927_C05
10	104.214	198	4	-0.308	0.048	7.292	2889	WS00927_C05
11	45.989	216	1	-0.210	0.056	4.259	2889	WS00927_C05
11	64.014	222	4	-0.356	0.054	5.111	2889	WS00927_C05
3	137.891	64	3	0.235	0.063	5.042	2890	WS00927_C15
4	142.917	85	1	0.277	0.062	3.908	2890	WS00927_C15
4	173.929	88	2	-0.218	0.048	5.993	2890	WS00927_C15
5	3.484	90	2	-0.197	0.051	4.307	2890	WS00927_C15
7	17.610	131	3	-0.235	0.108	4.224	2890	WS00927_C15
7	75.883	144	2	0.439	0.061	5.189	2890	WS00927_C15
9	49.013	173	2	0.226	0.041	4.779	2890	WS00927_C15
12	6.242	234	2	-0.205	0.034	4.924	2890	WS00927_C15
1	131.686	23	2	-0.405	0.055	4.666	2891	WS00927_D17
2	48.839	29	4	0.319	0.061	6.080	2891	WS00927_D17
3	116.958	63	4	-0.159	0.042	4.347	2891	WS00927_D17
5	7.672	92	4	0.216	0.047	4.796	2891	WS00927_D17
9	17.951	169	2	-0.330	0.058	8.139	2891	WS00927_D17
9	49.013	173	2	0.220	0.041	5.743	2891	WS00927_D17
9	69.046	181	2	0.263	0.078	4.380	2891	WS00927_D17
11	27.405	204	4	-0.283	0.048	4.401	2891	WS00927_D17
1	43.578	8	3	-0.213	0.046	4.417	2892	WS00927_E10
1	62.128	11	2	0.298	0.078	4.149	2892	WS00927_E10
1	69.854	13	2	0.220	0.048	5.848	2892	WS00927_E10
1	71.592	14	2	0.216	0.047	5.686	2892	WS00927_E10
1	77.177	15	2	0.246	0.060	7.326	2892	WS00927_E10
1	95.572	17	2	0.215	0.032	3.930	2892	WS00927_E10
5	54.072	100	4	0.187	0.045	4.245	2892	WS00927_E10
5	57.017	102	2	-0.209	0.035	4.243	2892	WS00927_E10
1	82.301	16	3	-0.191	0.042	3.935	2893	WS00927_F18
3	80.211	55	4	0.227	0.065	6.005	2893	WS00927_F18
10	21.864	188	1	0.415	0.141	9.981	2893	WS00927_F18
11	27.405	204	1	0.250	0.062	4.569	2893	WS00927_F18

11	27.405	204	4	-0.339	0.071	6.797	2893	WS00927_F18
11	45.989	216	1	-0.232	0.070	5.047	2893	WS00927_F18
1	95.572	17	2	-0.238	0.049	4.964	2894	WS00927_G03
2	120.617	38	4	0.304	0.063	6.075	2894	WS00927_G03
2	127.147	40	4	0.248	0.064	5.864	2894	WS00927_G03
3	9.953	42	3	-0.197	0.048	4.525	2894	WS00927_G03
3	9.953	42	4	0.179	0.042	3.913	2894	WS00927_G03
4	173.929	88	2	0.240	0.065	6.477	2894	WS00927_G03
6	45.897	117	1	-0.223	0.051	4.509	2894	WS00927_G03
7	15.555	130	3	0.266	0.067	5.423	2894	WS00927_G03
10	70.246	190	4	0.260	0.049	4.394	2894	WS00927_G03
10	78.479	193	4	0.311	0.088	7.662	2894	WS00927_G03
11	57.661	220	4	-0.253	0.052	4.817	2894	WS00927_G03
13	0.000	243	4	-0.369	0.118	4.147	2894	WS00927_G03
13	1.815	244	2	0.328	0.088	9.245	2894	WS00927_G03
13	1.815	244	4	-0.300	0.051	4.796	2894	WS00927_G03
2	48.839	29	4	0.224	0.044	3.947	2895	WS0264_D21
3	15.147	44	2	0.365	0.120	6.169	2895	WS0264_D21
5	16.219	93	2	-0.221	0.045	4.664	2895	WS0264_D21
5	31.191	95	1	0.313	0.093	7.725	2895	WS0264_D21
5	42.979	97	1	-0.482	0.170	14.198	2895	WS0264_D21
5	52.820	99	1	-0.360	0.116	9.398	2895	WS0264_D21
5	56.008	101	1	0.347	0.119	9.966	2895	WS0264_D21
6	26.350	115	2	0.184	0.041	4.244	2895	WS0264_D21
9	55.358	175	1	0.265	0.046	3.939	2895	WS0264_D21
10	21.864	188	1	0.319	0.049	4.065	2895	WS0264_D21
11	40.960	210	4	0.389	0.100	8.642	2895	WS0264_D21
11	44.884	215	4	-0.318	0.061	5.358	2895	WS0264_D21
11	48.979	218	4	0.325	0.063	5.748	2895	WS0264_D21
1	40.464	7	4	0.298	0.043	3.911	2896	WS0264_E05
3	83.563	56	3	-0.149	0.050	5.130	2896	WS0264_E05
6	69.073	121	4	-0.278	0.089	7.057	2896	WS0264_E05
8	11.974	150	1	0.248	0.085	6.018	2896	WS0264_E05
8	50.001	154	1	0.245	0.051	4.317	2896	WS0264_E05
9	17.951	169	2	0.187	0.048	6.237	2896	WS0264_E05
9	28.608	170	2	-0.369	0.096	12.064	2896	WS0264_E05
9	32.356	171	2	-0.216	0.040	4.638	2896	WS0264_E05
10	78.479	193	4	-0.211	0.058	4.729	2896	WS0264_E05
11	45.989	216	1	-0.212	0.075	5.313	2896	WS0264_E05
2	102.814	36	2	0.182	0.053	5.922	2897	WS0264_F11
4	92.039	79	2	0.133	0.037	3.892	2897	WS0264_F11
4	97.576	80	2	-0.220	0.068	7.196	2897	WS0264_F11
7	59.789	137	3	0.346	0.068	6.469	2897	WS0264_F11
7	70.278	141	3	-0.482	0.171	15.495	2897	WS0264_F11
7	91.487	146	3	-0.285	0.086	7.689	2897	WS0264_F11
9	0.000	163	1	-0.421	0.082	3.907	2897	WS0264_F11
9	28.608	170	3	0.308	0.062	4.787	2897	WS0264_F11
10	35.204	189	1	-0.317	0.042	3.854	2897	WS0264_F11
10	70.246	190	2	-0.187	0.049	4.855	2897	WS0264_F11
10	80.088	194	1	-0.229	0.074	6.499	2897	WS0264_F11
10	93.347	196	1	0.680	0.232	11.091	2897	WS0264_F11
10	104.214	198	4	-0.315	0.062	4.932	2897	WS0264_F11
11	64.014	222	3	0.325	0.073	6.701	2897	WS0264_F11
6	81.996	123	3	0.306	0.117	5.696	2898	WS0264_G03

7	70.278	141	3	-0.233	0.059	5.274	2898	WS0264_G03
10	93.347	196	1	0.514	0.130	6.331	2898	WS0264_G03
12	22.629	237	1	0.223	0.061	4.504	2898	WS0264_G03
12	32.473	238	1	0.231	0.064	4.695	2898	WS0264_G03
2	120.617	38	4	-0.206	0.055	5.745	2899	WS0264_H12
4	124.132	83	1	0.500	0.110	5.572	2899	WS0264_H12
4	142.917	85	1	-0.227	0.046	5.293	2899	WS0264_H12
5	0.000	89	1	0.424	0.105	11.268	2899	WS0264_H12
5	0.000	89	3	0.249	0.089	7.444	2899	WS0264_H12
5	7.672	92	1	0.233	0.061	8.823	2899	WS0264_H12
5	16.219	93	1	0.312	0.101	11.607	2899	WS0264_H12
5	16.219	93	2	0.347	0.057	4.003	2899	WS0264_H12
5	31.191	95	1	0.406	0.083	9.792	2899	WS0264_H12
5	42.979	97	1	-0.254	0.055	6.383	2899	WS0264_H12
5	52.820	99	1	-0.215	0.047	5.355	2899	WS0264_H12
6	45.897	117	1	0.287	0.035	4.326	2899	WS0264_H12
7	62.787	139	4	0.351	0.078	6.105	2899	WS0264_H12
7	70.278	141	3	-0.229	0.075	7.263	2899	WS0264_H12
7	91.487	146	3	-0.127	0.052	5.057	2899	WS0264_H12
8	87.463	158	2	-0.149	0.086	7.076	2899	WS0264_H12
9	17.951	169	2	-0.149	0.060	3.851	2899	WS0264_H12
9	28.608	170	2	0.280	0.052	4.094	2899	WS0264_H12
1	45.794	9	1	-0.195	0.048	4.415	2900	WS0264_I08
2	126.181	39	4	0.501	0.099	5.906	2900	WS0264_I08
3	9.953	42	4	-0.158	0.041	4.304	2900	WS0264_I08
3	144.593	66	4	-0.193	0.044	5.363	2900	WS0264_I08
3	148.745	68	4	0.273	0.034	4.093	2900	WS0264_I08
3	154.505	69	4	-0.293	0.054	6.329	2900	WS0264_I08
4	0.000	71	4	-0.254	0.036	4.129	2900	WS0264_I08
11	9.237	202	4	0.202	0.045	5.053	2900	WS0264_I08
12	14.406	230	4	-0.384	0.107	11.782	2900	WS0264_I08
1	131.686	23	1	-0.645	0.157	7.076	2901	WS0264_J19
4	43.146	74	3	0.241	0.054	4.622	2901	WS0264_J19
8	50.001	154	2	-0.141	0.036	4.184	2901	WS0264_J19
12	22.441	227	1	-0.197	0.055	4.004	2901	WS0264_J19
4	116.928	82	2	0.300	0.036	4.399	2902	WS0264_K07
6	87.891	124	2	0.206	0.043	5.174	2902	WS0264_K07
6	89.401	125	2	0.297	0.038	5.076	2902	WS0264_K07
8	50.001	154	2	-0.266	0.077	5.427	2902	WS0264_K07
10	21.864	188	1	0.367	0.101	7.023	2902	WS0264_K07
14	0.000	246	2	-0.205	0.048	5.619	2902	WS0264_K07
3	14.500	43	1	-0.273	0.034	3.977	2903	WS0058_G16
9	49.013	173	1	0.209	0.040	4.813	2903	WS0058_G16
9	81.073	183	1	0.255	0.038	4.469	2903	WS0058_G16
10	93.347	196	1	0.513	0.075	6.469	2903	WS0058_G16
12	14.406	230	4	0.234	0.069	4.326	2903	WS0058_G16
14	0.000	246	1	-0.242	0.050	5.794	2903	WS0058_G16
3	17.158	45	1	0.227	0.063	4.350	2904	WS0058_P07
4	0.000	71	4	0.237	0.042	4.273	2904	WS0058_P07
4	173.929	88	2	-0.210	0.041	4.894	2904	WS0058_P07
12	6.242	234	2	-0.345	0.076	9.030	2904	WS0058_P07
1	2.788	2	4	-0.223	0.030	5.114	2905	WS00924_A16
1	38.326	6	4	-0.327	0.047	5.794	2905	WS00924_A16
1	40.464	7	4	0.348	0.050	6.344	2905	WS00924_A16

6	25.914	114	1	-0.287	0.070	7.166	2905	WS00924_A16
7	73.169	142	1	0.527	0.088	5.511	2905	WS00924_A16
9	3.127	168	1	-0.257	0.032	4.106	2905	WS00924_A16
10	78.479	193	4	-0.223	0.033	4.063	2905	WS00924_A16
10	93.347	196	1	0.458	0.127	5.025	2905	WS00924_A16
11	46.026	217	1	-0.316	0.082	8.446	2905	WS00924_A16
12	14.406	230	4	0.282	0.032	4.073	2905	WS00924_A16
1	131.686	23	2	-0.422	0.053	4.506	2906	WS00924_D11
3	148.745	68	1	-0.322	0.089	6.525	2906	WS00924_D11
6	56.188	120	2	-0.277	0.046	5.192	2906	WS00924_D11
6	81.996	123	3	0.316	0.115	4.686	2906	WS00924_D11
11	57.661	220	4	0.153	0.034	4.060	2906	WS00924_D11
11	64.014	222	4	-0.387	0.083	9.465	2906	WS00924_D11
13	0.000	243	4	0.297	0.134	4.220	2906	WS00924_D11
14	0.000	246	2	-0.197	0.042	4.878	2906	WS00924_D11
6	106.702	128	2	0.596	0.153	8.747	2907	WS0083_D01
7	73.169	142	1	0.515	0.108	4.236	2907	WS0083_D01
1	131.686	23	2	-0.416	0.058	4.036	2908	WS0083_D21
1	133.992	24	2	-0.243	0.048	6.029	2908	WS0083_D21
3	9.953	42	3	0.258	0.055	4.922	2908	WS0083_D21
3	95.157	59	2	0.216	0.048	5.566	2908	WS0083_D21
4	124.132	83	1	-0.455	0.152	4.146	2908	WS0083_D21
8	50.001	154	1	-0.184	0.049	3.849	2908	WS0083_D21
9	0.000	163	2	-0.466	0.083	4.182	2908	WS0083_D21
9	17.951	169	2	-0.259	0.041	5.213	2908	WS0083_D21
1	131.686	23	2	-0.506	0.092	5.714	2909	WS0083_G05
2	48.839	29	4	0.280	0.051	5.802	2909	WS0083_G05
2	93.504	32	4	-0.293	0.041	4.417	2909	WS0083_G05
3	116.958	63	4	-0.243	0.062	7.016	2909	WS0083_G05
5	7.672	92	4	0.259	0.054	5.550	2909	WS0083_G05
10	104.214	198	2	-0.267	0.043	4.809	2909	WS0083_G05
12	38.543	232	4	0.348	0.093	3.855	2909	WS0083_G05
3	9.953	42	4	-0.193	0.049	4.920	2910	WS0083_G22
3	137.891	64	1	0.309	0.089	7.455	2910	WS0083_G22
3	154.505	69	1	-0.242	0.057	4.797	2910	WS0083_G22
6	106.702	128	2	0.421	0.087	4.325	2910	WS0083_G22
7	73.169	142	1	-0.507	0.098	4.110	2910	WS0083_G22
8	50.001	154	2	0.228	0.047	5.068	2910	WS0083_G22
9	59.963	177	4	-0.235	0.045	4.313	2910	WS0083_G22
11	29.069	205	2	-0.179	0.046	4.490	2910	WS0083_G22
1	82.301	16	4	-0.268	0.063	6.529	2911	WS0083_J11
2	120.617	38	4	-0.328	0.066	6.718	2911	WS0083_J11
5	31.191	95	1	0.216	0.052	4.591	2911	WS0083_J11
6	26.350	115	2	0.216	0.046	4.600	2911	WS0083_J11
7	17.610	131	3	0.344	0.135	5.630	2911	WS0083_J11
7	105.477	147	2	0.289	0.073	4.261	2911	WS0083_J11
9	0.000	166	2	0.223	0.056	5.389	2911	WS0083_J11
11	40.960	210	2	-0.403	0.081	4.893	2911	WS0083_J11
12	14.406	230	4	-0.264	0.043	4.226	2911	WS0083_J11
14	22.402	248	3	-0.307	0.059	5.138	2911	WS0083_J11
1	131.686	23	2	-0.409	0.057	3.850	2912	WS0083_K10
3	116.958	63	4	-0.218	0.049	5.697	2912	WS0083_K10
6	81.996	123	3	0.300	0.114	5.345	2912	WS0083_K10
10	80.088	194	1	-0.255	0.045	3.920	2912	WS0083_K10

10	93.347	196	1	0.499	0.127	6.008	2912	WS0083_K10
11	27.405	204	1	0.222	0.044	3.905	2912	WS0083_K10
11	27.405	204	4	-0.268	0.036	4.117	2912	WS0083_K10
13	0.000	243	4	0.386	0.123	4.788	2912	WS0083_K10
13	3.386	245	1	-0.274	0.082	7.029	2912	WS0083_K10
6	26.350	115	2	0.161	0.041	3.901	2913	WS0083_M22
6	56.188	120	2	-0.273	0.047	4.507	2913	WS0083_M22
7	17.610	131	3	0.344	0.090	4.012	2913	WS0083_M22
13	0.000	243	4	0.348	0.086	4.072	2913	WS0083_M22
14	0.000	246	1	0.211	0.056	5.157	2913	WS0083_M22
1	131.686	23	2	-0.412	0.056	4.657	2914	WS0083_O01
5	54.072	100	4	0.199	0.036	3.891	2914	WS0083_O01
10	9.580	187	3	-0.289	0.078	4.113	2914	WS0083_O01
11	60.464	221	2	0.280	0.048	4.400	2914	WS0083_O01
14	0.000	246	1	0.228	0.048	3.849	2914	WS0083_O01
1	77.177	15	2	0.171	0.037	4.454	2915	WS00925_A14
3	15.147	44	2	0.273	0.060	4.873	2915	WS00925_A14
3	144.593	66	4	0.227	0.042	5.655	2915	WS00925_A14
5	54.072	100	4	0.270	0.064	7.161	2915	WS00925_A14
6	56.188	120	2	-0.261	0.032	3.904	2915	WS00925_A14
6	81.996	123	3	0.302	0.098	3.941	2915	WS00925_A14
9	59.963	177	4	0.183	0.034	3.860	2915	WS00925_A14
14	0.000	246	2	-0.165	0.033	4.058	2915	WS00925_A14
4	173.929	88	2	-0.188	0.042	5.016	2916	WS00925_D16
5	3.484	90	2	-0.211	0.061	4.083	2916	WS00925_D16
5	57.017	102	2	-0.199	0.040	4.853	2916	WS00925_D16
11	56.921	219	2	-0.333	0.122	6.447	2916	WS00925_D16
1	77.177	15	2	-0.193	0.049	4.930	2917	WS0031_C11
1	95.572	17	2	-0.262	0.061	6.142	2917	WS0031_C11
1	95.572	17	3	0.204	0.065	5.532	2917	WS0031_C11
3	15.147	44	2	-0.332	0.099	5.195	2917	WS0031_C11
4	55.925	76	1	0.239	0.061	5.728	2917	WS0031_C11
4	75.958	77	3	-0.296	0.101	4.510	2917	WS0031_C11
4	116.928	82	2	-0.217	0.039	4.001	2917	WS0031_C11
5	86.669	106	4	-0.405	0.087	4.182	2917	WS0031_C11
6	25.914	114	1	-0.243	0.050	4.431	2917	WS0031_C11
9	59.963	177	4	-0.205	0.035	4.245	2917	WS0031_C11
9	81.073	183	1	-0.335	0.094	8.247	2917	WS0031_C11
10	77.676	192	1	-0.131	0.047	4.099	2917	WS0031_C11
10	80.088	194	1	-0.234	0.071	6.220	2917	WS0031_C11
11	46.026	217	1	-0.214	0.048	4.223	2917	WS0031_C11
12	51.382	242	1	-0.388	0.098	4.715	2917	WS0031_C11
1	38.326	6	2	0.230	0.043	4.968	2918	WS01019_B01
4	82.462	78	2	-0.225	0.039	4.400	2918	WS01019_B01
4	97.576	80	2	0.221	0.038	5.058	2918	WS01019_B01
9	0.000	166	1	0.301	0.085	6.178	2918	WS01019_B01
9	17.951	169	1	-0.338	0.097	6.950	2918	WS01019_B01
10	104.214	198	4	-0.200	0.034	4.222	2918	WS01019_B01
12	32.473	238	1	-0.274	0.077	5.589	2918	WS01019_B01
14	0.000	246	2	0.190	0.046	5.395	2918	WS01019_B01
14	43.392	250	1	-0.224	0.087	3.843	2918	WS01019_B01
14	44.898	251	3	0.306	0.083	4.585	2918	WS01019_B01
1	22.419	4	3	0.289	0.119	4.644	2919	WS0031_P11
1	40.464	7	3	0.340	0.089	7.844	2919	WS0031_P11

1	43.578	8	3	0.359	0.097	6.126	2919	WS0031_P11
1	45.794	9	3	-0.246	0.074	6.832	2919	WS0031_P11
1	69.854	13	2	0.289	0.065	7.005	2919	WS0031_P11
1	71.592	14	2	0.284	0.063	6.783	2919	WS0031_P11
1	77.177	15	2	0.224	0.037	4.056	2919	WS0031_P11
1	82.301	16	4	-0.298	0.059	4.168	2919	WS0031_P11
11	9.237	202	4	-0.185	0.040	3.978	2919	WS0031_P11
12	22.441	227	1	0.251	0.087	7.133	2919	WS0031_P11
1	107.333	19	3	-0.206	0.039	4.517	2920	WS01019_I17
3	14.500	43	1	-0.282	0.045	5.543	2920	WS01019_I17
3	17.158	45	1	0.228	0.044	5.390	2920	WS01019_I17
9	49.013	173	1	0.195	0.036	4.409	2920	WS01019_I17
10	93.347	196	1	0.444	0.052	5.060	2920	WS01019_I17
1	2.788	2	4	-0.226	0.042	4.126	2921	WS00810_G05
6	81.996	123	3	0.291	0.055	4.060	2921	WS00810_G05
10	93.347	196	1	0.551	0.098	7.308	2921	WS00810_G05
5	56.008	101	1	0.245	0.038	4.104	2922	WS00810_G23
9	36.227	165	4	0.319	0.048	4.488	2922	WS00810_G23
11	0.000	199	3	0.216	0.049	4.099	2922	WS00810_G23
11	40.960	210	2	-0.387	0.082	4.225	2922	WS00810_G23
11	43.491	214	4	-0.252	0.074	6.382	2922	WS00810_G23
11	46.026	217	1	-0.383	0.112	11.859	2922	WS00810_G23
11	57.661	220	4	0.336	0.085	8.425	2922	WS00810_G23
11	64.014	222	4	-0.293	0.051	4.658	2922	WS00810_G23
12	6.242	234	2	-0.239	0.046	4.224	2922	WS00810_G23
1	22.419	4	3	-0.241	0.088	4.126	2923	WS00810_J12
1	40.464	7	4	0.312	0.041	4.712	2923	WS00810_J12
1	95.572	17	3	0.206	0.065	4.793	2923	WS00810_J12
3	141.443	65	4	0.298	0.096	4.665	2923	WS00810_J12
3	154.505	69	1	0.252	0.039	4.226	2923	WS00810_J12
9	28.608	170	3	0.286	0.078	5.217	2923	WS00810_J12
10	9.580	187	3	-0.430	0.163	7.561	2923	WS00810_J12
10	80.088	194	1	-0.280	0.057	6.057	2923	WS00810_J12
10	93.347	196	1	0.618	0.196	9.152	2923	WS00810_J12
11	40.960	210	2	-0.380	0.072	4.099	2923	WS00810_J12
11	46.026	217	1	-0.339	0.086	9.139	2923	WS00810_J12
11	57.661	220	4	0.252	0.042	4.873	2923	WS00810_J12
4	92.039	79	4	-0.278	0.043	4.330	2924	WS00810_K04
7	15.555	130	2	0.188	0.051	4.750	2924	WS00810_K04
7	62.787	139	4	-0.250	0.044	5.468	2924	WS00810_K04
7	75.883	144	2	0.396	0.076	4.611	2924	WS00810_K04
7	75.883	144	4	-0.257	0.043	4.401	2924	WS00810_K04
9	28.608	170	3	0.304	0.063	4.943	2924	WS00810_K04
9	49.013	173	1	0.233	0.040	4.274	2924	WS00810_K04
11	17.088	203	1	-0.269	0.052	5.014	2924	WS00810_K04
11	46.026	217	1	-0.183	0.042	4.015	2924	WS00810_K04
11	57.661	220	4	0.233	0.053	5.447	2924	WS00810_K04
12	28.493	228	2	-0.244	0.080	4.633	2924	WS00810_K04
3	148.745	68	1	-0.243	0.061	4.600	2925	WS00810_L23
4	82.462	78	2	0.228	0.063	7.054	2925	WS00810_L23
4	97.576	80	2	-0.203	0.049	5.461	2925	WS00810_L23
8	0.000	148	4	-0.224	0.045	4.857	2925	WS00810_L23
9	0.000	166	2	0.314	0.086	8.509	2925	WS00810_L23
9	2.011	167	4	-0.276	0.049	5.318	2925	WS00810_L23

9	49.013	173	2	0.196	0.045	5.224	2925	WS00810_L23
11	9.237	202	4	-0.198	0.037	3.950	2925	WS00810_L23
14	0.000	246	2	-0.202	0.035	4.002	2925	WS00810_L23
1	69.854	13	2	0.217	0.041	5.084	2926	WS00810_M16
1	71.592	14	2	0.212	0.039	4.883	2926	WS00810_M16
1	77.177	15	2	0.216	0.040	5.003	2926	WS00810_M16
3	116.958	63	4	-0.187	0.057	6.370	2926	WS00810_M16
4	173.929	88	2	-0.177	0.039	4.856	2926	WS00810_M16
6	87.891	124	2	-0.230	0.057	7.062	2926	WS00810_M16
6	89.401	125	2	-0.316	0.055	6.918	2926	WS00810_M16
11	43.491	214	4	-0.234	0.051	5.615	2926	WS00810_M16
11	57.661	220	4	0.168	0.045	5.068	2926	WS00810_M16
11	64.014	222	4	-0.307	0.068	7.747	2926	WS00810_M16
1	107.333	19	3	-0.192	0.041	4.524	2927	WS00810_O14
4	124.132	83	1	-0.463	0.206	4.246	2927	WS00810_O14
4	173.929	88	2	-0.185	0.031	4.108	2927	WS00810_O14
10	70.246	190	2	-0.276	0.057	7.731	2927	WS00810_O14
10	78.479	193	3	0.238	0.044	4.910	2927	WS00810_O14
12	6.242	234	2	-0.277	0.048	6.614	2927	WS00810_O14
2	120.617	38	4	-0.306	0.066	6.690	2928	WS00810_P12
2	126.181	39	4	0.380	0.078	3.902	2928	WS00810_P12
3	95.157	59	2	0.210	0.041	3.968	2928	WS00810_P12
3	116.958	63	4	-0.299	0.083	8.413	2928	WS00810_P12
6	54.066	119	4	0.186	0.040	4.056	2928	WS00810_P12
6	56.188	120	2	-0.258	0.040	4.126	2928	WS00810_P12
11	64.014	222	4	-0.365	0.071	6.640	2928	WS00810_P12
13	3.386	245	1	-0.216	0.051	4.145	2928	WS00810_P12
14	0.000	246	2	-0.167	0.039	3.898	2928	WS00810_P12
1	69.854	13	2	0.259	0.048	5.611	2929	WS00930_A10
1	71.592	14	2	0.246	0.042	4.934	2929	WS00930_A10
1	77.177	15	2	0.272	0.055	6.399	2929	WS00930_A10
4	124.132	83	1	-0.469	0.154	4.359	2929	WS00930_A10
5	57.017	102	4	-0.207	0.040	5.002	2929	WS00930_A10
6	56.188	120	2	-0.265	0.041	5.284	2929	WS00930_A10
6	81.996	123	3	0.217	0.107	4.643	2929	WS00930_A10
7	75.883	144	2	0.412	0.052	4.481	2929	WS00930_A10
10	93.347	196	1	0.406	0.091	3.958	2929	WS00930_A10
10	104.214	198	2	-0.171	0.036	4.603	2929	WS00930_A10
11	43.491	214	4	-0.114	0.029	3.879	2929	WS00930_A10
1	123.316	20	4	0.242	0.037	3.972	2930	WS00930_A20
1	131.686	23	2	-0.469	0.067	7.223	2930	WS00930_A20
2	48.839	29	4	0.349	0.069	7.277	2930	WS00930_A20
3	116.958	63	4	-0.167	0.037	4.933	2930	WS00930_A20
5	57.017	102	2	-0.206	0.033	4.352	2930	WS00930_A20
6	25.914	114	1	-0.267	0.065	3.854	2930	WS00930_A20
7	75.883	144	2	0.378	0.042	4.517	2930	WS00930_A20
11	27.405	204	4	-0.388	0.091	8.540	2930	WS00930_A20
11	60.464	221	2	0.280	0.054	6.986	2930	WS00930_A20
1	123.316	20	4	0.251	0.045	4.769	2931	WS00930_B17
1	131.686	23	2	-0.452	0.069	7.190	2931	WS00930_B17
3	116.958	63	4	-0.201	0.040	5.498	2931	WS00930_B17
6	69.073	121	4	-0.258	0.041	5.433	2931	WS00930_B17
8	39.090	152	4	-0.203	0.030	4.161	2931	WS00930_B17
11	27.405	204	1	0.229	0.076	5.266	2931	WS00930_B17

11	27.405	204	4	-0.302	0.052	6.971	2931	WS00930_B17
11	60.464	221	2	0.341	0.077	9.056	2931	WS00930_B17
2	93.504	32	4	-0.255	0.046	4.974	2932	WS00930_C05
11	60.464	221	2	0.258	0.056	6.211	2932	WS00930_C05
13	3.386	245	1	-0.194	0.059	4.597	2932	WS00930_C05
1	95.572	17	3	0.327	0.067	6.411	2933	WS00930_D07
2	48.839	29	4	0.311	0.097	7.281	2933	WS00930_D07
3	154.505	69	1	-0.242	0.046	4.677	2933	WS00930_D07
4	55.925	76	1	0.229	0.053	5.211	2933	WS00930_D07
6	76.376	122	1	0.299	0.145	4.924	2933	WS00930_D07
8	0.000	148	4	-0.382	0.124	10.912	2933	WS00930_D07
8	39.090	152	4	0.266	0.053	4.685	2933	WS00930_D07
8	87.463	158	2	0.295	0.107	9.192	2933	WS00930_D07
12	0.000	226	3	0.177	0.042	4.161	2933	WS00930_D07
3	15.147	44	2	0.374	0.111	9.598	2934	WS00930_D16
3	80.211	55	4	0.233	0.049	5.019	2934	WS00930_D16
11	27.405	204	4	-0.373	0.084	8.961	2934	WS00930_D16
3	15.147	44	2	0.327	0.091	6.909	2935	WS00930_F06
5	54.072	100	4	0.222	0.051	5.238	2935	WS00930_F06
5	57.017	102	4	-0.219	0.048	4.078	2935	WS00930_F06
6	56.188	120	2	-0.312	0.051	6.431	2935	WS00930_F06
8	50.001	154	1	-0.268	0.076	5.309	2935	WS00930_F06
11	27.405	204	4	-0.345	0.066	7.139	2935	WS00930_F06
1	2.788	2	3	-0.223	0.054	4.201	2936	WS00930_F15
1	62.128	11	1	-0.503	0.141	4.815	2936	WS00930_F15
1	71.592	14	2	0.179	0.033	4.098	2936	WS00930_F15
1	77.177	15	2	0.186	0.036	4.389	2936	WS00930_F15
1	123.316	20	4	0.417	0.151	11.547	2936	WS00930_F15
3	112.868	61	2	-0.209	0.040	4.945	2936	WS00930_F15
3	114.747	62	2	-0.239	0.052	6.434	2936	WS00930_F15
7	15.555	130	2	0.372	0.097	12.206	2936	WS00930_F15
11	0.000	199	2	-0.219	0.036	4.461	2936	WS00930_F15
12	28.493	228	2	-0.295	0.067	5.130	2936	WS00930_F15
13	0.000	243	4	0.263	0.129	4.378	2936	WS00930_F15
6	81.996	123	3	0.379	0.120	6.168	2937	WS0084_B21
11	27.405	204	4	-0.260	0.046	4.723	2937	WS0084_B21
3	137.891	64	3	0.246	0.043	4.154	2938	WS0084_I06
5	52.820	99	3	-0.316	0.066	6.115	2938	WS0084_I06
8	50.001	154	2	-0.229	0.058	6.728	2938	WS0084_I06
9	59.963	177	4	0.285	0.054	4.934	2938	WS0084_I06
11	40.960	210	2	-0.406	0.070	4.434	2938	WS0084_I06
11	40.960	210	4	0.309	0.058	5.339	2938	WS0084_I06
12	22.441	227	3	0.378	0.113	10.808	2938	WS0084_I06
12	38.543	232	4	-0.344	0.124	3.842	2938	WS0084_I06
12	6.242	234	2	-0.192	0.025	4.307	2938	WS0084_I06
2	48.839	29	4	0.343	0.087	8.086	2939	WS01015_I23
3	47.928	49	1	-0.284	0.096	6.213	2939	WS01015_I23
5	7.672	92	4	-0.274	0.059	5.220	2939	WS01015_I23
6	76.376	122	1	-0.398	0.264	6.885	2939	WS01015_I23
6	89.401	125	1	0.343	0.137	8.869	2939	WS01015_I23
8	50.001	154	2	0.205	0.038	4.483	2939	WS01015_I23
12	22.441	227	3	-0.254	0.060	5.633	2939	WS01015_I23
2	48.839	29	4	-0.226	0.052	3.897	2940	WS01015_N13
3	15.147	44	2	0.272	0.062	4.133	2940	WS01015_N13

5	54.072	100	4	0.190	0.056	3.988	2940	WS01015_N13
8	87.463	158	2	-0.182	0.037	5.181	2940	WS01015_N13
9	36.227	165	3	-0.201	0.042	4.941	2940	WS01015_N13
1	127.805	21	4	-0.313	0.076	8.610	2941	WS0035_M21
1	131.686	23	1	-0.587	0.109	6.022	2941	WS0035_M21
5	86.669	106	4	-0.448	0.141	7.751	2941	WS0035_M21
6	49.100	118	2	-0.313	0.069	3.843	2941	WS0035_M21
6	49.100	118	4	0.328	0.071	3.907	2941	WS0035_M21
6	81.996	123	4	0.334	0.077	3.971	2941	WS0035_M21
7	75.883	144	2	0.383	0.065	3.913	2941	WS0035_M21
7	105.477	147	2	-0.325	0.089	5.133	2941	WS0035_M21
8	11.974	150	1	0.226	0.055	4.106	2941	WS0035_M21
9	55.358	175	1	0.273	0.062	4.721	2941	WS0035_M21
10	80.088	194	2	-0.348	0.067	5.961	2941	WS0035_M21
12	28.493	228	2	-0.298	0.072	4.136	2941	WS0035_M21
12	14.406	230	4	0.363	0.055	6.303	2941	WS0035_M21
14	9.923	247	1	0.222	0.055	4.085	2941	WS0035_M21
1	50.955	10	1	0.252	0.065	5.364	2942	IS0011_L18
2	63.261	30	3	-0.173	0.060	6.496	2942	IS0011_L18
4	124.132	83	1	0.466	0.216	4.402	2942	IS0011_L18
6	87.891	124	2	-0.229	0.052	5.495	2942	IS0011_L18
6	89.401	125	2	-0.324	0.066	7.560	2942	IS0011_L18
6	106.702	128	2	0.443	0.109	5.333	2942	IS0011_L18
9	69.046	181	2	-0.388	0.105	4.766	2942	IS0011_L18
10	9.580	187	3	0.240	0.085	6.091	2942	IS0011_L18
10	21.864	188	3	-0.177	0.043	4.764	2942	IS0011_L18
11	17.088	203	1	-0.279	0.079	6.488	2942	IS0011_L18
11	29.069	205	1	0.268	0.047	4.074	2942	IS0011_L18
14	22.402	248	3	-0.310	0.046	5.106	2942	IS0011_L18
1	95.572	17	3	0.235	0.035	4.470	2943	WS0043_I23
6	26.350	115	2	0.208	0.034	4.480	2943	WS0043_I23
10	78.479	193	3	-0.336	0.068	8.930	2943	WS0043_I23
10	86.151	195	3	0.231	0.036	4.798	2943	WS0043_I23
11	0.000	199	3	0.248	0.044	5.756	2943	WS0043_I23
3	9.953	42	3	0.238	0.055	4.956	2944	WS00927_H18
6	56.188	120	2	-0.217	0.034	4.067	2944	WS00927_H18
7	17.610	131	3	-0.266	0.085	4.414	2944	WS00927_H18
9	17.951	169	2	-0.273	0.046	4.405	2944	WS00927_H18
9	49.013	173	2	0.215	0.040	3.905	2944	WS00927_H18
14	0.000	246	2	-0.221	0.046	5.616	2944	WS00927_H18
1	69.854	13	2	0.152	0.045	4.834	2945	WS0044_B22
1	71.592	14	2	0.134	0.037	4.031	2945	WS0044_B22
2	127.147	40	4	-0.230	0.045	4.422	2945	WS0044_B22
3	116.958	63	4	-0.239	0.041	4.181	2945	WS0044_B22
10	21.864	188	1	0.383	0.065	5.102	2945	WS0044_B22
10	35.204	189	1	0.275	0.051	4.084	2945	WS0044_B22
11	29.069	205	2	-0.165	0.037	3.976	2945	WS0044_B22
11	43.491	214	3	-0.229	0.057	4.973	2945	WS0044_B22
11	56.921	219	2	0.430	0.136	5.751	2945	WS0044_B22
12	38.543	232	4	-0.408	0.122	5.057	2945	WS0044_B22
1	77.177	15	2	0.158	0.041	4.266	2946	WS00927_K24
1	95.572	17	2	0.251	0.059	6.132	2946	WS00927_K24
1	127.805	21	4	-0.318	0.074	7.659	2946	WS00927_K24
3	15.147	44	2	0.344	0.084	4.408	2946	WS00927_K24

3	47.928	49	1	-0.247	0.052	4.387	2946	WS00927_K24
6	56.188	120	2	-0.249	0.047	4.980	2946	WS00927_K24
8	50.001	154	1	-0.268	0.054	4.825	2946	WS00927_K24
4	0.000	71	4	0.250	0.040	4.604	2947	WS00722_K02
4	173.929	88	2	-0.203	0.046	5.078	2947	WS00722_K02
7	15.555	130	2	-0.265	0.041	4.559	2947	WS00722_K02
9	81.073	183	1	0.268	0.058	4.553	2947	WS00722_K02
10	78.479	193	3	0.320	0.082	6.148	2947	WS00722_K02
12	6.242	234	2	-0.246	0.041	5.120	2947	WS00722_K02
3	114.747	62	2	-0.232	0.037	4.332	2948	WS00722_L15
5	54.072	100	4	0.210	0.047	4.819	2948	WS00722_L15
5	57.017	102	4	-0.273	0.070	6.898	2948	WS00722_L15
9	17.951	169	2	-0.244	0.038	3.930	2948	WS00722_L15
11	37.143	209	3	0.212	0.056	4.735	2948	WS00722_L15
12	28.493	228	2	-0.224	0.047	4.000	2948	WS00722_L15
1	133.992	24	2	0.182	0.048	4.729	2949	WS00723_A17
2	101.076	35	3	-0.243	0.045	4.821	2949	WS00723_A17
5	0.000	89	1	0.290	0.059	5.216	2949	WS00723_A17
7	74.560	143	1	-0.274	0.048	4.308	2949	WS00723_A17
9	28.608	170	2	0.268	0.053	5.466	2949	WS00723_A17
9	28.608	170	3	-0.349	0.088	9.445	2949	WS00723_A17
9	55.358	175	3	-0.563	0.124	6.056	2949	WS00723_A17
10	21.864	188	3	-0.245	0.042	4.507	2949	WS00723_A17
11	64.014	222	4	0.213	0.050	4.108	2949	WS00723_A17
12	28.493	228	2	0.222	0.078	4.423	2949	WS00723_A17
1	69.854	13	2	0.164	0.041	4.117	2950	WS00723_B05
1	77.177	15	2	0.166	0.042	4.215	2950	WS00723_B05
6	81.996	123	3	0.280	0.072	4.092	2950	WS00723_B05
9	36.227	165	3	-0.213	0.042	4.486	2950	WS00723_B05
10	78.479	193	3	0.259	0.042	4.427	2950	WS00723_B05
2	120.617	38	3	-0.175	0.042	4.272	2951	WS00723_F09
4	124.132	83	1	-0.441	0.149	3.886	2951	WS00723_F09
8	50.001	154	2	-0.188	0.046	4.914	2951	WS00723_F09
9	59.963	177	4	0.370	0.081	5.806	2951	WS00723_F09
11	0.000	199	3	0.436	0.110	11.523	2951	WS00723_F09
11	17.088	203	1	0.232	0.051	4.027	2951	WS00723_F09
11	48.979	218	2	0.242	0.054	5.922	2951	WS00723_F09
12	22.441	227	3	0.205	0.039	3.962	2951	WS00723_F09
3	17.158	45	1	0.251	0.056	4.920	2952	WS00723_H06
3	69.071	52	1	-0.289	0.059	3.935	2952	WS00723_H06
7	105.477	147	2	-0.237	0.094	4.826	2952	WS00723_H06
9	36.227	165	3	-0.186	0.035	3.991	2952	WS00723_H06
11	17.088	203	1	0.323	0.115	9.954	2952	WS00723_H06
11	35.604	207	3	-0.227	0.050	5.800	2952	WS00723_H06
11	37.143	209	3	0.255	0.063	7.276	2952	WS00723_H06
11	43.491	214	3	-0.198	0.038	4.438	2952	WS00723_H06
11	44.884	215	3	0.220	0.041	4.686	2952	WS00723_H06
12	32.473	238	1	-0.244	0.054	4.695	2952	WS00723_H06
14	43.392	250	1	-0.346	0.099	6.577	2952	WS00723_H06
4	142.917	85	1	-0.217	0.057	4.907	2953	WS00723_O08
5	54.072	100	4	0.279	0.060	5.871	2953	WS00723_O08
6	45.897	117	4	-0.276	0.069	5.967	2953	WS00723_O08
6	49.100	118	2	-0.357	0.251	7.633	2953	WS00723_O08
6	81.996	123	3	0.291	0.100	5.928	2953	WS00723_O08

7	59.789	137	3	0.294	0.077	6.967	2953	WS00723_O08
7	70.278	141	3	-0.347	0.119	10.776	2953	WS00723_O08
7	91.487	146	3	-0.221	0.061	5.490	2953	WS00723_O08
9	3.127	168	1	-0.331	0.097	3.952	2953	WS00723_O08
9	28.608	170	3	0.285	0.058	4.905	2953	WS00723_O08
10	93.347	196	1	0.429	0.074	4.412	2953	WS00723_O08
4	43.146	74	3	-0.187	0.047	4.962	2954	WS00934_A08
5	31.191	95	1	0.242	0.050	5.228	2954	WS00934_A08
6	81.996	123	3	0.323	0.061	4.237	2954	WS00934_A08
8	78.389	157	1	0.236	0.047	4.877	2954	WS00934_A08
8	87.463	158	1	-0.232	0.047	4.835	2954	WS00934_A08
9	59.963	177	1	-0.252	0.038	4.023	2954	WS00934_A08
14	0.000	246	1	-0.228	0.039	4.022	2954	WS00934_A08
14	0.000	246	2	-0.216	0.076	6.409	2954	WS00934_A08
2	126.181	39	2	-0.304	0.042	4.652	2955	WS0039_E17
3	9.953	42	4	0.152	0.041	4.833	2955	WS0039_E17
6	25.914	114	1	-0.164	0.049	4.020	2955	WS0039_E17
11	27.405	204	4	-0.330	0.065	6.256	2955	WS0039_E17
11	37.143	209	3	0.183	0.049	4.999	2955	WS0039_E17
11	45.989	216	2	0.234	0.037	4.140	2955	WS0039_E17
11	60.464	221	2	0.255	0.061	6.480	2955	WS0039_E17
1	50.955	10	1	-0.261	0.088	6.051	2956	WS0039_F19
1	62.128	11	1	-0.566	0.167	6.090	2956	WS0039_F19
3	47.928	49	1	0.225	0.059	4.066	2956	WS0039_F19
5	0.000	89	3	0.344	0.100	5.553	2956	WS0039_F19
6	89.401	125	2	0.216	0.035	4.119	2956	WS0039_F19
7	15.555	130	2	0.243	0.047	5.590	2956	WS0039_F19
9	81.073	183	1	0.208	0.056	3.989	2956	WS0039_F19
4	82.462	78	2	-0.113	0.022	4.427	2957	WS0039_I20
4	92.039	79	2	-0.218	0.033	4.440	2957	WS0039_I20
5	52.820	99	3	-0.365	0.081	8.257	2957	WS0039_I20
8	50.001	154	2	-0.216	0.056	7.237	2957	WS0039_I20
9	36.227	165	4	0.191	0.044	4.573	2957	WS0039_I20
9	28.608	170	2	0.194	0.029	3.962	2957	WS0039_I20
9	28.608	170	3	-0.341	0.048	4.639	2957	WS0039_I20
9	59.963	177	4	0.207	0.037	4.166	2957	WS0039_I20
3	83.563	56	3	0.214	0.056	6.740	2958	WS0039_K06
3	137.891	64	1	-0.266	0.049	5.656	2958	WS0039_K06
3	141.443	65	3	-0.389	0.089	7.303	2958	WS0039_K06
5	7.672	92	1	-0.288	0.048	4.130	2958	WS0039_K06
11	0.000	199	3	0.264	0.034	4.410	2958	WS0039_K06
11	27.405	204	1	-0.402	0.079	9.062	2958	WS0039_K06
11	29.069	205	1	-0.267	0.040	4.577	2958	WS0039_K06
14	0.000	246	1	-0.275	0.041	4.679	2958	WS0039_K06
14	46.637	252	3	0.297	0.037	4.487	2958	WS0039_K06
1	38.326	6	4	-0.278	0.041	4.407	2959	WS0039_P23
1	40.464	7	4	0.376	0.068	8.246	2959	WS0039_P23
1	95.572	17	3	0.205	0.073	4.365	2959	WS0039_P23
1	107.333	19	4	-0.296	0.059	4.122	2959	WS0039_P23
3	15.147	44	2	-0.303	0.067	4.238	2959	WS0039_P23
4	142.917	85	1	-0.308	0.069	6.339	2959	WS0039_P23
8	11.974	150	1	0.215	0.050	4.615	2959	WS0039_P23
11	46.026	217	1	-0.286	0.090	8.204	2959	WS0039_P23
1	107.333	19	4	-0.247	0.047	4.677	2960	WS0055_B18

4	142.917	85	1	-0.245	0.059	4.192	2960	WS0055_B18
6	69.073	121	3	0.231	0.073	5.655	2960	WS0055_B18
6	81.996	123	3	0.347	0.139	4.983	2960	WS0055_B18
8	62.580	155	1	0.287	0.131	4.684	2960	WS0055_B18
9	17.951	169	2	-0.235	0.046	5.370	2960	WS0055_B18
9	28.608	170	2	0.301	0.065	7.586	2960	WS0055_B18
1	45.794	9	1	0.372	0.068	6.938	2961	WS0055_F20
1	50.955	10	1	-0.264	0.049	4.923	2961	WS0055_F20
3	137.891	64	1	-0.306	0.039	3.944	2961	WS0055_F20
4	116.928	82	4	-0.272	0.052	5.234	2961	WS0055_F20
5	84.347	105	1	-0.280	0.055	5.490	2961	WS0055_F20
6	25.914	114	1	-0.186	0.044	4.389	2961	WS0055_F20
6	45.897	117	1	0.261	0.050	4.981	2961	WS0055_F20
7	62.787	139	4	-0.214	0.041	4.136	2961	WS0055_F20
11	40.960	210	2	-0.430	0.106	5.249	2961	WS0055_F20
11	40.960	210	4	0.377	0.086	8.575	2961	WS0055_F20
11	46.026	217	1	-0.316	0.069	6.940	2961	WS0055_F20
14	0.000	246	1	-0.275	0.053	5.294	2961	WS0055_F20
2	48.839	29	4	-0.288	0.067	7.162	2962	WS0055_H14
10	70.246	190	2	-0.204	0.036	4.334	2962	WS0055_H14
1	69.854	13	2	0.193	0.044	4.655	2963	WS0085_L11
1	71.592	14	2	0.181	0.039	4.121	2963	WS0085_L11
1	77.177	15	2	0.203	0.049	5.103	2963	WS0085_L11
3	116.958	63	4	-0.173	0.050	4.206	2963	WS0085_L11
6	26.350	115	2	0.193	0.037	3.946	2963	WS0085_L11
6	81.996	123	3	0.289	0.073	4.364	2963	WS0085_L11
6	87.891	124	2	-0.241	0.057	6.012	2963	WS0085_L11
6	89.401	125	2	-0.255	0.048	4.957	2963	WS0085_L11
7	15.555	130	3	0.273	0.069	7.085	2963	WS0085_L11
11	64.014	222	4	-0.265	0.057	4.804	2963	WS0085_L11
1	71.592	14	2	0.190	0.032	4.021	2964	WS0086_D04
1	77.177	15	2	0.203	0.037	4.555	2964	WS0086_D04
5	3.484	90	2	-0.322	0.087	6.679	2964	WS0086_D04
5	52.820	99	3	-0.273	0.079	5.938	2964	WS0086_D04
5	57.017	102	2	-0.225	0.032	4.000	2964	WS0086_D04
11	64.014	222	4	-0.269	0.057	6.178	2964	WS0086_D04
13	0.000	243	4	0.293	0.146	4.848	2964	WS0086_D04
1	95.572	17	3	0.246	0.055	5.872	2965	IS0014_I16
3	95.157	59	2	0.218	0.042	5.713	2965	IS0014_I16
3	116.958	63	4	-0.228	0.052	4.162	2965	IS0014_I16
5	52.820	99	3	-0.269	0.045	4.189	2965	IS0014_I16
6	69.073	121	4	-0.304	0.069	5.351	2965	IS0014_I16
9	49.013	173	2	0.227	0.037	5.188	2965	IS0014_I16
10	70.246	190	2	0.259	0.036	5.071	2965	IS0014_I16
10	78.479	193	3	-0.353	0.067	7.337	2965	IS0014_I16
10	86.151	195	3	0.175	0.037	3.967	2965	IS0014_I16
1	133.992	24	2	-0.157	0.039	4.280	2966	WS0041_A24
1	141.021	25	2	0.158	0.040	4.386	2966	WS0041_A24
2	48.839	29	4	0.255	0.040	3.989	2966	WS0041_A24
3	9.953	42	3	0.198	0.071	6.153	2966	WS0041_A24
6	81.996	123	3	0.276	0.108	5.255	2966	WS0041_A24
1	62.128	11	2	-0.292	0.051	4.134	2967	WS01039_K09
2	75.550	31	2	-0.327	0.068	9.052	2967	WS01039_K09
4	92.039	79	2	-0.218	0.033	4.435	2967	WS01039_K09

4	142.917	85	3	0.241	0.045	5.004	2967	WS01039_K09
5	56.008	101	2	-0.225	0.045	6.147	2967	WS01039_K09
5	57.017	102	2	0.228	0.037	4.818	2967	WS01039_K09
5	84.347	105	2	-0.232	0.049	6.455	2967	WS01039_K09
7	17.610	131	3	0.234	0.048	4.287	2967	WS01039_K09
7	62.787	139	4	-0.295	0.071	5.643	2967	WS01039_K09
7	74.560	143	2	-0.335	0.059	7.823	2967	WS01039_K09
9	0.000	163	1	0.443	0.158	4.288	2967	WS01039_K09
9	36.227	165	3	-0.189	0.038	4.484	2967	WS01039_K09
9	69.046	181	2	-0.245	0.052	4.704	2967	WS01039_K09
14	22.402	248	4	-0.360	0.091	6.289	2967	WS01039_K09
14	44.898	251	1	-0.233	0.081	4.484	2967	WS01039_K09
1	69.854	13	2	-0.230	0.044	5.265	2968	WS01039_L13
1	71.592	14	2	-0.229	0.045	5.302	2968	WS01039_L13
1	77.177	15	2	-0.214	0.037	4.453	2968	WS01039_L13
2	126.181	39	4	-0.366	0.060	4.558	2968	WS01039_L13
4	173.929	88	2	0.177	0.042	4.984	2968	WS01039_L13
6	45.897	117	1	0.261	0.071	5.030	2968	WS01039_L13
7	17.610	131	3	0.277	0.125	5.406	2968	WS01039_L13
9	49.013	173	2	-0.164	0.032	3.892	2968	WS01039_L13
11	64.014	222	3	-0.284	0.062	5.786	2968	WS01039_L13
6	56.188	120	2	-0.231	0.037	5.248	2969	WS01039_O09
9	36.227	165	3	-0.215	0.075	7.305	2969	WS01039_O09
10	78.479	193	3	0.385	0.089	8.667	2969	WS01039_O09
11	46.026	217	1	0.197	0.082	4.817	2969	WS01039_O09
1	123.316	20	4	0.213	0.073	5.056	2970	WS01039_P21
3	15.147	44	2	-0.223	0.076	5.289	2970	WS01039_P21
3	80.211	55	4	0.320	0.070	6.954	2970	WS01039_P21
3	95.157	59	2	-0.208	0.044	4.880	2970	WS01039_P21
4	173.929	88	2	0.276	0.074	8.558	2970	WS01039_P21
7	17.610	131	3	0.244	0.127	5.847	2970	WS01039_P21
7	75.883	144	2	-0.461	0.086	6.119	2970	WS01039_P21
9	0.000	163	1	0.485	0.118	5.180	2970	WS01039_P21
9	59.963	177	1	-0.275	0.080	5.877	2970	WS01039_P21
9	69.046	181	1	0.277	0.095	4.277	2970	WS01039_P21
11	27.405	204	4	-0.185	0.045	4.144	2970	WS01039_P21
11	57.661	220	4	-0.187	0.047	4.599	2970	WS01039_P21
11	64.014	222	4	0.342	0.064	5.667	2970	WS01039_P21
4	92.039	79	2	-0.270	0.058	7.300	2971	WS00932_D12
4	116.928	82	2	0.294	0.044	5.539	2971	WS00932_D12
4	124.132	83	2	0.347	0.100	4.150	2971	WS00932_D12
4	140.962	84	3	0.202	0.050	4.726	2971	WS00932_D12
4	142.917	85	3	0.253	0.044	4.061	2971	WS00932_D12
5	57.017	102	2	0.212	0.047	5.873	2971	WS00932_D12
7	70.278	141	3	0.262	0.057	5.381	2971	WS00932_D12
8	11.974	150	1	-0.177	0.062	3.872	2971	WS00932_D12
9	49.013	173	2	-0.160	0.031	4.022	2971	WS00932_D12
11	46.026	217	1	0.235	0.101	6.357	2971	WS00932_D12
14	9.923	247	1	-0.198	0.075	4.730	2971	WS00932_D12
3	137.891	64	3	-0.219	0.059	5.691	2972	WS00932_E18
4	116.928	82	2	0.330	0.047	5.875	2972	WS00932_E18
4	124.132	83	2	0.404	0.113	6.601	2972	WS00932_E18
6	45.897	117	4	-0.384	0.065	5.785	2972	WS00932_E18
6	49.100	118	2	0.223	0.071	4.301	2972	WS00932_E18

7	54.200	136	2	-0.344	0.075	4.627	2972	WS00932_E18
7	70.278	141	3	0.292	0.073	7.161	2972	WS00932_E18
7	91.487	146	3	0.173	0.044	4.222	2972	WS00932_E18
8	50.001	154	2	0.254	0.051	5.707	2972	WS00932_E18
9	3.127	168	1	0.274	0.123	3.874	2972	WS00932_E18
9	49.013	173	2	-0.252	0.064	7.417	2972	WS00932_E18
9	55.358	175	1	0.288	0.071	4.648	2972	WS00932_E18
9	55.358	175	3	-0.484	0.141	5.803	2972	WS00932_E18
10	9.580	187	2	0.256	0.115	7.129	2972	WS00932_E18
11	44.884	215	3	0.189	0.041	4.022	2972	WS00932_E18
11	48.979	218	2	-0.304	0.042	5.274	2972	WS00932_E18
12	22.441	227	3	-0.314	0.062	5.912	2972	WS00932_E18
12	28.493	228	2	0.186	0.077	4.771	2972	WS00932_E18
12	0.000	233	1	0.286	0.093	6.025	2972	WS00932_E18
14	46.637	252	4	-0.249	0.052	3.863	2972	WS00932_E18
1	29.800	5	2	0.313	0.086	6.284	2973	WS00932_I14
1	50.955	10	1	-0.275	0.047	4.969	2973	WS00932_I14
1	131.686	23	2	0.445	0.137	4.590	2973	WS00932_I14
3	112.868	61	2	-0.229	0.049	4.099	2973	WS00932_I14
4	142.917	85	1	-0.219	0.064	6.655	2973	WS00932_I14
5	0.000	89	1	0.389	0.069	7.614	2973	WS00932_I14
5	0.000	89	3	-0.313	0.092	8.116	2973	WS00932_I14
5	84.347	105	1	-0.251	0.042	4.476	2973	WS00932_I14
6	54.066	119	4	-0.266	0.049	5.229	2973	WS00932_I14
6	89.401	125	2	0.299	0.053	4.918	2973	WS00932_I14
7	15.555	130	3	-0.155	0.044	4.286	2973	WS00932_I14
7	17.610	131	3	-0.356	0.136	6.508	2973	WS00932_I14
7	91.487	146	3	-0.243	0.060	4.992	2973	WS00932_I14
9	55.358	175	1	0.338	0.048	5.173	2973	WS00932_I14
1	82.301	16	3	0.217	0.051	4.387	2974	WS00932_J18
5	42.979	97	2	-0.476	0.124	12.845	2974	WS00932_J18
5	56.008	101	2	-0.268	0.045	4.183	2974	WS00932_J18
5	84.347	105	1	0.202	0.054	4.026	2974	WS00932_J18
5	84.347	105	2	-0.177	0.040	4.491	2974	WS00932_J18
7	15.555	130	2	0.285	0.055	6.411	2974	WS00932_J18
7	70.278	141	3	0.248	0.051	4.459	2974	WS00932_J18
9	0.000	166	1	0.157	0.057	4.274	2974	WS00932_J18
9	17.951	169	2	0.299	0.047	5.074	2974	WS00932_J18
10	78.479	193	4	-0.250	0.052	5.440	2974	WS00932_J18
14	22.402	248	4	-0.293	0.039	4.085	2974	WS00932_J18
14	46.637	252	4	-0.280	0.038	4.216	2974	WS00932_J18
1	29.800	5	2	0.296	0.051	5.088	2975	WS0263_J16
1	38.326	6	2	-0.221	0.049	4.885	2975	WS0263_J16
3	17.158	45	1	0.238	0.047	4.152	2975	WS0263_J16
3	141.443	65	3	-0.278	0.079	4.030	2975	WS0263_J16
4	0.000	71	4	0.234	0.054	4.032	2975	WS0263_J16
4	75.958	77	3	0.303	0.066	4.132	2975	WS0263_J16
7	17.610	131	3	0.335	0.115	7.169	2975	WS0263_J16
10	70.246	190	2	-0.242	0.044	4.564	2975	WS0263_J16
1	45.794	9	1	0.333	0.058	5.786	2976	WS0263_K04
11	43.491	214	3	-0.217	0.051	3.941	2976	WS0263_K04
3	14.500	43	1	-0.311	0.053	4.138	2977	WS0263_M02
3	17.158	45	1	0.190	0.053	4.098	2977	WS0263_M02
6	69.073	121	3	0.295	0.083	6.485	2977	WS0263_M02

11	27.405	204	1	-0.329	0.054	4.308	2977	WS0263_M02
1	62.128	11	1	-0.676	0.155	8.849	2978	WS0263_M18
1	131.686	23	1	-0.460	0.069	3.899	2978	WS0263_M18
3	47.928	49	1	-0.304	0.050	5.321	2978	WS0263_M18
3	80.211	55	4	-0.189	0.045	4.860	2978	WS0263_M18
6	89.401	125	1	0.249	0.048	4.976	2978	WS0263_M18
7	15.555	130	2	0.332	0.089	7.789	2978	WS0263_M18
11	0.000	199	3	0.354	0.060	4.426	2978	WS0263_M18
11	17.088	203	1	0.302	0.056	5.918	2978	WS0263_M18
11	27.405	204	1	-0.378	0.062	6.661	2978	WS0263_M18
11	27.405	204	4	0.346	0.069	7.875	2978	WS0263_M18
11	29.069	205	1	-0.267	0.043	4.500	2978	WS0263_M18
11	40.960	210	2	-0.387	0.078	4.273	2978	WS0263_M18
11	40.960	210	4	0.370	0.091	10.040	2978	WS0263_M18
11	60.464	221	1	-0.295	0.042	4.421	2978	WS0263_M18
3	15.147	44	2	0.198	0.065	4.055	2979	WS0263_O06
3	137.891	64	1	-0.268	0.043	4.489	2979	WS0263_O06
4	55.925	76	1	0.197	0.052	6.238	2979	WS0263_O06
4	116.928	82	4	-0.265	0.036	4.189	2979	WS0263_O06
7	75.883	144	2	0.403	0.074	4.534	2979	WS0263_O06
7	75.883	144	4	-0.262	0.044	5.124	2979	WS0263_O06
9	0.000	163	1	-0.508	0.129	5.677	2979	WS0263_O06
9	49.013	173	2	0.165	0.044	3.912	2979	WS0263_O06
11	45.989	216	2	0.171	0.051	4.514	2979	WS0263_O06
11	46.026	217	1	-0.280	0.088	8.913	2979	WS0263_O06
2	126.181	39	2	-0.275	0.055	4.355	2980	WS0263_O21
4	116.928	82	4	-0.234	0.059	5.870	2980	WS0263_O21
4	142.917	85	1	-0.228	0.058	6.343	2980	WS0263_O21
7	75.883	144	2	0.403	0.109	4.399	2980	WS0263_O21
9	3.127	168	1	-0.410	0.048	4.882	2980	WS0263_O21
9	28.608	170	3	0.283	0.059	4.769	2980	WS0263_O21
10	78.479	193	4	-0.272	0.050	4.370	2980	WS0263_O21
10	93.347	196	1	0.435	0.112	4.540	2980	WS0263_O21
10	95.795	197	4	0.143	0.039	4.211	2980	WS0263_O21
14	22.402	248	4	0.349	0.042	4.620	2980	WS0263_O21
14	46.637	252	4	0.353	0.057	6.448	2980	WS0263_O21
3	116.958	63	4	-0.224	0.040	4.729	2981	WS0264_B09
4	173.929	88	2	-0.470	0.167	21.755	2981	WS0264_B09
6	69.073	121	3	0.206	0.069	4.815	2981	WS0264_B09
9	0.000	166	2	0.299	0.065	8.364	2981	WS0264_B09
9	17.951	169	2	-0.284	0.047	6.252	2981	WS0264_B09
9	49.013	173	2	0.254	0.037	4.855	2981	WS0264_B09
14	0.000	246	2	-0.153	0.034	4.559	2981	WS0264_B09
1	107.333	19	4	0.165	0.043	4.677	2982	WS0264_B20
2	48.839	29	4	0.297	0.059	6.051	2982	WS0264_B20
2	126.181	39	4	0.326	0.048	3.913	2982	WS0264_B20
3	116.958	63	4	-0.292	0.064	6.699	2982	WS0264_B20
3	137.891	64	3	0.176	0.057	4.557	2982	WS0264_B20
5	54.072	100	4	0.248	0.043	4.541	2982	WS0264_B20
8	95.937	159	3	-0.170	0.060	5.005	2982	WS0264_B20
9	0.000	166	2	0.225	0.032	3.965	2982	WS0264_B20
9	17.951	169	2	-0.254	0.040	4.586	2982	WS0264_B20
11	45.989	216	1	-0.341	0.124	7.427	2982	WS0264_B20
14	0.000	246	1	0.207	0.085	5.107	2982	WS0264_B20

6	87.891	124	2	-0.271	0.077	9.571	2983	WS00818_P16
6	89.401	125	2	-0.342	0.058	6.349	2983	WS00818_P16
9	81.073	183	1	0.216	0.059	3.886	2983	WS00818_P16
10	93.347	196	1	0.429	0.097	4.426	2983	WS00818_P16
11	64.014	222	4	-0.223	0.047	4.096	2983	WS00818_P16
2	95.880	34	3	0.205	0.051	5.156	2984	WS01020_A22
5	52.820	99	1	0.261	0.105	5.908	2984	WS01020_A22
5	52.820	99	3	-0.301	0.042	4.223	2984	WS01020_A22
9	36.227	165	3	0.241	0.065	6.419	2984	WS01020_A22
9	49.013	173	2	0.296	0.077	10.154	2984	WS01020_A22
10	9.580	187	2	-0.166	0.058	3.890	2984	WS01020_A22
11	43.491	214	4	-0.180	0.054	4.002	2984	WS01020_A22
3	80.211	55	4	0.182	0.045	4.417	2985	WS01020_D21
4	0.000	71	4	-0.238	0.055	5.417	2985	WS01020_D21
4	142.917	85	1	-0.241	0.050	4.466	2985	WS01020_D21
5	52.820	99	1	0.276	0.044	3.992	2985	WS01020_D21
6	56.188	120	2	-0.220	0.045	4.586	2985	WS01020_D21
6	81.996	123	4	0.344	0.076	4.361	2985	WS01020_D21
7	75.883	144	2	0.405	0.090	4.233	2985	WS01020_D21
9	55.358	175	1	0.306	0.067	6.024	2985	WS01020_D21
11	17.088	203	1	0.224	0.059	5.245	2985	WS01020_D21
14	0.000	246	1	-0.191	0.043	3.845	2985	WS01020_D21
2	120.617	38	3	-0.206	0.055	4.616	2986	WS01020_E16
4	173.929	88	2	-0.215	0.041	4.196	2986	WS01020_E16
5	52.820	99	3	-0.278	0.062	4.360	2986	WS01020_E16
5	54.072	100	4	0.167	0.043	4.380	2986	WS01020_E16
12	6.242	234	2	-0.222	0.043	5.982	2986	WS01020_E16
1	2.788	2	4	-0.259	0.042	5.477	2987	WS01020_G24
1	131.686	23	1	0.509	0.082	4.491	2987	WS01020_G24
2	48.839	29	4	0.889	0.417	62.606	2987	WS01020_G24
2	75.550	31	2	-0.376	0.082	9.634	2987	WS01020_G24
2	93.504	32	4	0.344	0.060	9.074	2987	WS01020_G24
2	101.076	35	4	-0.302	0.054	7.247	2987	WS01020_G24
3	148.745	68	4	-0.205	0.027	4.283	2987	WS01020_G24
4	116.928	82	4	0.298	0.037	5.645	2987	WS01020_G24
5	7.672	92	4	0.212	0.035	5.755	2987	WS01020_G24
6	45.897	117	4	-0.210	0.027	4.362	2987	WS01020_G24
6	54.066	119	4	-0.185	0.028	4.259	2987	WS01020_G24
7	62.787	139	4	0.350	0.062	9.371	2987	WS01020_G24
10	21.864	188	1	-0.317	0.077	5.468	2987	WS01020_G24
10	78.479	193	4	0.267	0.058	8.205	2987	WS01020_G24
10	95.795	197	4	-0.330	0.091	13.563	2987	WS01020_G24
10	104.214	198	4	0.298	0.035	6.597	2987	WS01020_G24
12	14.406	230	4	0.254	0.035	5.239	2987	WS01020_G24
12	38.543	232	4	-0.314	0.095	5.164	2987	WS01020_G24
14	43.392	250	2	-0.237	0.063	6.842	2987	WS01020_G24
14	46.637	252	4	-0.322	0.049	7.408	2987	WS01020_G24
1	45.794	9	1	0.166	0.041	4.186	2988	WS01020_H12
1	62.128	11	1	-0.500	0.114	4.793	2988	WS01020_H12
1	71.592	14	2	0.171	0.050	4.291	2988	WS01020_H12
1	77.177	15	2	0.234	0.088	7.548	2988	WS01020_H12
4	92.039	79	2	-0.214	0.060	5.377	2988	WS01020_H12
4	142.917	85	1	0.178	0.047	4.788	2988	WS01020_H12
5	31.191	95	1	0.240	0.048	4.891	2988	WS01020_H12

6	69.073	121	4	0.243	0.054	4.037	2988	WS01020_H12
7	70.278	141	3	0.285	0.053	4.837	2988	WS01020_H12
8	78.389	157	1	0.239	0.044	4.516	2988	WS01020_H12
8	87.463	158	1	-0.228	0.040	4.121	2988	WS01020_H12
9	0.000	163	1	0.773	0.226	13.535	2988	WS01020_H12
10	9.580	187	3	0.420	0.084	4.140	2988	WS01020_H12
10	80.088	194	1	0.197	0.058	5.938	2988	WS01020_H12
10	93.347	196	1	-0.487	0.095	5.707	2988	WS01020_H12
11	17.088	203	1	0.322	0.078	7.920	2988	WS01020_H12
11	29.069	205	1	-0.294	0.041	4.480	2988	WS01020_H12
11	45.989	216	1	0.210	0.052	5.307	2988	WS01020_H12
12	6.242	234	2	-0.223	0.045	4.660	2988	WS01020_H12
14	46.637	252	4	-0.396	0.057	5.077	2988	WS01020_H12
1	43.578	8	2	-0.181	0.045	4.472	2989	WS01020_K09
1	62.128	11	1	-0.462	0.115	4.072	2989	WS01020_K09
1	77.177	15	2	0.222	0.049	4.792	2989	WS01020_K09
2	48.839	29	1	-0.295	0.071	6.470	2989	WS01020_K09
2	101.076	35	4	-0.306	0.064	3.925	2989	WS01020_K09
4	82.462	78	2	-0.195	0.039	4.129	2989	WS01020_K09
5	54.072	100	4	0.182	0.043	4.001	2989	WS01020_K09
6	106.702	128	3	0.339	0.084	6.981	2989	WS01020_K09
10	86.151	195	3	0.234	0.053	4.969	2989	WS01020_K09
11	17.088	203	1	0.179	0.043	3.885	2989	WS01020_K09
11	42.600	213	3	0.204	0.048	4.560	2989	WS01020_K09
11	64.014	222	3	0.244	0.052	4.878	2989	WS01020_K09
12	0.000	226	3	-0.209	0.057	5.396	2989	WS01020_K09
3	47.928	49	1	0.192	0.045	4.216	2990	WS01020_K19
3	95.157	59	2	-0.186	0.052	4.932	2990	WS01020_K19
3	104.681	60	2	0.218	0.045	4.242	2990	WS01020_K19
3	112.868	61	2	-0.286	0.091	8.586	2990	WS01020_K19
3	114.747	62	2	-0.246	0.069	6.529	2990	WS01020_K19
3	116.958	63	4	-0.244	0.044	5.150	2990	WS01020_K19
4	124.132	83	1	-0.437	0.068	4.160	2990	WS01020_K19
5	56.008	101	2	-0.298	0.094	7.862	2990	WS01020_K19
5	57.017	102	2	0.285	0.078	7.394	2990	WS01020_K19
8	39.090	152	4	-0.218	0.047	5.484	2990	WS01020_K19
9	17.951	169	1	-0.232	0.044	4.064	2990	WS01020_K19
10	9.580	187	2	0.274	0.080	4.456	2990	WS01020_K19
10	35.204	189	1	0.234	0.045	4.224	2990	WS01020_K19
11	45.989	216	1	0.212	0.056	5.213	2990	WS01020_K19
14	46.637	252	4	-0.330	0.065	7.652	2990	WS01020_K19
4	142.917	85	1	-0.220	0.052	4.458	2991	WS0041_I18
5	52.820	99	3	-0.313	0.055	5.759	2991	WS0041_I18
5	86.669	106	2	-0.236	0.102	4.226	2991	WS0041_I18
6	81.996	123	3	0.283	0.110	6.713	2991	WS0041_I18
10	80.088	194	1	-0.204	0.045	3.986	2991	WS0041_I18
10	93.347	196	1	0.526	0.107	6.636	2991	WS0041_I18
1	107.333	19	3	0.236	0.052	5.785	2992	WS0042_B02
4	142.917	85	1	-0.247	0.044	4.290	2992	WS0042_B02
10	93.347	196	2	0.433	0.086	3.945	2992	WS0042_B02
12	22.629	237	1	0.216	0.045	4.366	2992	WS0042_B02
1	131.686	23	1	-0.407	0.058	3.934	2993	WS00816_A11
3	14.500	43	1	-0.227	0.048	4.941	2993	WS00816_A11
3	17.158	45	1	0.185	0.047	4.908	2993	WS00816_A11

5	86.669	106	4	0.338	0.119	7.899	2993	WS00816_A11
9	59.963	177	4	0.284	0.039	3.922	2993	WS00816_A11
11	0.000	199	3	0.273	0.059	4.457	2993	WS00816_A11
12	6.242	234	4	-0.394	0.067	7.590	2993	WS00816_A11
13	0.000	243	4	-0.321	0.105	7.178	2993	WS00816_A11
3	154.505	69	1	0.195	0.053	5.082	2994	WS00816_G11
4	82.462	78	1	-0.218	0.053	4.953	2994	WS00816_G11
5	54.072	100	4	0.210	0.050	4.507	2994	WS00816_G11
5	57.017	102	4	-0.287	0.059	5.515	2994	WS00816_G11
9	59.963	177	4	0.256	0.046	3.868	2994	WS00816_G11
12	22.441	227	3	-0.219	0.044	5.238	2995	WS0262_K05
1	38.326	6	4	-0.318	0.038	4.187	2996	WS0262_O05
1	40.464	7	4	0.334	0.047	5.149	2996	WS0262_O05
4	55.925	76	1	0.198	0.039	4.134	2996	WS0262_O05
4	116.928	82	4	-0.268	0.057	6.611	2996	WS0262_O05
7	62.787	139	4	-0.175	0.030	4.005	2996	WS0262_O05
9	0.000	163	1	-0.448	0.114	4.417	2996	WS0262_O05
9	32.356	171	2	0.186	0.037	3.871	2996	WS0262_O05
10	9.580	187	3	-0.386	0.147	5.910	2996	WS0262_O05
10	93.347	196	1	0.560	0.194	7.533	2996	WS0262_O05
1	62.128	11	2	0.358	0.100	6.015	2997	WS0038_D18
1	141.021	25	2	0.238	0.040	5.153	2997	WS0038_D18
3	116.958	63	4	-0.313	0.089	7.689	2997	WS0038_D18
4	75.958	77	3	-0.269	0.070	4.562	2997	WS0038_D18
5	0.000	89	3	0.362	0.082	4.870	2997	WS0038_D18
5	31.191	95	1	-0.229	0.090	5.504	2997	WS0038_D18
6	25.914	114	1	-0.250	0.096	5.878	2997	WS0038_D18
7	15.555	130	2	0.248	0.034	4.429	2997	WS0038_D18
10	78.479	193	3	-0.439	0.108	10.953	2997	WS0038_D18
10	86.151	195	3	0.245	0.053	5.380	2997	WS0038_D18
10	93.347	196	2	0.466	0.076	4.775	2997	WS0038_D18
1	45.794	9	1	-0.273	0.053	4.613	2998	WS0038_M12
1	62.128	11	2	0.244	0.051	4.032	2998	WS0038_M12
4	142.917	85	1	-0.271	0.060	5.275	2998	WS0038_M12
2	63.261	30	3	0.184	0.034	3.950	2999	WS0046_A04
5	52.820	99	3	-0.235	0.037	4.101	2999	WS0046_A04
5	57.017	102	2	-0.236	0.046	6.433	2999	WS0046_A04
7	17.610	131	3	-0.224	0.044	3.964	2999	WS0046_A04
9	69.046	181	2	0.219	0.045	4.151	2999	WS0046_A04
11	27.405	204	4	-0.315	0.086	4.552	2999	WS0046_A04
14	44.898	251	3	-0.204	0.035	4.119	2999	WS0046_A04
3	9.953	42	3	0.222	0.073	7.639	3000	WS0046_B21
5	7.672	92	4	0.179	0.047	4.149	3000	WS0046_B21
5	52.820	99	3	-0.305	0.073	5.241	3000	WS0046_B21
9	49.013	173	2	0.178	0.040	5.586	3000	WS0046_B21
9	69.046	181	2	0.270	0.055	4.532	3000	WS0046_B21
10	21.864	188	3	0.212	0.045	4.975	3000	WS0046_B21
1	69.854	13	2	0.227	0.056	5.361	3001	WS0046_G10
1	71.592	14	2	0.220	0.053	5.078	3001	WS0046_G10
1	77.177	15	2	0.262	0.076	7.221	3001	WS0046_G10
4	75.958	77	3	0.273	0.130	4.213	3001	WS0046_G10
4	173.929	88	2	-0.171	0.041	3.901	3001	WS0046_G10
5	56.008	101	1	0.194	0.064	5.912	3001	WS0046_G10
6	89.401	125	1	0.328	0.056	5.265	3001	WS0046_G10

6	95.249	126	1	-0.313	0.076	4.436	3001	WS0046_G10
10	78.479	193	3	0.272	0.047	4.286	3001	WS0046_G10
10	78.479	193	3	0.317	0.048	3.929	3002	WS0046_H09
1	69.854	13	2	0.165	0.035	4.220	3003	WS0046_K07
1	77.177	15	2	0.185	0.043	5.164	3003	WS0046_K07
1	95.572	17	2	0.259	0.048	5.803	3003	WS0046_K07
3	15.147	44	2	0.309	0.062	4.724	3003	WS0046_K07
6	81.996	123	3	0.318	0.114	4.160	3003	WS0046_K07
11	27.405	204	4	-0.244	0.036	3.980	3003	WS0046_K07
1	69.854	13	2	0.233	0.054	5.883	3004	WS0046_M05
1	71.592	14	2	0.227	0.052	5.610	3004	WS0046_M05
1	77.177	15	2	0.242	0.058	6.298	3004	WS0046_M05
1	95.572	17	2	0.279	0.051	5.555	3004	WS0046_M05
14	0.000	246	2	-0.208	0.039	4.267	3004	WS0046_M05
1	2.788	2	4	-0.263	0.061	4.692	3005	WS0046_P19
2	120.617	38	3	-0.256	0.047	4.575	3005	WS0046_P19
4	75.958	77	3	0.265	0.076	4.526	3005	WS0046_P19
4	82.462	78	2	0.235	0.053	5.978	3005	WS0046_P19
4	97.576	80	2	-0.236	0.049	6.553	3005	WS0046_P19
6	56.188	120	2	-0.273	0.051	6.149	3005	WS0046_P19
7	54.200	136	2	-0.277	0.074	4.423	3005	WS0046_P19
8	62.580	155	1	-0.339	0.184	4.274	3005	WS0046_P19
9	17.951	169	2	-0.299	0.055	4.986	3005	WS0046_P19
9	59.963	177	4	0.271	0.054	4.867	3005	WS0046_P19
10	78.479	193	3	0.266	0.046	4.440	3005	WS0046_P19
1	62.128	11	1	-0.656	0.200	8.195	3006	WS0047_A14
4	173.929	88	2	-0.203	0.037	3.924	3006	WS0047_A14
6	89.401	125	2	0.231	0.038	3.864	3006	WS0047_A14
10	78.479	193	3	0.306	0.062	4.539	3006	WS0047_A14
11	27.405	204	4	-0.217	0.046	5.458	3006	WS0047_A14
12	6.242	234	2	-0.225	0.032	4.264	3006	WS0047_A14
13	1.815	244	4	0.244	0.045	4.602	3006	WS0047_A14
2	120.617	38	3	-0.254	0.042	4.136	3007	WS0071_C09
2	127.147	40	3	0.309	0.082	8.086	3007	WS0071_C09
4	55.925	76	1	0.211	0.046	4.092	3007	WS0071_C09
5	52.820	99	3	-0.333	0.076	7.400	3007	WS0071_C09
7	62.787	139	4	-0.212	0.031	4.000	3007	WS0071_C09
10	35.204	189	1	-0.326	0.072	5.612	3007	WS0071_C09
1	45.794	9	1	0.291	0.033	3.872	3008	WS0071_C19
1	95.572	17	3	0.251	0.057	4.519	3008	WS0071_C19
3	148.745	68	1	-0.249	0.046	5.543	3008	WS0071_C19
6	25.914	114	1	-0.310	0.106	12.091	3008	WS0071_C19
8	78.389	157	1	0.252	0.036	4.161	3008	WS0071_C19
10	93.347	196	1	0.495	0.124	5.883	3008	WS0071_C19
11	46.026	217	1	-0.371	0.081	9.403	3008	WS0071_C19
2	127.147	40	4	-0.111	0.024	4.286	3009	WS0071_E18
3	141.443	65	4	0.279	0.059	4.046	3009	WS0071_E18
3	144.647	67	1	0.400	0.100	6.087	3009	WS0071_E18
3	148.745	68	4	-0.147	0.036	5.143	3009	WS0071_E18
4	103.719	81	4	-0.313	0.051	7.332	3009	WS0071_E18
5	31.191	95	1	0.288	0.056	5.156	3009	WS0071_E18
5	42.979	97	1	-0.488	0.164	14.839	3009	WS0071_E18
5	52.820	99	1	-0.457	0.153	13.895	3009	WS0071_E18
5	56.008	101	1	0.190	0.062	5.669	3009	WS0071_E18

6	49.100	118	4	0.558	0.192	12.003	3009	WS0071_E18
6	81.996	123	4	-0.375	0.111	6.997	3009	WS0071_E18
7	15.555	130	2	0.309	0.049	5.148	3009	WS0071_E18
8	39.090	152	4	0.257	0.029	4.143	3009	WS0071_E18
8	87.463	158	1	-0.318	0.071	6.522	3009	WS0071_E18
12	22.629	237	1	0.305	0.058	5.319	3009	WS0071_E18
13	1.815	244	4	0.277	0.048	7.106	3009	WS0071_E18
1	62.128	11	1	-0.573	0.154	6.246	3010	WS0071_F17
4	0.000	71	4	-0.244	0.051	4.812	3010	WS0071_F17
6	69.073	121	3	0.321	0.074	6.974	3010	WS0071_F17
6	87.891	124	2	0.194	0.049	4.995	3010	WS0071_F17
6	89.401	125	2	0.258	0.046	4.657	3010	WS0071_F17
1	95.572	17	2	0.244	0.050	4.878	3011	WS0071_I01
2	63.261	30	3	0.316	0.107	11.949	3011	WS0071_I01
2	75.550	31	1	0.223	0.065	5.794	3011	WS0071_I01
2	95.880	34	3	0.320	0.059	6.735	3011	WS0071_I01
2	101.076	35	3	-0.300	0.057	6.386	3011	WS0071_I01
3	15.147	44	2	0.283	0.087	4.106	3011	WS0071_I01
5	52.820	99	3	-0.259	0.046	5.026	3011	WS0071_I01
6	81.996	123	3	0.313	0.069	4.198	3011	WS0071_I01
7	105.477	147	2	-0.348	0.130	6.198	3011	WS0071_I01
10	78.479	193	3	0.324	0.050	5.540	3011	WS0071_I01
1	2.788	2	4	-0.229	0.052	4.171	3012	WS0071_J10
3	14.500	43	1	-0.273	0.042	4.113	3012	WS0071_J10
3	17.158	45	1	0.220	0.054	5.196	3012	WS0071_J10
10	93.347	196	1	0.417	0.065	4.183	3012	WS0071_J10
1	15.589	3	2	-0.226	0.057	4.691	3013	WS0071_L22
1	29.800	5	1	-0.301	0.047	4.996	3013	WS0071_L22
3	148.745	68	1	-0.219	0.036	3.882	3013	WS0071_L22
4	92.039	79	4	-0.408	0.089	9.632	3013	WS0071_L22
4	103.719	81	4	-0.290	0.066	8.696	3013	WS0071_L22
5	0.000	89	1	-0.217	0.040	4.297	3013	WS0071_L22
5	7.672	92	4	-0.187	0.045	4.778	3013	WS0071_L22
7	67.154	140	4	-0.316	0.111	4.808	3013	WS0071_L22
9	59.963	177	4	0.287	0.043	4.634	3013	WS0071_L22
10	77.676	192	1	-0.221	0.042	4.417	3013	WS0071_L22
10	80.088	194	1	-0.379	0.087	9.249	3013	WS0071_L22
10	93.347	196	1	0.544	0.114	7.139	3013	WS0071_L22
11	64.014	222	3	0.234	0.062	4.858	3013	WS0071_L22
6	25.914	114	1	-0.227	0.059	4.948	3014	WS0071_M18
10	93.347	196	1	0.466	0.103	5.212	3014	WS0071_M18
11	64.014	222	4	-0.316	0.059	5.389	3014	WS0071_M18
12	6.242	234	2	-0.149	0.030	4.308	3014	WS0071_M18
1	40.464	7	4	0.401	0.074	7.027	3015	WS0071_O20
1	45.794	9	1	0.309	0.043	4.678	3015	WS0071_O20
1	50.955	10	1	-0.295	0.050	5.371	3015	WS0071_O20
1	141.021	25	2	-0.157	0.053	4.411	3015	WS0071_O20
3	154.505	69	1	0.196	0.039	4.208	3015	WS0071_O20
4	55.925	76	1	0.260	0.032	4.935	3015	WS0071_O20
5	52.820	99	3	-0.324	0.069	6.301	3015	WS0071_O20
6	45.897	117	1	0.281	0.047	4.939	3015	WS0071_O20
9	3.127	168	1	-0.465	0.081	7.197	3015	WS0071_O20
10	9.580	187	3	-0.308	0.114	5.827	3015	WS0071_O20
1	45.794	9	1	0.359	0.056	5.216	3016	WS0071_P12

1	45.794	9	3	-0.211	0.046	4.485	3016	WS0071_P12
4	75.958	77	3	0.370	0.136	7.165	3016	WS0071_P12
5	86.669	106	4	-0.407	0.091	4.099	3016	WS0071_P12
6	45.897	117	1	0.236	0.057	5.356	3016	WS0071_P12
6	69.073	121	4	0.271	0.084	6.894	3016	WS0071_P12
7	75.883	144	4	-0.406	0.086	7.128	3016	WS0071_P12
9	3.127	168	1	-0.426	0.081	5.718	3016	WS0071_P12
3	9.953	42	3	0.300	0.093	8.047	3017	WS0072_C15
4	124.132	83	1	-0.478	0.120	4.564	3017	WS0072_C15
12	32.473	238	1	-0.244	0.046	4.065	3017	WS0072_C15
12	51.382	242	1	0.447	0.149	5.711	3017	WS0072_C15
3	17.158	45	1	-0.264	0.058	5.498	3018	WS0072_D02
3	144.647	67	1	0.308	0.081	4.340	3018	WS0072_D02
4	92.039	79	4	0.385	0.089	9.578	3018	WS0072_D02
4	103.719	81	4	0.246	0.076	8.062	3018	WS0072_D02
9	55.358	175	1	-0.286	0.064	5.938	3018	WS0072_D02
9	55.358	175	3	0.505	0.146	6.630	3018	WS0072_D02
9	61.113	178	1	0.319	0.085	4.576	3018	WS0072_D02
11	64.014	222	3	-0.255	0.053	4.337	3018	WS0072_D02
12	32.473	238	1	0.248	0.052	4.954	3018	WS0072_D02
13	3.386	245	1	-0.272	0.057	5.443	3018	WS0072_D02
14	22.402	248	4	0.310	0.050	4.353	3018	WS0072_D02
1	82.301	16	3	-0.280	0.062	7.070	3019	WS0072_F05
4	82.462	78	1	-0.102	0.060	4.453	3019	WS0072_F05
6	89.401	125	1	0.282	0.061	4.728	3019	WS0072_F05
6	95.249	126	1	-0.476	0.063	4.258	3019	WS0072_F05
7	17.610	131	3	-0.269	0.073	3.890	3019	WS0072_F05
9	0.000	163	1	0.468	0.084	4.809	3019	WS0072_F05
9	17.951	169	2	-0.115	0.036	4.317	3019	WS0072_F05
9	28.608	170	2	0.407	0.101	12.801	3019	WS0072_F05
9	28.608	170	3	-0.345	0.035	4.502	3019	WS0072_F05
9	32.356	171	2	0.304	0.055	6.477	3019	WS0072_F05
11	41.984	212	2	-0.281	0.133	15.140	3019	WS0072_F05
11	45.989	216	2	-0.296	0.139	16.067	3019	WS0072_F05
11	56.921	219	2	0.404	0.158	8.549	3019	WS0072_F05
1	77.177	15	2	0.236	0.039	5.062	3020	WS0072_G14
1	95.572	17	2	0.305	0.046	5.982	3020	WS0072_G14
3	9.953	42	3	0.174	0.037	3.882	3020	WS0072_G14
5	52.820	99	3	-0.264	0.051	5.265	3020	WS0072_G14
5	56.008	101	2	0.253	0.041	4.738	3020	WS0072_G14
5	57.017	102	2	-0.291	0.065	8.428	3020	WS0072_G14
7	15.555	130	2	-0.241	0.030	3.935	3020	WS0072_G14
7	17.610	131	3	-0.267	0.064	4.269	3020	WS0072_G14
2	120.617	38	4	-0.350	0.075	6.596	3021	WS0072_J07
2	127.147	40	4	-0.224	0.064	5.603	3021	WS0072_J07
3	116.958	63	4	-0.232	0.048	4.270	3021	WS0072_J07
13	0.000	243	4	0.474	0.211	7.315	3021	WS0072_J07
3	9.953	42	3	0.210	0.042	3.928	3022	WS0072_K03
4	0.000	71	4	0.274	0.071	6.719	3022	WS0072_K03
5	52.820	99	3	-0.221	0.048	3.910	3022	WS0072_K03
5	57.017	102	2	-0.269	0.048	5.457	3022	WS0072_K03
7	17.610	131	3	-0.331	0.092	4.980	3022	WS0072_K03
8	50.001	154	1	-0.313	0.110	8.637	3022	WS0072_K03
1	45.794	9	1	0.315	0.062	5.123	3023	WS00931_F03

11	27.405	204	4	-0.212	0.046	4.994	3023	WS00931_F03
1	22.419	4	4	0.414	0.140	4.677	3024	WS00931_I06
1	40.464	7	3	-0.264	0.043	4.127	3024	WS00931_I06
2	48.839	29	1	-0.310	0.067	5.362	3024	WS00931_I06
2	75.550	31	1	0.178	0.051	4.013	3024	WS00931_I06
3	95.157	59	2	0.156	0.040	4.120	3024	WS00931_I06
6	87.891	124	2	0.302	0.071	7.755	3024	WS00931_I06
6	89.401	125	2	0.294	0.074	7.772	3024	WS00931_I06
7	15.555	130	3	-0.254	0.060	5.486	3024	WS00931_I06
7	59.789	137	3	-0.307	0.088	8.251	3024	WS00931_I06
7	70.278	141	3	0.215	0.043	4.060	3024	WS00931_I06
10	78.479	193	3	0.497	0.101	9.628	3024	WS00931_I06
10	86.151	195	3	-0.297	0.118	11.015	3024	WS00931_I06
11	40.960	210	2	0.459	0.090	5.836	3024	WS00931_I06
11	40.960	210	4	-0.327	0.059	5.691	3024	WS00931_I06
14	22.402	248	3	-0.293	0.048	4.545	3024	WS00931_I06
14	43.392	250	1	0.302	0.097	4.412	3024	WS00931_I06
1	95.572	17	2	0.207	0.040	4.345	3025	WS01025_K01
3	15.147	44	2	0.319	0.094	4.349	3025	WS01025_K01
5	57.017	102	2	-0.208	0.035	3.842	3025	WS01025_K01
13	0.000	243	4	0.352	0.147	4.379	3025	WS01025_K01
6	81.996	123	3	0.361	0.087	5.257	3026	WS01025_P05
8	50.001	154	1	-0.248	0.046	4.436	3026	WS01025_P05
8	95.937	159	3	-0.249	0.046	4.776	3026	WS01025_P05
9	59.963	177	4	0.372	0.106	7.926	3026	WS01025_P05
12	6.242	234	2	-0.202	0.034	3.866	3026	WS01025_P05
3	15.147	44	2	0.321	0.064	4.161	3027	WS01035_F18
4	0.000	71	4	0.190	0.049	4.374	3027	WS01035_F18
4	75.958	77	3	0.263	0.100	4.862	3027	WS01035_F18
4	173.929	88	2	-0.229	0.035	4.196	3027	WS01035_F18
8	50.001	154	1	-0.271	0.054	3.981	3027	WS01035_F18
9	59.963	177	4	0.319	0.076	6.385	3027	WS01035_F18
1	2.788	2	4	-0.240	0.042	4.133	3028	WS01035_G15
4	75.958	77	3	0.290	0.088	4.923	3028	WS01035_G15
9	59.963	177	4	0.301	0.054	4.992	3028	WS01035_G15
10	78.479	193	3	0.301	0.058	5.214	3028	WS01035_G15
3	15.147	44	2	0.313	0.080	6.507	3029	WS01035_K12
3	20.512	46	2	0.175	0.043	5.202	3029	WS01035_K12
3	116.958	63	4	0.371	0.096	10.233	3029	WS01035_K12
4	75.958	77	3	0.377	0.109	5.216	3029	WS01035_K12
4	97.576	80	2	0.244	0.038	5.956	3029	WS01035_K12
4	116.928	82	2	0.266	0.033	4.177	3029	WS01035_K12
4	124.132	83	1	-0.451	0.133	4.249	3029	WS01035_K12
5	57.017	102	2	-0.267	0.034	4.255	3029	WS01035_K12
6	25.914	114	1	0.163	0.060	3.947	3029	WS01035_K12
7	15.555	130	3	0.310	0.086	6.991	3029	WS01035_K12
7	74.560	143	2	-0.272	0.032	3.881	3029	WS01035_K12
8	78.389	157	1	0.190	0.080	5.266	3029	WS01035_K12
10	80.088	194	1	0.271	0.066	4.368	3029	WS01035_K12
11	0.000	199	2	0.247	0.033	4.002	3029	WS01035_K12
1	82.301	16	4	-0.270	0.061	5.215	3030	WS01035_L12
2	48.839	29	4	-0.258	0.048	5.046	3030	WS01035_L12
3	9.953	42	3	-0.237	0.059	4.870	3030	WS01035_L12
3	116.958	63	4	0.248	0.049	5.090	3030	WS01035_L12

3	144.593	66	4	-0.277	0.066	8.001	3030	WS01035_L12
3	148.745	68	4	0.416	0.126	12.160	3030	WS01035_L12
3	154.505	69	4	-0.346	0.081	8.556	3030	WS01035_L12
6	106.702	128	2	-0.399	0.075	4.005	3030	WS01035_L12
9	0.000	166	2	-0.238	0.047	6.050	3030	WS01035_L12
9	28.608	170	2	0.210	0.030	4.103	3030	WS01035_L12
9	28.608	170	3	-0.210	0.049	4.171	3030	WS01035_L12
9	49.013	173	2	-0.259	0.045	4.776	3030	WS01035_L12
10	78.479	193	3	0.295	0.053	4.380	3030	WS01035_L12
3	144.593	66	4	-0.319	0.066	7.649	3031	WS01035_O20
3	148.745	68	4	0.430	0.106	11.506	3031	WS01035_O20
3	154.505	69	4	-0.314	0.049	5.516	3031	WS01035_O20
5	54.072	100	4	0.185	0.046	5.187	3031	WS01035_O20
6	26.350	115	2	-0.176	0.028	3.966	3031	WS01035_O20
7	59.789	137	3	-0.239	0.059	4.452	3031	WS01035_O20
7	70.278	141	3	0.242	0.067	4.967	3031	WS01035_O20
9	59.963	177	4	0.326	0.061	6.610	3031	WS01035_O20
9	69.046	181	2	-0.230	0.102	4.576	3031	WS01035_O20
1	62.128	11	1	-0.502	0.128	4.932	3032	WS01035_P20
3	9.953	42	4	0.318	0.058	5.968	3032	WS01035_P20
4	0.000	71	4	-0.308	0.051	6.297	3032	WS01035_P20
4	116.928	82	2	0.305	0.032	3.936	3032	WS01035_P20
9	28.608	170	2	0.349	0.068	8.253	3032	WS01035_P20
9	28.608	170	3	-0.328	0.072	5.293	3032	WS01035_P20
11	17.088	203	1	0.261	0.055	4.029	3032	WS01035_P20
13	1.815	244	4	0.222	0.043	5.977	3032	WS01035_P20
1	131.686	23	2	0.537	0.096	6.637	3033	WS01036_C09
1	133.992	24	2	0.372	0.095	11.687	3033	WS01036_C09
1	141.021	25	2	-0.341	0.084	10.326	3033	WS01036_C09
4	82.462	78	2	-0.135	0.025	4.074	3033	WS01036_C09
4	92.039	79	2	-0.261	0.059	7.393	3033	WS01036_C09
4	97.576	80	2	0.259	0.050	6.229	3033	WS01036_C09
5	16.219	93	2	-0.252	0.042	6.248	3033	WS01036_C09
6	45.897	117	4	-0.246	0.055	4.286	3033	WS01036_C09
6	56.188	120	2	0.236	0.032	3.992	3033	WS01036_C09
6	87.891	124	2	0.251	0.040	4.960	3033	WS01036_C09
7	70.278	141	3	0.240	0.055	4.678	3033	WS01036_C09
8	87.463	158	2	0.148	0.031	3.998	3033	WS01036_C09
11	17.088	203	2	0.344	0.074	5.305	3033	WS01036_C09
12	6.242	234	2	-0.272	0.051	7.160	3033	WS01036_C09
1	131.686	23	2	0.486	0.072	5.601	3034	WS01036_D15
1	133.992	24	2	0.222	0.044	4.640	3034	WS01036_D15
3	148.745	68	1	0.255	0.058	4.815	3034	WS01036_D15
5	3.484	90	2	0.313	0.064	4.999	3034	WS01036_D15
6	25.914	114	1	-0.239	0.053	4.333	3034	WS01036_D15
7	105.477	147	2	-0.271	0.081	6.272	3034	WS01036_D15
11	35.604	207	3	-0.196	0.055	5.212	3034	WS01036_D15
11	42.600	213	3	-0.197	0.052	4.963	3034	WS01036_D15
14	0.000	246	2	0.217	0.054	5.403	3034	WS01036_D15
3	14.500	43	2	-0.223	0.052	6.524	3035	WS00724_O14
3	20.512	46	2	0.324	0.049	6.354	3035	WS00724_O14
9	28.608	170	3	0.198	0.039	3.888	3035	WS00724_O14
11	37.143	209	3	0.169	0.052	4.603	3035	WS00724_O14
11	56.921	219	2	-0.258	0.109	6.023	3035	WS00724_O14

1	69.854	13	2	0.320	0.070	8.731	3036	WS00725_A19
1	71.592	14	2	0.347	0.088	11.046	3036	WS00725_A19
1	77.177	15	2	0.388	0.107	13.417	3036	WS00725_A19
2	126.181	39	2	-0.243	0.033	4.088	3036	WS00725_A19
3	14.500	43	2	-0.203	0.041	5.541	3036	WS00725_A19
3	154.505	69	4	-0.142	0.049	5.102	3036	WS00725_A19
4	116.928	82	2	-0.298	0.049	5.993	3036	WS00725_A19
6	49.100	118	2	-0.355	0.129	6.649	3036	WS00725_A19
6	56.188	120	2	-0.239	0.037	4.414	3036	WS00725_A19
6	69.073	121	3	0.382	0.117	10.885	3036	WS00725_A19
6	81.996	123	3	0.297	0.197	8.205	3036	WS00725_A19
9	17.951	169	1	0.328	0.078	5.256	3036	WS00725_A19
9	28.608	170	3	0.307	0.071	7.139	3036	WS00725_A19
10	9.580	187	2	-0.169	0.068	4.955	3036	WS00725_A19
10	35.204	189	2	0.201	0.036	5.301	3036	WS00725_A19
11	64.014	222	4	-0.245	0.066	5.182	3036	WS00725_A19
1	131.686	23	2	-0.446	0.069	4.546	3037	WS00725_G01
3	83.563	56	3	-0.145	0.053	4.826	3037	WS00725_G01
4	97.576	80	2	-0.140	0.032	3.924	3037	WS00725_G01
4	116.928	82	2	-0.318	0.066	7.289	3037	WS00725_G01
6	69.073	121	4	-0.206	0.054	5.217	3037	WS00725_G01
7	70.278	141	3	-0.262	0.049	4.613	3037	WS00725_G01
9	0.000	166	1	-0.153	0.067	5.085	3037	WS00725_G01
9	28.608	170	3	0.425	0.118	9.565	3037	WS00725_G01
10	93.347	196	1	0.438	0.091	4.601	3037	WS00725_G01
1	131.686	23	2	-0.417	0.079	4.168	3038	WS00725_H11
5	57.017	102	2	-0.227	0.041	4.596	3038	WS00725_H11
6	69.073	121	3	0.271	0.051	5.518	3038	WS00725_H11
6	81.996	123	3	0.368	0.111	5.608	3038	WS00725_H11
6	106.702	128	2	-0.377	0.065	4.006	3038	WS00725_H11
1	82.301	16	3	-0.256	0.046	4.085	3039	WS00725_M08
5	52.820	99	3	-0.218	0.049	4.052	3039	WS00725_M08
5	57.017	102	2	-0.243	0.038	4.992	3039	WS00725_M08
7	17.610	131	3	-0.318	0.077	4.368	3039	WS00725_M08
11	27.405	204	4	-0.283	0.075	7.494	3039	WS00725_M08
2	63.261	30	3	0.207	0.041	3.844	3040	WS00725_N06
2	95.880	34	3	0.241	0.049	4.034	3040	WS00725_N06
3	15.147	44	2	0.266	0.071	4.645	3040	WS00725_N06
5	52.820	99	3	-0.320	0.065	4.214	3040	WS00725_N06
5	57.017	102	2	-0.231	0.043	5.658	3040	WS00725_N06
9	17.951	169	2	-0.302	0.049	5.687	3040	WS00725_N06
9	28.608	170	2	0.211	0.036	4.857	3040	WS00725_N06
9	32.356	171	2	0.168	0.031	4.178	3040	WS00725_N06
9	69.046	181	2	0.307	0.105	7.085	3040	WS00725_N06
10	21.864	188	3	0.265	0.048	4.126	3040	WS00725_N06
11	27.405	204	4	-0.361	0.081	7.037	3040	WS00725_N06
2	48.839	29	4	0.437	0.118	12.036	3041	WS00727_A02
4	0.000	71	4	-0.222	0.046	4.729	3041	WS00727_A02
6	106.702	128	2	0.399	0.125	4.620	3041	WS00727_A02
8	39.090	152	4	-0.253	0.038	3.989	3041	WS00727_A02
9	28.608	170	3	0.293	0.047	4.001	3041	WS00727_A02
10	104.214	198	2	0.186	0.028	5.187	3041	WS00727_A02
11	9.237	202	4	0.209	0.063	6.473	3041	WS00727_A02
11	27.405	204	4	-0.290	0.060	6.822	3041	WS00727_A02

1	15.589	3	2	0.236	0.054	7.151	3042	WS00727_B09
2	126.181	39	2	-0.219	0.038	5.101	3042	WS00727_B09
3	20.512	46	2	0.234	0.049	6.551	3042	WS00727_B09
6	106.702	128	2	0.549	0.105	8.318	3042	WS00727_B09
8	78.389	157	1	0.200	0.073	3.977	3042	WS00727_B09
11	27.405	204	4	-0.285	0.087	6.358	3042	WS00727_B09
11	45.989	216	2	0.187	0.032	4.059	3042	WS00727_B09
11	60.464	221	2	0.323	0.056	7.455	3042	WS00727_B09
1	131.686	23	2	-0.442	0.059	4.786	3043	WS01026_O06
4	173.929	88	2	-0.182	0.033	3.958	3043	WS01026_O06
6	81.996	123	3	0.308	0.098	4.271	3043	WS01026_O06
8	50.001	154	1	-0.231	0.067	4.878	3043	WS01026_O06
10	78.479	193	3	0.277	0.056	4.392	3043	WS01026_O06
11	27.405	204	4	-0.307	0.060	6.110	3043	WS01026_O06
1	22.419	4	3	-0.319	0.079	4.474	3044	WS00912_D14
2	48.839	29	4	0.333	0.060	4.719	3044	WS00912_D14
2	95.880	34	1	0.308	0.058	5.344	3044	WS00912_D14
6	69.073	121	4	-0.237	0.050	4.872	3044	WS00912_D14
7	73.169	142	2	-0.447	0.076	4.784	3044	WS00912_D14
8	50.001	154	2	-0.218	0.052	4.678	3044	WS00912_D14
11	60.464	221	2	0.282	0.058	5.643	3044	WS00912_D14
2	120.617	38	3	-0.253	0.061	5.752	3045	WS00819_D04
3	14.500	43	1	-0.269	0.046	3.924	3045	WS00819_D04
4	92.039	79	2	-0.224	0.048	4.972	3045	WS00819_D04
6	89.401	125	2	0.262	0.051	4.620	3045	WS00819_D04
9	36.227	165	4	0.195	0.042	3.924	3045	WS00819_D04
9	28.608	170	2	0.258	0.040	4.117	3045	WS00819_D04
13	1.815	244	4	0.253	0.060	4.863	3045	WS00819_D04
1	131.686	23	2	-0.401	0.048	4.240	3046	WS00912_I21
3	116.958	63	4	-0.251	0.057	6.737	3046	WS00912_I21
3	137.891	64	3	0.247	0.069	4.572	3046	WS00912_I21
4	173.929	88	2	-0.215	0.042	4.929	3046	WS00912_I21
6	25.914	114	1	-0.272	0.092	6.473	3046	WS00912_I21
8	39.090	152	4	-0.238	0.039	4.695	3046	WS00912_I21
11	27.405	204	4	-0.366	0.080	5.761	3046	WS00912_I21
11	46.026	217	1	-0.287	0.086	6.065	3046	WS00912_I21
1	82.301	16	4	0.249	0.042	5.100	3047	WS00829_H20
1	127.805	21	4	-0.203	0.036	4.526	3047	WS00829_H20
4	0.000	71	4	0.232	0.076	7.911	3047	WS00829_H20
6	56.188	120	2	-0.300	0.043	4.327	3047	WS00829_H20
8	87.463	158	2	-0.099	0.031	3.861	3047	WS00829_H20
9	17.951	169	2	-0.234	0.053	5.112	3047	WS00829_H20
3	20.512	46	2	0.208	0.039	4.063	3048	WS00830_C19
4	173.929	88	2	-0.221	0.044	4.515	3048	WS00830_C19
6	69.073	121	3	0.284	0.076	7.040	3048	WS00830_C19
7	105.477	147	2	-0.275	0.064	4.222	3048	WS00830_C19
9	59.963	177	4	0.251	0.047	4.481	3048	WS00830_C19
1	15.589	3	2	-0.192	0.038	5.116	3049	WS00931_M15
1	62.128	11	2	-0.367	0.092	4.566	3049	WS00931_M15
3	95.157	59	2	0.187	0.036	4.787	3049	WS00931_M15
11	27.405	204	4	0.309	0.049	4.774	3049	WS00931_M15
14	0.000	246	2	-0.216	0.042	5.189	3049	WS00931_M15
5	86.669	106	4	-0.342	0.079	3.962	3050	WS01029_B02
6	87.891	124	2	0.226	0.059	6.785	3050	WS01029_B02

6	89.401	125	2	0.396	0.083	6.518	3050	WS01029_B02
7	15.555	130	2	0.274	0.036	4.171	3050	WS01029_B02
8	39.090	152	4	-0.455	0.184	23.660	3050	WS01029_B02
10	78.479	193	3	0.361	0.076	4.610	3050	WS01029_B02
11	43.491	214	4	0.159	0.032	3.975	3050	WS01029_B02
12	6.242	234	4	-0.394	0.077	9.771	3050	WS01029_B02
1	45.794	9	1	0.293	0.043	3.975	3051	WS00913_J01
1	82.301	16	4	0.270	0.042	3.969	3051	WS00913_J01
2	102.814	36	2	0.271	0.064	5.628	3051	WS00913_J01
2	126.181	39	2	-0.241	0.040	3.870	3051	WS00913_J01
4	92.039	79	4	-0.290	0.045	3.943	3051	WS00913_J01
6	69.073	121	3	0.268	0.064	5.902	3051	WS00913_J01
11	46.026	217	1	-0.263	0.051	4.699	3051	WS00913_J01
1	15.589	3	2	0.189	0.043	4.345	3052	WS00913_O23
3	15.147	44	2	0.284	0.082	4.753	3052	WS00913_O23
3	116.958	63	4	-0.284	0.093	9.113	3052	WS00913_O23
3	148.745	68	1	-0.246	0.050	4.285	3052	WS00913_O23
4	116.928	82	2	-0.279	0.056	5.856	3052	WS00913_O23
4	116.928	82	4	0.259	0.040	3.942	3052	WS00913_O23
4	124.132	83	2	-0.279	0.094	4.716	3052	WS00913_O23
6	81.996	123	3	0.289	0.083	4.005	3052	WS00913_O23
9	2.011	167	4	-0.282	0.066	6.517	3052	WS00913_O23
10	70.246	190	4	-0.267	0.052	5.090	3052	WS00913_O23
11	57.661	220	4	0.214	0.050	4.928	3052	WS00913_O23
11	64.014	222	4	-0.417	0.122	11.409	3052	WS00913_O23
13	3.386	245	1	-0.251	0.051	4.430	3052	WS00913_O23
3	9.953	42	3	0.195	0.053	4.216	3053	WS01029_N14
6	81.996	123	3	0.279	0.102	4.303	3053	WS01029_N14
7	15.555	130	2	-0.255	0.041	4.760	3053	WS01029_N14
10	78.479	193	3	0.359	0.081	6.443	3053	WS01029_N14
11	27.405	204	4	-0.306	0.051	4.632	3053	WS01029_N14
14	0.000	246	2	-0.215	0.036	4.346	3053	WS01029_N14
2	48.839	29	4	0.340	0.072	6.513	3054	WS01029_O05
3	83.563	56	3	-0.203	0.042	4.260	3054	WS01029_O05
10	21.864	188	1	-0.291	0.058	4.216	3054	WS01029_O05
11	27.405	204	4	-0.315	0.063	5.637	3054	WS01029_O05
3	9.953	42	3	0.229	0.056	5.614	3055	WS01029_P21
3	95.157	59	2	0.159	0.029	3.842	3055	WS01029_P21
5	54.072	100	4	0.221	0.051	4.506	3055	WS01029_P21
5	56.008	101	2	0.219	0.026	3.893	3055	WS01029_P21
5	57.017	102	2	-0.249	0.048	7.205	3055	WS01029_P21
7	17.610	131	3	-0.355	0.119	8.957	3055	WS01029_P21
9	69.046	181	2	0.254	0.067	5.071	3055	WS01029_P21
11	27.405	204	4	-0.292	0.054	3.866	3055	WS01029_P21
1	2.788	2	3	0.219	0.033	4.270	3056	WS01030_A07
1	45.794	9	3	0.251	0.043	5.135	3056	WS01030_A07
1	62.128	11	2	0.248	0.045	4.928	3056	WS01030_A07
1	127.805	21	3	0.221	0.031	4.058	3056	WS01030_A07
1	131.686	23	2	-0.333	0.068	4.306	3056	WS01030_A07
1	133.992	24	2	-0.226	0.041	6.652	3056	WS01030_A07
3	83.563	56	3	-0.215	0.040	5.232	3056	WS01030_A07
3	116.958	63	4	-0.192	0.072	4.169	3056	WS01030_A07
7	105.477	147	2	0.275	0.088	4.269	3056	WS01030_A07
9	49.013	173	2	0.190	0.030	4.869	3056	WS01030_A07

9	69.046	181	2	0.282	0.061	6.961	3056	WS01030_A07
10	21.864	188	3	0.191	0.033	4.308	3056	WS01030_A07
10	78.479	193	3	-0.266	0.047	6.033	3056	WS01030_A07
10	86.151	195	3	0.202	0.037	4.778	3056	WS01030_A07
12	6.242	234	2	0.322	0.056	9.334	3056	WS01030_A07
3	95.157	59	2	0.229	0.044	5.451	3057	WS01030_C13
3	116.958	63	4	-0.216	0.046	3.908	3057	WS01030_C13
7	17.610	131	3	-0.304	0.081	5.994	3057	WS01030_C13
8	50.001	154	1	-0.218	0.063	3.972	3057	WS01030_C13
8	62.580	155	1	-0.349	0.155	4.486	3057	WS01030_C13
14	44.898	251	3	-0.253	0.054	5.564	3057	WS01030_C13
2	48.839	29	1	-0.284	0.046	6.319	3058	WS01030_C23
3	14.500	43	1	0.422	0.080	10.710	3058	WS01030_C23
3	17.158	45	1	-0.158	0.052	6.834	3058	WS01030_C23
3	141.443	65	4	-0.161	0.065	3.979	3058	WS01030_C23
3	144.593	66	4	-0.232	0.035	4.099	3058	WS01030_C23
4	82.462	78	1	0.276	0.031	4.336	3058	WS01030_C23
5	0.000	89	1	-0.321	0.035	4.908	3058	WS01030_C23
7	67.154	140	4	0.202	0.086	6.555	3058	WS01030_C23
7	75.883	144	2	-0.547	0.224	8.232	3058	WS01030_C23
7	75.883	144	4	0.562	0.132	15.156	3058	WS01030_C23
8	50.001	154	1	-0.172	0.057	7.207	3058	WS01030_C23
9	55.358	175	3	0.464	0.117	4.848	3058	WS01030_C23
11	9.237	202	4	-0.229	0.061	6.514	3058	WS01030_C23
11	27.405	204	1	0.211	0.028	3.863	3058	WS01030_C23
11	64.014	222	4	-0.219	0.077	6.793	3058	WS01030_C23
12	0.000	233	1	-0.601	0.191	25.781	3058	WS01030_C23
12	22.629	237	1	-0.832	0.338	45.841	3058	WS01030_C23
12	32.473	238	1	-0.641	0.186	25.402	3058	WS01030_C23
12	51.382	242	1	0.727	0.175	14.001	3058	WS01030_C23
13	1.815	244	4	0.097	0.032	3.993	3058	WS01030_C23
1	131.686	23	2	-0.447	0.068	6.243	3059	WS01030_E23
3	116.958	63	4	-0.257	0.063	7.484	3059	WS01030_E23
4	97.576	80	2	-0.185	0.032	4.117	3059	WS01030_E23
6	81.996	123	3	0.302	0.128	4.939	3059	WS01030_E23
7	70.278	141	3	-0.243	0.075	5.239	3059	WS01030_E23
8	39.090	152	4	-0.237	0.043	5.103	3059	WS01030_E23
11	60.464	221	2	0.255	0.044	5.536	3059	WS01030_E23
5	0.000	89	1	-0.261	0.048	4.060	3060	WS01030_F11
5	52.820	99	1	-0.267	0.048	3.977	3060	WS01030_F11
6	49.100	118	4	0.302	0.089	4.814	3060	WS01030_F11
6	81.996	123	4	-0.356	0.085	4.020	3060	WS01030_F11
8	11.974	150	1	0.321	0.111	9.213	3060	WS01030_F11
9	3.127	168	1	0.429	0.124	6.166	3060	WS01030_F11
12	22.441	227	1	0.288	0.067	5.661	3060	WS01030_F11
12	22.629	237	1	0.266	0.090	7.448	3060	WS01030_F11
12	32.473	238	1	0.228	0.066	5.487	3060	WS01030_F11
3	9.953	42	3	0.203	0.043	4.014	3061	WS00915_A16
3	15.147	44	2	0.343	0.099	7.332	3061	WS00915_A16
3	104.681	60	2	0.220	0.044	5.610	3061	WS00915_A16
3	112.868	61	2	-0.180	0.039	4.990	3061	WS00915_A16
3	114.747	62	2	-0.247	0.044	5.638	3061	WS00915_A16
3	144.593	66	4	0.230	0.044	4.288	3061	WS00915_A16
6	81.996	123	3	0.313	0.156	5.990	3061	WS00915_A16

6	106.702	128	2	0.481	0.106	5.933	3061	WS00915_A16
11	27.405	204	4	-0.357	0.083	6.514	3061	WS00915_A16
1	133.992	24	2	-0.209	0.030	4.014	3062	WS00915_A24
3	15.147	44	2	0.278	0.070	4.478	3062	WS00915_A24
3	20.512	46	2	0.209	0.030	3.940	3062	WS00915_A24
5	54.072	100	4	0.226	0.061	5.047	3062	WS00915_A24
6	56.188	120	2	-0.283	0.048	6.466	3062	WS00915_A24
9	69.046	181	2	0.234	0.064	4.597	3062	WS00915_A24
2	93.504	32	4	-0.232	0.045	4.096	3063	WS00915_C15
4	116.928	82	2	0.257	0.051	5.602	3064	WS00915_D12
4	173.929	88	2	-0.216	0.041	4.476	3064	WS00915_D12
5	57.017	102	2	-0.216	0.041	4.407	3064	WS00915_D12
1	2.788	2	4	-0.222	0.051	4.237	3065	WS00915_F05
4	173.929	88	2	-0.234	0.046	5.047	3065	WS00915_F05
6	81.996	123	3	0.346	0.097	4.845	3065	WS00915_F05
8	50.001	154	1	-0.241	0.051	4.085	3065	WS00915_F05
12	6.242	234	2	-0.196	0.038	4.304	3065	WS00915_F05
1	82.301	16	4	0.252	0.047	4.258	3066	WS00915_F23
4	173.929	88	2	-0.247	0.053	5.898	3066	WS00915_F23
1	77.177	15	2	0.146	0.035	3.849	3067	WS00915_I03
2	93.504	32	4	-0.347	0.061	6.481	3067	WS00915_I03
2	126.181	39	4	0.362	0.057	3.882	3067	WS00915_I03
3	14.500	43	1	-0.299	0.053	4.117	3067	WS00915_I03
3	17.158	45	1	0.231	0.082	6.192	3067	WS00915_I03
3	137.891	64	3	-0.300	0.055	4.120	3067	WS00915_I03
4	116.928	82	2	0.218	0.047	5.395	3067	WS00915_I03
5	86.669	106	4	0.400	0.165	6.356	3067	WS00915_I03
9	59.963	177	4	0.303	0.064	6.826	3067	WS00915_I03
11	27.405	204	1	-0.364	0.076	5.757	3067	WS00915_I03
11	29.069	205	1	-0.229	0.051	3.899	3067	WS00915_I03
12	22.441	227	1	-0.326	0.086	6.633	3067	WS00915_I03
5	54.072	100	4	0.213	0.049	3.898	3068	WS00915_I14
8	50.001	154	1	-0.241	0.032	4.780	3068	WS00915_I14
1	141.021	25	2	-0.239	0.049	6.232	3069	WS0053_M08
3	83.563	56	3	-0.195	0.068	8.198	3069	WS0053_M08
3	137.891	64	3	-0.508	0.110	13.056	3069	WS0053_M08
3	141.443	65	3	-0.415	0.254	11.449	3069	WS0053_M08
6	56.188	120	2	-0.306	0.033	4.339	3069	WS0053_M08
6	81.996	123	3	0.280	0.115	5.173	3069	WS0053_M08
10	93.347	196	1	0.402	0.086	3.870	3069	WS0053_M08
10	104.214	198	4	-0.286	0.067	5.817	3069	WS0053_M08
11	44.884	215	3	0.269	0.043	5.265	3069	WS0053_M08
11	64.014	222	3	0.246	0.032	3.966	3069	WS0053_M08
1	123.316	20	4	-0.264	0.060	4.874	3070	WS0053_M24
2	101.076	35	4	0.151	0.017	4.015	3070	WS0053_M24
4	6.032	72	4	-0.164	0.034	3.932	3070	WS0053_M24
4	97.576	80	2	-0.211	0.024	4.981	3070	WS0053_M24
6	89.401	125	2	0.217	0.043	5.635	3070	WS0053_M24
7	62.787	139	4	-0.189	0.028	4.375	3070	WS0053_M24
7	75.883	144	4	-0.295	0.036	4.073	3070	WS0053_M24
9	3.127	168	1	-0.438	0.149	5.538	3070	WS0053_M24
9	17.951	169	1	0.213	0.068	4.483	3070	WS0053_M24
10	80.088	194	1	-0.274	0.060	4.106	3070	WS0053_M24
2	93.504	32	4	-0.288	0.077	6.489	3071	WS0054_A01

2	120.617	38	4	-0.218	0.043	3.876	3071	WS0054_A01
3	47.928	49	1	0.156	0.033	4.116	3071	WS0054_A01
4	92.039	79	4	-0.339	0.064	5.323	3071	WS0054_A01
5	42.979	97	1	-0.239	0.043	5.353	3071	WS0054_A01
10	93.347	196	1	0.496	0.093	5.904	3071	WS0054_A01
12	38.543	232	4	0.260	0.075	4.598	3071	WS0054_A01
13	0.000	243	4	0.390	0.093	5.728	3071	WS0054_A01
1	95.572	17	2	0.246	0.049	4.383	3072	WS0054_B05
5	31.191	95	1	0.233	0.048	4.706	3072	WS0054_B05
5	52.820	99	3	-0.260	0.061	5.180	3072	WS0054_B05
7	105.477	147	2	-0.282	0.084	4.778	3072	WS0054_B05
9	3.127	168	1	-0.338	0.081	5.231	3072	WS0054_B05
10	93.347	196	1	0.444	0.090	4.732	3072	WS0054_B05
4	173.929	88	2	-0.196	0.043	4.075	3073	WS0054_F10
5	3.484	90	2	-0.212	0.063	4.023	3073	WS0054_F10
6	81.996	123	3	0.383	0.114	6.706	3073	WS0054_F10
9	2.011	167	4	-0.257	0.047	4.693	3073	WS0054_F10
1	29.800	5	1	-0.279	0.044	5.012	3074	WS0054_G07
1	69.854	13	2	0.164	0.056	4.198	3074	WS0054_G07
1	71.592	14	2	0.159	0.054	4.004	3074	WS0054_G07
1	77.177	15	2	0.162	0.055	4.080	3074	WS0054_G07
4	97.576	80	2	-0.221	0.054	3.844	3074	WS0054_G07
6	54.066	119	4	0.179	0.059	3.975	3074	WS0054_G07
7	70.278	141	3	-0.227	0.038	4.598	3074	WS0054_G07
9	55.358	175	3	0.434	0.051	4.249	3074	WS0054_G07
9	58.045	176	1	-0.386	0.106	9.894	3074	WS0054_G07
9	69.046	181	1	0.336	0.081	7.439	3074	WS0054_G07
11	64.014	222	3	0.176	0.033	3.915	3074	WS0054_G07
12	14.406	230	3	0.271	0.040	4.843	3074	WS0054_G07
1	62.128	11	1	-0.627	0.181	7.461	3075	WS0054_K07
4	173.929	88	2	-0.228	0.057	6.568	3075	WS0054_K07
11	60.464	221	2	0.301	0.053	6.133	3075	WS0054_K07
2	102.814	36	1	-0.212	0.061	3.893	3076	WS0054_K20
3	9.953	42	3	0.463	0.154	15.872	3076	WS0054_K20
4	173.929	88	2	-0.218	0.037	4.414	3076	WS0054_K20
5	0.000	89	3	0.191	0.044	4.506	3076	WS0054_K20
9	17.951	169	2	-0.225	0.034	3.945	3076	WS0054_K20
1	62.128	11	2	0.372	0.090	5.762	3077	WS00911_I16
4	124.132	83	1	0.455	0.111	4.158	3077	WS00911_I16
5	0.000	89	1	-0.265	0.072	5.239	3077	WS00911_I16
5	0.000	89	3	0.336	0.093	10.034	3077	WS00911_I16
6	45.897	117	1	0.305	0.065	4.953	3077	WS00911_I16
6	49.100	118	4	0.422	0.162	4.434	3077	WS00911_I16
9	69.046	181	2	0.233	0.062	4.148	3077	WS00911_I16
10	78.479	193	3	-0.435	0.092	9.540	3077	WS00911_I16
3	116.958	63	4	-0.199	0.050	4.455	3078	WS00911_N23
5	7.672	92	4	0.261	0.059	5.624	3078	WS00911_N23
5	52.820	99	3	-0.294	0.047	4.712	3078	WS00911_N23
7	17.610	131	3	-0.221	0.065	4.100	3078	WS00911_N23
9	17.951	169	2	-0.265	0.048	6.338	3078	WS00911_N23
3	15.147	44	2	0.332	0.060	4.322	3079	WS00828_A08
3	116.958	63	4	-0.246	0.043	4.791	3079	WS00828_A08
11	27.405	204	4	-0.214	0.035	4.162	3079	WS00828_A08
13	0.000	243	4	0.365	0.126	4.105	3079	WS00828_A08

1	123.316	20	4	0.273	0.055	3.873	3080	WS01031_C24
1	127.805	21	4	-0.207	0.050	4.995	3080	WS01031_C24
1	131.686	23	2	-0.459	0.075	4.359	3080	WS01031_C24
9	28.608	170	3	0.267	0.050	4.317	3080	WS01031_C24
10	93.347	196	1	0.462	0.078	5.420	3080	WS01031_C24
11	27.405	204	4	-0.293	0.052	5.006	3080	WS01031_C24
11	56.921	219	1	-0.363	0.181	5.153	3080	WS01031_C24
4	124.132	83	1	0.420	0.060	3.937	3081	WS00828_M17
6	69.073	121	3	0.177	0.057	4.305	3081	WS00828_M17
4	116.928	82	2	0.214	0.043	3.922	3082	WS01031_H12
5	52.820	99	3	-0.261	0.049	4.805	3082	WS01031_H12
6	81.996	123	3	0.413	0.118	8.099	3082	WS01031_H12
1	29.800	5	1	-0.301	0.063	7.226	3083	WS00935_G01
1	95.572	17	2	0.258	0.068	5.111	3083	WS00935_G01
1	95.572	17	3	-0.204	0.042	4.470	3083	WS00935_G01
4	82.462	78	1	-0.182	0.050	5.658	3083	WS00935_G01
4	124.132	83	1	-0.485	0.100	4.696	3083	WS00935_G01
4	142.917	85	1	0.450	0.121	13.914	3083	WS00935_G01
4	173.929	88	2	-0.203	0.061	4.488	3083	WS00935_G01
10	70.246	190	2	-0.245	0.065	4.690	3083	WS00935_G01
10	70.246	190	4	0.390	0.098	8.499	3083	WS00935_G01
11	45.989	216	1	0.219	0.043	4.911	3083	WS00935_G01
14	0.000	246	1	-0.334	0.088	10.083	3083	WS00935_G01
2	126.181	39	2	-0.210	0.036	3.909	3084	WS00935_G19
3	15.147	44	2	0.258	0.064	4.149	3084	WS00935_G19
6	56.188	120	2	-0.240	0.048	4.992	3084	WS00935_G19
7	15.555	130	3	0.231	0.063	6.478	3084	WS00935_G19
7	17.610	131	3	0.317	0.121	5.312	3084	WS00935_G19
9	17.951	169	2	-0.239	0.045	4.821	3084	WS00935_G19
9	28.608	170	2	0.209	0.042	4.599	3084	WS00935_G19
10	21.864	188	1	-0.300	0.056	4.451	3084	WS00935_G19
4	75.958	77	3	0.321	0.121	6.377	3085	WS00935_J01
4	173.929	88	2	-0.226	0.046	4.863	3085	WS00935_J01
6	81.996	123	3	0.353	0.097	5.111	3085	WS00935_J01
8	50.001	154	1	-0.283	0.070	5.927	3085	WS00935_J01
10	78.479	193	3	0.260	0.039	4.091	3085	WS00935_J01
1	29.800	5	1	-0.309	0.069	5.717	3086	WS00935_K12
1	95.572	17	2	0.221	0.043	4.450	3086	WS00935_K12
4	116.928	82	2	0.221	0.047	4.997	3086	WS00935_K12
5	84.347	105	1	0.203	0.057	4.746	3086	WS00935_K12
10	35.204	189	1	-0.235	0.056	4.658	3086	WS00935_K12
10	78.479	193	3	0.242	0.035	4.073	3086	WS00935_K12
1	29.800	5	1	-0.244	0.032	3.909	3087	WS00935_P11
4	173.929	88	2	-0.243	0.063	4.247	3087	WS00935_P11
14	0.000	246	1	-0.251	0.037	4.515	3087	WS00935_P11
1	29.800	5	1	-0.245	0.043	4.083	3088	WS00827_B02
4	75.958	77	3	0.294	0.104	5.249	3088	WS00827_B02
14	0.000	246	1	-0.358	0.090	8.663	3088	WS00827_B02
2	48.839	29	4	-0.274	0.064	4.242	3089	WS00827_I15
6	69.073	121	3	0.296	0.061	7.513	3089	WS00827_I15
11	0.000	199	2	-0.272	0.049	4.587	3089	WS00827_I15
11	0.000	199	3	0.341	0.054	6.737	3089	WS00827_I15
11	17.088	203	1	0.287	0.055	5.592	3089	WS00827_I15
11	27.405	204	1	-0.343	0.064	6.354	3089	WS00827_I15

11	29.069	205	1	-0.278	0.066	6.580	3089	WS00827_I15
11	45.989	216	1	0.157	0.040	3.951	3089	WS00827_I15
5	52.820	99	3	-0.294	0.047	4.545	3090	WS00827_J15
6	81.996	123	3	0.344	0.067	4.878	3090	WS00827_J15
11	0.000	199	3	0.372	0.067	5.340	3090	WS00827_J15
11	27.405	204	1	-0.314	0.054	5.169	3090	WS00827_J15
11	56.921	219	2	-0.250	0.101	4.127	3090	WS00827_J15
14	0.000	246	1	-0.292	0.057	5.404	3090	WS00827_J15
1	45.794	9	1	0.303	0.052	4.169	3091	WS00915_K20
3	154.505	69	1	0.208	0.058	4.862	3091	WS00915_K20
4	92.039	79	4	-0.287	0.041	4.382	3091	WS00915_K20
5	54.072	100	4	0.201	0.045	4.825	3091	WS00915_K20
5	57.017	102	4	-0.291	0.047	5.063	3091	WS00915_K20
1	2.788	2	4	-0.233	0.044	4.800	3092	WS00915_L22
4	75.958	77	3	0.323	0.119	6.098	3092	WS00915_L22
5	31.191	95	1	0.245	0.058	4.169	3092	WS00915_L22
9	59.963	177	4	0.279	0.051	4.819	3092	WS00915_L22
1	40.464	7	4	0.161	0.030	4.090	3093	WS00915_O19
3	148.745	68	4	0.182	0.033	4.473	3093	WS00915_O19
4	82.462	78	2	-0.322	0.077	9.063	3093	WS00915_O19
4	97.576	80	2	0.259	0.058	8.458	3093	WS00915_O19
5	3.484	90	2	0.272	0.048	4.744	3093	WS00915_O19
5	16.219	93	2	-0.238	0.034	4.159	3093	WS00915_O19
6	25.914	114	1	0.213	0.072	4.569	3093	WS00915_O19
6	69.073	121	4	-0.253	0.046	6.181	3093	WS00915_O19
9	17.951	169	2	0.178	0.035	3.897	3093	WS00915_O19
10	9.580	187	2	-0.327	0.089	8.892	3093	WS00915_O19
13	1.815	244	4	-0.292	0.038	5.226	3093	WS00915_O19
14	22.402	248	4	-0.354	0.034	3.913	3093	WS00915_O19
3	15.147	44	2	0.234	0.067	4.550	3094	WS00915_P15
4	173.929	88	2	-0.201	0.052	5.434	3094	WS00915_P15
6	81.996	123	3	0.307	0.083	4.388	3094	WS00915_P15
8	50.001	154	1	-0.190	0.049	4.309	3094	WS00915_P15
10	70.246	190	2	-0.213	0.040	4.262	3094	WS00915_P15
12	6.242	234	2	-0.249	0.054	6.322	3094	WS00915_P15
3	15.147	44	2	0.285	0.067	5.028	3095	WS00916_B06
3	137.891	64	3	0.247	0.053	4.146	3095	WS00916_B06
4	142.917	85	1	0.222	0.057	4.134	3095	WS00916_B06
4	142.917	85	3	-0.231	0.049	4.099	3095	WS00916_B06
4	173.929	88	2	-0.188	0.040	4.659	3095	WS00916_B06
6	81.996	123	3	0.339	0.109	5.007	3095	WS00916_B06
7	15.555	130	2	-0.251	0.040	4.601	3095	WS00916_B06
10	78.479	193	3	0.303	0.066	5.455	3095	WS00916_B06
11	27.405	204	4	-0.307	0.055	5.641	3095	WS00916_B06
1	2.788	2	4	-0.252	0.091	7.792	3096	WS00916_B20
3	20.512	46	2	0.159	0.042	4.364	3096	WS00916_B20
4	75.958	77	3	0.466	0.192	10.060	3096	WS00916_B20
6	89.401	125	2	0.238	0.036	3.959	3096	WS00916_B20
6	95.249	126	1	-0.480	0.134	7.720	3096	WS00916_B20
7	17.610	131	3	-0.403	0.129	6.802	3096	WS00916_B20
14	0.000	246	2	0.217	0.047	4.946	3096	WS00916_B20
3	9.953	42	3	0.279	0.062	6.762	3097	WS00916_D20
3	116.958	63	4	-0.240	0.096	7.571	3097	WS00916_D20
5	57.017	102	2	-0.177	0.028	3.843	3097	WS00916_D20

7	17.610	131	3	-0.243	0.071	5.310	3097	WS00916_D20
9	17.951	169	2	-0.261	0.033	3.877	3097	WS00916_D20
9	49.013	173	2	0.245	0.046	5.592	3097	WS00916_D20
9	69.046	181	2	0.221	0.063	4.779	3097	WS00916_D20
1	95.572	17	2	0.242	0.039	4.897	3098	WS00916_E07
3	15.147	44	2	0.320	0.090	5.401	3098	WS00916_E07
4	75.958	77	3	0.254	0.062	3.900	3098	WS00916_E07
5	54.072	100	4	0.215	0.067	5.854	3098	WS00916_E07
5	56.008	101	2	0.194	0.029	4.031	3098	WS00916_E07
5	57.017	102	2	-0.243	0.047	5.864	3098	WS00916_E07
7	15.555	130	2	-0.254	0.044	5.505	3098	WS00916_E07
7	17.610	131	3	-0.307	0.092	5.729	3098	WS00916_E07
8	50.001	154	1	-0.237	0.081	5.486	3098	WS00916_E07
9	17.951	169	2	-0.269	0.042	5.432	3098	WS00916_E07
11	27.405	204	4	-0.299	0.061	5.516	3098	WS00916_E07
3	15.147	44	2	0.271	0.090	6.164	3099	WS00727_E22
5	54.072	100	4	0.277	0.064	5.814	3099	WS00727_E22
5	57.017	102	2	-0.187	0.037	4.798	3099	WS00727_E22
6	56.188	120	2	-0.265	0.032	4.228	3099	WS00727_E22
7	15.555	130	2	-0.281	0.037	5.082	3099	WS00727_E22
7	17.610	131	3	-0.222	0.072	4.694	3099	WS00727_E22
9	17.951	169	2	-0.311	0.044	5.382	3099	WS00727_E22
2	63.261	30	3	0.184	0.036	3.895	3100	WS00727_F24
4	75.958	77	3	0.234	0.050	3.908	3100	WS00727_F24
5	56.008	101	2	0.214	0.031	4.374	3100	WS00727_F24
5	57.017	102	2	-0.237	0.047	6.315	3100	WS00727_F24
6	56.188	120	2	-0.242	0.033	4.620	3100	WS00727_F24
7	17.610	131	3	-0.243	0.056	4.323	3100	WS00727_F24
9	49.013	173	2	0.241	0.041	5.565	3100	WS00727_F24
1	69.854	13	2	0.236	0.049	6.196	3101	WS00727_I19
1	71.592	14	2	0.241	0.052	6.542	3101	WS00727_I19
1	77.177	15	2	0.248	0.054	6.838	3101	WS00727_I19
1	95.572	17	2	0.264	0.038	4.845	3101	WS00727_I19
1	123.316	20	4	0.294	0.058	4.783	3101	WS00727_I19
1	131.686	23	2	-0.520	0.089	6.231	3101	WS00727_I19
2	48.839	29	4	0.389	0.082	7.662	3101	WS00727_I19
3	116.958	63	4	-0.150	0.036	3.947	3101	WS00727_I19
4	124.132	83	1	-0.444	0.147	3.978	3101	WS00727_I19
5	54.072	100	4	0.256	0.066	7.233	3101	WS00727_I19
3	15.147	44	2	0.321	0.079	4.483	3102	WS00727_K05
3	47.928	49	1	-0.258	0.049	4.442	3102	WS00727_K05
4	75.958	77	3	0.211	0.111	4.122	3102	WS00727_K05
6	81.996	123	3	0.362	0.116	5.211	3102	WS00727_K05
7	75.883	144	2	0.374	0.063	3.910	3102	WS00727_K05
7	75.883	144	4	-0.277	0.052	5.499	3102	WS00727_K05
8	39.090	152	4	-0.203	0.040	4.216	3102	WS00727_K05
8	62.580	155	1	-0.324	0.094	4.325	3102	WS00727_K05
11	9.237	202	4	0.219	0.039	4.189	3102	WS00727_K05
3	9.953	42	4	0.190	0.038	5.030	3103	WS00727_P02
6	81.996	123	3	0.349	0.102	4.341	3103	WS00727_P02
9	59.963	177	4	0.381	0.101	10.264	3103	WS00727_P02
11	40.960	210	2	0.416	0.055	5.130	3103	WS00727_P02
11	48.979	218	2	0.337	0.060	7.403	3103	WS00727_P02
3	15.147	44	2	0.323	0.083	5.118	3104	WS00832_A06

3	20.512	46	2	0.250	0.047	5.366	3104	WS00832_A06
5	52.820	99	3	-0.335	0.074	6.407	3104	WS00832_A06
5	54.072	100	4	0.203	0.044	4.236	3104	WS00832_A06
9	17.951	169	2	-0.247	0.042	4.494	3104	WS00832_A06
9	59.963	177	4	0.238	0.047	3.931	3104	WS00832_A06
1	95.572	17	2	0.227	0.033	3.849	3105	WS00832_F22
3	15.147	44	2	0.399	0.141	8.499	3105	WS00832_F22
4	75.958	77	3	0.353	0.155	8.068	3105	WS00832_F22
5	54.072	100	4	0.216	0.071	6.248	3105	WS00832_F22
5	57.017	102	2	-0.196	0.040	4.582	3105	WS00832_F22
6	56.188	120	2	-0.242	0.040	5.023	3105	WS00832_F22
6	81.996	123	3	0.328	0.088	4.244	3105	WS00832_F22
9	59.963	177	4	0.302	0.088	7.059	3105	WS00832_F22
2	48.839	29	4	-0.188	0.046	4.019	3106	WS00832_H03
3	144.593	66	4	-0.180	0.046	4.274	3106	WS00832_H03
4	43.146	74	3	-0.244	0.054	4.729	3106	WS00832_H03
4	75.958	77	3	0.261	0.098	4.864	3106	WS00832_H03
4	140.962	84	3	-0.334	0.127	10.986	3106	WS00832_H03
4	142.917	85	1	0.244	0.069	4.659	3106	WS00832_H03
4	142.917	85	3	-0.418	0.119	10.541	3106	WS00832_H03
9	28.608	170	2	-0.251	0.031	3.930	3106	WS00832_H03
9	59.963	177	4	0.211	0.050	4.106	3106	WS00832_H03
11	43.491	214	4	-0.213	0.057	5.952	3106	WS00832_H03
12	0.000	226	3	-0.246	0.072	6.236	3106	WS00832_H03
12	6.242	234	2	-0.191	0.029	3.989	3106	WS00832_H03
2	48.839	29	4	-0.264	0.049	5.079	3107	WS00821_N10
3	9.953	42	4	0.226	0.045	4.387	3107	WS00821_N10
11	27.405	204	1	-0.188	0.050	4.269	3107	WS00821_N10
12	28.493	228	2	0.279	0.064	4.098	3107	WS00821_N10
12	22.629	237	1	-0.244	0.054	4.594	3107	WS00821_N10
12	32.473	238	1	-0.310	0.086	7.355	3107	WS00821_N10
14	22.402	248	4	-0.347	0.079	8.291	3107	WS00821_N10
14	46.637	252	4	-0.270	0.049	4.883	3107	WS00821_N10
1	2.788	2	4	-0.240	0.052	4.071	3108	WS01027_C16
1	29.800	5	1	-0.243	0.043	4.929	3108	WS01027_C16
3	14.500	43	1	-0.338	0.065	7.451	3108	WS01027_C16
3	17.158	45	1	0.175	0.043	5.001	3108	WS01027_C16
1	29.800	5	1	-0.251	0.033	4.001	3109	WS00823_H10
1	62.128	11	1	-0.470	0.125	4.198	3109	WS00823_H10
3	14.500	43	1	-0.269	0.043	5.198	3109	WS00823_H10
3	17.158	45	1	0.188	0.035	4.300	3109	WS00823_H10
4	173.929	88	2	-0.258	0.085	5.564	3109	WS00823_H10
9	36.227	165	3	-0.185	0.037	4.376	3109	WS00823_H10
14	0.000	246	1	-0.236	0.036	4.446	3109	WS00823_H10
5	57.017	102	2	0.250	0.061	6.095	3110	WS00823_N01
9	59.963	177	4	-0.188	0.048	3.864	3110	WS00823_N01
12	22.441	227	1	-0.270	0.067	5.889	3110	WS00823_N01
12	38.543	232	4	0.276	0.136	4.505	3110	WS00823_N01
1	123.316	20	4	0.300	0.054	4.990	3111	WS00822_N01
1	127.805	21	4	-0.161	0.038	4.601	3111	WS00822_N01
3	148.745	68	1	-0.224	0.055	3.859	3111	WS00822_N01
6	81.996	123	3	0.324	0.138	5.464	3111	WS00822_N01
11	27.405	204	4	-0.371	0.072	6.417	3111	WS00822_N01
12	6.242	234	2	-0.221	0.035	4.842	3111	WS00822_N01

14	22.402	248	4	0.180	0.032	3.949	3111	WS00822_N01
1	123.316	20	4	0.301	0.154	6.811	3112	WS00823_B08
1	133.992	24	2	0.231	0.046	4.974	3112	WS00823_B08
2	63.261	30	3	-0.278	0.041	5.031	3112	WS00823_B08
4	75.958	77	3	-0.242	0.086	5.470	3112	WS00823_B08
5	0.000	89	3	0.254	0.039	4.965	3112	WS00823_B08
6	95.249	126	1	-0.403	0.121	6.850	3112	WS00823_B08
7	17.610	131	3	0.244	0.084	5.299	3112	WS00823_B08
9	28.608	170	2	0.238	0.043	4.755	3112	WS00823_B08
11	0.000	199	2	-0.256	0.035	3.872	3112	WS00823_B08
11	0.000	199	3	0.278	0.034	4.226	3112	WS00823_B08
11	17.088	203	1	0.356	0.144	11.517	3112	WS00823_B08
13	1.815	244	4	0.181	0.065	4.334	3112	WS00823_B08
14	22.402	248	3	-0.213	0.047	5.596	3112	WS00823_B08
1	15.589	3	2	0.215	0.041	5.999	3113	WS00916_I07
1	29.800	5	2	-0.180	0.029	4.325	3113	WS00916_I07
1	62.128	11	2	0.261	0.062	4.021	3113	WS00916_I07
2	102.814	36	2	-0.212	0.033	4.303	3113	WS00916_I07
4	116.928	82	2	-0.208	0.036	5.271	3113	WS00916_I07
5	7.672	92	4	0.330	0.099	5.858	3113	WS00916_I07
9	0.000	166	2	0.182	0.039	5.768	3113	WS00916_I07
9	17.951	169	2	-0.217	0.036	5.655	3113	WS00916_I07
10	78.479	193	3	-0.356	0.082	9.398	3113	WS00916_I07
11	27.405	204	4	-0.271	0.077	5.481	3113	WS00916_I07
1	43.578	8	2	-0.180	0.036	4.531	3114	WS00916_N09
8	39.090	152	4	-0.231	0.044	4.552	3114	WS00916_N09
10	95.795	197	4	-0.227	0.042	4.328	3114	WS00916_N09
11	27.405	204	4	-0.371	0.068	6.299	3114	WS00916_N09
1	131.686	23	2	-0.370	0.040	4.178	3115	WS00825_N13
1	133.992	24	2	-0.191	0.031	4.049	3115	WS00825_N13
3	116.958	63	4	-0.173	0.037	4.606	3115	WS00825_N13
4	173.929	88	2	-0.194	0.038	4.964	3115	WS00825_N13
11	27.405	204	4	-0.238	0.031	4.303	3115	WS00825_N13
11	60.464	221	2	0.405	0.097	12.442	3115	WS00825_N13
12	6.242	234	4	0.379	0.054	5.686	3115	WS00825_N13
1	62.128	11	1	-0.511	0.121	5.079	3116	WS00825_O05
2	102.814	36	2	0.253	0.068	5.505	3116	WS00825_O05
3	137.891	64	1	0.333	0.056	5.778	3116	WS00825_O05
3	144.647	67	1	0.278	0.098	4.048	3116	WS00825_O05
4	43.146	74	3	-0.200	0.036	3.856	3116	WS00825_O05
4	116.928	82	2	0.277	0.067	5.885	3116	WS00825_O05
4	124.132	83	2	0.332	0.167	6.684	3116	WS00825_O05
6	54.066	119	4	0.248	0.069	5.656	3116	WS00825_O05
6	81.996	123	3	0.312	0.079	4.664	3116	WS00825_O05
6	81.996	123	4	-0.472	0.154	6.415	3116	WS00825_O05
7	62.787	139	4	0.231	0.051	4.637	3116	WS00825_O05
9	58.045	176	1	-0.305	0.089	5.219	3116	WS00825_O05
9	59.963	177	1	-0.285	0.045	4.479	3116	WS00825_O05
11	64.014	222	4	-0.321	0.075	4.641	3116	WS00825_O05
13	0.000	243	4	0.367	0.153	6.366	3116	WS00825_O05
1	127.805	21	4	-0.250	0.052	4.725	3117	WS0051_A23
6	81.996	123	3	0.427	0.140	7.364	3117	WS0051_A23
6	89.401	125	1	0.193	0.050	3.880	3117	WS0051_A23
6	95.249	126	1	-0.420	0.149	7.827	3117	WS0051_A23

13	1.815	244	4	0.218	0.040	4.014	3117	WS0051_A23
1	69.854	13	2	0.211	0.053	5.763	3118	WS0051_C01
1	71.592	14	2	0.196	0.047	5.055	3118	WS0051_C01
1	77.177	15	2	0.192	0.044	4.790	3118	WS0051_C01
3	17.158	45	1	0.211	0.050	3.970	3118	WS0051_C01
4	173.929	88	2	-0.238	0.057	6.193	3118	WS0051_C01
6	81.996	123	3	0.401	0.152	6.763	3118	WS0051_C01
8	50.001	154	1	-0.228	0.049	4.166	3118	WS0051_C01
14	0.000	246	2	-0.244	0.068	6.367	3118	WS0051_C01
3	80.211	55	4	-0.119	0.036	3.846	3119	WS0051_F15
4	116.928	82	2	0.273	0.036	4.208	3119	WS0051_F15
4	124.132	83	1	-0.641	0.222	8.295	3119	WS0051_F15
4	173.929	88	2	-0.245	0.060	6.739	3119	WS0051_F15
5	57.017	102	2	-0.247	0.036	4.217	3119	WS0051_F15
5	86.669	106	4	-0.311	0.172	7.016	3119	WS0051_F15
6	87.891	124	2	0.244	0.057	6.466	3119	WS0051_F15
6	89.401	125	2	0.353	0.073	7.522	3119	WS0051_F15
9	59.963	177	1	-0.277	0.056	4.572	3119	WS0051_F15
11	17.088	203	1	0.231	0.056	4.157	3119	WS0051_F15
1	95.572	17	3	0.196	0.033	3.991	3120	WS0051_H11
5	7.672	92	1	-0.281	0.109	7.608	3120	WS0051_H11
6	25.914	114	1	-0.267	0.058	3.918	3120	WS0051_H11
7	17.610	131	3	0.196	0.066	5.101	3120	WS0051_H11
8	50.001	154	2	-0.244	0.045	5.105	3120	WS0051_H11
11	46.026	217	1	-0.205	0.074	4.896	3120	WS0051_H11
12	6.242	234	2	0.309	0.099	12.976	3120	WS0051_H11
1	29.800	5	1	-0.344	0.059	6.252	3121	WS0051_L24
6	49.100	118	2	-0.313	0.111	4.575	3121	WS0051_L24
6	56.188	120	2	-0.233	0.048	3.869	3121	WS0051_L24
7	91.487	146	3	-0.169	0.034	4.192	3121	WS0051_L24
8	78.389	157	1	0.235	0.041	4.356	3121	WS0051_L24
10	93.347	196	1	0.534	0.084	6.872	3121	WS0051_L24
14	0.000	246	1	-0.245	0.043	4.555	3121	WS0051_L24
1	29.800	5	1	-0.249	0.042	4.803	3122	WS0051_O14
1	127.805	21	4	-0.274	0.060	4.381	3122	WS0051_O14
3	141.443	65	3	-0.346	0.074	6.341	3122	WS0051_O14
10	93.347	196	1	0.505	0.071	6.174	3122	WS0051_O14
12	14.406	230	4	0.275	0.058	4.285	3122	WS0051_O14
1	82.301	16	4	0.311	0.085	5.728	3123	WS0075_E20
4	75.958	77	3	0.223	0.111	4.216	3123	WS0075_E20
14	0.000	246	1	-0.259	0.039	4.146	3123	WS0075_E20
1	2.788	2	4	-0.124	0.051	4.352	3124	WS0075_F11
3	154.505	69	1	0.227	0.036	4.017	3124	WS0075_F11
5	54.072	100	4	0.238	0.054	4.265	3124	WS0075_F11
5	57.017	102	4	-0.203	0.056	4.429	3124	WS0075_F11
7	105.477	147	2	-0.202	0.104	5.202	3124	WS0075_F11
9	0.000	166	1	-0.232	0.039	4.332	3124	WS0075_F11
9	28.608	170	3	0.252	0.048	5.651	3124	WS0075_F11
11	43.491	214	4	-0.203	0.066	5.104	3124	WS0075_F11
12	22.629	237	1	-0.320	0.060	6.750	3124	WS0075_F11
12	32.473	238	1	-0.277	0.044	4.998	3124	WS0075_F11
14	0.000	246	1	-0.173	0.044	4.775	3124	WS0075_F11
1	29.800	5	1	-0.271	0.044	5.085	3125	WS0075_I06
6	69.073	121	3	0.199	0.035	4.261	3125	WS0075_I06

8	62.580	155	1	0.332	0.158	4.782	3125	WS0075_I06
1	40.464	7	4	0.239	0.049	4.424	3126	WS0075_I20
1	131.686	23	2	0.523	0.107	6.666	3126	WS0075_I20
3	9.953	42	3	-0.234	0.071	7.175	3126	WS0075_I20
3	83.563	56	3	0.176	0.043	4.322	3126	WS0075_I20
8	62.580	155	1	0.344	0.170	4.003	3126	WS0075_I20
8	78.389	157	1	0.282	0.075	5.399	3126	WS0075_I20
1	45.794	9	1	0.323	0.089	5.573	3127	WS0075_K15
2	48.839	29	4	-0.285	0.081	6.373	3127	WS0075_K15
3	9.953	42	3	-0.220	0.048	4.487	3127	WS0075_K15
4	82.462	78	2	-0.189	0.038	4.696	3127	WS0075_K15
4	75.958	77	3	0.255	0.107	4.125	3128	WS0075_L05
10	93.347	196	1	0.416	0.057	4.255	3128	WS0075_L05
14	0.000	246	1	-0.359	0.097	10.168	3128	WS0075_L05
3	9.953	42	3	0.231	0.045	4.226	3129	WS0075_N06
3	15.147	44	2	0.219	0.056	3.848	3129	WS0075_N06
4	173.929	88	2	-0.221	0.054	5.911	3129	WS0075_N06
6	81.996	123	3	0.325	0.092	5.176	3129	WS0075_N06
10	9.580	187	3	0.295	0.072	4.303	3129	WS0075_N06
11	27.405	204	4	-0.293	0.047	4.298	3129	WS0075_N06
12	6.242	234	2	-0.192	0.036	4.479	3129	WS0075_N06
1	43.578	8	2	-0.204	0.042	4.340	3130	WS0075_N19
3	15.147	44	2	0.290	0.074	5.515	3130	WS0075_N19
3	148.745	68	1	-0.295	0.053	4.569	3130	WS0075_N19
6	81.996	123	3	0.397	0.121	8.152	3130	WS0075_N19
8	50.001	154	1	-0.210	0.043	3.920	3130	WS0075_N19
8	87.463	158	2	-0.260	0.069	4.040	3130	WS0075_N19
10	93.347	196	1	0.509	0.102	6.901	3130	WS0075_N19
11	27.405	204	1	0.174	0.044	3.906	3130	WS0075_N19
11	27.405	204	4	-0.305	0.059	5.844	3130	WS0075_N19
14	22.402	248	4	0.180	0.041	4.302	3130	WS0075_N19
1	69.854	13	2	0.204	0.038	4.487	3131	WS00820_B01
1	71.592	14	2	0.199	0.037	4.317	3131	WS00820_B01
1	77.177	15	2	0.210	0.040	4.754	3131	WS00820_B01
3	15.147	44	2	0.236	0.060	4.359	3131	WS00820_B01
4	75.958	77	3	0.301	0.133	5.534	3131	WS00820_B01
4	142.917	85	1	0.226	0.059	4.111	3131	WS00820_B01
4	173.929	88	2	-0.203	0.049	5.800	3131	WS00820_B01
7	15.555	130	2	-0.283	0.046	5.450	3131	WS00820_B01
10	78.479	193	3	0.257	0.051	4.222	3131	WS00820_B01
12	6.242	234	2	-0.203	0.036	4.940	3131	WS00820_B01
1	69.854	13	2	0.200	0.035	4.121	3132	WS00820_B13
1	71.592	14	2	0.198	0.035	4.067	3132	WS00820_B13
1	77.177	15	2	0.210	0.038	4.510	3132	WS00820_B13
6	81.996	123	3	0.343	0.142	5.909	3132	WS00820_B13
11	27.405	204	4	-0.278	0.037	4.132	3132	WS00820_B13
4	173.929	88	2	-0.191	0.038	4.739	3133	WS00820_D17
5	54.072	100	4	0.199	0.039	4.741	3133	WS00820_D17
5	57.017	102	2	-0.180	0.033	3.990	3133	WS00820_D17
6	81.996	123	3	0.327	0.125	4.796	3133	WS00820_D17
11	27.405	204	4	-0.362	0.075	8.034	3133	WS00820_D17
13	1.815	244	4	0.234	0.043	5.058	3133	WS00820_D17
3	15.147	44	2	0.248	0.050	4.764	3134	WS00820_E02
3	116.958	63	4	-0.178	0.041	4.801	3134	WS00820_E02

4	173.929	88	2	-0.203	0.038	5.112	3134	WS00820_E02
5	54.072	100	4	0.250	0.049	5.796	3134	WS00820_E02
8	87.463	158	2	-0.192	0.030	3.856	3134	WS00820_E02
11	27.405	204	4	-0.313	0.050	5.326	3134	WS00820_E02
3	15.147	44	2	0.362	0.084	6.198	3135	WS00820_G03
4	116.928	82	2	0.272	0.040	5.351	3135	WS00820_G03
4	173.929	88	2	-0.216	0.047	6.169	3135	WS00820_G03
5	57.017	102	2	-0.212	0.031	4.042	3135	WS00820_G03
8	50.001	154	2	-0.195	0.035	4.177	3135	WS00820_G03
10	80.088	194	2	0.216	0.029	4.121	3135	WS00820_G03
11	27.405	204	4	-0.256	0.045	4.834	3135	WS00820_G03
6	69.073	121	3	0.225	0.046	4.385	3136	WS00820_G16
6	87.891	124	2	0.184	0.055	4.167	3136	WS00820_G16
6	95.249	126	1	-0.340	0.055	4.229	3136	WS00820_G16
9	69.046	181	1	0.412	0.077	5.909	3136	WS00820_G16
11	17.088	203	1	0.332	0.088	9.918	3136	WS00820_G16
11	27.405	204	1	-0.327	0.038	4.425	3136	WS00820_G16
11	29.069	205	1	-0.391	0.078	9.029	3136	WS00820_G16
11	45.989	216	1	0.255	0.066	7.388	3136	WS00820_G16
11	60.464	221	2	0.309	0.075	5.785	3136	WS00820_G16
3	15.147	44	2	0.309	0.058	4.345	3137	WS00820_I04
5	54.072	100	4	0.231	0.061	6.249	3137	WS00820_I04
6	56.188	120	2	-0.250	0.032	4.161	3137	WS00820_I04
12	6.242	234	2	-0.202	0.032	4.165	3137	WS00820_I04
2	48.839	29	1	-0.192	0.056	4.519	3138	WS00820_I21
3	20.512	46	2	0.216	0.064	6.727	3138	WS00820_I21
9	36.227	165	4	0.265	0.045	4.523	3138	WS00820_I21
13	3.386	245	1	0.196	0.062	5.081	3138	WS00820_I21
1	29.800	5	2	-0.250	0.062	6.784	3139	WS00832_N09
1	127.805	21	3	0.176	0.041	4.049	3139	WS00832_N09
3	141.443	65	3	-0.393	0.109	5.009	3139	WS00832_N09
3	154.505	69	1	-0.383	0.067	5.406	3139	WS00832_N09
5	86.669	106	2	-0.323	0.074	4.198	3139	WS00832_N09
7	15.555	130	2	0.286	0.060	6.825	3139	WS00832_N09
7	17.610	131	3	0.303	0.128	6.920	3139	WS00832_N09
8	50.001	154	2	-0.190	0.044	4.573	3139	WS00832_N09
9	36.227	165	3	0.288	0.043	4.355	3139	WS00832_N09
10	104.214	198	2	-0.184	0.024	3.892	3139	WS00832_N09
12	6.242	234	2	0.237	0.051	5.605	3139	WS00832_N09
14	46.637	252	4	-0.424	0.085	8.007	3139	WS00832_N09
1	15.589	3	2	-0.210	0.039	4.485	3140	WS00832_O15
2	48.839	29	1	-0.174	0.054	3.971	3140	WS00832_O15
2	63.261	30	3	0.382	0.149	15.206	3140	WS00832_O15
2	75.550	31	1	0.407	0.178	12.932	3140	WS00832_O15
2	75.550	31	2	0.178	0.037	4.251	3140	WS00832_O15
2	93.504	32	4	0.289	0.074	6.415	3140	WS00832_O15
4	75.958	77	3	0.276	0.101	5.183	3140	WS00832_O15
4	92.039	79	2	-0.241	0.033	3.884	3140	WS00832_O15
4	116.928	82	2	0.302	0.071	8.260	3140	WS00832_O15
4	142.917	85	1	0.230	0.058	4.343	3140	WS00832_O15
4	173.929	88	2	-0.206	0.036	4.100	3140	WS00832_O15
5	54.072	100	4	0.182	0.048	4.076	3140	WS00832_O15
5	57.017	102	2	-0.234	0.049	5.555	3140	WS00832_O15
6	56.188	120	2	-0.284	0.055	6.381	3140	WS00832_O15

6	106.702	128	2	-0.421	0.087	4.788	3140	WS00832_O15
10	93.347	196	2	-0.467	0.097	4.267	3140	WS00832_O15
4	75.958	77	3	0.272	0.112	4.862	3141	WS00833_D22
4	173.929	88	2	-0.241	0.049	5.632	3141	WS00833_D22
5	54.072	100	4	0.211	0.049	5.055	3141	WS00833_D22
8	50.001	154	1	-0.240	0.052	4.148	3141	WS00833_D22
12	6.242	234	2	-0.203	0.032	3.931	3141	WS00833_D22
1	95.572	17	3	-0.257	0.064	4.176	3142	WS00833_F14
3	15.147	44	2	0.284	0.089	5.367	3142	WS00833_F14
5	54.072	100	4	0.275	0.072	6.717	3142	WS00833_F14
5	86.669	106	2	-0.247	0.082	4.979	3142	WS00833_F14
5	86.669	106	4	0.316	0.111	4.041	3142	WS00833_F14
9	2.011	167	4	-0.216	0.047	4.408	3142	WS00833_F14
9	59.963	177	4	0.301	0.082	7.598	3142	WS00833_F14
10	70.246	190	2	-0.219	0.046	5.346	3142	WS00833_F14
10	93.347	196	2	-0.486	0.091	5.397	3142	WS00833_F14
10	104.214	198	4	-0.235	0.040	4.092	3142	WS00833_F14
1	62.128	11	1	-0.453	0.081	3.907	3143	WS00833_M01
1	69.854	13	2	0.160	0.038	3.960	3143	WS00833_M01
1	77.177	15	2	0.164	0.039	4.111	3143	WS00833_M01
3	114.747	62	2	-0.175	0.038	3.967	3143	WS00833_M01
7	75.883	144	4	0.293	0.053	4.215	3143	WS00833_M01
9	59.963	177	4	0.251	0.043	3.884	3143	WS00833_M01
11	64.014	222	4	-0.277	0.050	4.575	3143	WS00833_M01
13	0.000	243	4	0.343	0.124	5.876	3143	WS00833_M01
4	116.928	82	4	-0.483	0.132	11.822	3144	WS00833_N11
7	17.610	131	3	0.283	0.113	4.555	3144	WS00833_N11
9	59.963	177	4	0.213	0.040	3.932	3144	WS00833_N11
11	43.491	214	3	0.222	0.060	5.369	3144	WS00833_N11
11	44.884	215	3	-0.213	0.053	4.742	3144	WS00833_N11
11	46.026	217	1	-0.203	0.034	3.849	3144	WS00833_N11
11	56.921	219	2	-0.361	0.141	5.356	3144	WS00833_N11
11	64.014	222	3	-0.229	0.059	5.293	3144	WS00833_N11
3	14.500	43	1	-0.298	0.054	5.209	3145	WS00938_A20
3	17.158	45	1	0.210	0.061	5.878	3145	WS00938_A20
4	75.958	77	3	0.247	0.122	4.773	3145	WS00938_A20
2	48.839	29	4	-0.290	0.078	7.187	3146	WS00938_B16
3	154.505	69	1	0.211	0.065	4.718	3146	WS00938_B16
3	154.505	69	4	-0.222	0.039	3.943	3146	WS00938_B16
6	25.914	114	1	0.235	0.057	4.102	3146	WS00938_B16
11	17.088	203	1	0.308	0.100	7.142	3146	WS00938_B16
11	27.405	204	1	-0.313	0.086	6.350	3146	WS00938_B16
11	27.405	204	4	0.252	0.051	5.105	3146	WS00938_B16
11	29.069	205	1	-0.241	0.060	4.310	3146	WS00938_B16
11	44.884	215	3	-0.270	0.068	5.871	3146	WS00938_B16
11	64.014	222	3	-0.281	0.083	7.243	3146	WS00938_B16
12	22.441	227	1	-0.298	0.073	5.460	3146	WS00938_B16
14	0.000	246	2	0.201	0.039	4.354	3146	WS00938_B16
1	29.800	5	1	-0.241	0.090	6.976	3147	WS0101_E11
3	148.745	68	4	0.274	0.040	4.173	3147	WS0101_E11
6	106.702	128	3	0.340	0.047	4.548	3147	WS0101_E11
7	15.555	130	2	0.258	0.035	4.220	3147	WS0101_E11
7	17.610	131	3	0.270	0.089	4.055	3147	WS0101_E11
7	62.787	139	4	-0.228	0.052	5.183	3147	WS0101_E11

7	70.278	141	3	0.317	0.076	7.645	3147	WS0101_E11
7	91.487	146	3	0.296	0.062	6.261	3147	WS0101_E11
10	78.479	193	3	-0.275	0.052	5.404	3147	WS0101_E11
10	78.479	193	4	-0.322	0.046	4.252	3147	WS0101_E11
12	6.242	234	2	0.224	0.036	4.986	3147	WS0101_E11
14	0.000	246	2	0.234	0.043	4.917	3147	WS0101_E11
2	48.839	29	1	0.286	0.044	5.788	3148	WS0101_J14
2	48.839	29	4	-0.271	0.075	4.872	3148	WS0101_J14
2	126.181	39	2	-0.265	0.071	3.963	3148	WS0101_J14
3	14.500	43	1	-0.259	0.037	4.886	3148	WS0101_J14
3	141.443	65	3	-0.301	0.064	6.543	3148	WS0101_J14
7	15.555	130	3	-0.270	0.038	3.850	3148	WS0101_J14
9	0.000	166	1	-0.241	0.037	4.928	3148	WS0101_J14
9	28.608	170	3	0.238	0.055	6.908	3148	WS0101_J14
10	93.347	196	1	0.471	0.057	5.915	3148	WS0101_J14
1	141.021	25	2	0.189	0.038	4.049	3149	WS0079_I20
2	48.839	29	4	0.193	0.039	4.029	3149	WS0079_I20
4	82.462	78	1	-0.249	0.062	5.110	3149	WS0079_I20
8	50.001	154	1	-0.209	0.055	5.367	3149	WS0079_I20
8	50.001	154	2	-0.233	0.057	6.033	3149	WS0079_I20
1	77.177	15	2	0.216	0.042	4.046	3150	WS00938_H23
1	95.572	17	2	0.329	0.073	7.091	3150	WS00938_H23
3	112.868	61	2	-0.199	0.043	4.101	3150	WS00938_H23
3	114.747	62	2	-0.195	0.043	4.177	3150	WS00938_H23
6	25.742	113	3	-0.187	0.041	4.003	3150	WS00938_H23
6	25.914	114	1	0.181	0.051	4.612	3150	WS00938_H23
7	74.560	143	1	0.260	0.048	4.418	3150	WS00938_H23
7	75.883	144	2	-0.390	0.064	4.225	3150	WS00938_H23
8	50.001	154	1	-0.176	0.050	4.598	3150	WS00938_H23
9	49.013	173	1	0.264	0.050	4.420	3150	WS00938_H23
9	81.073	183	1	0.263	0.057	5.236	3150	WS00938_H23
10	9.580	187	3	0.380	0.145	7.636	3150	WS00938_H23
11	42.600	213	3	0.183	0.039	3.892	3150	WS00938_H23
11	56.921	219	1	-0.404	0.147	4.146	3150	WS00938_H23
11	57.661	220	4	0.168	0.048	4.286	3150	WS00938_H23
14	0.000	246	1	0.307	0.065	6.075	3150	WS00938_H23
1	69.854	13	2	0.162	0.043	4.491	3151	WS0079_P07
1	71.592	14	2	0.148	0.037	3.887	3151	WS0079_P07
4	173.929	88	2	-0.238	0.049	5.137	3151	WS0079_P07
8	50.001	154	1	-0.288	0.063	6.123	3151	WS0079_P07
2	48.839	29	4	-0.266	0.058	5.909	3152	WS00939_B18
2	63.261	30	3	-0.246	0.050	4.547	3152	WS00939_B18
3	137.891	64	3	-0.266	0.059	5.481	3152	WS00939_B18
3	144.593	66	4	-0.277	0.054	5.183	3152	WS00939_B18
3	148.745	68	4	0.290	0.072	6.929	3152	WS00939_B18
9	69.046	181	1	0.396	0.144	5.865	3152	WS00939_B18
11	40.960	210	2	0.430	0.086	6.334	3152	WS00939_B18
1	69.854	13	2	-0.247	0.060	6.601	3153	WS00813_A06
1	71.592	14	2	-0.249	0.061	6.766	3153	WS00813_A06
1	77.177	15	2	-0.242	0.057	6.273	3153	WS00813_A06
10	9.580	187	2	0.236	0.056	3.872	3153	WS00813_A06
10	35.204	189	2	-0.221	0.034	3.841	3153	WS00813_A06
11	42.600	213	3	0.221	0.055	4.074	3153	WS00813_A06
12	6.242	234	2	0.196	0.032	4.946	3153	WS00813_A06

14	9.923	247	1	-0.217	0.059	4.583	3153	WS00813_A06
14	22.402	248	3	-0.281	0.066	5.019	3153	WS00813_A06
4	82.462	78	1	-0.212	0.059	3.965	3154	WS00813_B07
4	173.929	88	2	-0.279	0.060	7.194	3154	WS00813_B07
5	3.484	90	2	-0.284	0.069	4.862	3154	WS00813_B07
5	7.672	92	4	0.289	0.061	5.280	3154	WS00813_B07
5	57.017	102	4	-0.297	0.054	5.303	3154	WS00813_B07
6	81.996	123	3	0.363	0.131	4.568	3154	WS00813_B07
9	17.951	169	2	-0.330	0.070	7.345	3154	WS00813_B07
9	28.608	170	2	0.238	0.038	4.547	3154	WS00813_B07
10	78.479	193	3	0.239	0.056	4.722	3154	WS00813_B07
11	64.014	222	3	0.196	0.055	4.655	3154	WS00813_B07
3	15.147	44	2	0.360	0.120	8.538	3155	WS00813_E10
3	116.958	63	4	-0.152	0.039	3.948	3155	WS00813_E10
4	173.929	88	2	-0.199	0.039	4.413	3155	WS00813_E10
5	54.072	100	4	0.200	0.041	4.161	3155	WS00813_E10
6	56.188	120	2	-0.270	0.040	4.620	3155	WS00813_E10
7	15.555	130	2	-0.290	0.048	5.445	3155	WS00813_E10
8	50.001	154	1	-0.238	0.063	5.359	3155	WS00813_E10
10	70.246	190	2	-0.225	0.048	5.586	3155	WS00813_E10
10	78.479	193	3	0.233	0.052	4.434	3155	WS00813_E10
12	6.242	234	2	-0.180	0.033	4.141	3155	WS00813_E10
1	69.854	13	2	0.240	0.039	4.261	3156	WS00813_E22
1	71.592	14	2	0.236	0.037	4.084	3156	WS00813_E22
3	15.147	44	2	0.280	0.107	6.921	3156	WS00813_E22
3	47.928	49	1	-0.140	0.057	4.638	3156	WS00813_E22
3	95.157	59	2	-0.243	0.028	3.987	3156	WS00813_E22
3	112.868	61	2	-0.192	0.045	4.789	3156	WS00813_E22
3	114.747	62	2	-0.246	0.054	5.714	3156	WS00813_E22
4	75.958	77	3	0.337	0.097	4.047	3156	WS00813_E22
10	78.479	193	3	0.345	0.054	5.119	3156	WS00813_E22
11	27.405	204	1	0.262	0.065	5.678	3156	WS00813_E22
11	48.979	218	2	0.158	0.039	4.288	3156	WS00813_E22
11	64.014	222	4	-0.219	0.060	5.832	3156	WS00813_E22
12	6.242	234	2	-0.236	0.031	4.488	3156	WS00813_E22
14	0.000	246	2	-0.223	0.036	4.777	3156	WS00813_E22
1	45.794	9	1	-0.273	0.092	7.090	3157	WS00813_I07
1	50.955	10	1	0.172	0.058	4.453	3157	WS00813_I07
2	126.181	39	2	-0.311	0.052	5.873	3157	WS00813_I07
2	126.181	39	4	0.353	0.066	3.876	3157	WS00813_I07
3	137.891	64	1	0.214	0.060	4.824	3157	WS00813_I07
5	52.820	99	1	-0.215	0.065	5.141	3157	WS00813_I07
11	60.464	221	2	0.416	0.095	10.098	3157	WS00813_I07
3	15.147	44	2	0.309	0.056	5.336	3158	WS00813_I23
6	56.188	120	2	-0.314	0.037	4.803	3158	WS00813_I23
7	70.278	141	3	-0.206	0.051	4.017	3158	WS00813_I23
11	27.405	204	4	-0.257	0.048	5.524	3158	WS00813_I23
12	6.242	234	2	-0.159	0.028	3.892	3158	WS00813_I23
14	0.000	246	2	-0.138	0.030	4.311	3158	WS00813_I23
1	95.572	17	2	0.275	0.049	5.851	3159	WS00813_N08
1	123.316	20	4	0.347	0.102	8.397	3159	WS00813_N08
1	127.805	21	4	-0.218	0.038	4.128	3159	WS00813_N08
6	81.996	123	4	-0.336	0.144	4.435	3159	WS00813_N08
6	106.702	128	2	0.425	0.111	4.506	3159	WS00813_N08

7	62.787	139	4	0.318	0.057	5.901	3159	WS00813_N08
10	80.088	194	2	0.226	0.035	4.207	3159	WS00813_N08
10	95.795	197	4	-0.218	0.036	4.143	3159	WS00813_N08
11	57.661	220	4	0.293	0.061	7.156	3159	WS00813_N08
11	64.014	222	4	-0.380	0.066	4.764	3159	WS00813_N08
12	28.493	228	2	-0.303	0.066	5.478	3159	WS00813_N08
13	0.000	243	4	0.470	0.206	6.920	3159	WS00813_N08
14	0.000	246	2	-0.219	0.053	4.946	3159	WS00813_N08
1	107.333	19	3	-0.231	0.042	4.668	3160	WS00813_O05
4	75.958	77	3	0.330	0.094	6.386	3160	WS00813_O05
4	116.928	82	2	0.279	0.057	6.131	3160	WS00813_O05
4	173.929	88	2	-0.266	0.068	7.232	3160	WS00813_O05
5	0.000	89	3	-0.315	0.061	6.838	3160	WS00813_O05
6	25.914	114	1	0.227	0.047	3.863	3160	WS00813_O05
10	78.479	193	3	0.415	0.084	9.298	3160	WS00813_O05
10	93.347	196	2	-0.501	0.084	5.588	3160	WS00813_O05
13	1.815	244	2	-0.257	0.046	4.888	3160	WS00813_O05
2	101.076	35	4	0.188	0.025	4.078	3161	WS00918_J13
7	75.883	144	2	0.447	0.119	6.834	3161	WS00918_J13
11	45.989	216	1	-0.301	0.062	5.935	3161	WS00918_J13
11	64.014	222	3	0.256	0.064	4.836	3161	WS00918_J13
14	22.402	248	4	0.390	0.041	4.764	3161	WS00918_J13
4	92.039	79	4	-0.304	0.043	3.870	3162	WS00918_K07
4	124.132	83	1	0.492	0.096	5.324	3162	WS00918_K07
4	142.917	85	1	-0.202	0.038	3.861	3162	WS00918_K07
6	81.996	123	3	0.231	0.085	4.098	3162	WS00918_K07
8	50.001	154	1	-0.214	0.041	4.117	3162	WS00918_K07
1	29.800	5	1	-0.197	0.047	4.635	3163	WS00918_M09
3	141.443	65	3	-0.299	0.056	3.876	3163	WS00918_M09
4	82.462	78	1	-0.152	0.059	5.819	3163	WS00918_M09
4	103.719	81	4	-0.277	0.052	4.753	3163	WS00918_M09
4	142.917	85	1	0.299	0.091	9.063	3163	WS00918_M09
8	0.000	148	4	-0.266	0.041	3.851	3163	WS00918_M09
8	87.463	158	2	0.221	0.048	4.025	3163	WS00918_M09
9	36.227	165	3	0.289	0.041	4.193	3163	WS00918_M09
11	45.989	216	1	0.262	0.073	7.255	3163	WS00918_M09
12	22.441	227	1	0.233	0.060	6.087	3163	WS00918_M09
12	28.493	228	2	-0.317	0.077	4.270	3163	WS00918_M09
1	29.800	5	1	-0.271	0.069	4.509	3164	WS00918_N02
1	133.992	24	2	0.184	0.044	5.390	3164	WS00918_N02
3	116.958	63	4	0.184	0.049	5.635	3164	WS00918_N02
3	154.505	69	4	-0.312	0.046	4.367	3164	WS00918_N02
6	25.742	113	3	0.185	0.058	4.166	3164	WS00918_N02
6	45.897	117	4	0.222	0.045	5.171	3164	WS00918_N02
6	89.401	125	1	0.174	0.061	4.033	3164	WS00918_N02
9	36.227	165	4	-0.217	0.042	4.881	3164	WS00918_N02
10	70.246	190	4	0.179	0.040	4.594	3164	WS00918_N02
11	9.237	202	4	0.195	0.041	4.751	3164	WS00918_N02
11	27.405	204	4	-0.248	0.044	4.854	3164	WS00918_N02
11	29.069	205	2	0.256	0.051	5.978	3164	WS00918_N02
11	40.960	210	2	0.342	0.047	3.875	3164	WS00918_N02
5	56.008	101	2	0.195	0.052	5.317	3165	WS00918_O04
5	57.017	102	2	-0.236	0.061	6.341	3165	WS00918_O04
6	69.073	121	3	0.202	0.047	4.092	3165	WS00918_O04

6	89.401	125	2	0.340	0.084	7.362	3165	WS00918_O04
6	106.702	128	2	-0.440	0.113	5.361	3165	WS00918_O04
9	28.608	170	2	0.375	0.096	10.109	3165	WS00918_O04
9	32.356	171	2	0.235	0.045	4.658	3165	WS00918_O04
10	93.347	196	1	0.408	0.095	3.992	3165	WS00918_O04
11	27.405	204	1	-0.429	0.129	11.123	3165	WS00918_O04
11	29.069	205	1	-0.327	0.088	7.490	3165	WS00918_O04
11	35.604	207	3	0.188	0.061	5.346	3165	WS00918_O04
11	37.143	209	3	-0.169	0.051	4.474	3165	WS00918_O04
13	0.000	243	4	0.393	0.124	5.329	3165	WS00918_O04
13	1.815	244	2	-0.294	0.077	8.140	3165	WS00918_O04
13	1.815	244	4	0.359	0.081	8.296	3165	WS00918_O04
14	44.898	251	1	-0.496	0.123	10.449	3165	WS00918_O04
1	50.955	10	1	-0.201	0.040	4.258	3166	WS00918_O12
10	9.580	187	3	-0.453	0.126	8.067	3166	WS00918_O12
10	104.214	198	4	0.206	0.028	4.331	3166	WS00918_O12
12	6.242	234	2	-0.156	0.036	4.776	3166	WS00918_O12
13	3.386	245	1	-0.231	0.037	3.972	3166	WS00918_O12
14	22.402	248	3	0.214	0.036	3.875	3166	WS00918_O12
1	45.794	9	1	0.257	0.049	6.166	3167	WS01010_A08
3	14.500	43	1	-0.300	0.035	4.353	3167	WS01010_A08
3	17.158	45	1	0.197	0.046	5.594	3167	WS01010_A08
3	137.891	64	1	-0.210	0.037	4.593	3167	WS01010_A08
3	141.443	65	3	-0.355	0.066	6.327	3167	WS01010_A08
4	142.917	85	1	0.267	0.044	5.403	3167	WS01010_A08
6	89.401	125	2	0.269	0.078	4.624	3167	WS01010_A08
6	95.249	126	1	-0.320	0.044	4.284	3167	WS01010_A08
6	106.702	128	2	-0.493	0.173	6.397	3167	WS01010_A08
6	106.702	128	3	0.285	0.037	4.845	3167	WS01010_A08
12	6.242	234	2	-0.240	0.079	5.508	3167	WS01010_A08
12	22.629	237	1	-0.212	0.050	6.087	3167	WS01010_A08
2	63.261	30	3	0.228	0.043	4.531	3168	WS01010_B03
4	124.132	83	1	0.493	0.112	4.984	3168	WS01010_B03
4	142.917	85	1	-0.285	0.082	7.579	3168	WS01010_B03
5	16.219	93	2	0.299	0.049	4.240	3168	WS01010_B03
6	25.742	113	3	-0.232	0.047	4.935	3168	WS01010_B03
6	25.914	114	1	-0.266	0.067	6.143	3168	WS01010_B03
6	106.702	128	2	0.425	0.086	5.225	3168	WS01010_B03
9	0.000	166	1	-0.167	0.059	5.329	3168	WS01010_B03
9	28.608	170	3	0.279	0.036	4.312	3168	WS01010_B03
9	69.046	181	2	0.309	0.073	4.714	3168	WS01010_B03
10	9.580	187	3	-0.473	0.178	7.521	3168	WS01010_B03
10	35.204	189	1	-0.428	0.103	9.584	3168	WS01010_B03
10	77.676	192	1	-0.173	0.062	5.620	3168	WS01010_B03
10	80.088	194	1	-0.338	0.105	9.552	3168	WS01010_B03
10	93.347	196	1	0.638	0.209	9.756	3168	WS01010_B03
11	64.014	222	3	0.241	0.038	4.037	3168	WS01010_B03
1	22.419	4	4	-0.384	0.107	4.311	3169	WS02612_A12
2	63.261	30	3	0.218	0.044	5.079	3169	WS02612_A12
4	75.958	77	3	0.235	0.048	4.409	3169	WS02612_A12
4	116.928	82	2	0.284	0.063	8.098	3169	WS02612_A12
4	116.928	82	4	-0.244	0.057	3.907	3169	WS02612_A12
5	52.820	99	3	-0.360	0.085	9.869	3169	WS02612_A12
5	57.017	102	2	-0.193	0.035	4.340	3169	WS02612_A12

5	57.017	102	4	0.232	0.067	4.770	3169	WS02612_A12
7	70.278	141	3	0.186	0.036	4.139	3169	WS02612_A12
9	3.127	168	1	-0.366	0.088	4.397	3169	WS02612_A12
11	43.491	214	3	0.226	0.049	5.619	3169	WS02612_A12
11	56.921	219	2	-0.333	0.091	8.697	3169	WS02612_A12
3	15.147	44	2	0.302	0.076	5.579	3170	WS02612_P04
6	56.188	120	2	-0.311	0.058	6.964	3170	WS02612_P04
6	81.996	123	3	0.311	0.093	4.201	3170	WS02612_P04
8	50.001	154	1	-0.223	0.058	4.206	3170	WS02612_P04
1	22.419	4	4	-0.296	0.082	5.097	3171	WS0091_A02
1	62.128	11	1	-0.466	0.057	4.362	3171	WS0091_A02
1	95.572	17	2	-0.172	0.049	4.104	3171	WS0091_A02
4	124.132	83	1	-0.455	0.056	4.314	3171	WS0091_A02
4	142.917	85	1	0.256	0.051	5.429	3171	WS0091_A02
7	15.555	130	3	0.230	0.057	4.054	3171	WS0091_A02
7	67.154	140	4	-0.359	0.070	5.308	3171	WS0091_A02
7	73.169	142	1	0.444	0.052	3.939	3171	WS0091_A02
7	75.883	144	4	-0.239	0.055	6.026	3171	WS0091_A02
8	62.580	155	1	-0.295	0.082	6.218	3171	WS0091_A02
8	87.463	158	1	0.230	0.039	4.155	3171	WS0091_A02
1	69.854	13	2	0.198	0.052	5.837	3172	WS00928_A08
1	71.592	14	2	0.197	0.051	5.782	3172	WS00928_A08
1	77.177	15	2	0.204	0.054	6.096	3172	WS00928_A08
1	95.572	17	2	0.269	0.046	5.288	3172	WS00928_A08
1	127.805	21	4	-0.184	0.035	4.090	3172	WS00928_A08
4	173.929	88	2	-0.197	0.034	3.879	3172	WS00928_A08
5	54.072	100	4	0.195	0.035	3.959	3172	WS00928_A08
5	57.017	102	4	-0.253	0.042	4.444	3172	WS00928_A08
8	50.001	154	1	-0.249	0.060	4.695	3172	WS00928_A08
10	70.246	190	2	-0.239	0.049	5.644	3172	WS00928_A08
10	70.246	190	4	0.267	0.036	4.220	3172	WS00928_A08
12	6.242	234	2	-0.204	0.034	4.243	3172	WS00928_A08
14	22.402	248	4	0.408	0.097	11.153	3172	WS00928_A08
1	95.572	17	3	-0.296	0.078	4.580	3173	WS0091_F08
4	92.039	79	4	-0.254	0.036	4.066	3173	WS0091_F08
5	16.219	93	2	0.231	0.040	4.260	3173	WS0091_F08
14	22.402	248	4	0.292	0.051	5.635	3173	WS0091_F08
1	127.805	21	4	-0.209	0.033	3.874	3174	WS00928_D24
4	173.929	88	2	-0.207	0.032	3.964	3174	WS00928_D24
6	81.996	123	3	0.318	0.099	3.935	3174	WS00928_D24
11	27.405	204	4	-0.279	0.065	7.504	3174	WS00928_D24
1	123.316	20	4	0.259	0.058	5.580	3175	WS0101_N13
1	127.805	21	4	-0.232	0.049	6.073	3175	WS0101_N13
1	131.686	23	2	-0.545	0.088	8.233	3175	WS0101_N13
6	89.401	125	1	0.264	0.064	3.988	3175	WS0101_N13
8	50.001	154	1	-0.332	0.128	8.662	3175	WS0101_N13
11	27.405	204	4	-0.339	0.081	8.814	3175	WS0101_N13
2	48.839	29	4	0.258	0.044	4.142	3176	WS0101_O04
4	75.958	77	3	0.308	0.114	5.075	3176	WS0101_O04
4	173.929	88	2	-0.190	0.036	4.494	3176	WS0101_O04
5	54.072	100	4	0.178	0.044	4.452	3176	WS0101_O04
6	81.996	123	3	0.364	0.129	5.408	3176	WS0101_O04
10	78.479	193	3	0.341	0.072	6.229	3176	WS0101_O04
11	27.405	204	4	-0.264	0.048	4.836	3176	WS0101_O04

4	75.958	77	3	0.324	0.125	7.709	3177	WS0102_A07
4	142.917	85	3	-0.320	0.071	5.923	3177	WS0102_A07
5	54.072	100	4	0.251	0.079	6.446	3177	WS0102_A07
5	86.669	106	2	-0.247	0.068	4.185	3177	WS0102_A07
7	17.610	131	3	-0.281	0.087	5.387	3177	WS0102_A07
8	39.090	152	4	-0.207	0.051	4.183	3177	WS0102_A07
9	59.963	177	4	0.266	0.070	4.990	3177	WS0102_A07
12	6.242	234	2	-0.301	0.063	9.488	3177	WS0102_A07
3	15.147	44	2	0.303	0.073	4.483	3178	WS0102_A22
6	56.188	120	2	-0.279	0.044	5.316	3178	WS0102_A22
8	87.463	158	2	-0.171	0.034	4.010	3178	WS0102_A22
4	75.958	77	3	0.280	0.087	3.894	3179	WS0102_C21
6	69.073	121	3	0.315	0.081	6.298	3179	WS0102_C21
6	87.891	124	2	0.171	0.037	4.280	3179	WS0102_C21
6	89.401	125	2	0.302	0.065	5.236	3179	WS0102_C21
13	1.815	244	2	-0.269	0.051	5.965	3179	WS0102_C21
13	1.815	244	4	0.257	0.049	5.497	3179	WS0102_C21
3	47.928	49	1	0.205	0.049	5.216	3180	WS0102_D16
3	137.891	64	3	-0.244	0.079	4.085	3180	WS0102_D16
4	0.000	71	4	-0.332	0.069	7.917	3180	WS0102_D16
4	103.719	81	4	-0.297	0.063	6.859	3180	WS0102_D16
4	116.928	82	4	0.312	0.055	6.293	3180	WS0102_D16
4	124.132	83	1	0.487	0.091	6.376	3180	WS0102_D16
5	42.979	97	1	-0.205	0.037	3.900	3180	WS0102_D16
6	45.897	117	1	0.229	0.039	4.172	3180	WS0102_D16
6	81.996	123	4	-0.375	0.101	7.175	3180	WS0102_D16
7	17.610	131	3	0.270	0.118	4.735	3180	WS0102_D16
7	75.883	144	4	0.286	0.062	4.032	3180	WS0102_D16
10	9.580	187	3	-0.230	0.092	3.928	3180	WS0102_D16
12	22.441	227	1	-0.224	0.038	4.023	3180	WS0102_D16
12	38.543	232	4	0.306	0.080	5.614	3180	WS0102_D16
13	1.815	244	4	0.261	0.048	5.479	3180	WS0102_D16
1	38.326	6	4	-0.314	0.042	4.646	3181	WS0102_F17
1	40.464	7	4	0.407	0.077	7.083	3181	WS0102_F17
1	45.794	9	1	0.411	0.085	7.766	3181	WS0102_F17
1	127.805	21	4	-0.285	0.056	6.340	3181	WS0102_F17
2	48.839	29	4	-0.292	0.059	6.392	3181	WS0102_F17
2	127.147	40	4	-0.229	0.038	4.034	3181	WS0102_F17
3	144.647	67	1	-0.254	0.093	4.503	3181	WS0102_F17
7	62.787	139	4	-0.165	0.031	4.134	3181	WS0102_F17
9	28.608	170	2	0.236	0.045	4.463	3181	WS0102_F17
10	104.214	198	4	-0.281	0.032	5.317	3181	WS0102_F17
11	40.960	210	2	-0.527	0.122	8.440	3181	WS0102_F17
11	40.960	210	4	0.288	0.039	4.351	3181	WS0102_F17
11	46.026	217	1	-0.312	0.067	6.145	3181	WS0102_F17
14	0.000	246	1	-0.261	0.045	4.175	3181	WS0102_F17
14	0.000	246	2	0.229	0.050	4.960	3181	WS0102_F17
14	44.898	251	3	0.269	0.070	5.491	3181	WS0102_F17
3	15.147	44	2	0.237	0.103	5.697	3182	WS0102_G06
11	17.088	203	1	0.239	0.048	4.363	3182	WS0102_G06
11	29.069	205	2	0.304	0.061	5.151	3182	WS0102_G06
11	60.464	221	2	0.220	0.070	5.972	3182	WS0102_G06
1	131.686	23	2	0.432	0.068	4.040	3183	WS0092_E12
2	93.504	32	4	-0.312	0.051	5.236	3183	WS0092_E12

11	64.014	222	3	-0.228	0.056	4.771	3183	WS0092_E12
12	28.493	228	2	0.360	0.077	5.209	3183	WS0092_E12
13	3.386	245	1	-0.222	0.043	3.904	3183	WS0092_E12
5	31.191	95	1	0.272	0.051	5.153	3184	WS0092_J09
5	52.820	99	3	-0.239	0.038	4.100	3184	WS0092_J09
6	81.996	123	3	0.358	0.068	5.134	3184	WS0092_J09
4	75.958	77	3	0.271	0.090	4.083	3185	WS01013_C18
5	31.191	95	1	0.194	0.041	3.972	3185	WS01013_C18
5	52.820	99	3	-0.255	0.046	5.588	3185	WS01013_C18
10	93.347	196	1	0.489	0.079	5.744	3185	WS01013_C18
1	15.589	3	2	-0.194	0.054	4.521	3186	WS01038_M10
1	95.572	17	2	0.277	0.051	4.406	3186	WS01038_M10
3	154.505	69	4	-0.205	0.052	5.308	3186	WS01038_M10
5	31.191	95	1	0.207	0.040	4.190	3186	WS01038_M10
8	50.001	154	1	-0.266	0.063	6.910	3186	WS01038_M10
11	17.088	203	1	-0.239	0.049	5.053	3186	WS01038_M10
11	46.026	217	1	-0.237	0.047	4.910	3186	WS01038_M10
2	126.181	39	2	-0.242	0.048	3.861	3188	WS0269_H24
4	92.039	79	4	-0.415	0.067	7.053	3188	WS0269_H24
9	3.127	168	1	-0.436	0.074	6.301	3188	WS0269_H24
10	93.347	196	1	0.411	0.097	4.056	3188	WS0269_H24
14	22.402	248	4	0.457	0.079	8.040	3188	WS0269_H24
1	22.419	4	3	-0.247	0.100	4.002	3189	WS00712_A24
1	95.572	17	3	0.259	0.064	5.397	3189	WS00712_A24
2	126.181	39	4	0.592	0.183	6.674	3189	WS00712_A24
3	9.953	42	3	-0.236	0.082	6.918	3189	WS00712_A24
5	54.072	100	4	0.261	0.050	5.201	3189	WS00712_A24
5	57.017	102	4	-0.395	0.079	8.384	3189	WS00712_A24
9	2.011	167	4	-0.150	0.045	4.620	3189	WS00712_A24
10	80.088	194	2	-0.192	0.040	4.273	3189	WS00712_A24
1	45.794	9	1	0.326	0.046	4.859	3190	WS00712_H16
1	45.794	9	3	-0.324	0.050	4.226	3190	WS00712_H16
4	97.576	80	2	-0.255	0.042	4.605	3190	WS00712_H16
5	0.000	89	1	0.317	0.043	4.486	3190	WS00712_H16
7	15.555	130	2	0.268	0.049	4.577	3190	WS00712_H16
7	74.560	143	1	-0.223	0.046	4.497	3190	WS00712_H16
7	75.883	144	2	0.471	0.113	7.195	3190	WS00712_H16
7	75.883	144	4	-0.406	0.052	6.256	3190	WS00712_H16
9	3.127	168	1	-0.463	0.056	4.453	3190	WS00712_H16
10	104.214	198	4	-0.312	0.042	4.200	3190	WS00712_H16
13	3.386	245	1	0.281	0.038	3.889	3190	WS00712_H16
1	131.686	23	1	-0.526	0.080	5.360	3191	WS0094_M02
6	69.073	121	4	0.167	0.042	4.293	3191	WS0094_M02
6	87.891	124	2	0.166	0.043	3.930	3191	WS0094_M02
6	89.401	125	2	0.345	0.053	3.858	3191	WS0094_M02
8	0.000	148	4	0.215	0.044	4.871	3191	WS0094_M02
12	6.242	234	4	-0.410	0.065	5.107	3191	WS0094_M02
1	123.316	20	4	-0.327	0.069	5.094	3192	WS0057_C08
1	131.686	23	2	0.775	0.199	13.250	3192	WS0057_C08
1	133.992	24	2	0.218	0.037	4.073	3192	WS0057_C08
2	102.814	36	1	0.240	0.050	4.187	3192	WS0057_C08
3	47.928	49	1	0.237	0.055	4.351	3192	WS0057_C08
6	87.891	124	2	0.268	0.060	6.593	3192	WS0057_C08
6	106.702	128	2	-0.477	0.148	5.648	3192	WS0057_C08

8	95.937	159	3	0.205	0.064	5.749	3192	WS0057_C08
9	0.000	163	1	-0.656	0.174	9.470	3192	WS0057_C08
11	27.405	204	1	-0.304	0.099	8.270	3192	WS0057_C08
11	27.405	204	4	0.343	0.071	6.910	3192	WS0057_C08
1	29.800	5	2	-0.259	0.053	4.081	3193	WS0094_P23
1	38.326	6	2	0.304	0.069	5.804	3193	WS0094_P23
1	62.128	11	2	0.275	0.085	4.344	3193	WS0094_P23
1	133.992	24	2	0.184	0.046	3.868	3193	WS0094_P23
5	31.191	95	1	-0.187	0.039	4.059	3193	WS0094_P23
7	91.487	146	3	0.220	0.069	7.047	3193	WS0094_P23
8	0.000	148	4	0.256	0.062	5.315	3193	WS0094_P23
1	95.572	17	3	0.183	0.042	3.877	3194	WS0057_K23
3	15.147	44	2	-0.259	0.073	4.521	3194	WS0057_K23
4	75.958	77	3	-0.297	0.099	4.008	3194	WS0057_K23
5	16.219	93	2	0.275	0.052	6.668	3194	WS0057_K23
6	95.249	126	1	0.369	0.123	5.985	3194	WS0057_K23
9	49.013	173	2	-0.158	0.034	3.921	3194	WS0057_K23
10	70.246	190	2	0.206	0.033	3.928	3194	WS0057_K23
1	123.316	20	4	-0.264	0.062	4.282	3195	WS01017_E17
1	127.805	21	4	0.247	0.056	5.711	3195	WS01017_E17
2	48.839	29	1	0.285	0.055	4.359	3195	WS01017_E17
5	16.219	93	2	0.284	0.056	5.655	3195	WS01017_E17
5	54.072	100	4	0.237	0.059	6.029	3195	WS01017_E17
5	86.669	106	4	-0.320	0.130	4.144	3195	WS01017_E17
7	15.555	130	3	-0.256	0.055	4.563	3195	WS01017_E17
1	40.464	7	4	0.281	0.074	4.119	3196	WS01017_F02
3	9.953	42	3	-0.212	0.038	5.056	3196	WS01017_F02
3	14.500	43	1	-0.223	0.031	3.942	3196	WS01017_F02
3	17.158	45	1	0.215	0.043	5.545	3196	WS01017_F02
3	141.443	65	3	-0.268	0.058	5.853	3196	WS01017_F02
7	15.555	130	2	0.283	0.071	4.282	3196	WS01017_F02
7	70.278	141	3	0.207	0.035	4.569	3196	WS01017_F02
9	58.045	176	1	0.264	0.048	4.855	3196	WS01017_F02
10	35.204	189	1	-0.215	0.030	3.928	3196	WS01017_F02
3	14.500	43	1	0.311	0.058	6.928	3197	WS01017_G15
3	17.158	45	1	-0.244	0.049	5.784	3197	WS01017_G15
4	0.000	71	4	-0.232	0.041	4.159	3197	WS01017_G15
4	55.925	76	1	0.260	0.049	6.423	3197	WS01017_G15
5	52.820	99	3	-0.230	0.059	5.492	3197	WS01017_G15
11	45.989	216	1	-0.194	0.034	4.054	3197	WS01017_G15
11	46.026	217	1	-0.247	0.045	5.331	3197	WS01017_G15
12	6.242	234	4	0.213	0.043	4.730	3197	WS01017_G15
12	51.382	242	1	-0.463	0.168	9.399	3197	WS01017_G15
1	38.326	6	4	-0.262	0.036	4.049	3198	WS01017_H04
1	62.128	11	1	-0.532	0.131	5.402	3198	WS01017_H04
1	95.572	17	3	0.378	0.101	9.250	3198	WS01017_H04
3	47.928	49	1	0.192	0.064	5.461	3198	WS01017_H04
4	103.719	81	4	-0.275	0.040	5.671	3198	WS01017_H04
6	45.897	117	1	0.304	0.086	7.866	3198	WS01017_H04
7	15.555	130	2	0.288	0.036	3.940	3198	WS01017_H04
9	28.608	170	2	0.303	0.047	5.051	3198	WS01017_H04
3	14.500	43	1	0.325	0.049	5.308	3199	WS01017_I14
3	17.158	45	1	-0.238	0.058	6.227	3199	WS01017_I14
3	83.563	56	3	-0.176	0.049	5.545	3199	WS01017_I14

4	116.928	82	2	-0.292	0.070	5.664	3199	WS01017_I14
6	87.891	124	2	-0.201	0.067	5.466	3199	WS01017_I14
6	89.401	125	2	-0.307	0.069	5.049	3199	WS01017_I14
7	15.555	130	3	0.277	0.044	4.113	3199	WS01017_I14
9	17.951	169	2	-0.294	0.061	4.627	3199	WS01017_I14
1	38.326	6	4	-0.166	0.045	4.140	3200	WS01017_I20
1	77.177	15	2	-0.203	0.043	4.734	3200	WS01017_I20
4	55.925	76	1	0.231	0.050	4.323	3200	WS01017_I20
6	26.350	115	2	-0.255	0.057	6.292	3200	WS01017_I20
6	45.897	117	1	0.305	0.074	5.893	3200	WS01017_I20
6	49.100	118	2	0.412	0.210	12.835	3200	WS01017_I20
6	56.188	120	1	0.300	0.061	4.852	3200	WS01017_I20
6	56.188	120	2	0.392	0.080	9.047	3200	WS01017_I20
6	69.073	121	3	-0.258	0.067	7.569	3200	WS01017_I20
6	81.996	123	3	-0.472	0.272	14.254	3200	WS01017_I20
6	87.891	124	2	0.246	0.064	7.049	3200	WS01017_I20
6	106.702	128	3	0.284	0.041	3.994	3200	WS01017_I20
12	22.441	227	1	0.249	0.059	4.675	3200	WS01017_I20
13	0.000	243	4	0.323	0.190	4.725	3200	WS01017_I20
1	29.800	5	1	-0.327	0.051	4.991	3201	WS01017_K13
1	50.955	10	1	0.242	0.048	4.627	3201	WS01017_K13
3	95.157	59	2	-0.232	0.066	5.493	3201	WS01017_K13
5	42.979	97	1	-0.215	0.043	4.166	3201	WS01017_K13
5	52.820	99	3	-0.235	0.051	4.465	3201	WS01017_K13
6	26.350	115	2	0.233	0.059	5.364	3201	WS01017_K13
12	0.000	233	1	0.191	0.040	3.878	3201	WS01017_K13
14	43.392	250	1	0.303	0.068	4.664	3201	WS01017_K13
1	107.333	19	3	0.200	0.036	4.279	3202	WS01017_L06
2	48.839	29	1	-0.267	0.043	3.918	3202	WS01017_L06
3	80.211	55	4	-0.231	0.058	4.006	3202	WS01017_L06
4	97.576	80	2	0.206	0.044	3.874	3202	WS01017_L06
7	75.883	144	2	0.482	0.109	5.689	3202	WS01017_L06
9	0.000	166	1	0.263	0.052	4.739	3202	WS01017_L06
10	35.204	189	1	0.254	0.054	4.912	3202	WS01017_L06
11	35.604	207	3	-0.178	0.035	4.123	3202	WS01017_L06
11	42.600	213	3	-0.197	0.039	4.595	3202	WS01017_L06
12	22.441	227	3	0.253	0.044	4.585	3202	WS01017_L06
1	38.326	6	4	-0.333	0.070	5.100	3203	WS00946_A08
1	40.464	7	4	0.349	0.064	5.477	3203	WS00946_A08
1	62.128	11	1	-0.542	0.153	5.618	3203	WS00946_A08
1	95.572	17	3	0.287	0.076	7.824	3203	WS00946_A08
3	9.953	42	3	-0.251	0.045	4.771	3203	WS00946_A08
6	89.401	125	1	-0.292	0.055	4.650	3203	WS00946_A08
7	17.610	131	3	0.241	0.078	4.452	3203	WS00946_A08
7	62.787	139	2	0.263	0.038	3.990	3203	WS00946_A08
7	74.560	143	1	-0.202	0.048	4.041	3203	WS00946_A08
9	59.963	177	4	-0.310	0.108	8.833	3203	WS00946_A08
3	144.593	66	4	-0.188	0.042	4.277	3204	WS00817_J10
3	148.745	68	4	0.198	0.044	4.177	3204	WS00817_J10
3	154.505	69	4	-0.245	0.064	5.690	3204	WS00817_J10
4	116.928	82	2	-0.236	0.039	4.038	3204	WS00817_J10
7	15.555	130	2	0.215	0.060	6.100	3204	WS00817_J10
7	74.560	143	1	-0.192	0.053	4.485	3204	WS00817_J10
9	0.000	163	1	-0.529	0.121	6.194	3204	WS00817_J10

10	9.580	187	2	-0.236	0.076	5.160	3204	WS00817_J10
11	46.026	217	1	-0.169	0.052	4.421	3204	WS00817_J10
12	28.493	228	2	-0.195	0.061	4.112	3204	WS00817_J10
1	82.301	16	3	-0.262	0.054	6.715	3205	WS00946_J09
1	95.572	17	3	0.251	0.040	4.910	3205	WS00946_J09
2	95.880	34	3	0.310	0.071	8.784	3205	WS00946_J09
2	101.076	35	3	-0.280	0.055	6.866	3205	WS00946_J09
3	95.157	59	2	-0.220	0.053	6.193	3205	WS00946_J09
3	104.681	60	2	0.244	0.048	5.993	3205	WS00946_J09
3	112.868	61	2	-0.169	0.037	4.538	3205	WS00946_J09
3	114.747	62	2	-0.248	0.044	5.443	3205	WS00946_J09
3	148.745	68	1	-0.257	0.065	4.156	3205	WS00946_J09
3	154.505	69	1	-0.331	0.112	7.242	3205	WS00946_J09
6	81.996	123	3	-0.365	0.139	7.224	3205	WS00946_J09
9	0.000	166	2	0.262	0.053	4.280	3205	WS00946_J09
10	78.479	193	3	-0.326	0.041	5.081	3205	WS00946_J09
3	104.681	60	2	-0.229	0.058	5.773	3206	WS00817_P08
3	112.868	61	2	0.238	0.059	5.849	3206	WS00817_P08
3	114.747	62	2	0.244	0.067	6.634	3206	WS00817_P08
5	0.000	89	3	0.323	0.061	6.952	3206	WS00817_P08
8	62.580	155	1	0.407	0.246	8.959	3206	WS00817_P08
8	78.389	157	1	0.319	0.101	8.928	3206	WS00817_P08
8	87.463	158	1	-0.281	0.080	7.087	3206	WS00817_P08
9	0.000	163	1	-0.513	0.113	5.931	3206	WS00817_P08
10	78.479	193	3	-0.376	0.066	7.019	3206	WS00817_P08
13	1.815	244	2	0.237	0.041	4.108	3206	WS00817_P08
1	95.572	17	2	-0.265	0.069	6.901	3207	WS00922_H13
1	95.572	17	3	0.229	0.048	4.471	3207	WS00922_H13
2	120.617	38	3	0.247	0.048	4.487	3207	WS00922_H13
4	82.462	78	2	-0.242	0.061	5.000	3207	WS00922_H13
9	0.000	163	1	-0.419	0.075	3.865	3207	WS00922_H13
11	35.604	207	3	-0.169	0.041	3.875	3207	WS00922_H13
1	38.326	6	4	-0.260	0.050	4.162	3208	WS00922_L04
1	40.464	7	4	0.239	0.052	4.709	3208	WS00922_L04
1	82.301	16	3	-0.165	0.039	4.276	3208	WS00922_L04
2	127.147	40	3	-0.219	0.038	4.187	3208	WS00922_L04
3	9.953	42	4	0.199	0.048	4.887	3208	WS00922_L04
6	25.914	114	1	-0.189	0.060	6.082	3208	WS00922_L04
6	45.897	117	1	0.353	0.060	6.401	3208	WS00922_L04
7	59.789	137	3	-0.253	0.053	5.866	3208	WS00922_L04
11	37.143	209	3	0.231	0.043	4.713	3208	WS00922_L04
14	0.000	246	2	-0.108	0.038	3.920	3208	WS00922_L04
14	22.402	248	4	-0.270	0.057	3.986	3208	WS00922_L04
1	38.326	6	4	-0.396	0.070	7.918	3209	WS0087_F18
1	40.464	7	4	0.384	0.056	5.855	3209	WS0087_F18
1	45.794	9	1	0.332	0.035	4.817	3209	WS0087_F18
3	17.158	45	1	0.225	0.055	7.494	3209	WS0087_F18
3	47.928	49	1	0.186	0.041	5.583	3209	WS0087_F18
4	124.132	83	1	0.446	0.054	5.057	3209	WS0087_F18
6	89.401	125	1	-0.312	0.036	4.949	3209	WS0087_F18
8	11.974	150	1	-0.247	0.060	8.149	3209	WS0087_F18
8	62.580	155	1	0.344	0.117	10.801	3209	WS0087_F18
8	78.389	157	1	0.349	0.078	10.612	3209	WS0087_F18
8	87.463	158	1	-0.289	0.050	6.841	3209	WS0087_F18

9	3.127	168	1	-0.402	0.061	7.797	3209	WS0087_F18
1	40.464	7	4	0.206	0.043	3.862	3210	WS0087_G17
1	82.301	16	3	-0.293	0.047	4.470	3210	WS0087_G17
5	0.000	89	3	-0.264	0.072	6.679	3210	WS0087_G17
6	106.702	128	2	-0.446	0.094	5.053	3210	WS0087_G17
7	17.610	131	3	-0.387	0.115	6.005	3210	WS0087_G17
7	75.883	144	2	0.403	0.073	4.306	3210	WS0087_G17
10	35.204	189	2	-0.227	0.053	5.459	3210	WS0087_G17
10	70.246	190	2	0.267	0.064	7.073	3210	WS0087_G17
12	6.242	234	4	0.312	0.061	4.858	3210	WS0087_G17
1	50.955	10	1	-0.266	0.056	5.027	3211	WS0087_I22
3	141.443	65	3	-0.317	0.059	4.171	3211	WS0087_I22
10	93.347	196	1	-0.393	0.057	3.960	3211	WS0087_I22
3	15.147	44	2	-0.288	0.144	5.273	3212	WS0087_J23
3	17.158	45	1	0.217	0.053	4.358	3212	WS0087_J23
6	56.188	120	2	0.274	0.063	6.962	3212	WS0087_J23
6	106.702	128	2	0.500	0.096	6.856	3212	WS0087_J23
7	73.169	142	1	0.513	0.079	4.225	3212	WS0087_J23
9	17.951	169	2	0.280	0.048	5.743	3212	WS0087_J23
9	69.046	181	2	-0.323	0.093	6.482	3212	WS0087_J23
1	43.578	8	2	0.199	0.037	4.320	3213	WS0087_M07
4	142.917	85	1	-0.267	0.067	4.787	3213	WS0087_M07
7	75.883	144	2	0.509	0.100	7.040	3213	WS0087_M07
8	87.463	158	2	0.291	0.074	8.543	3213	WS0087_M07
9	36.227	165	3	0.190	0.053	4.381	3213	WS0087_M07
9	17.951	169	1	-0.392	0.086	6.272	3213	WS0087_M07
9	17.951	169	2	0.260	0.042	5.828	3213	WS0087_M07
11	46.026	217	1	-0.385	0.164	11.952	3213	WS0087_M07
14	22.402	248	3	-0.290	0.061	5.004	3213	WS0087_M07
14	43.392	250	2	0.235	0.052	4.173	3213	WS0087_M07
1	38.326	6	4	-0.299	0.033	4.066	3214	WS0087_M24
1	40.464	7	4	0.312	0.036	4.439	3214	WS0087_M24
4	92.039	79	4	-0.331	0.043	5.301	3214	WS0087_M24
4	103.719	81	4	-0.194	0.032	5.329	3214	WS0087_M24
4	142.917	85	1	-0.171	0.032	3.970	3214	WS0087_M24
5	86.669	106	4	-0.420	0.071	5.334	3214	WS0087_M24
6	49.100	118	4	0.408	0.059	4.534	3214	WS0087_M24
7	15.555	130	2	0.323	0.085	5.415	3214	WS0087_M24
7	67.154	140	4	-0.310	0.100	7.567	3214	WS0087_M24
7	73.169	142	1	0.500	0.062	4.787	3214	WS0087_M24
7	74.560	143	1	-0.263	0.041	5.080	3214	WS0087_M24
7	75.883	144	4	-0.356	0.056	3.933	3214	WS0087_M24
9	3.127	168	1	-0.344	0.044	5.810	3214	WS0087_M24
3	154.505	69	1	0.250	0.074	6.358	3215	WS0087_P05
4	55.925	76	1	-0.176	0.046	3.916	3215	WS0087_P05
5	0.000	89	3	0.292	0.057	6.664	3215	WS0087_P05
5	56.008	101	2	-0.232	0.049	4.498	3215	WS0087_P05
5	57.017	102	2	0.275	0.065	6.862	3215	WS0087_P05
8	78.389	157	1	0.247	0.050	4.316	3215	WS0087_P05
11	0.000	199	2	-0.278	0.052	5.401	3215	WS0087_P05
4	43.146	74	3	0.199	0.050	4.751	3216	WS00715_A01
5	31.191	95	1	-0.243	0.067	5.704	3216	WS00715_A01
5	42.979	97	1	0.288	0.046	3.892	3216	WS00715_A01
5	42.979	97	2	-0.225	0.040	4.113	3216	WS00715_A01

7	74.560	143	1	0.312	0.060	5.126	3216	WS00715_A01
10	78.479	193	4	-0.238	0.055	5.147	3216	WS00715_A01
14	43.392	250	2	0.206	0.042	3.942	3216	WS00715_A01
4	142.917	85	1	-0.203	0.044	4.139	3217	WS00815_D09
5	16.219	93	2	0.304	0.055	4.726	3217	WS00815_D09
5	54.072	100	4	-0.272	0.068	6.705	3217	WS00815_D09
5	57.017	102	4	0.178	0.043	4.221	3217	WS00815_D09
6	81.996	123	4	0.532	0.139	7.882	3217	WS00815_D09
6	89.401	125	2	0.286	0.040	4.054	3217	WS00815_D09
7	74.560	143	1	-0.305	0.059	5.566	3217	WS00815_D09
7	75.883	144	4	-0.360	0.066	5.515	3217	WS00815_D09
9	55.358	175	1	0.400	0.089	8.630	3217	WS00815_D09
9	55.358	175	3	-0.384	0.103	4.361	3217	WS00815_D09
11	27.405	204	1	-0.252	0.050	4.963	3217	WS00815_D09
11	40.960	210	2	-0.396	0.100	4.652	3217	WS00815_D09
11	42.600	213	3	-0.213	0.044	3.923	3217	WS00815_D09
11	46.026	217	1	-0.281	0.045	4.449	3217	WS00815_D09
11	64.014	222	4	0.320	0.099	9.632	3217	WS00815_D09
12	6.242	234	4	-0.354	0.065	4.816	3217	WS00815_D09
14	44.898	251	3	0.204	0.041	3.880	3217	WS00815_D09
3	116.958	63	4	0.239	0.096	8.092	3218	WS00815_I12
9	0.000	163	1	-0.469	0.089	4.860	3218	WS00815_I12
13	3.386	245	1	0.291	0.064	5.784	3218	WS00815_I12
14	46.637	252	4	0.278	0.052	4.651	3218	WS00815_I12
1	2.788	2	4	0.146	0.029	4.844	3219	WS00715_C22
1	62.128	11	1	-0.463	0.081	5.143	3219	WS00715_C22
[truncated: 524,140 more chars]
